# Installing Axial Chirality by Atroposelective C(sp^3^)–H Bond Oxidation

**DOI:** 10.1021/jacs.6c06123

**Published:** 2026-05-12

**Authors:** Margarida Borrell, Marco Galeotti, Laia Vicens, Tomer Mintz, Arnau Call, Doron Pappo, Miquel Costas

**Affiliations:** † Institut de Química Computacional i Catàlisi (IQCC) and Departament de Química, 451767 Universitat de Girona, Campus Montilivi, Girona, Catalonia 17071, Spain; ‡ Department of Chemistry, 26732 Ben-Gurion University of the Negev, Beer-Sheva 8410501, Israel

## Abstract

The synthesis of optically enriched multifunctionalized biaryl compounds by a non-directed atroposelective C­(sp^3^)–H bond oxidation of 2,6-dimethyl-1,1′-biaryls is reported. The reaction is catalyzed by a novel family of manganese complexes bearing a highly structured oxidation site, which is shown to be the key factor controlling the excellent chemo- and enantioselectivity of the reaction. The system operates under mild conditions, using low catalyst loading and H_2_O_2_ as oxidant, enabling compatibility with configurationally labile substrates and tolerance toward a wide variety of functional groups that are typically sensitive in organometallic catalysis. The devoid of directing groups on the substrate core allows access to axially chiral biaryls (42 examples) decorated with a plethora of functionalities in high yields (up to 88%) and outstanding enantioselectivity ratios (up to >99% ee). Furthermore, the exquisite site- and enantioselective C–H oxidation activity of the catalysts enables subsequent site- and chemoselective oxidation of the second methyl group, offering a novel and versatile platform for elaboration of the biaryl core. Overall, the catalysts’ activity enables recognition of methyl groups as functional groups for synthetic manipulations, showcasing the strategic use of C–H bond functionalization reactions as a powerful approach for stereoselective synthesis.

## Graphical abstract

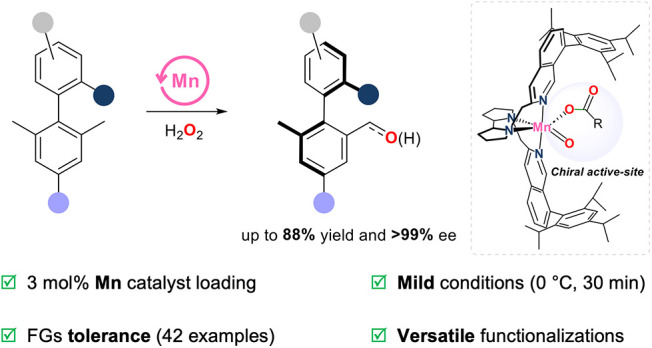

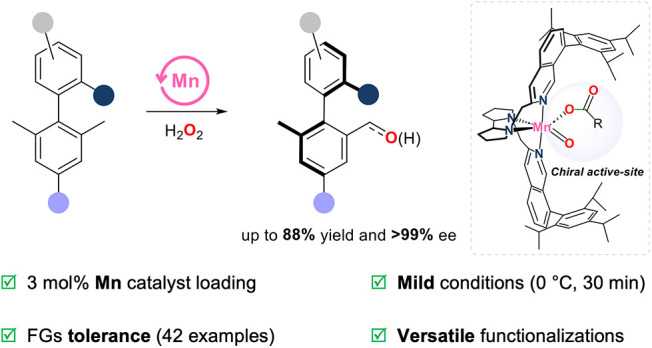

## Introduction

The enantioselective C­(sp^3^)–H bond functionalization has emerged as a powerful strategy in modern chemistry for simultaneously installing functionality and chiral information into ubiquitous C–H bonds.^1–3^ By circumventing the need for functional directing groups, these reactions offer excellent atom economy and powerful synthetic strategies for accessing a diverse array of chiral molecules from readily available achiral starting materials. Chiral iron and manganese oxidation catalysts that emulate the activity of iron-dependent hydroxylases by generating high-valent metal-oxo species that engage in site- and enantioselective hydrogen atom transfer (HAT)/radical recombination reactions (Figure 1A) successfully discriminate among enantiotopic C­(sp^3^)–H bonds in *meso*-molecules.^4–10^ While these chiral catalysts are highly successful at simultaneously uncovering point chirality at multiple remote enantiotopic centers,^11–14^ and axial chirality in spirocyclic compounds,^15,16^ their application for inducing axial chirality in biaryl compounds has never been achieved, probably because this challenging reaction requires catalysts with a highly structured oxidation site to distinguish between two remote atropotopic C­(sp^3^)–H bonds.

**Figure 1:**
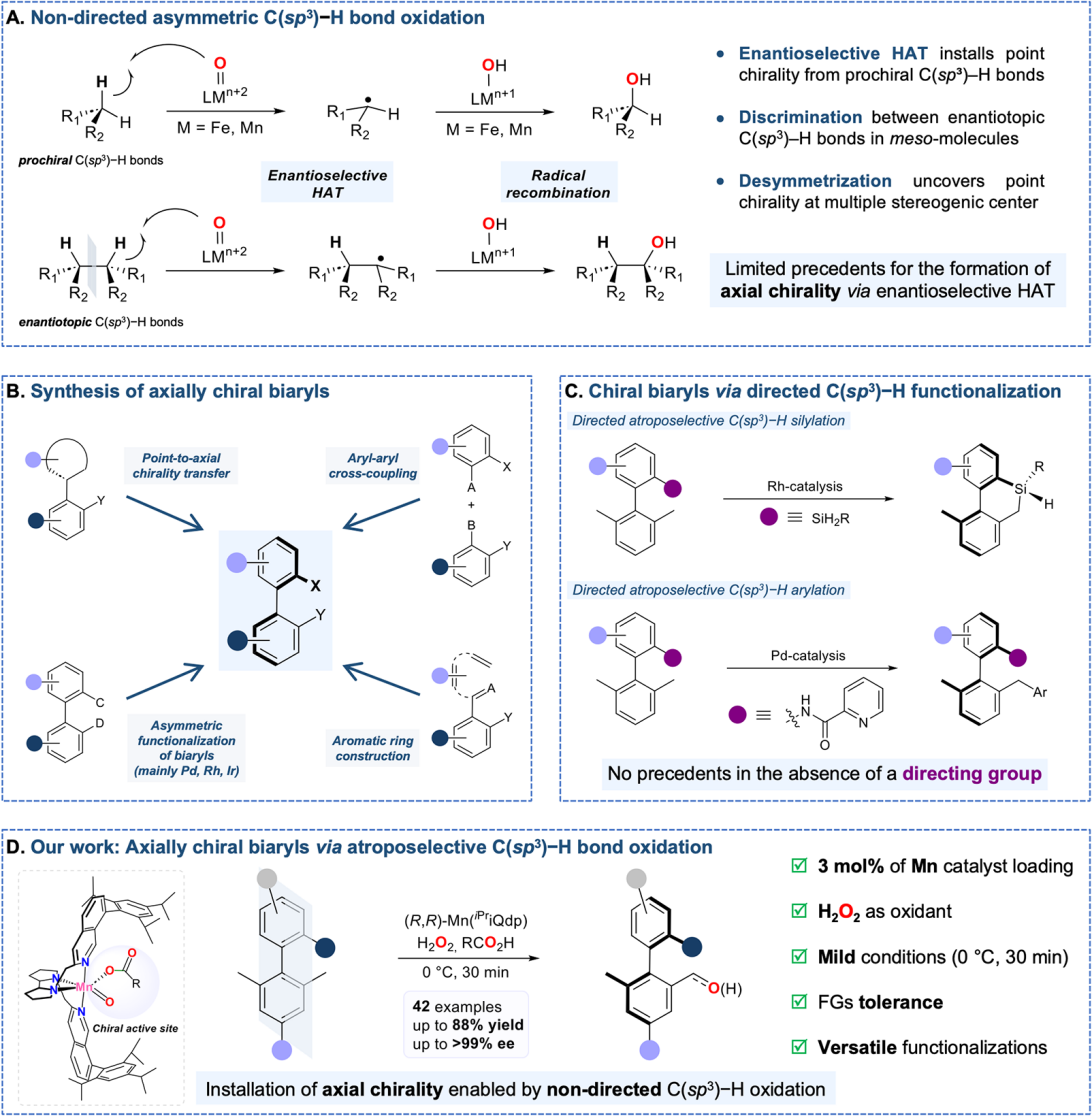
(A) Enantioselective C­(sp^3^)–H oxidation to install point chirality, (B, C) preparation of axially chiral biaryls and (D) overview of this work resulting in the installation of axial chirality. DG, directing group. FG, functional group.

Since the isolation of the first optically active atropoisomer in 1922,^17^ axially chiral compounds have been recognized as constituents of bioactive molecules^18^ and have become pervasive entities widely applied in asymmetric catalysis^19^ and functional materials.^20^ Accordingly, the past several decades have witnessed an explosion of applications and methods for the synthesis of axially chiral biaryls (Figure 1B). These methods have mainly relied on manipulating preexisting functionality.^21–26^ More recently, the advent of C–H bond functionalization reactions has provided new synthetic tools for this endeavor.^3,27–31^ Asymmetric C­(sp^2^)–H functionalizations have emerged as powerful reactions, providing new disconnections for atroposelective target-oriented synthesis.^32–36^ By contrast, the notorious limited reactivity of C­(sp^3^)–H bonds stands as a major challenge, and so far only two examples have been reported (Figure 1C), both of which rely on directing groups. In 2021, He and coworkers described a directed Rh-catalyzed enantioselective C­(sp^3^)–H silylation of biaryls.^37^ More recently, Akiyama and coworkers disclosed a directed approach for the synthesis of axially chiral biaryls via C­(sp^3^)–H desymmetrizative arylation, enabled by a chiral palladium phosphate catalyst.^38^ Recent breakthroughs have disclosed catalytic methods that utilize 3d-transition metals to install axial chirality,^39–69^ avoiding the traditional requirement of precious-metal catalysts (such as Rh, Pd and Ir). However, the necessity to install and remove directing groups remains a longstanding limitation in atroposelective C–H bond functionalization reactions.^32,35,36^

Herein, we show a non-directed manganese-catalyzed atroposelective C­(sp^3^)–H bond oxidation of 2,6-dimethyl-1,1′-biaryls (referred to as biaryls in this study) that afford chiral biaryl compounds with chemically versatile oxygenated functionality. The installation of axial chirality in exceptional site- and enantioselectivity proceeds via a HAT/radical recombination step within the chiral cavity of a novel Mn­(OTf)_2_(^iPr^iQdp) catalyst, allowing methyl groups to be recognized as orthogonal functional handles for further selective transformations, thereby highlighting the strategic potential of C–H bond oxidation as a powerful approach for the enantioselective construction of axially chiral biaryl frameworks.

## Results and Discussion

### Reaction Development

Manganese complexes bearing bulky tris­(isopropyl)­silyl (TIPS) substituents on the pyridine donors of tetradentate aminopyridine ligands have been shown to exhibit high enantioselectivity in the desymmetrization of cyclohexyl derivatives through nondirected C­(sp^3^)–H oxidation, proceeding via an initial enantioselective HAT.^12,14^ We considered applying the reaction to introduce axial chirality into biaryl moieties, which are widely used to transfer stereochemical information to metals in enantioselective catalysis (e.g., BINAP and CPA) and in metal–organic frameworks for a variety of applications.^19,20^ Therefore, we first explored the ability of Mn­(OTf)_2_(^TIPS^pdp) (**C1**) and Mn­(OTf)_2_(^TIPS^ecp) (**C2**) (where pdp = *N,N′*-*bis*(2-pyridylmethyl)-2,2′-bipyrrolidine and ecp = *N,N′*-diethyl *N,N′*-*bis*(2-pyridylmethyl)-1,2-*trans*-diaminocyclohexane, Figure 2B) to promote the oxidative desymmetrization of 4′-(*tert*-butyl)-2′,6′-dimethyl-[1,1′-biphenyl]-2-carbonitrile (**1**), which bears two atropotopic ortho-methyl groups (Figure 2A, entries 1–2).

**Figure 2:**
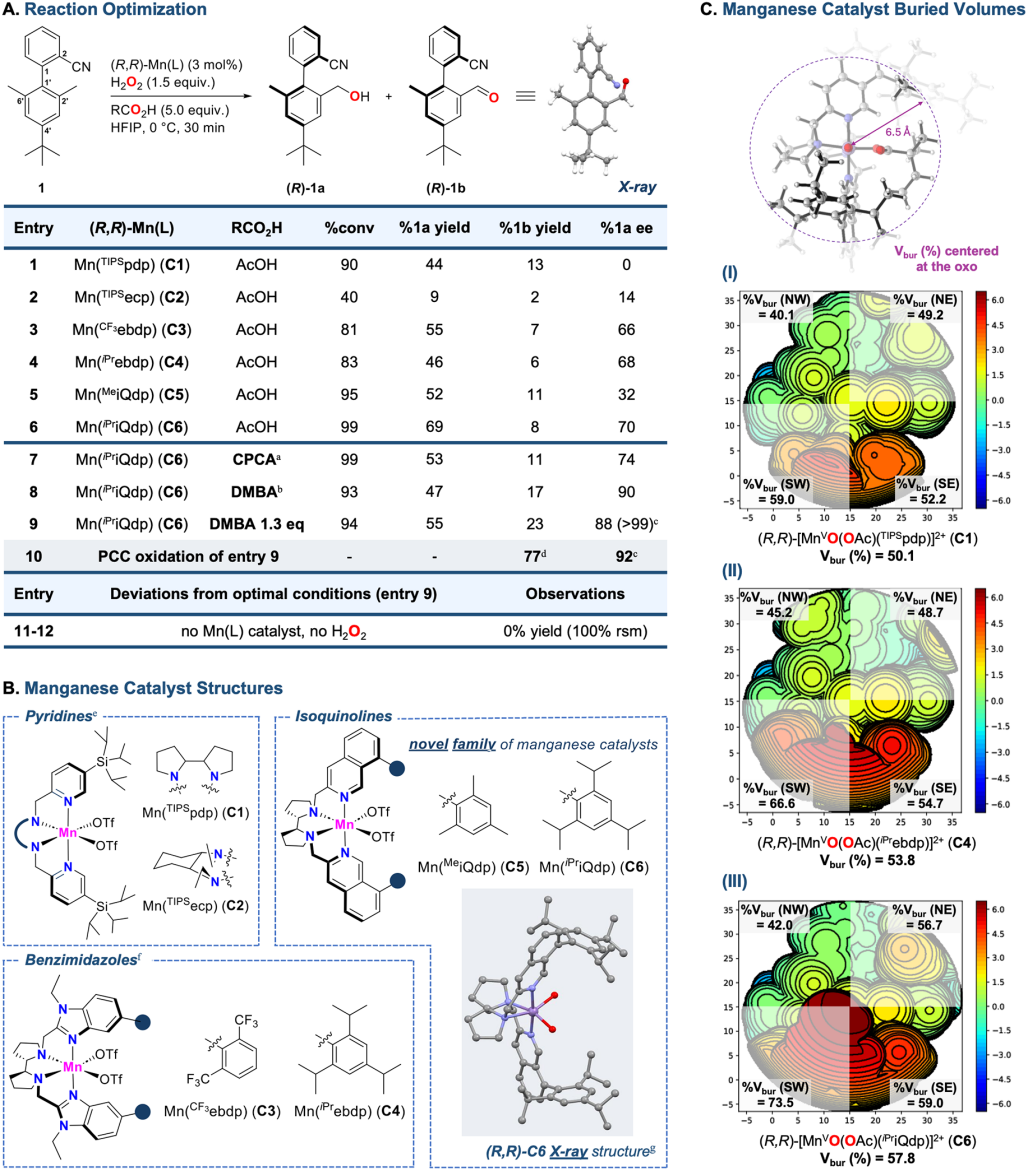
Reaction optimization and manganese catalysts exploration. Conversion and yields were determined by GC with reference to an internal standard. Ee values were determined by chiral SFC analysis. Absolute stereochemistry of **1b** (*R*) has been tentatively assigned in analogy to *R* **-28a** (Figure 4). (A) Evaluating the effects of the catalysts and carboxylic acids, as well as control experiments. ^a^Cyclopropanecarboxylic acid. ^b^2,2-dimethylbutanoic acid. ^c^%**1b** ee. ^d^Isolated yield. (B) Catalyst structures evaluated based on pyridines, benzimidazoles and isoquinolines. ^e^Ref 14. ^f^Ref 72. ^g^Recrystallized in DMSO (CCDC Number 2519089). With the exception of the Mn bound oxygen atoms, triflate groups are omitted for clarity. (C) Calculated steric maps of the DFT-calculated manganese-oxo-acetate species visualized by SambVa application at a radius of 6.5 Å with 0.03 mesh (details are provided in the Supporting Information).

Standard conditions involved 3 mol % of Mn catalyst, 5 equiv of AcOH and 1.5 equiv of H_2_O_2_, delivered over 30 min using a syringe pump in 1,1,1,3,3,3-hexafluoro-2-propanol (HFIP) at 0 °C. For both the catalysts, oxidation occurs selectively at the primary benzylic C–H bonds to deliver the alcohol product (**1a**) in yields of 44% and 9%, accompanied by the formation of the corresponding aldehyde (**1b**) in 13% and 2% yields, respectively. No products arising from the oxidation of the primary C–H bonds of the *tert*-butyl substituent were observed, in line with the lower bond dissociation energy of the benzylic C–H bonds of methyl groups at C-2′ and C-6′. However, poor enantioselectivities were measured with these catalysts (0% and 14% ee for **C1** and **C2**, respectively).

Building on prior work we hypothesized that the enantioselectivity of the HAT event will be sensitive to the shape of the coordination site where the reactive manganese-oxo is formed.^11^ We then pursued increasing the buried volume around the oxidation site using tetradentate ligand frameworks with rigid, sterically encumbered ligand substituents. Among these, given the aromatic nature of the targeted substrates, we strategically employed catalysts bearing aromatic substituents to provide shape complementarity and arene–arene interactions that could contribute to effective chiral recognition (Figure 2C). Accordingly, manganese complexes featuring sterically demanding bis-5-aryl-benzimidazole bipyrrolidine ligands with formula [Mn­(OTf)_2_(^Ar^ebdp)] (where ebdp = *N,N′*-bis­(ethylbenzimidazole)-2,2′-bipyrrolidine, and Ar = 2,4-trifluoromethylphenyl (Mn­(OTf)_2_(^CF3^ebdp), **C3**), 2,4,6-triisopropylphenyl (Mn­(OTf)_2_(^iPr^ebdp), **C4**), Figure 2B) were evaluated (entries 3–4), affording improved yields (46–55%) together with promising enantioselectivities (66–68% ee). The enhanced ee correlates with the increase in buried volume from **C1** (V_bur_ = 50.1%) to **C4** (V_bur_ = 53.8%) calculated in the active site of the putative manganese-oxo species, providing initial validation for our hypothesis (Figure 2C–I–II).

Encouraged by these results, we turned our attention to sterically hindered manganese catalysts bearing recently described bis-8-aryl-isoquinoline bipyrrolidine ligands.^70^ Two novel chiral manganese complexes with the general formula [Mn­(OTf)_2_(^Ar^iQdp)] (where iQdp = *N*,*N*′-bis­(isoquinoline)-2,2′-bipyrrolidine, and Ar = 2,4,6-trimethylphenyl (Mn­(OTf)_2_(^Me^iQdp), **C5**), 2,4,6-triisopropylphenyl (Mn­(OTf)_2_(^iPr^iQdp), **C6**), Figure 2B) were synthesized and evaluated under catalytic conditions (synthetic details are provided in the Supporting Information). Analysis of the active site of the manganese-oxo species of **C6** (Vbur = 57.8%) reveals a highly structured, rigid, and sterically constrained cleft, envisioned to be particularly well suited for recognizing aromatic substrates and rendering **C6** a promising candidate for enhanced enantioinduction (Figure 2C–III).

Consistent with these structural features, oxidation of **1** with **C5** afforded **1a** in 52% yield and modest 32% ee (entry 5), whereas **C6**, bearing additional steric bulk at the C8 position of the isoquinoline scaffold, delivered **1a** in 69% yield and an optimal 70% ee, highlighting the pronounced effect of steric congestion on atropodiscrimination (entry 6).

We next examined the role of the acid, which serves as a coligand in defining the active site (Figure 1D, on the left).^14^ When the oxidation of **1** with catalyst **C6** was performed using the more sterically demanding acids cyclopropane carboxylic acid (CPCA, entry 7) or 2,2-dimethylbutanoic acid (DMBA, entry 8) instead of AcOH, product **1a** was obtained in 74% ee and an outstanding 90% ee, respectively. Of notice, buried volume analysis of Mn^V^-oxo **C6** in combination with the sterically hindered 2,2-dimethylbutanoic acid (DMBA) revealed the formation of a particularly rigid and sterically congested active site, with a V_bur_ of 62.7% (Figure S5 in the Supporting Information). Further optimization revealed that oxidation of **1** in the presence of 1.3 equiv of DMBA, afforded **1a** and **1b** in a combined yield of 78% (**1a**:**1b** ratio of 2.4) with 88% and >99% ee, respectively (entry 9). Formation of **1b** originates from oxidation of **1a** and its high enantioselectivity benefits from a kinetic resolution in this reaction.^71^ When the reaction crude was treated with PCC (pyridinium chlorochromate), **1b** was obtained in 77% isolated yield with 92% ee (entry 10). Notably, the reaction proceeds with excellent mass balance. Of notice, the lack of oxidation products on the aryl rings demonstrates the outstanding chemoselectivity (C­(sp^3^)–H over C­(sp^2^)–H bonds) of the catalyst. Finally, control experiments confirmed that no product formation occurs in the absence of either the manganese catalysts or hydrogen peroxide (entries 11–12 and Supporting Information).

### Scope Exploration for the Atroposelective C­(sp^3^)–H Oxidation of Biaryls

With the optimized protocol in hand, we investigated the atroposelective C–H bond oxidation of various biaryl substrates having different functionalities at the C-2 position (Figure 3). Isolated yields and enantioselectivities of the corresponding alcohols were reported. However, upon detection of overoxidation products, the crude mixture was treated with PCC, and the corresponding aldehydes were isolated. For some biaryl substrates, using **C2** rather than **C6** was required to achieve higher enantioselectivities (deviations from the general procedure are detailed in Figure 3). Although buried volume analysis indicates that **C2** has a lower steric demand than **C6** (V_bur_ = 50.9%; see Figure S2 in the Supporting Information), the optimum performance of this catalyst in selected cases indicates that, beyond the steric properties of the catalyst, specific interactions between the catalyst and substrate functionalities may also influence atropodiscrimination during the HAT step.

**Figure 3:**
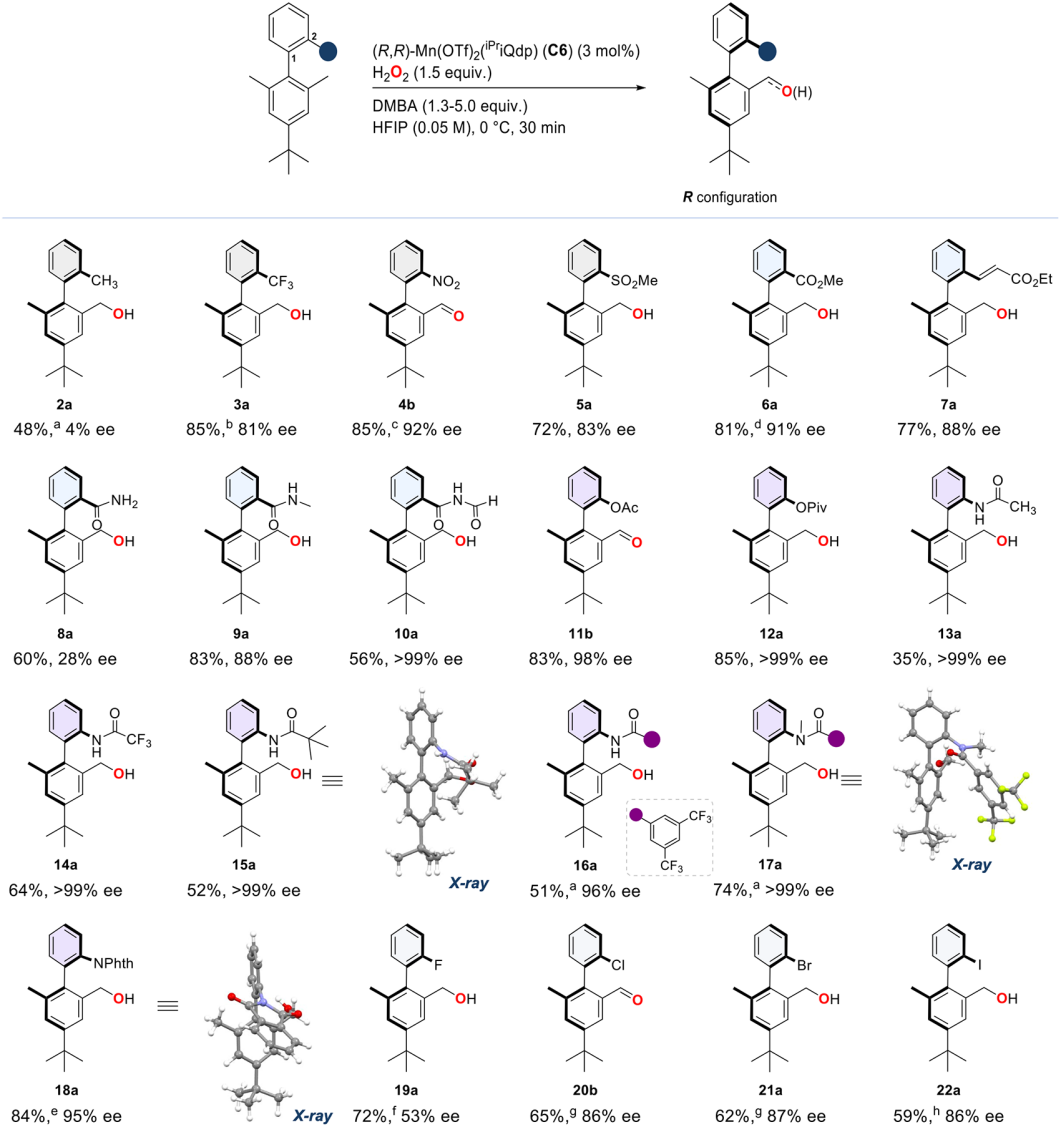
Functional group tolerance at the C-2 position of the biaryls. Yields are isolated yields of the alcohol or aldehyde products. Ee values were determined by chiral SFC analysis. Absolute stereochemistry of the atropisomeric products in the scope have been tentatively assigned in analogy to *R* **-28a** (Figure 4). Atropoisomeric products **3a**, **20b** and **21a** obtained with (*R,R*)-**C2** catalyst show the same absolute configuration of the other products (details are provided in the Supporting Information). Deviations from standard conditions: ^a^AcOH 5 equiv. ^b^Catalyst **C2** (3 mol %), HFIP/TFE 2:1, −20 °C. ^c^DMBA 1.3 equiv. ^d^DMBA 1.3 equiv, HFIP/TFE 2.1, −20 °C. ^e^0.5 mmol scale. ^f^AcOH 1.3 equiv, 25 °C. ^g^Catalyst **C2** (3 mol %), 25 °C. ^h^DMBA 1.3 equiv, 25 °C.

The atroposelective C–H oxidation of biaryl **2** with a methyl substituent at the C-2 position afforded biaryl **2a** in moderate 48% yield. To our delight, replacement of the methyl group with a trifluoromethyl substituent provided the corresponding alcohol product **3a** in 85% yield and 81% ee.

At this point, the functional group tolerance of the transformation was examined by reacting biaryl substrates bearing 2-nitro, 2-methylsulfonyl, 2-methyl carboxylate, and 2-ethyl acrylate groups (**4**–**7**). To our delight, the corresponding products were obtained in high yields (72–90%), and with excellent enantiomeric excesses (up to 91% ee for **6a**). Notably, the selective formation of **7a** demonstrates that the Mn­(OTf)_2_(^iPr^iQdp) catalyst enables chemo- and enantioselective C­(sp^3^)–H oxidation in the presence of α,β-unsaturated carbonyl groups, overriding the intrinsically favored epoxidation of the olefin moiety.^73,74^

Introduction of a primary amide group (−CONH_2_) resulted in product **8a** being obtained in 60% yield and only 28% ee, but satisfyingly, the oxidation of the *N*-methyl derivative of **9** led to the formation of **9a** in outstanding 83% yield and 88% ee.

Encouraged by the straightforward accessibility of chiral *N*-substituted amide compounds, we explored the synthesis of chiral *N*-formyl imides, which, owing to their highly reactive formyl group, serve as key intermediates in the preparation of valuable compounds such as triazoles, enamides, benzimidazoles, 4-quinazolinones, and *N*-formyl amines.^75^ Oxidation of the 2-(*N*-formyl benzamide)-biaryl derivative **10** afforded the corresponding alcohol product **10a** in 56% yield with >99% ee, representing a precursor for the synthesis of axially chiral biaryls featuring nitrogen-containing functional architectures. *Inter alia*, straightforward access to amino acids of interest for the preparation of recyclable polymer^76^ and to chiral biphenyl amidinate ligands employed in asymmetric catalysis is devised.^77^

Oxidation of the 2-acetate and 2-pivalate biaryl derivatives provided products **11b** and **12a** in remarkable yields (82–84%) and with 98% and >99% ee, respectively, demonstrating that biaryl scaffolds bearing C–O bonds are also well tolerated under these reaction conditions. Similarly, excellent enantiocontrol was achieved in the oxidation of the acetamide derivative, affording **13a** as a single atropoisomer (>99% ee), albeit in a modest 35% yield. Pleasingly, replacement of the acetamide group with trifluoroacetamide, pivalamide, 3,5-bis­(trifluoromethyl)­benzamide, and *N*-methyl-3,5-bis­(trifluoromethyl)­benzamide and phthalimide, led to the formation of **14a**-**18a** in good to excellent yields (51–83%) and with outstanding enantioselectivities (up to >99% ee).

Finally, we examined the compatibility of the method toward halide groups. Oxidation of 2-fluoro-2′,6′-dimethylbiaryl **19** afforded **19a** in 72% yield and moderate 53% ee. The low atroposelectivity observed in this example is likely due to the small size of the fluorine atom, which presumably facilitates atropoisomerization during the reaction, leading to partial loss of optical purity.^34^ Gratifyingly, oxidation of the larger chloro-, bromo-, and iodo derivatives (**20**–**22**) delivered the corresponding products in good yields (58–65%) and with significantly improved enantioselectivities (84–88% ee). These results highlight the excellent tolerance of the reaction toward halogen substituents, orthogonal to organometallic reactants, and underscore their value as versatile synthetic handles for preparing C1-symmetrical ligands and catalysts,^78^ as well as for accessing homochiral polyarenes that are increasingly employed in nanotechnology.^79^

### Translation to Poly Substituted Biaryls

The power of the new method in preparing optical pure biaryl compounds was demonstrated by reacting a series of C-3-, C-4- and C-5-substituted *N*-(4′-(*tert*-butyl)-2′,6′-dimethyl-[1,1′-biphenyl]-2-yl)­pivalamides under the optimized conditions (Figure 4A). The oxidation of substrates bearing nitrile and bromo substituents at the C-3 position afforded products **23a** and **24a** in 61% and 81% yields, respectively, with both compounds exhibiting outstanding enantioselectivities (>99% ee). Furthermore, oxidation of 3,5-difluoro biaryl derivative **25** delivered the corresponding aldehyde **25b** in 87% yield and >99% ee. These examples collectively demonstrate the compatibility of multiple functional groups. Moving to C-4 substituted biaryls, oxidation of the *tert*-butyl derivative furnished alcohol **26a** in 59% yield and 98% ee. Excellent yields (63–99%) and enantioselectivities (>99% ee) were also observed for biaryls bearing electron-withdrawing groups (**27**–**29**). Notably, we were able to determine the absolute stereochemistry of **28a** (*R*) by X-ray crystallography (all others were tentatively assigned by analogy). To demonstrate generality, the 4-fluoro-2-CO_2_Me derivative was also tested, affording alcohol **30a** in 68% yield and 97% ee. Finally, oxidation of 5-nitro (**31**) and 5-halogenated 2-pivaloylamide biaryls (**32**–**34**) provided the corresponding oxidation products in excellent yields (71–90%) and enantioselectivities (98–>99% ee).

**Figure 4:**
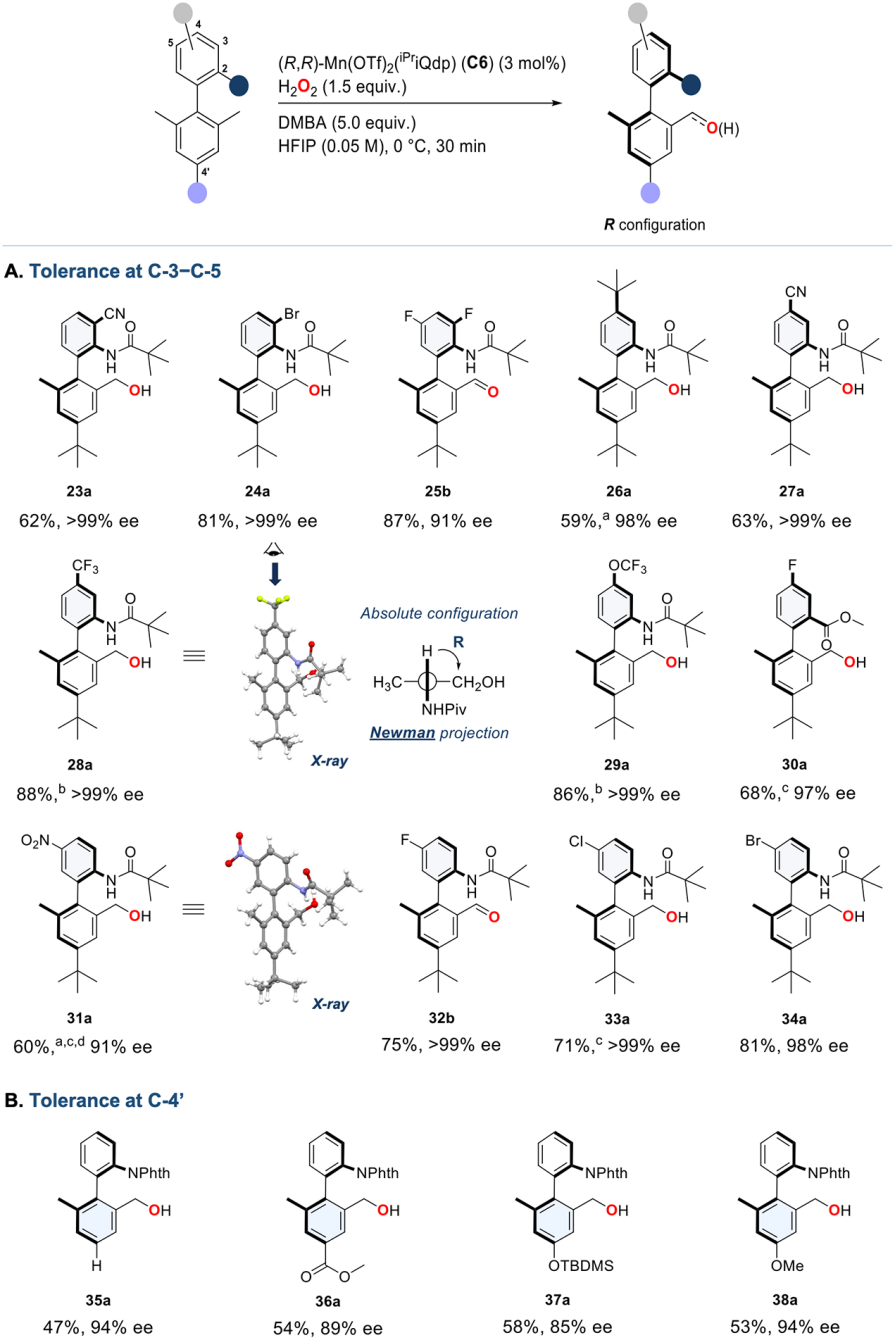
Extended scope of the reaction. Yields are isolated yields of the alcohol or aldehyde products. Ee values were determined by chiral SFC analysis. Absolute stereochemistry of the atropisomeric products in the scope have been tentatively assigned in analogy to *R*-**28a**. (A) Evaluating biaryls with functional groups at C-3–C-5. *^a^* H_2_O_2_ 2.0 equiv. *^b^* DMBA 1.3 equiv. *^c^* AcOH 5 equiv. ^d^15% yield and 96% ee with DMBA 5 equiv. (B) Evaluating biaryls with functional groups at C-4′.

At this point, we turned our attention to extending the oxidation protocol to biaryl substrates lacking the *tert*-butyl substituent at the C-4′ position (Figure 4B). Pivalamide substituted substrates proved somewhat unstable to the standard oxidation conditions (see Supporting Information). However, phthalamide substitution provided a satisfactory solution. The oxidation of 2-phthalimido biaryl **35** that lacks a substituent at the C-4′ position afforded the desired alcohol **35a** in 47% yield and with 96% ee. The oxidation of the 4′-methyl carboxylate derivative provided **36a** with an excellent 89% ee. Electron-donating substituents such as 4′-OTBDMS (**37**) and 4′-OMe (**38**), which are valuable synthetic handles, were also well tolerated under the oxidation conditions, retaining high enantioselectivity, with the major products having a similar *R* configuration. Interestingly, no products resulting from the oxidation of the methoxy group in substrate **38** were detected. This behavior may be attributed to the fluorinated alcohol solvent, which likely deactivates otherwise reactive primary C–H bonds through hydrogen bonding with the oxygen atom,^80^ and to the orientation of the substrate within the catalytic site.^70^

### Simultaneous Installation of Point and Axial Chirality

Encouraged by the broad scope of this catalytic system, we next examined the possibility of simultaneously generating axial and point chirality, which represents a long-standing challenge in the synthesis of such chiral architectures.^81^ As an illustrative application, substrate **39**, chosen because of the particularly high stability of the phthalamide group, bearing ethyl groups at the C-2′ and C-6′ positions, was oxidized to give a mixture of diastereomeric alcohols (**39a** and **39a′**) in 80% combined yield with a 92:8 d.r. and ee values of 83% and >99%, respectively (Figure 5A). Subsequent PCC oxidation of the mixture, which removes the point chirality while preserving the axial configuration, afforded ketone **39b** in quantitative yield and 81% ee, which matches the enantioselectivity of the major diastereomer. This outcome establishes the feasibility of concurrent axial and point chirality generation under the present catalytic manifold and indicates that both **39a** and **39a′** share the same axial configuration, with the observed diastereoselectivity arising from differentiation at a single stereogenic center that is erased upon oxidation.

**Figure 5:**
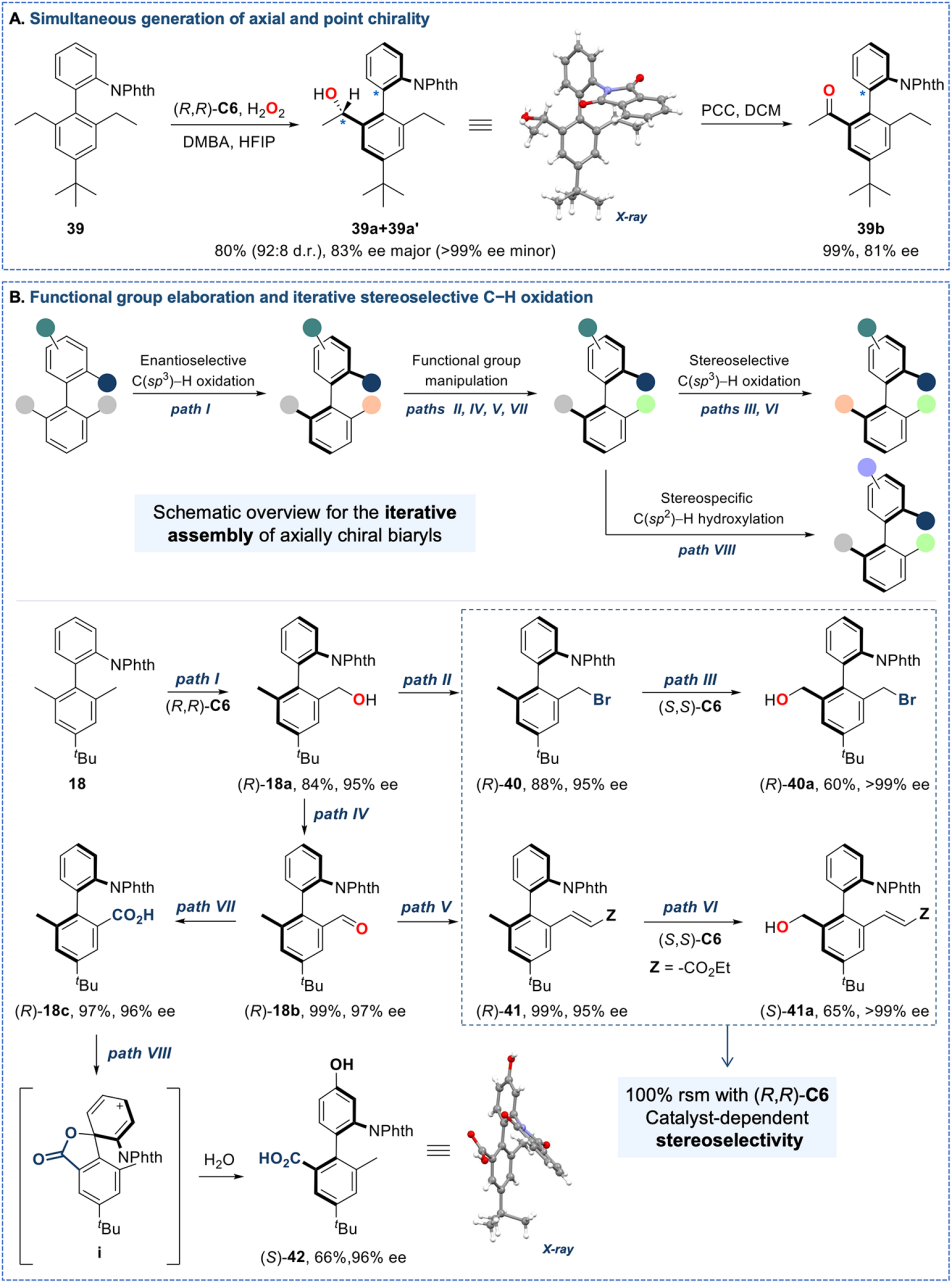
Synthetic applications. Yields are isolated yields of the products. Ee values were determined by chiral SFC analysis. Synthetic details are reported in the Supporting Information. Absolute stereochemistry of the atropisomeric products in the scope have been tentatively assigned in analogy to *R*-28a. (A) Viability of generating axial and point chirality in one step. (B) Elaboration of the chiral biaryl products. Paths: (I) Catalyst (*R*,*R*)-**C6,** H_2_O_2_, DMBA, HFIP; (II) CBr_4_, PPh_3_, DCM; (III) Catalyst (*S*,*S*)-**C6**, H_2_O_2_, DMBA, HFIP; (IV) PCC, DCM; (V) Triethyl phosphonoacetate, NaH, THF; (VI) Catalyst (*S,S*)-**C6**, H_2_O_2_, DMBA, HFIP; (VII) NaClO_2_, NaH_2_PO_4_, 2-mehyl-2-butene, *^t^* BuOH/H_2_O; (VIII) Cu­(OTf)_2_; Cu­(MeCN)_4_BF_4_; TBAF­(*^t^* BuOH)_4_; MeCN, purple LED. rsm, recovered starting material.

### Functional Group Elaboration and Iterative Oxidation of Methyl Groups

We reasoned that the exquisite chemoselective oxidation displayed by the catalysts may enable recognition of the second methyl site as a point for elaboration. This feature will enable rapid expansion of molecular complexity (Figure 5B).

To investigate this direction, (*R*)-**18a**, prepared from biaryl **18** using catalyst (*R*,*R*)-**C6** (path I), was converted into the corresponding alkyl bromide (*R*)-**40** in 88% yield and 95% ee (path II). No oxidation product was formed when biaryl (*R*)-**40** was reacted with the (*R*,*R*)-**C6** catalyst. In contrast, with the (*S*,*S*)-**C6** catalyst, exclusive formation of alcohol (*R*)-**40a** was formed in 60% isolated yield and >99% ee (path III). Interestingly, owing to the combined electronic and steric deactivation, the benzyl bromide group in compound (*R*)-**40** remained unoxidized, emphasizing the exquisite chemoselectivity of the catalyst. Furthermore, PCC oxidation of (*R*)-**18a** delivered the corresponding aldehyde (*R*)-**18b** (path IV), which was subsequently converted into the (E)-ethyl acrylate derivative (*R*)-**41** (path V). Gratifyingly, oxidation of (*R*)-**41** with catalyst (*S,S*)-**C6** furnished the corresponding primary alcohol (*S*)-**41a** in 65% isolated yield and >99% ee, with no detectable epoxidation of the conjugated double bond (path VI). These two paths are paradigmatic examples of kinetic resolution reactions, in which the catalyst’s chirality governs the selective functionalization of a single face of the biaryl framework.

Motivated by the potential for straightforward use of carboxylic acid directed reactions, (*R*)-**18b** was converted into the corresponding carboxylic acid (*R*)-**18c** (path VII) and subjected to the decarboxylative fluorination photoirradiation conditions recently reported by Ritter and co-workers.^82,83^ Surprisingly, selective hydroxylation at the remote C-4 position took place, affording phenol (*S*)-**42** in 66% yield and 96% ee, presumably, via an initial dearomatizative spirolactonization intermediate **i**, followed by nucleophilic attack of water at the C-4 and rearomatization (path VIII). This result is particularly noteworthy for providing facile access to axially chiral, multifunctionalized biaryl carboxyphenols, a valuable core that resembles those found first generation biphenyl-based antihypertensives,^84^ and could serve as a versatile building block for exploring uncharted chemical space in atropisomeric drug discovery.^85^

### Mechanistic Studies

Experimental and computational studies were performed to elucidate the mechanism and the origin of the enantioselectivity. C–H bond oxygenation mediated by this class of catalysts proceeds through a well-established radical mechanism, involving hydrogen atom transfer (HAT) from the substrate C–H bond to a high-valent manganese-oxo species followed by a fast OH rebound (Figure 1A).^4–7^ In agreement with this scenario, oxidation of *N*-(4′-(*tert*-butyl)-2′,6′-bis­(methyl-d_2_)-[1,1′-biphenyl]-2-yl)­pivalamide (**43**) provided an intramolecular kinetic isotope effect (KIE) of 2.7, suggesting that C–H bond cleavage constitutes the rate-determining step (Figure 6A).

**Figure 6:**
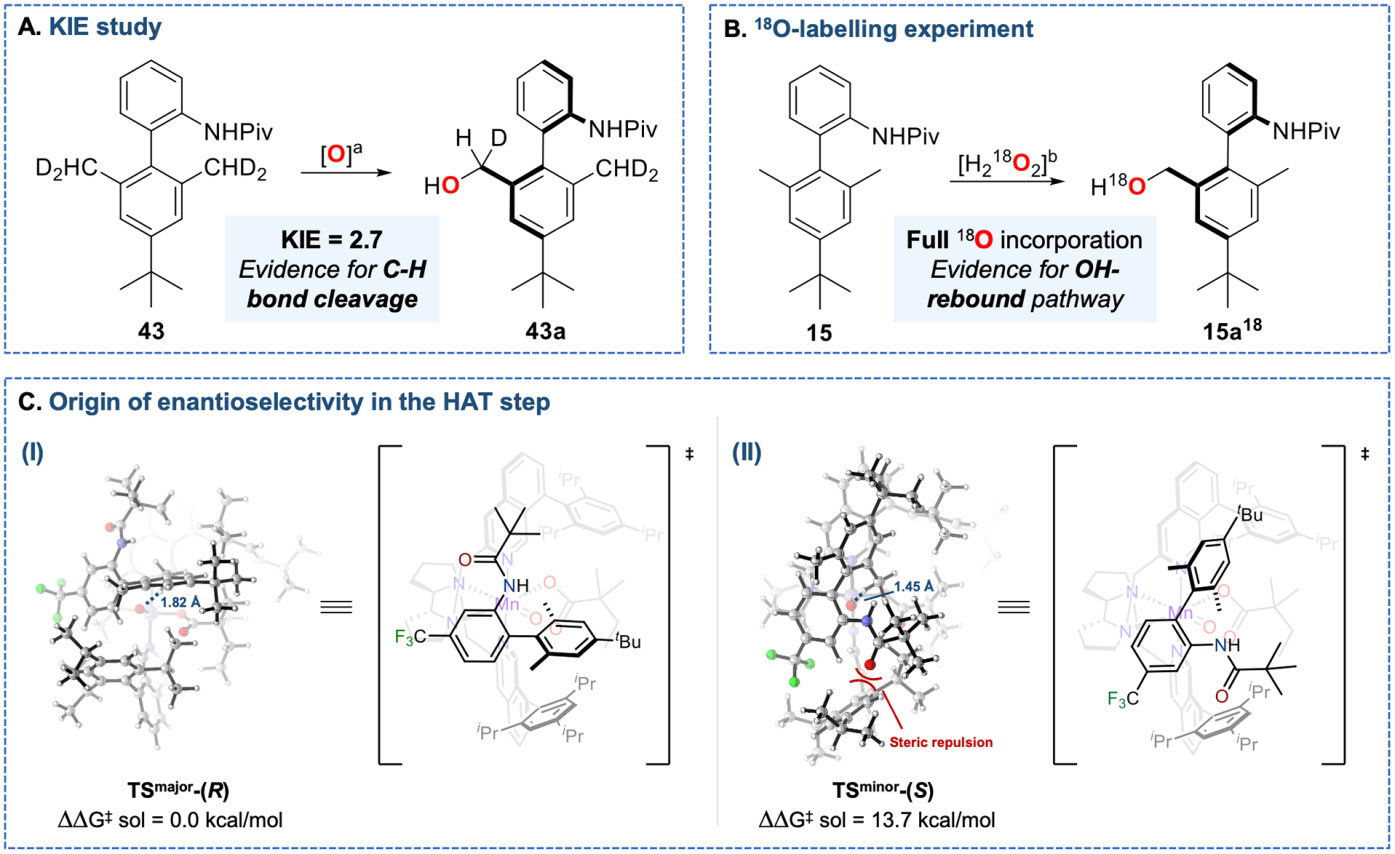
Experimental and computational studies. (A) Kinetic Isotope Effect (KIE) study. *^a^* Catalyst **C6** (3 mol %), DMBA (1.3 equiv) H_2_O_2_ (0.5 equiv), HFIP, 0 °C, 30 min. Full details are reported in the Supporting Information. (B) H_2_^18^O_2_ labeling experiment. *^b^* Catalyst **C6** (3 mol %), DMBA (1.3 equiv), H_2_^18^O_2_ (0.1 equiv), HFIP, 0 °C, 30 min. Full details are reported in the Supporting Information. (C) DFT-calculated TS structures that lead to the major (**TS_major_-(** *R* **))** and (**TS_major_-(** *S* **))** enantiomers of product **28a** (details are provided in the Supporting Information).

Furthermore, oxidation of **15** with labeled H_2_^18^O_2_ provided insight into the OH rebound pathway: the ^18^O label was quantitatively retained in the resulting alcohol, confirming that the hydroxyl group is transferred from the manganese catalyst after the first HAT process (Figure 6B).

To gain further insight into the origins of the exceptional atroposelectivity observed in the oxidation of 2,6-dimethyl-1,1′-biaryl substrates, density functional theory (DFT) calculations were performed on the enantiodetermining C–H bond cleavage step using the catalytically active (*R*,*R*)-[Mn^V^O­(ODMBA)­(^*i*Pr^iQdp]^2+^ oxidant and substrate **28**. The transition state leading to the major enantiomer (**TS** ^**major**^ **-(** *R* **)**) is 13.7 kcal mol^–1^ lower in energy than that leading to the minor enantiomer (**TS** ^**minor**^ **-(** *S* **)**), fully accounting for the experimentally observed >99% ee (ΔΔG^‡^ > 2.5 kcal mol^–1^) for (*R)*-**27a** (Figure 6C–I). In **TS** ^**major**^ **-(** *R* **)**, substrate **28** nests perfectly within the chiral catalyst cavity with minimal distortion, positioning the bulky pivalamide group in a region with low steric congestion (NW quadrant of the V_bur_ steric map, see Figure S1 in the Supporting Information). In contrast, **TS** ^**minor**^ **-(** *S* **)** is destabilized by steric clash between the pivalamide group and the 2,4,6-triisopropylphenyl group at C8 of the isoquinoline (Figure 6C–II, red curves). Overall, these results indicate that the exceptional enantioselectivity arises from the precise structural complementarity between the substrate and the catalyst’s chiral cavity, with unfavorable steric interactions destabilizing the minor pathway.

## Conclusions

On closing, the work describes the development of a non-directed atroposelective C­(sp^3^)–H bond oxidation of 2,6-dimethyl-1,1′-biaryls catalyzed by a novel Mn­(OTf)_2_(^iPr^iQdp) catalyst. The methodology pioneers the use of enantioselective C­(sp^3^)–H bond oxidation as a tool for installing axial chirality in multifunctionalized biaryl moieties, a useful chemical motif across multiple fields. The undirected nature of the reaction makes it agnostic to a specific functionality. This aspect, combined with the use of a HAT/radical recombination reaction as a chirality-defining element, endows the reaction with an unusually broad scope. Furthermore, iterative chemoselective oxidation of C­(sp^3^)–H bonds enable rapidly building of molecular complexity and the expansion of accessible chemical space. Further development of the reaction’s potential to access heterocyclic and polycyclic structures is envisioned. Finally, the use of catalysts based on earth-abundant metals and hydrogen peroxide as oxidant makes the reaction readily suitable for large-scale applications with minimum environmental impact.

## Supplementary Material



## Notes

Experimental procedures for the preparation of metal complexes and substrates, catalytic reactions, details on the isolation and characterization of the reaction products, computational data, HPLC and GC traces, and NMR spectra (PDF)
