# Supplementary material for: Installing Axial Chirality by Atroposelective C(sp3)–H Bond Oxidation
Source: J Am Chem Soc. 2026 May 12;148(20):21080–91. doi: 10.1021/jacs.6c06123 (PMC13220272; doi:10.1021/jacs.6c06123)
Supplement: Supplementary file 1 [file ja6c06123_si_001.pdf]

Supporting Information for

Installing Axial Chirality by Atroposelective C(sp^3)–H Bond Oxidation

Margarida Borrell,[1,≠] Marco Galeotti, [1,≠] Laia Vicens,[1,≠] Tomer Mintz,[2] Arnau Call,[1] Doron Pappo,[2,*] Miquel Costas[1,*]

[1]Institut de Química Computacional i Catàlisi (IQCC) and Departament de Química, Universitat de Girona; Campus Montilivi, 17071 Girona, Catalonia, Spain.
[2]Department of Chemistry, Ben-Gurion University of the Negev, Beer-Sheva 8410501, Israel

*Corresponding author. Email: miquel.costas@udg.edu, pappod@bgu.ac.il

[≠]These authors contributed equally to this work

Table of content

1. Materials and methods **3**
1.1. Materials 3
1.2. Instrumentation 3
2. Synthesis of the catalysts **5**
2.1. Synthesis of the ligands 5
2.2. Synthesis of the complexes 8
3. Synthesis of the substrates **9**
3.1. General procedures 9
3.2. Synthesis of 5-(*tert*-butyl)-1,3-dimethylbenzene derivatives 11
3.3. Synthesis and characterization data 13
3.4. Non-reactive substrates 46
4. Catalytic studies **53**
4.1. General procedure for catalytic oxidations 53
4.2. General procedure for isolation of the oxidized products 53
4.3. General procedure for oxidative work-up with PCC 53
4.4. General procedure for reductive work-up with $NaBH_4$ 54
5. Additional experiments **55**
6. Characterization of the isolated products **56**
6.1. Product scope 56
6.2. Product elaboration 70
7. KIE experiment **75**
8. Labelling experiment **77**
9. X-ray Structures **79**
9.1. Solid structures of manganese catalysts 79
9.2. Solid structures of the atropoisomeric products 83
10. Computational studies **101**
10.1. Buried volume analysis of Mn-oxo species 101
10.2. Computational details for DFT calculations 104
10.3. Atomic coordinates of DFT-calculated structures 106
11. References **134**
11.1. NMR spectra of the ligands 138
11.2. NMR spectra of the substrates 143
11.3. NMR spectra of the isolated products 236
12. SFC traces of the isolated products **292**

1. Materials and methods

1.1. Materials

All commercially available reagents, substrates and solvents were purchased by Sigma Aldrich, Fluorochem, Scharlab, TCI, BLD Pharmatech, Fisher Scientific or VWR, and were used as received unless otherwise stated. 1,1,1,3,3,3-Hexafluoro-2-propanol (99% from Fluorochem) was employed for oxidation reactions.

Anhydrous solvents were either bought from Thermo Scientific or Sigma Aldrich (stored under inert atmosphere and with molecular sieves) or purified and dried by passing through an activated alumina purification system (M-Braun SPS-800).

Note: names for the non-commercially available compounds are those generated by ChemDraw Professional 21.0 software (Perkin Elmer), following the IUPAC nomenclature.

1.2. Instrumentation

NMR spectra were recorded at 400 MHz, using Bruker Ultrashield ASCEND Nanobay or Bruker 400 MHz Advance III HD spectrometers for ^{1}H, ^{13}C and ^{19}F acquisitions. All NMR spectra were recorded at 25ºC unless otherwise stated. Chemical shifts (d) are reported in parts per million (ppm) and referenced to the residual proton solvent peaks for ^{1}H and ^{13}C. Coupling constansts (*J*) are given in Hz and refer to apparent multiplicities (s = singlet, d = doublet, t = triplet, quint = quintuplet, br = broad signal, dd = doublet of doublets, etc.).

High Resolution mass spectrometry (HRMS) was performed on a Bruker Elute-Compact UHPLC-MS instrument with an electrospray ionization source (ESI) and a quadrupole analyzer. Samples were introduced into the mass spectrometer ion source by direct infusion through a syringe pump and were externally calibrate using sodium formate.

GC analyses were carried out on an Agilent 7820A or Agilent 8860 gas chromatograph (HP5 column, 30 m x 0.32 mm, 0.25 mm, Agilent J&W) with a flame ionization detector. GC-MS spectral analyses were performed on an Agilent 7890A gas chromatograph (HP-5MS column, 30 m x 0.25 mm, 0.25 mm, Agilent J&W) interfaced with an Agilent 5975X mass spectrometer with a triple-axis detector. NH_3 was used as the ionization gas.

Thin layer chromatography (TLC) was performed using VWR F254 fluorescent treated silica, which was visualized under UV light.

X-ray diffraction analyses were carried out on a Bruker D8 QUEST ECCO diffractometer using graphite-monochromated Mo Ka radiation (l = 0.71 Å) from an X-ray tube.

Optical rotations ($[a]_D^{25}$) were recorded at room temperature (25ºC) using a Jasco P-2000 iRM-800 polarimeter (l = 589 nm, sodium lamp). Concentration is expressed in g/100 mL. The cell was 10 cm long with 1 mL, 2 mL or 5 mL of capacity.

Chromatographic resolution of enantiomers was performed on an Agilent 1260 Infinity II SFC system using CHIRALPAK-IA, CHIRALPAK-IB-N, CHIRALPAK-IC, CHIRALPAK-IG (100

x 3 mm, 3 mm, Daicel Corporation Chiral) or CHIRALPAK-IJ (150 x 3 mm, 3 mm, Daicel Corporation Chiral) columns. Enantiomers were identified by comparison of those observed in racemic samples prepared using standard catalysis procedures using racemic catalysts.

Column chromatography was carried out using SilicaFlash P60 (230-400, mesh SiliCycle) silica gel. Automated flash chromatography was performed ona Biotage Isolera One.

IR spectra were recorded neat as a thin film on a Bruker Alpha FT-IR spectrometer using a MKII Golden Gate single reflection ATR system. Selected absorption maxima (n_{max}) are reported in wavenumbers (cm^{-1}).

Elemental analyses of C, H and N were performed on a PerkinElmer CHNS-O EA2400 series II elemental analyzer.

Photochemical reaction was carried out using one purple LED (Kessil PR160-390 nm LED).

2. Synthesis of the catalysts

2.1. Synthesis of the ligands

^{TIPS}pdp,[1] ^{TIPS}ecp,[2] ^{CF3}ebdp,[3] ^{iPr}ebdp[3] and ^{iPr}iQdp[4] were prepared according to reported procedures. **L1** (^{Me}ebdp) was synthesized according to the procedures reported in this section.

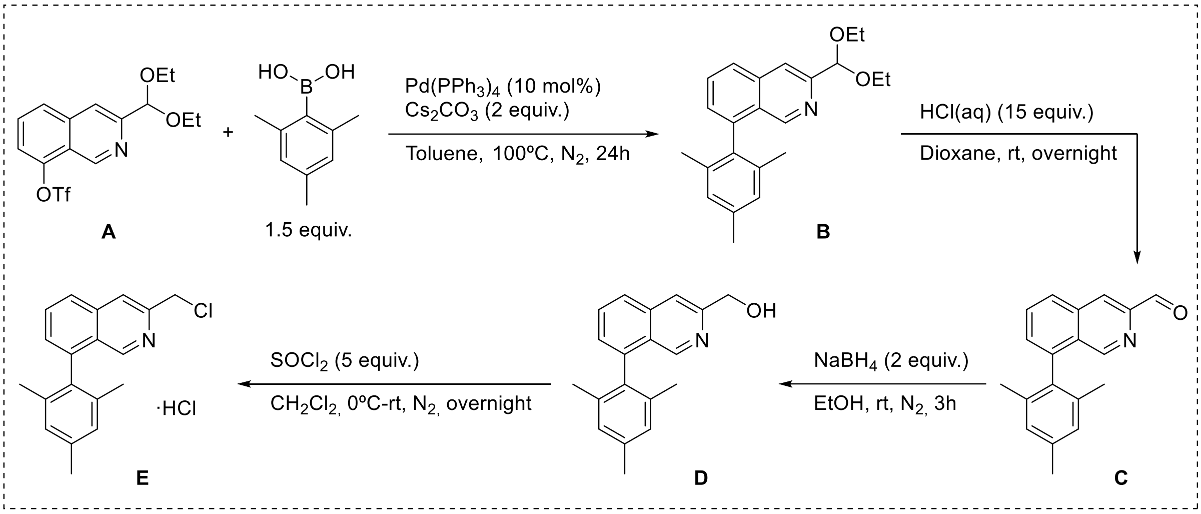

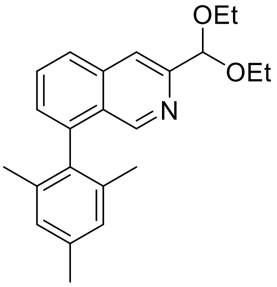

3-(diethoxymethyl)-8-mesitylisoquinoline (B). It was prepared according to a modified reported procedure.[4] A Schlenk tube containing a magnetic stir bar was charged with **A**[4] (354 mg, 0.941 mmol, 1 equiv.), mesityl boronic acid (244 mg, 1.41 mmol, 1.5 equiv.), $Pd(PPh_3)_4$ (111 mg, 0.09941 mmol, 10 mol%) and Cs_2CO_3 (618 mg, 1.88 mmol, 2 equiv.) and dissolved in toluene (3.8 mL, 0.25 M) under N_2 atmosphere. The reaction was stirred at room temperature for 10 minutes and at 100ºC for 24h. The reaction was cooled to room temperature, quenched with H_2O (10 mL) and diluted with EtOAc (10 mL). The phases were separated and the product was extracted with Et_2O (3x10 mL). The combined organic phases were washed with brine (10 mL) and water (10 mL), dried over anhydrous $MgSO_4$, filtered and the solvent was evaporated under reduced pressure. The crude was purified by column chromatography over silica gel (hexane:EtOAc, 95:5) to afford the product as a colorless solid (300 mg, 0.858 mmol, 91% yield). ^{1}H-NMR (400 MHz, $CDCl_3$) δ, ppm: 8.76 (s, 1H), 7.99 (s, 1H), 7.86 (dt, *J* = 8.3, 1.1 Hz, 1H), 7.74 (dd, *J* = 8.3, 7.1 Hz, 1H), 7.35 (dd, *J* = 7.1, 1.1 Hz, 1H), 7.00 (s, 2H), 5.66 (s, 1H), 3.82-3.64 (m, 4H), 2.38 (s, 3H), 1.86 (s, 6H), 1.29 (t, *J* = 7.1 Hz, 6H). ^{13}C-NMR (100 MHz, $CDCl_3$) δ, ppm: 151.1, 150.6, 140.0, 137.6, 136.8, 136.7, 134.7, 130.6, 128.6, 128.4, 127.0, 126.4, 118.0, 102.7, 62.5, 21.3, 20.6, 15.4. HRMS (ESI-MS) *m/z* calculated for $C_{23}H_{27}NO_2$ $[M+H]^+$ 350.2115, found 350.2112.

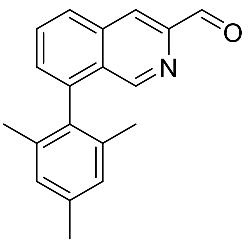

8-mesitylisoquinoline-3-carbaldehyde (C). It was prepared according to a modified reported procedure.[4] **B** (300 mg, 0.858 mmol, 1 equiv.) was dissolved in a 1:6 mixture of 1,4-dioxane (3.5 mL) and aqueous 2M HCl (21 mL) and the solution was stirred at room temperature overnight. The mixture was quenched with saturated $NaHCO_3$ until pH = 10 and the product was extracted with EtOAc (3 x 50 mL). The combined organic

phases were dried over anhydrous $MgSO_4$, filtered and the solvent was evaporated under reduced pressure to obtain the product as a pale yellow solid (170 mg, 0.617 mmol, 72% yield), that was used in the next step without further purification. ^{1}H-NMR (400 MHz, $CDCl_3$) δ, ppm: 10.25 (s, 1H), 8.90 (s, 1H), 8.45 (d, *J* = 1.1 Hz, 1H), 8.03 (dt, *J* = 8.3, 1.1 Hz, 1H), 7.86 (dd, *J* = 8.3, 7.1 Hz, 1H), 7.54 (dd, *J* = 7.1, 1.1 Hz, 1H), 7.04 (s, 2H), 2.40 (s, 3H), 1.87 (s, 6H). ^{13}C-NMR (100 MHz, $CDCl_3$) δ, ppm: 193.7, 151.7, 147.0, 140.4, 138.0, 136.6, 135.8, 134.0, 131.5, 131.4, 129.1, 128.6, 127.9, 121.8, 21.3, 20.6. HRMS (ESI-MS) *m/z* calculated for $C_{19}H_{17}NO$ $[M+H]^+$ 276.1383, found 276.1382.

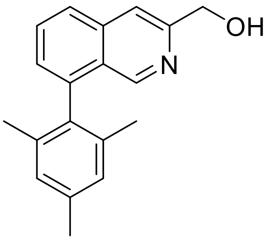

(8-mesitylisoquinolin-3-yl)methanol (D). It was prepared according to a modified reported procedure.[4] Under N_2 atmosphere, **C** (170 mg, 0.617 mmol, 1 equiv.) was dissolved in anhydrous MeOH (4.1 mL, 0.15M) and $NaBH_4$ (47.7 mg, 1.24 mmol, 2 equiv.) was added in small portions. The solution was stirred at room temperature for 3 hours. The solvent was then evaporated under reduced pressure, the crude was solved in H_2O (10 mL) and the product was extracted with CH_2Cl_2 (3 x 15 mL). The combined organic phases were dried over anhydrous $MgSO_4$, filtered and the solvent was evaporated under reduced pressure to obtain the product as a colorless solid (162 mg, 0.584 mmol, 95% yield), that was used in the next step without further purification. ^{1}H-NMR (400 MHz, $CDCl_3$) δ, ppm: 8.75 (s, 1H), 7.82 (d, *J* = 8.5 Hz, 1H), 7.76 (ddd, *J* = 8.5, 6.8, 1.0 Hz, 1H), 7.70 (s, 1H), 7.35 (dt, *J* = 6.8, 1.1 Hz, 1H), 7.02 (s, 2H), 4.91 (s, 2H), 2.39 (s, 3H), 1.87 (s, 6H). ^{13}C-NMR (100 MHz, $CDCl_3$) δ, ppm: 152.5, 150.3, 140.2, 137.7, 137.0, 136.7, 134.6, 131.0, 128.4, 128.3, 126.6, 125.8, 117.3, 64.8, 21.3, 20.5. HRMS (ESI-MS) *m/z* calculated for $C_{19}H_{19}NO$ $[M+H]^+$ 278.1539, found 278.1537.

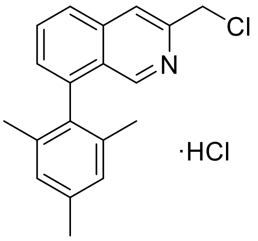

3-(chloromethyl)-8-mesitylisoquinoline hydrochloride (E). It was prepared according to a modified reported procedure.[4] Under N_2 atmosphere, **D** (162 mg, 0.584 mmol, 1 equiv.) was dissolved in anhydrous CH_2Cl_2 (12 mL, 0.05M). At 0ºC, $SOCl_2$ (175 mL, 2.36 mmol, 4 equiv.) was added dropwise and the mixture was stirred at room temperature for 3h. The solvent was then evaporated under reduced pressure to obtain the product as a yellow solid (100 mg, 0.301 mmol, 52% yield). ^{1}H-NMR (400 MHz, CD_3OD) δ, ppm: 8.86 (s, 1H), 8.63 (s, 1H), 8.36-8.30 (m, 2H), 7.83 (dd, *J* = 5.1, 3.2 Hz, 1H), 7.12 (s, 2H), 5.13 (s, 2H), 2.40 (s, 3H), 1.88 (s, 6H). ^{13}C-NMR (100 MHz, $CDCl_3$) δ, ppm: 147.4, 144.2, 142.9, 141.2, 140.2, 138.5, 137.8, 134.0, 133.4, 129.8, 128.3, 127.3, 126.6, 41.9, 21.2, 20.5. HRMS (ESI-MS) *m/z* calculated for $C_{19}H_{18}ClN$ $[M+H]^+$ 296.1201, found 296.1196.

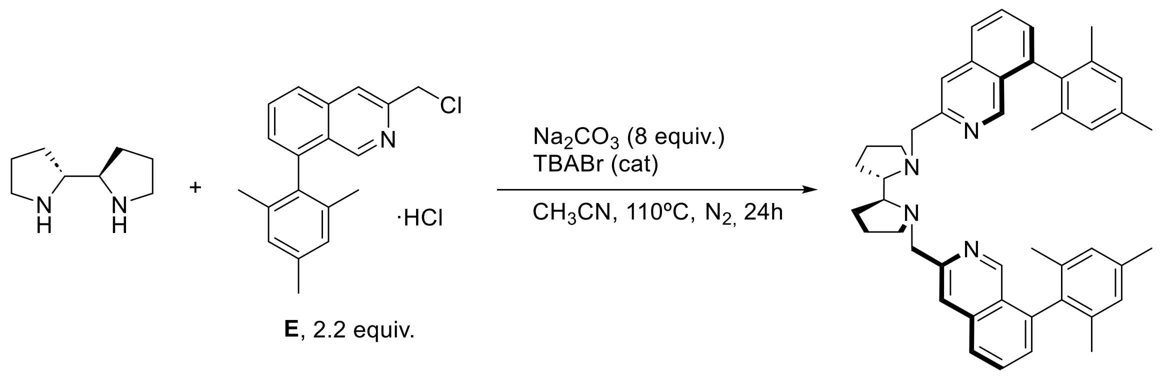

E, 2.2 equiv.

L1

1-((8-mesitylisoquinolin-3-yl)methyl)-1'-((8-mesitylisoquinolin-3-yl)methyl)-2,2'-bipyrrolidine (L1). It was prepared according to a modified reported procedure.[5] In a sealed vial and under N_2 atmosphere, **E** (100 mg, 0.325 mmol, 2.2 equiv.) were dissolved in anhydrous CH_3CN (3 mL, 0.1 M). 2,2'-bispyrrolidine (20.9 mg, 0.148 mmol, 1 equiv.), solid Na_2CO_3 (277 mg, 2.60 mmol, 8 equiv.) and a scoop of TBABr were then added and the solution was heated 24h at 110ºC. The mixture was cooled down to room temperature, the resultant solids were filtered-off and the solvent was removed under reduced pressure. The crude was solved in CH_2Cl_2 (15 mL) and washed with 2M $NaOH_{(aq)}$ (2x10 mL). The combined organic phases were dried over anhydrous $MgSO_4$, filtered and the solvent was evaporated under reduced pressure. The crude was purified by column chromatography over silica gel (CH_2Cl_2:MeOH:NH_3, 95:8:2) to afford the product as a colorless solid (83.0 mg, 0.126 mmol, 39% yield). ^{1}H-NMR (400 MHz, $CDCl_3$) δ, ppm: 8.70 (s, 2H), 7.74 (d, *J* = 8.2 Hz, 4H), 7.67 (dd, *J* = 8.2, 6.8 Hz, 2H), 7.30-7.25 (m, 2H), 6.99 (s, 4H), 4.39 (d, *J* = 14.4 Hz, 2H), 3.65 (d, *J* = 14.4 Hz, 2H), 3.16 (s, 2H), 2.93 (s, 2H), 2.38 (s, 6H), 2.35-2.25 (m, 2H), 1.92-1.87 (m, 2H), 1.85 (s, 12H), 1.81-1.65 (m, 6H). ^{13}C-NMR (100 MHz, $CDCl_3$) δ, ppm: 150.4, 139.7, 137.3, 136.8, 136.6, 134.8, 130.2, 128.2, 127.6, 126.0, 125.6, 118.7, 110.0, 65.7, 60.9, 55.4, 29.3, 23.5, 21.1, 20.4. HRMS (ESI-MS) *m/z* calculated for $C_{46}H_{50}N_4$ $[M+H]^+$ 659.4108, found 659.4097. $[a]_D^{25}$ = +46.89 (c 0.550, $CHCl_3$).

2.2. Synthesis of the complexes

Mn(^{TIPS}pdp) (**C1**),[2] Mn(^{TIPS}ecp) (**C2**),[2] Mn(^{CF3}ebdp) (**C3**),[3] and Mn(^{iPr}ebdp) (**C4**)[3] were prepared according to reported procedures.

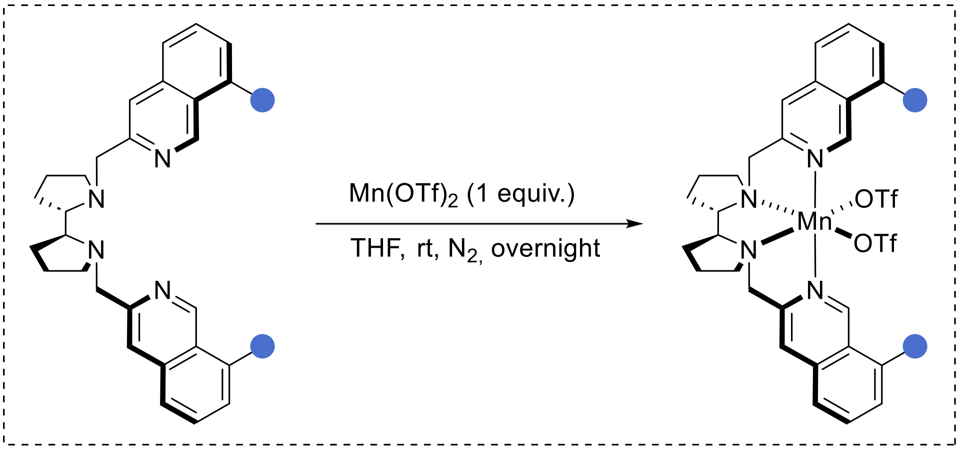

C5 [Mn(^{Me}iQdp)] and **C6** [Mn(^{iPr}iQdp)] were synthesized according to a modified reported procedure.[6] Under N_2 atmosphere, $Mn(OTf)_2$ (1 equiv.) was added to a vigorously stirred solution of the ligand (1 equiv.) in THF (1 mL). The solution was stirred at room temperature overnight and the solvent was then removed under reduced pressure. The crude was dissolved in CH_2Cl_2 (3 mL) and the solution was filtered-off through a celite© plug. The complexes were then purified by crystallization by slow ether diffusion in a CH_2Cl_2 solution.

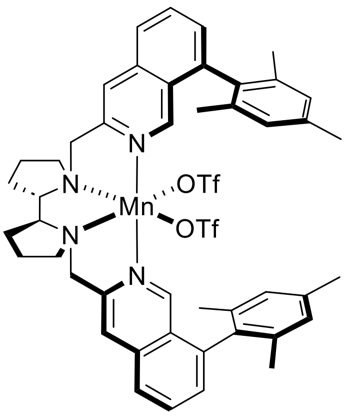

Mn(^{Me}iQdp) (C5). It was prepared according to the procedure using $^{Mes\text{-}IQ}$pdp (83.0 mg, 0.126 mmol, 1 equiv.) and $Mn(OTf)_2$ (44.5 mg, 0.126 mmol, 1 equiv.). Purification by crystallization by slow ether diffusion in a CH_2Cl_2 solution affords the complex as colorless solid (90.3 mg, 0.0892 mmol, 71% yield). HRMS (ESI-MS) *m/z* calculated for $C_{47}H_{50}F_3MnN_4O_3S$ $[M\text{-}OTf]^+$ 862.2931, found 862.2918. Elemental analysis calculated (%) for $C_{48}H_{50}F_6MnN_4O_6S_2$ (MW = 1012.00): C 56.97, H 4.98, N 5.54, found C 56.92, H 5.25, N 5.48. FT-IR(ATR) n, cm^{-1}: 2974-2832 (C-H *sp*^{3}), 1625, 1304, 1208, 1031, 894, 763, 636, 513. XRD structure found in CCDC Number 2519084.

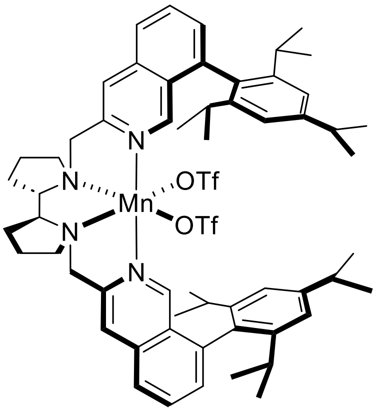

Mn(^{iPr}iQdp) (C6). It was prepared according to the procedure using $^{iPr\text{-}IQ}$pdp (104 mg, 0.126 mmol, 1 equiv.) and $Mn(OTf)_2$ (44.5 mg, 0.126 mmol, 1 equiv.). Purification by crystallization by slow ether diffusion in a CH_2Cl_2 solution affords the complex as colorless solid (129.4 mg, 0.110 mmol, 87% yield). HRMS (ESI-MS) *m/z* calculated for $C_{58}H_{74}MnN_4$ $[M\text{-}2OTf]^{2+}$ 440.7641, found 440.7650. Elemental analysis calculated (%) for $C_{60}H_{74}F_6MnN_4O_6S_2$ (MW = 1180.32): C 61.06, H 6.32, N 4.65, found C 60.52, H 6.61, N 5.06. FT-IR(ATR) n, cm^{-1}: 2982-2843 (C-H *sp*^{3}), 1627, 1310, 1209, 1164, 1029, 760, 636, 514. XRD structure found in CCDC Number 2519089.

3. Synthesis of the substrates

3.1. General procedures

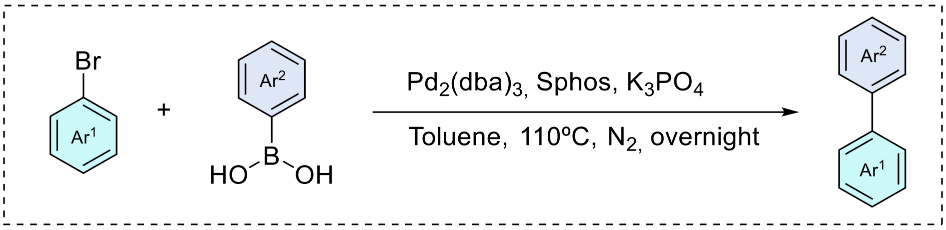

General procedure A for cross-coupling reactions. It was adapted from reported procedures.[7, 8] A Schlenk tube containing a magnetic stir bar was charged with aryl halide, aryl boronic acid, $Pd_2(dba)_3$, SPhos and K_3PO_4 and dissolved in anhydrous toluene under N_2 atmosphere. The reaction was stirred at 110ºC overnight. The reaction was quenched with H_2O and diluted with EtOAc. The phases were separated and the product was extracted with EtOAc (x3). The organic phases were washed with brine, dried over anhydrous $MgSO_4$, filtered and the solvent was evaporated under reduced pressure. The crude was purified by column chromatography over silica gel.

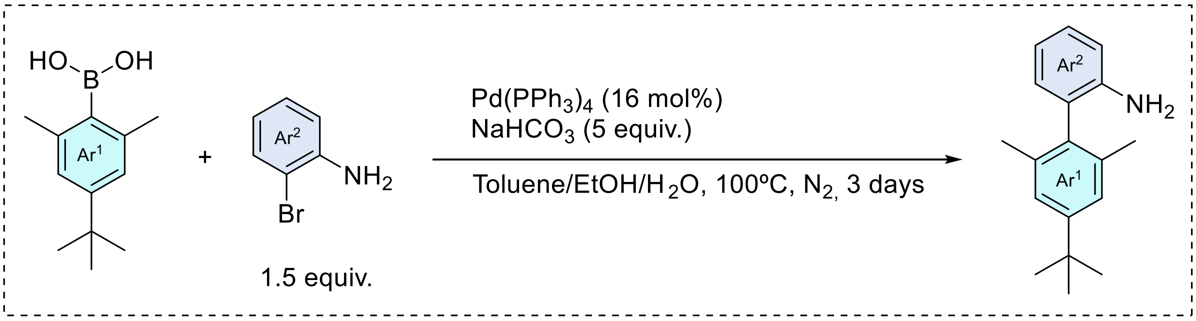

General procedure B for cross-coupling reactions. It was adapted from a reported procedure.[9] A Schlenk tube containing a magnetic stir bar was charged with (4-(*tert*-butyl)-2,6-dimethylphenyl)boronic acid (1 equiv.), the substituted 2-bromoaniline (1.5 equiv.), $Pd(PPh_3)_4$ (16 mol%) and $NaHCO_3$ (5 equiv.) were dissolved in a mixture of toluene/EtOH/H_2O (1:2:1 v/v, 0.08 M) under N_2 atmosphere. The reaction was stirred at 100ºC for 3 days. The reaction was cooled to room temperature and H_2O was added in the mixture. The product was extracted with EtOAc (x3). The organic phases were dried over anhydrous $MgSO_4$, filtered and the solvent was evaporated under reduced pressure. The crude was purified by column chromatography over silica gel. Note: EtOH and H_2O were degassed with a N_2 flux for several minutes before adding to the reaction

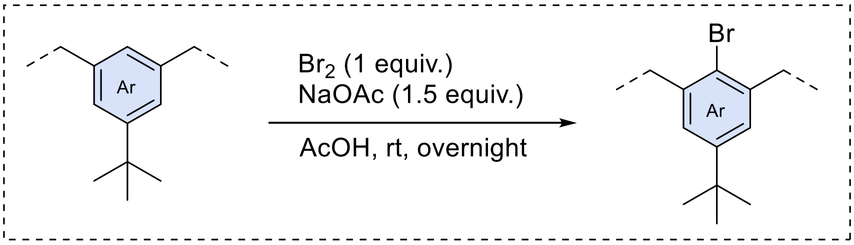

General procedure C for the bromination of arenes. It was adapted from a reported procedure.[10] A solution of bromine (1 equiv.) in glacial acetic acid (0.5 M) was added dropwise to a solution of arene (1 equiv.) and NaOAc (1.5 equiv.). The mixture was stirred at room

temperature overnight and the solvent was then removed under reduced pressure. H_2O was added to the residue and the product was extracted with EtOAc (x3). The organic phases were washed with saturated $NaHCO_3$, dried over anhydrous $MgSO_4$, filtered and the solvent was evaporated under reduced pressure. The crude was purified either by column chromatography over silica gel or by recrystallization from absolute EtOH.

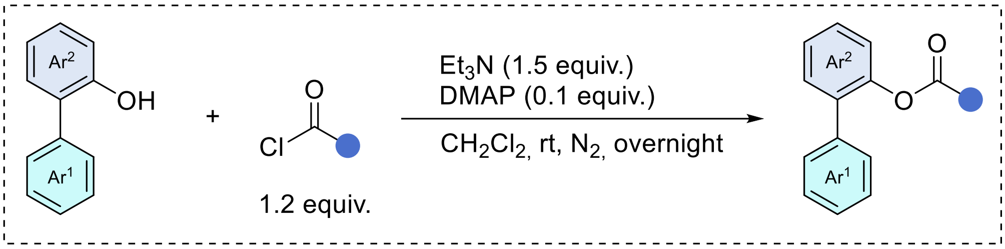

General procedure D for the synthesis of esters. It was adapted from a reported procedure.[11] Under N_2 atmosphere, the alcohol (1 equiv.) was dissolved in anhydrous CH_2Cl_2 (0.2 M). Et_3N (1.2 equiv.), DMAP (0.1 equiv.) and the corresponding acyl chloride (1.2 equiv.) were added to the solution and the reaction was stirred at room temperature overnight. The reaction was quenched with 2M $HCl_{(aq)}$ and the layers were separated. The product was extracted with CH_2Cl_2 (x3). The combined organic layers were washed with saturated $NaHCO_3$ solution, dried over anhydrous $MgSO_4$, filtered and the solvent was evaporated under reduced pressure. The crude was purified by column chromatography over silica gel.

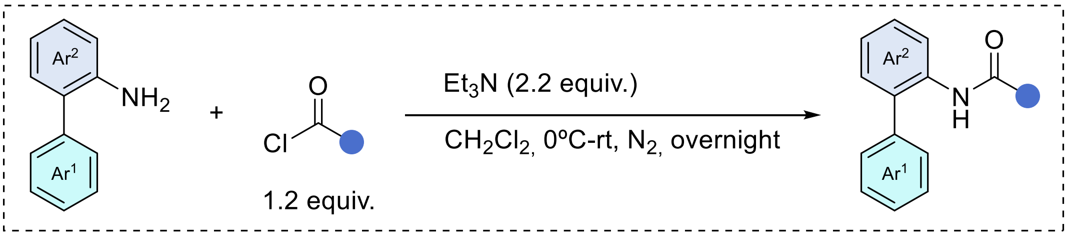

General procedure E for the synthesis of secondary amides. It was adapted from reported procedures.[12, 13] Under N_2 atmosphere, the amine (1 equiv.) was dissolved in anhydrous CH_2Cl_2 (0.2 M) and cooled to 0ºC. Et_3N (2.2 equiv.) and the corresponding acyl chloride (1.2 equiv.) were added to the solution and the reaction was stirred at room temperature overnight. The reaction was quenched with 2M $HCl_{(aq)}$ and the layers were separated. The product was extracted with CH_2Cl_2 (x3). The combined organic layers were washed with saturated $NaHCO_3$ solution, dried over anhydrous $MgSO_4$, filtered and the solvent was evaporated under reduced pressure. The crude was purified by column chromatography over silica gel.

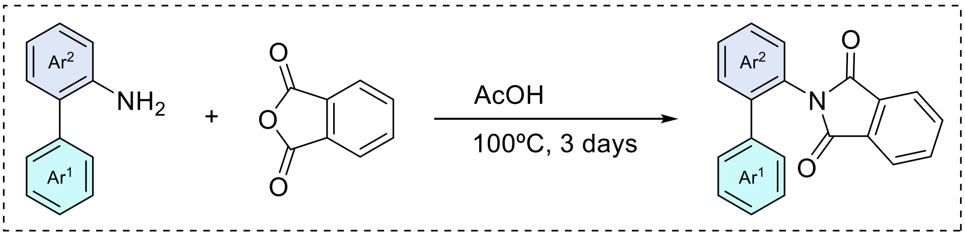

General procedure F for the synthesis of phthalimides. It was adapted from a reported procedure. It was prepared according to a modified reported procedure.[14] In a crimped vial, the amine (1 equiv.) and phtalic anhydride (1 equiv.) were dissolved in AcOH (0.5 M) and the mixture was stirred at 100ºC for 3 days. The reaction was cooled to room temperature and quenched with H_2O. The product was extracted with EtOAc (x3), and the combined organic

layers were washed with saturated $NaHCO_3$ solution and brine, dried over anhydrous $MgSO_4$, filtered and the solvent was evaporated under reduced pressure. The crude was purified by column chromatography over silica gel.

3.2. Synthesis of 5-(*tert*-butyl)-1,3-dimethylbenzene derivatives

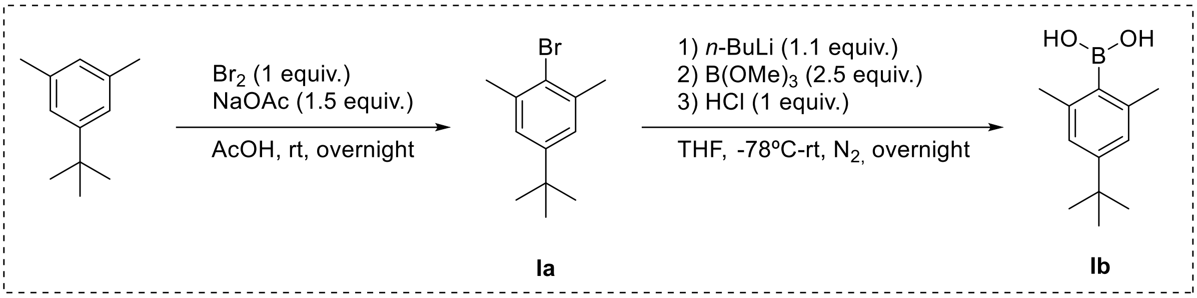

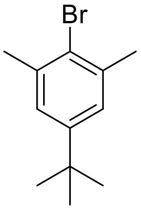

2-bromo-5-(*tert*-butyl)-1,3-dimethylbenzene (Ia). It was prepared according to the General Procedure C using 1-(*tert*-butyl)-3,5-dimethylbenzene (13.0 g, 78.5 mmol, 1 equiv.), Br_2 (12.5 g, 78.5 mmol, 1 equiv.) and NaOAc (117 mmol, 9.69 mmol, 1.5 equiv.) in AcOH (350 mL). The crude was purified by recrystallization from EtOH to afford the product as a colorless solid (15.0 g, 62.2 mmol, 79% yield). Spectral data match those reported in literature.[15] ^{1}H-NMR (400 MHz, $CDCl_3$) δ, ppm: 7.20 (s, 2H), 2.52 (s, 6H), 1.40 (s, 9H). ^{13}C-NMR (100 MHz, $CDCl_3$) δ, ppm: 149.8, 137.7, 125.6, 124.5, 34.4, 31.4, 24.2. GC-MS (CI) *m/z* for $C_{12}H_{17}Br$ $[M+H]^+$ 240.0. TLC (SiO_2, hexane), R_f = 0.84

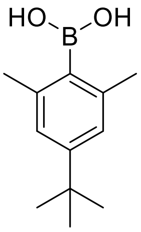

(4-(*tert*-butyl)-2,6-dimethylphenyl)boronic acid. It was prepared according to a modified reported procedure.[15] **Ia** (5.00 g, 20.7 mmol, 1 equiv.) was dissolved in anhydrous THF (47 mL, 0.44 M) and cooled to -78ºC. *n*-BuLi (2.5M in hexane, 9.1 mL, 22.8 mmol, 1.1 equiv.) was added dropwise and the reaction was stirred for 2h at this temperature. Then, $B(OMe)_3$ (5.90 mL, 51.8 mmol, 2.5 equiv.) was added dropwise and the reaction mixture was stirred for 2h at -78ºC and at room temperature overnight. After this time, 1M $HCl_{(aq)}$ (20.7 mL, 20.7 mmol, 1 equiv.) was added to the solution and the mixture was stirred at room temperature for 5h. The product was then extracted with CH_2Cl_2 (3x100 mL). The combined organic layers were washed with water, dried over anhydrous $MgSO_4$, filtered and the solvent was evaporated under reduced pressure. The crude was purified by column chromatography over silica gel (hexane:EtOAc, 100:0 to 80:20) to afford the product as a colorless solid (2.24 g, 10.9 mmol, 53% yield). Spectral data match those reported in literature.[15] ^{1}H-NMR (400 MHz, $CDCl_3$) δ, ppm: 7.03 (s, 2H), 4.56 (s, 2H), 2.39 (s, 6H), 1.30 (s, 9H). HRMS (ESI-MS) *m/z* calculated for $C_{12}H_{19}BO_2$ $[M-H]^-$ 251.1463, found 251.1462. TLC (SiO_2, hexane:EtOAc 8:2), R_f = 0.32.

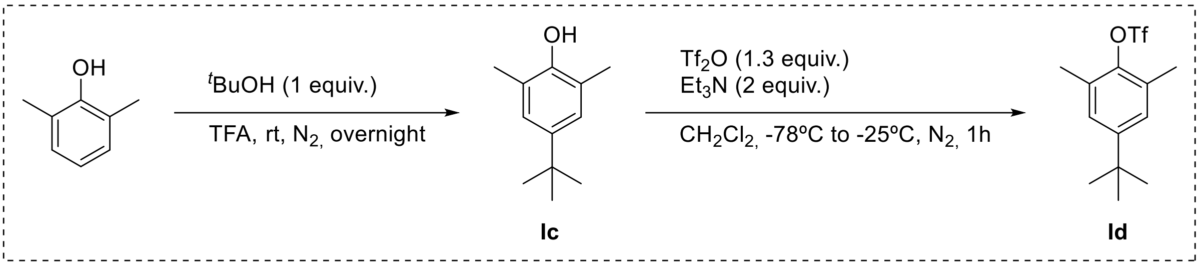

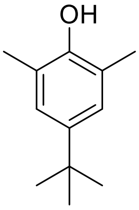

4-(*tert*-butyl)-2,6-dimethylphenol (Ic). It was prepared according to a modified reported procedure.[16] Under N_2 atmosphere, 2,6-dimethylphenol (1.75 g, 14.2 mmol, 1 equiv.) and *tert*-butanol (1.10 mL, 14.2 mmol, 1 equiv.) were dissolved in trifluoroacetic acid (18 mL, 0.8 M) and the mixture was stirred at room temperature overnight. The reaction was quenched with H_2O and the product was extracted with CH_2Cl_2 (x3). The organic layers were washed with saturated $NaHCO_3$ solution, dried over anhydrous $MgSO_4$, filtered and the solvent was evaporated under reduced pressure. The product was obtained as a colorless solid (1.75 g, 9.82 mmol, 69% yield) and was used in the next step without further purification. Spectral data match those reported in literature.[17] ^{1}H-NMR (400 MHz, $CDCl_3$) δ, ppm: 7.00 (s, 2H), 4.48 (br, 1H), 2.25 (s, 6H), 1.29 (s, 9H). ^{13}C-NMR (100 MHz, $CDCl_3$) δ, ppm: 150.0, 143.0, 125.7, 122.4, 34.0, 31.7, 16.3. HRMS (ESI-MS) *m/z* calculated for $C_{12}H_{18}O$ $[M-H]^-$ 177.1285, found 177.1285. TLC (SiO_2, hexane:EtOAc 9:1), R_f = 0.49.

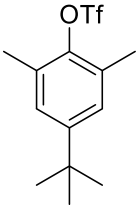

4-(*tert*-butyl)-2,6-dimethylphenyl trifluoromethanesulfonate (Id). It was prepared according to a modified reported procedure.[18] Under N_2 atmosphere, **Ic** (1.75 g, 9.82 mmol, 1 equiv.) was dissolved in anhydrous CH_2Cl_2 (20 mL, 0.5 M) and Et_3N (2.80 mL, 19.6 mmol, 2 equiv.) was added. The mixture was cooled to -78ºC and a solution of Tf_2O (2.19 mL, 11.9 mmol, 1.3 equiv.) in anhydrous CH_2Cl_2 (3.5 mL, 3.6 M) was carefully added. The mixture was stirred at -25ºC for 1 hour. The reaction was quenched with saturated $NaHCO_3$ (20 mL) and the phases were separated. The organic phase was washed with brine, dried over anhydrous $MgSO_4$, filtered and the solvent was evaporated under reduced pressure. The crude was purified by column chromatography over silica gel (hexane:EtOAc, 100:0 to 93:7) to afford the product as a colorless oil (1.53 g, 4.93 mmol, 50% yield). ^{1}H-NMR (400 MHz, $CDCl_3$) δ, ppm: 7.11 (s, 2H), 2.38 (s, 6H), 1.30 (s, 9H). ^{13}C-NMR (100 MHz, $CDCl_3$) δ, ppm: 151.1, 144.9, 130.7, 127.1, 118.8 (q, J = 319.7 Hz), 34.5, 31.4, 17.5. ^{19}F-NMR (377 MHz, $CDCl_3$) δ, ppm: -74.6. GC-MS (CI) *m/z* for $C_{13}H_{17}F_3O_3S$ $[M+NH_4]^+$ 328.1. TLC (SiO_2, hexane:EtOAc 9:1), R_f = 0.84.

3.3. Synthesis and characterization data

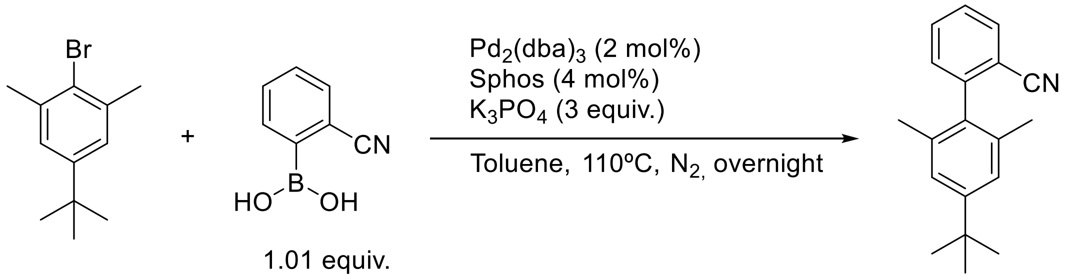

Ia **1**

4'-(*tert*-butyl)-2',6'-dimethyl-[1,1'-biphenyl]-2-carbonitrile (1). It was prepared according to General Procedure A[8] using **Ia** (482 mg, 2.00 mmol, 1 equiv.), (2-cyanophenyl)boronic acid (330 mg, 2.02 mmol, 1.01 equiv.), $Pd_2(dba)_3$ (37.0 mg, 0.040 mmol, 2 mol%), Sphos (34.0 mg, 0.080 mmol, 4 mol%) and K_3PO_4 (1.30 g, 6.00 mmol, 3 equiv.) in toluene (10 mL). The crude was purified by column chromatography over silica gel (hexane:EtOAc, 95:5) to obtain the product as a colorless solid (269 mg, 1.02 mmol, 51% yield). ^{1}H-NMR (400 MHz, $CDCl_3$) δ, ppm: 7.77 (dd, *J* = 7.8, 1.3 Hz, 1H), 7.65 (td, *J* = 7.7, 1.4 Hz, 1H), 7.45 (td, *J* = 7.7, 1.3 Hz, 1H), 7.31 (dd, *J* = 7.8 Hz, 1.2 Hz, 1H), 7.15 (s, 2H), 2.03 (s, 6H), 1.35 (s, 9H). ^{13}C-NMR (100 MHz, $CDCl_3$) δ, ppm: 151.2, 145.7, 135.3, 134.9, 133.1, 132.9, 130.7, 127.4, 124.8, 118.0, 113.3, 34.5, 31.4, 20.7. HRMS (ESI-MS) *m/z* calculated for $C_{19}H_{21}N$ $[M+Na]^+$ 281.2012, found 281.2014. TLC (SiO_2, hexane:EtOAc 95:5), R_f = 0.49.

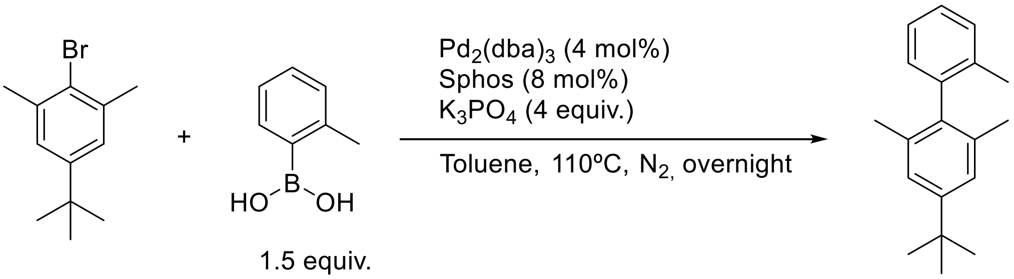

Ia **2**

4-(*tert*-butyl)-2,2',6-trimethyl-1,1'-biphenyl (2). It was prepared according to General Procedure A[7] using **Ia** (355 mg, 1.47 mmol, 1 equiv.), *ortho*-tolyl boronic acid (316 mg, 2.20 mmol, 1.5 equiv.), $Pd_2(dba)_3$ (54.5 mg, 0.059 mmol, 4 mol%), Sphos (49.3 mg, 0.118 mmol, 8 mol%) and K_3PO_4 (1.28 g, 5.88 mmol, 4 equiv.) in toluene (3 mL). The crude was purified by column chromatography over silica gel (hexane:EtOAc, 95:5) to obtain the product as a colorless solid (256 mg, 0.954 mmol, 69% yield). ^{1}H-NMR (400 MHz, $CDCl_3$) δ, ppm: 7.35-7.28 (m, 3H), 7.19 (s, 2H), 7.13-7.09 (m, 1H), 2.05 (s, 3H), 2.03 (s, 6H), 1.43 (s, 9H). ^{13}C-NMR (100 MHz, $CDCl_3$) δ, ppm: 149.5, 140.8, 138.1, 136.0, 135.2, 129.9, 129.2, 126.8, 125.9, 124.2, 34.3, 31.5, 20.6, 19.5. GC-MS (CI) *m/z* for $C_{19}H_{24}$ $[M+H]^+$ 253.1. TLC (SiO_2, hexane), R_f = 0.72.

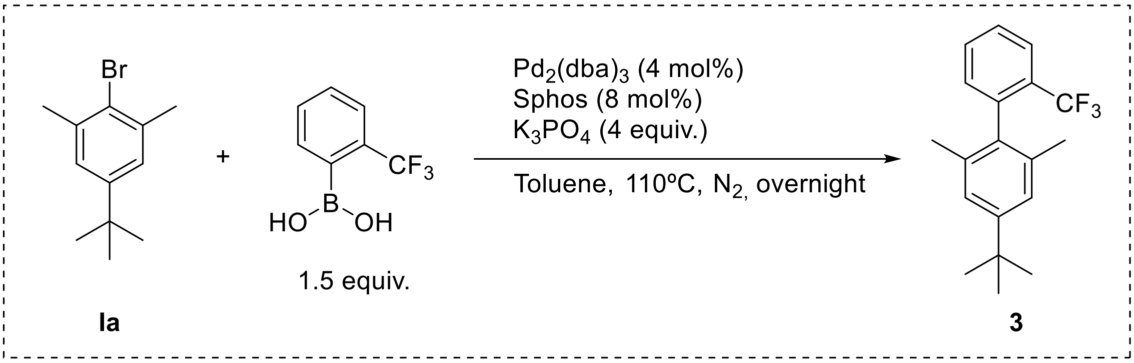

4-(*tert*-butyl)-2,6-dimethyl-2'-(trifluoromethyl)-1,1'-biphenyl (3). It was prepared according to General Procedure A[7] using **Ia** (241 mg, 1.00 mmol, 1 equiv.), (2-(trifluoromethyl)phenyl)boronic acid (285 mg, 1.50 mmol, 1.5 equiv.), $Pd_2(dba)_3$ (37.0 mg, 0.040 mmol, 4 mol%), Sphos (34.0 mg, 0.080 mmol, 8 mol%) and K_3PO_4 (866 mg, 4.00 mmol, 4 equiv.) in toluene (5 mL). The crude was purified by column chromatography over silica gel (hexane) to obtain the product as a colorless solid (222 mg, 0.725 mmol, 73% yield). ^{1}H-NMR (400 MHz, $CDCl_3$) δ, ppm: 7.77 (d, J = 7.7 Hz, 1H), 7.58 (t, J = 7.5 Hz, 1H), 7.46 (t, J = 7.7 Hz, 1H), 7.19 (d, J = 7.5 Hz, 1H), 7.08 (s, 2H), 1.93 (s, 6H), 1.35 (s, 9H). ^{13}C-NMR (100 MHz, $CDCl_3$) δ, ppm: 150.4, 140.5 (q, J = 2.0 Hz), 135.8, 136.0, 132.0, 131.6, 129.0 (q, J = 29.5 Hz), 127.2, 126.4 (q, J = 5.3 Hz), 125.5 (q, J = 274.6 Hz), 124.0, 34.5, 31.6, 20.9. ^{19}F-NMR (377 MHz, $CDCl_3$) δ, ppm: -62.1. GC-MS (CI) m/z for $C_{19}H_{21}F_3$ $[M+NH_4]^+$ 324.1. TLC (SiO_2, hexane), R_f = 0.70.

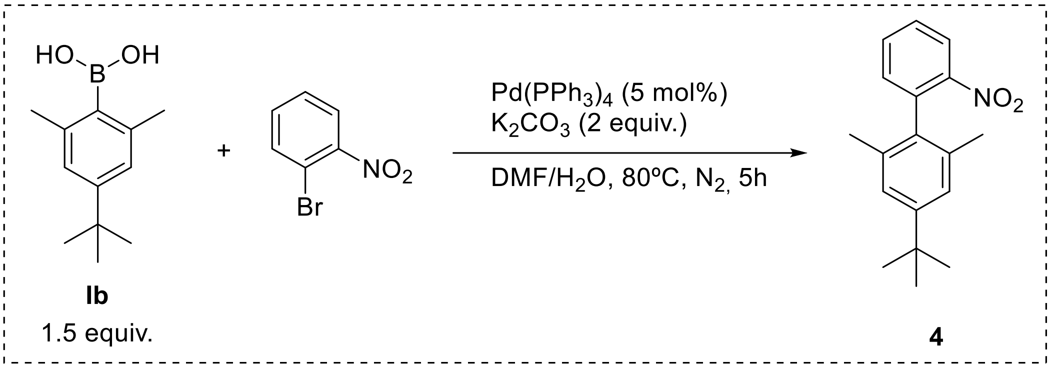

4-(*tert*-butyl)-2,6-dimethyl-2'-nitro-1,1'-biphenyl (4). It was prepared according to a modified reported procedure.[19] A Schlenk tube containing a magnetic stir bar was charged with 1-bromo-2-nitrobenzene (206 mg, 1.00 mmol, 1 equiv.), **Ib** (309 mg, 1.50 mmol, 1.5 equiv.), $Pd(PPh_3)_4$ (59.0 mg, 0.050 mmol, 5 mol%) and K_2CO_3 (276 mg, 2.00 mmol, 2 equiv.) and dissolved in DMF/H_2O (5:1 v/v, 4 mL, 0.25 M) under N_2 atmosphere. The reaction was stirred at 80ºC for 5h. The reaction was quenched with H_2O (15 mL) and diluted with EtOAc (10 mL). The phases were separated and the product was extracted with EtOAc (3x10 mL). The combined organic phases were dried over anhydrous $MgSO_4$, filtered and the solvent was evaporated under reduced pressure. The crude was purified by column chromatography over silica gel (hexane) to afford the product as a yellow solid (36.6 mg, 0.129 mmol, 13% yield). ^{1}H-NMR (400 MHz, $CDCl_3$) δ, ppm: 7.99 (dd, J = 8.2, 1.3 Hz, 1H), 7.65 (td, J = 7.5, 1.3 Hz, 1H), 7.51 (ddd, J = 8.2, 7.5, 1.5 Hz, 1H), 7.28 (dd, J = 7.5, 1.5 Hz, 1H), 7.10 (s, 2H), 1.99 (s, 6H), 1.34 (s, 9H). ^{13}C-NMR (100 MHz, $CDCl_3$) δ, ppm: 150.7, 149.6, 136.1, 135.1, 134.1,

133.0, 132.2, 128.2, 124.5, 124.2, 34.5, 31.5, 20.8. HRMS (ESI-MS) *m/z* calculated for $C_{18}H_{21}NO_2$ $[M+H]^+$ 284.1645, found 284.1641. TLC (SiO_2, hexane), R_f = 0.33.

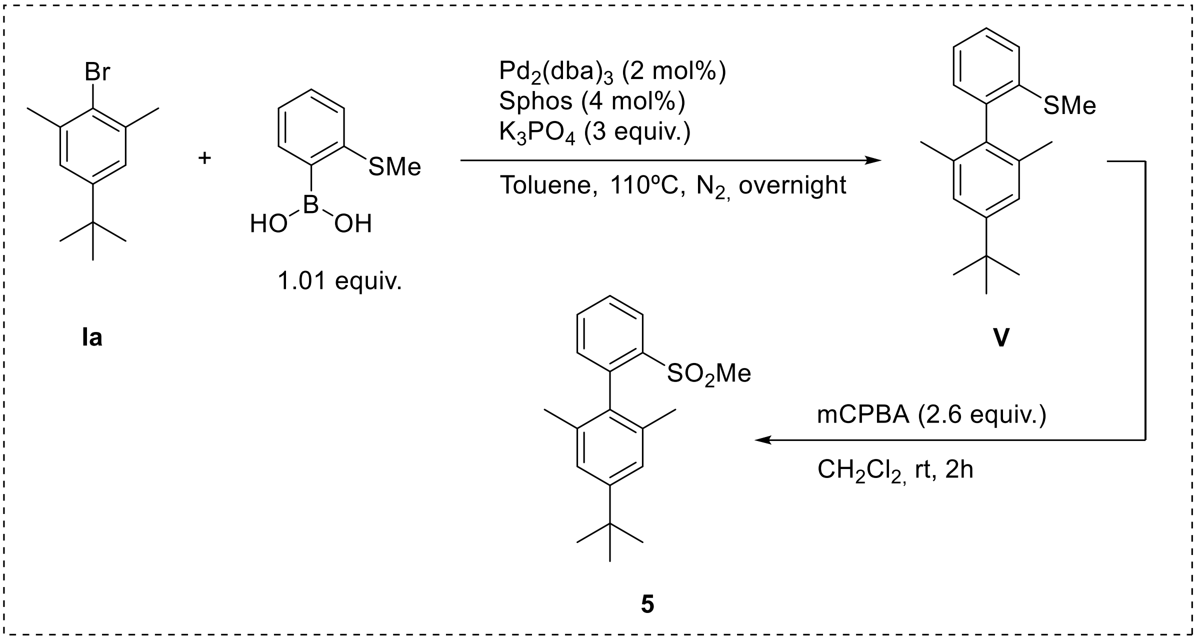

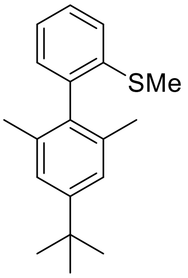

(4'-(*tert*-butyl)-2',6'-dimethyl-[1,1'-biphenyl]-2-yl)(methyl)sulfane (V). It was prepared according to General Procedure A[8] using **Ia** (240 mg, 1.00 mmol, 1 equiv.), (2-(methylthio)phenyl)boronic acid (175 mg, 1.01 mmol, 1.01 equiv.), $Pd_2(dba)_3$ (18.5 mg, 0.020 mmol, 2 mol%), Sphos (16.8 mg, 0.04 mmol, 4 mol%) and K_3PO_4 (637 mg, 3.00 mmol, 3 equiv.) in toluene (5 mL). The crude was purified by column chromatography over silica gel (hexane) to obtain the product as a colorless solid (187 mg, 0.657 mmol, 66% yield). ^{1}H-NMR (400 MHz, $CDCl_3$) δ, ppm: 7.42 (td, *J* = 7.5, 1.5 Hz, 1H), 7.34 – 7.25 (m, 2H), 7.24 (s, 2H), 7.13 (dd, *J* = 7.5, 1.5 Hz, 1H), 2.42 (s, 3H), 2.12 (s, 6H), 1.46 (s, 9H). ^{13}C-NMR (100 MHz, $CDCl_3$) δ, ppm: 150.3, 139.1, 138.1, 136.6, 135.8, 129.4, 127.6, 124.6, 124.4, 123.7, 34.4, 31.6, 20.6, 14.8. HRMS (ESI-MS) *m/z* calculated for $C_{19}H_{24}S$ $[M+H]^+$ 285.1671, found 285.1676. TLC (SiO_2, hexane), R_f = 0.79.

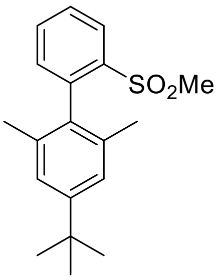

4-(*tert*-butyl)-2,6-dimethyl-2'-(methylsulfonyl)-1,1'-biphenyl (5). It was prepared according to a modified reported procedure.[20] **V** (187 mg, 0.658 mmol, 1 equiv.) was dissolved in CH_2Cl_2 (6.5 mL, 0.1 M). mCPBA (383 mg, 1.71 mmol, 2.6 equiv.) was then added and the reaction was stirred at room temperature for 2 hours. The reaction was quenched a with saturated $NaHCO_3$ solution (10 mL) and the product was extracted with CH_2Cl_2 (3 x 20 mL). The combined organic layers were dried over anhydrous $MgSO_4$, filtered and the solvent was evaporated under reduced pressure. The crude was purified by column chromatography over silica gel (hexane:EtOAc, 80:20) to obtain the product as a colorless solid (85.5 mg, 0.270 mmol, 41% yield). ^{1}H-NMR (400 MHz, $CDCl_3$) δ, ppm: 8.25 (dd, *J* = 7.8, 1.4 Hz, 1H), 7.66 (td, *J* = 7.7, 1.4 Hz, 1H), 7.55 (td, *J* = 7.7, 1.4 Hz, 1H), 7.23 (dd, *J* = 7.7, 1.4 Hz, 1H), 7.13 (s, 2H), 2.67 (s, 3H), 2.00 (s, 6H), 1.34 (s, 9H). ^{13}C-NMR (100 MHz, $CDCl_3$) δ, ppm: 151.4, 140.5, 139.2, 136.1, 134.5, 133.7, 132.1, 128.8, 127.9,

124.4. HRMS (ESI-MS) m/z calculated for $C_{19}H_{24}O_2S$ $[M+Na]^+$ 339.1389, found 339.1383. TLC (SiO_2, hexane:EtOAc 8:2), R_f = 0.35.

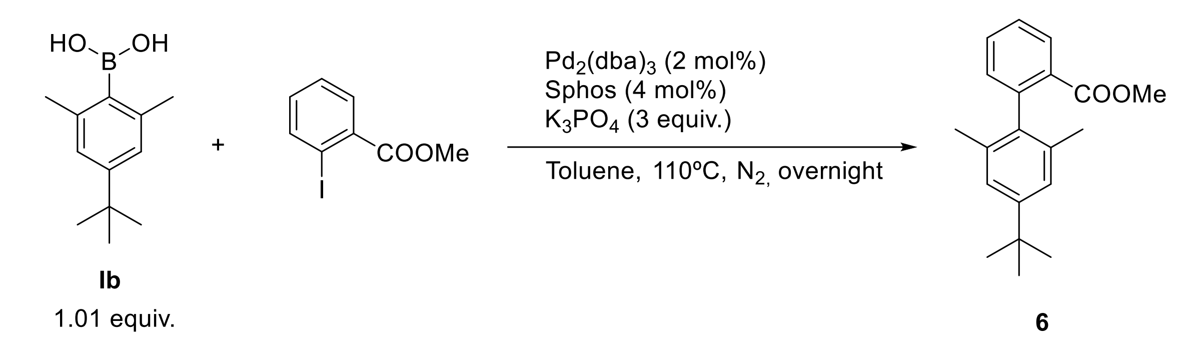

Methyl 4'-(*tert*-butyl)-2',6'-dimethyl-[1,1'-biphenyl]-2-carboxylate (6). It was prepared according to General Procedure A[8] using methyl 2-iodobenzoate (265 mg, 1.00 mmol, 1 equiv.), **Ib** (208 mg, 1.01 mmol, 1.01 equiv.), $Pd_2(dba)_3$ (18.5 mg, 0.020 mmol, 2 mol%), Sphos (16.8 mg, 0.04 mmol, 4 mol%) and K_3PO_4 (637 mg, 3.00 mmol, 3 equiv.) in toluene (5 mL). The crude was purified by column chromatography over silica gel (hexane:EtOAc, 100:0 to 97:3) to obtain the product as a colorless solid (45.8 mg, 0.155 mmol, 16% yield). ^{1}H-NMR (400 MHz, $CDCl_3$) δ, ppm: 7.99 (dd, J = 7.7, 1.5 Hz, 1H), 7.55 (td, J = 7.6, 1.5 Hz, 1H), 7.41 (td, J = 7.6, 1.3 Hz, 1H), 7.17 (dd, J = 7.7, 1.3 Hz, 1H), 7.08 (s, 2H), 3.59 (s, 3H), 1.94 (s, 6H), 1.35 (s, 9H). ^{13}C-NMR (100 MHz, $CDCl_3$) δ, ppm: 167.8, 149.6, 142.6, 138.2, 134.7, 132.2, 131.0, 130.6, 130.3, 127.0, 124.0, 51.9, 34.4, 31.6, 21.0. HRMS (ESI-MS) m/z calculated for $C_{20}H_{24}O_2$ $[M+Na]^+$ 319.1669, found 319.16670. TLC (SiO_2, hexane:EtOAc 9:1), R_f = 0.71.

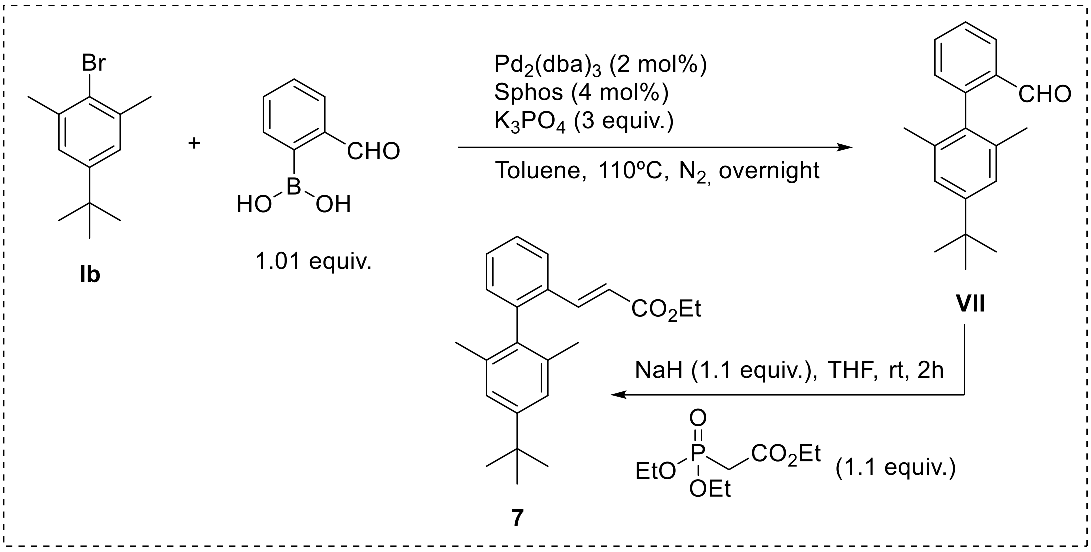

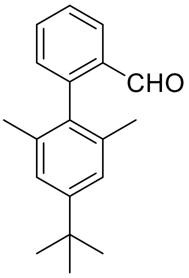

4'-(*tert*-butyl)-2',6'-dimethyl-[1,1'-biphenyl]-2-carbaldehyde (VII). It was prepared according to General Procedure A[8] using **Ia** (240 mg, 1.00 mmol, 1 equiv.), (2-formylphenyl)boronic acid (151 mg, 1.01 mmol, 1.01 equiv.), $Pd_2(dba)_3$ (18.5 mg, 0.020 mmol, 2 mol%), Sphos (16.8 mg, 0.04 mmol, 4 mol%) and K_3PO_4 (637 mg, 3.00 mmol, 3 equiv.) in toluene (5 mL). The crude was purified by column chromatography over silica gel using hexane/AcOEt (8:2) to obtain the product as a colorless solid (253 mg, 0.951 mmol, 95% yield). ^{1}H-NMR (400 MHz, $CDCl_3$) δ, ppm: 9.67 (s, 1H), 8.06 (dd, *J* = 7.8, 1.5 Hz, 1H), 7.68 (td, *J* = 7.5, 1.5 Hz, 1H), 7.55 – 7.47 (m, 1H), 7.30 – 7.23 (m, 1H), 7.16 (s, 2H) 2.00 (s, 6H), 1.38 (s, 9H). ^{13}C-NMR (100 MHz, $CDCl_3$) δ, ppm: 192.6, 150.9, 145.5, 135.8, 134.3, 134.1, 133.8, 130.7, 127.6, 127.1, 124.5, 34.4, 31.4, 21.2. HRMS (ESI-MS) *m/z* calculated for $C_{19}H_{22}O$ $[M+H]^+$ 267.1743, found 267.1745. TLC (SiO_2, hexane/AcOEt 8:2), R_f = 0.72.

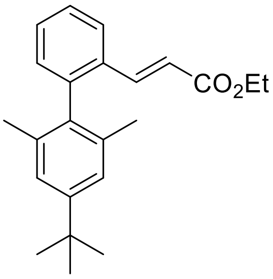

Ethyl (E)-3-(4'-(tert-butyl)-2',6'-dimethyl-[1,1'-biphenyl]-2-yl)acrylate (7). It was prepared according to a modified reported procedure.[21] To a suspension of NaH (20.8 mg, 0.868 mmol, 1.1 equiv.) in THF (600 μL), a solution of triethyl phosphonoacetate (194 mg, 0.868 mmol, 1,1 equiv.) in THF (300 μL) was slowly added. The mixture was stirred at room temperature for 30 min. Then, **VII** (210 mg, 0.790 mmol, 1.0 equiv.) in THF (200 μL) was added at 0 °C, and the mixture was stirred at room temperature. After confirmation of consumption of the aldehyde by GC, a solution of saturated aqueous sodium bicarbonate (2 mL) was added. The mixture was extracted with EtOAc (3 x 2 mL), washed with brine (5 mL) and dried over Mg_2SO_4. The solvent was removed under reduced pressure and the crude was purified by silica-gel column chromatography using hexane/EtOAc (95:5) to give **7** (260 mg, 0.774 mmol, 98% yield) as a colorless liquid. ^{1}H-NMR (400 MHz, $CDCl_3$) δ, ppm: 7.77 (dd, J = 7.6, 1.5 Hz, 1H), 7.50 – 7.33 (m, 3H), 7.17 (d, J = 12.1 Hz, 3H), 6.32 (d, J = 16.0 Hz, 1H), 4.18 (q, J = 7.1 Hz, 2H), 1.96 (s, 6H), 1.40 (s, 9H), 1.28 (t, J = 7.1 Hz, 3H). ^{13}C-NMR (100 MHz, $CDCl_3$) δ, ppm: 167.0, 150.3, 142.8, 142.4, 136.2, 135.5, 133.0, 130.4, 130.2, 127.4, 126.5, 124.5, 118.9, 60.3, 34.4, 31.4, 20.9, 14.2. HRMS (ESI-MS) *m/z* calculated for $C_{23}H_{28}O_2$ $[M+Na]^+$ 337.2162, found 337.2165. TLC (SiO_2, hexane:EtOAc 95:5), R_f = 0.7.

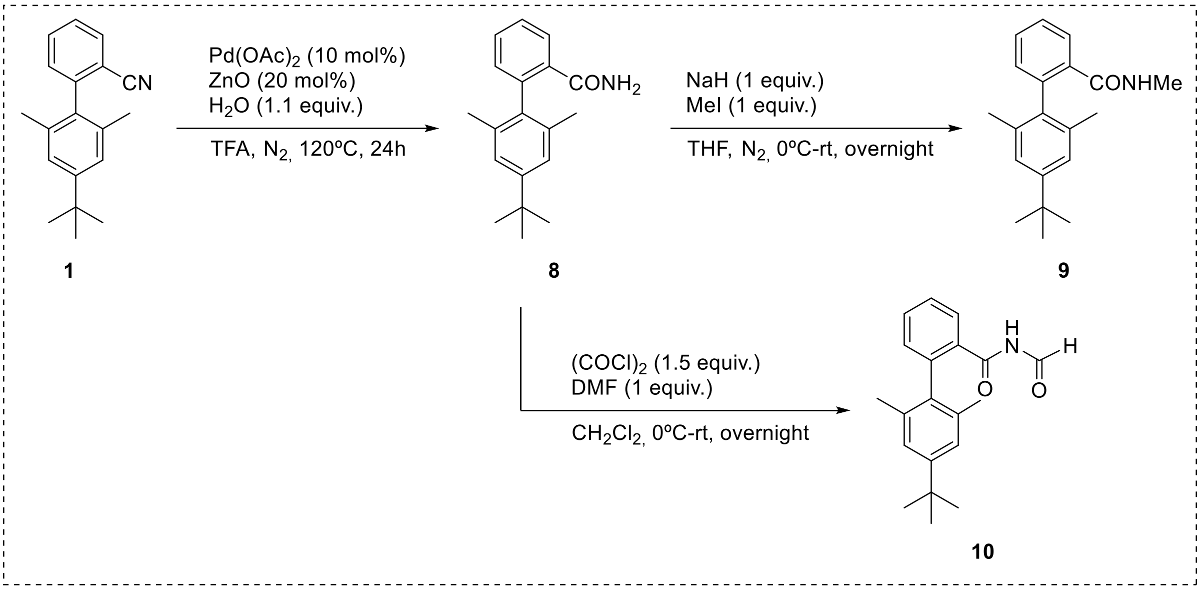

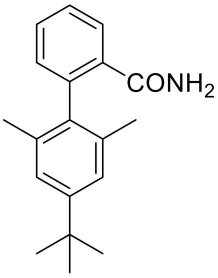

4'-(tert-butyl)-2',6'-dimethyl-[1,1'-biphenyl]-2-carboxamide (8). It was prepared according to a modified reported procedure.[22] In a crimped vial and under N_2 atmosphere, **1** (184 mg, 0.697 mmol, 1 equiv.), $Pd(OAc)_2$ (16.0 mg, 0.0697 mmol, 10 mol%) and ZnO (11.7 mg, 0.139 mmol, 20 mol%) were dissolved in TFA (1.5 mL, 0.45M). The mixture was stirred for 30 minutes at room temperature and then 1.1 equiv. of H_2O (13.8 mL, 0.766 mmol, 1.1 equiv.) was slowly added. The reaction was stirred at 120ºC for 24h. The crude reaction mixture was diluted with CH_2Cl_2 and filtered through a celite plug. The solvent was removed under reduced pressure and the crude was purified by column chromatography over silica gel (hexane:EtOAc, 100:0 to 80:20) to afford the product as a colorless solid (74.9 mg, 0.266 mmol, 38% yield). ^{1}H-NMR (400 MHz, $CDCl_3$) δ, ppm: 8.21 (dd, *J* = 7.7, 1.5 Hz, 1H), 7.52 (td, *J* = 7.5, 1.5 Hz, 1H), 7.44 (td, *J* = 7.7, 1.5 Hz, 1H), 7.16 (s, 2H), 7.09 (dd, *J* = 7.5, 1.5 Hz, 1H), 6.10 (br, 1H), 5.51 (br, 1H), 2.00 (s, 6H), 1.34 (s, 9H). ^{13}C-NMR (100 MHz, $CDCl_3$) δ, ppm: 169.5, 151.3, 139.4, 136.9, 135.5, 132.2, 131.8, 130.8, 130.5, 127.6, 125.3, 34.5, 31.5, 20.9. HRMS (ESI-MS) *m/z* calculated for $C_{19}H_{23}NO$ $[M+Na]^+$ 304.1672, found 304.1669. TLC (SiO_2, hexane:EtOAc 8:2), R_f = 0.23.

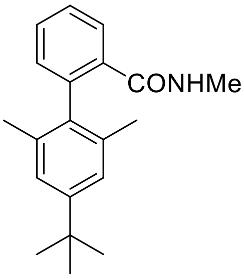

4'-(tert-butyl)-*N*,2',6'-trimethyl-[1,1'-biphenyl]-2-carboxamide (9). It was prepared according to a modified reported procedure.[23] Under N_2 atmosphere, **8** (85.7 mg, 0.304 mmol, 1 equiv.) was dissolved in anhydrous THF (1.5 mL, 0.2M). The solution was cooled to 0ºC and NaH (8.12 mg, 0.304 mmol, 1 equiv.) was slowly added. The mixture was further stirred at 0ºC for 15 minutes and then iodomethane (19.2 mL, 0.304 mmol, 1 equiv.) was added dropwise and the reaction was stirred at room temperature overnight. The reaction was quenched with H_2O (2 mL) and the product was extracted with EtOAc (3x10 mL). The combined organic phases were dried over anhydrous $MgSO_4$, filtered and the solvent was evaporated under reduced pressure. The crude was purified by column chromatography over silica gel (hexane:EtOAc, 100:0 to 73:27) to obtain the product as a colorless solid (53.0 mg, 0.179 mmol, 59% yield). ^{1}H-NMR (400 MHz, $CDCl_3$) δ, ppm: 8.14 (dd, *J* = 7.5, 1.5 Hz, 1H), 7.52-7.41 (m, 2H), 7.16 (s, 2H), 7.10 (dd, *J* = 7.5, 1.5 Hz, 1H), 5.40 (br, 1H), 2.59 (d, *J* = 4.8 Hz, 3H), 1.99 (s, 6H), 1.35

(s, 9H). ^{13}C-NMR (100 MHz, $CDCl_3$) δ, ppm: 168.3, 151.2, 138.5, 136.9, 135.6, 133.7, 131.1, 130.4, 130.3, 127.7, 125.2, 34.6, 31.5, 26.9, 21.0. HRMS (ESI-MS) *m/z* calculated for $C_{20}H_{25}NO$ $[M+H]^+$ 296.2009, found 296.2013. TLC (SiO_2, hexane:EtOAc 7:3), R_f = 0.33.

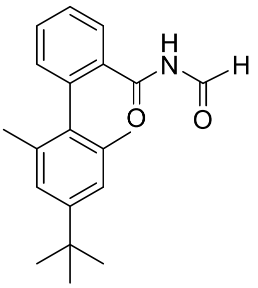

4'-(tert-butyl)-*N*-formyl-2',6'-dimethyl-[1,1'-biphenyl]-2-carboxamide (10). It was prepared according to a modified reported procedure.[24] Under N_2 atmosphere, **8** (148 mg, 0.527 mmol, 1 equiv.) was disdissolved in anhydrous CH_2Cl_2 (2.6 mL, 0.2 M) and cooled to 0ºC. Oxalyl chloride (68 mL, 0.788 mmol, 1.5 equiv.) and DMF (40.8 mL, 0.527 mmol, 1 equiv.) were added. The mixture was stirred at room temperature overnight. The reaction was quenched with H_2O (5 mL) and the product was extracted with CH_2Cl_2 (3x10 mL). The combined organic phases were dried over anhydrous $MgSO_4$, filtered and the solvent was evaporated under reduced pressure. The crude was purified by column chromatography over silica gel (hexane:EtOAc, 100:0 to 80:20) to obtain the product as a colorless solid (23.8 mg, 0.0769 mmol, 15% yield). ^{1}H-NMR (400 MHz, $CDCl_3$) δ, ppm: 9.09 (d, *J* = 9.7 Hz, 1H), 8.19 (dd, *J* = 7.8, 1.5 Hz, 1H), 7.78 (s, 1H), 7.65 (td, *J* = 7.6, 1.4 Hz, 1H), 7.52 (td, *J* = 7.6, 1.4 Hz, 1H), 7.23-7.19 (m, 3H), 2.00 (s, 6H), 1.35 (s, 9H). ^{13}C-NMR (100 MHz, $CDCl_3$) δ, ppm: 167.2, 162.3, 152.5, 140.3, 135.4, 135.3, 133.4, 131.1, 131.0, 130.7, 128.1, 125.8, 34.7, 31.4, 21.0. HRMS (ESI-MS) *m/z* calculated for $C_{20}H_{23}NO_2$ $[M+Na]^+$ 332.1626, found 332.1624. TLC (SiO_2, hexane:EtOAc 9:1), R_f = 0.31.

***Note**: this product was obtained as a byproduct in the synthesis of other compound and was decided to use as substrate under the catalytic conditions.*

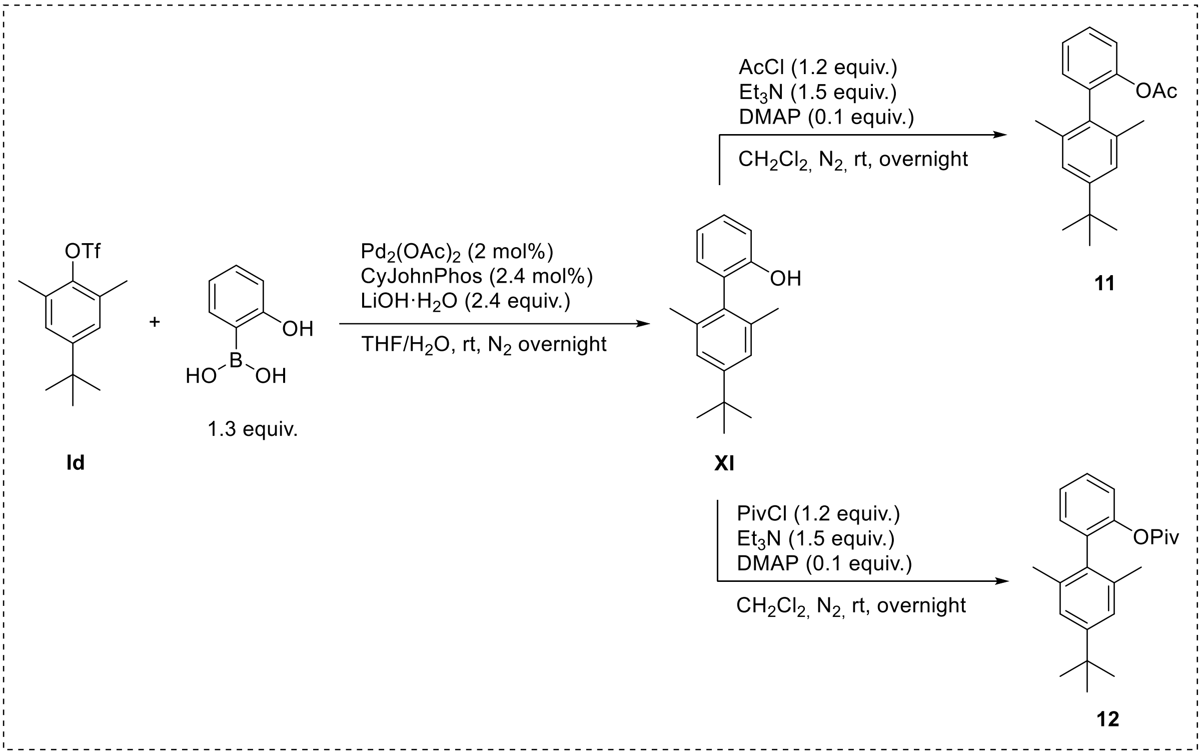

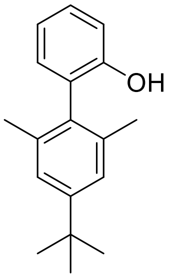

4'-(*tert*-butyl)-2',6'-dimethyl-[1,1'-biphenyl]-2-ol (XI). It was prepared according to a modified reported procedure.[25] A Schlenk tube containing a magnetic stir bar was charged with **Id** (1.53 g, 4.93 mmol, 1 equiv.), (2-hydroxyphenyl)boronic acid (902 mg, 6.41 mmol, 1.3 equiv.), $Pd(OAc)_2$ (22.4 mg, 0.0986 mmol, 2 mol%), CyJohnPhos (42.3 mg, 0.118 mmol, 2.4 mol%) and LiOH·H_2O (507 mg, 11.8 mmol, 2.4 equiv.) and dissolved in a mixture of THF/H_2O (8:2 v/v, 5 mL, 1 M) under N_2 atmosphere. The reaction was stirred at room temperature overnight. The reaction was quenched with 2N $HCl_{(aq)}$ (50 mL) and the product was extracted with EtOAc (3x50 mL). The combined organic layers were washed with brine, dried over anhydrous $MgSO_4$, filtered and the solvent was evaporated under reduced pressure. The crude was purified by column chromatography over silica gel (hexane:EtOAc, 100:0 to 94:6) to obtain the product as a colorless solid (479 mg, 1.88 mmol, 38% yield). ^{1}H-NMR (400 MHz, $CDCl_3$) δ, ppm: 7.33 (ddd, *J* = 8.2, 7.2, 1.9 Hz, 1H), 7.26 (s, 2H), 7.12-7.01 (m, 3H), 4.79 (s, 1H), 2.13 (s, 6H), 1.43 (s, 9H). ^{13}C-NMR (100 MHz, $CDCl_3$) δ, ppm: 152.6, 151.3, 137.5, 131.8, 130.1, 129.0, 126.7, 125.1, 120.8, 115.2, 34.5, 31.5, 20.7. HRMS (ESI-MS) *m/z* calculated for $C_{18}H_{22}O$ $[M-H]^-$ 253.1598, found 253.1600. TLC (SiO_2, hexane:EtOAc 9:1), R_f = 0.38.

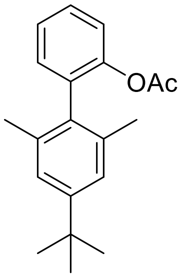

4'-(*tert*-butyl)-2',6'-dimethyl-[1,1'-biphenyl]-2-yl acetate (11). It was prepared according to General Procedure D using **XI** (146 mg, 0.574 mmol, 1 equiv.), acetyl chloride (49.9 mL, 0.688 mmol, 1.2 equiv), Et_3N (121 mL, 0.860 mmol, 1.5 equiv.) and DMAP (7.15 mg, 0.0574 mmol, 0.1 equiv.) in CH_2Cl_2 (3 mL). The crude was purified by column chromatography over silica gel (hexane:EtOAc, 100:0 to 90:10) to obtain the product as a colorless oil (131 mg, 0.442 mmol, 77% yield). ^{1}H-NMR (400 MHz, $CDCl_3$) δ, ppm: 7.40 (ddd, *J* = 7.9, 7.4, 1.8 Hz, 1H), 7.32 (td, *J* = 7.4, 1.3 Hz, 1H), 7.25 (dd, *J* = 7.4, 1.8 Hz, 1H), 7.18 (dd, *J* = 8.0, 1.3 Hz, 1H), 7.13 (s, 2H), 2.07 (s, 6H), 1.87 (s, 3H), 1.37 (s, 9H). ^{13}C-NMR (100 MHz, $CDCl_3$) δ, ppm: 169.3, 150.3, 148.8, 136.3, 134.1, 133.6, 131.3, 128.3, 126.2, 124.2, 122.6, 34.4, 31.5, 20.7, 20.4. HRMS (ESI-MS) *m/z* calculated for $C_{20}H_{24}O_2$ $[M+H]^+$ 297.1849, found 297.1850. TLC (SiO_2, hexane:EtOAc 95:5), R_f = 0.29.

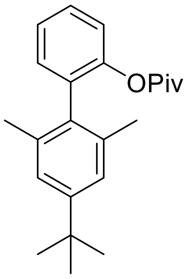

4'-(*tert*-butyl)-2',6'-dimethyl-[1,1'-biphenyl]-2-yl pivalate (12). It was prepared according to General Procedure D using **XI** (113 mg, 0.445 mmol, 1 equiv.), pivaloyl chloride (66.0 mL, 0.534 mmol, 1.2 equiv.), Et_3N (94.0 mL, 0.668 mmol, 1.5 equiv.) and DMAP (5.56 mg, 0.0445 mmol, 0.1 equiv.) in CH_2Cl_2 (2 mL). The crude was purified by column chromatography over silica gel (hexane:EtOAc, 100:0 to 95:5) to obtain the product as a colorless solid (28.5 mg, 0.0842 mmol, 19% yield). ^{1}H-NMR (400 MHz, $CDCl_3$) δ, ppm: ^{1}H NMR (400 MHz, $CDCl_3$) δ 7.38 (td, *J* = 7.8, 1.8 Hz, 1H), 7.30 (td, *J* = 7.5, 1.3 Hz, 1H), 7.23 (dd, *J* = 7.5, 1.8 Hz, 1H), 7.13 (dd, *J* = 7.8, 1.3 Hz, 1H), 7.08 (s, 2H), 2.03 (s, 6H), 1.31 (d, *J* = 1.6 Hz, 9H), 0.91 (d, *J* = 1.4 Hz, 9H). ^{13}C-NMR (100 MHz, $CDCl_3$) δ, ppm: 176.7, 150.5, 148.9, 136.5, 134.3, 133.7, 130.9, 128.3, 126.1, 124.2, 122.8, 38.8, 34.4, 31.5, 26.7, 20.7. HRMS (ESI-MS) *m/z* calculated for $C_{23}H_{30}O_2$ $[M+H]^+$ 339.2319, found 339.2311. TLC (SiO_2, hexane:EtOAc 95:5), R_f = 0.14.

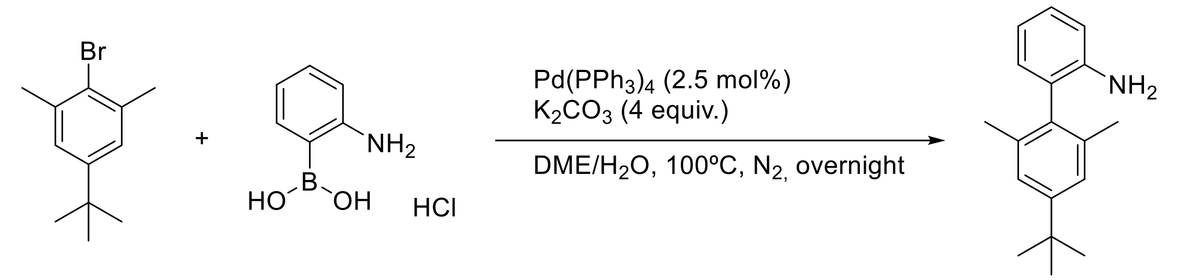

Ia
1.1 equiv.

XIII

4'-(*tert*-butyl)-2',6'-dimethyl-[1,1'-biphenyl]-2-amine (XIII). It was prepared according to a modified reported procedure.[26] A Schlenk tube containing a magnetic stir bar was charged with **Ia** (2.12 g, 8.79 mmol, 1.1 equiv.), (2-aminophenyl)boronic acid hydrochloride (1.38 g, 7.96 mmol, 1 equiv.), $Pd(PPh_3)_4$ (236 mg, 0.200 mmol, 2.5 mol%) and K_2CO_3 (4.42 g, 32.0 mmol, 4 equiv.) and dissolved in a mixture of DME/H_2O (2:1 v/v, 10 mL, 0.8 M) under N_2 atmosphere. The reaction was stirred at 100ºC overnight. The reaction was diluted with H_2O (50 mL) and the product was extracted with EtOAc (3x50 mL). The organic phases were washed with brine, dried over anhydrous $MgSO_4$, filtered and the solvent was evaporated under reduced pressure. The crude was purified by column chromatography over silica gel (hexane:EtOAc, 100:0 to 93:7) to obtain the product as a colorless solid (1.09 g, 4.30 mmol, 54% yield). ^{1}H-NMR (400 MHz, $CDCl_3$) δ, ppm: 7.19-7.12 (m, 3H), 6.94 (ddd, *J* = 7.5, 1.6, 0.5 Hz, 1H), 6.84-6.76 (m, 2H), 3.40 (br, 2H), 2.05 (s, 6H), 1.34 (s, 9H). ^{13}C-NMR (100 MHz, $CDCl_3$) δ, ppm: 150.3, 143.8, 136.7, 135.0, 130.1, 128.1, 126.5, 124.8, 118.6, 115.1, 34.5, 31.6, 20.6. HRMS (ESI-MS) *m/z* calculated for $C_{18}H_{23}N$ $[M+H]^+$ 254.1903, found 254.1906. TLC (SiO_2, hexane:EtOAc 10:1), R_f = 0.68. ***Note****: DME and H_2O was degassed with a N_2 flux for several minutes before adding to the reaction.*

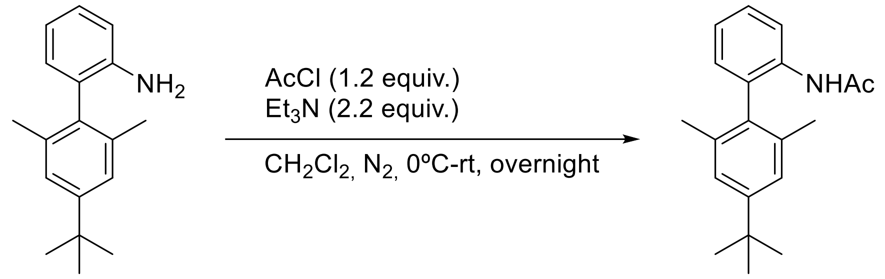

XIII

13

4'-(*tert*-butyl)-2',6'-dimethyl-[1,1'-biphenyl]-2-yl acetamide (13). It was prepared according to General Procedure E using **XIII** (200 mg, 0.789 mmol, 1 equiv.), acetyl chloride (68.7 mL, 0.947 mmol, 1.2 equiv) and Et_3N (242 mL, 1.73 mmol, 2.2 equiv.) in CH_2Cl_2 (4 mL). The crude was purified by column chromatography over silica gel (hexane:EtOAc, 100:0 to 80:20) to obtain the product as a colorless solid (149 mg, 0.504 mmol, 64% yield). ^{1}H-NMR (400 MHz, $CDCl_3$) δ, ppm: 8.40 (d, *J* = 8.3 Hz, 1H), 7.35 (td, *J* = 7.9, 1.7 Hz, 1H), 7.20-7.11 (m, 3H), 7.05 (dd, *J* = 7.6, 1.7 Hz, 1H), 6.76 (br, 1H), 2.00 (s, 7H), 1.93 (s, 3H), 1.36 (s, 9H). ^{13}C-NMR (100 MHz, $CDCl_3$) δ, ppm: 168.2, 151.2, 136.6, 135.6, 133.2, 129.9, 129.7, 128.2, 125.1, 124.2, 120.3, 34.6, 31.5, 25.0, 20.7. HRMS (ESI-MS) *m/z* calculated for $C_{20}H_{25}NO$ $[M+H]^+$ 296.2009, found 296.2006. TLC (SiO_2, hexane:EtOAc 8:2), R_f = 0.40.

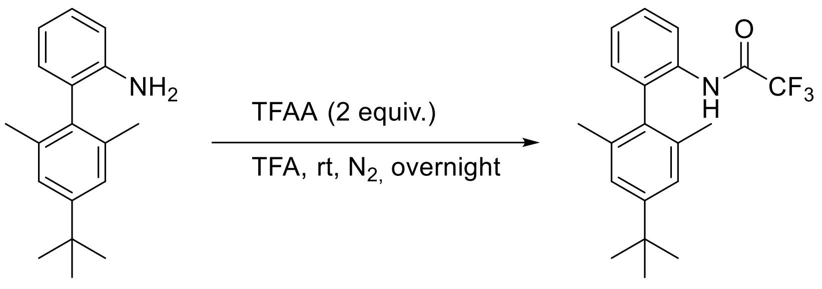

***N*-(4'-(*tert*-butyl)-2',6'-dimethyl-[1,1'-biphenyl]-2-yl)-2,2,2-trifluoroacetamide (14)**. It was prepared according to a modified reported procedure.[27] Under N_2 atmosphere, **XIII** (200 mg, 0.789 mmol, 1 equiv.) was dissolved in trifluoroacetic acid (1 mL, 0.8 M) and cooled to 0ºC. Trifluoroacetic anhydride (222 mL, 1.58 mmol, 2 equiv.) was added to the solution and the reaction was stirred at room temperature overnight. The reaction was quenched carefully with H_2O (2 mL) and the product was extracted with CH_2Cl_2 (3x10 mL). The combined organic layers were washed with saturated $NaHCO_3$ solution, dried over anhydrous $MgSO_4$, filtered and the solvent was evaporated under reduced pressure. The crude was purified by column chromatography over silica gel (hexane:EtOAc, 100:0 to 96:4) to obtain the product as a colorless solid (169 mg, 0.484 mmol, 61% yield). ^{1}H-NMR (400 MHz, $CDCl_3$) δ, ppm: 8.44 (d, *J* = 8.3 Hz, 1H), 7.56 (br, 1H), 7.45 (td, *J* = 7.8, 1.6 Hz, 1H), 7.32 (td, *J* = 7.5, 1.1 Hz, 1H), 7.25 (s, 2H), 7.21 (dd, *J* = 7.7, 1.5 Hz, 1H), 2.02 (s, 6H), 1.39 (s, 9H). ^{13}C-NMR (100 MHz, $CDCl_3$) δ, ppm: 154.4 (q, *J* = 37.0 Hz), 152.1, 136.4, 133.1, 131.7, 131.3, 130.0, 128.6, 126.2, 125.3, 120.9, 117.1 (q, *J* = 293.6 Hz), 34.6, 31.4, 20.5. ^{19}F-NMR (377 MHz, $CDCl_3$) δ, ppm: -77.4. HRMS (ESI-MS) *m/z* calculated for $C_{20}H_{22}F_3NO$ $[M+H]^+$ 350.1726, found 350.1727. TLC (SiO_2, hexane:EtOAc 95:5), R_f = 0.46.

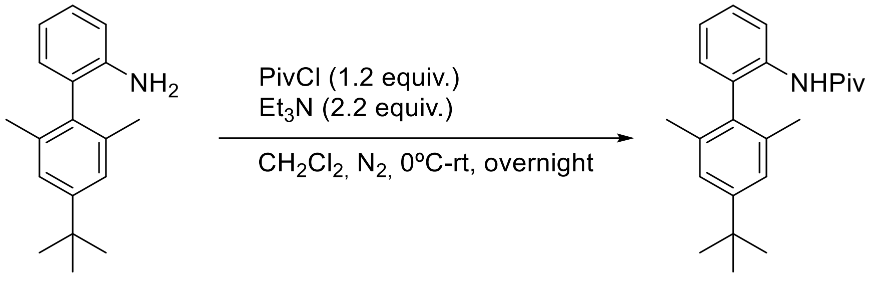

4'-(*tert*-butyl)-2',6'-dimethyl-[1,1'-biphenyl]-2-yl pivalamide (15). It was prepared according to General Procedure E using **XIII** (200 mg, 0.789 mmol, 1 equiv.), pivaloyl chloride (118 mL, 0.947 mmol, 1.2 equiv) and Et_3N (244 mL, 1.74 mmol, 2.2 equiv.) in CH_2Cl_2 (4 mL). The crude was purified by column chromatography over silica gel (hexane:EtOAc, 100:0 to 94:6) to obtain the product as a colorless solid (174 mg, 0.516 mmol, 65% yield). ^{1}H-NMR (400 MHz, $CDCl_3$) δ, ppm: 8.44 (dd, *J* = 8.3, 1.1 Hz, 1H), 7.40-7.34 (m, 1H), 7.21 (s, 2H), 7.18-7.09 (m, 2H), 7.03 (br, 1H), 1.99 (s, 6H), 1.35 (s, 9H), 0.95 (s, 9H). ^{13}C-NMR (100 MHz, $CDCl_3$) δ, ppm: 176.3, 151.6, 136.7, 135.6, 133.3, 130.1, 129.0, 128.3, 125.0, 123.9, 119.7, 39.7, 34.6, 31.4, 27.2, 20.5. HRMS (ESI-MS) *m/z* calculated for $C_{23}H_{31}NO$ $[M+Na]^+$ 360.2298, found 360.2293. TLC (SiO_2, hexane:EtOAc 9:1), R_f = 0.64.

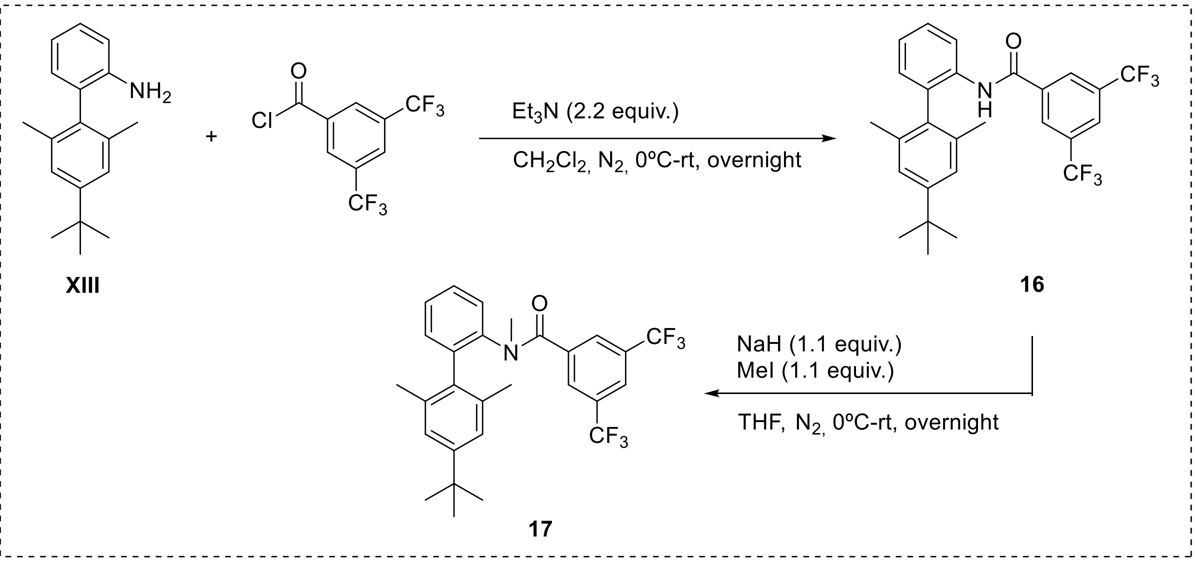

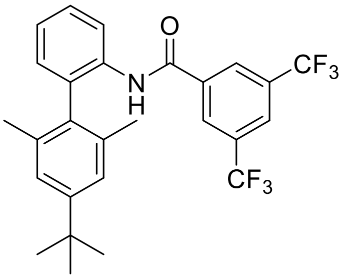

***N*-(4'-(*tert*-butyl)-2',6'-dimethyl-[1,1'-biphenyl]-2-yl)-3,5-bis(trifluoromethyl)benzamide (16)**. It was prepared according to General Procedure E using **XIII** (400 mg, 1.58 mmol, 1 equiv.), 3,5-bis(trifluoromethyl)benzoyl chloride (354 mL, 1.89 mmol, 1.2 equiv) and Et_3N (500 mL, 3.47 mmol, 2.2 equiv.) in CH_2Cl_2 (8 mL). The crude was purified by column chromatography over silica gel (hexane:EtOAc, 100:0 to 93:7) to obtain the product as a colorless solid (583 mg, 1.18 mmol, 75% yield). ^{1}H-NMR (400 MHz, $CDCl_3$) δ, ppm: 8.69 (dd, *J* = 8.3, 1.3 Hz, 1H), 7.99- 7.95 (m, 1H), 7.92-7.87 (m, 2H), 7.74 (br, 1H), 7.48 (ddd, *J* = 8.6, 7.5, 1.7 Hz, 1H), 7.32-7.26 (m, 3H), 7.24-7.18 (m, 1H), 2.09 (s, 6H), 1.39 (s, 9H). ^{13}C-NMR (100 MHz, $CDCl_3$) δ, ppm: 161.4, 152.1, 136.9, 136.5, 134.7, 132.4 (q, *J* = 34.8 Hz), 132.3, 130.3, 129.6, 128.5, 126.9 (q, *J* = 3.8 Hz), 125.4, 125.0, 122.7 (q, *J* = 273.3 Hz), 119.6, 34.5, 31.2, 20.6. ^{19}F-NMR (377 MHz, $CDCl_3$) δ, ppm: -64.0. HRMS (ESI-MS) *m/z* calculated for $C_{27}H_{25}F_6NO$ $[M+Na]^+$ 516.1733, found 516.1733. TLC (SiO_2, hexane:EtOAc 95:5), R_f = 0.33.

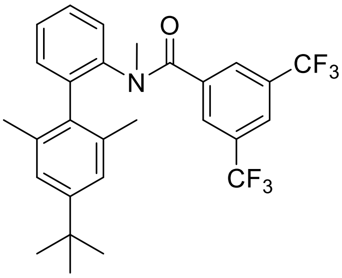

***N*-(4'-(*tert*-butyl)-2',6'-dimethyl-[1,1'-biphenyl]-2-yl)-*N*-methyl-3,5-bis(trifluoromethyl)benzamide (17)**. It was prepared according to a modified reported procedure.[23] Under N_2 atmosphere, **16** (208 mg, 0.427 mmol, 1 equiv.) was dissolved in anhydrous THF (2.15 mL, 0.2M). The solution was cooled to 0ºC and NaH (12.4 mg, 0.463 mmol, 1.1 equiv.) was slowly added. The mixture was further stirred at 0ºC for 15 minutes and then iodomethane (29.0 mL, 0.463 mmol, 1.1 equiv.) was added dropwise and the reaction was stirred at room temperature overnight. The reaction was quenched with H_2O (2 mL) and the product was extracted with EtOAc (3x10 mL). The combined organic phases were dried over anhydrous $MgSO_4$, filtered and the solvent was evaporated under reduced pressure. The crude was purified by column chromatography over silica gel (hexane:EtOAc, 100:0 to 80:20) to obtain the product as a colorless solid (137 mg, 0.270 mmol, 63% yield). **In the NMR spectra, a 2:1 mixture of non-interchangeable*

conformations is observed. ¹H-NMR (400 MHz, $CDCl_3$, 228K) δ, ppm: 7.89 (s, $1H_{minor}$), 7.75 (s, $1H_{major}$), 7.62-7.48 (m, 3H), 7.37-7.31 (m, 1H), 7.30-7.28 (m, $1H_{major}$), 7.23-7.06 (m, 2H), 6.83-6.81 (m, $1H_{minor}$). ^{19}F-NMR (377 MHz, $CDCl_3$, 228K) δ, ppm: -63.6 (minor), -63.8 (major). HRMS (ESI-MS) *m/z* calculated for $C_{28}H_{27}NO$ $[M+H]^+$ 508.2070, found 508.2069. TLC (SiO_2, hexane:EtOAc 8:2), R_f = 0.73.

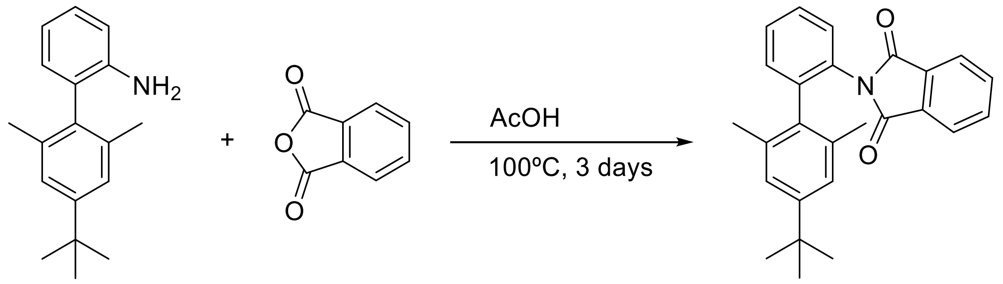

XIII 18

2-(4'-(*tert*-butyl)-2',6'-dimethyl-[1,1'-biphenyl]-2-yl)isoindoline-1,3-dione (18). It was prepared according to General Procedure F using **XIII** (200 mg, 0.789 mmol, 1 equiv.) and phthalic anhydride (118 mg, 0.789 mmol, 1 equiv.) in acetic acid (1.6 mL). The crude was purified by column chromatography over silica gel (hexane:EtOAc, 100:0 to 90:10) to obtain the product as a colorless solid (137 mg, 0.357 mmol, 45% yield). ^{1}H-NMR (400 MHz, $CDCl_3$) δ, ppm: 7.80 (dd, *J* = 5.5, 3.1 Hz, 2H), 7.69 (dd, *J* = 5.5, 3.1 Hz, 2H), 7.59- 7.48 (m, 2H), 7.42-7.33 (m, 2H), 7.01 (s, 2H), 2.11 (s, 6H), 1.25 (s, 9H). ^{13}C-NMR (100 MHz, $CDCl_3$) δ, ppm: 167.2, 150.0, 140.5, 136.3, 134.5, 134.1, 131.9, 131.9, 131.0, 129.5, 129.3, 128.0, 124.3, 123.5. HRMS (ESI-MS) *m/z* calculated for $C_{26}H_{25}NO_2$ $[M+H]^+$ 384.1958, found 384.1960. TLC (SiO_2, hexane:EtOAc 90:10), R_f = 0.25.

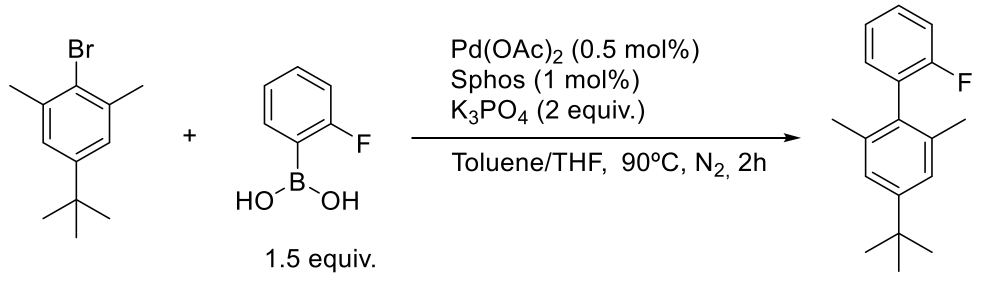

Ia 19

4-(*tert*-butyl)-2'-fluoro-2,6-dimethyl-1,1'-biphenyl (19). It was prepared according to a modified reported procedure.[7] A Schlenk tube containing a magnetic stir bar was charged with **Ia** (241 mg, 1.00 mmol, 1 equiv.), (2-fluorophenyl)boronic acid (221 mg, 1.50 mmol, 1.5 equiv.), $Pd(OAc)_2$ (59.0 mg, 0.005 mmol, 0.5 mol%), Sphos (4.20 mg, 0.010 mmol, 1 mol%), K_3PO_4 (433 mg, 2.00 mmol, 2 equiv.) and dissolved in toluene/THF (1:1 v/v, 4 mL, 0.25 M) under N_2 atmosphere. The reaction was stirred at 90ºC for 2h. The solution was then diluted with EtOAc (20 mL) and filtered through a pad of silica gel. The crude was purified by column chromatography over silica gel (hexane) to afford the product as a colorless oil (163 mg, 0.635 mmol, 64% yield). ^{1}H-NMR (400 MHz, $CDCl_3$) δ, ppm: 7.46-7.39 (m, 1H), 7.31-7.21 (m, 5H), 2.20 (s, 6H), 1.49 (s, 9H). ^{13}C-NMR (100 MHz, $CDCl_3$) δ, ppm: 159.9 (d, *J* = 244.3 Hz), 150.5, 136.2, 132.5, 131.8 (d, *J* = 4.0 Hz), 128.9 (d, *J* = 8.0 Hz), 128.3 (d, *J* = 18.0 Hz), 124.5, 124.2

(d, J = 3.6 Hz), 115.8 (d, J = 22.6 Hz), 34.5, 31.6, 20.9. ^{19}F-NMR (377 MHz, $CDCl_3$) δ, ppm: -115.6 (dt, J = 11.8, 5.9 Hz). GC-MS (CI) m/z for $C_{18}H_{21}F$ $[M+NH_4]^+$ 274.1. TLC (SiO_2, hexane), R_f = 0.61.

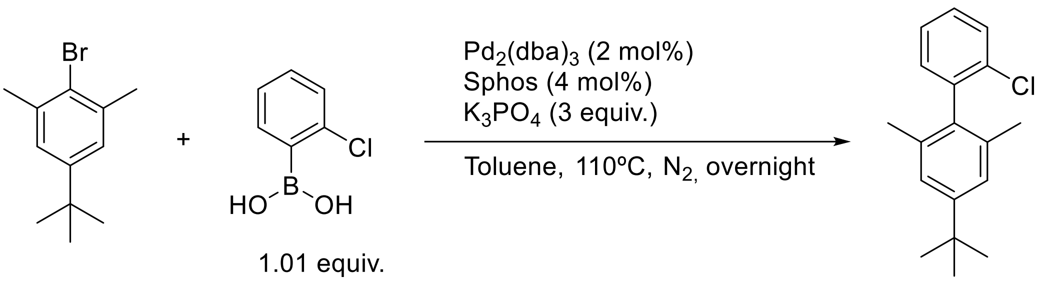

Ia **20**

4-(*tert*-butyl)-2'-chloro-2,6-dimethyl-1,1'-biphenyl (20). It was prepared according to General Procedure A[8] using **Ia** (241 mg, 1.00 mmol, 1 equiv.), (2-chlorophenyl)boronic acid (159 mg, 1.01 mmol, 1.01 equiv.), $Pd_2(dba)_3$ (18.5 mg, 0.020 mmol, 2 mol%), Sphos (16.8 mg, 0.04 mmol, 4 mol%) and K_3PO_4 (649 mg, 3.00 mmol, 3 equiv.) in toluene (5 mL). The crude was purified by column chromatography over silica gel (hexane) to obtain the product as a colorless solid (154 mg, 0.564 mmol, 56% yield). ^{1}H-NMR (400 MHz, $CDCl_3$) δ, ppm: 7.53-7.49 (m, 1H), 7.37-7.28 (m, 2H), 7.22-7.18 (m, 1H), 7.16 (s, 2H), 2.04 (s, 6H), 1.40 (s, 9H). ^{13}C-NMR (100 MHz, $CDCl_3$) δ, ppm: 150.4, 140.1, 136.2, 135.6, 133.9, 131.2, 129.6, 128.4, 127.0, 124.3, 34.5, 31.6, 20.7. GC-MS (CI) m/z for $C_{18}H_{21}Cl$ $[M+NH_4]^+$ 290.1. TLC (SiO_2, hexane), R_f = 0.58.

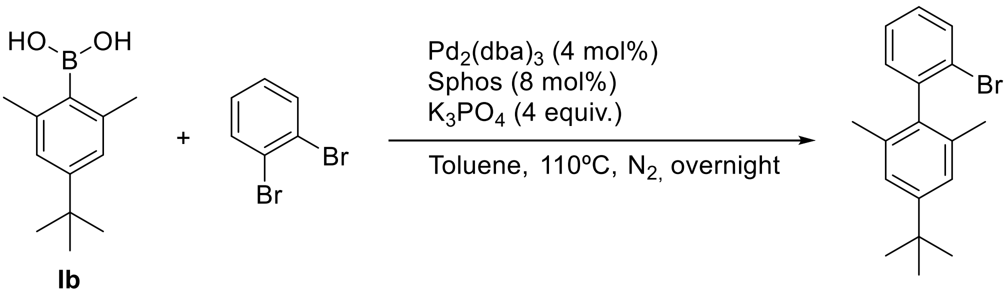

Ib
1.5 equiv. **21**

2'-bromo-4-(*tert*-butyl)-2,6-dimethyl-1,1'-biphenyl (21). It was prepared according to General Procedure A[7] using 1,2-dibromobenzene (238 mg, 1.00 mmol, 1 equiv.), **Ib** (309 mg, 1.50 mmol, 1.5 equiv.), $Pd_2(dba)_3$ (37.0 mg, 0.040 mmol, 4 mol%), Sphos (34.0 mg, 0.080 mmol, 8 mol%) and K_3PO_4 (848 mg, 4.00 mmol, 4 equiv.) in toluene (2 mL). The crude was purified by column chromatography over silica gel (hexane) to obtain the product as a colorless solid (91.6 mg, 0.289 mmol, 29% yield). ^{1}H-NMR (400 MHz, $CDCl_3$) δ, ppm: 7.70 (dd, J = 8.1, 1.2 Hz, 1H), 7.38 (td, J = 7.5, 1.2 Hz, 1H), 7.25 – 7.17 (m, 2H), 7.15 (s, 2H), 2.02 (s, 6H), 1.39 (s, 9H). ^{13}C-NMR (100 MHz, $CDCl_3$) δ, ppm: 150.4, 142.2, 138.0, 135.3, 132.8, 131.0, 128.6, 127.7, 124.4, 124.3, 34.5, 31.6, 20.7. TLC (SiO_2, hexane), R_f =0.40.

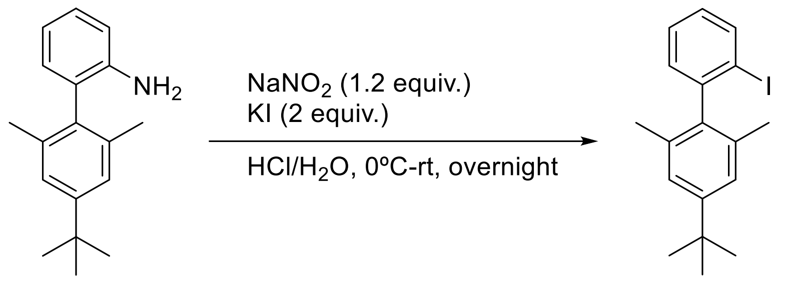

XIII **22**

4-(*tert*-butyl)-2'-iodo-2,6-dimethyl-1,1'-biphenyl (22). It was prepared according to modified reported procedures.[28, 29] A solution of $NaNO_2$ (86.4 mg, 1.22 mmol, 1.2 equiv.) in H_2O (0.6 mL, 2 M) was added to a suspension of **XIII** (242 mg, 1.01 mmol, 1 equiv.) in H_2O / concentrated HCl (5:1 v/v, 2.4 mL, 0.4M) at 0ºC. The reaction was stirred at this temperature for 45 minutes and a solution of KI (340 mg, 2.03 mmol, 2 equiv.) in H_2O (0.6 mL, 3.4 M) was added dropwise. The mixture was stirred at room temperature overnight. The product was extracted with Et_2O (4x20 mL) and the organic phase was washed with 2N HCl(aq), saturated NaHCO3 and brine. The combined organic phases were dried over anhydrous $MgSO_4$, filtered and the solvent was evaporated under reduced pressure. The crude was purified by column chromatography over silica gel (hexane) to obtain the product as a colorless solid (149 mg, 0.425 mmol, 42% yield). ^{1}H-NMR (400 MHz, $CDCl_3$) δ, ppm: 7.96 (dd, *J* = 8.0, 1.2 Hz, 1H), 7.41 (td, *J* = 7.5, 1.2 Hz, 1H), 7.17 (dd, *J* = 7.5, 1.7 Hz, 1H), 7.13 (s, 2H), 7.04 (ddd, *J* = 8.0, 7.5, 1.7 Hz, 1H), 1.98 (s, 6H), 1.38 (s, 9H). ^{13}C-NMR (100 MHz, $CDCl_3$) δ, ppm: 150.6, 146.4, 141.2, 139.2, 135.1, 129.9, 128.6, 128.5, 124.4, 101.1, 34.5, 31.6, 20.8. GC-MS (CI) *m/z* for $C_{18}H_{21}I$ $[M+NH_4]^+$ 382.1. TLC (SiO_2, hexane), R_f = 0.66.

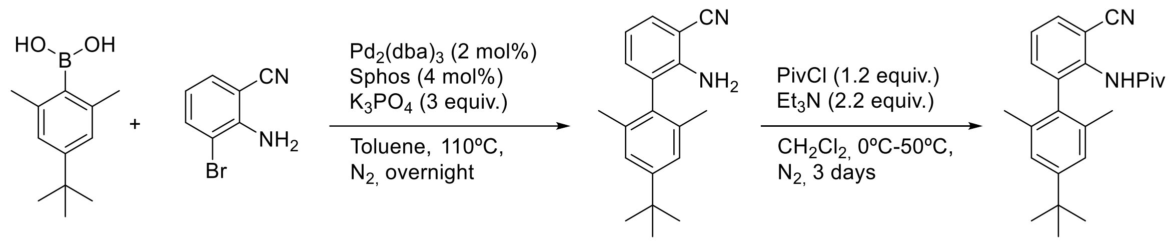

Ib
1.01 equiv.
XXIII **23**

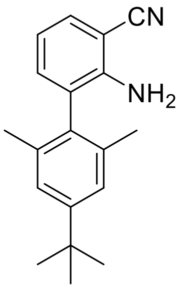

2-amino-4'-(*tert*-butyl)-2',6'-dimethyl-[1,1'-biphenyl]-3-carbonitrile (XXIII). It was prepared according to General Procedure A[8] using methyl 2-amino-3-bromobenzonitrile (207 mg, 1.00 mmol, 1 equiv.), **Ib** (208 mg, 1.01 mmol, 1.01 equiv.), $Pd_2(dba)_3$ (18.5 mg, 0.020 mmol, 2 mol%), Sphos (16.8 mg, 0.04 mmol, 4 mol%) and K_3PO_4 (649 mg, 3.00 mmol, 3 equiv.) in toluene (5 mL). The crude was purified by column chromatography over silica gel (hexane:EtOAc, 100:0 to 95:5) to obtain the product as a colorless solid (182 mg, 0.654 mmol, 65% yield). ^{1}H-NMR (400 MHz, $CDCl_3$) δ, ppm: 7.40 (dd, *J* = 7.6, 1.6 Hz, 1H), 7.17 (s, 2H), 7.13 (dd, *J* = 7.6, 1.6 Hz, 1H), 6.80 (t, *J* = 7.6 Hz, 1H), 4.20 (br, 2H), 2.03 (s, 6H), 1.35 (s, 9H). ^{13}C-NMR (100 MHz, $CDCl_3$) δ, ppm: 151.3, 147.3, 136.6, 134.8, 132.7, 131.4, 126.8, 125.1, 118.2, 118.0, 95.8, 34.5, 31.5, 20.4. HRMS (ESI-MS) *m/z*

calculated for $C_{19}H_{22}N_2$ $[M+H]^+$ 279.1856, found 279.1857. TLC (SiO_2, hexane:EtOAc 95:5), R_f = 0.19.

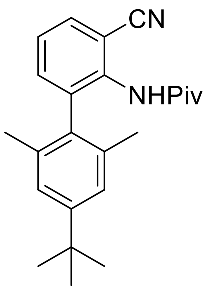

***N*-(4'-(*tert*-butyl)-3-cyano-2',6'-dimethyl-[1,1'-biphenyl]-2-yl) pivalamide (23)**. It was prepared according to General Procedure E using **XXIII** (182 mg, 0.654 mmol, 1 equiv.), pivaloyl chloride (97 mL, 0.783 mmol, 1.2 equiv.) and Et_3N (202 mL, 1.44 mmol, 2.2 equiv.) in CH_2Cl_2 (3.2 mL). The crude was purified by column chromatography over silica gel (hexane:EtOAc, 100:0 to 90:10) to obtain the product as a colorless solid (65.3 mg, 0.180 mmol, 28% yield). ^{1}H-NMR (400 MHz, $CDCl_3$) δ, ppm: 7.70 (dd, *J* = 7.3, 2.0 Hz, 1H), 7.48-7.35 (m, 2H), 7.16 (s, 2H), 6.69 (br, 1H), 1.94 (s, 6H), 1.32 (s, 9H), 0.98 (s, 9H). ^{13}C-NMR (100 MHz, $CDCl_3$) δ, ppm: 176.4, 151.9, 137.6, 137.4, 135.9, 134.3, 132.7, 132.4, 126.7, 125.1, 117.0, 111.8, 39.3, 34.6, 31.4, 27.0, 20.6. HRMS (ESI-MS) *m/z* calculated for $C_{24}H_{30}N_2O$ $[M+H]^+$ 363.2431, found 363.2429. TLC (SiO_2, hexane:EtOAc 9:1), R_f = 0.37. ***Note**: The reaction was performed in a crimpled vial at 50ºC for 3 days.*

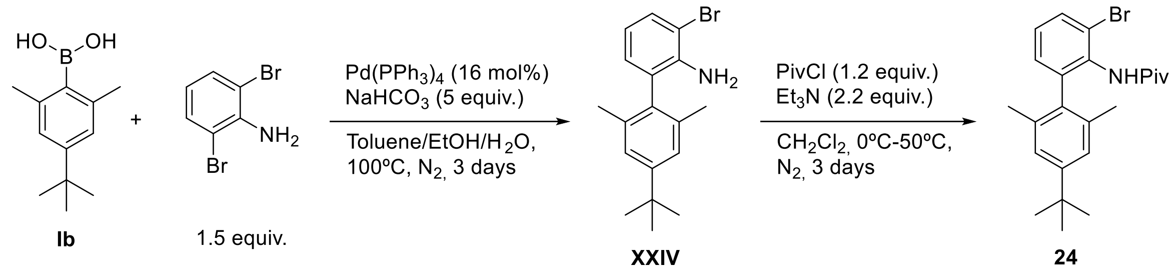

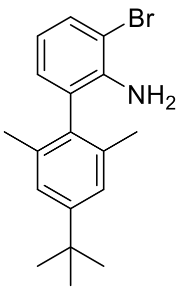

3-bromo-4'-(*tert*-butyl)-2',6'-dimethyl-[1,1'-biphenyl]-2-amine (XXIV). It was prepared according to General Procedure B using 2,6-dibromoaniline (2 mg, 1.50 mmol, 1.5 equiv.), **Ib** (206 mg, 1.00 mmol, 1 equiv.), $Pd_2(dba)_3$ (185 mg, 0.160 mmol, 16 mol%) and $NaHCO_3$ (420 mg, 5.00 mmol, 5 equiv.) in toluene / EtOH / H_2O (1:2:1 v/v, 20 mL). The crude was purified by column chromatography over silica gel (hexane) to obtain the product as a colorless solid (134 mg, 0.403 mmol, 40% yield). ^{1}H-NMR (400 MHz, $CDCl_3$) δ, ppm: 7.42 (dd, *J* = 7.8, 1.5 Hz, 1H), 7.16 (s, 2H), 6.90 (dd, *J* = 7.4, 1.5 Hz, 1H), 6.67 (t, *J* = 7.8 Hz, 1H), 3.75 (br, 2H), 2.05 (s, 6H), 1.36 (s, 9H). ^{13}C-NMR (100 MHz, $CDCl_3$) δ, ppm: 150.9, 140.9, 136.6, 134.2, 131.5, 129.3, 128.0, 125.0, 119.6, 109.8, 34.5, 31.5, 20.6. HRMS (ESI-MS) *m/z* calculated for $C_{18}H_{22}BrN$ $[M+H]^+$ 332.1008, found 332.1006. TLC (SiO_2, hexane), R_f = 0.52.

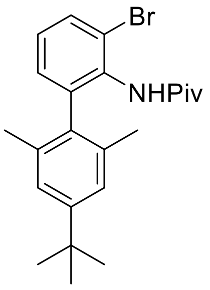

***N*-(3-bromo-4'-(*tert*-butyl)-2',6'-dimethyl-[1,1'-biphenyl]-2-yl) pivalamide (24)**. It was prepared according to General Procedure E using **XXIV** (85.8 mg, 0.258 mmol, 1 equiv.), pivaloyl chloride (38.5 mL, 0.309 mmol, 1.2 equiv.) and Et_3N (80.0 mL, 0.568 mmol, 2.2 equiv.) in CH_2Cl_2 (1.3 mL). The crude was purified by column chromatography over silica gel (hexane:EtOAc, 100:0 to 95:5) to obtain the product as a colorless solid (64.9 mg, 0.156 mmol, 60% yield). ^{1}H-NMR (400 MHz, $CDCl_3$) δ, ppm: 7.63

(dd, J = 7.8, 1.7 Hz, 1H), 7.24-7.15 (m, 2H), 7.11 (s, 2H), 6.56 (br, 1H), 2.01 (s, 6H), 1.31 (s, 9H), 0.93 (s, 9H). ^{13}C-NMR (100 MHz, $CDCl_3$) δ, ppm: 175.9, 150.8, 140.8, 135.7, 135.0, 134.4, 132.1, 129.3, 128.4, 124.6, 123.4, 39.1, 34.5, 31.5, 37.2, 20.7. HRMS (ESI-MS) *m/z* calculated for $C_{23}H_{30}BrNO$ $[M+H]^+$ 416.1584, found 416.1584. TLC (SiO_2, hexane:EtOAc 95:5), R_f = 0.17. ***Note**: The reaction was performed in a crimpled vial at 50ºC for 3 days.*

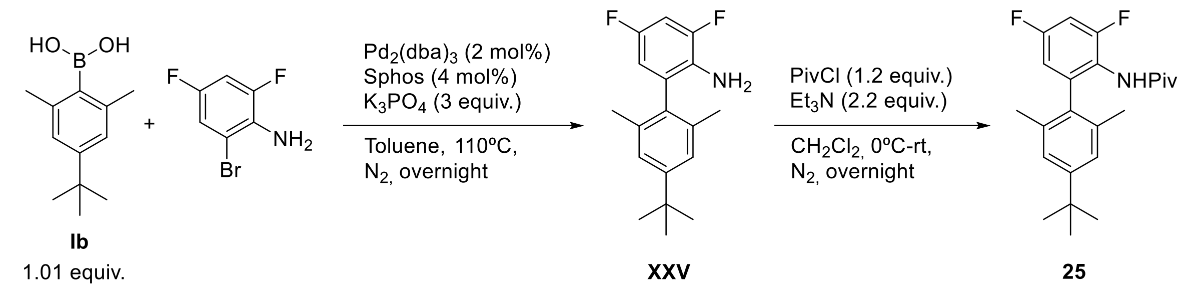

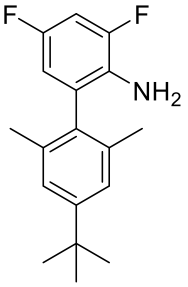

4'-(*tert*-butyl)-3,5-difluoro-2',6'-dimethyl-[1,1'-biphenyl]-2-amine (XXV). It was prepared according to General Procedure A[8] using 2-bromo-4,6-difluoroaniline (208 mg, 1.00 mmol, 1 equiv.), **Ib** (208 mg, 1.01 mmol, 1.01 equiv.), $Pd_2(dba)_3$ (18.5 mg, 0.020 mmol, 2 mol%), Sphos (16.8 mg, 0.04 mmol, 4 mol%) and K_3PO_4 (649 mg, 3.00 mmol, 3 equiv.) in toluene (5 mL). The crude was purified by column chromatography over silica gel (hexane:EtOAc, 100:0 to 96:4 to obtain the product as a colorless solid (135 mg, 0.468 mmol, 47% yield). ^{1}H-NMR (400 MHz, $CDCl_3$) δ, ppm: 7.18 (d, J = 0.7 Hz, 2H), 6.84-6.78 (m, 1H), 6.57 (ddd, J = 8.9, 2.9, 1.6 Hz, 1H), 3.35 (br, 2H), 2.08 (s, 6H), 1.37 (s, 9H). ^{13}C-NMR (100 MHz, $CDCl_3$) δ, ppm: 54.8 (dd, J = 238.3, 12.3 Hz), 151.2 (dd, J = 241.7, 12.5 Hz), 136.4, 132.8 (dd, J = 3.2, 1.7 Hz), 128.9 (dd, J = 8.9, 4.5 Hz), 128.7 (dd, J = 12.1, 2.8 Hz), 125.0, 111.7 (dd, J = 21.5, 3.3 Hz), 102.4 (dd, J = 26.4, 23.0 Hz), 34.5, 31.5, 20.4. ^{19}F-NMR (377 MHz, $CDCl_3$) δ, ppm: -124.9 (t, J = 8.7 Hz), -130.3 (d, J = 10.9 Hz). HRMS (ESI-MS) *m/z* calculated for $C_{18}H_{21}F_2N$ $[M+H]^+$ 290.1715, found 290.1712. TLC (SiO_2, hexane:EtOAc 9:1), R_f = 0.72.

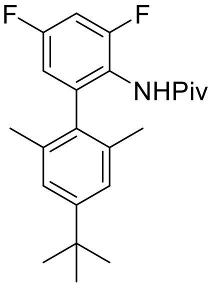

***N*-(4'-(*tert*-butyl)-3,5-difluoro-2',6'-dimethyl-[1,1'-biphenyl]-2-yl) pivalamide (25)**. It was prepared according to General Procedure E using **XXV** (135 mg, 0.468 mmol, 1 equiv.), pivaloyl chloride (70.0 mL, 0.561 mmol, 1.2 equiv.) and Et_3N (145 mL, 1.03 mmol, 2.2 equiv.) in CH_2Cl_2 (2.3 mL). The crude was purified by column chromatography over silica gel (hexane:EtOAc, 100:0 to 95:5) to obtain the product as a colorless solid (107 mg, 0.286 mmol, 61% yield). ^{1}H-NMR (400 MHz, $CDCl_3$) δ, ppm: 7.13 (s, 2H), 6.91 (ddd, J = 9.7, 8.6, 2.8 Hz, 1H), 6.75 (ddd, J = 8.4, 2.8, 1.5 Hz, 1H), 6.21 (br, 1H), 1.99 (s, 6H), 1.32 (s, 9H), 0.95 (s, 9H). ^{13}C-NMR (100 MHz, $CDCl_3$) δ, ppm: 176.5, 161.1 (dd, J = 248.3, 12.3 Hz), 158.4 (dd, J = 253.8, 13.2 Hz), 151.5, 140.4 (dd, J = 9.7, 2.8 Hz), 135.7, 132.9 (t, J = 2.2 Hz), 124.8, 119.5 (dd, J = 12.8, 3.8 Hz), 112.0 (dd, J = 21.8, 3.5 Hz), 103.7 (dd, J = 26.3, 24.5 Hz), 39.1, 34.5, 31.4, 27.2, 20.5. ^{19}F-NMR (377 MHz, $CDCl_3$) δ, ppm: -113.0 (q, J = 8.2 Hz), -113.9 (t, J = 8.6 Hz). HRMS (ESI-MS) *m/z* calculated for $C_{23}H_{29}F_2NO$ $[M+Na]^+$ 396.2109, found 396.2110. TLC (SiO_2, hexane:EtOAc 9:1), R_f = 0.25.

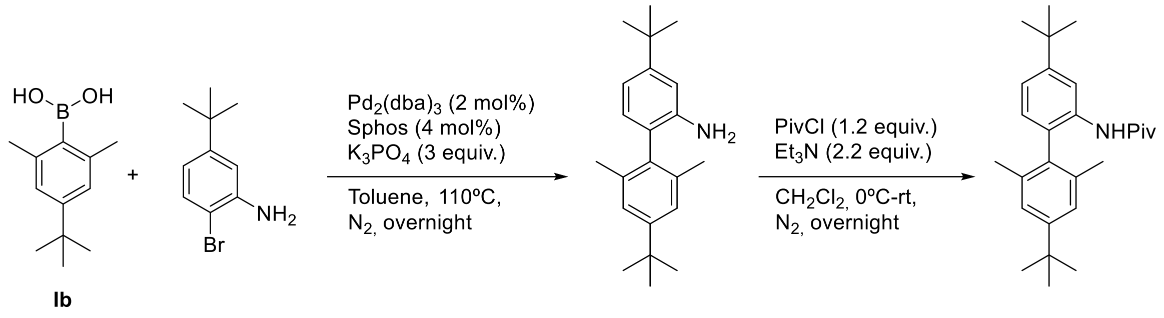

Ib 1.01 equiv. **XXVI** **26**

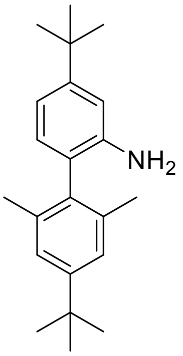

4,4'-di-tert-butyl-2',6'-dimethyl-[1,1'-biphenyl]-2-amine (XXVI). It was prepared according to General Procedure A[8] using 2-bromo-5-(*tert*-butyl)aniline (235 mg, 1.00 mmol, 1 equiv.), **Ib** (208 mg, 1.01 mmol, 1.01 equiv.), $Pd_2(dba)_3$ (18.5 mg, 0.020 mmol, 2 mol%), Sphos (16.8 mg, 0.04 mmol, 4 mol%) and K_3PO_4 (649 mg, 3.00 mmol, 3 equiv.) in toluene (5 mL). The crude was purified by column chromatography over silica gel (hexane:EtOAc, 100:0 to 95:5) to obtain the product as a colorless solid (196 mg, 0.633 mmol, 63% yield). ^{1}H-NMR (400 MHz, $CDCl_3$) δ, ppm: 7.20 (dd, *J* = 8.3, 2.4 Hz, 1H), 7.18 (s, 1H), 7.01 (d, *J* = 2.4 Hz, 1H), 6.75 (d, *J* = 8.3 Hz, 1H), 3.32 (br, 2H), 2.10 (s, 6H), 1.39 (s, 9H), 1.31 (s, 9H). ^{13}C-NMR (100 MHz, $CDCl_3$) δ, ppm: 150.2, 141.5, 141.1, 136.7, 135.7, 127.2, 126.2, 124.7, 124.6, 114.7, 34.5, 34.1, 31.8, 31.6, 20.7. HRMS (ESI-MS) *m/z* calculated for $C_{22}H_{31}N$ $[M+H]^+$ 310.2529, found 310.2520. TLC (SiO_2, hexane:EtOAc 95:5), R_f = 0.43.

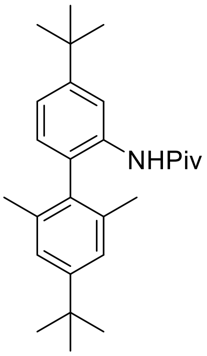

***N*-(4,4'-di-*tert*-butyl-2',6'-dimethyl-[1,1'-biphenyl]-2-yl)pivalamide (26).** It was prepared according to General Procedure E using **XXVI** (186 mg, 0.602 mmol, 1 equiv.), pivaloyl chloride (89.9 mL, 0.723 mmol, 1.2 equiv.) and Et_3N (187 mL, 1.32 mmol, 2.2 equiv.) in CH_2Cl_2 (3 mL). The crude was purified by column chromatography over silica gel (hexane:EtOAc, 100:0 to 95:5) to obtain the product as a colorless solid (124 mg, 0.315 mmol, 52% yield). ^{1}H-NMR (400 MHz, $CDCl_3$) δ, ppm: 8.31 (d, *J* = 8.6 Hz, 1H), 7.37 (dd, *J* = 8.6, 2.4 Hz, 1H), 7.21 (s, 2H), 7.14 (d, *J* = 2.4z Hz, 1H), 6.96 (br, 1H), 1.99 (s, 6H), 1.35 (s, 9H), 1.31 (s, 9H), 0.95 (s, 9H). ^{13}C-NMR (100 MHz, $CDCl_3$) δ, ppm: 176.2, 151.5, 146.9, 136.8, 134.0, 133.0, 129.7, 126.1, 125.0, 124.9, 119.4, 39.7, 34.6, 34.6, 31.6, 31.5, 27.2, 20.6. HRMS (ESI-MS) *m/z* calculated for $C_{27}H_{39}NO$ $[M+Na]^+$ 416.2924, found 416.2928. TLC (SiO_2, hexane:EtOAc 9:1), R_f = 0.41.

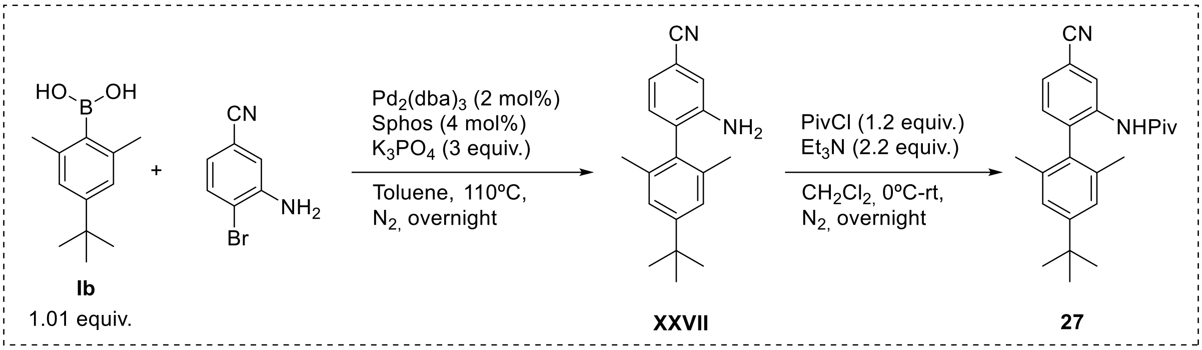

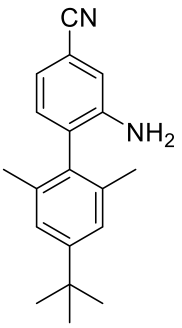

2-amino-4'-(*tert*-butyl)-2',6'-dimethyl-[1,1'-biphenyl]-4-carbonitrile (XXVII). It was prepared according to General Procedure A[8] using 3-amino-4-bromobenzonitrile (207 mg, 1.00 mmol, 1 equiv.), **Ib** (208 mg, 1.01 mmol, 1.01 equiv.), $Pd_2(dba)_3$ (18.5 mg, 0.020 mmol, 2 mol%), Sphos (16.8 mg, 0.04 mmol, 4 mol%) and K_3PO_4 (649 mg, 3.00 mmol, 3 equiv.) in toluene (5 mL). The crude was purified by column chromatography over silica gel (hexane:EtOAc, 100:0 to 95:5) to obtain the product as a colorless solid (156 mg, 0.560 mmol, 56% yield). ^{1}H-NMR (400 MHz, $CDCl_3$) δ, ppm: 7.15 (s, 2H), 7.08 (dd, *J* = 7.7, 1.5 Hz, 1H), 7.04-6.98 (m, 2H), 3.65 (br, 2H), 2.02 (s, 6H), 1.34 (s, 9H). ^{13}C-NMR (100 MHz, $CDCl_3$) δ, ppm: 151.2, 144.6, 136.1, 133.1, 131.2, 131.0, 125.1, 122.0, 119.6, 117.5, 111.7, 34.5, 31.5, 20.4. HRMS (ESI-MS) *m/z* calculated for $C_{19}H_{22}N_2$ $[M+H]^+$ 279.1856, found 279.1860. TLC (SiO_2, hexane:EtOAc 95:5), R_f = 0.26.

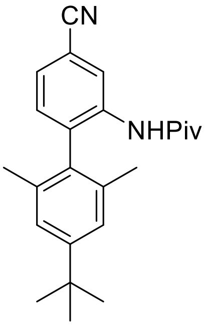

***N*-(4'-(*tert*-butyl)-4-cyano-2',6'-dimethyl-[1,1'-biphenyl]-2-yl) pivalamide (27)**. It was prepared according to General Procedure E using **XXVII** (156 mg, 0.559 mmol, 1 equiv.), pivaloyl chloride (83.5 mL, 0.671 mmol, 1.2 equiv.) and Et_3N (173 mL, 1.23 mmol, 2.2 equiv.) in CH_2Cl_2 (2.8 mL). The crude was purified by column chromatography over silica gel (hexane:EtOAc, 10:1) to obtain the product as a colorless solid (74.6 mg, 0.206 mmol, 37% yield). ^{1}H-NMR (400 MHz, $CDCl_3$) δ, ppm: 8.86 (d, *J* = 1.6 Hz, 1H), 7.44 (dd, *J* = 7.8, 1.6 Hz, 1H), 7.24-7.19 (m, 3H), 7.11 (br, 1H), 1.96 (s, 6H), 1.34 (s, 9H), 0.95 (s, 9H). ^{13}C-NMR (100 MHz, $CDCl_3$) δ, ppm: 176.7, 152.6, 136.6, 136.1, 134.8, 131.4, 130.0, 127.5, 125.4, 122.8, 118.9, 112.3, 39.9, 34.7, 31.4, 27.0, 20.4. HRMS (ESI-MS) *m/z* calculated for $C_{24}H_{30}N_2O$ $[M+H]^+$ 363.2431, found 363.2432. TLC (SiO_2, hexane:EtOAc 9:1), R_f = 0.30.

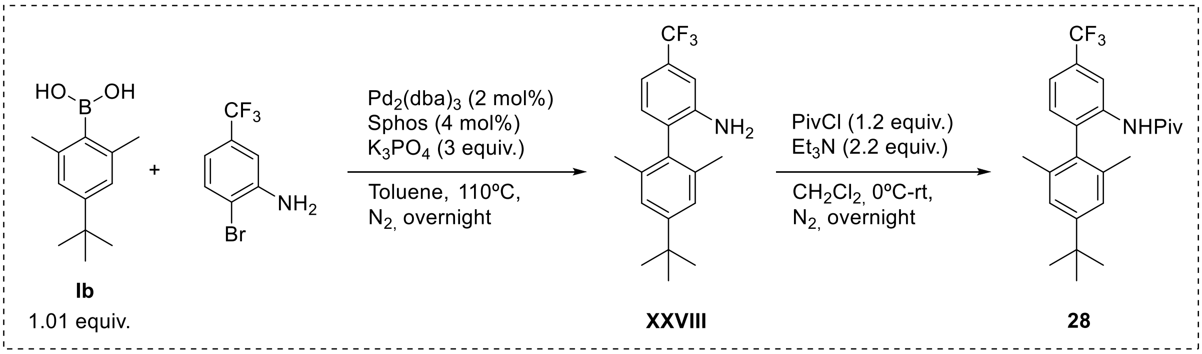

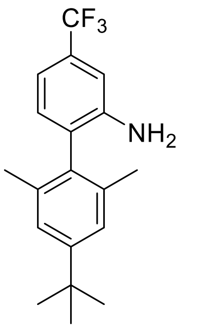

4'-(*tert*-butyl)-2',6'-dimethyl-4-(trifluoromethyl)-[1,1'-biphenyl]-2-amine (XXVIII). It was prepared according to General Procedure A[8] using 2-bromo-5-(trifluoromethyl)aniline (253 mg, 1.00 mmol, 1 equiv.), **Ib** (208 mg, 1.01 mmol, 1.01 equiv.), $Pd_2(dba)_3$ (18.5 mg, 0.020 mmol, 2 mol%), Sphos (16.8 mg, 0.04 mmol, 4 mol%) and K_3PO_4 (649 mg, 3.00 mmol, 3 equiv.) in toluene (5 mL). The crude was purified by column chromatography over silica gel (hexane:EtOAc, 100:0 to 95:5) to obtain the product as a colorless solid (125 mg, 0.389 mmol, 39% yield). ^{1}H-NMR (400 MHz, $CDCl_3$) δ, ppm: 7.16 (s, 2H), 7.07-7.03 (m, 2H), 7.01-6.97 (m, 1H), 3.61 (br, 2H), 2.04 (s, 6H), 1.35 (s, 9H). ^{13}C-NMR (100 MHz, $CDCl_3$) δ, ppm: 150.9, 144.2, 136.4, 133.6, 130.6, 130.4 (q, *J* = 32.0 Hz), 129.7, 125.0, 124.4 (q, *J* = 272.2 Hz), 115.0 (q, *J* = 3.9 Hz), 111.4 (q, *J* = 3.8 Hz), 34.5, 31.5, 20.5. ^{19}F-NMR (377 MHz, $CDCl_3$) δ, ppm: -63.6. HRMS (ESI-MS) *m/z* calculated for $C_{19}H_{22}F_3N$ $[M+H]^+$ 332.1777, found 332.1778. TLC (SiO_2, hexane:EtOAc 95:5), R_f = 0.41.

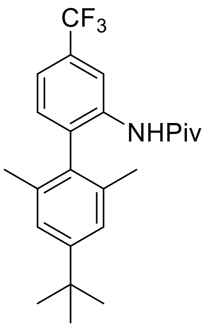

***N*-(4'-(*tert*-butyl)-2',6'-dimethyl-4-(trifluoromethyl)-[1,1'-biphenyl]-2-yl)pivalamide (28)**. It was prepared according to General Procedure E using **XXVIII** (125 mg, 0.388 mmol, 1 equiv.), pivaloyl chloride (57.8 mL, 0.465 mmol, 1.2 equiv.) and Et_3N (120 mL, 0.853 mmol, 2.2 equiv.) in CH_2Cl_2 (2 mL). The crude was purified by column chromatography over silica gel (hexane:EtOAc, 100:0 to 95:5) to obtain the product as a colorless solid (71.0 mg, 0.175 mmol, 45% yield). ^{1}H-NMR (400 MHz, $CDCl_3$) δ, ppm: 8.85-8.83 (m, 1H), 7.42 (ddd, *J* = 7.9, 1.8, 0.8 Hz, 1H), 7.26-7.21 (m, 3H), 7.14 (br, 1H), 1.98 (s, 6H), 1.35 (s, 9H), 0.96 (s, 9H). ^{13}C-NMR (100 MHz, $CDCl_3$) δ, ppm: 176.6, 152.3, 136.4, 136.2, 133.4, 132.0, 130.7 (q, *J* = 32.4 Hz), 129.5, 125.6, 124.2 (q, *J* = 272.3 Hz), 120.5 (q, *J* = 3.8 Hz), 116.6 (q, *J* = 4.0 Hz), 39.9, 34.7, 31.4, 27.1, 20.5. ^{19}F-NMR (377 MHz, $CDCl_3$) δ, ppm: -63.5. HRMS (ESI-MS) *m/z* calculated for $C_{24}H_{30}F_3NO$ $[M+Na]^+$ 428.2172, found 428.2169. TLC (SiO_2, hexane:EtOAc 9:1), R_f = 0.55.

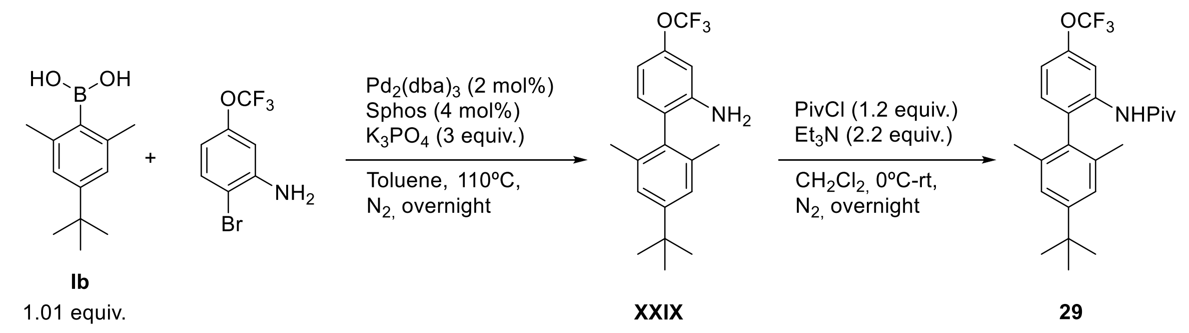

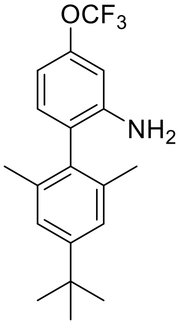

4'-(*tert*-butyl)-2',6'-dimethyl-4-(trifluoromethoxy)-[1,1'-biphenyl]-2-amine (XXIX). It was prepared according to General Procedure A[8] using 2-bromo-5-(trifluoromethoxy)aniline (269 mg, 1.00 mmol, 1 equiv.), **Ib** (208 mg, 1.01 mmol, 1.01 equiv.), $Pd_2(dba)_3$ (18.5 mg, 0.020 mmol, 2 mol%), Sphos (16.8 mg, 0.04 mmol, 4 mol%) and K_3PO_4 (649 mg, 3.00 mmol, 3 equiv.) in toluene (5 mL). The crude was purified by column chromatography over silica gel (hexane:EtOAc, 100:0 to 95:5) to obtain the product as a colorless solid (193 mg, 0.572 mmol, 57% yield). ^{1}H-NMR (400 MHz, $CDCl_3$) δ, ppm: 7.17 (s, 2H),

6.94 (d, J = 8.2 Hz, 1H), 6.70-6.64 (m, 1H), 6.65-6.61 (m, 1H), 3.55 (br, 2H), 2.06 (s, 6H), 1.36 (s, 9H). ^{13}C-NMR (100 MHz, $CDCl_3$) δ, ppm: 150.8, 149.4 (q, J = 1.8 Hz), 145.1, 136.8, 133.7, 131.0, 124.9, 124.8, 120.9 (q, J = 256.3 Hz), 110.3, 107.0, 34.5, 31.5, 20.5f. ^{19}F-NMR (377 MHz, $CDCl_3$) δ, ppm: -58.5. HRMS (ESI-MS) m/z calculated for $C_{19}H_{22}F_3NO$ $[M+H]^+$ 338.1726, found 338.1717. TLC (SiO_2, hexane:EtOAc 95:5), R_f = 0.42.

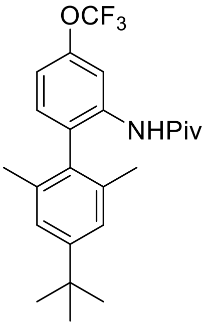

***N*-(4'-(*tert*-butyl)-2',6'-dimethyl-4-(trifluoromethoxy)-[1,1'-biphenyl]-2-yl)pivalamide (29).** It was prepared according to General Procedure E using **XXIX** (193 mg, 0.572 mmol, 1 equiv.), pivaloyl chloride (85.2 mL, 0.685 mmol, 1.2 equiv.) and Et_3N (177 mL, 1.26 mmol, 2.2 equiv.) in CH_2Cl_2 (3 mL). The crude was purified by column chromatography over silica gel (hexane:EtOAc, 100:0 to 95:5) to obtain the product as a colorless solid (172 mg, 0.408 mmol, 71% yield). ^{1}H-NMR (400 MHz, $CDCl_3$) δ, ppm: 8.50 (dd, J = 2.5, 1.1 Hz, 1H), 7.25 (s, 2H), 7.15 (d, J = 8.3 Hz, 1H), 7.12 (s, 1H), 7.04 (ddd, J = 8.3, 2.5, 1.1 Hz, 1H), 2.01 (s, 6H), 1.37 (s, 9H), 0.98 (s, 9H). ^{13}C-NMR (100 MHz, $CDCl_3$) δ, ppm: 176.5, 152.2, 149.0 (q, J = 1.9 Hz), 136.9, 136.8, 132.0, 129.9, 128.2, 125.3, 120.7 (q, J = 257.1 Hz), 115.8, 112.4. ^{19}F-NMR (377 MHz, $CDCl_3$) δ, ppm: -58.6. HRMS (ESI-MS) m/z calculated for $C_{24}H_{30}F_3NO_2$ $[M+H]^+$ 442.2301, found 422.2303. TLC (SiO_2, hexane:EtOAc 9:1), R_f = 0.58.

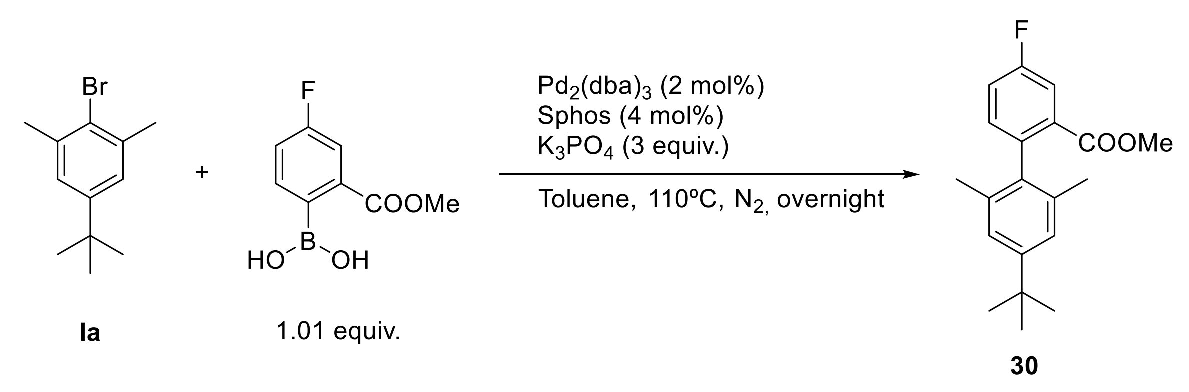

Methyl 4'-(*tert*-butyl)-4-fluoro-2',6'-dimethyl-[1,1'-biphenyl]-2-carboxylate (30). It was prepared according to General Procedure A[8] using **Ia** (241 mg, 1.00 mmol, 1 equiv.), (4-fluoro-2-(methoxycarbonyl)phenyl)boronic acid (204 mg, 1.01 mmol, 1.01 equiv.), $Pd_2(dba)_3$ (18.5 mg, 0.020 mmol, 2 mol%), Sphos (16.8 mg, 0.04 mmol, 4 mol%) and K_3PO_4 (649 mg, 3.00 mmol, 3 equiv.) in toluene (5 mL). The crude was purified by column chromatography over silica gel (hexane:EtOAc, 100:0 to 94:6) to obtain the product as a colorless solid (113 mg, 0.359 mmol, 36% yield). ^{1}H-NMR (400 MHz, $CDCl_3$) δ, ppm: 7.72 (dd, J = 9.3, 2.8 Hz, 1H), 7.34-7.22 (m, 1H), 7.21-7.13 (m, 1H), 7.11 (s, 2H), 3.62 (s, 3H), 1.96 (s, 6H), 1.37 (s, 9H). ^{13}C-NMR (100 MHz, $CDCl_3$) δ, ppm: 166.4 (d, J = 2.8 Hz), 161.3 (d, J = 246.6 Hz), 149. 8, 138.4 (d, J = 3.7 Hz), 137.1, 134.9, 132.6 (d, J = 7.3 Hz), 132.1 (d, J = 7.1 Hz), 124.0, 119.2 (d, J = 20.9 Hz), 117.1 (d, J = 23.1 Hz), 52.1, 34.3, 31.5, 20.9. ^{19}F-NMR (377 MHz, $CDCl_3$) δ, ppm: -116.0 (td, J = 8.7, 5.6 Hz). HRMS (ESI-MS) m/z calculated for $C_{20}H_{23}FO_2$ $[M+Na]^+$ 337.1574, found 337.1578. TLC (SiO_2, hexane:EtOAc 95:5) R_f = 0.43.

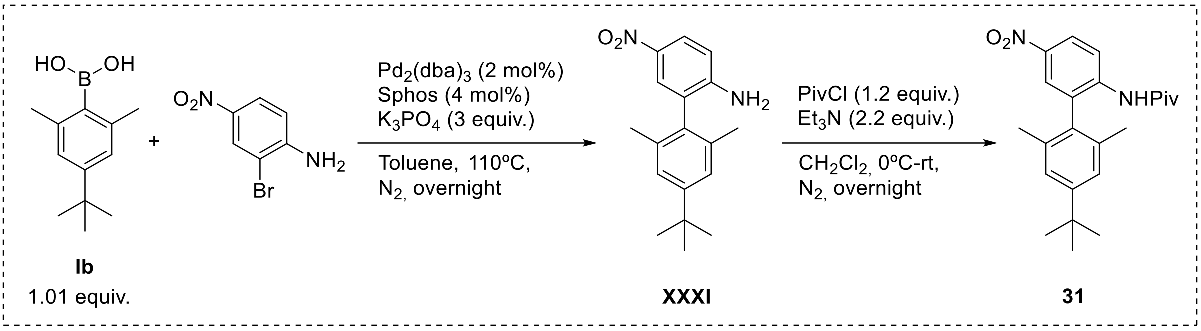

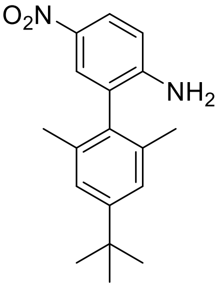

4'-(*tert*-butyl)-2',6'-dimethyl-5-nitro-[1,1'-biphenyl]-2-amine (XXXI). It was prepared according to General Procedure A[8] using 2-bromo-4-nitroaniline (298 mg, 1.00 mmol, 1 equiv.), **Ib** (208 mg, 1.01 mmol, 1.01 equiv.), $Pd_2(dba)_3$ (18.5 mg, 0.020 mmol, 2 mol%), Sphos (16.8 mg, 0.04 mmol, 4 mol%) and K_3PO_4 (649 mg, 3.00 mmol, 3 equiv.) in toluene (5 mL). The crude was purified by column chromatography over silica gel (hexane:EtOAc, 100:0 to 82:12) to obtain the product as a yellow solid (133 mg, 0.446 mmol, 45% yield). ^{1}H-NMR (400 MHz, $CDCl_3$) δ, ppm: 8.09 (dd, *J* = 8.9, 2.6 Hz, 1H), 7.91 (d, *J* = 2.6 Hz, 1H), 7.17 (s, 2H), 6.74 (d, *J* = 8.9 Hz, 1H), 4.23 (br, 2H), 2.05 (s, 6H), 1.35 (s, 9H). ^{13}C-NMR (100 MHz, $CDCl_3$) δ, ppm: 151.5, 150.4, 139.1, 136.6, 132.1, 126.9, 125.3, 125.2, 113.4, 34.5, 31.4, 20.4. HRMS (ESI-MS) *m/z* calculated for $C_{18}H_{22}N_2O_2$ $[M+Na]^+$ 321.1573, found 321.1571. TLC (SiO_2, hexane:EtOAc 9:1), R_f = 0.24.

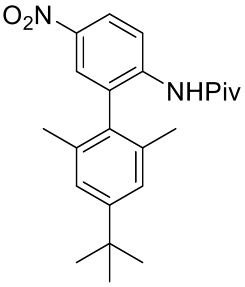

***N*-(4'-(*tert*-butyl)-2',6'-dimethyl-5-nitro-[1,1'-biphenyl]-2-yl) pivalamide (31)**. It was prepared according to General Procedure E using **XXXI** (121 mg, 0.404 mmol, 1 equiv.), pivaloyl chloride (89.3 mL, 0.485 mmol, 1.2 equiv.) and Et_3N (125 mL, 0.888 mmol, 2.2 equiv.) in CH_2Cl_2 (2 mL). The crude was purified by column chromatography over silica gel (hexane:EtOAc, 100:0 to 95:5) to obtain the product as a pale yellow solid (59.3 mg, 0.155 mmol, 38% yield). ^{1}H-NMR (400 MHz, $CDCl_3$) δ, ppm: 8.72 (d, *J* = 9.2 Hz, 1H), 8.30 (dd, *J* = 9.2, 2.7 Hz, 1H), 8.08 (d, *J* = 2.7 Hz, 1H), 7.36 (br, 1H), 7.28 (s, 2H), 2.02 (s, 6H), 1.38 (s, 9H), 1.00 (s, 9H). ^{13}C-NMR (100 MHz, $CDCl_3$) δ, ppm: 176-7, 152.7, 143.2, 141.6, 136.4, 130.7, 130.4, 125.4, 124.8, 124.5, 118.8, 40.0, 34.6, 31.2, 26.9, 20.4. HRMS (ESI-MS) *m/z* calculated for $C_{23}H_{30}N_2O_3$ $[M+H]^+$ 383.2329, found 383.2332. TLC (SiO_2, hexane:EtOAc 9:1), R_f = 0.33.

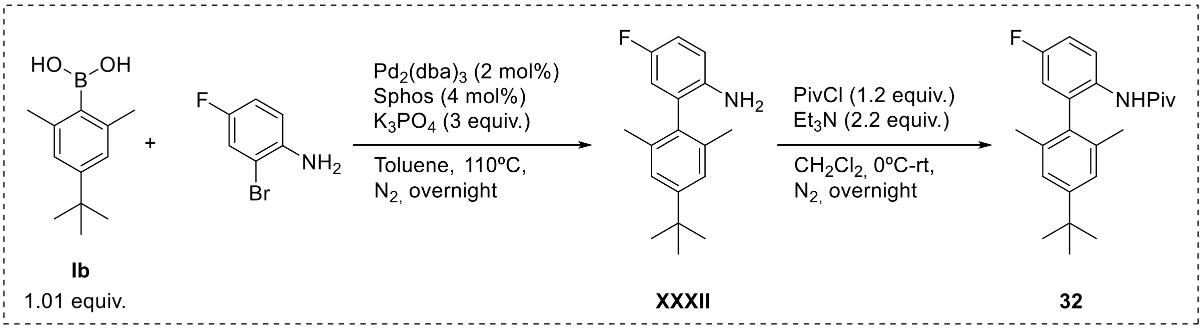

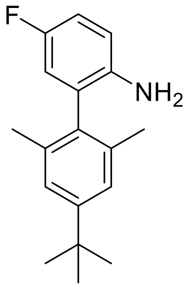

4'-(*tert*-butyl)-5-fluoro-2',6'-dimethyl-[1,1'-biphenyl]-2-amine (XXXII). It was prepared according to General Procedure A[8] using 2-bromo-4-fluoroaniline (323 mg, 1.67 mmol, 1 equiv.), **Ib** (347 mg, 1.68 mmol, 1.01 equiv.), $Pd_2(dba)_3$ (30.8 mg, 0.033 mmol, 2 mol%), Sphos (27.9 mg, 0.066 mmol, 4 mol%) and K_3PO_4 (1.08 g, 5.00 mmol, 3 equiv.) in toluene (8.4 mL). The crude was purified by column chromatography over silica gel (hexane:EtOAc, 100:0 to 90:10) to obtain the product as a colorless solid (199 mg, 0.733 mmol, 44% yield). ^{1}H-NMR (400 MHz, $CDCl_3$) δ, ppm: 7.14 (s, 2H), 6.91-6.83 (m, 1H), 6.74-6.68 (m, 2H), 2.06 (s, 6H), 1.34 (s, 9H). ^{13}C-NMR (100 MHz, $CDCl_3$) δ, ppm: 157.1 (d, J = 237.7 Hz), 150.9, 138.1, 136.5, 133.6, 129.0 (d, J = 7.5 Hz), 125.0, 117.2 (d, J = 8.2 Hz), 116.5 (d, J = 21.7 Hz), 114.8 (d, J = 22.3 Hz), 34.5, 31.5, 20.5. ^{19}F-NMR (377 MHz, $CDCl_3$) δ, ppm: -128.0 (td, J = 8.7, 4.8 Hz). HRMS (ESI-MS) *m/z* calculated for $C_{18}H_{22}FN$ $[M+H]^+$ 272.1809, found 272.1813. TLC (SiO_2, hexane:EtOAc 9:1), R_f = 0.41.

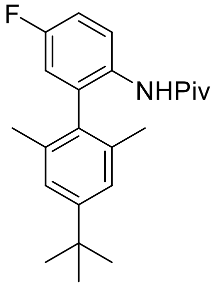

***N*-(4'-(*tert*-butyl)-5-fluoro-2',6'-dimethyl-[1,1'-biphenyl]-2-yl) pivalamide (32).** It was prepared according to General Procedure E using **XXXII** (102 mg, 0.375 mmol, 1 equiv.), pivaloyl chloride (56.0 mL, 0.451 mmol, 1.2 equiv.) and Et_3N (116 mL, 0.826 mmol, 2.2 equiv.) in CH_2Cl_2 (2 mL). The crude was purified by column chromatography over silica gel (hexane:EtOA, 100:0 to 92:8) to obtain the product as a colorless solid (53.5 mg, 0.150 mmol, 40% yield). ^{1}H-NMR (400 MHz, $CDCl_3$) δ, ppm: 8.40 (dd, J = 9.1, 5.4 Hz, 1H), 7.21 (s, 2H), 7.09-7.01 (m, 1H), 6.94 (s, 1H), 6.85 (dd, J = 8.7, 3.0 Hz, 1H), 1.99 (s, 6H), 1.34 (s, 9H), 0.94 (s, 9H). ^{13}C-NMR (100 MHz, $CDCl_3$) δ, ppm: 176.2, 159.0 (d, J = 243.5 Hz), 152.0, 132.3 (d, J = 1.5 Hz), 132.1 (d, J = 7.7 Hz), 131.9 (d, J = 2.8 Hz), 125.2, 121.4 (d, J = 8.0 Hz), 115.8 (d, J = 22.2 Hz), 114.7 (d, J = 21.8 Hz), 39.7, 34.6, 31.4, 27.1, 20.4. ^{19}F-NMR (377 MHz, $CDCl_3$) δ, ppm: -119.7 (td, J = 8.5, 5.3 Hz). HRMS (ESI-MS) *m/z* calculated for $C_{23}H_{30}FNO$ $[M+H]^+$ 356.2384, found 356.2385. TLC (SiO_2, hexane:EtOAc 9:1), R_f = 0.33.

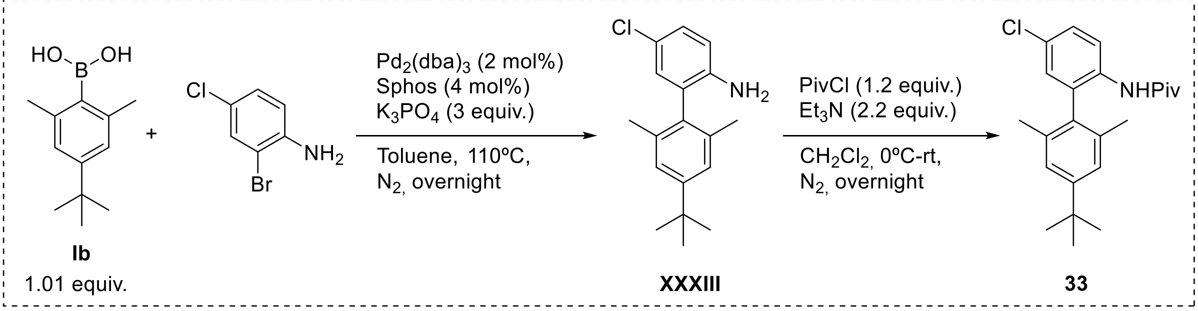

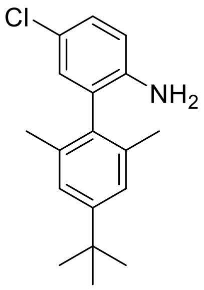

4'-(*tert*-butyl)-5-chloro-2',6'-dimethyl-[1,1'-biphenyl]-2-amine (XXXIII). It was prepared according to General Procedure A[8] using 2-bromo-4-chloroxyaniline (211 mg, 1.00 mmol, 1 equiv.), **Ib** (208 mg, 1.01 mmol, 1.01 equiv.), $Pd_2(dba)_3$ (18.5 mg, 0.020 mmol, 2 mol%), Sphos (16.8 mg, 0.04 mmol, 4 mol%) and K_3PO_4 (649 mg, 3.00 mmol, 3 equiv.) in toluene (5 mL). The crude was purified by column chromatography over silica gel (hexane:EtOAc, 100:0 to 95:5) to obtain the product as a colorless solid (144 mg, 0.500 mmol, 50% yield). ^{1}H-NMR (400 MHz, $CDCl_3$) δ, ppm: 7.15 (s, 2H), 7.11 (dd, J = 8.5, 2.5 Hz, 1H), 6.94 (d, J = 2.5 Hz, 1H), 6.70 (d, J = 8.5 Hz, 1H), 3.43 (br,

2H), 2.07 (s, 6H), 1.35 (s, 9H). ^{13}C-NMR (100 MHz, $CDCl_3$) δ, ppm: 150.8, 142.5, 136.5, 133.7, 129.7, 128.0, 127.9, 124.9, 122.9, 116.1, 34.5, 31.5, 20.5. HRMS (ESI-MS) *m/z* calculated for $C_{18}H_{22}ClN$ [M+H]$^+$ 288.1514, found 288.1518. TLC (SiO_2, hexane:EtOAc 9:1), R_f = 0.46.

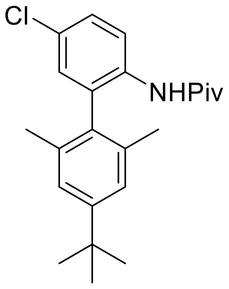

***N*-(4'-(*tert*-butyl)-5-chloro-2',6'-dimethyl-[1,1'-biphenyl]-2-yl) pivalamide (33)**. It was prepared according to General Procedure E using **XXXIII** (144 mg, 0.500 mmol, 1 equiv.), pivaloyl chloride (74.4 mL, 0.600 mmol, 1.2 equiv.) and Et_3N (154 mL, 1.10 mmol, 2.2 equiv.) in CH_2Cl_2 (2.5 mL). The crude was purified by column chromatography over silica gel (hexane:EtOAc, 95:5) to obtain the product as a colorless solid (72.7 mg, 0.195 mmol, 39% yield). ^{1}H-NMR (400 MHz, $CDCl_3$) δ, ppm: 8.41 (d, *J* = 8.9 Hz, 1H), 7.33 (dd, *J* = 8.8, 2.5 Hz, 1H), 7.20 (s, 2H), 7.11 (d, *J* = 2.5 Hz, 1H), 7.00 (br, 1H), 1.99 (s, 6H), 1.34 (s, 9H), 0.94 (s, 9H). ^{13}C-NMR (100 MHz, $CDCl_3$) δ, ppm: 176.3, 152.1, 136.5, 134.4, 132.0, 131.8, 128.9, 128.7, 128.3, 125.2, 120.9, 39.8, 34.6, 31.4, 27.1, 20.5. HRMS (ESI-MS) *m/z* calculated for $C_{23}H_{30}ClNO$ [M+H]$^+$ 372.2089, found 372.2082. TLC (SiO_2, hexane:EtOAc 9:1), R_f = 0.41.

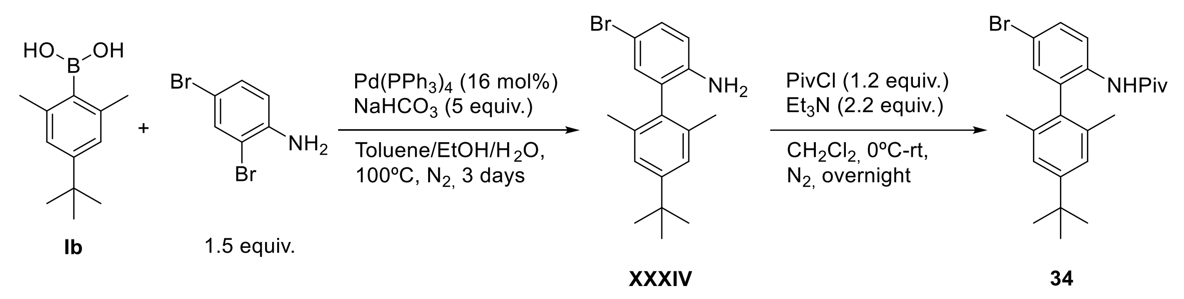

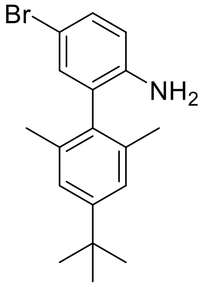

5-bromo-4'-(*tert*-butyl)-2',6'-dimethyl-[1,1'-biphenyl]-2-amine (XXXIV). It was prepared according to General Procedure B using 2,4-dibromoaniline (384 mg, 1.50 mmol, 1.5 equiv.), **Ib** (206 mg, 1.00 mmol, 1 equiv.), $Pd_2(dba)_3$ (189 mg, 0.160 mmol, 16 mol%) and $NaHCO_3$ (420 mg, 5.00 mmol, 5 equiv.) in toluene / EtOH / H_2O (1:2:1 v/v, 20 mL). The crude was purified by column chromatography over silica gel (hexane:EtOAc, 90:10) to obtain the product as a colorless solid (92.3 mg, 0.278 mmol, 28% yield). ^{1}H-NMR (400 MHz, $CDCl_3$) δ, ppm: 7.24 (dd, *J* = 8.6, 2.5 Hz, 1H), 7.14 (s, 2H), 7.08 (d, *J* = 2.3 Hz, 1H), 6.66 (d, *J* = 8.5 Hz, 1H), 3.24 (br, 2H), 2.06 (s, 6H), 1.34 (s, 9H). ^{13}C-NMR (100 MHz, $CDCl_3$) δ, ppm: 150.8, 142.9, 136.5, 133.6, 132.5, 130.9, 128.5, 124.9, 116.6, 110.1, 34.5, 31.5, 20.6. HRMS (ESI-MS) *m/z* calculated for $C_{18}H_{22}BrN$ [M+H]$^+$ 332.1008, found 332.1013. TLC (SiO_2, hexane:EtOAc 9:1), R_f = 0.38.

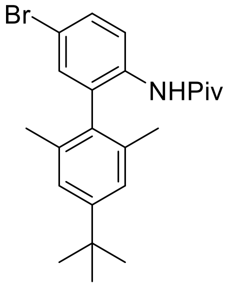

***N*-(5-bromo-4'-(*tert*-butyl)-2',6'-dimethyl-[1,1'-biphenyl]-2-yl) pivalamide (34)**. It was prepared according to General Procedure E using **XXXIV** (92.3 mg, 0.278 mmol, 1 equiv.), pivaloyl chloride (41.6 mL, 0.334 mmol, 1.2 equiv.) and Et_3N (86.2 mL, 0.612 mmol, 2.2 equiv.) in CH_2Cl_2 (1 mL). The crude was purified by column chromatography over silica gel (hexane:EtOAc 100:0 to 95:5) to obtain the product as a colorless solid (48.7 mg, 0.117 mmol, 42% yield). ^{1}H-NMR (400 MHz, $CDCl_3$) δ, ppm: 8.36 (d, *J* = 8.8 Hz, 1H), 7.49 (d, *J* = 2.4 Hz, 1H), 7.26 (m, 1H)*, 7.20 (s,

2H), 7.00 (br, 1H), 1.99 (s, 6H), 1.34 (s, 9H), 0.94 (s, 9H). ^{13}C-NMR (100 MHz, $CDCl_3$) δ, ppm: 176.4, 152.2, 136.6, 134.9, 132.1, 131.9, 131.7, 131.3, 125.2, 121.3, 116.4, 39.8, 34.6, 31.4, 27.1, 20.5. HRMS (ESI-MS) *m/z* calculated for $C_{23}H_{30}BrNO$ $[M+H]^+$ 416.1584, found 416.1576. TLC (SiO_2, hexane:EtOAc 91:9), R_f = 0.39. **Signal overlapped with the residual $CHCl_3$ (confirmed by HSQC).*

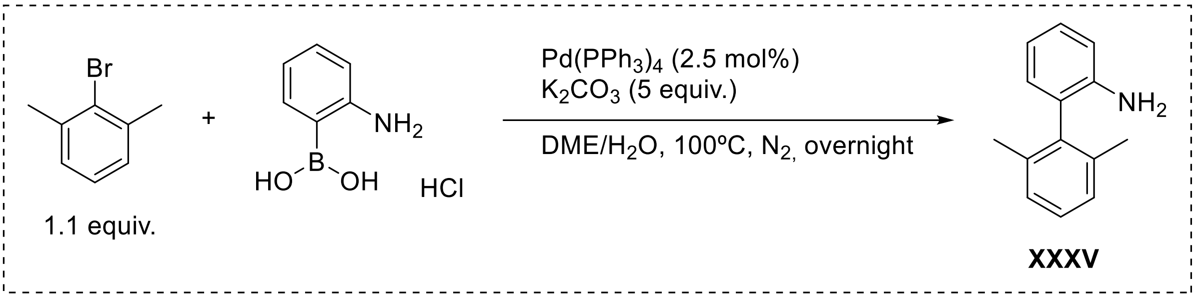

2',6'-dimethyl-[1,1'-biphenyl]-2-amine (XXV). It was prepared according to a modified reported procedure.[26] A Schlenk tube containing a magnetic stir bar was charged with 2-bromo-1,3-dimethylbenzene (1.17 g, 6.32 mmol, 1.1 equiv.), (2-aminophenyl)boronic acid hydrochloride (997 mg, 5.75 mmol, 1 equiv.), $Pd(PPh_3)_4$ (169 mg, 0.144 mmol, 2.5 mol%) and K_2CO_3 (3.97 g, 28.7 mmol, 5 equiv.) and dissolved in a mixture of DME/H_2O (2:1 v/v, 7.2 mL, 0.8 M) under N_2 atmosphere. The reaction was stirred at 100ºC overnight. The reaction was diluted with H_2O (50 mL) and the product was extracted with EtOAc (3x50 mL). The organic phases were washed with brine, dried over anhydrous $MgSO_4$, filtered and the solvent was evaporated under reduced pressure. The crude was purified by column chromatography over silica gel (hexane:EtOAc, 100:0 to 95:5) to obtain the product as a colorless solid (413 mg, 2.09 mmol, 36% yield). Spectral data match those reported in literature.[30] ^{1}H-NMR (400 MHz, $CDCl_3$) δ, ppm: 7.25-7.15 (m, 4H), 6.96 (dd, J = 7.5, 1.6 Hz, 1H), 6.86 (td, J = 7.4, 1.2 Hz, 1H), 6.81 (dd, J = 8.0, 1.2 Hz, 1H), 3.41 (br, 2H), 2.10 (s, 6H). ^{13}C-NMR (100 MHz, $CDCl_3$) δ, ppm: 143.5, 138.0, 137.3, 129.8, 128.3, 127.7, 127.6, 126.3, 118.6, 115.2, 20.3. HRMS (ESI-MS) *m/z* calculated for $C_{14}H_{15}N$ $[M+H]^+$ 198.1277, found 198.1272. TLC (SiO_2, hexane:EtOAc 95:5), R_f = 0.24. ***Note**: DME and H_2O was degassed with a N_2 flux for several minutes before adding to the reaction.*

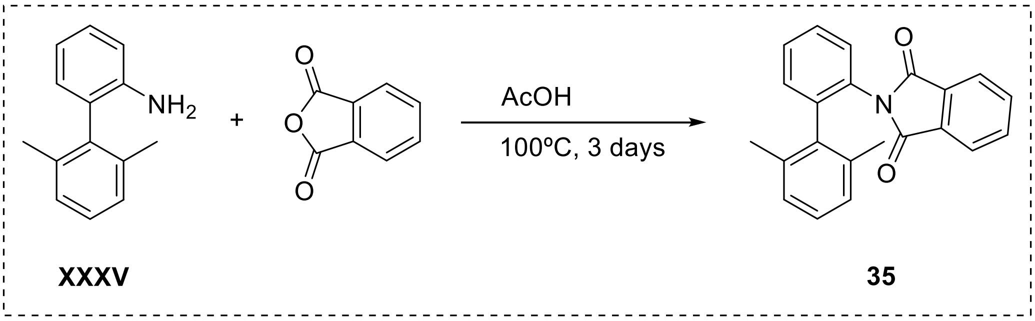

2-(2',6'-dimethyl-[1,1'-biphenyl]-2-yl)isoindoline-1,3-dione (35). It was prepared according to General Procedure F using **XXXV** (227 mg, 1.15 mmol, 1 equiv.) and phthalic anhydride (172 mg, 1.15 mmol, 1 equiv.) in acetic acid (2.3 mL). The crude was purified by column chromatography over silica gel (hexane:EtOAc, 100:0 to 90:10) to obtain the product as a colorless solid (220 mg, 0.672 mmol, 58% yield). ^{1}H-NMR (400 MHz, $CDCl_3$) δ, ppm: 7.78 (dd,

J = 5.5, 3.1 Hz, 2H), 7.66 (dd, J = 5.5, 3.01 Hz, 2H), 7.59-7.49 (m, 2H), 7.43-7.38 (m, 1H), 7.37-7.31 (m, 1H), 7.06-6.96 (m, 3H), 2.13 (s, 6H). ^{13}C-NMR (100 MHz, $CDCl_3$) δ, ppm: 167.1, 140.3, 137.4, 136.9, 134.1, 131.7, 131.5, 130.8, 129.5, 129.4, 128.2, 127.4, 127.3, 123.5. HRMS (ESI-MS) *m/z* calculated for $C_{22}H_{17}NO_2$ $[M+H]^+$ 328.1332, found 328.1332. TLC (SiO_2, hexane:EtOAc 8:2), R_f = 0.40.

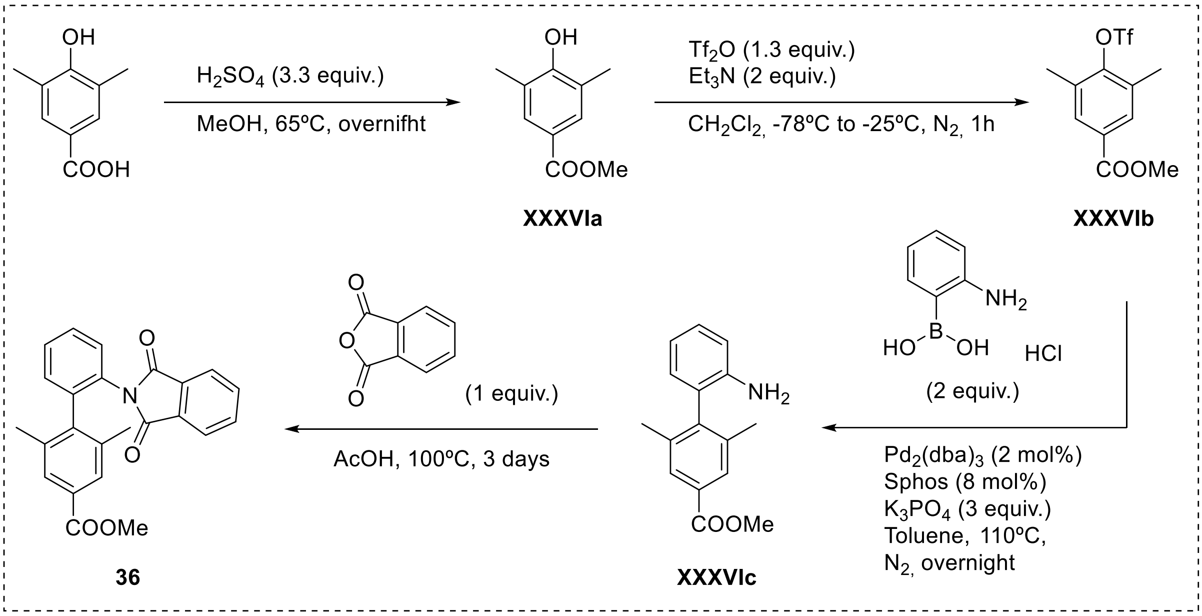

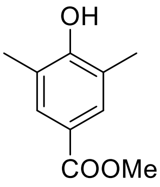

Methyl 4-hydroxy-3,5-dimethylbenzoate (XXXVIa). It was prepared according to a modified reported procedure.[18] 4-hydroxy-3,5-dimethylbenzoic acid (2.04 g, 12.3 mmol, 1 equiv.) was dissolved in MeOH (41 mL, 0.3M) and concentrated H_2SO_4 (2.29 mL, 40.8 mmol, 3.3 equiv.) was added. The reaction was heated at reflux (65ºC) overnight. The solvent was then evaporated under reduced pressure. The residue was diluted with EtOAc (50 mL) and washed with H_2O, saturated $NaHCO_3$ solution and brine. The combined organic phases were dried over anhydrous $MgSO_4$, filtered and the solvent was evaporated under reduced pressure. The product was obtained as a colorless solid (1.65 g, 9.16 mmol, 74% yield) and was used in the next step without further purification. Spectral data match those reported in literature.[18] ^{1}H-NMR (400 MHz, $CDCl_3$) δ, ppm: 7.69 (s, 2H), 5.83 (br, 1H), 3.87 (s, 3H), 2.26 (s, 9H). ^{13}C-NMR (100 MHz, $CDCl_3$) δ, ppm: 167.7, 156.9, 130.6, 123.3, 121.6, 52.0, 16.0. HRMS (ESI-MS) *m/z* calculated for $C_{10}H_{12}O_3$ $[M+H]^+$ 181.0859, found 181.0856. TLC (SiO_2, hexane:EtOAc 9:1), R_f = 0.22.

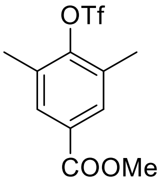

Methyl 3,5-dimethyl-4-(((trifluoromethyl)sulfonyl)oxy)benzoate (XXXVIb). It was prepared according to a modified reported procedure.[18] Under N_2 atmosphere, **XXXVIa** (1.65 g, 9.15 mmol, 1 equiv.) was dissolved in anhydrous CH_2Cl_2 (18 mL, 0.5 M) and Et_3N (2.58 mL, 18.3 mmol, 1.3 equiv.) was added. The mixture was cooled to -78ºC and a solution of Tf_2O (2.04 mL, 11.9 mmol, 1.3 equiv.) in anhydrous CH_2Cl_2 (3.3 mL, 3.6 M) was carefully added. The mixture was stirred at -25ºC for 1 hour. The reaction was quenched with saturated $NaHCO_3$ (20 mL) and the phases were separated. The organic phase was washed with brine, dried over anhydrous $MgSO_4$, filtered and the solvent was evaporated under reduced pressure. The crude was purified by column chromatography over silica gel (hexane:EtOAc, 100:00 to 91:9) to afford

the product as a colorless solid (2.44 g, 7.81 mmol,85% yield). Spectral data match those reported in literature.[18] ^{1}H-NMR (400 MHz, $CDCl_3$) δ, ppm: 7.81 (s, 2H), 3.91 (s, 3H), 2.43 (s, 6H). ^{13}C-NMR (100 MHz, $CDCl_3$) δ, ppm: 166.0, 150.0, 132.1, 131.3, 129.8, 118.7 (q, *J* = 319.8), 52.5, 17.3. ^{19}F-NMR (377 MHz, $CDCl_3$) δ, ppm: -74.3. HRMS (ESI-MS) *m/z* calculated for $C_{11}H_{11}F_3O_5S$ $[M+H]^+$ 313.0352, found 313.0352. TLC (SiO_2, hexane:EtOAc 95:5), R_f = 0.29.

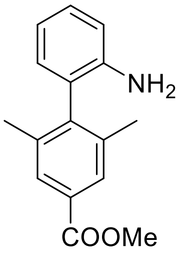

Methyl 2'-amino-2,6-dimethyl-[1,1'-biphenyl]-4-carboxylate (XXXVIc). It was prepared according to General Procedure A[7] using **XXXVIb** (590 mg, 1.89 mmol, 1 equiv.), (2-aminophenyl)boronic acid hydrochloride (572 mg, 3.78 mmol, 2 equiv.), $Pd_2(dba)_3$ (34.9 mg, 0.0378 mmol, 2 mol%), Sphos (63.3 mg, 0.151 mmol, 8 mol%) and K_3PO_4 (1.23 g, 5.67 mmol, 3 equiv.) in toluene (9.5 mL). The crude was purified by column chromatography over silica gel (hexane:EtOAc, 100:0 to 85:15) to obtain the product as a colorless solid (269 mg, 1.05 mmol, 56% yield). ^{1}H-NMR (400 MHz, $CDCl_3$) δ, ppm: 7.81 (s, 2H), 7.19 (ddd, *J* = 8.0, 7.1, 1.9 Hz, 1H), 6.90-6.77 (m, 3H), 3.92 (s, 3H), 3.36 (br, 2H), 2.10 (s, 6H). ^{13}C-NMR (100 MHz, $CDCl_3$) δ, ppm: 167.4, 143.1, 142.9, 137.8, 129.2, 129.2, 128.8, 128.7, 125.3, 118.8, 115.4, 52.1, 20.3. HRMS (ESI-MS) *m/z* calculated for $C_{16}H_{17}NO_2$ $[M+H]^+$ 256.1332, found 256.1331. TLC (SiO_2, hexane:EtOAc 8:2), R_f = 0.26.

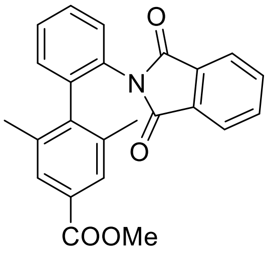

Methyl 2'-(1,3-dioxoisoindolin-2-yl)-2,6-dimethyl-[1,1'-biphenyl]-4-carboxylate (36). It was prepared according to General Procedure F using **XXXVIc** (159 mg, 0.624 mmol, 1 equiv.) and phthalic anhydride (93.4 mg, 0.624 mmol, 1 equiv.) in acetic acid (1.25 mL). The crude was purified by column chromatography over silica gel (hexane:EtOAc, 100:0 to 90:10) to obtain the product as a colorless solid (117 mg, 0.304 mmol, 49% yield). ^{1}H-NMR (400 MHz, $CDCl_3$) δ, ppm: 7.76 (dd, *J* = 5.5, 3.1 Hz, 2H), 7.68 (dd, *J* = 5.5, 3.1 Hz, 2H), 7.65 (s, 2H), 7.58-7.52 (m, 2H), 7.41-7.37 (m, 1H), 7.31-7.27 (m, 1H), 3.85 (s, 3H), 2.13 (s, 6H). ^{13}C-NMR (100 MHz, $CDCl_3$) δ, ppm: 167.3, 167.0, 142.3, 139.3, 137.5, 134.2, 131.6, 130.9, 130.4, 129.6, 129.4, 129.0, 128.6, 128.4, 123.6, 52.0, 20.5. HRMS (ESI-MS) *m/z* calculated for $C_{24}H_{19}NO_4$ $[M+H]^+$ 386.1387, found 386.1382. TLC (SiO_2, hexane:EtOAc 8:2), R_f = 0.32.

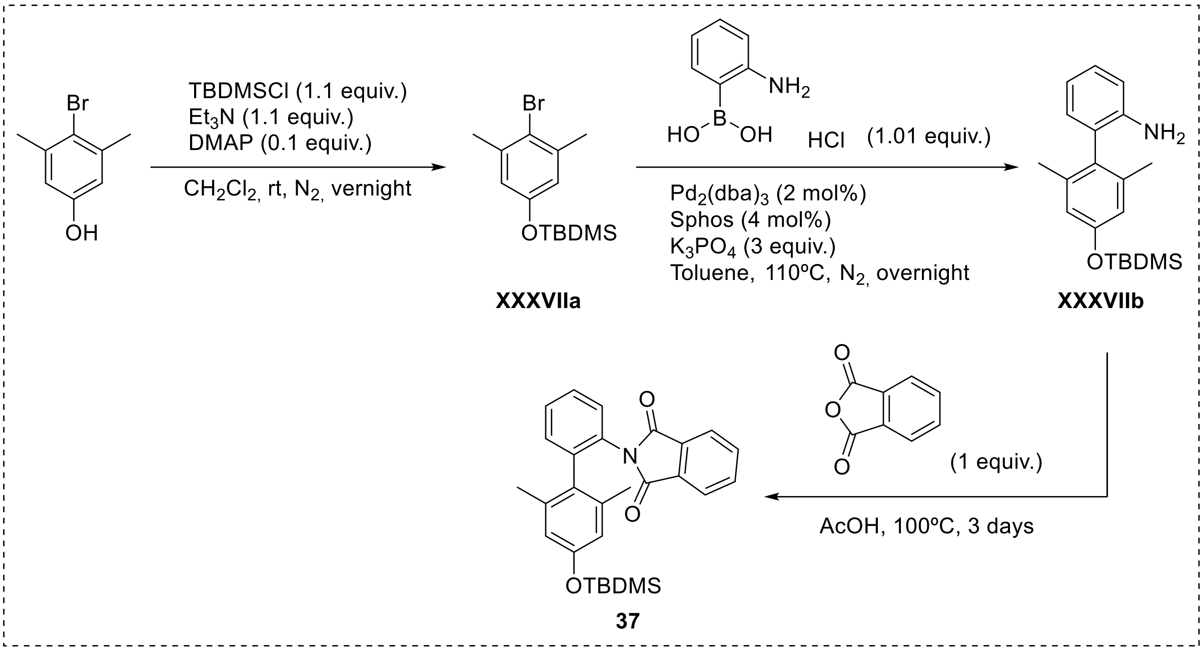

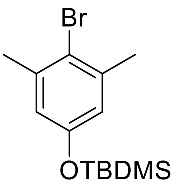

(4-bromo-3,5-dimethylphenoxy)(*tert*-butyl)dimethylsilane (XXXVIIa). It was prepared according to a modified reported procedure.[31] Under N_2 atmosphere, 4-bromo-3,5-dimethylphenol (3.04 g, 14.8 mmol, 1 equiv.) was dissolved in anhydrous CH_2Cl_2 (20 mL, 0.74 M) and Et_3N (2.29 mL, 16.3 mmol, 1.1 equiv.) and DMAP (183 mg, 1.48 mmol, 0.1 equiv.) were added. The mixture was cooled to 0ºC and TBDMSCl (2.51 g, 16.3 mmol, 1.1 equiv.) was added and stirred at room temperature overnight. The reaction was diluted with CH_2Cl_2 (20 mL) and it was washed with 2N $HCl_{(aq)}$ (20 mL) and saturated $NaHCO_3$ (20 mL). The combined organic layers were dried over anhydrous $MgSO_4$, filtered and the solvent was evaporated under reduced pressure. The crude was purified by column chromatography over silica gel (hexane) to afford the product as a colorless oil (3.45 g, 10.9 mmol, 74% yield). Spectral data match those reported in literature.[31] ^{1}H-NMR (400 MHz, $CDCl_3$) δ, ppm: 6.58 (s, 2H), 2.35 (s, 6H), 0.98 (s, 9H), 0.19 (s, 6H). ^{13}C-NMR (100 MHz, $CDCl_3$) δ, ppm: 154.3, 139.2, 120.1, 119.1, 25.8, 24.0, 18.3, -4.3. HRMS (ESI-MS) *m/z* calculated for $C_{14}H_{23}BrOSi$ $[M+H]^+$ 315.0774, found 315.0770. TLC (SiO_2, hexane), R_f = 0.65.

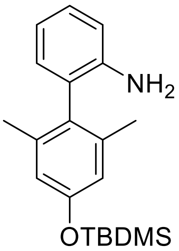

4'-((*tert*-butyldimethylsilyl)oxy)-2',6'-dimethyl-[1,1'-biphenyl]-2-amine (XXXVIIb). It was prepared according to General Procedure A[8] using **XXXVIIa** (560 mg, 1.78 mmol, 1 equiv.), (2-aminophenyl)boronic acid hydrochloride (321 mg, 1.79 mmol, 1.01 equiv.), $Pd_2(dba)_3$ (32.9 mg, 0.0355 mmol, 2 mol%), Sphos (29.8 mg, 0.0710 mmol, 4 mol%) and K_3PO_4 (1.92 mg, 8.86 mmol, 5 equiv.) in toluene (9 mL). The crude was purified by column chromatography over silica gel (hexane:EtOAc, 100:0 to 95:5) to obtain the product as a colorless solid (139 mg, 0.425 mmol, 24% yield). ^{1}H-NMR (400 MHz, $CDCl_3$) δ, ppm: 7.16 (ddd, *J* = 7.9, 7.4, 1.6 Hz, 1H), 6.92 (dd, *J* = 7.4, 1.6 Hz, 1H), 6.84-6.75 (m, 2H), 6.63 (s, 2H), 3.18 (br, 2H), 2.00 (s, 6H), 1.02 (s, 9H), 0.25 (s, 6H). ^{13}C-NMR (100 MHz, $CDCl_3$) δ, ppm: 154.8, 143.9, 138.6, 130.9, 130.5, 128.2, 126.3, 119.1, 118.6, 115.1, 25.8, 20.4, 18.3, -4.2. HRMS (ESI-MS) *m/z* calculated for $C_{20}H_{29}NOSi$ $[M+H]^+$ 328.2091, found 328.2097. TLC (SiO_2, hexane:EtOAc 95:5), R_f = 0.13.

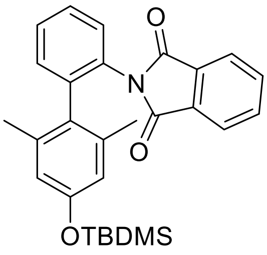

2-(4'-((*tert*-butyldimethylsilyl)oxy)-2',6'-dimethyl-[1,1'-biphenyl]-2-yl)isoindoline-1,3-dione (37). It was prepared according to General Procedure F using **XXXVIIb** (65.7 mg, 0.204 mmol, 1 equiv.) and phthalic anhydride (30.5 mg, 0.624 mmol, 1 equiv.) in acetic acid (0.4 mL). The crude was purified by column chromatography over silica gel (hexane:EtOAc, 100:0 to 90:10) to obtain the product as a colorless solid (27.1 mg, 0.0592 mmol, 29% yield). ^{1}H-NMR (400 MHz, $CDCl_3$) δ, ppm: 7.77 (dd, J = 5.5, 3.1 Hz, 2H), 7.67 (dd, J = 5.5, 3.1 Hz, 2H), 7.56-7.46 (m, 2H), 7.38 -7.30 (m, 2H), 6.45 (s, 2H), 2.01 (s, 6H), 0.90 (s, 9H), 0.08 (s, 6H). ^{13}C-NMR (100 MHz, $CDCl_3$) δ, ppm: 167.2, 154.6, 140.4, 138.3, 134.1, 132.1, 131.9, 131.3, 130.7, 129.6, 129.4, 128.1, 123.6, 119.0, 25.8, 20.7, 18.3, -4.3. HRMS (ESI-MS) m/z calculated for $C_{28}H_{31}NO_3Si$ $[M+H]^+$ 458.2146, found 458.2148. TLC (SiO_2, hexane:EtOAc 9:1), R_f = 0.40.

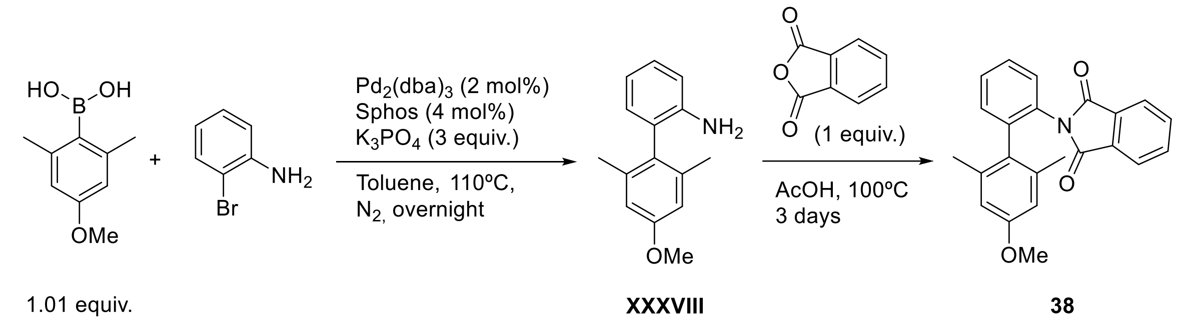

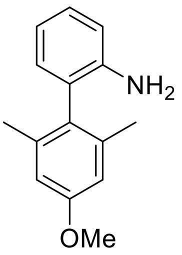

4'-methoxy-2',6'-dimethyl-[1,1'-biphenyl]-2-amine (XXXVIII). It was prepared according to General Procedure A[8] using 2-bromoaniline (175 mg, 1.00 mmol, 1 equiv.), (4-methoxy-2,6-dimethylphenyl)boronic acid (187 mg, 1.01 mmol, 1.01 equiv.), $Pd_2(dba)_3$ (18.4 mg, 0.020 mmol, 2 mol%), Sphos (16.7 mg, 0.040 mmol, 4 mol%) and K_3PO_4 (649 mg, 3.00 mmol, 3 equiv.) in toluene (5 mL). The crude was purified by column chromatography over silica gel (hexane:EtOAc, 100:0 to 90:10) to obtain the product as a colorless solid (120 mg, 0.528 mmol, 53% yield). ^{1}H-NMR (400 MHz, $CDCl_3$) δ, ppm: 7.17 (ddd, J = 7.9, 7.4, 1.7 Hz, 1H), 6.93 (dd, J = 7.4, 1.6 Hz, 1H), 6.86-6.77 (m, 2H), 6.72 (s, 2H), 3.83 (s, 3H), 3.43 (br, 2H), 2.05 (s, 6H). ^{13}C-NMR (100 MHz, $CDCl_3$) δ, ppm: 158.7, 143.9, 138.7, 130.4, 130.3, 128.1, 125.9, 118.5, 115.0, 113.0, 55.2, 20.5. HRMS (ESI-MS) m/z calculated for $C_{15}H_{17}NO$ $[M+H]^+$ 228.1383, found 228.1377. TLC (SiO_2, hexane:EtOAc 9:1), R_f = 0.35.

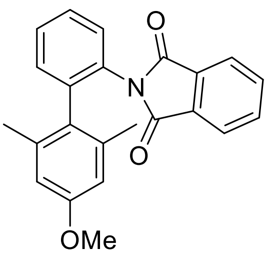

2-(4'-methoxy-2',6'-dimethyl-[1,1'-biphenyl]-2-yl)isoindoline-1,3-dione (38). It was prepared according to General Procedure F using **XXXVIII** (96.2 mg, 0.423 mmol, 1 equiv.) and phthalic anhydride (63.3 mg, 0.423 mmol, 1 equiv.) in acetic acid (0.8 mL). The crude was purified by column chromatography over silica gel (hexane:EtOAc, 100:0 to 90:10) to obtain the product as a colorless solid (65.9 mg, 0.184 mmol, 44% yield). ^{1}H-NMR (400 MHz, $CDCl_3$) δ, ppm: 7.79 (dd, J = 5.5, 3.1 Hz, 2H), 7.67 (dd, J = 5.5, 3.1 Hz, 2H), 7.55-7.46 (m, 2H), 7.40-7.33 (m, 1H), 7.33-7.28 (m, 1H), 6.54 (s, 2H), 3.71 (s, 3H), 2.07 (s, 6H). ^{13}C-NMR (100 MHz, $CDCl_3$) δ, ppm: 167.2, 158.5, 140.2, 138.4, 134.2, 132.2, 131.8, 131.3, 130.1, 129.5, 129.4, 128.1, 123.6,

112.7, 55.0, 20.9. HRMS (ESI-MS) *m/z* calculated for $C_{23}H_{19}NO_3$ $[M+H]^+$ 358.1438, found 358.1432. TLC (SiO_2, hexane:EtOAc 8:2), R_f = 0.44.

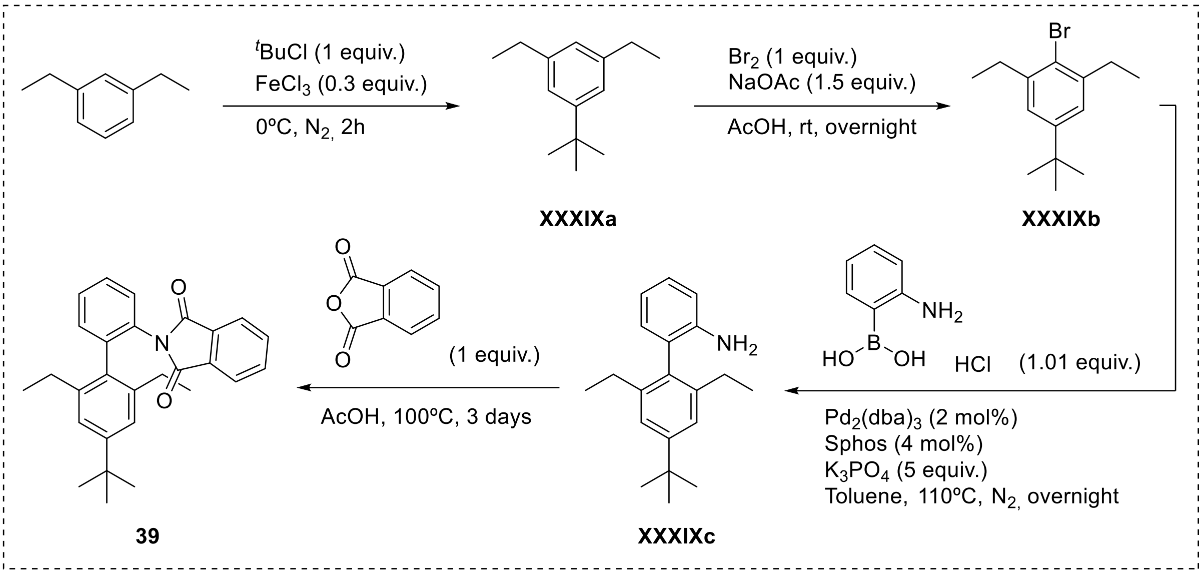

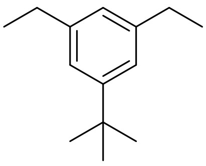

1-(*tert*-butyl)-3,5-diethylbenzene (XXXIXa). It was prepared according to a modified reported procedure.[32] Under N_2 atmosphere, 1,3-diethylbenzene (5 mL, 31.4 mmol, 1 equiv.) was mixed with $FeCl_3$ (1.58 g, 9.42 mmol, 0.3 equiv.) and cooled to 0ºC. *tert*-butyl chloride (3.45 mL, 31.4 mmol, 1 equiv) was added dropswise over a period of 1h and stirred for 2 hours at 0ºC. The reaction was quenched with H_2O (5 mL) and the product was extracted with EtOAc (3x10 mL). The combined organic phases were dried over anhydrous $MgSO_4$, filtered and the solvent was evaporated under reduced pressure. The crude was purified by column chromatography over silica gel (hexane) to afford the product as a colorless oil (1.02 g, 5.36 mmol,17% yield). **The product was not completely pure but was used in the next step without further purification.* ^{1}H-NMR (400 MHz, $CDCl_3$) δ, ppm: 7.07 (d, *J* = 1.6 Hz, 2H), 6.90-6.88 (m, 1H), 2.65 (q, *J* = 7.6 Hz, 4H), 1.34 (s, 9H), 1.26 (d, *J* = 7.6 Hz, 6H). ^{13}C-NMR (100 MHz, $CDCl_3$) δ, ppm: 151.1, 143.8, 124.5, 122.3, 34.6, 31.5, 29.1, 15.7. GC-MS (CI) for *m/z* $C_{14}H_{22}$ $[M+H]^+$ 291.1. TLC (SiO_2, hexane), R_f = 0.40.

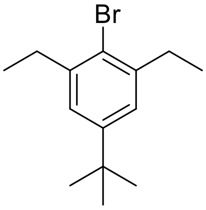

2-bromo-5-(*tert*-butyl)-1,3-diethylbenzene (XXXIXb). It was prepared according to General Procedure C using **XXXIXa** (1.00 g, 5.25 mmol, 1 equiv.), Br_2 (840 mg, 5.25 mmol, 1 equiv.) and NaOAc (653 mg, 7.88 mmol, 1.5 equiv.) in AcOH (30 mL). The crude was purified by column chromatography over silica gel (hexane) to obtain the product as a colorless oil (1.13 g, 4.20 mmol, 80% yield). **The product was not completely pure but was used in the next step without further purification.* ^{1}H-NMR (400 MHz, $CDCl_3$) δ, ppm: 7.11 (s, 2H), 2.81 (q, *J* = 7.5 Hz, 5H), 1.32 (s, 9H), 1.24 (d, *J* = 7.5 Hz, 6H). ^{13}C-NMR (100 MHz, $CDCl_3$) δ, ppm: 150.2, 143.3, 126.8, 124.4, 34.6, 31.5, 30.6, 14.7. GC-MS (CI) *m/z* for $C_{14}H_{21}Br$ $[M+NH_4]^+$ 285.9. TLC (SiO_2, hexane), R_f = 0.81.

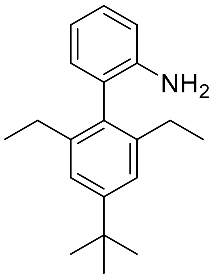

4'-(*tert*-butyl)-2',6'-diethyl-[1,1'-biphenyl]-2-amine (XXXIXc). It was prepared according to General Procedure A[8] using **XXXIXb** (305.9 mg, 1.14 mmol, 1 equiv.), (2-aminophenyl)boronic acid hydrochloride (205 mg, 1.15 mmol, 1.01 equiv.), $Pd_2(dba)_3$ (21.0 mg, 0.0227 mmol, 2 mol%), Sphos (19.0 mg, 0.0454 mmol, 4 mol%) and K_3PO_4 (1.23 g, 5.68 mmol, 5 equiv.) in toluene (5 mL). The crude was purified by column chromatography over silica gel (hexane:EtOAc, 100:0 to 95:5) to obtain the product as a colorless solid (82.3 mg, 0.292 mmol, 26% yield). ^{1}H-NMR (400 MHz, $CDCl_3$) δ, ppm: 7.20- 7.15 (m, 3H), 7.03-6.97 (m, 1H), 6.84-6.74 (m, 2H), 3.15 (br, 2H), 2.44-2.30 (m, 4H), 1.37 (s, 9H), 1.06 (t, J = 7.6 Hz, 6H). ^{13}C-NMR (100 MHz, $CDCl_3$) δ, ppm: 150.7, 144.1, 142.8, 133.7, 130.9, 128.1, 125.9, 123.2, 118.3, 115.0, 34.7, 31.6, 27.0, 15.7. HRMS (ESI-MS) *m/z* calculated for $C_{20}H_{27}N$ $[M+H]^+$ 282.2216, found 282.2215. TLC (SiO_2, hexane:EtOAc 95:5), R_f = 0.31.

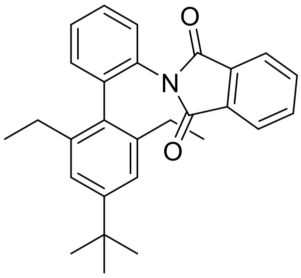

2-(4'-(*tert*-butyl)-2',6'-diethyl-[1,1'-biphenyl]-2-yl)isoindoline-1,3-dione (39). It was prepared according to General Procedure F using **XXXIXc** (82.3 mg, 0.292 mmol, 1 equiv.) and phthalic anhydride (43.8 mg, 0.292 mmol, 1 equiv.) in acetic acid (0.6 mL). The crude was purified by column chromatography over silica gel (hexane:EtOAc, 100:0 to 95:5) to obtain the product as a colorless solid (31.4 mg, 0.0763 mmol, 26% yield). ^{1}H-NMR (400 MHz, $CDCl_3$) δ, ppm: 7.74 (dd, J = 5.5, 3.1 Hz, 2H), 7.65 (dd, J = 5.5, 3.1 Hz, 2H), 7.53-7.50 (m, 2H), 7.41-7.34 (m, 2H), 7.00 (s, 2H), 2.37 (q, J = 7.6 Hz, 5H), 1.22 (s, 9H), 1.05 (t, J = 7.6 Hz, 6H). ^{13}C-NMR (100 MHz, $CDCl_3$) δ, ppm: 167.1, 150.4, 142.4, 140.4, 134.0, 133.0, 132.2, 131.9, 131.3, 129.5, 129.0, 128.1, 123.4, 122.3, 34.6, 31.4, 26.8, 15.9. HRMS (ESI-MS) *m/z* calculated for $C_{28}H_{29}NO_2$ $[M+H]^+$ 412.2271, found 412.2267. TLC (SiO_2, hexane:EtOAc 9:1), R_f = 0.43.

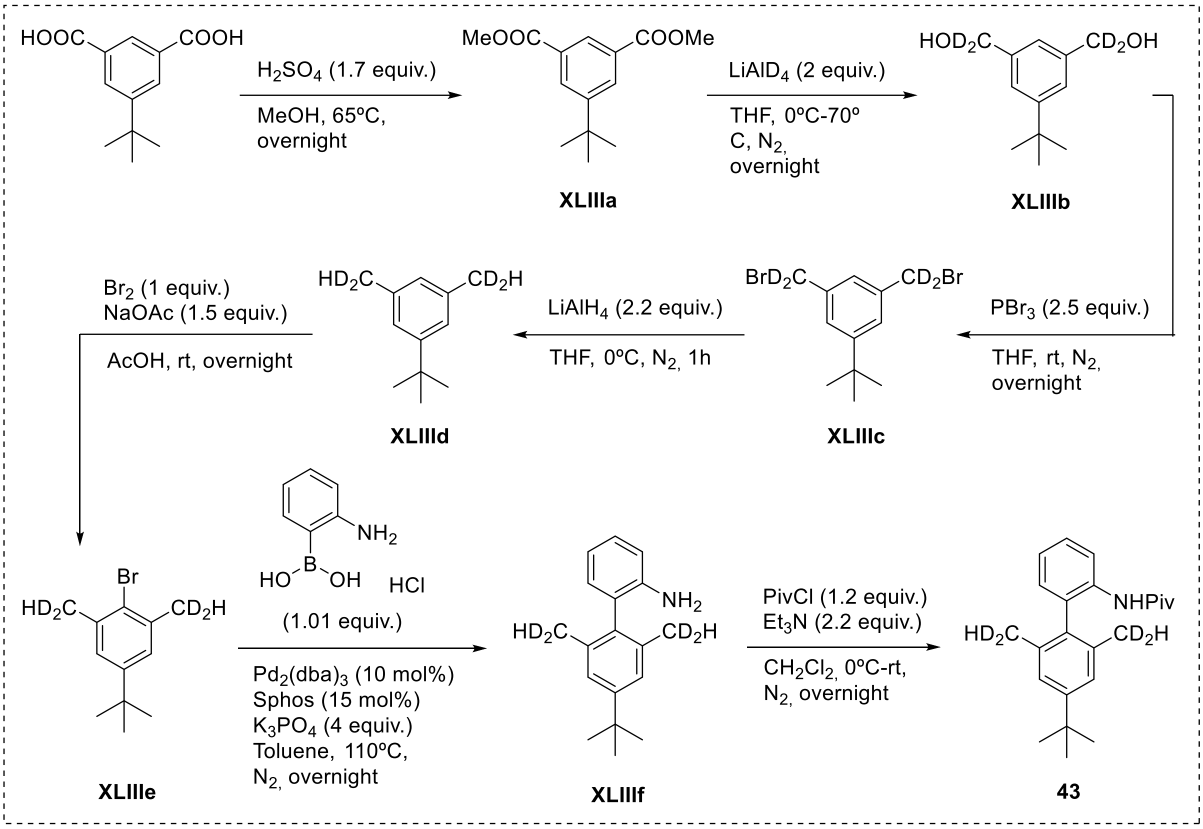

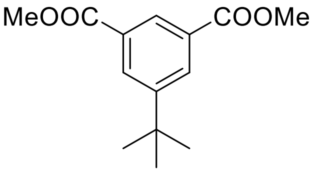

Dimethyl 5-(*tert*-butyl)isophthalate (XLIIIa). It was prepared according to a modified reported procedure.[33] 5-(*tert*-butyl)isophthalic acid (5.02 g, 21.5 mmol, 1 equiv.) was dissolved in MeOH (43 mL, 0.5M) and H_2SO_4 (2 mL, 36.8 mmol, 1.7 equiv.) was added. The reaction was refluxed at 65ºC overnight. After being cooled to room temperature, the solvent was evaporated under reduced pressure. The crude was dissolved in EtOAc and the organic phase was washed with 1M $NaOH_{(aq)}$ and brine. The combined organic layers were dried over anhydrous $MgSO_4$, filtered and the solvent was evaporated under reduced pressure. The product was obtained as a colorless solid (3.52 g, 14.1 mmol, 66% yield) and was used in the next step without further purification. Spectral data match those reported in literature.[34] ^{1}H-NMR (400 MHz, $CDCl_3$) δ, ppm: 8.50 (t, *J* = 1.6 Hz, 1H), 8.26 (d, *J* = 1.6 Hz, 2H), 3.94 (s, 6H), 1.37 (s, 9H). ^{13}C-NMR (100 MHz, $CDCl_3$) δ, ppm: 166.8, 152.3, 131.1, 130.5, 128.2, 52.4, 35.1, 31.3. HRMS (ESI-MS) *m/z* calculated for $C_{14}H_{18}O_4$ $[M+H]^+$ 251.1278, found 251.1278. TLC (SiO_2, hexane:EtOAc 9:1), R_f = 0.45.

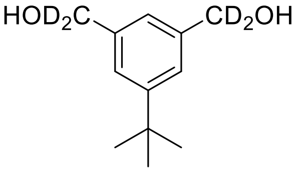

(5-(*tert*-butyl)-1,3-phenylene)bis(methan-d_2-ol) (XLIIIb). It was prepared according to modified reported procedures.[33, 35] Under N_2 atmosphere, $LiAlD_4$ (691 mg, 16.3 mmol, 2 equiv.) was dissolved in anhydrous THF (10 mL, 1.6 M). The solution was cooled at 0ºC and **XLIIIa** (2.04 g, 8.15 mmol, 1 equiv.) was added in small portions and the mixture was refluxed at 70ºC overnight. The reaction was then cooled to 0ºC and quenched with H_2O (1 mL), 1M $NaOH_{(aq)}$ (1 mL) and H_2O (3 mL). The solid was filtered off and washed with EtOAc (3x10 mL). The filtrates were evaporated under reduced pressure. The product was obtained as a colorless oil (676 mg, 3.41 mmol, 42% yield) and was used in the next step without further purification. ^{1}H-NMR (400 MHz, $CDCl_3$) δ, ppm: 7.29-7.27 (m, 2H),

7.17-7.14 (m, 1H), 2.38 (br, 2H), 1.31 (s, 9H). 13C-NMR (100 MHz, $CDCl_3$) δ, ppm: 152.0, 140.9, 125.3, 123.1, 64.8 (quint, J = 21.2 Hz), 34.9, 31.5. HRMS (ESI-MS) m/z calculated for $C_{12}H_{14}D_4O_2$ $[M+Na]^+$ 221.1450, found 221.1449.

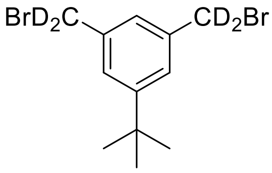

(3-(bromomethyl-*d*₂)-5-(*tert*-butyl)phenyl)methan-*d*₂-ol (XLIIIc). It was prepared according to modified reported procedures.[35, 36] Under N_2 atmosphere, **XLIIIb** (676 mg, 3.41 mmol, 1 equiv.) was dissolved in anhydrous THF (17 mL, 0.2 M) and PBr_3 (0.818 mL, 8.53 mmol, 2.5 equiv.) was added dropwise. The mixture was stirred at room temperature overnight. The reaction was quenched with saturated NH_4Cl (10 mL) and the product was extracted with Et_2O (3x30 mL). The organic phases were dried over anhydrous $MgSO_4$, filtered and the solvent was evaporated under reduced pressure. The crude was purified by column chromotagraphy over silica gel (hexane) to obtain the product as a colorless oil (432 mg, 1.33 mmol, 39% yield). 1H-NMR (400 MHz, $CDCl_3$) δ, ppm: 7.35 (d, J = 1.7 Hz, 2H), 7.26 (t, J = 1.7 Hz, 1H), 1.34 (s, 9H). 13C-NMR (100 MHz, $CDCl_3$) δ, ppm: 152.6, 138.0, 126.9, 126.3, 34.9, 33.1 (quint, J = 23.1 Hz), 31.3. GC-MS (CI) m/z for $C_{12}H_{12}D_4Br_2$ $[M+NH_4]^+$ 342.0. TLC (SiO_2, hexane), R_f = 0.19.

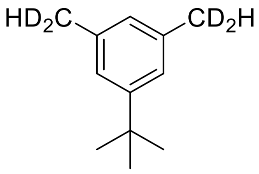

1-(tert-butyl)-3,5-bis(methyl-d2)benzene (XLIIId). It was prepared according to a modified reported procedure.[37] Under N_2 atmosphere, $LiAlH_4$ (117 mg, 2.94 mmol, 2.2 equiv.) was dissolved in anhydrous THF (2 mL, 1.5 M). The solution was cooled at 0ºC and a solution of **XLIIIc** (432 mg, 1.33 mmol, 1 equiv.) in anhydrous THF (2 mL, 0.7 M) was added dropwise. The solution was further stirred at 0ºC for 1 hour. The reaction was diluted with anhydrous THF (4 mL) and quenched with H_2O (1 mL), 1M $NaOH_{(aq)}$ (1 mL) and H_2O (3 mL). The mixture was stirred at room temperature for 15 minutes and anhydrous $MgSO_4$ was subsequently added. After stirring for further 15 minutes, the solid was filtered off and washed with Et_2O (3x10 mL). The filtrates were evaporated under reduced pressure. The product was obtained as a colorless oil and was used immediately in the next step without further purification. 1H-NMR (400 MHz, $CDCl_3$) δ, ppm: 7.05-6.95 (m, 2H), 6.88-6.78 (m, 1H), 2.30 (s, 2H), 1.31 (s, 9H). 13C-NMR (100 MHz, $CDCl_3$) δ, ppm: 151.2, 137.4, 127.2, 123.3, 34.6, 31.6, 21.2 (quint, J = 19.3 Hz).

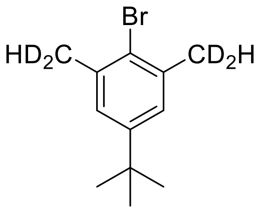

2-bromo-5-(*tert*-butyl)-1,3-bis(methyl-*d*₂)benzene (XLIIIe). It was prepared according to General Procedure C using **XLIIId**, Br_2 (212 mg, 1.33 mmol, 1 equiv. respect to **XLIIIc**) and NaOAc (166 mg, 2.00 mmol, 1.5 equiv. respect to **XLIIIc**) in AcOH (5.4 mL). The crude was purified by recrystallization from ethanol to obtain the product as a colorless solid (61.8 mg, 0.253 mmol, 19% yield over two steps). 1H-NMR (400 MHz, $CDCl_3$) δ, ppm: 7.10 (s, 2H), 2.39 (s, 2H), 1.28 (s, 9H). 13C-NMR (100 MHz, $CDCl_3$) δ, ppm: 149.7, 137.6, 125.5, 125.4, 34.1, 31.4, 23.6 (quint, J = 19.6 Hz). GC-MS (CI) m/z for $C_{12}H_{13}D_4Br$ $[M+H]^+$ 244.0.

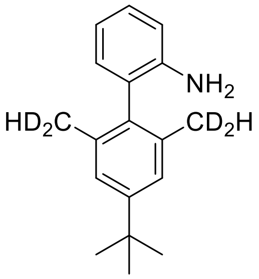

4'-(*tert*-butyl)-2',6'-bis(methyl-*d*$_2$)-[1,1'-biphenyl]-2-amine (XLIIIf). It was prepared according to General Procedure A using **XLIIIe** (61.8 mg, 0.253 mmol, 1 equiv.), (2-aminophenyl)boronic acid hydrochloride (67.9 mg, 0.380 mmol, 1.01 equiv.), $Pd_2(dba)_3$ (23.4 mg, 0.0253 mmol, 10 mol%), Sphos (15.9 mg, 0.0380 mmol, 15 mol%) and K_3PO_4 (219 mg, 1.01 mmol, 4 equiv.) in toluene (1 mL). The crude was purified by column chromatography over silica gel (hexane:EtOAc, 100:0 to 95:5) to obtain the product as a colorless solid (23.7 mg, 0.0921 mmol, 36% yield). ^{1}H-NMR (400 MHz, $CDCl_3$) δ, ppm: 7.19-7.13 (m, 3H), 6.94 (dd, J = 7.4, 1.6 Hz, 1H), 6.84-6.75 (m, 2H), 3.41 (br, 2H), 2.02 (s, 2H), 1.35 (s, 9H). ^{13}C-NMR (100 MHz, $CDCl_3$) δ, ppm: 150.3, 143.7, 136.6, 135.0, 130.1, 128.1, 126.5, 124.8, 118.6, 115.1, 34.5, 31.6, 20.1 (quint, J = 19.3 Hz). HRMS (ESI-MS) *m/z* calculated for $C_{18}H_{19}D_4N$ $[M+H]^+$ 258.2154, found 258.2157. TLC (SiO_2, hexane:EtOAc 95:5), R_f = 0.40.

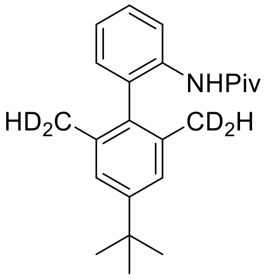

***N*-(4'-(*tert*-butyl)-2',6'-bis(methyl-*d*$_2$)-[1,1'-biphenyl]-2-yl) pivalamide (43)**. It was prepared according to General Procedure E using **XLIIIf** (23.7 mg, 0.0921 mmol, 1 equiv.), pivaloyl chloride (13.7 mL, 0.110 mmol, 1.2 equiv.) and Et_3N (28.5 mL, 0.203 mmol, 2.2 equiv.) in CH_2Cl_2 (1 mL). The crude was purified by column chromatography over silica gel (hexane:EtOAc, 100:0 to 95:5) to obtain the product as a colorless solid (13.9 mg, 0.0407 mmol, 44% yield). ^{1}H-NMR (400 MHz, $CDCl_3$) δ, ppm: 8.43 (dd, J = 8.3, 1.1 Hz, 1H), 7.39-7.33 (m, 1H), 7.20 (s, 2H), 7.16- 7.09 (m, 2H), 7.03 (br, 1H), 1.95 (s, 2H), 1.34 (s, 9H), 0.95 (s, 9H). ^{13}C-NMR (100 MHz, $CDCl_3$) δ, ppm: 176.3, 151.6, 136.7, 135.7, 133.4, 130.1, 129.0, 128.4, 125.1, 123.9, 119.7, 39.8, 34.6, 31.5, 27.2, 20.0 (quint, J = 19.3 Hz). HRMS (ESI-MS) *m/z* calculated for $C_{23}H_{27}D_4NO$ $[M+Na]^+$ 364.2549, found 364.2543. TLC (SiO_2, hexane:EtOAc 9:1), R_f = 0.23.

3.4. Non-reactive substrates

The following substrates were tested under the reaction conditions, but they found to be non-reactive or provide only traces of reaction products.

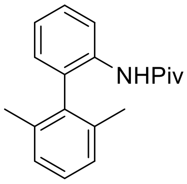

44

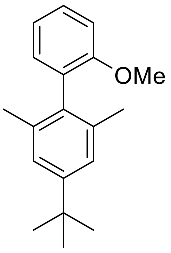

45

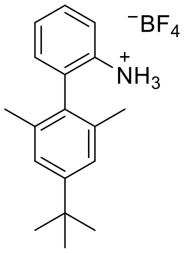

46

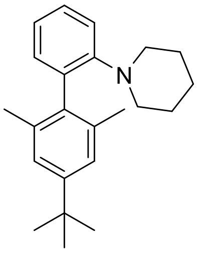

47

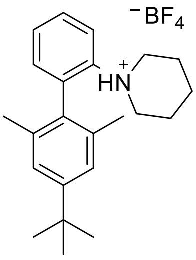

48

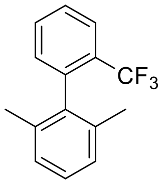

49

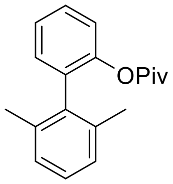

50

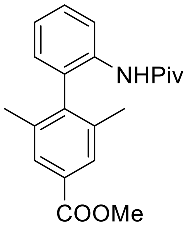

51

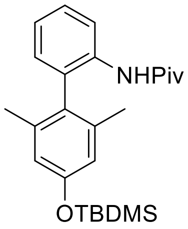

52

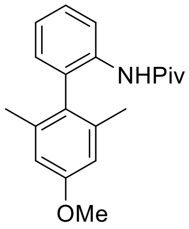

53

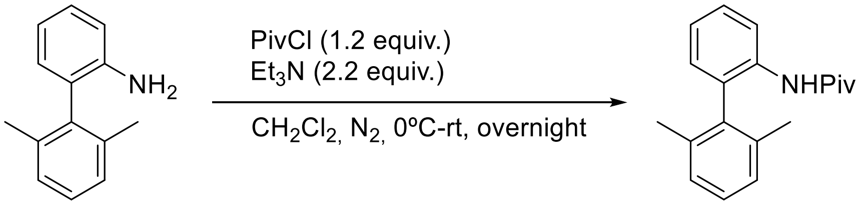

XXXV **44**

***N*-(2',6'-dimethyl-[1,1'-biphenyl]-2-yl)pivalamide (44)**. It was prepared according to General Procedure E using **XXXV** (115 mg, 0.583 mmol, 1 equiv.), pivaloyl chloride (87.1 mL, 0.700 mmol, 1.2 equiv.) and Et_3N (181 mL, 1.28 mmol, 2.2 equiv.) in CH_2Cl_2 (3 mL). The crude was purified by column chromatography over silica gel (hexane:EtOAc, 100:0 to 95:5) to obtain the product as a colorless oil (113 mg, 0.402 mmol, 69% yield). ^{1}H-NMR (400 MHz, $CDCl_3$) δ, ppm: 8.50 (dd, *J* = 8.3, 1.2 Hz, 1H), 7.43-7.36 (m, 1H), 7.29 (dd, *J* = 6.0, 2.8 Hz, 1H), 7.25-7.16 (m, 3H), 7.11 (dd, *J* = 7.6, 1.7 Hz, 1H), 7.08 (s, 1H), 2.02 (s, 6H), 1.00 (s, 9H). ^{13}C-NMR (100 MHz, $CDCl_3$) δ, ppm: 176.3, 137.1, 136.2, 135.4, 129.8, 128.8, 128.5, 128.4, 128.0, 124.0, 119.8, 39.7, 27.2, 20.2. HRMS (ESI-MS) *m/z* calculated for $C_{19}H_{23}NO$ $[M+H]^+$ 198.1277, found 198.1272. TLC (SiO_2, hexane:EtOAc 9:1), R_f = 0.50.

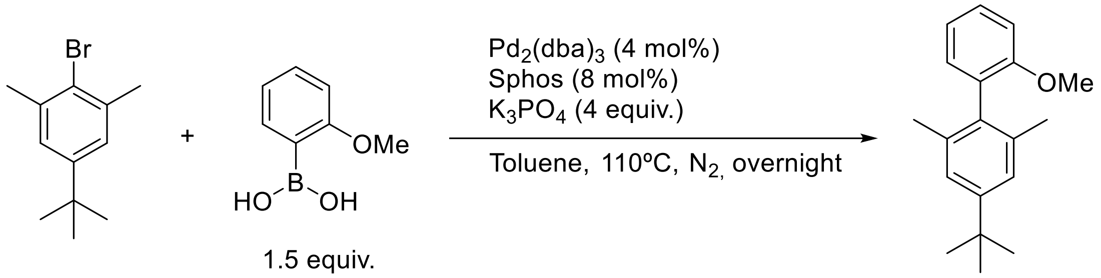

Ia **45**

4-(*tert*-butyl)-2'-methoxy-2,6-dimethyl-1,1'-biphenyl (45). It was prepared according to General Procedure A[7] using **Ia** (241 mg, 1.00 mmol, 1 equiv.), (2-methoxyphenyl)boronic acid (204 mg, 1.50 mmol, 1.5 equiv.), $Pd_2(dba)_3$ (37.0 mg, 0.040 mmol, 4 mol%), Sphos (34.0 mg, 0.080 mmol, 8 mol%) and K_3PO_4 (866 mg, 4.00 mmol, 4 equiv.) in toluene (5 mL). The crude was purified by column chromatography over silica gel (hexane) to obtain the product as a colorless oil (108 mg, 0.402 mmol, 40% yield). ^{1}H-NMR (400 MHz, $CDCl_3$) δ, ppm: 7.35 (ddd, *J* = 8.2, 7.3, 2.0 Hz, 1H), 7.14 (s, 2H), 7.10-6.97 (m, 3H), 3.76 (s, 3H), 2.05 (s, 6H), 1.38 (s, 9H). ^{13}C-NMR (100 MHz, $CDCl_3$) δ, ppm: 156.7, 149.4, 135.9, 135.2, 131.1, 129.7, 128.2, 124.1, 120.6, 110.8, 55.5, 34.3, 31.5, 20.8. HRMS (ESI-MS) *m/z* calculated for $C_{19}H_{24}O$ $[M+H]^+$ 269.1900, found 269.1898. TLC (SiO_2, hexane:EtOAc 9:1), R_f = 0.79.

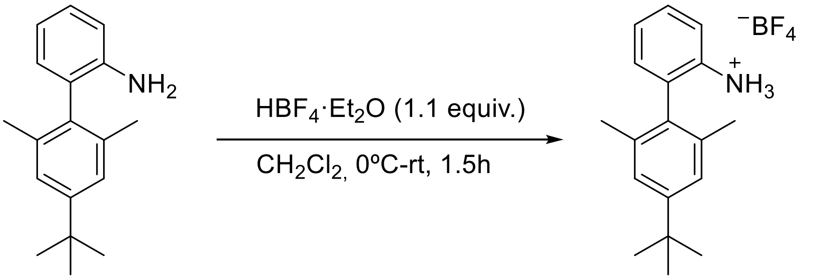

XIII **46**

4'-(*tert*-butyl)-2',6'-dimethyl-[1,1'-biphenyl]-2-aminium tetrafluoroborate (46). It was prepared according to a modified reported procedure.[38] **XII** (128 mg, 0.503 mmol, 1 equiv.) was dissolved in anhydrous CH_2Cl_2 (50 mL, 0.01 M) and cooled to 0ºC. $HBF_4·Et_2O$ (75.4 mL, 0.554 mmol, 1.1 equiv.) was added dropwise. The solution was stirred at 0ºC for 30 minutes and at room temperature for 1 h. The solvent was then evaporated under reduced pressure and the crude was dried on high vacuum for 2 hours. The resultant solid was then washed with hexane and recrystallized from CH_2Cl_2 to afford the product as a colorless solid (61.1 mg, 0.179 mmol, 36% yield). ^{1}H-NMR (400 MHz, $CDCl_3$) δ, ppm: 7.59-7.55 (m, 1H), 7.53-7.43 (m, 2H), 7.26-7.22 (m, 1H), 7.16 (s, 2H), 1.94 (s, 6H), 1.28 (s, 9H). ^{13}C-NMR (100 MHz, $CDCl_3$) δ, ppm: 152.4, 136.2, 135.7, 131.1, 130.3, 130.1, 129.4, 127.0, 125.5, 124.0, 34.6, 31.3, 20.4. HRMS (ESI-MS) *m/z* calculated for $C_{18}H_{24}N$ $[M]^+$ 254.1903, found 254.1904; *m/z* calculated for BF_4 $[M]^-$ 87.0035, found 87.0035.

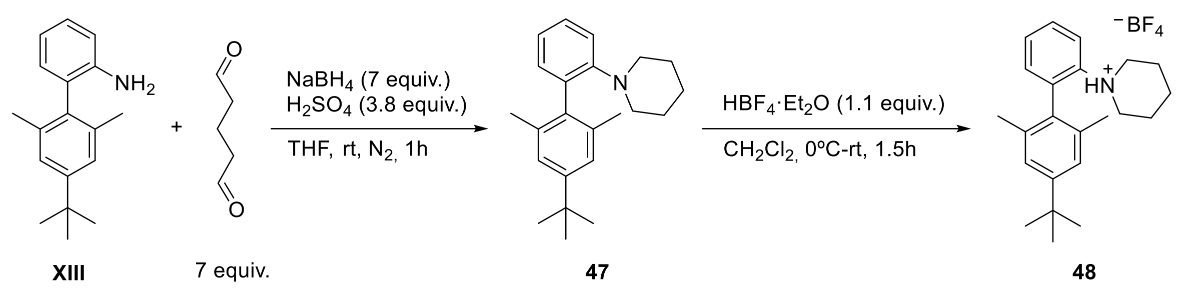

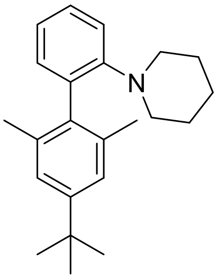

1-(4'-(*tert*-butyl)-2',6'-dimethyl-[1,1'-biphenyl]-2-yl)piperidine (47). It was prepared according to a modified reported procedure.[39] Under N_2 atmosphere, a solution of **XIII** (154 mg, 0.607 mmol, 1 equiv.) in anhydrou THF (6 mL, 0.1 M) was added dropwise to a solution of glutaraldehyde (0.769 mL, 4.25 mmol, 7 equiv.) and 20% aqueous H_2SO_4 (0.6 mL, 2.33 mmol, 3.8 equiv.) in THF (3 mL, 0.2 M). The mixture was stirred at room temperature overnight and the reaction was quenched with saturated Na_2CO_3 (10 mL). The product was extracted with EtOAc (3 x 20 mL) and the organic phase was dried over anhydrous $MgSO_4$, filtered and the solvent was evaporated under reduced pressure. The crude was purified by column chromotagraphy over silica gel (hexane) to obtain the product as an orange oil (81.6 mg, 0.254 mmol, 42% yield). ^{1}H-NMR (400 MHz, $CDCl_3$) δ, ppm: 7.32-7.26 (m, 1H), 7.13 (s, 2H), 7.08-7.00 (m, 3H), 2.79-2.69 (m, 4H), 2.11 (s, 6H), 1.42-1.34 (m, 2H), 1.37 (s, 9H), 1.33-1.24 (m, 2H). ^{13}C-NMR (100 MHz, $CDCl_3$) δ, ppm: 152.3, 149.4, 137.7, 135.7, 135.0, 131.5, 127.8, 124.4, 121.8, 118.7, 52.4, 34.4, 31.6, 26.4, 24.5, 21.1. HRMS (ESI-MS) *m/z* calculated for $C_{23}H_{31}N$ $[M+H]^+$ 322.2529, found 322.2525. TLC (SiO_2, hexane), R_f = 0.49.

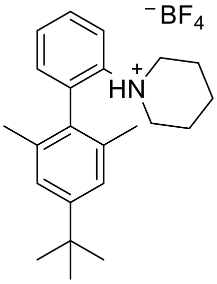

1-(4'-(*tert*-butyl)-2',6'-dimethyl-[1,1'-biphenyl]-2-yl)piperidin-1-ium tetrafluoroborate (48). It was prepared according to a modified reported procedure.[38] **XII** (34.3 mg, 0.107 mmol, 1 equiv.) was dissolved in anhydrous CH_2Cl_2 (8 mL, 0.01 M) and cooled to 0ºC. A solution of $HBF_4 \cdot Et_2O$ (16 mL, 0.117 mmol, 1.1 equiv.) in anhydrous CH_2Cl_2 (200 mL, 0.6 M) was added dropwise. The solution was stirred at 0ºC for 30 minutes and at room temperature for 1 h. The solvent was then evaporated under reduced pressure and the crude was dried on high vacuum for 2 hours. The resultant solid was dissolved in Et_2O (15 mL) and the solution was filtered. The solvent was removed under reduced pressure to afford the product as a yellow solid (18.9 mg, 0.0462 mmol, 43% yield). ^{1}H-NMR (400 MHz, $CDCl_3$) δ, ppm: 8.05-7.97 (m, 1H), 7.68-7.55 (m, 2H), 7.37 (s, 1H), 7.26 (s, 2H), 7.25-7.21 (m, 1H), 3.76-3.63 (m, 2H), 3.55-3.43 (m, 2H), 2.01 (s, 6H), 2.01-1.93 (m, 2H), 1.83-1.68 (m, 3H), 1.60-1.44 (m, 1H), 1.36 (s, 9H). ^{13}C-NMR (100 MHz, $CDCl_3$) δ, ppm: 153.3, 138.7, 136.3, 133.7, 132.4, 131.3, 130.8, 130.6, 125.9, 122.7, 57.5, 34.7, 31.3, 24.4, 21.0, 20.5. HRMS (ESI-MS) *m/z* calculated for $C_{23}H_{32}N$ $[M]^+$ 322.2529, found 322.2535; *m/z* calculated for BF_4 $[M]^-$ 87.0035, found 87.0038.

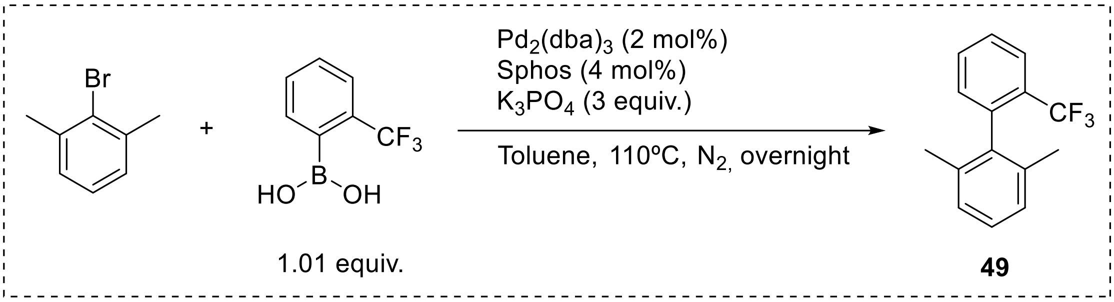

2,6-dimethyl-2'-(trifluoromethyl)-1,1'-biphenyl (49). It was prepared according to General Procedure A[8] using 2-bromo-1,3-dimethylbenzene (387 mg, 2.09 mmol, 1 equiv.), (2-(trifluoromethyl)phenyl)boronic acid (409 mg, 2.11 mmol, 1.01 equiv.), $Pd_2(dba)_3$ (38.7 mg, 0.0418 mmol, 2 mol%), Sphos (35.1 mg, 0.0836 mmol, 4 mol%) and K_3PO_4 (1.36 mg, 6.27 mmol, 3 equiv.) in toluene (10 mL). The crude was purified by column chromatography over silica gel (hexane) to obtain the product as a colorless oil (166 mg, 0.663 mmol, 32% yield). Spectral data match those reported in literature.[40] ^{1}H-NMR (400 MHz, $CDCl_3$) δ, ppm: 7.80 (dd, J = 8.0, 1.4 Hz, 1H), 7.62 (td, J = 7.6, 1.3 Hz, 1H), 7.49 (tt, J = 7.6, 1.1 Hz, 1H), 7.24-7.16 (m, 2H), 7.11 (d, J = 7.6 Hz, 2H), 1.97 (s, 6H). ^{13}C-NMR (100 MHz, $CDCl_3$) δ, ppm: 140.2 (J = 2.1 Hz), 138.8, 136.4, 132.2, 131.3, 128.8 (q, J = 29.7 Hz,), 127.8, 127.4, 127.1, 126.5 (q, J = 5.2 Hz), 124.1 (q, J = 273.8), 20.6. ^{19}F-NMR (377 MHz, $CDCl_3$) δ, ppm: -62.2. GC-MS (CI) m/z for $C_{15}H_{13}F_3$ $[M+NH_4]^+$ 268.0. TLC (SiO$_2$, hexane), R_f = 0.79.

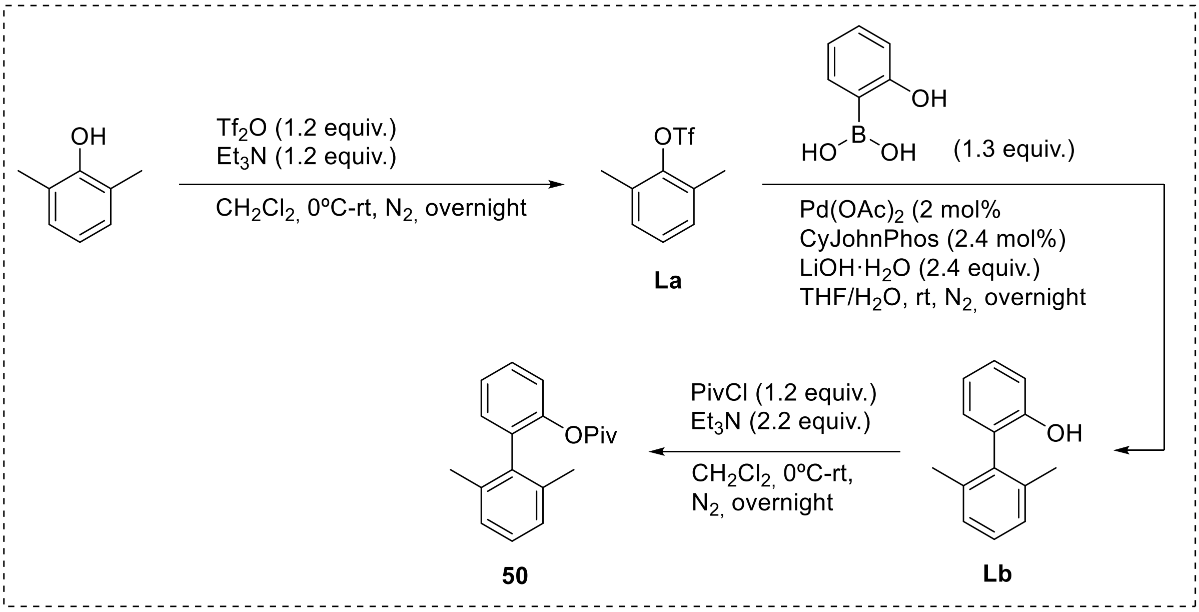

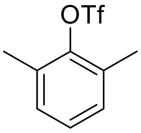

2,6-dimethylphenyl trifluoromethanesulfonate (La). It was prepared according to a modified reported procedure.[41] Under N_2 atmosphere, 2,6-dimethylphenol (4.00 g, 32.4 mmol, 1 equiv.) was dissolved in anhydrous CH_2Cl_2 (160 mL, 0.2 M) and cooled to 0°C. Et_3N (5.50 mL, 38.9 mmol, 1.2 equiv.) was added dropwise, followed by the addition of Tf_2O (6.70 mL, 38.9 mmol, 1.2 equiv.). The mixture was stirred at room temperature overnight. The reaction was quenched with H_2O (150 mL) and the phases were separated. The organic phase was washed with brine, dried over anhydrous $MgSO_4$, filtered and the solvent was evaporated under reduced pressure. The crude was purified by column chromatography over silica gel (hexane:EtOAc, 90:10) to afford

the product as a colorless oil (6.13 g, 24.1 mmol, 74% yield). Spectral data match those reported in literature.[42] 1H-NMR (400 MHz, $CDCl_3$) δ, ppm: 7.20-7.11 (m, 3H), 2.41 (s, 6H). 13C-NMR (100 MHz, $CDCl_3$) δ, ppm: 147.1, 131.6, 130.0, 128.1, 118.8 (q, J = 319.6 Hz), 17.2. 19F-NMR (377 MHz, $CDCl_3$) δ, ppm: -74.6. HRMS (ESI-MS) m/z calculated for $C_9H_9F_3O_3S$ $[M+NH_4]^+$ 272.0563, found 272.0566. TLC (SiO_2, hexane:EtOAc 90:10), R_f = 0.61.

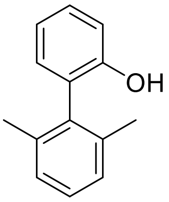

2',6'-dimethyl-[1,1'-biphenyl]-2-ol (Lb). It was prepared according to a modified reported procedure.[25] A Schlenk tube containing a magnetic stir bar was charged with **La** (551 mg, 2.17 mmol, 1 equiv.), (2-hydroxyphenyl)boronic acid (397 mg, 2.82 mmol, 1.3 equiv.), $Pd(OAc)_2$ 9.83 mg, 0.0433 mmol, 2 mol%), CyJohnPhos (18.6 mg, 0.0520 mmol, 2.4 mol%) and $LiOH{\cdot}H_2O$ (227 mg, 5.20 mmol, 2.4 equiv.) and dissolved in a mixture of THF/H_2O (8:2 v/v, 2 mL, 1 M) under N_2 atmosphere. The reaction was stirred at room temperature overnight. The reaction was quenched with 2N $HCl_{(aq)}$ (50 mL) and the product was extracted with EtOAc (3x25 mL). The combined organic layers were washed with brine, dried over anhydrous $MgSO_4$, filtered and the solvent was evaporated under reduced pressure. The crude was purified by column chromatography over silica gel (hexane:EtOAc, , 100:0 to 92:8) to obtain the product as a colorless solid (72.8 mg, 0.367 mmol, 17% yield). Spectral data match those reported in literature.[25] 1H-NMR (400 MHz, $CDCl_3$) δ, ppm: 7.30 (ddd, J = 8.7, 6.6, 2.3 Hz, 1H), 7.23 (dd, J = 8.6, 6.2 Hz, 1H), 7.20-7.14 (m, 2H), 7.06-6.97 (m, 3H), 4.65 (br, 1H), 2.06 (s, 6H). 13C-NMR (100 MHz, $CDCl_3$) δ, ppm: 152.4, 138.1, 134.9, 129.9, 129.1, 128.5, 128.1, 126.5, 120.9, 115.4, 20.4. TLC (SiO_2, hexane:EtOAc 90:10), R_f = 0.50.

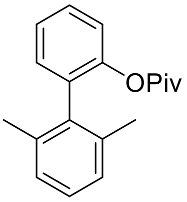

2',6'-dimethyl-[1,1'-biphenyl]-2-yl pivalate (50). It was prepared according to General Procedure D using **Lb** (72.8 mg, 0.367 mmol, 1 equiv.), pivaloyl chloride (55.0 mL, 0.441 mmol, 1.2 equiv.), Et_3N (78.0 mL, 0.551 mmol, 1.5 equiv.) and DMAP (4.58 mg, 0.0367 mmol. 0.1 equiv.) in CH_2Cl_2 (2 mL). The crude was purified by column chromatography over silica gel (hexane:EtOAc, 100:0 to 95:5) to obtain the product as a colorless oil (42.8 mg, 0.152 mmol, 41% yield). 1H-NMR (400 MHz, $CDCl_3$) δ, ppm: 7.41 (td, J = 7.7, 1.8 Hz, 1H), 7.32 (td, J = 7.5, 1.3 Hz, 1H), 7.21 (dd, J = 7.5, 1.8 Hz, 1H), 7.19 – 7.11 (m, 2H), 7.10 – 7.04 (m, 2H), 2.05 (s, 6H), 0.95 (s, 9H). 13C-NMR (100 MHz, $CDCl_3$) δ, ppm: 176.7, 148.6, 137.0, 136.6, 134.0, 130.8, 128.4, 127.6, 127.2, 126.1, 122.9, 38.8, 26.7, 20.4. HRMS (ESI-MS) m/z calculated for $C_{19}H_{22}O_2$ $[M+H]^+$ 283.1693, found 283.1695. TLC (SiO_2, hexane:EtOAc 95:5), R_f = 0.28.

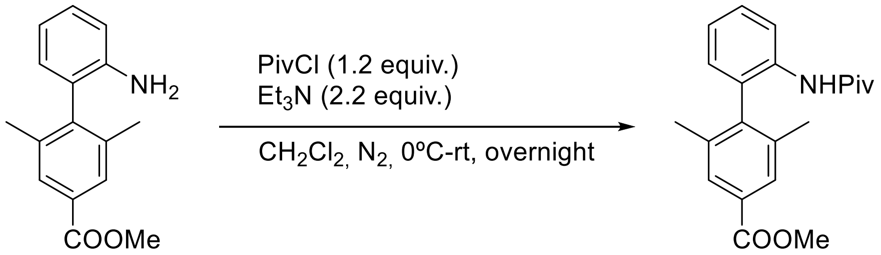

XXXVIc **51**

Methyl 2,6-dimethyl-2'-pivalamido-[1,1'-biphenyl]-4-carboxylate (51). It was prepared according to General Procedure E using **XXXVIc** (127 mg, 0.497 mmol, 1 equiv.), pivaloyl chloride (74.3 mL, 0.597 mmol, 1.2 equiv) and Et_3N (154 mL, 1.09 mmol, 2.2 equiv.) in CH_2Cl_2

(2.5 mL). The crude was purified by column chromatography over silica gel (hexane:EtOAc, 80:20) to obtain the product as a colorless solid (45.3 mg, 0.133 mmol, 27% yield). ^{1}H-NMR (400 MHz, $CDCl_3$) δ, ppm: 8.44 (dd, *J* = 8.4, 1.2 Hz, 1H), 7.87 (s, 2H), 7.40 (ddd, *J* = 8.4, 7.6, 1.6 Hz, 1H), 7.17 (dd, *J* = 7.5, 1.2 Hz, 1H), 7.03 (dd, *J* = 7.6, 1.6 Hz, 1H), 6.90 (s, 1H), 3.95 (s, 3H), 2.04 (s, 6H), 0.97 (s, 9H). ^{13}C-NMR (100 MHz, $CDCl_3$) δ, ppm: 176.4, 167.2, 141.5, 137.8, 135.0, 130.1, 129.1, 128.9, 128.5, 124.3, 120.4, 52.4, 39.8, 27.3, 20.3. HRMS (ESI-MS) *m/z* calculated for $C_{21}H_{25}NO_3$ $[M+Na]^+$ 362.1727, found 362.1721. TLC (SiO_2, hexane:EtOAc 9:1), R_f = 0.24.

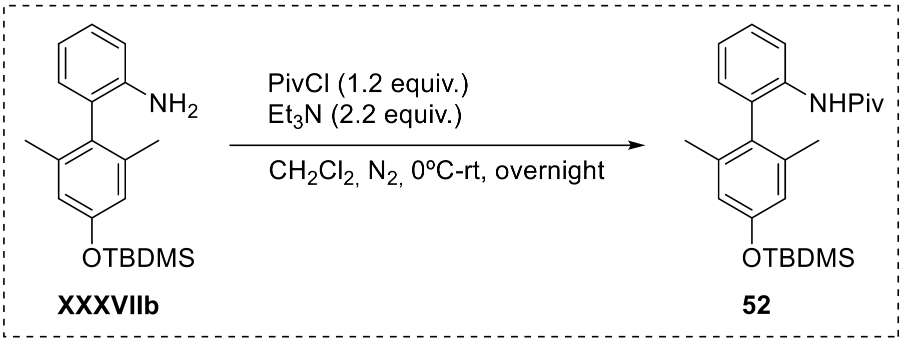

***N*-(4'-((*tert*-butyldimethylsilyl)oxy)-2',6'-dimethyl-[1,1'-biphenyl]-2-yl)pivalamide (52)**. It was prepared according to General Procedure E using **XXXVIIb** (139 mg, 0.425 mmol, 1 equiv.), pivaloyl chloride (63.4 mL, 0.510 mmol, 1.2 equiv) and Et_3N (132 mL, 0.935 mmol, 2.2 equiv.) in CH_2Cl_2 (2 mL). The crude was purified by column chromatography over silica gel (hexane:EtOAc, 80:20) to obtain the product as a colorless Aoil (45.3 mg, 0.133 mmol, 27% yield). ^{1}H-NMR (400 MHz, $CDCl_3$) δ, ppm: 8.45 (dd, *J* = 8.4, 1.2 Hz, 1H), 7.35 (ddd, *J* = 8.4, 7.5, 1.7 Hz, 1H), 7.13 (td, *J* = 7.5, 1.2 Hz, 2H), 7.06 (dd, *J* = 7.6, 1.7 Hz, 1H), 6.68 (s, 2H), 1.92 (s, 6H), 1.01 (s, 9H), 1.00 (s, 9H), 0.22 (s, 6H). ^{13}C-NMR (100 MHz, $CDCl_3$) δ, ppm: 176.3, 155.6, 138.6, 135.9, 129.7, 129.6, 129.3, 128.4, 123.9, 119.7, 119.6, 39.8, 27.3, 25.9, 20.4, 18.4, -4.2. HRMS (ESI-MS) *m/z* calculated for $C_{25}H_{37}NO_2Si$ $[M+H]^+$ 412.2666, found 412.2669. TLC (SiO_2, hexane:EtOAc 9:1), R_f = 0.4

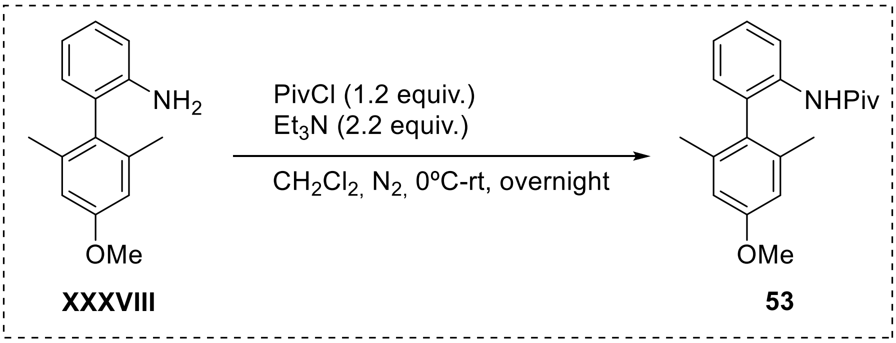

***N*-(4'-methoxy-2',6'-dimethyl-[1,1'-biphenyl]-2-yl)pivalamide (53)**. It was prepared according to General Procedure E using **XXXVIII** (120 mg, 0.528 mmol, 1 equiv.), pivaloyl chloride (78.8 mL, 0.633 mmol, 1.2 equiv) and Et_3N (164 mL, 1.16 mmol, 2.2 equiv.) in CH_2Cl_2 (2.6 mL). The crude was purified by column chromatography over silica gel (hexane:EtOAc, , 100:0 to 95:5) to obtain the product as a pale yellow solid (98.8 mg, 0.317 mmol, 60% yield). ^{1}H-NMR (400 MHz, $CDCl_3$) δ, ppm: 8.45 (dd, *J* = 8.4, 1.2 Hz, 1H), 7.36 (ddd, *J* = 8.4, 7.5, 1.7 Hz, 1H), 7.14 (td, *J* = 7.5, 1.2 Hz, 2H), 7.05 (dd, *J* = 7.5, 1.7 Hz, 1H), 6.75 (s, 2H), 3.84 (s, 3H), 1.97 (s, 6H), 1.00 (s, 9H). ^{13}C-NMR (100 MHz, $CDCl_3$) δ, ppm: 176.4, 159.5, 138.7, 136.0,

129.7, 129.6, 128.6, 128.4, 123.9, 119.7, 113.4, 55.4, 39.8, 27.4, 20.6. HRMS (ESI-MS) *m/z* calculated for $C_{20}H_{25}NO_2$ $[M+Na]^+$ 334.1777, found 334.1776. TLC (SiO_2, hexane:EtOAc 9:1), R_f = 0.28.

4. Catalytic studies

4.1. General procedure for catalytic oxidations

An HFIP solution (280 mL 0.05 M) of substrate (14.1 mmol, 1 equiv.), catalyst (0.424 mmol, 3 mol%) and carboxylic acid (1.3 or 5 equiv.) was prepared in a 2 mL vial equipped with a stirring bar. The resulting mixture was cooled to 0ºC with an ice bath and 150 mL of of H_2O_2 (21.2 mmol, 1.5 equiv.) from a 0.14 M solution in HFIP, diluted from 50% in water, Aldrich) were added dropwise by syringe pump over 30 minutes. The solution was taken off from the ice bath and 0.5 equiv. of internal standard (biphenyl) was added. The solution was filtered through a small plug of silica and basic alumina, which was subsequently rinsed with 5 mL of EtOAc twice. The mixture was then analyzed by GC to determine the conversion and product yields.

SFC analysis: after GC analysis, the crude mixture was purified through a silica plug (hexane:EtOAc) to isolate the product. It was solved in MeOH, EtOH or 2-propanol (according to SFC conditions) and its ee was determined by SFC. In case that both alcohol and aldehyde were detected in the reaction crude, either an oxidative or reductive work-up was performed prior to isolation of the product.

4.2. General procedure for isolation of the oxidized products

An HFIP solution (0.05 M) of substrate (1 equiv.), catalyst (3 mol%) and carboxylic acid (1.3 or 5 equiv.) was prepared in a 25 mL round-bottom flask equipped with a stirring bar. The resulting mixture was cooled to 0ºC with an ice bath and 1.5 equiv. of H_2O_2 (from a 0.14 solution in HFIP, diluted from 50% in water, Aldrich) were added dropwise by syringe pump over 30 minutes. Then, the solution was taken off from the ice bath, the solvent was removed under reduced pressure and the crude was purified by column chromatography over silica gel (hexane:EtOAc). Characterization of the products thus obtained was performed by NMR and HRMS, and the ee of the products was analyzed by SFC.

Considerations:

- *(R,R)*-Mn(^{iPr}iQdp) (**C6**) or *(R,R)*-Mn(^{TIPS}ecp) (**C2**) were used as catalysts.
- Acetic acid (AcOH) or 2,2-dimethylbutyric acid (DMBA) were used as carboxylic acid, either in 1.3 equiv. or 5 equiv. The conditions for each case are specified together with the characterization of the isolated products.
- Reactions were perfomed on a 0.0485 – 0.58 mmol scale.
- For substrates **1**, **4**, **19**, **24** and **33** an oxidative work-up with PCC was performed prior the isolation of the products.
- For substrates **8** and **35** a reductive work-up with $NaBH_4$ was performed prior the isolation of the products.

4.3. General procedure for oxidative work-up with PCC

Oxidation of the reaction mixture was done according to a reported procedure.[43] Before isolation of the product and after removal of the HFIP, the crude was solved in CH_2Cl_2 (0.01

M) and 2.5 equiv. of celite® and 2.5 equiv. of PCC were added. The reaction was stirred at room temperature overnight. After this time, the mixture was filtered through a pad of silica, eluted with EtOAc and the solvent was removed under reduced pressure. The crude material was then purified by column chromatography over silica gel (hexane:EtOAc).

4.4. General procedure for reductive work-up with $NaBH_4$

Oxidation of the reaction mixture was done according to a reported procedure.[44] Before isolation of the product and after removal of the HFIP, the crude was solved in anhydrous MeOH (0.01 M) and $NaBH_4$ (2.0 equiv.) was added. The reaction was stirred at room temperature overnight. After this time, the solvent was removed under reduced pressure. The crude material was rinsed with CH_2Cl_2 (2.0 mL), washed with H_2O (2 x 2.0 mL) and then purified by column chromatography over silica gel (hexane:EtOAc).

5. Additional experiments

Table S1. Solvent effect on the oxidation of **1** with H_2O_2 catalyzed by **C6**.

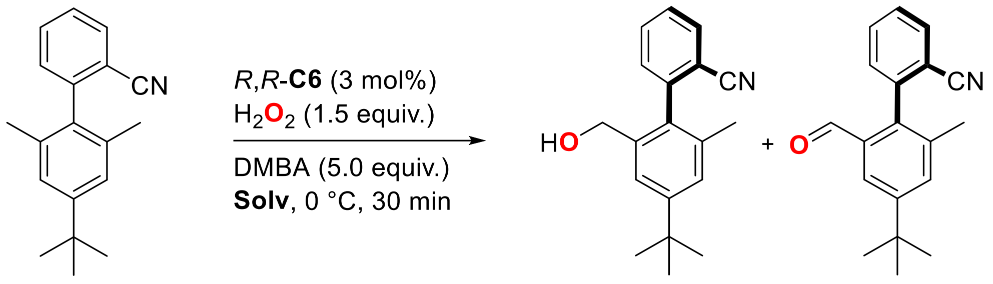

1 **1a** **1b**

Entry	Solv	% conv	% 1a yield	% 1b yield	% 1a ee	% 1b ee
**1**	MeCN	-	-	-	-	-
**2**	TFE	72	42	9	73	-
**3**	**HFIP**	**94**	**55**	**23**	**88**	**>99**

Table S2. Stability of axial chirality under oxidative and reductive treatment.

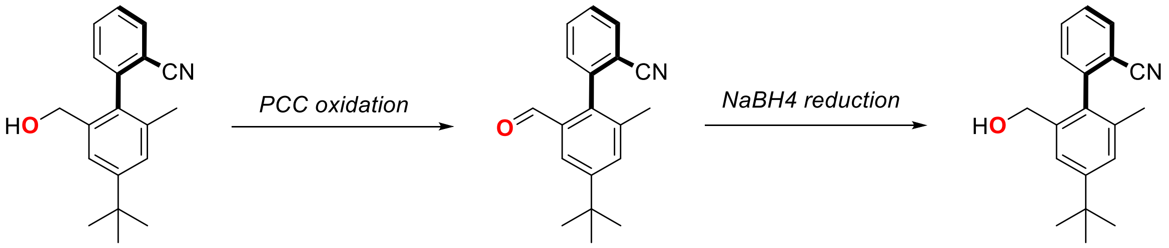

(*R*)-1a 73% ee **(*R*)-1b** 73% ee **(*R*)-1a** 73% ee

6. Characterization of the isolated products

6.1. Product scope

The absolute stereochemistry of the atropisomeric products (*R*) has been tentatively assigned in analogy to compound *R*-**28a** (*vide infra*). Oxidation products **3a**, **20b** and **21a** obtained with (*R*,*R*)-**C2** catalyst show the same absolute configuration of the other products. As example, the major enantiomer of product **20b** obtained with (*R*,*R*)-**C2** (86% ee) and (*R*,*R*)-**C6** (42% ee) appears in the same retention time (see HPLC traces in the section 12).

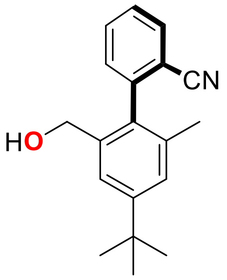

4'-(*tert*-butyl)-2'-(hydroxymethyl)-6'-methyl-[1,1'-biphenyl]-2-carbonitrile (1a). It was isolated according to the General Procedure on an 0.197 mmol scale using **C6** as catalyst and DMBA (1.3 equiv.) as carboxylic acid in HFIP at 0ºC. It was purified by column chromatography over silica gel (hexane:EtOAc, 100:0 to 80:20) to obtain the product as a colorless solid (55.3 mg, 0.109 mmol, 55% yield, 88% ee). **11.5 mg of **1b** (0.041 mmol, 21% yield, >99% ee) were also isolated.* ^{1}H-NMR (400 MHz, $CDCl_3$) δ, ppm: 7.76 (dd, *J* = 7.8, 0.8 Hz, 1H), 7.65 (td, *J* = 7.7, 1.4 Hz, 1H), 7.47 (td, *J* = 7.7, 1.3 Hz, 1H), 7.41 (d, *J* = 2.0 Hz, 1H), 7.36-7.31 (m, 1H), 7.28-7.22 (m, 1H), 4.36 (d, *J* = 12.6 Hz, 1H), 4.30 (d, *J* = 12.6 Hz, 1H), 2.03 (s, 3H), 1.37 (s, 9H). ^{13}C-NMR (100 MHz, $CDCl_3$) δ, ppm: 151.9, 144.2, 138.2, 135.9, 134.1, 133.0, 132.9, 130.8, 127.9, 126.9, 123.0, 118.1, 113.6, 63.9, 34.8, 31.5, 20.5. HRMS (ESI-MS) *m/z* calculated for $C_{19}H_{21}NO$ $[M+NH_4]^+$ 297.1961, found 297.1964. TLC (SiO_2, hexane:EtOAc 8:2), R_f = 0.24. $[a]_D^{25}$ = +8.41 (c 1.00, $CHCl_3$).

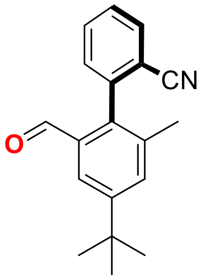

4'-(*tert*-butyl)-2'-formyl-6'-methyl-[1,1'-biphenyl]-2-carbonitrile (1b). It was isolated according to the General Procedure on an 0.197 mmol scale using **C6** as catalyst and DMBA (1.3 equiv.) as carboxylic acid in HFIP at 0ºC. The crude mixture was oxidized with PCC and purified by column chromatography over silica gel (hexane:EtOAc, 100:0 to 90:10) to obtain the product as a colorless solid (38.7 mg, 0.140 mmol, 71% yield, 92% ee). ^{1}H-NMR (400 MHz, $CDCl_3$) δ, ppm: 9.67 (s, 1H), 7.89 (d, *J* = 2.1 Hz, 1H), 7.80 (dd, *J* = 7.8, 1.4 Hz, 1H), 7.68 (td, *J* = 7.7, 1.4 Hz, 1H), 7.58 (d, *J* = 2.1 Hz, 1H), 7.53 (td, *J* = 7.7, 1.2 Hz, 1H), 7.33 (dd, *J* = 7.8, 1.2 Hz, 1H), 2.12 (s, 3H), 1.39 (s, 9H). ^{13}C-NMR (100 MHz, $CDCl_3$) δ, ppm: 191.6, 152.3, 142.0, 137.7, 137.2, 134.1, 133.3, 133.1, 132.8, 131.1, 128.4, 123.9, 117.6, 113.7. HRMS (ESI-MS) *m/z* calculated for $C_{19}H_{19}NO$ $[M+H]^+$ 278.1539, found 278.1544. TLC (SiO_2, hexane:EtOAc 8:2), R_f = 0.55. $[a]_D^{25}$ = +24.2 (c 0.500, $CHCl_3$). XRD structure found in CCDC Number 2519154.

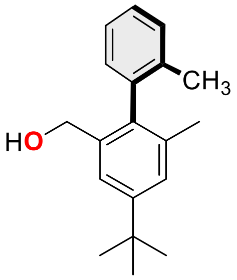

(4-(*tert*-butyl)-2',6-dimethyl-[1,1'-biphenyl]-2-yl)methanol (2a). It was isolated according to the General Procedure on a 0.141 mmol scale using **C6** as catalyst and AcOH (5 equiv.) as carboxylic acid in HFIP at room temperature. It was purified by column chromatography over silica gel (hexane) to obtain the product as a colorless solid (18.3 mg, 0.0682 mmol, 48% yield, 4% ee). ^{1}H-NMR (400 MHz, $CDCl_3$) δ, ppm: 7.36 (d, *J* = 2.0 Hz, 1H), 7.30-7.26 (m, 2H), 7.25-7.20 (m, 2H), 7.09-7.04 (m, 1H), 4.30 (s, 2H), 1.98 (s, 3H), 1.96 (s, 3H), 1.37 (s, 9H). ^{13}C-NMR (100 MHz, $CDCl_3$) δ, ppm: 150.2, 139.2, 137.8, 137.0, 136.2, 135.7, 130.1, 129.2, 127.4, 126.2, 126.0,

122.2, 64.1, 34.5, 31.4, 20.3, 19.7. HRMS (ESI-MS) *m/z* calculated for $C_{19}H_{24}O$ $[M+Na]^+$ 291.1719, found 291.1717. TLC (SiO_2, hexane:EtOAc 9:1), R_f = 0.33. $[a]_D^{25}$ = -1.76 (c 0.380, $CHCl_3$).

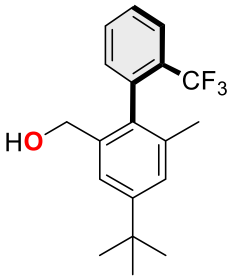

(4-(*tert*-butyl)-6-methyl-2'-(trifluoromethyl)-[1,1'-biphenyl]-2-yl)methanol (3a). It was isolated according to the General Procedure on a 0.081 mmol scale using **C2** as catalyst and DMBA (1.3 equiv.) as carboxylic acid in HFIP at 0ºC. It was purified by column chromatography over silica gel (hexane:EtOAc, 8:2) to obtain the product as a colorless solid (22.5 mg, 0.07 mmol, 85% yield, 81% ee). ^{1}H-NMR (400 MHz, $CDCl_3$) δ, ppm: 7.77 (d, *J* = 7.9 Hz, 1H), 7.59 (t, *J* = 7.6 Hz, 1H), 7.49 (t, *J* = 7.7 Hz, 1H), 7.40 (d, *J* = 2.0 Hz, 1H), 7.25-7.18 (m, 2H), 4.30 (d, *J* = 12.8 Hz, 1H), 4.23 (d, *J* = 12.8 Hz, 1H), 1.94 (s, 3H), 1.37 (s, 9H). ^{13}C-NMR (100 MHz, $CDCl_3$) δ, ppm: 151.0, 138.3, 136.0, 134.3, 131,9, 131.8, 127.6, 126.4 (q, *J* = 5.1 Hz), 126.0, 124.0 (q, *J* = 274.0 Hz), 121.8, 63.6, 34.6, 31.4, 20.5. ^{19}F-NMR (377 MHz, $CDCl_3$) δ, ppm: -61.6. HRMS (ESI-MS) *m/z* calculated for $C_{19}H_{21}F_3O$ $[M+Na]^+$ 345.1437, found 345.1435. TLC (SiO_2, hexane:EtOAc 8:2), R_f = 0.38. $[a]_D^{25}$ = +0.844 (c 0.940, $CHCl_3$).

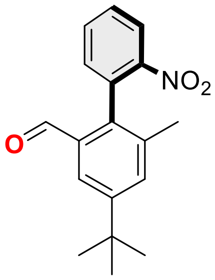

4-(*tert*-butyl)-6-methyl-2'-nitro-[1,1'-biphenyl]-2-carbaldehyde (4b). It was isolated according to the General Procedure on an 0.140 mmol scale using **C6** as catalyst and DMBA (1.3 equiv.) as carboxylic acid in HFIP at 0ºC. The crude mixture was oxidized with PCC and purified by column chromatography over silica gel (hexane:EtOAc, 9:1) to obtain the product as a pale yellow solid (35.4 mg, 0.119 mmol, 85% yield, 92% ee). ^{1}H-NMR (400 MHz, $CDCl_3$) δ, ppm: δ 9.70 (s, 1H), 8.13 (dd, J = 8.2, 1.4 Hz, 1H), 7.84 (d, J = 2.1 Hz, 1H), 7.69 (td, J = 7.5, 1.4 Hz, 1H), 7.60 (ddd, J = 8.9, 7.5, 1.5 Hz, 1H), 7.52 (d, J = 2.1 Hz, 1H), 7.29-7.22 (m, 1H), 2.02 (s, 3H), 1.39 (s, 9H). ^{13}C-NMR (100 MHz, $CDCl_3$) δ, ppm: 191.9, 151.7, 149.2, 137.2, 136.7, 133.8, 133.4, 133.2, 133.0, 132.4, 129.1, 124.6, 124.3, 34.9, 31.3, 20.0. HRMS (ESI-MS) *m/z* calculated for $C_{18}H_{19}NO_3$ $[M+H]^+$ 298.1438, found 298.1438. TLC (SiO_2, hexane:EtOAc 8:2), R_f = 0.5. $[a]_D^{25}$ = +23.0 (c 0.910, $CHCl_3$).

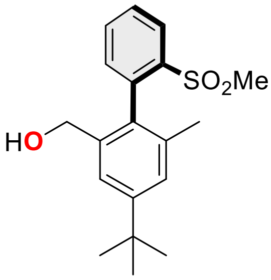

(4-(*tert*-butyl)-6-methyl-2'-(methylsulfonyl)-[1,1'-biphenyl]-2-yl)methanol (5a). It was isolated according to the General Procedure on an 0.101 mmol scale using **C6** as catalyst and DMBA (5 equiv.) as carboxylic acid in HFIP at 0ºC. It was purified by column chromatography over silica gel (hexane:EtOAc, 5:1) to obtain the product as a colorless solid (21.5 mg, 0.0725 mmol, 72% yield, 83% ee). ^{1}H-NMR (400 MHz, $CDCl_3$) δ, ppm: 8.26 (dd, *J* = 8.0, 1.4 Hz, 1H), 7.68 (td, *J* = 7.6, 1.4 Hz, 1H), 7.59 (td, *J* = 7.6, 1.4 Hz, 1H), 7.52 (d, *J* = 2.0 Hz, 1H), 7.28-719 (m, 2H), 4.29-4.19 (m, 2H), 2.71 (br, 1H), 2.69 (s, 3H), 1.97 (s, 3H), 1.37 (s, 9H). ^{13}C-NMR (100 MHz, $CDCl_3$) δ, ppm: 152.4, 140.3, 139.4, 139.0, 135.1, 133.8, 133.1, 132.8, 129.0, 128.4, 126.3, 123.9, 63.4, 42.7, 34.8, 31.5, 21.1. HRMS (ESI-MS) *m/z* calculated for $C_{19}H_{24}O_3S$ $[M+NH_4]^+$ 350.1784, found 350.1784. TLC (SiO_2, hexane:EtOAc 8:2), R_f = 0.09. $[a]_D^{25}$ = +5.52 (c 0.917, $CHCl_3$). XRD structure found in CCDC Number 2519155.

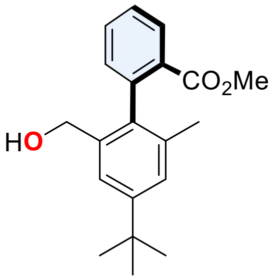

Methyl 4'-(*tert*-butyl)-2'-(hydroxymethyl)-6'-methyl-[1,1'-biphenyl]-2-carboxylate (6a). It was isolated according to the General Procedure on a 0.089 mmol scale using **C6** as catalyst and DMBA (1.3 equiv.) as carboxylic acid in HFIP:TFE (2:1) at -26ºC (cooled with an *o*-xylene / liquid N_2 bath). It was purified by column chromatography over silica gel (hexane:EtOAc, 5:1) to obtain the product as a colorless solid (72.5 mg, 0.073 mmol, 81% yield, 91% ee). ^{1}H-NMR (400 MHz, $CDCl_3$) δ, ppm: 7.93 (dd, *J* = 7.8, 1.5 Hz, 1H), 7.56 (td, *J* = 7.6, 1.5 Hz, 1H), 7.44 (td, *J* = 7.6, 1.3 Hz, 1H), 7.36 (s, 1H), 7.20-7.15 (m, 2H), 4.36-4.21 (m, 2H), 3.66 (s, 3H), 2.36 (br, 1H), 1.90 (s, 3H), 1.36 (s, 9H). ^{13}C-NMR (100 MHz, $CDCl_3$) δ, ppm: 168.6, 150.5, 141.0, 137.7, 137.2, 135.0, 132.1, 131.2, 131.0, 130.1, 127.5, 126.3, 123.4, 64.5, 52.4, 34.6, 31.5, 20.7. HRMS (ESI-MS) *m/z* calculated for $C_{20}H_{24}O_3$ $[M+H]^+$ 313.1798, found 313.1790. TLC (SiO_2, hexane:EtOAc 8:2), R_f = 0.25. $[a]_D^{25}$ = +5.75 (c 0.750, $CHCl_3$).

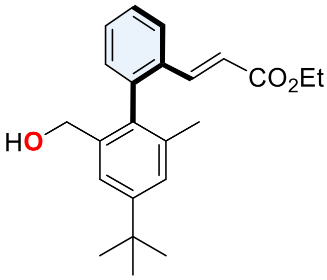

Ethyl (E)-3-(4'-(tert-butyl)-2'-(hydroxymethyl)-6'-methyl-[1,1'-biphenyl]-2-yl)acrylate (7a). It was isolated according to the General Procedure on a 0.05 mmol scale using **C6** as catalyst and DMBA (1.3 equiv.) as carboxylic acid in HFIP at 0ºC. It was purified by column chromatography over silica gel (hexane:EtOAc, 8:2) to obtain the product as a colorless solid (15.2 mg, 0.0426 mmol, 77% yield, 88% ee). ^{1}H-NMR (400 MHz, $CDCl_3$) δ, ppm: 7.76 (dd, *J* = 7.4, 1.8 Hz, 1H), 7.48 – 7.38 (m, 3H), 7.32 (d, *J* = 16.0 Hz, 1H), 7.26 (d, *J* = 2.0 Hz, 1H), 7.21 – 7.17 (m, 1H), 6.30 (d, *J* = 16.0 Hz, 1H), 4.28 (s, 2H), 4.16 (q, *J* = 7.1 Hz, 2H), 1.95 (s, 3H), 1.40 (s, 9H), 1.26 (t, *J* = 7.1 Hz, 3H). ^{13}C-NMR (100 MHz, $CDCl_3$) δ, ppm: 166.9, 151.0, 142.4, 140.6, 138.2, 135.9, 135.0, 133.2, 130.5, 130.2, 127.8, 126.6, 126.4, 122.4, 119.2, 63.9, 60.4, 34.6, 31.4, 20.6, 14.2. HRMS (ESI-MS) *m/z* calculated for $C_{23}H_{28}O_3$ $[M+NH_4]^+$ 370.2377, found 370.2379. TLC (SiO_2, hexane:EtOAc 8:2), R_f = 0.3. $[a]_D^{25}$ = +16.42 (c 0.390, $CHCl_3$).

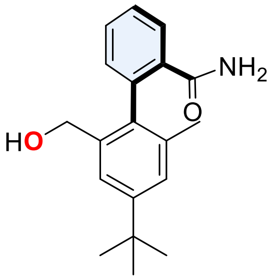

4'-(*tert*-butyl)-2'-(hydroxymethyl)-6'-methyl-[1,1'-biphenyl]-2-carboxamide (8a). It was isolated according to the General Procedure on a 0.101 mmol scale using **C6** as catalyst and DMBA (5 equiv.) as carboxylic acid in HFIP at 0ºC. It was purified by column chromatography over silica gel (hexane:EtOAc, 5:1 to 3:1) to obtain the product as a colorless (18.0 mg, 0.0605 mmol, 60% yield, 28% ee). ^{1}H-NMR (400 MHz, $CDCl_3$) δ, ppm: 7.77 (dd, *J* = 7.6, 1.5 Hz, 1H), 7.49 (td, *J* = 7.5, 1.5 Hz, 1H), 7.42 (td, *J* = 7.5, 1.4 Hz, 1H), 7.31 (d, *J* = 2.1 Hz, 1H), 7.24-7.18 (m, 1H), 7.12 (dd, *J* = 7.5, 1.4 Hz, 1H), 6.48 (br, 1H), 5.48 (br, 1H), 4.50 (dd, *J* = 11.2, 3.3 Hz, 1H), 4.31 (dd, *J* = 11.2, 4.9 Hz, 1H), 3.04 (br, 1H), 1.95 (s, 3H), 1.34 (s, 9H). ^{13}C-NMR (100 MHz, $CDCl_3$) δ, ppm: 171.6, 151.2, 138.0, 137.3, 136.8, 136.0, 135.8, 130.8, 130.2, 128.7, 127.7, 127.2, 124.3, 64.4, 34.6, 31.5, 20.7. HRMS (ESI-MS) *m/z* calculated for $C_{19}H_{23}NO_2$ $[M+Na]^+$ 320.1621, found 320.1617. TLC (SiO_2, hexane:EtOAc 7:3), R_f = 0.06. $[a]_D^{25}$ = +15.7 (c 1.000, $CHCl_3$).

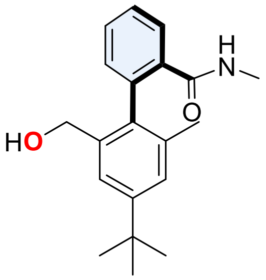

4'-(*tert*-butyl)-2'-(hydroxymethyl)-*N*,6'-dimethyl-[1,1'-biphenyl]-2-carboxamide (9a). It was isolated according to the General Procedure on a 0.099 mmol scale using **C6** as catalyst and DMBA (5 equiv.) as carboxylic acid in HFIP at 0ºC. The crude was reduced with $NaBH_4$ and purified by column chromatography over silica gel (hexane:EtOAc, 3:1 to 1:1) to obtain the product as a colorless solid (25.9 mg, 0.083 mmol, 83% yield, 88% ee). ^{1}H-NMR (400 MHz, $CDCl_3$) δ, ppm: 7.66 (dd, *J* = 7.6, 1.6 Hz, 1H), 7.47 (td, *J* = 7.5, 1.5 Hz, 1H), 7.41 (td, *J* = 7.6, 1.5 Hz, 1H), 7.31 (d, *J* = 2.0 Hz, 1H), 7.21-7.18 (m, 1H), 7.13 (dd, *J* = 7.5, 1.4 Hz, 1H), 6.46 (br, 1H), 4.50 (d, *J* = 11.0 Hz, 1H), 4.31 (d, *J* = 11.0 Hz, 1H), 2.62 (d, *J* = 4.7 Hz, 3H), 1.94 (s, 3H), 1.35 (s, 9H). ^{13}C-NMR (100 MHz, $CDCl_3$) δ, ppm: 170.4, 151.0, 137.9 , 137.4, 137.1, 136.9, 135.8, 130.3, 130.1, 128.2, 127.7, 127.0, 124.4, 64.4, 34.6, 31.5, 26.7, 20.7. HRMS (ESI-MS) *m/z* calculated for $C_{20}H_{25}NO_2$ $[M+H]^+$ 312.1958, found 312.1954. TLC (SiO_2, hexane:EtOAc 7:3), R_f = 0.22. $[a]_D^{25}$ = +26.3 (c 0.740, $CHCl_3$).

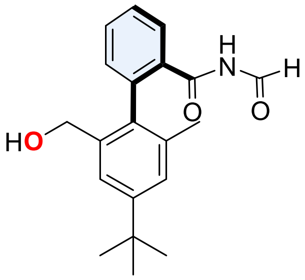

4'-(*tert*-butyl)-*N*-formyl-2'-(hydroxymethyl)-6'-methyl-[1,1'-biphenyl]-2-carboxamide (10a). It was isolated according to the General Procedure on an 0.049 mmol scale using **C6** as catalyst and DMBA (5 equiv.) as carboxylic acid in HFIP at 0ºC. It was purified by column chromatography over silica gel (hexane:EtOAc, 10:1 to 5:1) to obtain the product as a colorless solid (8.90 mg, 0.0274 mmol, 56% yield, >99% ee). ^{1}H-NMR (400 MHz, $CDCl_3$) δ, ppm: 9.93 (d, *J* = 9.5 Hz, 1H), 8.96 (d, *J* = 9.5 Hz, 1H), 7.79 (dd, *J* = 7.6, 1.5 Hz, 1H), 7.59 (td, *J* = 7.6, 1.5 Hz, 1H), 7.51 (td, *J* = 7.6, 1.3 Hz, 1H), 7.29 (d, *J* = 2.0 Hz, 1H), 7.27-7.22 (m, 1H), 7.20 (dd, *J* = 7.6, 1.3 Hz, 1H), 4.70 (d, *J* = 10.9 Hz, 1H), 4.47 (d, *J* = 10.9 Hz, 1H), 1.92 (s, 3H), 1.34 (s, 9H). ^{13}C-NMR (100 MHz, $CDCl_3$) δ, ppm: 170.0, 162.6, 152.0, 138.1, 136.6, 136.1, 136.0, 135.1, 132.1, 130.0, 129.3, 128.1, 127.8, 124.9, 64.8, 34.7, 31.4, 20.5. HRMS (ESI-MS) *m/z* calculated for $C_{20}H_{23}NO_3$ $[M+Na]^+$ 348.1570, found 348.1565. TLC (SiO_2, hexane:EtOAc 7:3), R_f = 0.71. $[a]_D^{25}$ = +34.9 (c 0.220, $CHCl_3$).

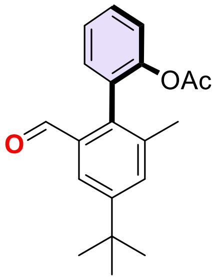

4'-(*tert*-butyl)-2'-formyl-6'-methyl-[1,1'-biphenyl]-2-yl acetate (11b). It was isolated according to the General Procedure on a 0.169 mmol scale using **C6** as catalyst and DMBA (5 equiv.) as carboxylic acid in HFIP at 0ºC. The crude mixture was oxidized with PCC and purified by column chromatography over silica gel (hexane:EtOAc, 90:10) to obtain the product as a colorless oil (43.5 mg, 0.140 mmol, 83% yield, 98% ee). ^{1}H-NMR (400 MHz, $CDCl_3$) δ, ppm: 9.67 (s, 1H), 7.87 (d, *J* = 2.1 Hz, 1H), 7.52 (d, *J* = 2.1 Hz, 1H), 7.46 (td, *J* = 7.8, 1.7 Hz, 1H), 7.35 (td, *J* = 7.5, 1.2 Hz, 1H), 7.28-7.23 (m, 1H), 7.19 (dd, *J* = 8.1, 1.2 Hz, 1H), 2.12 (s, 3H), 1.84 (s, 3H), 1.37 (s, 9H). ^{13}C-NMR (100 MHz, $CDCl_3$) δ, ppm: 193.1, 169.1, 151.4, 149.0, 137.7, 137.5, 134.1, 132.8, 131.8, 130.4, 129.6, 126.3, 122.8, 121.4, 34.9, 31.3, 20.4, 20.0. HRMS (ESI-MS) *m/z* calculated for $C_{20}H_{22}O_3$ $[M+Na]^+$ 333.1461, found 333.1461. TLC (SiO_2, hexane:EtOAc 9:1), R_f = 0.23. $[a]_D^{25}$ = -18.0 (c 0.990, $CHCl_3$).

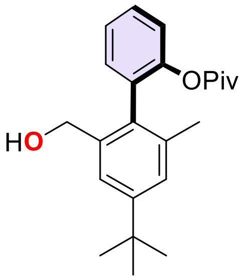

4'-(*tert*-butyl)-2'-(hydroxymethyl)-6'-methyl-[1,1'-biphenyl]-2-yl pivalate (12a). It was isolated according to the General Procedure on an 0.086 mmol scale using **C6** as catalyst and AcOH (5 equiv.) as carboxylic acid in HFIP at 0ºC. It was purified by column chromatography over silica gel (hexane:EtOAc, 10:1) to obtain the product as a colorless oil (8.9 mg, 0.0251 mmol, 85% yield, >99% ee). ^{1}H-NMR (400 MHz, $CDCl_3$) δ, ppm: 7.44-7.37 (m, 2H), 7.32 (td, *J* = 7.5, 1.3 Hz, 1H), 7.24-7.16 (m, 2H), 7.11 (dd, *J* = 8.1, 1.3 Hz, 1H), 4.41 (dd, *J* = 12.4, 4.2 Hz, 1H), 4.28 (dd, *J* = 12.4, 8.8 Hz, 1H), 2.13 (dd, *J* = 8.8, 4.1 Hz, 1H), 2.03 (s, 3H), 1.33 (s, 9H), 0.90 (s, 9H). ^{13}C-NMR (100 MHz, $CDCl_3$) δ, ppm: 177.7, 151.3, 148.9, 139.6, 136.7, 133.0, 132.4, 131.3, 128.9, 126.3, 126.1, 123.5, 122.7, 63.5, 38.9, 34.7, 31.5, 26.7, 20.5. HRMS (ESI-MS) *m/z* calculated for $C_{23}H_{30}O_3$ $[M+Na]^+$ 377.2087, found 377.2086. TLC (SiO$_2$, hexane:EtOAc 8:2), R_f = 0.46. $[a]_D^{25}$ = +5.48 (c 0.200, $CHCl_3$).

***Note**: to determine the ee, this product was derivatized to the corresponding aldehyde (**11b**).*[43]

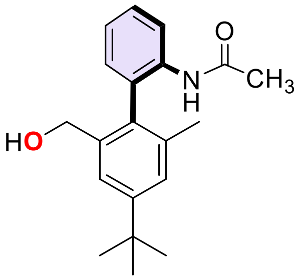

***N*-(4'-(*tert*-butyl)-2'-(hydroxymethyl)-6'-methyl-[1,1'-biphenyl]-2-yl)acetamide (13a).** It was isolated according to the General Procedure on an 0.150 mmol scale using **C6** as catalyst and DMBA (1.3 equiv.) as carboxylic acid in HFIP at 0ºC. It was purified by column chromatography over silica gel (hexane:EtOAc, 10:1 to 3:1) to obtain the product as a colorless oil (16.5 mg, 0.053 mmol, 35% yield, >99% ee). ^{1}H-NMR (400 MHz, $CDCl_3$) δ, ppm: 8.23 (d, *J* = 8.2 Hz, 1H), 7.41-7.36 (m, 2H), 7.30-7.28 (m, 1H), 7.18 (t, *J* = 7.4 Hz, 1H), 7.08-7.04 (m, 1H), 7.01 (br, 1H), 4.35 (s, 2H), 1.98 (s, 3H), 1.92 (s, 3H), 1.38 (s, 9H). ^{13}C-NMR (100 MHz, $CDCl_3$) δ, ppm: 168.5, 151.5, 138.5, 136.9, 135.8, 133.0, 129.9, 129.7, 128.5, 127.1, 124.5, 123.5, 121.9, 64.0, 34.6, 31.4, 29.7, 20.4. HRMS (ESI-MS) *m/z* calculated for $C_{20}H_{25}NO_2$ $[M+Na]^+$ 334.1775, found 334.1777. TLC (SiO$_2$, hexane:EtOAc 7:3), R_f = 0.08. $[a]_D^{25}$ = -18.4 (c 0.630, $CHCl_3$).

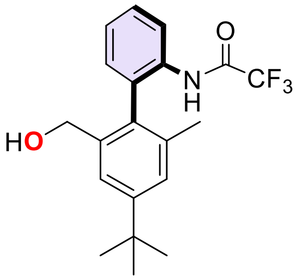

***N*-(4'-(*tert*-butyl)-2'-(hydroxymethyl)-6'-methyl-[1,1'-biphenyl]-2-yl)-2,2,2-trifluoroacetamide (14a).** It was isolated according to the General Procedure on an 0.100 mmol scale using **C6** as catalyst and DMBA (5 equiv.) as carboxylic acid in HFIP at 0ºC. It was purified by column chromatography over silica gel (hexane:EtOAc, 90:10) to obtain the product as a colorless solid (23.4 mg, 0.064 mmol, 64% yield, >99%% ee). ^{1}H-NMR (400 MHz, $CDCl_3$) δ, ppm: 8.28 (br, 1H), 8.08 (dd, *J* = 8.1, 1.2 Hz, 1H), 7.46 (td, *J* = 7.7, 1.6 Hz, 1H), 7.38 – 7.28 (m, 3H), 7.18 (dd, *J* = 7.7, 1.6 Hz, 1H), 4.39 (d, *J* = 11.5 Hz, 1H), 4.31 (d, *J* = 11.5 Hz, 1H), 1.96 (s, 3H), 1.37 (s, 9H). ^{13}C-NMR (100 MHz, $CDCl_3$) δ, ppm: 154.9 (q, *J* = 37.0 Hz), 152.1, 137.8, 137.0, 133.4, 132.6, 132.4, 130.1, 128.7, 127.5, 123.9, 122.9, 115.7 (q, *J* = 288.7 Hz), 64.4, 34.6, 31.3, 20.4. ^{19}F-NMR (377 MHz, $CDCl_3$) δ, ppm: -77.3. HRMS (ESI-MS) *m/z* calculated for $C_{20}H_{22}F_3NO_2$ $[M+Na]^+$ 388.1495, found 388.1492. TLC (SiO$_2$, hexane:EtOAc 8:2), R_f = 0.43. $[a]_D^{25}$ = -20.0 (c 0.330, $CHCl_3$).

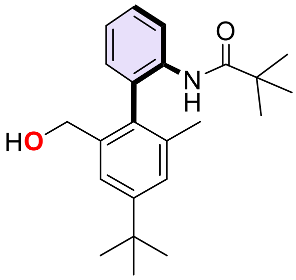

***N*-(4'-(*tert*-butyl)-2'-(hydroxymethyl)-6'-methyl-[1,1'-biphenyl]-2-yl)pivalamide (15a).** It was isolated according to the General Procedure on a 0.148 mmol scale using **C6** as catalyst and DMBA as carboxylic acid in HFIP at 0ºC. It was purified by column chromatography over silica gel (hexane:EtOAc, 10:1 to 5:1) to obtain the product as a colorless solid (27.3 mg, 0.077 mmol, 52% yield, >99% ee). ^{1}H-NMR (400 MHz, $CDCl_3$) δ, ppm: 8.23 (dd, *J* = 8.2, 1.2 Hz, 1H), 7.43 (d, *J* = 2.0 Hz, 1H), 7.39 (td, *J* = 7.8, 1.8 Hz, 1H), 7.31 (d, *J* = 2.0 Hz, 1H), 7.19 (td, *J* = 7.4, 1.2 Hz, 1H), 7.16-7.11 (m, 2H), 4.34 (*d*, J = 5.4 Hz, 2H), 1.97 (s, 3H), 1.66 (t, *J* = 5.4 Hz, 1H), 1.57 (s, 3H), 1.36 (s, 9H), 0.93 (s, 9H). ^{13}C-NMR (100 MHz, $CDCl_3$) δ, ppm: 176.7, 152.0, 139.0, 137.1, 136.0, 133.2, 130.3, 129.2, 128.7, 127.0, 124.5, 123.6, 121.7, 64.0, 39.6, 34.8, 31.5, 27.2, 20.3. HRMS (ESI-MS) *m/z* calculated for $C_{23}H_{31}NO_2$ $[M+Na]^+$ 376.2247, found 376.2253. TLC (SiO_2, hexane:EtOAc 7:3), R_f = 0.50. $[a]_D^{25}$ = -12.6 (c 0.130, $CHCl_3$). XRD structure found in CCDC Number 2519156.

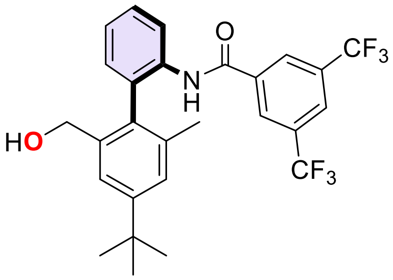

***N*-(4'-(*tert*-butyl)-2'-(hydroxymethyl)-6'-methyl-[1,1'-biphenyl]-2-yl)-3,5-bis(trifluoromethyl)benzamide (16a).** It was isolated according to the General Procedure on an 0.099 mmol scale using **C6** as catalyst and AcOH (5 equiv.) as carboxylic acid in HFIP at 0ºC. It was purified by column chromatography over silica gel (hexane:EtOAc, 15:1) to obtain the product as a colorless solid (25.8 mg, 0.051 mmol, 51% yield, 96% ee). ^{1}H-NMR (400 MHz, $CDCl_3$) δ, ppm: 8.34 (d, *J* = 8.4 Hz, 1H), 8.27 (br, 1H), 7.98 (s, 2H), 7.92 (s, 1H), 7.49 (td, *J* = 7.8, 1.6 Hz, 1H), 7.44 (d, *J* = 2.0 Hz, 1H), 7.34-7.27 (m, 2H), 7.19 (dd, *J* = 7.5, 1.6 Hz, 1H), 4.47 (d, *J* = 11.3 Hz, 1H), 4.41 (d, *J* = 11.3 Hz, 1H), 2.00 (s, 3H), 1.36 (s, 9H). ^{13}C-NMR (100 MHz, $CDCl_3$) δ, ppm: 162.1, 152.3, 138.0, 137.2, 137.0, 135.4, 133.3, 132.3 (q, *J* = 33.8 Hz), 131.6, 130.0, 128.9, 127.7, 127.5 (q, *J* = 4.0 Hz), 125.7, 125.1, 124.4 (q, *J* = 275 Hz), 122.5 ^{19}F-NMR (377 MHz, $CDCl_3$) δ, ppm: -64.0. HRMS (ESI-MS) *m/z* calculated for $C_{27}H_{25}F_6NO_2$ $[M+Na]^+$ 532.1682, found 532.1685. TLC (SiO_2, hexane:EtOAc 8:2), R_f = 0.45. $[a]_D^{25}$ = -6.11 (c 0.490, $CHCl_3$).

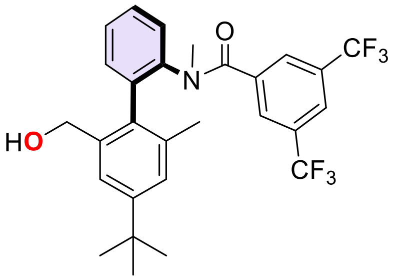

***N*-(4'-(*tert*-butyl)-2'-(hydroxymethyl)-6'-methyl-[1,1'-biphenyl]-2-yl)-*N*-methyl-3,5-bis(trifluoromethyl) benzamide (17a).** It was isolated according to the General Procedure on an 0.086 mmol scale using **C6** as catalyst and AcOH (5 equiv.) as carboxylic acid in HFIP at 0ºC. It was purified by column chromatography over silica gel (hexane:EtOAc, 80:20) to obtain the product as a colorless solid (25.4 mg, 0.064 mmol, 74% yield, >99% ee). Structure confirmed by XRD, NMR obtained as a non-interchangeable mixture of conformers. ^{19}F-NMR (377 MHz, $CDCl_3$, 263K) δ, ppm: -63.7, -63.9, -64.0. HRMS (ESI-MS) *m/z* calculated for $C_{28}H_{27}F_6NO_2$ $[M+H]^+$ 524.2019, found 524.2029. TLC (SiO_2, hexane:EtOAc 8:2), R_f = 0.21. $[a]_D^{25}$ = -0.535 (c 0.580, $CHCl_3$). XRD structure found in CCDC Number 2525663.

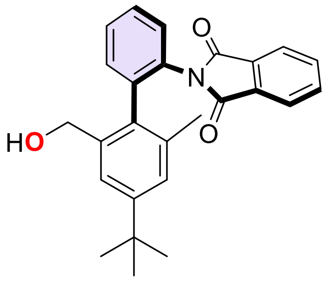

2-(4'-(*tert*-butyl)-2'-(hydroxymethyl)-6'-methyl-[1,1'-biphenyl]-2-yl)isoindoline-1,3-dione (18a). It was isolated according to the General Procedure on an 0.104 mmol scale using **C6** as catalyst and DMBA (1.3 equiv.) as carboxylic acid in HFIP at 0ºC. It was purified by column chromatography over silica gel (hexane:EtOAc, 80:20) to obtain the product as a colorless solid (35.0 mg, 0.088 mmol, 84% yield, 95% ee). ^{1}H-NMR (400 MHz, $CDCl_3$) δ, ppm: 7.86-7.78 (m, 1H), 7.74-7.63 (m, 3H), 7.55-7.48 (m, 2H), 7.41-7.34 (m, 2H), 7.33-7.28 (m, 1H), 7.01 (d, *J* = 2.0 Hz, 1H), 4.54 (d, *J* = 12.4 Hz, 1H), 4.39 (d, *J* = 12.4 Hz, 1H), 2.63 (s, 1H), 1.97 (s, 3H), 1.23 (s, 9H). ^{13}C-NMR (100 MHz, $CDCl_3$) δ, ppm: 167.5, 167.4, 150.8, 139.7, 139.2, 135.9, 134.4, 134.4, 133.3, 132.1, 131.8, 131.7, 130.9, 129.6, 129.4, 128.5, 126.0, 123.8, 123.7, 123.4, 63.5, 34.5, 31.4, 20.5. HRMS (ESI-MS) *m/z* calculated for $C_{26}H_{25}NO_3$ $[M+Na]^+$ 422.1727, found 422.1711. TLC (SiO_2, hexane:EtOAc 7:3), R_f = 0.38. $[a]_D^{25}$ = -13.5 (c 0.720, $CHCl_3$). XRD structure found in CCDC Number 2519273.

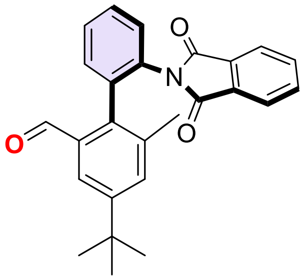

4-(*tert*-butyl)-2'-(1,3-dioxoisoindolin-2-yl)-6-methyl-[1,1'-biphenyl]-2-carbaldehyde (18b). It was isolated according to the General Procedure on an 0.580 mmol scale using **C6** as catalyst and DMBA (1.3 equiv.) as carboxylic acid in HFIP at 0ºC. The crude mixture was oxidized with PCC and purified by column chromatography over silica gel (hexane:EtOAc, 85:15) to obtain the product as a colorless solid (185 mg, 0.466 mmol, 80% yield, 97% ee). ^{1}H-NMR (400 MHz, $CDCl_3$) δ, ppm: 9.86 (s, 1H), 7.82 (dd, *J* = 5.9, 1.8 Hz, 2H), 7.78 – 7.73 (m, 1H), 7.72 – 7.66 (m, 2H), 7.63 – 7.57 (m, 2H), 7.46 – 7.42 (m, 1H), 7.42 – 7.36 (m, 2H), 2.11 (s, 3H), 1.28 (s, 9H). ^{13}C-NMR (100 MHz, $CDCl_3$) δ, ppm: 192.9, 167.2, 166.8, 151.0, 138.3, 137.2, 136.8, 134.3, 134.2, 134.2, 132.4, 132.0, 131.7, 131.5, 131.5, 129.6, 129.3, 129.1, 123.9, 123.5, 121.2, 34.6, 31.1, 19.8. HRMS (ESI-MS) *m/z* calculated for $C_{26}H_{23}NO_3$ $[M+Na]^+$ 420.1570, found 420.1569. TLC (SiO_2, hexane:EtOAc 8:2), R_f = 0.45. $[a]_D^{25}$ = -7.86 (c 0.940, $CHCl_3$).

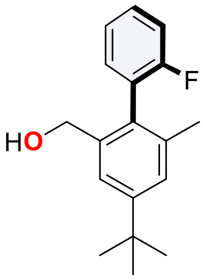

(4-(*tert*-butyl)-2'-fluoro-6-methyl-[1,1'-biphenyl]-2-yl)methanol (19a). It was isolated according to the General Procedure on a 0.051 mmol scale using **C6** as catalyst and AcOH (1.3 equiv.) as carboxylic acid in HFIP at 0ºC. It was purified by column chromatography over silica gel (hexane:EtOAc, 90:10) to obtain the product as a colorless oil (38.1 mg, 95.6 mmol, 72% yield, 56% ee). ^{1}H-NMR (400 MHz, $CDCl_3$) δ, ppm: 7.45 (d, *J* = 2.1 Hz, 1H), 7.43-7.35 (m, 1H), 7.31-7.29 (m, 1H), 7.25-7.16 (m, 3H), 4.44 (d, *J* = 12.6 Hz, 1H), 4.40 (d, *J* = 12.6 Hz, 1H), 2.11 (s, 3H), 1.41 (s, 9H). ^{13}C-NMR (100 MHz, $CDCl_3$) δ, ppm: 159.9 (d, *J* = 243.9 Hz), 151.2, 13.8, 136.7, 131.8 (d, *J* = 3.6 Hz), 131.3, 129.5 (d, *J* = 7.8 Hz), 126.8 (d, *J* = 17.7 Hz), 126.5, 124.3 (d, *J* = 3.7 Hz), 122.7, 115.9 (d, *J* = 22.4 Hz), 64.1, 34.7, 31.5, 20.6z. ^{19}F-NMR (377 MHz, $CDCl_3$) δ, ppm: -115.3 (dt, *J* = 9.4, 5.9 Hz). HRMS (ESI-MS) *m/z* calculated for $C_{18}H_{21}FO$ $[M-H]^-$ 271.1504, found 271.1501. TLC (SiO_2, hexane:EtOAc 9:1), R_f = 0.23. $[a]_D^{25}$ = -5.52 (c 0.180, $CHCl_3$).

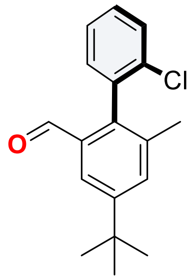

4-(*tert*-butyl)-2'-chloro-6-methyl-[1,1'-biphenyl]-2-carbaldehyde (20b). It was isolated according to the General Procedure on an 0.197 mmol scale using **C2** as catalyst and DMBA (5 equiv.) as carboxylic acid in HFIP at 0ºC. The crude mixture was oxidized with PCC and purified by column chromatography over silica gel (hexane:EtOAc, 50:1 to 10:1) to obtain the product as a colorless oil (19.8 mg, 0.069 mmol, 65% yield, 86% ee). ^{1}H-NMR (400 MHz, $CDCl_3$) δ, ppm: 9.63 (s, 1H), 7.89 (d, *J* = 2.1 Hz, 1H), 7.57-7.54 (m, 1H), 7.54-7.49 (m, 1H), 7.40-7.33 (m, 2H), 7.23-7.20 (m, 1H), 2.08 (s, 3H), 1.39 (s, 9H). ^{13}C-NMR (100 MHz, $CDCl_3$) δ, ppm: 192.5, 151.5, 139.9, 137.2, 136.2, 134.2, 133.9, 133.1, 131.8, 129.8, 129.5, 127.0, 121.9, 34.9, 31.4, 19.9. HRMS (ESI-MS) *m/z* calculated for $C_{18}H_{19}ClO$ $[M+Na]^+$ 309.1017, found 309.1007. TLC (SiO_2, hexane:EtOAc 9:1), R_f = 0.66. $[a]_D^{25}$ = -14.6 (c 0.560, $CHCl_3$).

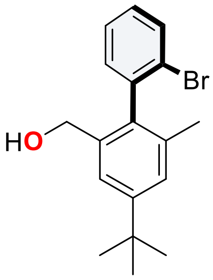

(2'-bromo-4-(*tert*-butyl)-6-methyl-[1,1'-biphenyl]-2-yl)methanol (21a). It was isolated according to the General Procedure on a 0.170 mmol scale using **C2** as catalyst and AcOH (1.3 equiv.) as carboxylic acid in HFIP at room temperature. It was purified by column chromatography over silica gel (hexane:EtOAc, 90:10) to obtain the product as a colorless oil (35.2 mg, 0.106 mmol, 62% yield, 87% ee). ^{1}H-NMR (400 MHz, $CDCl_3$) δ, ppm: 7.68 (dd, *J* = 8.0, 1.3 Hz, 1H), 7.41 – 7.35 (m, 2H), 7.26 – 7.21 (m, 2H), 7.18 (dd, *J* = 7.5, 1.7 Hz, 1H), 4.35 (d, *J* = 12.7 Hz, 1H), 4.29 (d, *J* = 12.7 Hz, 1H), 2.00 (s, 3H), 1.58 (br, 1H), 1.38 (s, 9H). ^{13}C-NMR (100 MHz, $CDCl_3$) δ, ppm: 151.2, 140.7, 137.9, 136.8, 135.8, 132.9, 131.2, 129.1, 127.7, 126.4, 124.4, 122.5, 64.0, 34.7, 31.5, 20.4. HRMS (ESI-MS) *m/z* calculated for $C_{18}H_{21}BrO$ $[M+H]^+$ 355.0668, found 355.0665. TLC (SiO_2, hexane:EtOAc 9:1), R_f = 0.14. $[a]_D^{25}$ = -6.96 (c 0.927, $CHCl_3$).

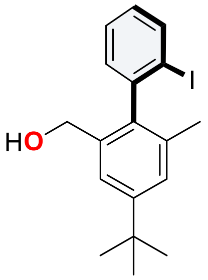

(4-(*tert*-butyl)-2'-iodo-6-methyl-[1,1'-biphenyl]-2-yl)methanol (22a). It was isolated according to the General Procedure on a 0.113 mmol scale using **C6** as catalyst and DMBA (1.3 equiv.) as carboxylic acid in HFIP at room temperature. It was purified by column chromatography over silica gel (hexane:EtOAc, 90:10) to obtain the product as a colorless oil (25.2 mg, 0.0663 mmol, 59% yield, 86% ee). ^{1}H-NMR (400 MHz, $CDCl_3$) δ, ppm: 7.95 (dd, *J* = 8.0, 1.2 Hz, 1H), 7.44-7.38 (m, 2H), 7.23 (d, *J* = 2.1 Hz, 1H), 7.18 (dd, *J* = 7.6, 1.7 Hz, 1H), 7.06 (td, *J* = 7.6, 1.7 Hz, 1H), 4.33 (d, *J* = 12.7 Hz, 1H), 4.27 (d, *J* = 12.7 Hz, 1H), 1.97 (s, 3H), 1.38 (s, 9H). ^{13}C-NMR (100 MHz, $CDCl_3$) δ, ppm: 151.3, 144.9, 140.0, 139.3, 137.6, 135.6, 130.2, 129.0, 128.6, 126.5, 122.5, 101.1, 64.0, 34.8, 31.6, 29.9, 20.5. HRMS (ESI-MS) *m/z* calculated for $C_{18}H_{21}IO$ $[M+Na]^+$ 403.0529, found 403.0524. TLC (SiO_2, hexane:EtOAc 9:1), R_f = 0.22. $[a]_D^{25}$ = -20.6 (c 0.280, $CHCl_3$).

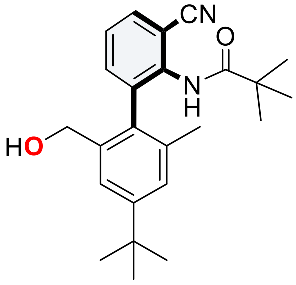

***N*-(4'-(*tert*-butyl)-3-cyano-2'-(hydroxymethyl)-6'-methyl-[1,1'-biphenyl]-2-yl)pivalamide (23a).** It was isolated according to the General Procedure on an 0.099 mmol scale using **C6** as catalyst and DMBA (5 equiv.) as carboxylic acid at 0ºC. It was purified by column chromatography over silica gel (hexane:EtOAc, 5:1 to 3:1) to obtain the product as a colorless solid (23.0 mg, 0.061 mmol, 62% yield, >99% ee). ^{1}H-NMR (400 MHz, $CDCl_3$) δ, ppm: 7.80 (s, 1H), 7.72 (dd, *J* = 5.8, 3.5 Hz, 1H), 7.46-7.38 (m, 2H), 7.33 (d, *J* = 2.0 Hz, 1H), 4.39 (dd, *J* = 10.7, 3.1 Hz, 1H), 4.28 (dd, *J* = 10.7, 3.1 Hz, 1H), 2.32 (br, 1H), 1.91 (s, 3H), 1.34 (s,

9H), 0.93 (s, 9H). ^{13}C-NMR (100 MHz, $CDCl_3$) δ, ppm: 177.4, 152.0, 139.3, 138.9, 137.5, 136.5, 134.4, 133.8, 132.5, 127.4, 127.2, 124.6, 116.9, 113.6, 64.4, 39.1, 34.7, 31.4, 27.0, 20.6. HRMS (ESI-MS) *m/z* calculated for $C_{24}H_{30}N_2O_2$ $[M+Na]^+$ 401.2199, found 401.2205. TLC (SiO_2, hexane:EtOAc 8:2), R_f = 0.18. $[a]_D^{25}$ = +4.46 (c 0.867, $CHCl_3$).

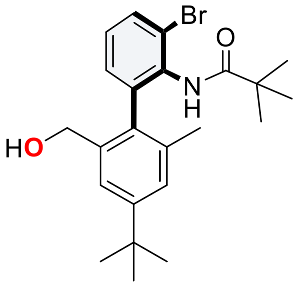

***N*-(3-bromo-4'-(*tert*-butyl)-2'-(hydroxymethyl)-6'-methyl-[1,1'-biphenyl]-2-yl)pivalamide (24a).** It was isolated according to the General Procedure on an 0.101 mmol scale using **C6** as catalyst and DMBA (5 equiv.) as carboxylic acid at 0ºC. It was purified by column chromatography over silica gel (hexane:EtOAc, 10:1 to 5:1) to obtain the product as a colorless solid (35.2 mg, 0.0814 mmol, 81% yield, >99% ee). ^{1}H-NMR (400 MHz, $CDCl_3$) δ, ppm: 7.65 (dd, *J* = 8.0, 1.5 Hz, 1H), 7.36 (d, *J* = 2.1 Hz, 1H), 7.34 (br, 1H), 7.25-7.19 (m, 2H), 7.15 (dd, *J* = 7.6, 1.5 Hz, 1H), 4.38 (dd, *J* = 11.0, 2.0 Hz, 1H), 4.32 (dd, *J* = 11.4, 5.6 Hz, 1H), 2.88 (br, 1H), 1.97 (s, 3H), 1.32 (s, 9H), 0.91 (s, 9H). ^{13}C-NMR (100 MHz, $CDCl_3$) δ, ppm: 177.1, 151.3, 140.7, 138.5, 135.7, 135.1, 134.9, 132.3, 129.6, 128.6, 126.5, 124.6, 124.1. HRMS (ESI-MS) *m/z* calculated for $C_{23}H_{30}BrNO_2$ $[M+Na]^+$ 454.1352, found 454.1358. TLC (SiO_2, hexane:EtOAc 8:2), R_f = 0.41. $[a]_D^{25}$ = -5.14 (c 0.892, $CHCl_3$).

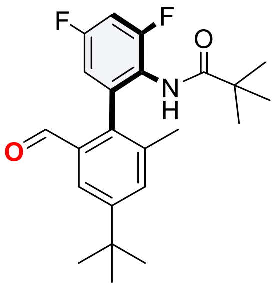

***N*-(4'-(*tert*-butyl)-3,5-difluoro-2'-formyl-6'-methyl-[1,1'-biphenyl]-2-yl)pivalamide (25b).** It was isolated according to the General Procedure on an 0.099 mmol scale using **C6** as catalyst and DMBA (5 equiv.) as carboxylic acid in at 0ºC. The crude mixture was oxidized with PCC and purified by column chromatography over silica gel (hexane:EtOAc, 20:1 to 10:1) to obtain the product as a colorless solid (33.2 mg, 0.086 mmol, 87% yield, 91% ee). ^{1}H-NMR (400 MHz, $CDCl_3$) δ, ppm: 9.73 (s, 1H), 7.82 (d, *J* = 2.1 Hz, 1H), 7.53 (d, *J* = 2.1 Hz, 1H), 7.01-6.92 (m, 1H), 6.82 (ddd, *J* = 8.3, 2.8, 1.5 Hz, 1H), 6.49 (br, 1H), 2.14 (s, 3H), 1.35 (s, 9H), 0.90 (s, 9H). ^{13}C-NMR (100 MHz, $CDCl_3$) δ, ppm: 192.5, 176.9, 162.4 (d, *J* = 14.9 Hz), 159.8 (d, *J* = 22.0 Hz), 157.2 (d, *J* = 13.3 Hz), 138.9 (d, *J* = 8.7 Hz), 137.5, 136.3, 133.1, 122.8, 113.2 (d, *J* = 19.2 Hz), 104.4 (t, *J* = 25.5 Hz), 39.1, 34.9, 31.3, 27.2, 20.0. ^{19}F-NMR (377 MHz, $CDCl_3$) δ, ppm: -111.7 (q, *J* = 8.1 Hz), -116.3 (t, *J* = 8.5 Hz). HRMS (ESI-MS) *m/z* calculated for $C_{23}H_{27}F_2NO_2$ $[M+H]^+$ 388.2083, found 388.2087. TLC (SiO_2, hexane:EtOAc 8:2), R_f = 0.53. $[a]_D^{25}$ = -29.2 (c 0.630, $CHCl_3$).

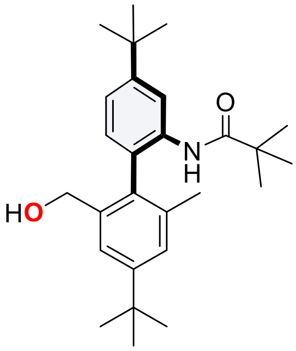

***N*-(4,4'-di-*tert*-butyl-2'-(hydroxymethyl)-6'-methyl-[1,1'-biphenyl]-2-yl)pivalamide (26a).** It was isolated according to the General Procedure on an 0.099 mmol scale using **C6** as catalyst and DMBA (5 equiv.) as carboxylic acid in at 0ºC. It was purified by column chromatography over silica gel (hexane:EtOAc, 10:1 to 5:1) to obtain the product as a colorless solid (24.0 mg, 58.6 mmol, 59% yield, 98% ee). ^{1}H-NMR (400 MHz, $CDCl_3$) δ, ppm: 8.07 (d, *J* = 8.6 Hz, 1H), 7.43 (d, *J* = 1.6 Hz, 1H), 7.40 (dd, *J* = 8.6, 2.3 Hz, 1H), 7.30 (d, *J* = 1.6 Hz, 1H), 7.16 (d, *J* = 2.3 Hz, 1H), 7.10 (br, 1H), 4.35 (s, 2H), 1.98 (s, 3H), 1.36 (s, 9H), 1.31 (s, 9H), 0.93 (s, 9H). ^{13}C-NMR (100 MHz, $CDCl_3$) δ, ppm: 176.7, 151.8, 147.6, 139.0, 137.0, 133.8, 133.3, 130.2, 126.9, 126.2, 125.4, 123.5, 121.7, 64.0, 39.5, 34.8, 34.6, 31.6, 31.5, 27.2, 20.4. HRMS (ESI-MS) *m/z*

calculated for $C_{27}H_{39}NO_2$ $[M+Na]^+$ 432.2873, found 432.2879. TLC (SiO_2, hexane:EtOAc 8:2), R_f = 0.43. $[a]_D^{25}$ = -5.71 (c 0.993, $CHCl_3$).

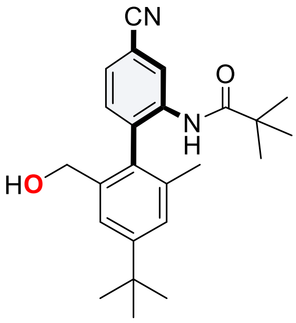

***N*-(4'-(*tert*-butyl)-4-cyano-2'-(hydroxymethyl)-6'-methyl-[1,1'-biphenyl]-2-yl)pivalamide (27a).** It was isolated according to the General Procedure on a 0.055 mmol scale using **C6** as catalyst and DMBA (5 equiv.) as carboxylic acid in HFIP at 0ºC. It was purified by column chromatography over silica gel (hexane:EtOAc, 5:1 to 3:1) to obtain the product as a colorless solid (13.1 mg, 0.035 mmol, 63% yield, >99% ee). ^{1}H-NMR (400 MHz, $CDCl_3$) δ, ppm: 8.66 (d, *J* = 1.6 Hz, 1H), 7.47 (dd, *J* = 7.8, 1.6 Hz, 1H), 7.45 (d, *J* = 2.0 Hz, 1H), 7.33 (d, *J* = 2.0 Hz, 1H), 7.27-7.23 (m, 1H), 4.35 (d, *J* = 11.9 Hz, 1H), 4.30 (d, *J* = 11.9 Hz, 1H), 1.94 (s, 3H), 1.36 (s, 9H), 0.94 (s, 9H). ^{13}C-NMR (100 MHz, $CDCl_3$) δ, ppm: 176.8, 153.0, 138.3, 137.2, 136.6, 135.1, 131.5, 130.2, 127.8, 127.4, 124.7, 124.0, 118.7, 112.6, 63.7, 39.8, 34.9, 31.4, 27.1, 20.2. HRMS (ESI-MS) *m/z* calculated for $C_{24}H_{30}N_2O_2$ $[M+H]^+$ 361.2274, found 361.2271. TLC (SiO_2, hexane:EtOAc 8:2), R_f = 0.14. $[a]_D^{25}$ = +13.8 (c 0.290, $CHCl_3$).

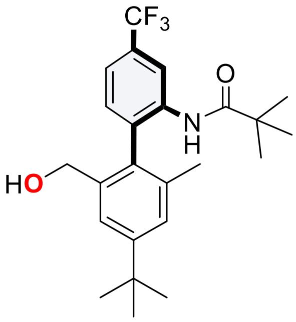

***N*-(4'-(*tert*-butyl)-4-chloro-2'-(hydroxymethyl)-6'-methyl-[1,1'-biphenyl]-2-yl)pivalamide (28a).** It was isolated according to the General Procedure on a 0.087 mmol scale using **C6** as catalyst and DMBA (1.3 equiv.) as carboxylic acid in HFIP at 0ºC. It was purified by column chromatography over silica gel (hexane, 90:10) to obtain the product as a colorless solid (32.3 mg, 0.077 mmol, 88% yield, >99% ee). ^{1}H-NMR (400 MHz, $CDCl_3$) δ, ppm: 8.67 (d, *J* = 1.8 Hz, 1H), 7.51-7.43 (m, 2H), 7.37-7.33 (m, 1H), 7.30-7.29 (m, 1H), 4.38 (d, *J* = 12.0 Hz, 1H), 4.34 (d, *J* = 12.0 Hz, 1H), 1.99 (s, 3H), 1.39 (s, 9H), 0.97 (s, 9H). ^{13}C-NMR (100 MHz, $CDCl_3$) δ, ppm: 176.7, 152.5, 138.4, 136.7, 136.6, 135.5, 131.8, 130.8 (q, *J* = 32.5 Hz), 129.6, 127.1, 125.3 (q, *J* = 272 Hz), 123.7, 120.8 (q, *J* = 3.8 Hz), 118.2 (*J* = 4.2 Hz), 63.6, 39.6, 34.7, 31.3, 27.0, 20.1. ^{19}F-NMR (377 MHz, $CDCl_3$) δ, ppm: -63.5.z HRMS (ESI-MS) *m/z* calculated for $C_{24}H_{30}F_3NO_2$ $[M+Na]^+$ 444.2121, found 444.2119. TLC (SiO_2, hexane:EtOAc 9:1), R_f = 0.30. $[a]_D^{25}$ = -13.8 (c 0.390, $CHCl_3$). XRD structure found in CCDC Number 2519278.

X-ray structure of *R*-28a

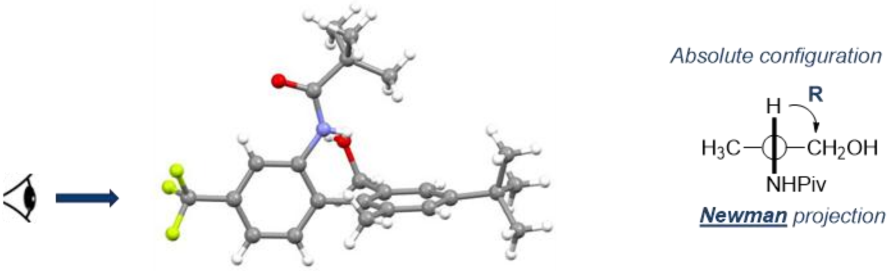

Crystals of the purified sample were grown via slow evaporation from chloroform, which were analyzed by x-ray diffraction. The quality of the crystals obtained allowed to determine the absolute configuration which is *R*. The structure was deposited in the Cambridge Crystallographic Data Centre (deposition no. CCDC 2519278). The absolute stereochemistry

of the other atropisomeric products in the scope have been tentatively assigned in analogy to this compound (in all the cases the crystals have been obtained via slow evaporation from chloroform).

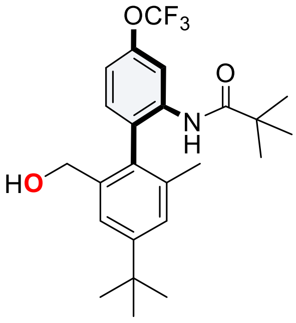

***N*-(4'-(*tert*-butyl)-2'-(hydroxymethyl)-6'-methyl-4-(trifluoromethoxy)-[1,1'-biphenyl]-2-yl)pivalamide (29a).** It was isolated according to the General Procedure on a 0.085 mmol scale using **C6** as catalyst and DMBA (1.3 equiv.) as carboxylic acid in HFIP at 0ºC. It was purified by column chromatography over silica gel (hexane, 90:10) to obtain the product as a colorless solid (32.0 mg, 0.073 mmol, 86% yield, >99% ee). ^{1}H-NMR (400 MHz, $CDCl_3$) δ, ppm: 8.30 (d, *J* = 1.3 Hz, 1H), 7.45 (d, *J* = 2.2 Hz, 1H), 7.32 (d, *J* = 1.7 Hz, 1H), 7.19 (br, 1H), 7.15 (d, *J* = 8.3 Hz, 1H), 7.06-7.00 (m, 1H), 4.34 (s, 1H), 4.33 (s, 1H), 1.97 (s, 3H), 1.36 (s, 9H), 0.94 (s, 9H). ^{13}C-NMR (100 MHz, $CDCl_3$) δ, ppm: 176.6, 152.4, 149.1, 138.9, 137.2, 137.1, 131.6, 130.0, 127.9, 127.1, 123.5, 120.5 (q, *J* = 257.5 Hz), 116.0, 113.7, 63.6, 39.6, 34.7, 31.3, 27.0, 20.2. ^{19}F-NMR (377 MHz, $CDCl_3$) δ, ppm: -58.6. HRMS (ESI-MS) *m/z* calculated for $C_{24}H_{30}F_3NO_3$ $[M+H]^+$ 438.2251, found 438.2243. TLC (SiO_2, hexane:EtOAc 9:1), R_f = 0.34. $[a]_D^{25}$ = +7.40 (c 0.750, $CHCl_3$).

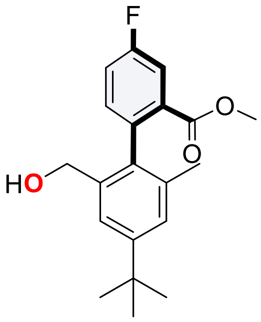

Methyl 4'-(*tert*-butyl)-4-fluoro-2'-(hydroxymethyl)-6'-methyl-[1,1'-biphenyl]-2-carboxylate (30a). It was isolated according to the General Procedure on a 0.140 mmol scale using **C6** as catalyst and AcOH (5 equiv.) as carboxylic acid in HFIP at 0ºC. It was purified by column chromatography over silica gel (hexane:EtOAc, 9:1) to obtain the product as a colorless solid (31.6 mg, 0.096 mmol, 68% yield, >99% ee). ^{1}H-NMR (400 MHz, $CDCl_3$) δ, ppm: 7.65 (dd, *J* = 9.2, 2.8 Hz, 1H), 7.35 (d, *J* = 2.0 Hz, 1H), 7.27 (td, *J* = 8.2, 2.8 Hz, 1H), 7.19 (d, *J* = 2.0 Hz, 1H), 7.15 (dd, *J* = 8.5, 5.5 Hz, 1H), 4.31 (d, *J* = 11.7 Hz, 1H), 4.26 (d, *J* = 11.7 Hz, 1H), 3.67 (s, 3H), 2.23 (br, 1H), 1.90 (s, 3H), 1.36 (s, 9H). ^{13}C-NMR (100 MHz, $CDCl_3$) δ, ppm: 167.3 (d, *J* = 2.6 Hz), 161.6 (d, *J* = 247.6 Hz), 150.8, 137.9, 136.9 (d, *J* = 3.7 Hz), 136.2, 135.3, 132.9 (d, *J* = 7.4 Hz), 132.6 (d, *J* = 7.1 Hz), 126.4, 123.4, 119.3 (d, *J* = 20.9 Hz), 117.1 (d, *J* = 23.3 Hz), 64.4, 52.6, 34.6, 31.5, 20.7. ^{19}F-NMR (377 MHz, $CDCl_3$) δ, ppm: -115.2 (td, *J* = 8.5, 5.4 Hz). HRMS (ESI-MS) *m/z* calculated for $C_{20}H_{23}FO_3$ $[M+Na]^+$ 353.1523, found 353.1526. TLC (SiO_2, hexane:EtOAc 9:1), R_f = 0.05.

***Note**: to determine the ee, this product was derivatized to the corresponding benzyl imine (**30c**) by a first oxidation to the aldehyde with PCC[43] and subsequent reaction with benzylamine in CH_3CN.[45]*

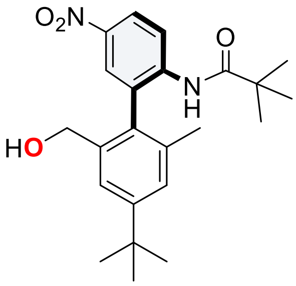

***N*-(4'-(*tert*-butyl)-2'-(hydroxymethyl)-6'-methyl-5-nitro-[1,1'-biphenyl]-2-yl)pivalamide (31a).** It was isolated according to the General Procedure on a 0.081 mmol scale using **C6** as catalyst and AcOH (5 equiv.) as carboxylic acid in HFIP at 0ºC. It was purified by column chromatography over silica gel (hexane:EtOAc, 10:1 to 5:1) to obtain the product as a colorless solid (19.4 mg, 0.049 mmol, 60% yield, 91% ee). ^{1}H-NMR (400 MHz, $CDCl_3$) δ, ppm: 8.58 (d, *J* = 9.1 Hz, 1H), 8.28 (dd, *J* = 9.1, 2.7 Hz, 1H), 8.06 (d, *J* = 2.7 Hz, 1H), 7.48 (d, *J* = 2.1 Hz, 1H), 7.40 (s, 1H), 7.36 (d, *J* = 2.1 Hz, 1H), 4.38 (dd, *J* = 12.1, 3.5 Hz, 1H), 4.33 (dd, *J* = 12.1, 4.3 Hz, 1H), 1.98 (s, 3H), 1.59 (br, 1H), 1.37 (s, 9H), 0.96 (s, 9H). ^{13}C-NMR (100

MHz, $CDCl_3$) δ, ppm: 176.9, 153.3, 143.4, 142.3, 138.8, 137.0, 310.6, 130.1, 127.5, 124.9, 124.7, 124.0, 120.1, 63.7, 40.1, 34.9, 31.4, 27.0, 20.2. HRMS (ESI-MS) *m/z* calculated for $C_{23}H_{30}N_2O_4$ $[M+Na]^+$ 421.2098, found 421.2092. TLC (SiO_2, hexane:EtOAc 8:2), R_f = 0.17. $[a]_D^{25}$ = -6.90 (c 0.575, $CHCl_3$). XRD structure found in CCDC Number 2519281.

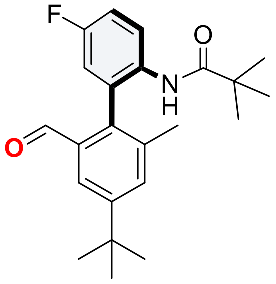

***N*-(4'-(*tert*-butyl)-5-fluoro-2'-formyl-6'-methyl-[1,1'-biphenyl]-2-yl)pivalamide (32b).** It was isolated according to the General Procedure on an 0.049 mmol scale using **C6** as catalyst and DMBA (5 equiv.) as carboxylic acid in HFIP at 0ºC. The crude mixture was oxidized with PCC and purified by column chromatography over silica gel (hexane:EtOAc, 10:1) to obtain the product as a colorless solid (12.1 mg, 0.033 mmol, 67% yield, 97% ee). ^{1}H-NMR (400 MHz, $CDCl_3$) δ, ppm: 9.68 (s, 1H), 8.29 (dd, *J* = 8.9, 5.3 Hz, 1H), 7.95 (d, *J* = 2.1 Hz, 1H), 7.63 (dd, *J* = 2.2, 0.7 Hz, 1H), 7.15 (ddd, *J* = 8.9, 8.4, 3.0 Hz, 1H), 6.93 (dd, *J* = 8.4, 3.0 Hz, 1H), 6.77 (br, 1H), 2.10 (s, 3H), 1.38 (s, 9H), 0.93 (s, 9H). ^{13}C-NMR (100 MHz, $CDCl_3$) δ, ppm: 191.9, 176.3, 159.1 (d, *J* = 245.4 Hz), 152.9, 138.0, 136.3, 134.4, 133.6, 132.5, 129.0, 123.2 (d, *J* = 8.1 Hz), 122.7, 116.9, 116.0 (d, *J* = 21.9 Hz), 39.7, 35.0, 31.3, 27.2, 19.7. ^{19}F-NMR (377 MHz, $CDCl_3$) δ, ppm: -118.7 (q, *J* = 7.5 Hz). HRMS (ESI-MS) *m/z* calculated for $C_{23}H_{28}FNO_2$ $[M+H]^+$ 370.2177, found 370.2175. TLC (SiO_2, hexane:EtOAc 8:2), R_f = 0.50. $[a]_D^{25}$ = -15.0 (c 0.420, $CHCl_3$).

***Note**: to determine the ee, this product was derivatized to the corresponding benzyl imine (**33c**) by a first oxidation to the aldehyde with PCC[43] and subsequent reaction with benzylamine in CH_3CN.[45]*

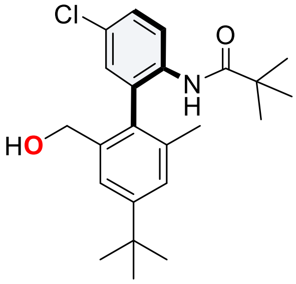

***N*-(4'-(*tert*-butyl)-5-chloro-2'-(hydroxymethyl)-6'-methyl-[1,1'-biphenyl]-2-yl)pivalamide (33a).** It was isolated according to the General Procedure on a 0.085 mmol scale using **C6** as catalyst and AcOH (5 equiv.) as carboxylic acid in HFIP at 0ºC. It was purified by column chromatography over silica gel (hexane, 90:10 to 75:25) to obtain the product as a colorless solid (23.4 mg, 0.060 mmol, 71% yield, >99% ee). ^{1}H-NMR (400 MHz, $CDCl_3$) δ, ppm: 8.21 (d, *J* = 8.8 Hz, 1H), 7.46 (d, *J* = 2.1 Hz, 1H), 7.38 (dd, *J* = 8.8, 2.5 Hz, 1H), 7.33 (d, *J* = 2.1 Hz, 1H), 7.17 (br, 1H), 7.16 (d, *J* = 2.5 Hz, 1H), 4.40 (d, *J* = 12.2 Hz, 1H), 4.37 (d, *J* = 12.2 Hz, 1H), 2.00 (s, 3H), 1.38 (s, 9H), 0.95 (s, 9H). ^{13}C-NMR (100 MHz, $CDCl_3$) δ, ppm: 176.5, 152.3, 138.5, 136.8, 134.7, 132.0, 131.8, 129.3, 128.9, 128.5, 127.0, 123.6, 122.9, 63.7, 39.5, 34.7, 31.3, 27.0, 20.2. HRMS (ESI-MS) *m/z* calculated for $C_{23}H_{30}ClNO_2$ $[M+Na]^+$ 410.1857, found 410.1850. TLC (SiO_2, hexane:EtOAc 3:1), R_f = 0.60. $[a]_D^{25}$ = -5.05 (c 0.805, $CHCl_3$).

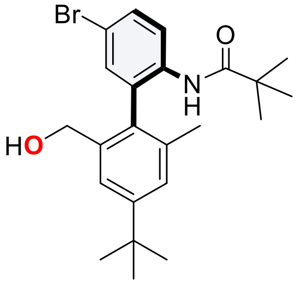

***N*-(5-bromo-4'-(*tert*-butyl)-2'-(hydroxymethyl)-6'-methyl-[1,1'-biphenyl]-2-yl)pivalamide (34a).** It was isolated according to the General Procedure on a 0.026 mmol scale using **C6** as catalyst and DMBA (5 equiv.) as carboxylic acid in HFIP at 0ºC. The crude was reduced with $NaBH_4$ and purified by column chromatography over silica gel (hexane:EtOAc, 20:1 to 10:1) to obtain the product as a colorless solid (9.1 mg, 0.021 mmol, 81% yield, 98% ee). ^{1}H-NMR (400 MHz, $CDCl_3$) δ, ppm: 8.15 (d, *J* = 8.8 Hz, 1H), 7.50 (dd, *J* = 8.8, 2.3 Hz, 1H), 7.43 (d, *J* = 2.0 Hz, 1H), 7.30 (d, *J* = 2.0 Hz, 1H), 7.28 (d, *J* = 2.3 Hz, 1H), 7.14

(br, 1H), 4.38 (d, J = 11.8 Hz, 1H), 4.34 (d, J = 11.8 Hz, 1H), 1.98 (s, 3H), 1.36 (s, 9H), 0.93 (s, 9H). ^{13}C-NMR (100 MHz, $CDCl_3$) δ, ppm: 176.7, 152.5, 138.7, 136.9, 135.4, 132.4, 131.9, 131.8, 131.6, 127.2, 123.7, 123.2, 117.1, 63.8, 39.7, 34.8, 31.4, 27.1, 20.3. HRMS (ESI-MS) *m/z* calculated for $C_{23}H_{30}BrNO_2$ $[M+H]^+$ 432.1533, found 432.1522. TLC (SiO_2, hexane:EtOAc 8:2), R_f = 0.29. $[a]_D^{25}$ = -11.3 (c 0.430, $CHCl_3$).

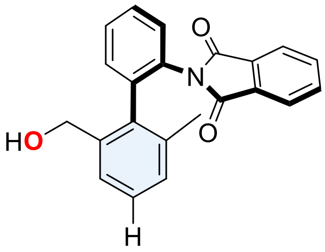

2-(2'-(hydroxymethyl)-6'-methyl-[1,1'-biphenyl]-2-yl)isoindoline-1,3-dione (35a). It was isolated according to the General Procedure on an 0.067 mmol scale using **C6** as catalyst and AcOH (5 equiv.) as carboxylic acid in HFIP at 0ºC. It was purified by column chromatography over silica gel (hexane:EtOAc, 8:2) to obtain the product as a colorless solid (10.9 mg, 0.0.032 mmol, 47% yield, 94% ee). ^{1}H-NMR (400 MHz, $CDCl_3$) δ, ppm: 7.86 – 7.81 (m, 1H), 7.73 – 7.65 (m, 3H), 7.57 – 7.51 (m, 2H), 7.41 – 7.34 (m, 2H), 7.32 – 7.28 (m, 1H), 7.18 (t, J = 7.6 Hz, 1H), 7.04 – 6.99 (m, 1H), 4.55 (d, J = 12.6 Hz, 1H), 4.40 (d, J = 12.6 Hz, 1H), 2.63 (br, 1H), 1.98 (s, 3H). ^{13}C-NMR (100 MHz, $CDCl_3$) δ, ppm: 167.4, 167.3, 140.2, 138.9, 136.4, 135.9, 134.3, 131.8, 131.6, 131.5, 130.5, 129.5, 129.3, 128.8, 128.6, 128.0, 126.3, 123.7, 123.7, 63.1, 53.8, 31.7, 29.7, 29.3, 20.1. HRMS (ESI-MS) *m/z* calculated for $C_{22}H_{17}NO_3$ $[M+Na]^+$ 366.1098, found 366.1101. TLC (SiO_2, hexane:EtOAc 7:3), R_f = 0.16. $[a]_D^{25}$ = -6.43 (c 0.18, $CHCl_3$).

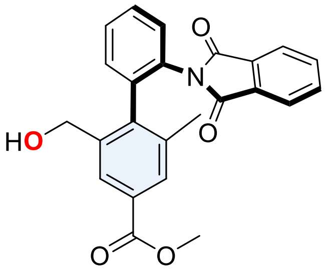

Methyl 2'-(1,3-dioxoisoindolin-2-yl)-2-(hydroxymethyl)-6-methyl-[1,1'-biphenyl]-4-carboxylate (36a). It was isolated according to the General Procedure on a 0.100 mmol scale using **C6** as catalyst and DMBA (5 equiv.) as carboxylic acid in HFIP at 0ºC. It was purified by column chromatography over silica gel (hexane:EtOAc, 3:1 to 2:1) to obtain the product as a colorless solid (21.6 mg, 0.054 mmol, 54% yield, 89% ee). ^{1}H-NMR (400 MHz, $CDCl_3$) δ, ppm: 8.07 (d, J = 1.7 Hz, 1H), 7.83 (d, J = 6.7 Hz, 1H), 7.74-7.68 (m, 4H), 7.60-7.54 (m, 2H), 7.44-7.33 (m, 1H), 7.28-7.26 (m, 1H), 4.59 (d, J = 12.9 Hz, 1H), 4.42 (d, J = 12.9 Hz, 1H), 3.87 (s, 3H), 2.02 (s, 3H). ^{13}C-NMR (100 MHz, $CDCl_3$) δ, ppm: 167.4, 167.3, 167.2, 141.0, 140.8, 138.1, 137.2, 134.6, 131.6, 131.5, 131.3, 130.3, 129.9, 129.8, 129.6, 129.1, 127.5, 124.0, 123.9. HRMS (ESI-MS) *m/z* calculated for $C_{24}H_{19}NO_5$ $[M+H]^+$ 402.1336, found 402.1330. TLC (SiO_2, hexane:EtOAc 7:3), R_f = 0.19. $[a]_D^{25}$ = -25.4 (c 0.230, $CHCl_3$).

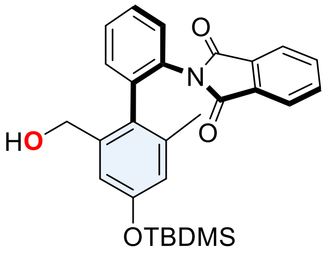

2-(4'-((*tert*-butyldimethylsilyl)oxy)-2'-(hydroxymethyl)-6'-methyl-[1,1'-biphenyl]-2-yl)isoindoline-1,3-dione (37a). It was isolated according to the General Procedure on a 0.035 mmol scale using **C6** as catalyst and DMBA (5 equiv.) as carboxylic acid in HFIP at 0ºC. It was purified by column chromatography over silica gel (hexane:EtOAc, 10:1 to 5:1) to obtain the product as a colorless solid (9.5 mg, 0.02 mmol, 58% yield, 85% ee). ^{1}H-NMR (400 MHz, $CDCl_3$) δ, ppm: 7.88 – 7.83 (m, 1H), 7.73 (dt, J = 4.9, 1.9 Hz, 3H), 7.57 – 7.53 (m, 2H), 7.40 – 7.36 (m, 1H), 7.34 – 7.30 (m, 1H), 6.90 (d, J = 2.5 Hz, 1H), 6.51 (d, J = 2.5 Hz, 1H), 4.50 (d, J = 12.7 Hz, 1H), 4.34 (d, J = 12.8 Hz, 1H), 1.93 (s, 3H), 0.93 (s, 9H), 0.12 (s, 6H). ^{13}C-NMR (100 MHz, $CDCl_3$) δ, ppm: 167.4, 167.3, 155.1, 141.7, 139.0, 137.8, 134.3,

132.1, 131.6, 131.5, 130.9, 129.5, 129.3, 129.0, 128.4, 123.7, 123.6, 120.4, 117.5, 63.0, 25.7, 20.1, 18.2, -4.4. HRMS (ESI-MS) *m/z* calculated for $C_{28}H_{31}NO_4Si$ $[M+H]^+$ 474.2095, found 474.2090. TLC (SiO_2, hexane:EtOAc 8:2), R_f = 0.34. $[a]_D^{25}$ = -25.9 (c 0.250, $CHCl_3$).

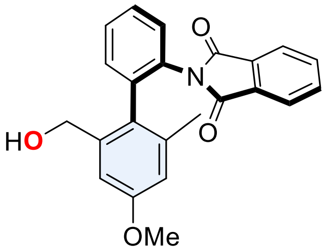

2-(2'-(hydroxymethyl)-4'-methoxy-6'-methyl-[1,1'-biphenyl]-2-yl)isoindoline-1,3-dione (38a). It was isolated according to the General Procedure on a 0.100 mmol scale using **C6** as catalyst and DMBA (5 equiv.) as carboxylic acid in HFIP at 0ºC. It was purified by column chromatography over silica gel (hexane:EtOAc, 10:1 to 3:1) to obtain the product as a colorless liquid (19.8 mg, 0.053 mmol, 53% yield, 94% ee). ^{1}H-NMR (400 MHz, $CDCl_3$) δ, ppm: 7.90 – 7.83 (m, 1H), 7.80 – 7.67 (m, 3H), 7.55 (dd, *J* = 5.8, 3.4 Hz, 2H), 7.38 (dt, *J* = 5.4, 3.7 Hz, 1H), 7.30 (d, *J* = 3.4 Hz, 1H), 6.97 (d, *J* = 2.7 Hz, 1H), 6.59 (d, *J* = 2.7 Hz, 1H), 4.55 (d, *J* = 12.7 Hz, 1H), 4.38 (d, *J* = 12.9 Hz, 1H), 3.78 (s, 3H), 1.97 (s, 3H). ^{13}C-NMR (100 MHz, $CDCl_3$) δ, ppm: 167.4, 167.3, 159.0, 141.8, 138.9, 138.0, 134.3, 132.3, 131.6, 131.5, 131.0, 129.5, 129.3, 128.5, 128.4, 123.8, 123.7, 114.8, 110.7, 63.2, 55.1, 20.3. HRMS (ESI-MS) *m/z* calculated for $C_{23}H_{19}NO_4$ $[M+Na]^+$ 396.1206, found 396.1205. TLC (SiO_2, hexane:EtOAc 1.1), R_f = 0.42. $[a]_D^{25}$ = -22.2 (c 0.370, $CHCl_3$).

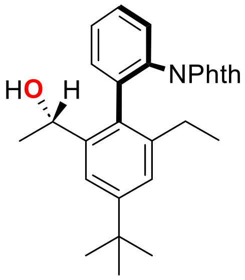

2-(4'-(*tert*-butyl)-2'-ethyl-6'-(1-hydroxyethyl)-[1,1'-biphenyl]-2-yl)isoindoline-1,3-dione (39a). It was isolated according to the General Procedure on a 0.0398 mmol scale using **C6** as catalyst and DMBA (5 equiv.) as carboxylic acid in HFIP at 01C. It was purified by column chromatography over silica gel (hexane:EtOAc, 10:1 to 5:1) to obtain the product as a colorless solid (13.8 mg, 0.0323 mmol, 81% yield, d.r = 93:1, 83% ee for the major diastereoisomer, >99% for the minor). ^{1}H-NMR (400 MHz, $CDCl_3$) δ, ppm: 7.86-7.79 (m, 1H), 7.77-7.65 (m, 3H), 7.58-7.52 (m, 2H), 7.51-7.45 (m, 1H), 7.40-7.33 (m, 1H), 7.31-7.25 (m, 1H), 7.11 (d, *J* = 2.0 Hz, 1H), 4.80 (q, *J* = 6.3 Hz, 1H), 2.34 (qd, *J* = 7.5, 2.2 Hz, 2H), 1.50 (d, *J* = 6.3 Hz, 3H), 1.26 (s, 9H), 1.01 (t, *J* = 7.5 Hz, 3H). ^{13}C-NMR (100 MHz, $CDCl_3$) δ, ppm: 167.1, 167.0, 150.8, 142.7, 142.6, 139.4, 134.1, 132.7, 132.3, 131.8, 131.7, 131.0, 129.3, 129.0, 128.3, 124.4, 123.5, 123.4, 119.6, 67.6, 34.6, 31.3, 26.5, 23.1, 15.8. HRMS (ESI-MS) *m/z* calculated for $C_{28}H_{29}NO_3$ $[M+Na]^+$ 450.2040, found 450.2039. TLC (SiO_2, hexane:EtOAc 7:3), R_f = 0.46. $[a]_D^{25}$ = +9.20 (c 0.460, $CHCl_3$). XRD structure found in CCDC Number 2519371.

***Note**: the ee of the corresponding ketone (**39b**), obtained after oxidation with PCC,[43] was also measured (81% ee).*

6.2. Product elaboration

The following products have been prepared and characterized from (*R*)-**18a** and (*R*)-**18b**.

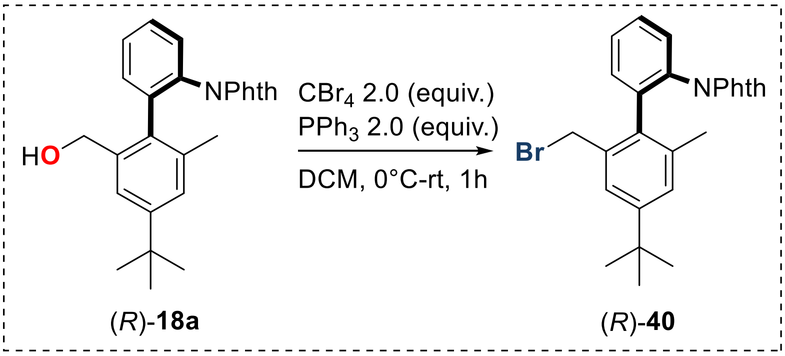

(*R*)-2-(2'-(bromomethyl)-4'-(tert-butyl)-6'-methyl-[1,1'-biphenyl]-2-yl)isoindoline-1,3-dione ((*R*)-40). It was prepared according to a modified reported procedure.[46] A sealed vial equipped with a septum and kept under nitrogen was charged with alcohol (*R*)-**18a** (50 mg, 0.125 mmol, 1 equiv.), carbon tetrabromide (83 mg, 0.250 mmol, 2.0 equiv.), and 1 mL of dry dichloromethane. The solution was then cooled to 0°C. At this point, triphenylphosphine (66 mg, 0.250 mmol, 2.0 equiv.) was added as a solid under nitrogen flow into the mixture, and the reaction was left stirring for 2 hours, allowing it to warm up to room temperature. The reaction mixture was then evaporated to dryness, and the crude product was purified by flash chromatography over silica using hexane:EtOAc (6:1) to obtain the product as a colorless solid (51 mg, 88% yield, 95% ee). ^{1}H-NMR (400 MHz, $CDCl_3$) δ, ppm: 7.86 – 7.79 (m, 1H), 7.77 – 7.53 (m, 6H), 7.39 (dt, *J* = 7.0, 1.5 Hz, 1H), 7.27 (d, *J* = 2.0 Hz, 1H), 7.07 (dd, *J* = 1.9, 0.8 Hz, 1H), 4.48 – 4.36 (m, 2H), 2.02 (s, 3H), 1.24 (s, 9H). ^{13}C-NMR (100 MHz, $CDCl_3$) δ, ppm: 167.2, 167.1, 150.9, 138.6, 136.8, 136.4, 134.1, 134.1, 131.7, 131.6, 131.6, 130.9, 129.4, 129.3, 128.7, 127.1, 125.0, 123.6, 123.4, 34.4, 34.2, 31.1, 20.5. HRMS (ESI-MS) *m/z* calculated for $C_{26}H_{24}BrNO_2$ $[M+H]^+$ 461.1063, found 461.1062. TLC (SiO_2, hexane:EtOAc 5:1), R_f = 0.39. $[a]_D^{25}$ = -17.9 (c 0.600, $CHCl_3$).

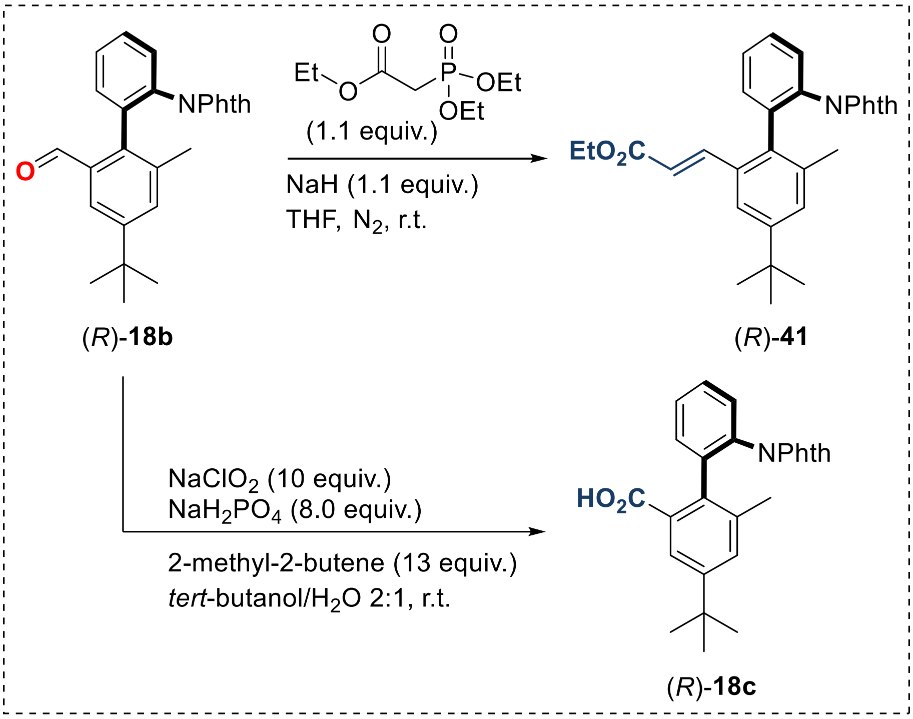

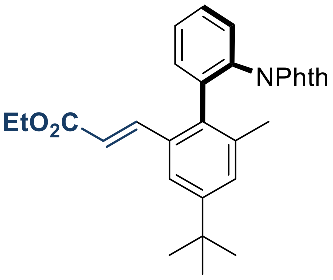

Ethyl (*R*)-3-(4-(tert-butyl)-2'-(1,3-dioxoisoindolin-2-yl)-6-methyl-[1,1'-biphenyl]-2-yl)acrylate ((*R*)-41). It was prepared according to a modified reported procedure.[21] To a suspension of NaH (0.73 mg, 0.03 mmol, 1.1 equiv.) in THF (25 μL), a solution of triethyl phosphonoacetate (6.7 mg, 0.03 mmol, 1,1 equiv.) in THF (15 μL) was slowly added. The mixture was stirred at room temperature for 30 min. Then, (*R*)-**18b** (0.028 mmol, 1.0 equiv.) in THF (25 μL) was added at 0 °C, and the mixture was stirred at room temperature. After confirmation of consumption of the aldehyde by GC, a solution of saturated aqueous sodium bicarbonate (1.0 mL) was added. The mixture was extracted with EtOAc (3 x 1.0 mL), washed with brine (3.0 mL) and dried over Mg_2SO_4. The solvent was removed under reduced pressure and the crude was purified by silica-gel column chromatography using hexane/EtOAc (8:2) to give the product as a colorless liquid (13.1 mg, 99% yield, 95% ee). ^{1}H-NMR (400 MHz, $CDCl_3$) δ, ppm: 7.79 – 7.72 (m, 2H), 7.69 (dd, *J* = 5.5, 3.1 Hz, 2H), 7.59 – 7.48 (m, 3H), 7.45 – 7.39 (m, 2H), 7.35 – 7.30 (m, 1H), 7.22 (d, *J* = 2.0 Hz, 1H), 6.15 (d, *J* = 15.9 Hz, 1H), 4.23 (qt, *J* = 7.1, 3.7 Hz, 2H), 2.13 (s, 3H), 1.27 (m, *J* = 4.8 Hz, 12H). ^{13}C-NMR (100 MHz, $CDCl_3$) δ, ppm: 166.9, 166.72 166.4, 150.4, 144.5, 137.9, 137.1, 135.6, 134.1, 134.0, 133.4, 132.1, 131.8, 131.7, 131.1, 129.3, 129.0, 128.9, 128.6, 123.5, 123.3, 120.5, 118.8, 60.2, 34.5, 31.2, 20.6, 14.3. HRMS (ESI-MS) *m/z* calculated for $C_{30}H_{29}NO_4$ $[M+H]^+$ 468.2169, found 468.2166. TLC (SiO_2, hexane:EtOAc 8:2), R_f = 0.9. $[a]_D^{25}$ = -152.03 (c 0.930, $CHCl_3$).

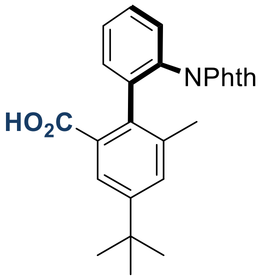

(*R*)-4-(tert-butyl)-2'-(1,3-dioxoisoindolin-2-yl)-6-methyl-[1,1'-biphenyl]-2-carboxylic acid ((*R*)-18c). It was prepared according to a modified reported procedure.[47, 48] To a stirred solution of compound (*R*)-**18b** (40 mg, 0.1 mmol, 1.0 equiv.) and 2-methylbut-2-ene (140 µL, 1.3 mmol, 13.0 equiv.) in *t*BuOH (1.6 mL) were added 0.8 mL water solution of $NaClO_2$ (113 mg, 1.0 mmol) and NaH_2PO_4 (96 mg, 0.95 mmol). The solution was stirred at room temperature for 4 hours. The mixture was quenched with saturated NH_4Cl (3 mL) and extracted with EtOAc (2 x 5 mL). The combined organic layers were washed with brine, dried over Mg_2SO_4, filtered and concentrated the product as a pale-yellow solid (40.1 mg, 97%, 96% ee).
^{1}H-NMR (400 MHz, $CDCl_3$) δ, ppm: 7.94 (dt, *J* = 7.2, 1.0 Hz, 1H), 7.85 – 7.74 (m, 3H), 7.63 – 7.53 (m, 3H), 7.45 – 7.38 (m, 2H), 7.23 (dd, *J* = 2.1, 0.7 Hz, 1H), 1.99 (s, 3H), 1.29 (s, 9H). ^{13}C-NMR (100 MHz, $CDCl_3$) δ, ppm 170.6, 168.6, 167.0, 151.1, 137.5, 136.0, 134.9, 134.8, 134.4, 132.4, 131.9, 131.3, 131.2, 130.0, 129.9, 129.5, 129.1, 128.7, 124.4, 124.1, 34.5, 31.1, 19.9. HRMS (ESI-MS) *m/z* calculated for $C_{26}H_{23}NO_4$ $[M-H]^-$ 412.1554, found 412.1550. TLC (SiO_2, hexane:EtOAc 1:1), R_f = 0.43. $[a]_D^{25}$ = -17.22 (c 0.840, $CHCl_3$).

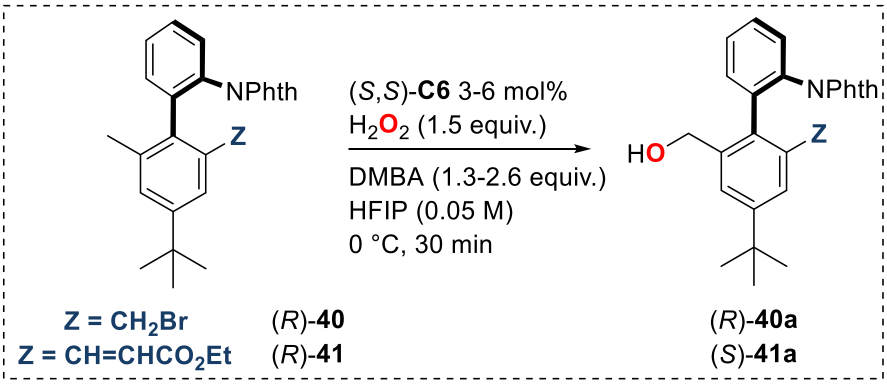

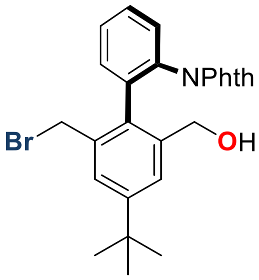

(*R*)-2-(2'-(bromomethyl)-4'-(tert-butyl)-6'-(hydroxymethyl)-[1,1'-biphenyl]-2-yl)isoindoline-1,3-dione ((*R*)-40a). It was isolated according to the General Procedure on an 0.025 mmol scale using *(S,S)*-**C6** as catalyst and DMBA (1.3 equiv.) as carboxylic acid in HFIP at 0ºC. **Additional 3 mol% of (S,S)-Mn($^{iPr\text{-}IQ}$pdp) and 1.3 equiv. of DMBA were added after 15 minutes.* It was purified by column chromatography over silica gel (hexane:EtOAc 3:1) to obtain the product as a colorless solid (6.2 mg, 0.015 mmol, 60% yield, >99% ee). ^{1}H-NMR (400 MHz, $CDCl_3$) δ, ppm: 7.82 – 7.75 (m, 2H), 7.73 – 7.71 (dd, *J* = 8.5, 1.5 Hz, 2H), 7.64 – 7.58 (m, 2H), 7.53 (dt, *J* = 6.3, 2.2 Hz, 1H), 7.50 (d, *J* = 2.0 Hz, 1H), 7.41 – 7.37 (m, 1H), 7.33 (d, *J* = 2.1 Hz, 1H), 4.48 – 4.35 (m, 2H), 4.34 – 4.28 (m, 2H), 1.26 (s, 9H). ^{13}C-NMR (100 MHz, $CDCl_3$) δ, ppm: 167.6, 167.4, 151.7, 140.2, 137.4, 136.1, 134.3, 134.3, 132.6, 132.2, 131.5, 131.5, 130.7, 129.5, 129.4, 129.1, 126.7, 125.9, 123.7, 123.6, 62.9, 34.6, 33.0, 31.1. HRMS (ESI-MS) *m/z* calculated for $C_{26}H_{24}BrNO_3$ $[M+Na]^+$ 500.0832, found 500.0827. TLC (SiO_2, hexane:EtOAc 7:3), R_f = 0.33. $[a]_D^{25}$ = -32.83 (c 0.300, $CHCl_3$).

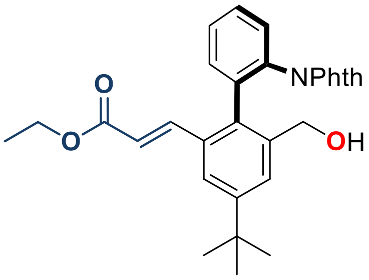

Ethyl (*S*)-3-(4-(tert-butyl)-2'-(1,3-dioxoisoindolin-2-yl)-6-(hydroxymethyl)-[1,1'-biphenyl]-2-yl)acrylate (*S*)-41a. It was isolated according to the General Procedure on an 0.03 mmol scale using *(S,S)-* **C6** as catalyst and AcOH (1.3 equiv.) as carboxylic acid in HFIP at 0ºC. **6 mol% of (S,S)-* **C6** *was used.* It was purified by column chromatography over silica gel (hexane:EtOAc 8:2) to obtain the product as a colorless liquid (9.3 mg, 0.020 mmol, 65% yield, >99% ee). ^{1}H-NMR (400 MHz, $CDCl_3$) δ, ppm: 7.81 – 7.78 (m, 1H), 7.74 – 7.64 (m, 4H), 7.62 – 7.54 (m, 2H), 7.44 – 7.40 (m, 2H), 7.37 – 7.32 (m, 2H), 6.05 (d, *J* = 15.8 Hz, 1H), 4.64 (d, *J* = 12.5 Hz, 1H), 4.44 (d, *J* = 12.5 Hz, 1H), 1.32 (t, *J* = 7.1 Hz, 3H), 1.30 (s, 9H). ^{13}C-NMR (100 MHz, $CDCl_3$) δ, ppm: 167.0, 166.5, 166.4, 151.3, 143.5, 140.3, 136.6, 134.3, 134.3, 134.1, 133.0, 132.1, 131.8, 131.4, 131.1, 129.5, 129.1, 129.0, 128.1, 123.6, 123.4, 122.1, 119.7, 63.0, 60.3, 34.7, 31.1, 14.3. HRMS (ESI-MS) *m/z* calculated for $C_{30}H_{29}NO_5$ $[M+H]^+$ 484.2118, found 484.2119. TLC (SiO_2, hexane:EtOAc 8:2), R_f = 0.3. $[a]_D^{25}$ = -113.34 (c 0.250, $CHCl_3$).

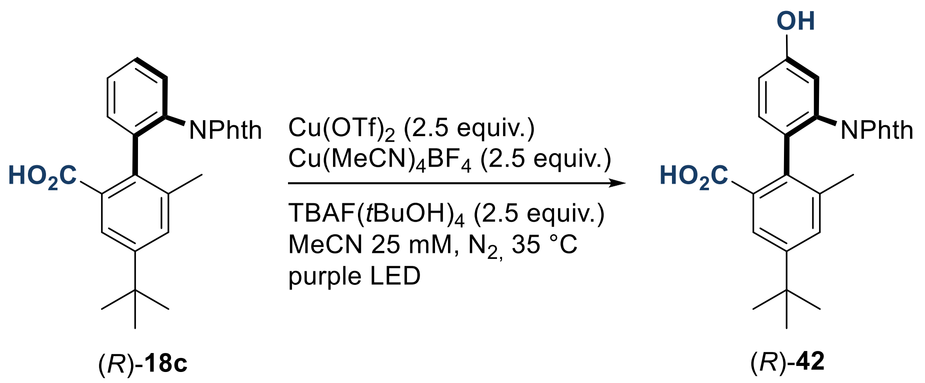

(*R*)-4-(*tert*-butyl)-2'-(1,3-dioxoisoindolin-2-yl)-4'-hydroxy-6-methyl-[1,1'-biphenyl]-2-carboxylic acid ((*R*)-42). It was prepared according to a modified reported procedure.[49] Under nitrogen atmosphere, a 6 mL vial equipped with a magnetic stir bar was charged with (*R*)-**18c** (33.0 mg, 0.08 mmol, 1.00 equiv.), $Cu(OTf)_2$ (72.0 mg, 0.2 mmol, 2.5 equiv.), $Cu(MeCN)_4BF_4$ (63.0 mg, 0.2 mmol, 2.5 equiv.), and TBAF(*t*BuOH)$_4$ (112 mg, 0.2 mmol, 2.5 equiv.). Anhydrous MeCN (3.2 mL, 25 mM) was then added into the vial. The vial was sealed, stirred for 30 min and kept under LED irradiation (KessilPR160-390 nm LED) at room temperature for 24 h. After irradiation, the reaction mixture was transferred to a 10 mL round-bottom flask and evaporated to dryness. The residue was dissolved in DCM (10 mL), transferred into a separatory funnel containing 5 mL saturated aqueous NH_4Cl and 5 mL H_2O, and the layers were separated. The organic layer was collected, and the aqueous layer was further extracted with DCM (3 × ca. 10 mL). The combined organic layer was dried over Mg_2SO_4, filtered, and the solvent was removed under reduced pressure. The residue was purified by chromatography on silica gel using hexane/AcOEt (1:1) to obtain the product as a pale yellow solid (23.2 mg, 0.054 mmol, 67% yield, 96% ee). ^{1}H-NMR (400 MHz, $CDCl_3$) δ, ppm: 7.96 (d, *J* = 7.3 Hz, 1H), 7.86 – 7.70 (m, 3H), 7.57 (d, *J* = 2.1 Hz, 1H), 7.21 (dd, *J* = 5.3, 3.2 Hz, 2H), 7.04 (dd, *J* = 8.4, 2.5 Hz, 1H), 6.92 (d, *J* = 2.5 Hz, 1H), 2.67 (s, 1H), 1.99 (s, 3H), 1.29 (s, 9H). ^{13}C-NMR (100 MHz, $CDCl_3$) δ, ppm: 171.9, 168.8, 166.8, 157.2, 150.9, 136.3, 135.0, 134.9, 134.8, 132.5, 132.2, 131.2, 131.1, 130.1, 129.9, 128.4, 124.1, 117.6, 116.0, 31.1, 28.8, 19.8. HRMS (ESI-MS) *m/z* calculated for $C_{26}H_{23}NO_5$ $[M-H]^-$ 428.1492, found

428.1490. TLC (SiO_2, hexane:EtOAc 1:1), R_f = 0.28. $[a]_D^{25}$ = -45.74 (c 0.170, $CHCl_3$). XRD structure found in CCDC Number 2527977.

7. KIE experiment

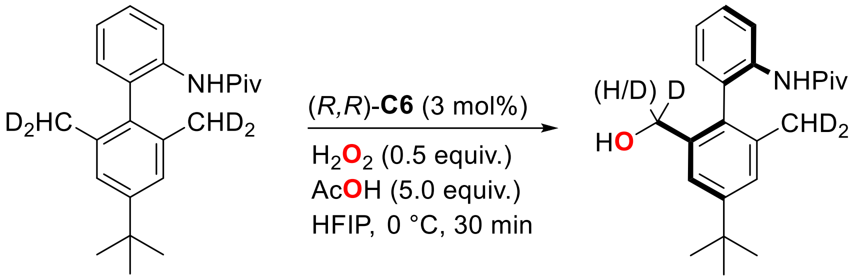

43 **43a**

43 was synthetized in order to calculate the intramolecular Kinetic Isotopic Effect, KIE (see above for more details about the synthetic procedure).

The reaction was performed following the general procedure reported in section **4.1** but using 0.5 equivalents of H_2O_2, resulting in ~10% conversion, in order to minimize formation of the overoxidized aldehyde byproduct, which would otherwise bias the KIE determination.

The crude reaction mixture was directly analyzed by GC–MS, and the KIE was determined from the relative isotopologue distribution of the product, reflecting the competition between hydrogen and deuterium at the reactive site (**Figure S1**). The isotopomeric products arising from oxidation at H or D were not separable by GC or NMR analysis.

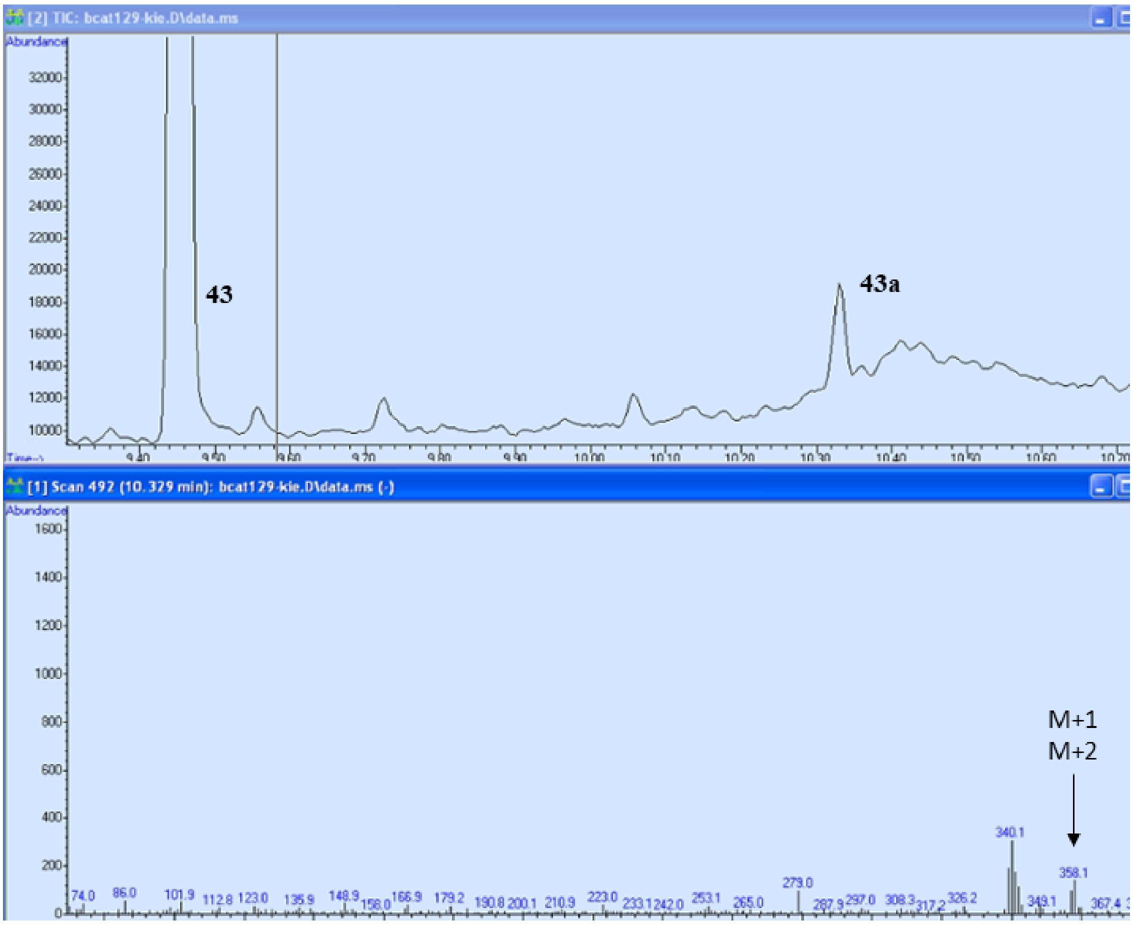

Figure S1. GC–MS spectrum of the crude reaction mixture after oxidation of **43**.

Scan 492 (10.329 min): bcat129-kie.D\data.ms

m/z	Abundance
348.00	60.0
348.80	20.0
349.10	21.0
352.10	16.0
353.20	13.0
354.00	15.0
355.20	20.0
356.20	19.0
357.10	112.0
358.10	191.0
359.30	36.0
360.00	77.0
361.00	20.0
361.70	12.0
362.00	11.0
363.10	13.0
364.00	157.0
364.90	41.0
366.00	18.0
366.90	10.0
367.40	12.0
[illegible]	[illegible]

[M+1] = 357.10 m/z

[M+2] = 358.10 m/z

Table S3. [M+1] and [M+2] values used for calculation of the KIE.

The KIE was extracted from GC–MS isotopologue distributions, corrected for natural abundance effects of ^{13}C and for statistical H/D site multiplicity. In particular, the signals at m/z 357.10 ([M+1], associated with D abstraction) and m/z 358.10 ([M+2], associated with H abstraction) as reported in **Table S3**, were used to calculate the KIE according to the following equation. The [M+2] signal was corrected for the contribution of ^{13}C natural abundance to the [M+1] isotopologue.

$$KIE = \frac{M+2_{corrected\ 13C}/n_H}{M+1/n_D} = \frac{162.7/2}{112/4} = 2.9$$

A duplicate of the experiment provided KIE = 2.5.

KIE = 2.7±0.2

8. Labelling experiment

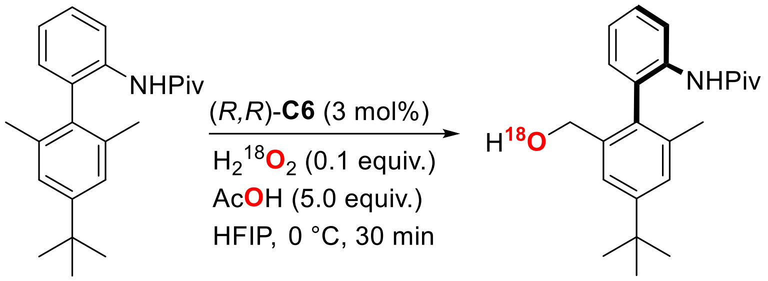

15 **15a**[18]

The oxidation of **15** with ^{18}O-labelled peroxide (purchased from Gioxcat) was performed following the general procedure reported in section **4.1** but using 0.1 equivalents of $H_2{}^{18}O_2$.

Composition of labelled hydrogen peroxide: ^{18}O-^{18}O 78.8%; ^{18}O-^{16}O 12.8%; ^{16}O-^{16}O 8.4% (GC-MS analysis). Relative percentual of ^{18}O: **85.2%**.

After catalysis **88%** ^{18}**O** incorporation in **14a**[18] was observed (GC-MS analysis, **Figure S2** and **Table S4**). This result shows that ^{18}**O** derived from labelled hydrogen peroxide was fully incorporated in the resulting product.

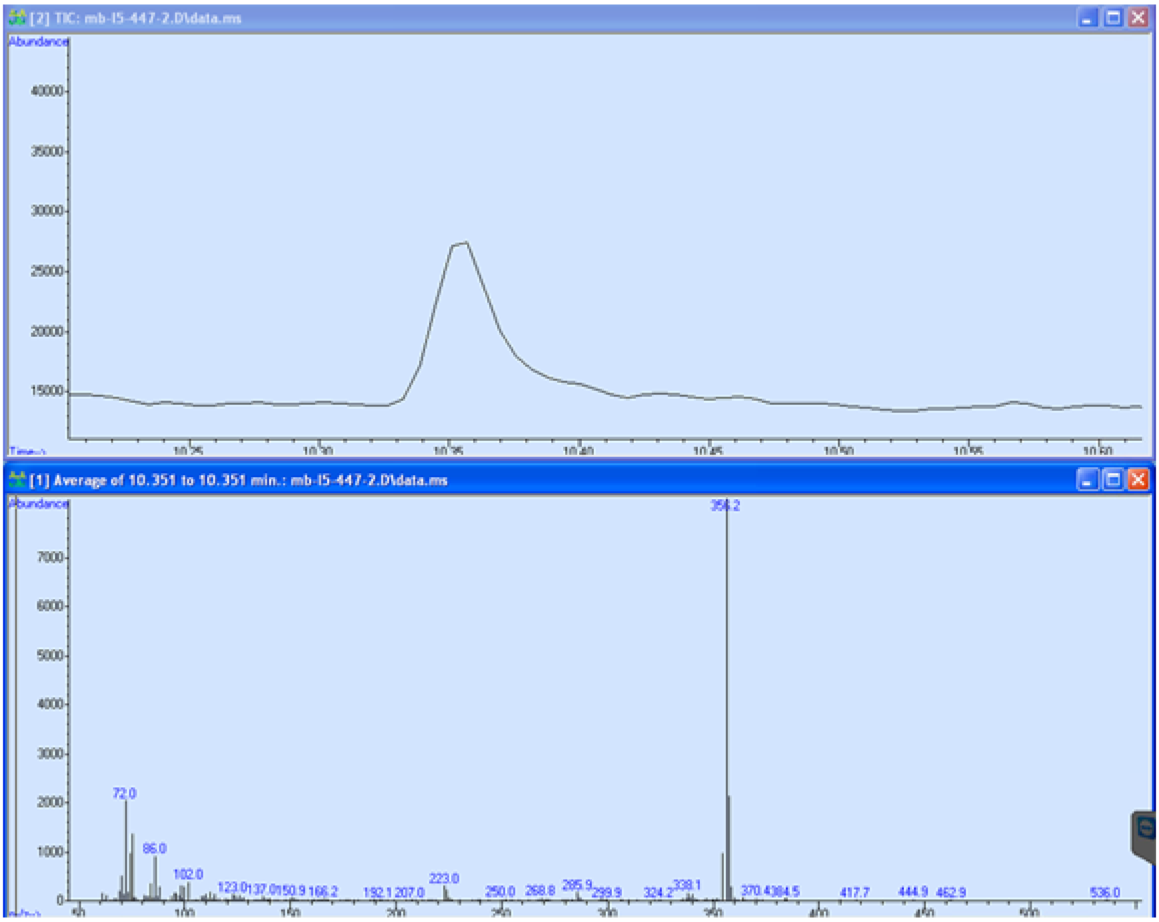

Figure S2. GC–MS spectrum of the crude reaction mixture after oxidation of **15**.

m/z	Abundance
346.80	53.0
347.90	19.0
349.00	19.0
349.70	14.0
350.10	14.0
351.20	12.0
352.00	49.0
352.60	12.0
354.20	979.0
356.20	8205.0
357.20	2128.0
358.20	281.0
359.90	65.0
360.90	13.0
361.80	13.0
362.90	11.0
363.90	76.0
364.80	21.0
365.90	18.0
367.90	13.0
370.40	51.0
[illegible]	[illegible]

Table S4. Values used for calculation of the ^{18}O incorporation in **15a**.

9. **X-ray Structures**

9.1. Solid structures of manganese catalysts

<u>(*R*,*R*)-Mn(^{Me}iQdp)</u> (**C5**)

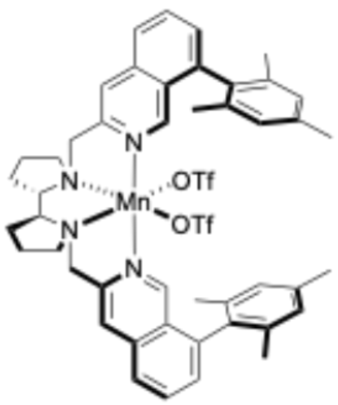

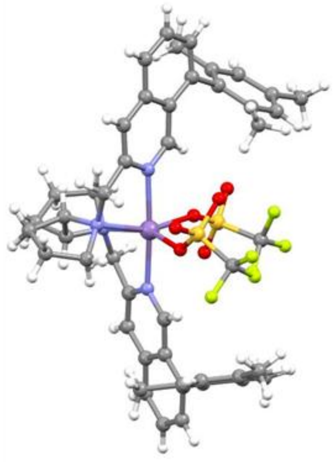

Table S5. Crystal data for (*R*,*R*)-**C5** (CCDC Number 2519084)

**Chemical formula**	$C_{49}H_{52}Cl_2F_6MnN_4O_6S_2$	
**Formula weight**	1096.90 g/mol	
**Temperature**	100(2) K	
**Wavelength**	0.71073 Å	
**Crystal size**	0.060 x 0.090 x 0.400 mm	
**Crystal habit**	colorless needle	
**Crystal system**	monoclinic	
**Space group**	C 1 2 1	
**Unit cell dimensions**	a = 28.565(4) Å	α = 90°
	b = 13.0456(14) Å	β = 121.019(3)°
	c = 18.938(2) Å	γ = 90°
**Volume**	6048.0(12) $Å^3$	
**Z**	4	
**Density (calculated)**	1.205 g/cm^3	
**Absorption coefficient**	0.438 mm^{-1}	
**F(000)**	2268	
**Diffractometer**	D8 QUEST ECO three-circle diffractometer	
**Radiation source**	Ceramic x-ray tube (Mo Kα, λ = 0.71073 Å)	
**Theta range for data collection**	2.18 to 27.57°	
**Index ranges**	-37<=h<=37, -16<=k<=16, -24<=l<=24	
**Reflections collected**	83832	

**Independent reflections**	13921 [R(int) = 0.1351]	
**Coverage of independent reflections**	99.6%	
**Absorption correction**	Multi-Scan	
**Max. and min. transmission**	0.9740 and 0.8440	
**Structure solution technique**	direct methods	
**Structure solution program**	XT, VERSION 2018/2	
**Refinement method**	Full-matrix least-squares on F2	
**Refinement program**	SHELXL-2019/1 (Sheldrick, 2019)	
**Function minimized**	Σ w(Fo2 - Fc2)2	
**Data / restraints / parameters**	13921 / 1 / 638	
**Goodness-of-fit on F2**	1.027	
**Δ/σmax**	0.001	
**Final R indices**	9056 data; I>2σ(I)	R1 = 0.0791, wR2 = 0.1906
	all data	R1 = 0.1340, wR2 = 0.2254
**Weighting scheme**	$w=1/[\sigma^2(F_o^2)+(0.1171P)^2+10.3657P]$ where $P=(F_o^2+2F_c^2)/3$	
**Absolute structure parameter**	0.043(13)	
**Largest diff. peak and hole**	0.895 and -0.876 eÅ^{-3}	
**R.M.S. deviation from mean**	0.086 eÅ^{-3}	

(*R*,*R*)-Mn(^{iPr}iQdp) (**C6**)

The crystalline material of the manganese catalyst containing OTf ligands showed significant disorder, preventing the determination of a reliable X-ray structure. Consequently, the crystal structure was obtained after recrystallization of the complex from DMSO, resulting in coordination of DMSO ligands in place of OTf.

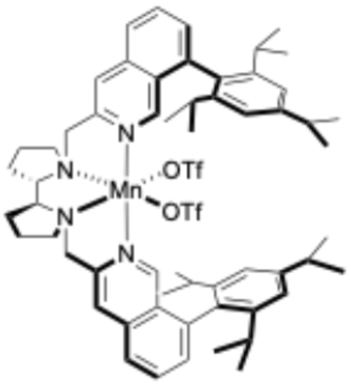

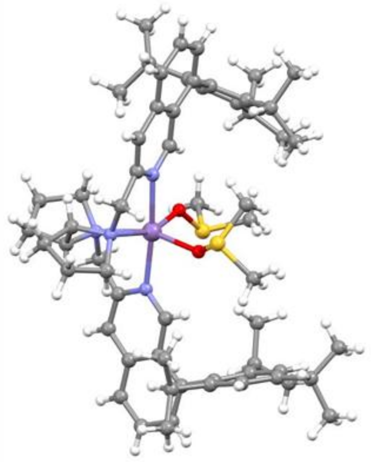

Table S6. Crystal data for (*R*,*R*)-**C6** (CCDC Number 2519089)

**Chemical formula**	$C_{134}H_{192}F_{12}Mn_2N_8O_{20}S_{11}$
**Formula weight**	2925.48 g/mol
**Temperature**	130(2) K
**Wavelength**	0.71073 Å
**Crystal size**	0.030 x 0.170 x 0.210 mm
**Crystal habit**	yellow prism
**Crystal system**	monoclinic
**Space group**	C 1 2 1
**Unit cell dimensions**	a = 22.9428(9) Å α = 90°
	b = 15.7159(6) Å β = 109.6140(10)°
	c = 22.5517(9) Å γ = 90°
**Volume**	7659.6(5) $Å^3$
**Z**	2
**Density (calculated)**	1.268 g/cm^3
**Absorption coefficient**	0.391 mm^{-1}
**F(000)**	3092
**Diffractometer**	D8 QUEST ECO three-circle diffractometer

**Radiation source**	Ceramic x-ray tube (Mo Kα, λ = 0.71073 Å)
**Theta range for data collection**	2.24 to 27.55°
**Index ranges**	-29<=h<=29, -20<=k<=20, -29<=l<=29
**Reflections collected**	91164
**Independent reflections**	17582 [R(int) = 0.0201]
**Coverage of independent reflections**	99.6%
**Absorption correction**	Multi-Scan
**Max. and min. transmission**	0.9880 and 0.9220
**Structure solution technique**	direct methods
**Structure solution program**	XT, VERSION 2018/2
**Refinement method**	Full-matrix least-squares on F2
**Refinement program**	SHELXL-2019/1 (Sheldrick, 2019)
**Function minimized**	Σ w(Fo2 - Fc2)2
**Data / restraints / parameters**	17582 / 1 / 874
**Goodness-of-fit on F2**	1.083
**Δ/σmax**	0.038
**Final R indices**	16173 data; I>2σ(I) R1 = 0.0810, wR2 = 0.2234
	all data R1 = 0.0909, wR2 = 0.2404
**Weighting scheme**	$w=1/[\sigma^2(F_o^2)+(0.1834P)^2+7.5287P]$ where $P=(F_o^2+2F_c^2)/3$
**Absolute structure parameter**	-0.010(3)
**Extinction coefficient**	0.0040(7)
**Largest diff. peak and hole**	1.629 and -0.976 eÅ^{-3}
**R.M.S. deviation from mean**	0.251 eÅ^{-3}

9.2. Solid structures of the atropoisomeric products

(*R*)-4'-(*tert*-butyl)-2'-formyl-6'-methyl-[1,1'-biphenyl]-2-carbonitrile (*R*)-**1b**

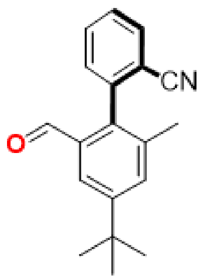

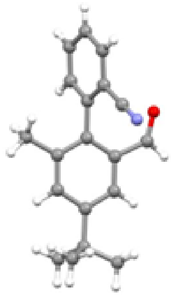

Table S7. Crystal data for (*R*)-**1b** (CCDC Number 2519154)

**Chemical formula**	$C_{19}H_{19}NO$	
**Formula weight**	277.35 g/mol	
**Temperature**	100(2) K	
**Wavelength**	0.71073 Å	
**Crystal size**	0.060 x 0.080 x 0.300 mm	
**Crystal habit**	colorless needle	
**Crystal system**	monoclinic	
**Space group**	P 1 21 1	
**Unit cell dimensions**	a = 6.9288(4) Å	α = 90°
	b = 20.5859(9) Å	β = 90.208(2)°
	c = 10.7759(5) Å	γ = 90°
**Volume**	1537.02(13) $Å^3$	
**Z**	4	
**Density (calculated)**	1.199 g/cm^3	
**Absorption coefficient**	0.074 mm^{-1}	
**F(000)**	592	
**Diffractometer**	D8 QUEST ECO three-circle diffractometer	
**Radiation source**	Ceramic x-ray tube (Mo Kα, λ = 0.71073 Å)	
**Theta range for data collection**	2.94 to 27.54°	
**Index ranges**	-9<=h<=9, -26<=k<=26, -13<=l<=14	
**Reflections collected**	50011	
**Independent reflections**	7029 [R(int) = 0.0748]	
**Coverage of independent reflections**	99.6%	
**Absorption correction**	Multi-Scan	
**Max. and min. transmission**	0.9960 and 0.9780	
**Structure solution technique**	direct methods	

**Structure solution program**	XT, VERSION 2018/2	
**Refinement method**	Full-matrix least-squares on F2	
**Refinement program**	SHELXL-2019/1 (Sheldrick, 2019)	
**Function minimized**	Σ w(Fo2 - Fc2)2	
**Data / restraints / parameters**	7029 / 1 / 387	
**Goodness-of-fit on F2**	1.060	
**Final R indices**	6270 data; I>2σ(I)	R1 = 0.0424, wR2 = 0.1012
	all data	R1 = 0.0530, wR2 = 0.1074
**Weighting scheme**	$w=1/[\sigma^2(F_o^2)+(0.0489P)^2+0.3919P]$<br>where $P=(F_o^2+2F_c^2)/3$	
**Absolute structure parameter**	-0.1(6)	
**Largest diff. peak and hole**	0.293 and -0.194 eÅ^{-3}	
**R.M.S. deviation from mean**	0.044 eÅ^{-3}	

(*R*)-(4-(*tert*-butyl)-6-methyl-2'-(methylsulfonyl)-[1,1'-biphenyl]-2-yl)methanol (*R*)-**5a**

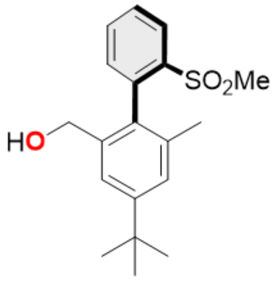

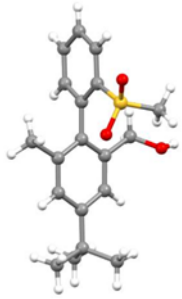

Table S8. Crystal data for (*R*)-**5a** (CCDC Number 2519155)

**Chemical formula**	$C_{19}H_{24}O_3S$
**Formula weight**	332.44 g/mol
**Temperature**	130(2) K
**Wavelength**	0.71073 Å
**Crystal size**	0.280 x 0.280 x 0.320 mm
**Crystal habit**	colorless prism
**Crystal system**	monoclinic
**Space group**	C 1 2 1
**Unit cell dimensions**	a = 26.665(2) Å α = 90°
	b = 8.1061(7) Å β = 91.806(3)°
	c = 16.3334(14) Å γ = 90°
**Volume**	3528.7(5) Å^3
**Z**	8
**Density (calculated)**	1.252 g/cm^3
**Absorption coefficient**	0.196 mm^{-1}
**F(000)**	1424
**Diffractometer**	D8 QUEST ECO three-circle diffractometer
**Radiation source**	Ceramic x-ray tube (Mo Kα, λ = 0.71073 Å)
**Theta range for data collection**	2.88 to 28.37°
**Index ranges**	-35<=h<=35, -10<=k<=10, -21<=l<=21
**Reflections collected**	73216
**Independent reflections**	8785 [R(int) = 0.0332]
**Coverage of independent reflections**	99.4%
**Absorption correction**	Multi-Scan
**Max. and min. transmission**	0.9470 and 0.9400
**Structure solution technique**	direct methods
**Structure solution program**	XT, VERSION 2018/2

**Refinement method**	Full-matrix least-squares on F2	
**Refinement program**	SHELXL-2019/1 (Sheldrick, 2019)	
**Function minimized**	Σ w(Fo2 - Fc2)2	
**Data / restraints / parameters**	8785 / 1 / 463	
**Goodness-of-fit on F2**	1.033	
**Δ/σmax**	0.005	
**Final R indices**	7758 data; I>2σ(I)	R1 = 0.0538, wR2 = 0.1487
	all data	R1 = 0.0615, wR2 = 0.1566
**Weighting scheme**	$w=1/[\sigma^2(F_o^2)+(0.0915P)^2+3.2560P]$ where $P=(F_o^2+2F_c^2)/3$	
**Absolute structure parameter**	0.00(2)	
**Largest diff. peak and hole**	0.562 and -0.398 eÅ^{-3}	
**R.M.S. deviation from mean**	0.066 eÅ^{-3}	

N-(4'-(*tert*-butyl)-2'-(hydroxymethyl)-6'-methyl-[1,1'-biphenyl]-2-yl)pivalamide (*R*)-**15a**

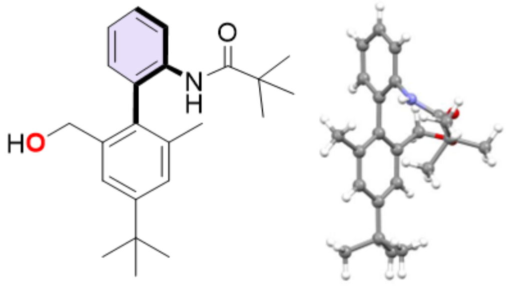

Table S9. Crystal data for (*R*)-**15a** (CCDC Number 2519156)

**Chemical formula**	$C_{23}H_{31}NO_2$	
**Formula weight**	353.49 g/mol	
**Temperature**	100(2) K	
**Wavelength**	0.71076 Å	
**Crystal size**	0.100 x 0.120 x 0.480 mm	
**Crystal habit**	yellow prism	
**Crystal system**	orthorhombic	
**Space group**	P 21 21 21	
**Unit cell dimensions**	a = 9.405(2) Å	α = 90°
	b = 12.744(3) Å	β = 90°
	c = 16.440(4) Å	γ = 90°
**Volume**	1970.5(8) $Å^3$	
**Z**	4	
**Density (calculated)**	1.191 g/cm^3	
**Absorption coefficient**	0.075 mm^{-1}	
**F(000)**	768	
**Diffractometer**	D8 QUEST ECO three-circle diffractometer	
**Radiation source**	Ceramic x-ray tube (Mo Kα, λ = 0.71076 Å)	
**Theta range for data collection**	2.50 to 27.73°	
**Index ranges**	-12<=h<=12, -16<=k<=16, -21<=l<=21	
**Reflections collected**	38703	
**Independent reflections**	4613 [R(int) = 0.1951]	
**Coverage of independent reflections**	99.4%	
**Absorption correction**	Multi-Scan	
**Max. and min. transmission**	0.9930 and 0.9650	
**Structure solution technique**	direct methods	
**Structure solution program**	XT, VERSION 2018/2	
**Refinement method**	Full-matrix least-squares on F2	

**Refinement program**	SHELXL-2019/1 (Sheldrick, 2019)
**Function minimized**	Σ w(Fo2 - Fc2)2
**Data / restraints / parameters**	4613 / 0 / 231
**Goodness-of-fit on F2**	1.085
**Final R indices**	3305 data; $I>2\sigma(I)$ R1 = 0.1110, wR2 = 0.2075
	all data R1 = 0.1544, wR2 = 0.2284
**Weighting scheme**	$w=1/[\sigma^2(F_o^2)+(0.0208P)^2+8.7195P]$ where $P=(F_o^2+2F_c^2)/3$
**Absolute structure parameter**	-1.0(10)
**Largest diff. peak and hole**	0.576 and -0.496 $eÅ^{-3}$
**R.M.S. deviation from mean**	0.089 $eÅ^{-3}$

N-(4'-(*tert*-butyl)-2'-(hydroxymethyl)-6'-methyl-[1,1'-biphenyl]-2-yl)-*N*-methyl-3,5-bis(trifluoromethyl) benzamide (*R*)-**17a**

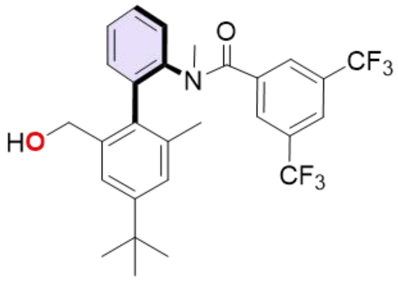

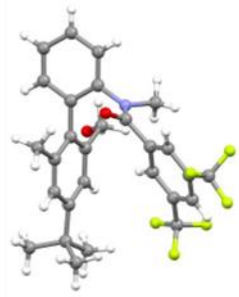

Table S10. Crystal data for (*R*)-**17a** (CCDC Number 2525663)

**Chemical formula**	$C_{28}H_{27}F_6NO_2$	
**Formula weight**	523.50 g/mol	
**Temperature**	100(2) K	
**Wavelength**	0.71073 Å	
**Crystal size**	0.080 x 0.080 x 0.100 mm	
**Crystal system**	monoclinic	
**Space group**	P 1 21 1	
**Unit cell dimensions**	a = 13.6519(11) Å	α = 90°
	b = 13.7714(11) Å	β = 103.077(2)°
	c = 28.499(2) Å	γ = 90°
**Volume**	5219.0(7) $Å^3$	
**Z**	8	
**Density (calculated)**	1.333 g/cm^3	
**Absorption coefficient**	0.112 mm^{-1}	
**F(000)**	2176	
**Diffractometer**	D8 QUEST ECO three-circle diffractometer	
**Radiation source**	Ceramic x-ray tube (Mo Kα, λ = 0.71073 Å)	
**Theta range for data collection**	2.14 to 27.58°	
**Index ranges**	-17<=h<=17, -17<=k<=17, -37<=l<=37	
**Reflections collected**	152330	
**Independent reflections**	23674 [R(int) = 0.0528]	
**Coverage of independent reflections**	99.2%	
**Absorption correction**	Multi-Scan	
**Max. and min. transmission**	0.9910 and 0.9890	
**Structure solution technique**	direct methods	
**Structure solution program**	XT, VERSION 2018/2	

**Refinement method**	Full-matrix least-squares on F2	
**Refinement program**	SHELXL-2019/1 (Sheldrick, 2019)	
**Function minimized**	Σ w(Fo2 - Fc2)2	
**Data / restraints / parameters**	23674 / 2 / 1333	
**Goodness-of-fit on F2**	1.144	
**Final R indices**	21612 data; I>2σ(I)	R1 = 0.1050, wR2 = 0.2543
	all data	R1 = 0.1130, wR2 = 0.2600
**Weighting scheme**	$w=1/[\sigma^2(F_o^2)+(0.0961P)^2+20.7394P]$<br>where $P=(F_o^2+2F_c^2)/3$	
**Absolute structure parameter**	0.06(13)	
**Largest diff. peak and hole**	0.872 and -0.890 eÅ^{-3}	
**R.M.S. deviation from mean**	0.116 eÅ^{-3}	

2-(4'-(*tert*-butyl)-2'-(hydroxymethyl)-6'-methyl-[1,1'-biphenyl]-2-yl)isoindoline-1,3-dione
(*R*)-**18a**

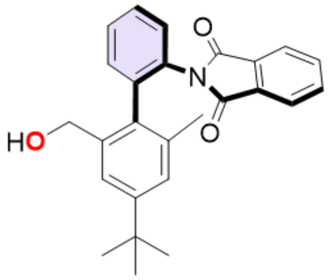

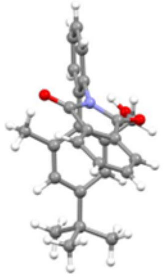

Table S11. Crystal data for *(R)*-**18a** (CCDC Number 2519273)

**Chemical formula**	$C_{26}H_{25}NO_3$	
**Formula weight**	399.47 g/mol	
**Temperature**	100(2) K	
**Wavelength**	0.71073 Å	
**Crystal size**	0.020 x 0.060 x 0.300 mm	
**Crystal habit**	colorless needle	
**Crystal system**	orthorhombic	
**Space group**	P b c a	
**Unit cell dimensions**	a = 14.571(8) Å	α = 90°
	b = 13.197(8) Å	β = 90°
	c = 21.944(11) Å	γ = 90°
**Volume**	4220.(4) $Å^3$	
**Z**	8	
**Density (calculated)**	1.258 g/cm^3	
**Absorption coefficient**	0.082 mm^{-1}	
**F(000)**	1696	
**Diffractometer**	D8 QUEST ECO three-circle diffractometer	
**Radiation source**	Ceramic x-ray tube (Mo Kα, λ = 0.71073 Å)	
**Theta range for data collection**	2.32 to 27.10°	
**Index ranges**	-18<=h<=18, -16<=k<=16, -28<=l<=28	
**Reflections collected**	115096	
**Independent reflections**	4650 [R(int) = 0.2466]	
**Coverage of independent reflections**	99.9%	
**Absorption correction**	Multi-Scan	
**Max. and min. transmission**	0.9980 and 0.9760	
**Structure solution technique**	direct methods	
**Structure solution program**	XT, VERSION 2018/2	
**Refinement method**	Full-matrix least-squares on F2	

**Refinement program**	SHELXL-2019/1 (Sheldrick, 2019)	
**Function minimized**	Σ w(Fo2 - Fc2)2	
**Data / restraints / parameters**	4650 / 0 / 288	
**Goodness-of-fit on F2**	1.191	
**Final R indices**	3295 data; I>2σ(I)	R1 = 0.1153, wR2 = 0.2131
	all data	R1 = 0.1590, wR2 = 0.2359
**Weighting scheme**	$w=1/[\sigma^2(F_o^2)+(0.0001P)^2+29.7058P]$<br>where $P=(F_o^2+2F_c^2)/3$	
**Largest diff. peak and hole**	0.393 and -0.451 eÅ^{-3}	
**R.M.S. deviation from mean**	0.093 eÅ^{-3}	

N-(4'-(*tert*-butyl)-4-chloro-2'-(hydroxymethyl)-6'-methyl-[1,1'-biphenyl]-2-yl)pivalamide (*R*)-**28a**

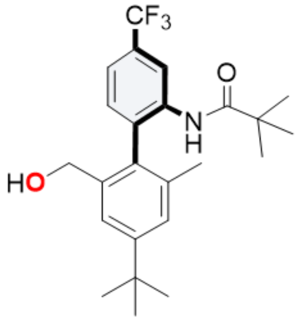

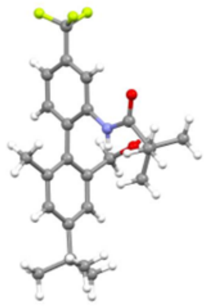

Table S12. Crystal data for (*R*)-**28a** (CCDC Number 2519278)

**Chemical formula**	$C_{24}H_{30}F_3NO_2$	
**Formula weight**	421.49 g/mol	
**Temperature**	100(2) K	
**Wavelength**	0.71073 Å	
**Crystal size**	0.080 x 0.100 x 0.400 mm	
**Crystal habit**	colorless needle	
**Crystal system**	orthorhombic	
**Space group**	P 21 21 21	
**Unit cell dimensions**	a = 10.5692(7) Å	α = 90°
	b = 14.3515(9) Å	β = 90°
	c = 14.6956(9) Å	γ = 90°
**Volume**	2229.1(2) $Å^3$	
**Z**	4	
**Density (calculated)**	1.256 g/cm^3	
**Absorption coefficient**	0.096 mm^{-1}	
**F(000)**	896	
**Diffractometer**	D8 QUEST ECO three-circle diffractometer	
**Radiation source**	Ceramic x-ray tube (Mo Kα, λ = 0.71073 Å)	
**Theta range for data collection**	3.12 to 27.50°	
**Index ranges**	-13<=h<=13, -18<=k<=18, -19<=l<=19	
**Reflections collected**	41505	
**Independent reflections**	5118 [R(int) = 0.1147]	
**Coverage of independent reflections**	99.8%	
**Absorption correction**	Multi-Scan	
**Max. and min. transmission**	0.9920 and 0.9630	
**Structure solution technique**	direct methods	

**Structure solution program**	XT, VERSION 2018/2
**Refinement method**	Full-matrix least-squares on F2
**Refinement program**	SHELXL-2019/1 (Sheldrick, 2019)
**Function minimized**	Σ w(Fo2 - Fc2)2
**Data / restraints / parameters**	5118 / 0 / 286
**Goodness-of-fit on F2**	1.035
**Final R indices**	3903 data; I>2σ(I) R1 = 0.0460, wR2 = 0.0819
	all data R1 = 0.0776, wR2 = 0.0935
**Weighting scheme**	$w=1/[\sigma^2(F_o^2)+(0.0289P)^2+0.6227P]$<br>where $P=(F_o^2+2F_c^2)/3$
**Absolute structure parameter**	-0.4(5)
**Largest diff. peak and hole**	0.228 and -0.253 eÅ^{-3}
**R.M.S. deviation from mean**	0.047 eÅ^{-3}

N-(4'-(*tert*-butyl)-2'-(hydroxymethyl)-6'-methyl-5-nitro-[1,1'-biphenyl]-2-yl)pivalamide (*R*)-**31a**

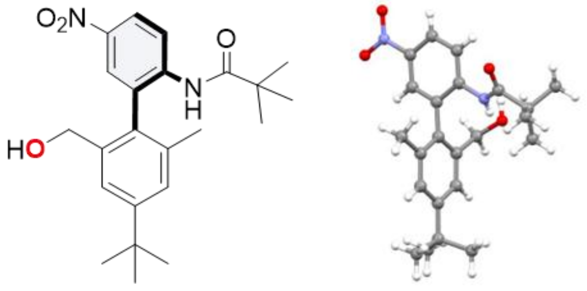

Table S13. Crystal data for (*R*)-**31a** (CCDC Number 2519281)

**Chemical formula**	$C_{23}H_{30}N_2O_4$	
**Formula weight**	398.49 g/mol	
**Temperature**	100(2) K	
**Wavelength**	0.71073 Å	
**Crystal size**	0.200 x 0.200 x 0.220 mm	
**Crystal habit**	colorless prism	
**Crystal system**	monoclinic	
**Space group**	P 1 21 1	
**Unit cell dimensions**	a = 9.0729(8) Å	α = 90°
	b = 18.1621(15) Å	β = 91.231(3)°
	c = 13.3771(12) Å	γ = 90°
**Volume**	2203.8(3) $Å^3$	
**Z**	4	
**Density (calculated)**	1.201 g/cm^3	
**Absorption coefficient**	0.082 mm^{-1}	
**F(000)**	856	
**Diffractometer**	D8 QUEST ECO three-circle diffractometer	
**Radiation source**	Ceramic x-ray tube (Mo Kα, λ = 0.71073 Å)	
**Theta range for data collection**	2.25 to 28.50°	
**Index ranges**	-12<=h<=12, -23<=k<=24, -17<=l<=17	
**Reflections collected**	63488	
**Independent reflections**	10969 [R(int) = 0.0843]	
**Coverage of independent reflections**	99.0%	
**Absorption correction**	Multi-Scan	
**Max. and min. transmission**	0.9840 and 0.9820	
**Structure solution technique**	direct methods	
**Structure solution program**	XT, VERSION 2018/2	

**Refinement method**	Full-matrix least-squares on F2	
**Refinement program**	SHELXL-2019/1 (Sheldrick, 2019)	
**Function minimized**	Σ w(Fo2 - Fc2)2	
**Data / restraints / parameters**	10969 / 1 / 545	
**Goodness-of-fit on F2**	1.026	
**Final R indices**	8615 data; I>2σ(I)	R1 = 0.0767, wR2 = 0.1907
	all data	R1 = 0.1003, wR2 = 0.2057
**Weighting scheme**	$w=1/[\sigma^2(F_o^2)+(0.0984P)^2+2.4943P]$<br>where $P=(F_o^2+2F_c^2)/3$	
**Absolute structure parameter**	-0.3(6)	
**Largest diff. peak and hole**	0.391 and -0.356 eÅ^{-3}	
**R.M.S. deviation from mean**	0.073 eÅ^{-3}	

2-(4'-(*tert*-butyl)-2'-ethyl-6'-(1-hydroxyethyl)-[1,1'-biphenyl]-2-yl)isoindoline-1,3-dione **39a**

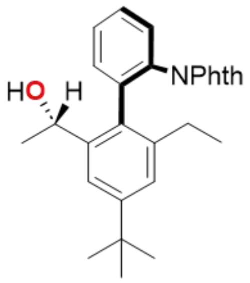

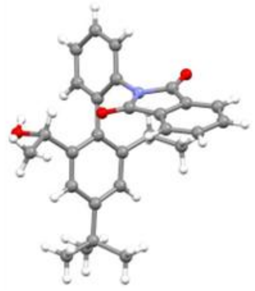

Table S14. Crystal data for **39a** (CCDC Number 2519371)

**Chemical formula**	$C_{28}H_{29}NO_3$	
**Formula weight**	427.52 g/mol	
**Temperature**	100(2) K	
**Wavelength**	0.71073 Å	
**Crystal size**	0.050 x 0.140 x 0.170 mm	
**Crystal habit**	colorless plate	
**Crystal system**	monoclinic	
**Space group**	C 1 2/c 1	
**Unit cell dimensions**	a = 29.513(6) Å	α = 90°
	b = 8.6955(18) Å	β = 115.442(6)°
	c = 19.820(4) Å	γ = 90°
**Volume**	4593.1(17) $Å^3$	
**Z**	8	
**Density (calculated)**	1.236 g/cm^3	
**Absorption coefficient**	0.080 mm^{-1}	
**F(000)**	1824	
**Diffractometer**	D8 QUEST ECO three-circle diffractometer	
**Radiation source**	Ceramic x-ray tube (Mo Kα, λ = 0.71073 Å)	
**Theta range for data collection**	2.28 to 28.18°	
**Index ranges**	-38<=h<=38, -11<=k<=11, -25<=l<=25	
**Reflections collected**	56809	
**Independent reflections**	5465 [R(int) = 0.1535]	
**Coverage of independent reflections**	96.6%	
**Absorption correction**	Multi-Scan	
**Max. and min. transmission**	0.9960 and 0.9870	
**Structure solution technique**	direct methods	
**Structure solution program**	XT, VERSION 2018/2	

**Refinement method**	Full-matrix least-squares on F2
**Refinement program**	SHELXL-2019/1 (Sheldrick, 2019)
**Function minimized**	Σ w(Fo2 - Fc2)2
**Data / restraints / parameters**	5465 / 1 / 337
**Goodness-of-fit on F2**	1.190
**Final R indices**	3702 data; I>2σ(I) R1 = 0.1158, wR2 = 0.2272
	all data R1 = 0.1643, wR2 = 0.2481
**Weighting scheme**	$w=1/[\sigma^2(F_o^2)+(0.0593P)^2+23.3270P]$ where $P=(F_o^2+2F_c^2)/3$
**Largest diff. peak and hole**	0.373 and -0.409 eÅ^{-3}
**R.M.S. deviation from mean**	0.073 eÅ^{-3}

(*R*)-4-(*tert*-butyl)-2'-(1,3-dioxoisoindolin-2-yl)-4'-hydroxy-6-methyl-[1,1'-biphenyl]-2-carboxylic acid ((*R*)-**42**).

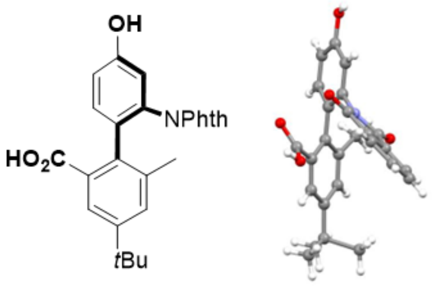

Table S15. Crystal data for **42** (CCDC Number 2527977)

**Chemical formula**	$C_{27}H_{25}Cl_2NO_5$	
**Formula weight**	514.38 g/mol	
**Temperature**	100(2) K	
**Wavelength**	0.71073 Å	
**Crystal size**	0.030 x 0.060 x 0.260 mm	
**Crystal habit**	colorless needle	
**Crystal system**	triclinic	
**Space group**	P -1	
**Unit cell dimensions**	a = 9.64(5) Å	α = 82.17(12)°
	b = 10.87(5) Å	β = 76.67(14)°
	c = 13.04(6) Å	γ = 78.12(13)°
**Volume**	1295.(11) $Å^3$	
**Z**	2	
**Density (calculated)**	1.318 g/cm^3	
**Absorption coefficient**	0.288 mm^{-1}	
**F(000)**	536	
**Diffractometer**	D8 QUEST ECO three-circle diffractometer	
**Radiation source**	Ceramic x-ray tube (Mo Kα, λ = 0.71073 Å)	
**Theta range for data collection**	2.99 to 27.46°	
**Index ranges**	-12<=h<=12, -13<=k<=14, -16<=l<=16	
**Reflections collected**	31941	
**Independent reflections**	5673 [R(int) = 0.1095]	
**Coverage of independent reflections**	95.5%	
**Absorption correction**	Multi-Scan	
**Max. and min. transmission**	0.9910 and 0.9290	
**Structure solution technique**	direct methods	
**Structure solution program**	XT, VERSION 2018/2	
**Refinement method**	Full-matrix least-squares on F2	

**Refinement program**	SHELXL-2019/1 (Sheldrick, 2019)
**Function minimized**	Σ w(Fo2 - Fc2)2
**Data / restraints / parameters**	5673 / 18 / 374
**Goodness-of-fit on F2**	1.141
**Final R indices**	4125 data; I>2σ(I) R1 = 0.0955, wR2 = 0.1730
	all data R1 = 0.1299, wR2 = 0.1869
**Weighting scheme**	$w=1/[\sigma^2(F_o^2)+(0.0341P)^2+2.9142P]$ where $P=(F_o^2+2F_c^2)/3$
**Largest diff. peak and hole**	0.529 and -0.516 eÅ^{-3}
**R.M.S. deviation from mean**	0.068 eÅ^{-3}

10. Computational studies

10.1. Buried volume analysis of Mn-oxo species

To elucidate the factors governing atroposelectivity in the non-directed oxidation of the *meso*-biaryl substrates, we performed a buried volume (V_{bur} (%)) analysis, [50] centered on the oxo unit of the catalytically active manganese-oxo carboxylato species, responsible for hydrogen atom transfer (HAT).[51] In this structure, the lone pair of the carbonyl group of the η^1-acetate ligand engages in a weak O···O interaction with the unpaired p-electron density of the oxo ligand. Previous studies on iron[52] and manganese[53] catalysts have shown that such interactions energetically stabilize the active species, thereby facilitating HAT reactivity.[51]
To facilitate direct comparison of the occupied quadrants, steric maps were generated for all catalysts in their R,R configuration. The steric maps were calculated for the most stable conformer of each manganese-oxo carboxylato species using the SambVca application,[54] employing a sphere radius of 6.5 Å centered on the oxo unit and a mesh size of 0.03. The oxo ligand was aligned along the Mn=O vector, with the z axis oriented toward the reader. Quadrant-resolved V_{bur} (%)) values (NW, NE, SW, and SE) were extracted and correlated with the experimentally observed enantioselectivity.

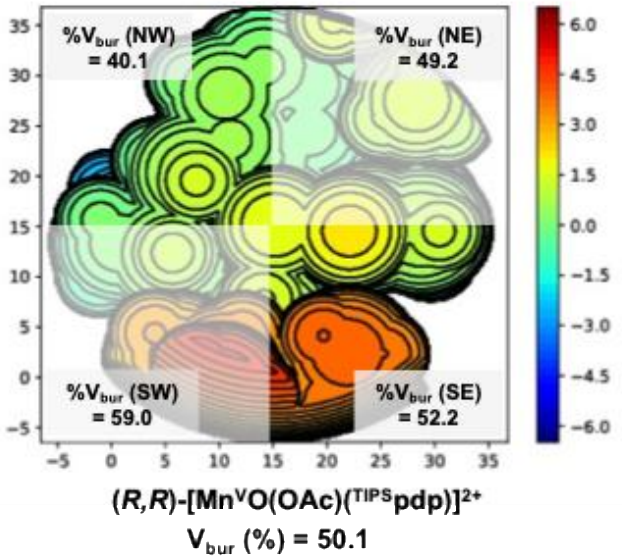

Figure S3. Calculated steric map of (*R*,*R*)-[Mn^V**O**(**O**Ac)(^{TIPS}pdp)]$^{2+}$ (**C1**)

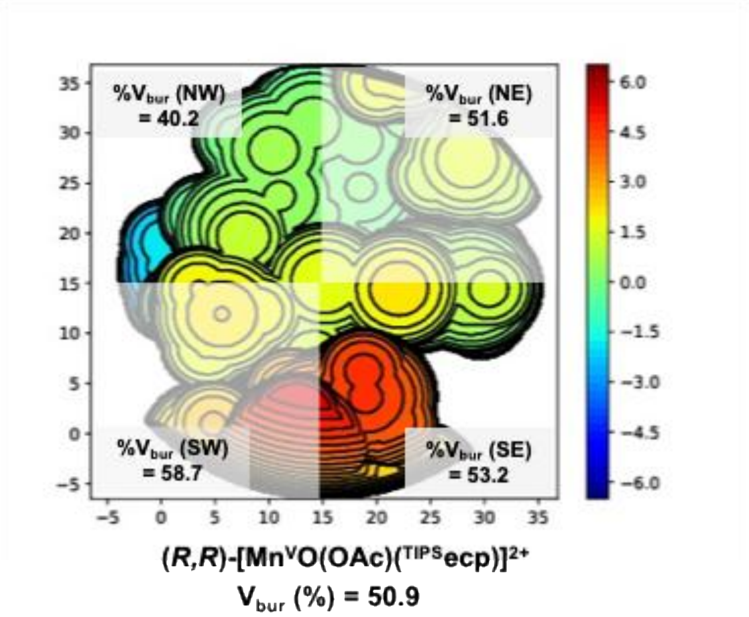

Figure S4. Calculated steric map of (*R*,*R*)-[Mn^{V}**O**(**O**Ac)(^{TIPS}ecp)]$^{2+}$ (**C2**)

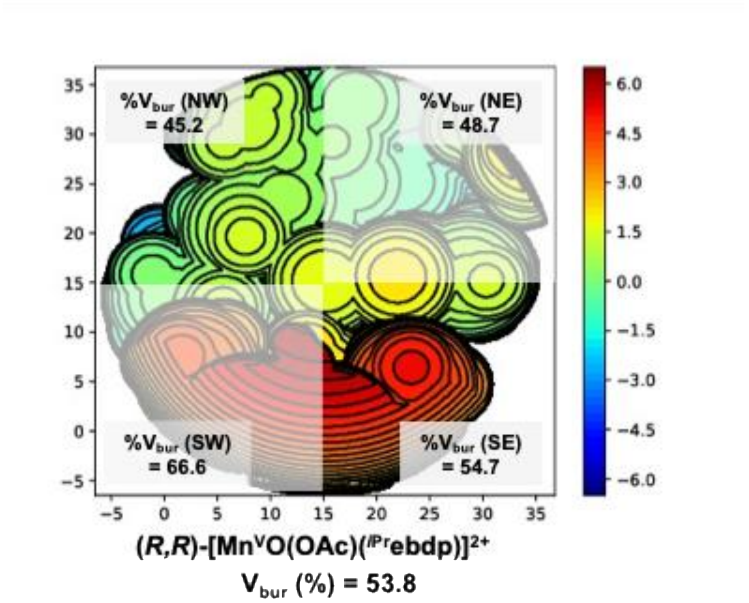

Figure S5. Calculated steric map of (*R*,*R*)-[Mn^{V}**O**(**O**Ac)(^{iPr}ebdp)]$^{2+}$ (**C4**)

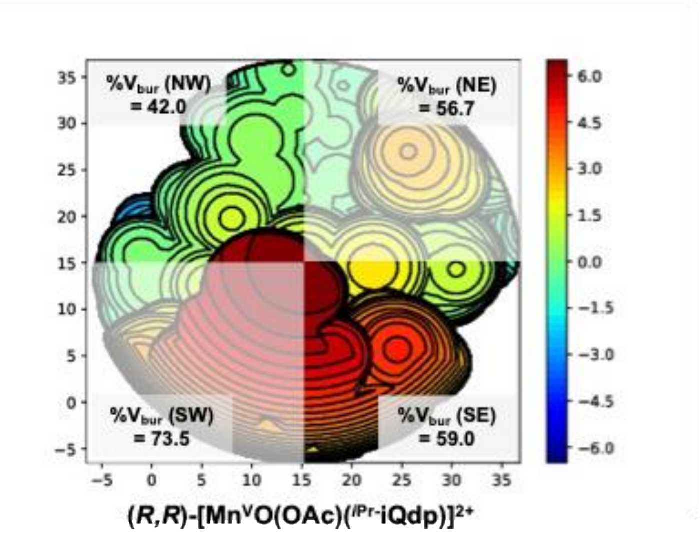

Figure S6. Calculated steric map of (*R*,*R*)-[Mn^{V}**O**(**O**Ac)(^{iPr}iQdp)]$^{2+}$ (**C6**)

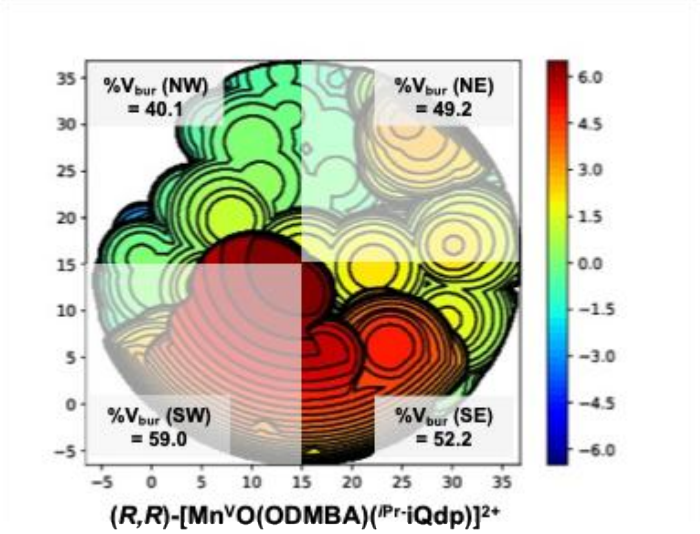

Figure S7. Calculated steric map of (*R*,*R*)-[Mn^{V}**O**(**O**DMB)(^{iPr}iQdp)]$^{2+}$ (**C6**)

10.2. Computational details for DFT calculations

The optimization of reaction intermediates and transition states geometries were performed by using the Gaussian program,[55] at the DFT (B3LYP) level using the LanL2DZ basis set and the pseudopotential for Mn, with 6-31+G(d,p) for the other atoms, incorporating dispersion corrections using the Grimme algorithm GD3BJ.[56, 57] Single-point electronic energy corrections of reaction intermediates and transition states were performed by increasing the basis set quality to triple-ζ Def2-TZVPP[58] and adding solvent (acetonitrile) corrections through the Solvation Model based on Density (SMD),[59] including GD3BJ dispersion corrections. All stationary points in the PES were characterized by means of analytical vibrational frequency calculations and connected through IRC calculations. Therefore, the whole methodology of the study can be denoted as B3LYP-GD3BJ/def2-TZVPP/SMD(MeCN)//B3LYP-GB3BJ/6-31G(d,p)(Mn-LANL2DZ).

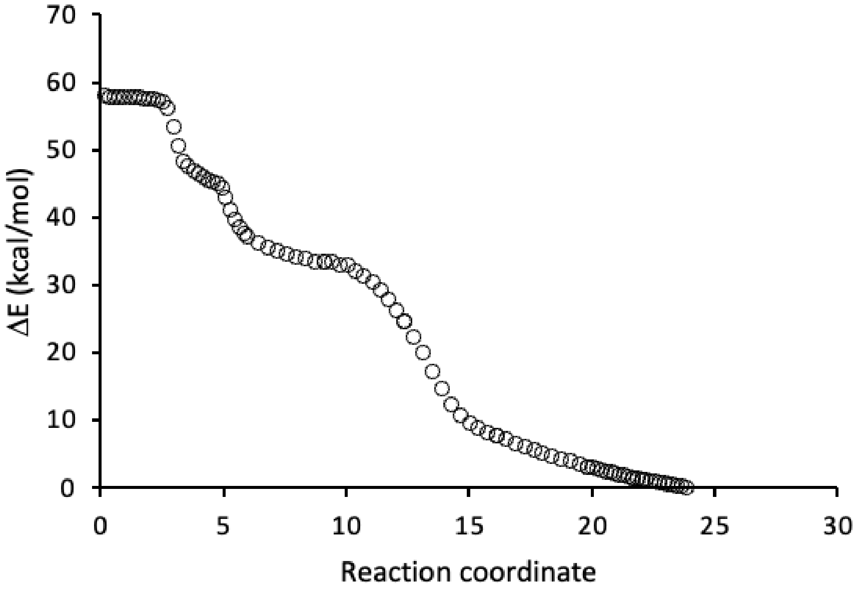

Figure S8. Intrinsic Reaction Coordinate (IRC) plot computed at the B3LYP-GB3BJ/6-31G(d,p)(Mn-LANL2DZ) level of theory, illustrating the reaction pathway from the transition state **TS^{major}-(*R*)** to the hydroxylated product. The energy profile (in kcal·mol^{-1}) plotted along the reaction coordinate confirms that **TS^{major}-(*R*)** is directly connected to the hydroxylated product, indicating a barrierless rebound step following hydrogen atom transfer.

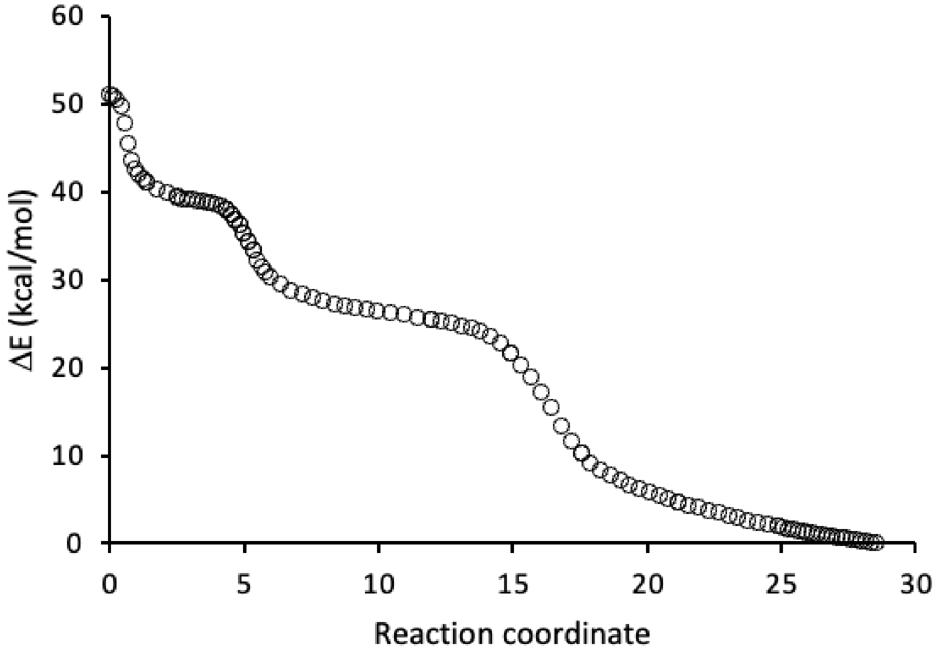

Figure S9. Intrinsic Reaction Coordinate (IRC) plot computed at the B3LYP-GB3BJ/6-31G(d,p)(Mn-LANL2DZ) level of theory, illustrating the reaction pathway from the transition state **TS^{minor}-(*S*)** to the hydroxylated product. The energy profile (in kcal·mol^{-1}) plotted along the reaction coordinate confirms that **TS^{minor}-(*S*)** is directly connected to the hydroxylated product, indicating a barrierless rebound step following hydrogen atom transfer.

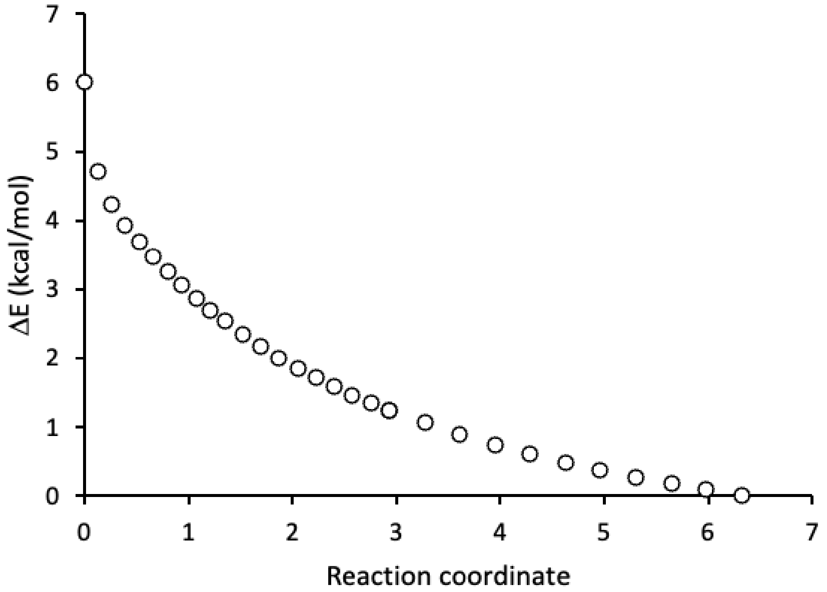

Figure S10. Intrinsic Reaction Coordinate (IRC) plot computed at the B3LYP-GB3BJ/6-31G(d,p)(Mn-LANL2DZ) level of theory, illustrating the reaction pathway from the transition state **TS^{minor}-(*S*)** to the reactant species. In contrast, **TS^{minor}-(*R*)** could not be directly connected to the reactant by IRC calculations due to the very small energy difference between the two structures (0.49 kcal·mol^{-1}); however, connectivity was confirmed through geometry optimization.

10.3. Atomic coordinates of DFT-calculated structures

Geometry optimizations for all reported structures were performed in the triplet spin state.

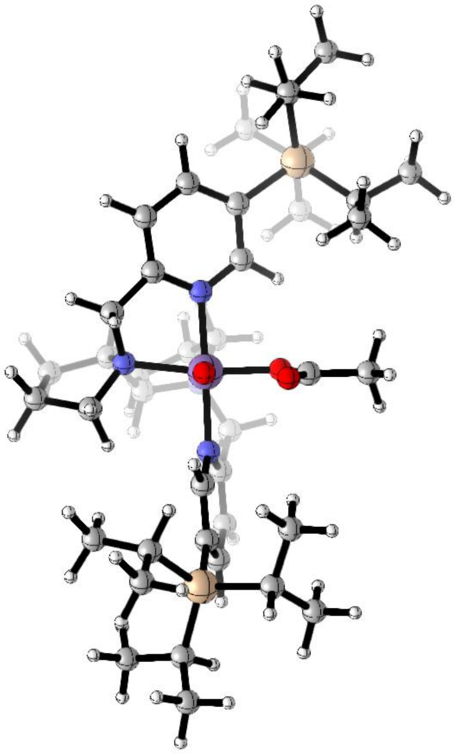

(*R*,*R*)-[Mn^{V}O(OAc)(^{TIPS}pdp)]$^{2+}$ (**C1**)

Mn	-0.034254000	0.378802000	0.077417000
Si	5.654612000	-0.961390000	0.071679000
N	1.845206000	0.551318000	0.784592000
O	-0.509236000	-0.618318000	1.323222000
C	2.919513000	-0.078624000	0.279922000
H	2.739360000	-0.635963000	-0.627790000
Si	-5.644916000	-1.140990000	0.174646000
N	-0.256839000	2.072949000	1.236233000
C	4.177471000	-0.029690000	0.876697000
N	0.229545000	1.809015000	-1.501456000
C	4.272677000	0.735045000	2.047526000
H	5.220873000	0.818666000	2.565222000
N	-1.867851000	0.357209000	-0.739479000
O	0.472651000	-1.033494000	-1.063317000
C	3.160397000	1.393452000	2.570572000
H	3.233261000	1.982071000	3.476799000
C	1.943965000	1.269871000	1.920962000
C	0.648233000	1.842874000	2.408316000
H	0.801738000	2.767202000	2.967806000
H	0.156678000	1.117294000	3.060447000
C	-1.645527000	2.404758000	1.702546000

H	-1.946704000	1.705788000	2.479871000
H	-2.318760000	2.282432000	0.857130000
C	-1.565607000	3.871667000	2.128957000
H	-2.539853000	4.353779000	2.060221000
H	-1.239625000	3.958767000	3.166031000
C	-0.525561000	4.496032000	1.163710000
H	-1.006570000	5.129110000	0.417112000
H	0.187934000	5.123929000	1.695527000
C	0.178803000	3.301974000	0.471056000
H	1.264583000	3.364766000	0.542843000
C	-0.215899000	3.156856000	-0.996535000
H	-1.302394000	3.202719000	-1.077202000
C	0.479379000	4.209741000	-1.899179000
H	-0.240306000	4.731847000	-2.528147000
H	0.968248000	4.967917000	-1.285648000
C	1.509473000	3.418456000	-2.742913000
H	2.479502000	3.912061000	-2.787415000
H	1.164653000	3.300326000	-3.771006000
C	1.603925000	2.062786000	-2.038582000
H	2.289544000	2.114594000	-1.194969000
H	1.912097000	1.233573000	-2.672507000
C	-0.678592000	1.341913000	-2.587127000
H	-0.858484000	2.124102000	-3.327953000
H	-0.183678000	0.506273000	-3.087264000
C	-1.966825000	0.859004000	-1.988976000
C	-3.183699000	0.852487000	-2.649167000
H	-3.259585000	1.269316000	-3.646026000
C	-4.293925000	0.294833000	-2.015117000
H	-5.244956000	0.291120000	-2.534578000
C	-4.195038000	-0.256534000	-0.730166000
C	-2.936748000	-0.196849000	-0.135362000
H	-2.740082000	-0.596153000	0.849688000
C	4.891778000	-2.498213000	-0.751975000
H	4.218458000	-2.092815000	-1.522611000
C	4.051453000	-3.382757000	0.185376000
H	3.569392000	-4.189391000	-0.376502000
H	4.671552000	-3.857179000	0.947262000
H	3.268533000	-2.826402000	0.709719000
C	5.954735000	-3.336160000	-1.487361000
H	6.639302000	-3.811626000	-0.781335000
H	5.483586000	-4.135479000	-2.066991000
H	6.552880000	-2.737778000	-2.178914000
C	6.930193000	-1.252617000	1.439695000
H	7.089678000	-0.257978000	1.880162000
C	8.292421000	-1.713542000	0.886436000
H	8.687659000	-1.032301000	0.129416000
H	9.028993000	-1.769463000	1.692693000
H	8.227493000	-2.707729000	0.438754000

C	6.446831000	-2.188040000	2.561811000
H	6.385578000	-3.221370000	2.213195000
H	7.148839000	-2.174231000	3.400209000
H	5.461890000	-1.913123000	2.951243000
C	6.342792000	0.222655000	-1.247225000
H	7.178293000	-0.313408000	-1.714065000
C	5.324148000	0.549464000	-2.350717000
H	5.772736000	1.194580000	-3.112088000
H	4.955751000	-0.345851000	-2.858027000
H	4.461762000	1.087935000	-1.940992000
C	6.900353000	1.512233000	-0.621347000
H	6.111955000	2.085660000	-0.120534000
H	7.683605000	1.312484000	0.112894000
H	7.332919000	2.160417000	-1.389116000
C	-5.351816000	-0.828416000	2.026344000
H	-4.379831000	-1.299308000	2.230830000
C	-6.388708000	-1.551955000	2.905667000
H	-6.463461000	-2.615678000	2.667641000
H	-6.121095000	-1.470866000	3.963224000
H	-7.382698000	-1.113958000	2.788668000
C	-5.240959000	0.654535000	2.420235000
H	-6.204694000	1.160575000	2.342406000
H	-4.908815000	0.752618000	3.458895000
H	-4.536330000	1.205171000	1.788344000
C	-7.245947000	-0.465866000	-0.580029000
H	-7.123167000	-0.612262000	-1.662624000
C	-8.481312000	-1.286063000	-0.161775000
H	-8.367093000	-2.350143000	-0.380702000
H	-8.685637000	-1.185944000	0.906668000
H	-9.368998000	-0.935366000	-0.695617000
C	-7.473853000	1.035623000	-0.334841000
H	-8.314710000	1.399078000	-0.932476000
H	-6.600621000	1.644772000	-0.589529000
C	-5.425351000	-2.983245000	-0.232252000
H	-6.253215000	-3.493331000	0.276186000
C	-5.560339000	-3.255657000	-1.739962000
H	-4.765291000	-2.759295000	-2.307841000
H	-5.481905000	-4.326903000	-1.946912000
H	-6.518545000	-2.917191000	-2.140774000
C	-4.110813000	-3.561976000	0.315988000
H	-3.237266000	-3.079420000	-0.136154000
H	-4.025865000	-3.453924000	1.399702000
H	-4.038748000	-4.629965000	0.089928000
C	0.232853000	-2.262712000	-0.660924000
O	-0.278513000	-2.530377000	0.425005000
H	-7.716877000	1.230861000	0.711932000
C	0.629640000	-3.348438000	-1.622054000
H	0.430303000	-4.327423000	-1.193530000

H	1.691960000	-3.246562000	-1.852263000
H	0.068143000	-3.222137000	-2.550426000

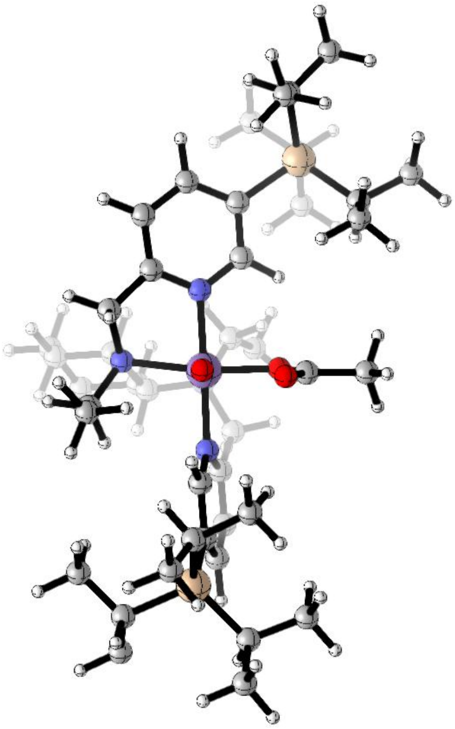

(*R*,*R*)-[Mn^{V}**O**(**O**Ac)(^{TIPS}ecp)]$^{2+}$ (**C2**)

Mn	-0.034190000	0.271100000	0.125110000
C	0.203030000	-2.285740000	-0.904490000
O	-0.297930000	-2.672650000	0.148300000
H	1.943740000	0.710380000	-1.894490000
C	0.582550000	-3.253480000	-1.991240000
H	0.385850000	-4.274990000	-1.675650000
H	1.641680000	-3.128420000	-2.224700000
H	0.008250000	-3.020550000	-2.890700000
Si	5.621780000	-1.242549000	0.041040000
O	-0.485320000	-0.869830000	1.254480000
N	1.857810000	0.337580000	0.848780000
C	2.909370000	-0.311320000	0.320210000
H	2.712320000	-0.829850000	-0.605550000
Si	-5.710330000	-1.159741000	0.132390000
N	-0.219760000	1.887970000	1.446680000
C	4.170060000	-0.331869000	0.910230000
N	0.246970000	1.809430000	-1.390360000
C	4.296830000	0.376611000	2.112980000

H	5.249720000	0.407631000	2.627940000
N	-1.885070000	0.329000000	-0.670830000
O	0.449350000	-1.020160000	-1.165780000
C	3.206990000	1.041070000	2.671890000
H	3.298650000	1.579290000	3.607220000
C	1.985350000	0.990320000	2.021300000
C	6.392950000	0.052891000	-1.118810000
H	7.198980000	-0.468579000	-1.649330000
C	7.019460000	1.222071000	-0.340960000
H	6.262840000	1.774241000	0.227990000
H	7.787010000	0.888861000	0.360540000
C	5.398080000	0.570641000	-2.170120000
H	4.976120000	-0.233389000	-2.778980000
H	4.570240000	1.106021000	-1.691430000
C	6.843080000	-1.744009000	1.397250000
H	7.059150000	-0.804349000	1.925470000
C	8.182070000	-2.249479000	0.827180000
H	8.900180000	-2.414449000	1.635170000
H	8.060470000	-3.200939000	0.304440000
H	8.632940000	-1.540569000	0.128640000
C	6.271420000	-2.734379000	2.426480000
H	6.955430000	-2.843309000	3.272780000
H	5.301520000	-2.420869000	2.824220000
H	6.142640000	-3.726879000	1.989420000
C	4.818510000	-2.635029000	-0.977450000
H	4.151540000	-2.112589000	-1.680540000
C	3.961880000	-3.617330000	-0.159640000
H	3.447680000	-4.322860000	-0.820360000
H	4.577690000	-4.211879000	0.516810000
H	3.203930000	-3.118430000	0.452020000
C	5.852950000	-3.391629000	-1.831940000
H	5.356130000	-4.097559000	-2.504110000
H	6.455700000	-2.720209000	-2.448150000
H	6.535570000	-3.970379000	-1.205880000
C	0.710300000	1.550970000	2.568360000
H	0.237980000	0.779380000	3.175030000
H	0.883020000	2.424090000	3.200830000
C	-1.627780000	2.094390000	1.965180000
H	-2.270690000	2.177030000	1.090930000
H	-1.650961000	3.053380000	2.478300000
C	-2.151140000	1.035580000	2.929090000
H	-3.231510000	1.164170000	3.008610000
H	-1.952130000	0.017290000	2.601850000
H	-1.740620000	1.161560000	3.931670000
C	0.313809000	3.120840000	0.711790000
H	1.398809000	2.995020000	0.708570000
C	-0.000621000	4.449680000	1.417170000
H	0.382429000	4.431620000	2.440300000

H	-1.080211000	4.599000000	1.477690000
C	0.576199000	5.638160000	0.643600000
H	1.668819000	5.570640000	0.590300000
H	0.344179000	6.559660000	1.180740000
C	-0.016981000	5.662790000	-0.763140000
H	0.370169000	6.508490000	-1.334640000
C	0.326989000	4.364540000	-1.496650000
H	1.412829000	4.322390000	-1.604300000
C	-0.156691000	3.115580000	-0.743050000
H	-1.250351000	3.118410000	-0.745030000
C	1.660290000	1.759460000	-1.914210000
H	2.288380000	2.273960000	-1.188440000
C	1.928910000	2.278010000	-3.327770000
H	2.999120000	2.167240000	-3.515800000
H	1.413570000	1.687170000	-4.086100000
C	-0.716000000	1.442510000	-2.459060000
H	-0.890930000	2.272290000	-3.146720000
H	-0.275630000	0.623410000	-3.030930000
C	-1.998810000	0.948260000	-1.864230000
C	-3.222880000	1.008940000	-2.510230000
C	-4.325930000	0.389449000	-1.927790000
H	-5.278810000	0.426419000	-2.442510000
C	-4.212990000	-0.289961000	-0.705300000
C	-2.950610000	-0.288350000	-0.120200000
H	-2.746390000	-0.802570000	0.805560000
C	-6.641830000	-2.032861000	-1.269410000
H	-6.984910000	-1.215101000	-1.918700000
C	-7.900090000	-2.759221000	-0.758120000
H	-8.494240000	-3.131471000	-1.597360000
H	-7.636440000	-3.621871000	-0.141240000
H	-8.545650000	-2.107871000	-0.163500000
C	-5.774280000	-2.968321000	-2.128930000
H	-6.343970000	-3.332501000	-2.988780000
H	-4.878920000	-2.471751000	-2.514990000
H	-5.448020000	-3.843191000	-1.563430000
C	-4.998300000	-2.249791000	1.514730000
H	-4.343600000	-1.580481000	2.092330000
C	-6.087250000	-2.743681000	2.486670000
H	-5.635560000	-3.297751000	3.314640000
H	-6.664870000	-1.922811000	2.917560000
H	-6.789130000	-3.418251000	1.991550000
C	-4.141810000	-3.427051000	1.015580000
H	-3.637270000	-3.916680000	1.853660000
H	-4.762890000	-4.181371000	0.528060000
H	-3.370270000	-3.124910000	0.301480000
C	-6.762760000	0.235369000	0.881820000
H	-7.594800000	-0.273921000	1.383900000
C	-5.988750000	1.028509000	1.947700000

H	-6.627790000	1.787219000	2.408950000
H	-5.612310000	0.386909000	2.748490000
H	-5.135880000	1.553379000	1.500630000
C	-7.362720000	1.180459000	-0.171250000
H	-8.010250000	1.923959000	0.302650000
H	-6.582210000	1.733969000	-0.705060000
H	-7.966330000	0.649699000	-0.910660000

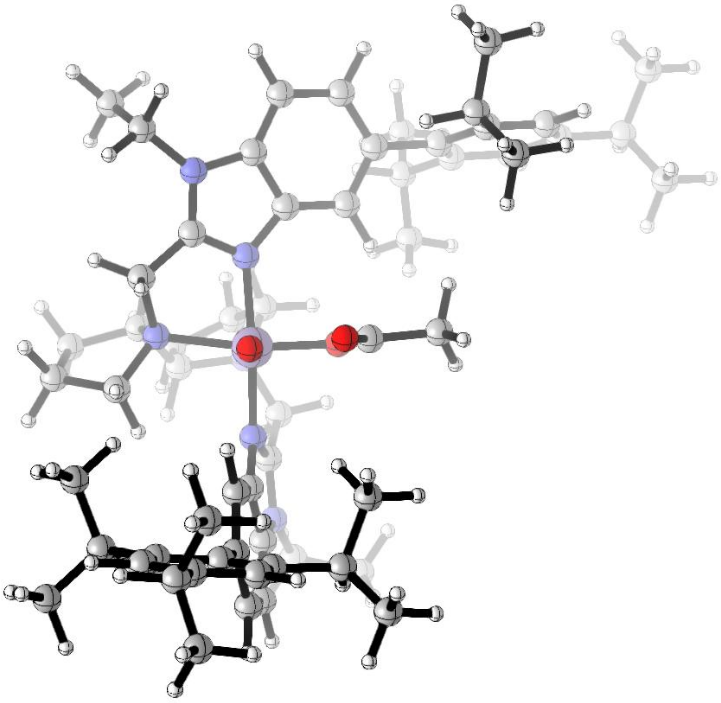

(*R*,*R*)-[Mn^{V}**O**(**O**Ac)(^{iPr}ebdp)]$^{2+}$ (**C4**)

Mn	-0.140426000	-1.087665000	0.010436000
O	-1.039066000	-0.079626000	-0.943138000
O	0.822176000	0.285723000	0.820543000
N	-1.438924000	-1.105895000	1.470707000
N	-1.945452000	-1.416164000	3.607169000
N	0.730193000	-2.582283000	1.354086000
N	-0.858939000	-2.783907000	-0.988766000
N	1.279314000	-1.350684000	-1.350314000
N	1.773680000	-1.921479000	-3.436436000
C	-2.706349000	-0.553042000	1.668944000
C	-3.579697000	0.090611000	0.793935000
H	-3.306453000	0.270222000	-0.236796000

C	-4.806512000	0.522964000	1.299332000
C	-5.129313000	0.301056000	2.662167000
H	-6.092969000	0.646552000	3.020405000
C	-4.261919000	-0.331602000	3.546525000
H	-4.530388000	-0.482701000	4.586008000
C	-3.039116000	-0.753907000	3.023294000
C	-1.017928000	-1.585785000	2.646705000
C	0.355850000	-2.156391000	2.734770000
H	0.419732000	-2.980901000	3.452553000
H	1.060026000	-1.378922000	3.047779000
C	2.197471000	-2.863688000	1.266153000
H	2.466562000	-2.877647000	0.210154000
H	2.761051000	-2.061150000	1.740566000
C	2.372717000	-4.244934000	1.905260000
H	3.264352000	-4.750465000	1.530597000
H	2.486919000	-4.160398000	2.989288000
C	1.066665000	-4.997657000	1.542832000
H	0.659011000	-5.536920000	2.399450000
H	1.240250000	-5.740147000	0.759859000
C	0.082407000	-3.904803000	1.038010000
H	-0.876832000	-3.941370000	1.559915000
C	-0.175112000	-4.025993000	-0.465865000
H	0.784290000	-4.112879000	-0.980026000
C	-1.134728000	-5.200456000	-0.800928000
H	-0.720087000	-5.843948000	-1.578673000
H	-1.279424000	-5.828857000	0.081113000
C	-2.465963000	-4.543393000	-1.251759000
H	-3.338156000	-5.003017000	-0.784119000
H	-2.600428000	-4.633188000	-2.332738000
C	-2.324216000	-3.075250000	-0.840541000
H	-2.582471000	-2.926080000	0.207332000
H	-2.905223000	-2.367267000	-1.430348000
C	-0.505016000	-2.577186000	-2.433219000
H	-0.563449000	-3.515553000	-2.992410000
H	-1.226737000	-1.870454000	-2.854180000
C	0.860295000	-1.979893000	-2.448331000
C	2.543774000	-0.830800000	-1.621798000
C	3.428245000	-0.091450000	-0.837712000
H	3.181983000	0.180487000	0.178922000
C	4.647752000	0.288249000	-1.398896000
C	4.960772000	-0.097299000	-2.727435000
H	5.920088000	0.202600000	-3.133915000
C	4.091307000	-0.839787000	-3.518738000
H	4.358314000	-1.123383000	-4.530553000
C	2.870753000	-1.197748000	-2.943618000
C	-5.766996000	1.220486000	0.393719000
C	-6.565810000	0.467814000	-0.488049000
C	-7.431668000	1.146981000	-1.351705000

H	-8.056126000	0.579127000	-2.035302000
C	-7.525276000	2.538187000	-1.360650000
C	-6.719551000	3.260418000	-0.473144000
H	-6.781722000	4.342803000	-0.472512000
C	-5.839771000	2.631636000	0.407909000
C	-1.819000000	-1.735340000	5.037435000
H	-2.786975000	-2.124658000	5.362164000
H	-1.095832000	-2.548535000	5.134650000
C	-1.402985000	-0.517196000	5.859105000
H	-0.420790000	-0.149243000	5.548512000
H	-2.126055000	0.294743000	5.750216000
H	-1.348602000	-0.787784000	6.916438000
C	5.606204000	1.099309000	-0.592308000
C	6.281038000	0.513908000	0.500392000
C	7.161123000	1.303750000	1.246539000
H	7.690810000	0.861057000	2.085446000
C	7.397088000	2.644681000	0.942411000
C	6.714205000	3.199606000	-0.143493000
H	6.883006000	4.242195000	-0.393555000
C	5.819019000	2.458842000	-0.917090000
C	1.694735000	-2.559102000	-4.759406000
H	0.637567000	-2.667515000	-5.012939000
C	0.614749000	1.508089000	0.380555000
O	-0.203757000	1.786575000	-0.505217000
C	1.472695000	2.578663000	0.999815000
H	1.529396000	2.429090000	2.080045000
H	1.076855000	3.565941000	0.765431000
H	2.483775000	2.487778000	0.587912000
C	-4.923476000	3.455323000	1.306235000
C	-6.488047000	-1.053342000	-0.540233000
C	6.096081000	-0.949114000	0.887646000
C	5.098040000	3.144922000	-2.072050000
C	-7.869055000	-1.721967000	-0.457891000
H	-8.487877000	-1.483575000	-1.327621000
H	-8.409700000	-1.396331000	0.434996000
H	-7.765986000	-2.811319000	-0.417782000
C	-5.729004000	-1.509416000	-1.800111000
H	-4.737617000	-1.043825000	-1.851104000
H	-6.267688000	-1.218988000	-2.707337000
H	-5.612624000	-2.599971000	-1.815347000
C	-5.569873000	4.748435000	1.822255000
H	-6.539931000	4.552758000	2.287930000
H	-5.721540000	5.477089000	1.020222000
H	-4.921779000	5.220755000	2.566605000
C	-3.600112000	3.761068000	0.577257000
H	-2.931645000	4.344423000	1.220039000
H	-3.789984000	4.343325000	-0.330092000
H	-3.078505000	2.845172000	0.281223000

C	4.319075000	4.385952000	-1.603598000
H	4.987771000	5.161774000	-1.219691000
H	3.612925000	4.135534000	-0.804757000
H	3.753912000	4.818724000	-2.434719000
C	6.078534000	3.506865000	-3.201060000
H	6.606751000	2.622225000	-3.570033000
H	6.833946000	4.217659000	-2.852300000
H	5.549090000	3.965484000	-4.041987000
C	5.385113000	-1.071445000	2.247403000
H	4.425223000	-0.540138000	2.242850000
H	5.988438000	-0.635486000	3.049254000
H	5.207010000	-2.122758000	2.503152000
C	7.428549000	-1.716691000	0.892624000
H	8.115249000	-1.327759000	1.649812000
H	7.926386000	-1.640818000	-0.077919000
H	7.262076000	-2.776857000	1.110745000
H	-5.914500000	-1.392288000	0.328911000
H	-4.678447000	2.847190000	2.184356000
H	4.364458000	2.445516000	-2.484688000
H	5.456183000	-1.423519000	0.137418000
C	-8.476971000	3.249144000	-2.306256000
H	-8.986238000	2.476035000	-2.895168000
C	-9.551139000	4.034459000	-1.534711000
H	-10.106993000	3.382448000	-0.854686000
H	-10.262591000	4.493024000	-2.228019000
H	-9.102673000	4.836847000	-0.939439000
C	-7.718835000	4.161072000	-3.285781000
H	-6.968620000	3.599791000	-3.851154000
H	-7.204733000	4.969866000	-2.755743000
H	-8.412058000	4.619551000	-3.997385000
C	8.367043000	3.473621000	1.765008000
H	8.769553000	2.819232000	2.548309000
C	9.548278000	3.964535000	0.910846000
H	10.069491000	3.127965000	0.436651000
H	10.267163000	4.509999000	1.529350000
H	9.209709000	4.641764000	0.119637000
C	7.654836000	4.649096000	2.455675000
H	6.828748000	4.300047000	3.083016000
H	7.246462000	5.350916000	1.720750000
H	8.354676000	5.202568000	3.088721000
C	2.422684000	-3.900701000	-4.792614000
H	2.364906000	-4.328417000	-5.796497000
H	3.477359000	-3.782586000	-4.531791000
H	1.972021000	-4.611156000	-4.092485000
H	2.121720000	-1.854535000	-5.477000000

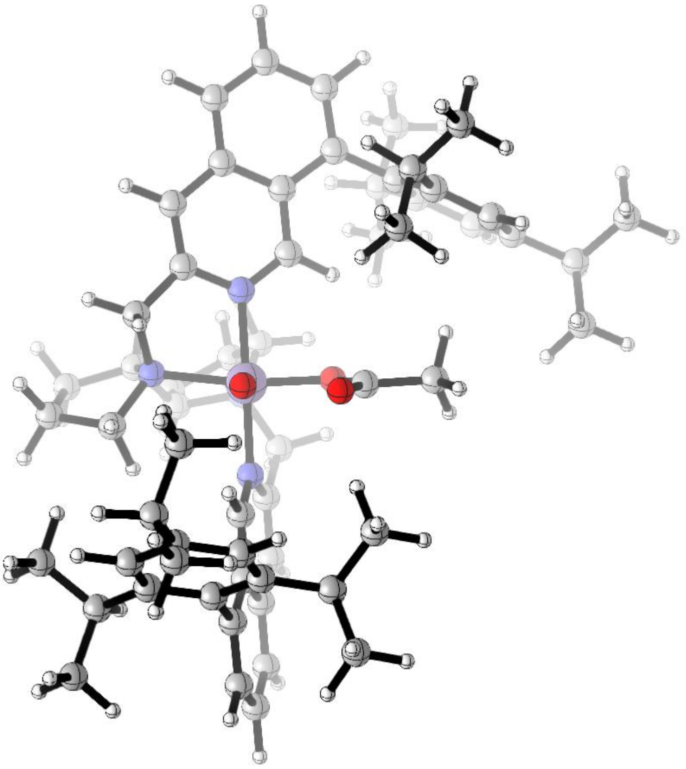

(*R*,*R*)-[Mn^{V}**O**(**O**Ac)(^{iPr}iQdp)]$^{2+}$ (**C6**)

N	-1.544029000	-1.168517000	1.229295000
N	0.696440000	-2.572722000	1.091814000
N	-0.601981000	-2.454622000	-1.375962000
N	1.531145000	-0.865423000	-1.325626000
C	-3.800859000	-0.803615000	1.940872000
C	-2.737956000	-0.624791000	1.036874000
H	-2.876858000	-0.038894000	0.137438000
C	-5.064830000	-0.174680000	1.716189000
C	-6.075500000	-0.421758000	2.626551000
H	-7.040621000	0.053586000	2.487247000
C	-5.869135000	-1.260186000	3.749263000
H	-6.689397000	-1.423873000	4.441084000
C	-4.645336000	-1.855928000	3.988094000
H	-4.493436000	-2.482262000	4.861168000
C	-3.579505000	-1.634398000	3.087784000
C	-2.279348000	-2.186242000	3.262420000
H	-2.067150000	-2.803027000	4.130201000
C	-1.290425000	-1.922292000	2.351338000
C	0.140532000	-2.351860000	2.468924000
H	0.731841000	-1.552350000	2.925445000
H	0.241484000	-3.254382000	3.077510000
C	2.181176000	-2.784736000	1.153108000
H	2.629000000	-2.029708000	1.798841000
H	2.581121000	-2.653420000	0.148193000

C	2.342521000	-4.235022000	1.616395000
H	2.316868000	-4.302492000	2.706904000
H	3.300328000	-4.646559000	1.293727000
C	1.134051000	-4.971631000	0.981019000
H	1.445268000	-5.580406000	0.128077000
H	0.654946000	-5.647087000	1.692068000
C	0.158653000	-3.859514000	0.509462000
H	-0.849488000	-4.001046000	0.905125000
C	-0.760195000	-4.877835000	-1.651204000
H	-0.232453000	-5.345771000	-2.484293000
H	-0.951466000	-5.666220000	-0.918416000
C	0.085569000	-3.742622000	-1.012769000
H	1.100395000	-3.709344000	-1.416449000
C	-2.049962000	-2.825291000	-1.442107000
H	-2.431078000	-2.886167000	-0.422393000
H	-2.605544000	-2.045312000	-1.962196000
C	-2.077914000	-4.202823000	-2.115560000
H	-2.105615000	-4.104882000	-3.203794000
H	-2.963814000	-4.770048000	-1.824046000
C	-0.124530000	-1.964602000	-2.703064000
H	-0.803996000	-1.163705000	-3.008915000
H	-0.160440000	-2.753323000	-3.460749000
C	1.268322000	-1.423601000	-2.556987000
C	2.213333000	-1.414454000	-3.548528000
H	1.991267000	-1.864813000	-4.511131000
C	3.480435000	-0.805049000	-3.329356000
C	4.491855000	-0.746371000	-4.312498000
H	4.318894000	-1.180369000	-5.291983000
C	5.691108000	-0.126503000	-4.014661000
H	6.468562000	-0.072011000	-4.770317000
C	5.933123000	0.438398000	-2.739216000
H	6.887728000	0.912733000	-2.539106000
C	4.979512000	0.397661000	-1.739052000
C	3.726070000	-0.223712000	-2.041190000
C	2.699787000	-0.277507000	-1.081652000
H	2.828130000	0.166410000	-0.101125000
C	-5.232648000	0.750637000	0.554594000
C	-4.889663000	2.112407000	0.706967000
C	-5.028190000	2.963023000	-0.395232000
C	-5.504047000	2.507878000	-1.629970000
C	-5.825630000	1.152432000	-1.752158000
C	-5.695540000	0.256984000	-0.684348000
C	5.218158000	0.958772000	-0.376458000
C	5.789069000	0.139669000	0.621132000
C	5.982944000	0.683428000	1.896006000
C	5.626213000	1.999387000	2.204331000
C	5.056765000	2.783424000	1.195292000
C	4.841124000	2.289859000	-0.093935000

C	-0.630152000	1.636947000	-0.692416000
O	-0.884860000	0.372364000	-0.968077000
O	0.797346000	0.135255000	1.017921000
O	0.179365000	1.990575000	0.173175000
C	-1.417819000	2.638532000	-1.489636000
H	-1.314505000	2.419509000	-2.555413000
H	-2.476415000	2.538297000	-1.225735000
H	-1.078947000	3.650471000	-1.272256000
Mn	0.038224000	-0.933344000	0.008370000
C	-4.375222000	2.671840000	2.029005000
H	-4.456768000	1.885885000	2.785796000
C	-5.691343000	3.458277000	-2.800736000
H	-6.104091000	2.870565000	-3.629986000
C	-6.100751000	-1.203029000	-0.847602000
H	-5.568662000	-1.784555000	-0.084431000
C	6.245551000	-1.281807000	0.311442000
H	5.695433000	-1.627532000	-0.572320000
C	4.265518000	3.196552000	-1.174935000
H	3.920334000	2.562547000	-1.999775000
C	5.857247000	2.560100000	3.596667000
H	6.310269000	1.760123000	4.195781000
H	-4.779602000	4.013576000	-0.279393000
H	-6.201362000	0.793984000	-2.705426000
H	4.777182000	3.807506000	1.418569000
H	6.432825000	0.073474000	2.673447000
C	-5.224745000	3.857154000	2.519880000
H	-4.889812000	4.179199000	3.510746000
H	-5.143629000	4.717604000	1.848238000
H	-6.282236000	3.584727000	2.587325000
C	-2.888055000	3.057338000	1.932975000
H	-2.525538000	3.439402000	2.892364000
H	-2.265160000	2.198176000	1.662379000
H	-2.729646000	3.839861000	1.183313000
C	-6.702190000	4.569635000	-2.465404000
H	-6.328674000	5.218572000	-1.665860000
H	-6.885832000	5.196104000	-3.343662000
H	-7.658254000	4.149728000	-2.139009000
C	-4.352830000	4.053009000	-3.273400000
H	-3.885494000	4.653270000	-2.484284000
H	-3.651039000	3.265053000	-3.567734000
H	-4.507896000	4.706781000	-4.137078000
C	-7.610171000	-1.378445000	-0.588939000
H	-8.191876000	-0.802116000	-1.315616000
H	-7.899486000	-2.430681000	-0.677921000
H	-7.887978000	-1.031956000	0.410058000
C	-5.724155000	-1.789816000	-2.217871000
H	-4.678731000	-1.584169000	-2.472353000
H	-5.876695000	-2.873888000	-2.219157000

H	-6.343309000	-1.378243000	-3.020394000
C	5.955253000	-2.277994000	1.445816000
H	4.910058000	-2.227093000	1.768263000
H	6.168770000	-3.299618000	1.114476000
H	6.578853000	-2.088407000	2.324338000
C	7.744737000	-1.301429000	-0.045445000
H	8.343984000	-0.952502000	0.801751000
H	8.071483000	-2.315005000	-0.300536000
H	7.960934000	-0.648857000	-0.895769000
C	3.054031000	4.012329000	-0.694637000
H	3.338447000	4.755839000	0.056355000
H	2.615321000	4.558495000	-1.536618000
H	2.284209000	3.370292000	-0.256434000
C	5.364537000	4.117435000	-1.738922000
H	4.968678000	4.745427000	-2.543788000
H	5.755995000	4.774870000	-0.955739000
H	6.203967000	3.538903000	-2.137125000
C	4.531933000	2.957886000	4.271099000
H	4.711748000	3.300100000	5.295154000
H	4.041604000	3.773796000	3.728774000
H	3.835967000	2.112945000	4.308716000
C	6.843415000	3.741686000	3.571913000
H	6.433414000	4.587716000	3.009594000
H	7.050371000	4.087813000	4.589382000
H	7.792507000	3.456964000	3.10768200

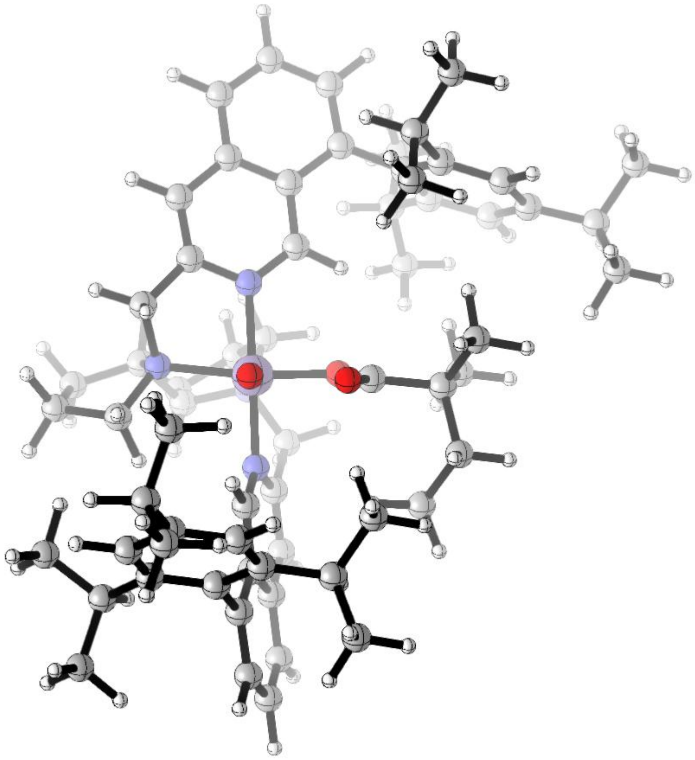

(*R*,*R*)-[Mn^{V}**O**(**O**DMBA)(^{iPr}iQdp)]$^{2+}$ (**C6**)

N	1.395518000	1.556489000	1.116135000
N	-0.929692000	2.827865000	0.823065000
N	0.367119000	2.425483000	-1.615590000
N	-1.679578000	0.773093000	-1.316529000
C	3.704656000	1.502839000	1.747480000
C	2.614825000	1.053322000	0.978531000
H	2.755046000	0.291183000	0.222470000
C	5.006452000	0.939022000	1.581117000
C	6.041885000	1.488240000	2.315838000
H	7.037757000	1.071448000	2.210463000
C	5.826158000	2.552953000	3.221370000
H	6.668047000	2.946532000	3.781765000
C	4.566547000	3.081917000	3.417324000
H	4.403664000	3.883810000	4.129742000
C	3.477651000	2.565725000	2.683037000
C	2.146537000	3.042502000	2.829857000
H	1.928561000	3.819127000	3.555973000
C	1.138203000	2.514417000	2.069739000
C	-0.314006000	2.858501000	2.190564000
H	-0.826417000	2.098123000	2.787519000
H	-0.459628000	3.833609000	2.662284000
C	-2.422536000	2.946450000	0.913111000
H	-2.795408000	2.271675000	1.683679000
H	-2.840236000	2.634344000	-0.043537000
C	-2.673596000	4.435906000	1.154653000
H	-2.594159000	4.678222000	2.217315000
H	-3.675814000	4.724549000	0.834656000
C	-1.561758000	5.138378000	0.335747000
H	-1.957434000	5.537904000	-0.601619000
H	-1.127902000	5.978610000	0.879879000
C	-0.500651000	4.044774000	0.035538000
H	0.492216000	4.326812000	0.392537000
C	0.346460000	4.780144000	-2.261654000
H	-0.203750000	5.060716000	-3.161191000
H	0.461500000	5.689976000	-1.667259000
C	-0.415130000	3.698125000	-1.449899000
H	-1.423643000	3.527160000	-1.833565000
C	1.784033000	2.886644000	-1.735434000
H	2.146973000	3.125073000	-0.735167000
H	2.401949000	2.082977000	-2.133530000
C	1.720243000	4.146967000	-2.606863000
H	1.775833000	3.889753000	-3.667414000
H	2.554540000	4.818622000	-2.397376000
C	-0.062442000	1.708706000	-2.851040000
H	0.664047000	0.909996000	-3.023606000
H	-0.063121000	2.368940000	-3.723707000

C	-1.423106000	1.116309000	-2.624203000
C	-2.343275000	0.872154000	-3.608167000
H	-2.124270000	1.152389000	-4.633649000
C	-3.578237000	0.240480000	-3.297747000
C	-4.559587000	-0.061445000	-4.265420000
H	-4.386311000	0.191927000	-5.306031000
C	-5.728309000	-0.683568000	-3.873228000
H	-6.482453000	-0.924286000	-4.615699000
C	-5.972126000	-1.010693000	-2.518079000
H	-6.904805000	-1.491745000	-2.245067000
C	-5.048321000	-0.726797000	-1.530471000
C	-3.823052000	-0.104007000	-1.927522000
C	-2.820845000	0.175706000	-0.983356000
H	-2.942608000	-0.100297000	0.057307000
C	5.232671000	-0.247826000	0.701763000
C	5.215991000	-1.534692000	1.283548000
C	5.529244000	-2.634476000	0.479518000
C	5.864994000	-2.495663000	-0.870403000
C	5.832136000	-1.214634000	-1.427383000
C	5.514556000	-0.082208000	-0.670630000
C	-5.289144000	-1.030181000	-0.089352000
C	-5.886965000	-0.052680000	0.734462000
C	-6.085610000	-0.354323000	2.084955000
C	-5.706512000	-1.581418000	2.634182000
C	-5.108100000	-2.525207000	1.794437000
C	-4.884941000	-2.274268000	0.439164000
C	0.565455000	-1.521173000	-0.436655000
O	0.766280000	-0.285404000	-0.843674000
O	-0.881282000	0.116448000	1.131971000
O	-0.150324000	-1.794553000	0.534198000
C	1.230320000	-2.633493000	-1.260379000
Mn	-0.189656000	1.080010000	-0.019968000
C	4.875688000	-1.750917000	2.753685000
H	4.732373000	-0.771288000	3.218495000
C	6.305875000	-3.689422000	-1.699949000
H	6.465013000	-3.330896000	-2.724512000
C	5.540617000	1.300886000	-1.309031000
H	4.899772000	1.958247000	-0.707970000
C	-6.364895000	1.276500000	0.161582000
H	-5.784636000	1.480181000	-0.746811000
C	-4.265498000	-3.348136000	-0.445931000
H	-3.930209000	-2.870798000	-1.373795000
C	-5.936406000	-1.879082000	4.105051000
H	-6.430139000	-1.001370000	4.540454000
H	5.536691000	-3.623335000	0.927154000
H	6.083099000	-1.100753000	-2.477145000
H	-4.810192000	-3.482814000	2.207612000
H	-6.556310000	0.379219000	2.731964000

C	6.024297000	-2.443295000	3.505690000
H	5.786716000	-2.529075000	4.570110000
H	6.203282000	-3.452894000	3.123819000
H	6.957112000	-1.880404000	3.407104000
C	3.555373000	-2.523344000	2.916345000
H	3.303467000	-2.631294000	3.975614000
H	2.724963000	-2.006782000	2.421970000
H	3.626128000	-3.528207000	2.488414000
C	7.647477000	-4.238636000	-1.181624000
H	7.544682000	-4.630395000	-0.164266000
H	8.000450000	-5.053648000	-1.820220000
H	8.414298000	-3.459111000	-1.165340000
C	5.242633000	-4.797316000	-1.752997000
H	5.025387000	-5.192734000	-0.755222000
H	4.306413000	-4.428923000	-2.182251000
H	5.592028000	-5.630900000	-2.368862000
C	6.962470000	1.890691000	-1.274008000
H	7.653351000	1.254907000	-1.835943000
H	6.981718000	2.890879000	-1.718424000
H	7.335591000	1.966170000	-0.249350000
C	4.989614000	1.307658000	-2.743808000
H	4.028822000	0.784409000	-2.810730000
H	4.857797000	2.335941000	-3.095957000
H	5.673218000	0.818326000	-3.443067000
C	-6.151535000	2.463124000	1.113604000
H	-5.125525000	2.495923000	1.493908000
H	-6.363048000	3.404678000	0.596895000
H	-6.818955000	2.416417000	1.978597000
C	-7.845568000	1.182432000	-0.251418000
H	-8.470360000	0.955962000	0.617954000
H	-8.191202000	2.126669000	-0.683890000
H	-8.003659000	0.391462000	-0.989118000
C	-3.034977000	-4.010378000	0.191762000
H	-3.297697000	-4.577739000	1.089211000
H	-2.587489000	-4.718833000	-0.512797000
H	-2.279010000	-3.270738000	0.471341000
C	-5.324025000	-4.398489000	-0.827957000
H	-4.897415000	-5.157088000	-1.491637000
H	-5.705943000	-4.904084000	0.064405000
H	-6.175574000	-3.937896000	-1.337563000
C	-4.604276000	-2.084699000	4.846730000
H	-4.780062000	-2.244092000	5.914753000
H	-4.069772000	-2.960086000	4.462997000
H	-3.948701000	-1.214967000	4.733439000
C	-6.870744000	-3.085663000	4.296629000
H	-6.420377000	-4.002456000	3.901966000
H	-7.073942000	-3.246959000	5.359379000
H	-7.825212000	-2.932953000	3.785048000

C	0.079691000	-3.398789000	-1.976954000
H	-0.482703000	-3.953109000	-1.221189000
H	0.561590000	-4.140073000	-2.623610000
C	1.949676000	-3.592412000	-0.293480000
H	2.313656000	-4.456290000	-0.854529000
H	2.812469000	-3.110522000	0.170604000
H	1.274634000	-3.944344000	0.489027000
C	2.229406000	-2.046370000	-2.266975000
H	2.670572000	-2.866731000	-2.839372000
H	1.755118000	-1.362030000	-2.973716000
H	3.046595000	-1.520998000	-1.766923000
C	-0.887240000	-2.541576000	-2.795625000
H	-1.610140000	-3.177893000	-3.312782000
H	-1.457914000	-1.864107000	-2.154023000
H	-0.373064000	-1.944783000	-3.554951000

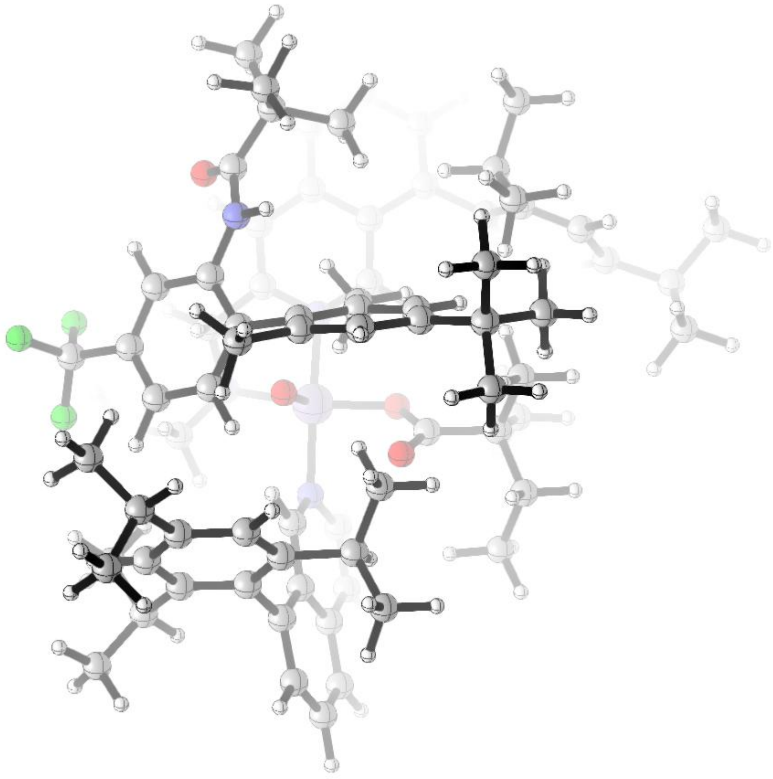

TS^{major}-(*R*)

N	-1.518781000	-1.339145000	1.535681000
N	0.686414000	-2.687785000	2.160468000
N	-0.721403000	-4.023696000	0.183281000
N	1.361760000	-2.735255000	-0.887995000
C	-3.692055000	-0.451499000	2.017383000
C	-2.714846000	-0.939741000	1.125579000
H	-2.911854000	-1.012986000	0.063328000

C	-4.994374000	-0.059532000	1.569448000
C	-5.893814000	0.378099000	2.525179000
H	-6.894505000	0.653090000	2.212315000
C	-5.550398000	0.467083000	3.894555000
H	-6.291526000	0.824660000	4.602076000
C	-4.299058000	0.099031000	4.336742000
H	-4.033370000	0.160725000	5.386576000
C	-3.347176000	-0.379042000	3.407230000
C	-2.051016000	-0.801091000	3.798143000
H	-1.751483000	-0.726810000	4.836637000
C	-1.168535000	-1.267895000	2.861962000
C	0.248193000	-1.657928000	3.147789000
H	0.887274000	-0.787629000	3.003737000
H	0.369831000	-2.011305000	4.174516000
C	2.165424000	-2.899599000	2.223707000
H	2.669399000	-1.938244000	2.256537000
H	2.465147000	-3.407673000	1.306757000
C	2.381224000	-3.791553000	3.449165000
H	2.491539000	-3.182220000	4.347440000
H	3.290302000	-4.387096000	3.349240000
C	1.100738000	-4.666098000	3.514830000
H	1.306146000	-5.704108000	3.241435000
H	0.679401000	-4.682485000	4.521529000
C	0.110585000	-4.032410000	2.501311000
H	-0.878466000	-3.870460000	2.936338000
C	-0.929008000	-6.110536000	1.427884000
H	-0.463881000	-7.004099000	1.008043000
H	-1.073432000	-6.302762000	2.494060000
C	-0.035283000	-4.857150000	1.222238000
H	0.958557000	-5.115415000	0.847752000
C	-2.172080000	-4.279046000	0.418296000
H	-2.484164000	-3.688486000	1.280393000
H	-2.746369000	-3.938978000	-0.441253000
C	-2.269857000	-5.779567000	0.720412000
H	-2.375250000	-6.354765000	-0.202988000
H	-3.138191000	-6.008376000	1.340868000
C	-0.327735000	-4.455982000	-1.181337000
H	-1.070357000	-4.045771000	-1.870190000
H	-0.335157000	-5.546344000	-1.284236000
C	1.018311000	-3.893512000	-1.541326000
C	1.811299000	-4.402903000	-2.534199000
H	1.523553000	-5.318796000	-3.040498000
C	2.979192000	-3.708263000	-2.945478000
C	3.797744000	-4.151950000	-4.007459000
H	3.551342000	-5.073040000	-4.525301000
C	4.891872000	-3.400433000	-4.379381000
H	5.519957000	-3.730827000	-5.200465000
C	5.219713000	-2.203583000	-3.701352000

H	6.098853000	-1.643950000	-4.002303000
C	4.460114000	-1.735686000	-2.644597000
C	3.308693000	-2.491101000	-2.263420000
C	2.450575000	-2.057254000	-1.235974000
H	2.625793000	-1.131331000	-0.702640000
C	-5.399954000	-0.121669000	0.129384000
C	-5.453923000	1.069092000	-0.632098000
C	-5.940286000	1.010075000	-1.940223000
C	-6.371269000	-0.186022000	-2.518582000
C	-6.285351000	-1.351062000	-1.754978000
C	-5.809103000	-1.349238000	-0.439815000
C	4.891374000	-0.503794000	-1.913755000
C	5.838532000	-0.626418000	-0.873701000
C	6.353531000	0.535389000	-0.293073000
C	5.979850000	1.809349000	-0.731880000
C	5.017038000	1.900084000	-1.740836000
C	4.444125000	0.766973000	-2.331372000
C	-0.714937000	-0.890432000	-2.219681000
O	-1.086207000	-1.598223000	-1.146144000
O	0.855338000	-0.579801000	0.565479000
O	0.356926000	-0.320639000	-2.318229000
C	-1.780364000	-0.829274000	-3.328042000
Mn	-0.024346000	-1.956922000	0.327563000
C	-5.027002000	2.411251000	-0.048237000
H	-4.359764000	2.206479000	0.793720000
C	-6.952744000	-0.216768000	-3.921384000
H	-7.181045000	-1.264075000	-4.154837000
C	-5.820067000	-2.638633000	0.374997000
H	-4.995051000	-2.589320000	1.095447000
C	6.301596000	-1.985786000	-0.363828000
H	5.878998000	-2.755040000	-1.016394000
C	3.401558000	0.921331000	-3.431005000
H	2.867428000	-0.028920000	-3.525040000
C	6.628789000	3.055564000	-0.154921000
H	6.108047000	3.921231000	-0.584737000
H	-5.994801000	1.924316000	-2.521366000
H	-6.617899000	-2.283263000	-2.198796000
H	4.713786000	2.884720000	-2.084680000
H	7.090803000	0.443713000	0.498288000
C	-6.234087000	3.202681000	0.487107000
H	-5.912192000	4.167041000	0.893812000
H	-6.950160000	3.397802000	-0.317262000
H	-6.759996000	2.662407000	1.276608000
C	-4.248615000	3.278608000	-1.049981000
H	-3.784163000	4.125646000	-0.535469000
H	-3.465325000	2.706285000	-1.550221000
H	-4.902387000	3.689627000	-1.824969000
C	-8.270740000	0.575480000	-3.983415000

H	-8.101338000	1.637694000	-3.777464000
H	-8.720653000	0.495579000	-4.977429000
H	-8.991073000	0.202331000	-3.250041000
C	-5.956364000	0.290034000	-4.976501000
H	-5.677473000	1.333294000	-4.792950000
H	-5.041001000	-0.309359000	-4.979481000
H	-6.397822000	0.237607000	-5.976006000
C	-7.128654000	-2.758619000	1.180098000
H	-7.988259000	-2.784383000	0.503370000
H	-7.135410000	-3.678403000	1.773684000
H	-7.260875000	-1.914648000	1.860724000
C	-5.613407000	-3.900699000	-0.476762000
H	-4.775740000	-3.792911000	-1.172908000
H	-5.423340000	-4.763795000	0.168890000
H	-6.501991000	-4.137611000	-1.068854000
C	5.760402000	-2.244398000	1.052279000
H	4.668601000	-2.173036000	1.068142000
H	6.045111000	-3.242216000	1.401680000
H	6.151435000	-1.517748000	1.771113000
C	7.829852000	-2.137160000	-0.416946000
H	8.329983000	-1.429838000	0.251476000
H	8.124381000	-3.145371000	-0.110435000
H	8.207086000	-1.963718000	-1.428755000
C	2.345204000	1.984565000	-3.096540000
H	2.770078000	2.992894000	-3.070411000
H	1.559395000	1.976941000	-3.857284000
H	1.869156000	1.780153000	-2.135744000
C	4.079025000	1.218012000	-4.780788000
H	3.332362000	1.304029000	-5.576592000
H	4.638388000	2.158224000	-4.736741000
H	4.780327000	0.425503000	-5.057260000
C	8.104394000	3.145161000	-0.584174000
H	8.556577000	4.071828000	-0.218233000
H	8.680040000	2.307127000	-0.177777000
H	8.201194000	3.121904000	-1.673204000
C	6.495720000	3.133507000	1.374817000
H	7.035605000	2.315172000	1.862196000
H	6.919599000	4.070789000	1.746987000
H	5.450852000	3.079222000	1.693547000
C	-1.054231000	-0.951317000	-4.689550000
H	-0.376874000	-0.098316000	-4.790394000
H	-1.812078000	-0.855307000	-5.475735000
C	-2.423968000	0.569649000	-3.221242000
H	-3.114855000	0.728398000	-4.053106000
H	-3.001166000	0.666196000	-2.297530000
H	-1.655973000	1.347221000	-3.251158000
C	-2.867811000	-1.901560000	-3.171624000
H	-3.571815000	-1.833001000	-4.005758000

H	-2.453388000	-2.913542000	-3.175093000
H	-3.439138000	-1.766711000	-2.251301000
C	-0.259703000	-2.244176000	-4.887809000
H	0.218280000	-2.252432000	-5.871242000
H	0.532262000	-2.335949000	-4.138572000
H	-0.896113000	-3.133153000	-4.829192000
C	-0.827312000	1.715927000	0.171163000
C	-0.098757000	2.925050000	-0.277346000
H	-1.753169000	1.570711000	-0.384438000
H	-1.040484000	1.706112000	1.241130000
C	1.141839000	3.311073000	0.328724000
C	-0.562585000	3.634180000	-1.398237000
C	1.750218000	2.463272000	1.380938000
C	1.847677000	4.419912000	-0.178428000
H	-1.483231000	3.303311000	-1.854774000
C	0.116028000	4.727576000	-1.915957000
C	1.181264000	2.334681000	2.674359000
C	2.919323000	1.755249000	1.082081000
C	1.315402000	5.113212000	-1.266169000
C	3.154505000	4.872060000	0.424822000
C	-0.374187000	5.513461000	-3.129318000
N	0.069735000	3.120657000	2.986042000
C	1.775611000	1.485792000	3.619746000
H	3.353228000	1.843900000	0.094701000
C	3.502832000	0.902589000	2.008075000
H	1.852608000	5.984241000	-1.626721000
H	3.155484000	4.778081000	1.513545000
H	3.360051000	5.913447000	0.167843000
H	3.988040000	4.269666000	0.052469000
C	0.720135000	5.477433000	-4.221950000
C	-1.669314000	4.927037000	-3.713631000
C	-0.638950000	6.977655000	-2.708174000
H	-0.087369000	3.911204000	2.376419000
C	-0.918865000	2.841078000	3.920263000
C	2.921608000	0.775460000	3.276327000
H	1.334226000	1.389189000	4.600450000
H	4.393889000	0.348205000	1.743807000
H	1.656972000	5.927304000	-3.881895000
H	0.383944000	6.038706000	-5.098687000
H	0.930344000	4.449575000	-4.531580000
H	-1.533862000	3.892271000	-4.044841000
H	-1.974394000	5.513894000	-4.583772000
H	-2.491995000	4.955818000	-2.992327000
H	-1.410213000	7.029279000	-1.933574000
H	-0.984032000	7.554216000	-3.571354000
H	0.261603000	7.464619000	-2.324008000
O	-0.877429000	1.836049000	4.617123000
C	-2.080255000	3.848159000	3.955040000

C	3.562653000	-0.101240000	4.320175000
C	-3.003360000	3.527402000	2.757473000
C	-2.851839000	3.649929000	5.267647000
C	-1.579581000	5.302867000	3.859257000
F	4.354152000	-1.047768000	3.759041000
F	2.621023000	-0.762668000	5.048908000
F	4.312270000	0.597309000	5.181902000
H	-3.363284000	2.496881000	2.810856000
H	-2.496278000	3.668443000	1.797328000
H	-3.873998000	4.189491000	2.774650000
H	-3.208627000	2.623059000	5.357870000
H	-3.712163000	4.324183000	5.293478000
H	-2.220511000	3.864112000	6.133809000
H	-1.140679000	5.543116000	2.883725000
H	-0.840007000	5.526386000	4.633129000
H	-2.423538000	5.984613000	3.994936000
H	-0.169376000	0.828115000	0.022061000

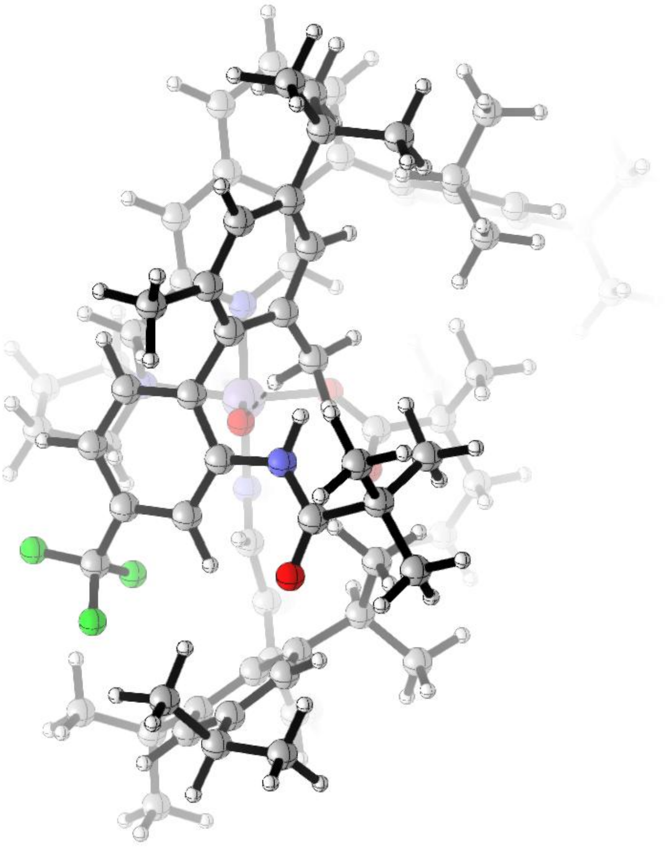

TS^{minor}-(S)

N	1.929897000	-0.414739000	-1.704327000
N	-0.184955000	-1.216608000	-3.132664000
N	1.098619000	-3.306639000	-1.897494000
N	-1.127648000	-2.729924000	-0.508163000

C	4.213649000	0.288559000	-1.548286000
C	3.075645000	-0.364880000	-1.038646000
H	3.104194000	-0.880292000	-0.086664000
C	5.456985000	0.279808000	-0.840219000
C	6.528252000	0.931540000	-1.425610000
H	7.481633000	0.938710000	-0.908802000
C	6.417462000	1.569126000	-2.683048000
H	7.289183000	2.060717000	-3.102649000
C	5.228863000	1.568250000	-3.381656000
H	5.151198000	2.050993000	-4.350304000
C	4.098600000	0.929004000	-2.826747000
C	2.843795000	0.867018000	-3.492240000
H	2.726080000	1.340952000	-4.461506000
C	1.788533000	0.203846000	-2.923274000
C	0.406160000	0.103915000	-3.498179000
H	-0.230264000	0.866869000	-3.047024000
H	0.415806000	0.248897000	-4.582530000
C	-1.661408000	-1.240612000	-3.377631000
H	-2.102812000	-0.331697000	-2.984951000
H	-2.074691000	-2.083820000	-2.823163000
C	-1.794415000	-1.456678000	-4.883544000
H	-1.701530000	-0.507760000	-5.419633000
H	-2.765648000	-1.879333000	-5.145486000
C	-0.615611000	-2.402927000	-5.223341000
H	-0.960602000	-3.433741000	-5.341004000
H	-0.124586000	-2.124588000	-6.157336000
C	0.356896000	-2.306070000	-4.014309000
H	1.363451000	-2.014544000	-4.323258000
C	1.320591000	-4.672279000	-3.898520000
H	0.858476000	-5.659492000	-3.844565000
H	1.444223000	-4.441416000	-4.959361000
C	0.436488000	-3.601176000	-3.209466000
H	-0.575093000	-3.960932000	-3.007098000
C	2.553651000	-3.419693000	-2.207212000
H	2.865189000	-2.511443000	-2.722626000
H	3.119407000	-3.489409000	-1.280960000
C	2.673495000	-4.636567000	-3.135969000
H	2.820360000	-5.552942000	-2.559425000
H	3.529439000	-4.540536000	-3.806373000
C	0.706480000	-4.307903000	-0.878177000
H	1.406183000	-4.202511000	-0.047089000
H	0.781617000	-5.330532000	-1.262487000
C	-0.685816000	-4.030704000	-0.392511000
C	-1.444359000	-4.983357000	0.222944000
H	-1.072916000	-6.000097000	0.303059000
C	-2.687332000	-4.644320000	0.810390000
C	-3.438704000	-5.590041000	1.536991000
H	-3.074353000	-6.608242000	1.624235000

C	-4.609922000	-5.196132000	2.145451000
H	-5.187379000	-5.905990000	2.728870000
C	-5.074485000	-3.872220000	2.012219000
H	-6.004911000	-3.587470000	2.489802000
C	-4.398924000	-2.912066000	1.272921000
C	-3.150326000	-3.289620000	0.677390000
C	-2.311129000	-2.374803000	-0.002102000
H	-2.591769000	-1.333838000	-0.113604000
C	5.628714000	-0.430286000	0.467553000
C	5.595865000	0.301748000	1.674425000
C	5.900190000	-0.354156000	2.870177000
C	6.220673000	-1.712165000	2.910385000
C	6.206980000	-2.422826000	1.708544000
C	5.923359000	-1.813868000	0.482360000
C	-5.053738000	-1.576015000	1.141021000
C	-6.238889000	-1.482776000	0.375692000
C	-6.992933000	-0.309075000	0.455734000
C	-6.622886000	0.768645000	1.265654000
C	-5.426760000	0.664625000	1.977986000
C	-4.630634000	-0.487400000	1.935348000
C	0.685657000	-1.605955000	1.711041000
O	1.199016000	-1.816111000	0.484456000
O	-0.572075000	-0.053253000	-0.793455000
O	-0.346739000	-0.997432000	1.905194000
C	1.546497000	-2.160061000	2.856705000
Mn	0.320084000	-1.440779000	-1.098559000
C	5.291735000	1.794222000	1.701327000
H	4.902359000	2.070604000	0.716040000
C	6.615435000	-2.392158000	4.209990000
H	6.803124000	-3.447913000	3.978565000
C	6.024260000	-2.623482000	-0.806390000
H	5.330179000	-2.186266000	-1.534685000
C	-6.750695000	-2.627722000	-0.493905000
H	-6.001865000	-3.425939000	-0.486245000
C	-3.415015000	-0.589035000	2.846541000
H	-2.742401000	-1.355298000	2.456425000
C	-7.528016000	1.988519000	1.357745000
H	-8.555155000	1.615963000	1.247549000
H	5.899317000	0.214141000	3.794422000
H	6.452110000	-3.479153000	1.733270000
H	-5.120175000	1.489000000	2.605372000
H	-7.915344000	-0.236820000	-0.113571000
C	6.578379000	2.605919000	1.931783000
H	6.370802000	3.679662000	1.914053000
H	7.021367000	2.362852000	2.902351000
H	7.326120000	2.390404000	1.163306000
C	4.216933000	2.158839000	2.738260000
H	4.020124000	3.234387000	2.727171000

H	3.275449000	1.639045000	2.541442000
H	4.528646000	1.902049000	3.754194000
C	7.921192000	-1.794277000	4.763319000
H	7.788511000	-0.741863000	5.035596000
H	8.239280000	-2.333025000	5.660651000
H	8.726560000	-1.851481000	4.025656000
C	5.495885000	-2.337061000	5.261595000
H	5.250283000	-1.303664000	5.528980000
H	4.583743000	-2.819224000	4.897408000
H	5.806000000	-2.849433000	6.176887000
C	7.441869000	-2.523379000	-1.402833000
H	8.177849000	-2.920358000	-0.697145000
H	7.513742000	-3.100991000	-2.329966000
H	7.713715000	-1.489420000	-1.625744000
C	5.643119000	-4.102022000	-0.627031000
H	4.708931000	-4.223471000	-0.068649000
H	5.533966000	-4.584968000	-1.603085000
H	6.417459000	-4.653259000	-0.086386000
C	-6.948017000	-2.211218000	-1.962149000
H	-6.007338000	-1.892670000	-2.414191000
H	-7.341108000	-3.053463000	-2.540111000
H	-7.660092000	-1.386172000	-2.056441000
C	-8.054947000	-3.211822000	0.078758000
H	-8.854121000	-2.464335000	0.075411000
H	-8.390993000	-4.063700000	-0.520515000
H	-7.926781000	-3.550784000	1.110769000
C	-2.601838000	0.710490000	2.907548000
H	-3.164320000	1.516378000	3.385272000
H	-1.686032000	0.542685000	3.478958000
H	-2.314709000	1.036218000	1.904377000
C	-3.844704000	-1.032449000	4.257608000
H	-2.971000000	-1.141375000	4.909054000
H	-4.517422000	-0.295377000	4.707578000
H	-4.370045000	-1.991557000	4.229590000
C	-7.269278000	2.971428000	0.199143000
H	-7.966699000	3.812938000	0.259196000
H	-6.252148000	3.368107000	0.257976000
H	-7.400104000	2.491642000	-0.773306000
C	-7.434914000	2.714004000	2.706943000
H	-6.467810000	3.211376000	2.820751000
H	-8.211334000	3.482033000	2.769753000
H	-7.573762000	2.024193000	3.545216000
C	0.600618000	-2.625375000	3.990059000
H	0.041063000	-1.755056000	4.344284000
H	1.230669000	-2.960480000	4.822258000
C	2.384511000	-0.963737000	3.354551000
H	2.960008000	-1.252505000	4.237226000
H	3.098042000	-0.637970000	2.594256000

H	1.738266000	-0.123851000	3.624303000
C	2.486859000	-3.288426000	2.409502000
H	3.065373000	-3.642667000	3.267361000
H	1.936627000	-4.146080000	2.012262000
H	3.197158000	-2.945349000	1.653843000
C	-0.387871000	-3.728845000	3.607689000
H	-1.015987000	-3.990786000	4.463450000
H	-1.051995000	-3.398030000	2.805217000
H	0.116647000	-4.644342000	3.282246000
F	-4.265712000	-0.421260000	-1.763889000
F	-4.709744000	0.447724000	-3.704523000
F	-5.837523000	1.076538000	-1.953686000
O	-4.211997000	3.487589000	1.683249000
N	-2.142943000	3.697027000	0.745622000
H	-1.284864000	4.224975000	0.821940000
C	-3.569316000	1.784656000	-2.109786000
C	-3.439232000	2.282687000	-0.811578000
H	-4.165657000	2.042359000	-0.047922000
C	-2.339175000	3.095791000	-0.501927000
C	-1.339115000	3.312908000	-1.475635000
C	-1.556408000	2.886851000	-2.786693000
H	-0.817472000	3.134296000	-3.542693000
C	-2.674922000	2.131751000	-3.121287000
H	-2.836023000	1.792096000	-4.136874000
C	-0.030683000	3.940632000	-1.150211000
C	0.313572000	5.179860000	-1.696513000
C	1.626289000	5.659134000	-1.529864000
H	1.863458000	6.626119000	-1.956597000
C	2.622434000	4.937357000	-0.858609000
C	2.253985000	3.708613000	-0.304243000
H	2.978654000	3.129730000	0.257443000
C	0.933803000	3.210272000	-0.382524000
C	-4.618027000	0.746490000	-2.389403000
C	-3.074542000	3.924939000	1.746942000
C	-2.553417000	4.784893000	2.910605000
C	-3.588853000	4.746210000	4.042620000
H	-3.716895000	3.730634000	4.426892000
H	-4.562700000	5.096710000	3.696435000
H	-3.258620000	5.385602000	4.865812000
C	-1.199202000	4.251948000	3.424396000
H	-1.270169000	3.200576000	3.716824000
H	-0.893620000	4.827907000	4.302461000
H	-0.394196000	4.347601000	2.686404000
C	-2.392367000	6.235515000	2.400959000
H	-3.339531000	6.623393000	2.015588000
H	-1.642394000	6.315700000	1.605828000
H	-2.071149000	6.881009000	3.223421000
C	-0.681524000	6.014194000	-2.465708000

H	-0.685977000	5.751496000	-3.529687000
H	-1.699317000	5.872349000	-2.096567000
C	4.050405000	5.476252000	-0.700735000
C	5.042441000	4.446512000	-1.277586000
H	4.960256000	3.475116000	-0.786293000
H	6.070406000	4.798033000	-1.146806000
H	4.868440000	4.296049000	-2.346827000
C	4.258970000	6.809540000	-1.438361000
H	4.070770000	6.717374000	-2.512802000
H	5.295331000	7.133995000	-1.313085000
H	3.620204000	7.603445000	-1.039929000
C	4.336334000	5.701191000	0.799076000
H	3.645251000	6.435462000	1.223462000
H	5.355247000	6.075597000	0.937057000
H	4.237160000	4.776253000	1.370442000
C	0.580232000	2.001808000	0.376083000
H	-0.291964000	2.105850000	1.027394000
H	0.135489000	1.106592000	-0.288217000
H	1.419316000	1.578574000	0.928997000
H	-0.430115000	7.075086000	-2.395494000

11. References

(1) Font, D.; Canta, M.; Milan, M.; Cussó, O.; Ribas, X.; Klein Gebbink, R. J.; Costas, M. Readily Accessible Bulky Iron Catalysts exhibiting Site Selectivity in the Oxidation of Steroidal Substrates. *Angew. Chem. Int. Ed.* **2016**, *55* (19), 5776-5779.

(2) Milan, M.; Bietti, M.; Costas, M. Highly Enantioselective Oxidation of Nonactivated Aliphatic C–H Bonds with Hydrogen Peroxide Catalyzed by Manganese Complexes. *ACS Cent. Sci.* **2017**, *3* (3), 196-204. DOI: 10.1021/acscentsci.6b00368.

(3) Call, A.; Capocasa, G.; Palone, A.; Vicens, L.; Aparicio, E.; Choukairi Afailal, N.; Siakavaras, N.; López Saló, M. E.; Bietti, M.; Costas, M. Highly Enantioselective Catalytic Lactonization at Nonactivated Primary and Secondary γ-C–H Bonds. *J. Am. Chem. Soc.* **2023**, *145* (32), 18094-18103. DOI: 10.1021/jacs.3c06231.

(4) Mintz, T.; Liu, L.; Pappo, D. Chiral Bis-8-Aryl-isoquinoline Bis-alkylamine Iron Catalysts for Asymmetric Oxidation Reactions. *Org. Lett.* **2025**, *27* (4), 1078-1083. DOI: 10.1021/acs.orglett.5c00050.

(5) Cussó, O.; Garcia-Bosch, I.; Ribas, X.; Lloret-Fillol, J.; Costas, M. Asymmetric epoxidation with H2O2 by manipulating the electronic properties of non-heme iron catalysts. *J. Am. Chem. Soc.* **2013**, *135* (39), 14871-14878. DOI: 10.1021/ja4078446.

(6) Cussó, O.; Garcia-Bosch, I.; Font, D.; Ribas, X.; Lloret-Fillol, J.; Costas, M. Highly stereoselective epoxidation with H2O2 catalyzed by electron-rich aminopyridine manganese catalysts. *Org. Lett.* **2013**, *15* (24), 6158-6161.

(7) Barder, T. E.; Walker, S. D.; Martinelli, J. R.; Buchwald, S. L. Catalysts for Suzuki−Miyaura Coupling Processes: Scope and Studies of the Effect of Ligand Structure. *J. Am. Chem. Soc.* **2005**, *127* (13), 4685-4696. DOI: 10.1021/ja042491j.

(8) Li, C.; Cai, S.-Z.; Ye, J.; Fang, X. Enantioselective Synthesis of Axially and Centrally Chiral Styrenes via Nickel-Catalyzed Desymmetric Hydrocyanation of Biaryl Dienes. *Org. Lett.* **2024**, *26* (18), 3867-3871. DOI: 10.1021/acs.orglett.4c01022.

(9) Uchikura, T.; Kato, S.; Makino, Y.; Fujikawa, M. J.; Yamanaka, M.; Akiyama, T. Chiral Phosphoric Acid–Palladium(II) Complex Catalyzed Asymmetric Desymmetrization of Biaryl Compounds by C(sp3)–H Activation. *J. Am. Chem. Soc.* **2023**, *145* (29), 15906-15911. DOI: 10.1021/jacs.3c03552.

(10) Prisinzano, T.; Law, H.; Dukat, M.; Slassi, A.; MaClean, N.; Demchyshyn, L.; Glennon, R. A. Imidazoline-modified benzylimidazolines as h5-HT1D/1B serotonergic ligands. *Biorg. Med. Chem.* **2001**, *9* (3), 613-619. DOI: https://doi.org/10.1016/S0968-0896(00)00275-3.

(11) Bringmann, G.; Holenz, J.; Wiesen, B.; Nugroho, B. W.; Proksch, P. Dioncophylline A as a Growth-Retarding Agent against the Herbivorous Insect Spodoptera littoralis: Structure−Activity Relationships. *J. Nat. Prod.* **1997**, *60* (4), 342-347. DOI: 10.1021/np960707s.

(12) Milan, M.; Carboni, G.; Salamone, M.; Costas, M.; Bietti, M. Tuning Selectivity in Aliphatic C–H Bond Oxidation of N-Alkylamides and Phthalimides Catalyzed by Manganese Complexes. *ACS Catal.* **2017**, *7* (9), 5903-5911. DOI: 10.1021/acscatal.7b02151.

(13) Shi, Y.; Zhang, L.; Lan, J.; Zhang, M.; Zhou, F.; Wei, W.; You, J. Oxidative C−H/C−H Cross-Coupling Reactions between N-Acylanilines and Benzamides Enabled by a Cp*-Free RhCl3/TFA Catalytic System. *Angew. Chem. Int. Ed.* **2018**, *57* (29), 9108-9112. DOI: https://doi.org/10.1002/anie.201804528.

(14) Bie, J.; Lang, M.; Wang, J. Enantioselective N-Heterocyclic Carbene-Catalyzed Kinetic Resolution of Anilides. *Org. Lett.* **2018**, *20* (18), 5866-5871. DOI: 10.1021/acs.orglett.8b02538.

(15) Liu, J.; Liu, J.; Li, H.; Bin, Z.; You, J. Boron-Dipyrromethene-Based Fluorescent Emitters Enable High-Performance Narrowband Red Organic Light-Emitting Diodes. *Angew. Chem. Int. Ed.* **2023**, *62* (31), e202306471. DOI: https://doi.org/10.1002/anie.202306471.
(16) Cabrera-Afonso, M. J.; Carreño, M. C.; Urbano, A. Site-selective Oxidative Dearomatization of Phenols and Naphthols into ortho-Quinols or Epoxy ortho-Quinols using Oxone as the Source of Dimethyldioxirane. *Adv. Synth. Catal.* **2019**, *361* (19), 4468-4473. DOI: https://doi.org/10.1002/adsc.201900660.
(17) Furukawa, T.; Tobisu, M.; Chatani, N. C–H Functionalization at Sterically Congested Positions by the Platinum-Catalyzed Borylation of Arenes. *J. Am. Chem. Soc.* **2015**, *137* (38), 12211-12214. DOI: 10.1021/jacs.5b07677.
(18) Lu, Y.; Lum, T. K.; Leow Augustine, Y. W.; Weltrowska, G.; Nguyen, T. M. D.; Lemieux, C.; Chung, N. N.; Schiller, P. W. Replacement of the N-terminal Tyrosine Residue in Opioid Peptides with 3-(2,6-Dimethyl-4-carbamoylphenyl)propanoic Acid (Dcp) Results in Novel Opioid Antagonists. *J. Med. Chem.* **2006**, *49* (17), 5382-5385. DOI: 10.1021/jm060369k.
(19) Gao, H.; Xu, Q.-L.; Yousufuddin, M.; Ess, D. H.; Kürti, L. Rapid Synthesis of Fused N-Heterocycles by Transition-Metal-Free Electrophilic Amination of Arene C□H Bonds. *Angew. Chem. Int. Ed.* **2014**, *53* (10), 2701-2705. DOI: https://doi.org/10.1002/anie.201309973.
(20) Crich, D.; Hutton, T. K.; Ranganathan, K. Is There a Homolytic Substitution Chemistry (SH2) of Sulfones? *J. Org. Chem.* **2005**, *70* (19), 7672-7678. DOI: 10.1021/jo050990c.
(21) Clarasó, C.; Vicens, L.; Polo, A.; Costas, M. Enantioselective Epoxidation of β,β-Disubstituted Enamides with a Manganese Catalyst and Aqueous Hydrogen Peroxide. *Org. Lett.* **2019**, *21* (7), 2430-2435. DOI: 10.1021/acs.orglett.9b00729.
(22) Wan, J.-C.; Huang, J.-M.; Jhan, Y.-H.; Hsieh, J.-C. Novel Syntheses of Fluorenones via Nitrile-Directed Palladium-Catalyzed C–H and Dual C–H Bond Activation. *Org. Lett.* **2013**, *15* (11), 2742-2745. DOI: 10.1021/ol401063w.
(23) Nascimento, V. R.; Suenaga, M. L. S.; Andrade, L. H. An efficient approach for the synthesis of new (±)-coixspirolactams. *Org. Biomol. Chem.* **2020**, *18* (28), 5458-5465. DOI: 10.1039/D0OB01104E.
(24) Huang, Y.; Chan, G. H.; Chiba, S. Amide-Directed C−H Sodiation by a Sodium Hydride/Iodide Composite. *Angew. Chem. Int. Ed.* **2017**, *56* (23), 6544-6547. DOI: https://doi.org/10.1002/anie.201702512.
(25) Ishikawa, S.; Manabe, K. Synthetic method for multifunctionalized oligoarenes using pinacol esters of hydroxyphenylboronic acids. *Chem. Commun.* **2006**, (24), 2589-2591. DOI: 10.1039/B603574D.
(26) Haldar, C.; Bisht, R.; Chaturvedi, J.; Guria, S.; Hassan, M. M. M.; Ram, B.; Chattopadhyay, B. Ligand- and Substrate-Controlled para C–H Borylation of Anilines at Room Temperature. *Org. Lett.* **2022**, *24* (44), 8147-8152. DOI: 10.1021/acs.orglett.2c03188.
(27) Dobrydnev, A. V.; Volovnenko, T. A.; Volovenko, Y. M.; Palamarchuk, G. V.; Shishkin, O. V. Cyclic α-amino acids as precursors for synthesis of 2-amino-3-hetarylpyrrolin-4-ones and their spiro derivatives. *Monatshefte für Chemie - Chemical Monthly* **2012**, *143* (5), 779-789. DOI: 10.1007/s00706-012-0727-3.
(28) Poriel, C.; Ferrand, Y.; Juillard, S.; Le Maux, P.; Simonneaux, G. Synthesis and stereochemical studies of di and tetra 9,9′-spirobifluorene porphyrins: new building blocks for catalytic material. *Tetrahedron* **2004**, *60* (1), 145-158. DOI: https://doi.org/10.1016/j.tet.2003.10.080.
(29) Bonnaventure, I.; Charette, A. B. Probing the Importance of the Hemilabile Site of Bis(phosphine) Monoxide Ligands in the Copper-Catalyzed Addition of Diethylzinc to N-Phosphinoylimines: Discovery of New Effective Chiral Ligands. *J. Org. Chem.* **2008**, *73* (16), 6330-6340. DOI: 10.1021/jo800969x.

(30) Evoniuk, C. J.; Hill, S. P.; Hanson, K.; Alabugin, I. V. Double C–H amination by consecutive SET oxidations. *Chem. Commun.* **2016**, *52* (44), 7138-7141, 10.1039/C6CC03106D. DOI: 10.1039/C6CC03106D.
(31) Bos, M. E.; Loncaric, C.; Wu, C.; Wulff, W. D. Studies on the Synthesis of Richardianidin-1 via the Tautomer-Arrested Annulation of Fischer Carbene Complexes. *Synthesis* **2006**, *2006* (21), 3679-3705. DOI: 10.1055/s-2006-950313.
(32) Cheon, C. H.; Yamamoto, H. A Brønsted Acid Catalyst for the Enantioselective Protonation Reaction. *J. Am. Chem. Soc.* **2008**, *130* (29), 9246-9247. DOI: 10.1021/ja8041542.
(33) Murai, K.; Fukushima, S.; Nakamura, A.; Shimura, M.; Fujioka, H. C3-Symmetric chiral trisimidazoline: the role of a third imidazoline and its application to the nitro Michael reaction and the α-amination of β-ketoesters. *Tetrahedron* **2011**, *67* (26), 4862-4868. DOI: https://doi.org/10.1016/j.tet.2011.05.005.
(34) Sapala, A. R.; Maurya, G. P.; Singh, H.; Mehta, N.; Karmakar, T.; Haridas, V. Expanded triazolophanes: a topological analysis of vesicular assembly. *Org. Biomol. Chem.* **2023**, *21* (26), 5372-5376. DOI: 10.1039/D3OB00868A.
(35) Neuhaus, P.; Grote, D.; Sander, W. Matrix Isolation, Spectroscopic Characterization, and Photoisomerization of m-Xylylene. *J. Am. Chem. Soc.* **2008**, *130* (10), 2993-3000. DOI: 10.1021/ja073453d.
(36) Maeda, H.; Endo, S.; Ouchi, T.; Mizuno, K.; Segi, M. Synthesis and Conformational Analysis of 2,11-Dioxa[3.3]metacyclophanes. *Chem. Lett.* **2017**, *46* (9), 1357-1360. DOI: 10.1246/cl.170527.
(37) Suzuki, H.; Nakamura, K. Nitration of Pentamethylnitrobenzene, Pentamethylbenzoic Acid and Its Methyl Ester, Pentamethylacetanilide, and Pentamethylphenol and Its Methyl Ether. Orienting Effect of the Substituents for the Side-chain Nitrooxylation. *Bulletin of the Chemical Society of Japan* **1971**, *44* (1), 227-231. DOI: 10.1246/bcsj.44.227.
(38) Howell, J. M.; Feng, K.; Clark, J. R.; Trzepkowski, L. J.; White, M. C. Remote Oxidation of Aliphatic C–H Bonds in Nitrogen-Containing Molecules. *J. Am. Chem. Soc.* **2015**, *137* (46), 14590-14593. DOI: 10.1021/jacs.5b10299.
(39) Vyskočil, Š.; Jaracz, S.; Smrčina, M.; Štícha, M.; Hanuš, V.; Polášek, M.; Kočovský, P. Synthesis of N-Alkylated and N-Arylated Derivatives of 2-Amino-2‘-hydroxy-1,1‘-binaphthyl (NOBIN) and 2,2‘-Diamino-1,1‘-binaphthyl and Their Application in the Enantioselective Addition of Diethylzinc to Aromatic Aldehydes. *J. Org. Chem.* **1998**, *63* (22), 7727-7737. DOI: 10.1021/jo9807565.
(40) Carbó López, M.; Royal, G.; Philouze, C.; Chavant, P. Y.; Blandin, V. Imidazolidinone Nitroxides as Catalysts in the Aerobic Oxidation of Alcohols, en Route to Atroposelective Oxidative Desymmetrization. *Eur. J. Org. Chem.* **2014**, *2014* (22), 4884-4896. DOI: https://doi.org/10.1002/ejoc.201402324.
(41) Liu, W.; Li, J.; Querard, P.; Li, C.-J. Transition-Metal-Free C–C, C–O, and C–N Cross-Couplings Enabled by Light. *J. Am. Chem. Soc.* **2019**, *141* (16), 6755-6764. DOI: 10.1021/jacs.9b02684.
(42) Si, T.; Li, B.; Xiong, W.; Xu, B.; Tang, W. Efficient cross-coupling of aryl/alkenyl triflates with acyclic secondary alkylboronic acids. *Org. Biomol. Chem.* **2017**, *15* (46), 9903-9909. DOI: 10.1039/C7OB02531A.
(43) Borrell, M.; Gil-Caballero, S.; Bietti, M.; Costas, M. Site-Selective and Product Chemoselective Aliphatic C–H Bond Hydroxylation of Polyhydroxylated Substrates. *ACS Catal.* **2020**, *10*, 4702-4709. DOI: 10.1021/acscatal.9b05423.
(44) Sakakura, A.; Kondo, R.; Matsumura, Y.; Akakura, M.; Ishihara, K. Rational Design of Highly Effective Asymmetric Diels−Alder Catalysts Bearing 4,4′-Sulfonamidomethyl Groups. *J. Am. Chem. Soc.* **2009**, *131* (49), 17762-17764. DOI: 10.1021/ja906098b.

(45) Atherton, J. H.; Blacker, J.; Crampton, M. R.; Grosjean, C. The Strecker reaction: kinetic and equilibrium studies of cyanide addition to iminium ions. *Org. Biomol. Chem.* **2004**, *2* (18), 2567-2571. DOI: 10.1039/B407853E.
(46) Palone, A.; Casadevall, G.; Ruiz-Barragan, S.; Call, A.; Osuna, S.; Bietti, M.; Costas, M. C–H Bonds as Functional Groups: Simultaneous Generation of Multiple Stereocenters by Enantioselective Hydroxylation at Unactivated Tertiary C–H Bonds. *J. Am. Chem. Soc.* **2023**, *145* (29), 15742-15753. DOI: 10.1021/jacs.2c10148.
(47) Andrus, M. B.; Asgari, D.; Sclafani, J. A. Efficient Synthesis of 1,1'-Binaphthyl and 2,2'-Bi-o-tolyl-2,2'-bis(oxazoline)s and Preliminary Use for the Catalytic Asymmetric Allylic Oxidation of Cyclohexene. *J. Org. Chem.* **1997**, *62* (26), 9365-9368. DOI: 10.1021/jo9713619.
(48) Liao, G.; Li, B.; Chen, H.-M.; Yao, Q.-J.; Xia, Y.-N.; Luo, J.; Shi, B.-F. Pd-Catalyzed Atroposelective C−H Allylation through β-O Elimination: Diverse Synthesis of Axially Chiral Biaryls. *Angew. Chem. Int. Ed.* **2018**, *57* (52), 17151-17155. DOI: https://doi.org/10.1002/anie.201811256.
(49) Xu, P.; López-Rojas, P.; Ritter, T. Radical Decarboxylative Carbometalation of Benzoic Acids: A Solution to Aromatic Decarboxylative Fluorination. *J. Am. Chem. Soc.* **2021**, *143* (14), 5349-5354. DOI: 10.1021/jacs.1c02490.
(50) Escayola, S.; Bahri-Laleh, N.; Poater, A. %V_{Bur} index and steric maps: from predictive catalysis to machine learning. *Chem Soc Rev* **2024**, *53* (2), 853-882. DOI: 10.1039/d3cs00725a.
(51) Bryliakov, K. P.; Talsi, E. P. Active sites and mechanisms of bioinspired oxidation with H_2O_2, catalyzed by non-heme Fe and related Mn complexes. *Coord. Chem. Rev.* **2014**, *276*, 73-96. DOI: 10.1016/j.ccr.2014.06.009.
(52) Fan, R.; Serrano-Plana, J.; Oloo, W. N.; Draksharapu, A.; Delgado-Pinar, E.; Company, A.; Martin-Diaconescu, V.; Borrell, M.; Lloret-Fillol, J.; García-España, E.; et al. Spectroscopic and DFT Characterization of a Highly Reactive Nonheme Fe(V)-Oxo Intermediate. *J. Am. Chem. Soc.* **2018**, *140* (11), 3916-3928. DOI: 10.1021/jacs.7b11400 From NLM Medline.
(53) Call, A.; Cianfanelli, M.; Besalú-Sala, P.; Olivo, G.; Palone, A.; Vicens, L.; Ribas, X.; Luis, J. M.; Bietti, M.; Costas, M. Carboxylic Acid Directed *γ*-Lactonization of Unactivated Primary C–H Bonds Catalyzed by Mn Complexes: Application to Stereoselective Natural Product Diversification. *J. Am. Chem. Soc.* **2022**, *144* (42), 19542-19558. DOI: 10.1021/jacs.2c08620.
(54) Falivene, L.; Cao, Z.; Petta, A.; Serra, L.; Poater, A.; Oliva, R.; Scarano, V.; Cavallo, L. Towards the online computer-aided design of catalytic pockets. *Nat. Chem.* **2019**, *11* (10), 872-879. DOI: 10.1038/s41557-019-0319-5 From NLM Medline.
(55) *Gaussian 16 Rev. C.01*; Wallingford, CT, 2016.
(56) Grimme, S.; Ehrlich, S.; Goerigk, L. Effect of the damping function in dispersion corrected density functional theory. *J. Comput. Chem.* **2011**, *32* (7), 1456-1465. DOI: https://doi.org/10.1002/jcc.21759.
(57) Grimme, S.; Antony, J.; Ehrlich, S.; Krieg, H. A consistent and accurate ab initio parametrization of density functional dispersion correction (DFT-D) for the 94 elements H-Pu. *J. Chem. Phys.* **2010**, *132* (15), 154104-154119. DOI: 10.1063/1.3382344.
(58) Schäfer, A.; Huber, C.; Ahlrichs, R. Fully optimized contracted Gaussian basis sets of triple zeta valence quality for atoms Li to Kr. *J. Chem. Phys.* **1994**, *100* (8), 5829-5835. DOI: 10.1063/1.467146.
(59) Marenich, A. V.; Cramer, C. J.; Truhlar, D. G. Universal Solvation Model Based on Solute Electron Density and on a Continuum Model of the Solvent Defined by the Bulk Dielectric Constant and Atomic Surface Tensions. *J. Phys. Chem. B* **2009**, *113* (18), 6378-6396.

All NMR spectra for all compounds are shown on a single page, with ^{1}H spectra on top and ^{13}C spectra below. When F atoms are present in the compounds, ^{19}F spectra are provided.

11.1. NMR spectra of the ligands

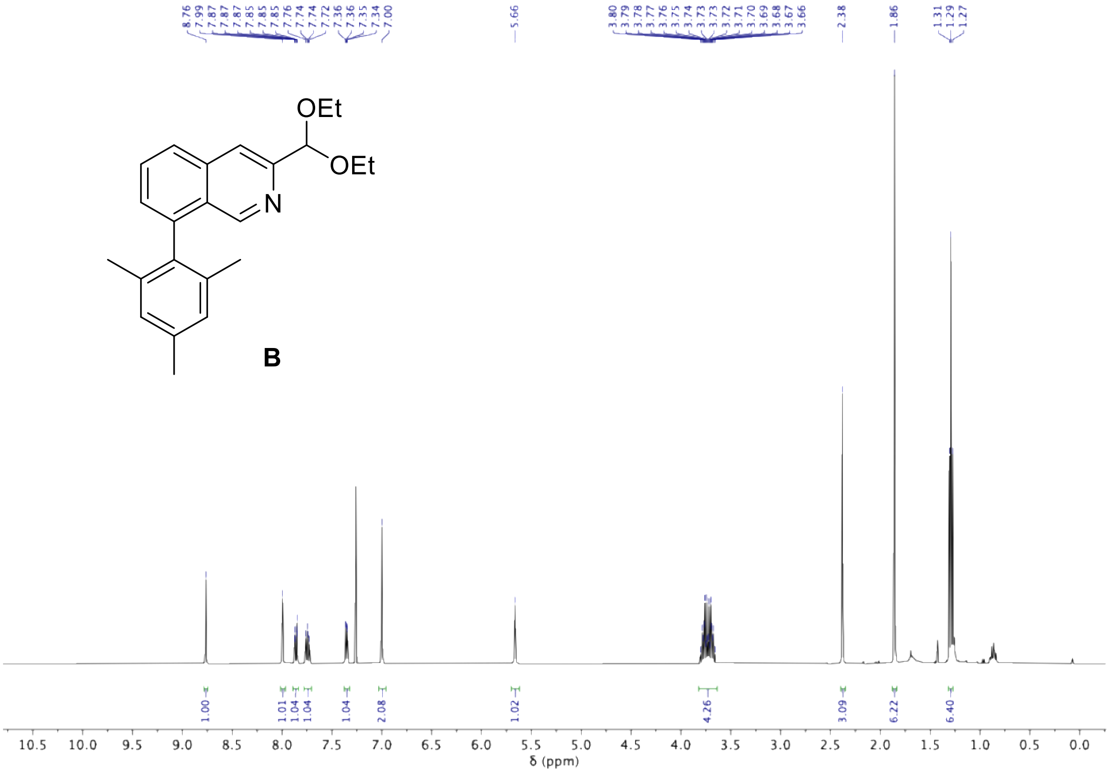

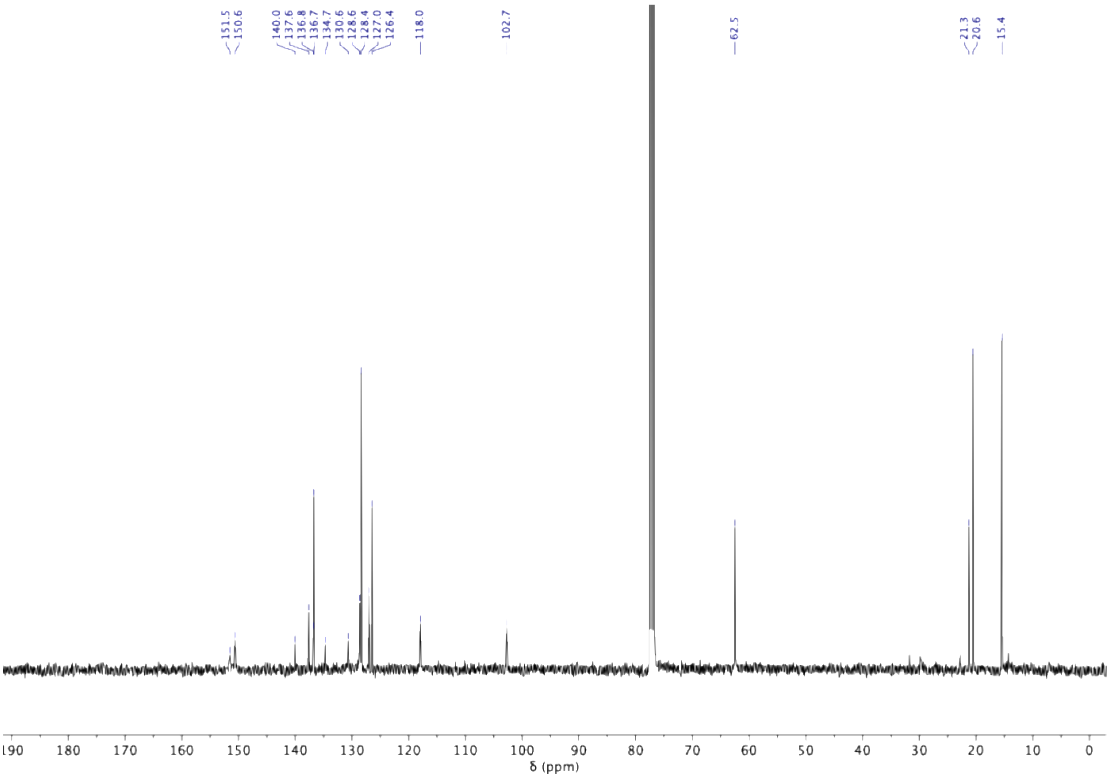

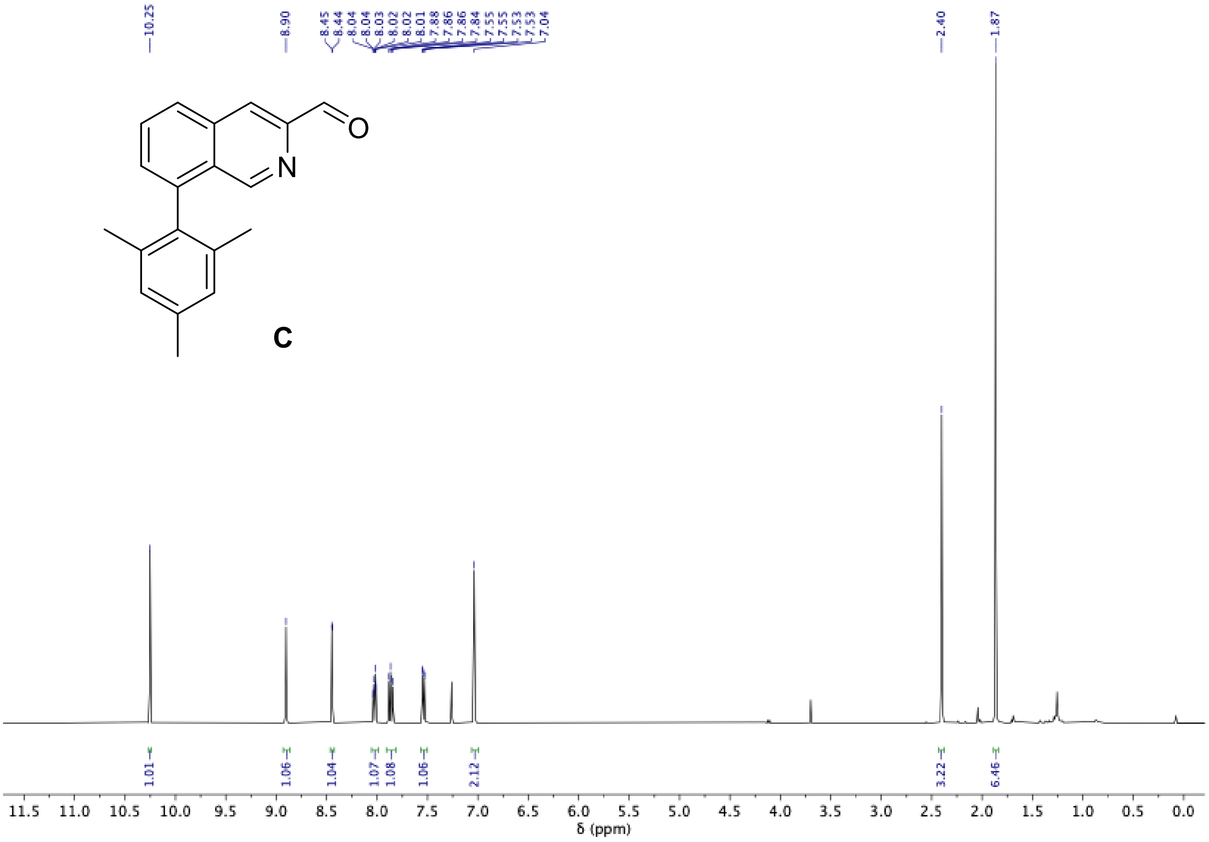

C

10.25
8.90
8.45
8.44
8.04
8.04
8.03
8.02
8.02
8.01
7.88
7.86
7.86
7.84
7.55
7.55
7.53
7.53
7.04
2.40
1.87
1.01
1.06
1.04
1.07
1.08
1.06
2.12
3.22
6.46
11.5 11.0 10.5 10.0 9.5 9.0 8.5 8.0 7.5 7.0 6.5 6.0 5.5 5.0 4.5 4.0 3.5 3.0 2.5 2.0 1.5 1.0 0.5 0.0
δ (ppm)

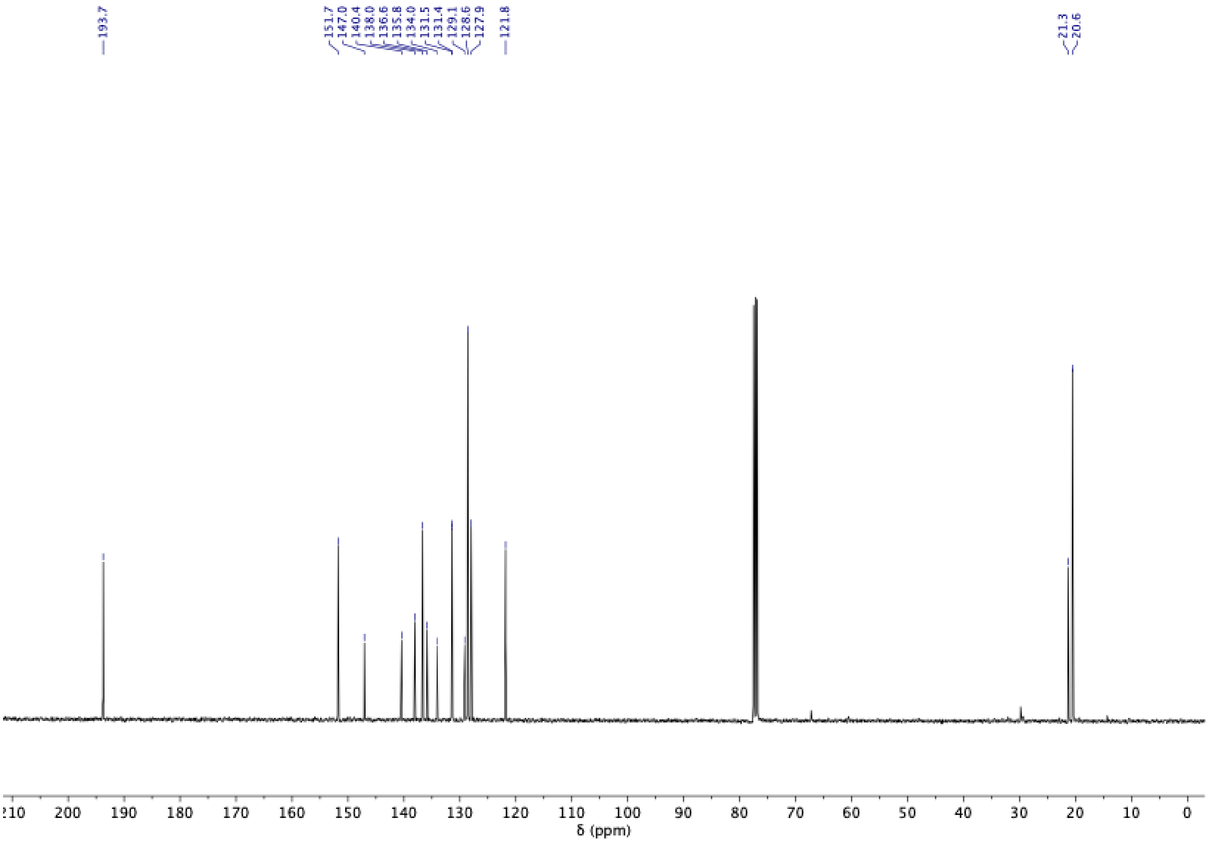

193.7
151.7
147.0
140.4
138.0
136.6
135.8
134.0
131.5
131.4
129.1
128.6
127.9
121.8
21.3
20.6
210 200 190 180 170 160 150 140 130 120 110 100 90 80 70 60 50 40 30 20 10 0
δ (ppm)

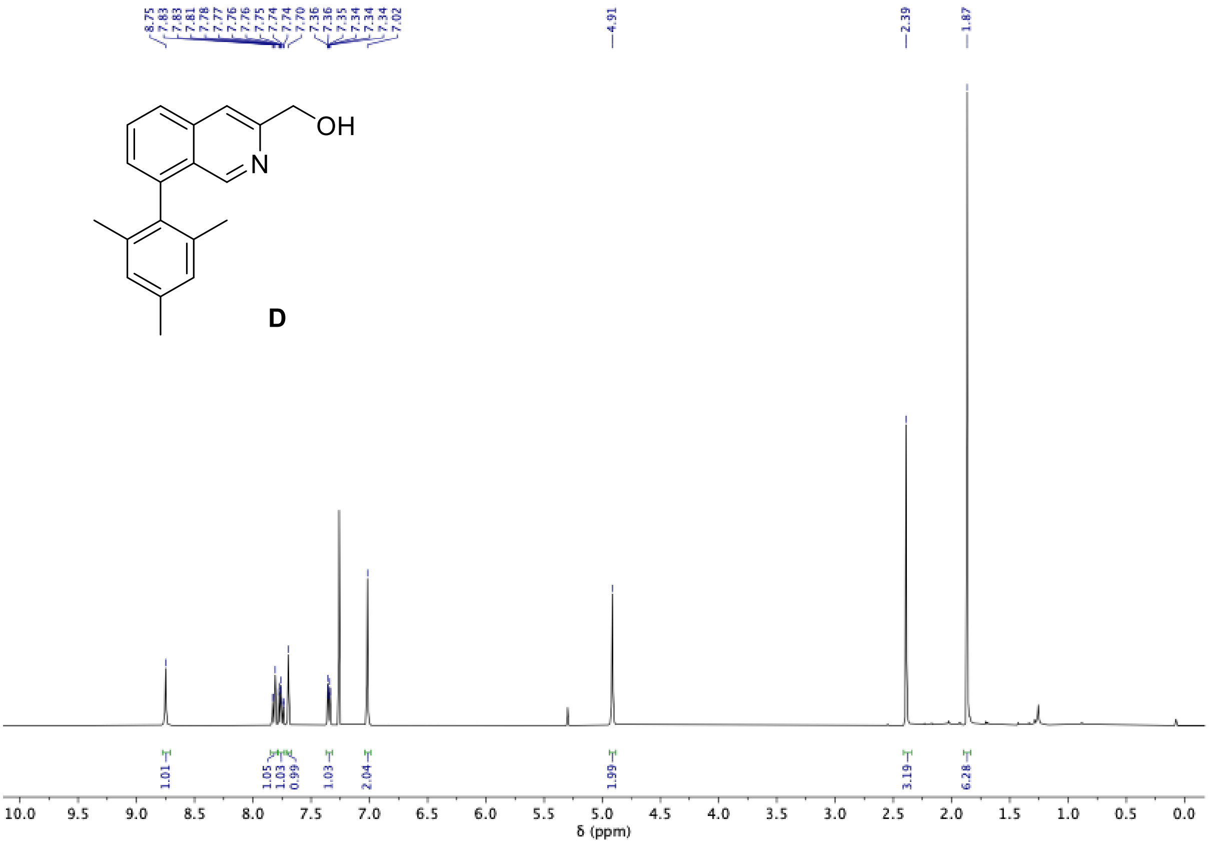
8.75 7.83 7.83 7.81 7.78 7.77 7.76 7.76 7.75 7.74 7.74 7.70 7.36 7.36 7.35 7.34 7.34 7.34 7.02
4.91
2.39
1.87
OH
N
1.01
1.05 1.03 0.99
1.03
2.04
1.99
3.19
6.28
10.0 9.5 9.0 8.5 8.0 7.5 7.0 6.5 6.0 5.5 5.0 4.5 4.0 3.5 3.0 2.5 2.0 1.5 1.0 0.5 0.0
δ (ppm)

D

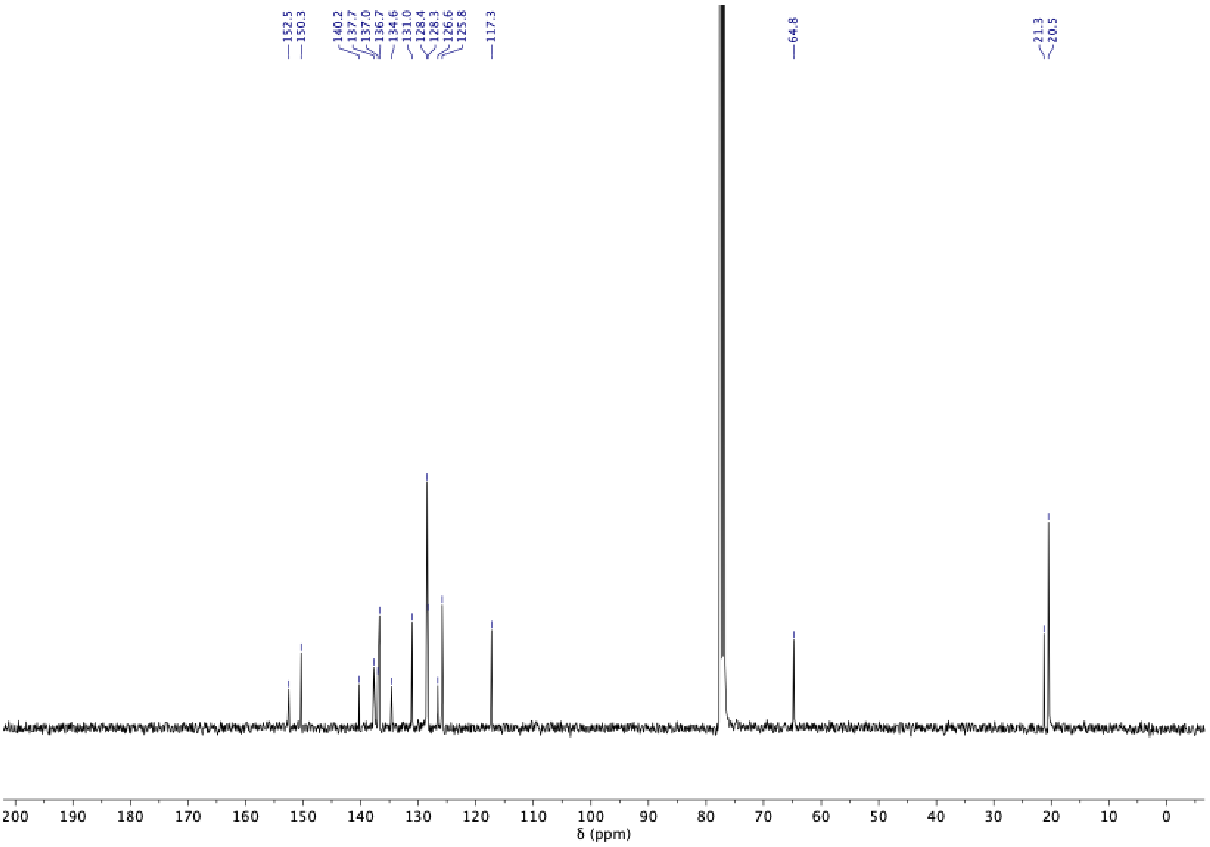
152.5 150.3
140.2 137.7 137.0 136.7 134.6 131.0 128.4 128.3 126.6 125.8
117.3
64.8
21.3 20.5
200 190 180 170 160 150 140 130 120 110 100 90 80 70 60 50 40 30 20 10 0
δ (ppm)

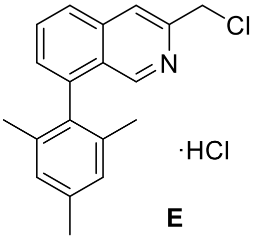
Cl
N
·HCl

E

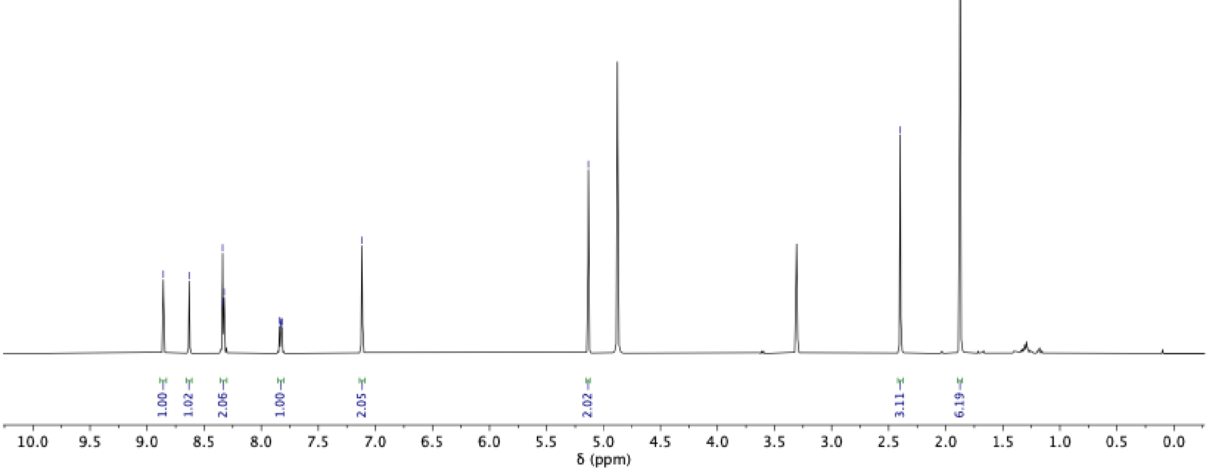
8.86
8.63
8.34
8.33
8.32
7.84
7.83
7.82
7.82
7.12
5.13
2.40
1.88
1.00
1.02
2.06
1.00
2.05
2.02
3.11
6.19
10.0 9.5 9.0 8.5 8.0 7.5 7.0 6.5 6.0 5.5 5.0 4.5 4.0 3.5 3.0 2.5 2.0 1.5 1.0 0.5 0.0
δ (ppm)

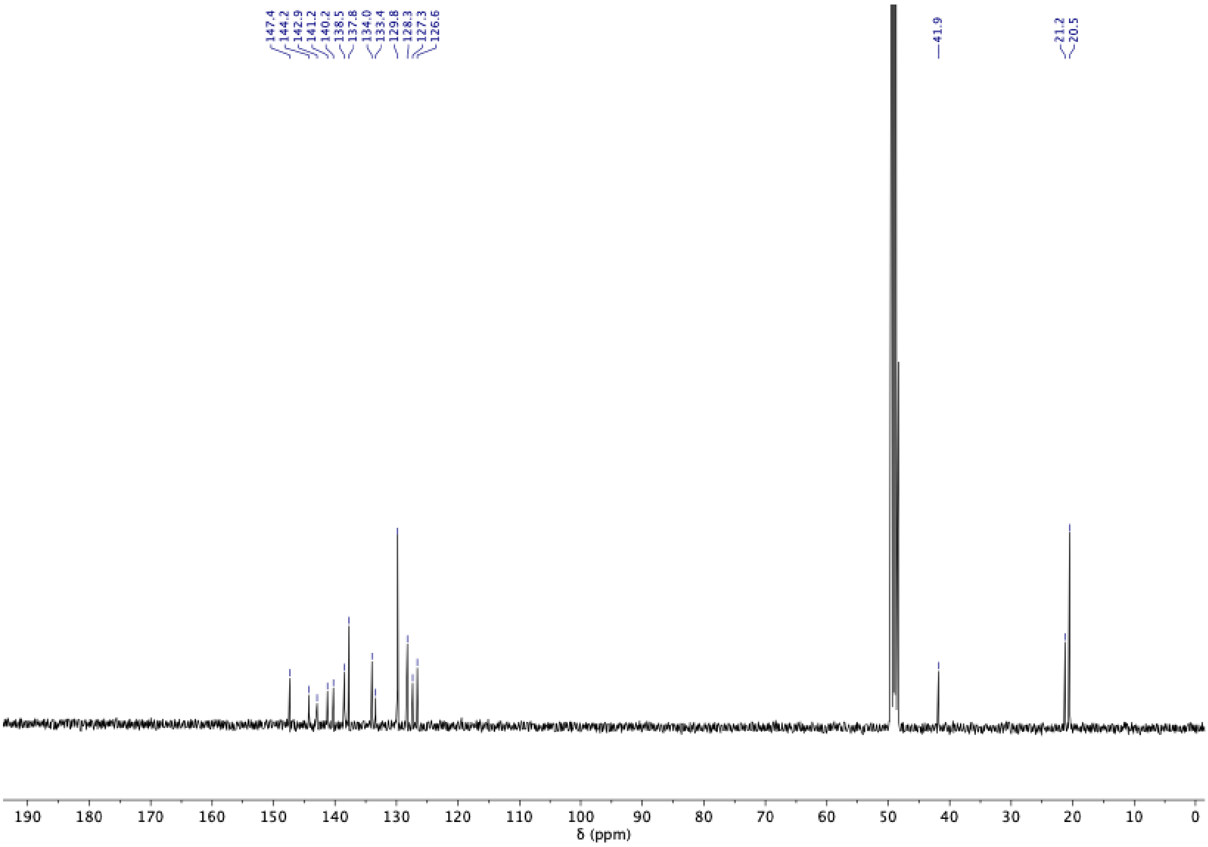
147.4
144.2
142.9
141.2
140.2
138.5
137.8
134.0
133.4
129.8
128.3
127.3
126.6
41.9
21.2
20.5
190 180 170 160 150 140 130 120 110 100 90 80 70 60 50 40 30 20 10 0
δ (ppm)

L1

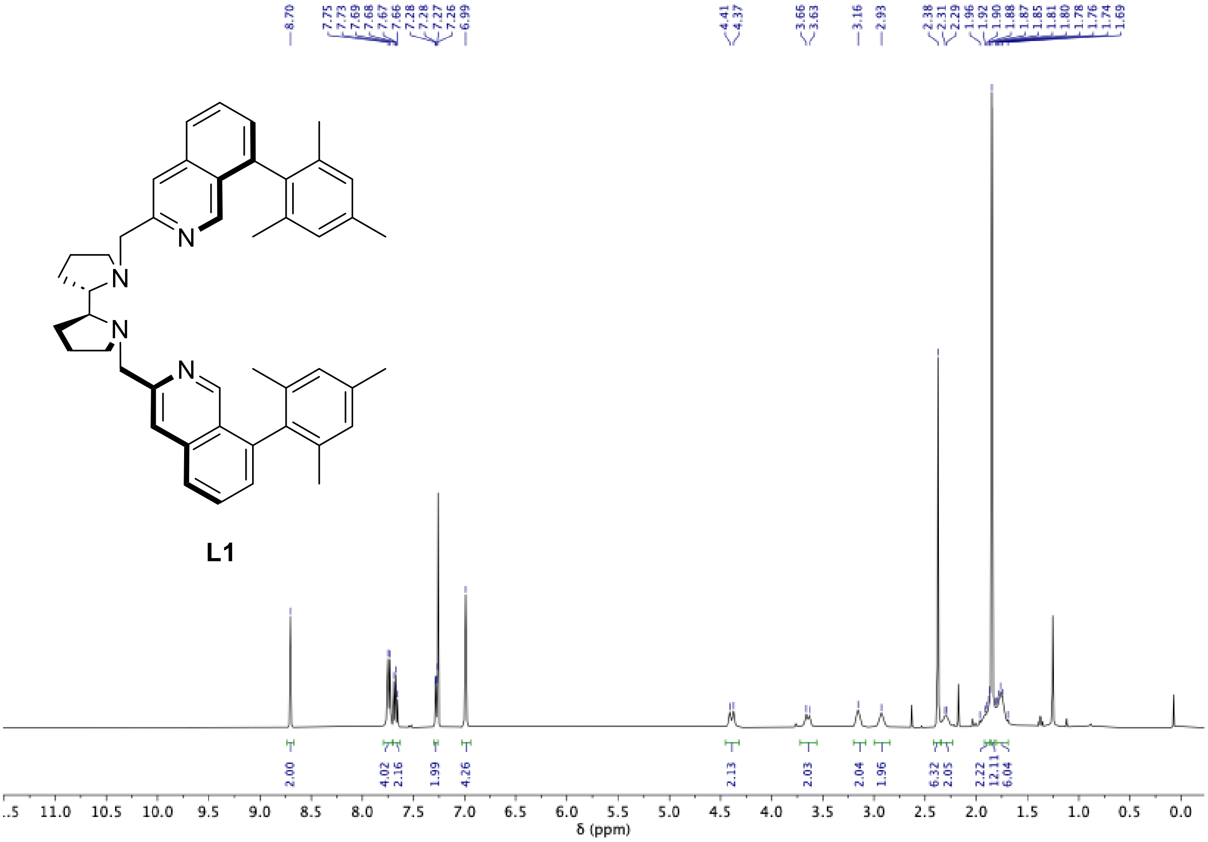
8.70
7.75 7.73 7.69 7.68 7.67 7.66 7.28 7.28 7.27 7.26 6.99
4.41 4.37
3.66 3.63
3.16
2.93
2.38 2.31 2.29 1.96 1.92 1.90 1.88 1.87 1.85 1.81 1.80 1.78 1.76 1.74 1.69
2.00 4.02 2.16 1.99 4.26 2.13 2.03 2.04 1.96 6.32 2.05 2.22 12.11 6.04
δ (ppm)

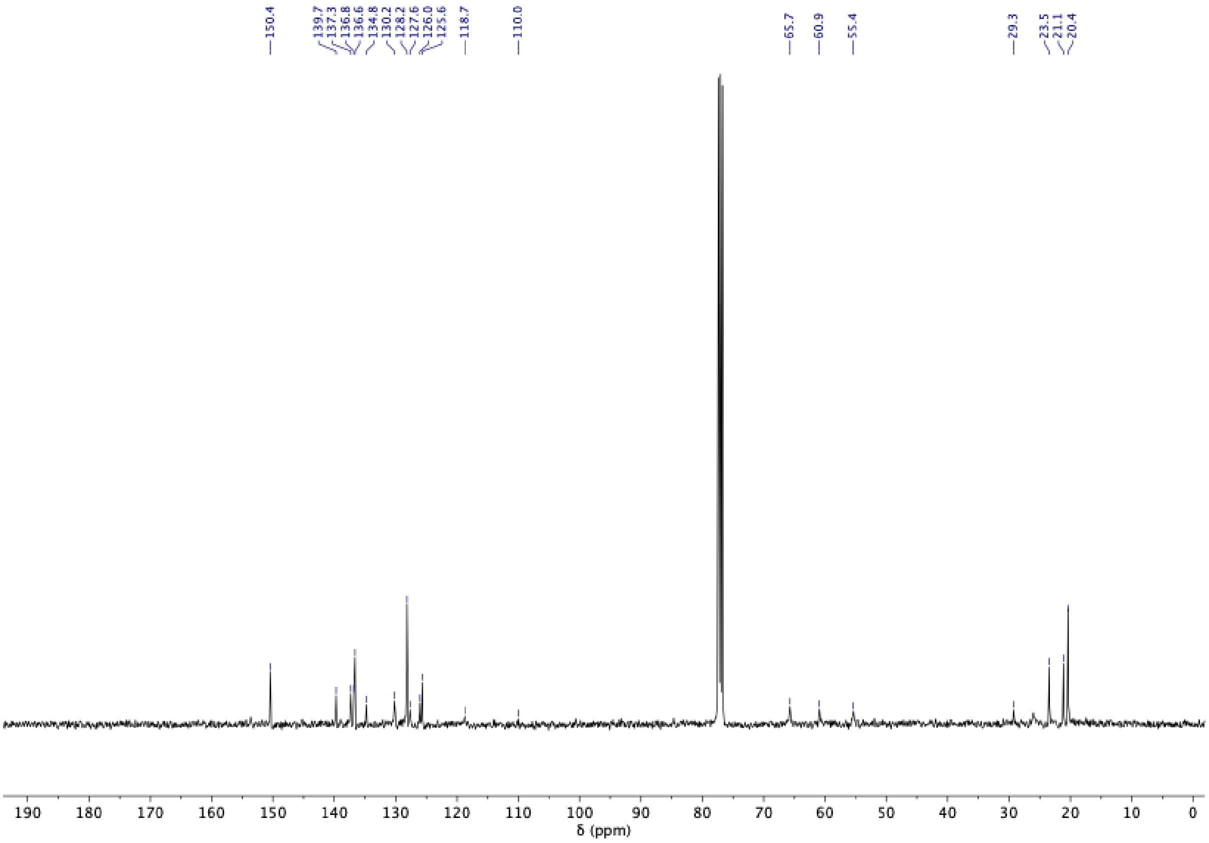
150.4
139.7 137.3 136.8 136.6 134.8 130.2 128.2 127.6 126.0 125.6
118.7
110.0
65.7
60.9
55.4
29.3
23.5 21.1 20.4
δ (ppm)

11.2. NMR spectra of the substrates

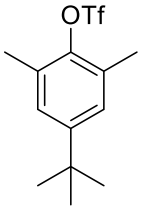

Id

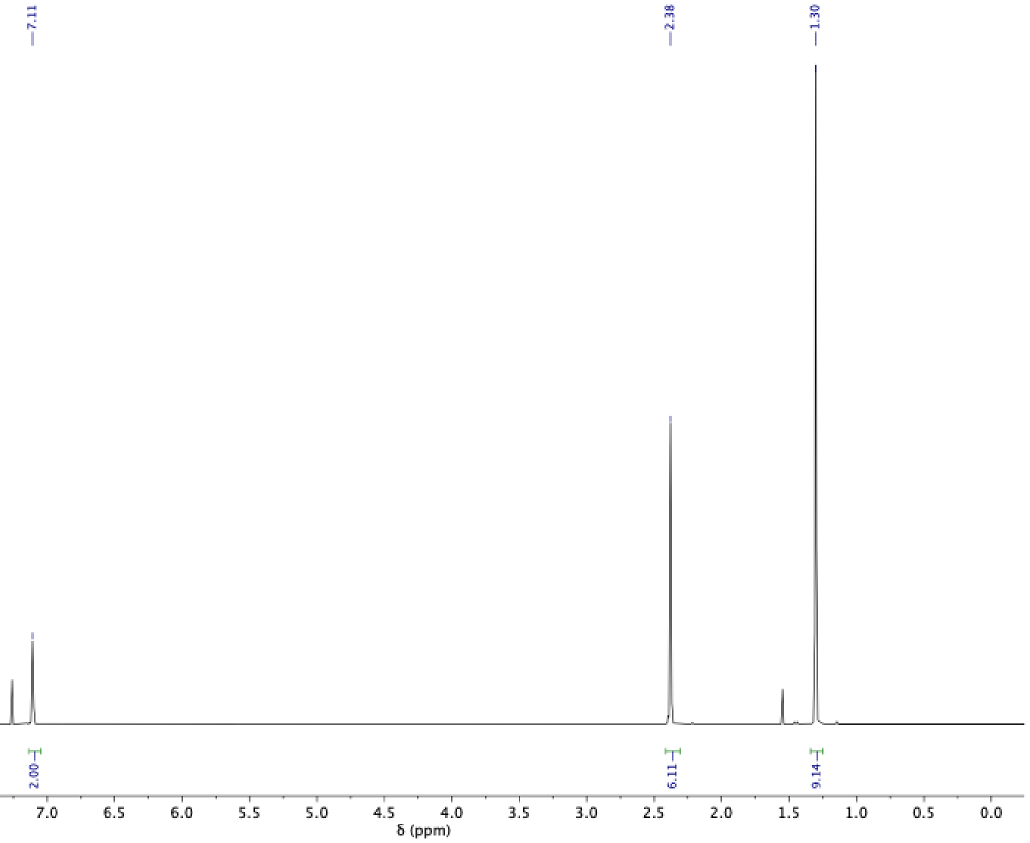

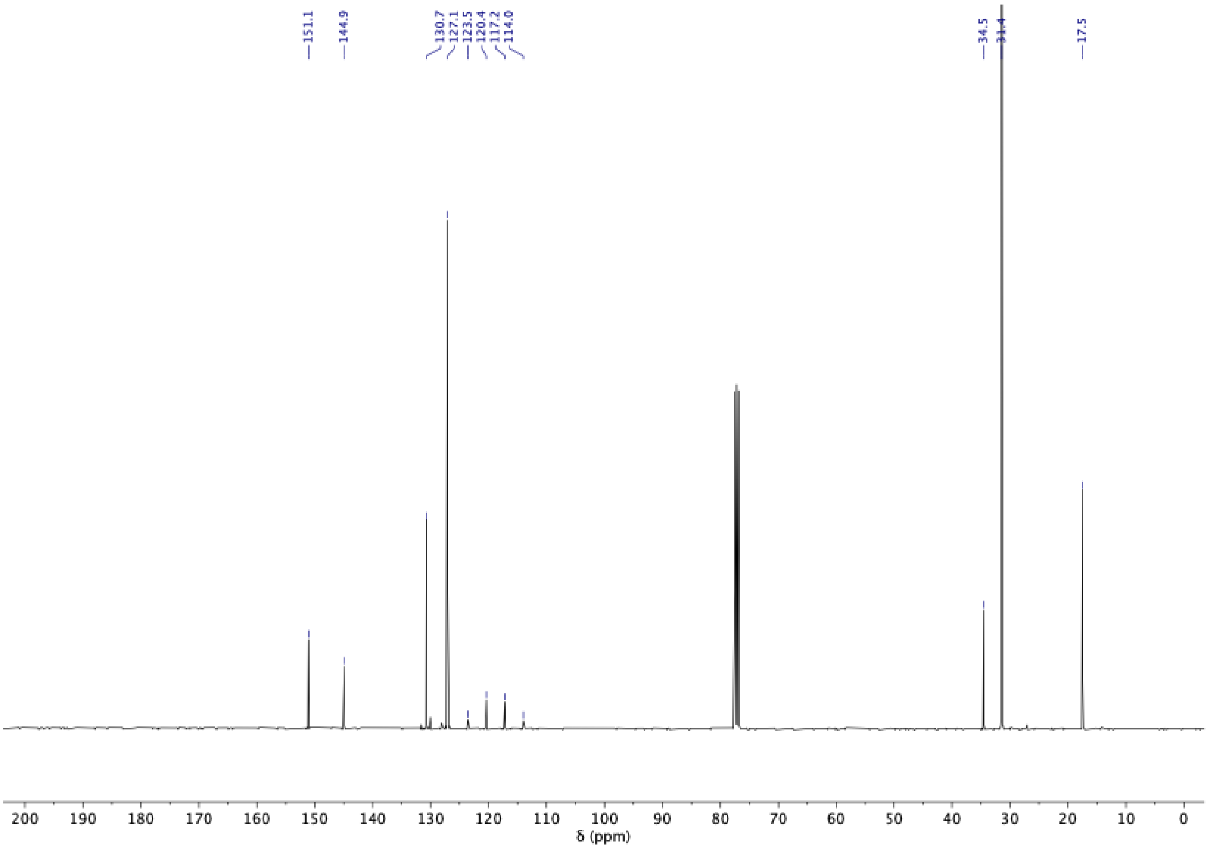

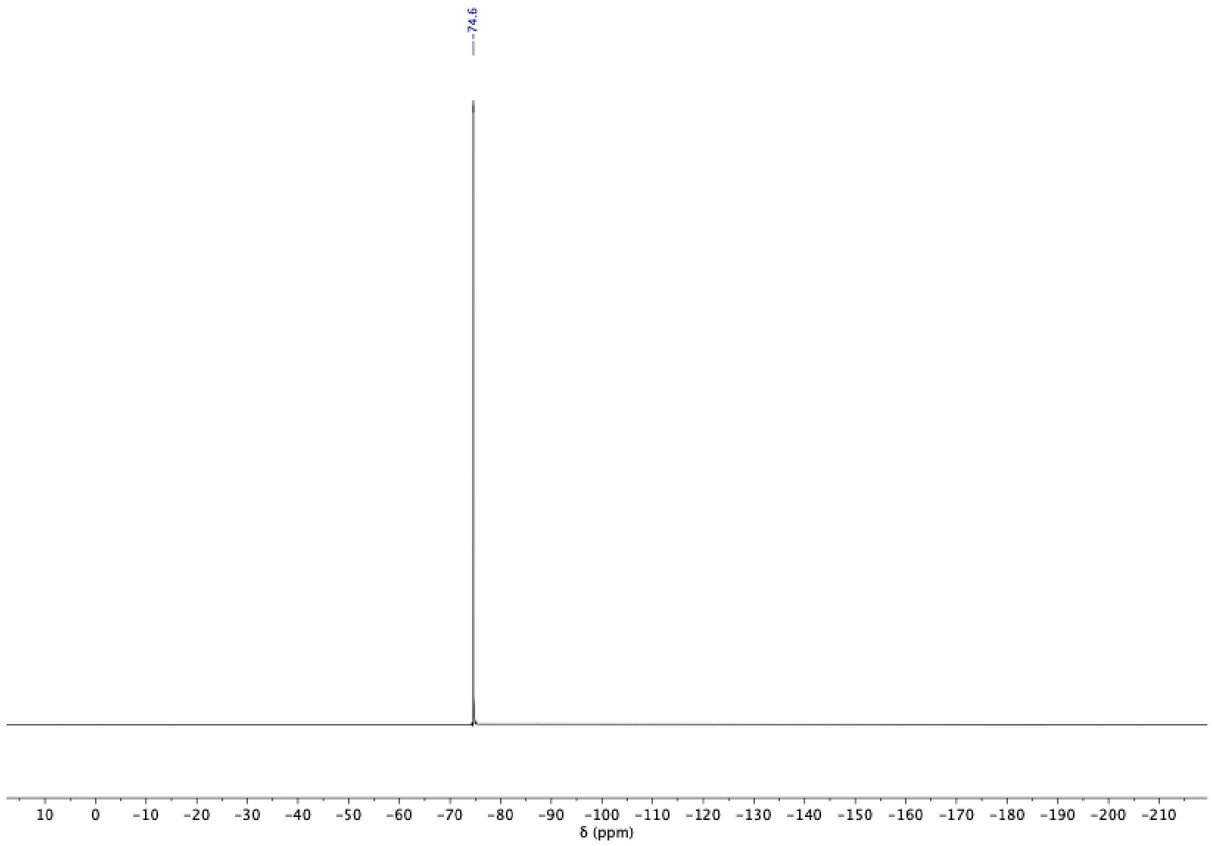

-74.6
10 0 −10 −20 −30 −40 −50 −60 −70 −80 −90 −100 −110 −120 −130 −140 −150 −160 −170 −180 −190 −200 −210
δ (ppm)

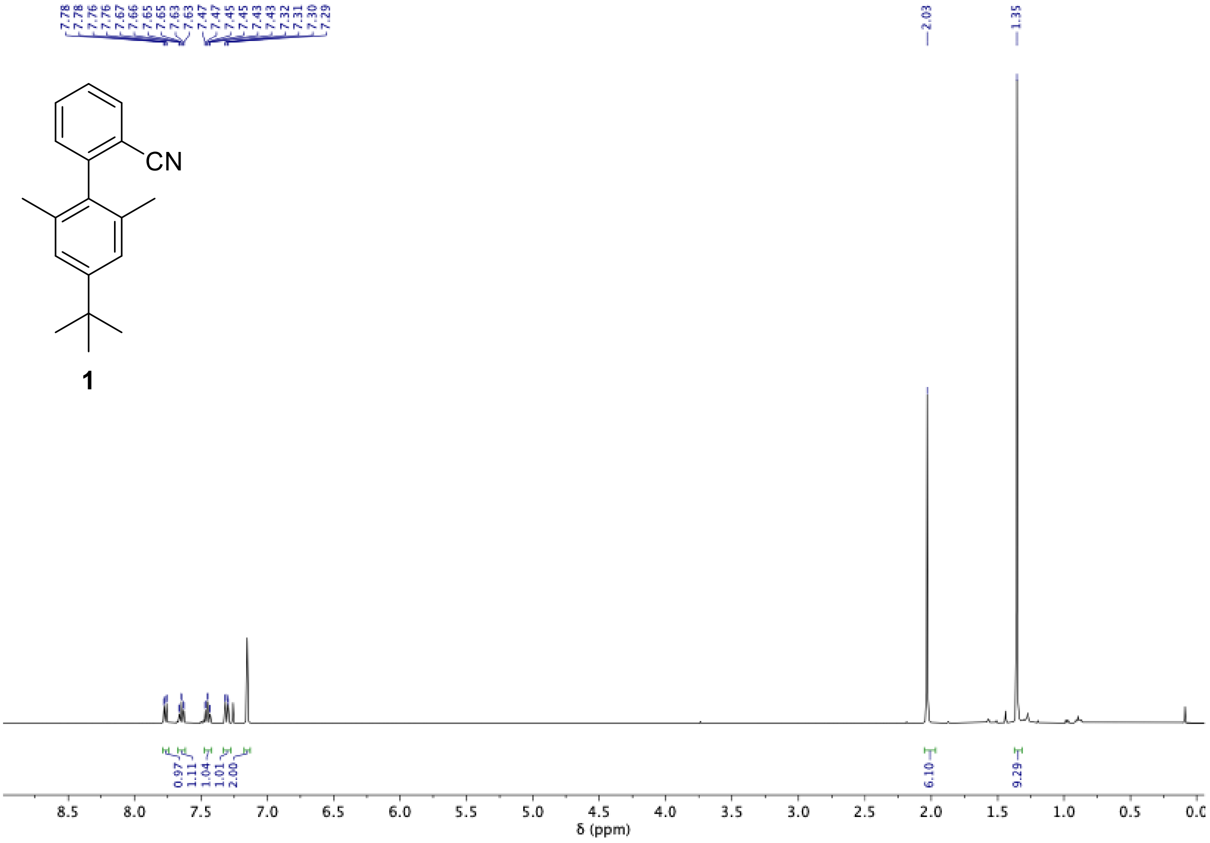

1
7.78
7.78
7.76
7.76
7.67
7.66
7.65
7.65
7.63
7.63
7.47
7.47
7.45
7.45
7.43
7.43
7.32
7.31
7.30
7.29
2.03
1.35
0.97
1.11
1.04
1.01
2.00
6.10
9.29
δ (ppm)

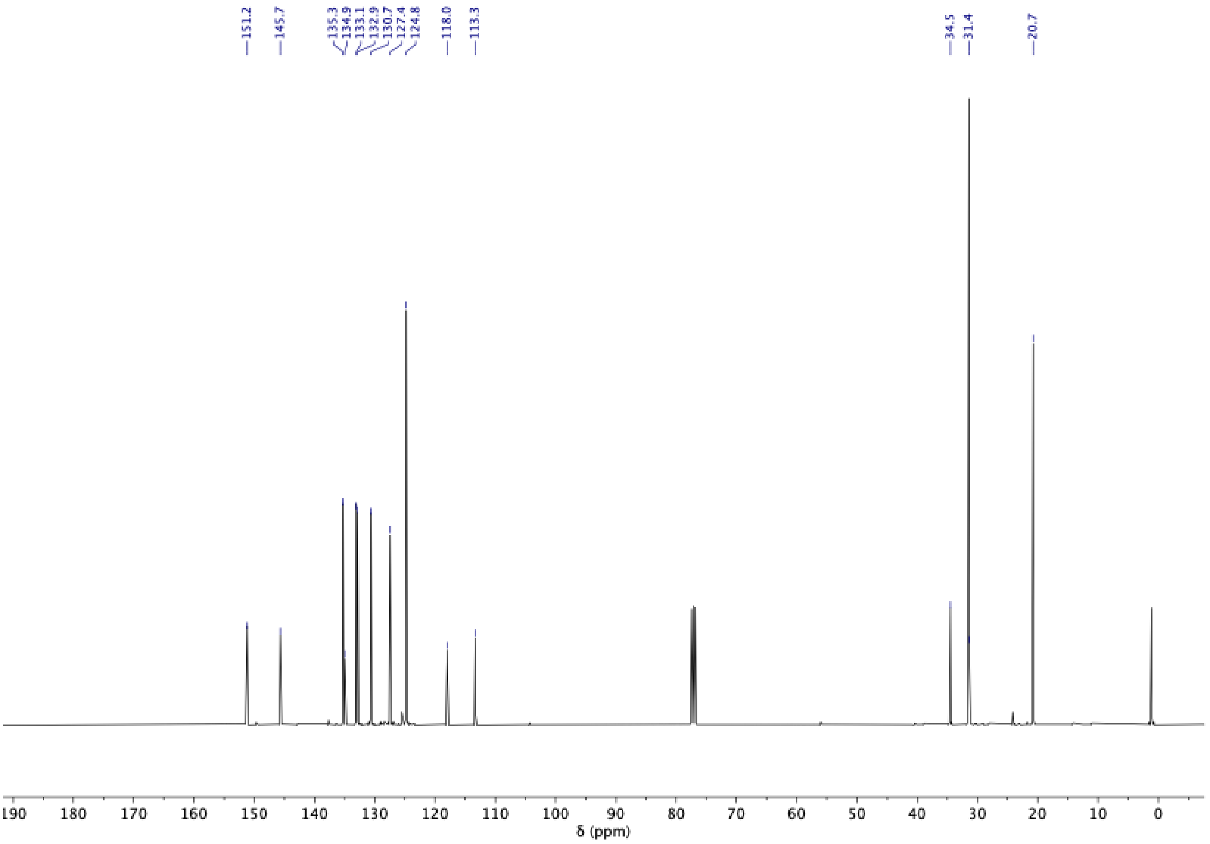
151.2
145.7
135.3
134.9
133.1
132.9
130.7
127.4
124.8
118.0
113.3
34.5
31.4
20.7
δ (ppm)

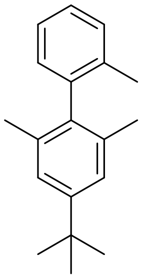

2

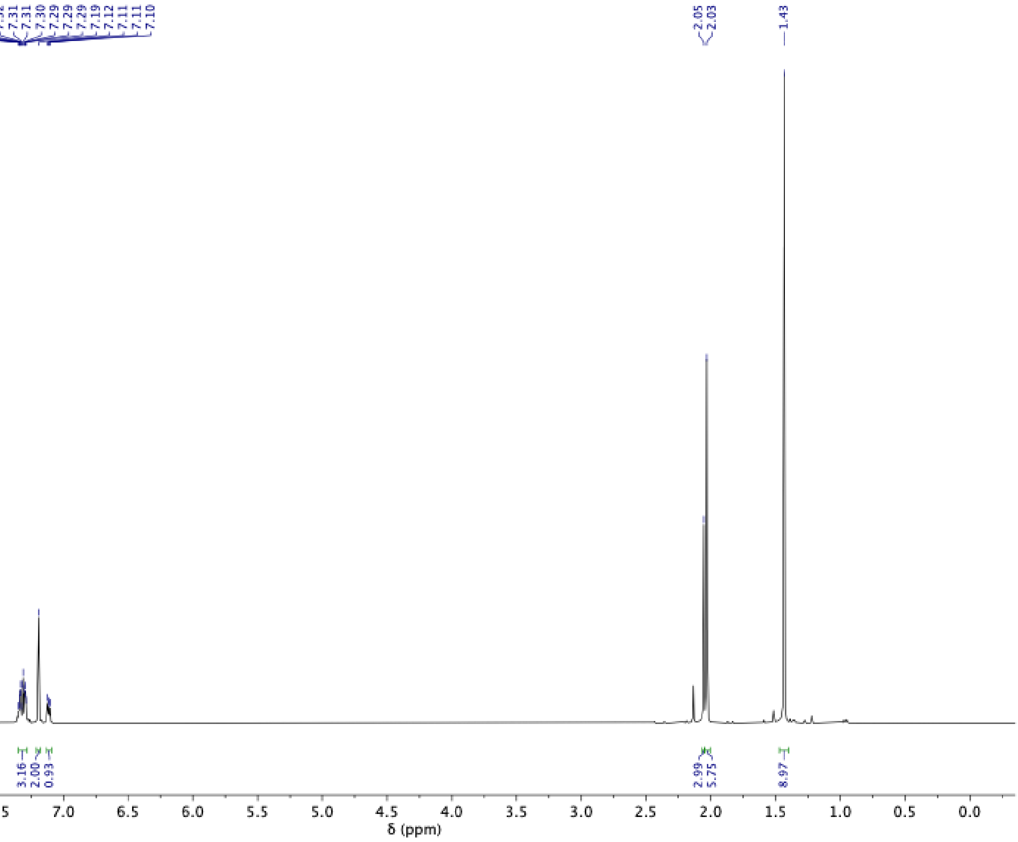
7.35 7.34 7.34 7.34 7.33 7.33 7.32 7.31 7.31 7.30 7.29 7.29 7.29 7.19 7.12 7.11 7.11 7.10
2.05 2.03
1.43
3.16 2.00 0.93
2.99 5.75
8.97
8.5 8.0 7.5 7.0 6.5 6.0 5.5 5.0 4.5 4.0 3.5 3.0 2.5 2.0 1.5 1.0 0.5 0.0
δ (ppm)

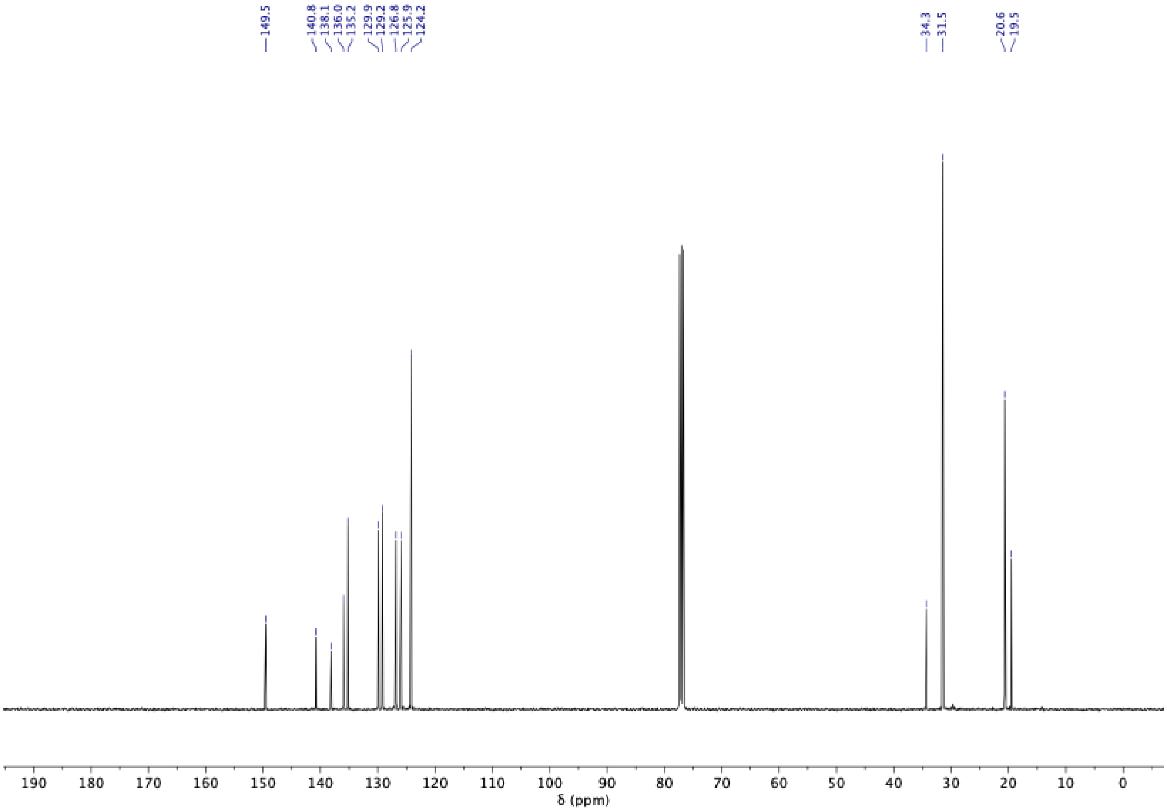
149.5
140.8 138.1 136.0 135.2 129.9 129.2 126.8 125.9 124.2
34.3 31.5
20.6 19.5
190 180 170 160 150 140 130 120 110 100 90 80 70 60 50 40 30 20 10 0
δ (ppm)

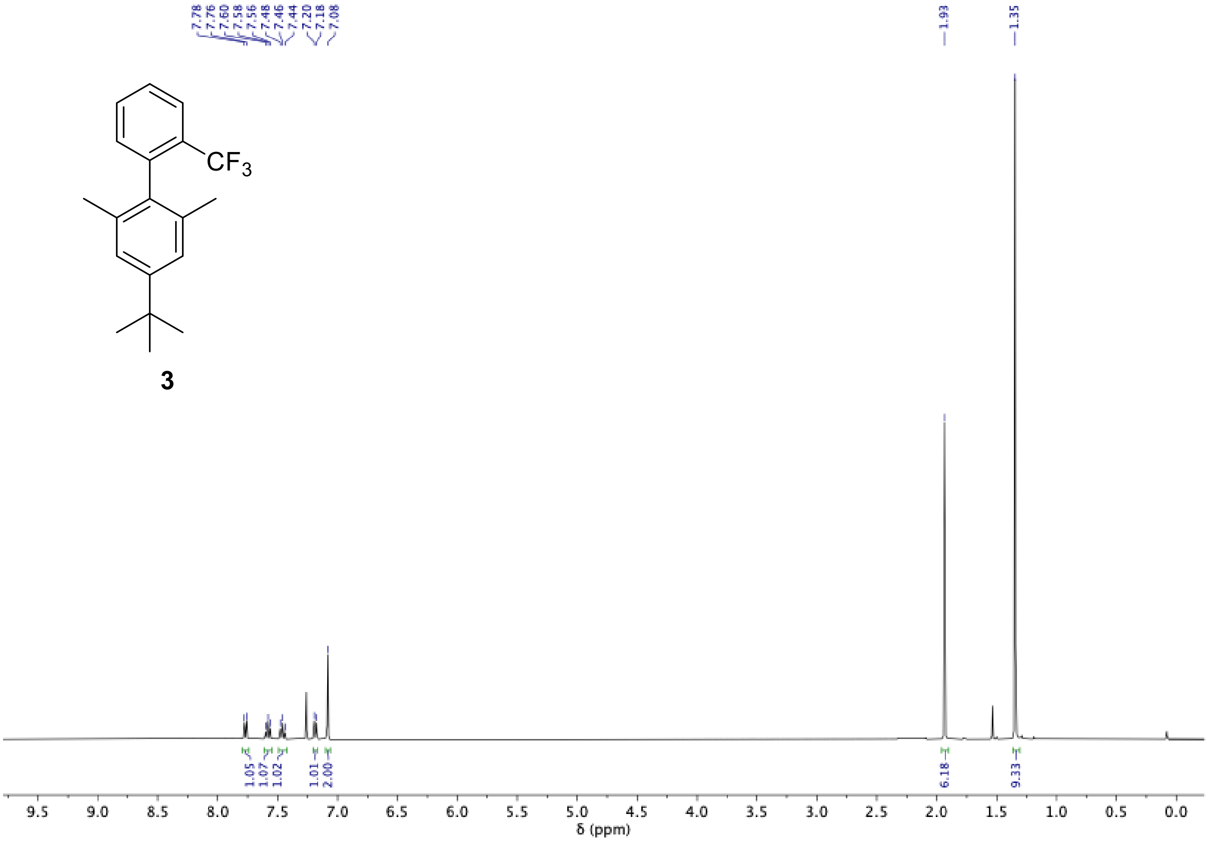
CF3
3
7.78
7.76
7.60
7.58
7.56
7.48
7.46
7.44
7.20
7.18
7.08
1.93
1.35
1.05
1.07
1.02
1.01
2.00
6.18
9.33
9.5 9.0 8.5 8.0 7.5 7.0 6.5 6.0 5.5 5.0 4.5 4.0 3.5 3.0 2.5 2.0 1.5 1.0 0.5 0.0
δ (ppm)

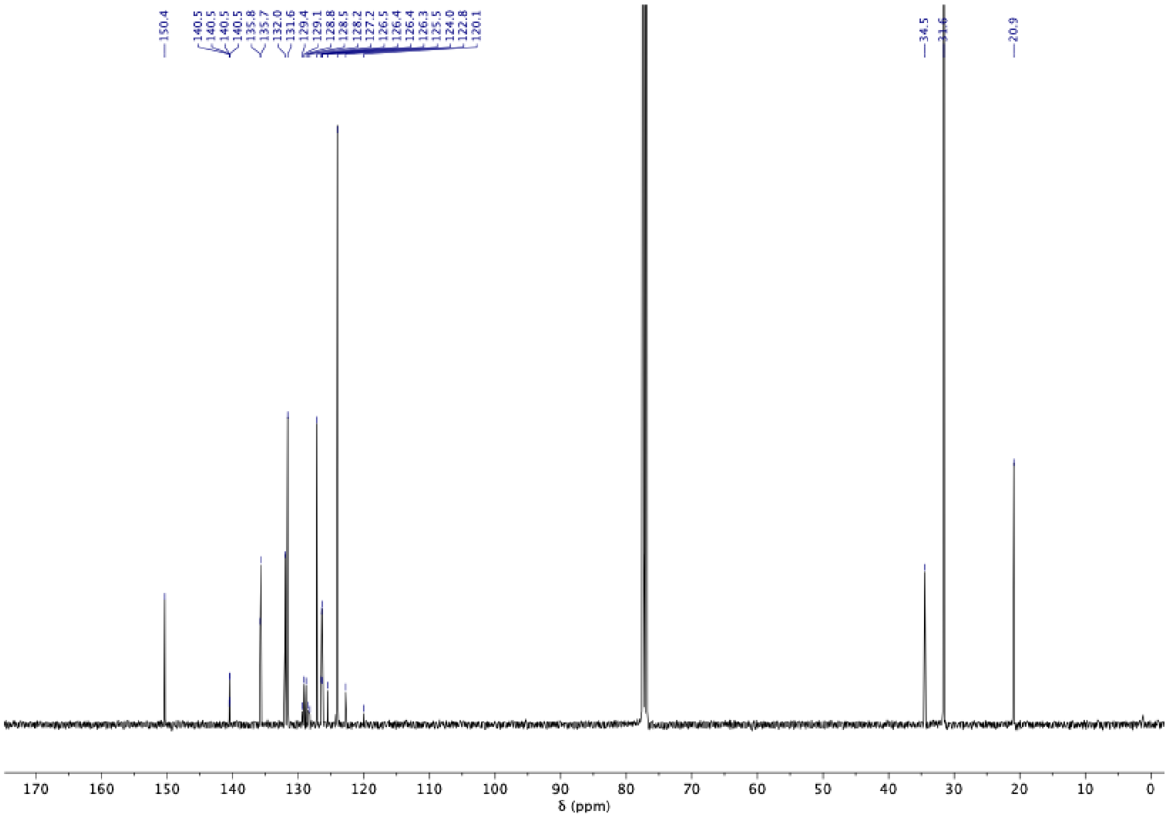
150.4
140.5
140.5
140.5
140.5
135.8
135.7
132.0
131.6
129.4
129.1
128.8
128.5
128.2
127.2
126.5
126.4
126.3
125.5
124.0
122.8
120.1
34.5
31.6
20.9
170 160 150 140 130 120 110 100 90 80 70 60 50 40 30 20 10 0
δ (ppm)

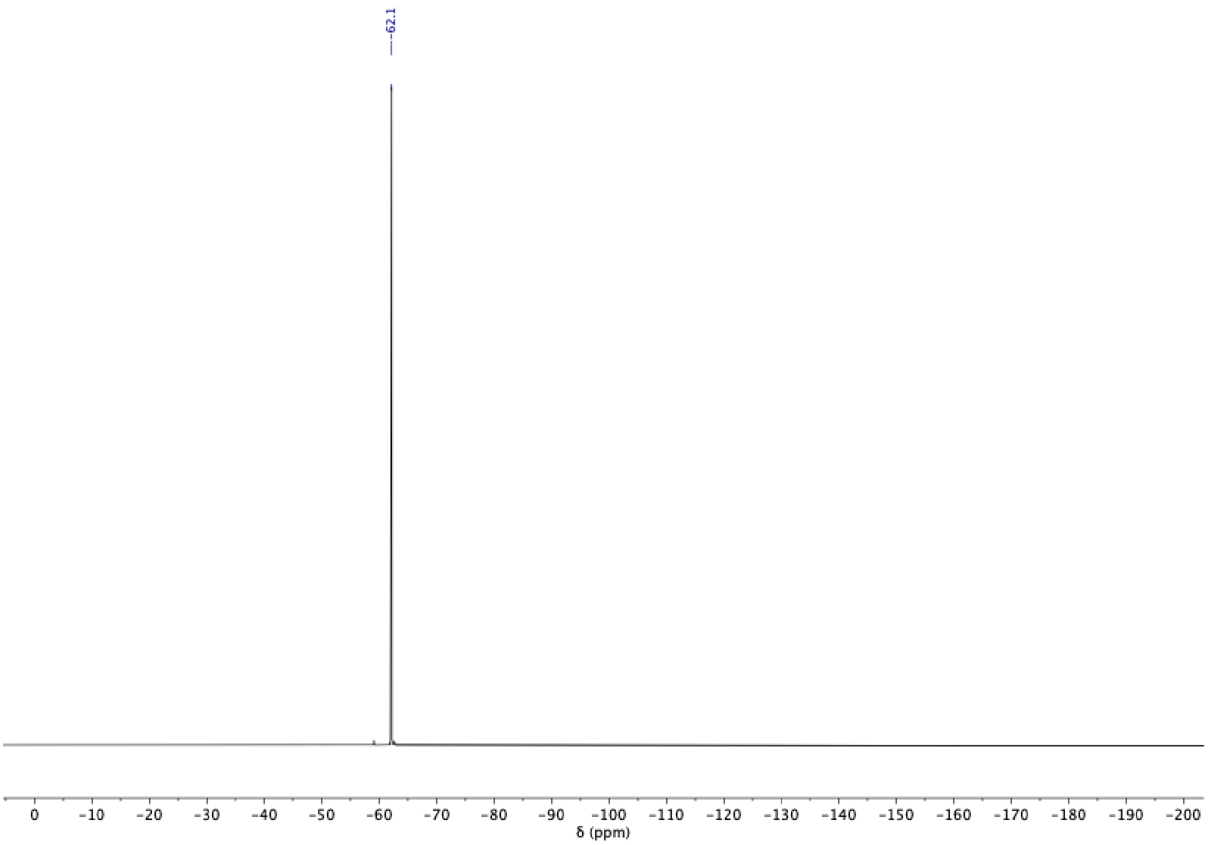
-62.1
0 -10 -20 -30 -40 -50 -60 -70 -80 -90 -100 -110 -120 -130 -140 -150 -160 -170 -180 -190 -200
δ (ppm)

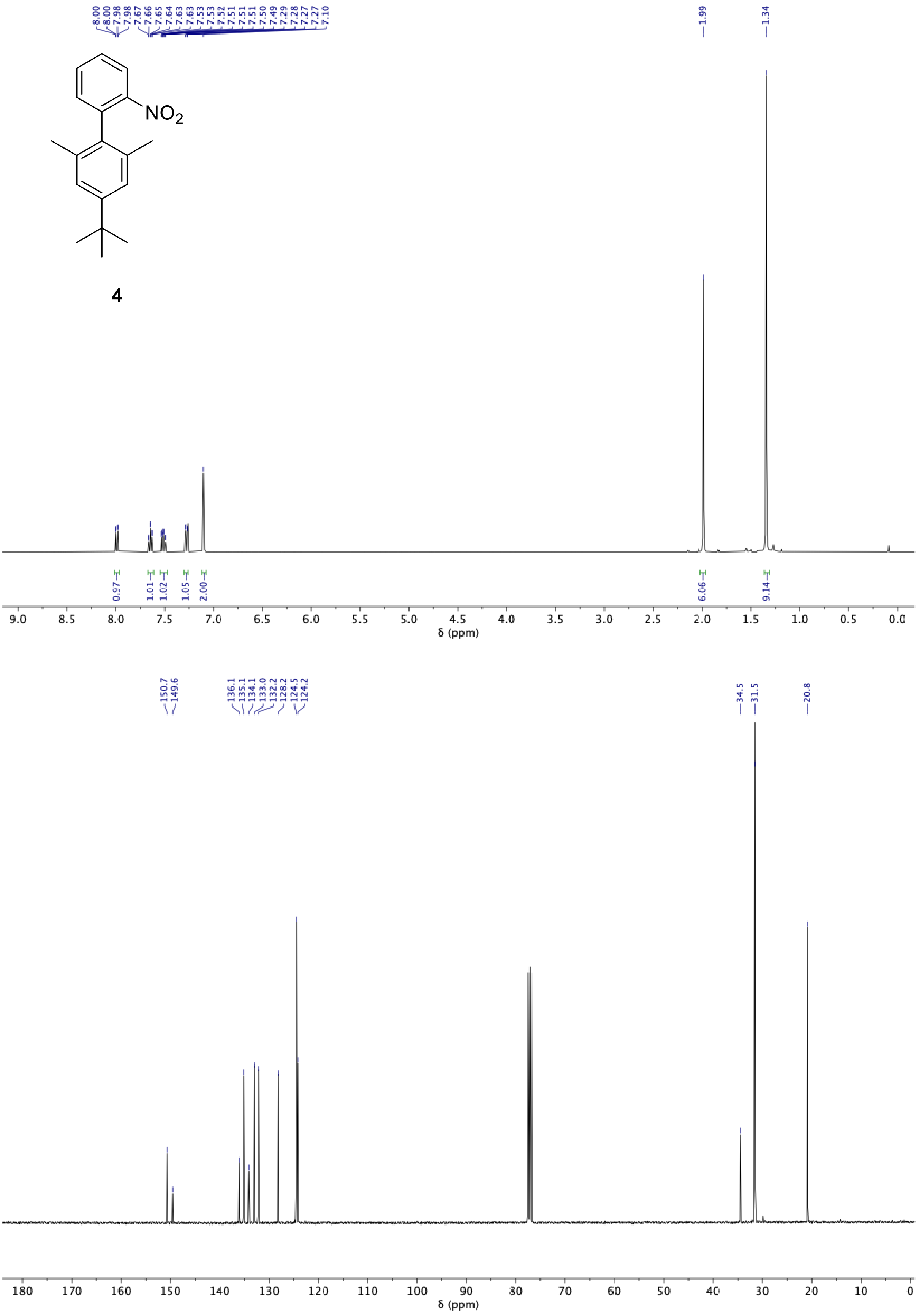
NO2
4
8.00
8.00
7.98
7.98
7.67
7.66
7.65
7.64
7.64
7.63
7.63
7.53
7.53
7.52
7.51
7.51
7.51
7.50
7.49
7.29
7.28
7.27
7.27
7.10
1.99
1.34
0.97
1.01
1.02
1.05
2.00
6.06
9.14
δ (ppm)
150.7
149.6
136.1
135.1
134.1
133.0
132.2
128.2
124.5
124.2
34.5
31.5
20.8
δ (ppm)

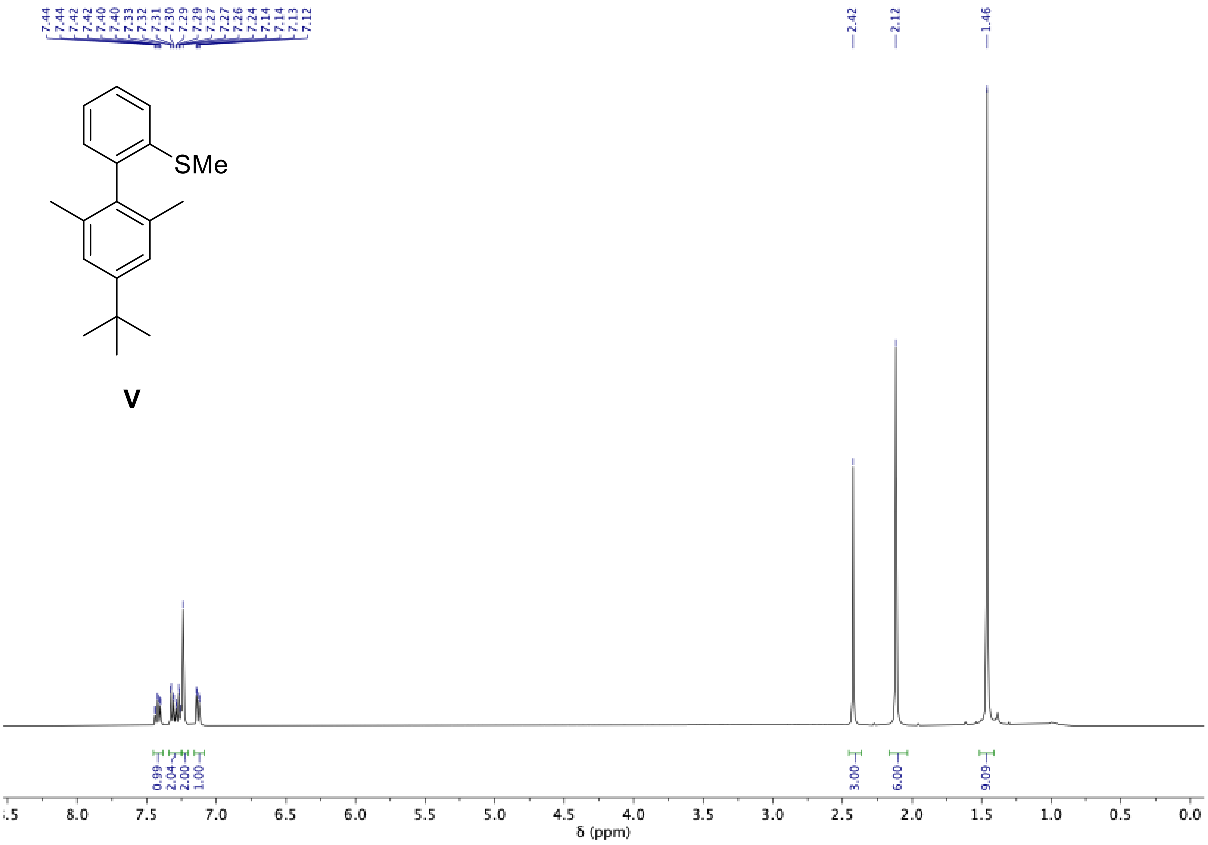
SMe
V

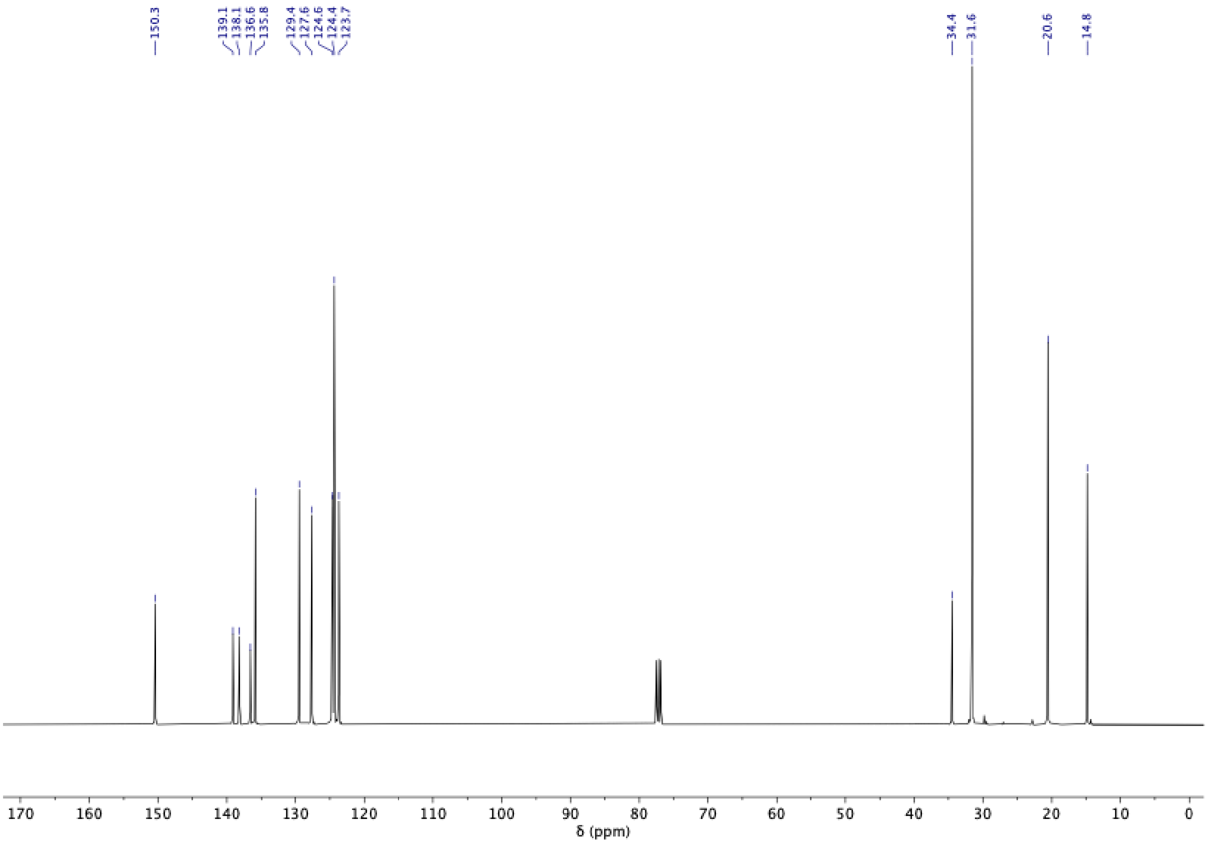

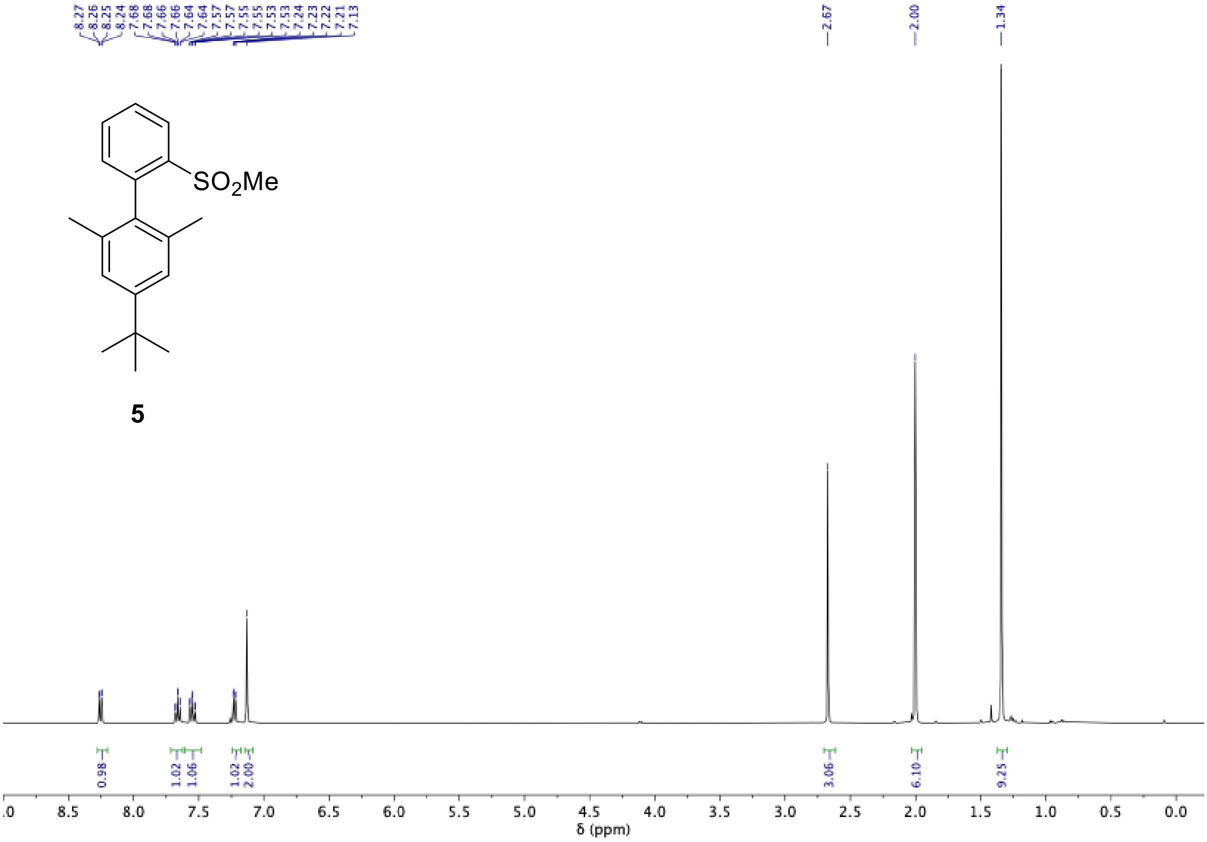
SO2Me
8.27
8.26
8.25
8.24
7.68
7.66
7.66
7.64
7.64
7.57
7.55
7.55
7.53
7.53
7.24
7.23
7.22
7.21
7.13
2.67
2.00
1.34
0.98
1.02
1.06
1.02
2.00
3.06
6.10
9.25
δ (ppm)

5

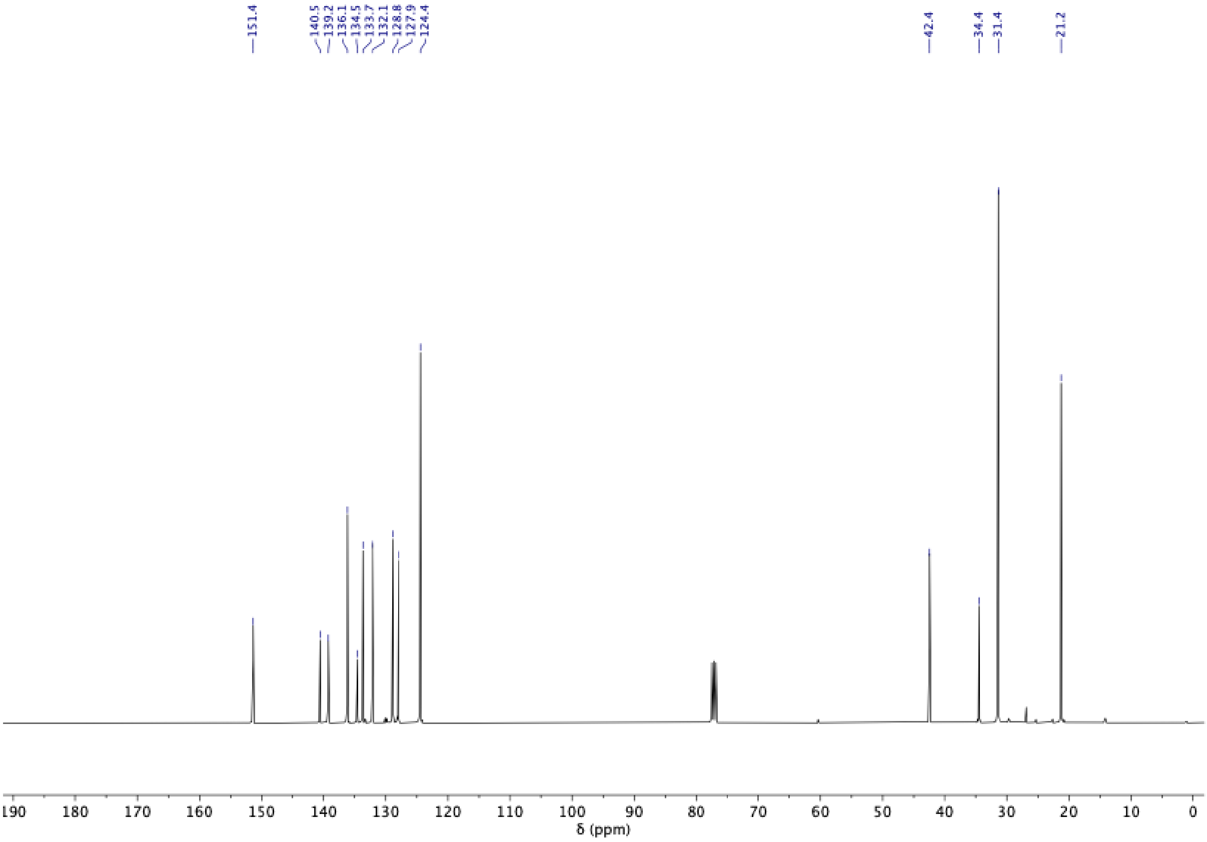
151.4
140.5
139.2
136.1
134.5
133.7
132.1
128.8
127.9
124.4
42.4
34.4
31.4
21.2
δ (ppm)

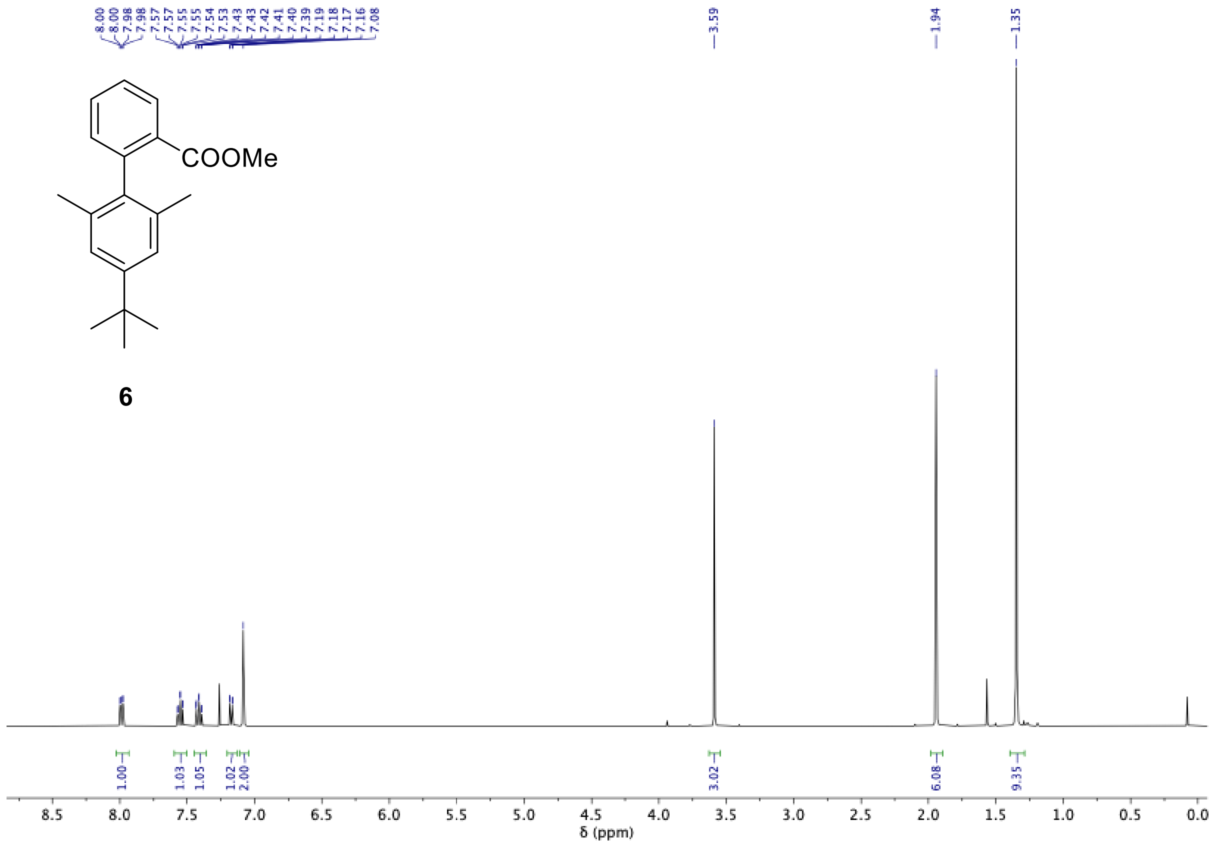
8.00
8.00
7.98
7.98
7.57
7.57
7.55
7.55
7.54
7.53
7.43
7.43
7.42
7.41
7.40
7.39
7.19
7.18
7.17
7.16
7.08
3.59
1.94
1.35
COOMe
6
1.00
1.03
1.05
1.02
2.00
3.02
6.08
9.35
8.5 8.0 7.5 7.0 6.5 6.0 5.5 5.0 4.5 4.0 3.5 3.0 2.5 2.0 1.5 1.0 0.5 0.0
δ (ppm)

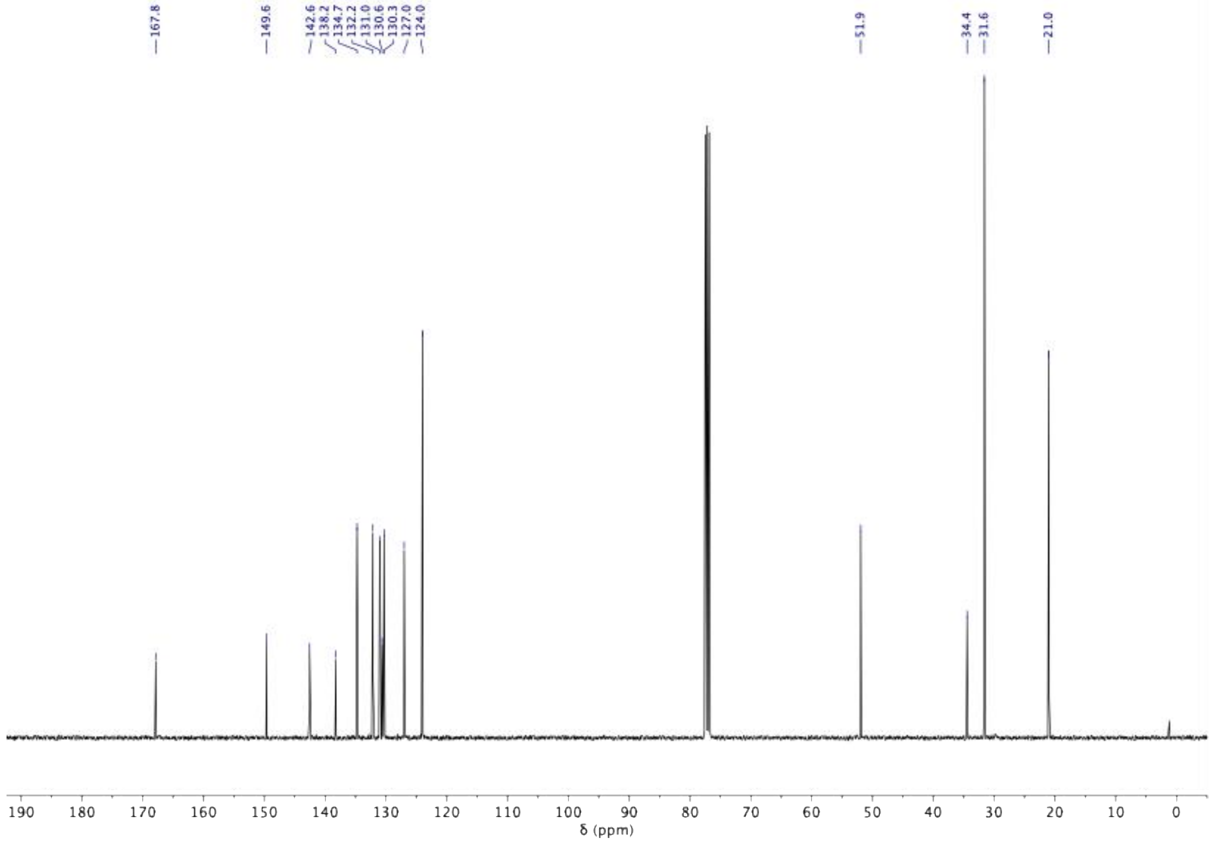
167.8
149.6
142.6
138.2
134.7
132.2
131.0
130.6
130.3
127.0
124.0
51.9
34.4
31.6
21.0
190 180 170 160 150 140 130 120 110 100 90 80 70 60 50 40 30 20 10 0
δ (ppm)

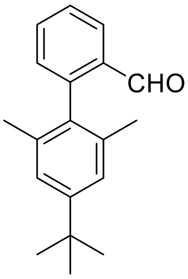

VII

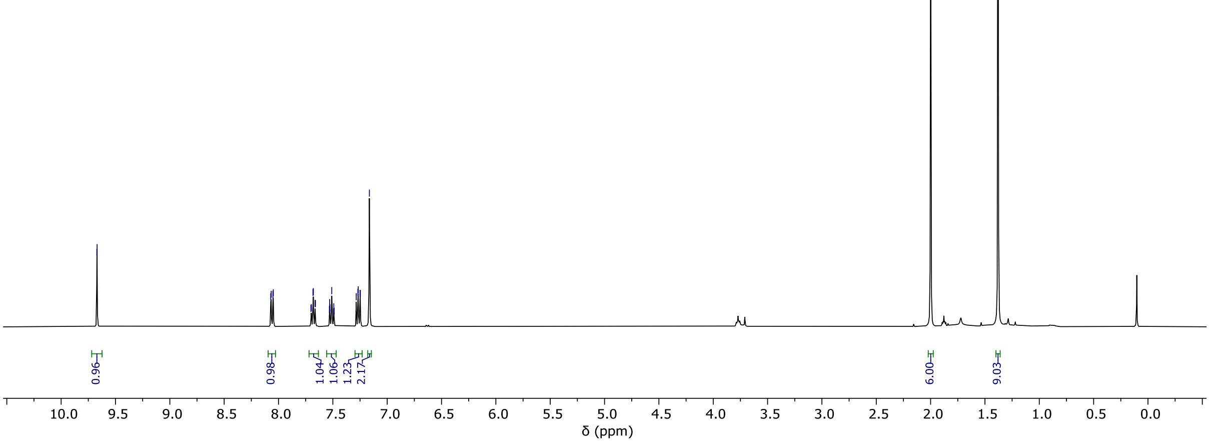
9.67
9.67
8.07
8.07
8.05
8.05
7.70
7.70
7.68
7.68
7.66
7.66
7.53
7.53
7.51
7.49
7.49
7.28
7.27
7.27
7.25
7.25
7.16
2.00
1.38
0.96
0.98
1.04
1.06
1.23
2.17
6.00
9.03
δ (ppm)

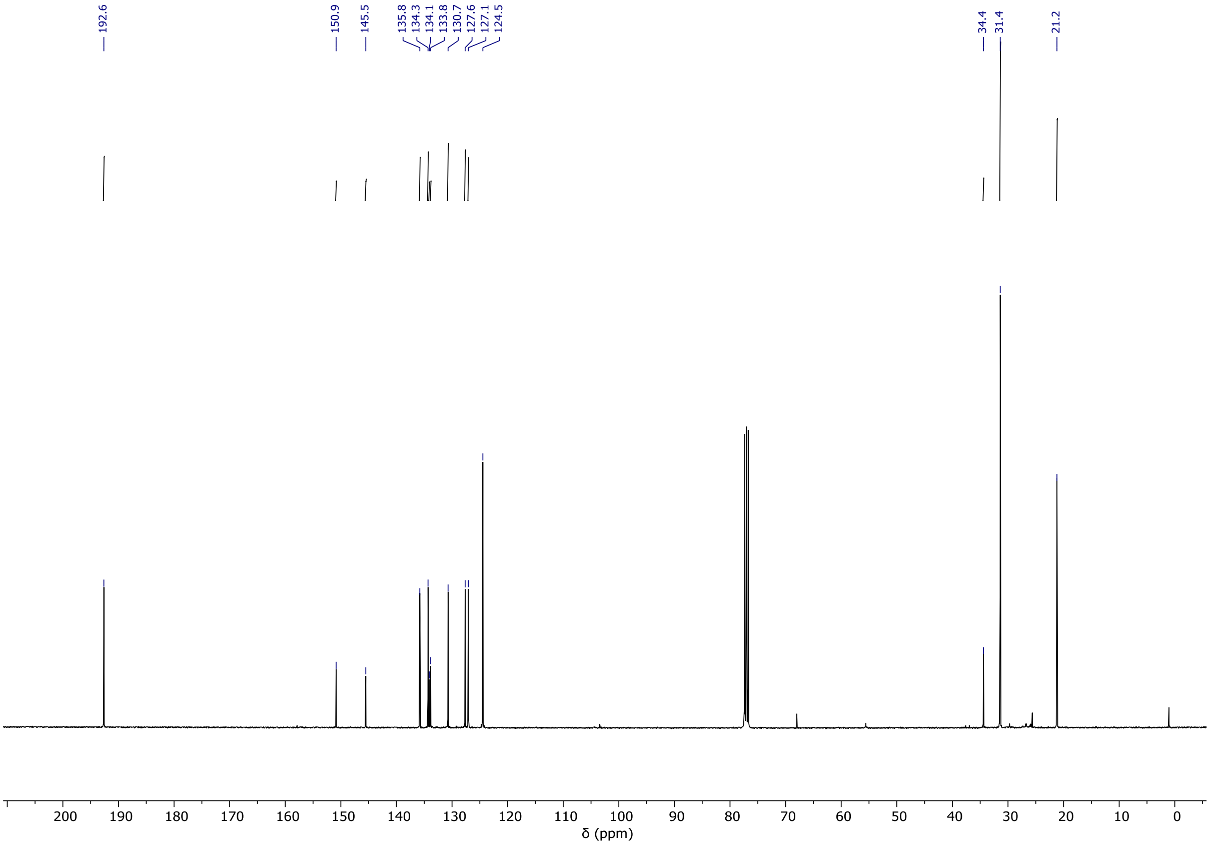
192.6
150.9
145.5
135.8
134.3
134.1
133.8
130.7
127.6
127.1
124.5
34.4
31.4
21.2
δ (ppm)

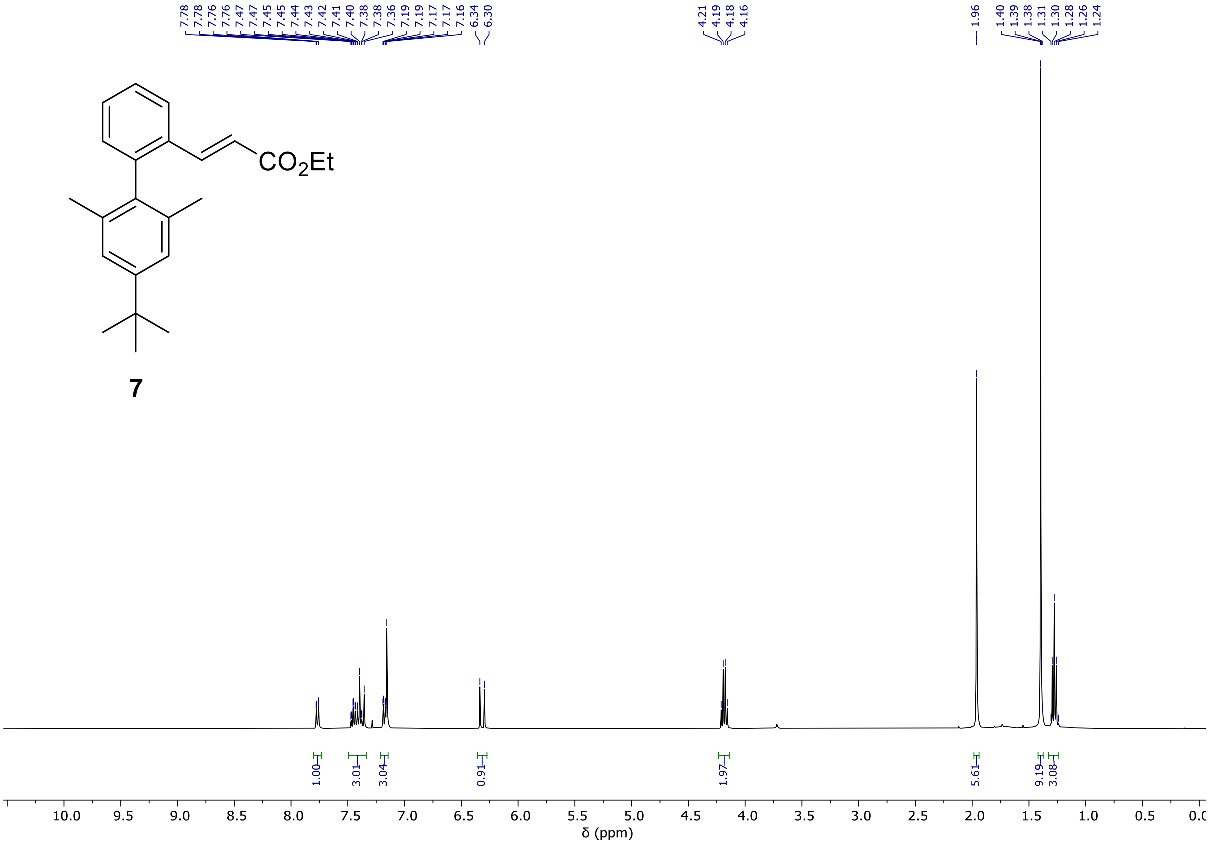
CO2Et
7
7.78
7.78
7.76
7.76
7.47
7.47
7.45
7.45
7.44
7.43
7.42
7.41
7.40
7.38
7.38
7.36
7.19
7.19
7.17
7.17
7.16
6.34
6.30
4.21
4.19
4.18
4.16
1.96
1.40
1.39
1.38
1.31
1.30
1.28
1.26
1.24
1.00
3.01
3.04
0.91
1.97
5.61
9.19
3.08
10.0 9.5 9.0 8.5 8.0 7.5 7.0 6.5 6.0 5.5 5.0 4.5 4.0 3.5 3.0 2.5 2.0 1.5 1.0 0.5 0.0
δ (ppm)

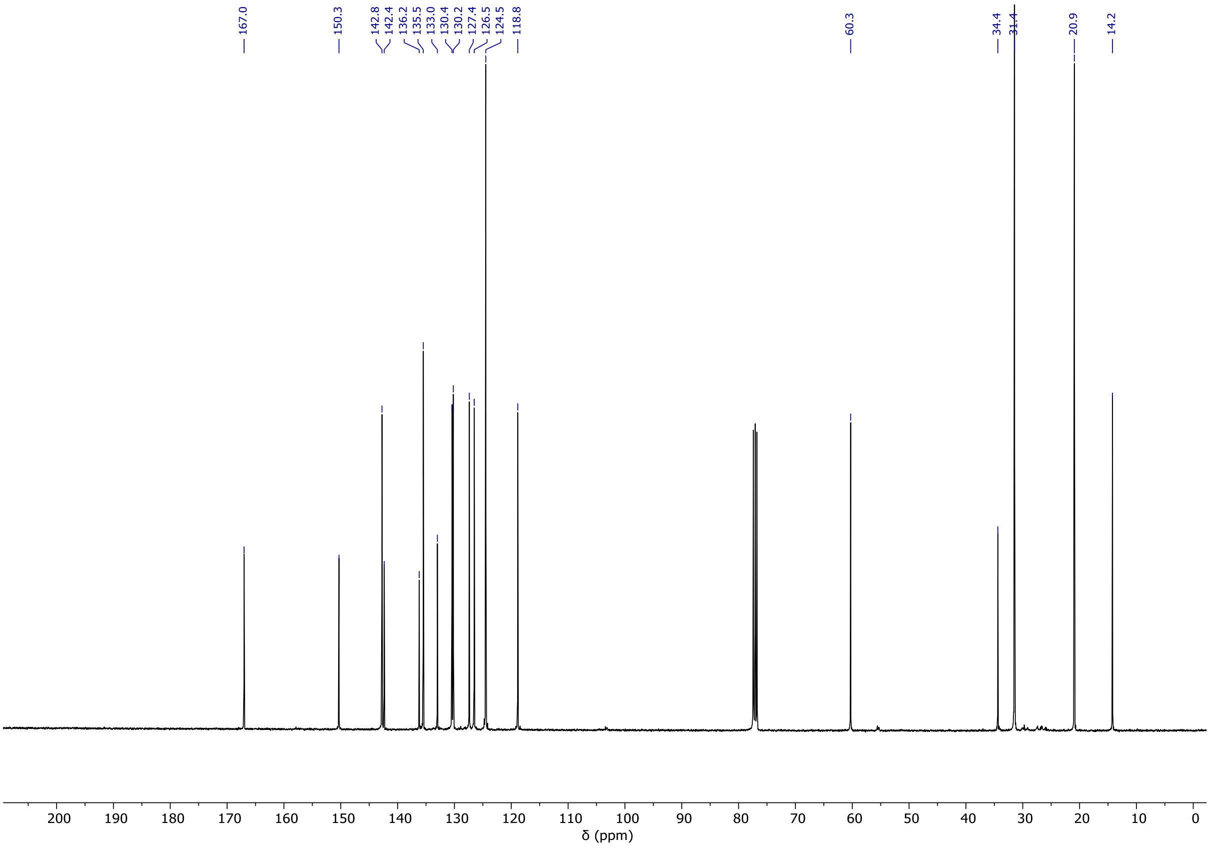
167.0
150.3
142.8
142.4
136.2
135.5
133.0
130.4
130.2
127.4
126.5
124.5
118.8
60.3
34.4
31.4
20.9
14.2
200 190 180 170 160 150 140 130 120 110 100 90 80 70 60 50 40 30 20 10 0
δ (ppm)

8

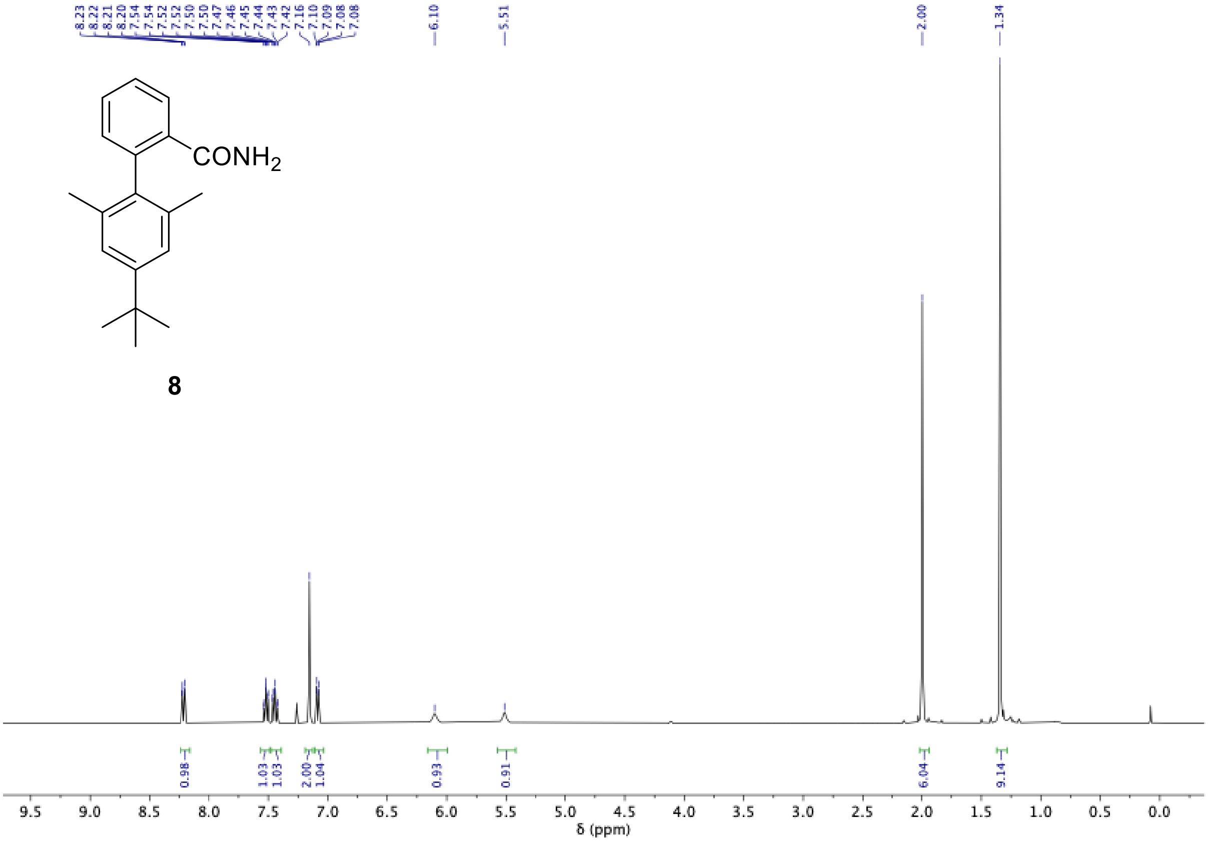
CONH2
8.23
8.22
8.21
8.20
7.54
7.54
7.52
7.52
7.50
7.50
7.47
7.46
7.45
7.44
7.43
7.42
7.16
7.10
7.09
7.08
7.08
6.10
5.51
2.00
1.34
0.98
1.03
1.03
2.00
1.04
0.93
0.91
6.04
9.14
9.5
9.0
8.5
8.0
7.5
7.0
6.5
6.0
5.5
5.0
4.5
4.0
3.5
3.0
2.5
2.0
1.5
1.0
0.5
0.0
δ (ppm)

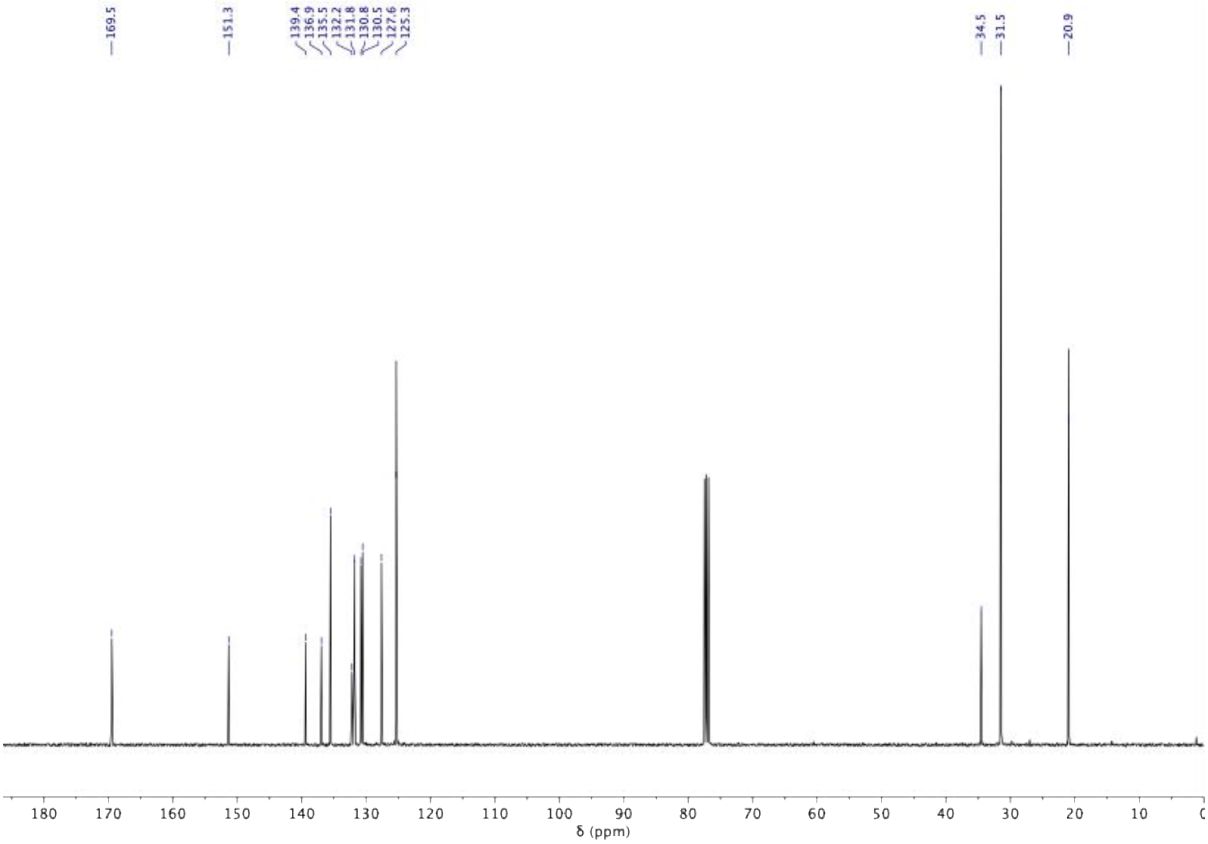
169.5
151.3
139.4
136.9
135.5
132.2
131.8
130.8
130.5
127.6
125.3
34.5
31.5
20.9
180
170
160
150
140
130
120
110
100
90
80
70
60
50
40
30
20
10
δ (ppm)

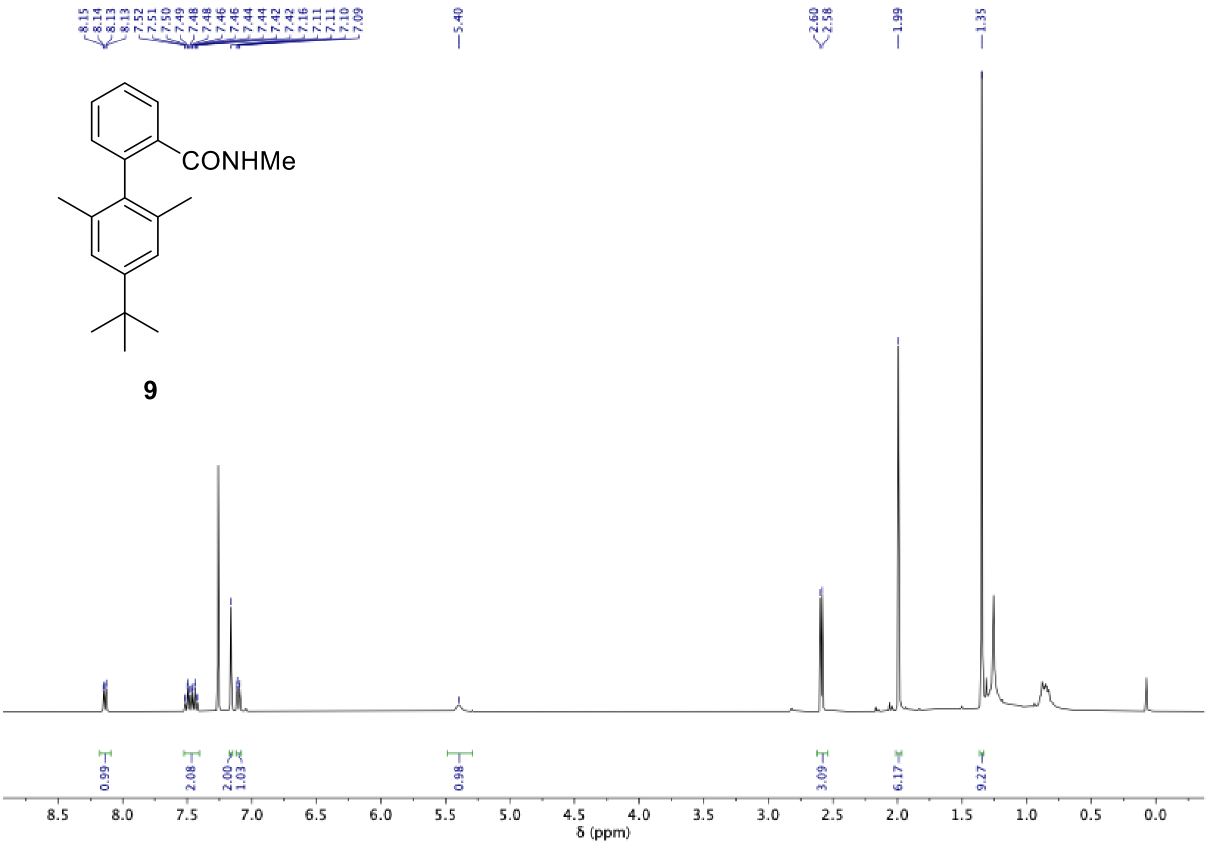
CONHMe
9
8.15
8.14
8.13
8.13
7.52
7.51
7.50
7.49
7.48
7.48
7.46
7.46
7.44
7.44
7.42
7.42
7.16
7.11
7.11
7.10
7.09
5.40
2.60
2.58
1.99
1.35
0.99
2.08
2.00
1.03
0.98
3.09
6.17
9.27
8.5
8.0
7.5
7.0
6.5
6.0
5.5
5.0
4.5
4.0
3.5
3.0
2.5
2.0
1.5
1.0
0.5
0.0
δ (ppm)

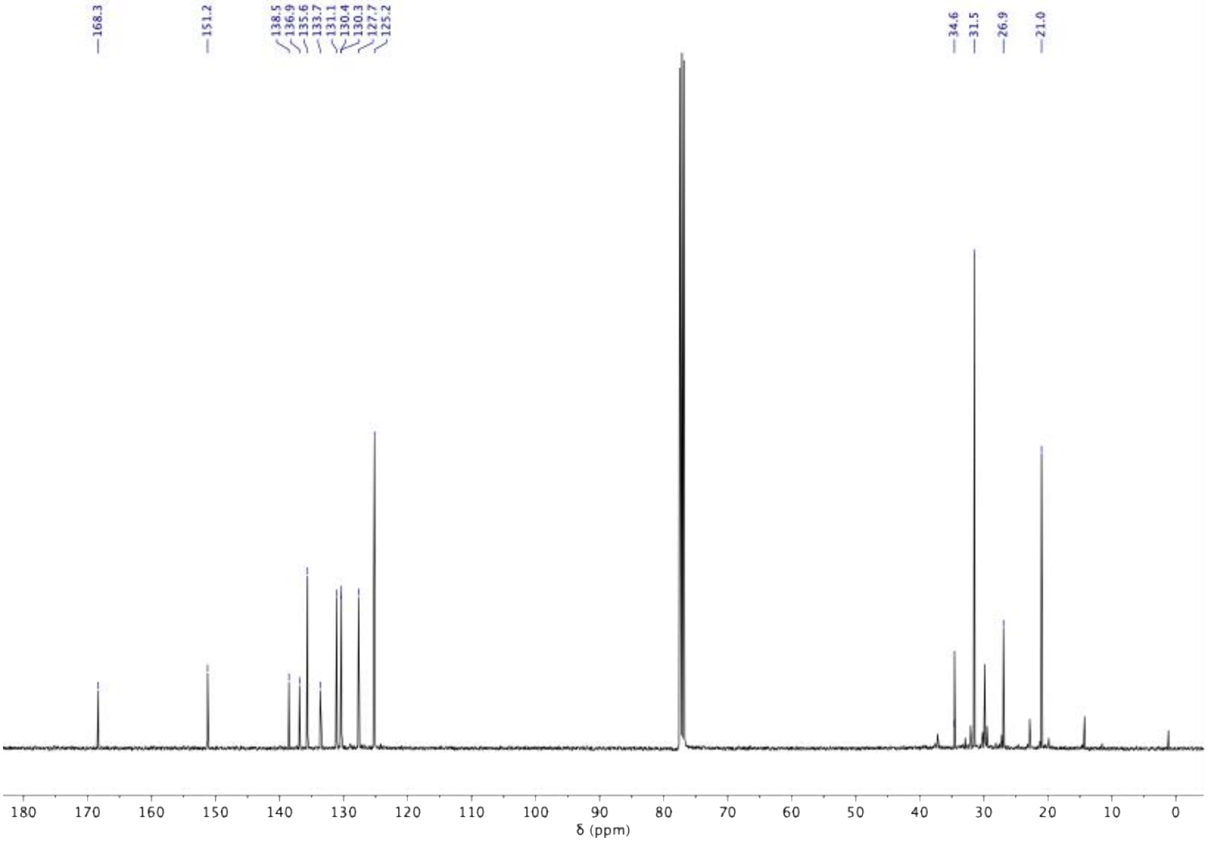
168.3
151.2
138.5
136.9
135.6
133.7
131.1
130.4
130.3
127.7
125.2
34.6
31.5
26.9
21.0
180
170
160
150
140
130
120
110
100
90
80
70
60
50
40
30
20
10
0
δ (ppm)

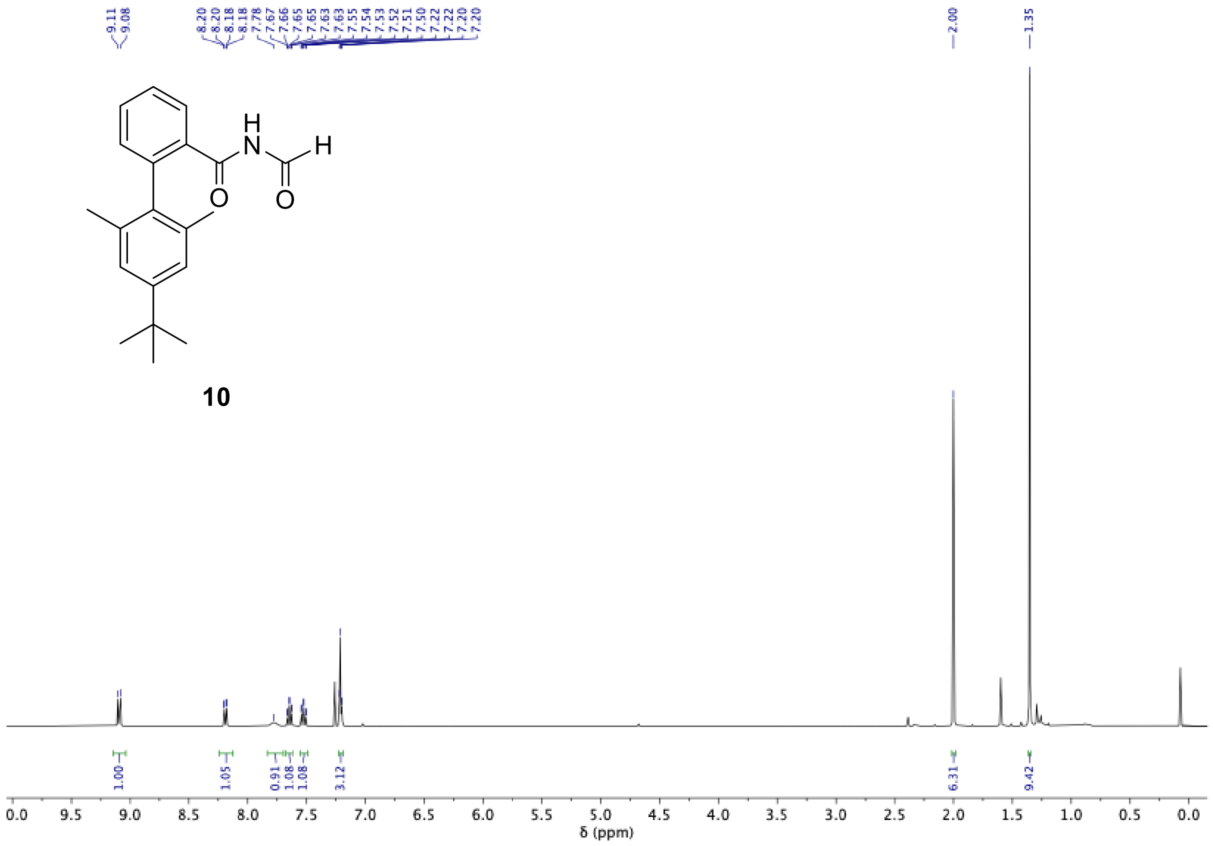
10
9.11
9.08
8.20
8.20
8.18
8.18
7.78
7.67
7.66
7.65
7.65
7.63
7.63
7.55
7.54
7.53
7.52
7.51
7.50
7.22
7.22
7.20
7.20
2.00
1.35
1.00
1.05
0.91
1.08
1.08
3.12
6.31
9.42
δ (ppm)

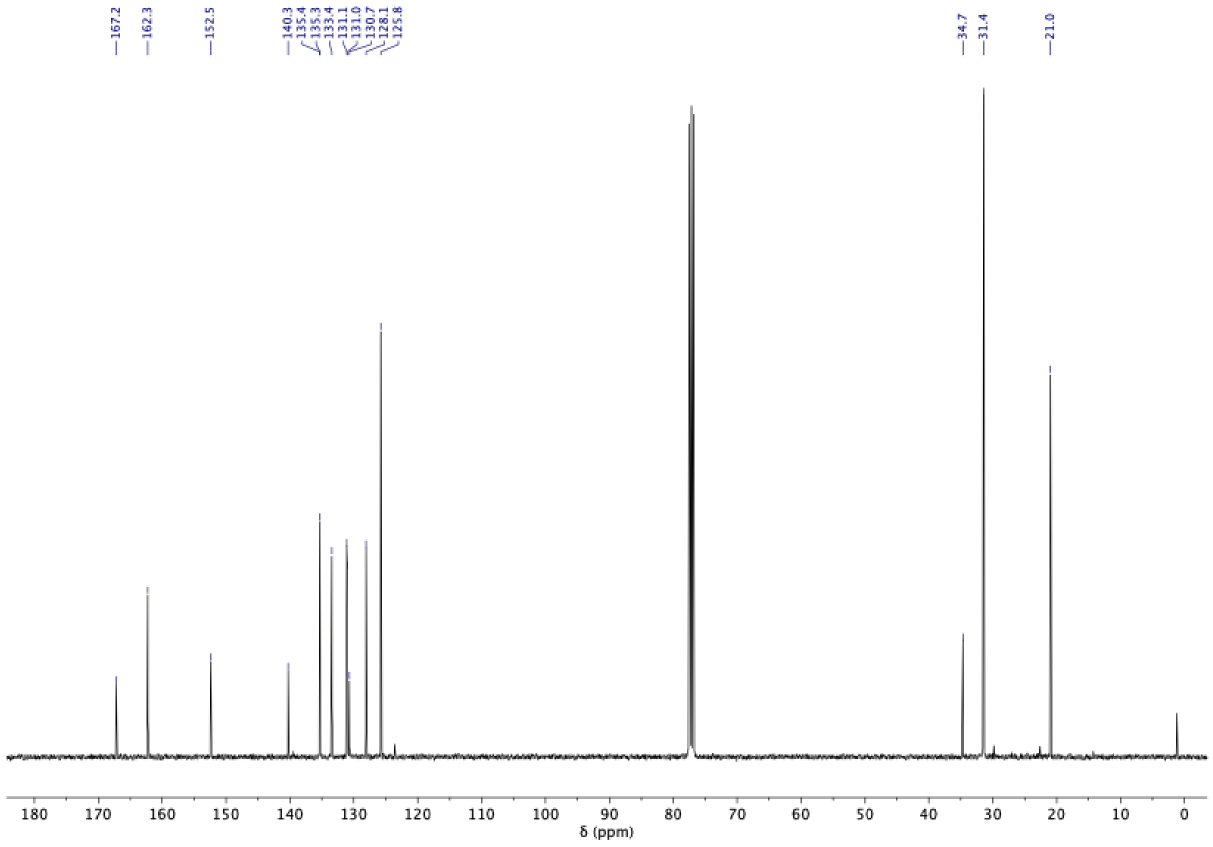
167.2
162.3
152.5
140.3
135.4
135.3
133.4
131.1
131.0
130.7
128.1
125.8
34.7
31.4
21.0
δ (ppm)

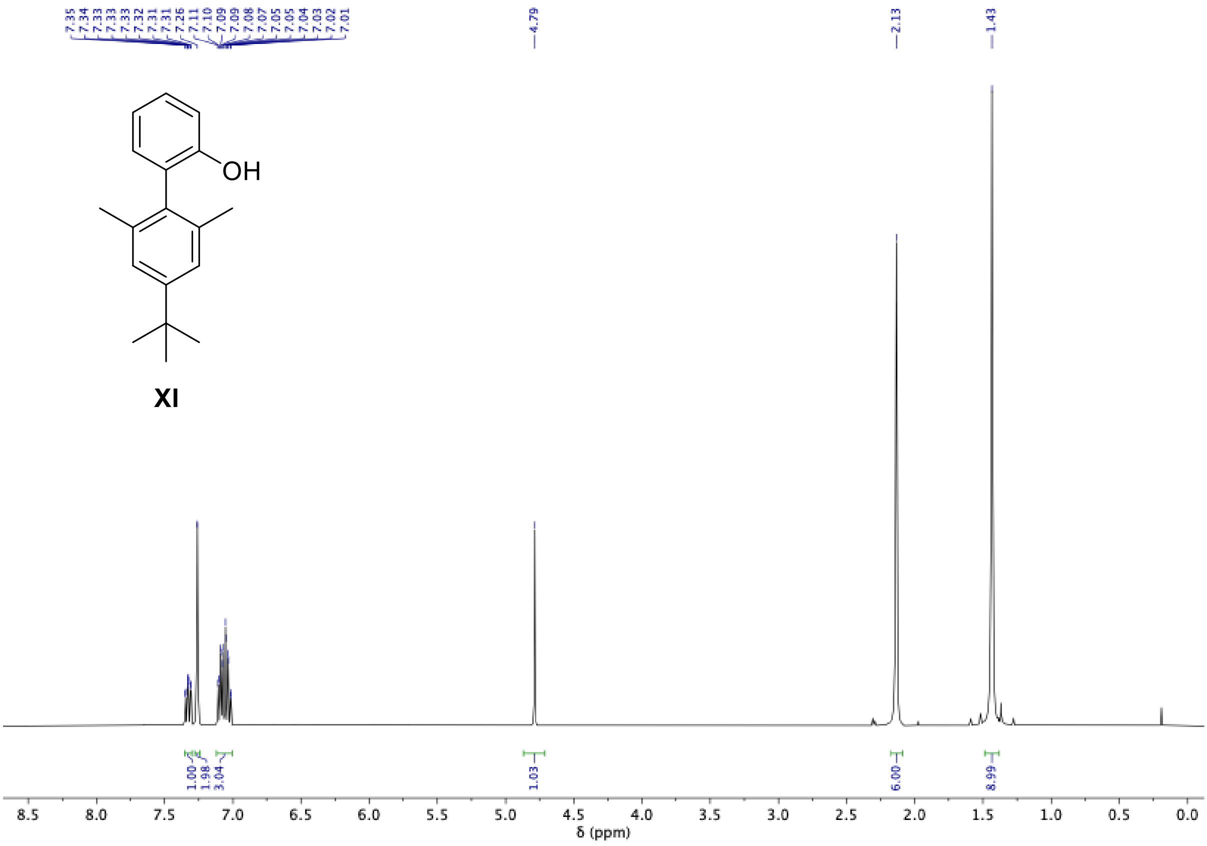
7.35 7.34 7.33 7.33 7.33 7.32 7.31 7.31 7.26 7.11 7.10 7.09 7.09 7.08 7.07 7.05 7.05 7.04 7.03 7.02 7.01
4.79
2.13
1.43
OH
XI
1.00
1.98
3.04
1.03
6.00
8.99
8.5 8.0 7.5 7.0 6.5 6.0 5.5 5.0 4.5 4.0 3.5 3.0 2.5 2.0 1.5 1.0 0.5 0.0
δ (ppm)

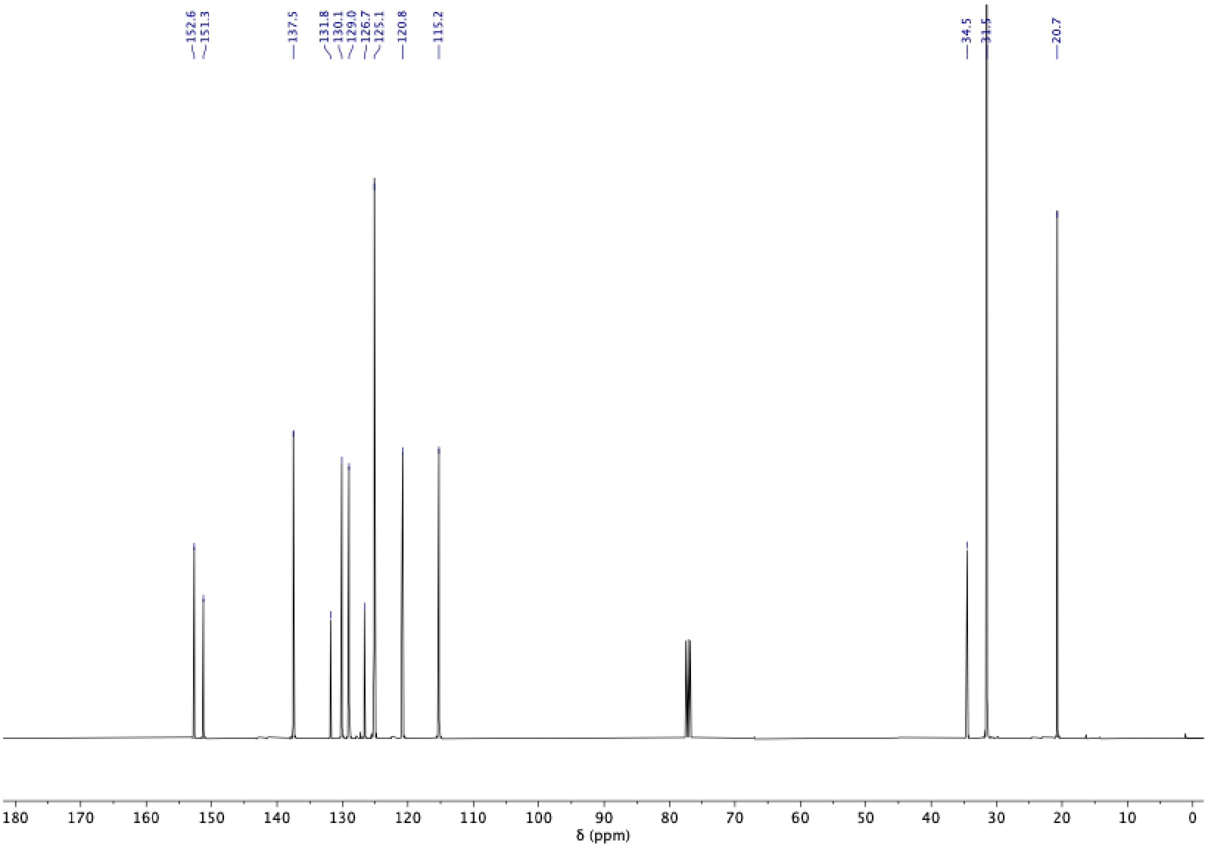
152.6
151.3
137.5
131.8
130.1
129.0
126.7
125.1
120.8
115.2
34.5
31.5
20.7
180 170 160 150 140 130 120 110 100 90 80 70 60 50 40 30 20 10 0
δ (ppm)

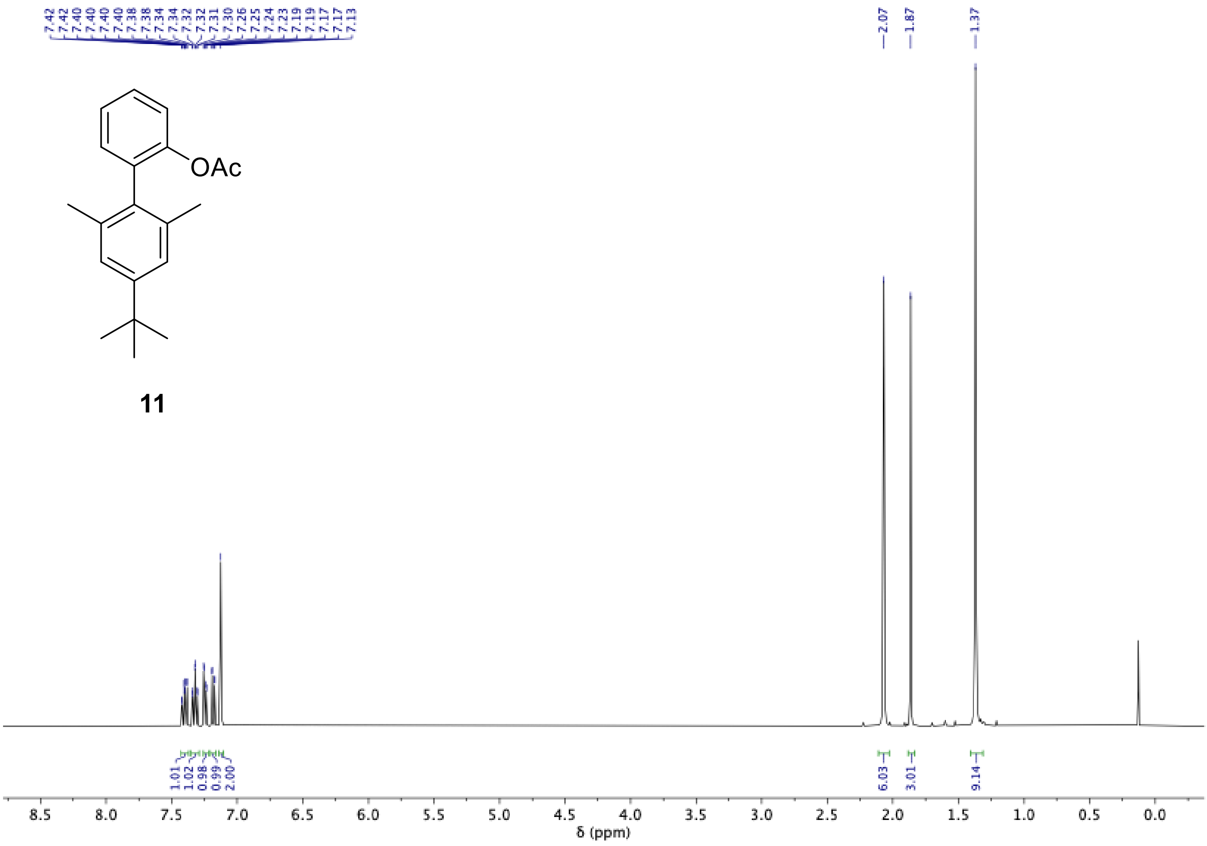

11

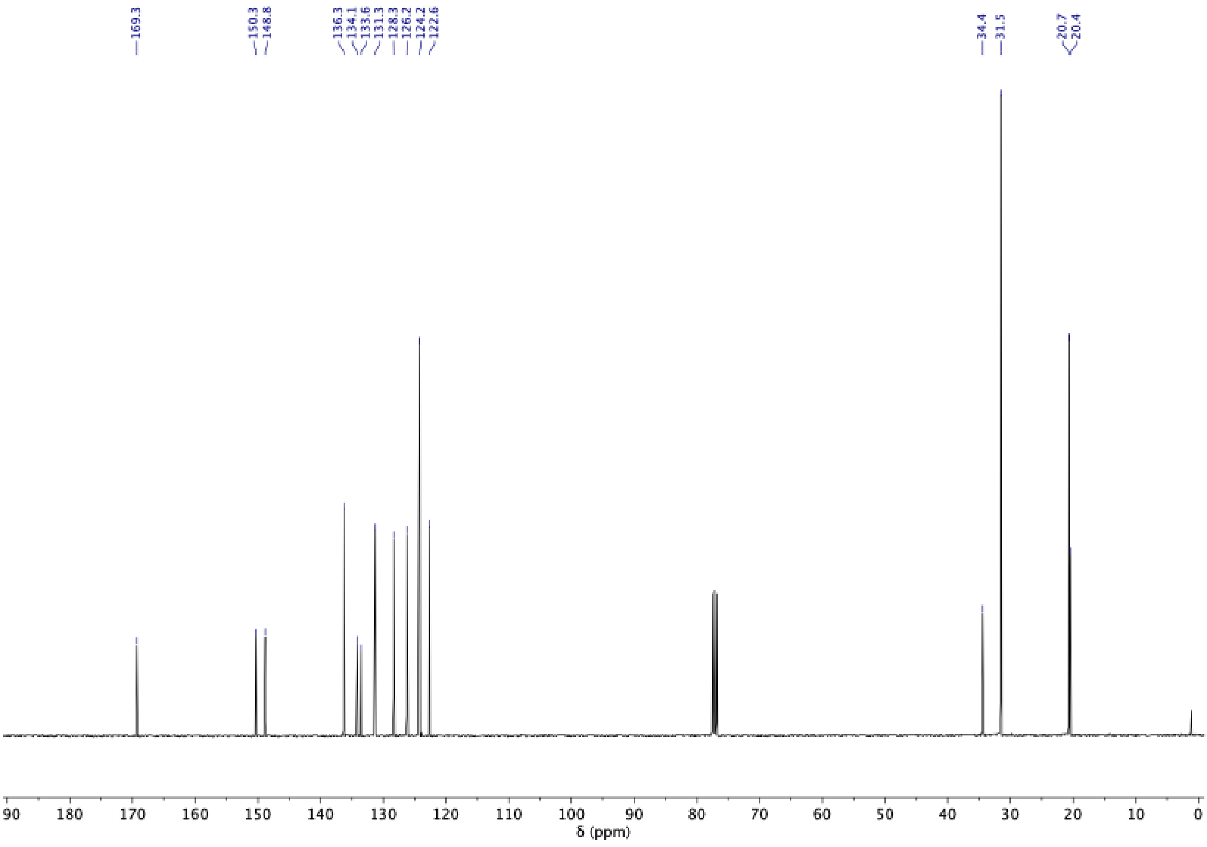

169.3
150.3
148.8
136.3
134.1
133.6
131.3
128.3
126.2
124.2
122.6
34.4
31.5
20.7
20.4
δ (ppm)

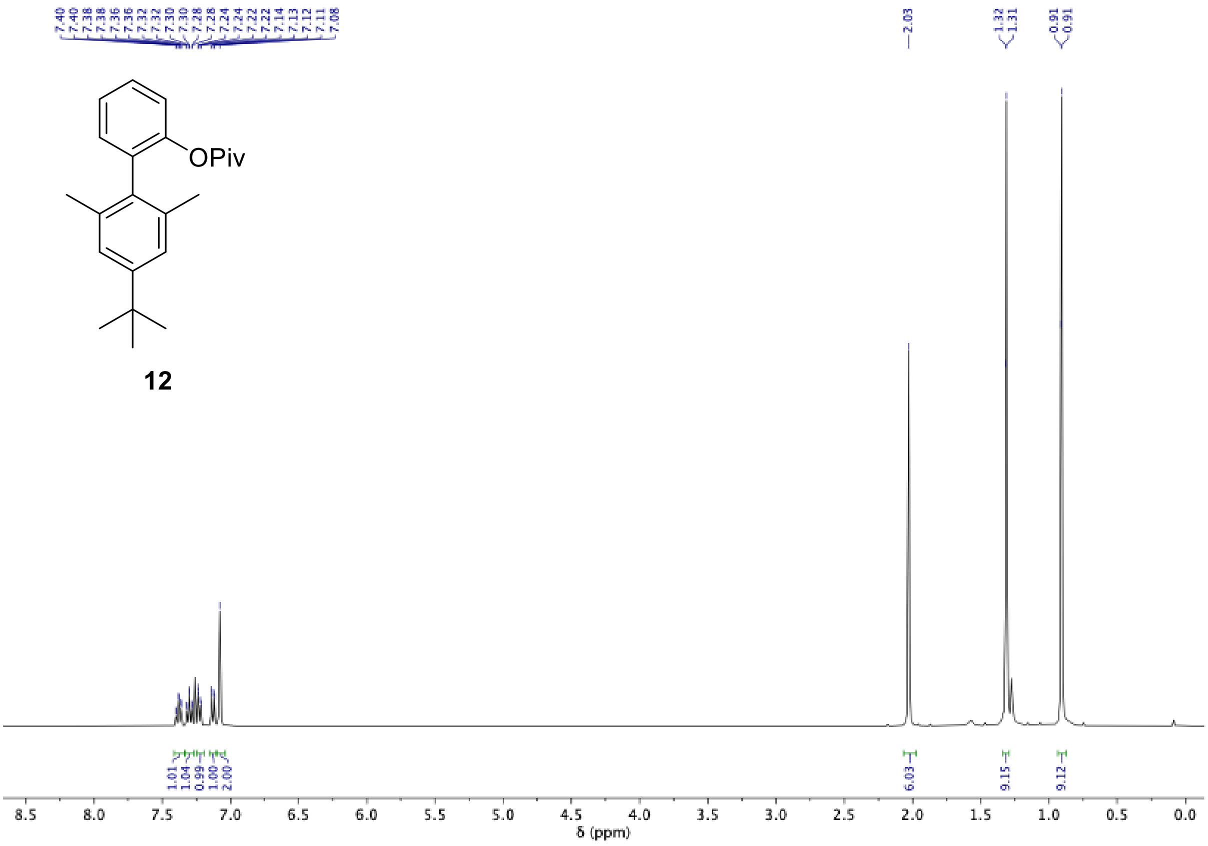
7.40
7.40
7.38
7.38
7.36
7.36
7.32
7.32
7.30
7.30
7.28
7.28
7.24
7.24
7.22
7.22
7.14
7.13
7.12
7.11
7.08
2.03
1.32
1.31
0.91
0.91
OPiv
1.01
1.04
0.99
1.00
2.00
6.03
9.15
9.12
8.5
8.0
7.5
7.0
6.5
6.0
5.5
5.0
4.5
4.0
3.5
3.0
2.5
2.0
1.5
1.0
0.5
0.0
δ (ppm)

12

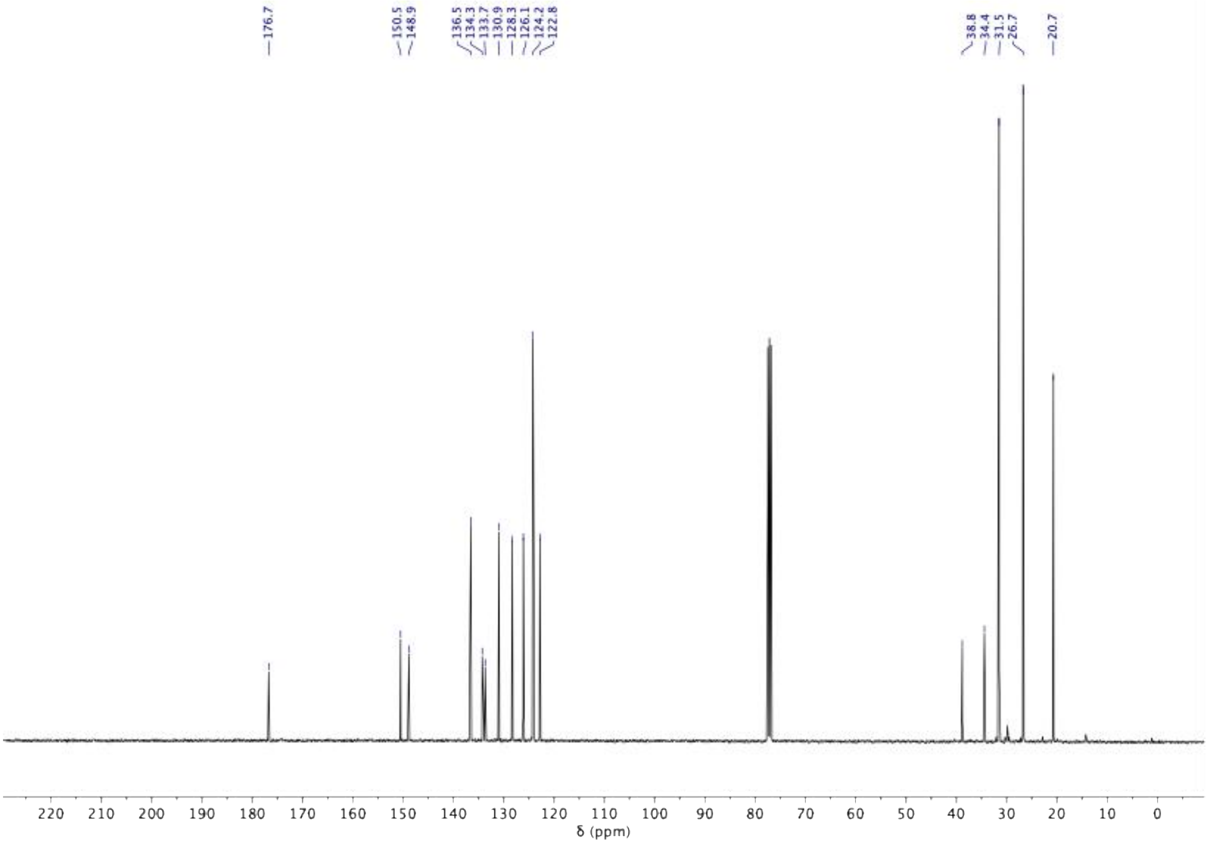
176.7
150.5
148.9
136.5
134.3
133.7
130.9
128.3
126.1
124.2
122.8
38.8
34.4
31.5
26.7
20.7
220
210
200
190
180
170
160
150
140
130
120
110
100
90
80
70
60
50
40
30
20
10
0
δ (ppm)

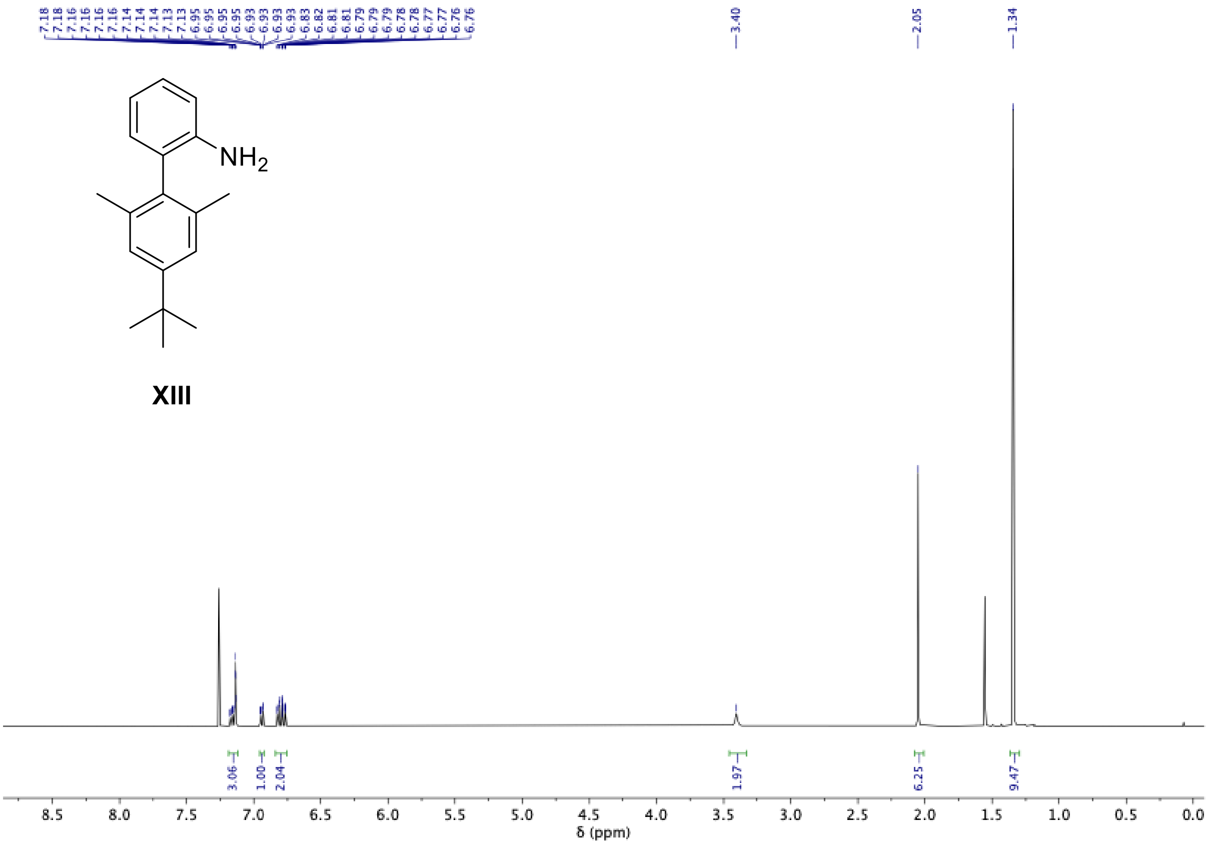
NH2
3.40
2.05
1.34
3.06
1.00
2.04
1.97
6.25
9.47
8.5 8.0 7.5 7.0 6.5 6.0 5.5 5.0 4.5 4.0 3.5 3.0 2.5 2.0 1.5 1.0 0.5 0.0
δ (ppm)

XIII

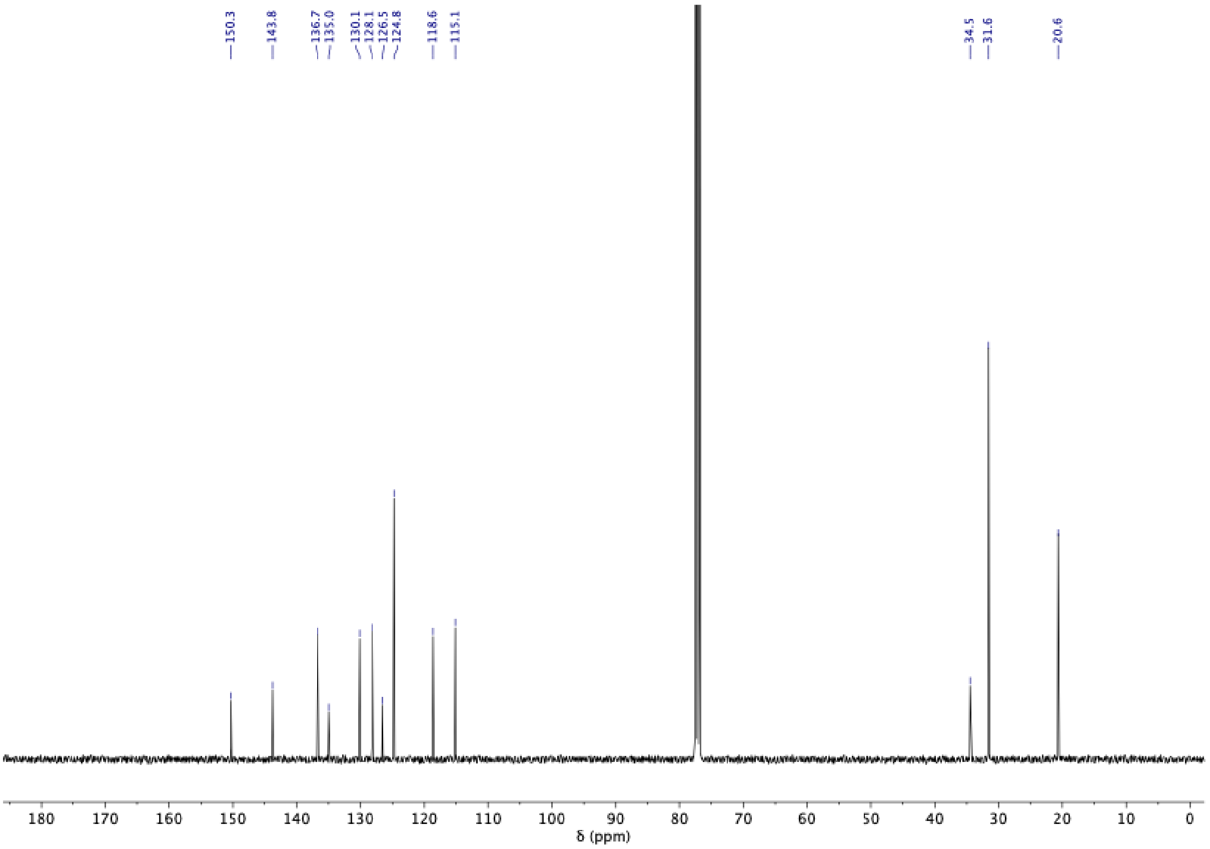
150.3
143.8
136.7
135.0
130.1
128.1
126.5
124.8
118.6
115.1
34.5
31.6
20.6
180 170 160 150 140 130 120 110 100 90 80 70 60 50 40 30 20 10 0
δ (ppm)

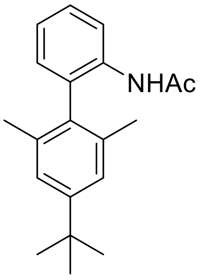
NHAc

13

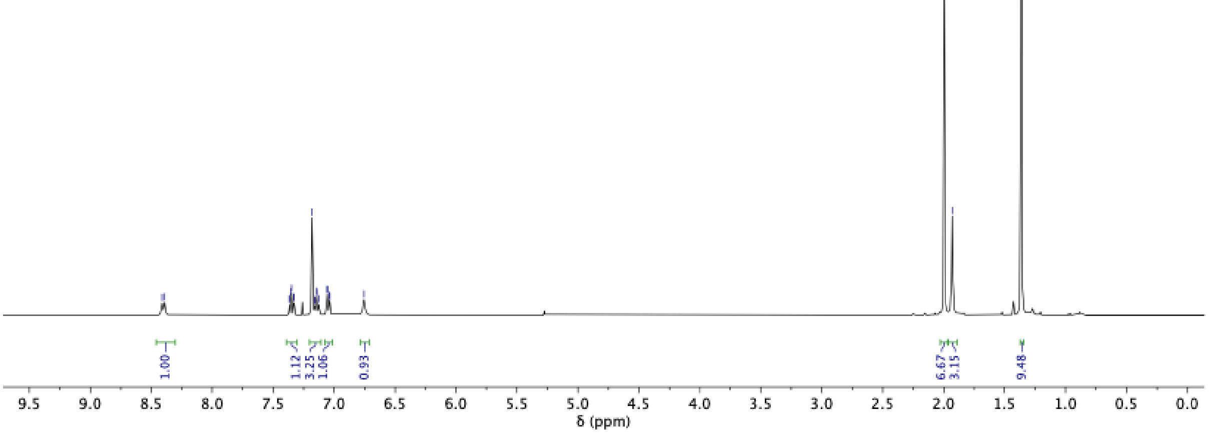
8.41
8.39
7.37
7.37
7.35
7.35
7.33
7.33
7.18
7.16
7.16
7.14
7.14
7.12
7.06
7.05
7.04
7.03
6.76
2.00
1.93
1.36
1.00
1.12
3.25
1.06
0.93
6.67
3.15
9.48
9.5 9.0 8.5 8.0 7.5 7.0 6.5 6.0 5.5 5.0 4.5 4.0 3.5 3.0 2.5 2.0 1.5 1.0 0.5 0.0
δ (ppm)

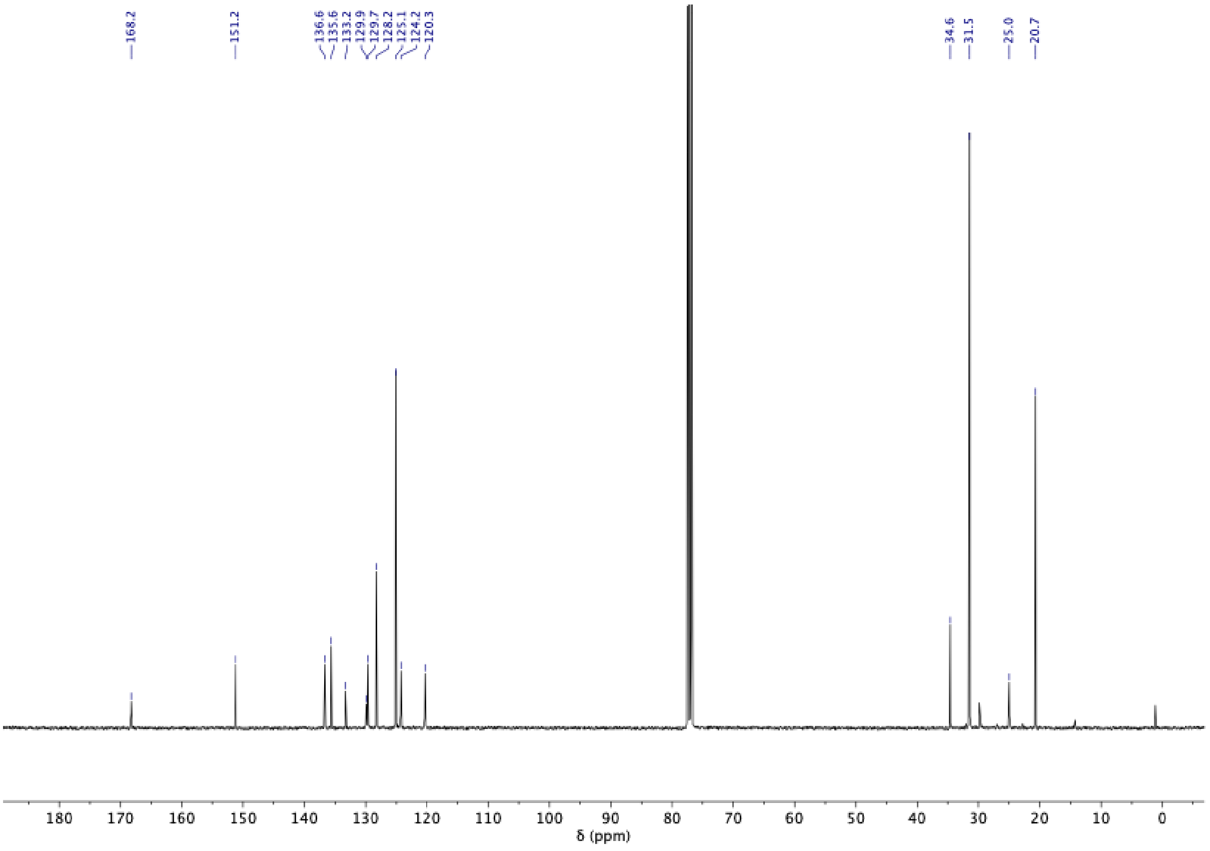
168.2
151.2
136.6
135.6
133.2
129.9
129.7
128.2
125.1
124.2
120.3
34.6
31.5
25.0
20.7
180 170 160 150 140 130 120 110 100 90 80 70 60 50 40 30 20 10 0
δ (ppm)

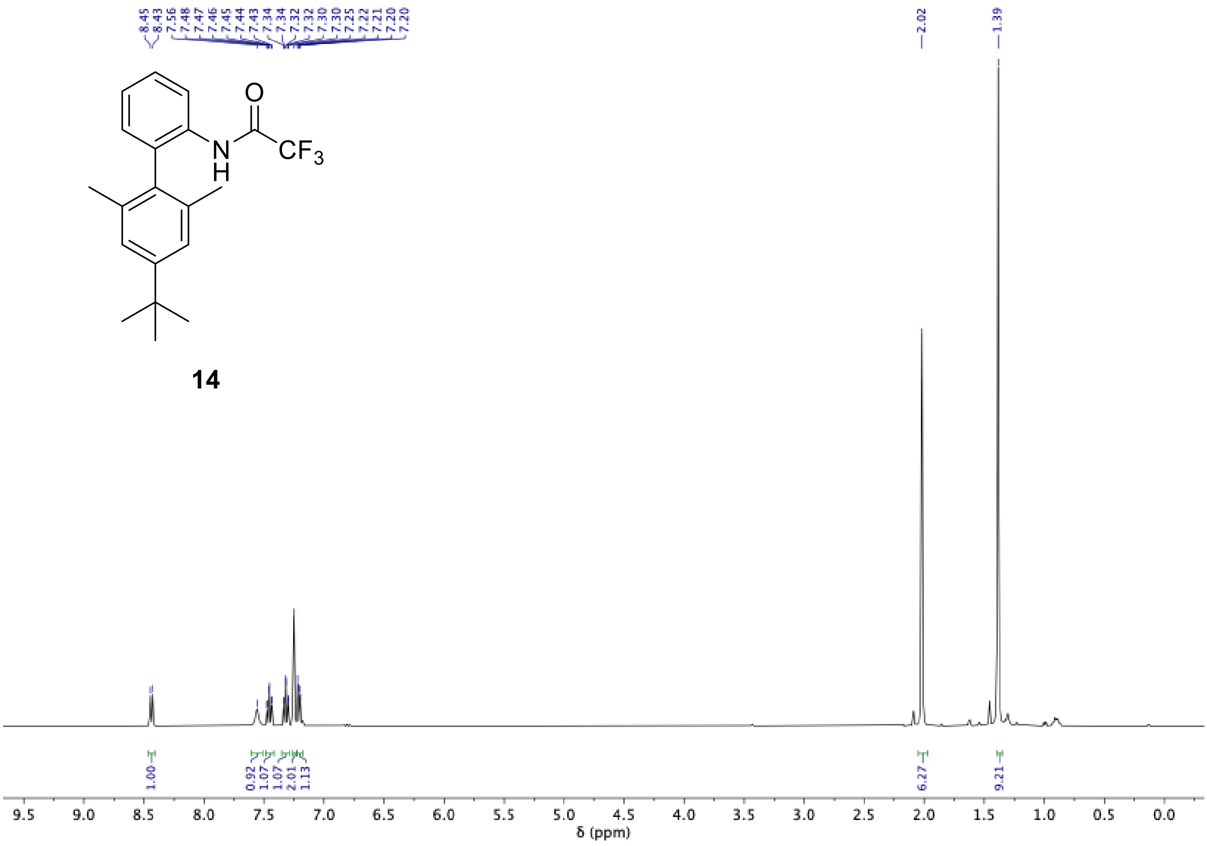
O
N
H
CF3
14
8.45
8.43
7.56
7.48
7.47
7.46
7.45
7.44
7.43
7.34
7.34
7.32
7.32
7.30
7.30
7.25
7.22
7.21
7.20
7.20
2.02
1.39
1.00
0.92
1.07
1.07
2.01
1.13
6.27
9.21
9.5 9.0 8.5 8.0 7.5 7.0 6.5 6.0 5.5 5.0 4.5 4.0 3.5 3.0 2.5 2.0 1.5 1.0 0.5 0.0
δ (ppm)

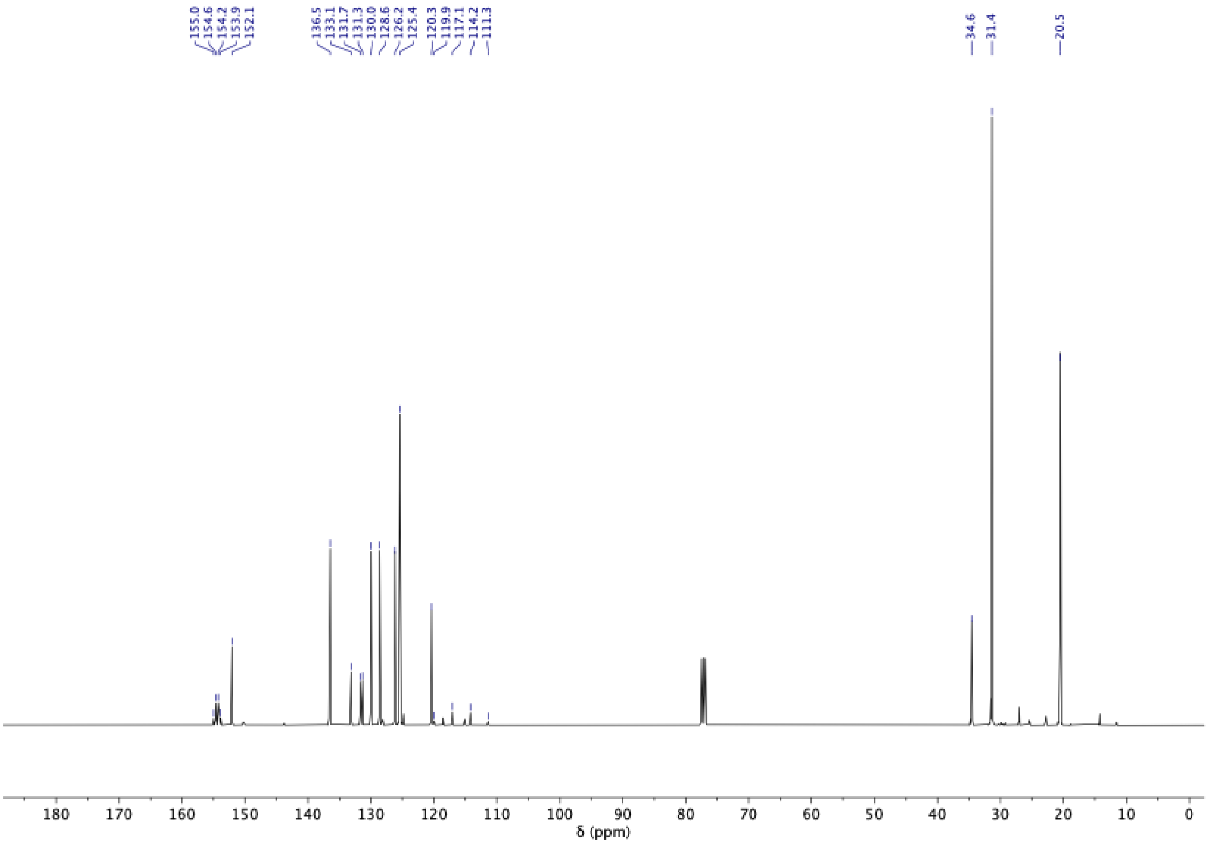
155.0
154.6
154.2
153.9
152.1
136.5
133.1
131.7
131.3
130.0
128.6
126.2
125.4
120.3
119.9
117.1
114.2
111.3
34.6
31.4
20.5
180 170 160 150 140 130 120 110 100 90 80 70 60 50 40 30 20 10 0
δ (ppm)

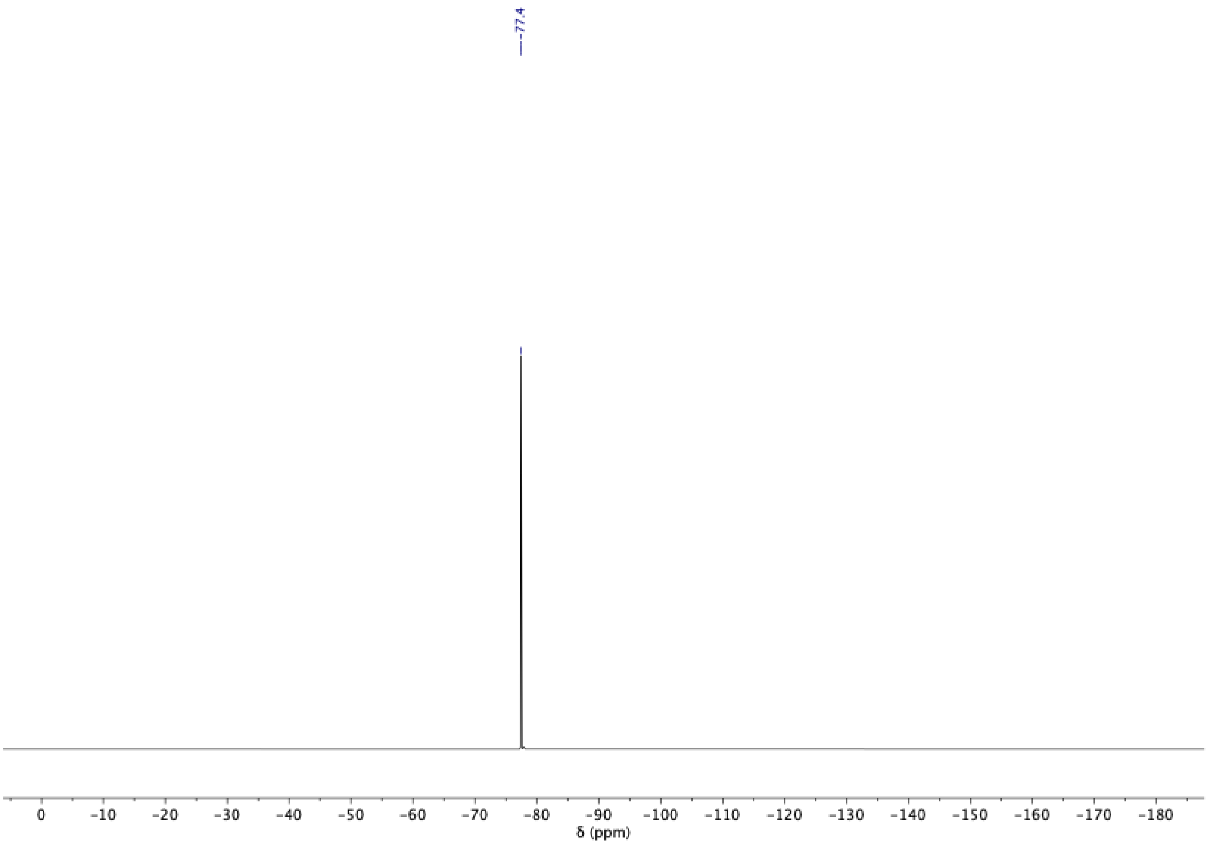
-77.4
0
-10
-20
-30
-40
-50
-60
-70
-80
-90
-100
-110
-120
-130
-140
-150
-160
-170
-180
δ (ppm)

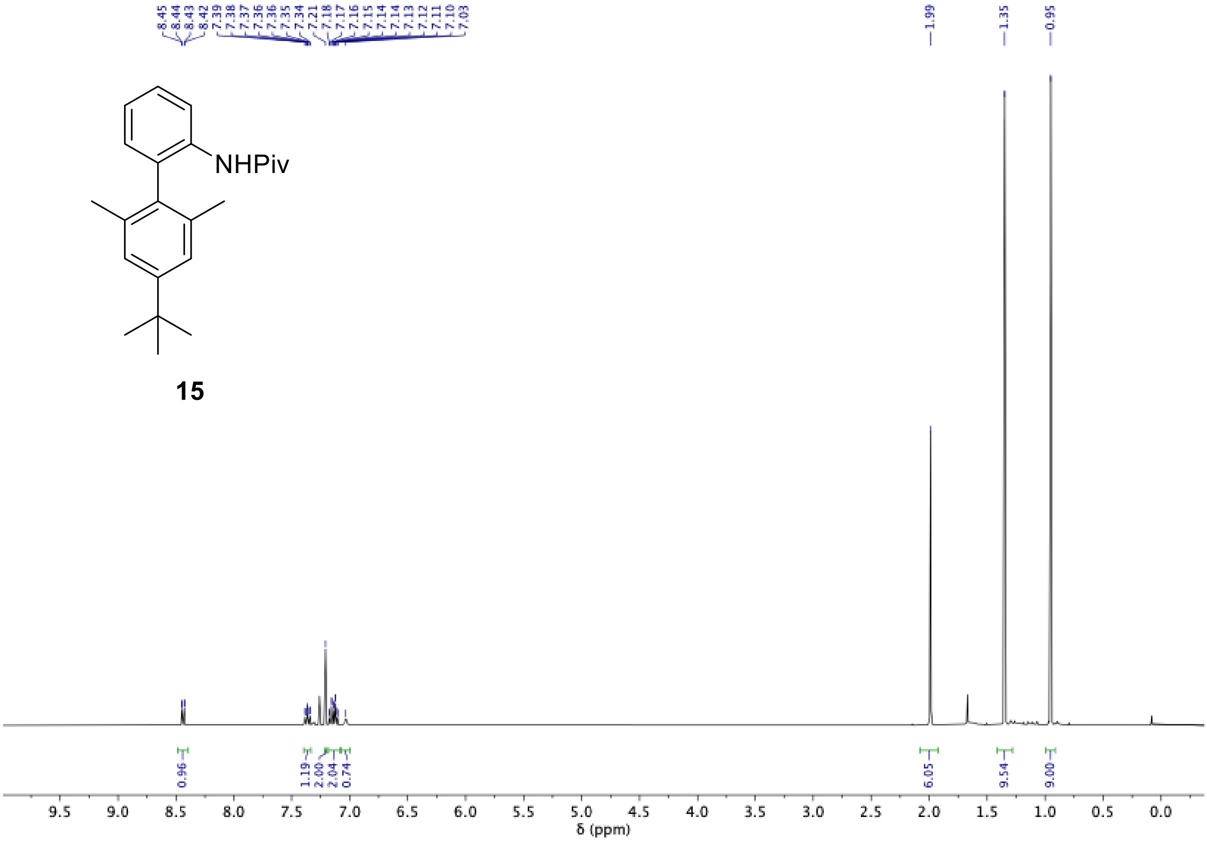
NHPiv
8.45
8.44
8.43
8.42
7.39
7.38
7.37
7.36
7.36
7.35
7.34
7.21
7.18
7.17
7.16
7.15
7.14
7.14
7.13
7.12
7.11
7.10
7.03
1.99
1.35
0.95
0.96
1.19
2.00
2.04
0.74
6.05
9.54
9.00
δ (ppm)

15

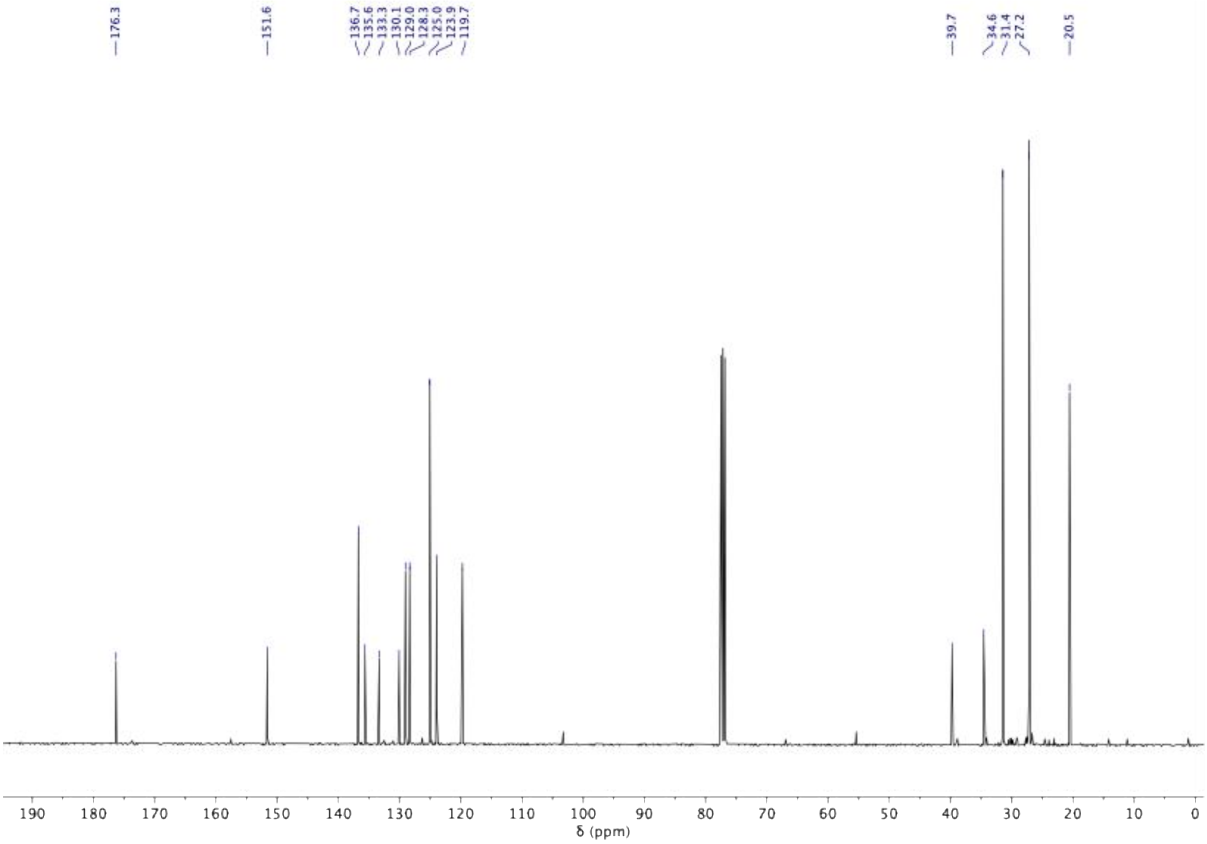
176.3
151.6
136.7
135.6
133.3
130.1
129.0
128.3
125.0
123.9
119.7
39.7
34.6
31.4
27.2
20.5
δ (ppm)

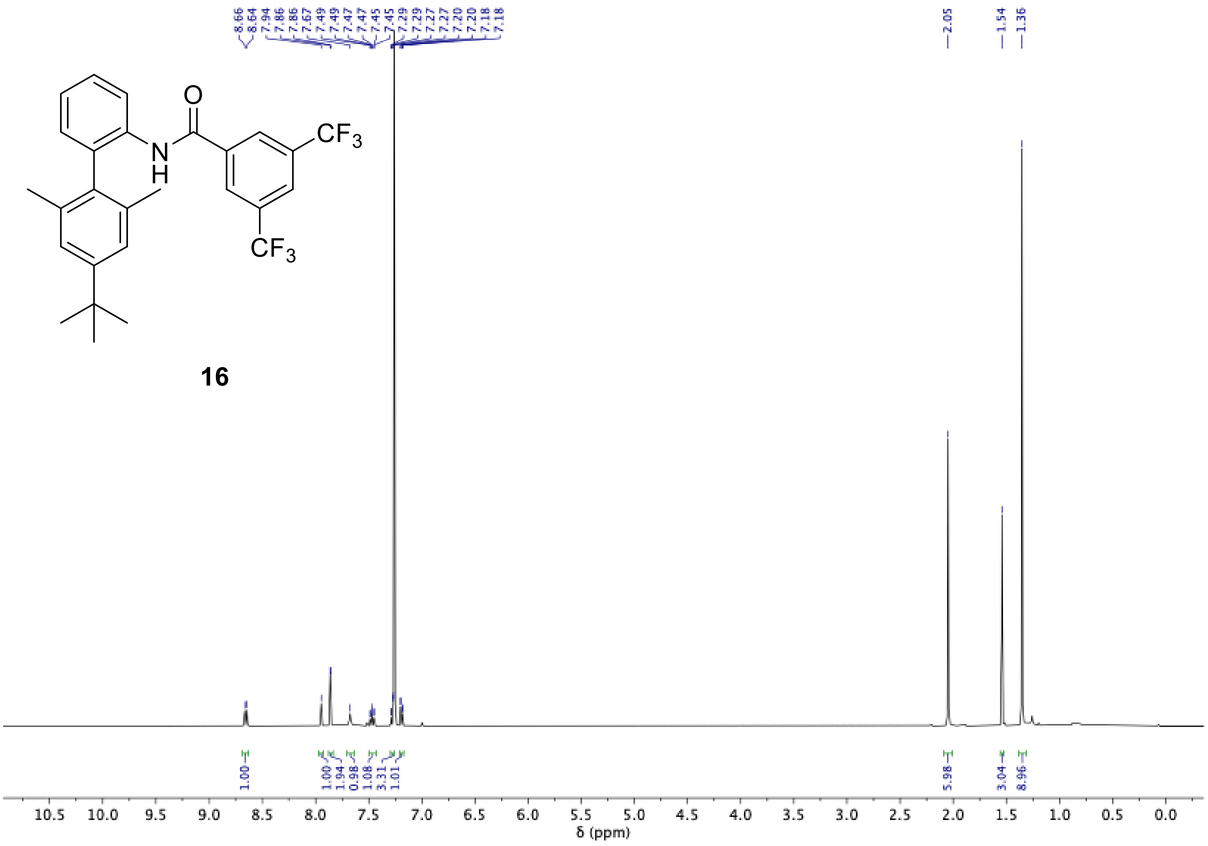
O
N
H
CF3
CF3
16
8.66
8.64
7.94
7.86
7.86
7.67
7.49
7.49
7.47
7.47
7.45
7.29
7.29
7.27
7.27
7.20
7.20
7.18
7.18
2.05
1.54
1.36
1.00
1.00
1.94
0.98
1.08
3.31
1.01
5.98
3.04
8.96
δ (ppm)

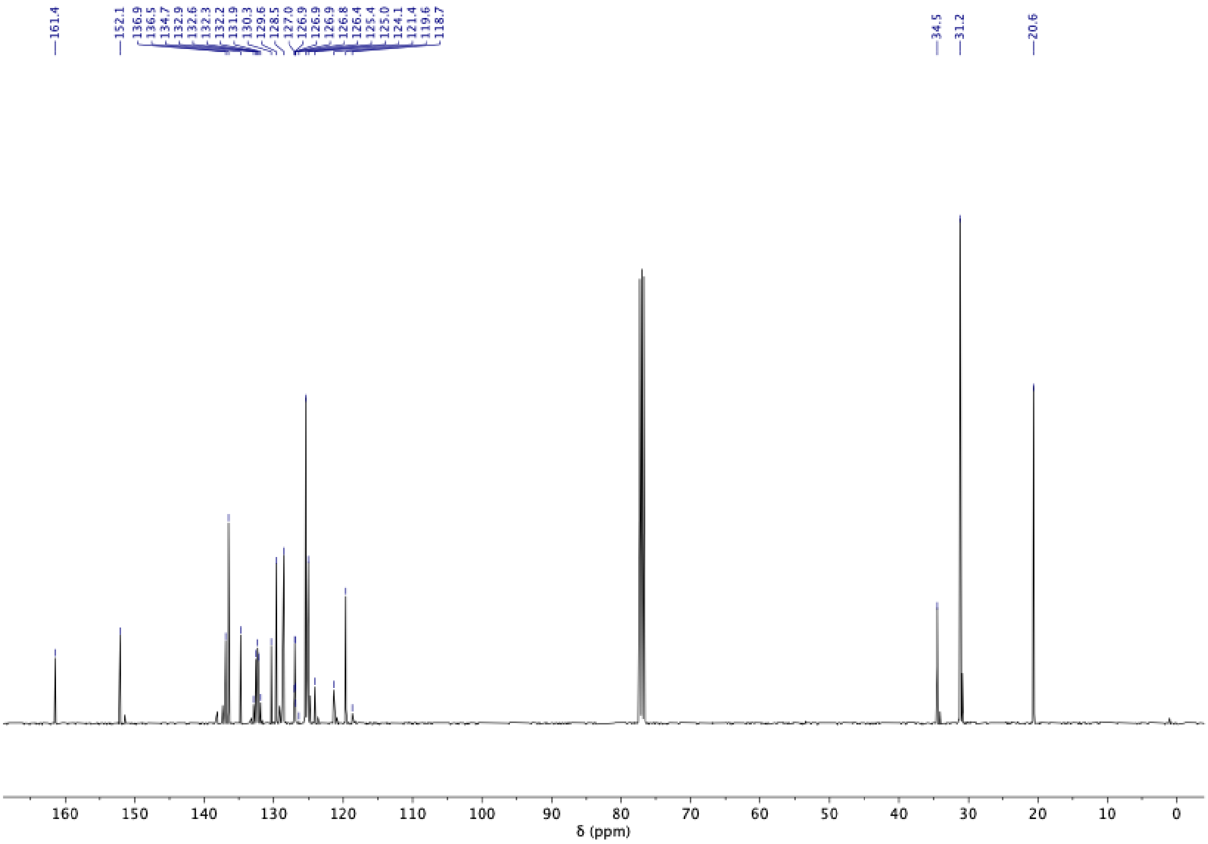
161.4
152.1
136.9
136.5
134.7
132.9
132.6
132.3
132.2
131.9
130.3
129.6
128.5
127.0
126.9
126.9
126.8
126.4
125.4
125.0
124.1
121.4
119.6
118.7
34.5
31.2
20.6
δ (ppm)

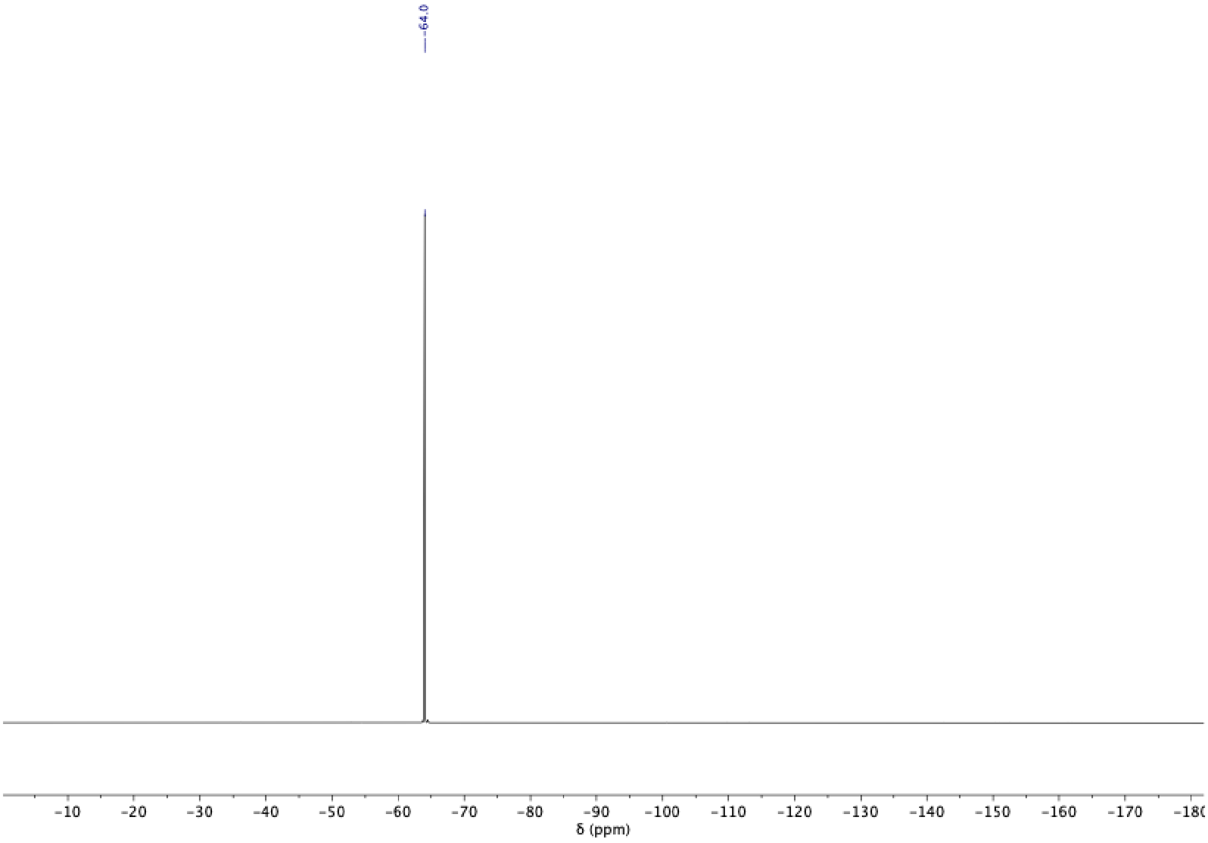
-64.0
-10
-20
-30
-40
-50
-60
-70
-80
-90
-100
-110
-120
-130
-140
-150
-160
-170
δ (ppm)

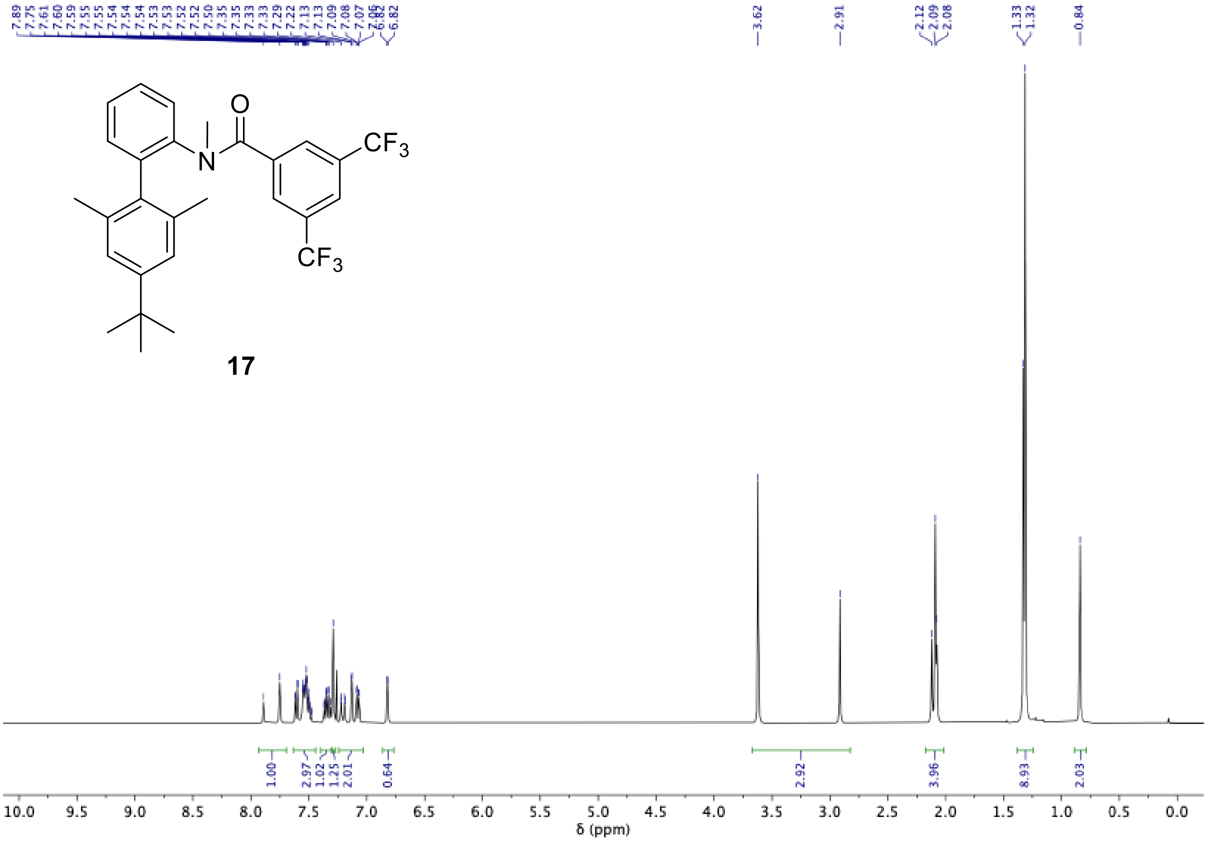
7.89 7.75 7.61 7.60 7.59 7.55 7.55 7.54 7.54 7.54 7.53 7.53 7.52 7.52 7.50 7.35 7.35 7.33 7.33 7.29 7.22 7.13 7.13 7.09 7.08 7.07 7.06 6.82 6.82
3.62
2.91
2.12 2.09 2.08
1.33 1.32
0.84
O
N
CF3
CF3
17
1.00 2.97 1.02 1.25 2.01 0.64 2.92 3.96 8.93 2.03
10.0 9.5 9.0 8.5 8.0 7.5 7.0 6.5 6.0 5.5 5.0 4.5 4.0 3.5 3.0 2.5 2.0 1.5 1.0 0.5 0.0
δ (ppm)

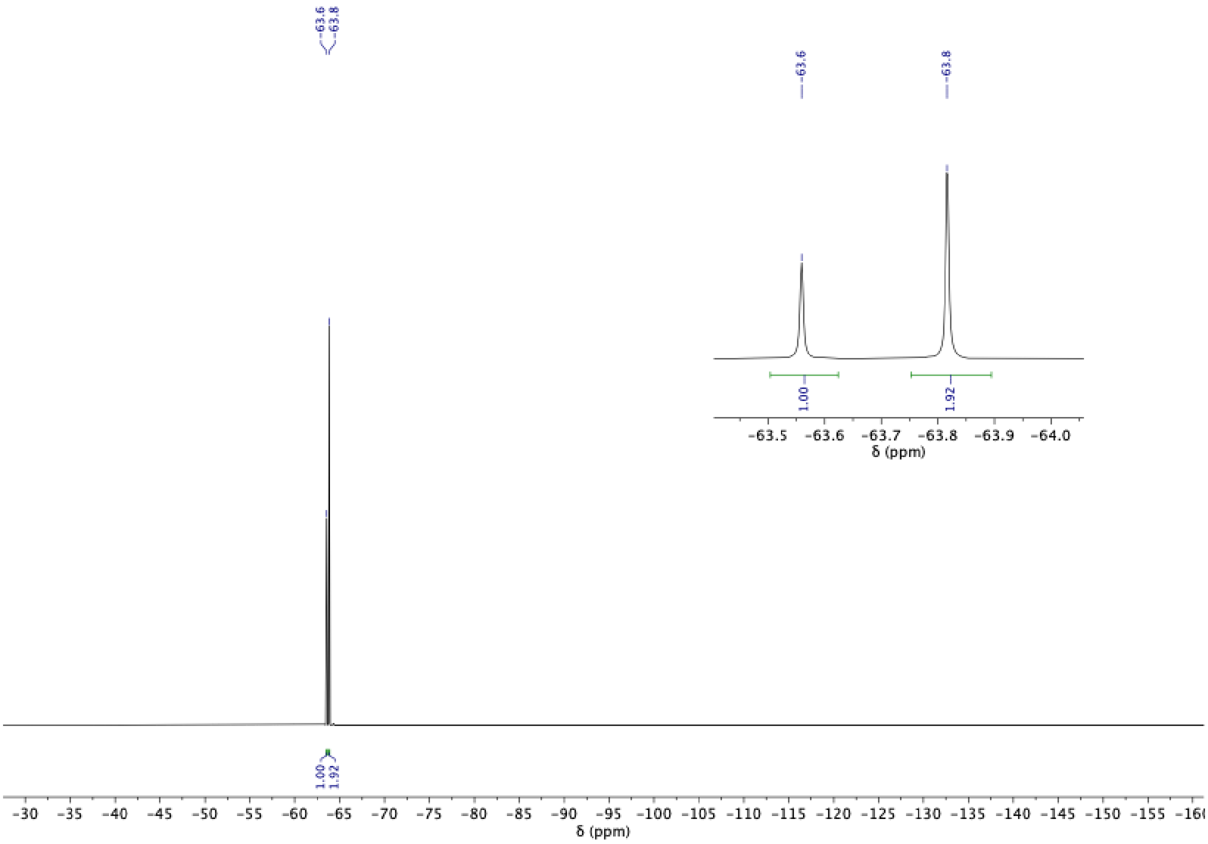
-63.6 -63.8
-63.6
-63.8
1.00
1.92
-63.5 -63.6 -63.7 -63.8 -63.9 -64.0
δ (ppm)
1.00 1.92
-30 -35 -40 -45 -50 -55 -60 -65 -70 -75 -80 -85 -90 -95 -100 -105 -110 -115 -120 -125 -130 -135 -140 -145 -150 -155 -160
δ (ppm)

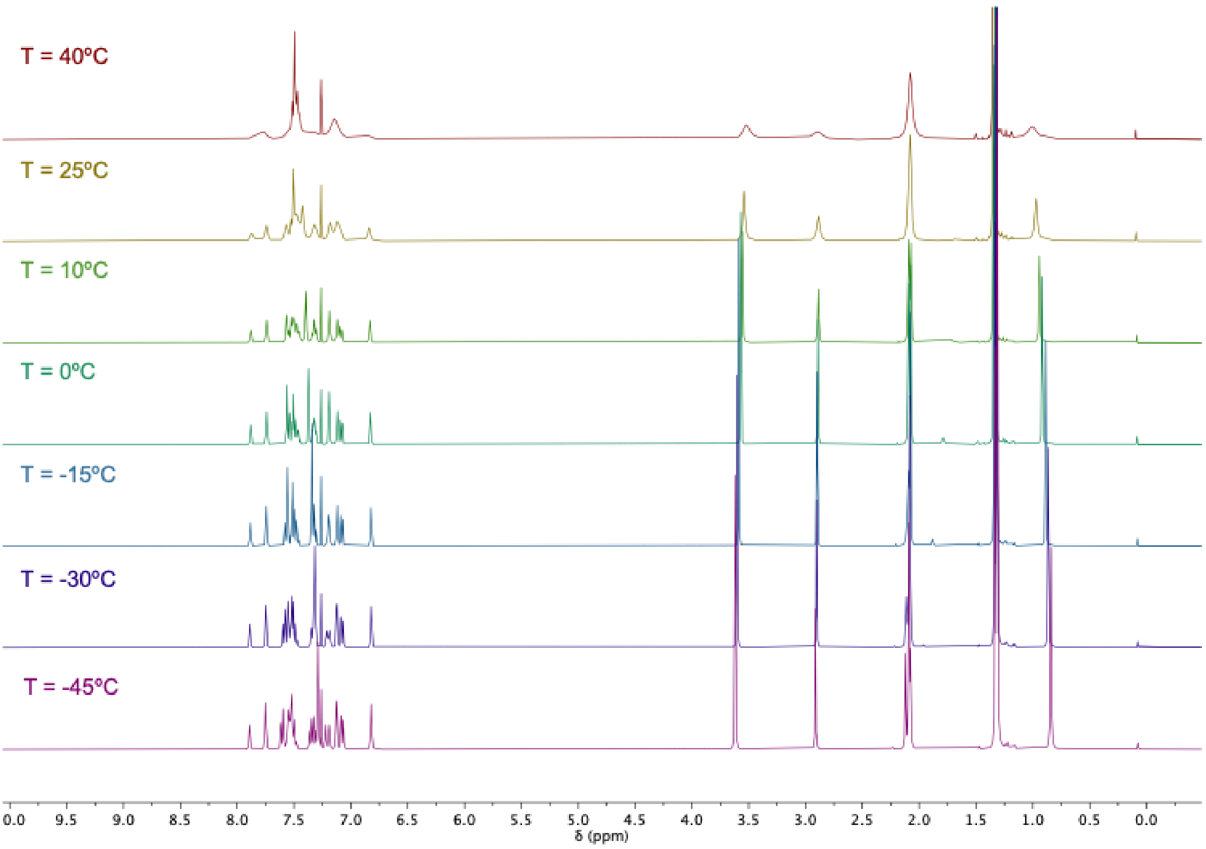
T = 40ºC
T = 25ºC
T = 10ºC
T = 0ºC
T = -15ºC
T = -30ºC
T = -45ºC
δ (ppm)

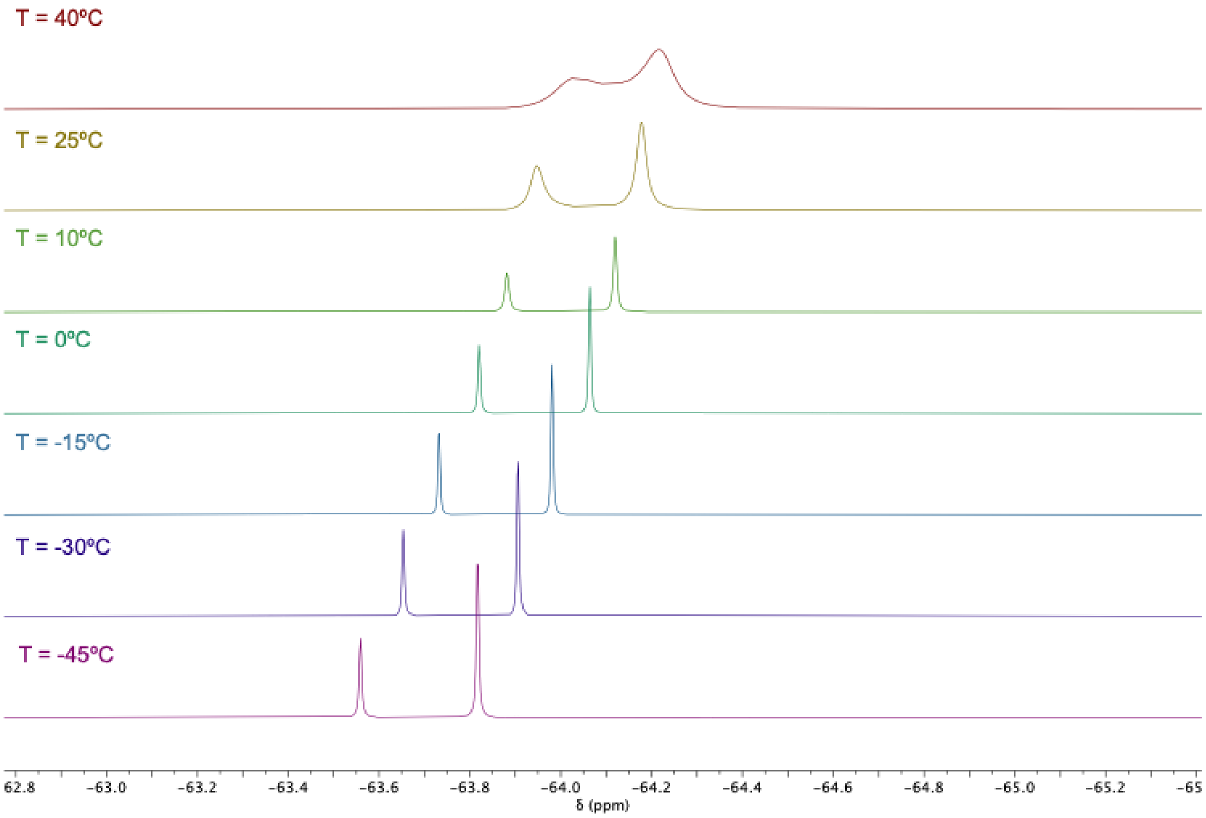
T = 40ºC
T = 25ºC
T = 10ºC
T = 0ºC
T = -15ºC
T = -30ºC
T = -45ºC
δ (ppm)

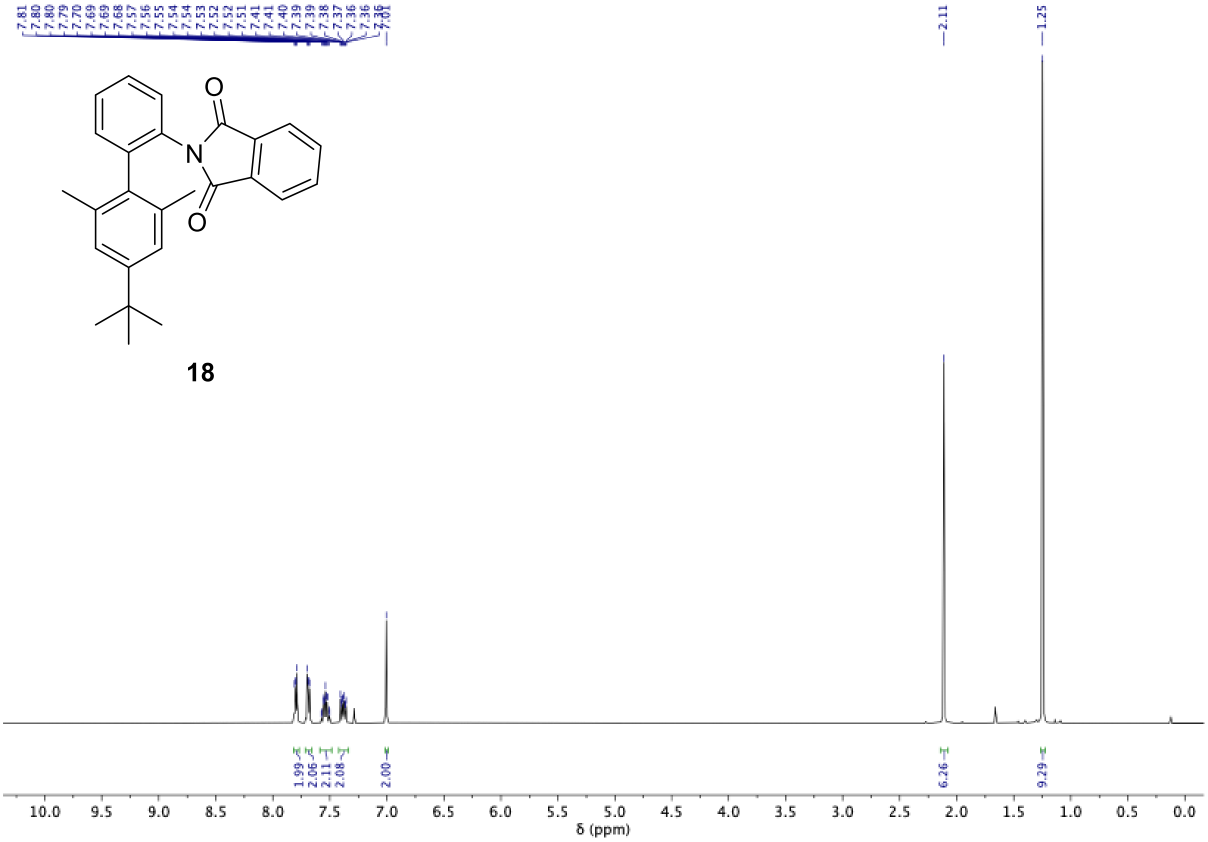
7.81
7.80
7.80
7.79
7.70
7.69
7.69
7.68
7.57
7.56
7.55
7.54
7.54
7.54
7.53
7.52
7.52
7.51
7.41
7.41
7.40
7.39
7.39
7.38
7.37
7.36
7.36
7.36
7.01
2.11
1.25
O
N
O
18
1.99
2.06
2.11
2.08
2.00
6.26
9.29
10.0 9.5 9.0 8.5 8.0 7.5 7.0 6.5 6.0 5.5 5.0 4.5 4.0 3.5 3.0 2.5 2.0 1.5 1.0 0.5 0.0
δ (ppm)

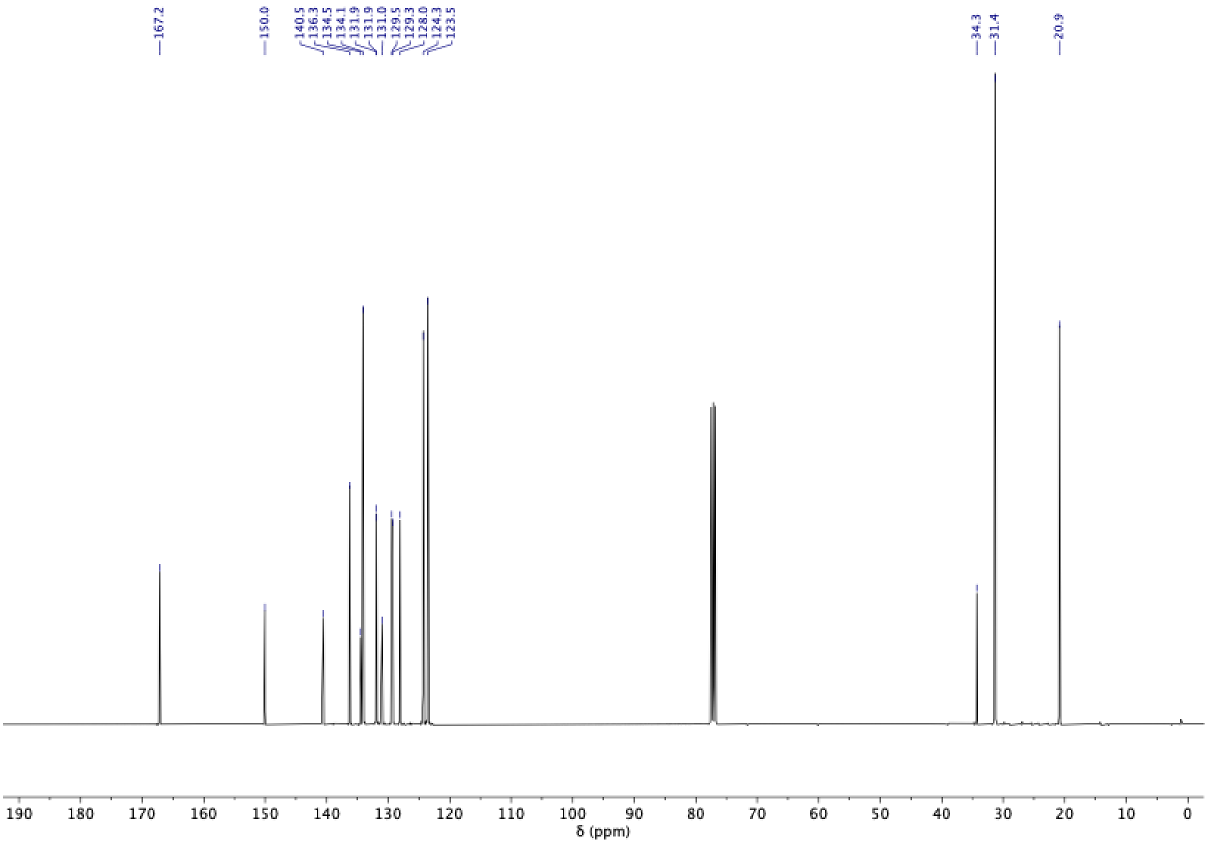
167.2
150.0
140.5
136.3
134.5
134.1
131.9
131.0
129.5
129.3
128.0
124.3
123.5
34.3
31.4
20.9
190 180 170 160 150 140 130 120 110 100 90 80 70 60 50 40 30 20 10 0
δ (ppm)

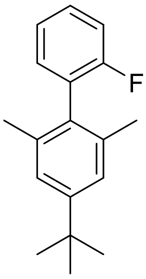
F

19

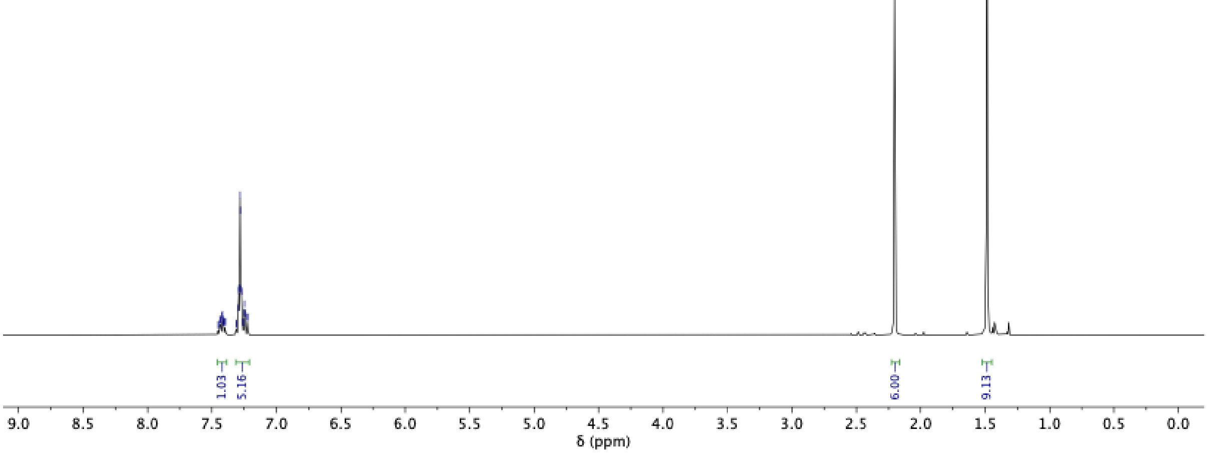
7.45
7.44
7.44
7.43
7.43
7.42
7.42
7.42
7.41
7.41
7.40
7.40
7.31
7.31
7.29
7.29
7.29
7.29
7.28
7.28
7.27
7.27
7.26
7.26
7.25
7.25
7.24
7.23
7.22
2.20
1.49
1.03
5.16
6.00
9.13
9.0 8.5 8.0 7.5 7.0 6.5 6.0 5.5 5.0 4.5 4.0 3.5 3.0 2.5 2.0 1.5 1.0 0.5 0.0
δ (ppm)

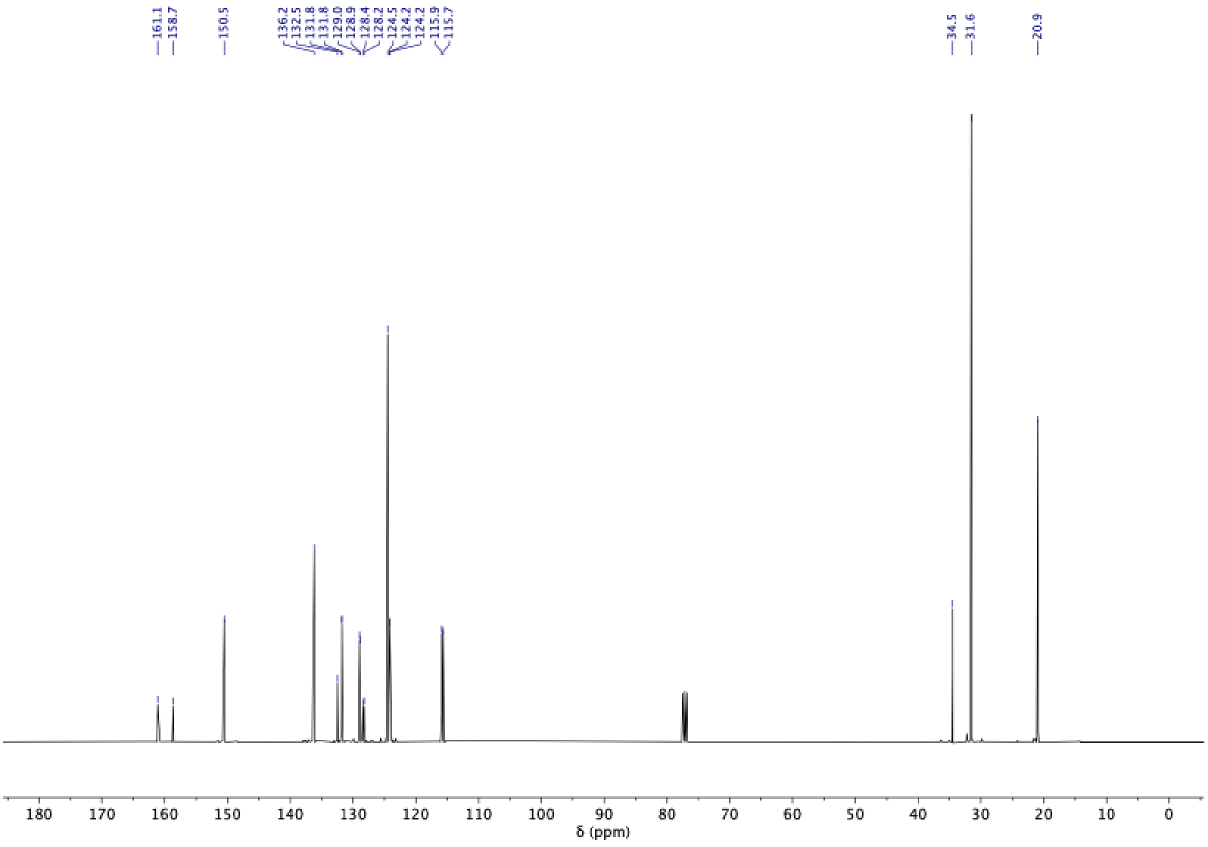
161.1
158.7
150.5
136.2
132.5
131.8
131.8
129.0
128.9
128.4
128.2
124.5
124.2
124.2
115.9
115.7
34.5
31.6
20.9
180 170 160 150 140 130 120 110 100 90 80 70 60 50 40 30 20 10 0
δ (ppm)

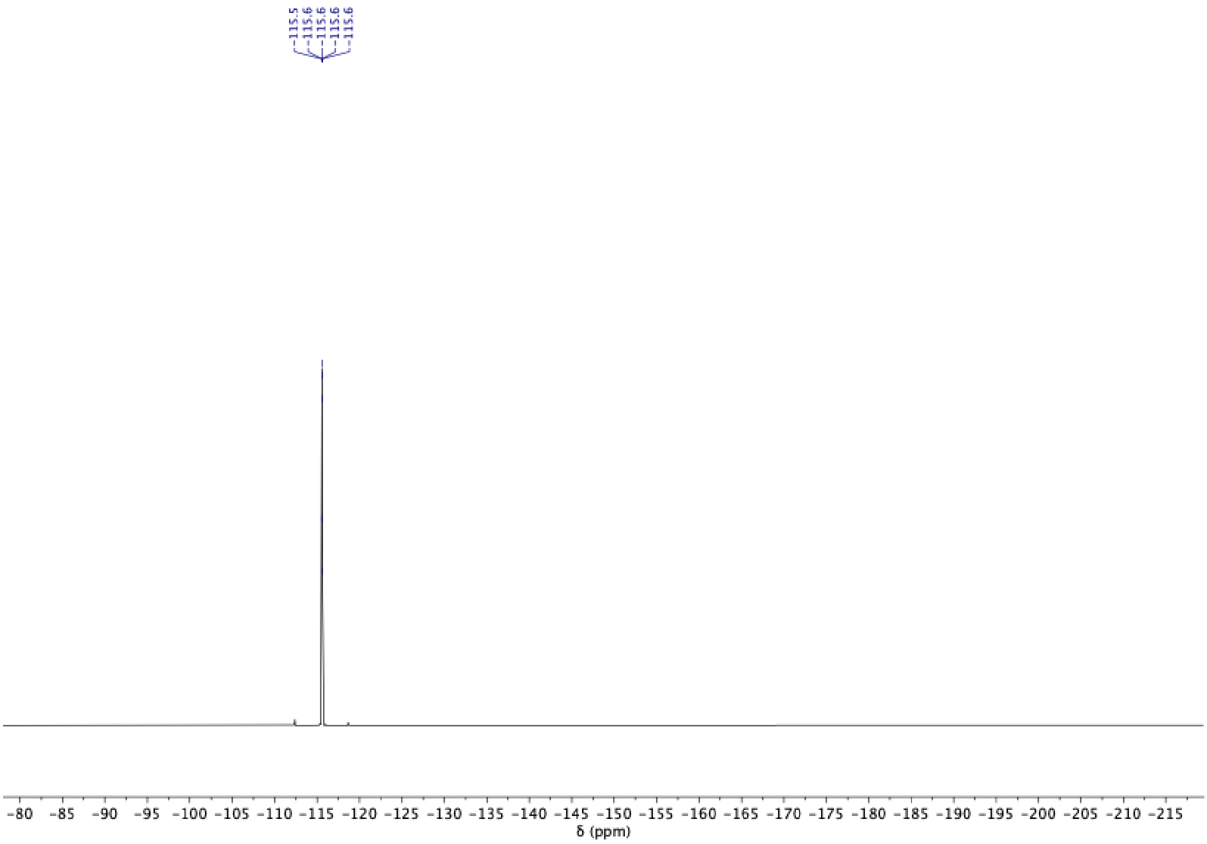

-115.5
-115.6
-115.6
-115.6
-115.6
-80 -85 -90 -95 -100 -105 -110 -115 -120 -125 -130 -135 -140 -145 -150 -155 -160 -165 -170 -175 -180 -185 -190 -195 -200 -205 -210 -215
δ (ppm)

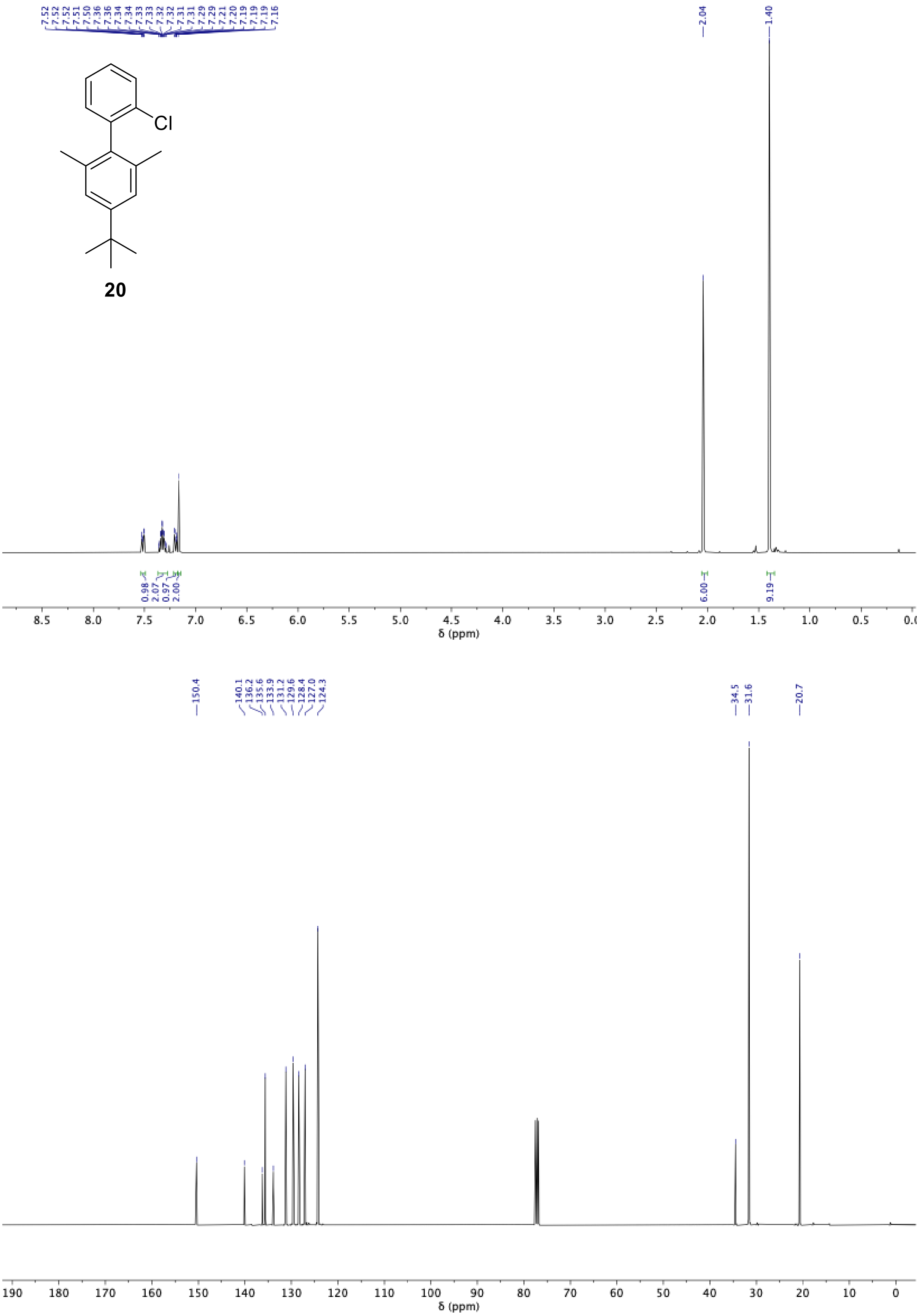
Cl
20
δ (ppm)
δ (ppm)

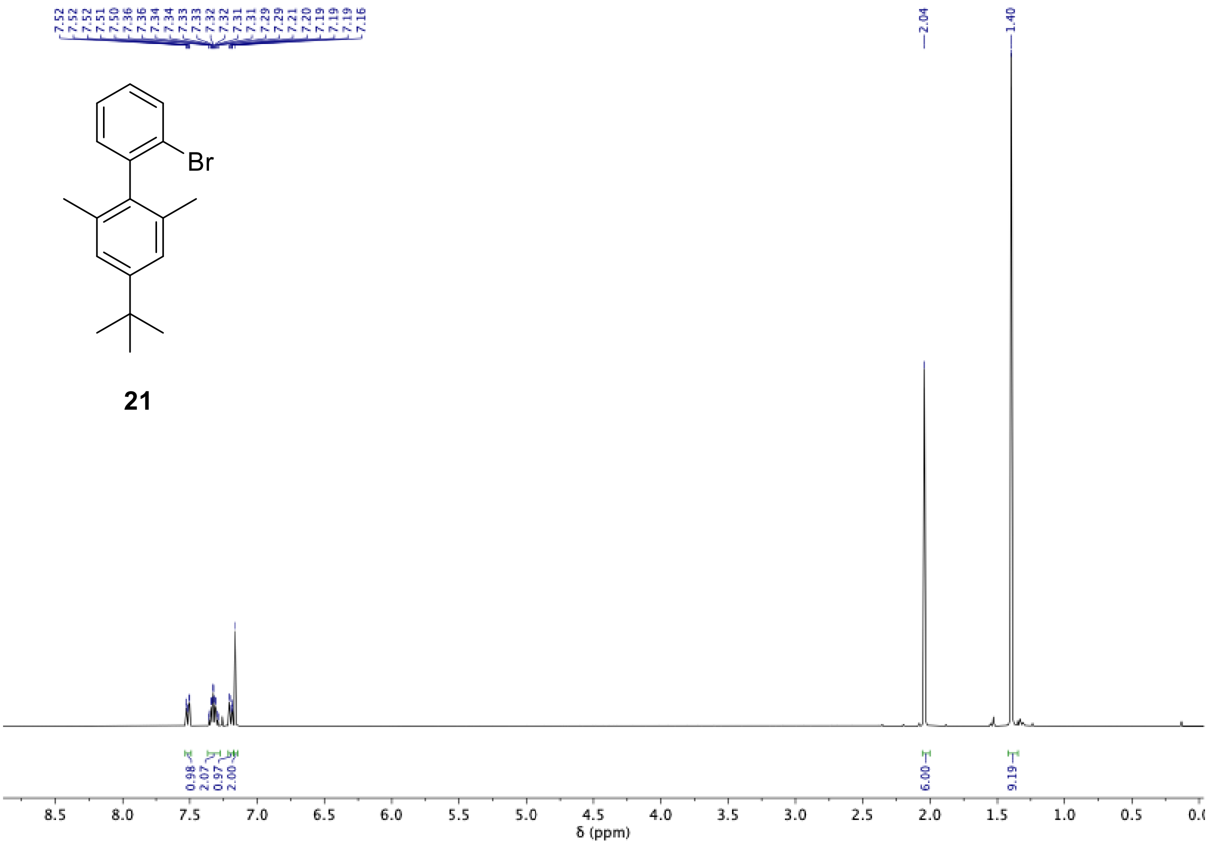
7.52 7.52 7.52 7.51 7.50 7.36 7.36 7.34 7.34 7.33 7.33 7.32 7.32 7.31 7.31 7.29 7.29 7.21 7.20 7.19 7.19 7.19 7.16
2.04
1.40
Br
21
0.98 2.07 0.97 2.00
6.00
9.19
δ (ppm)

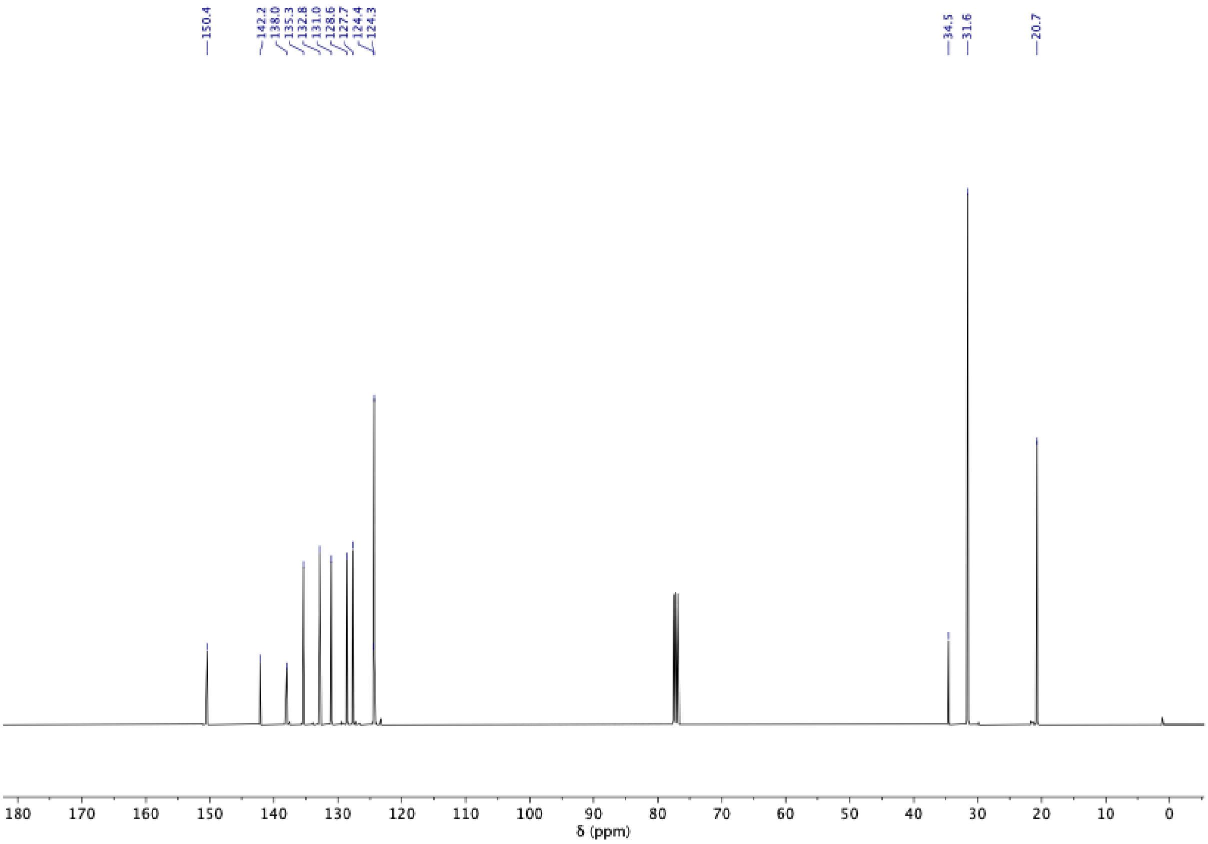
150.4
142.2 138.0 135.3 132.8 131.0 128.6 127.7 124.4 124.3
34.5 31.6
20.7
δ (ppm)

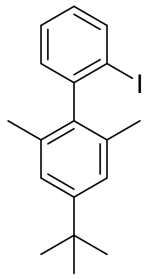

22

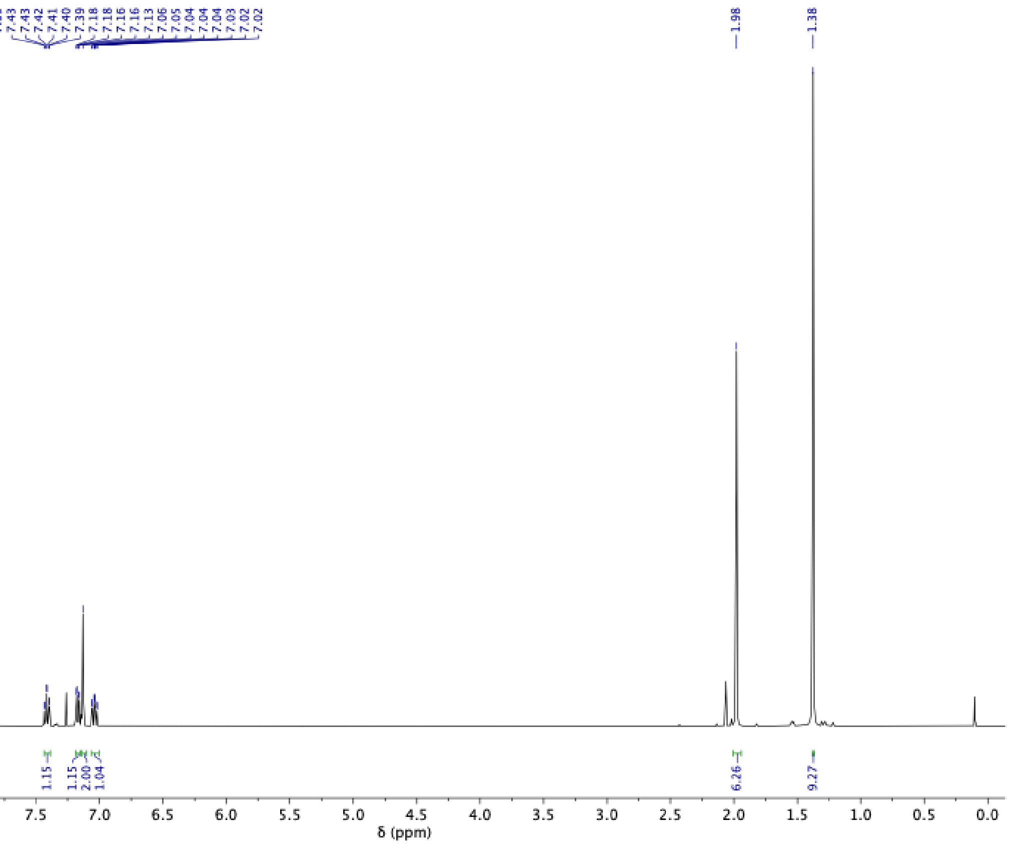
7.97
7.97
7.95
7.95
7.43
7.43
7.42
7.41
7.40
7.39
7.18
7.18
7.16
7.16
7.13
7.06
7.05
7.04
7.04
7.04
7.03
7.02
7.02
1.98
1.38
1.00
1.15
1.15
2.00
1.04
6.26
9.27
9.0
8.5
8.0
7.5
7.0
6.5
6.0
5.5
5.0
4.5
4.0
3.5
3.0
2.5
2.0
1.5
1.0
0.5
0.0
δ (ppm)

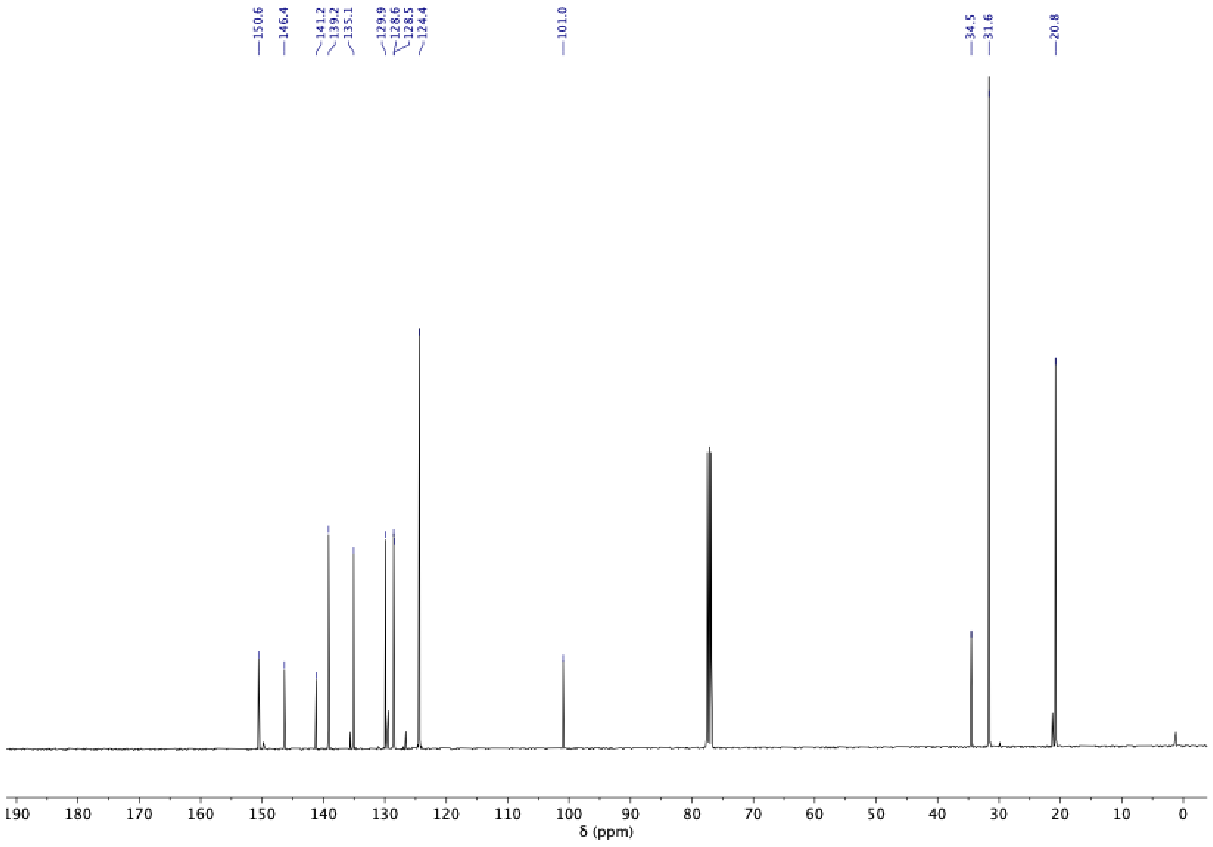
150.6
146.4
141.2
139.2
135.1
129.9
128.6
128.5
124.4
101.0
34.5
31.6
20.8
190
180
170
160
150
140
130
120
110
100
90
80
70
60
50
40
30
20
10
0
δ (ppm)

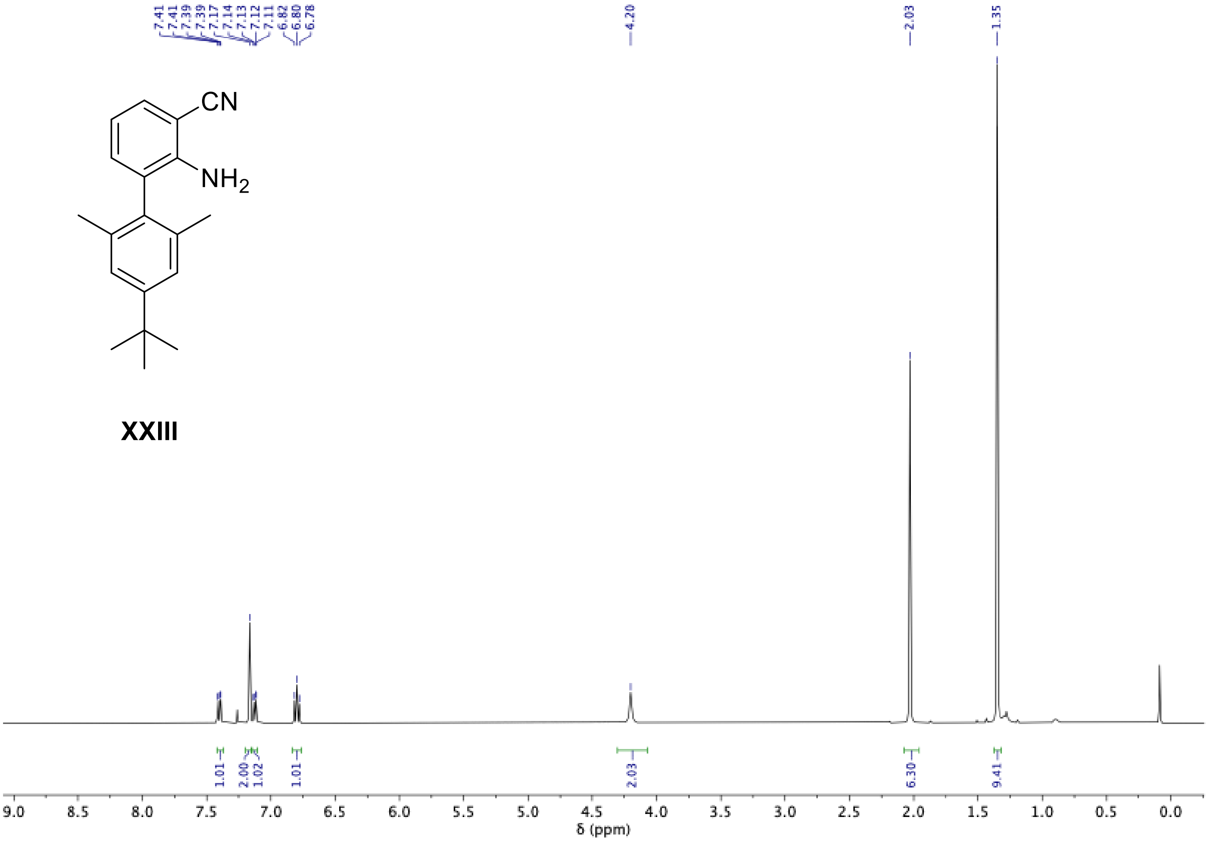
CN
NH2
XXIII
7.41
7.41
7.39
7.39
7.17
7.14
7.13
7.12
7.11
6.82
6.80
6.78
4.20
2.03
1.35
1.01
2.00
1.02
1.01
2.03
6.30
9.41
9.0 8.5 8.0 7.5 7.0 6.5 6.0 5.5 5.0 4.5 4.0 3.5 3.0 2.5 2.0 1.5 1.0 0.5 0.0
δ (ppm)

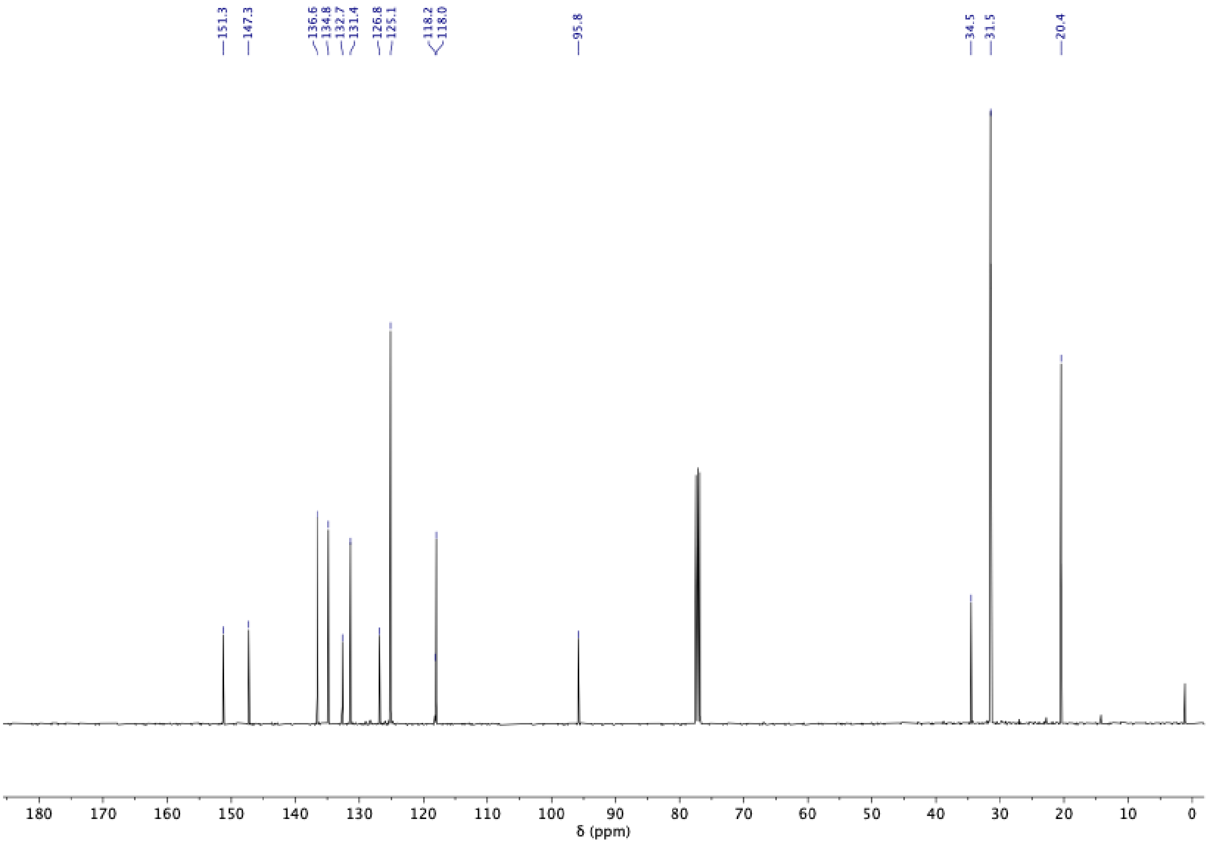
151.3
147.3
136.6
134.8
132.7
131.4
126.8
125.1
118.2
118.0
95.8
34.5
31.5
20.4
180 170 160 150 140 130 120 110 100 90 80 70 60 50 40 30 20 10 0
δ (ppm)

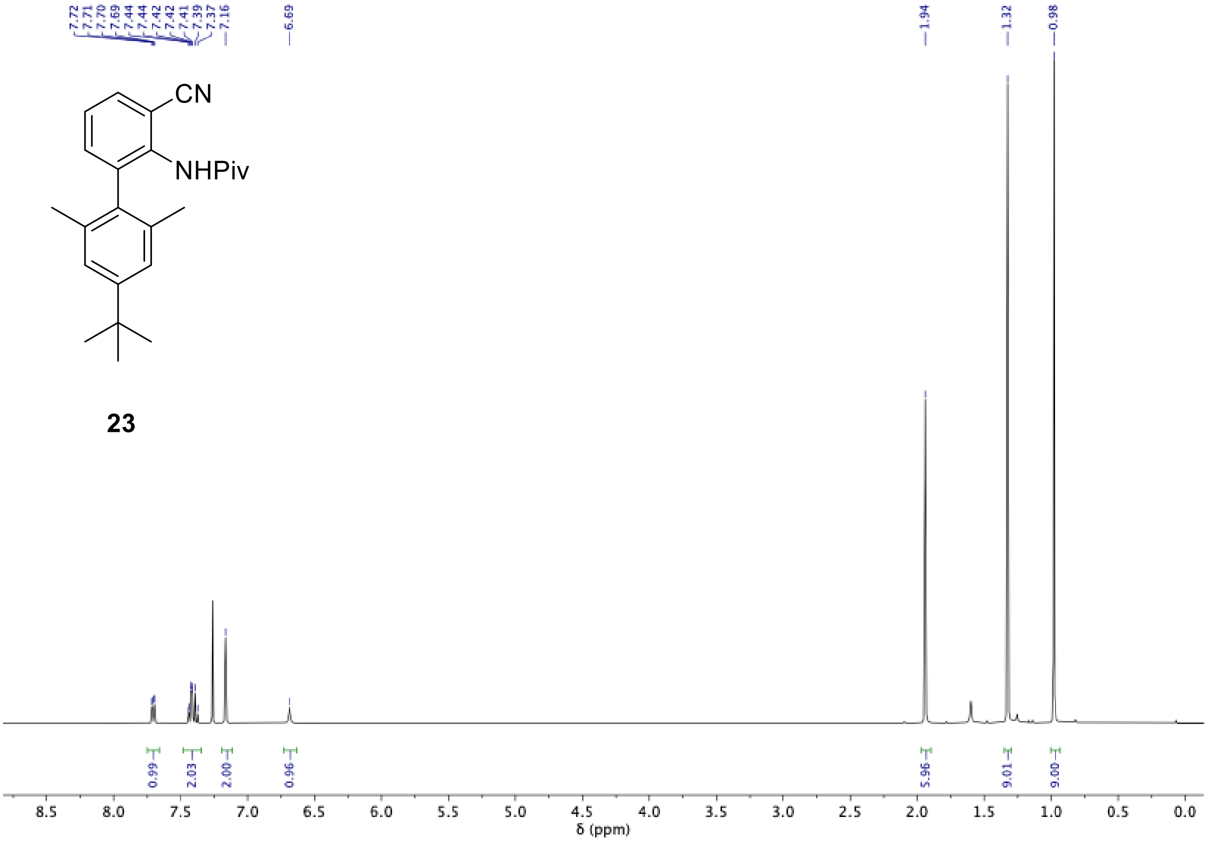
CN
NHPiv
7.72
7.71
7.70
7.69
7.44
7.44
7.42
7.42
7.41
7.39
7.37
7.16
6.69
1.94
1.32
0.98
0.99
2.03
2.00
0.96
5.96
9.01
9.00
8.5 8.0 7.5 7.0 6.5 6.0 5.5 5.0 4.5 4.0 3.5 3.0 2.5 2.0 1.5 1.0 0.5 0.0
δ (ppm)

23

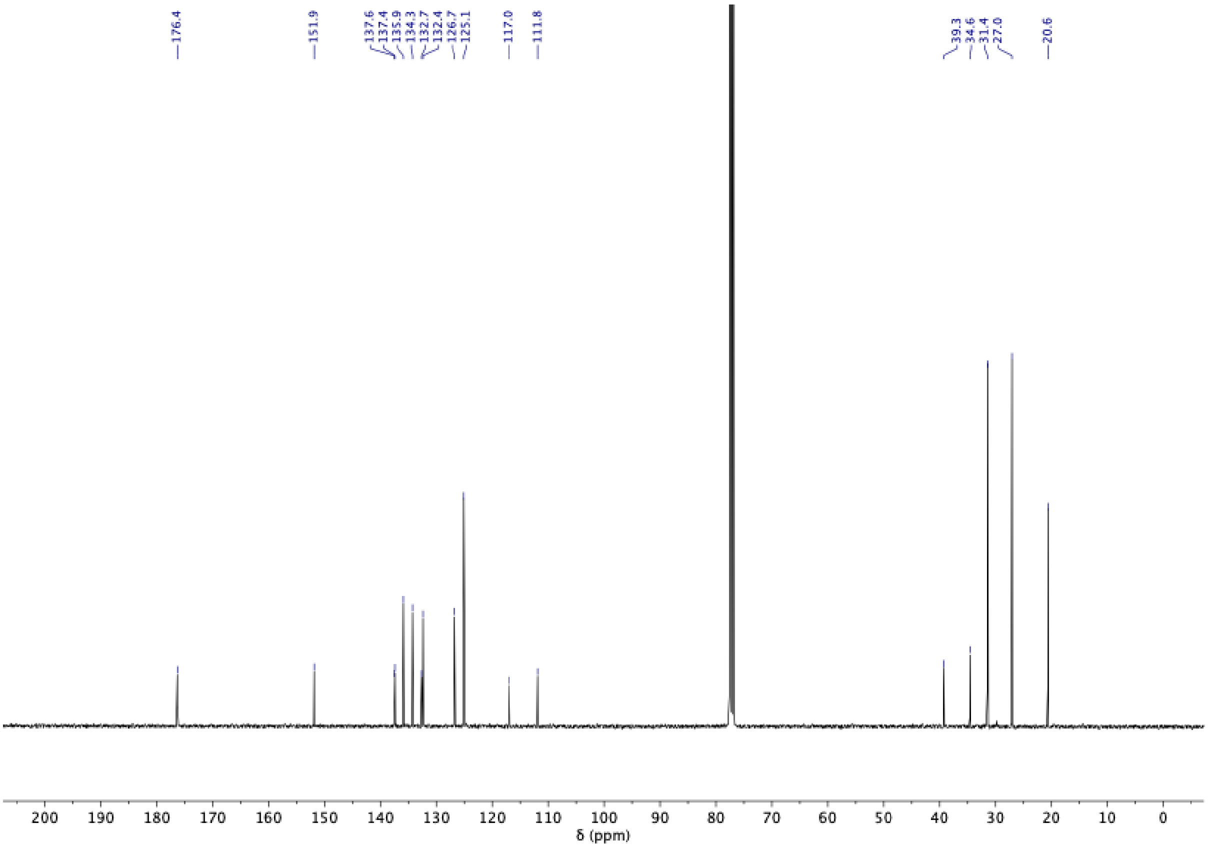
176.4
151.9
137.6
137.4
135.9
134.3
132.7
132.4
126.7
125.1
117.0
111.8
39.3
34.6
31.4
27.0
20.6
200 190 180 170 160 150 140 130 120 110 100 90 80 70 60 50 40 30 20 10 0
δ (ppm)

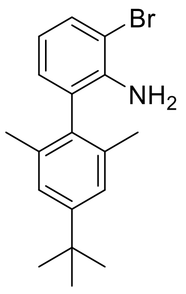

XXIV

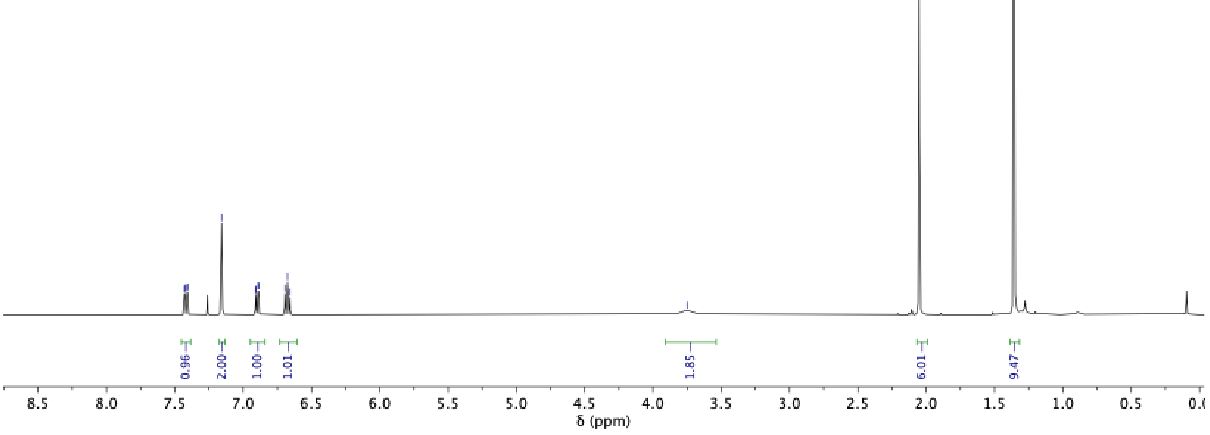
7.43
7.43
7.41
7.41
7.16
6.91
6.91
6.89
6.89
6.69
6.67
6.66
3.75
2.05
1.36
0.96
2.00
1.00
1.01
1.85
6.01
9.47
δ (ppm)

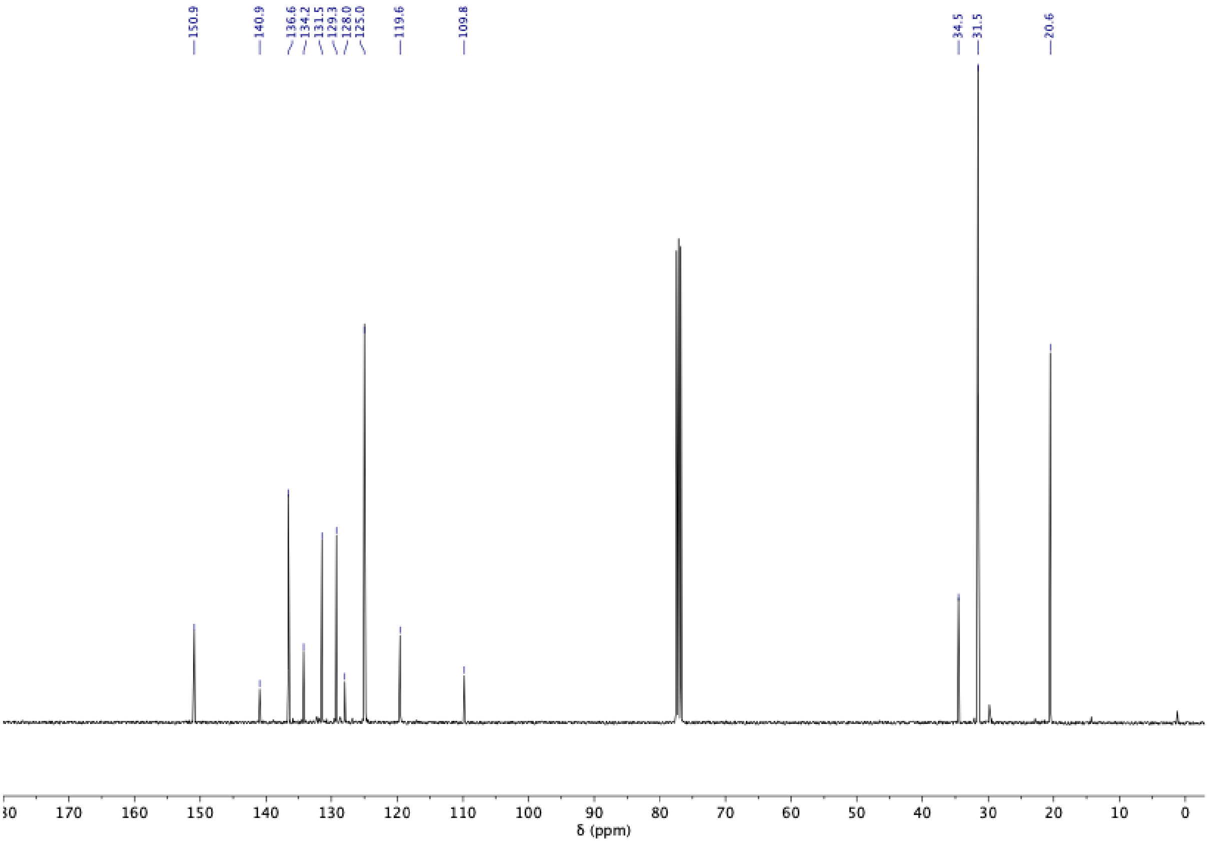
150.9
140.9
136.6
134.2
131.5
129.3
128.0
125.0
119.6
109.8
34.5
31.5
20.6
δ (ppm)

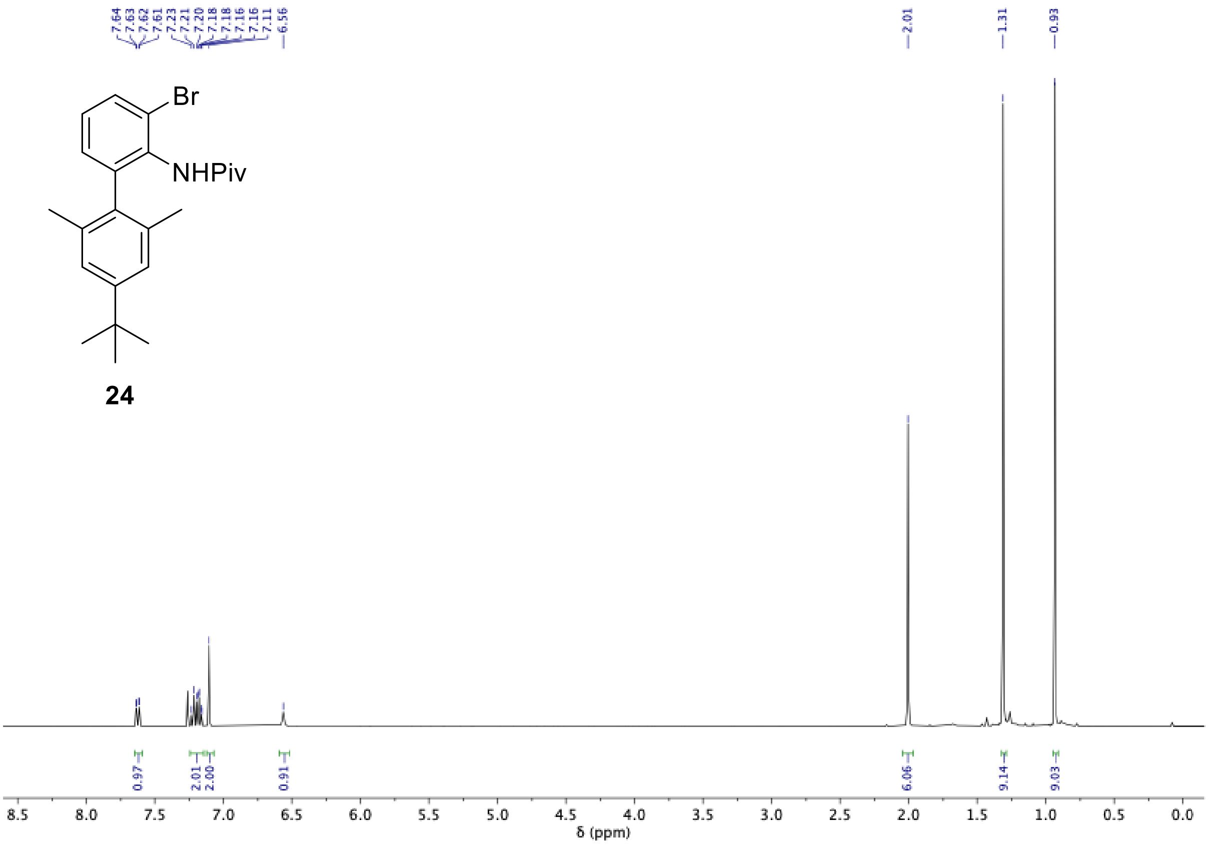
Br
NHPiv
24
7.64
7.63
7.62
7.61
7.23
7.21
7.20
7.18
7.18
7.16
7.16
7.11
6.56
2.01
1.31
0.93
0.97
2.01
2.00
0.91
6.06
9.14
9.03
δ (ppm)

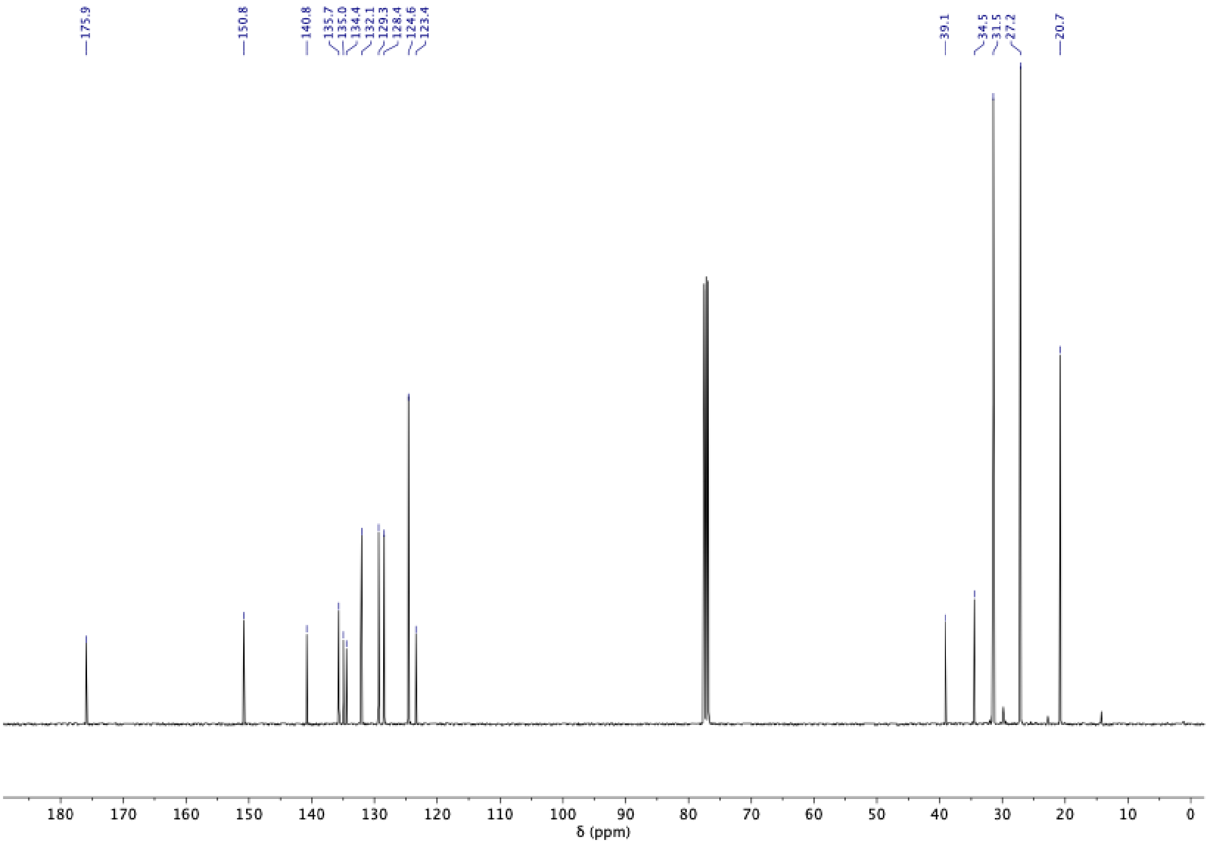
175.9
150.8
140.8
135.7
135.0
134.4
132.1
129.3
128.4
124.6
123.4
39.1
34.5
31.5
27.2
20.7
δ (ppm)

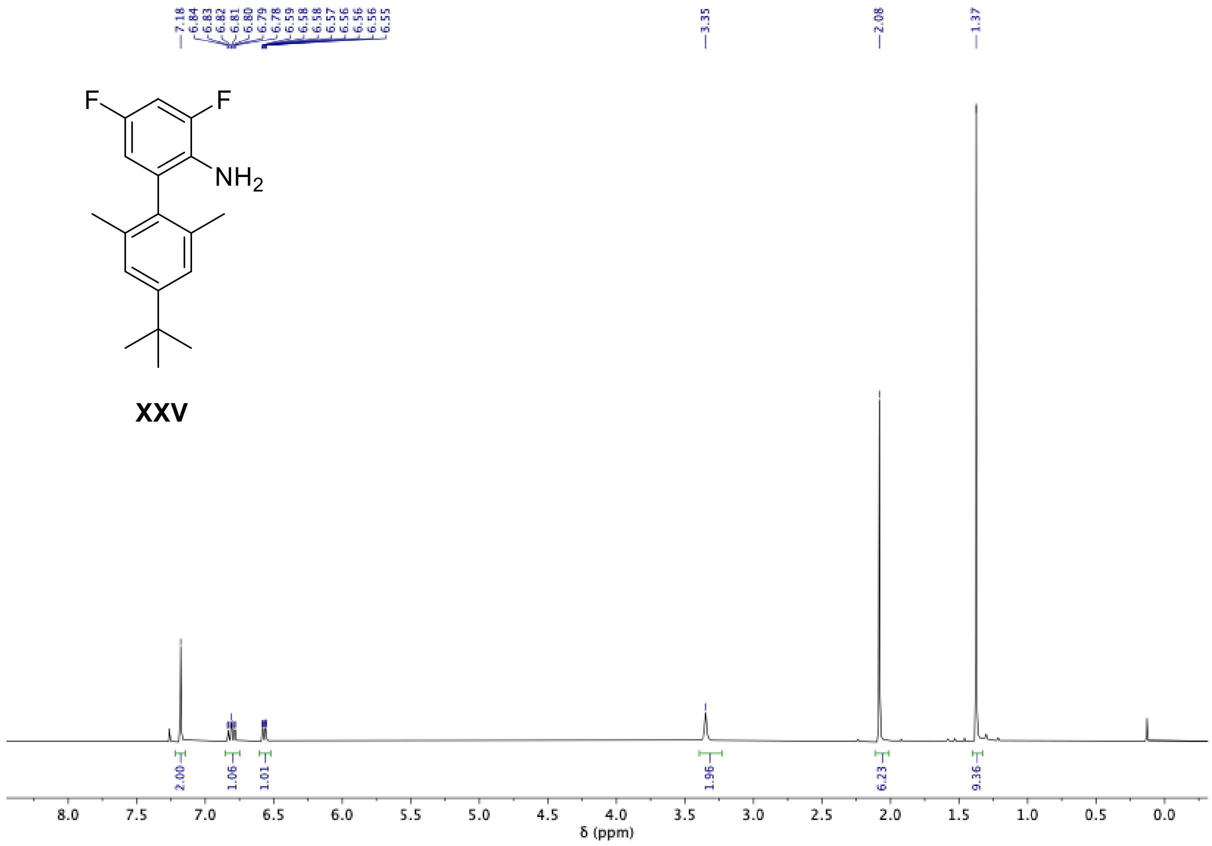
F
F
NH2
XXV
7.18
6.84
6.83
6.82
6.81
6.80
6.79
6.78
6.59
6.58
6.58
6.57
6.56
6.56
6.56
6.55
3.35
2.08
1.37
2.00
1.06
1.01
1.96
6.23
9.36
8.0 7.5 7.0 6.5 6.0 5.5 5.0 4.5 4.0 3.5 3.0 2.5 2.0 1.5 1.0 0.5 0.0
δ (ppm)

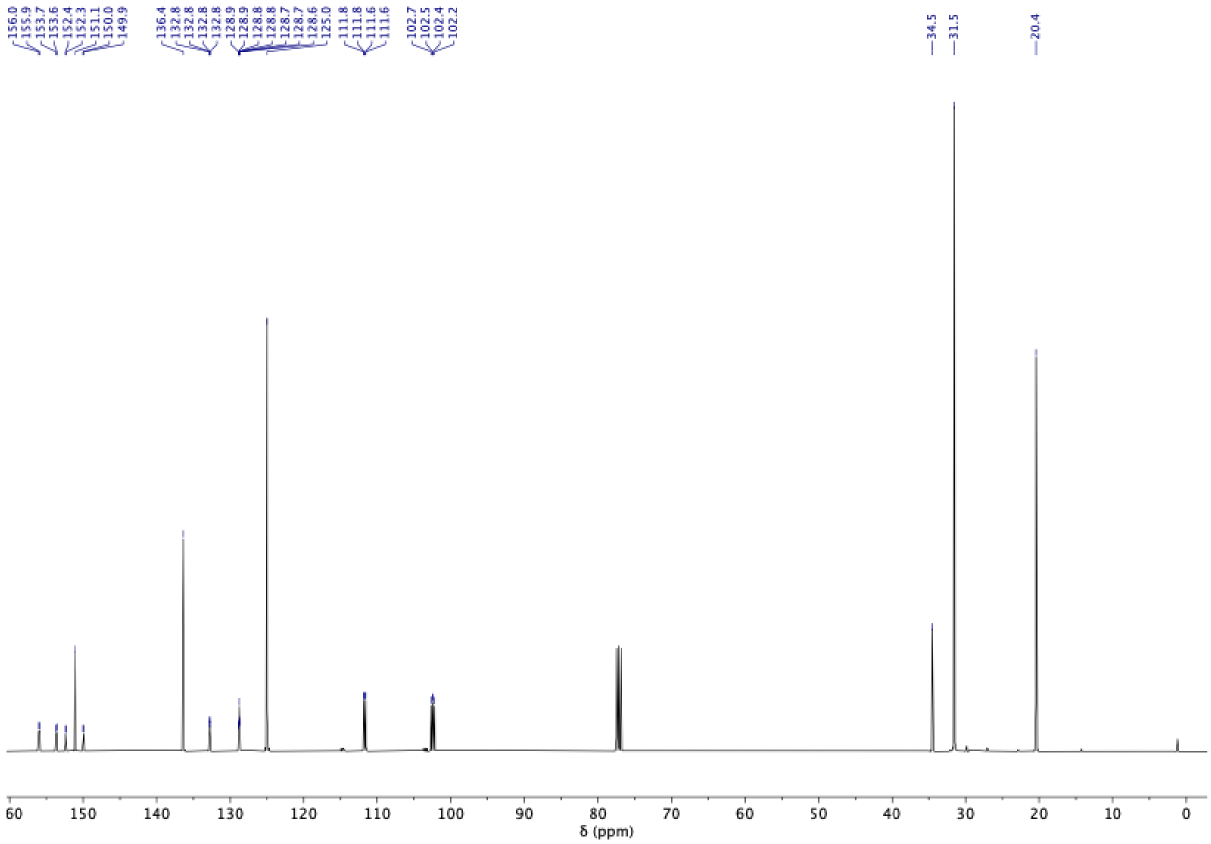
156.0
155.9
153.7
153.6
152.4
152.3
151.1
150.0
149.9
136.4
132.8
132.8
132.8
132.8
128.9
128.9
128.8
128.8
128.7
128.7
128.6
125.0
111.8
111.8
111.6
111.6
102.7
102.5
102.4
102.2
34.5
31.5
20.4
160 150 140 130 120 110 100 90 80 70 60 50 40 30 20 10 0
δ (ppm)

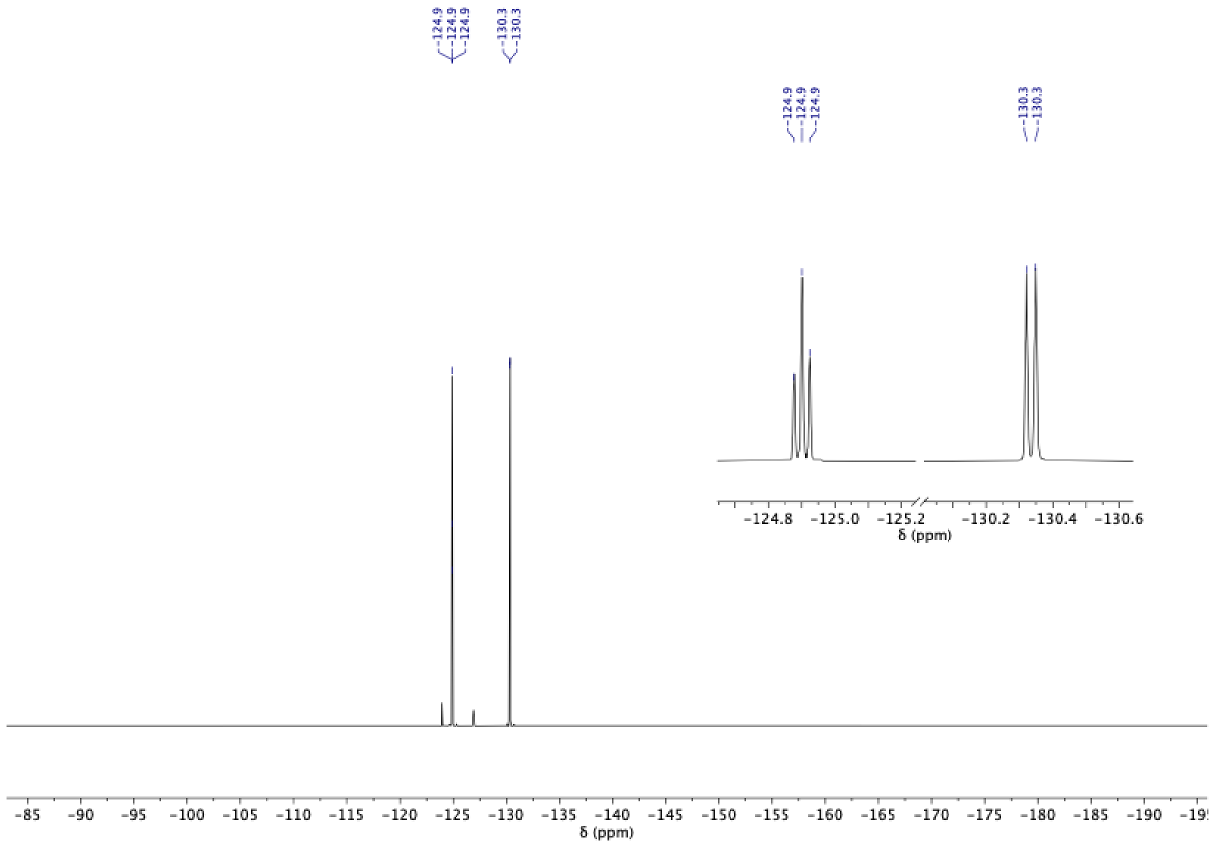
-124.9
-124.9
-124.9
-130.3
-130.3
-124.8 -125.0 -125.2 -130.2 -130.4 -130.6
δ (ppm)
-85 -90 -95 -100 -105 -110 -115 -120 -125 -130 -135 -140 -145 -150 -155 -160 -165 -170 -175 -180 -185 -190
δ (ppm)

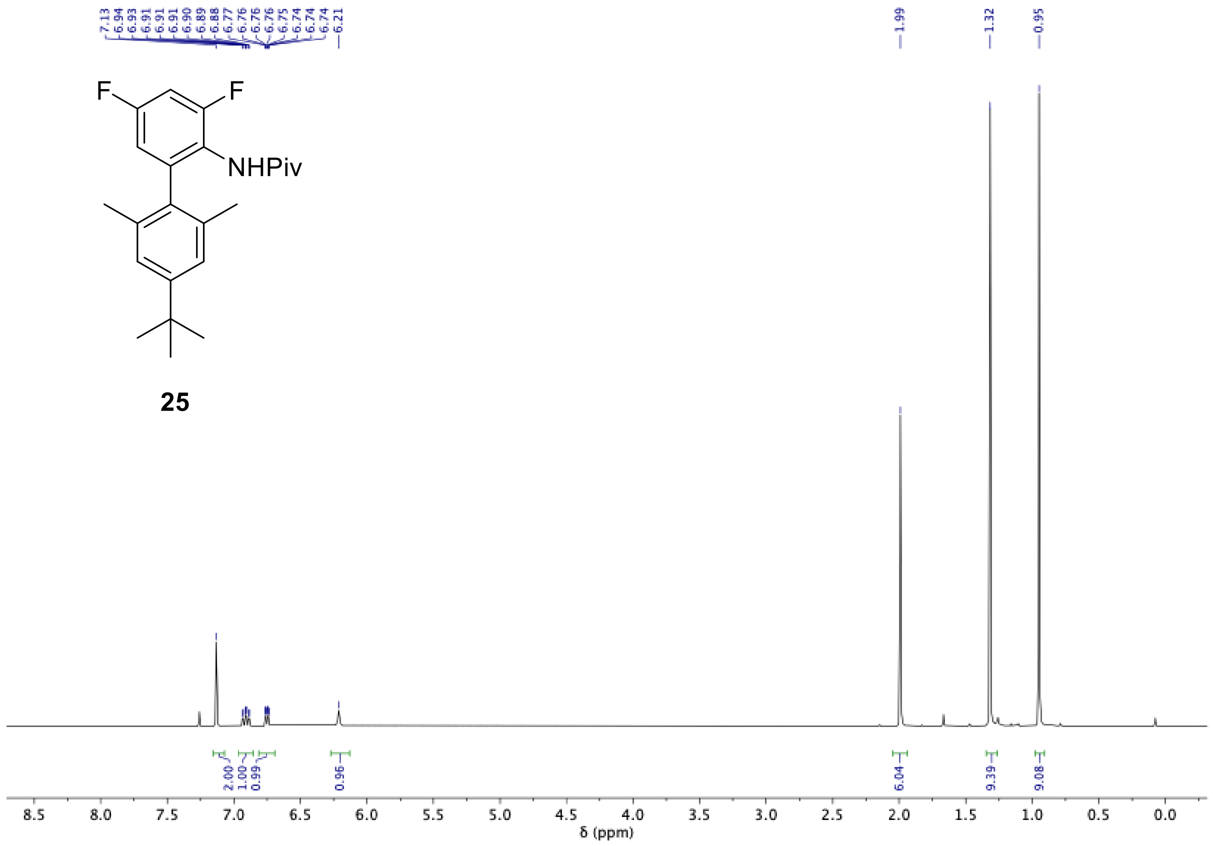
7.13 6.94 6.93 6.91 6.91 6.91 6.90 6.89 6.88 6.77 6.76 6.76 6.75 6.74 6.74 6.74 6.21
1.99
1.32
0.95
F
F
NHPiv
25
2.00 1.00 0.99
0.96
6.04
9.39
9.08
8.5 8.0 7.5 7.0 6.5 6.0 5.5 5.0 4.5 4.0 3.5 3.0 2.5 2.0 1.5 1.0 0.5 0.0
δ (ppm)

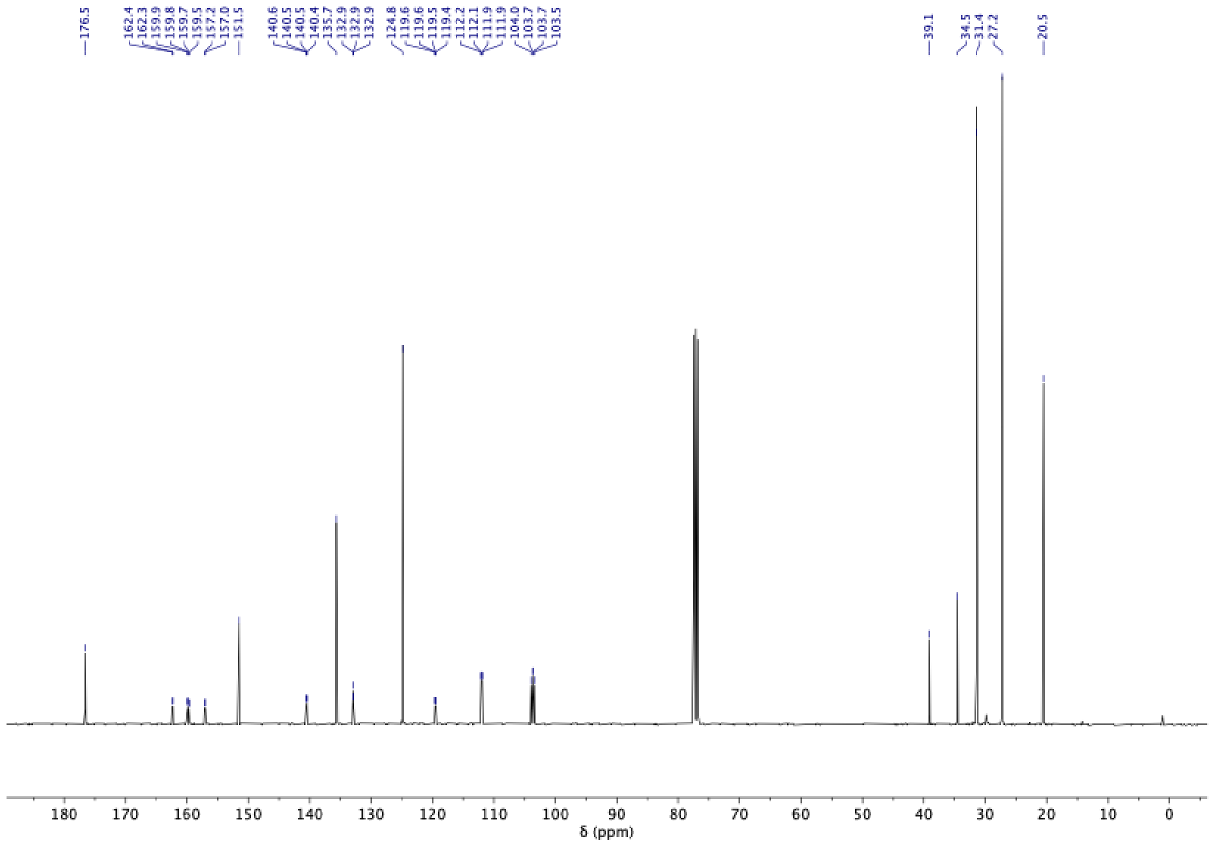
176.5
162.4 162.3 159.9 159.8 159.7 159.5 157.2 157.0 151.5
140.6 140.5 140.5 140.4 135.7 132.9 132.9 132.9
124.8 119.6 119.6 119.5 119.4 112.2 112.1 111.9 111.9 104.0 103.7 103.7 103.5
39.1
34.5 31.4 27.2
20.5
180 170 160 150 140 130 120 110 100 90 80 70 60 50 40 30 20 10 0
δ (ppm)

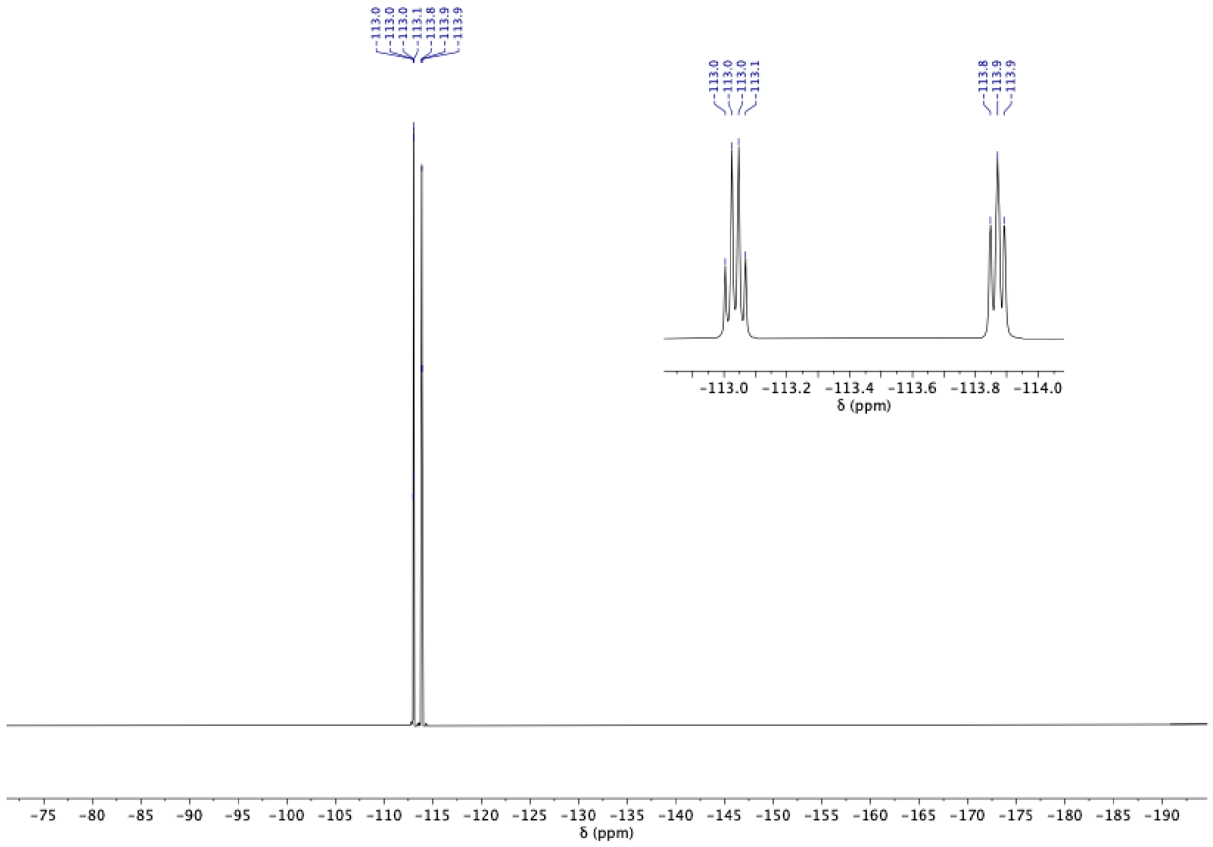
-113.0
-113.0
-113.0
-113.1
-113.8
-113.9
-113.9
-113.0 -113.2 -113.4 -113.6 -113.8 -114.0
δ (ppm)
-75 -80 -85 -90 -95 -100 -105 -110 -115 -120 -125 -130 -135 -140 -145 -150 -155 -160 -165 -170 -175 -180 -185 -190
δ (ppm)

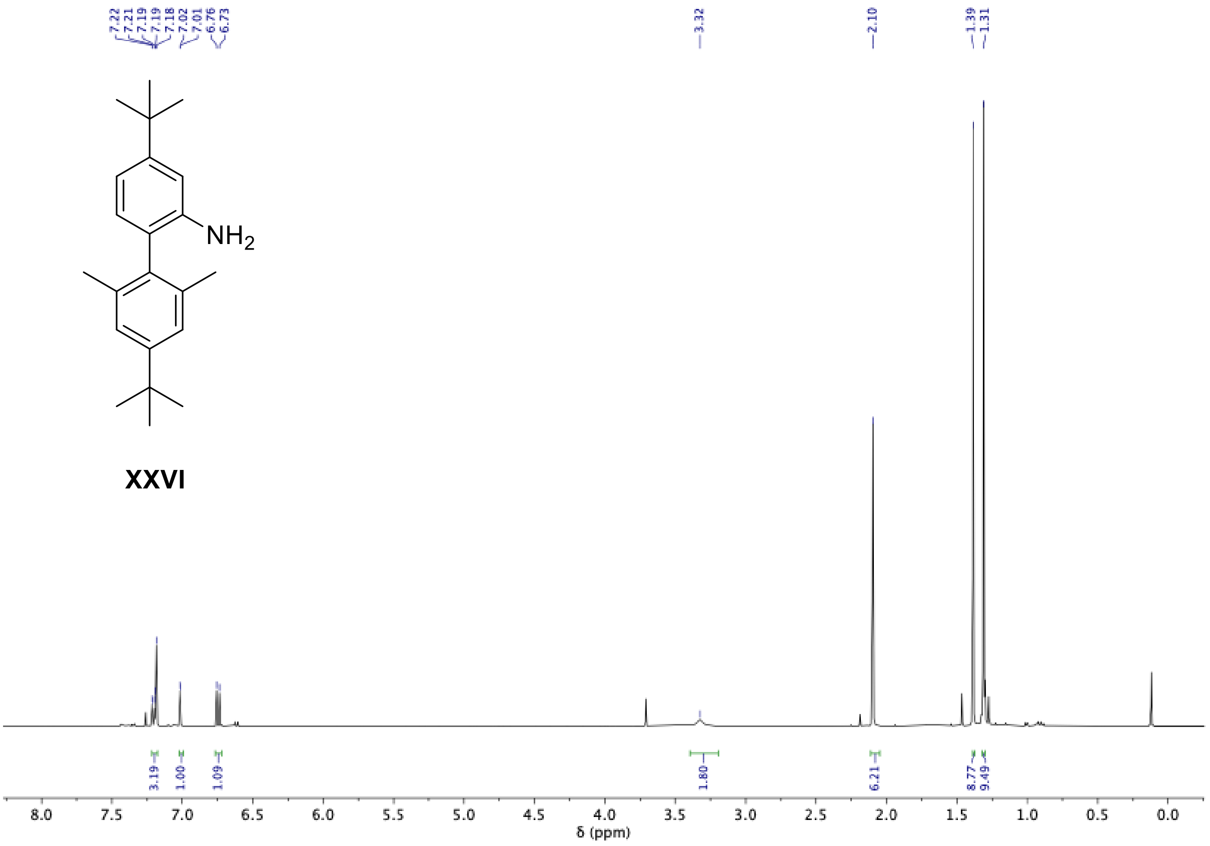
NH2
7.22
7.21
7.19
7.19
7.18
7.02
7.01
6.76
6.73
3.32
2.10
1.39
1.31
3.19
1.00
1.09
1.80
6.21
8.77
9.49
δ (ppm)

XXVI

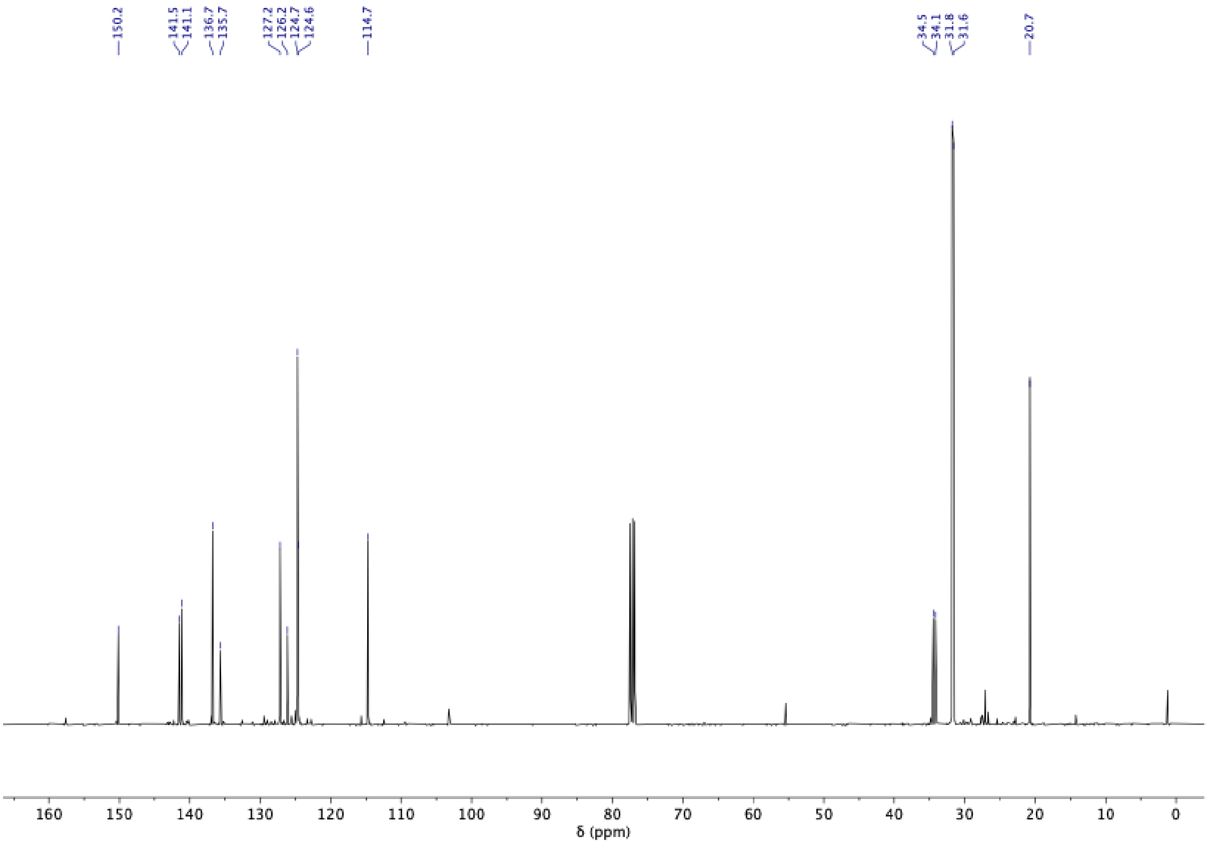
150.2
141.5
141.1
136.7
135.7
127.2
126.2
124.7
124.6
114.7
34.5
34.1
31.8
31.6
20.7
δ (ppm)

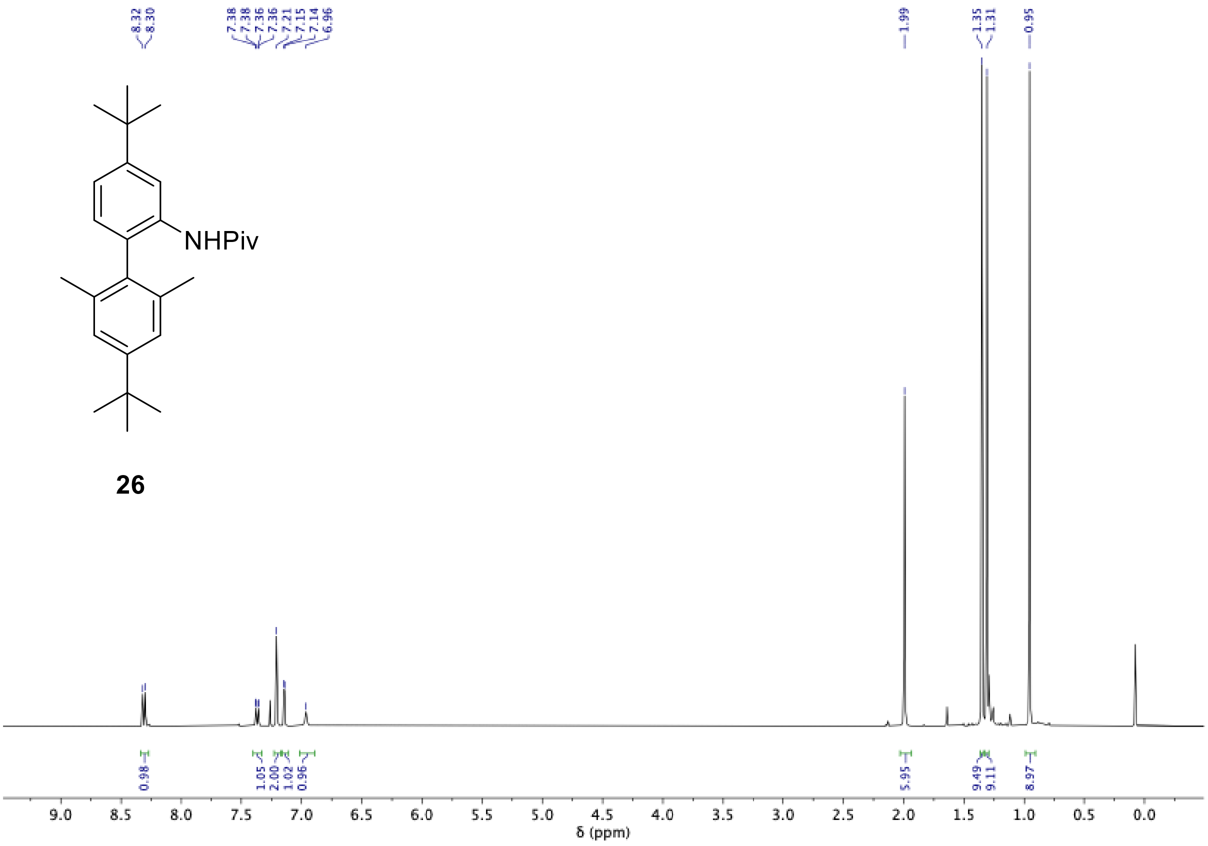
NHPiv
26
8.32
8.30
7.38
7.38
7.36
7.36
7.21
7.15
7.14
6.96
1.99
1.35
1.31
0.95
0.98
1.05
2.00
1.02
0.96
5.95
9.49
9.11
8.97
9.0 8.5 8.0 7.5 7.0 6.5 6.0 5.5 5.0 4.5 4.0 3.5 3.0 2.5 2.0 1.5 1.0 0.5 0.0
δ (ppm)

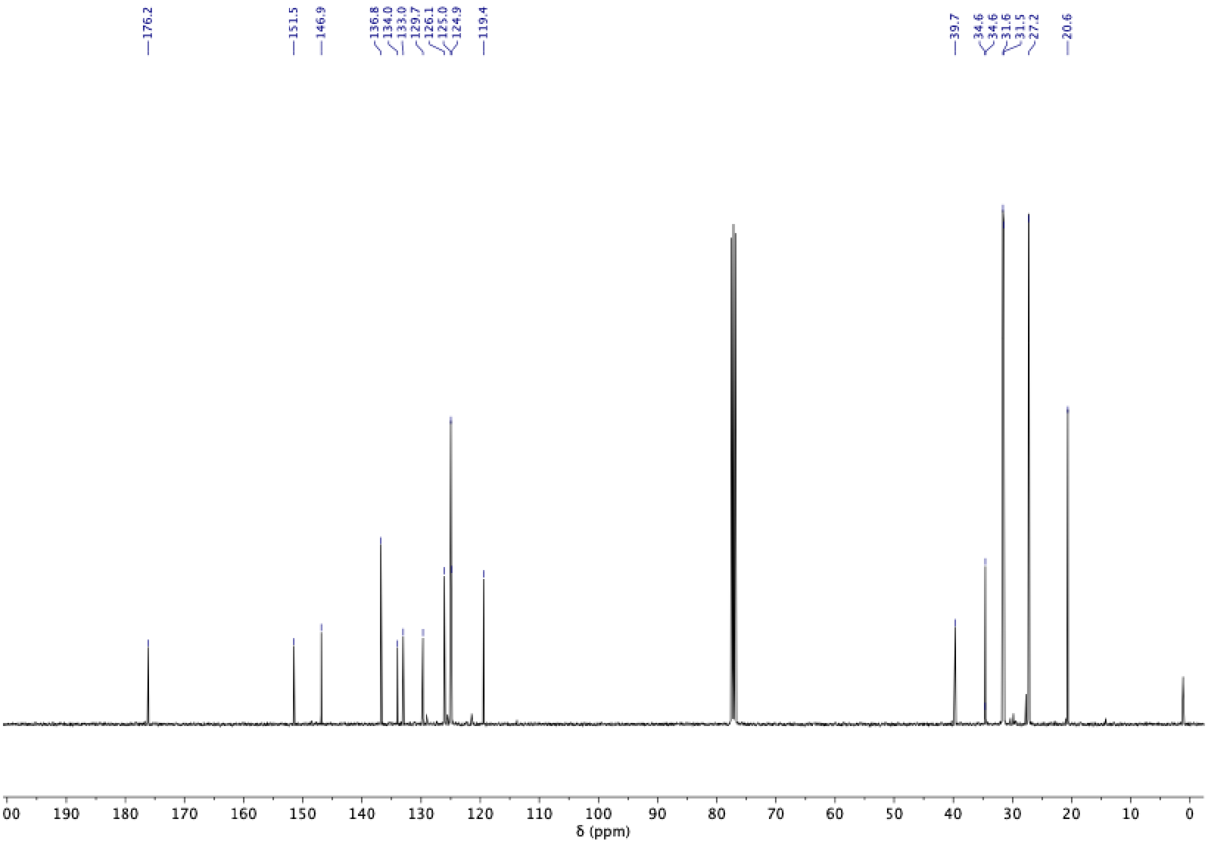
176.2
151.5
146.9
136.8
134.0
133.0
129.7
126.1
125.0
124.9
119.4
39.7
34.6
34.6
31.6
31.5
27.2
20.6
00 190 180 170 160 150 140 130 120 110 100 90 80 70 60 50 40 30 20 10 0
δ (ppm)

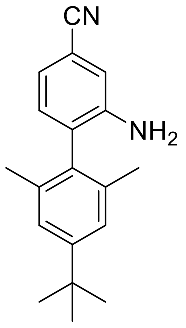
CN
NH2

XXVII

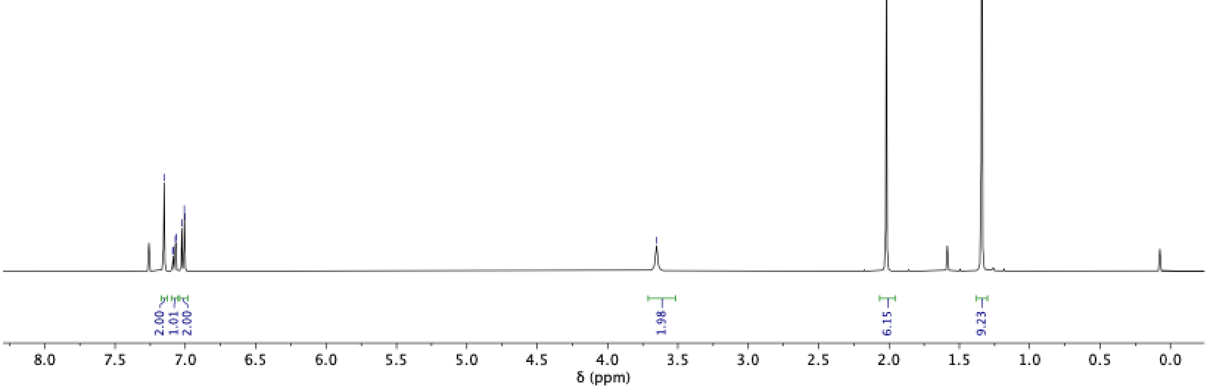
7.15
7.09
7.09
7.07
7.07
7.02
7.01
7.00
3.65
2.02
1.34
2.00
1.01
2.00
1.98
6.15
9.23
δ (ppm)

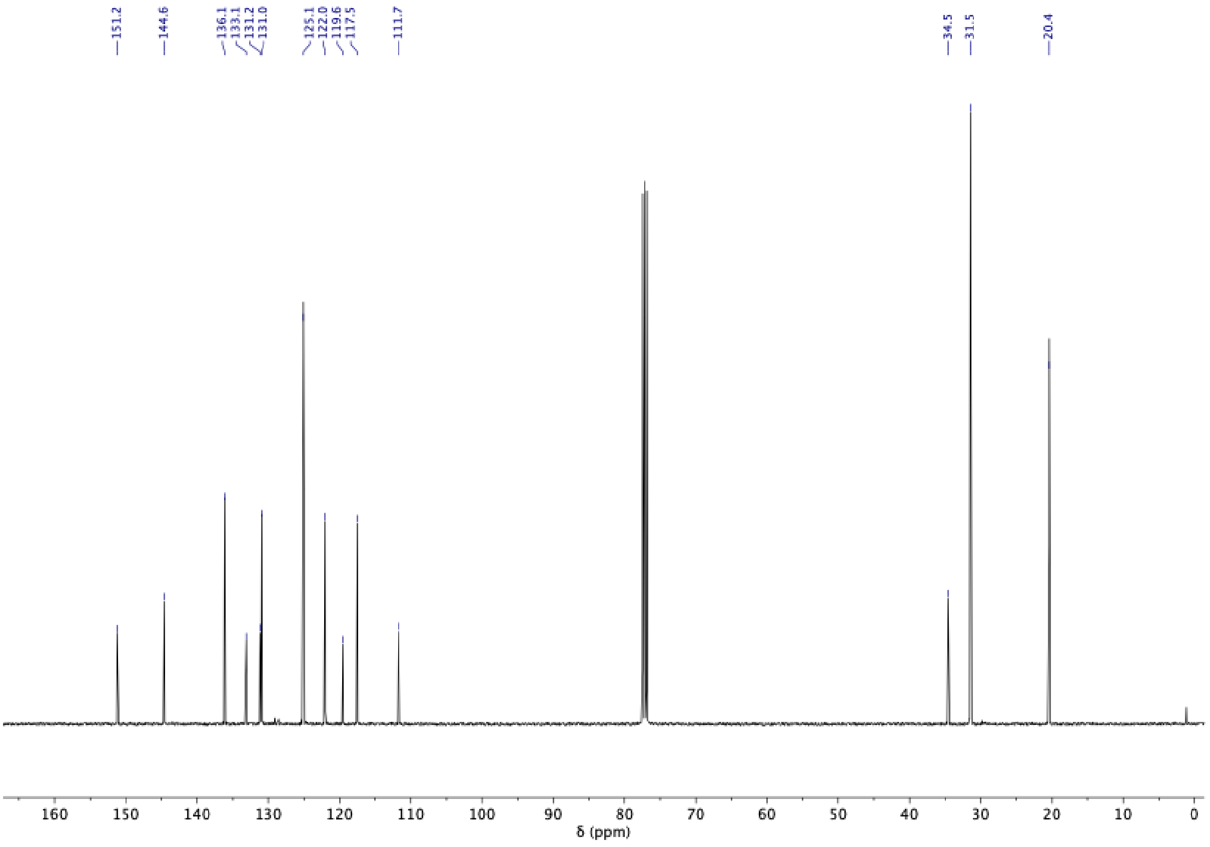
151.2
144.6
136.1
133.1
131.2
131.0
125.1
122.0
119.6
117.5
111.7
34.5
31.5
20.4
δ (ppm)

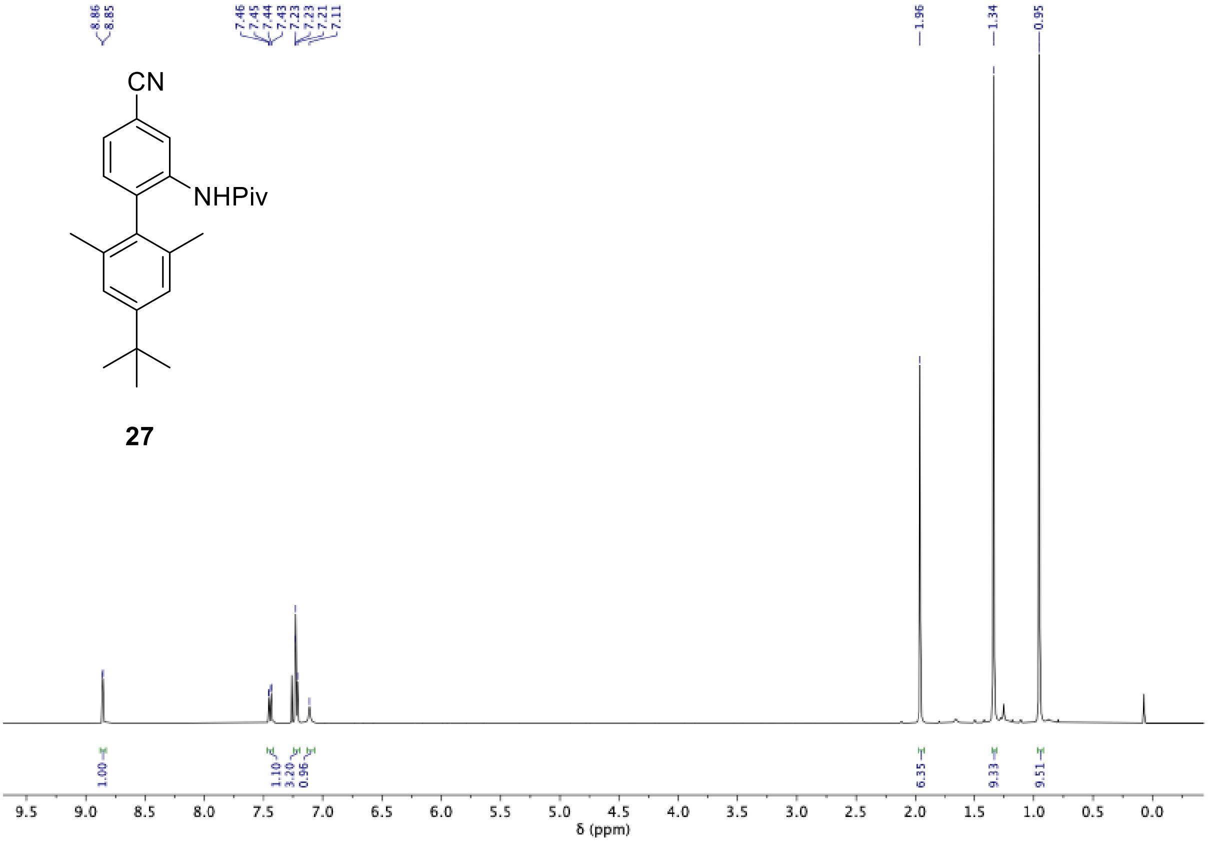
CN
NHPiv

27

8.86
8.85
7.46
7.45
7.44
7.43
7.23
7.23
7.21
7.11
1.96
1.34
0.95
1.00
1.10
3.20
0.98
6.35
9.33
9.51
δ (ppm)

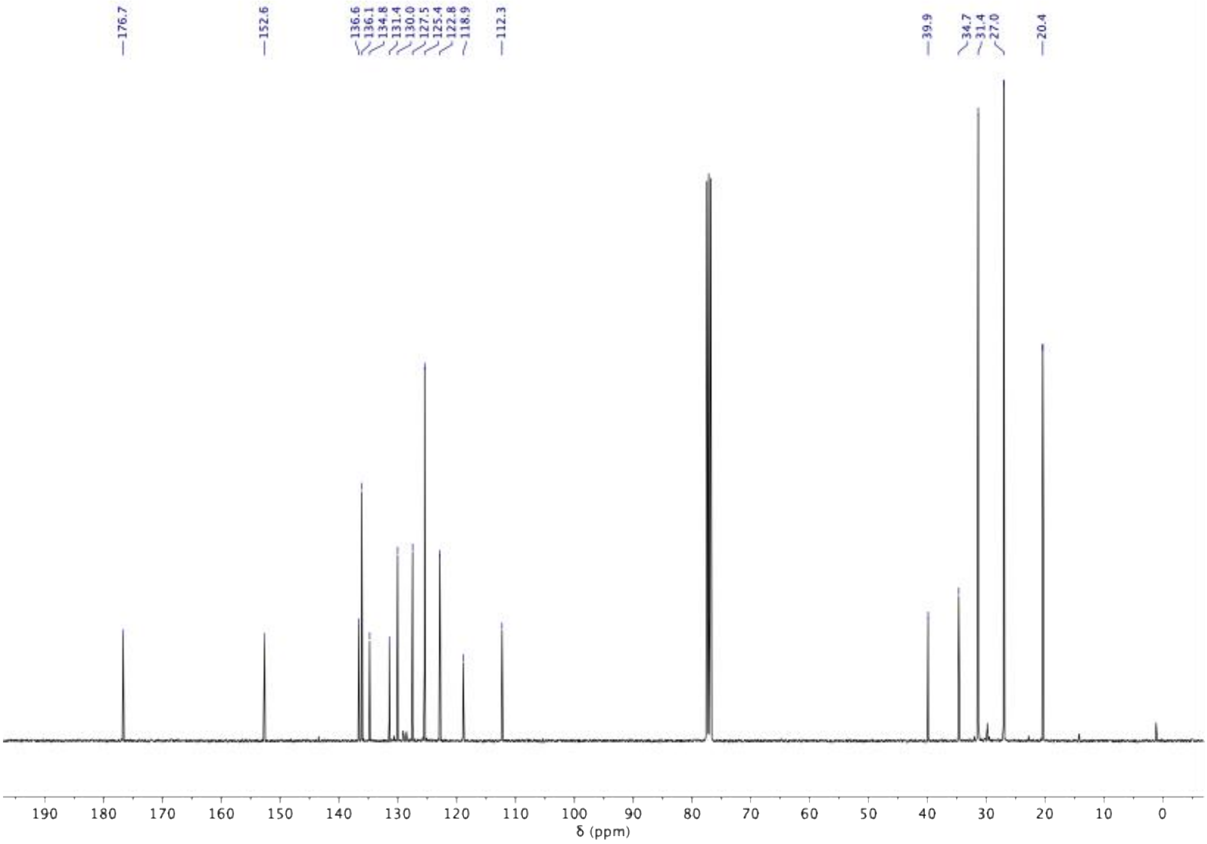
176.7
152.6
136.6
136.1
134.8
131.4
130.0
127.5
125.4
122.8
118.9
112.3
39.9
34.7
31.4
27.0
20.4
δ (ppm)

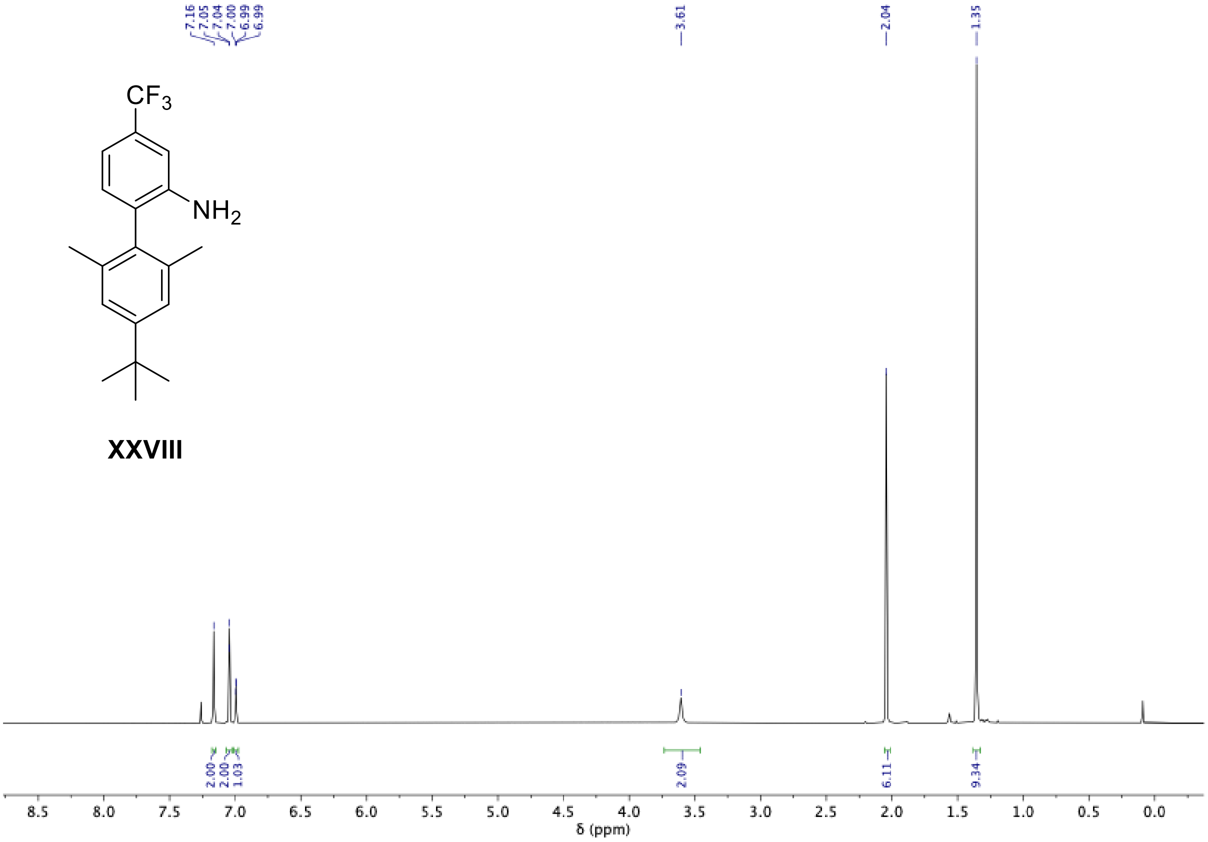
7.16
7.05
7.04
7.00
6.99
6.99
3.61
2.04
1.35
CF3
NH2
2.00
2.00
1.03
2.09
6.11
9.34
8.5 8.0 7.5 7.0 6.5 6.0 5.5 5.0 4.5 4.0 3.5 3.0 2.5 2.0 1.5 1.0 0.5 0.0
δ (ppm)

XXVIII

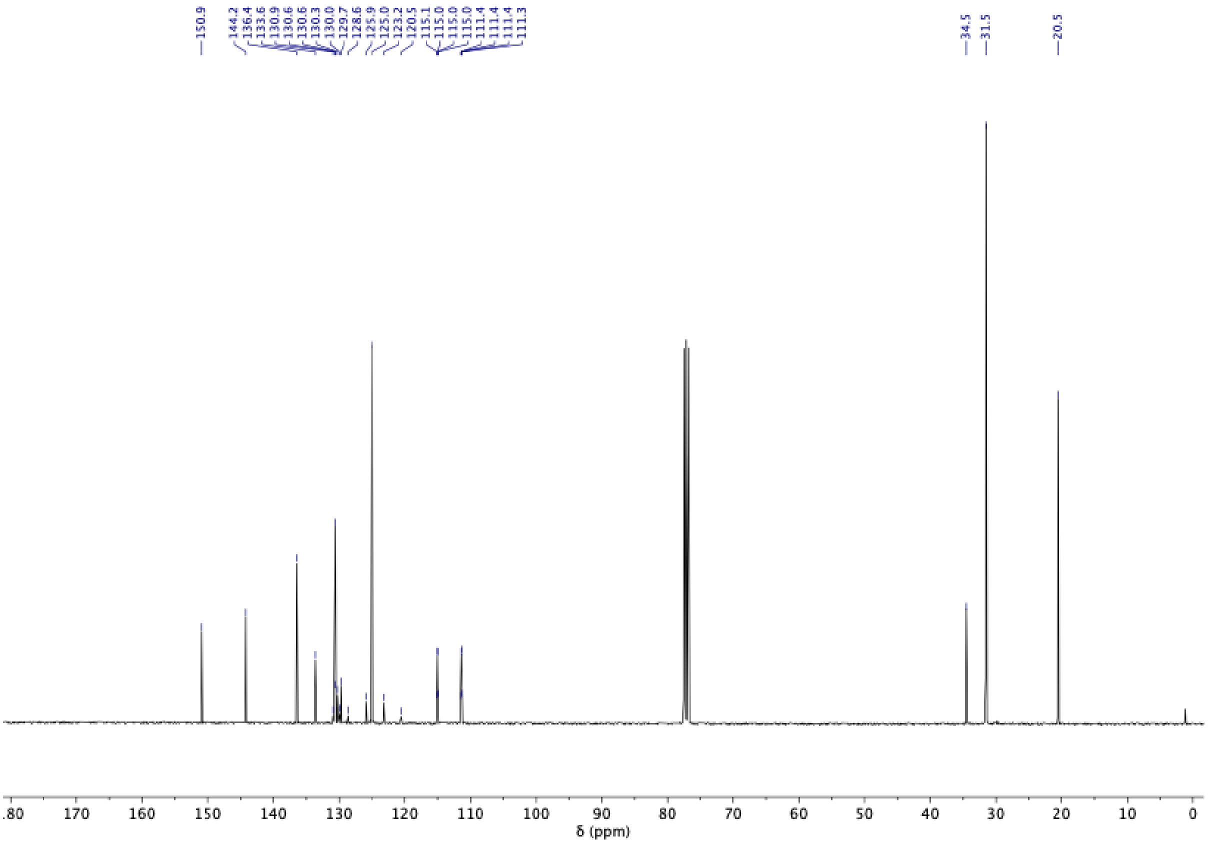
150.9
144.2
136.4
133.6
130.9
130.6
130.6
130.3
130.0
129.7
128.6
125.9
125.0
123.2
120.5
115.1
115.0
115.0
115.0
111.4
111.4
111.4
111.3
34.5
31.5
20.5
180 170 160 150 140 130 120 110 100 90 80 70 60 50 40 30 20 10 0
δ (ppm)

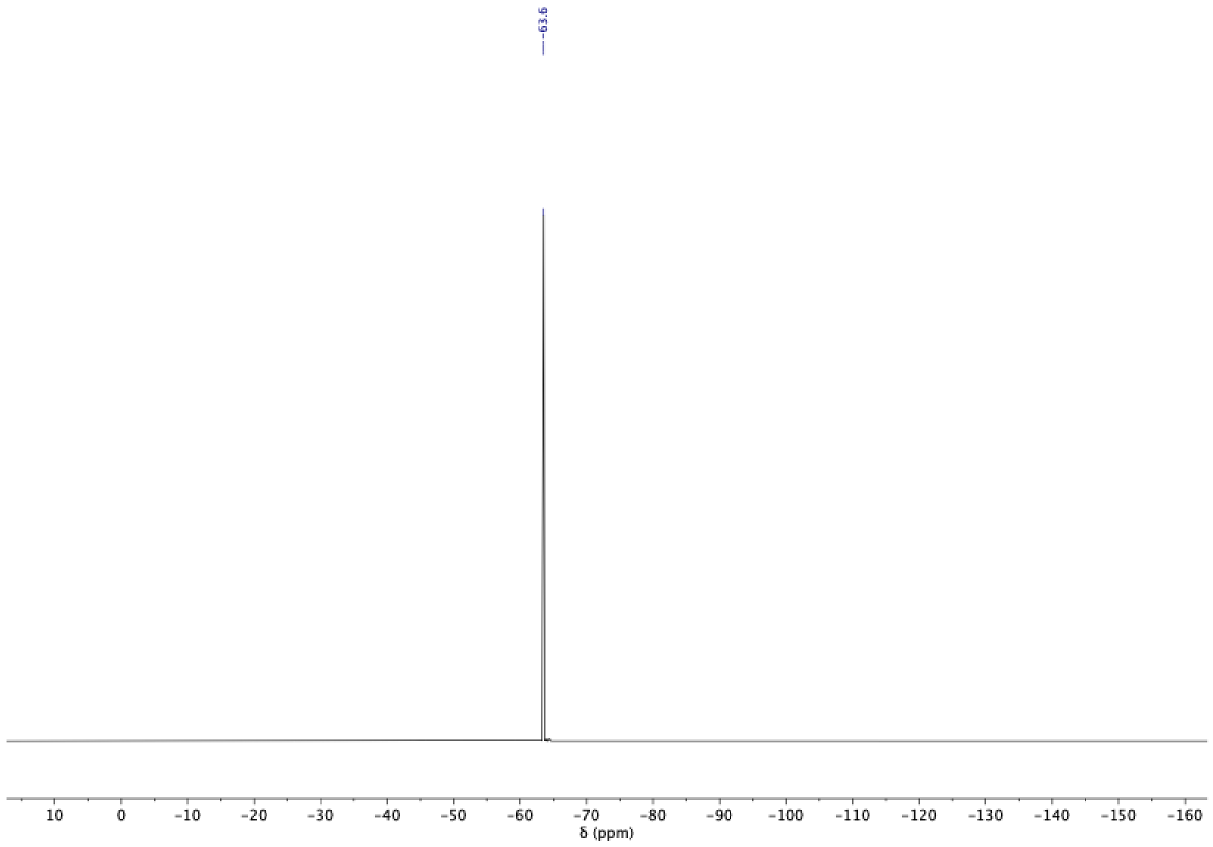
-63.6
10
0
-10
-20
-30
-40
-50
-60
-70
-80
-90
-100
-110
-120
-130
-140
-150
-160
δ (ppm)

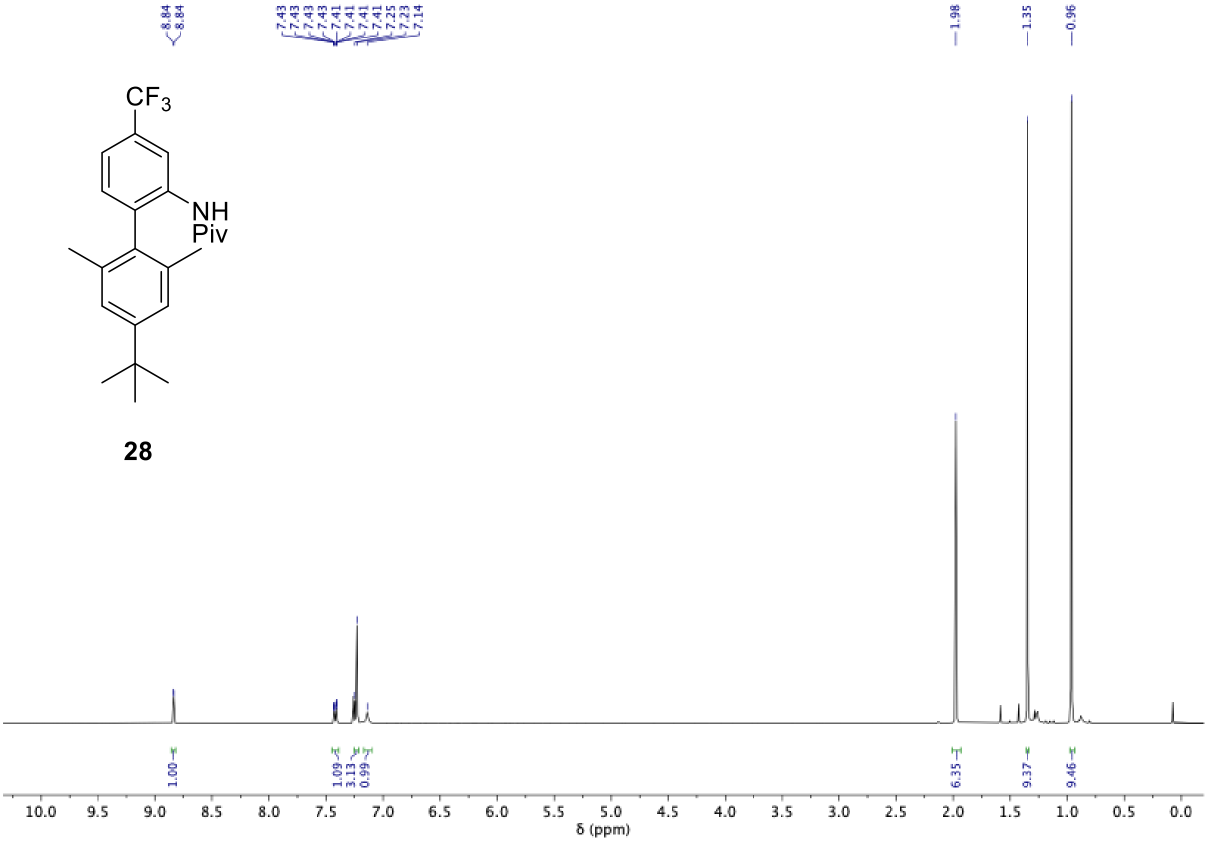
CF3
NH
Piv
28
8.84
8.84
7.43
7.43
7.43
7.43
7.41
7.41
7.41
7.41
7.25
7.23
7.14
1.98
1.35
0.96
1.00
1.09
3.13
0.99
6.35
9.37
9.46
10.0 9.5 9.0 8.5 8.0 7.5 7.0 6.5 6.0 5.5 5.0 4.5 4.0 3.5 3.0 2.5 2.0 1.5 1.0 0.5 0.0
δ (ppm)

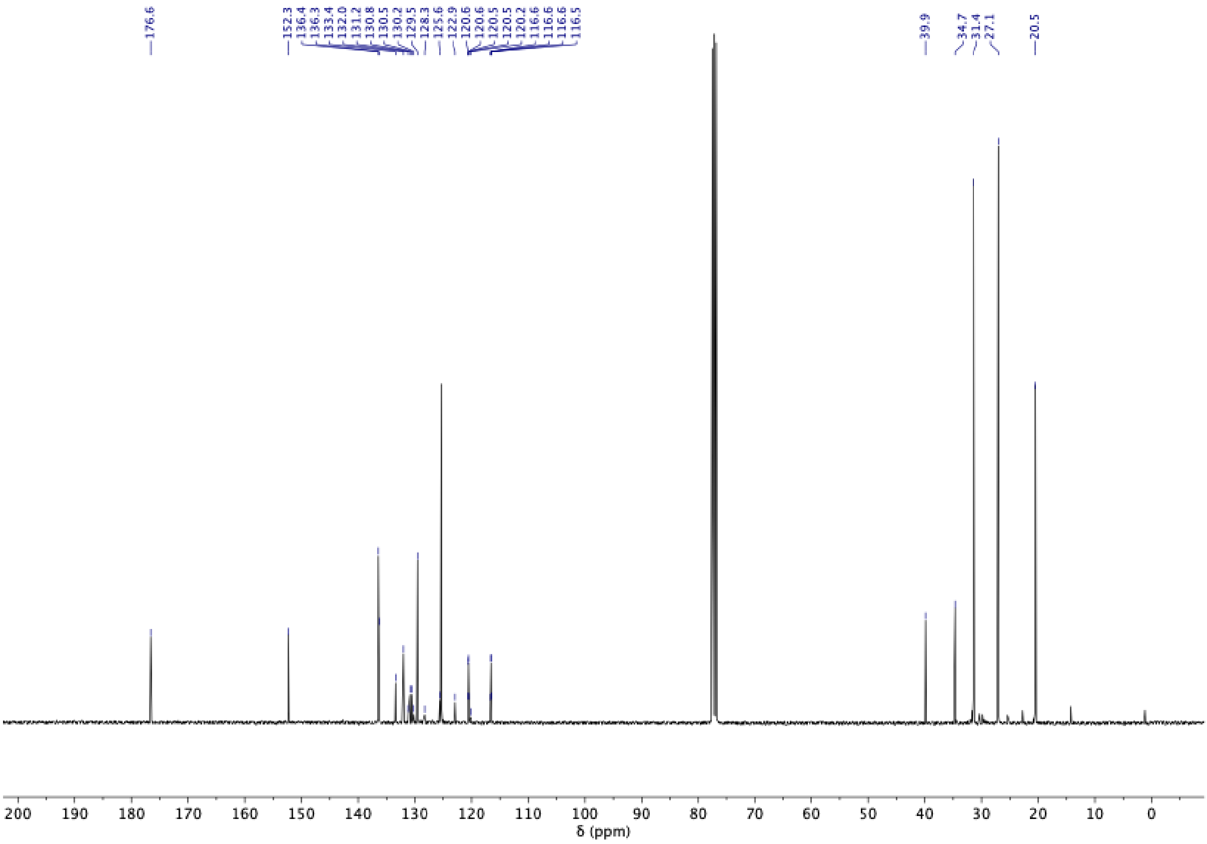
176.6
152.3
136.4
136.3
133.4
132.0
131.2
130.8
130.5
130.2
129.5
128.3
125.6
122.9
120.6
120.6
120.5
120.5
120.2
116.6
116.6
116.6
116.5
39.9
34.7
31.4
27.1
20.5
200 190 180 170 160 150 140 130 120 110 100 90 80 70 60 50 40 30 20 10 0
δ (ppm)

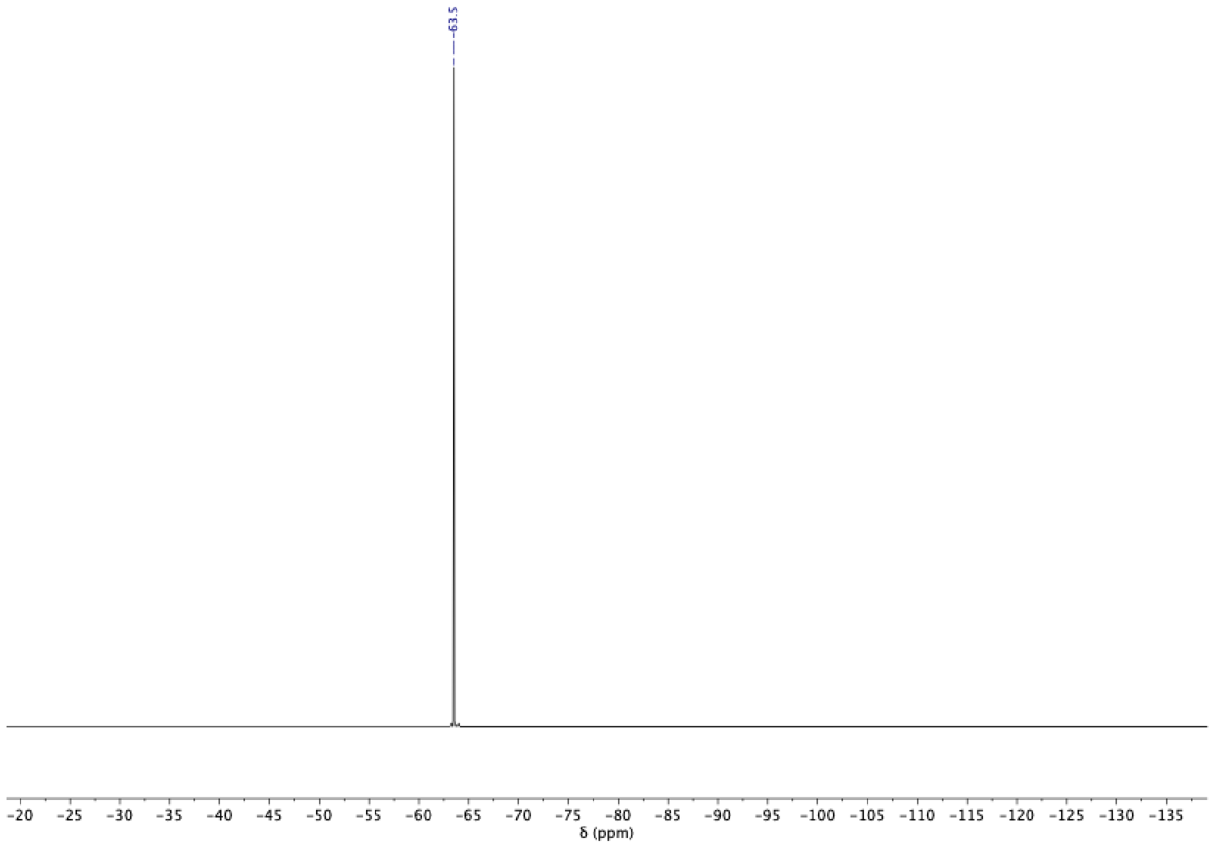
-63.5
-20 -25 -30 -35 -40 -45 -50 -55 -60 -65 -70 -75 -80 -85 -90 -95 -100 -105 -110 -115 -120 -125 -130 -135
δ (ppm)

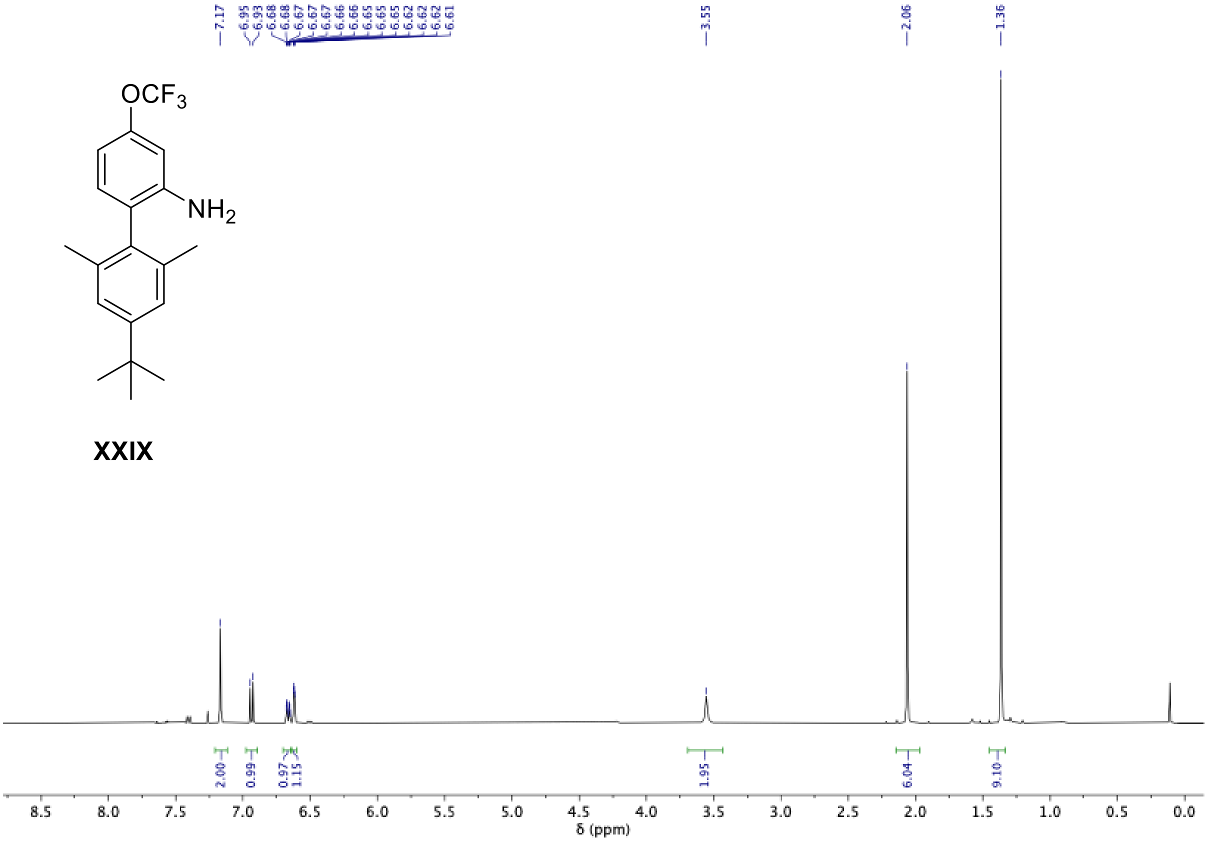
OCF$_3$
NH$_2$
XXIX
7.17
6.95
6.93
6.68
6.67
6.67
6.66
6.66
6.65
6.65
6.65
6.62
6.62
6.61
3.55
2.06
1.36
2.00
0.99
0.97
1.15
1.95
6.04
9.10
δ (ppm)

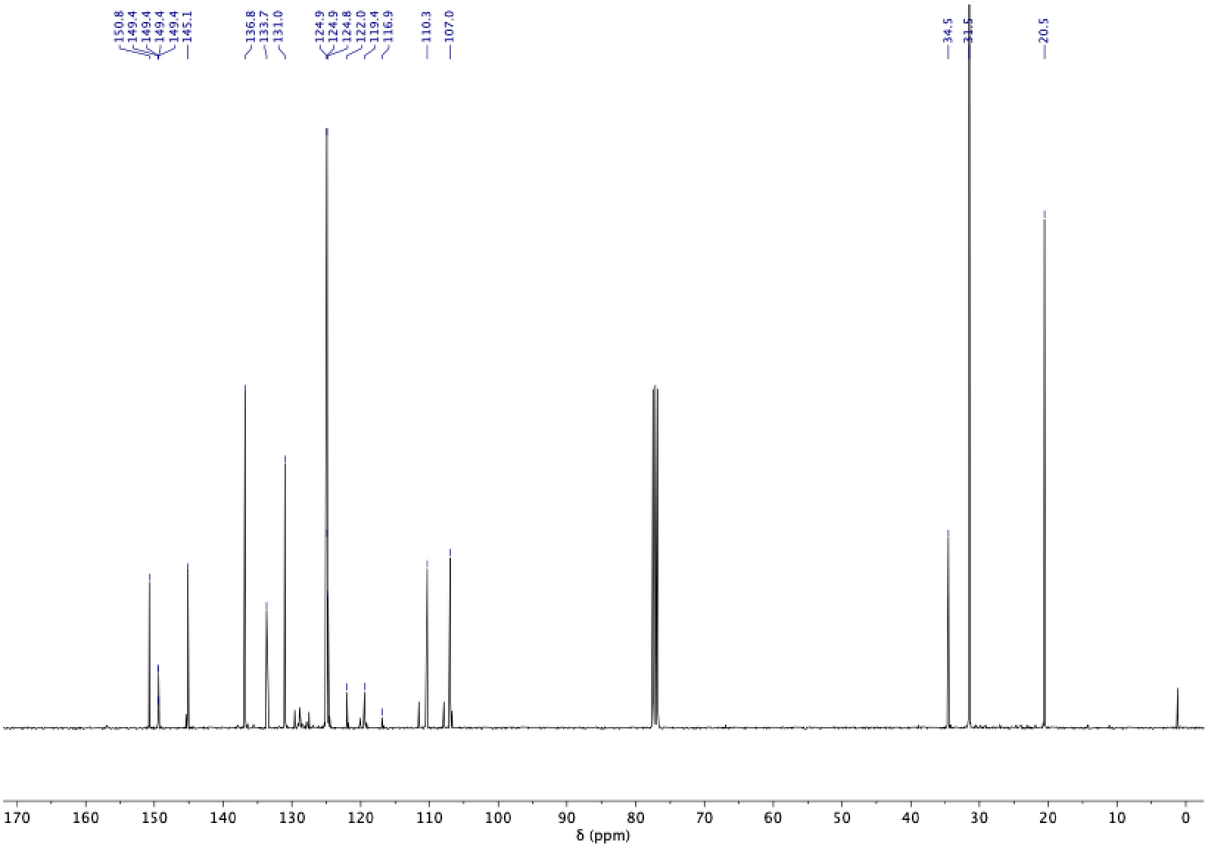
150.8
149.4
149.4
149.4
149.4
145.1
136.8
133.7
131.0
124.9
124.9
124.8
122.0
119.4
116.9
110.3
107.0
34.5
31.5
20.5
δ (ppm)

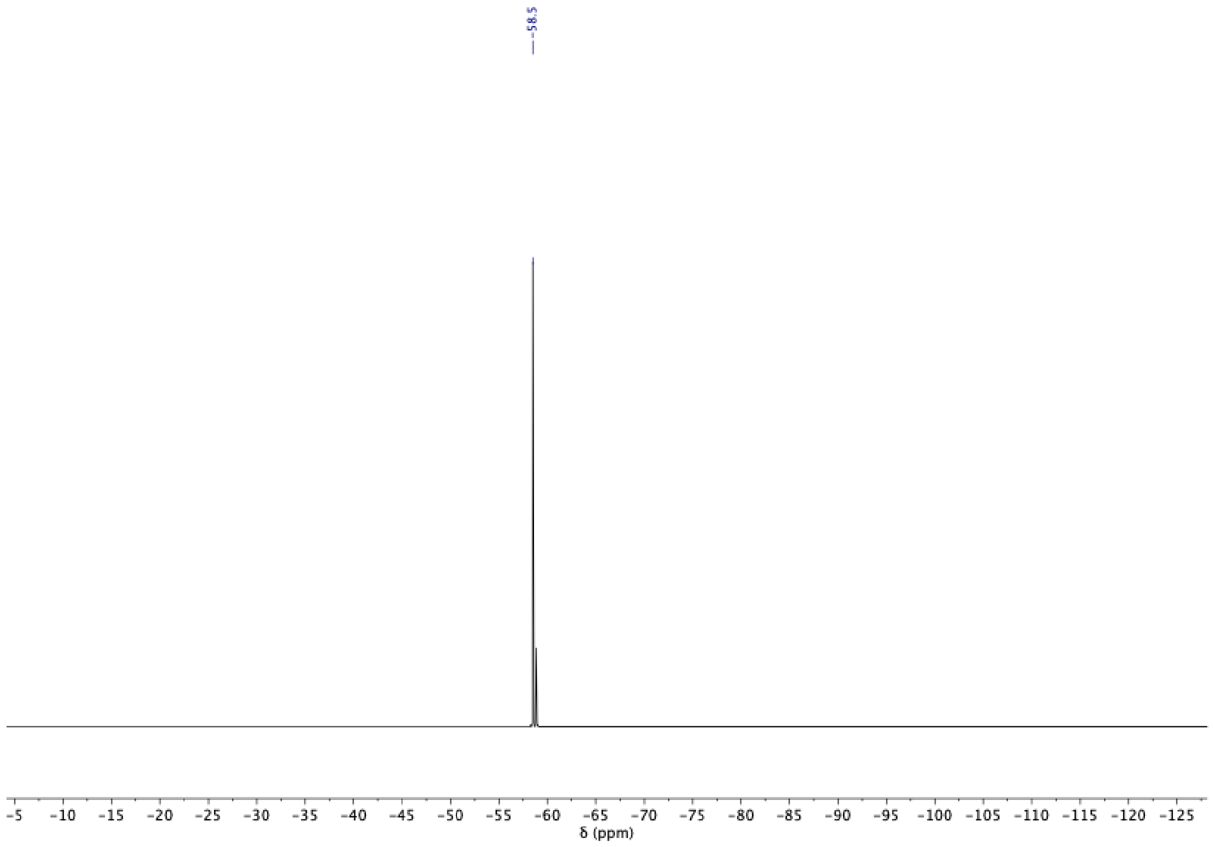
-58.5
-5 -10 -15 -20 -25 -30 -35 -40 -45 -50 -55 -60 -65 -70 -75 -80 -85 -90 -95 -100 -105 -110 -115 -120 -125
δ (ppm)

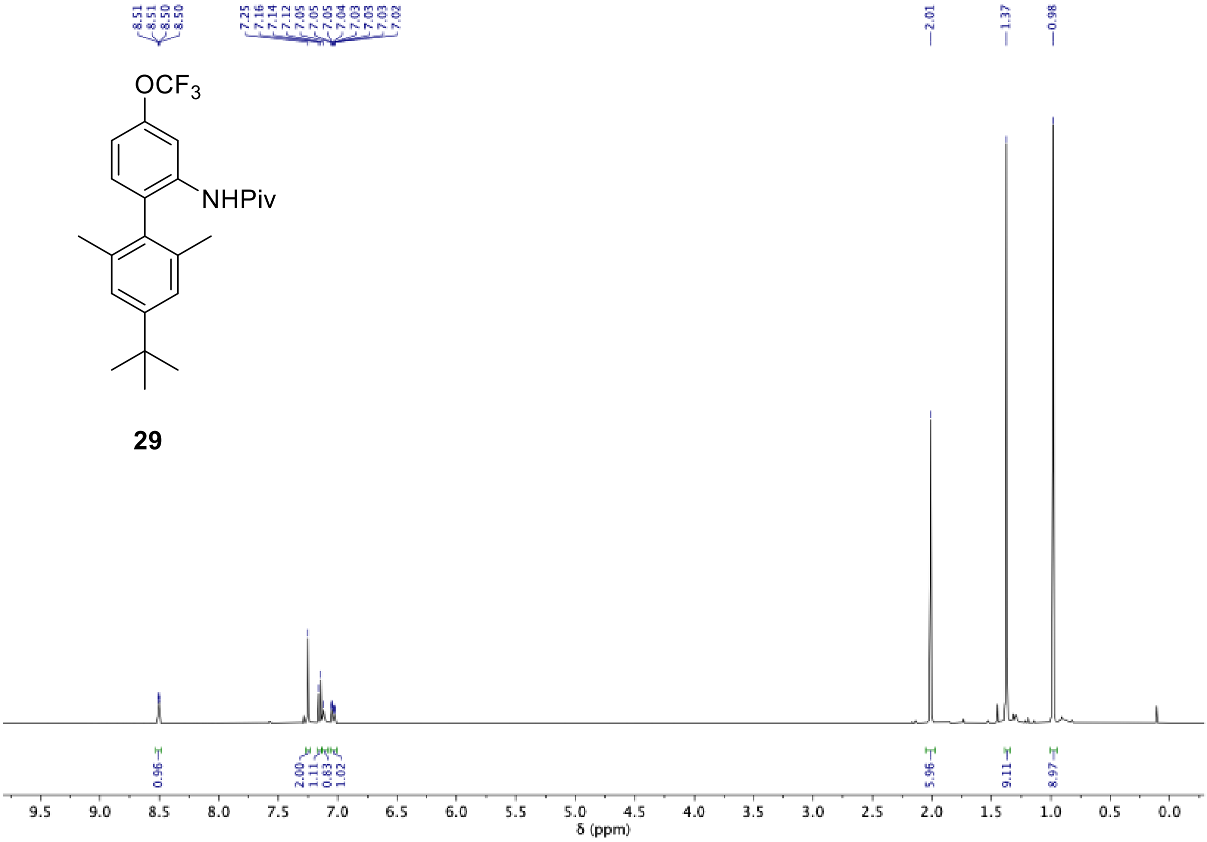

8.51 8.51 8.50 8.50
7.25 7.16 7.14 7.12 7.05 7.05 7.05 7.04 7.03 7.03 7.02
2.01
1.37
0.98
OCF3
NHPiv
0.96
2.00 1.11 0.83 1.02
5.96
9.11
8.97
9.5 9.0 8.5 8.0 7.5 7.0 6.5 6.0 5.5 5.0 4.5 4.0 3.5 3.0 2.5 2.0 1.5 1.0 0.5 0.0
δ (ppm)

29

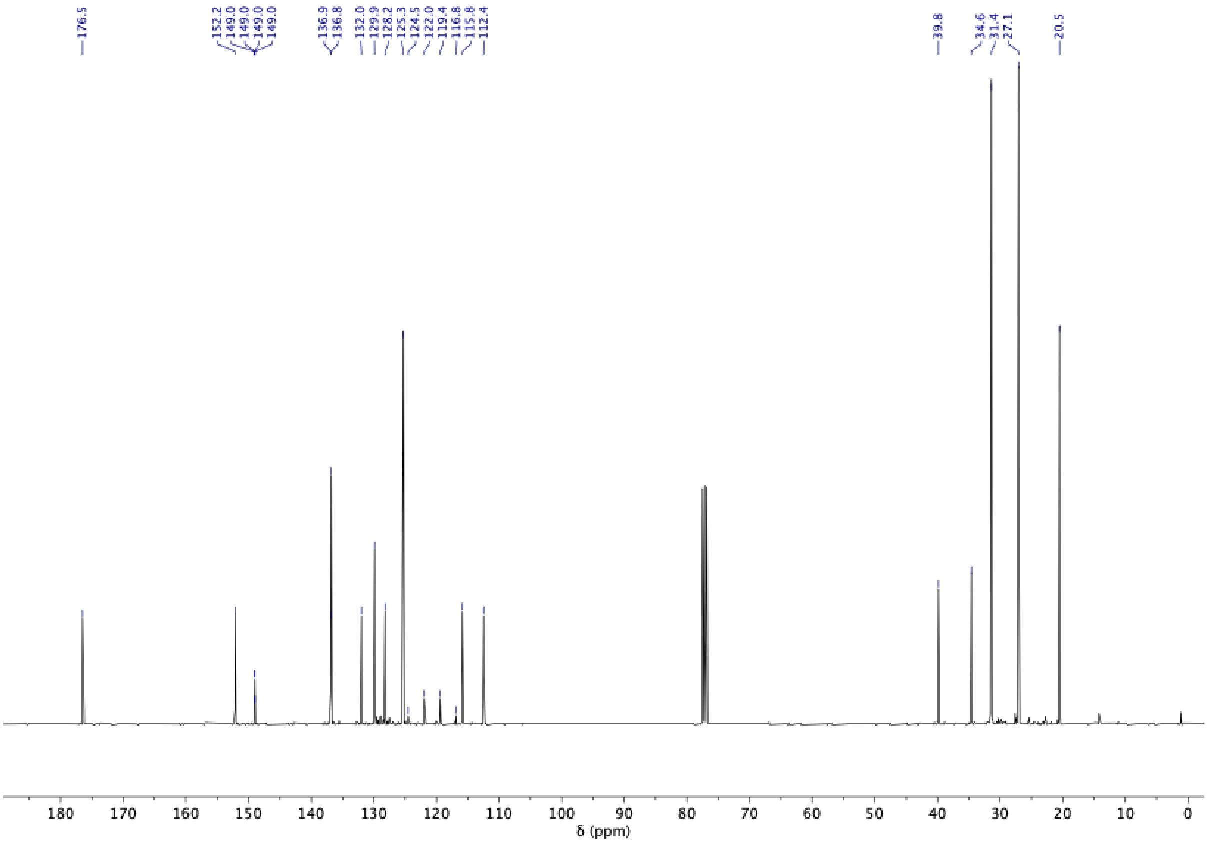

176.5
152.2 149.0 149.0 149.0 149.0
136.9 136.8
132.0 129.9 128.2 125.3 124.5 122.0 119.4 116.8 115.8 112.4
39.8
34.6 31.4 27.1
20.5
180 170 160 150 140 130 120 110 100 90 80 70 60 50 40 30 20 10 0
δ (ppm)

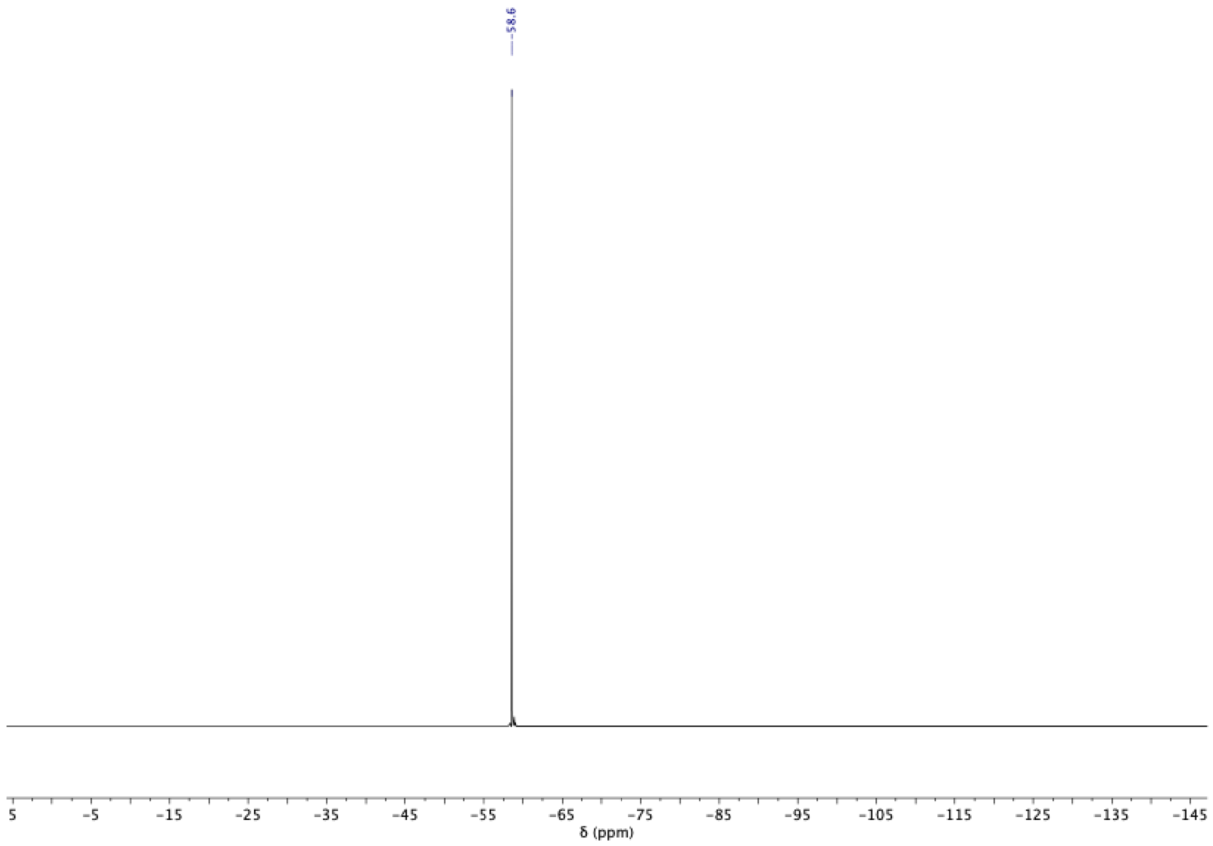
-58.6
5
-5
-15
-25
-35
-45
-55
-65
-75
-85
-95
-105
-115
-125
-135
-145
δ (ppm)

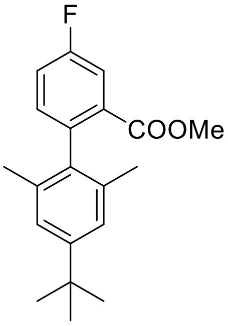
F
COOMe

30

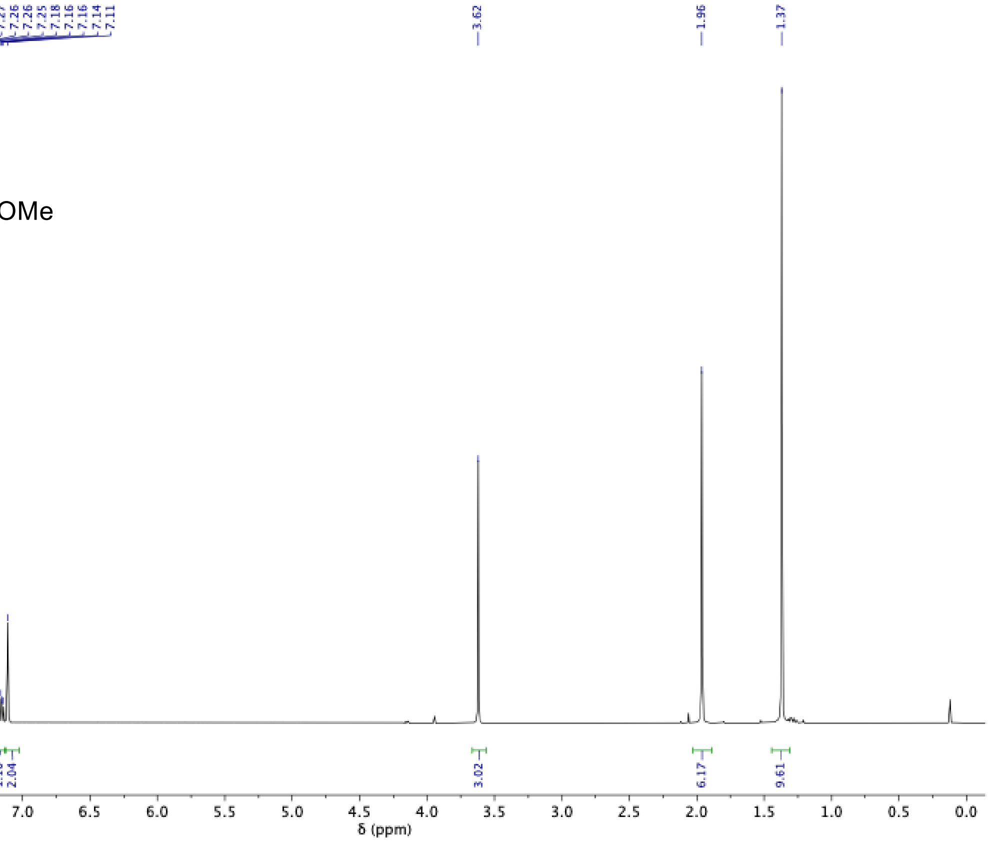
7.73
7.73
7.71
7.70
7.30
7.29
7.28
7.27
7.26
7.26
7.25
7.18
7.16
7.16
7.14
7.11
3.62
1.96
1.37
1.00
1.16
1.10
2.04
3.02
6.17
9.61
δ (ppm)

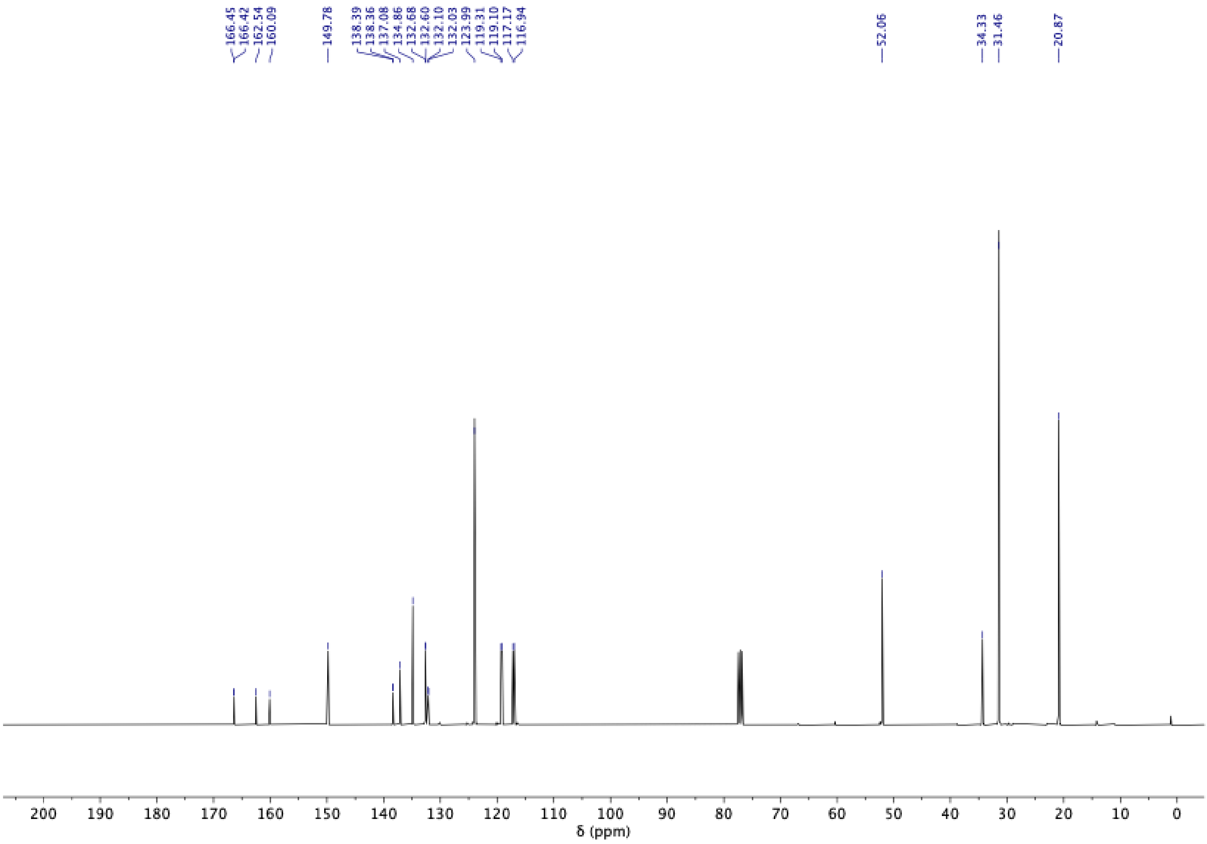
166.45
166.42
162.54
160.09
149.78
138.39
138.36
137.08
134.86
132.68
132.60
132.10
132.03
123.99
119.31
119.10
117.17
116.94
52.06
34.33
31.46
20.87
δ (ppm)

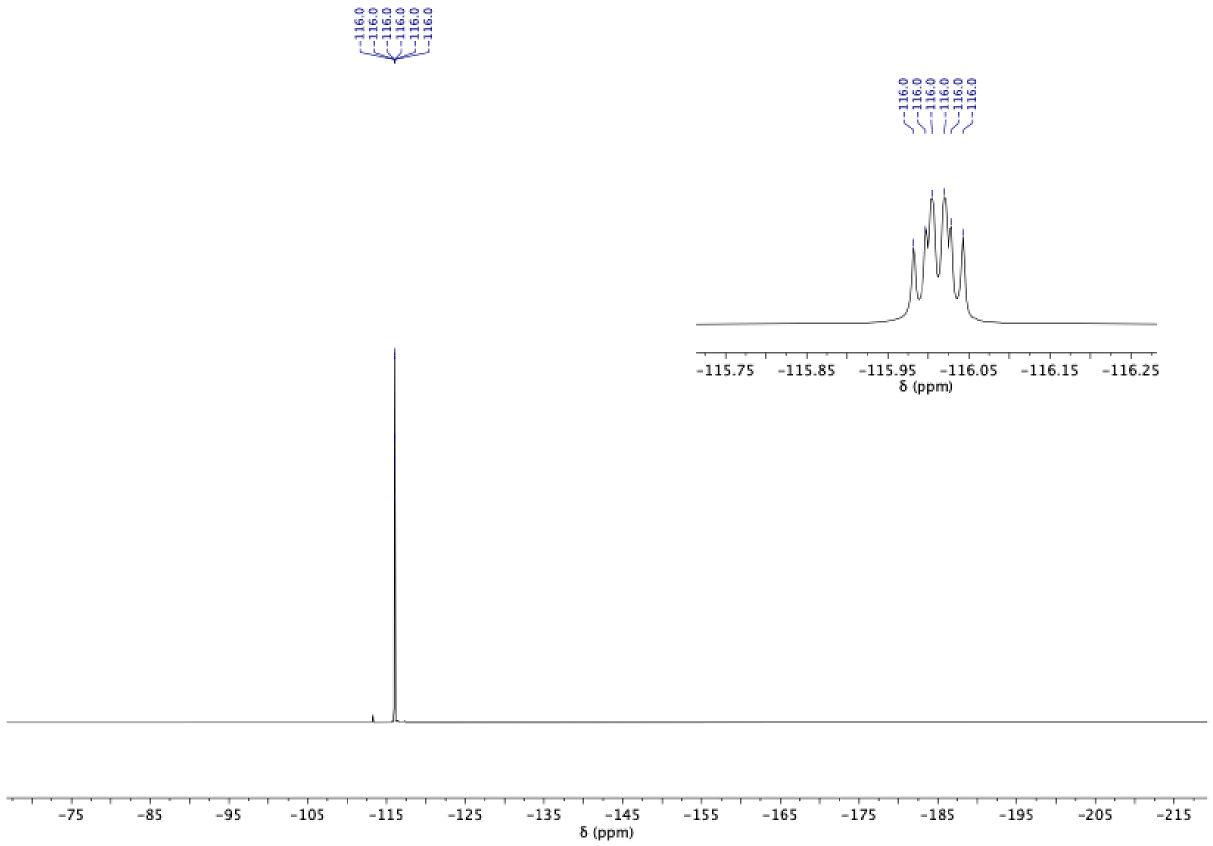
-116.0
-116.0
-116.0
-116.0
-116.0
-116.0
-115.75 -115.85 -115.95 -116.05 -116.15 -116.25
δ (ppm)
-75 -85 -95 -105 -115 -125 -135 -145 -155 -165 -175 -185 -195 -205 -215
δ (ppm)

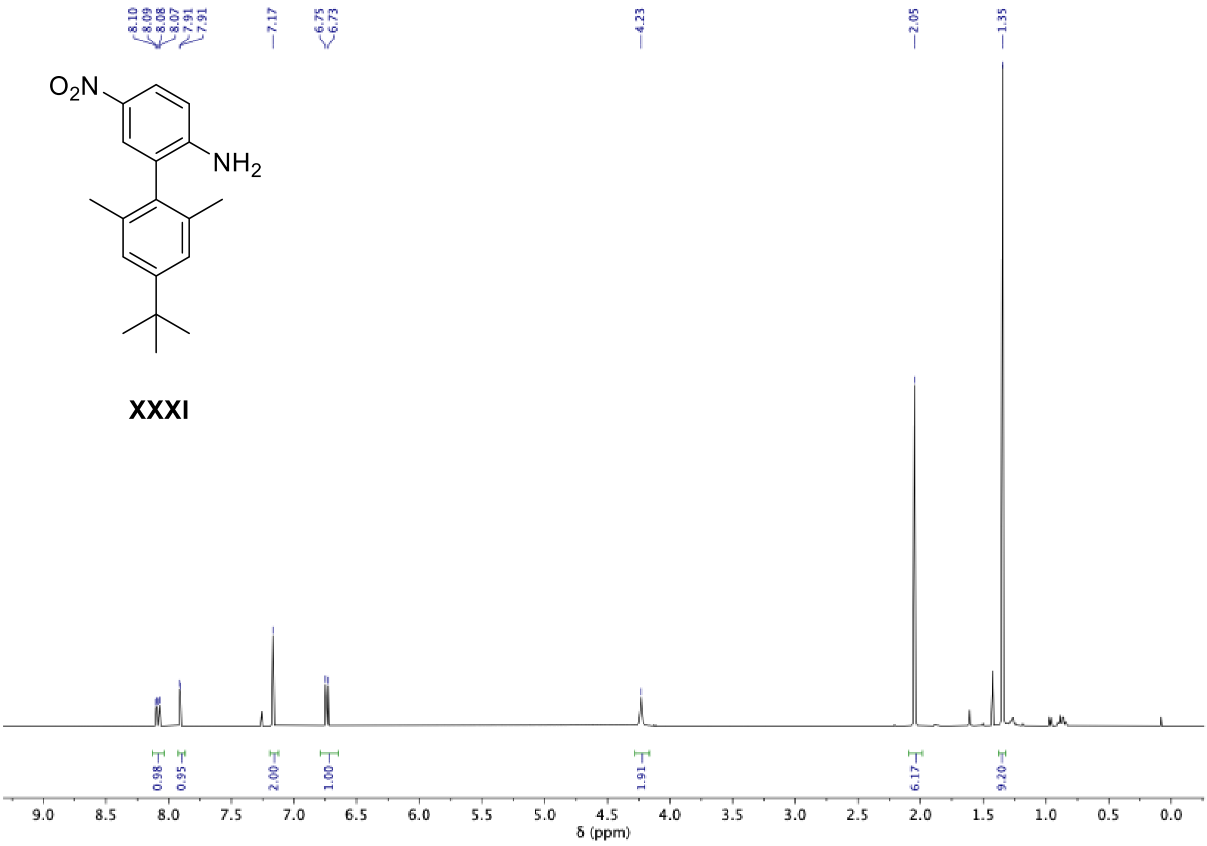
O_2N
NH_2
8.10
8.09
8.08
8.07
7.91
7.91
7.17
6.75
6.73
4.23
2.05
1.35
0.98
0.95
2.00
1.00
1.91
6.17
9.20
δ (ppm)

XXXI

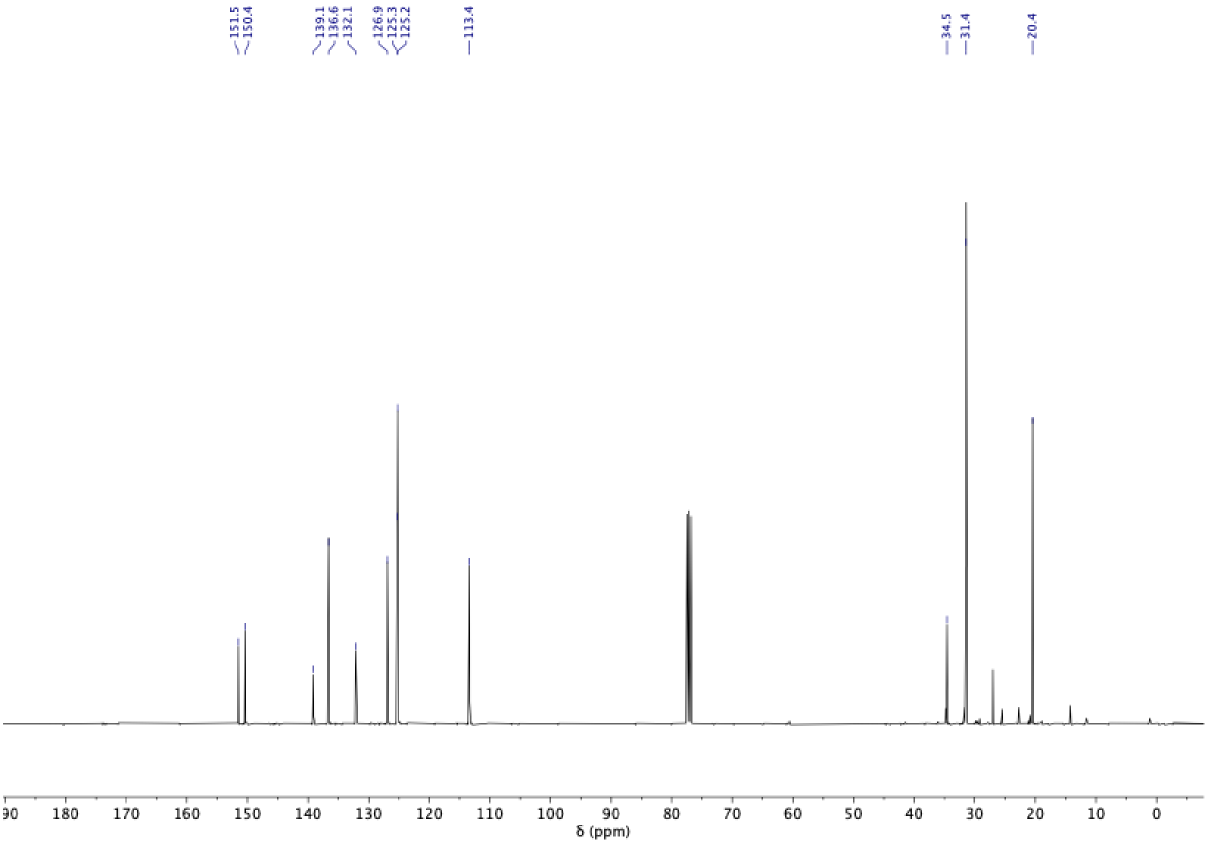
151.5
150.4
139.1
136.6
132.1
126.9
125.3
125.2
113.4
34.5
31.4
20.4
δ (ppm)

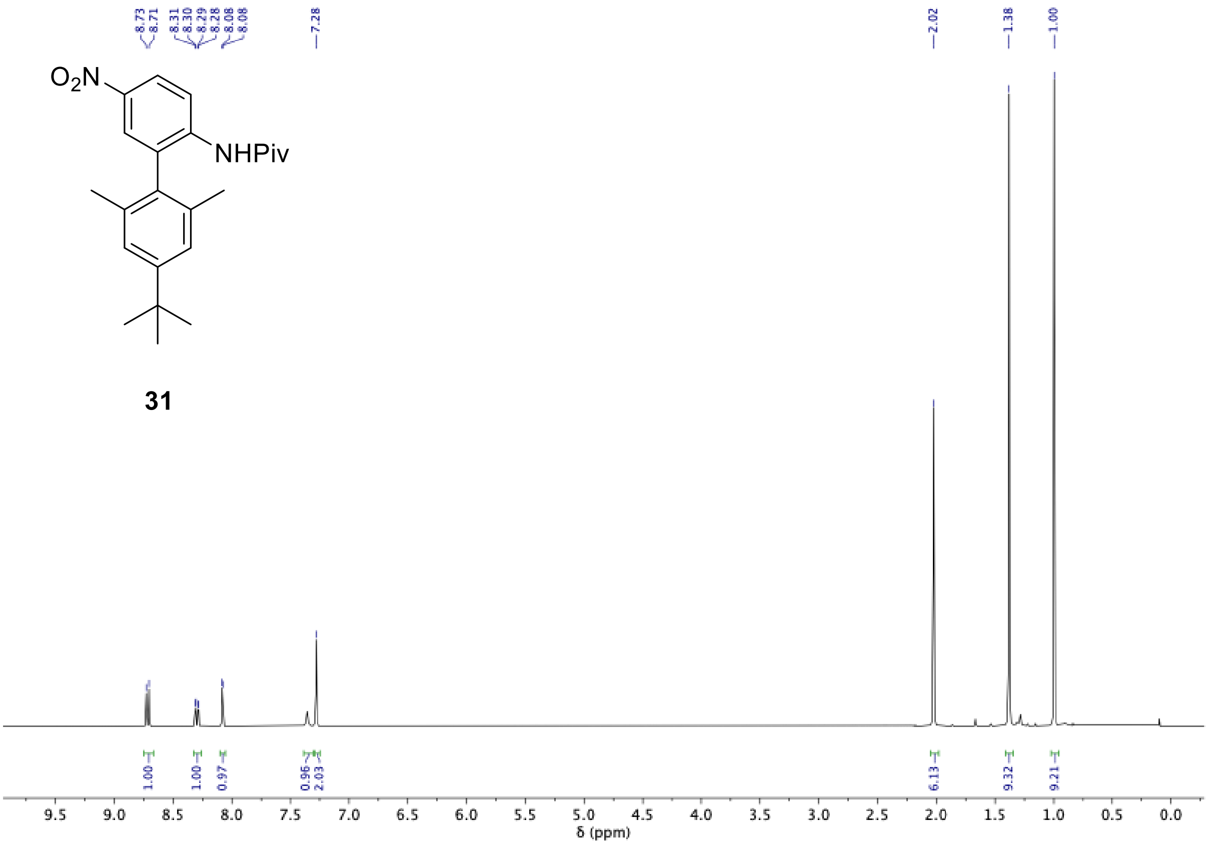

31

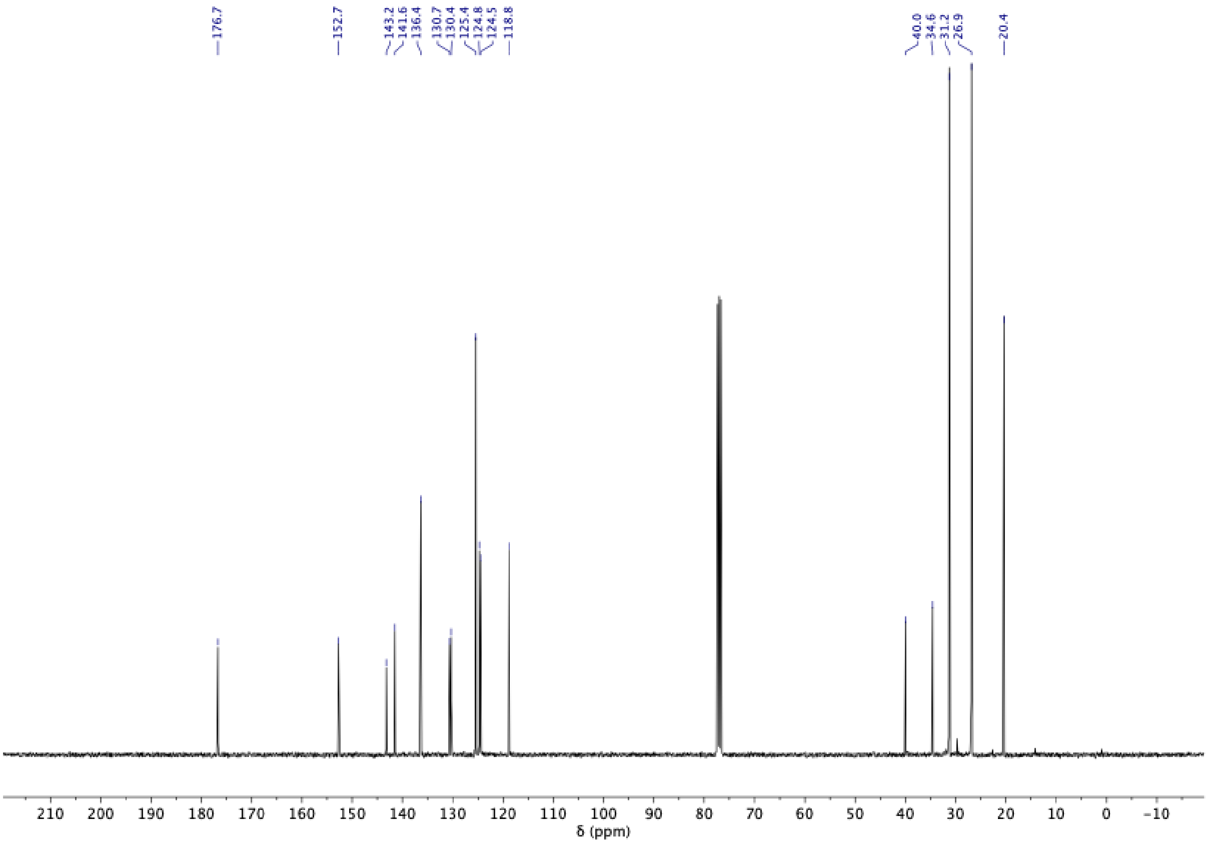

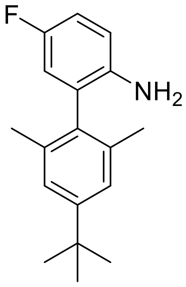
F
NH2

XXXII

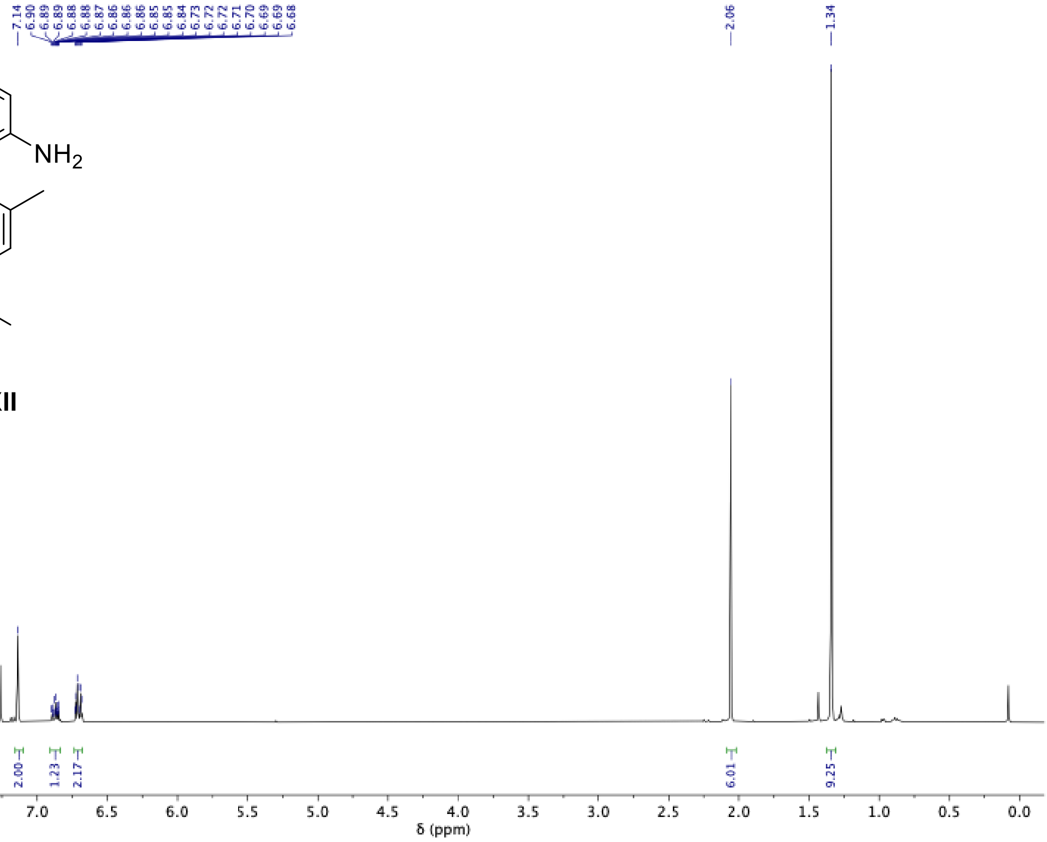
7.14
6.90
6.89
6.89
6.88
6.88
6.87
6.86
6.86
6.86
6.85
6.85
6.84
6.73
6.72
6.72
6.71
6.70
6.69
6.69
6.68
2.06
1.34
2.00
1.23
2.17
6.01
9.25
8.0 7.5 7.0 6.5 6.0 5.5 5.0 4.5 4.0 3.5 3.0 2.5 2.0 1.5 1.0 0.5 0.0
δ (ppm)

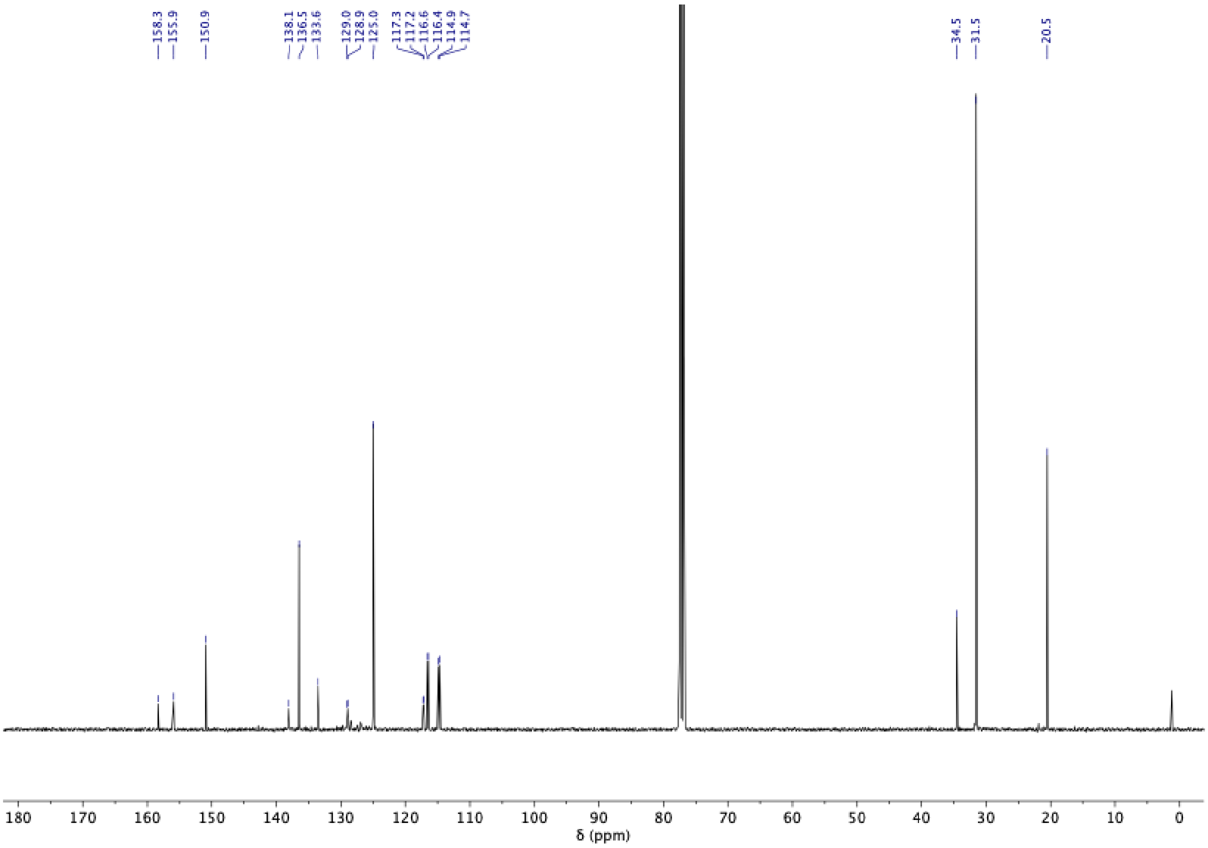
158.3
155.9
150.9
138.1
136.5
133.6
129.0
128.9
125.0
117.3
117.2
116.6
116.4
114.9
114.7
34.5
31.5
20.5
180 170 160 150 140 130 120 110 100 90 80 70 60 50 40 30 20 10 0
δ (ppm)

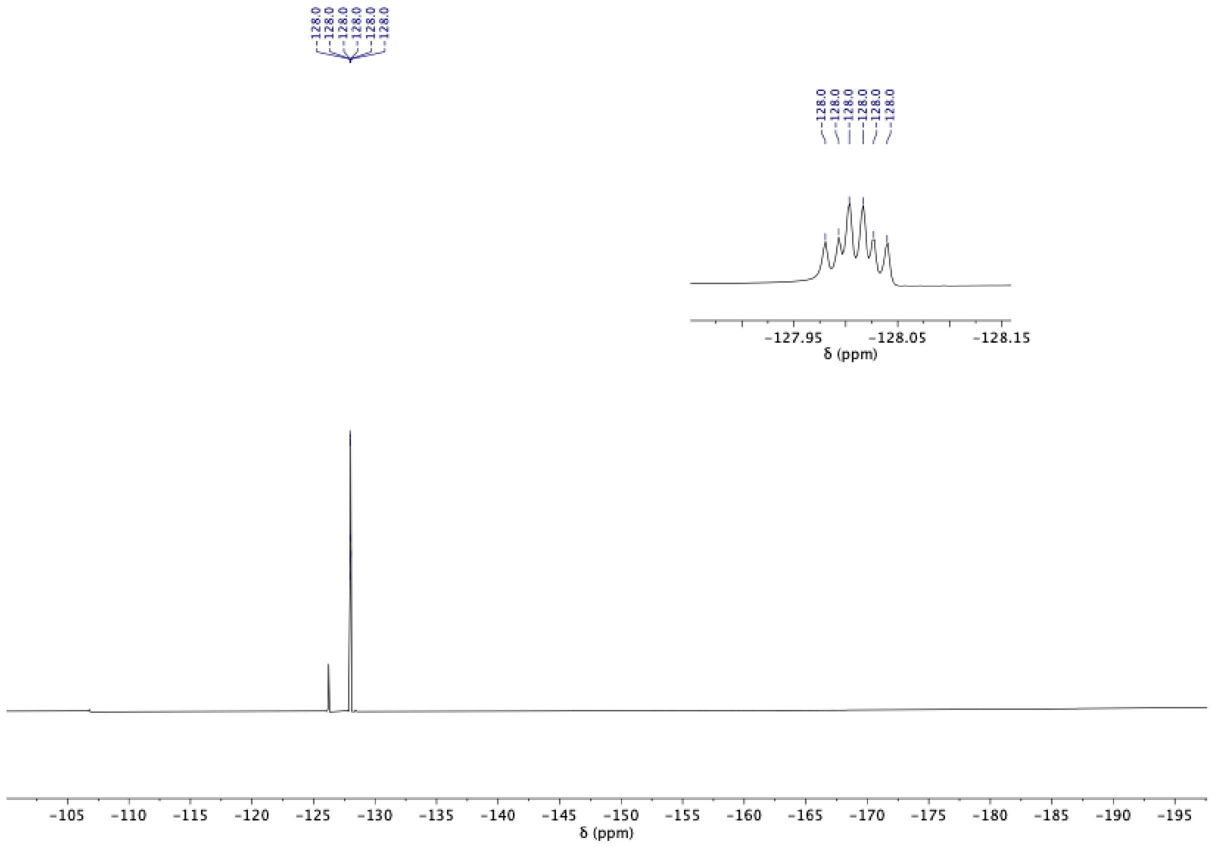
-128.0
-128.0
-128.0
-128.0
-128.0
-128.0
-127.95
-128.05
-128.15
δ (ppm)
-105
-110
-115
-120
-125
-130
-135
-140
-145
-150
-155
-160
-165
-170
-175
-180
-185
-190
-195
δ (ppm)

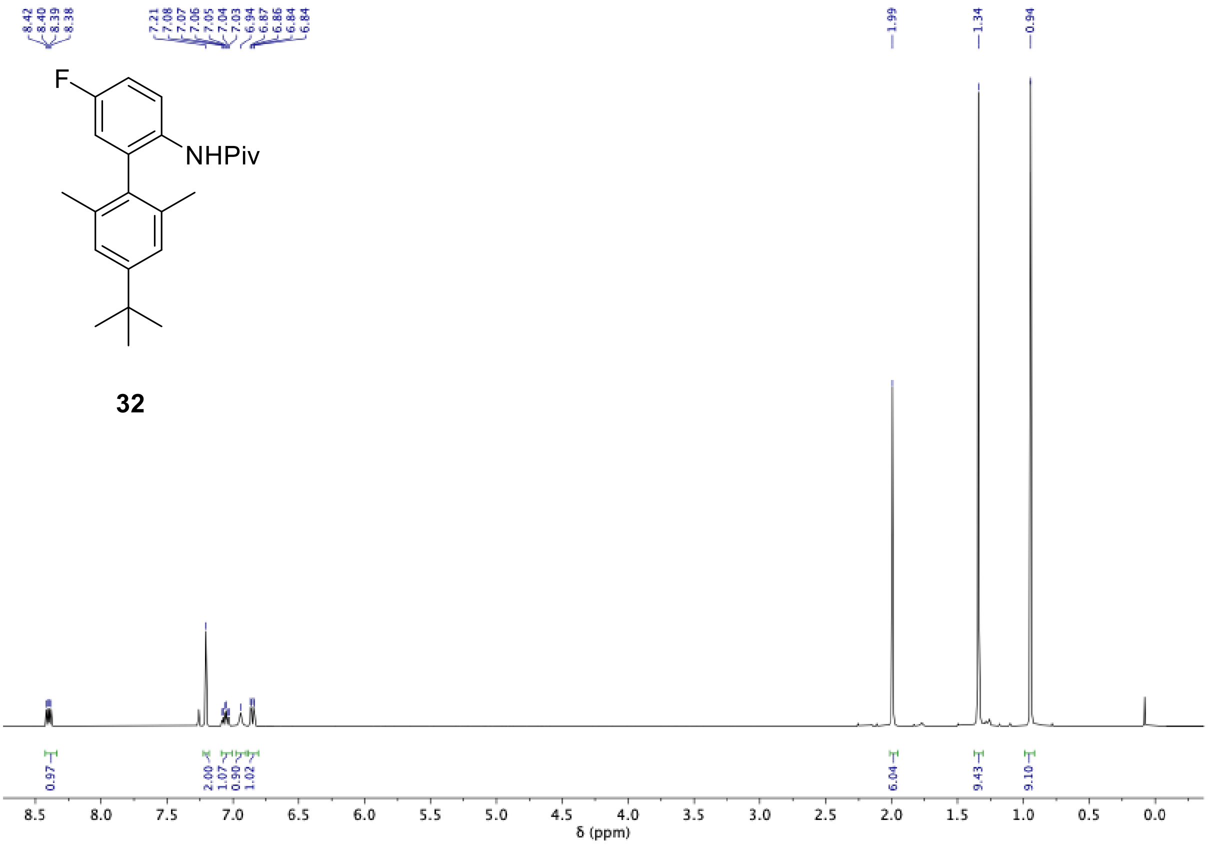
F
NHPiv
8.42
8.40
8.39
8.38
7.21
7.08
7.07
7.06
7.05
7.04
7.03
6.94
6.87
6.86
6.84
6.84
1.99
1.34
0.94
0.97
2.00
1.07
0.90
1.02
6.04
9.43
9.10
8.5
8.0
7.5
7.0
6.5
6.0
5.5
5.0
4.5
4.0
3.5
3.0
2.5
2.0
1.5
1.0
0.5
0.0
δ (ppm)

32

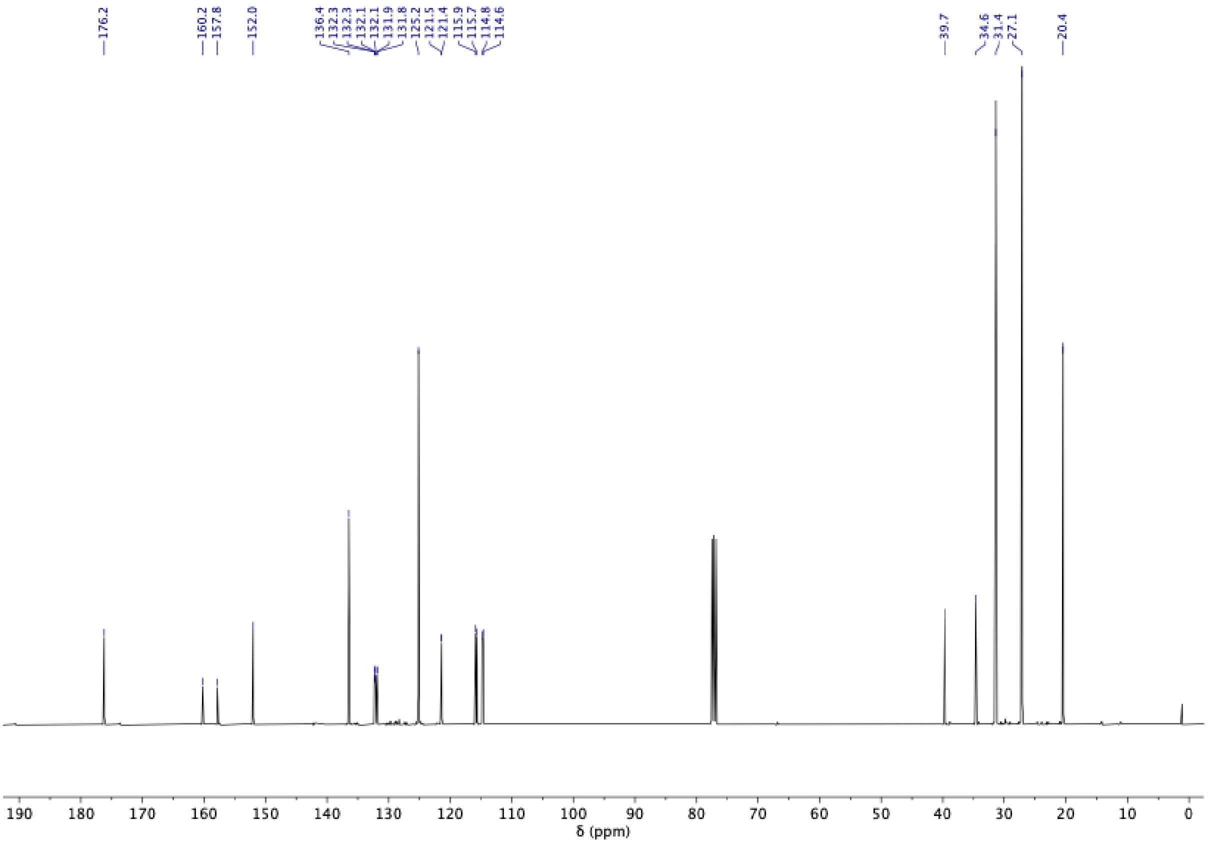
176.2
160.2
157.8
152.0
136.4
132.3
132.3
132.1
132.1
131.9
131.8
125.2
121.5
121.4
115.9
115.7
114.8
114.6
39.7
34.6
31.4
27.1
20.4
190
180
170
160
150
140
130
120
110
100
90
80
70
60
50
40
30
20
10
0
δ (ppm)

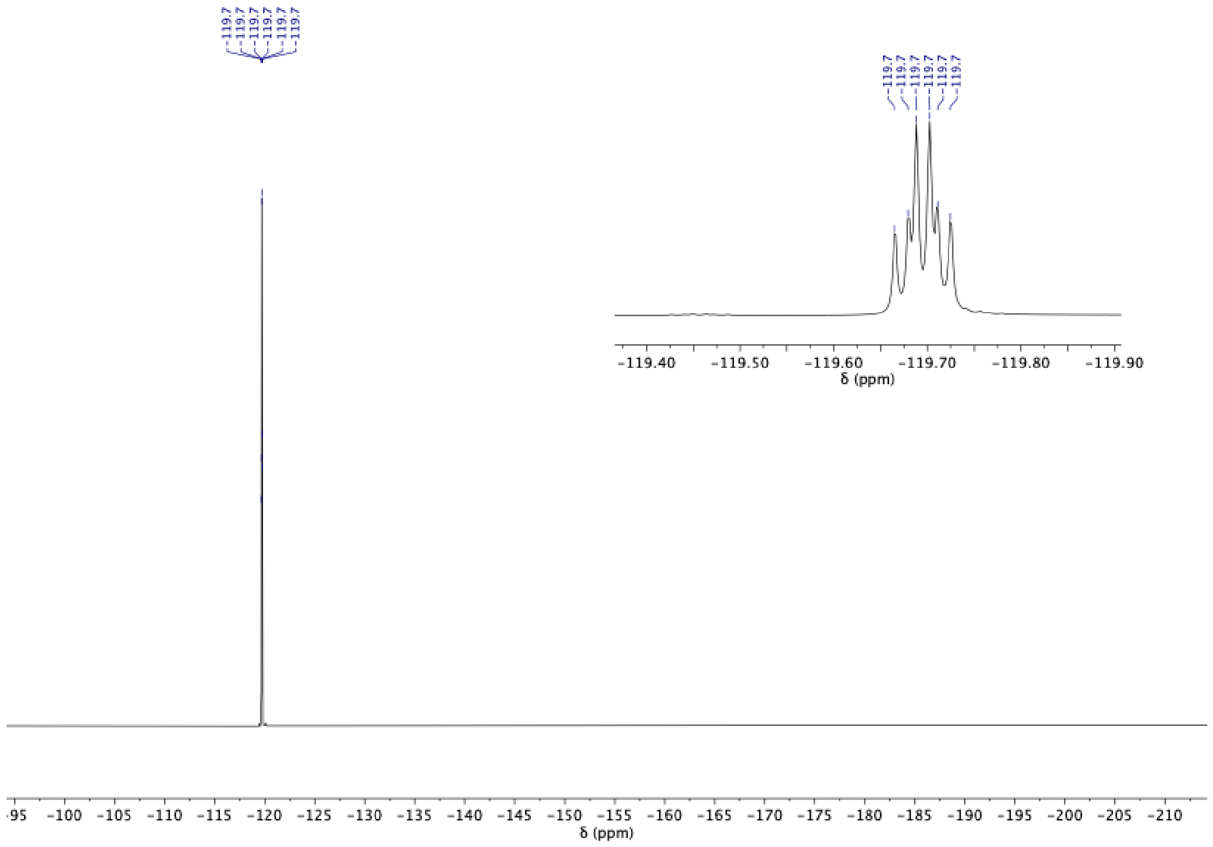

-119.7
-119.7
-119.7
-119.7
-119.7
-119.7
-119.40
-119.50
-119.60
-119.70
-119.80
-119.90
δ (ppm)
-100
-105
-110
-115
-120
-125
-130
-135
-140
-145
-150
-155
-160
-165
-170
-175
-180
-185
-190
-195
-200
-205
-210
δ (ppm)

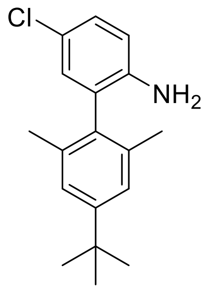
Cl
NH2

XXXIII

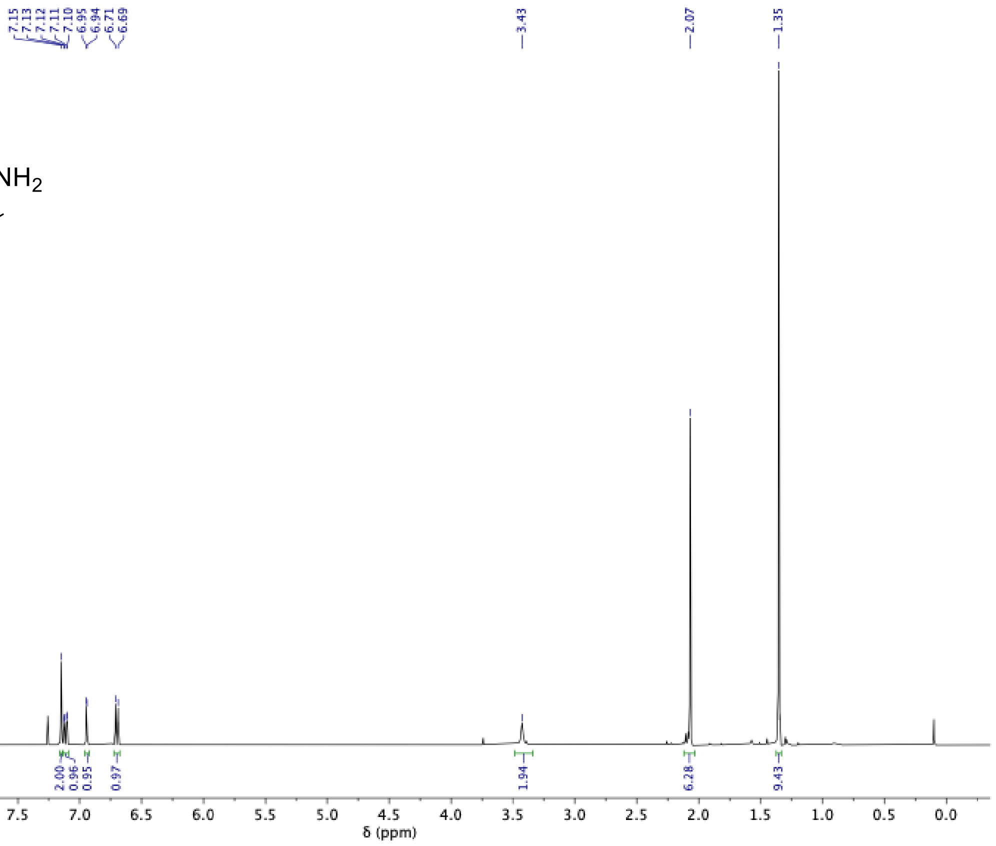
7.15
7.13
7.12
7.11
7.10
6.95
6.94
6.71
6.69
3.43
2.07
1.35
2.00
0.96
0.95
0.97
1.94
6.28
9.43
9.0
8.5
8.0
7.5
7.0
6.5
6.0
5.5
5.0
4.5
4.0
3.5
3.0
2.5
2.0
1.5
1.0
0.5
0.0
δ (ppm)

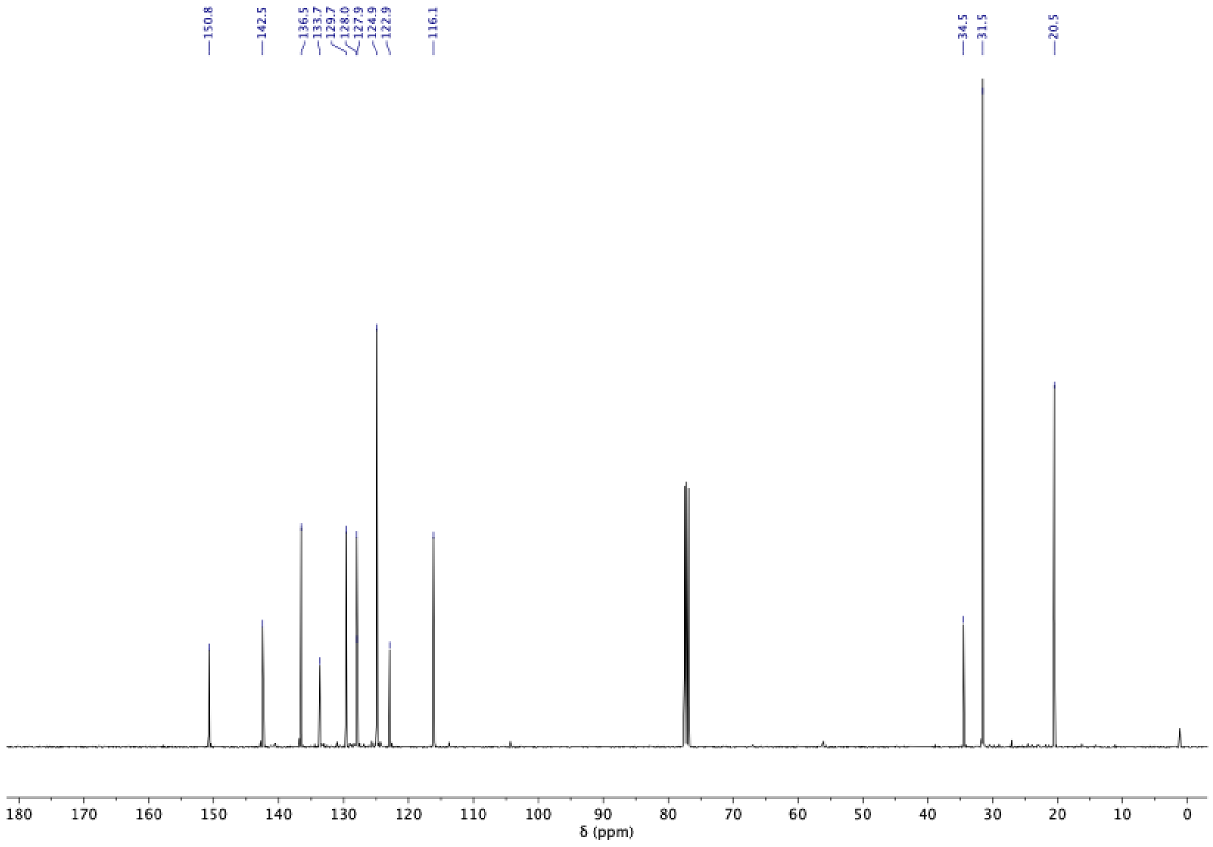
150.8
142.5
136.5
133.7
129.7
128.0
127.9
124.9
122.9
116.1
34.5
31.5
20.5
180
170
160
150
140
130
120
110
100
90
80
70
60
50
40
30
20
10
0
δ (ppm)

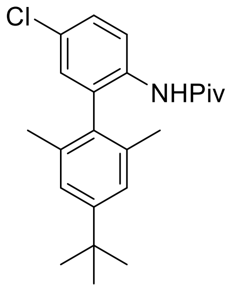
Cl
NHPiv

33

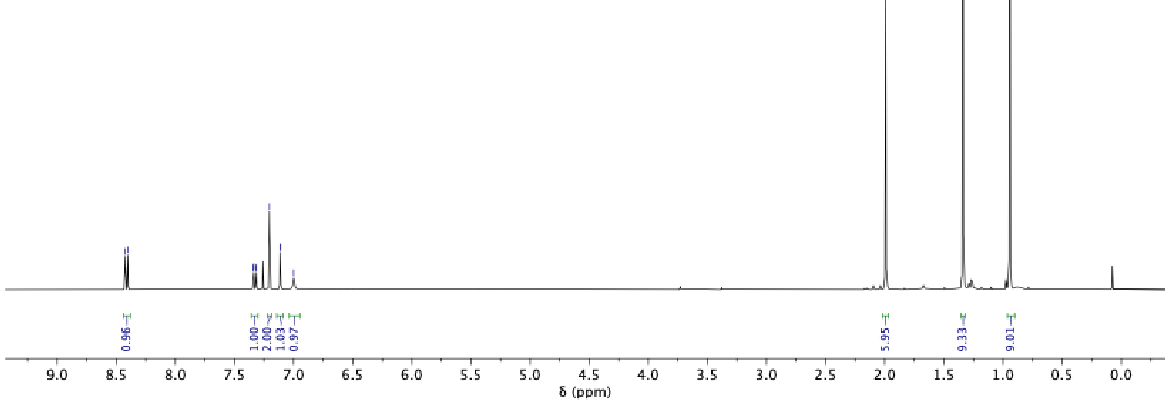
8.42
8.40
7.34
7.34
7.32
7.31
7.20
7.12
7.11
7.00
1.99
1.34
0.94
0.96
1.00
2.00
1.03
0.97
5.95
9.33
9.01
9.0 8.5 8.0 7.5 7.0 6.5 6.0 5.5 5.0 4.5 4.0 3.5 3.0 2.5 2.0 1.5 1.0 0.5 0.0
δ (ppm)

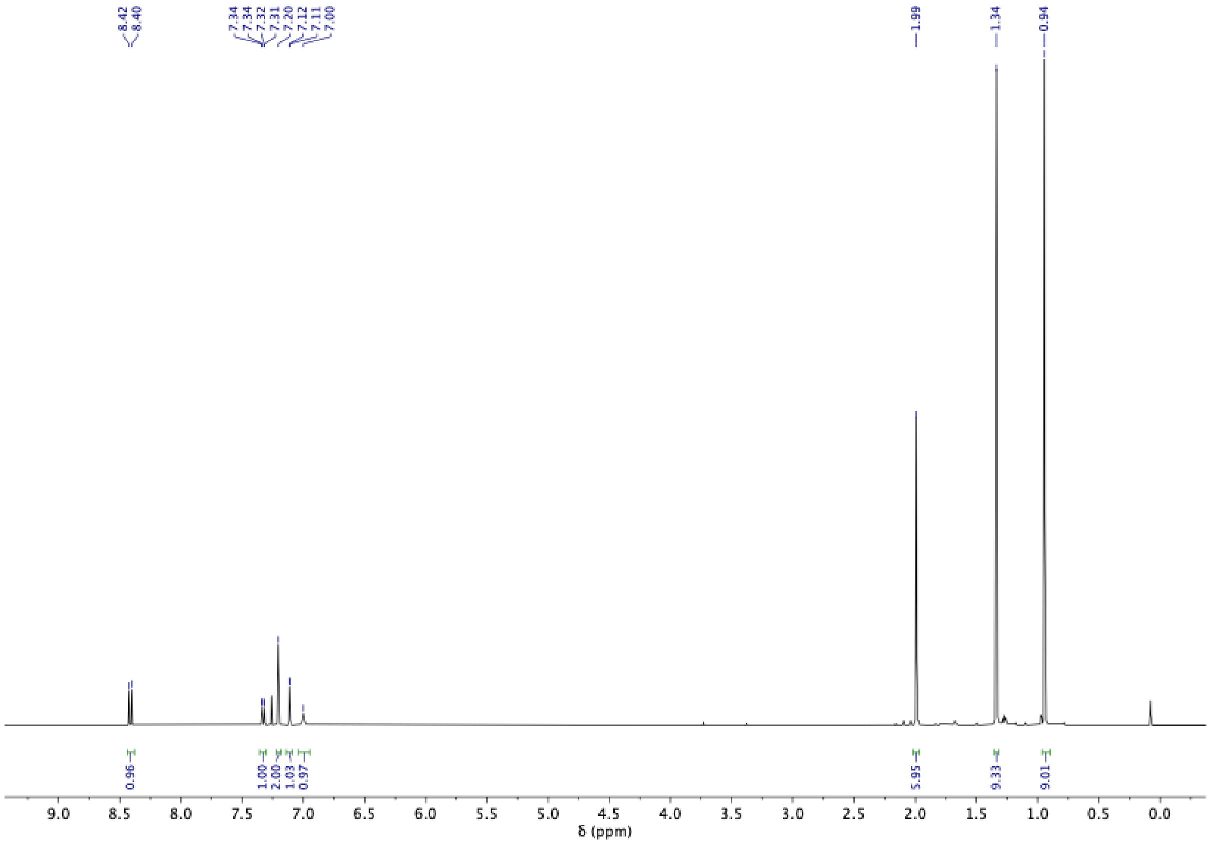
8.42
8.40
7.34
7.34
7.32
7.31
7.20
7.12
7.11
7.00
1.99
1.34
0.94
0.96
1.00
2.00
1.03
0.97
5.95
9.33
9.01
9.0 8.5 8.0 7.5 7.0 6.5 6.0 5.5 5.0 4.5 4.0 3.5 3.0 2.5 2.0 1.5 1.0 0.5 0.0
δ (ppm)

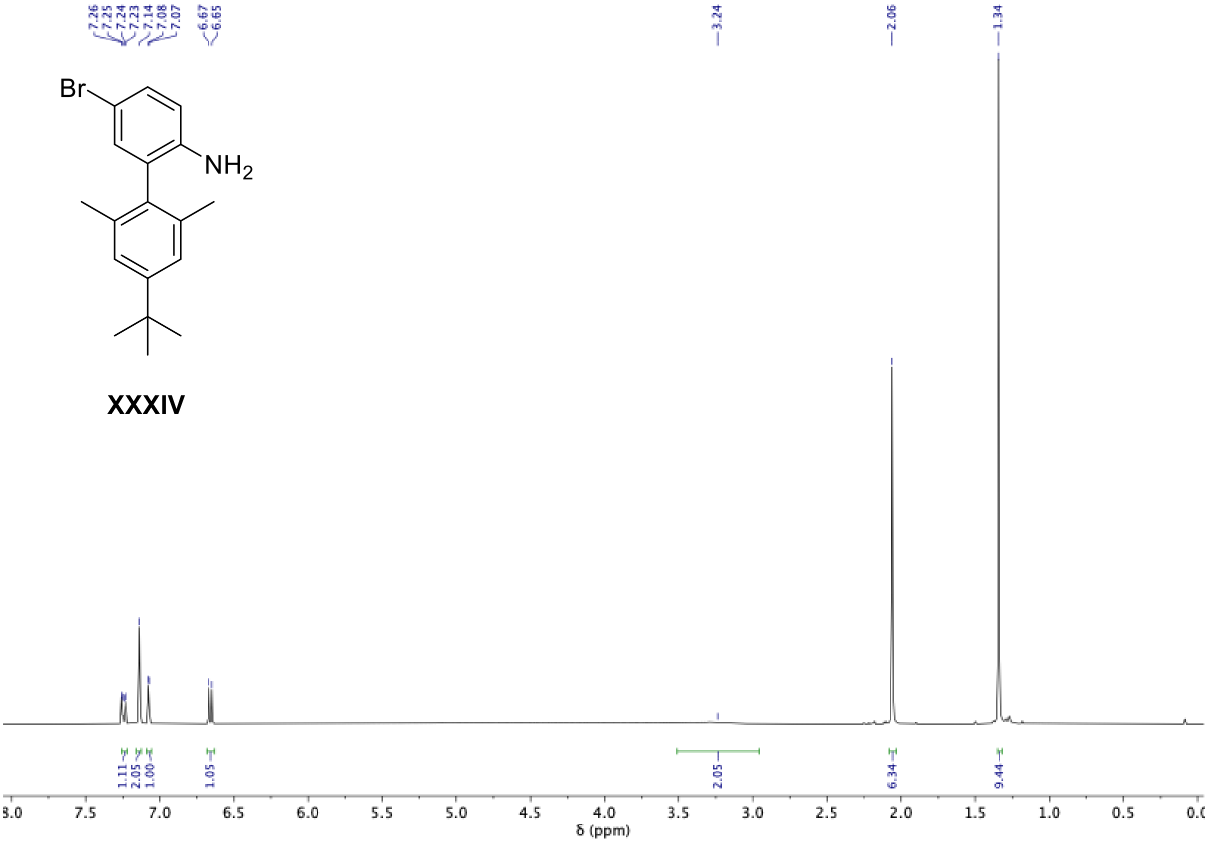
Br
NH2
XXXIV
7.26
7.25
7.24
7.23
7.14
7.08
7.07
6.67
6.65
3.24
2.06
1.34
1.11
2.05
1.00
1.05
2.05
6.34
9.44
δ (ppm)

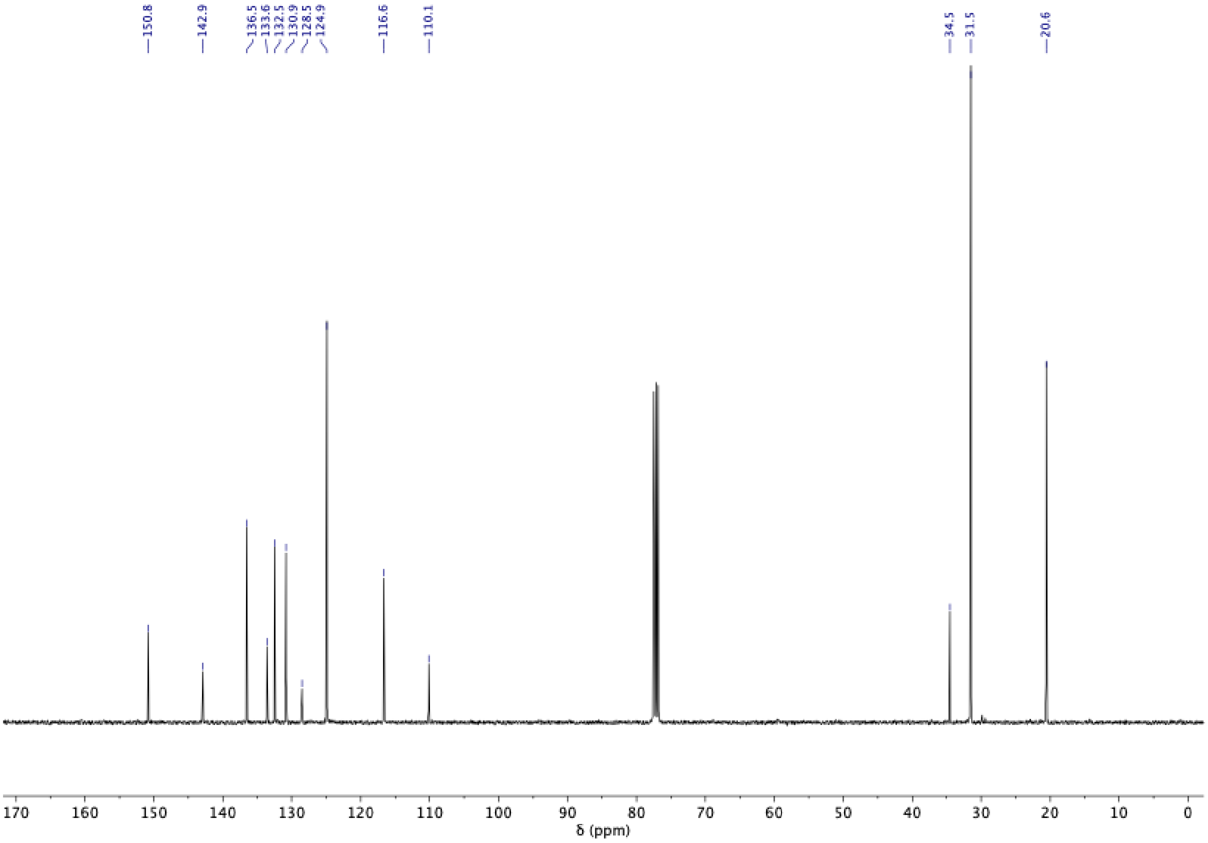
150.8
142.9
136.5
133.6
132.5
130.9
128.5
124.9
116.6
110.1
34.5
31.5
20.6
δ (ppm)

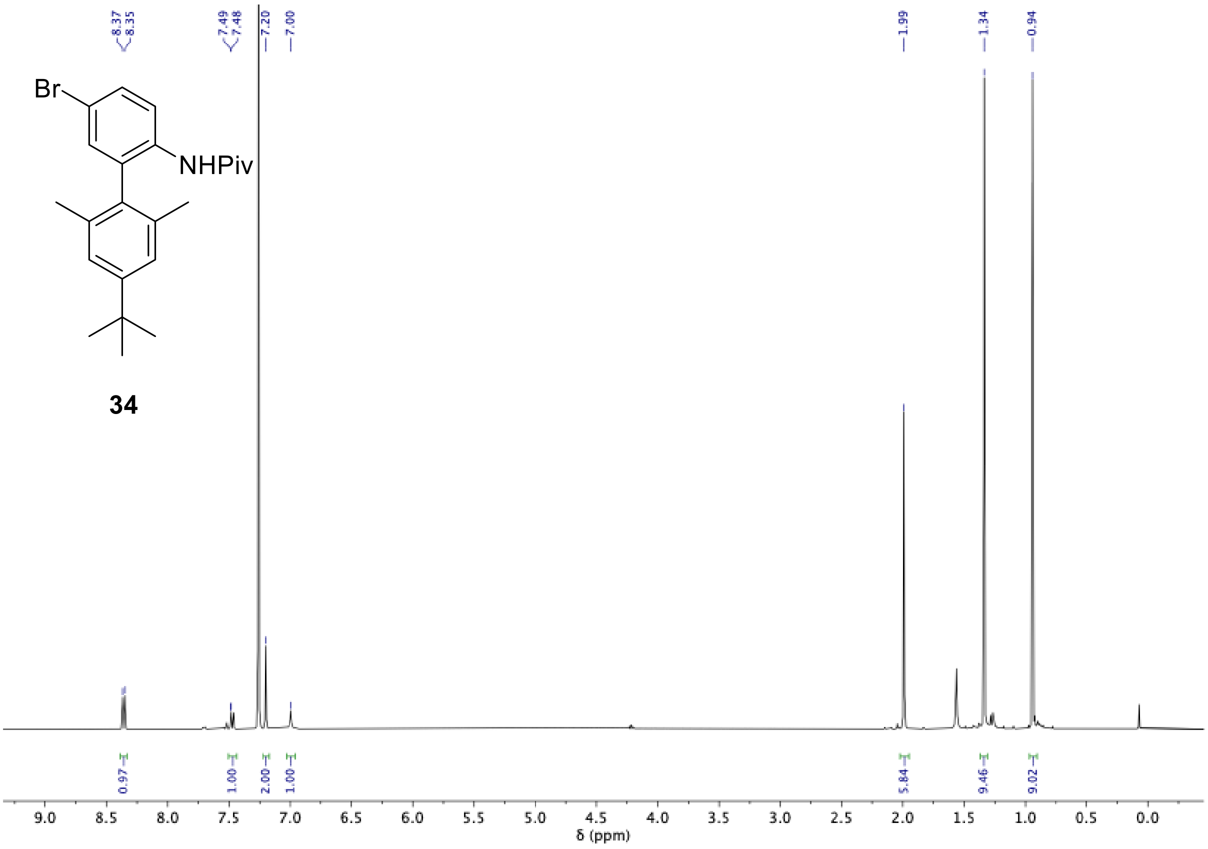
Br
NHPiv
8.37
8.35
7.49
7.48
7.20
7.00
1.99
1.34
0.94
0.97
1.00
2.00
1.00
5.84
9.46
9.02
δ (ppm)

34

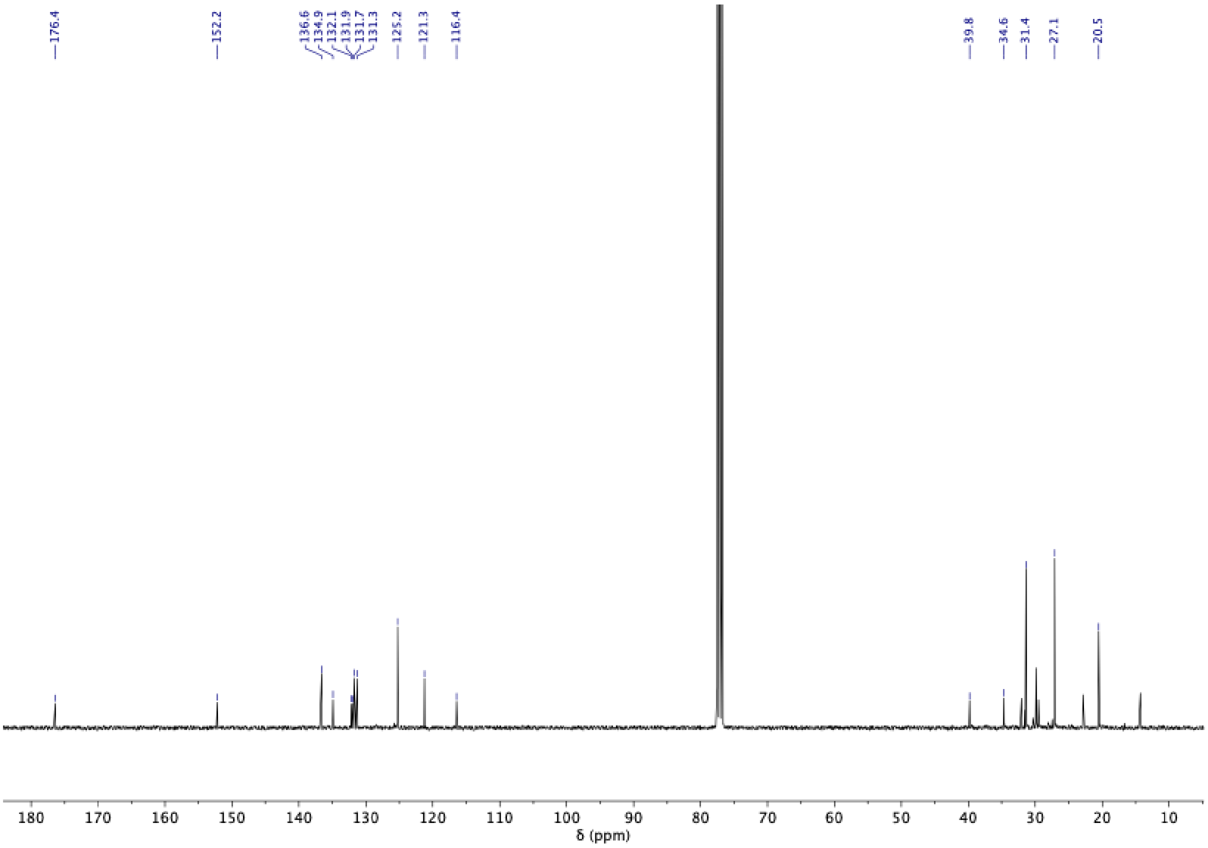
176.4
152.2
136.6
134.9
132.1
131.9
131.7
131.3
125.2
121.3
116.4
39.8
34.6
31.4
27.1
20.5
δ (ppm)

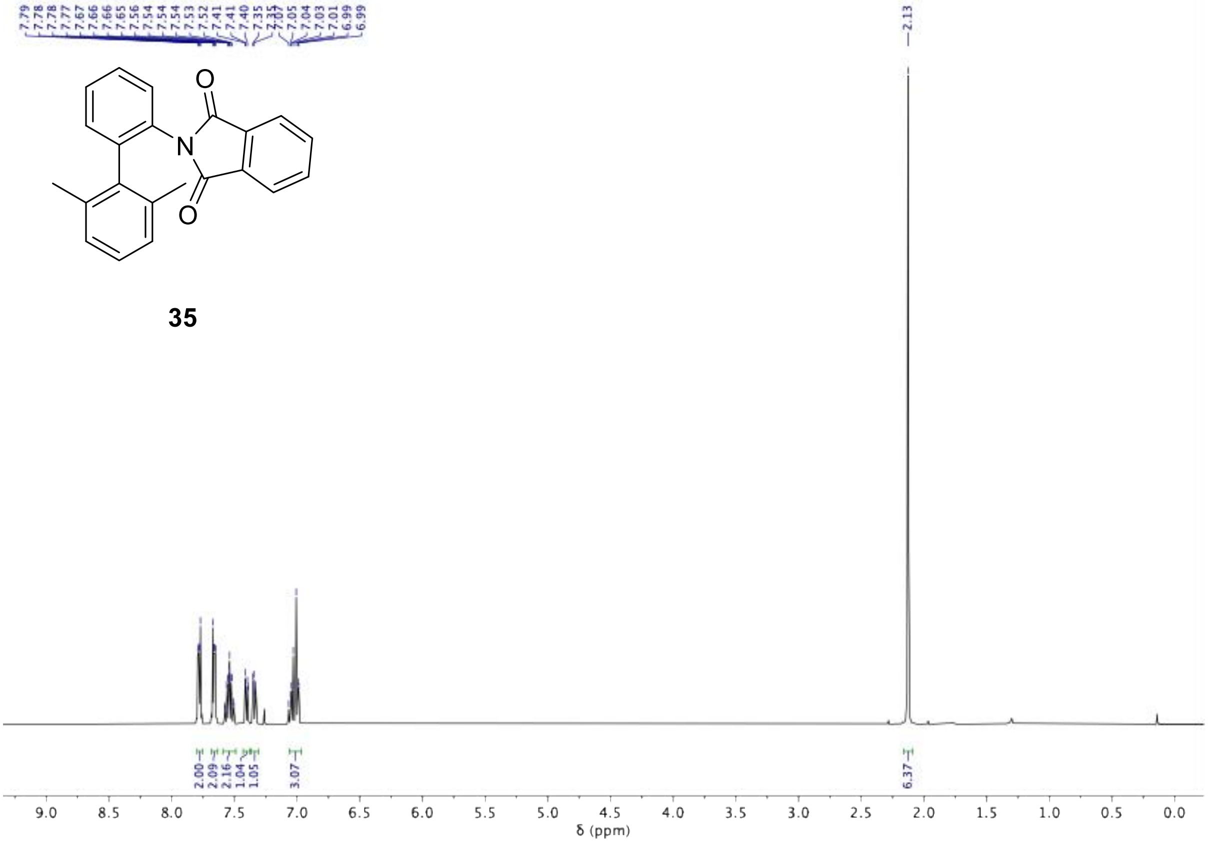
7.79 7.78 7.78 7.77 7.67 7.66 7.66 7.65 7.56 7.54 7.54 7.54 7.53 7.52 7.41 7.40 7.35 7.35 7.07 7.05 7.04 7.03 7.01 6.99 6.99
2.13
O
N
O
2.00 2.09 2.16 1.04 1.05 3.07 6.37
9.0 8.5 8.0 7.5 7.0 6.5 6.0 5.5 5.0 4.5 4.0 3.5 3.0 2.5 2.0 1.5 1.0 0.5 0.0
δ (ppm)

35

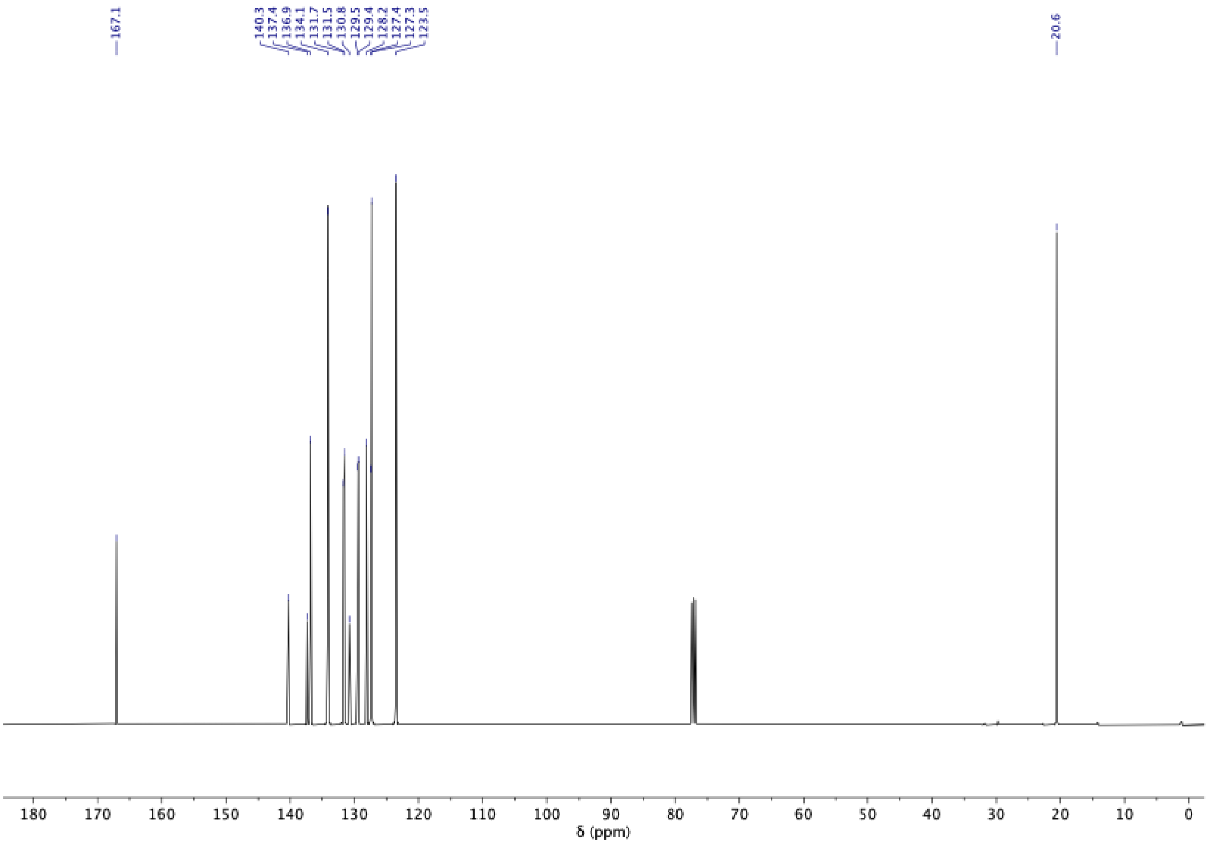
167.1
140.3 137.4 136.9 134.1 131.7 131.5 130.8 129.5 129.4 128.2 127.4 127.3 123.5
20.6
180 170 160 150 140 130 120 110 100 90 80 70 60 50 40 30 20 10 0
δ (ppm)

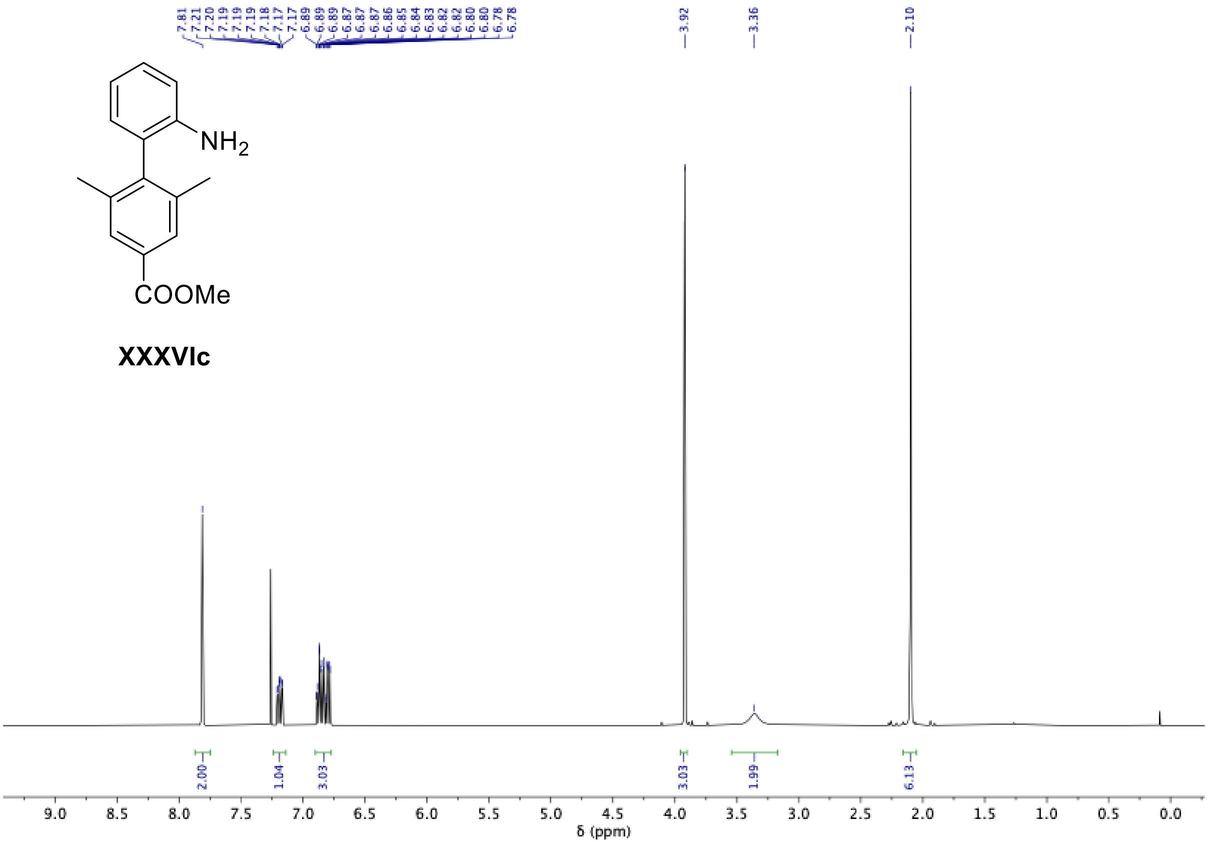

XXXVIc

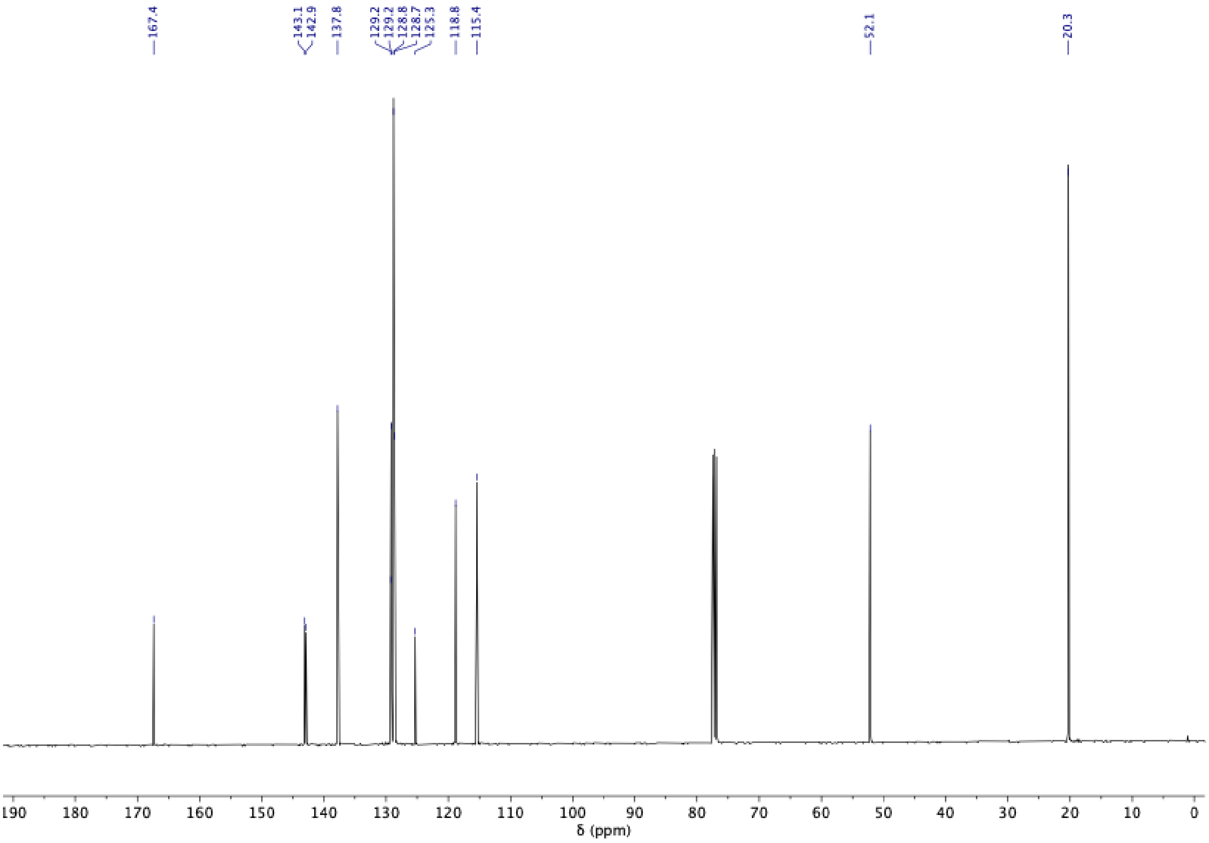

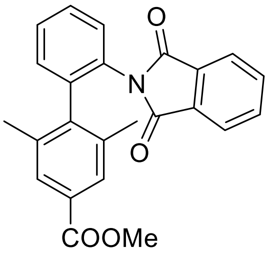
O
N
O
COOMe

36

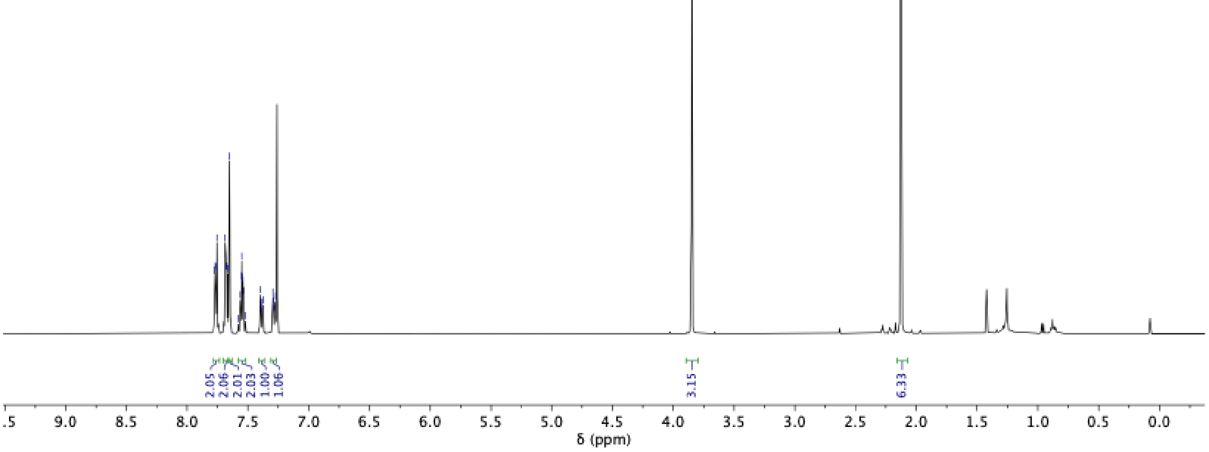
7.78 7.77 7.76 7.75 7.69 7.68 7.67 7.67 7.65 7.58 7.56 7.56 7.55 7.54 7.54 7.53 7.52 7.40 7.40 7.39 7.38 7.38 7.29 7.29 7.29 7.28 7.28 7.27
3.85
2.13
2.05 2.06 2.01 2.03 1.00 1.06
3.15
6.33
δ (ppm)

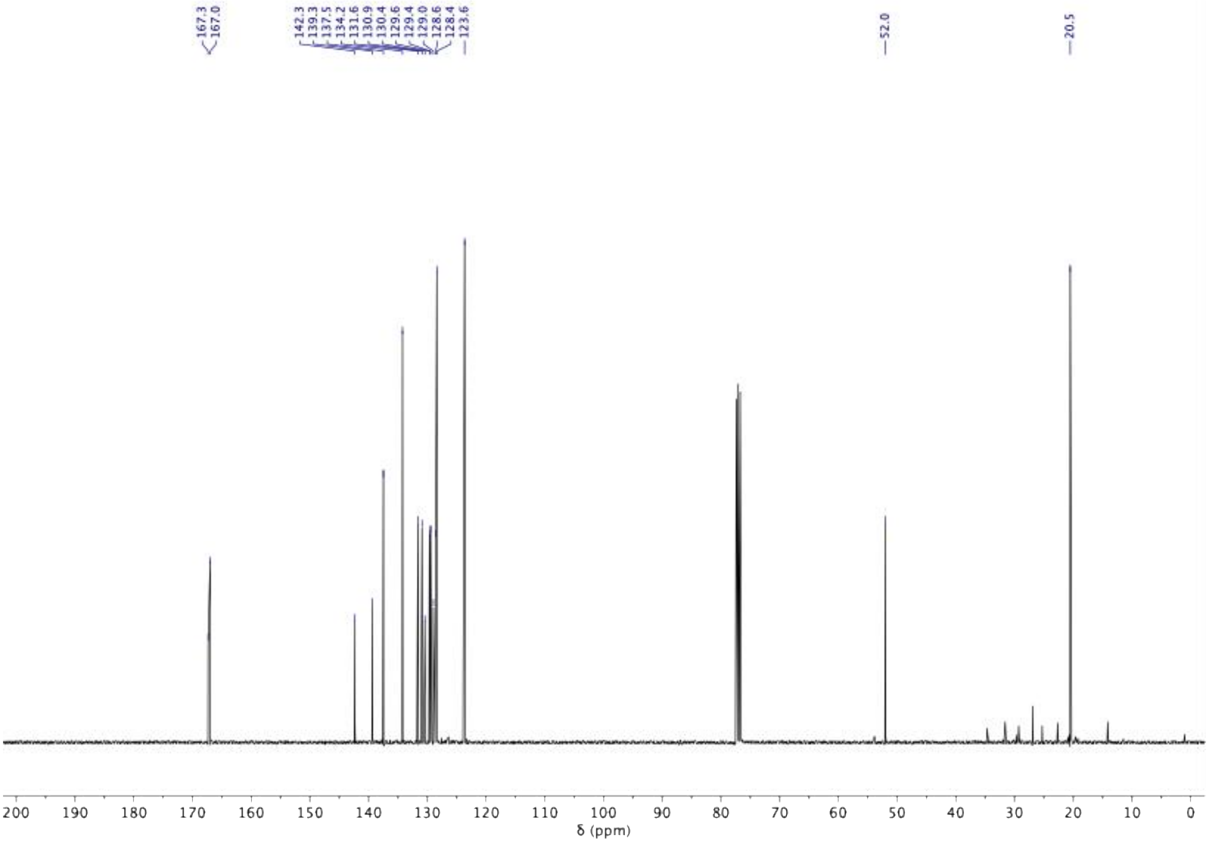
167.3 167.0
142.3 139.3 137.5 134.2 131.6 130.9 130.4 129.6 129.4 129.0 128.6 128.4 123.6
52.0
20.5
δ (ppm)

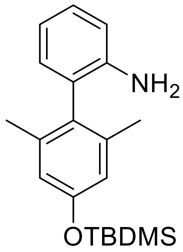
NH2
OTBDMS

XXXVIIb

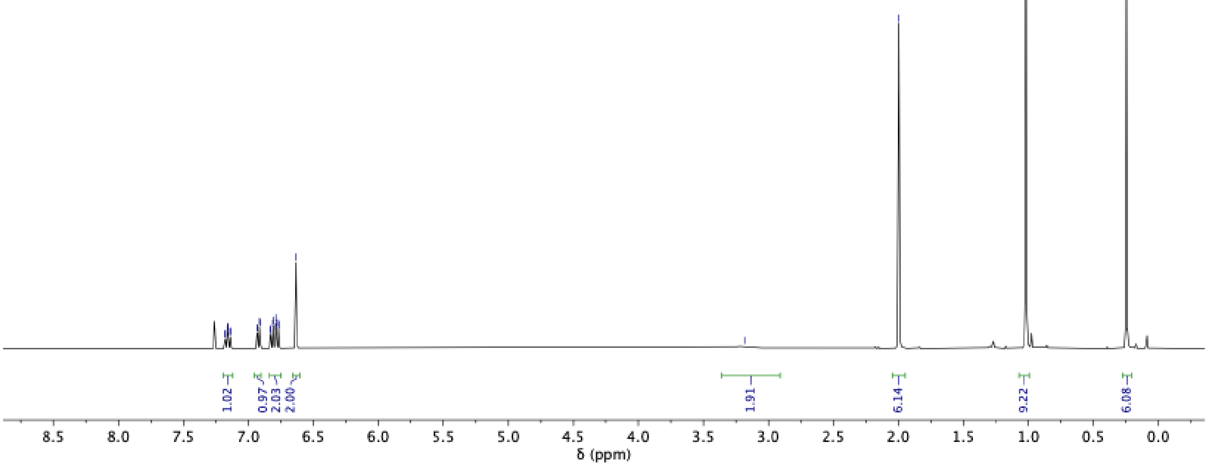
7.18
7.18
7.16
7.16
7.16
7.16
7.14
7.14
6.93
6.93
6.92
6.91
6.83
6.83
6.81
6.81
6.79
6.79
6.78
6.77
6.76
6.63
3.18
2.00
1.02
0.25
1.02
0.97
2.03
2.00
1.91
6.14
9.22
6.08
δ (ppm)

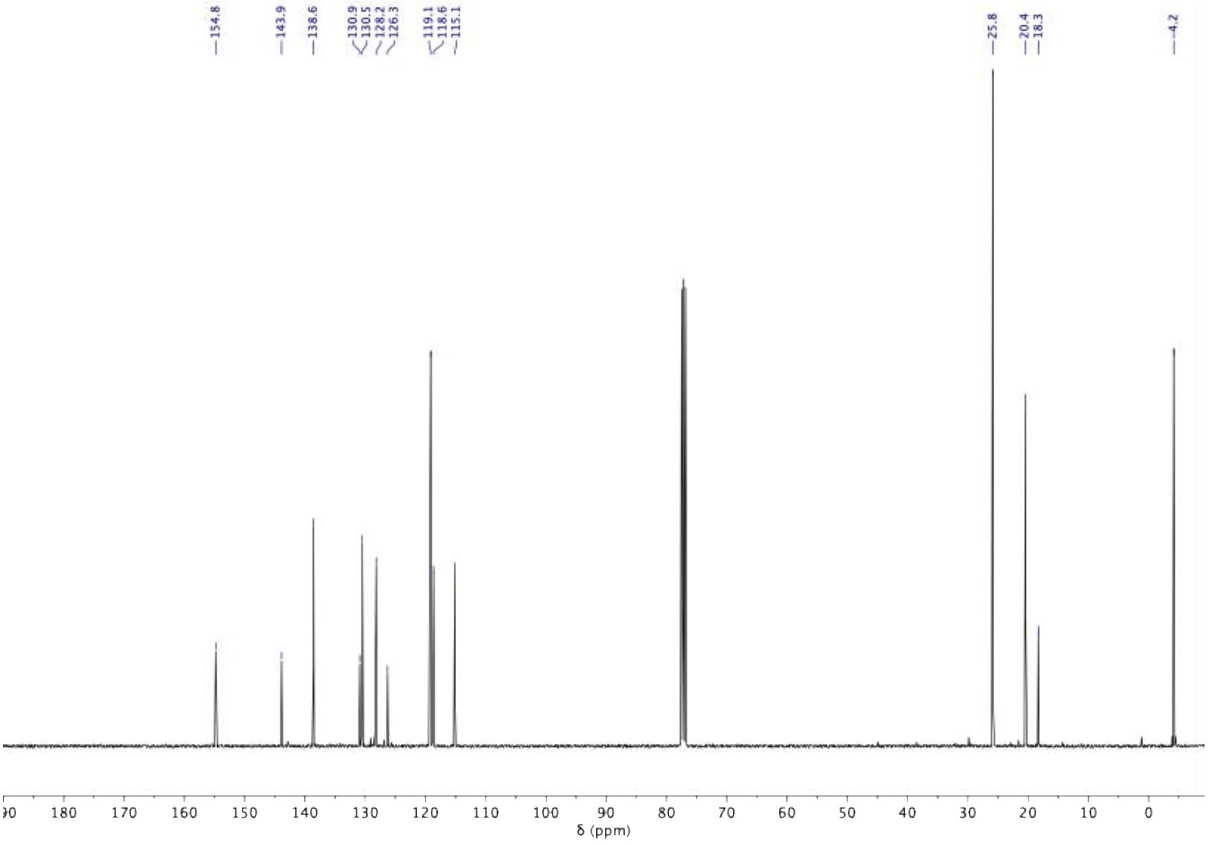
154.8
143.9
138.6
130.9
130.5
128.2
126.3
119.1
118.6
115.1
25.8
20.4
18.3
-4.2
δ (ppm)

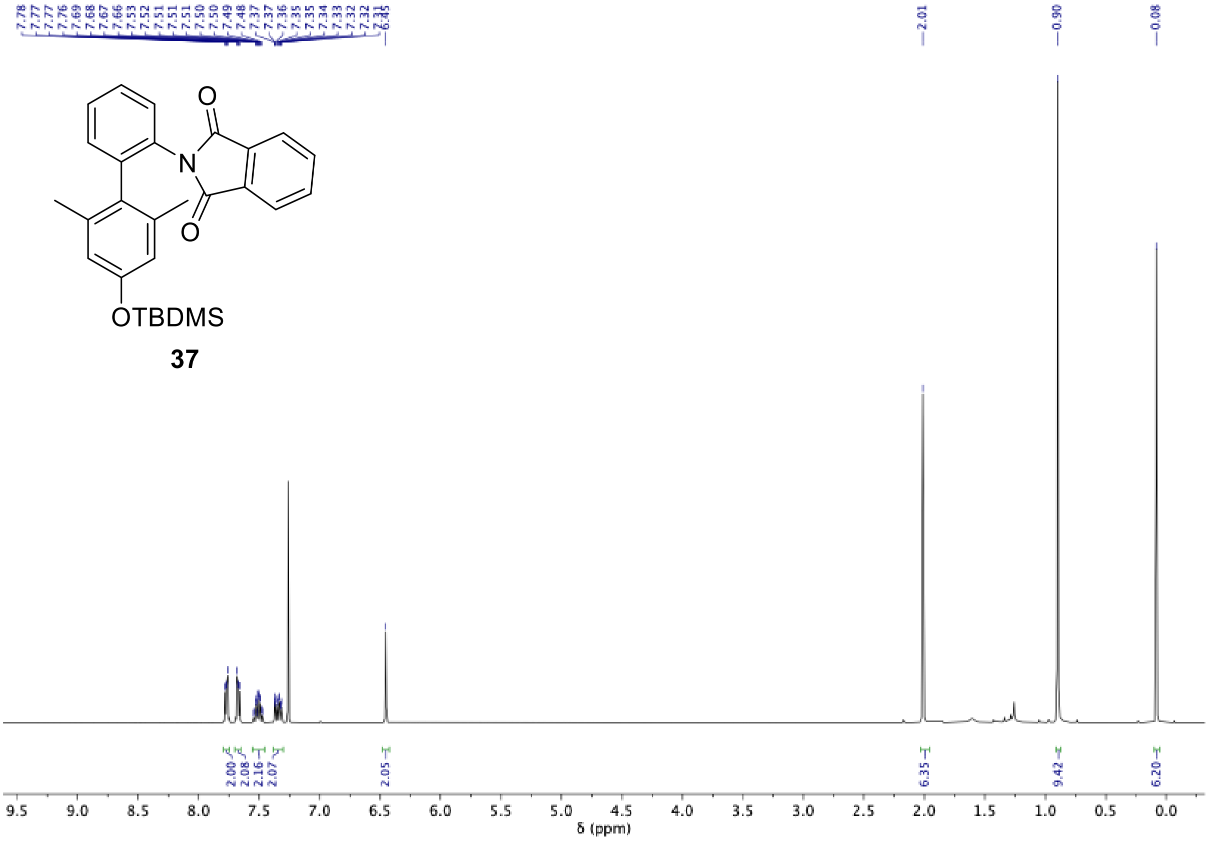
O
N
O
OTBDMS
37
7.78
7.77
7.77
7.76
7.69
7.68
7.67
7.66
7.53
7.52
7.51
7.51
7.51
7.50
7.49
7.48
7.37
7.37
7.36
7.35
7.35
7.34
7.33
7.32
7.32
7.31
6.45
2.01
0.90
0.08
2.00
2.08
2.16
2.07
2.05
6.35
9.42
6.20
9.5 9.0 8.5 8.0 7.5 7.0 6.5 6.0 5.5 5.0 4.5 4.0 3.5 3.0 2.5 2.0 1.5 1.0 0.5 0.0
δ (ppm)

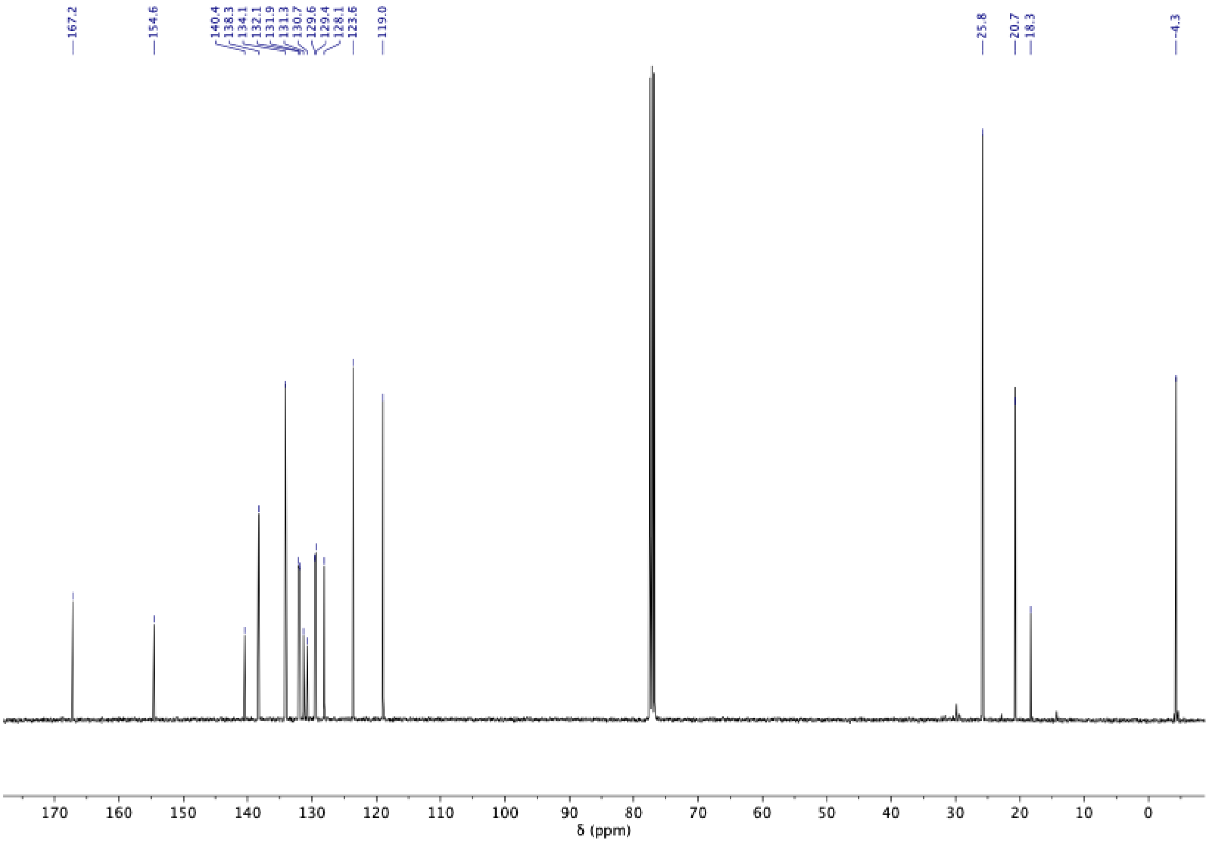
167.2
154.6
140.4
138.3
134.1
132.1
131.9
131.3
130.7
129.6
129.4
128.1
123.6
119.0
25.8
20.7
18.3
-4.3
170 160 150 140 130 120 110 100 90 80 70 60 50 40 30 20 10 0
δ (ppm)

XXXVIII

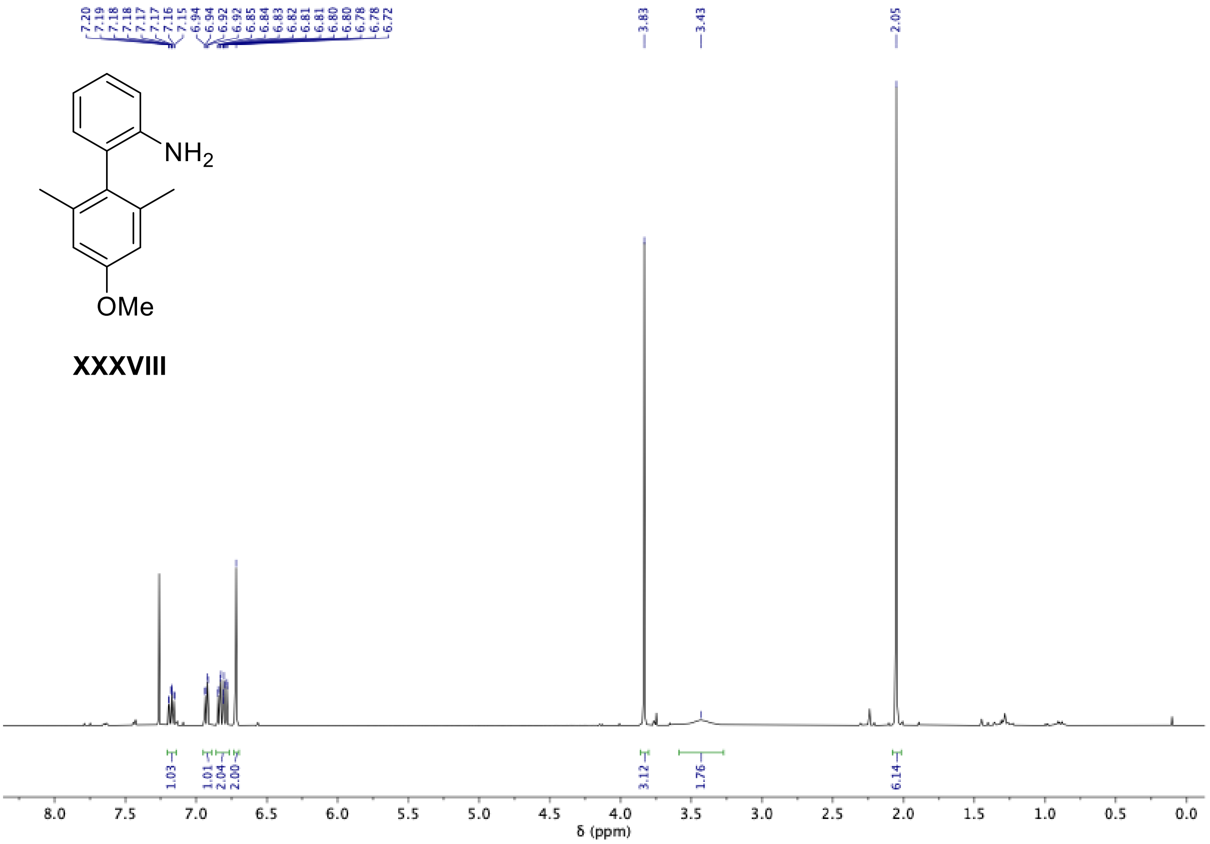
NH2
OMe
7.20
7.19
7.18
7.18
7.17
7.17
7.16
7.15
6.94
6.94
6.92
6.92
6.85
6.84
6.83
6.82
6.81
6.81
6.80
6.80
6.78
6.78
6.72
3.83
3.43
2.05
1.03
1.01
2.04
2.00
3.12
1.76
6.14
δ (ppm)

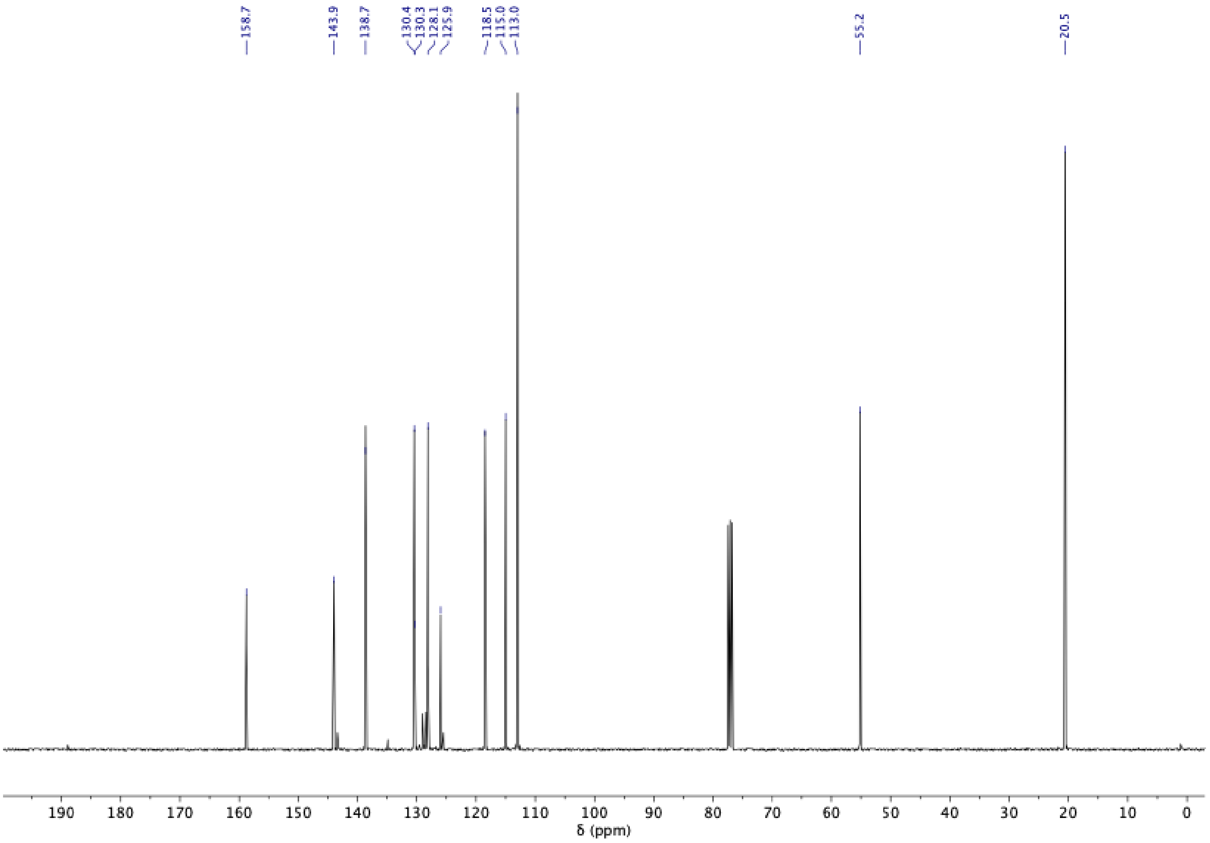
158.7
143.9
138.7
130.4
130.3
128.1
125.9
118.5
115.0
113.0
55.2
20.5
δ (ppm)

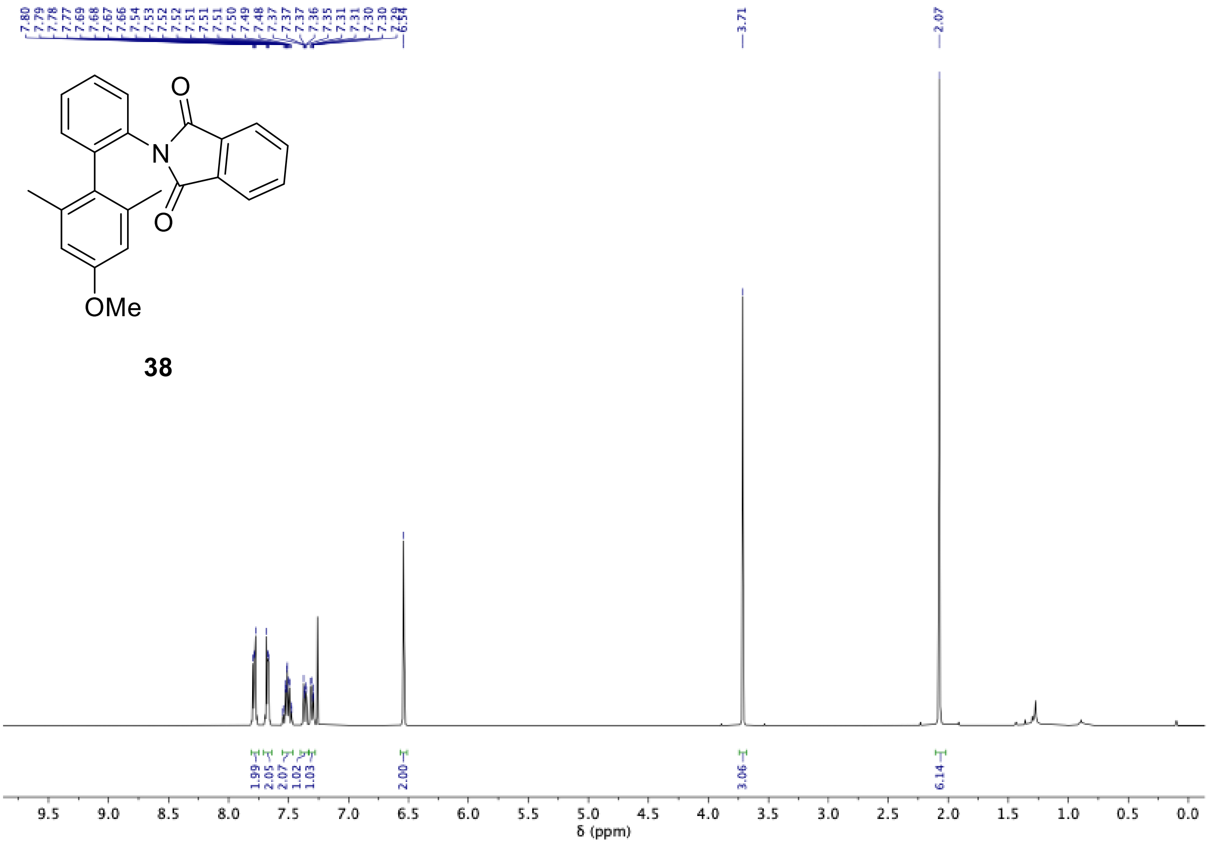
O
N
O
OMe
7.80
7.79
7.78
7.77
7.69
7.68
7.67
7.66
7.54
7.53
7.52
7.52
7.51
7.51
7.51
7.50
7.49
7.48
7.37
7.37
7.37
7.36
7.35
7.31
7.31
7.30
7.29
6.54
3.71
2.07
1.99
2.05
2.07
1.02
1.03
2.00
3.06
6.14
9.5 9.0 8.5 8.0 7.5 7.0 6.5 6.0 5.5 5.0 4.5 4.0 3.5 3.0 2.5 2.0 1.5 1.0 0.5 0.0
δ (ppm)

38

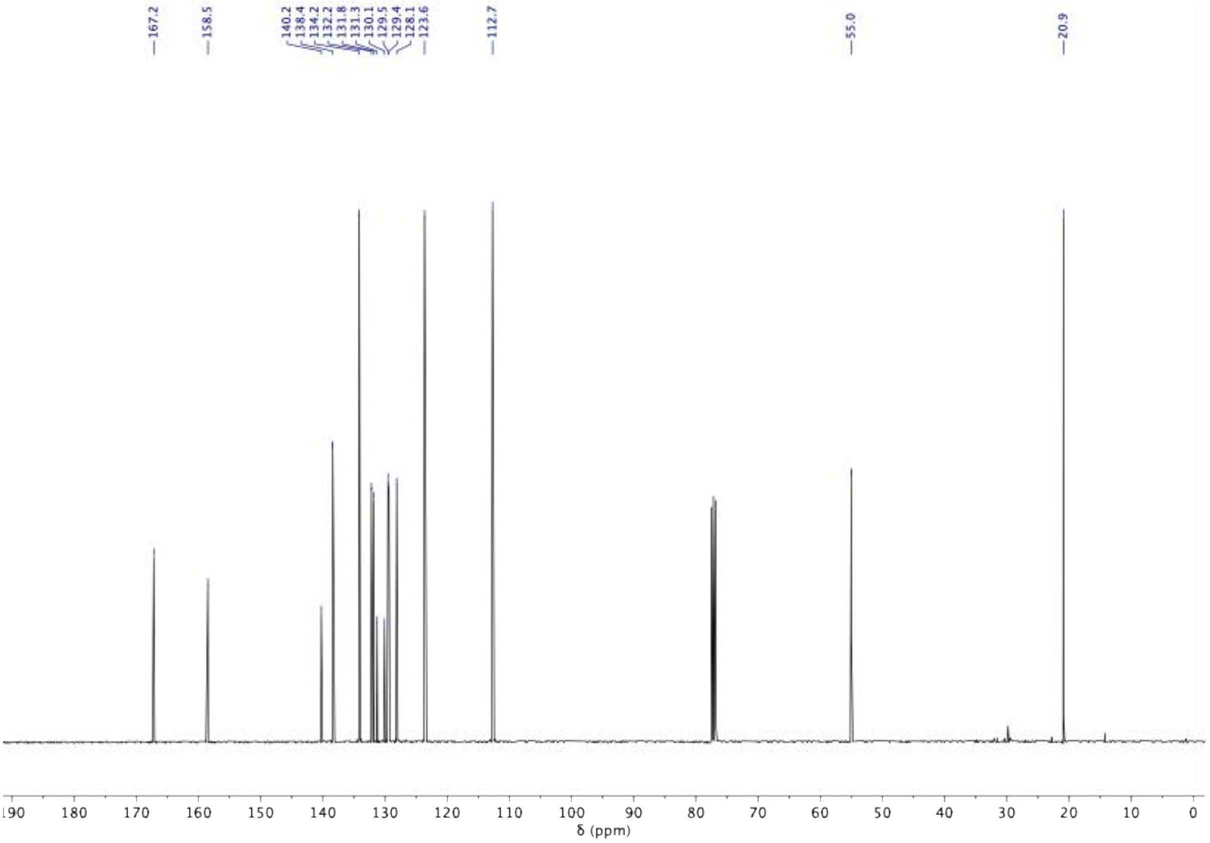
167.2
158.5
140.2
138.4
134.2
132.2
131.8
131.3
130.1
129.5
129.4
128.1
123.6
112.7
55.0
20.9
190 180 170 160 150 140 130 120 110 100 90 80 70 60 50 40 30 20 10 0
δ (ppm)

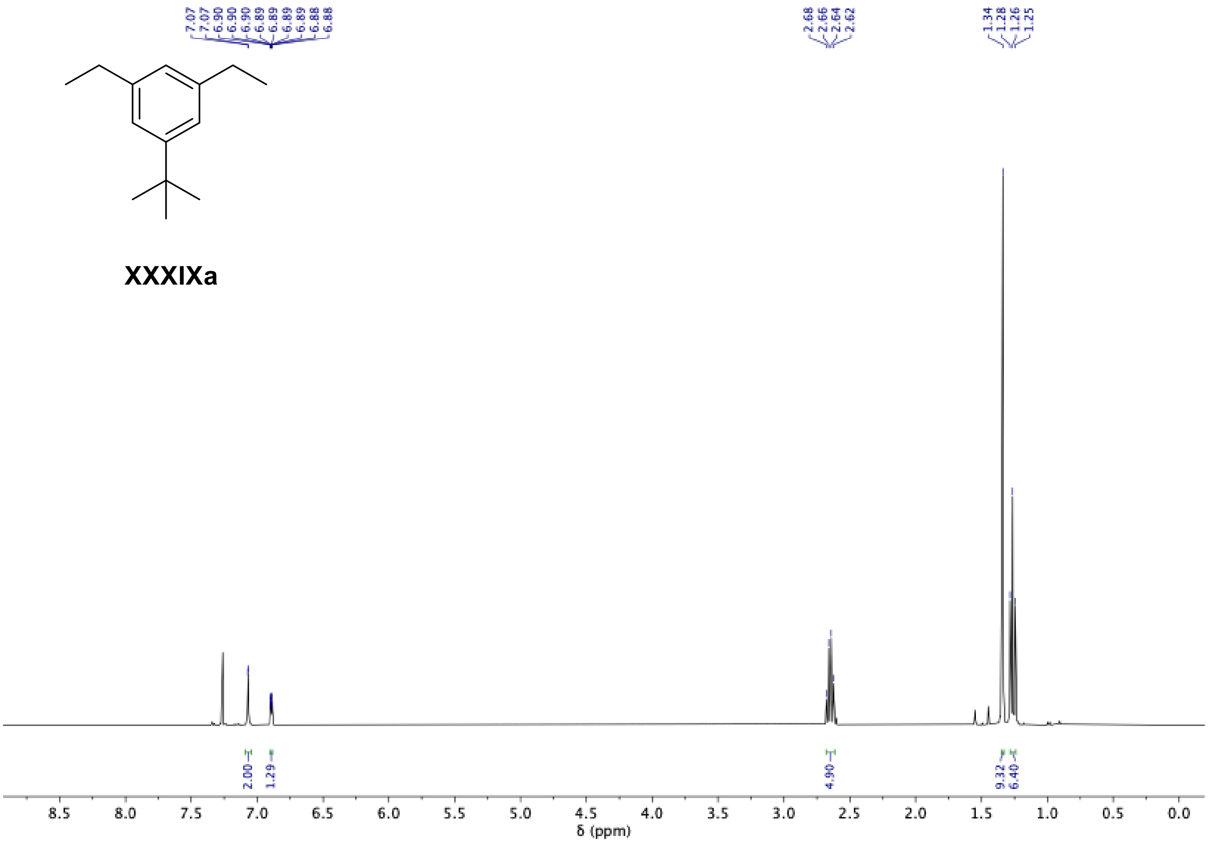

XXXIXa
7.07
7.07
6.90
6.90
6.90
6.89
6.89
6.89
6.89
6.88
6.88
2.68
2.66
2.64
2.62
1.34
1.28
1.26
1.25
2.00
1.29
4.90
9.32
6.40
8.5 8.0 7.5 7.0 6.5 6.0 5.5 5.0 4.5 4.0 3.5 3.0 2.5 2.0 1.5 1.0 0.5 0.0
δ (ppm)

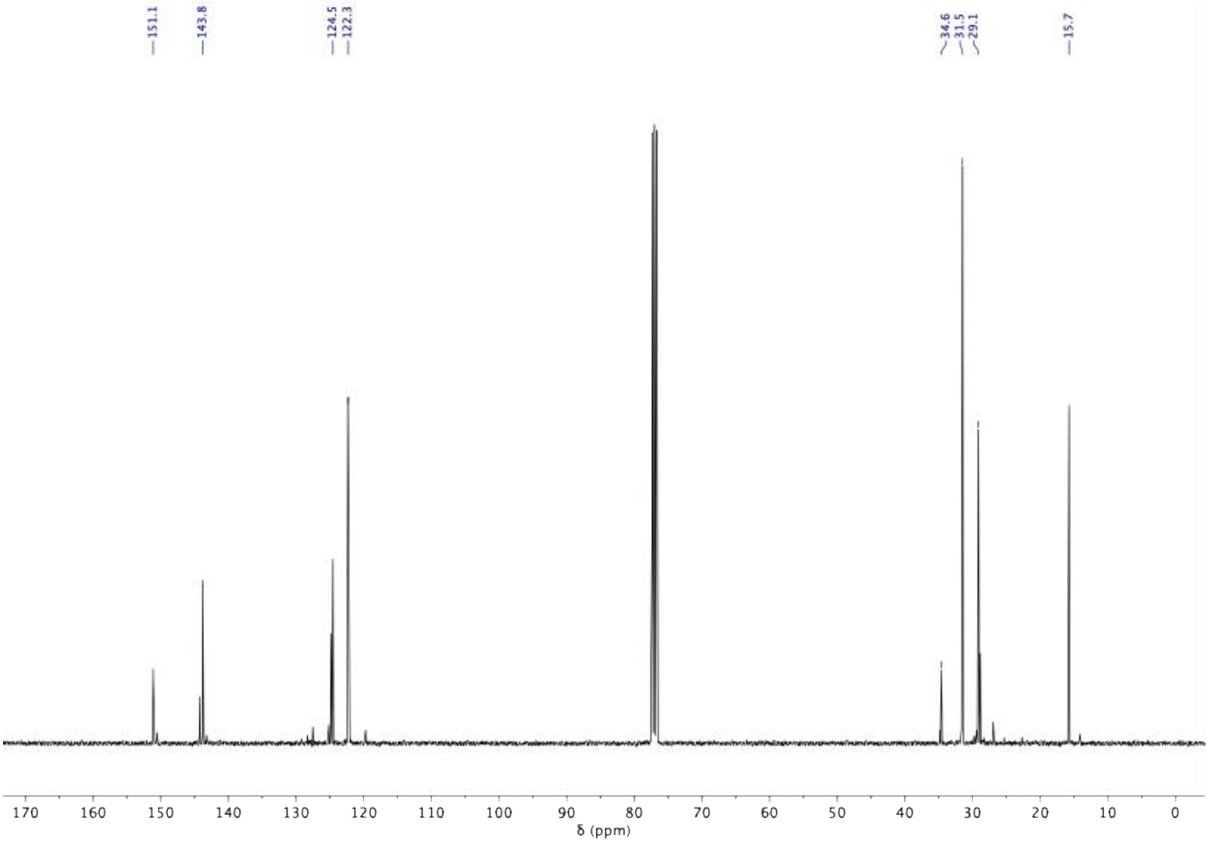
151.1
143.8
124.5
122.3
34.6
31.5
29.1
15.7
170 160 150 140 130 120 110 100 90 80 70 60 50 40 30 20 10 0
δ (ppm)

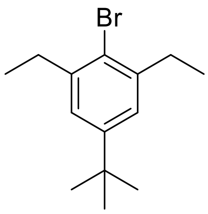
Br

XXXIXb

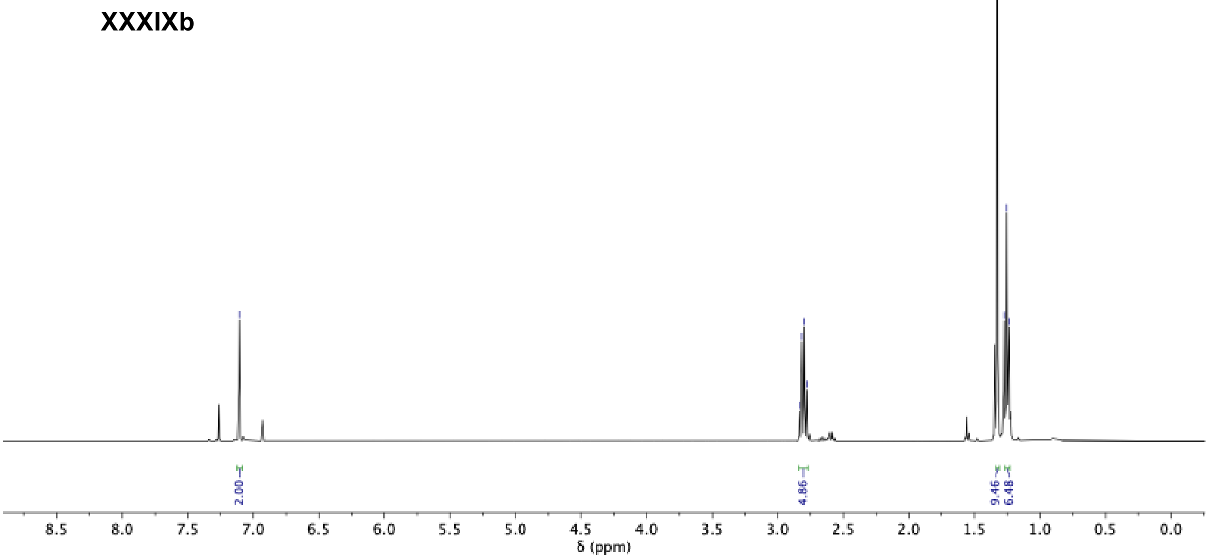
7.11
2.83
2.82
2.80
2.78
1.32
1.27
1.25
1.24
2.00
4.86
9.46
6.48
8.5 8.0 7.5 7.0 6.5 6.0 5.5 5.0 4.5 4.0 3.5 3.0 2.5 2.0 1.5 1.0 0.5 0.0
δ (ppm)

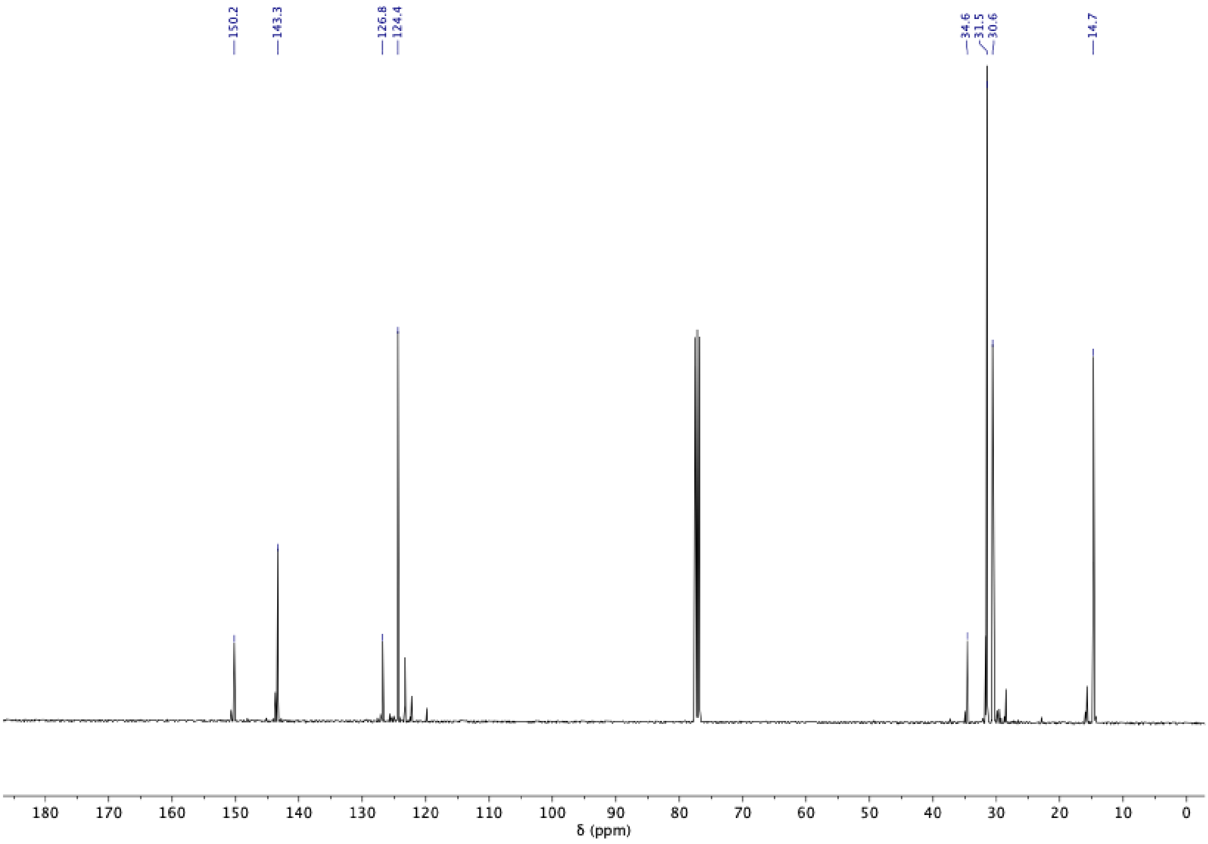
150.2
143.3
126.8
124.4
34.6
31.5
30.6
14.7
180 170 160 150 140 130 120 110 100 90 80 70 60 50 40 30 20 10 0
δ (ppm)

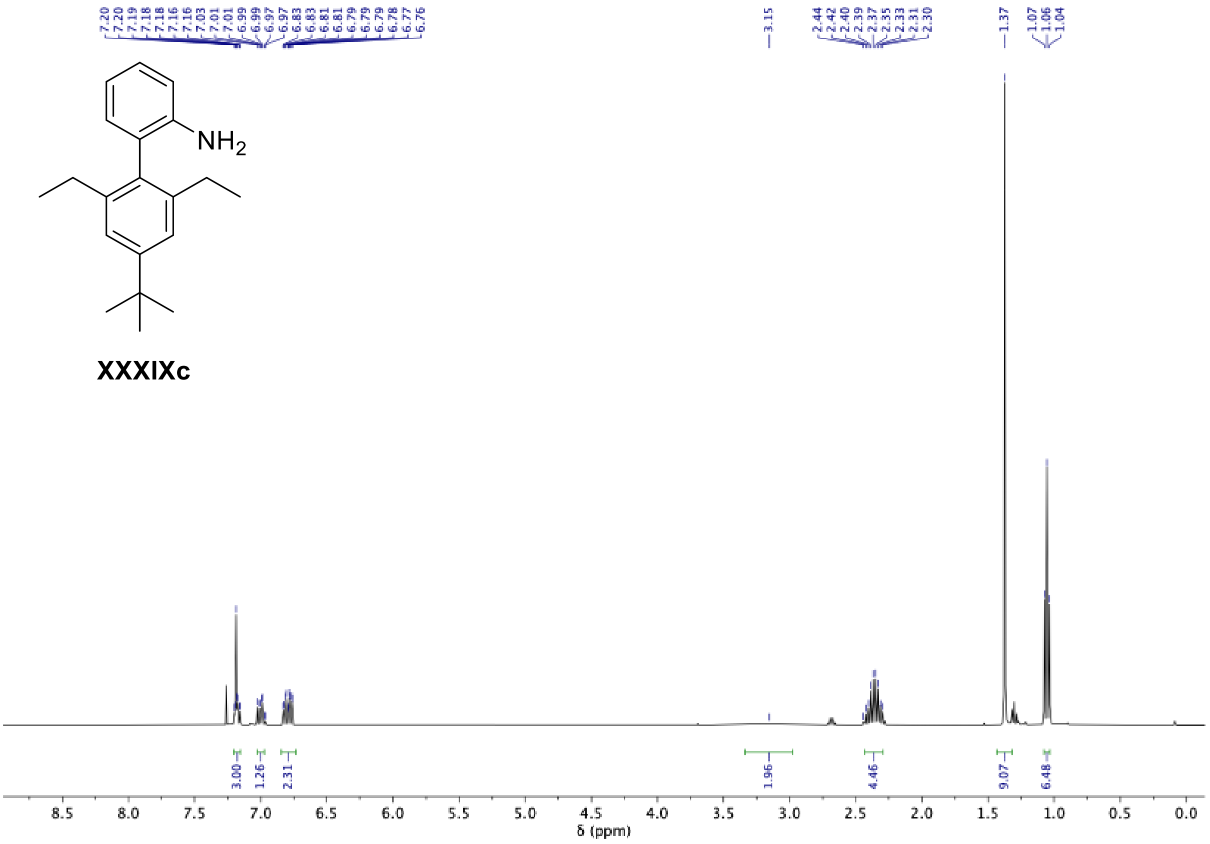
NH2
XXXIXc
7.20 7.20 7.19 7.18 7.18 7.16 7.16 7.03 7.01 7.01 6.99 6.99 6.97 6.97 6.83 6.83 6.81 6.81 6.79 6.79 6.78 6.77 6.76
3.15
2.44 2.42 2.40 2.39 2.37 2.35 2.33 2.31 2.30
1.37
1.07 1.06 1.04
3.00 1.26 2.31 1.96 4.46 9.07 6.48
8.5 8.0 7.5 7.0 6.5 6.0 5.5 5.0 4.5 4.0 3.5 3.0 2.5 2.0 1.5 1.0 0.5 0.0
δ (ppm)

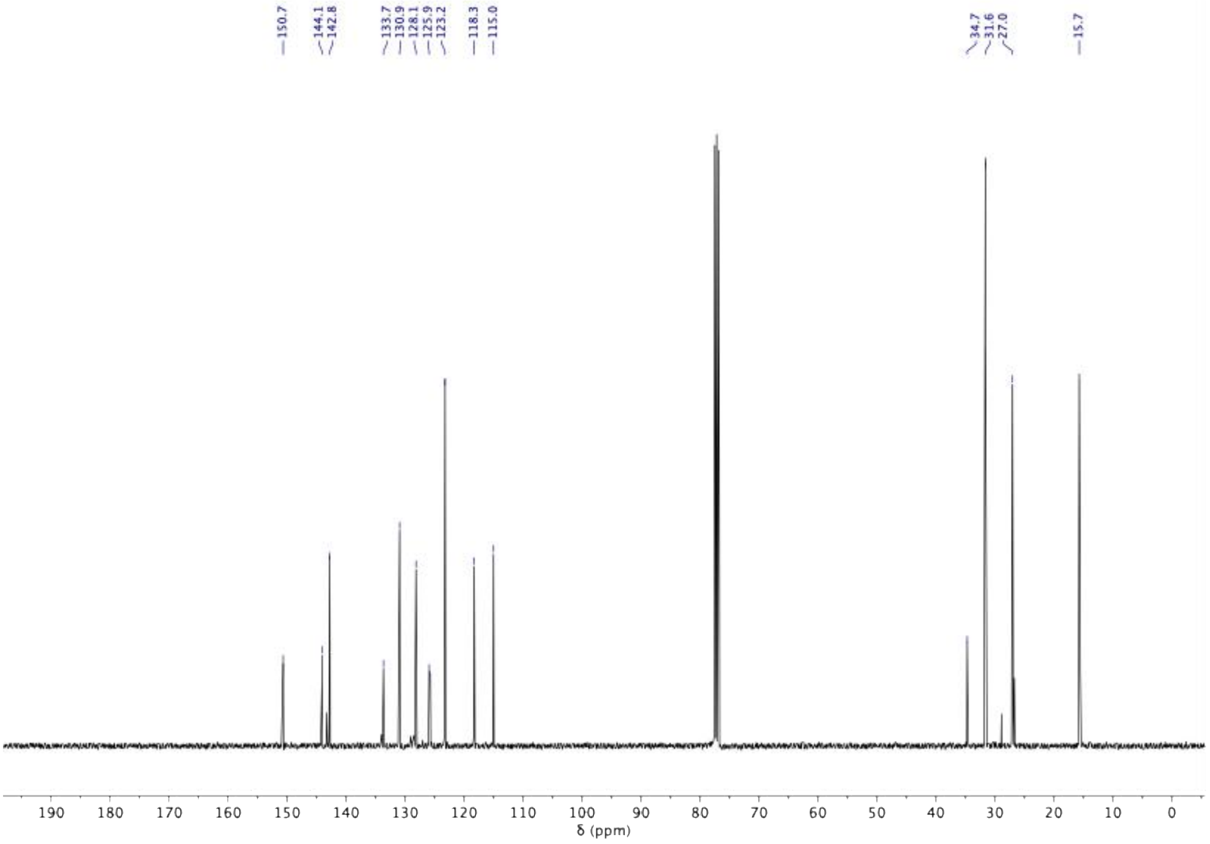
150.7
144.1 142.8
133.7 130.9 128.1 125.9 123.2
118.3 115.0
34.7 31.6 27.0
15.7
190 180 170 160 150 140 130 120 110 100 90 80 70 60 50 40 30 20 10 0
δ (ppm)

39

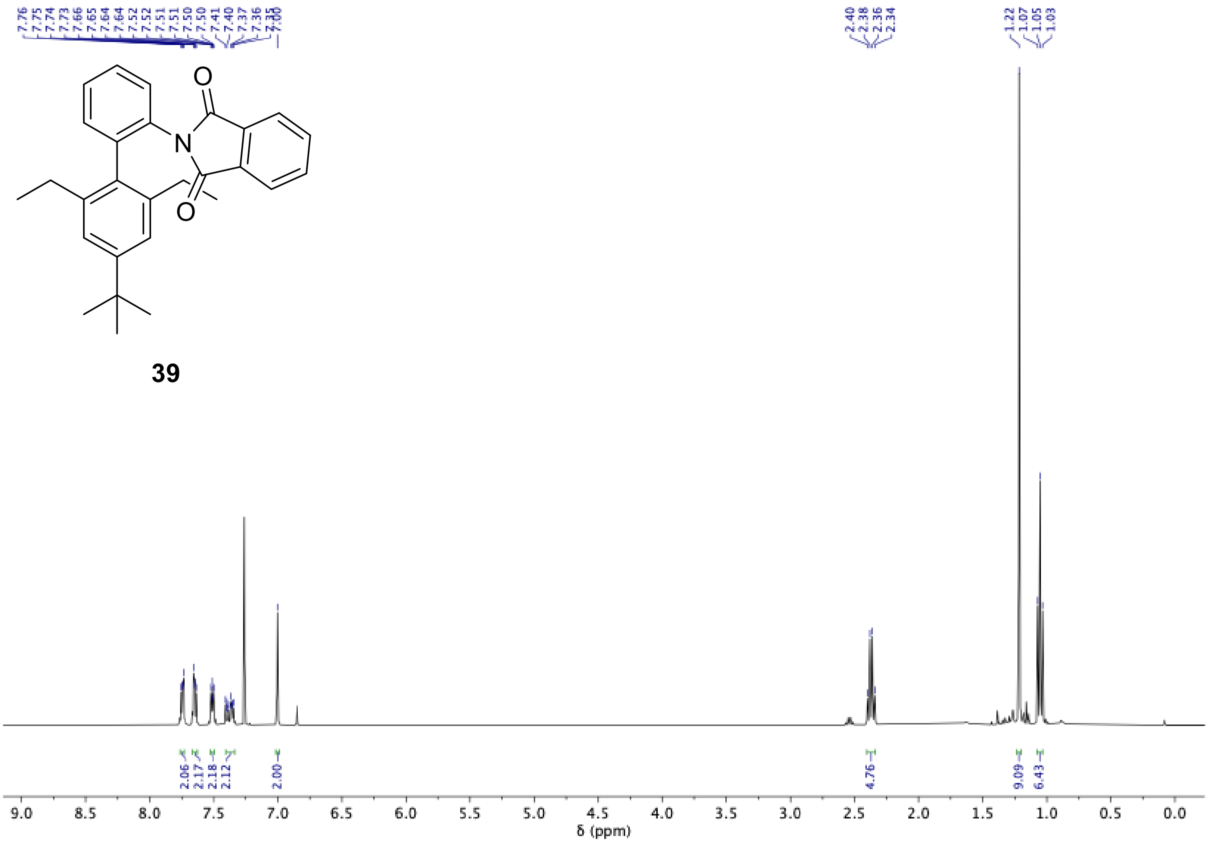

O
N
O
7.76
7.75
7.74
7.73
7.66
7.65
7.64
7.64
7.52
7.52
7.51
7.51
7.50
7.50
7.41
7.40
7.37
7.36
7.35
7.00
2.40
2.38
2.36
2.34
1.22
1.07
1.05
1.03
2.06
2.17
2.18
2.12
2.00
4.76
9.09
6.43
9.0 8.5 8.0 7.5 7.0 6.5 6.0 5.5 5.0 4.5 4.0 3.5 3.0 2.5 2.0 1.5 1.0 0.5 0.0
δ (ppm)

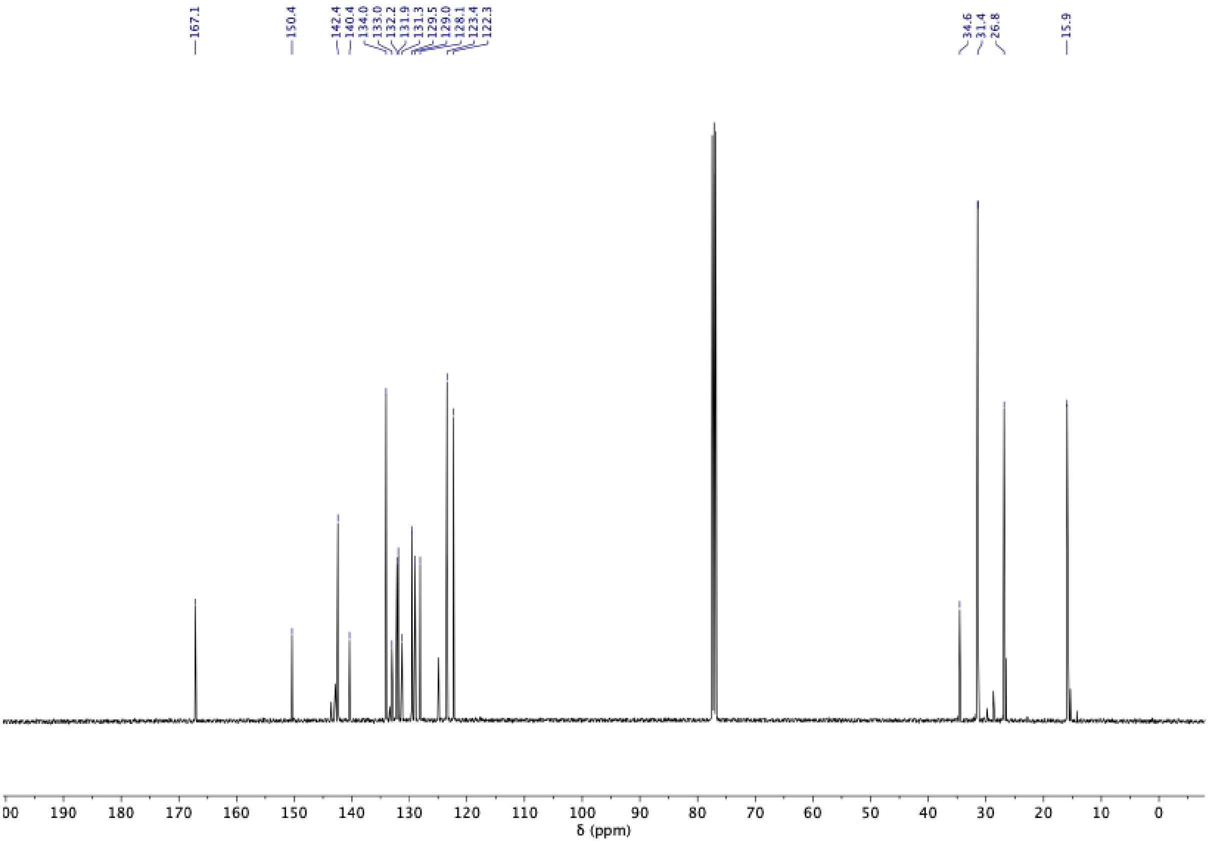

167.1
150.4
142.4
140.4
134.0
133.0
132.2
131.9
131.3
129.5
129.0
128.1
123.4
122.3
34.6
31.4
26.8
15.9
00 190 180 170 160 150 140 130 120 110 100 90 80 70 60 50 40 30 20 10 0
δ (ppm)

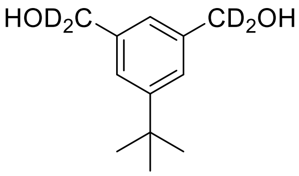
HOD2C
CD2OH

XLIIIb

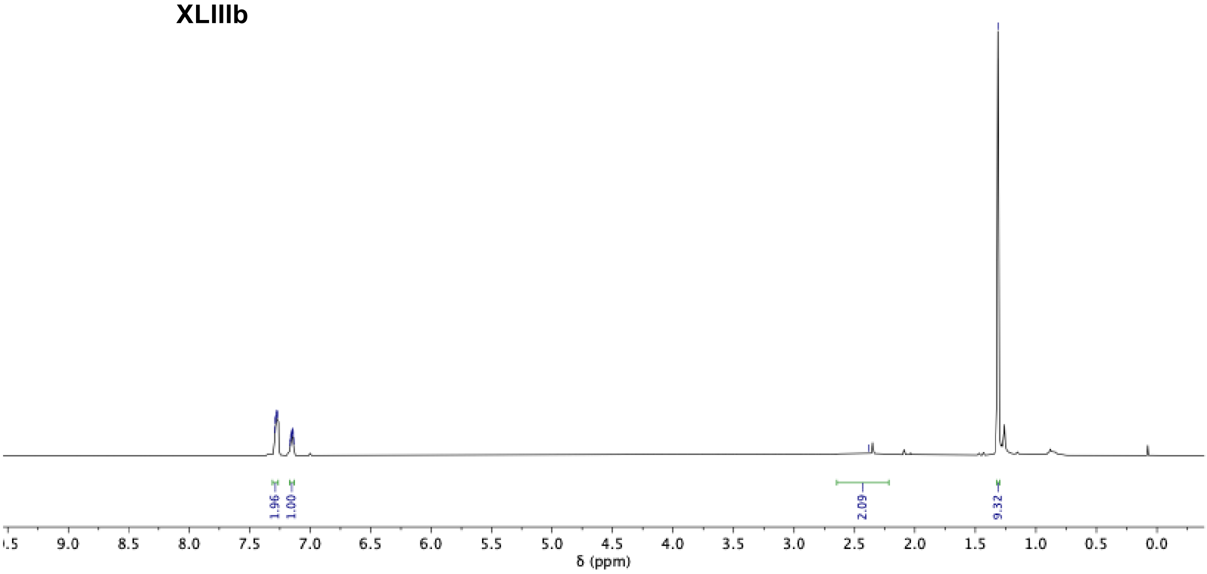
7.29
7.29
7.28
7.28
7.27
7.27
7.17
7.16
7.16
7.15
7.15
7.15
7.14
7.14
2.38
1.31
1.96
1.00
2.09
9.32
δ (ppm)

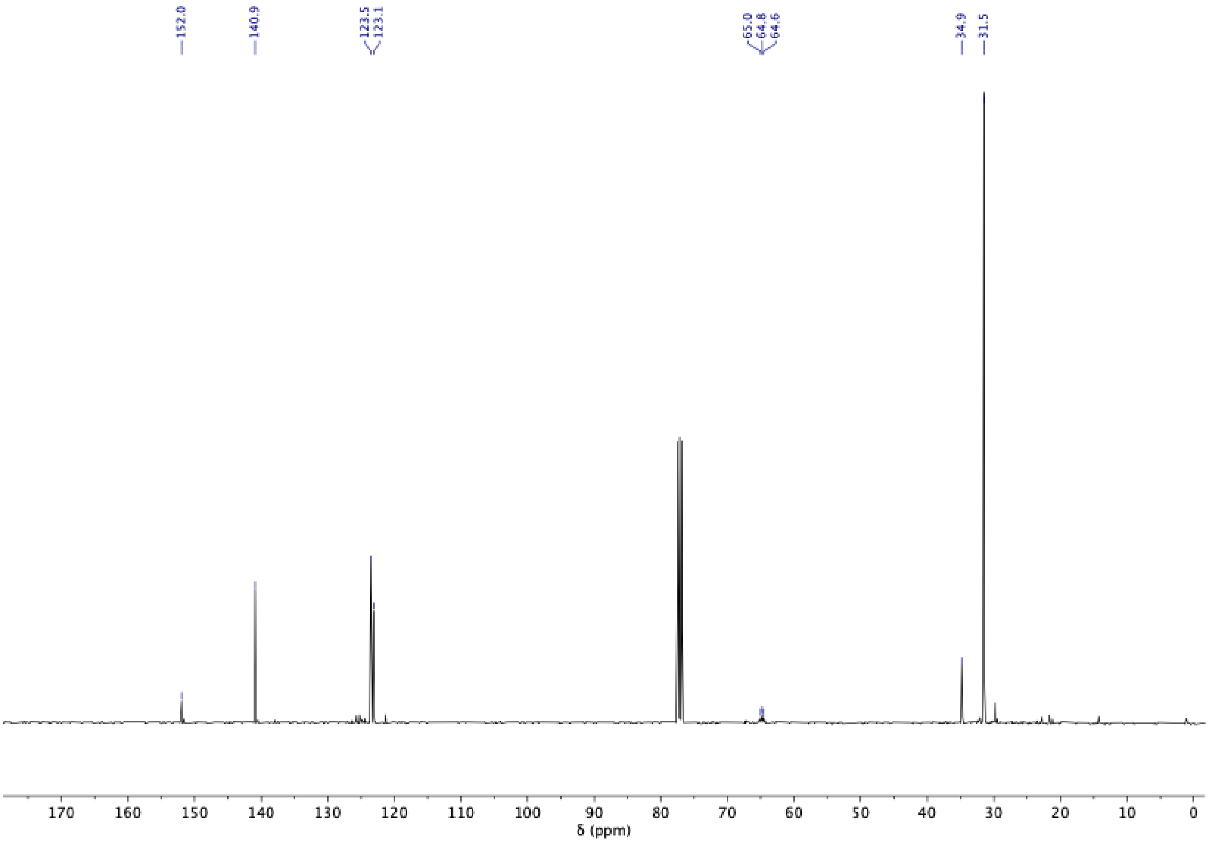
152.0
140.9
123.5
123.1
65.0
64.8
64.6
34.9
31.5
δ (ppm)

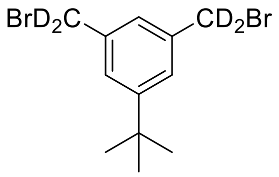
BrD2C
CD2Br

XLIIIc

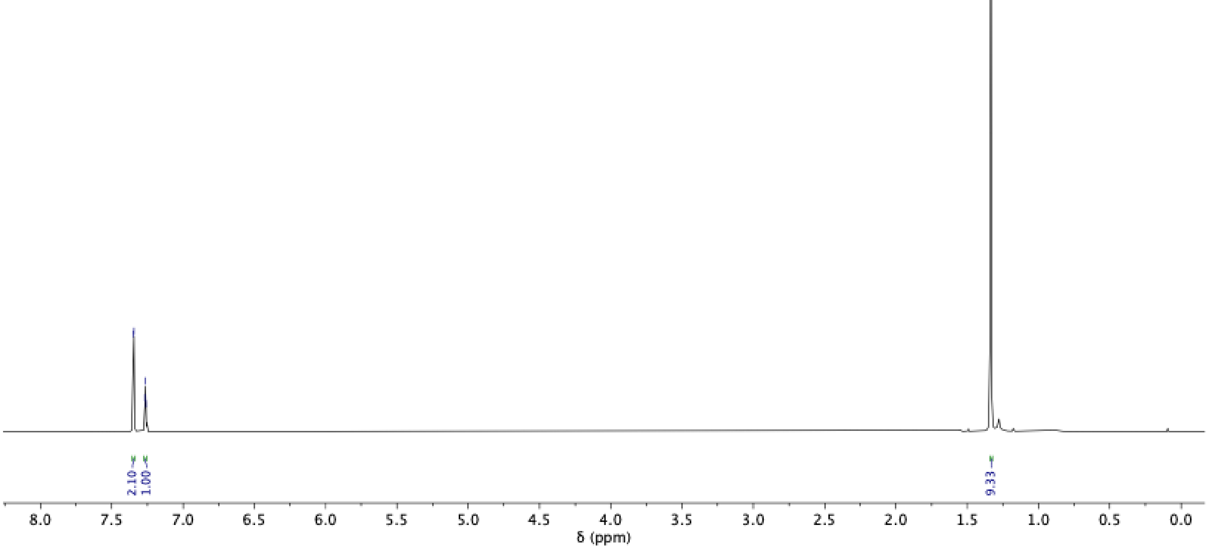
7.35
7.34
7.27
7.26
7.26
1.34
2.10
1.00
9.33
8.0
7.5
7.0
6.5
6.0
5.5
5.0
4.5
4.0
3.5
3.0
2.5
2.0
1.5
1.0
0.5
0.0
δ (ppm)

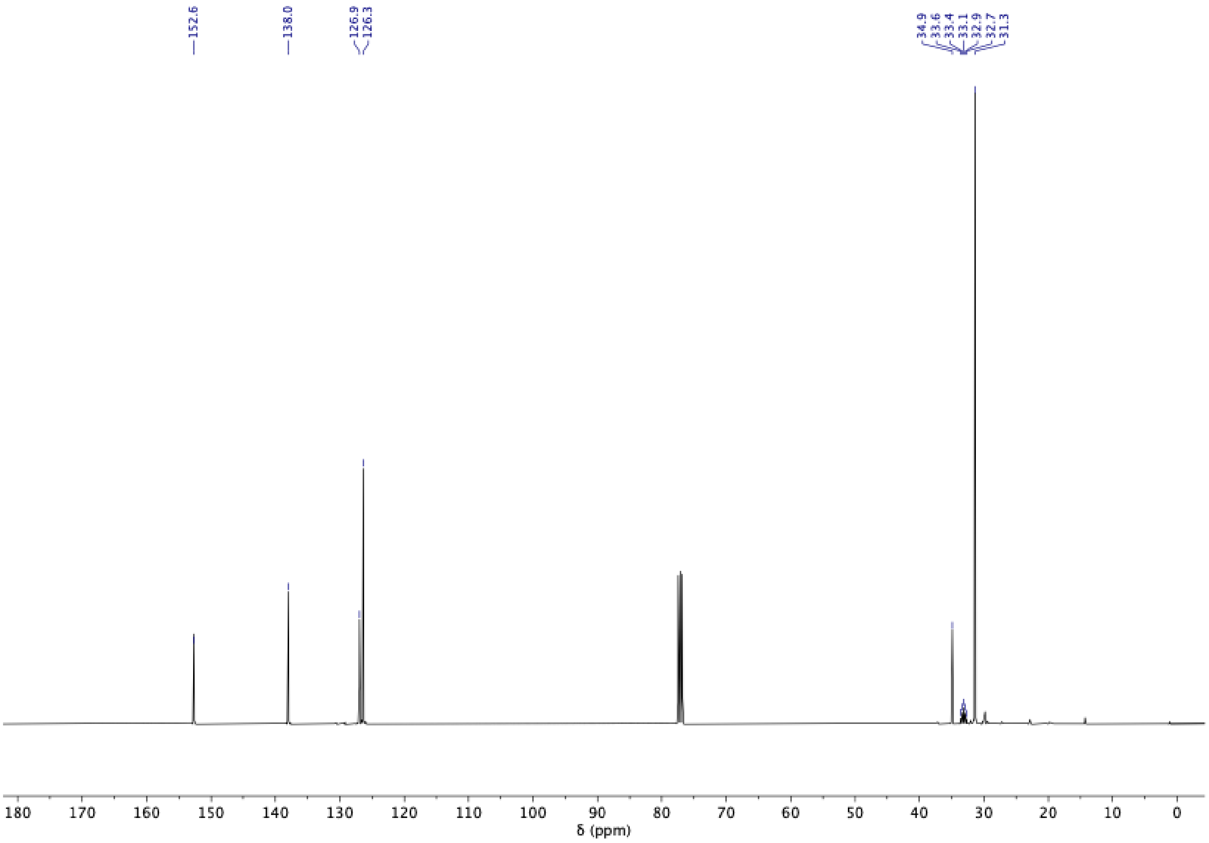
152.6
138.0
126.9
126.3
34.9
33.6
33.4
33.1
32.9
32.7
31.3
180
170
160
150
140
130
120
110
100
90
80
70
60
50
40
30
20
10
0
δ (ppm)

XLIIId

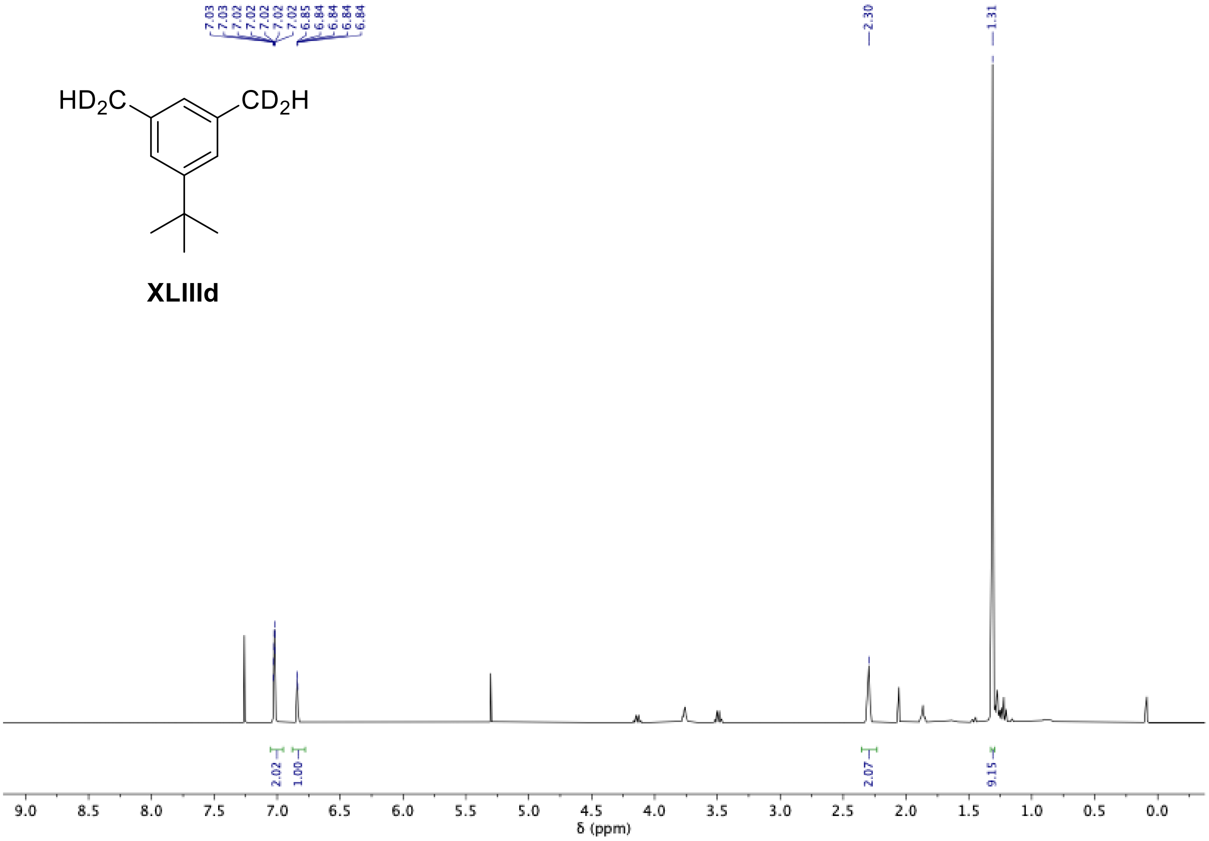
HD2C
CD2H
7.03
7.03
7.02
7.02
7.02
7.02
7.02
6.85
6.84
6.84
6.84
6.84
2.30
1.31
2.02
1.00
2.07
9.15
9.0 8.5 8.0 7.5 7.0 6.5 6.0 5.5 5.0 4.5 4.0 3.5 3.0 2.5 2.0 1.5 1.0 0.5 0.0
δ (ppm)

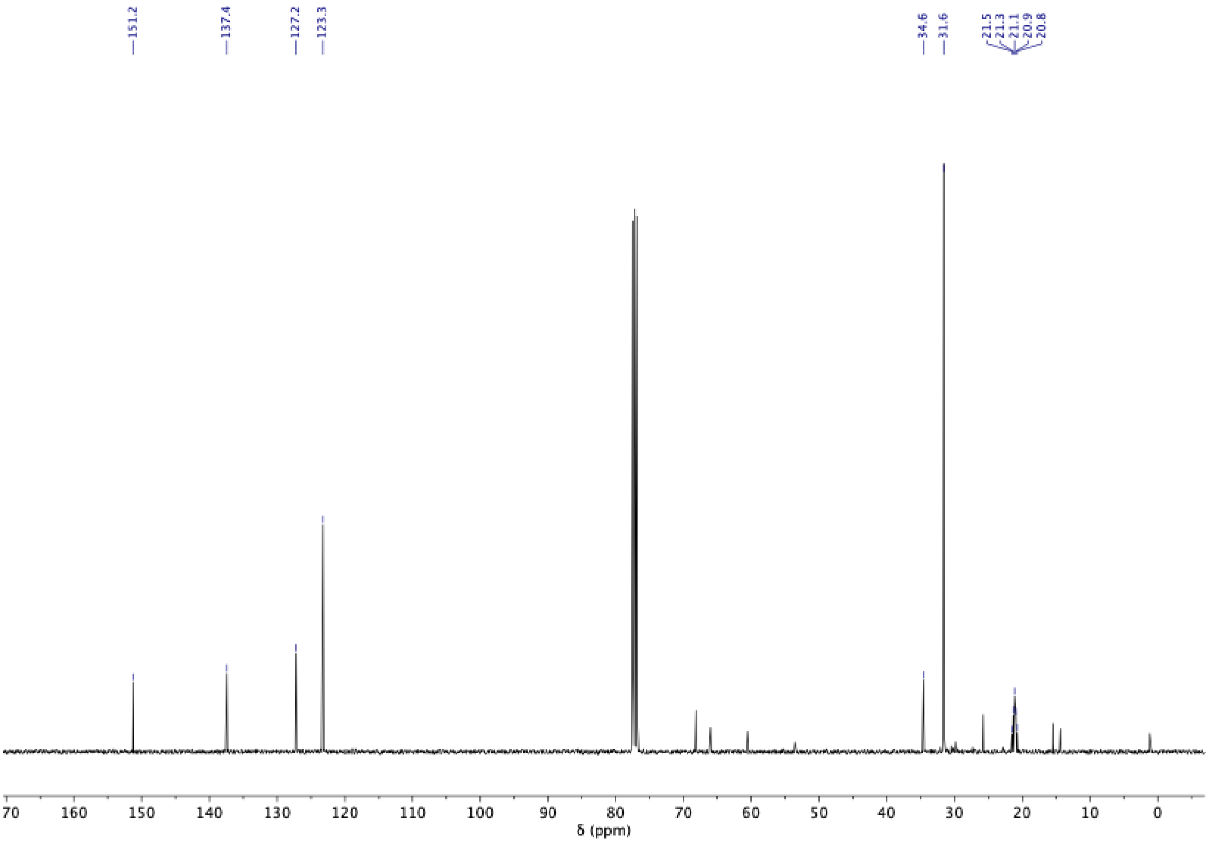
151.2
137.4
127.2
123.3
34.6
31.6
21.5
21.3
21.1
20.9
20.8
70 160 150 140 130 120 110 100 90 80 70 60 50 40 30 20 10 0
δ (ppm)

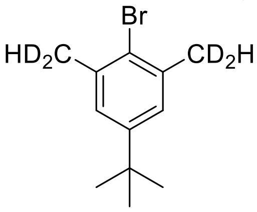
Br
HD_2C
CD_2H

XLIIIe

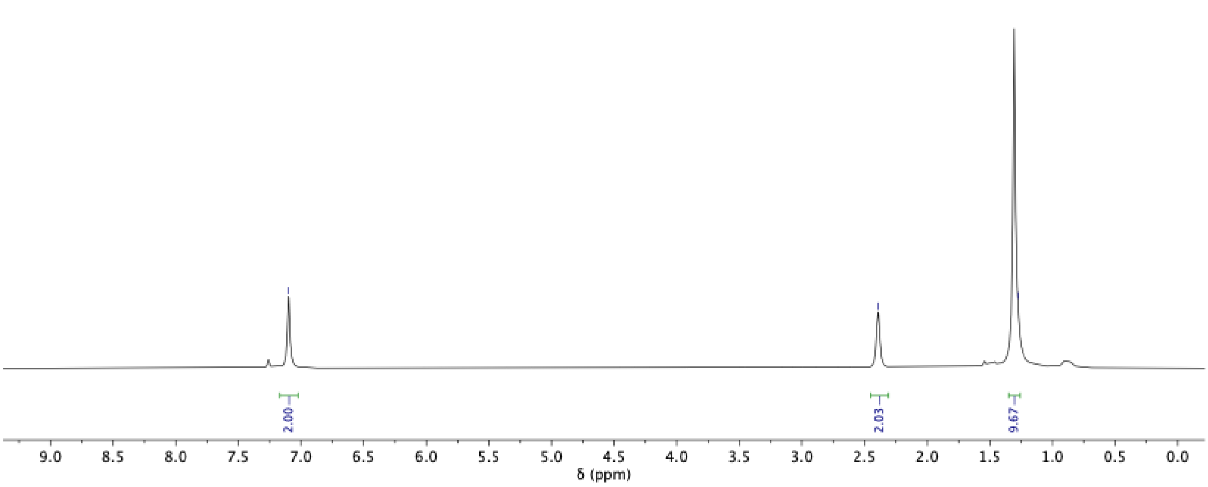
7.10
2.39
1.28
2.00
2.03
9.67
9.0 8.5 8.0 7.5 7.0 6.5 6.0 5.5 5.0 4.5 4.0 3.5 3.0 2.5 2.0 1.5 1.0 0.5 0.0
δ (ppm)

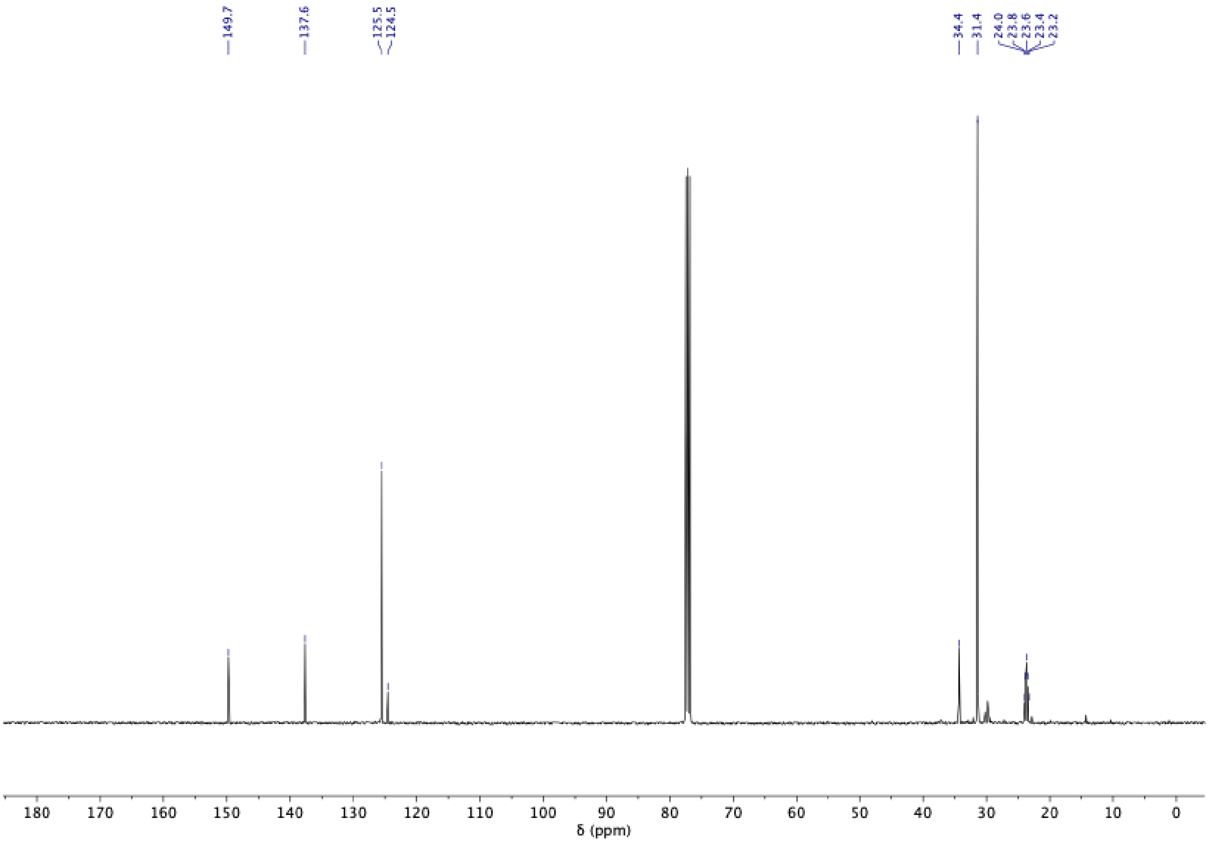
149.7
137.6
125.5
124.5
34.4
31.4
24.0
23.8
23.6
23.4
23.2
180 170 160 150 140 130 120 110 100 90 80 70 60 50 40 30 20 10 0
δ (ppm)

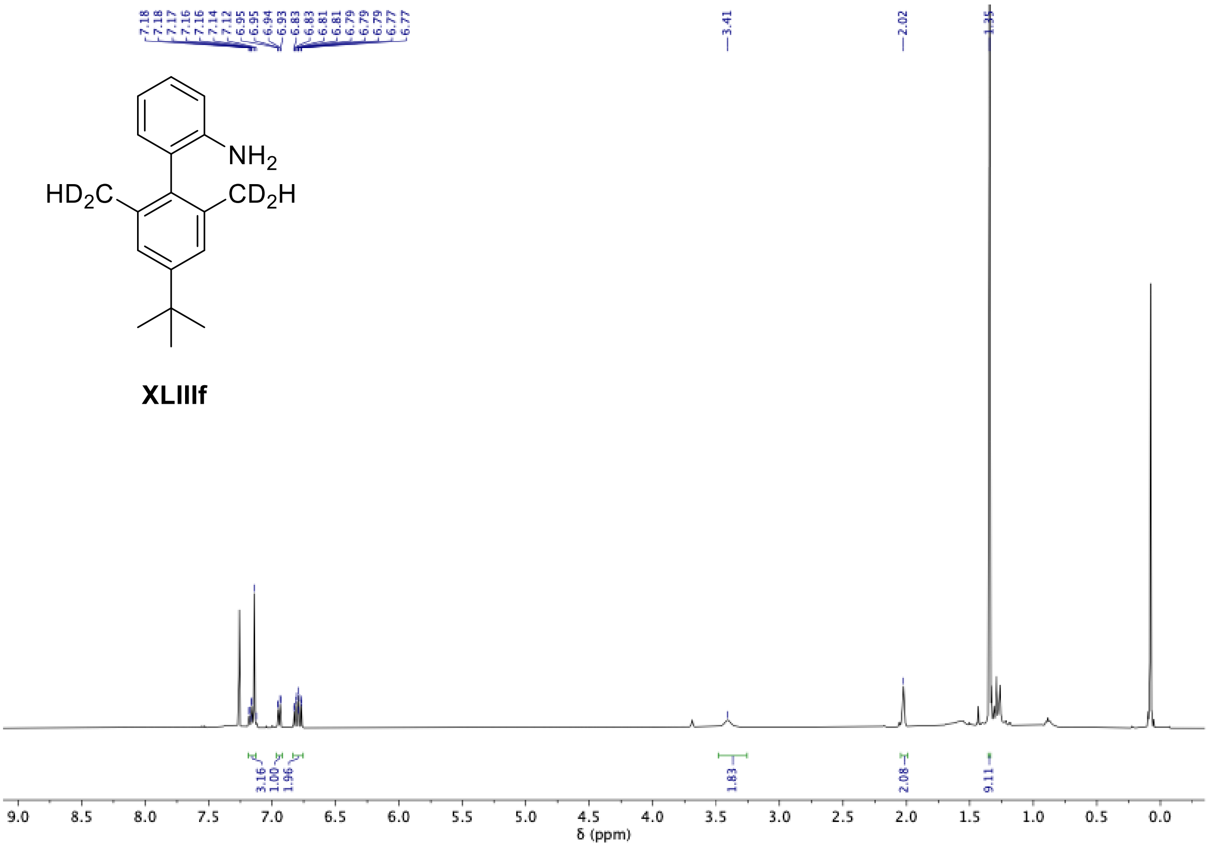
NH2
HD2C
CD2H
7.18
7.18
7.17
7.16
7.16
7.14
7.12
6.95
6.95
6.94
6.93
6.83
6.83
6.81
6.81
6.79
6.79
6.77
6.77
3.41
2.02
1.35
3.16
1.00
1.96
1.83
2.08
9.11
9.0 8.5 8.0 7.5 7.0 6.5 6.0 5.5 5.0 4.5 4.0 3.5 3.0 2.5 2.0 1.5 1.0 0.5 0.0
δ (ppm)

XLIIIf

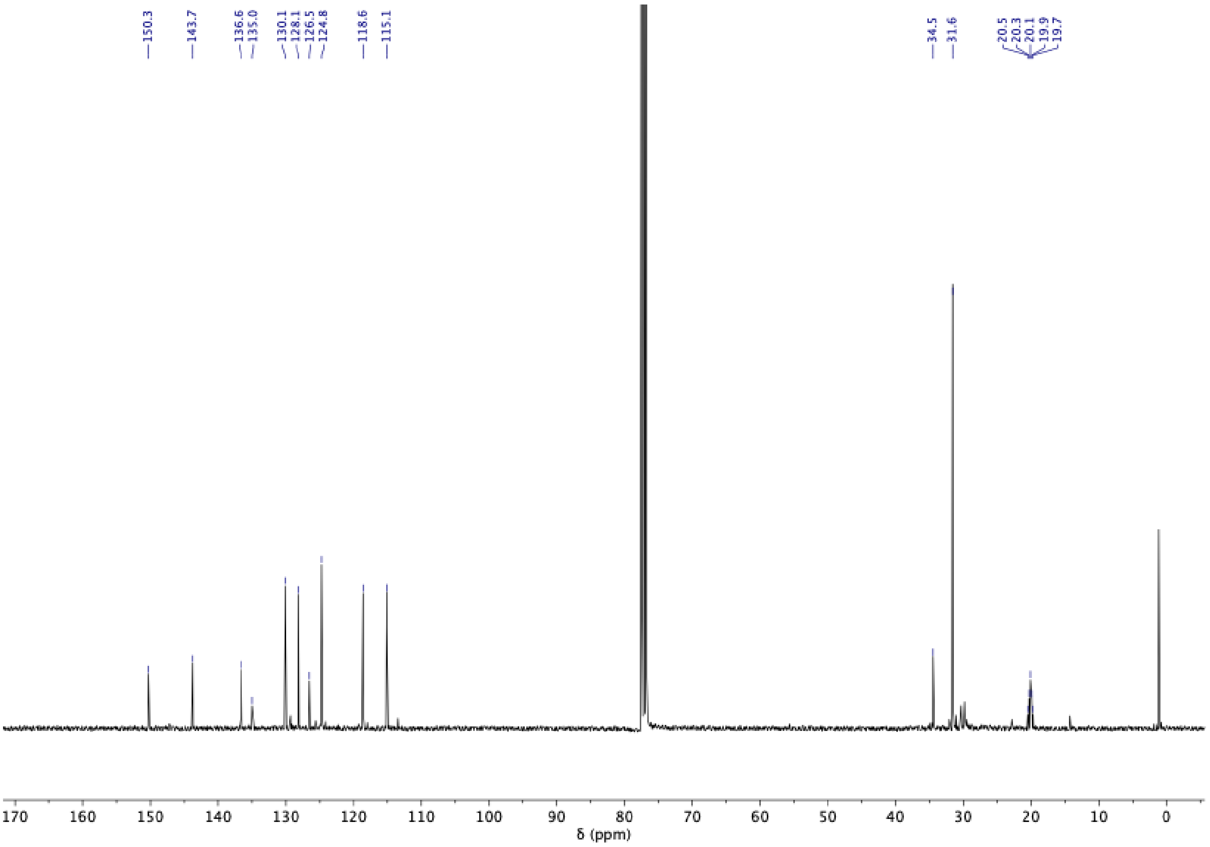
150.3
143.7
136.6
135.0
130.1
128.1
126.5
124.8
118.6
115.1
34.5
31.6
20.5
20.3
20.1
19.9
19.7
170 160 150 140 130 120 110 100 90 80 70 60 50 40 30 20 10 0
δ (ppm)

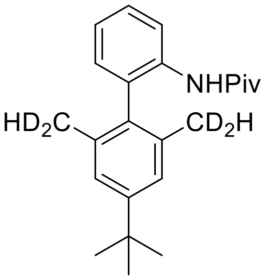
NHPiv
HD2C
CD2H

43

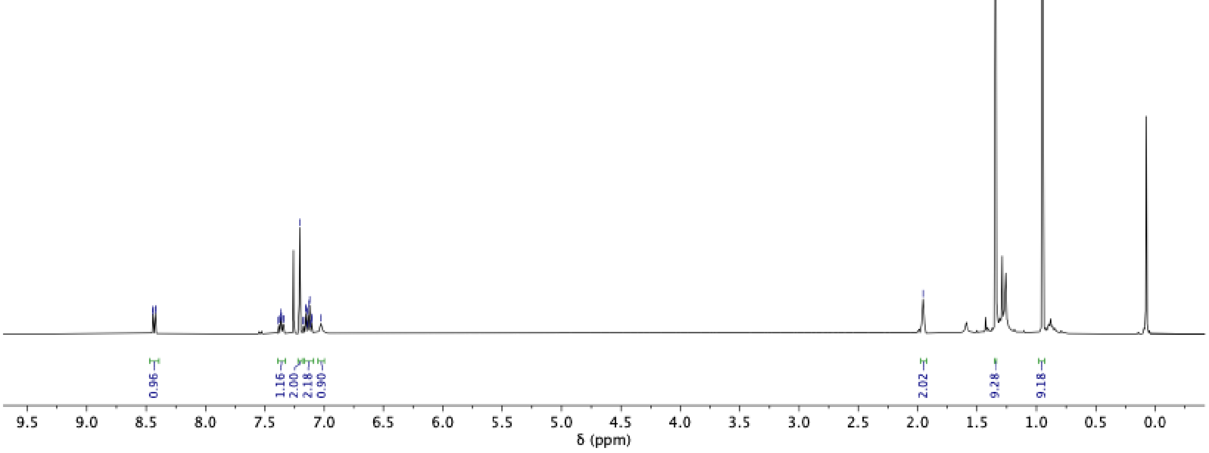
8.45
8.44
8.43
8.42
7.39
7.38
7.37
7.36
7.36
7.35
7.34
7.20
7.18
7.17
7.16
7.15
7.14
7.14
7.14
7.13
7.12
7.12
7.11
7.10
7.03
1.95
1.34
0.95
0.96
1.16
2.00
2.18
0.90
2.02
9.28
9.18
9.5 9.0 8.5 8.0 7.5 7.0 6.5 6.0 5.5 5.0 4.5 4.0 3.5 3.0 2.5 2.0 1.5 1.0 0.5 0.0
δ (ppm)

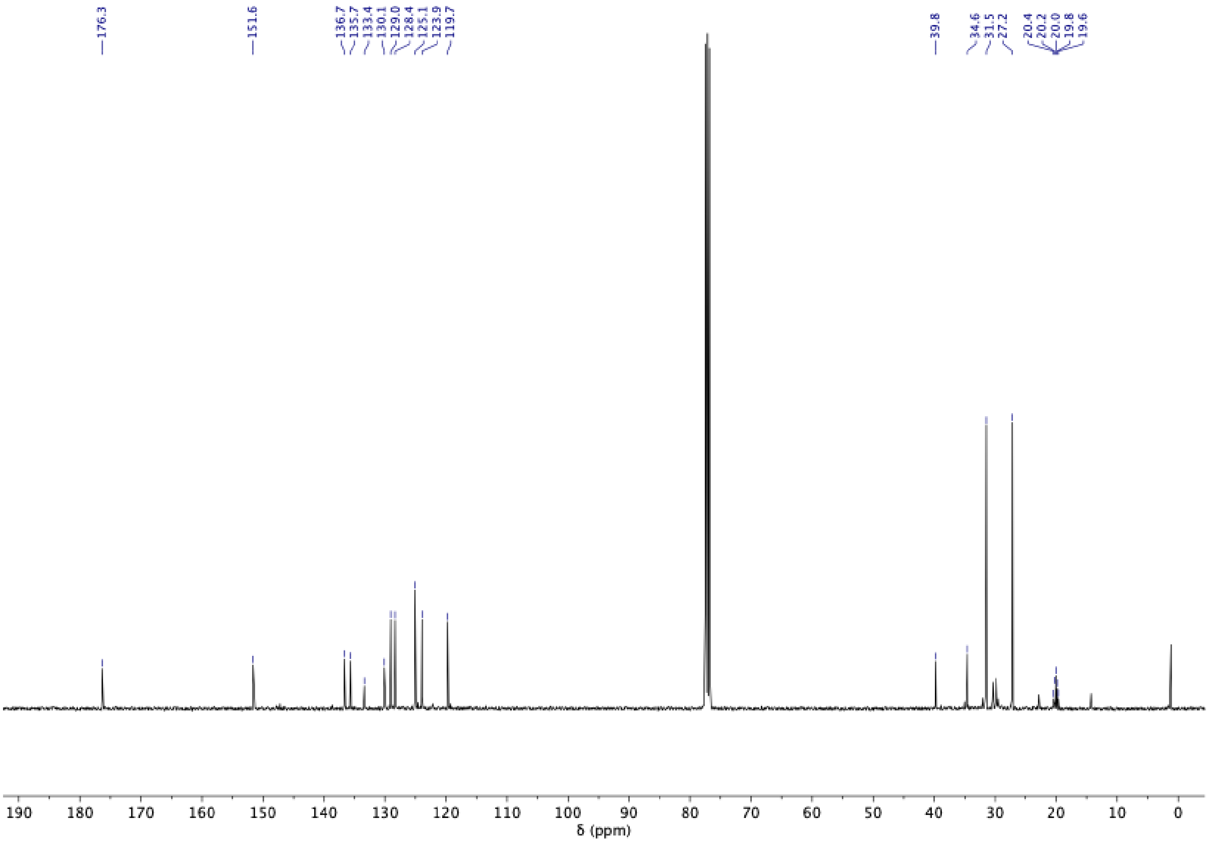
176.3
151.6
136.7
135.7
133.4
130.1
129.0
128.4
125.1
123.9
119.7
39.8
34.6
31.5
27.2
20.4
20.2
20.0
19.8
19.6
190 180 170 160 150 140 130 120 110 100 90 80 70 60 50 40 30 20 10 0
δ (ppm)

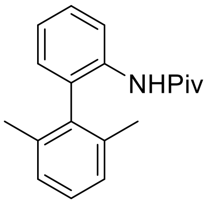
NHPiv

44

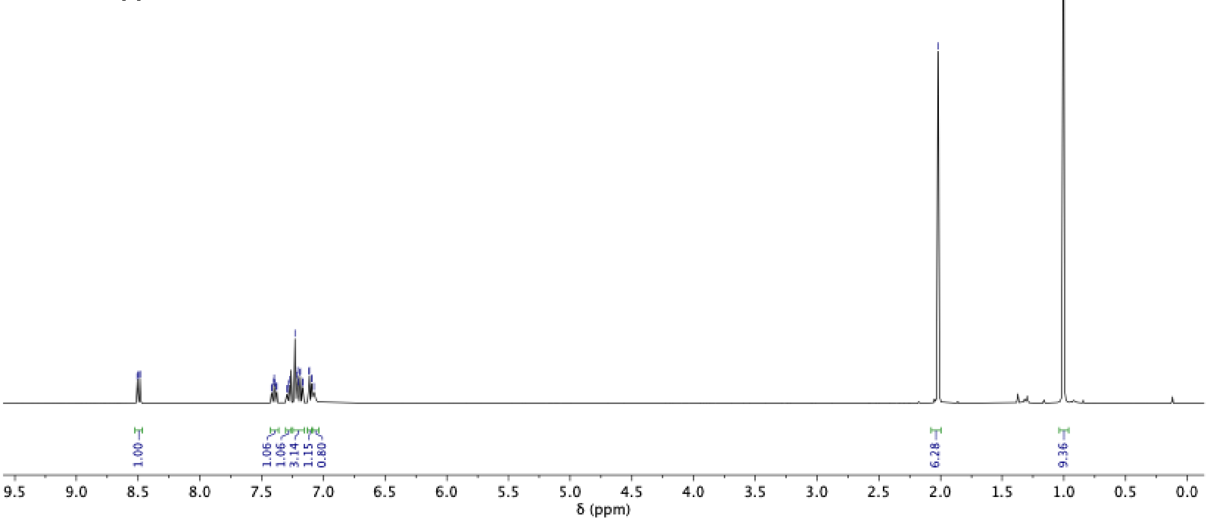
8.51
8.50
8.49
8.48
7.42
7.42
7.40
7.39
7.38
7.38
7.30
7.29
7.28
7.28
7.23
7.21
7.21
7.20
7.19
7.18
7.17
7.17
7.12
7.11
7.10
7.09
7.08
2.02
1.00
1.00
1.06
1.06
3.14
1.15
0.80
6.28
9.36
9.5 9.0 8.5 8.0 7.5 7.0 6.5 6.0 5.5 5.0 4.5 4.0 3.5 3.0 2.5 2.0 1.5 1.0 0.5 0.0
δ (ppm)

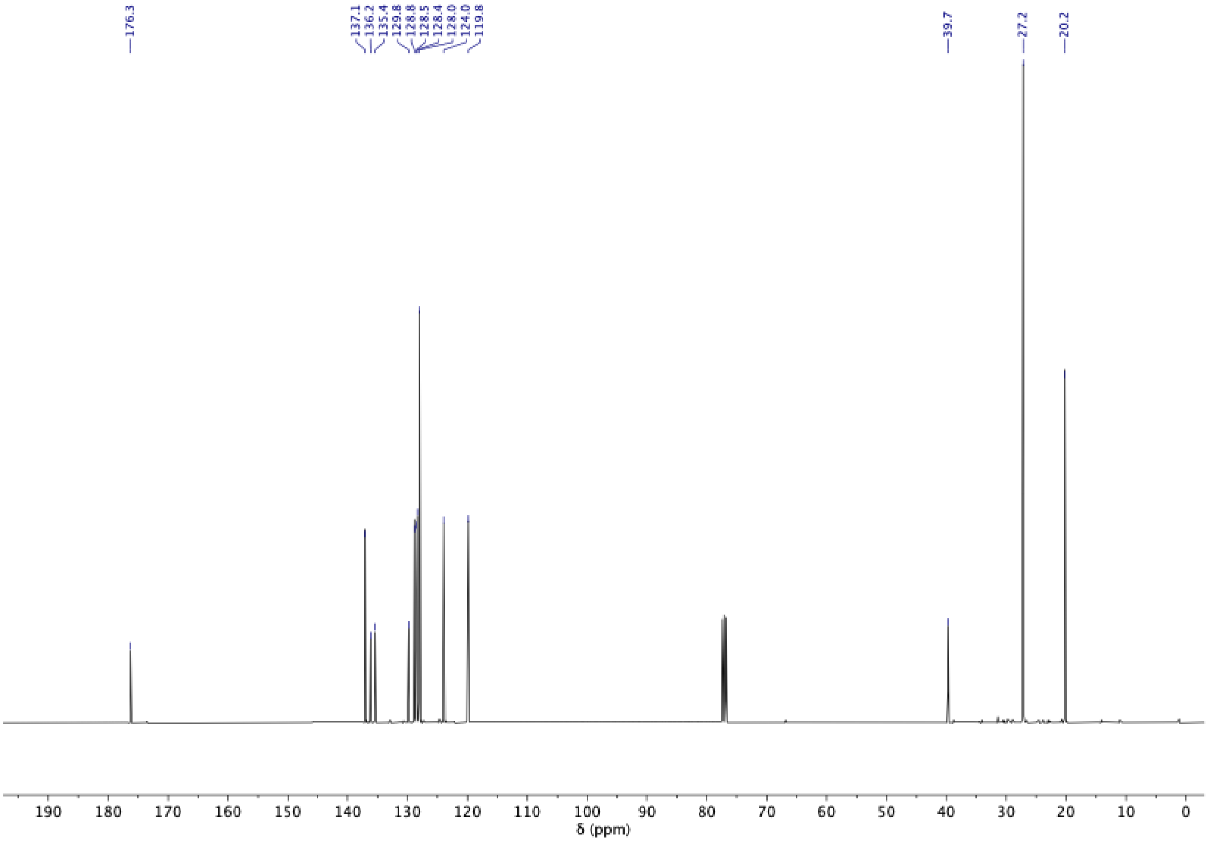
176.3
137.1
136.2
135.4
129.8
128.8
128.5
128.4
128.0
124.0
119.8
39.7
27.2
20.2
190 180 170 160 150 140 130 120 110 100 90 80 70 60 50 40 30 20 10 0
δ (ppm)

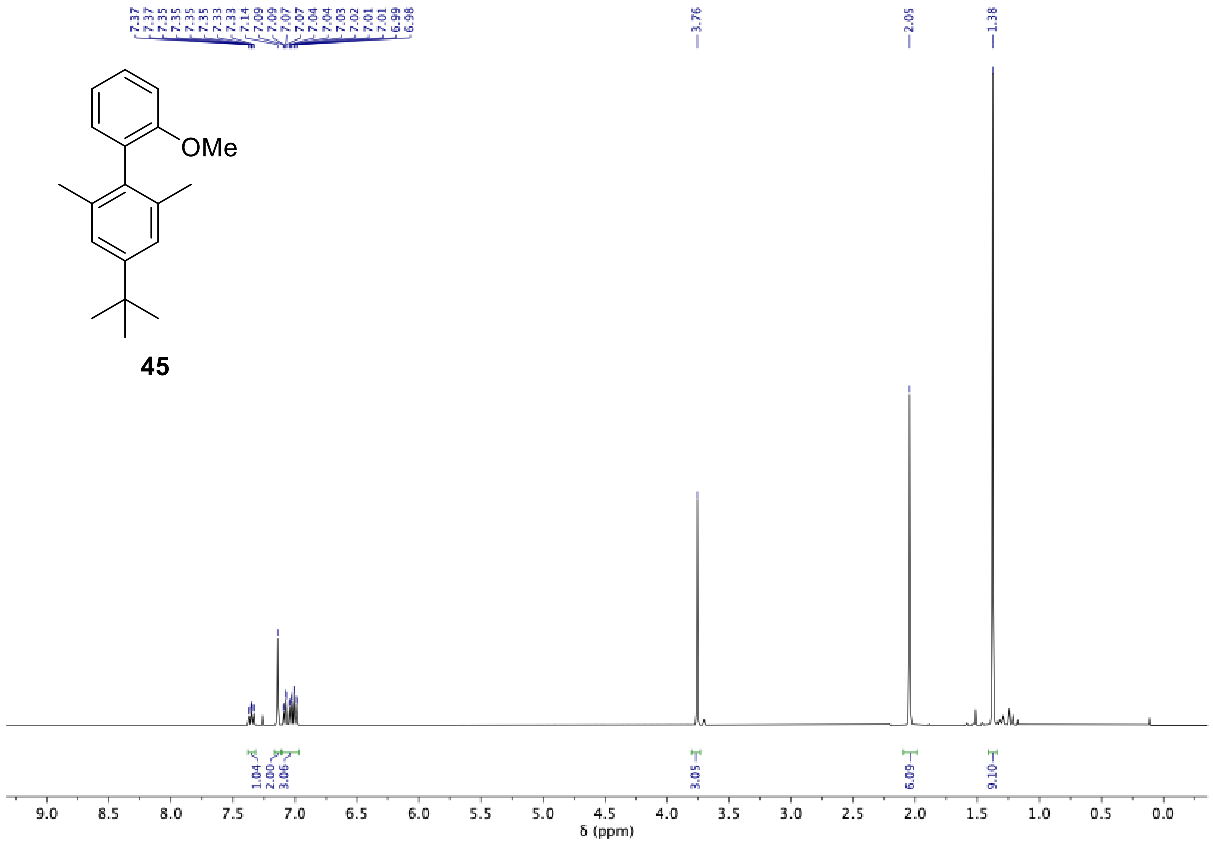
45
7.37
7.37
7.35
7.35
7.35
7.33
7.33
7.14
7.09
7.09
7.07
7.07
7.04
7.04
7.03
7.02
7.01
7.01
6.99
6.98
3.76
2.05
1.38
1.04
2.00
3.06
3.05
6.09
9.10
δ (ppm)

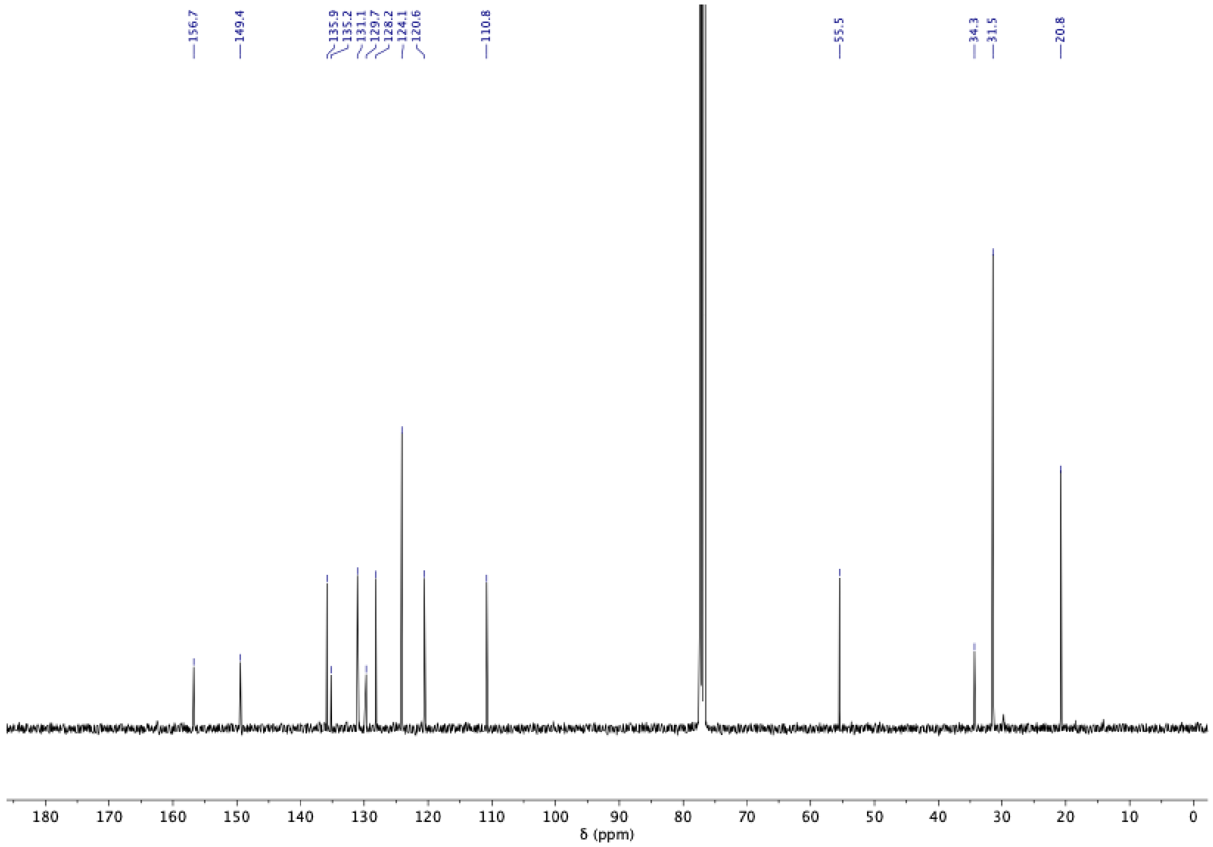
156.7
149.4
135.9
135.2
131.1
129.7
128.2
124.1
120.6
110.8
55.5
34.3
31.5
20.8
δ (ppm)

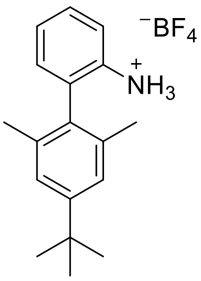

⁻BF4
+
NH3

46

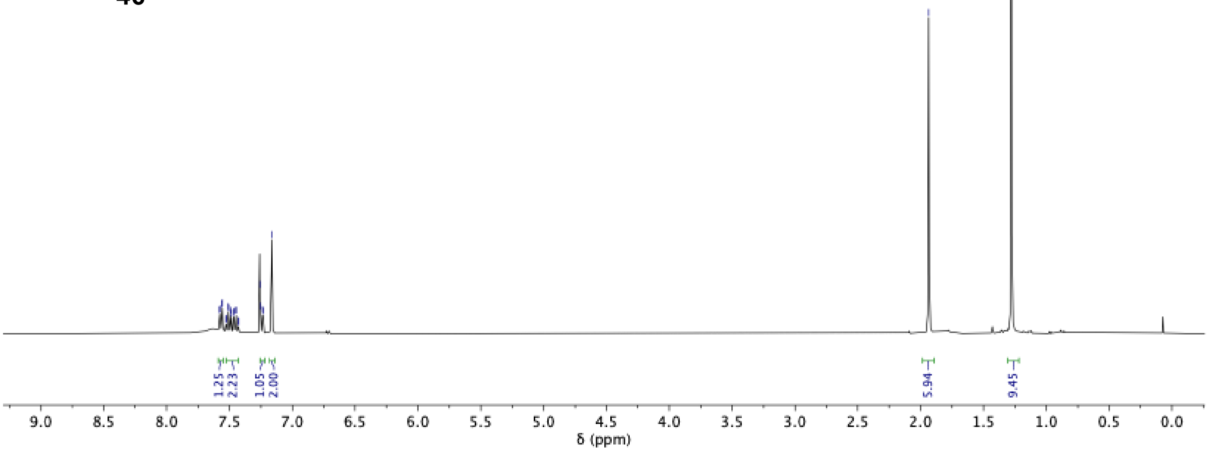

7.58
7.58
7.56
7.56
7.53
7.53
7.51
7.51
7.49
7.49
7.47
7.47
7.45
7.45
7.43
7.43
7.26
7.25
7.24
7.23
7.16
1.94
1.28
1.25
2.23
1.05
2.00
5.94
9.45
9.0
8.5
8.0
7.5
7.0
6.5
6.0
5.5
5.0
4.5
4.0
3.5
3.0
2.5
2.0
1.5
1.0
0.5
0.0
δ (ppm)

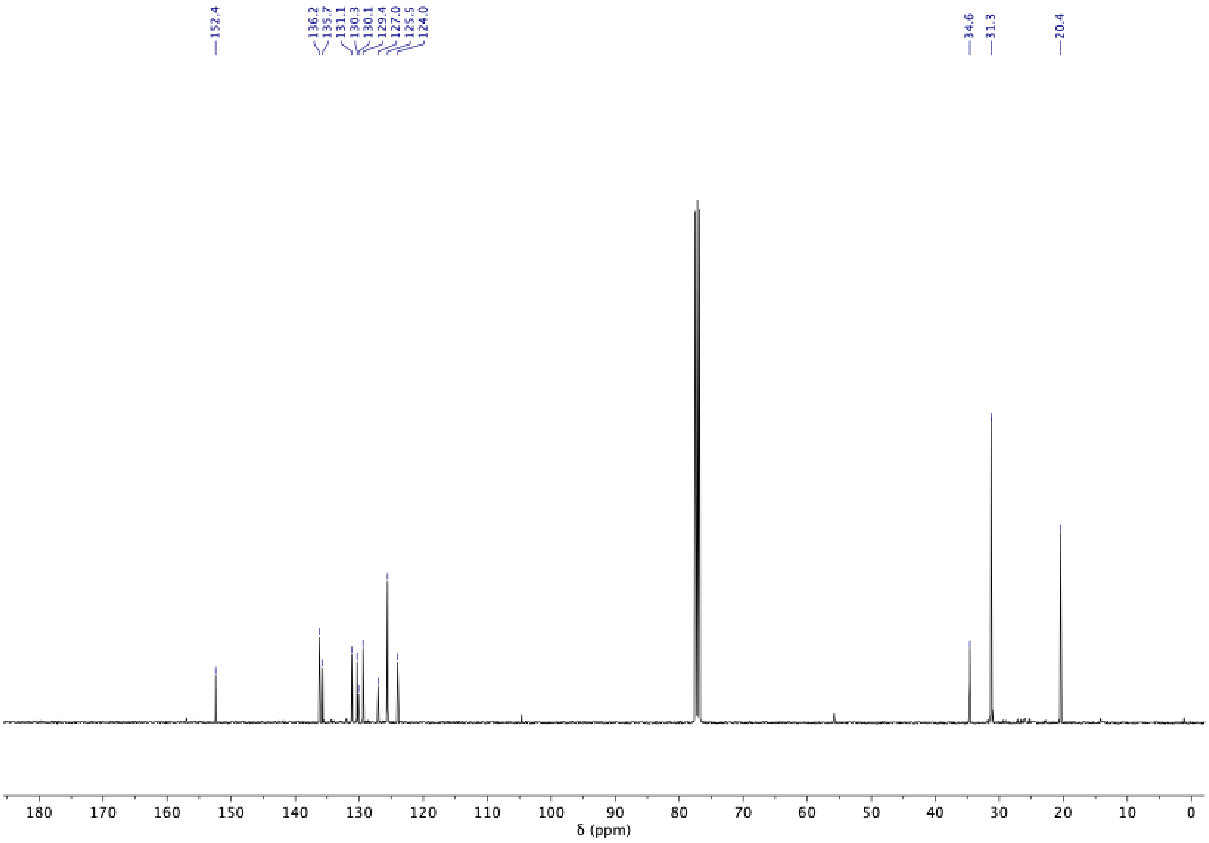

152.4
136.2
135.7
131.1
130.3
130.1
129.4
127.0
125.5
124.0
34.6
31.3
20.4
180
170
160
150
140
130
120
110
100
90
80
70
60
50
40
30
20
10
0
δ (ppm)

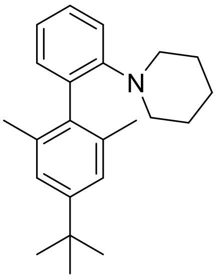

47

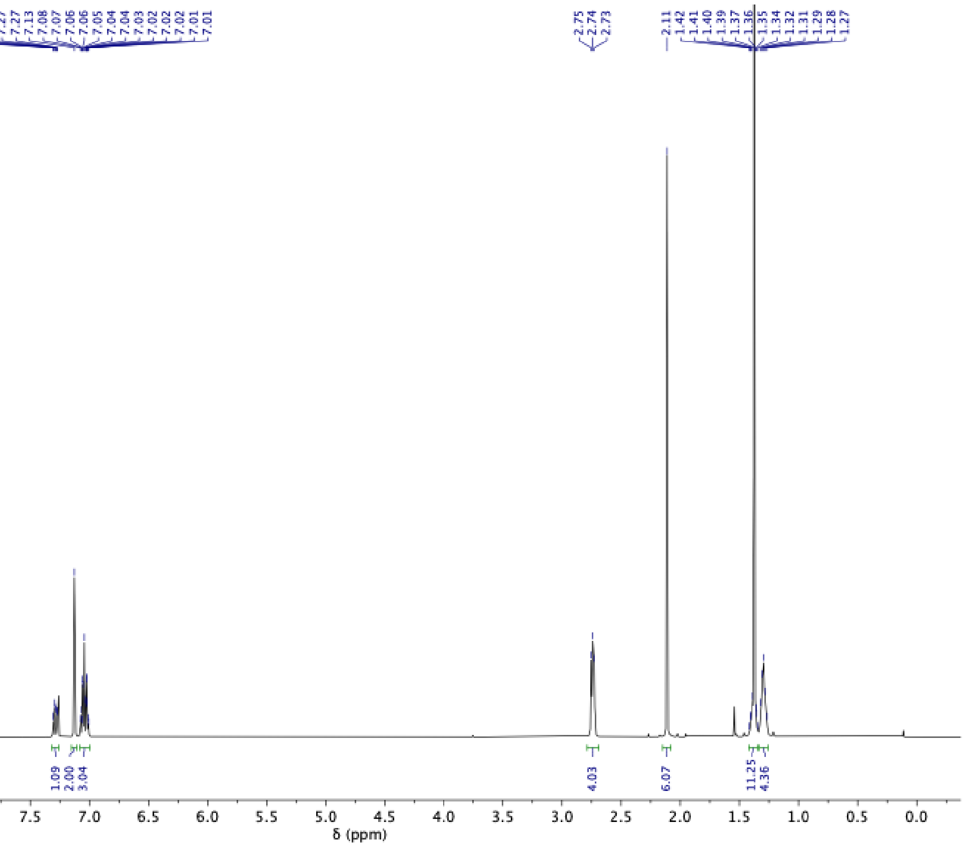
7.31
7.31
7.29
7.29
7.27
7.13
7.08
7.07
7.06
7.06
7.05
7.04
7.04
7.03
7.02
7.02
7.02
7.01
7.01
2.75
2.74
2.73
2.11
1.42
1.41
1.40
1.39
1.37
1.36
1.35
1.34
1.32
1.31
1.29
1.28
1.27
1.09
2.00
3.04
4.03
6.07
11.25
4.36
δ (ppm)

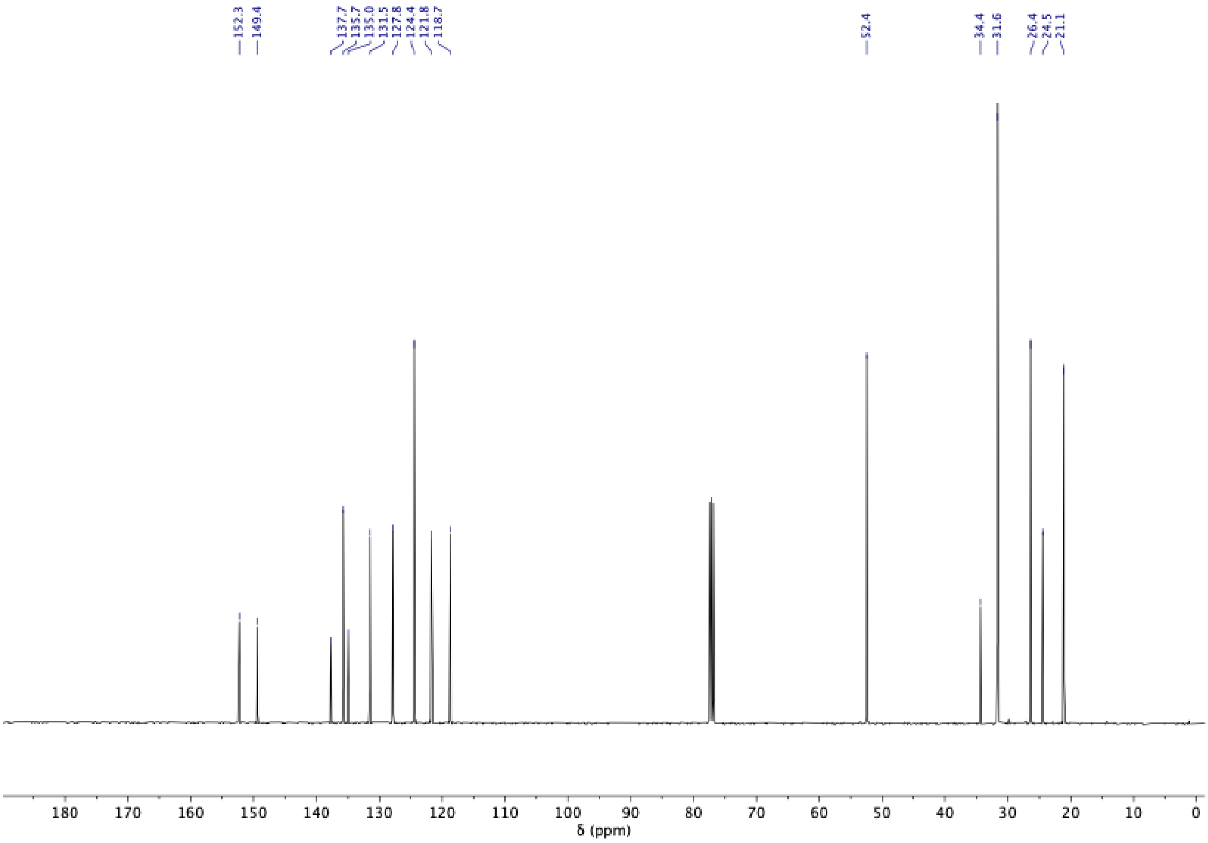
152.3
149.4
137.7
135.7
135.0
131.5
127.8
124.4
121.8
118.7
52.4
34.4
31.6
26.4
24.5
21.1
δ (ppm)

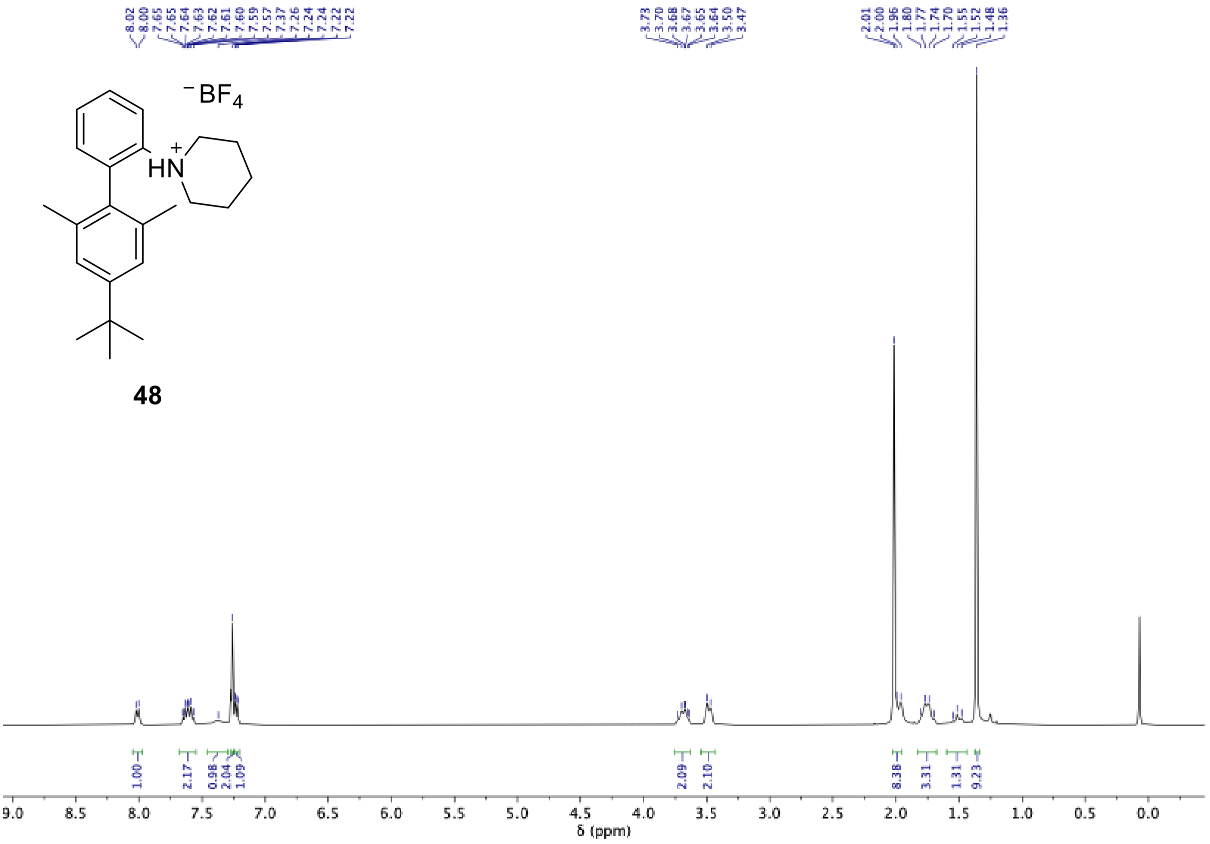
$^{-}BF_4$
HN+
48
8.02
8.00
7.65
7.64
7.63
7.62
7.61
7.60
7.59
7.57
7.37
7.26
7.24
7.24
7.22
7.22
3.73
3.70
3.68
3.67
3.65
3.64
3.50
3.47
2.01
2.00
1.96
1.80
1.77
1.74
1.70
1.55
1.52
1.48
1.36
1.00
2.17
0.98
2.04
1.09
2.09
2.10
8.38
3.31
1.31
9.23
δ (ppm)

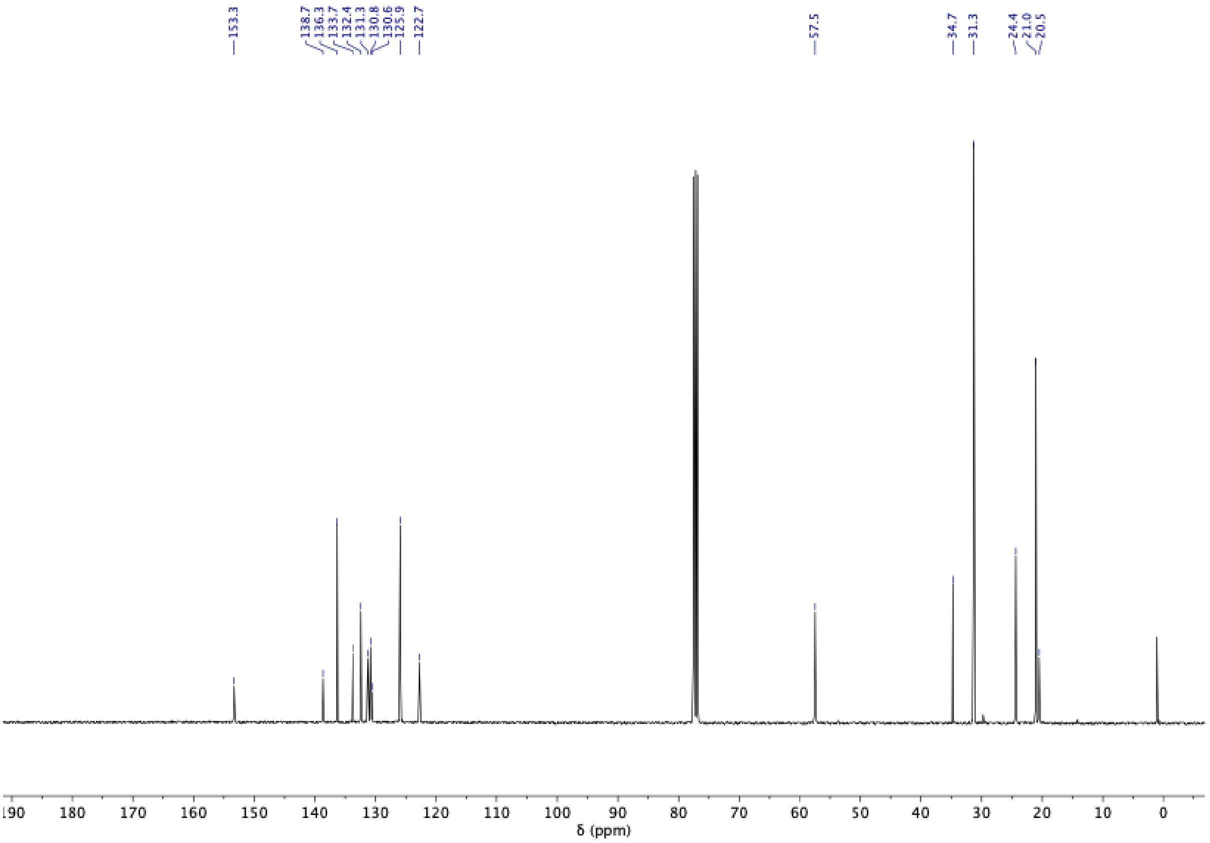
153.3
138.7
136.3
133.7
132.4
131.3
130.8
130.6
125.9
122.7
57.5
34.7
31.3
24.4
21.0
20.5
δ (ppm)

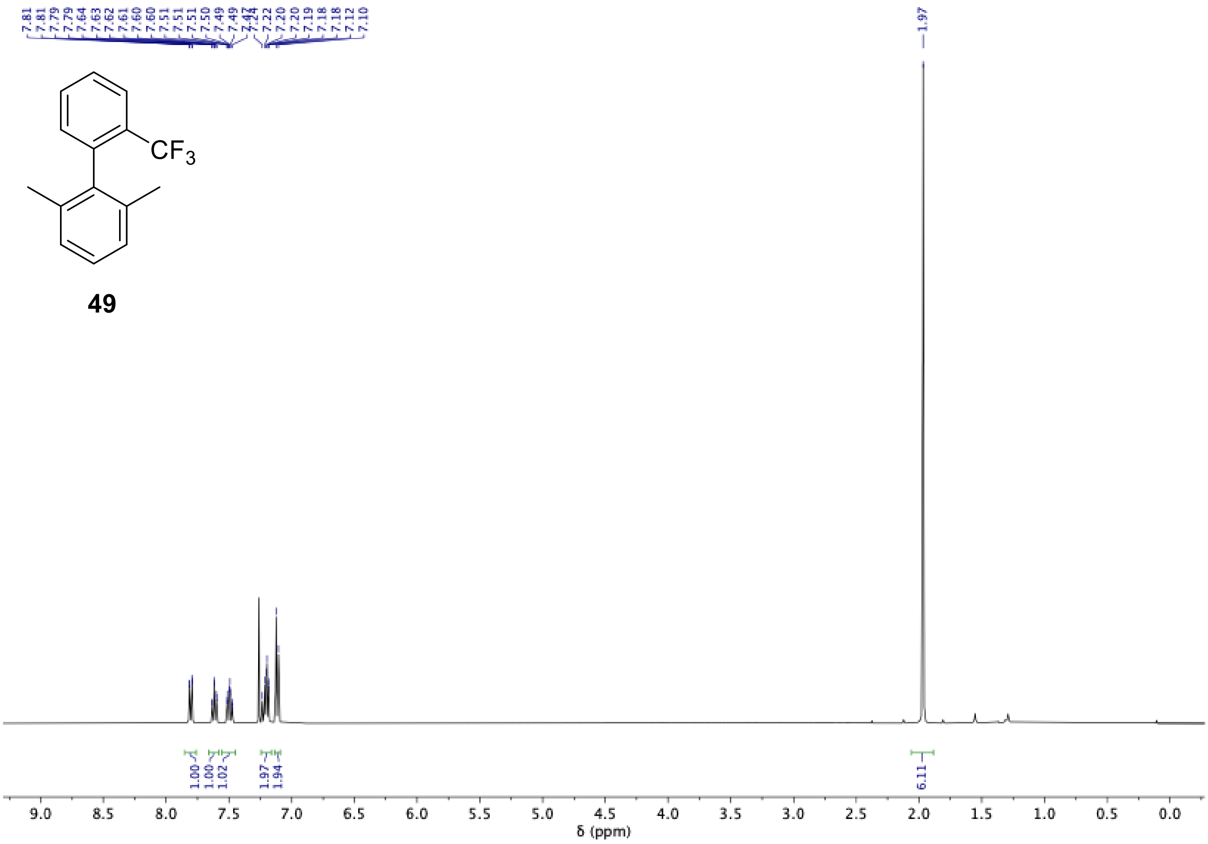
7.81
7.81
7.79
7.64
7.63
7.62
7.61
7.60
7.51
7.51
7.50
7.49
7.49
7.47
7.24
7.22
7.20
7.20
7.19
7.18
7.18
7.12
7.10
1.97
1.00
1.00
1.02
1.97
1.94
6.11
9.0
8.5
8.0
7.5
7.0
6.5
6.0
5.5
5.0
4.5
4.0
3.5
3.0
2.5
2.0
1.5
1.0
0.5
0.0
δ (ppm)
CF3

49

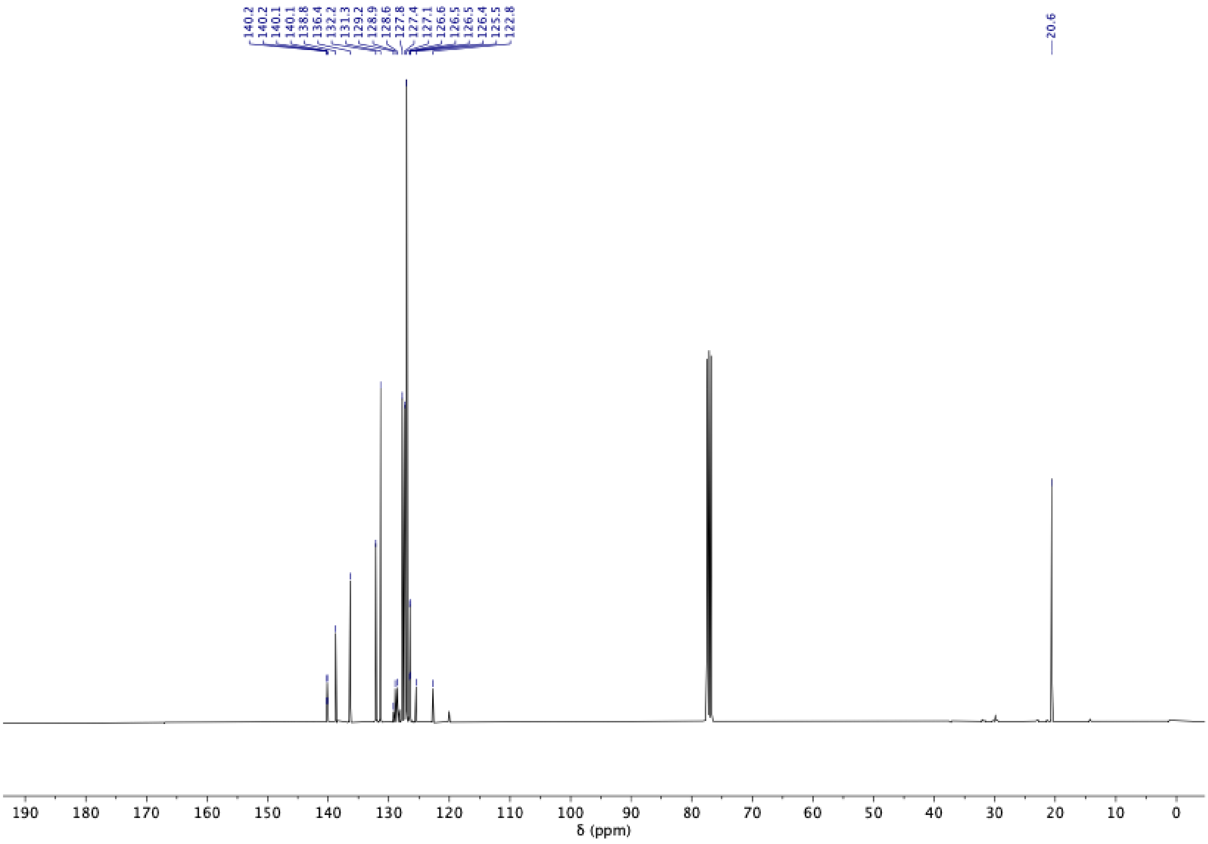
140.2
140.2
140.1
140.1
138.8
136.4
132.2
131.3
129.2
128.9
128.6
127.8
127.4
127.1
126.6
126.5
126.4
125.5
122.8
20.6
190
180
170
160
150
140
130
120
110
100
90
80
70
60
50
40
30
20
10
0
δ (ppm)

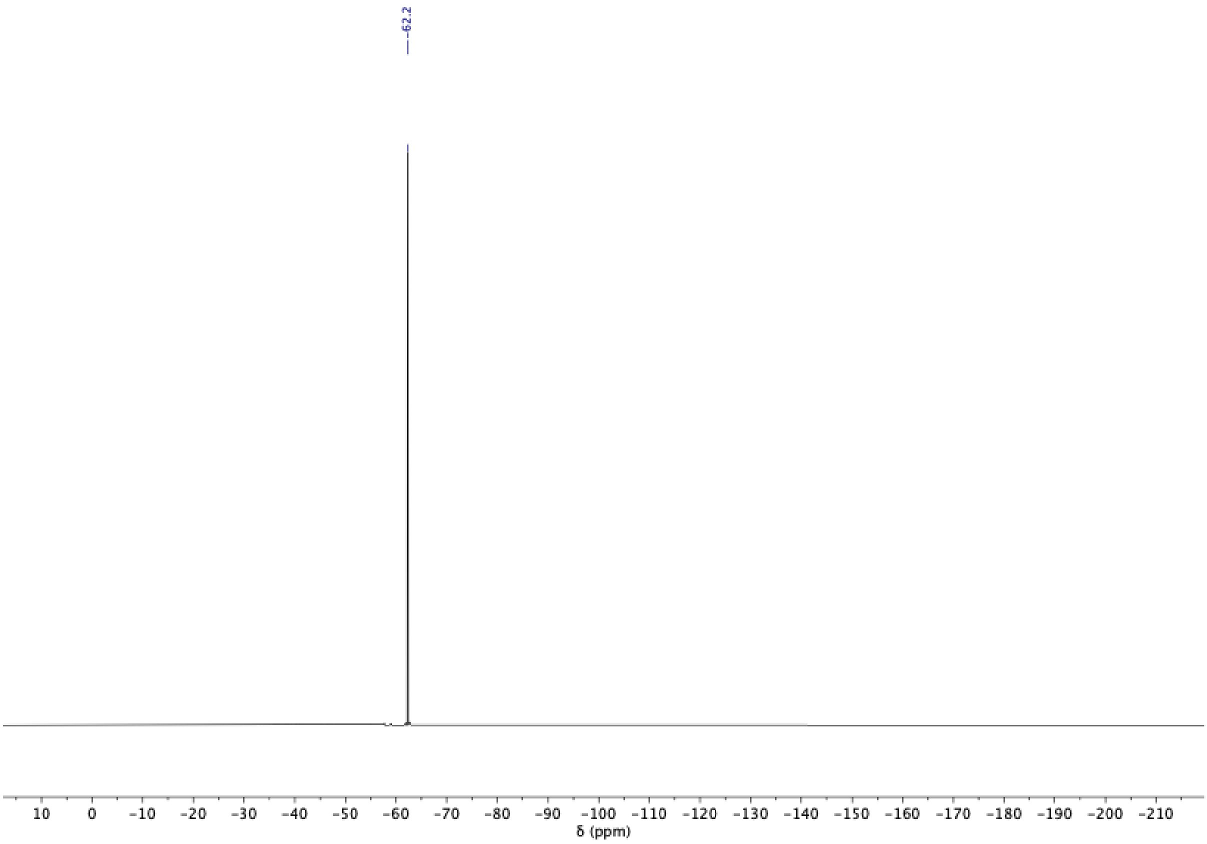

-62.2
10
0
-10
-20
-30
-40
-50
-60
-70
-80
-90
-100
-110
-120
-130
-140
-150
-160
-170
-180
-190
-200
-210
δ (ppm)

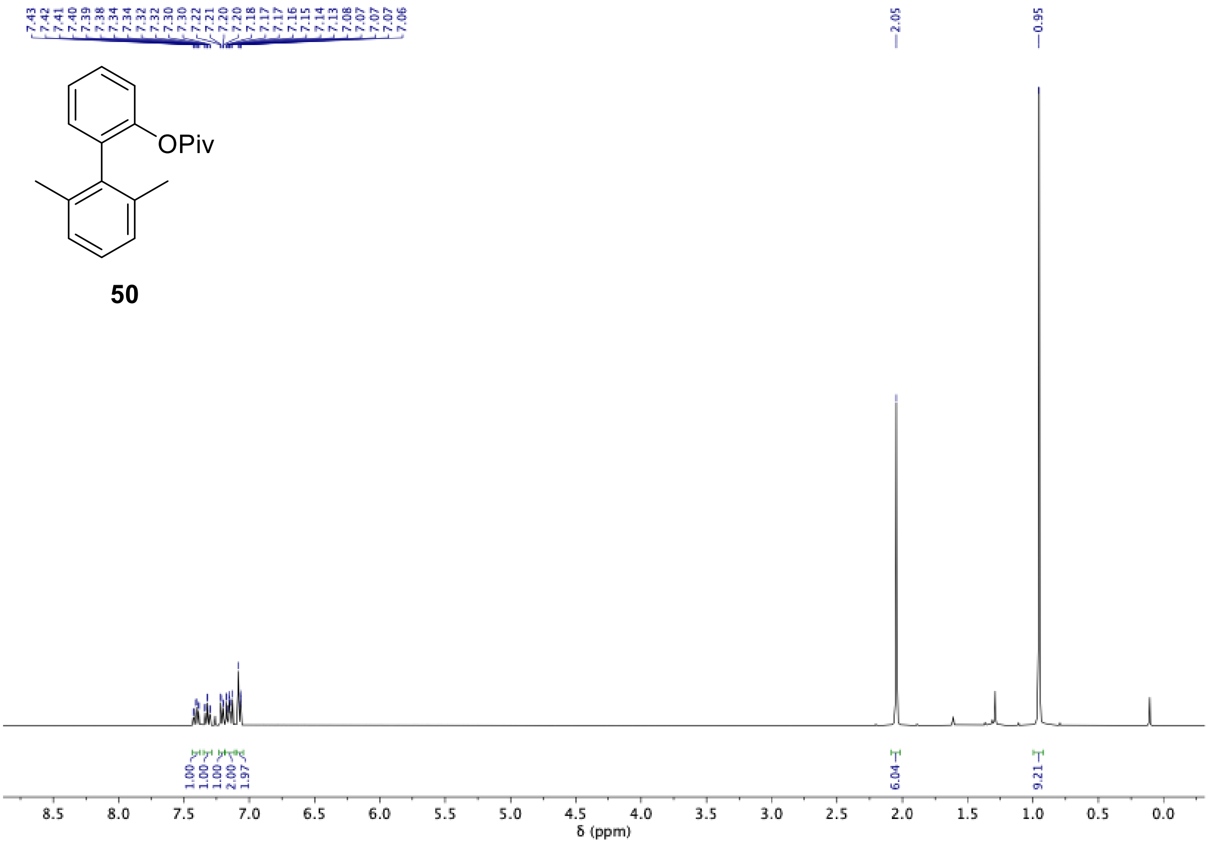
7.43
7.42
7.41
7.40
7.39
7.38
7.34
7.34
7.32
7.32
7.30
7.30
7.22
7.21
7.20
7.20
7.18
7.17
7.17
7.16
7.15
7.14
7.13
7.08
7.07
7.07
7.06
2.05
0.95
OPiv
1.00
1.00
1.00
2.00
1.97
6.04
9.21
8.5
8.0
7.5
7.0
6.5
6.0
5.5
5.0
4.5
4.0
3.5
3.0
2.5
2.0
1.5
1.0
0.5
0.0
δ (ppm)

50

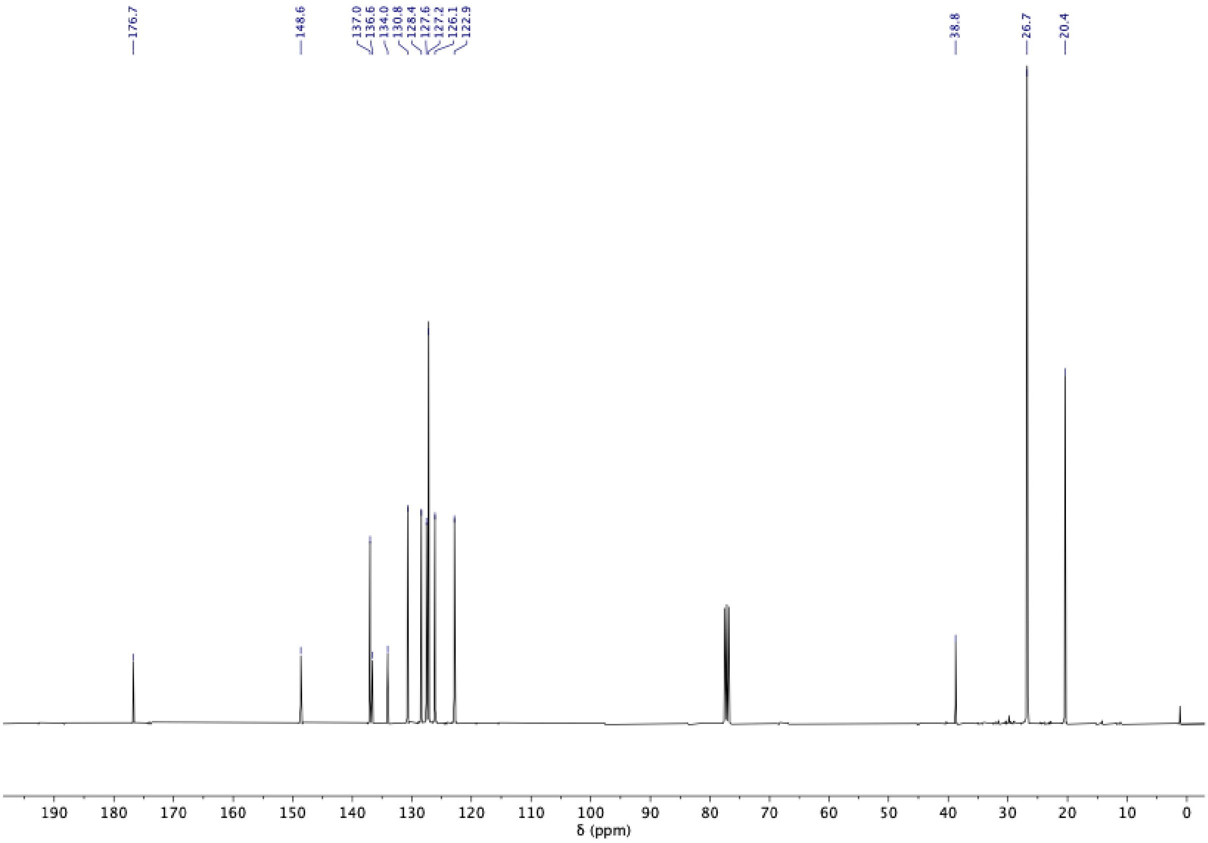
176.7
148.6
137.0
136.6
134.0
130.8
128.4
127.6
127.2
126.1
122.9
38.8
26.7
20.4
190
180
170
160
150
140
130
120
110
100
90
80
70
60
50
40
30
20
10
0
δ (ppm)

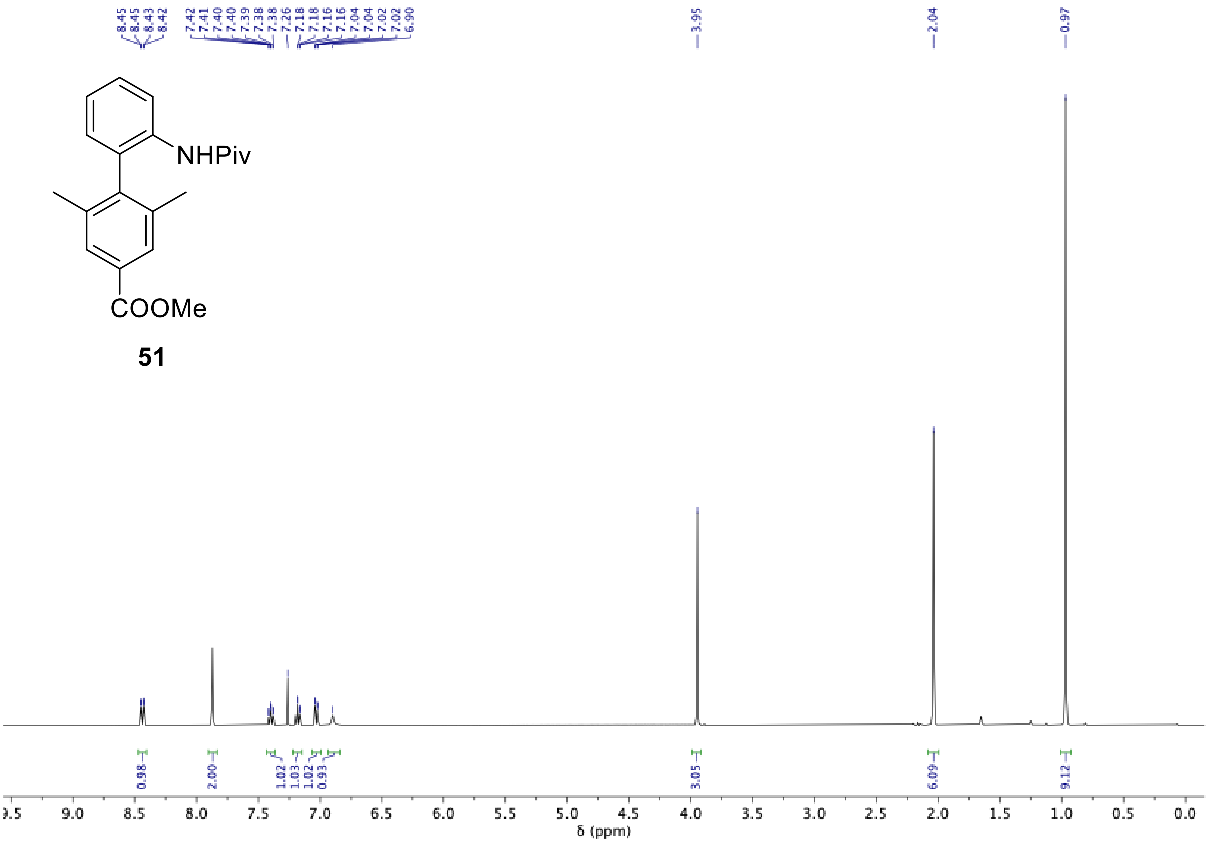
NHPiv
COOMe
51
8.45 8.45 8.43 8.42 7.42 7.41 7.40 7.40 7.39 7.38 7.38 7.26 7.18 7.18 7.16 7.16 7.04 7.04 7.02 7.02 6.90
3.95
2.04
0.97
0.98 2.00 1.02 1.03 1.02 0.93 3.05 6.09 9.12
9.0 8.5 8.0 7.5 7.0 6.5 6.0 5.5 5.0 4.5 4.0 3.5 3.0 2.5 2.0 1.5 1.0 0.5 0.0
δ (ppm)

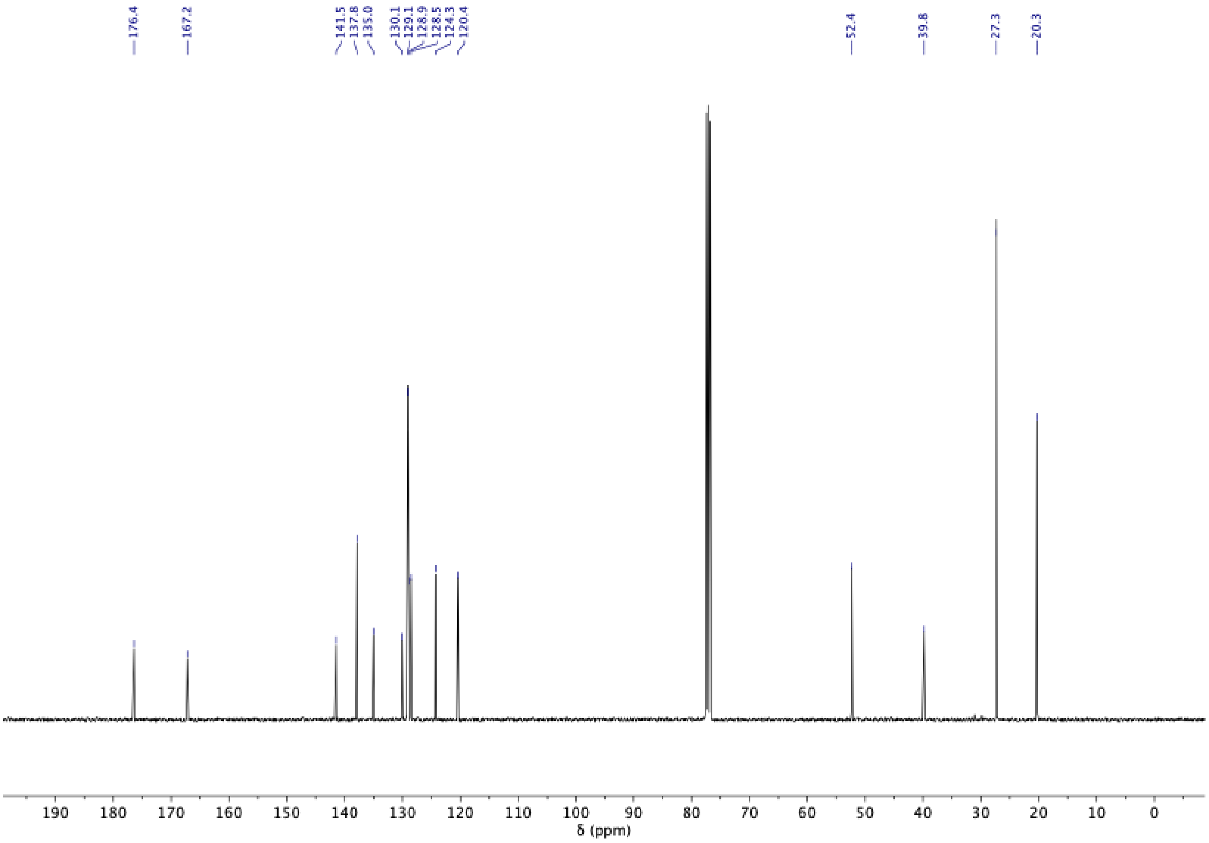
176.4
167.2
141.5 137.8 135.0 130.1 129.1 128.9 128.5 124.3 120.4
52.4
39.8
27.3
20.3
190 180 170 160 150 140 130 120 110 100 90 80 70 60 50 40 30 20 10 0
δ (ppm)

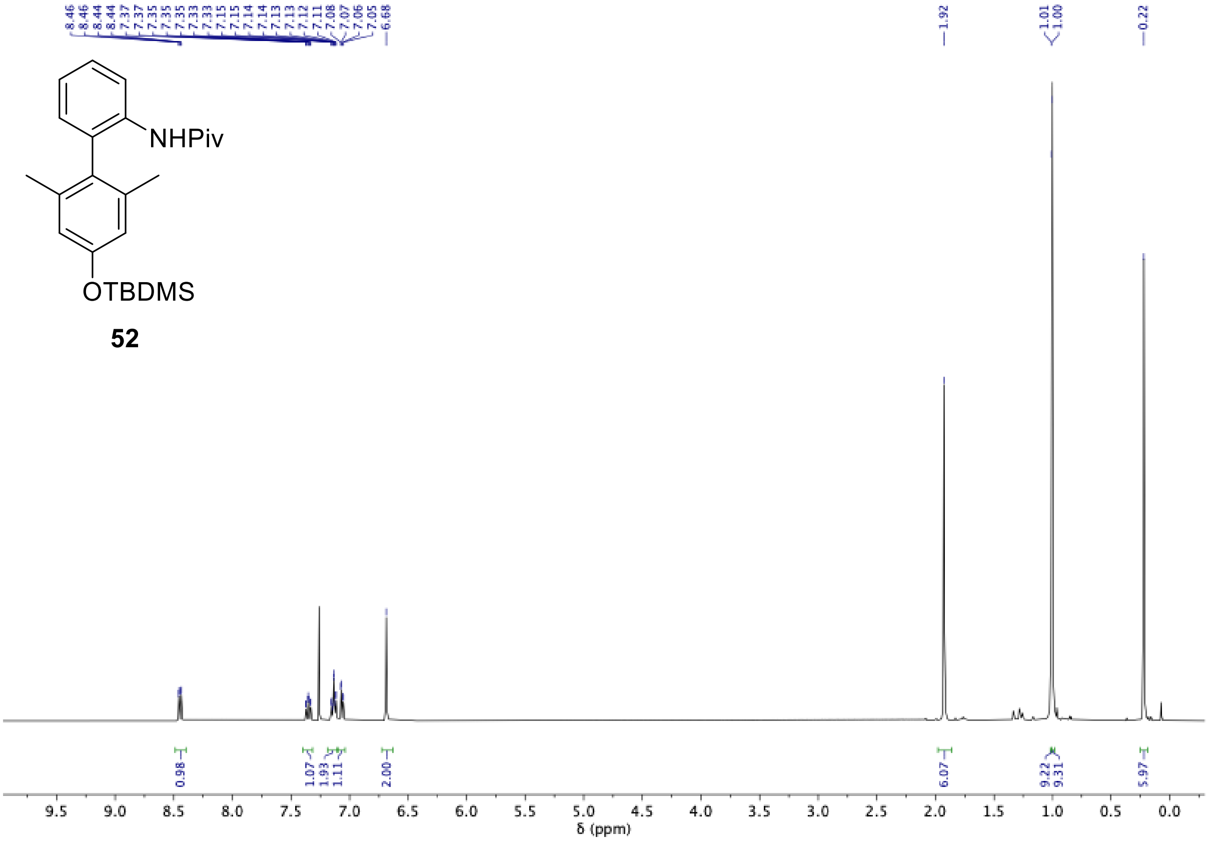
8.46 8.46 8.44 8.44 7.37 7.37 7.35 7.35 7.35 7.33 7.33 7.15 7.15 7.14 7.14 7.13 7.13 7.12 7.11 7.08 7.07 7.06 7.05 6.68
1.92
1.01 1.00
0.22
NHPiv
OTBDMS
0.98
1.07 1.93 1.11
2.00
6.07
9.22 9.31
5.97
9.5 9.0 8.5 8.0 7.5 7.0 6.5 6.0 5.5 5.0 4.5 4.0 3.5 3.0 2.5 2.0 1.5 1.0 0.5 0.0
δ (ppm)

52

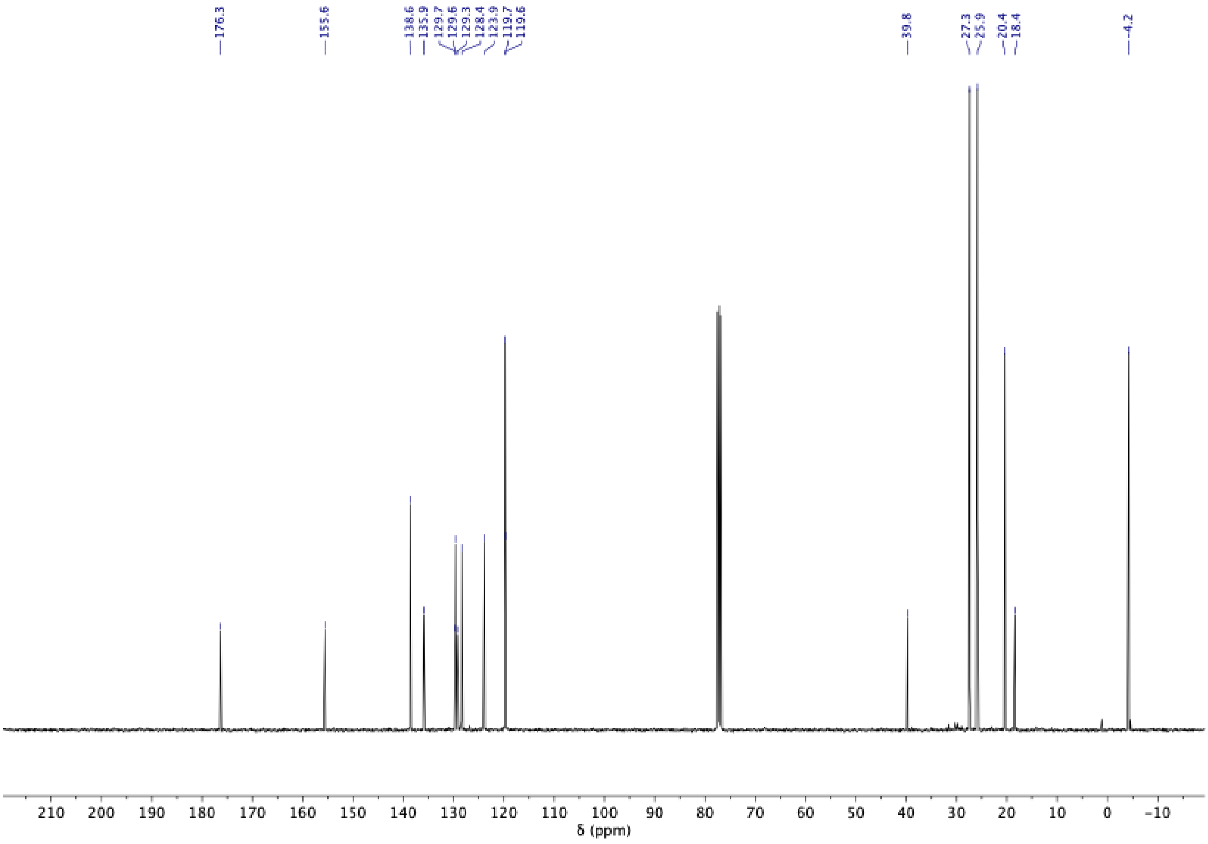
176.3
155.6
138.6 135.9 129.7 129.6 129.3 128.4 123.9 119.7 119.6
39.8
27.3 25.9
20.4 18.4
-4.2
210 200 190 180 170 160 150 140 130 120 110 100 90 80 70 60 50 40 30 20 10 0 -10
δ (ppm)

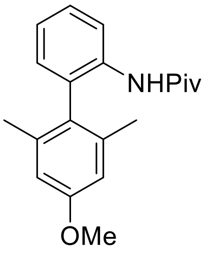
NHPiv
OMe

53

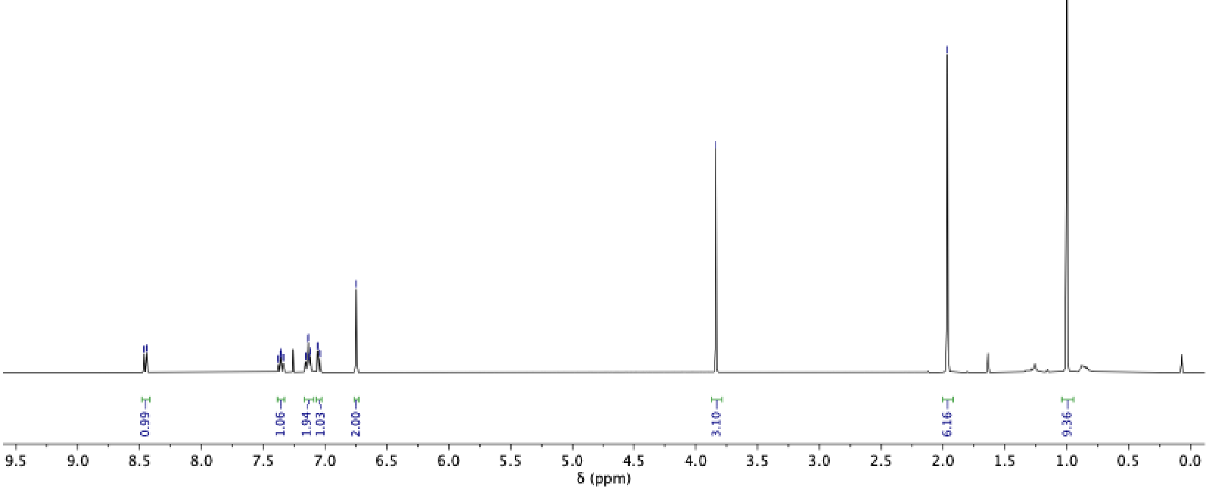
8.47
8.46
8.44
8.44
7.38
7.36
7.36
7.36
7.34
7.34
7.16
7.15
7.14
7.14
7.13
7.12
7.12
7.06
7.06
7.04
7.04
6.75
3.84
1.97
1.00
0.99
1.06
1.94
1.03
2.00
3.10
6.16
9.36
δ (ppm)

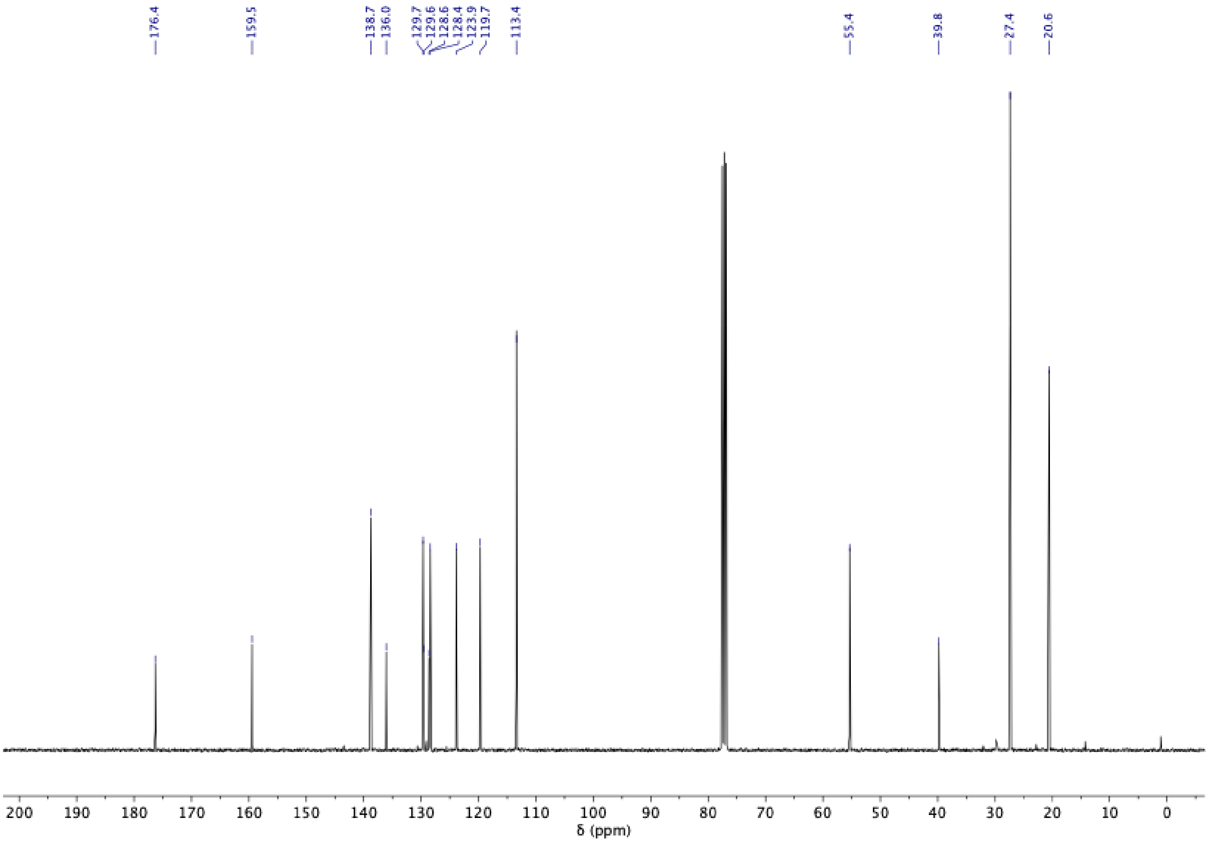
176.4
159.5
138.7
136.0
129.7
129.6
128.6
128.4
123.9
119.7
113.4
55.4
39.8
27.4
20.6
δ (ppm)

11.3. NMR spectra of the isolated products

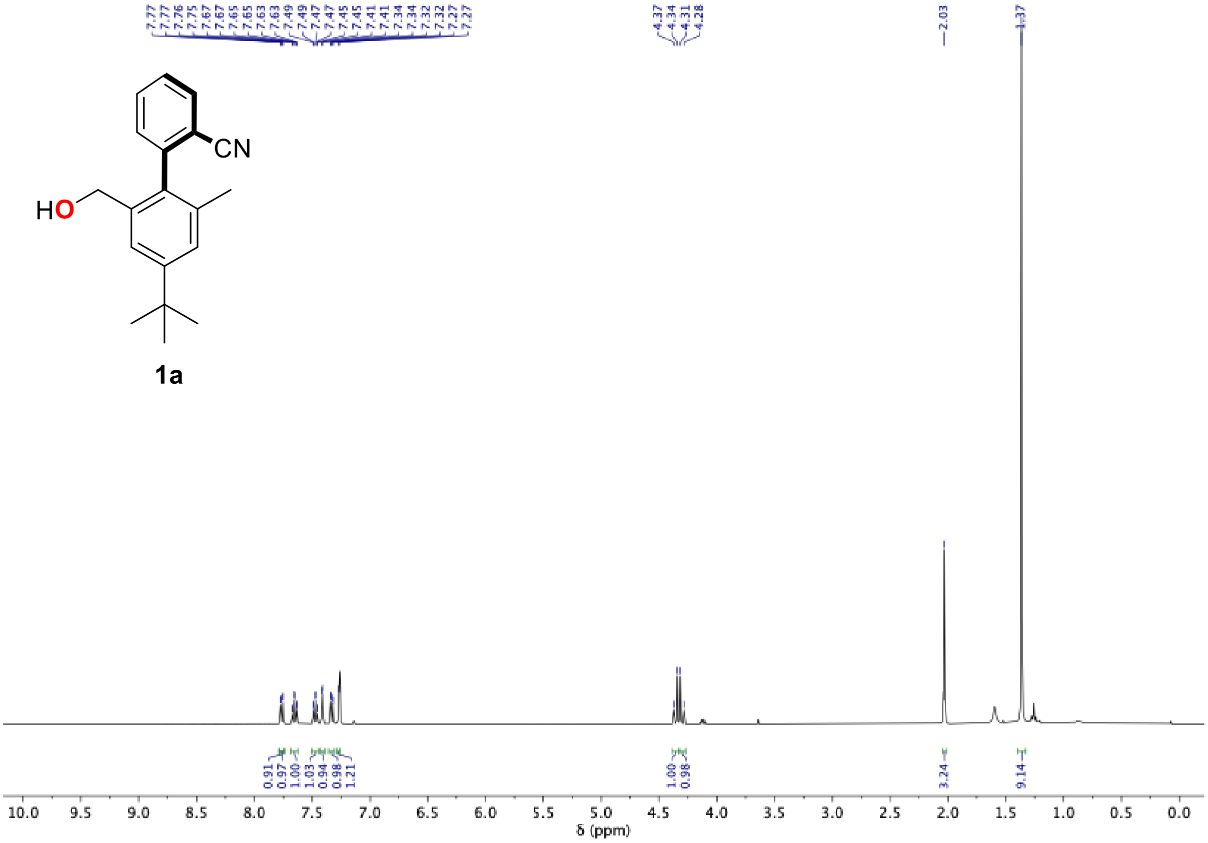

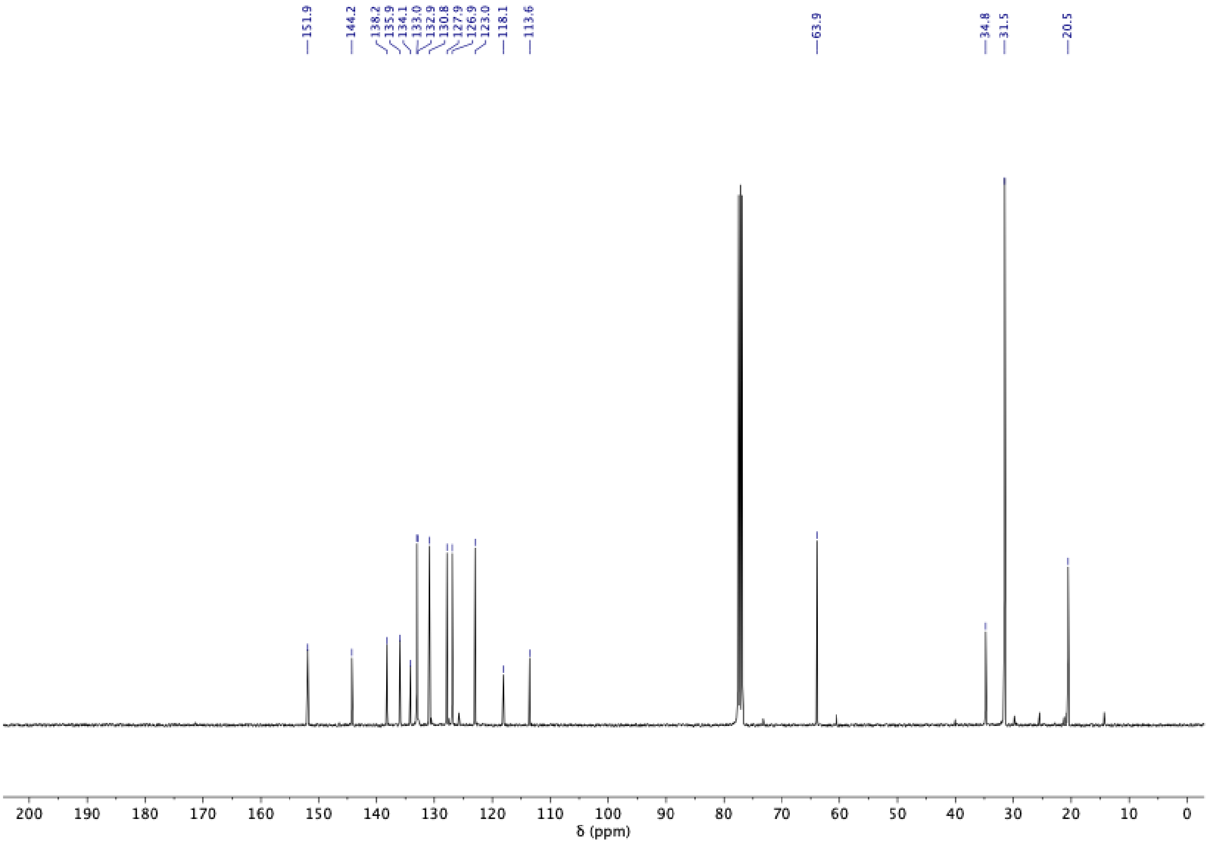

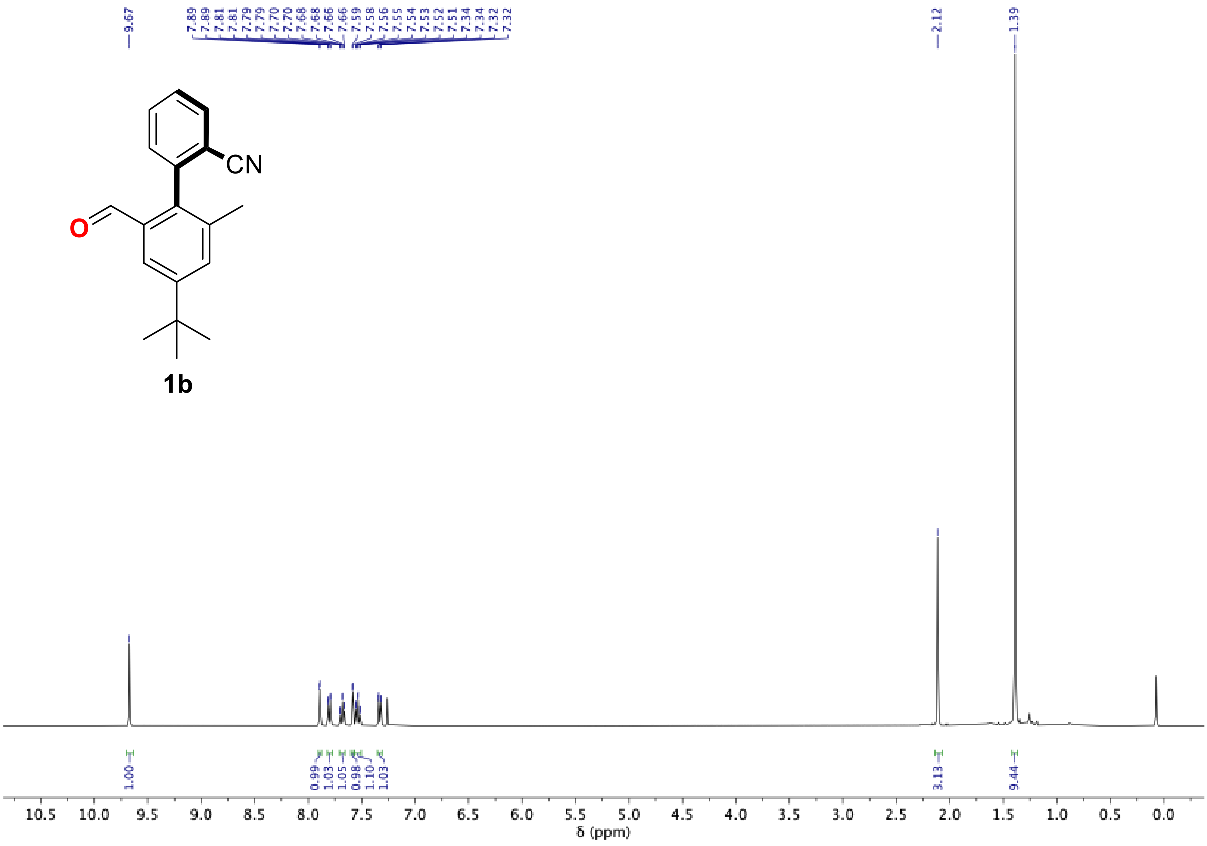
CN
O
9.67
7.89
7.89
7.81
7.81
7.79
7.79
7.70
7.70
7.68
7.68
7.66
7.66
7.59
7.58
7.56
7.55
7.54
7.53
7.52
7.51
7.34
7.34
7.32
7.32
2.12
1.39
1.00
0.99
1.03
1.05
0.98
1.10
1.03
3.13
9.44
10.5
10.0
9.5
9.0
8.5
8.0
7.5
7.0
6.5
6.0
5.5
5.0
4.5
4.0
3.5
3.0
2.5
2.0
1.5
1.0
0.5
0.0
δ (ppm)

1b

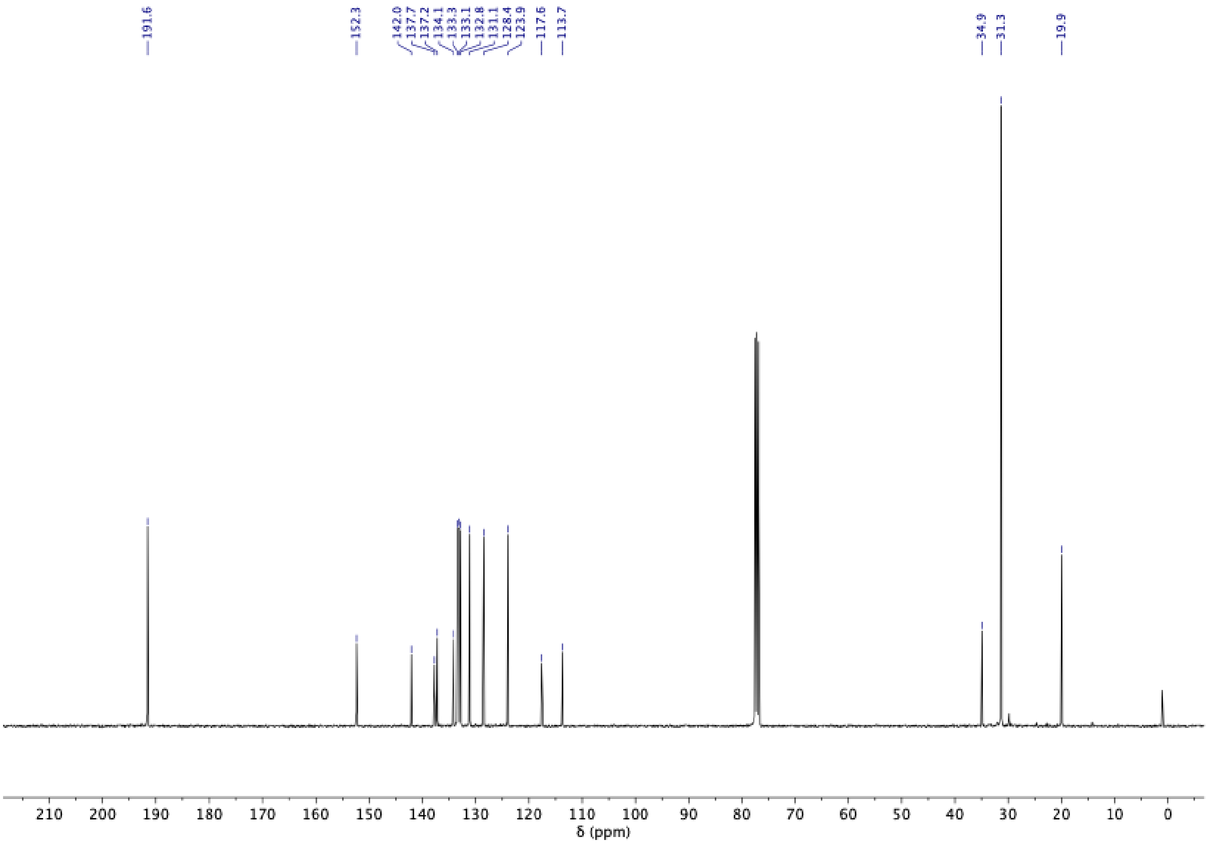
191.6
152.3
142.0
137.7
137.2
134.1
133.3
133.1
132.8
131.1
128.4
123.9
117.6
113.7
34.9
31.3
19.9
210
200
190
180
170
160
150
140
130
120
110
100
90
80
70
60
50
40
30
20
10
0
δ (ppm)

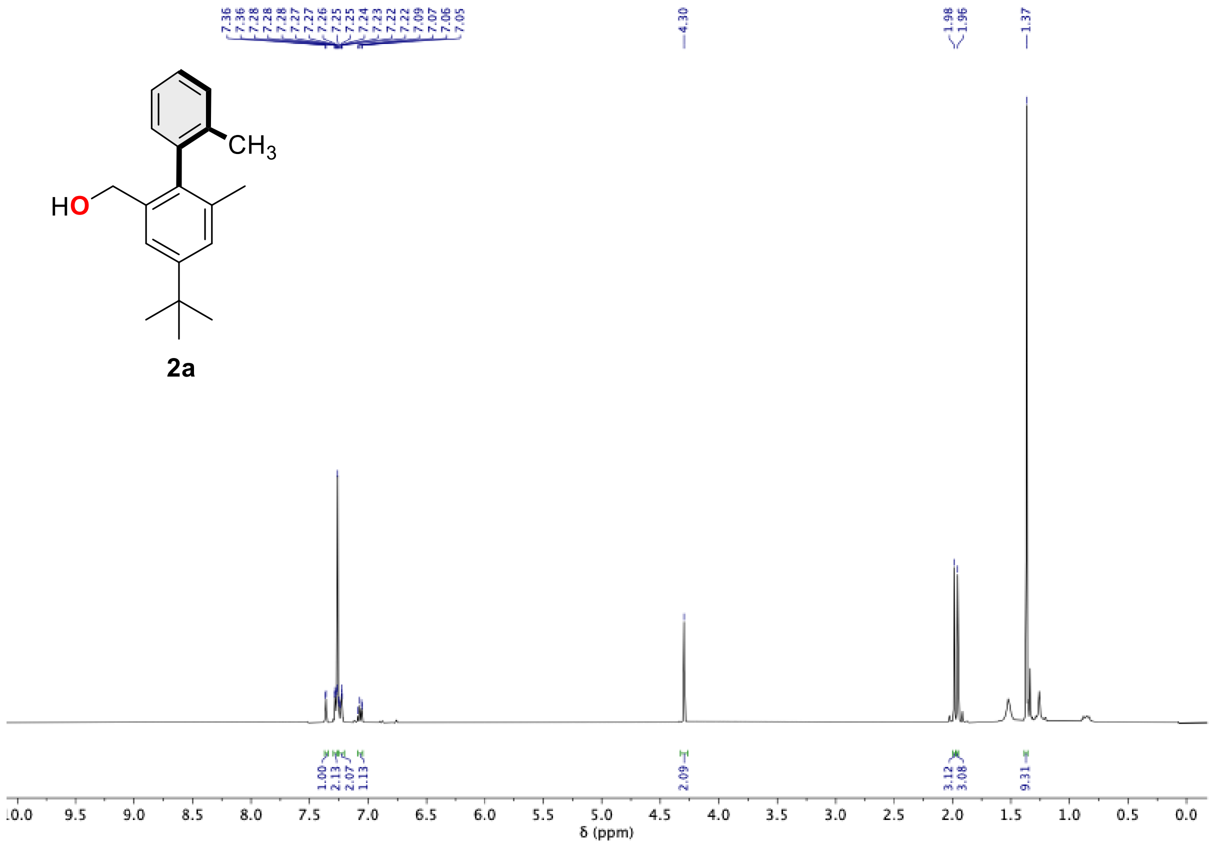
HO
CH3
2a
7.36
7.36
7.28
7.28
7.28
7.27
7.27
7.26
7.25
7.25
7.24
7.23
7.22
7.22
7.09
7.07
7.06
7.05
4.30
1.98
1.96
1.37
1.00
2.13
2.07
1.13
2.09
3.12
3.08
9.31
δ (ppm)

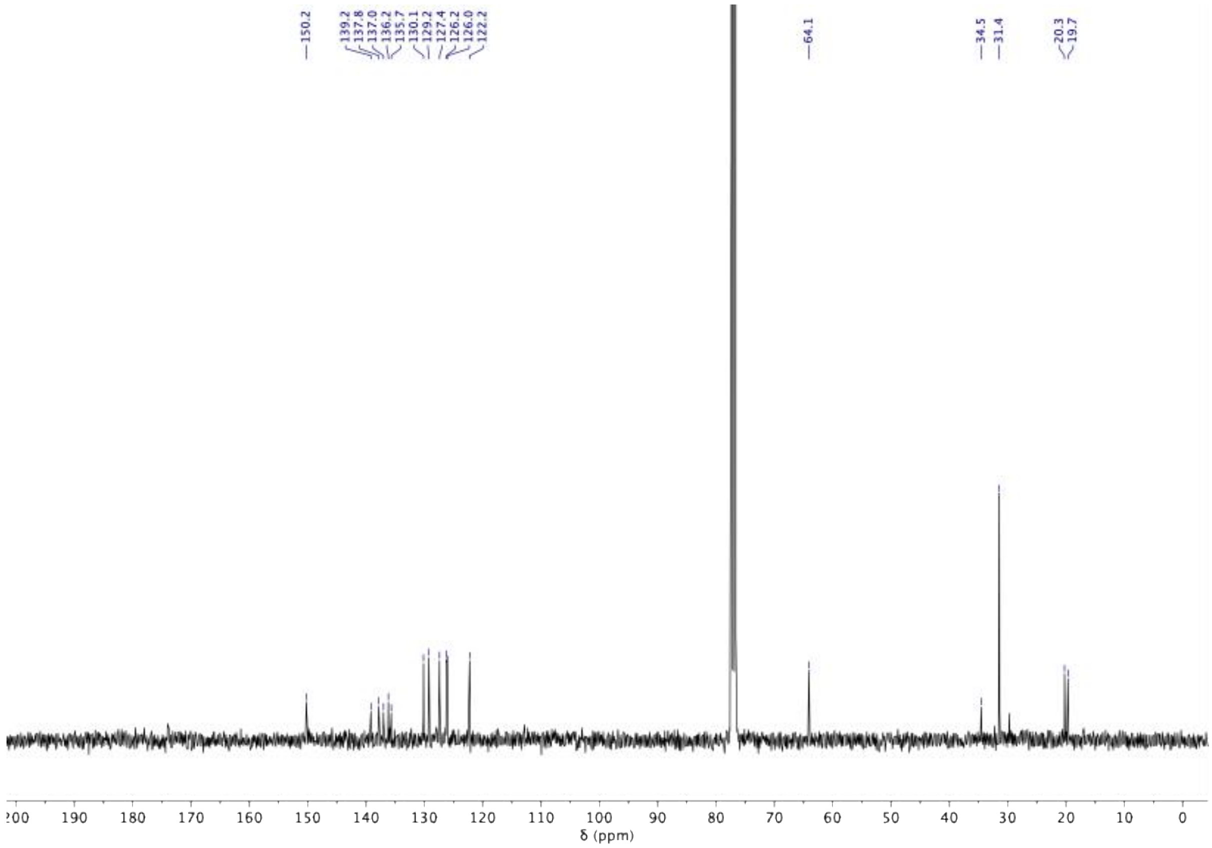
150.2
139.2
137.8
137.0
136.2
135.7
130.1
129.2
127.4
126.2
126.0
122.2
64.1
34.5
31.4
20.3
19.7
δ (ppm)

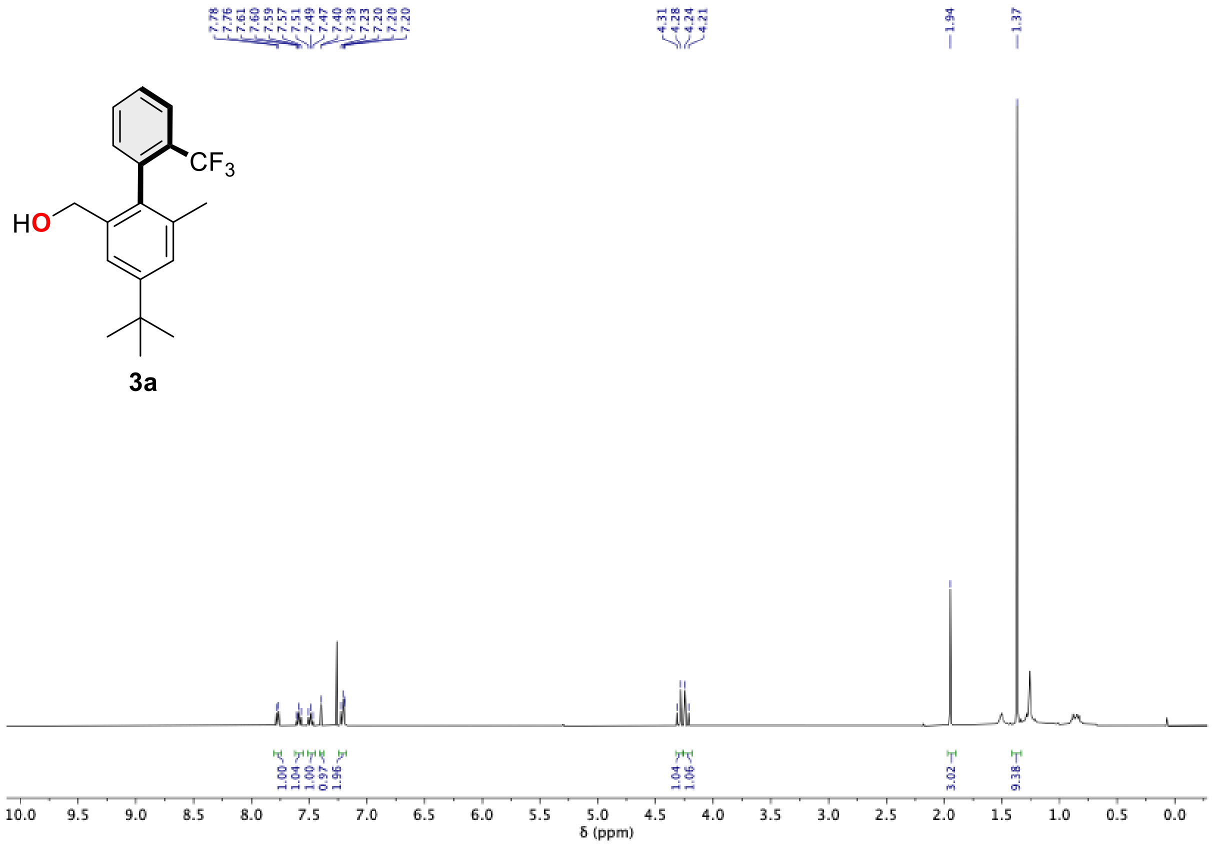
CF3
HO
3a
7.78 7.76 7.61 7.60 7.59 7.57 7.51 7.49 7.47 7.40 7.39 7.23 7.20 7.20 7.20
4.31 4.28 4.24 4.21
1.94
1.37
1.00 1.04 1.00 0.97 1.96
1.04 1.06
3.02
9.38
δ (ppm)

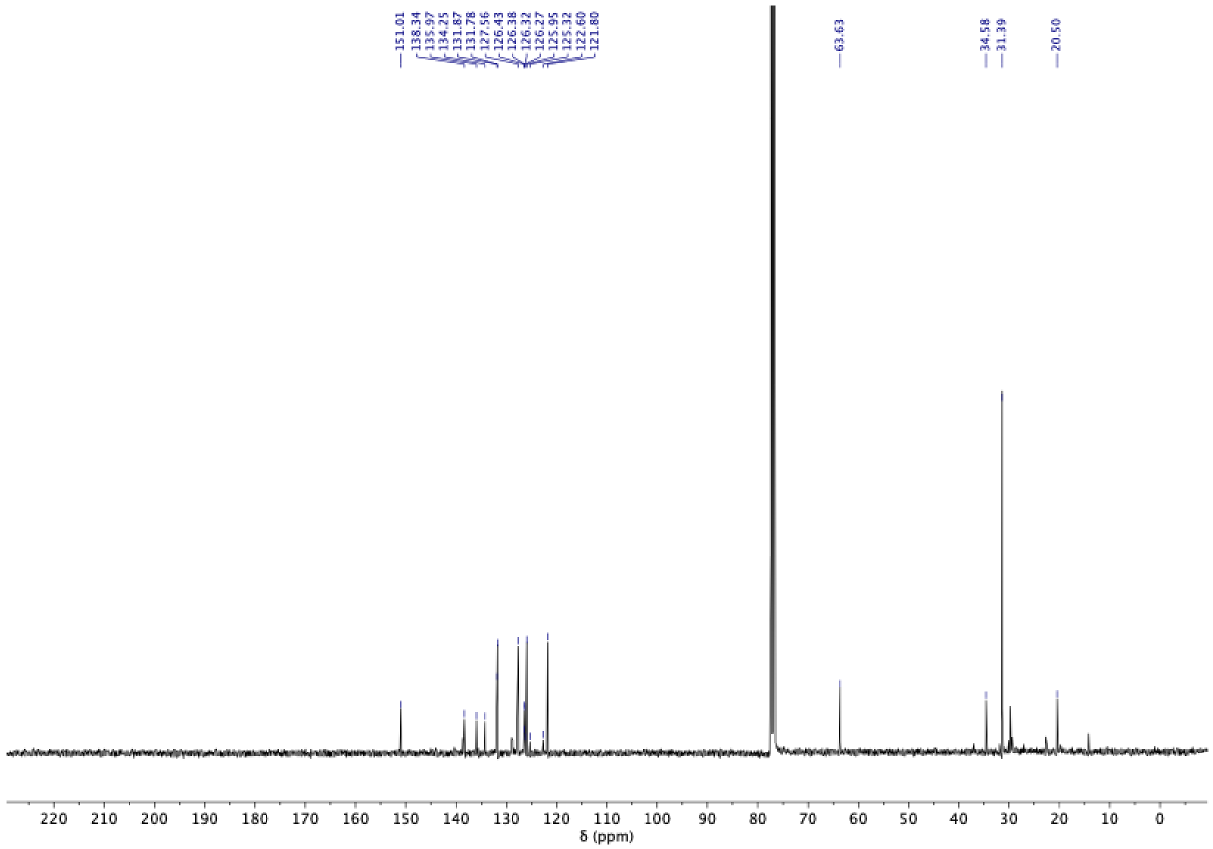
151.01 138.34 135.97 134.25 131.87 131.78 127.56 126.43 126.38 126.32 126.27 125.95 125.32 122.60 121.80
63.63
34.58 31.39
20.50
δ (ppm)

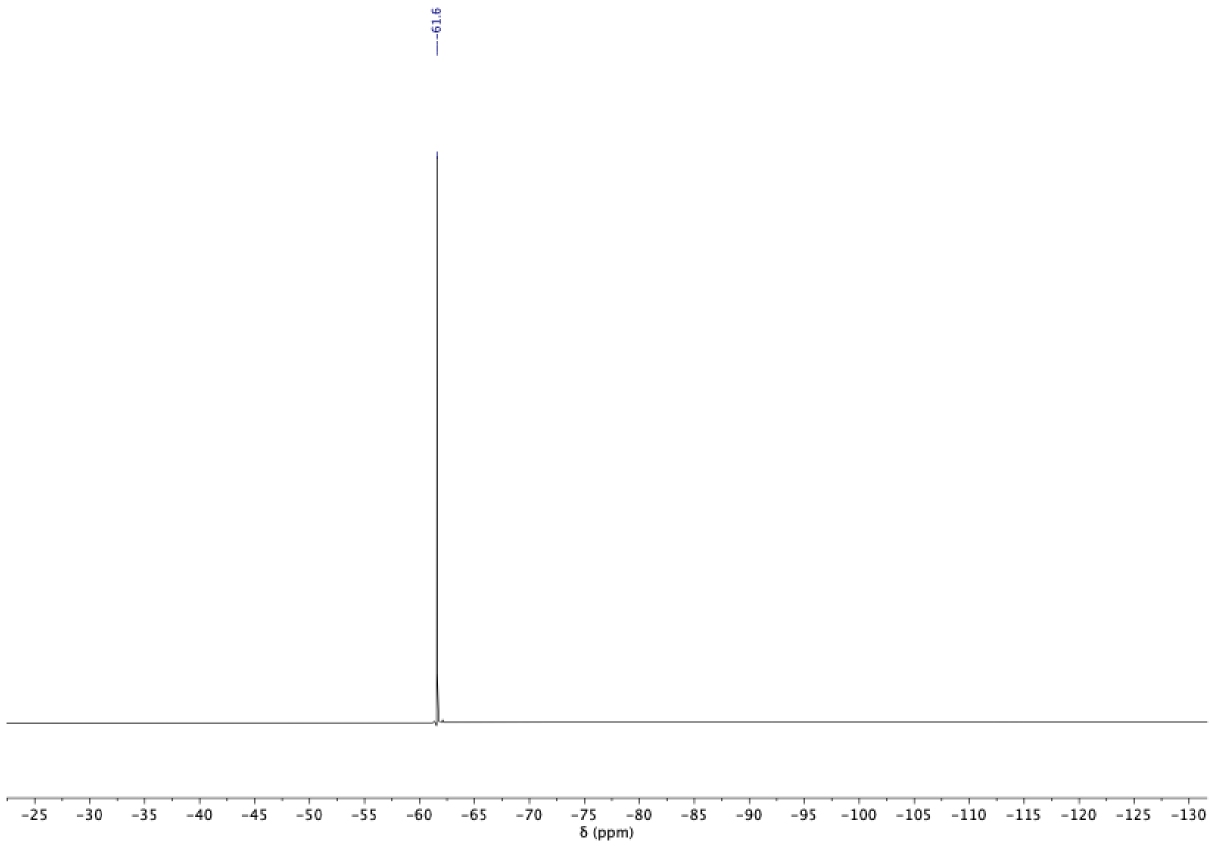

-61.6
-25
-30
-35
-40
-45
-50
-55
-60
-65
-70
-75
-80
-85
-90
-95
-100
-105
-110
-115
-120
-125
-130
δ (ppm)

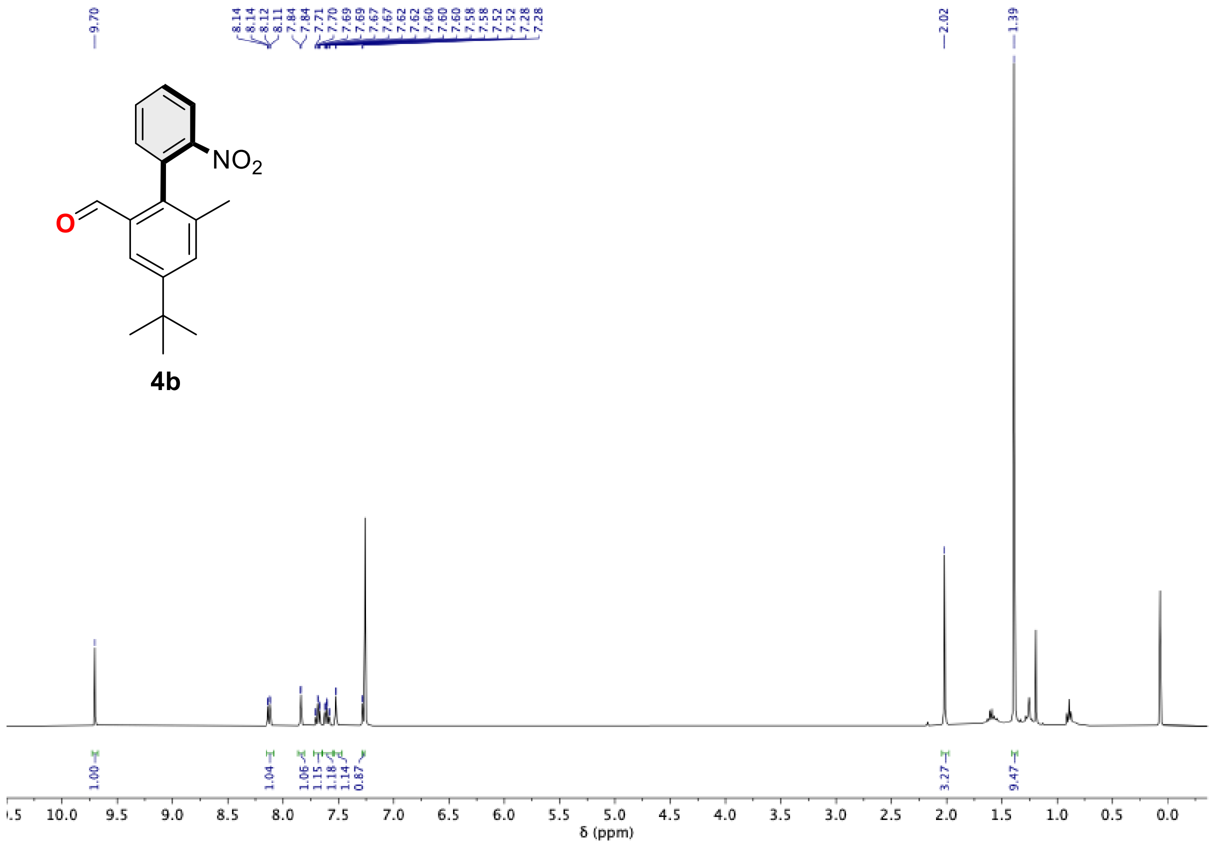
NO2
O

4b

9.70
8.14
8.14
8.12
8.11
7.84
7.84
7.71
7.70
7.69
7.69
7.67
7.67
7.62
7.62
7.60
7.60
7.60
7.58
7.58
7.52
7.52
7.28
7.28
2.02
1.39
1.00
1.04
1.06
1.15
1.18
1.14
0.87
3.27
9.47
δ (ppm)

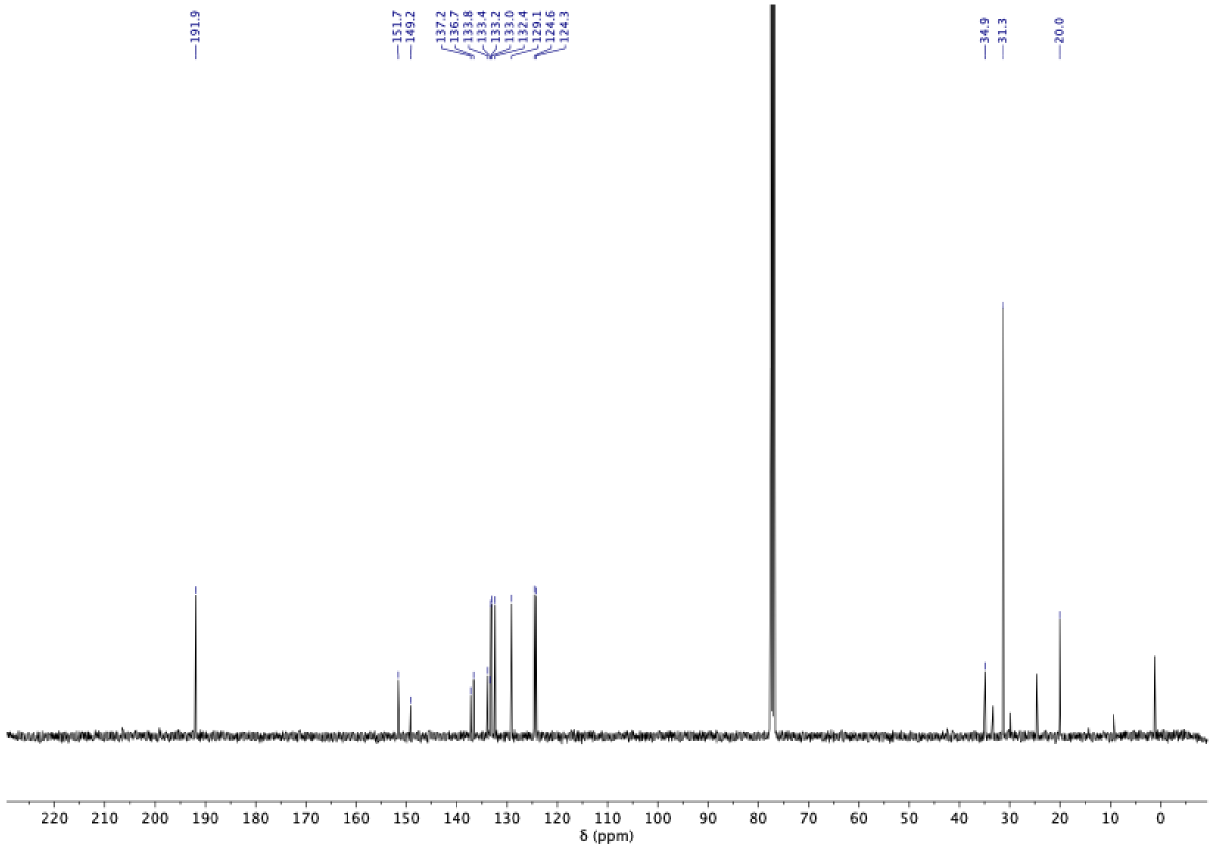
191.9
151.7
149.2
137.2
136.7
133.8
133.4
133.2
133.0
132.4
129.1
124.6
124.3
34.9
31.3
20.0
δ (ppm)

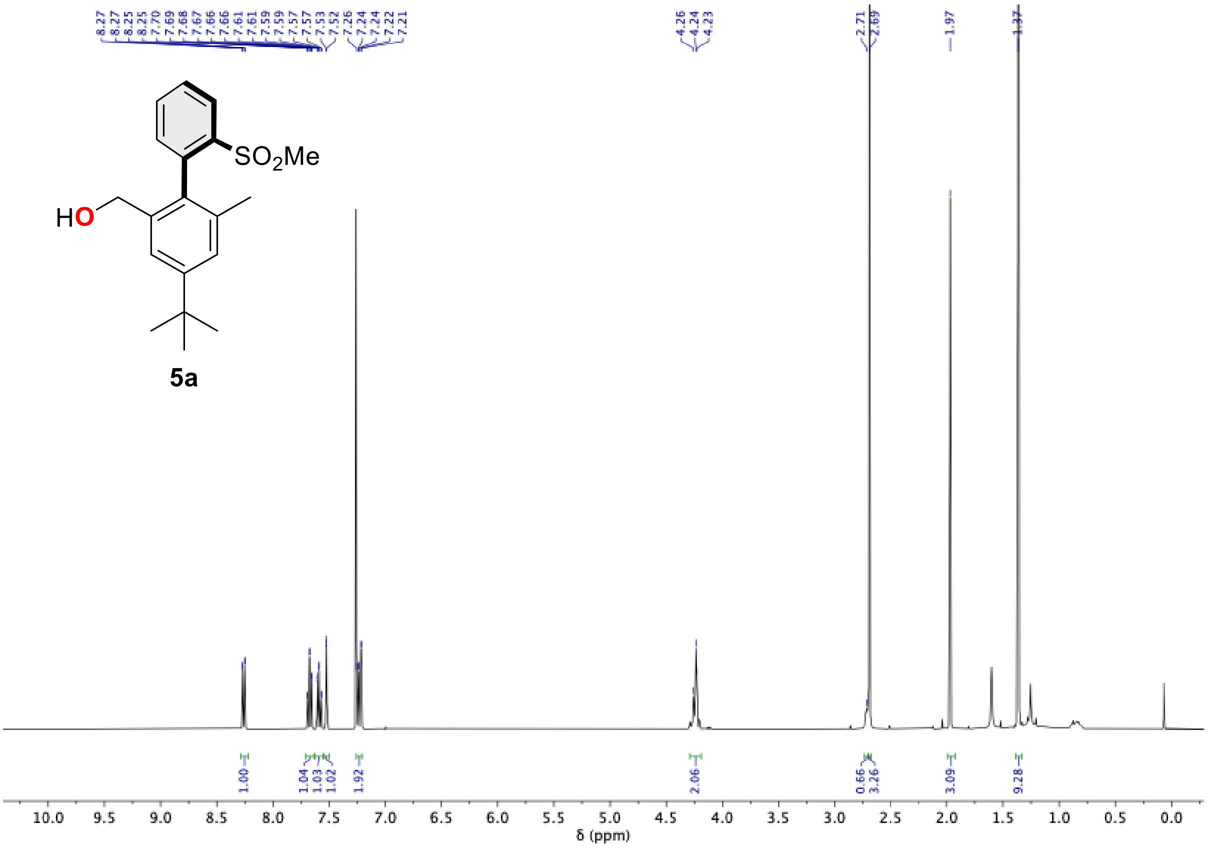
SO2Me
HO
5a
8.27
8.27
8.25
8.25
7.70
7.69
7.68
7.67
7.66
7.66
7.61
7.61
7.59
7.59
7.57
7.57
7.53
7.52
7.26
7.24
7.24
7.22
7.21
4.26
4.24
4.23
2.71
2.69
1.97
1.37
1.00
1.04
1.03
1.02
1.92
2.06
0.66
3.26
3.09
9.28
δ (ppm)

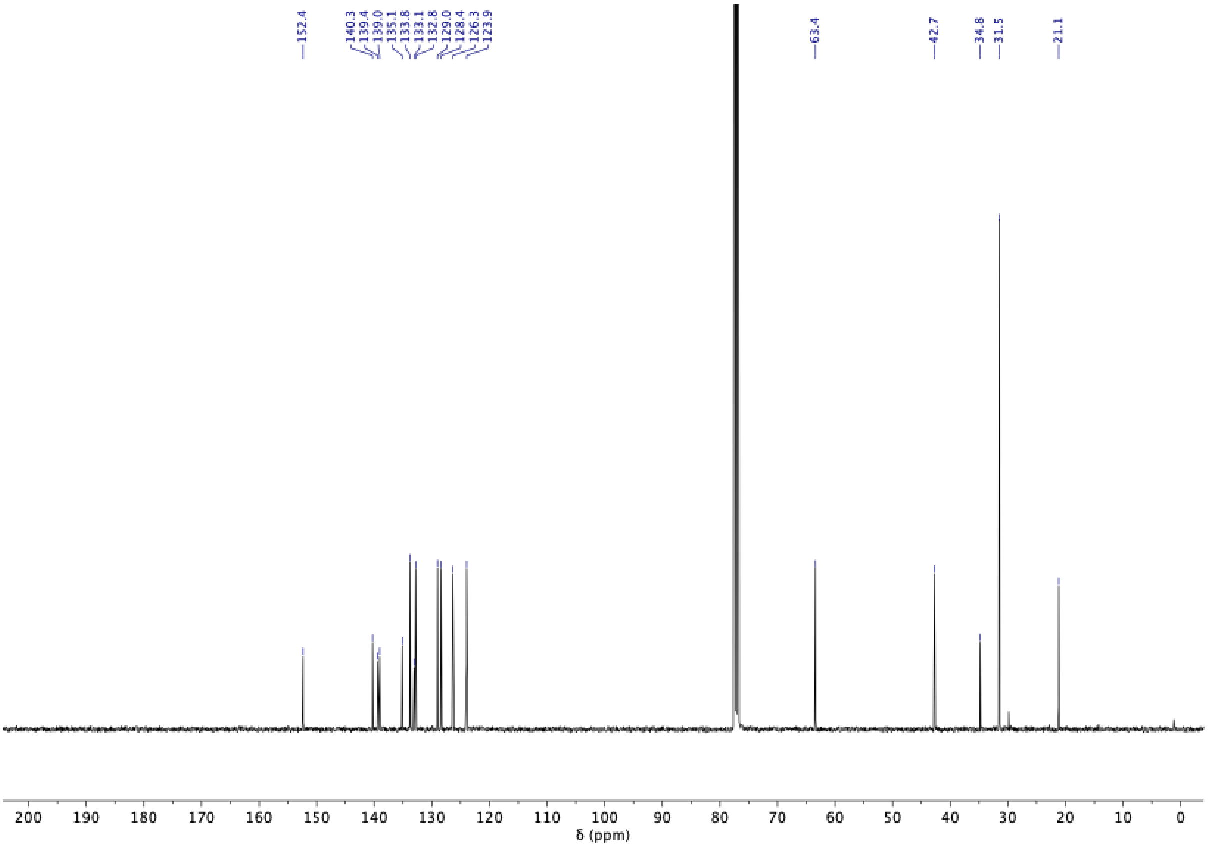
152.4
140.3
139.4
139.0
135.1
133.8
133.1
132.8
129.0
128.4
126.3
123.9
63.4
42.7
34.8
31.5
21.1
δ (ppm)

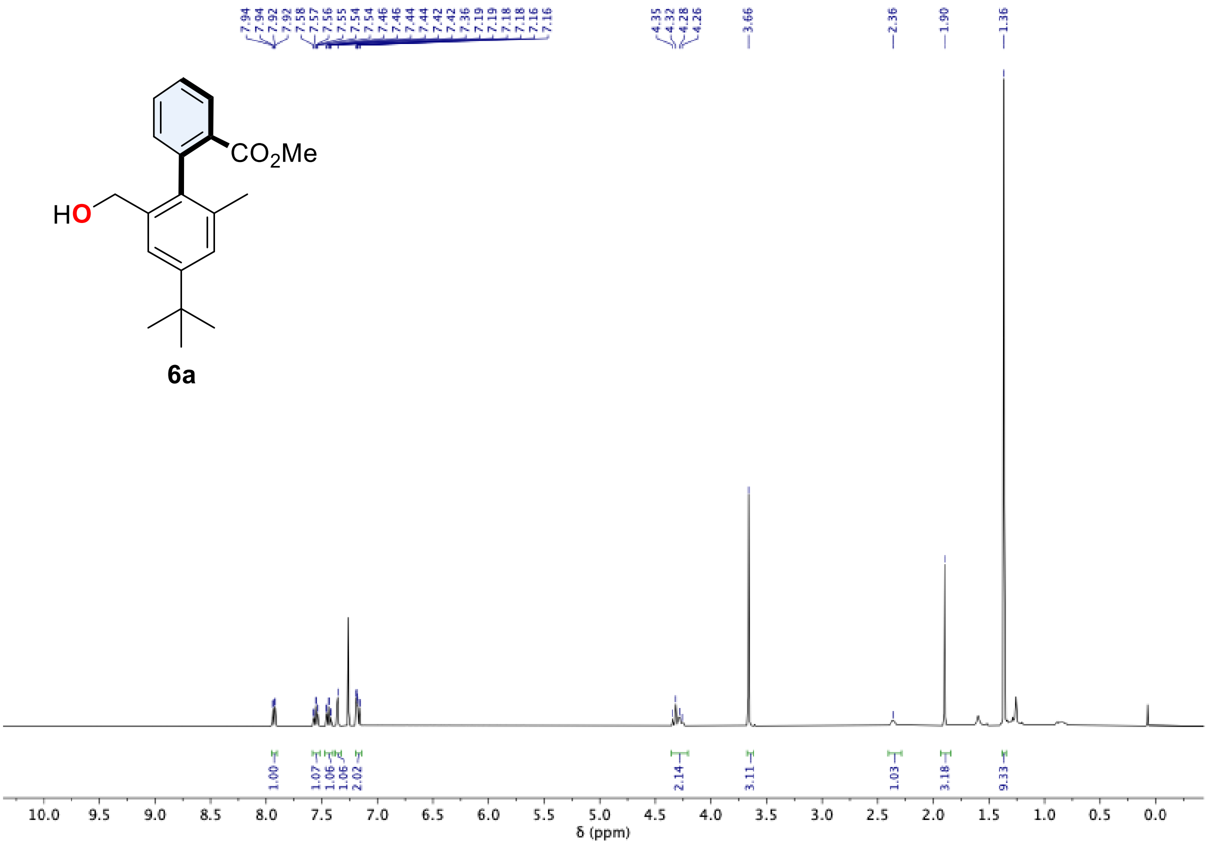
HO
CO2Me
6a
7.94 7.94 7.92 7.92 7.58 7.57 7.56 7.55 7.54 7.54 7.46 7.46 7.44 7.44 7.42 7.42 7.36 7.19 7.19 7.18 7.18 7.16 7.16
4.35 4.32 4.28 4.26
3.66
2.36
1.90
1.36
1.00 1.07 1.06 1.06 2.02 2.14 3.11 1.03 3.18 9.33
10.0 9.5 9.0 8.5 8.0 7.5 7.0 6.5 6.0 5.5 5.0 4.5 4.0 3.5 3.0 2.5 2.0 1.5 1.0 0.5 0.0
δ (ppm)

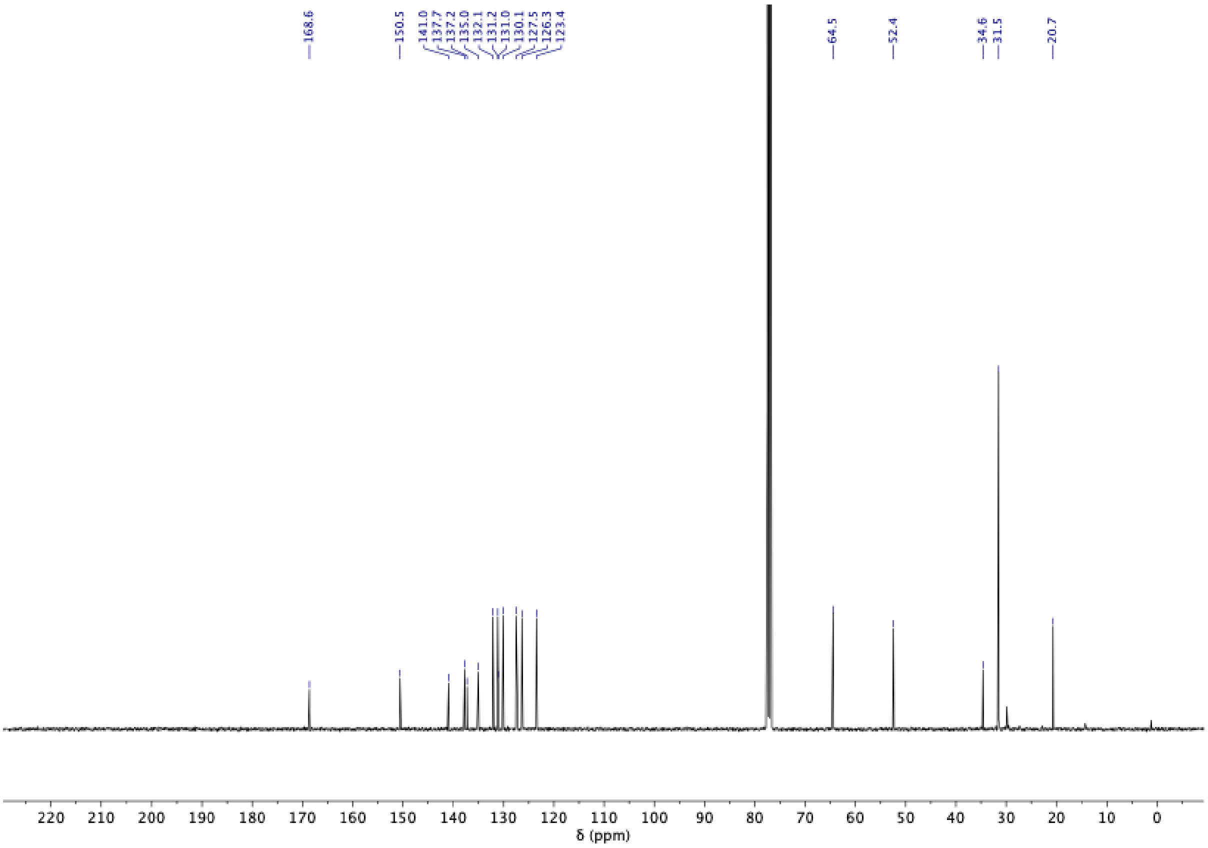
168.6
150.5
141.0 137.7 137.2 135.0 132.1 131.2 131.0 130.1 127.5 126.3 123.4
64.5
52.4
34.6 31.5
20.7
220 210 200 190 180 170 160 150 140 130 120 110 100 90 80 70 60 50 40 30 20 10 0
δ (ppm)

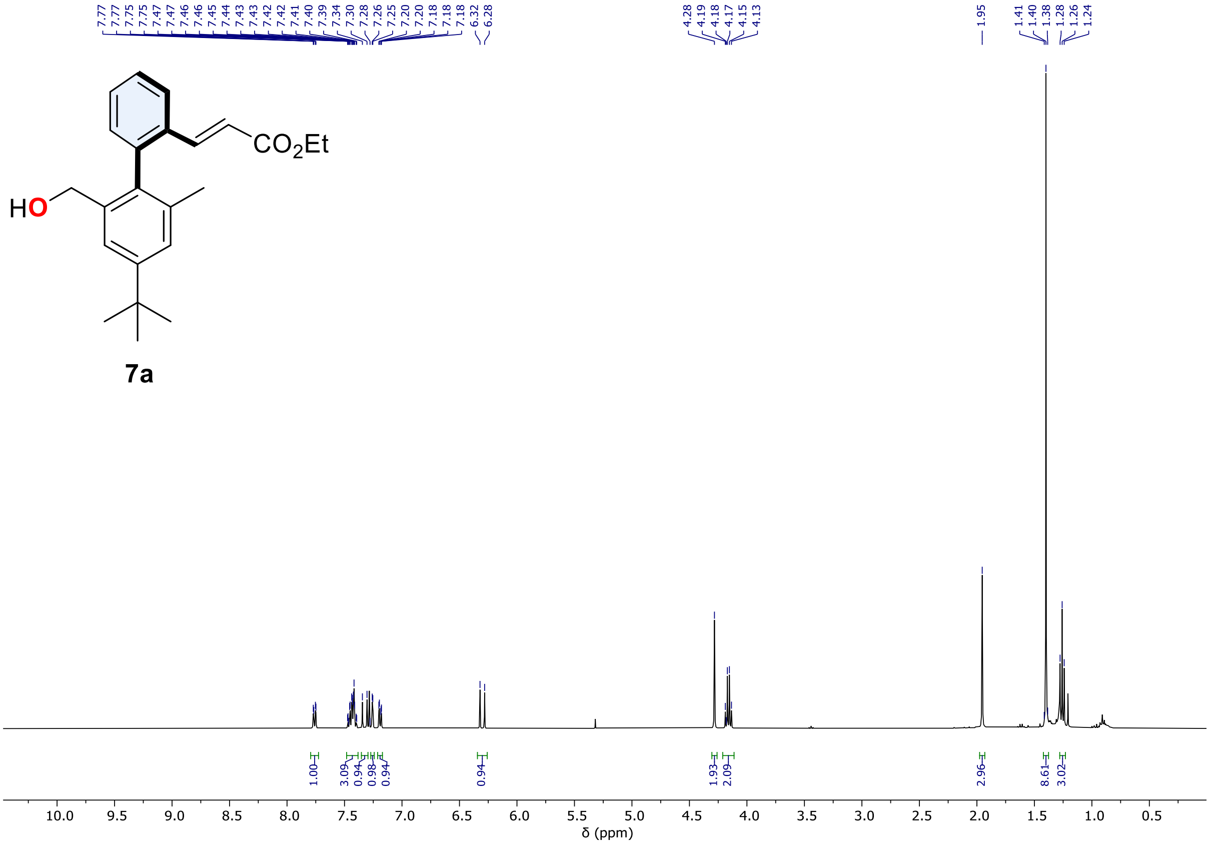
HO
CO2Et
7a
7.77 7.77 7.75 7.75 7.47 7.47 7.46 7.46 7.45 7.44 7.43 7.43 7.42 7.42 7.41 7.40 7.39 7.34 7.30 7.28 7.26 7.25 7.20 7.20 7.18 7.18 7.18 6.32 6.28
4.28 4.19 4.18 4.17 4.15 4.13
1.95
1.41 1.40 1.38 1.28 1.26 1.24
1.00 3.09 0.94 0.98 0.94 0.94 1.93 2.09 2.96 8.61 3.02
δ (ppm)

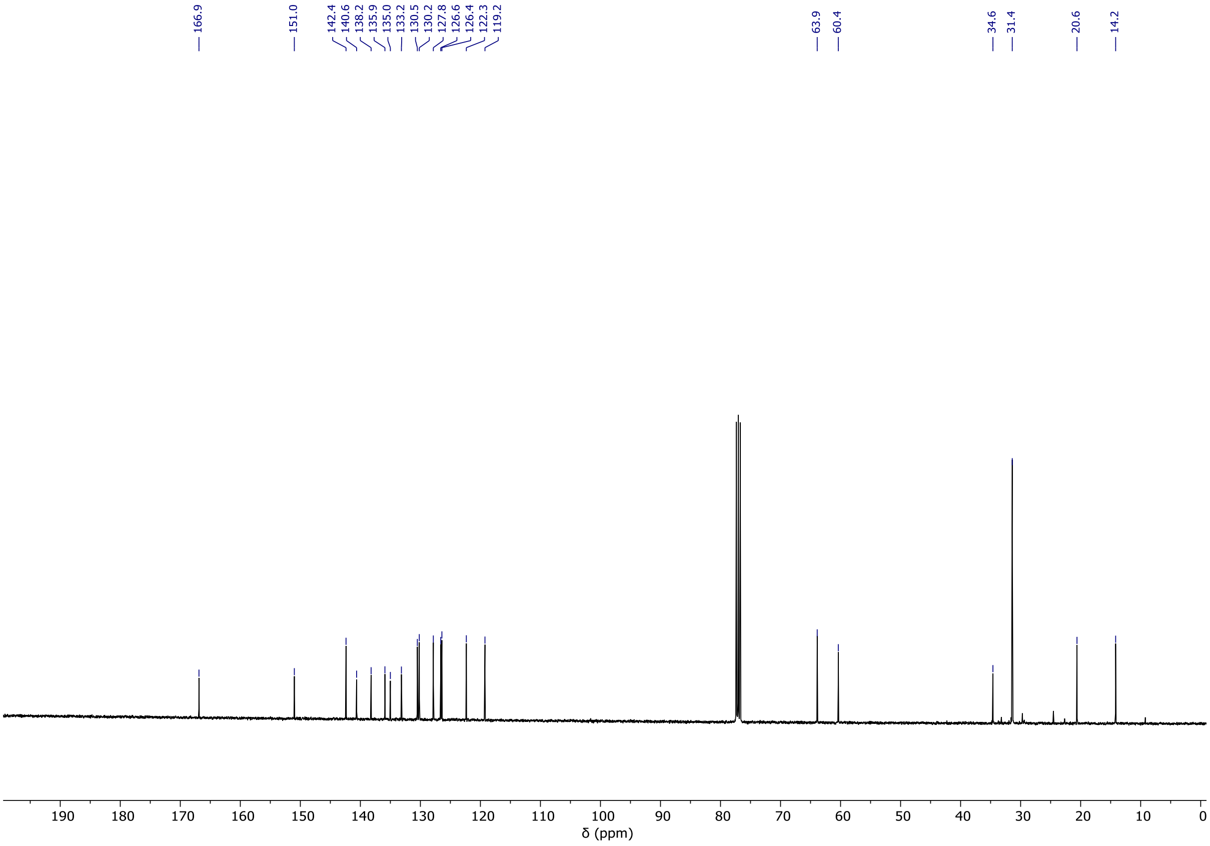
166.9
151.0
142.4 140.6 138.2 135.9 135.0 133.2 130.5 130.2 127.8 126.6 126.4 122.3 119.2
63.9 60.4
34.6 31.4
20.6
14.2
δ (ppm)

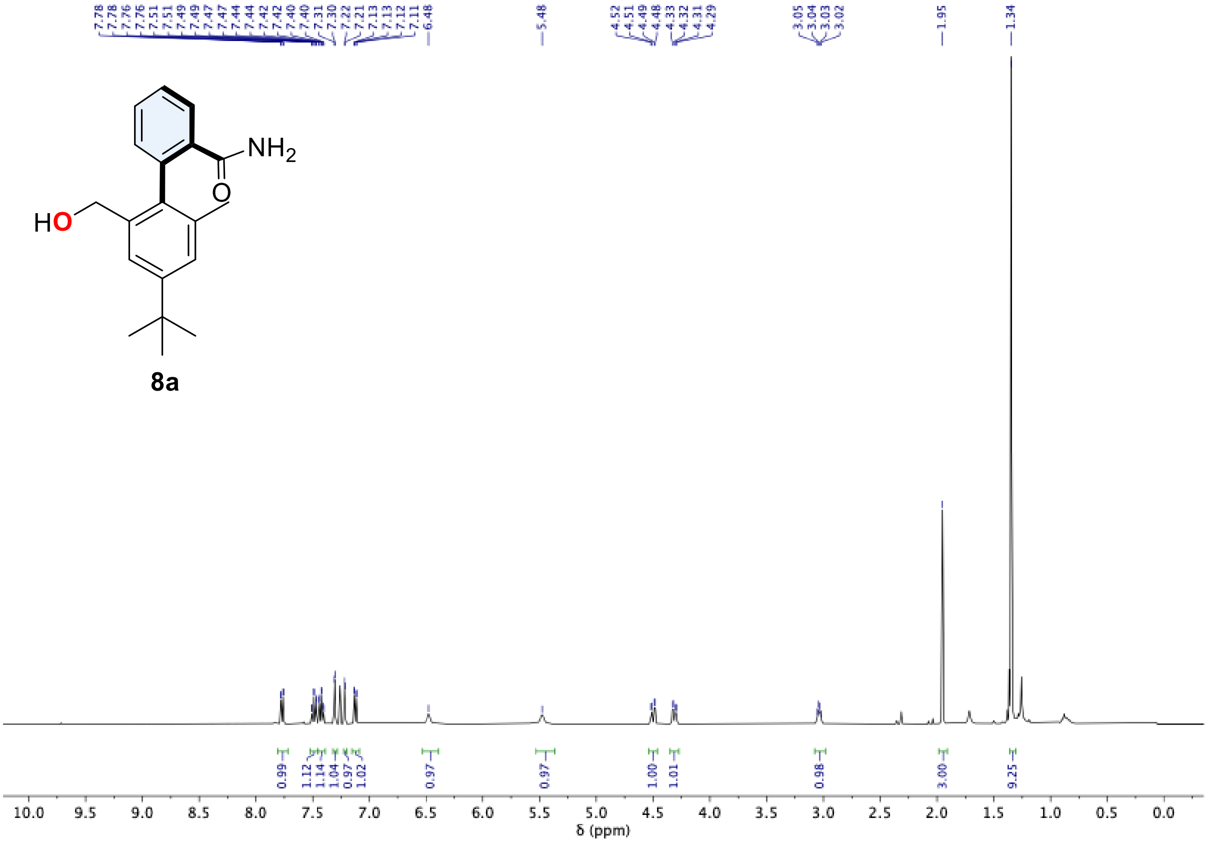
NH2
O
HO
8a
7.78 7.78 7.76 7.76 7.51 7.51 7.49 7.49 7.47 7.47 7.44 7.44 7.42 7.42 7.40 7.40 7.31 7.30 7.22 7.21 7.13 7.13 7.12 7.11 6.48
5.48
4.52 4.51 4.49 4.48 4.33 4.32 4.31 4.29
3.05 3.04 3.03 3.02
1.95
1.34
0.99 1.12 1.14 1.04 0.97 1.02 0.97 0.97 1.00 1.01 0.98 3.00 9.25
10.0 9.5 9.0 8.5 8.0 7.5 7.0 6.5 6.0 5.5 5.0 4.5 4.0 3.5 3.0 2.5 2.0 1.5 1.0 0.5 0.0
δ (ppm)

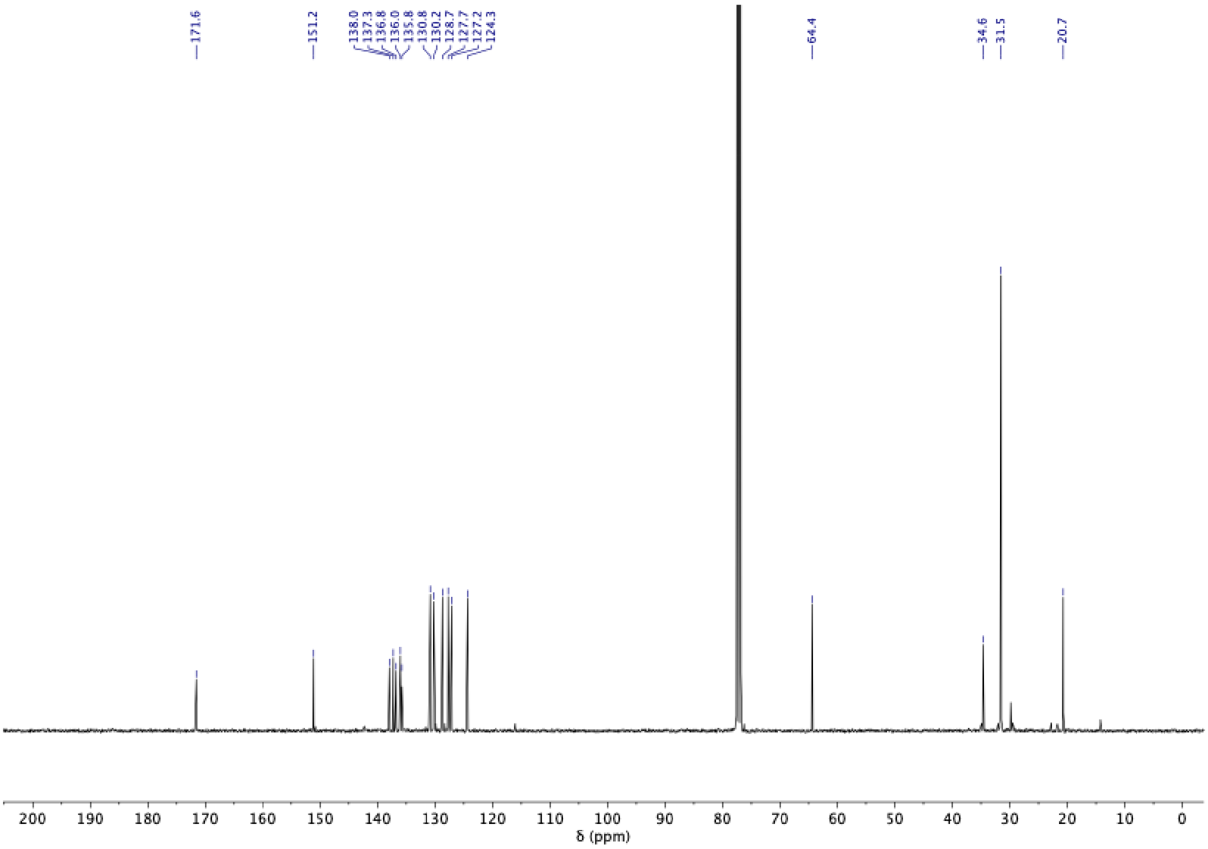
171.6
151.2
138.0 137.3 136.8 136.0 135.8 130.8 130.2 128.7 127.7 127.2 124.3
64.4
34.6 31.5
20.7
200 190 180 170 160 150 140 130 120 110 100 90 80 70 60 50 40 30 20 10 0
δ (ppm)

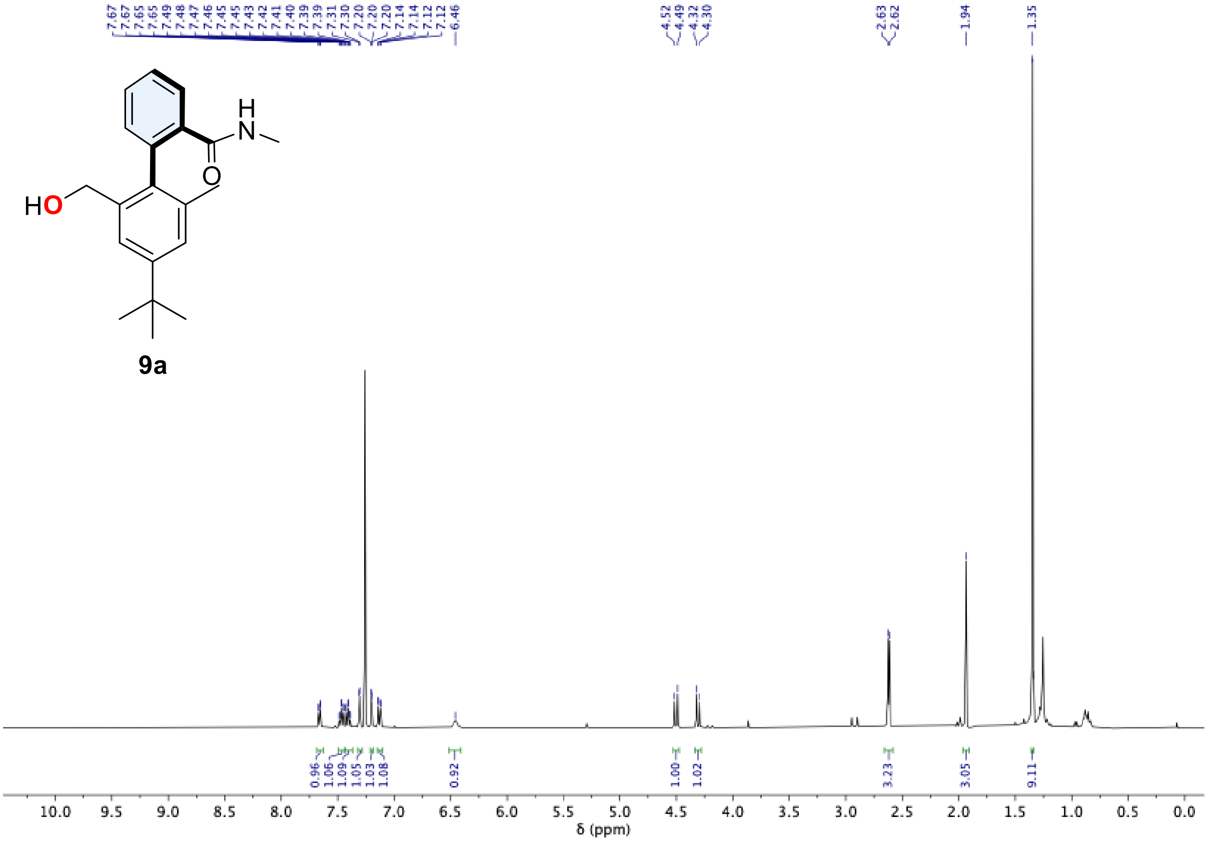

HO
H
N
O
9a
7.67 7.67 7.65 7.65 7.49 7.48 7.47 7.46 7.45 7.45 7.43 7.42 7.41 7.40 7.39 7.39 7.31 7.30 7.20 7.20 7.20 7.14 7.14 7.12 7.12 6.46
4.52 4.49 4.32 4.30
2.63 2.62
1.94
1.35
0.96 1.06 1.09 1.05 1.03 1.08 0.92 1.00 1.02 3.23 3.05 9.11
10.0 9.5 9.0 8.5 8.0 7.5 7.0 6.5 6.0 5.5 5.0 4.5 4.0 3.5 3.0 2.5 2.0 1.5 1.0 0.5 0.0
δ (ppm)

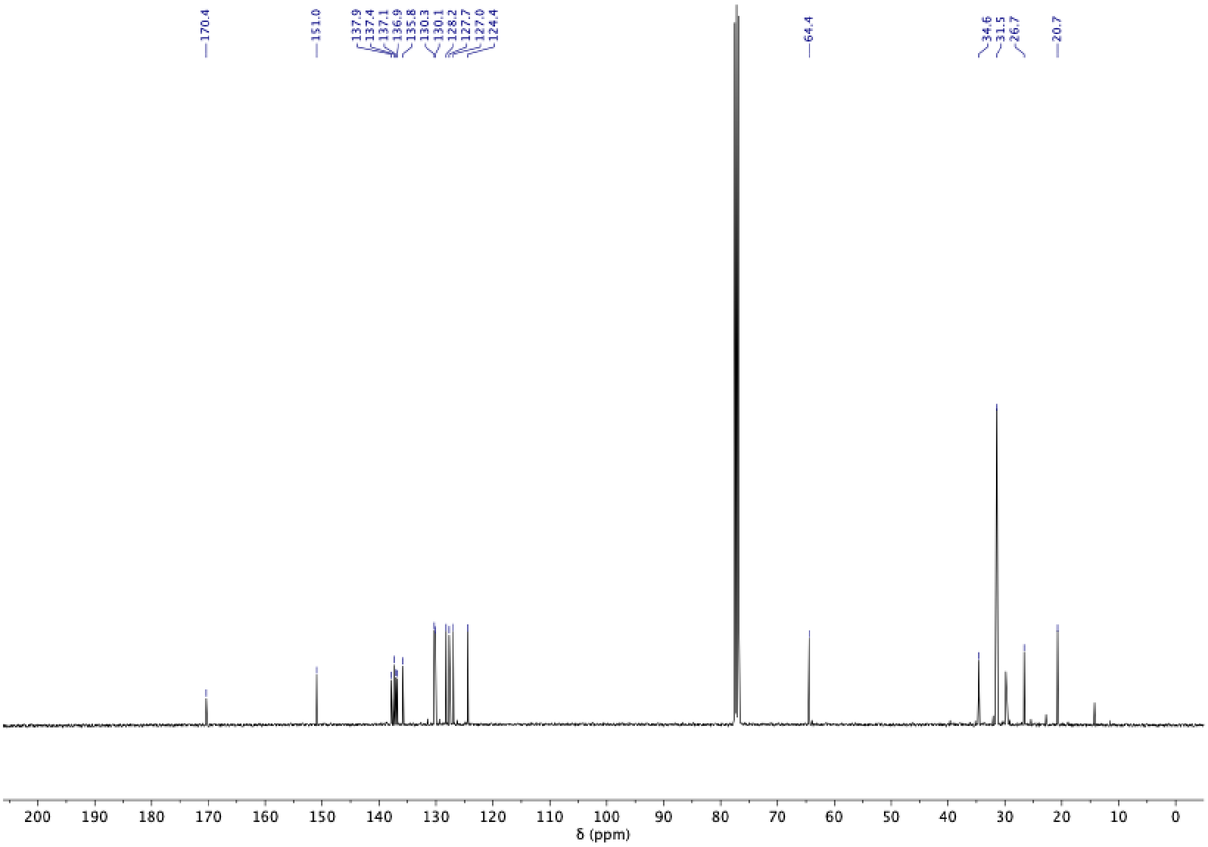

170.4
151.0
137.9 137.4 137.1 136.9 135.8 130.3 130.1 128.2 127.7 127.0 124.4
64.4
34.6 31.5 26.7
20.7
200 190 180 170 160 150 140 130 120 110 100 90 80 70 60 50 40 30 20 10 0
δ (ppm)

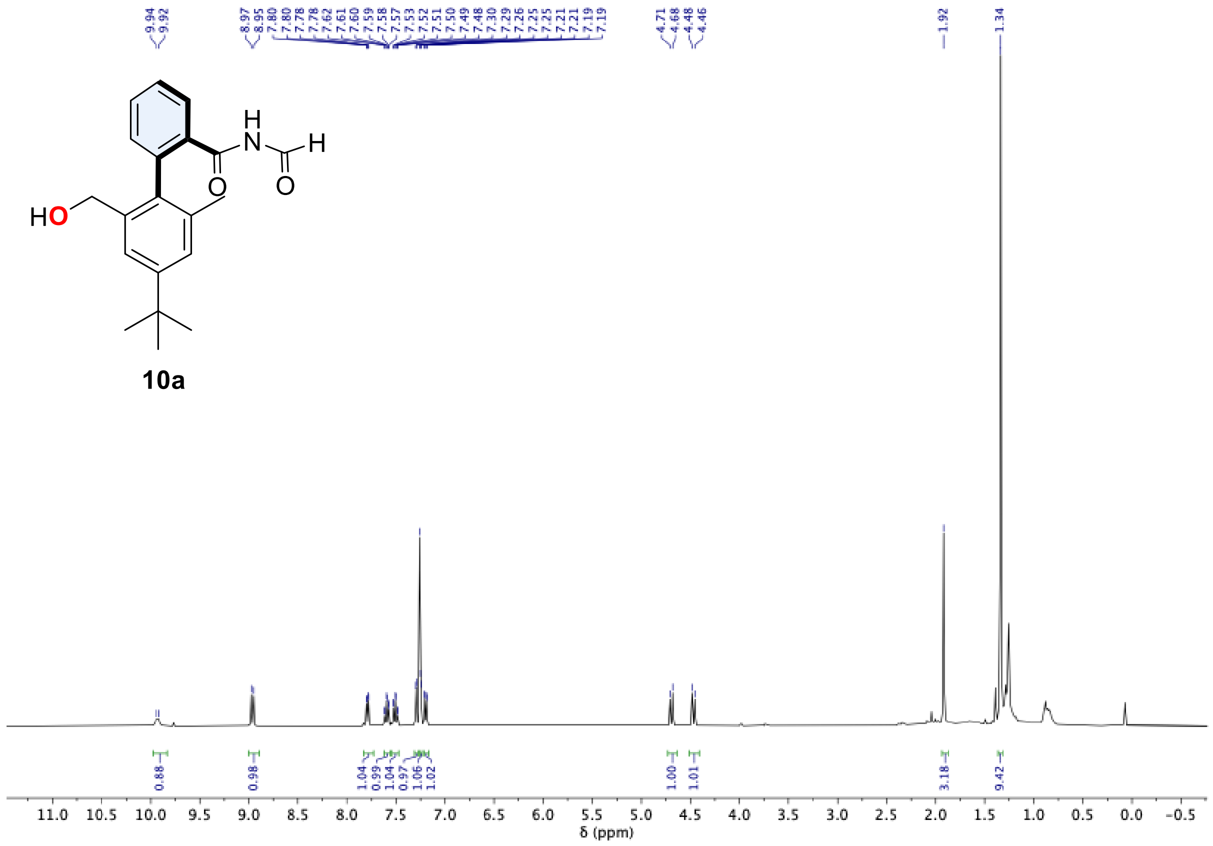
HO
H
N
H
O
O
10a
δ (ppm)

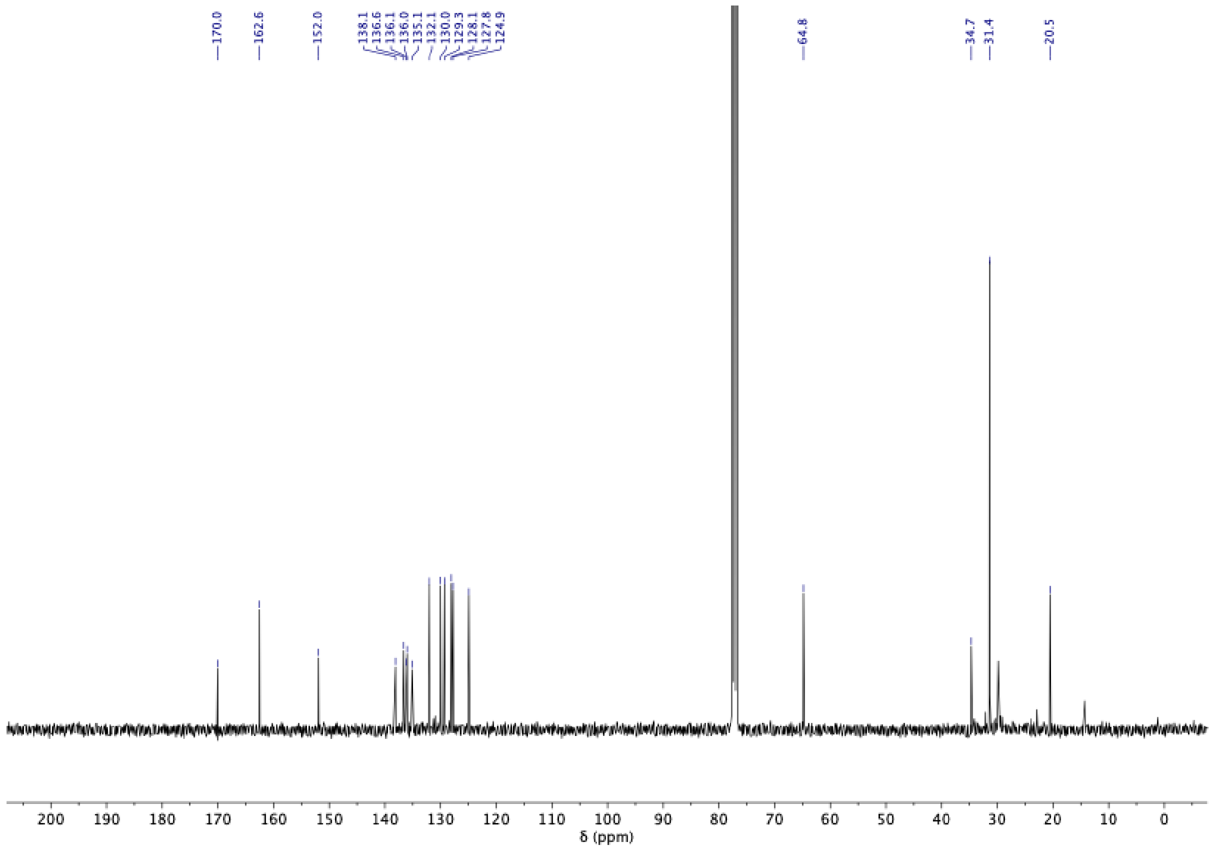
δ (ppm)

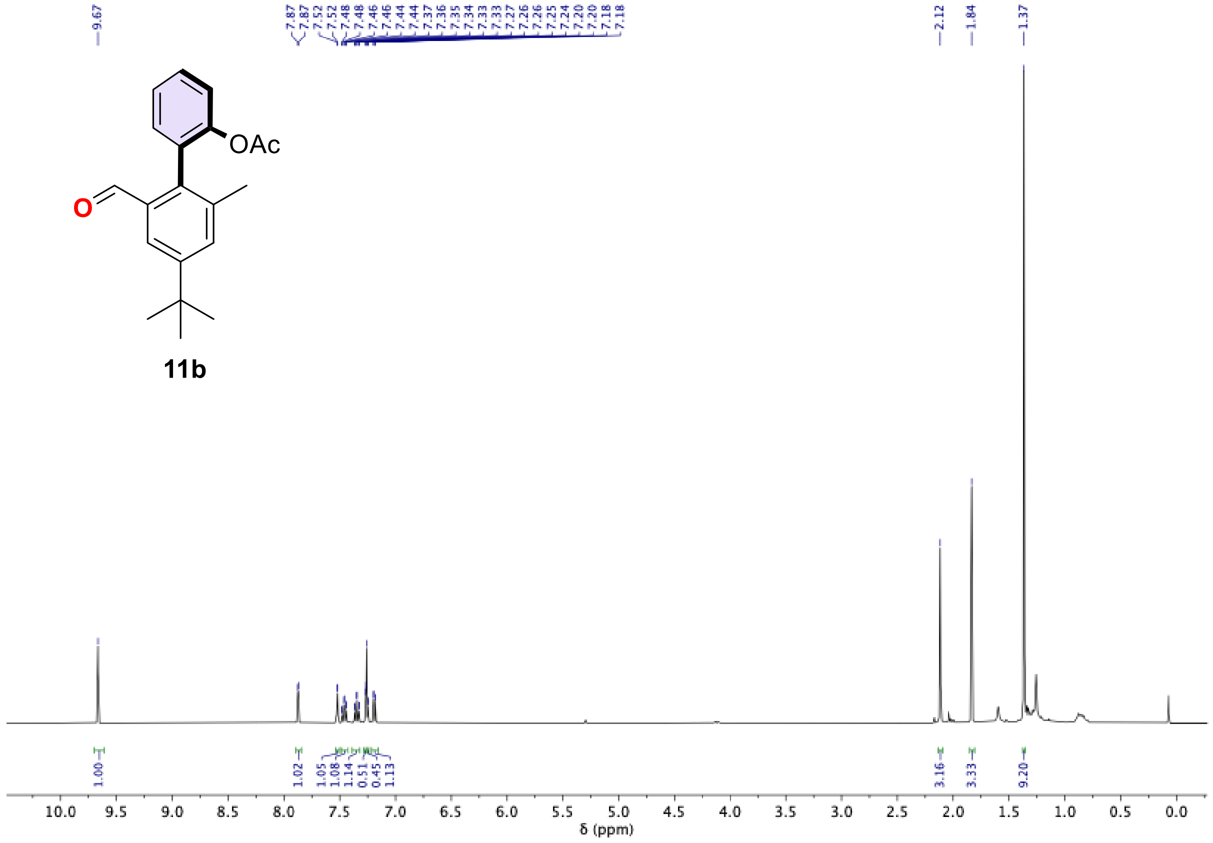
OAc
O
9.67
7.87
7.87
7.52
7.52
7.48
7.48
7.46
7.46
7.44
7.44
7.37
7.36
7.35
7.34
7.33
7.33
7.27
7.26
7.26
7.25
7.24
7.20
7.20
7.18
7.18
2.12
1.84
1.37
1.00
1.02
1.05
1.08
1.14
0.51
0.45
1.13
3.16
3.33
9.20
10.0 9.5 9.0 8.5 8.0 7.5 7.0 6.5 6.0 5.5 5.0 4.5 4.0 3.5 3.0 2.5 2.0 1.5 1.0 0.5 0.0
δ (ppm)

11b

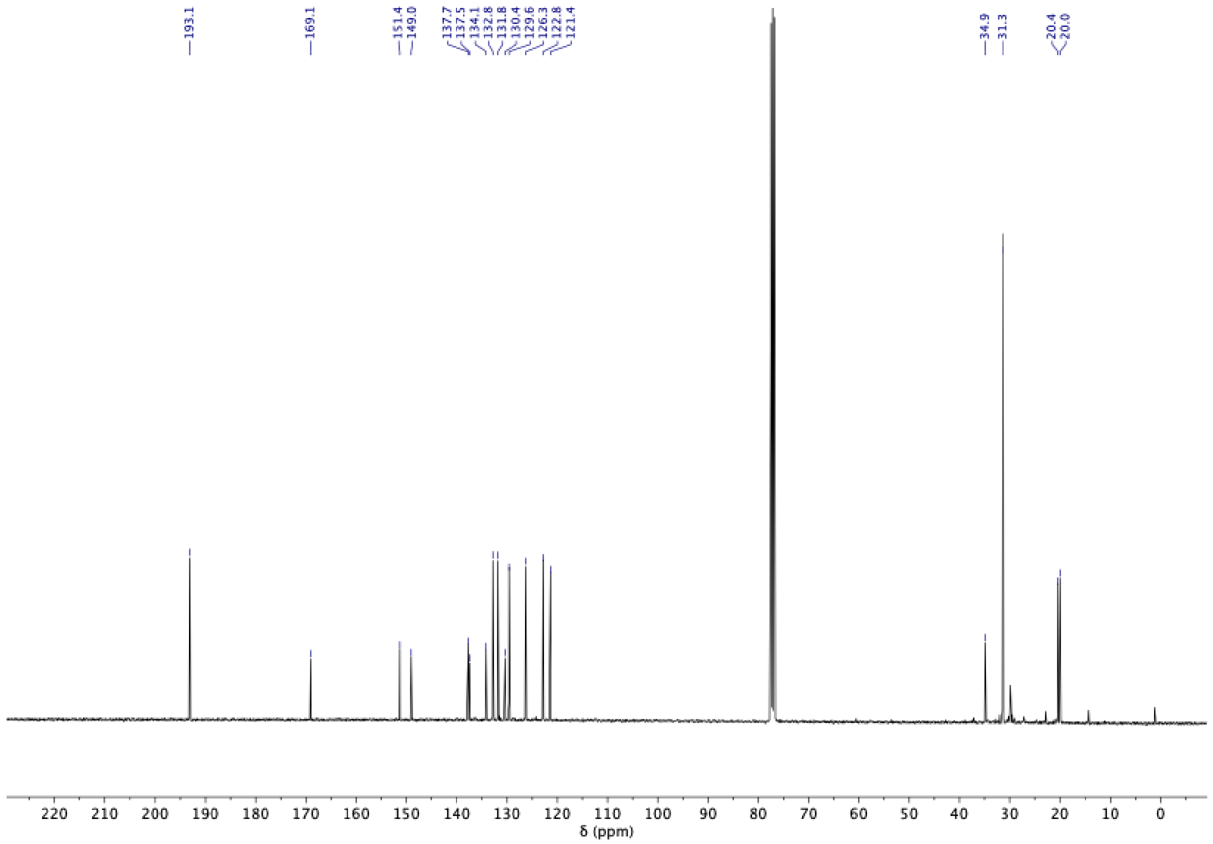
193.1
169.1
151.4
149.0
137.7
137.5
134.1
132.8
131.8
130.4
129.6
126.3
122.8
121.4
34.9
31.3
20.4
20.0
220 210 200 190 180 170 160 150 140 130 120 110 100 90 80 70 60 50 40 30 20 10 0
δ (ppm)

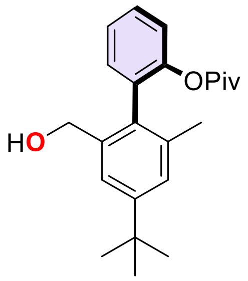
OPiv
HO

12a

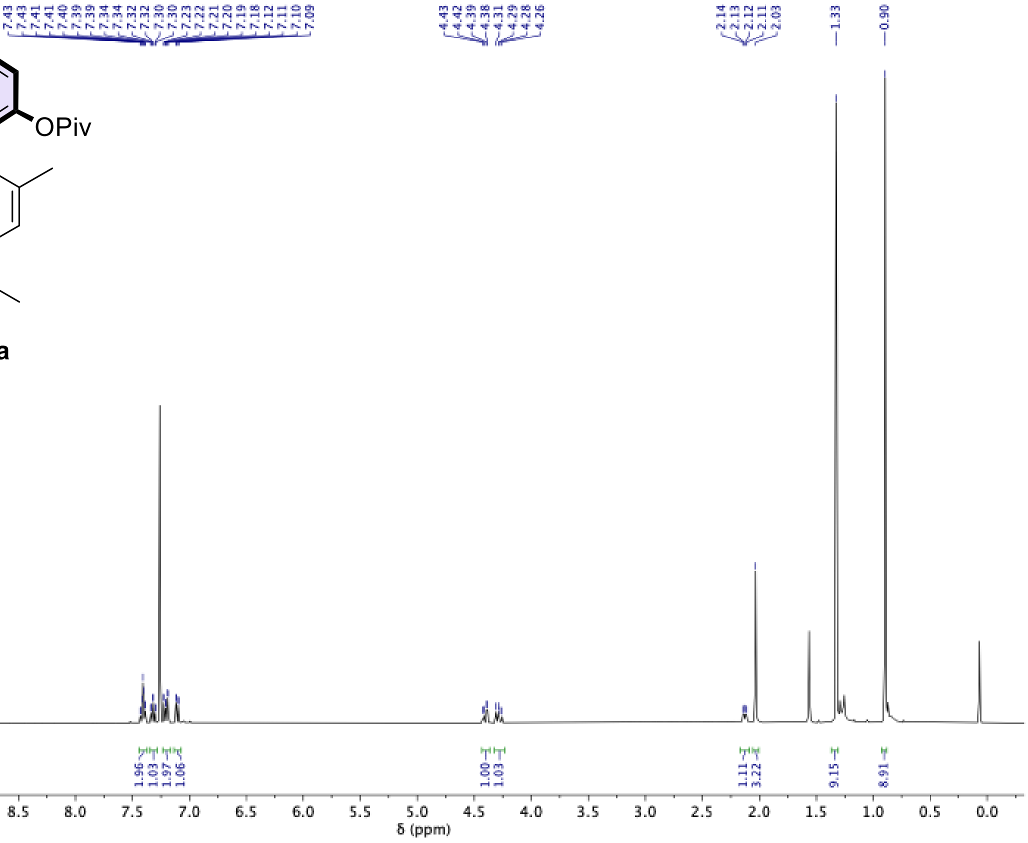
7.43
7.43
7.41
7.40
7.39
7.39
7.34
7.34
7.32
7.32
7.30
7.30
7.23
7.22
7.21
7.20
7.19
7.18
7.12
7.11
7.10
7.09
4.43
4.42
4.39
4.38
4.31
4.29
4.28
4.26
2.14
2.13
2.12
2.11
2.03
1.33
0.90
1.96
1.03
1.97
1.06
1.00
1.03
1.11
3.22
9.15
8.91
10.0
9.5
9.0
8.5
8.0
7.5
7.0
6.5
6.0
5.5
5.0
4.5
4.0
3.5
3.0
2.5
2.0
1.5
1.0
0.5
0.0
δ (ppm)

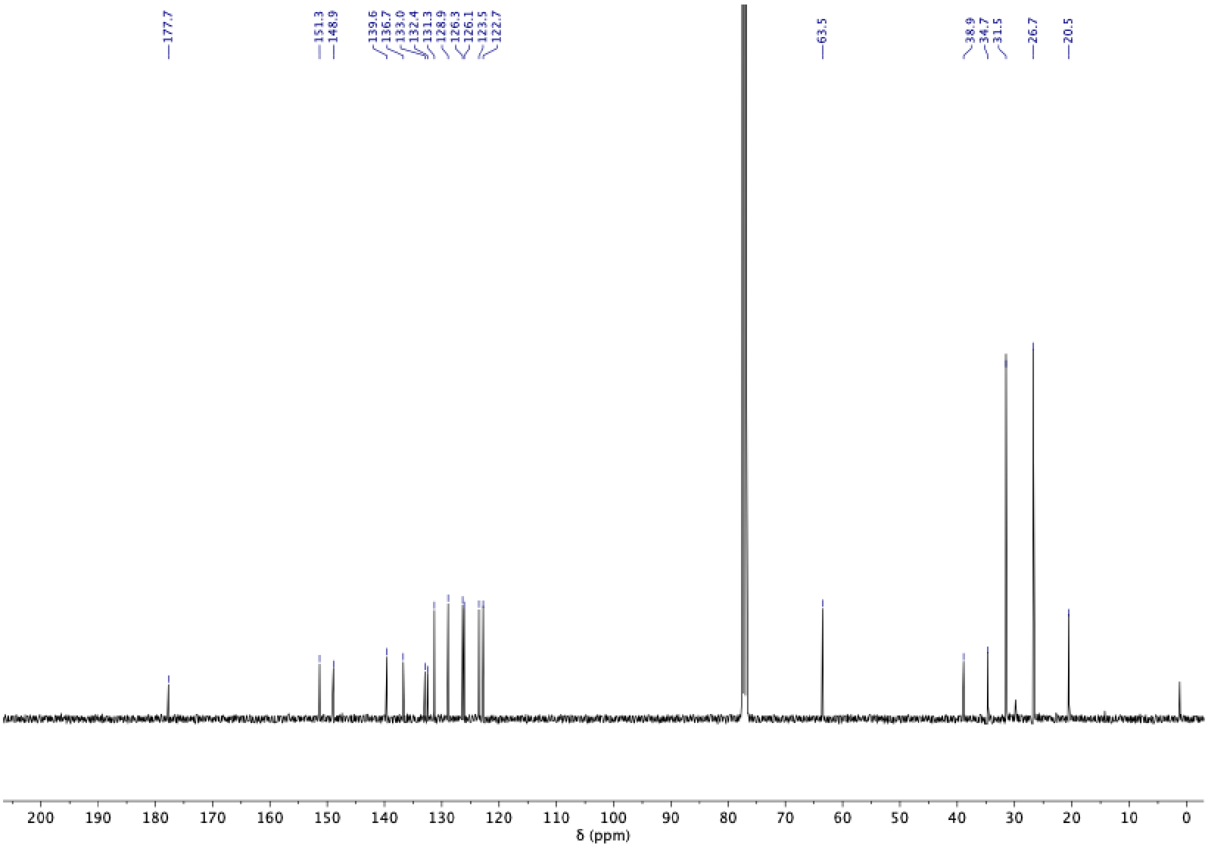
177.7
151.3
148.9
139.6
136.7
133.0
132.4
131.3
128.9
126.3
126.1
123.5
122.7
63.5
38.9
34.7
31.5
26.7
20.5
200
190
180
170
160
150
140
130
120
110
100
90
80
70
60
50
40
30
20
10
0
δ (ppm)

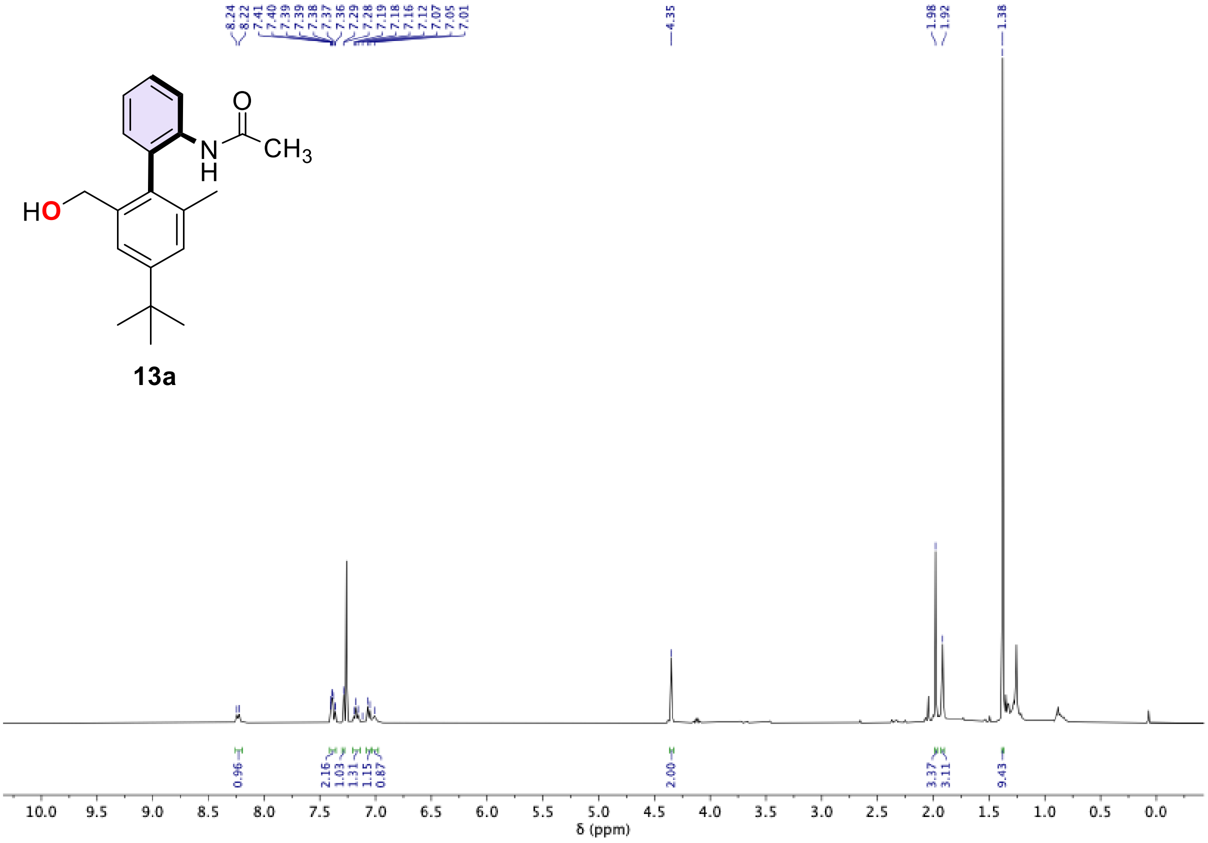
HO
O
N
H
CH3
13a
8.24
8.22
7.41
7.40
7.39
7.39
7.38
7.37
7.36
7.29
7.28
7.19
7.18
7.16
7.12
7.07
7.05
7.01
4.35
1.98
1.92
1.38
0.96
2.16
1.03
1.31
1.15
0.87
2.00
3.37
3.11
9.43
10.0 9.5 9.0 8.5 8.0 7.5 7.0 6.5 6.0 5.5 5.0 4.5 4.0 3.5 3.0 2.5 2.0 1.5 1.0 0.5 0.0
δ (ppm)

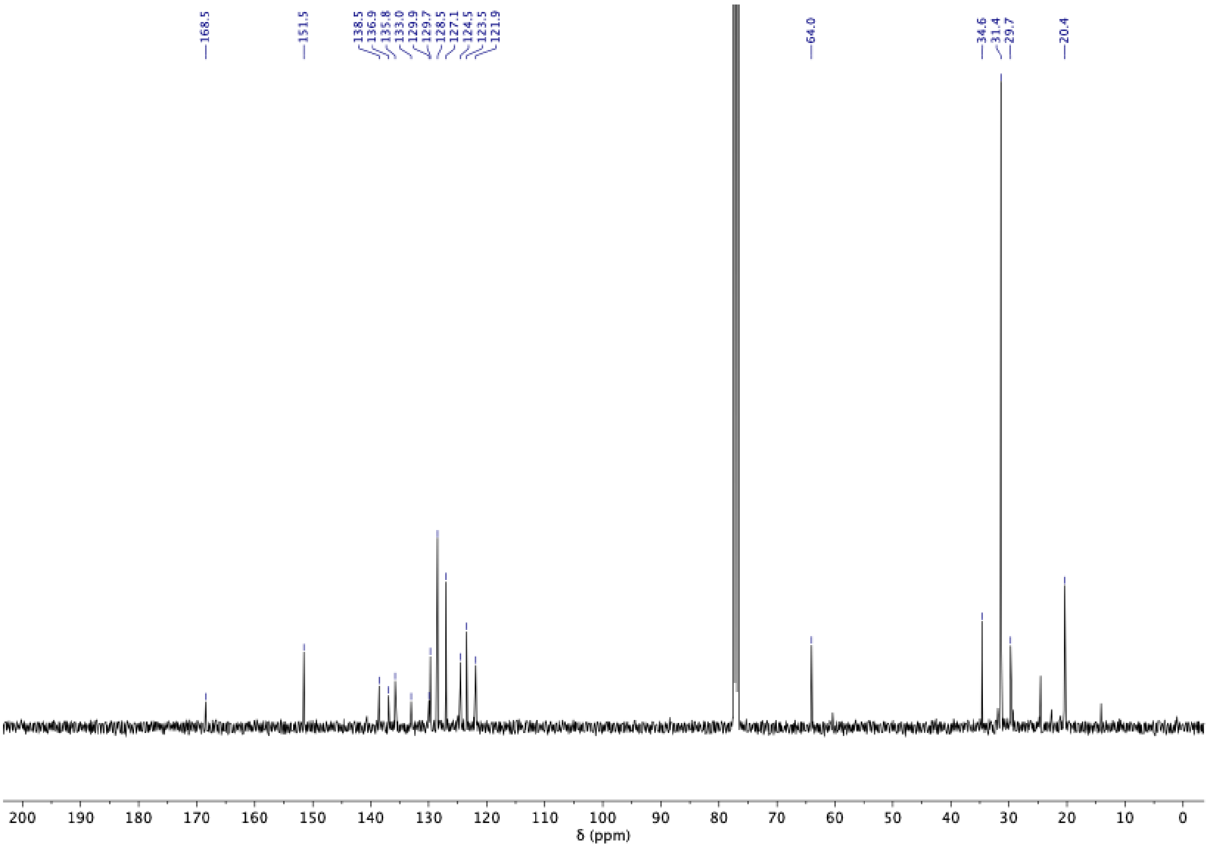
168.5
151.5
138.5
136.9
135.8
133.0
129.9
129.7
128.5
127.1
124.5
123.5
121.9
64.0
34.6
31.4
29.7
20.4
200 190 180 170 160 150 140 130 120 110 100 90 80 70 60 50 40 30 20 10 0
δ (ppm)

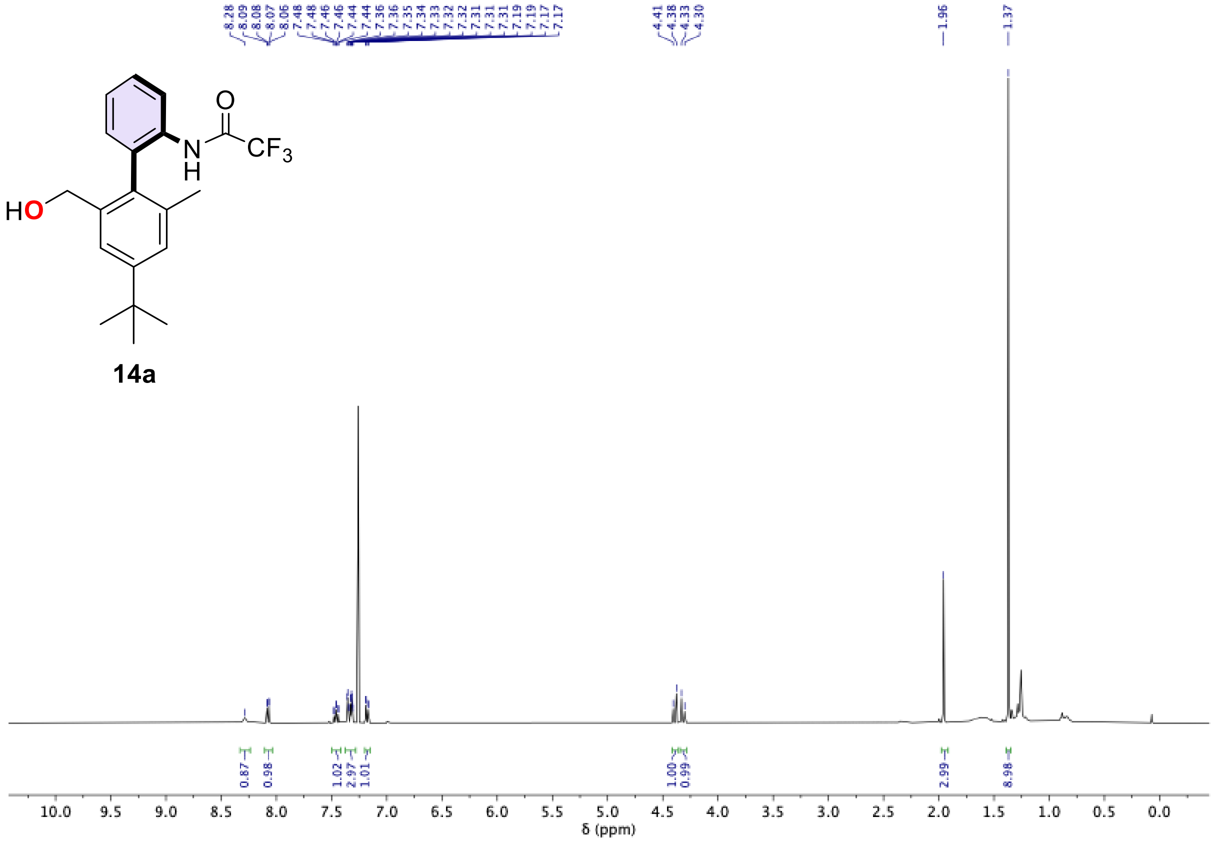
HO
O
N
H
CF3
14a
8.28
8.09
8.08
8.07
8.06
7.48
7.48
7.46
7.46
7.44
7.44
7.36
7.36
7.35
7.34
7.33
7.32
7.32
7.31
7.31
7.31
7.19
7.19
7.17
7.17
4.41
4.38
4.33
4.30
1.96
1.37
0.87
0.98
1.02
2.97
1.01
1.00
0.99
2.99
8.98
10.0
9.5
9.0
8.5
8.0
7.5
7.0
6.5
6.0
5.5
5.0
4.5
4.0
3.5
3.0
2.5
2.0
1.5
1.0
0.5
0.0
δ (ppm)

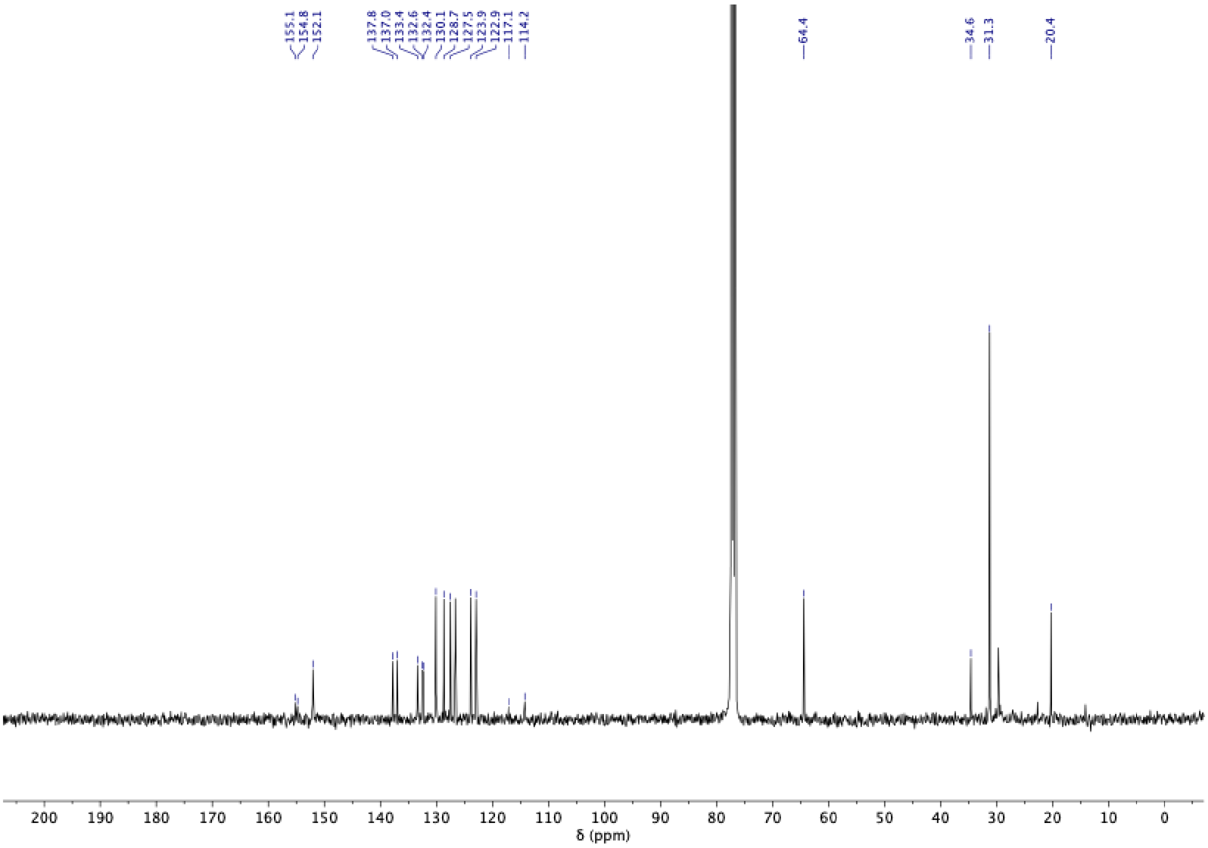
155.1
154.8
152.1
137.8
137.0
133.4
132.6
132.4
130.1
128.7
127.5
123.9
122.9
117.1
114.2
64.4
34.6
31.3
20.4
200
190
180
170
160
150
140
130
120
110
100
90
80
70
60
50
40
30
20
10
0
δ (ppm)

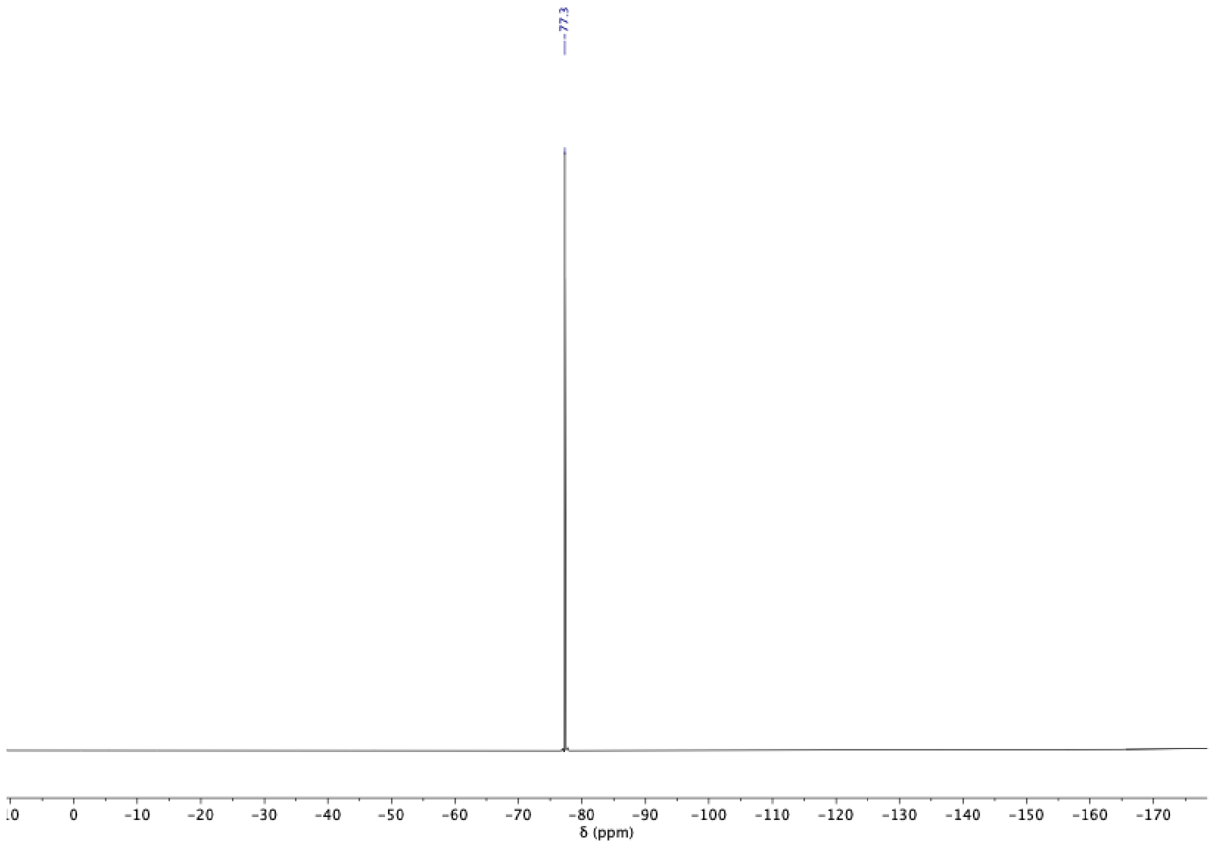
-77.3
10
0
-10
-20
-30
-40
-50
-60
-70
-80
-90
-100
-110
-120
-130
-140
-150
-160
-170
δ (ppm)

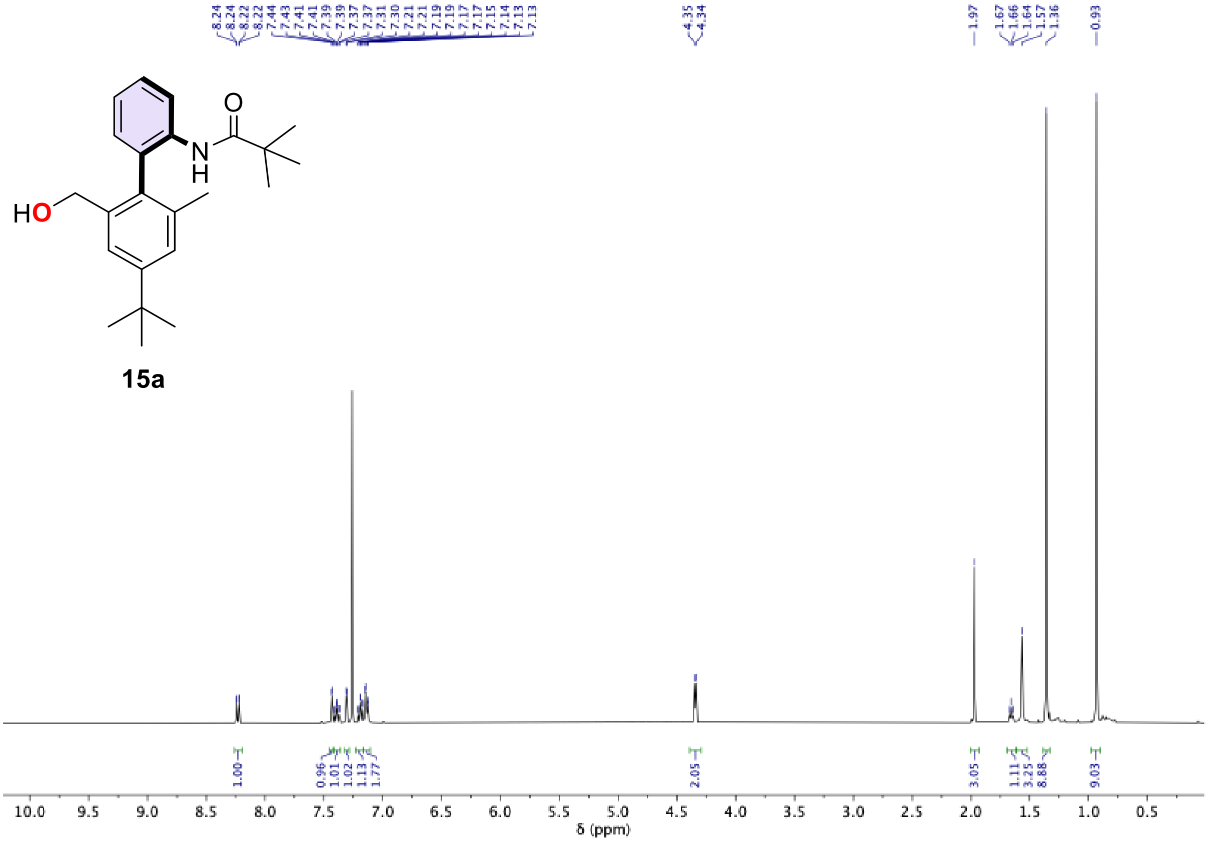
8.24 8.24 8.22 8.22 7.44 7.43 7.41 7.41 7.39 7.39 7.37 7.37 7.31 7.30 7.21 7.21 7.19 7.19 7.17 7.17 7.15 7.14 7.13 7.13
4.35 4.34
1.97 1.67 1.66 1.64 1.57 1.36
0.93
O
N
H
HO
15a
1.00 0.96 1.01 1.02 1.13 1.77 2.05 3.05 1.11 3.25 8.88 9.03
10.0 9.5 9.0 8.5 8.0 7.5 7.0 6.5 6.0 5.5 5.0 4.5 4.0 3.5 3.0 2.5 2.0 1.5 1.0 0.5
δ (ppm)

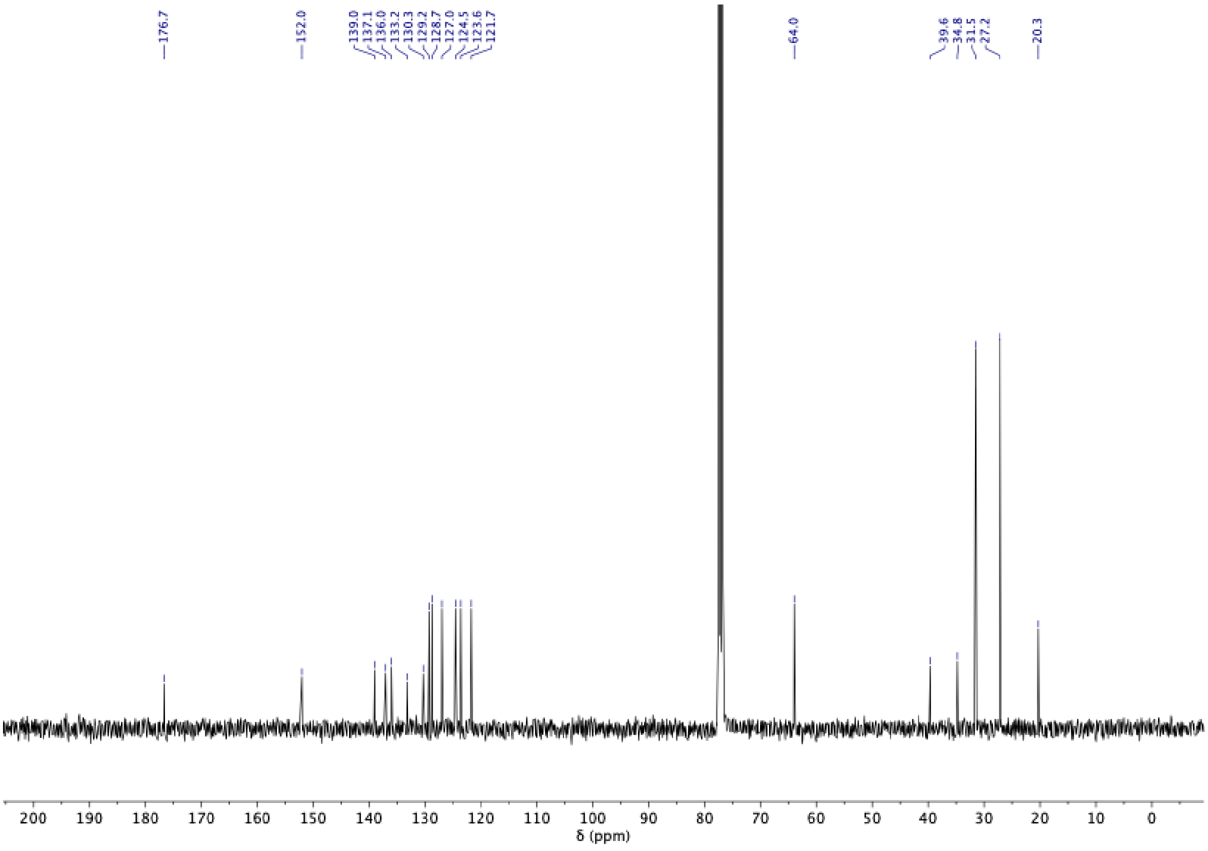
176.7
152.0
139.0 137.1 136.0 133.2 130.3 129.2 128.7 127.0 124.5 123.6 121.7
64.0
39.6 34.8 31.5 27.2
20.3
200 190 180 170 160 150 140 130 120 110 100 90 80 70 60 50 40 30 20 10 0
δ (ppm)

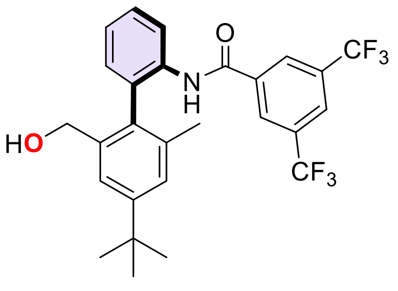
O
N
H
CF3
HO
CF3

16a

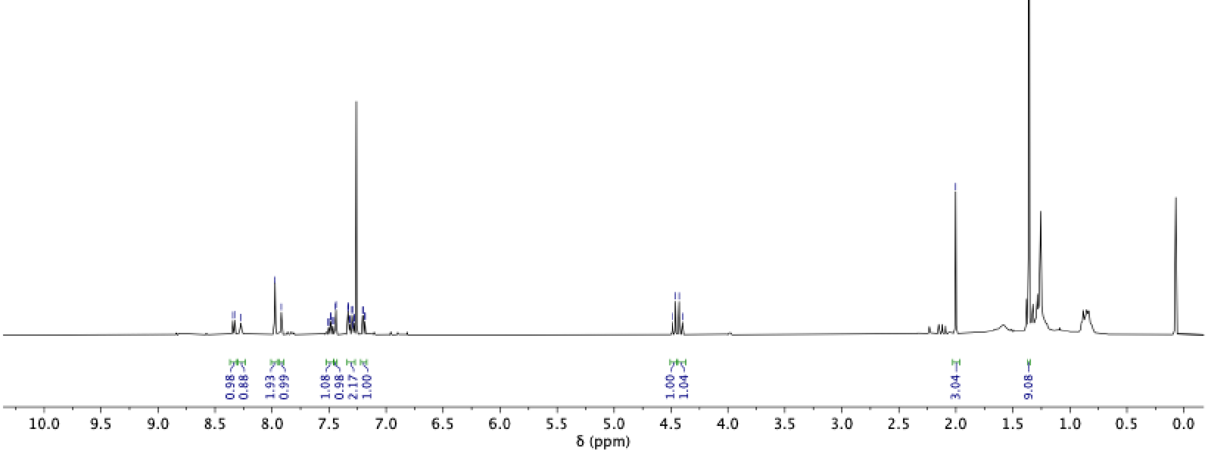
8.35
8.32
8.27
7.98
7.92
7.51
7.50
7.49
7.48
7.47
7.46
7.44
7.44
7.33
7.33
7.33
7.32
7.31
7.30
7.29
7.28
7.28
7.21
7.20
7.19
7.18
4.49
4.46
4.43
4.40
2.00
1.36
0.98
0.88
1.93
0.99
1.08
0.98
2.17
1.00
1.00
1.04
3.04
9.08
10.0 9.5 9.0 8.5 8.0 7.5 7.0 6.5 6.0 5.5 5.0 4.5 4.0 3.5 3.0 2.5 2.0 1.5 1.0 0.5 0.0
δ (ppm)

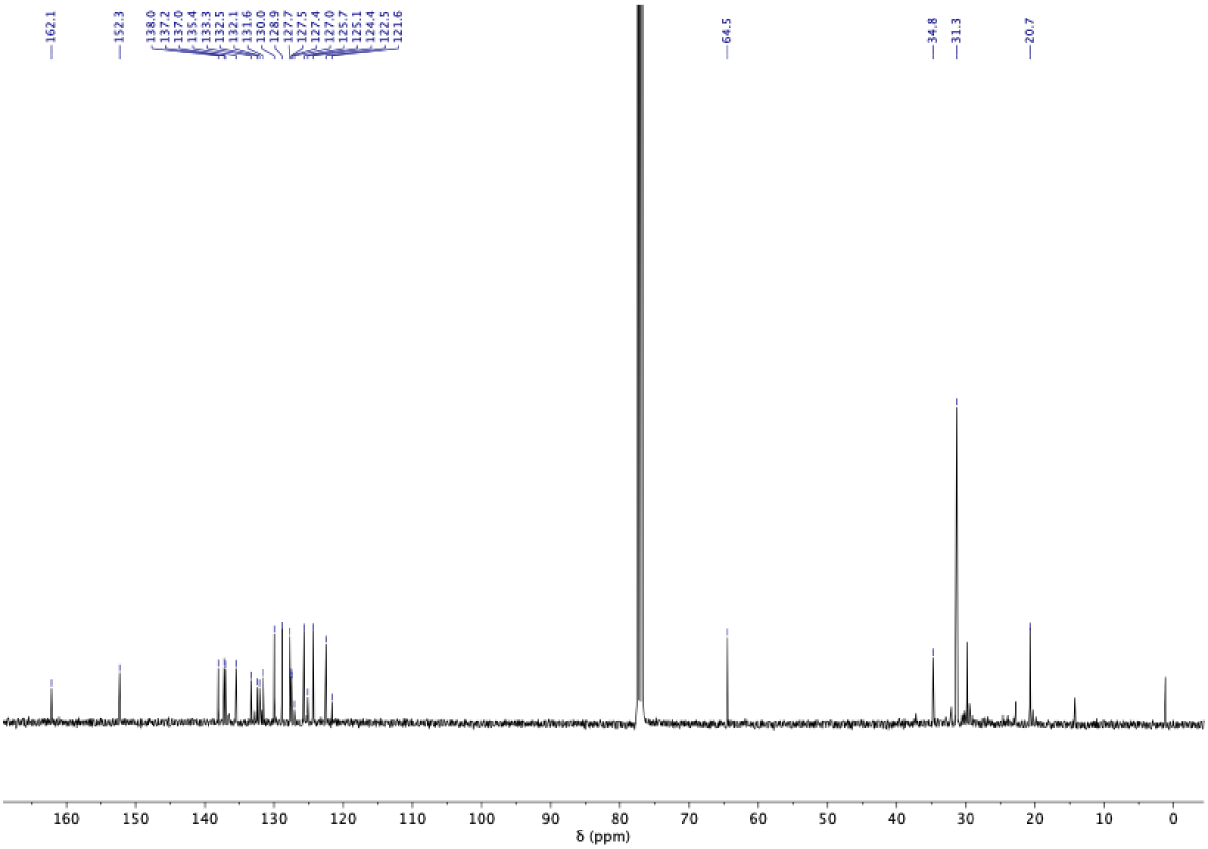
162.1
152.3
138.0
137.2
137.0
135.4
133.3
132.5
132.1
131.6
130.0
128.9
127.7
127.5
127.4
127.0
125.7
125.1
124.4
122.5
121.6
64.5
34.8
31.3
20.7
160 150 140 130 120 110 100 90 80 70 60 50 40 30 20 10 0
δ (ppm)

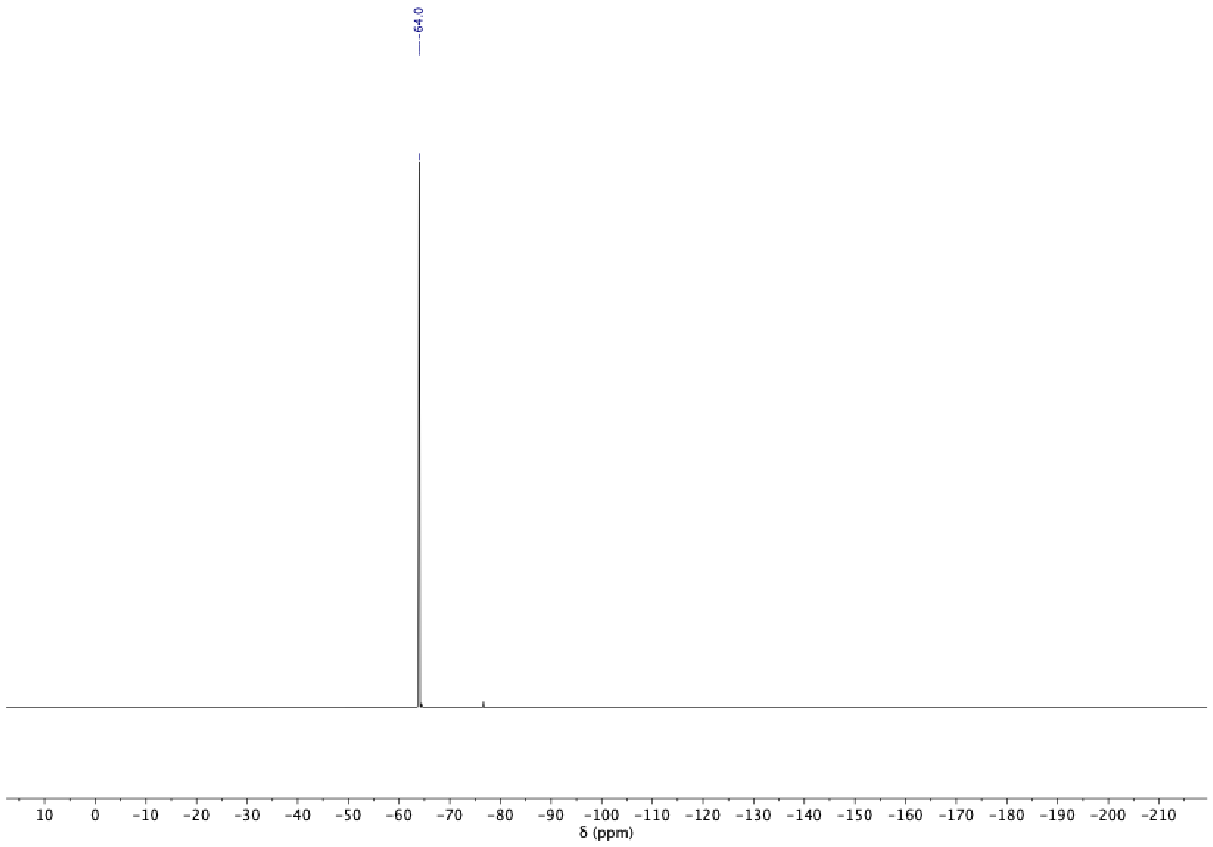

-64.0
10 0 −10 −20 −30 −40 −50 −60 −70 −80 −90 −100 −110 −120 −130 −140 −150 −160 −170 −180 −190 −200 −210
δ (ppm)

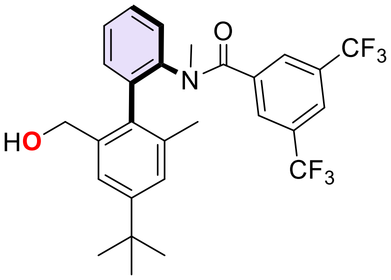
HO
O
N
CF3
CF3

17a

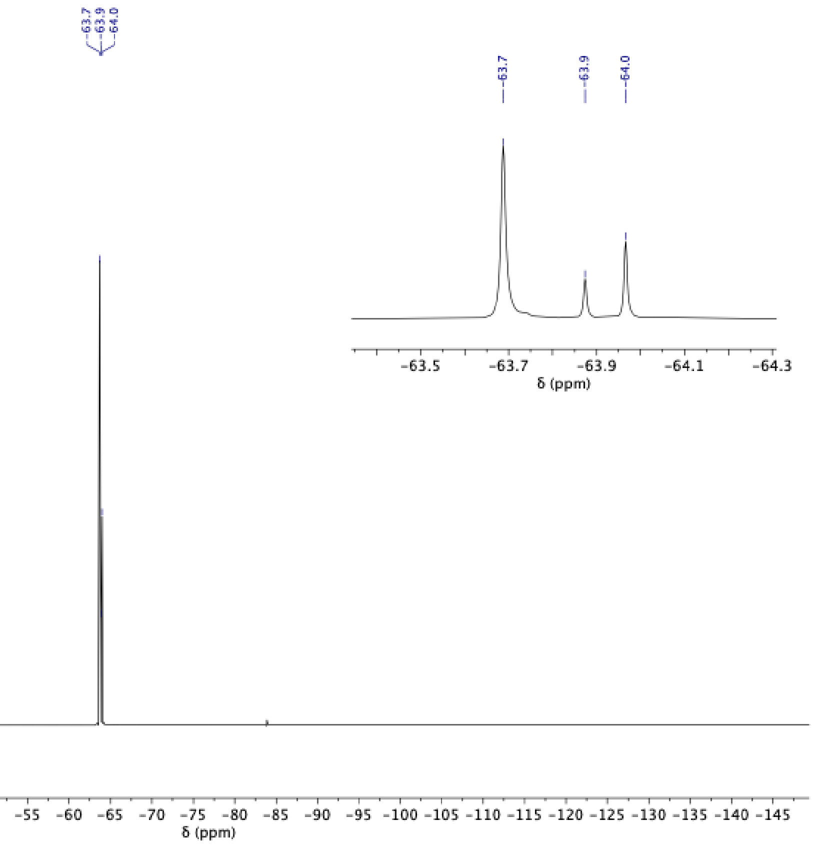
-63.7
-63.9
-64.0
-63.5 -63.7 -63.9 -64.1 -64.3
δ (ppm)
-5 -10 -15 -20 -25 -30 -35 -40 -45 -50 -55 -60 -65 -70 -75 -80 -85 -90 -95 -100 -105 -110 -115 -120 -125 -130 -135 -140 -145
δ (ppm)

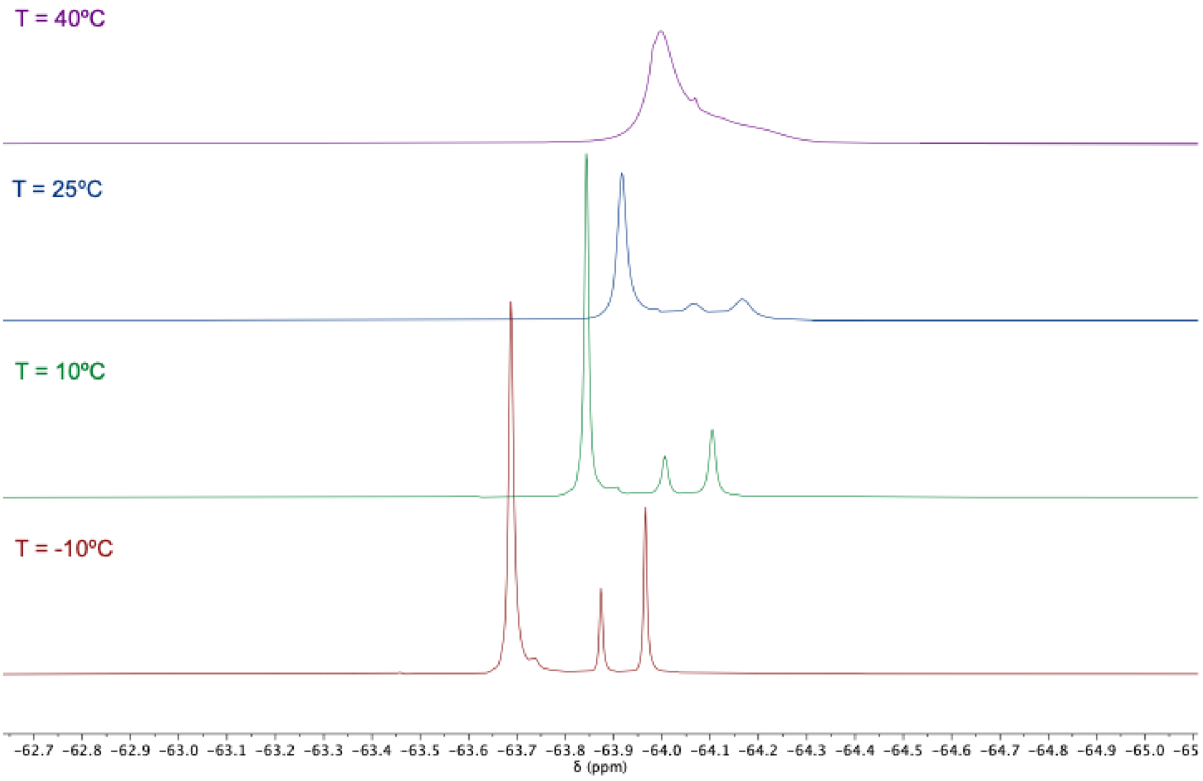
T = 40ºC
T = 25ºC
T = 10ºC
T = -10ºC
-62.7 -62.8 -62.9 -63.0 -63.1 -63.2 -63.3 -63.4 -63.5 -63.6 -63.7 -63.8 -63.9 -64.0 -64.1 -64.2 -64.3 -64.4 -64.5 -64.6 -64.7 -64.8 -64.9 -65.0 -65
δ (ppm)

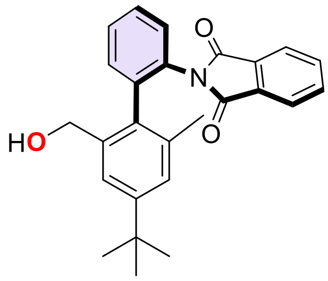
HO
N
O
O

18a

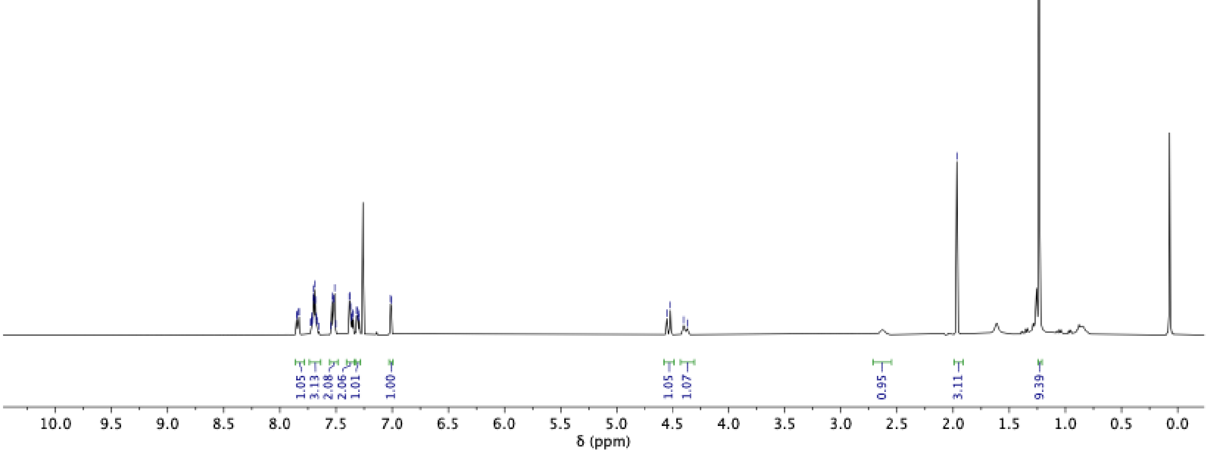
7.85
7.85
7.83
7.83
7.71
7.70
7.70
7.70
7.69
7.68
7.68
7.53
7.53
7.53
7.52
7.51
7.38
7.37
7.37
7.36
7.36
7.35
7.34
7.32
7.31
7.30
7.29
7.01
7.01
4.55
4.52
4.40
4.37
1.97
1.23
1.05
3.13
2.08
2.06
1.01
1.00
1.05
1.07
0.95
3.11
9.39
10.0 9.5 9.0 8.5 8.0 7.5 7.0 6.5 6.0 5.5 5.0 4.5 4.0 3.5 3.0 2.5 2.0 1.5 1.0 0.5 0.0
δ (ppm)

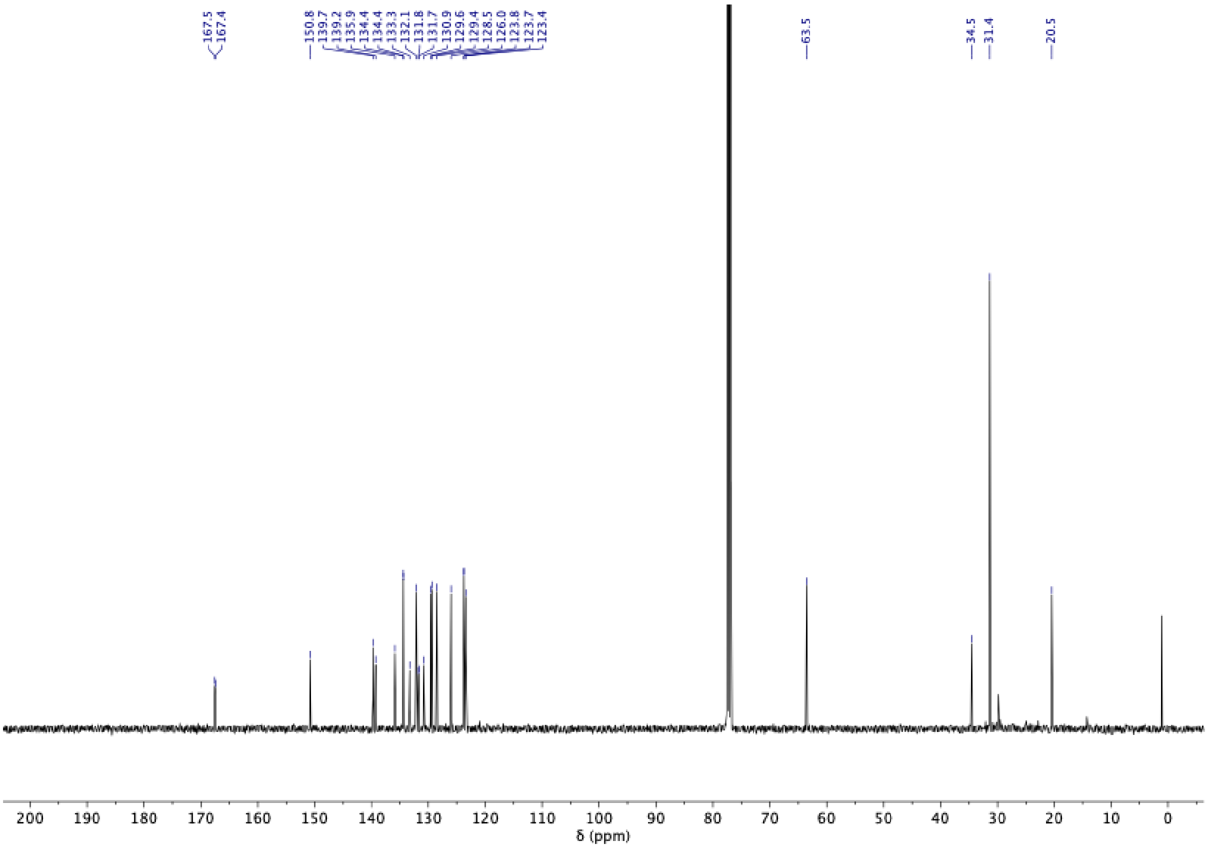
167.5
167.4
150.8
139.7
139.2
135.9
134.4
134.4
133.3
132.1
131.8
131.7
130.9
129.6
129.4
128.5
126.0
123.8
123.7
123.4
63.5
34.5
31.4
20.5
200 190 180 170 160 150 140 130 120 110 100 90 80 70 60 50 40 30 20 10 0
δ (ppm)

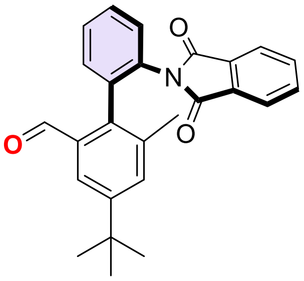

18b

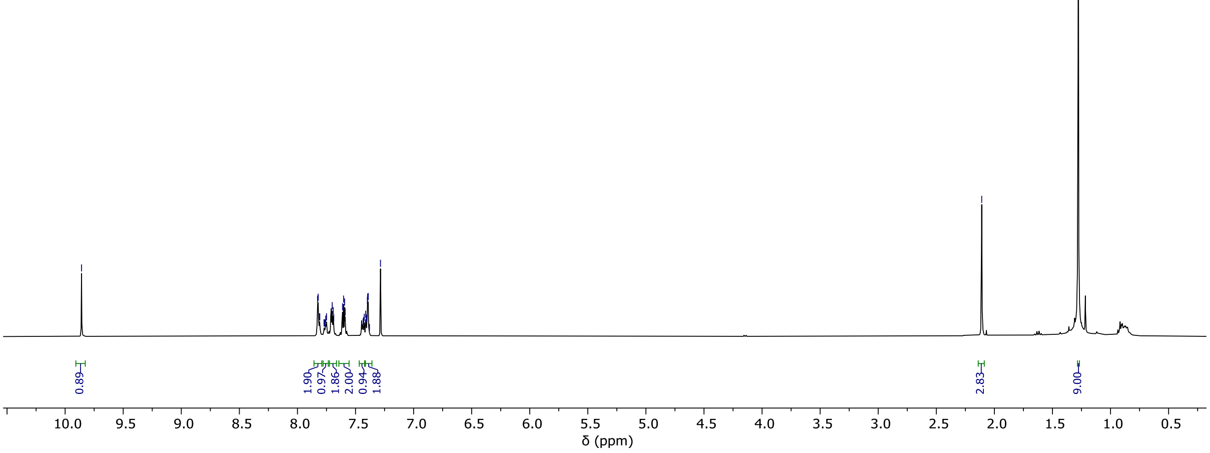

9.86
7.83 7.82 7.81 7.81 7.77 7.77 7.76 7.76 7.75 7.70 7.69 7.69 7.61 7.60 7.59 7.59 7.43 7.43 7.42 7.41 7.41 7.40 7.40 7.39 7.38 7.29
2.11
1.28
0.89
1.90 0.97 1.86 2.00 0.94 1.88
2.83
9.00
10.0 9.5 9.0 8.5 8.0 7.5 7.0 6.5 6.0 5.5 5.0 4.5 4.0 3.5 3.0 2.5 2.0 1.5 1.0 0.5
δ (ppm)

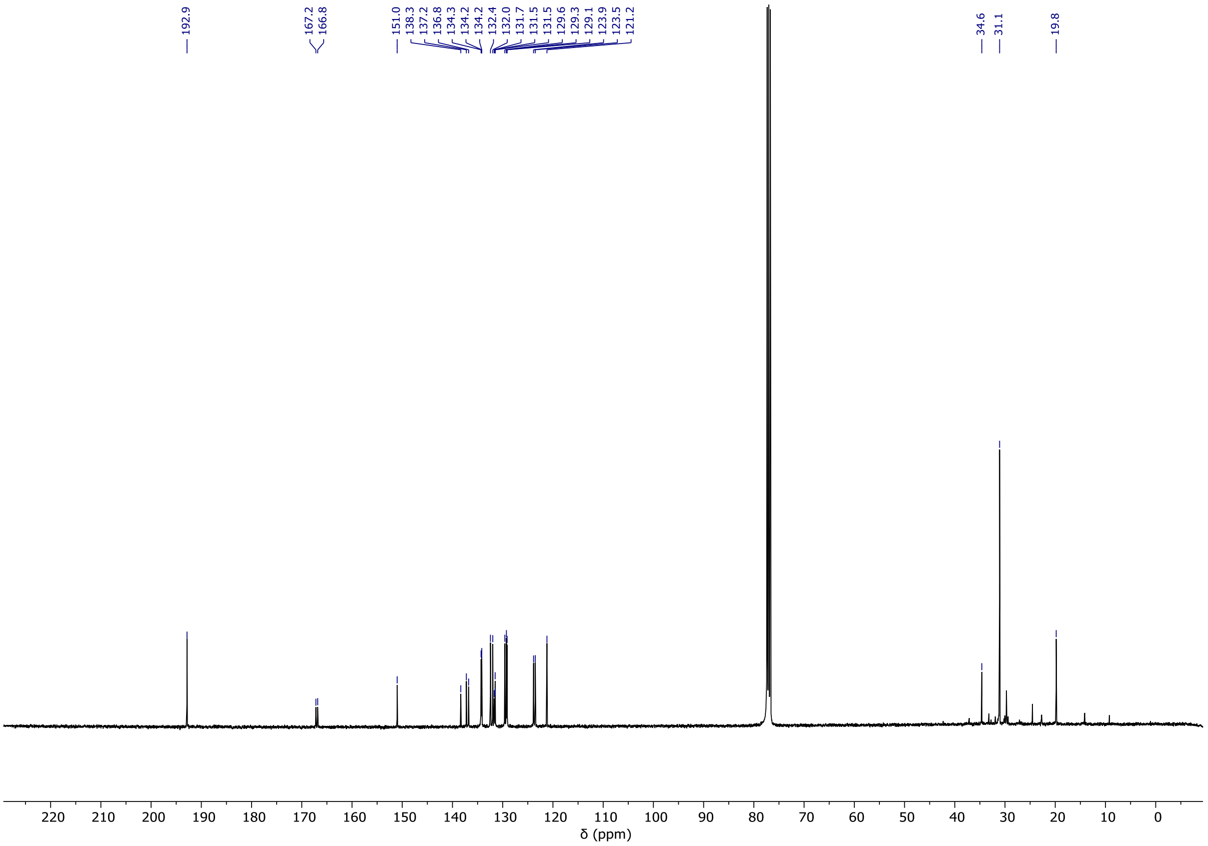

192.9
167.2 166.8
151.0 138.3 137.2 136.8 134.3 134.2 132.4 132.0 131.7 131.5 129.6 129.3 129.1 123.9 123.5 121.2
34.6 31.1
19.8
220 210 200 190 180 170 160 150 140 130 120 110 100 90 80 70 60 50 40 30 20 10 0
δ (ppm)

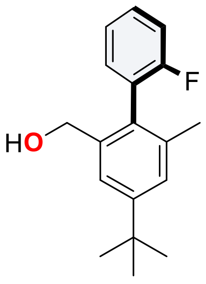
F
HO

19a

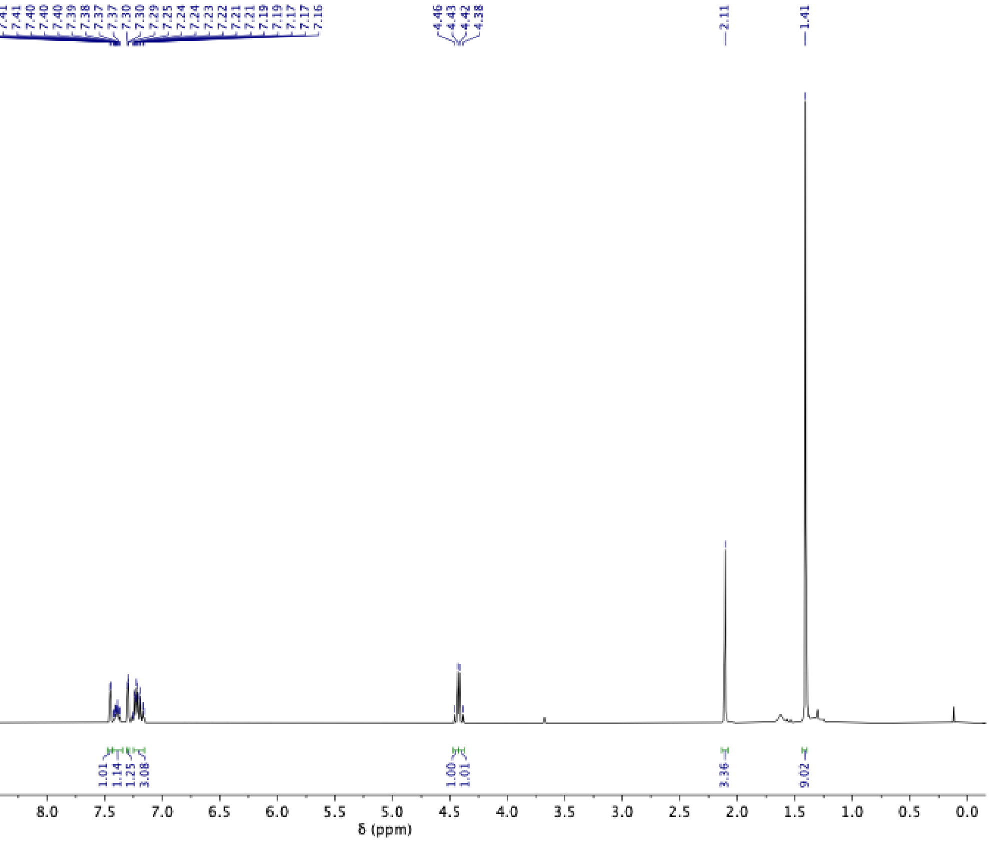
7.45
7.45
7.43
7.42
7.41
7.41
7.41
7.40
7.40
7.39
7.38
7.37
7.37
7.30
7.30
7.29
7.25
7.24
7.24
7.23
7.22
7.21
7.21
7.19
7.19
7.17
7.17
7.16
4.46
4.43
4.42
4.38
2.11
1.41
1.01
1.14
1.25
3.08
1.00
1.01
3.36
9.02
10.0 9.5 9.0 8.5 8.0 7.5 7.0 6.5 6.0 5.5 5.0 4.5 4.0 3.5 3.0 2.5 2.0 1.5 1.0 0.5 0.0
δ (ppm)

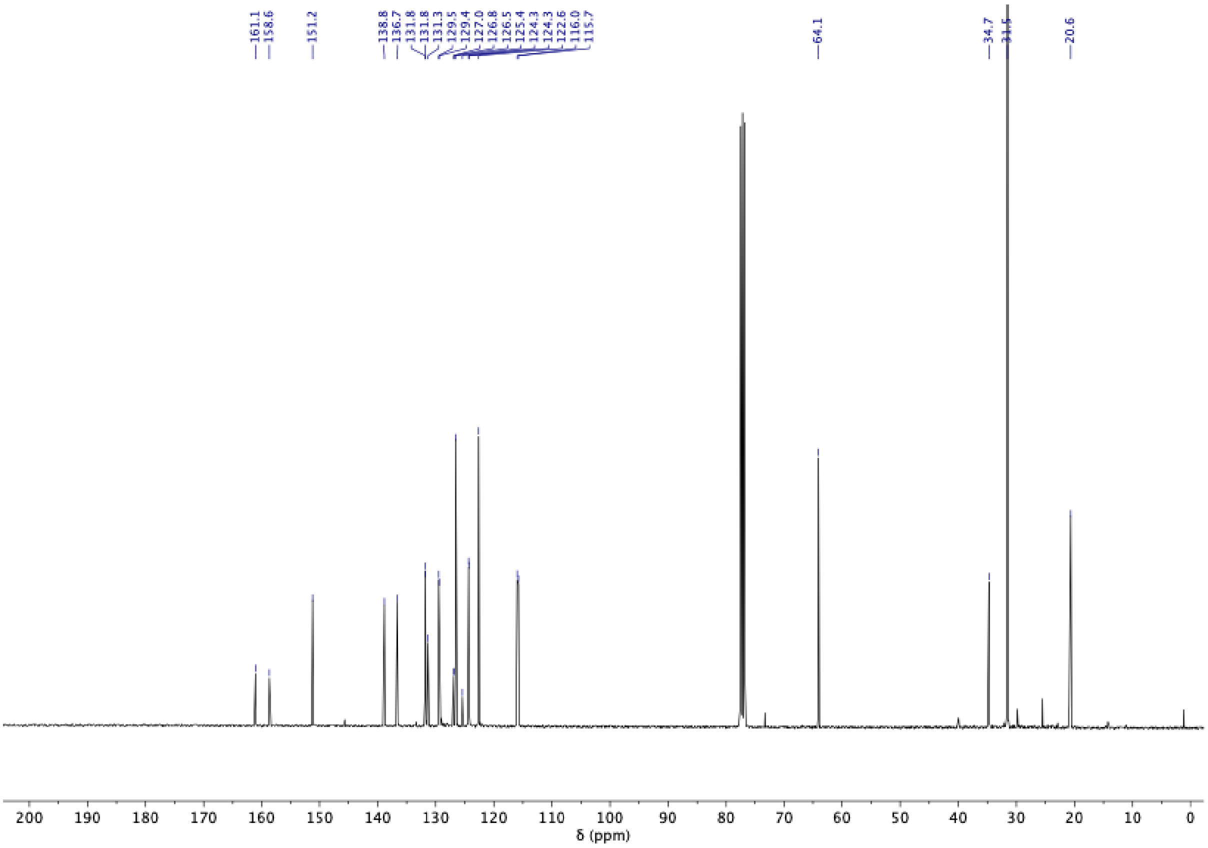
161.1
158.6
151.2
138.8
136.7
131.8
131.3
129.5
129.4
127.0
126.8
126.5
125.4
124.3
124.3
122.6
116.0
115.7
64.1
34.7
31.5
20.6
200 190 180 170 160 150 140 130 120 110 100 90 80 70 60 50 40 30 20 10 0
δ (ppm)

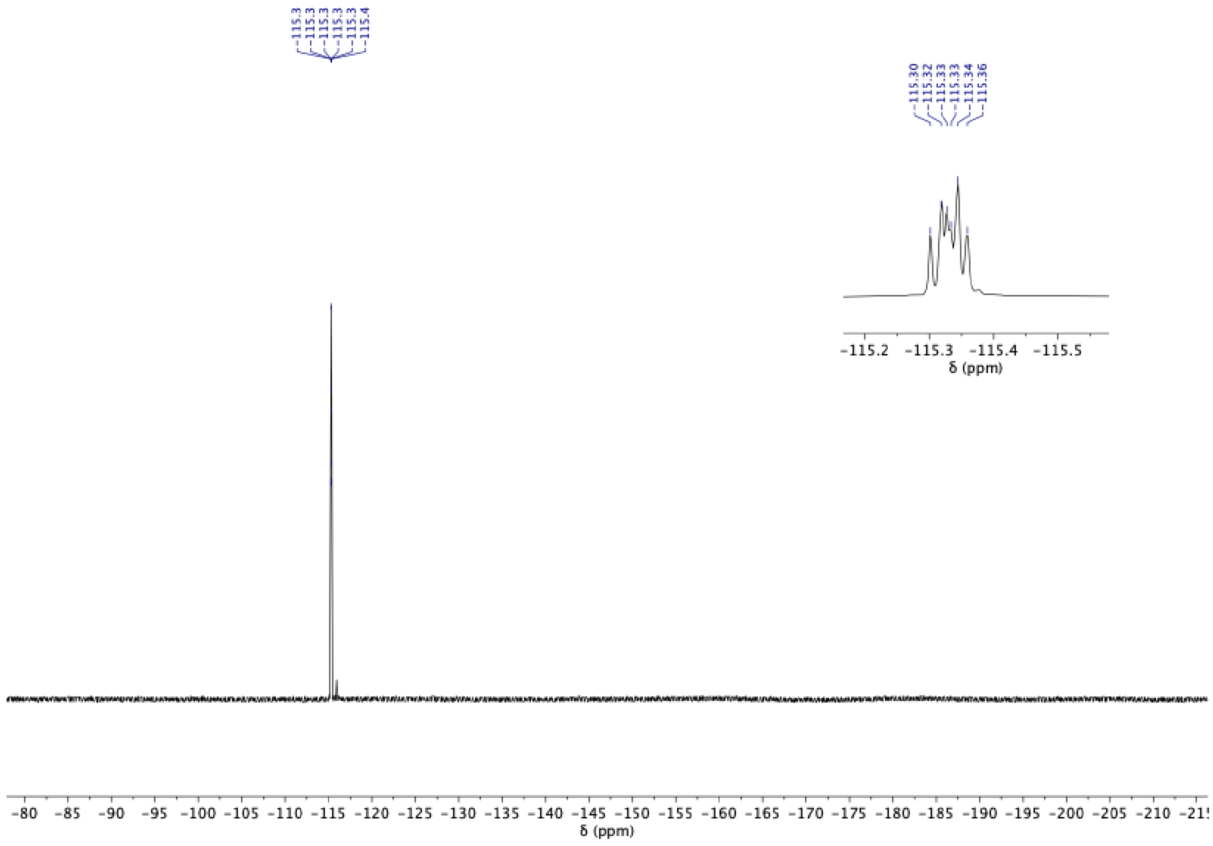
-115.3
-115.3
-115.3
-115.3
-115.3
-115.4
-115.30
-115.32
-115.33
-115.33
-115.34
-115.36
-115.2 -115.3 -115.4 -115.5
δ (ppm)
-80 -85 -90 -95 -100 -105 -110 -115 -120 -125 -130 -135 -140 -145 -150 -155 -160 -165 -170 -175 -180 -185 -190 -195 -200 -205 -210
δ (ppm)

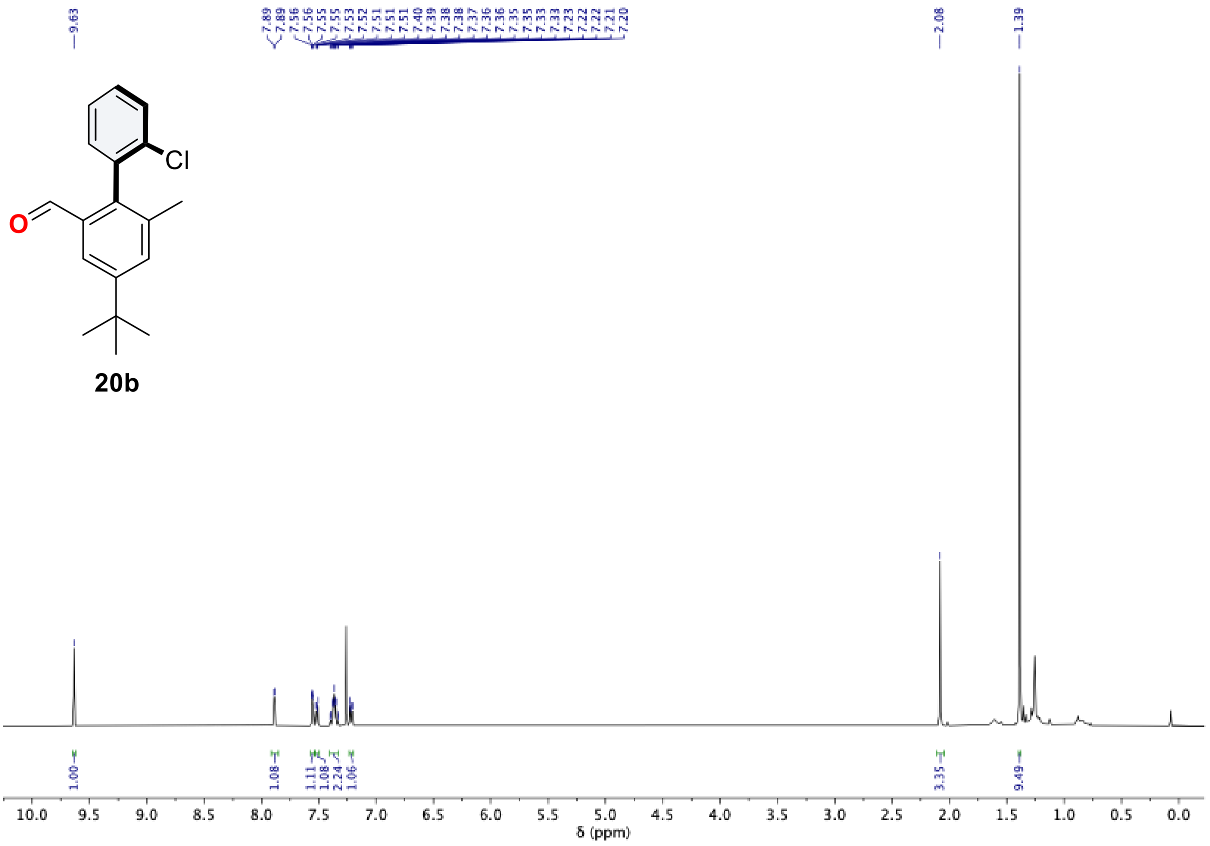
Cl
O
20b
9.63
7.89 7.89 7.56 7.55 7.55 7.53 7.52 7.51 7.51 7.51 7.40 7.39 7.38 7.38 7.37 7.36 7.36 7.35 7.35 7.33 7.33 7.23 7.22 7.22 7.21 7.20
2.08
1.39
1.00
1.08
1.11 1.08 2.24 1.06
3.35
9.49
10.0 9.5 9.0 8.5 8.0 7.5 7.0 6.5 6.0 5.5 5.0 4.5 4.0 3.5 3.0 2.5 2.0 1.5 1.0 0.5 0.0
δ (ppm)

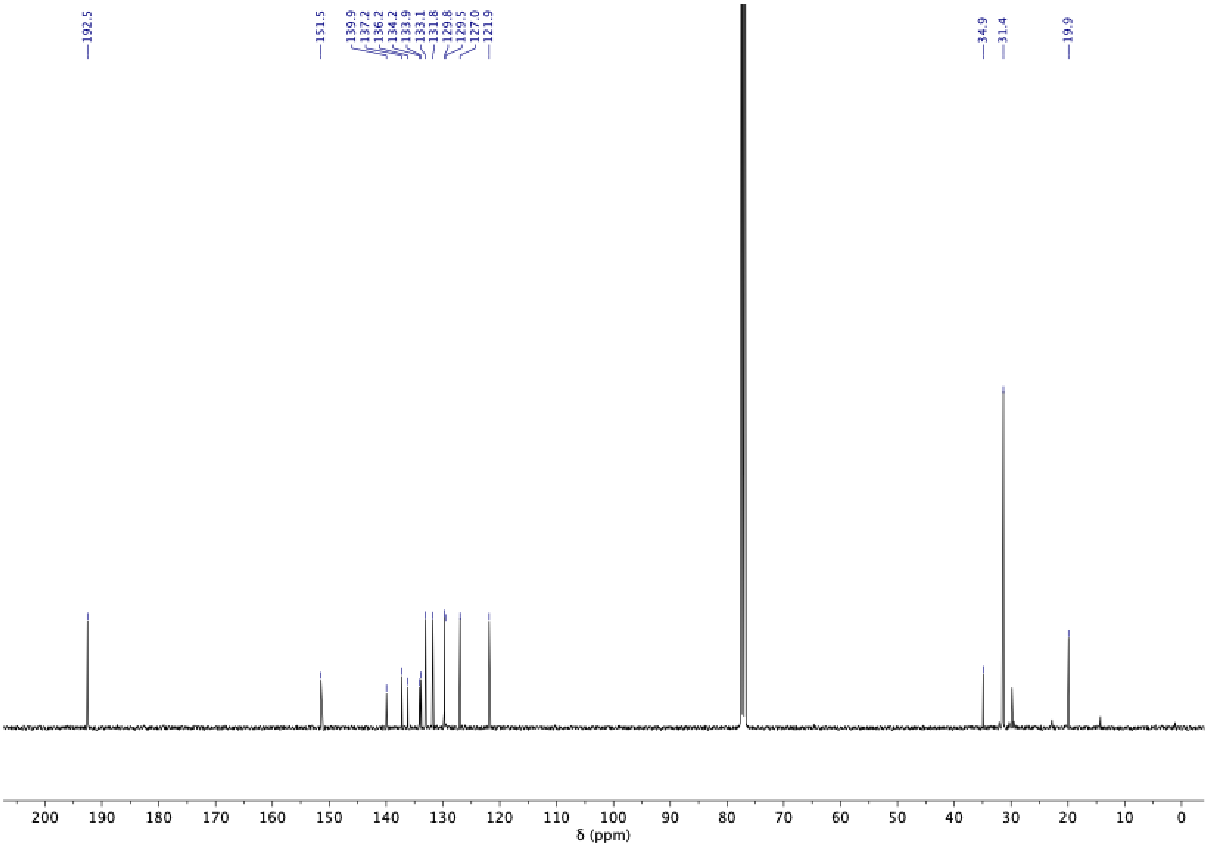
192.5
151.5
139.9 137.2 136.2 134.2 133.9 133.1 131.8 129.8 129.5 127.0 121.9
34.9 31.4
19.9
200 190 180 170 160 150 140 130 120 110 100 90 80 70 60 50 40 30 20 10 0
δ (ppm)

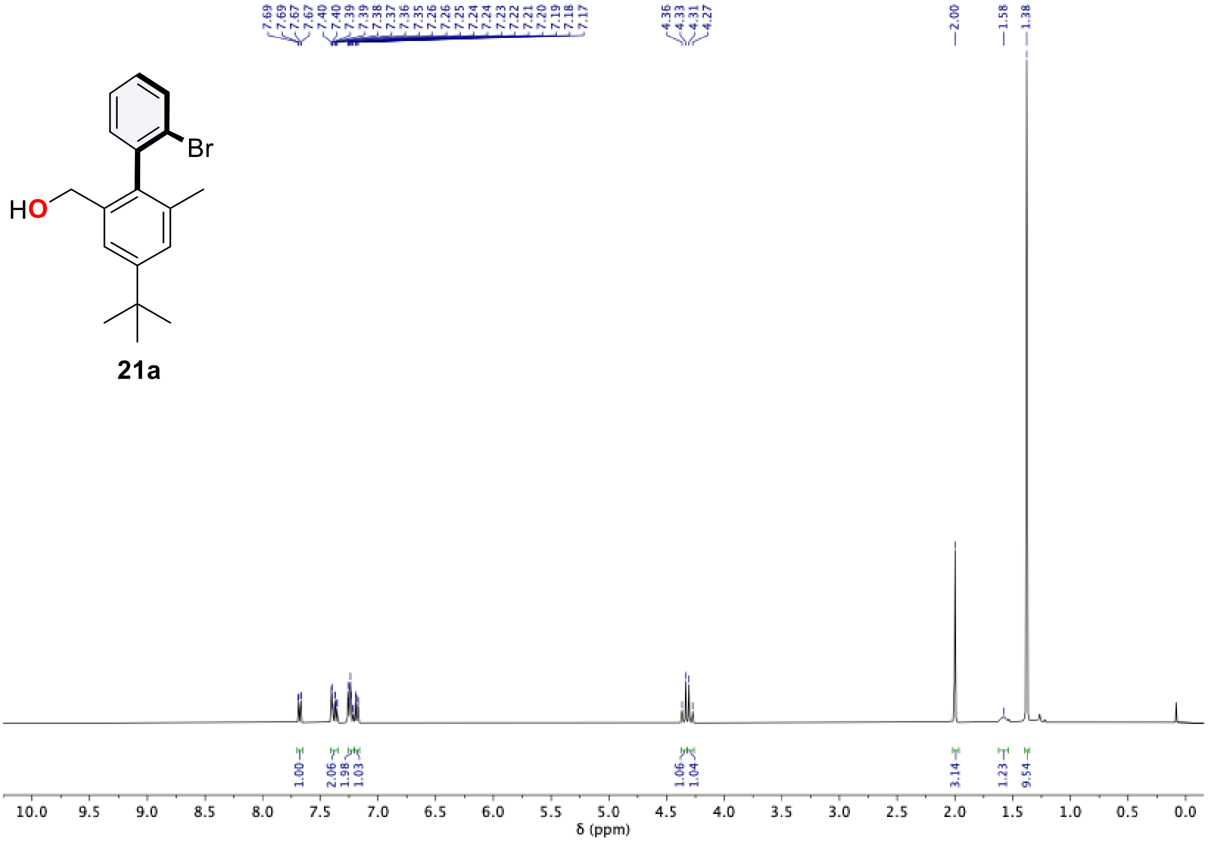
Br
HO
7.69
7.69
7.67
7.67
7.40
7.40
7.39
7.39
7.38
7.37
7.36
7.35
7.26
7.26
7.25
7.24
7.24
7.23
7.22
7.21
7.20
7.19
7.18
7.17
4.36
4.33
4.31
4.27
2.00
1.58
1.38
1.00
2.06
1.98
1.03
1.06
1.04
3.14
1.23
9.54
10.0 9.5 9.0 8.5 8.0 7.5 7.0 6.5 6.0 5.5 5.0 4.5 4.0 3.5 3.0 2.5 2.0 1.5 1.0 0.5 0.0
δ (ppm)

21a

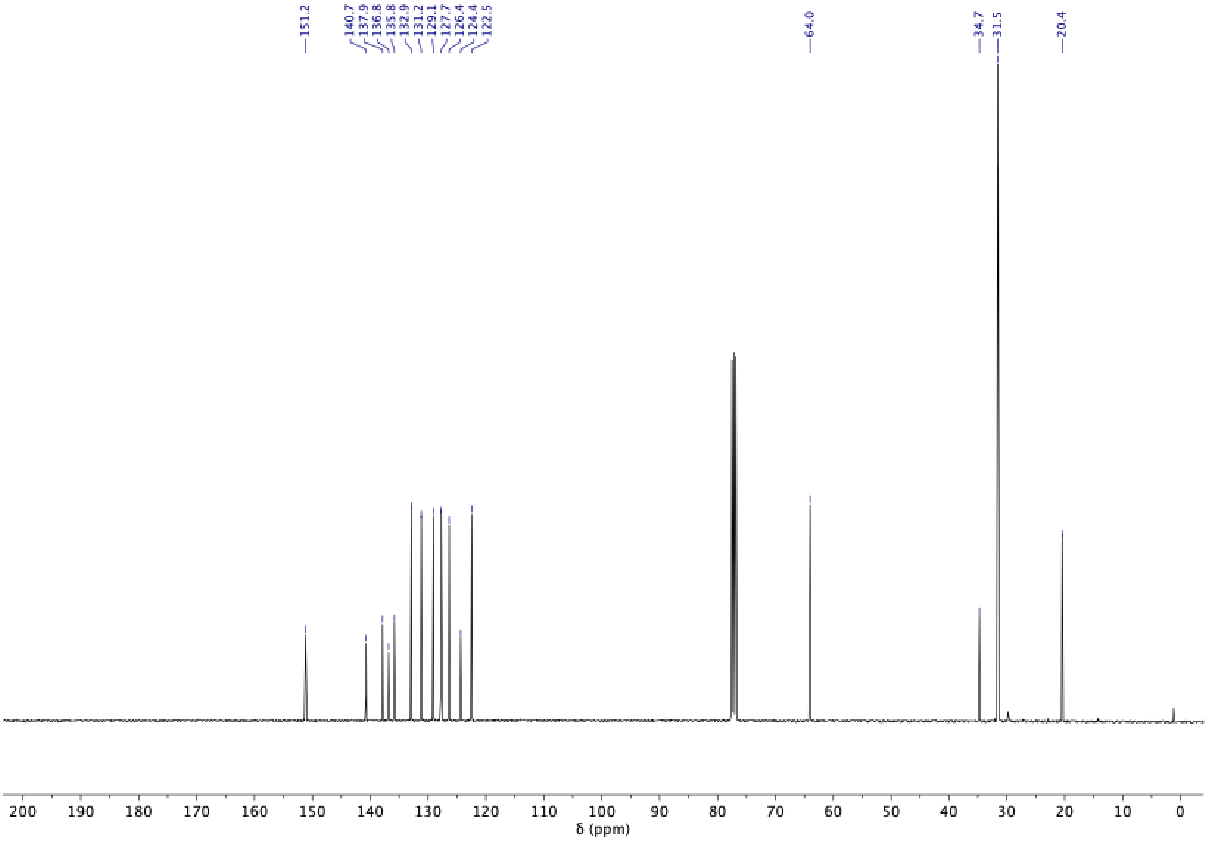
151.2
140.7
137.9
136.8
135.8
132.9
131.2
129.1
127.7
126.4
124.4
122.5
64.0
34.7
31.5
20.4
200 190 180 170 160 150 140 130 120 110 100 90 80 70 60 50 40 30 20 10 0
δ (ppm)

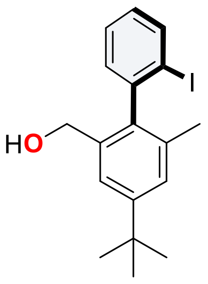
HO
I

22a

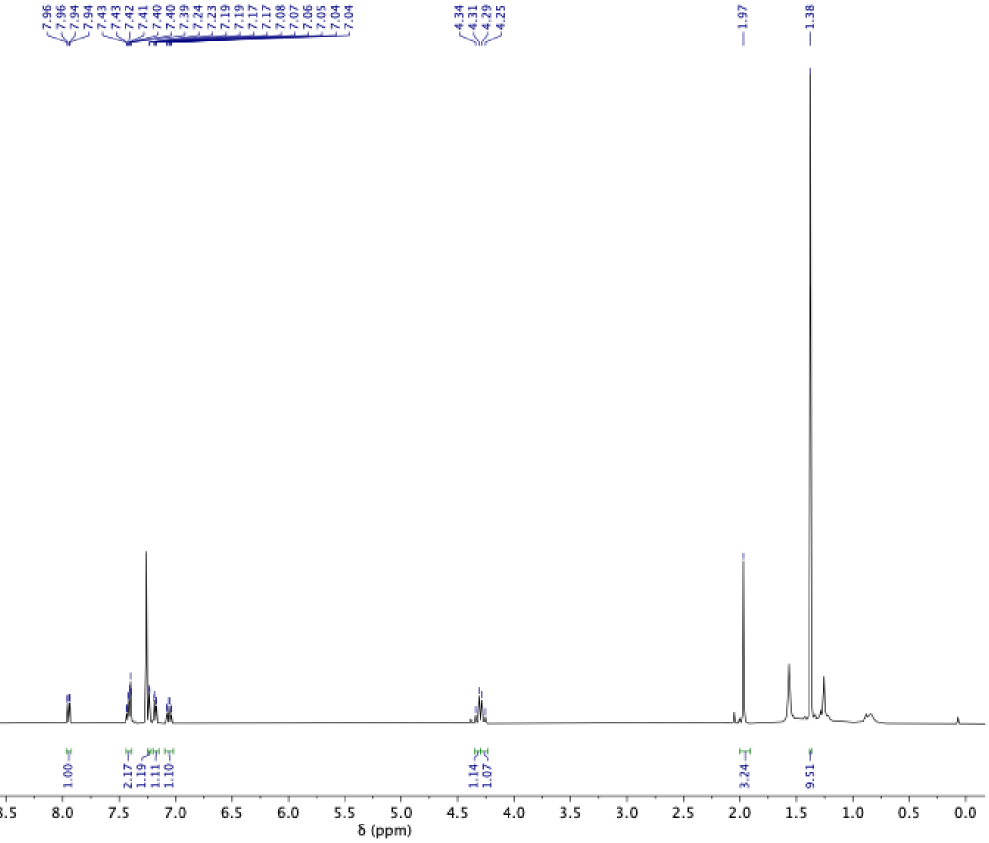
7.96
7.96
7.94
7.94
7.43
7.43
7.42
7.41
7.40
7.40
7.39
7.24
7.23
7.19
7.19
7.17
7.17
7.08
7.07
7.06
7.05
7.04
7.04
4.34
4.31
4.29
4.25
1.97
1.38
1.00
2.17
1.19
1.11
1.10
1.14
1.07
3.24
9.51
10.0 9.5 9.0 8.5 8.0 7.5 7.0 6.5 6.0 5.5 5.0 4.5 4.0 3.5 3.0 2.5 2.0 1.5 1.0 0.5 0.0
δ (ppm)

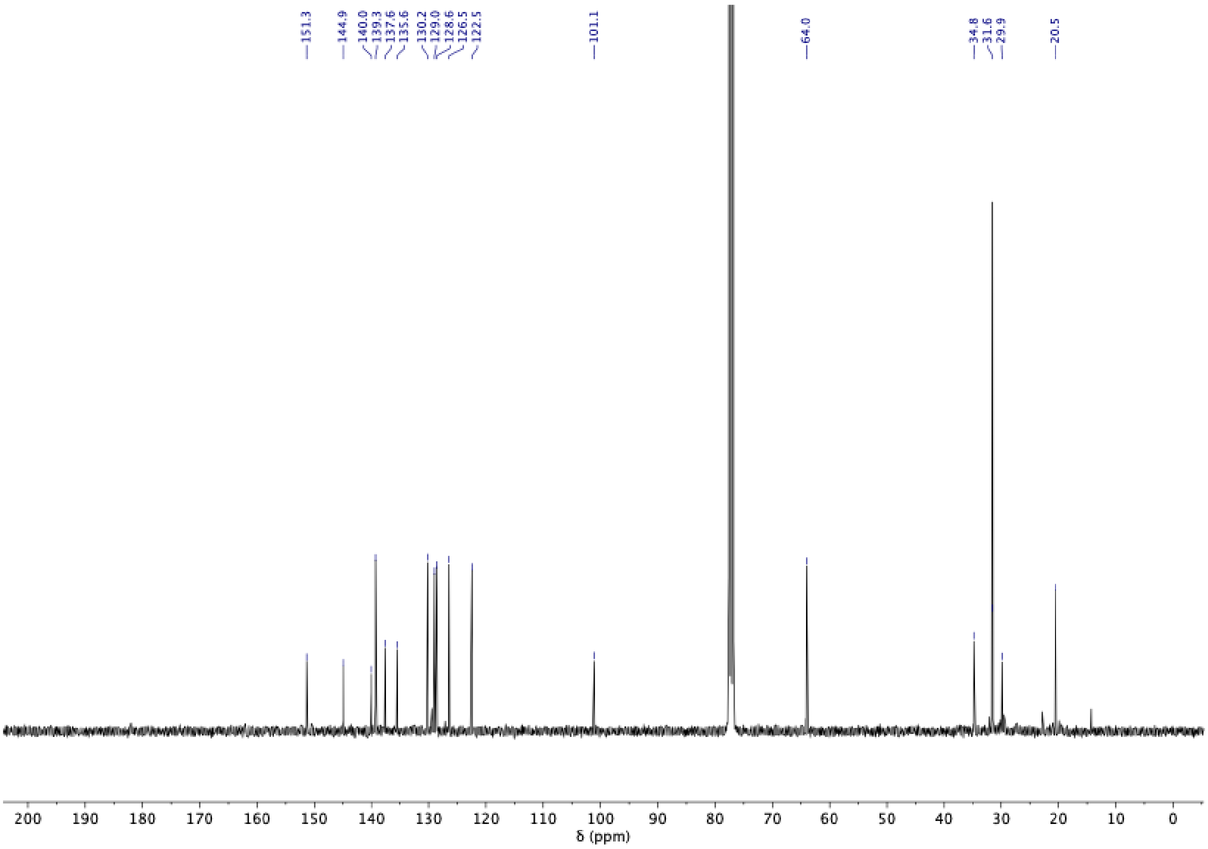
151.3
144.9
140.0
139.3
137.6
135.6
130.2
129.0
128.6
126.5
122.5
101.1
64.0
34.8
31.6
29.9
20.5
200 190 180 170 160 150 140 130 120 110 100 90 80 70 60 50 40 30 20 10 0
δ (ppm)

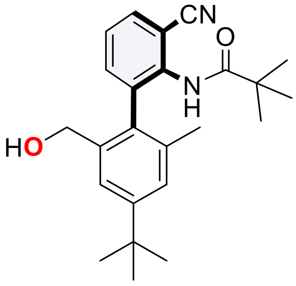
CN
O
N
H
HO

23a

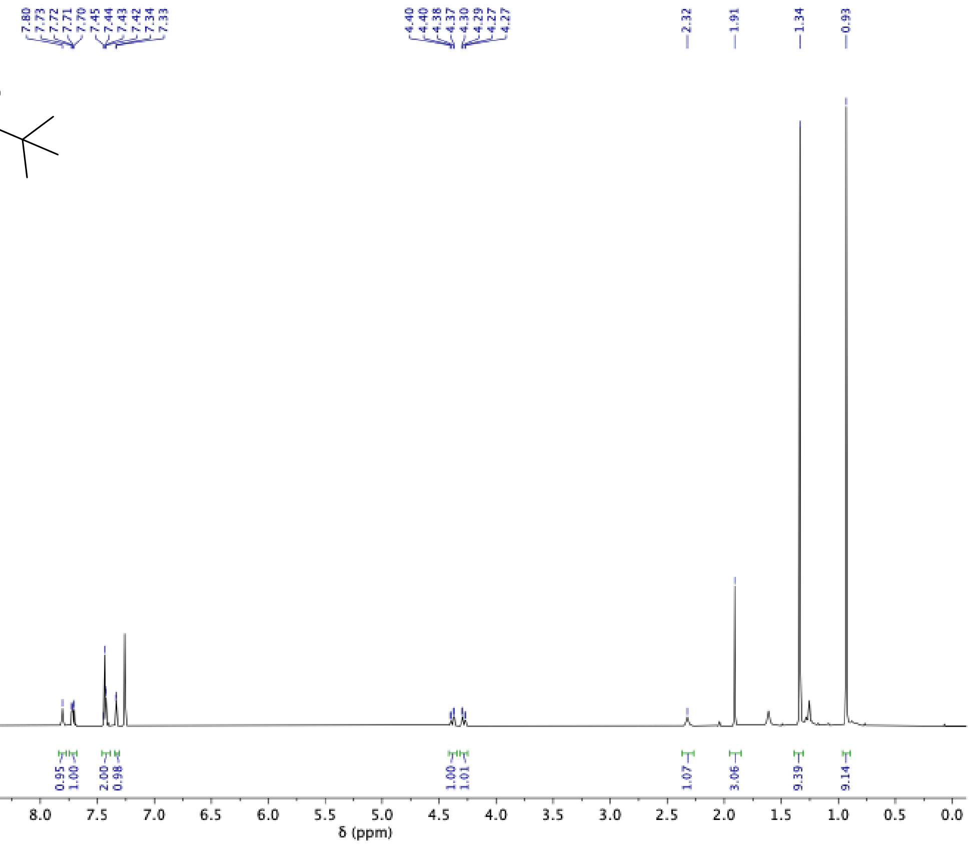
7.80
7.73
7.72
7.71
7.70
7.45
7.44
7.43
7.42
7.34
7.33
4.40
4.40
4.38
4.37
4.30
4.29
4.27
4.27
2.32
1.91
1.34
0.93
0.95
1.00
2.00
0.98
1.00
1.01
1.07
3.06
9.39
9.14
10.0 9.5 9.0 8.5 8.0 7.5 7.0 6.5 6.0 5.5 5.0 4.5 4.0 3.5 3.0 2.5 2.0 1.5 1.0 0.5 0.0
δ (ppm)

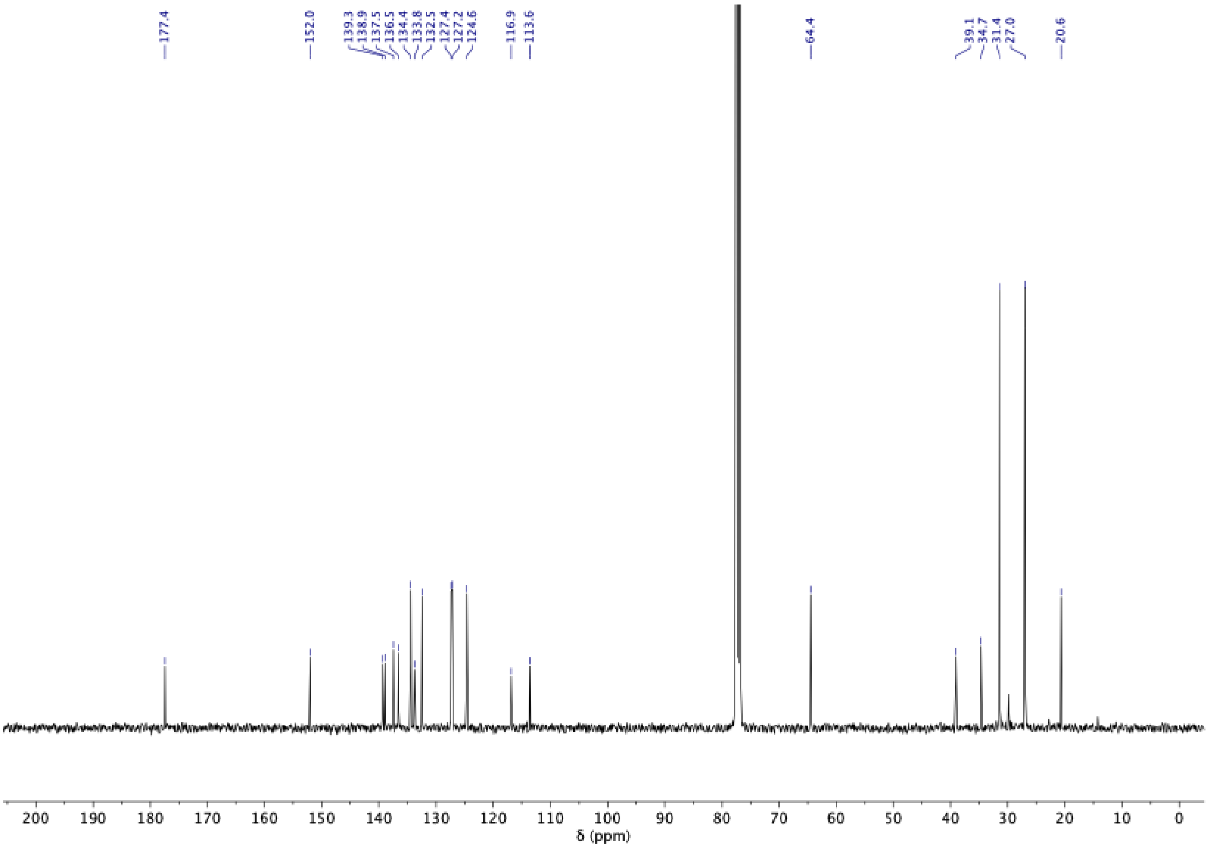
177.4
152.0
139.3
138.9
137.5
136.5
134.4
133.8
132.5
127.4
127.2
124.6
116.9
113.6
64.4
39.1
34.7
31.4
27.0
20.6
200 190 180 170 160 150 140 130 120 110 100 90 80 70 60 50 40 30 20 10 0
δ (ppm)

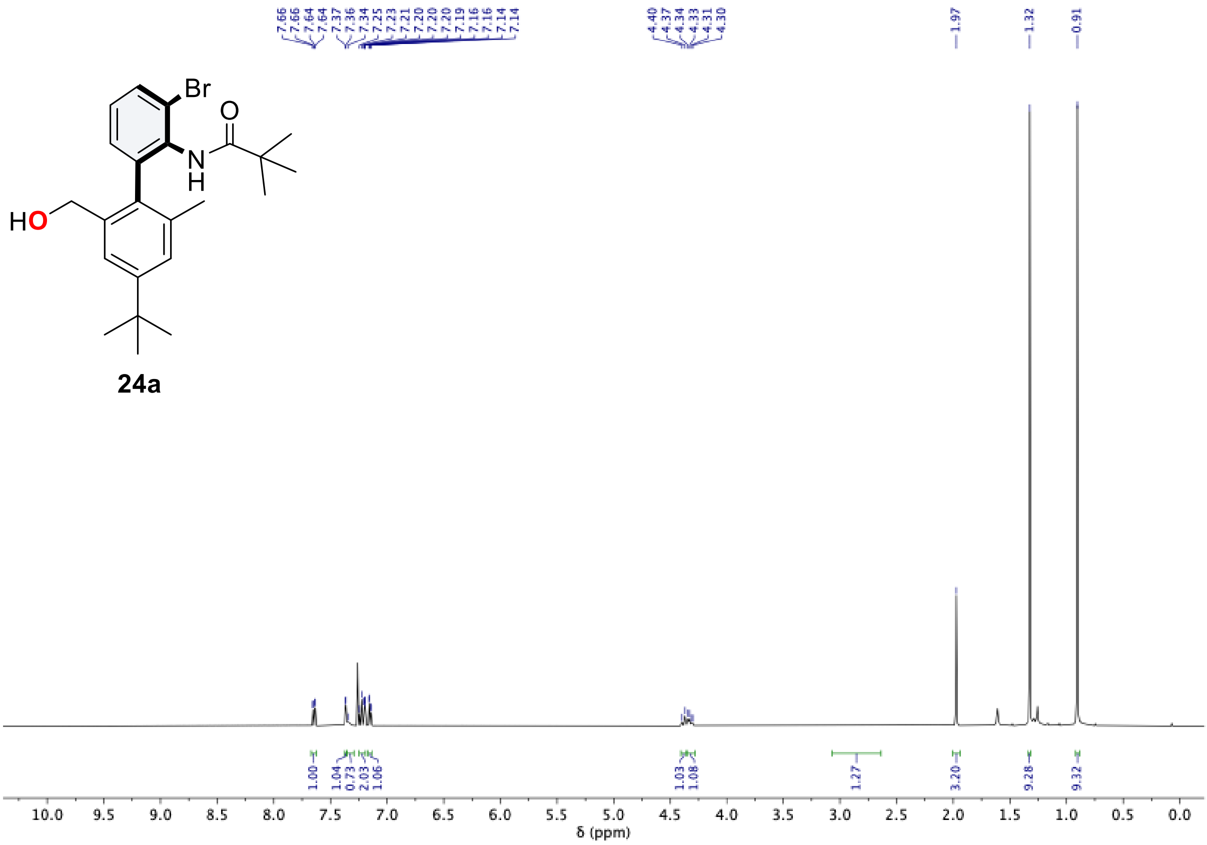
Br
O
N
H
HO
24a
7.66
7.66
7.64
7.64
7.37
7.36
7.34
7.25
7.23
7.21
7.20
7.20
7.20
7.19
7.16
7.16
7.14
7.14
4.40
4.37
4.34
4.33
4.31
4.30
1.97
1.32
0.91
1.00
1.04
0.73
2.03
1.06
1.03
1.08
1.27
3.20
9.28
9.32
10.0 9.5 9.0 8.5 8.0 7.5 7.0 6.5 6.0 5.5 5.0 4.5 4.0 3.5 3.0 2.5 2.0 1.5 1.0 0.5 0.0
δ (ppm)

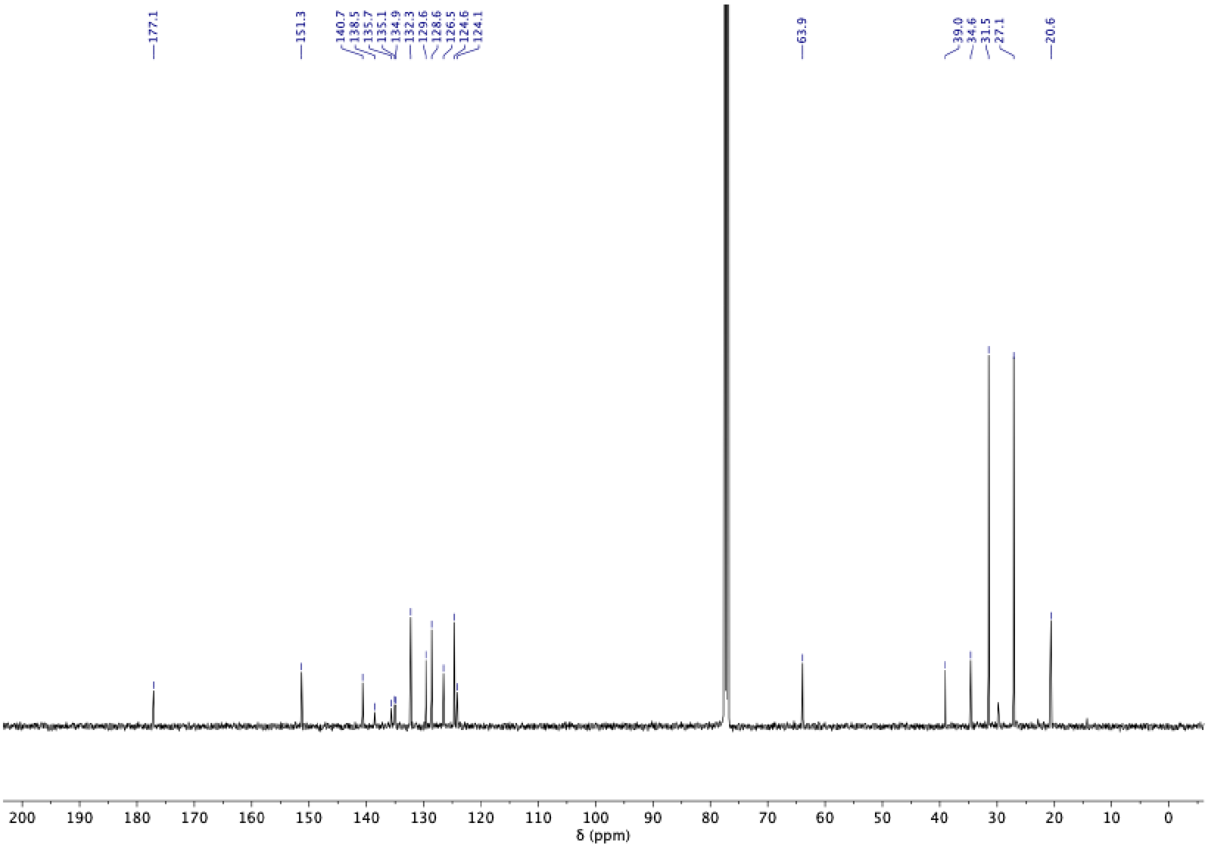
177.1
151.3
140.7
138.5
135.7
135.1
134.9
132.3
129.6
128.6
126.5
124.6
124.1
63.9
39.0
34.6
31.5
27.1
20.6
200 190 180 170 160 150 140 130 120 110 100 90 80 70 60 50 40 30 20 10 0
δ (ppm)

25b

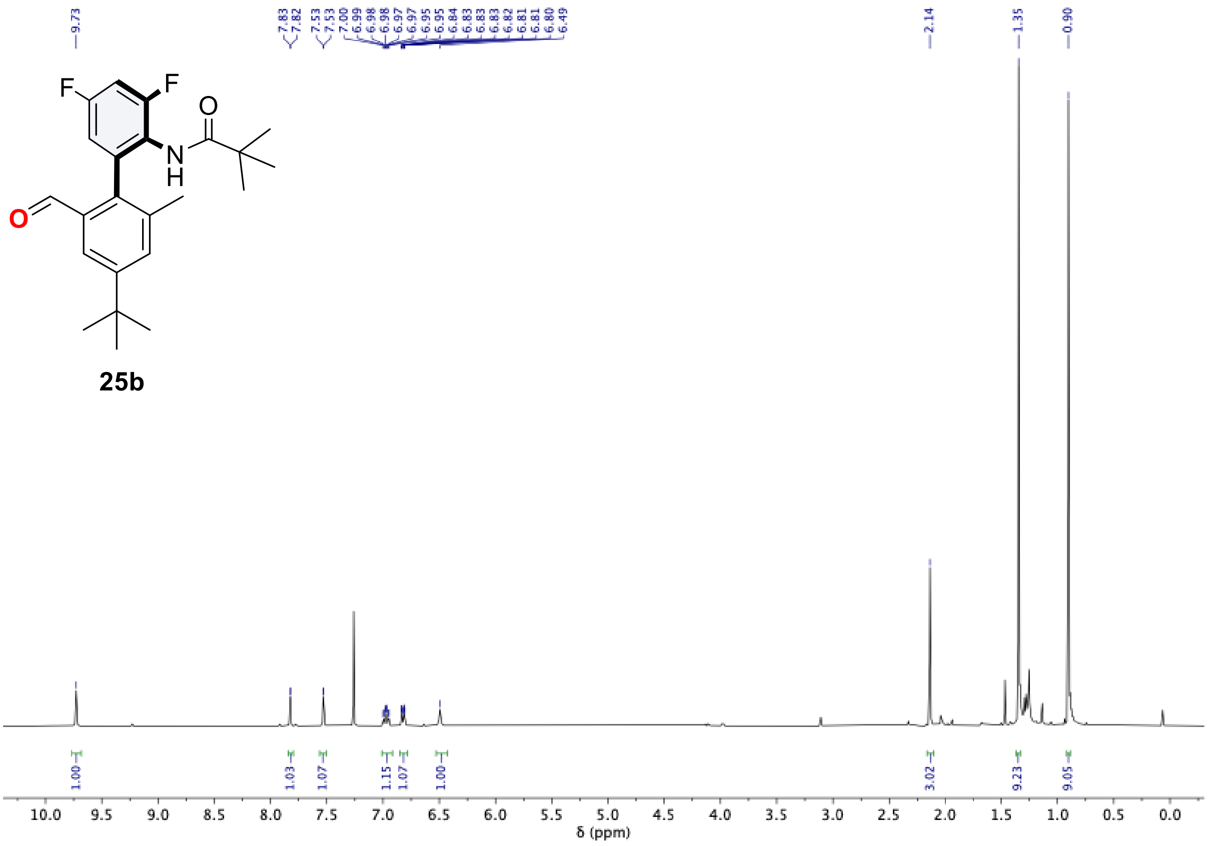
F
F
O
N
H
O
9.73
7.83
7.82
7.53
7.53
7.00
6.99
6.98
6.98
6.97
6.97
6.95
6.95
6.84
6.83
6.83
6.83
6.82
6.81
6.81
6.80
6.49
2.14
1.35
0.90
1.00
1.03
1.07
1.15
1.07
1.00
3.02
9.23
9.05
10.0 9.5 9.0 8.5 8.0 7.5 7.0 6.5 6.0 5.5 5.0 4.5 4.0 3.5 3.0 2.5 2.0 1.5 1.0 0.5 0.0
δ (ppm)

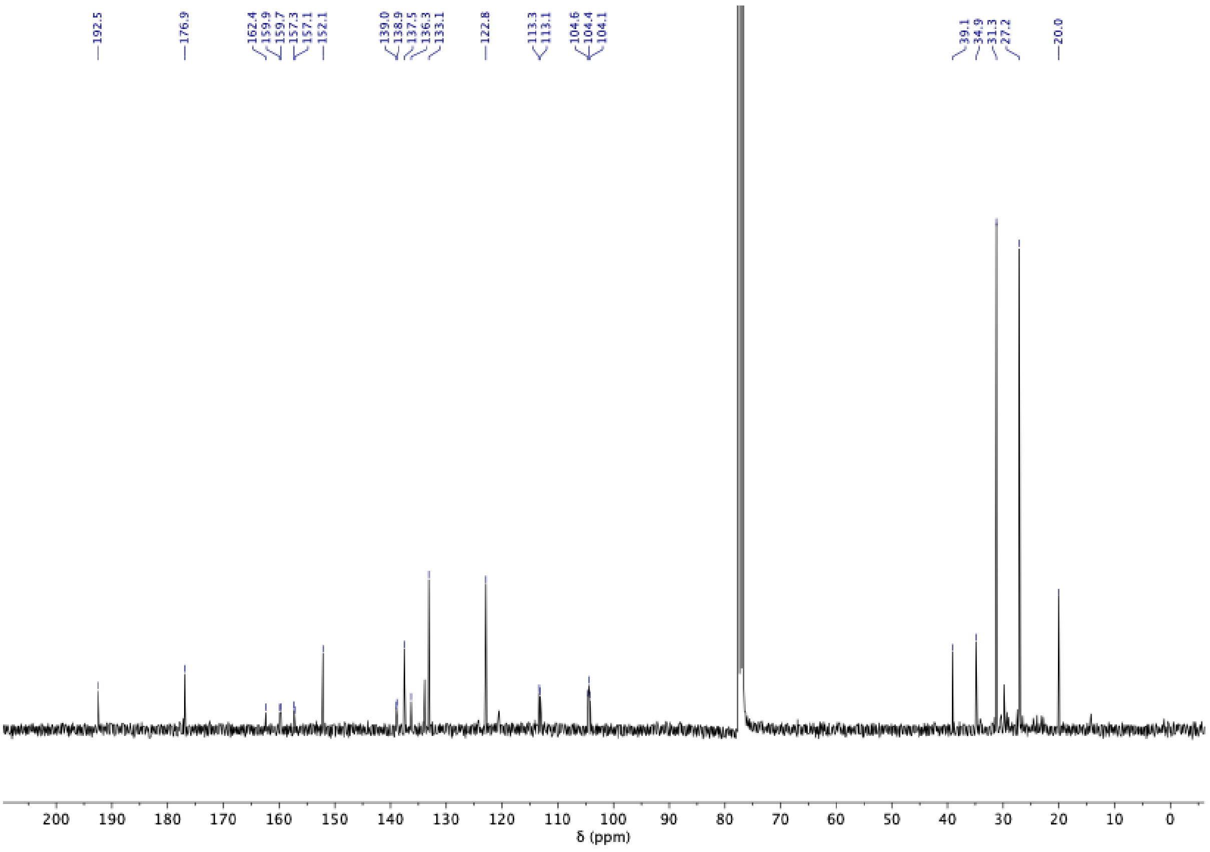
192.5
176.9
162.4
159.9
159.7
157.3
157.1
152.1
139.0
138.9
137.5
136.3
133.1
122.8
113.3
113.1
104.6
104.4
104.1
39.1
34.9
31.3
27.2
20.0
200 190 180 170 160 150 140 130 120 110 100 90 80 70 60 50 40 30 20 10 0
δ (ppm)

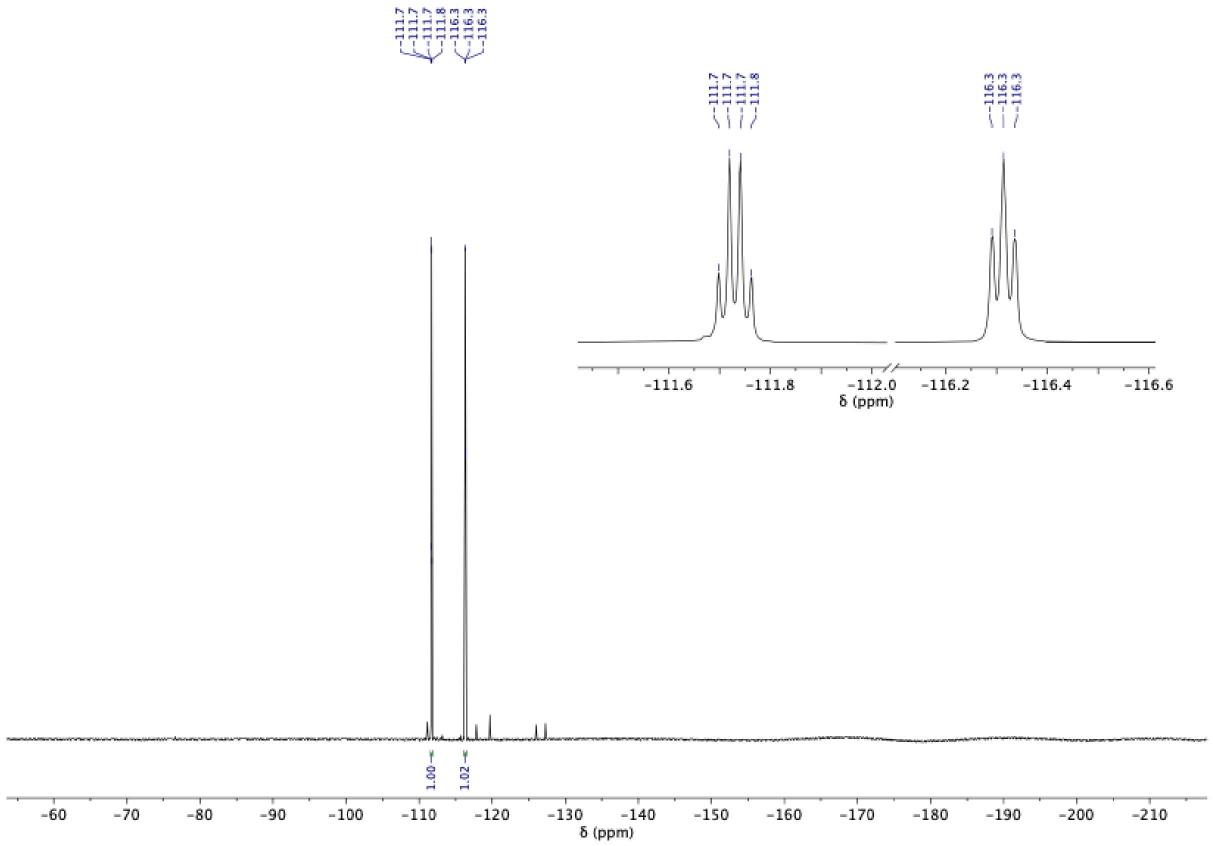

111.7
111.7
111.7
111.8
116.3
116.3
116.3
1.00
1.02
-60
-70
-80
-90
-100
-110
-120
-130
-140
-150
-160
-170
-180
-190
-200
-210
δ (ppm)
-111.6
-111.8
-112.0
-116.2
-116.4
-116.6

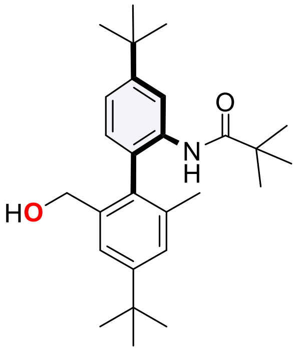
O
N
H
HO

26a

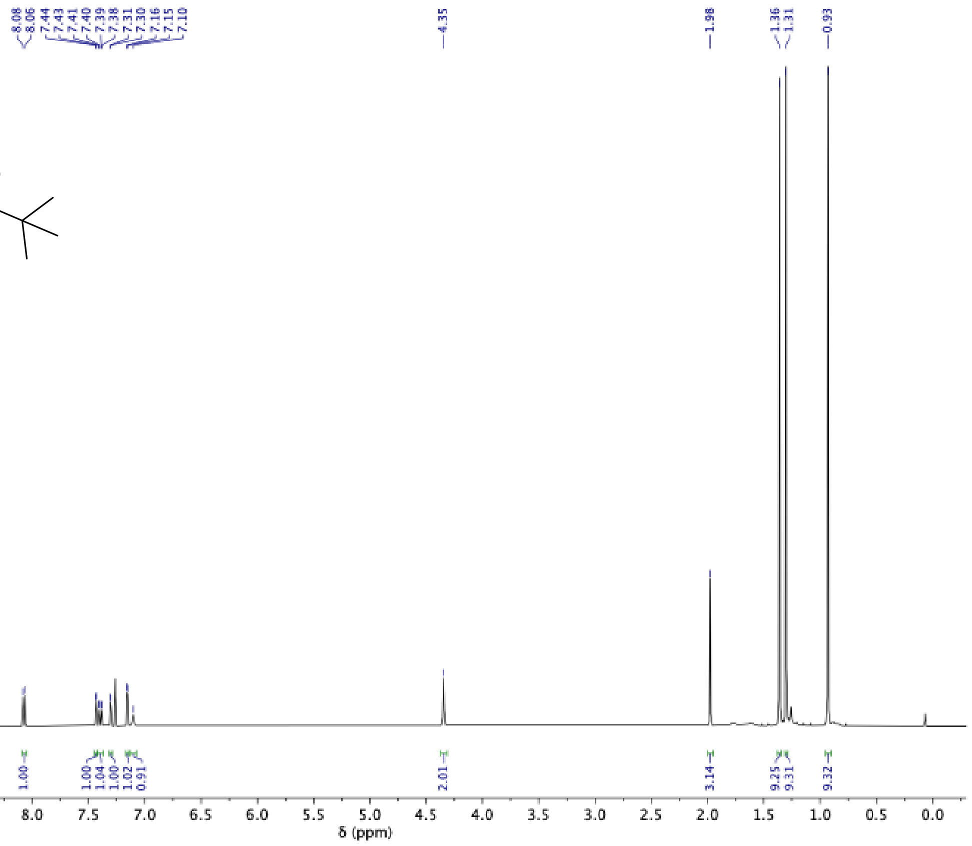
8.08
8.06
7.44
7.43
7.41
7.40
7.39
7.38
7.31
7.30
7.16
7.15
7.10
4.35
1.98
1.36
1.31
0.93
1.00
1.00
1.04
1.00
1.02
0.91
2.01
3.14
9.25
9.31
9.32
10.0 9.5 9.0 8.5 8.0 7.5 7.0 6.5 6.0 5.5 5.0 4.5 4.0 3.5 3.0 2.5 2.0 1.5 1.0 0.5 0.0
δ (ppm)

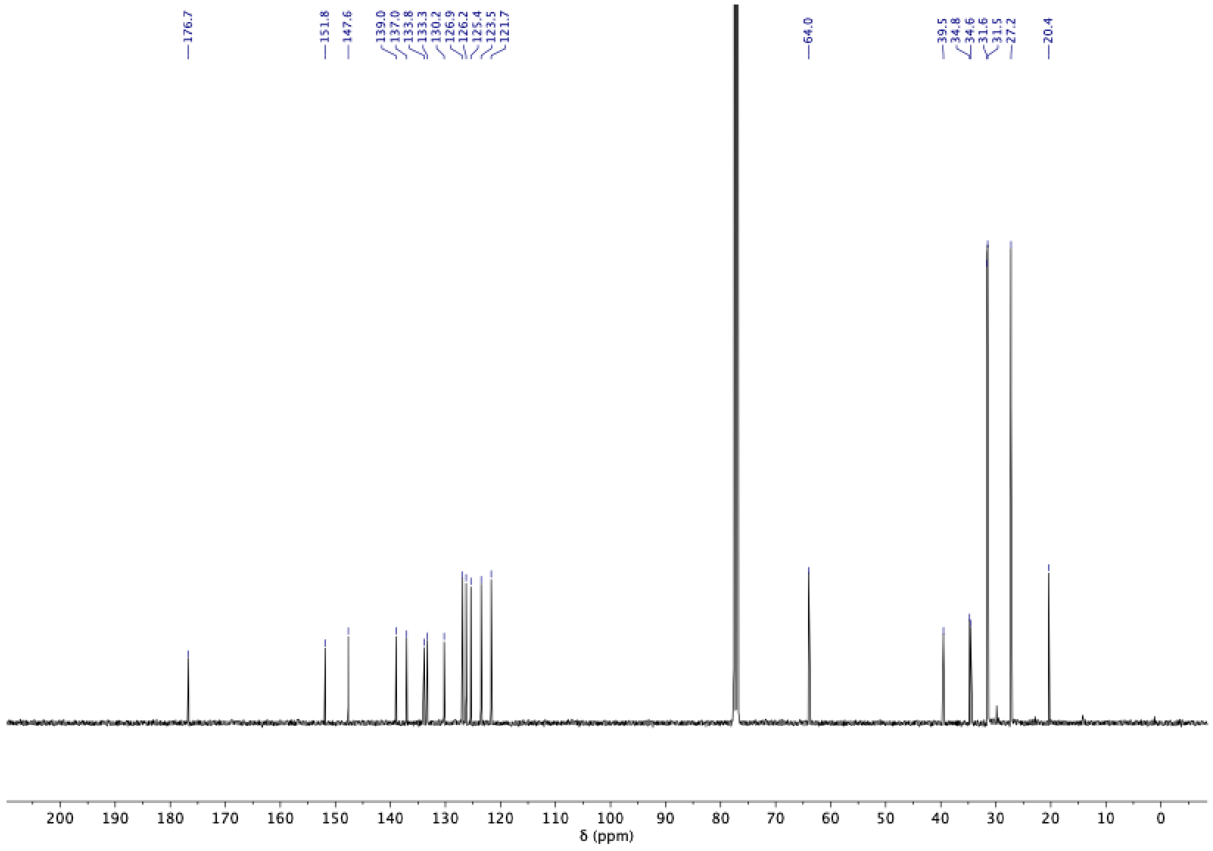
176.7
151.8
147.6
139.0
137.0
133.8
133.3
130.2
126.9
126.2
125.4
123.5
121.7
64.0
39.5
34.8
34.6
31.6
31.5
27.2
20.4
200 190 180 170 160 150 140 130 120 110 100 90 80 70 60 50 40 30 20 10 0
δ (ppm)

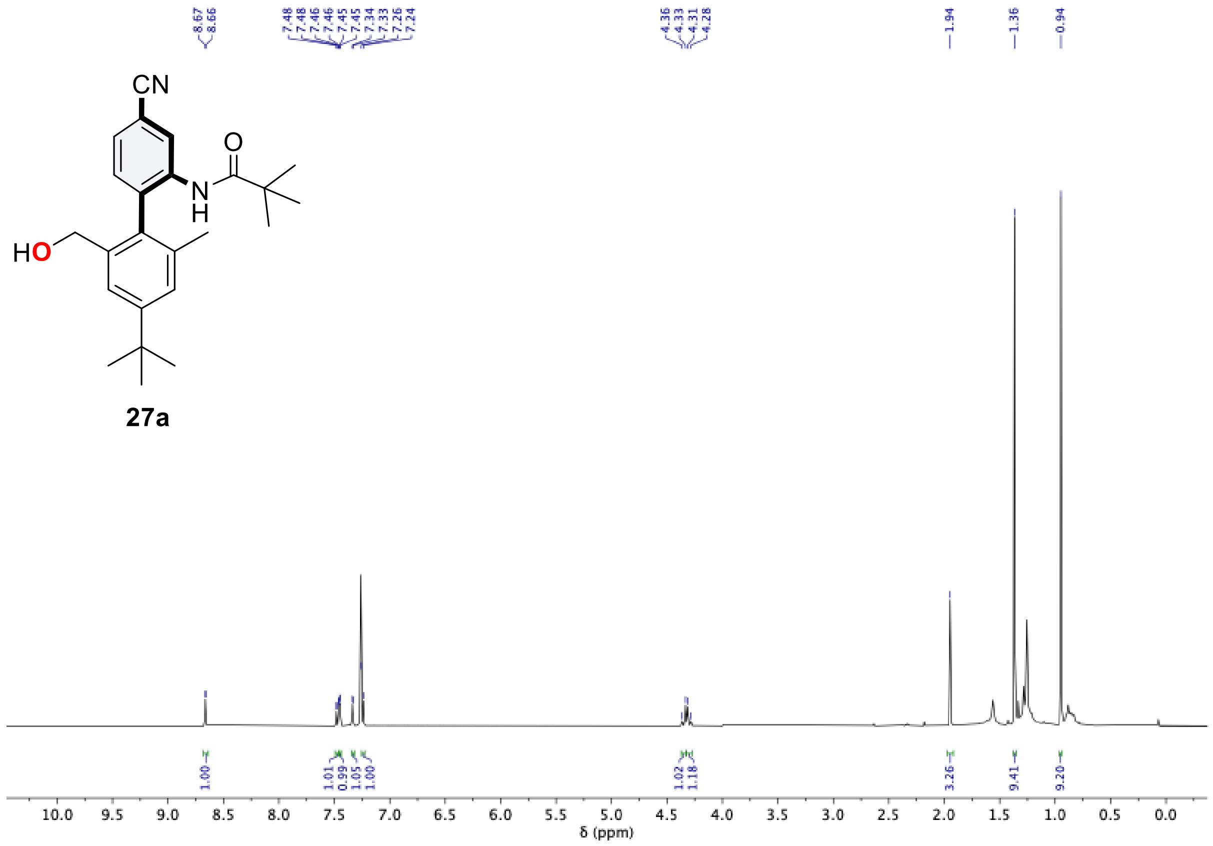
CN
O
N
H
HO
27a
8.67
8.66
7.48
7.48
7.46
7.46
7.45
7.45
7.34
7.33
7.26
7.24
4.36
4.33
4.31
4.28
1.94
1.36
0.94
1.00
1.01
0.99
1.05
1.00
1.02
1.18
3.26
9.41
9.20
10.0 9.5 9.0 8.5 8.0 7.5 7.0 6.5 6.0 5.5 5.0 4.5 4.0 3.5 3.0 2.5 2.0 1.5 1.0 0.5 0.0
δ (ppm)

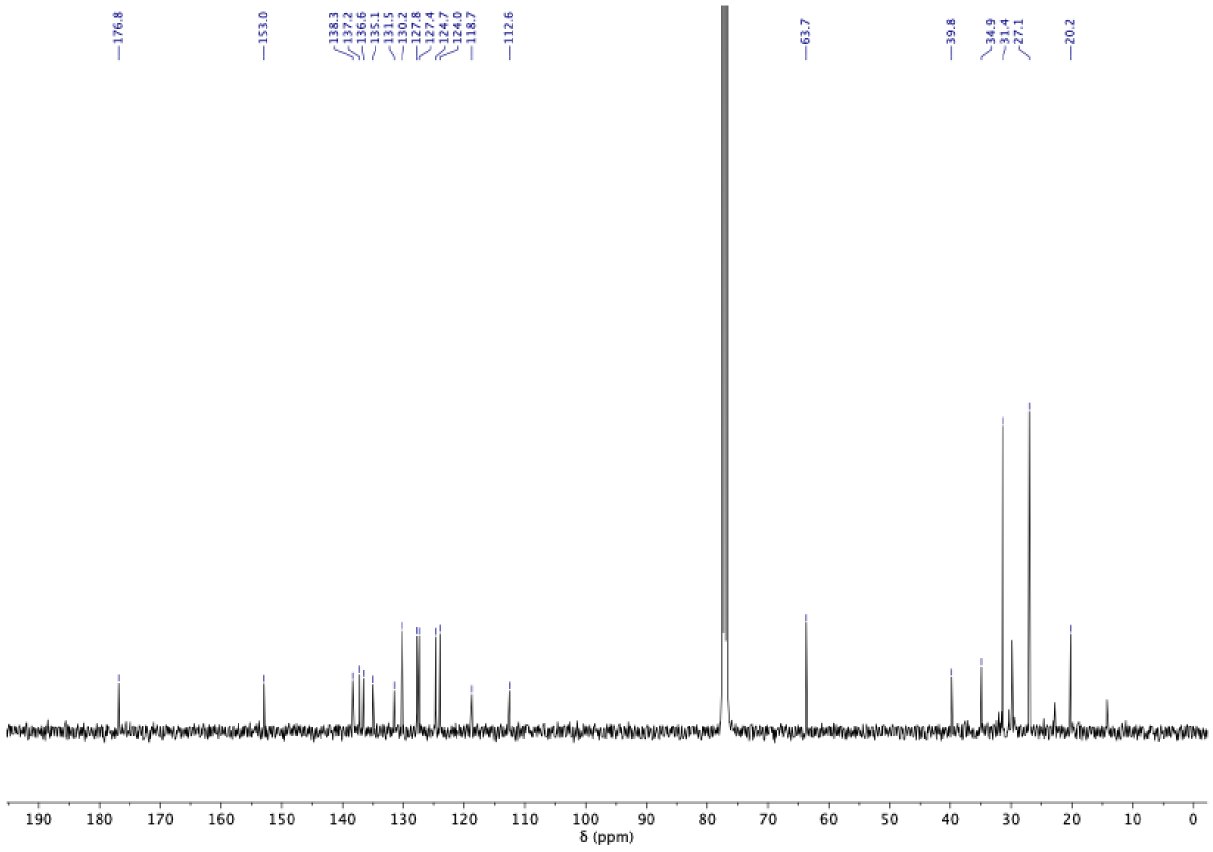
176.8
153.0
138.3
137.2
136.6
135.1
131.5
130.2
127.8
127.4
124.7
124.0
118.7
112.6
63.7
39.8
34.9
31.4
27.1
20.2
190 180 170 160 150 140 130 120 110 100 90 80 70 60 50 40 30 20 10 0
δ (ppm)

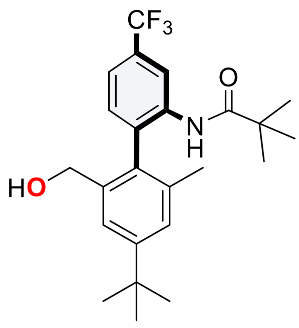
CF3
O
N
H
HO

28a

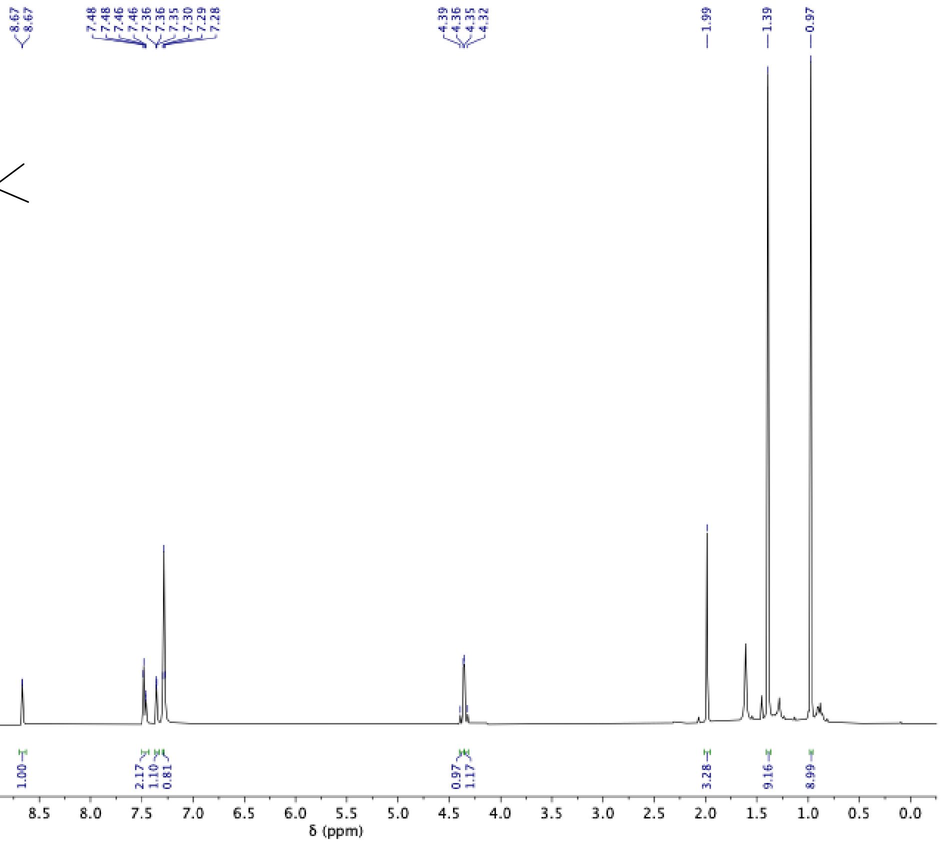
8.67
8.67
7.48
7.48
7.46
7.46
7.36
7.36
7.35
7.30
7.29
7.28
4.39
4.36
4.35
4.32
1.99
1.39
0.97
1.00
2.17
1.10
0.81
0.97
1.17
3.28
9.16
8.99
11.0 10.5 10.0 9.5 9.0 8.5 8.0 7.5 7.0 6.5 6.0 5.5 5.0 4.5 4.0 3.5 3.0 2.5 2.0 1.5 1.0 0.5 0.0
δ (ppm)

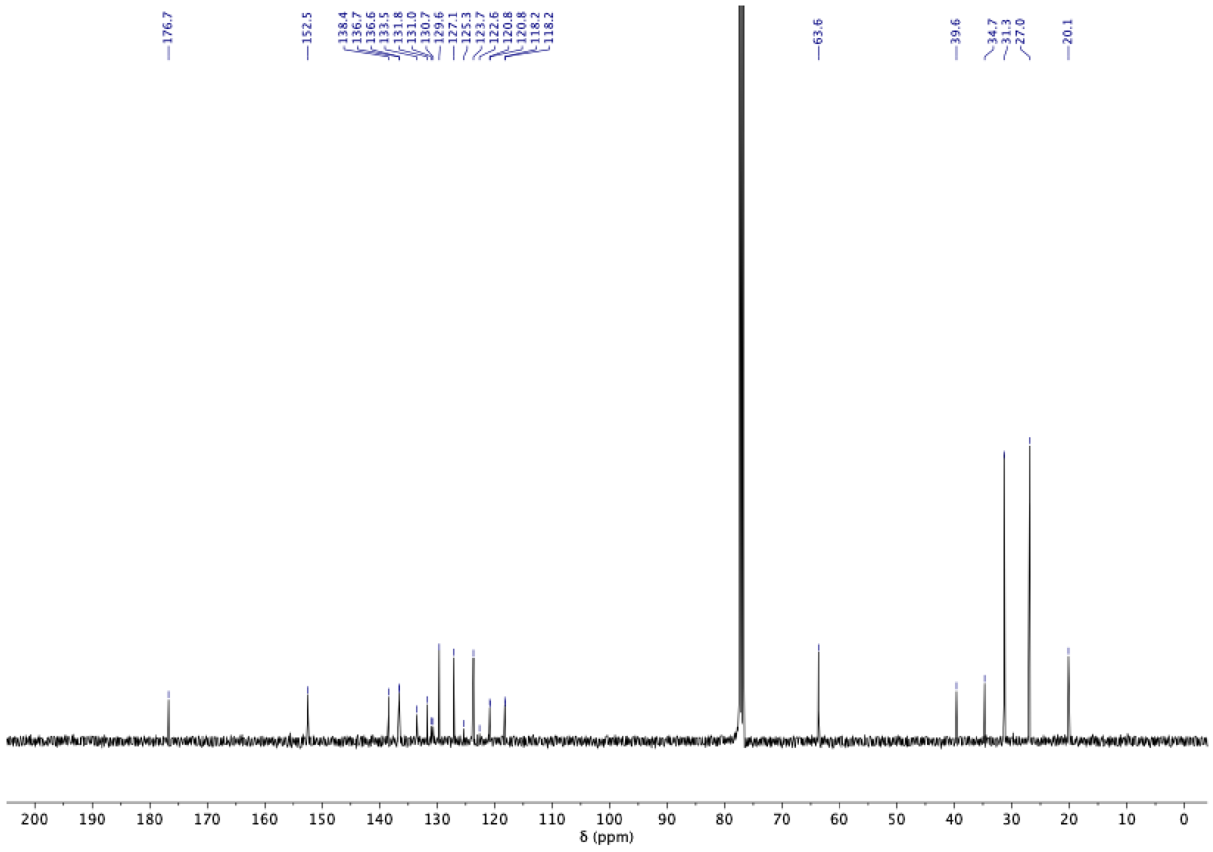
176.7
152.5
138.4
136.7
136.6
133.5
131.8
131.0
130.7
129.6
127.1
125.3
123.7
122.6
120.8
120.8
118.2
118.2
63.6
39.6
34.7
31.3
27.0
20.1
200 190 180 170 160 150 140 130 120 110 100 90 80 70 60 50 40 30 20 10 0
δ (ppm)

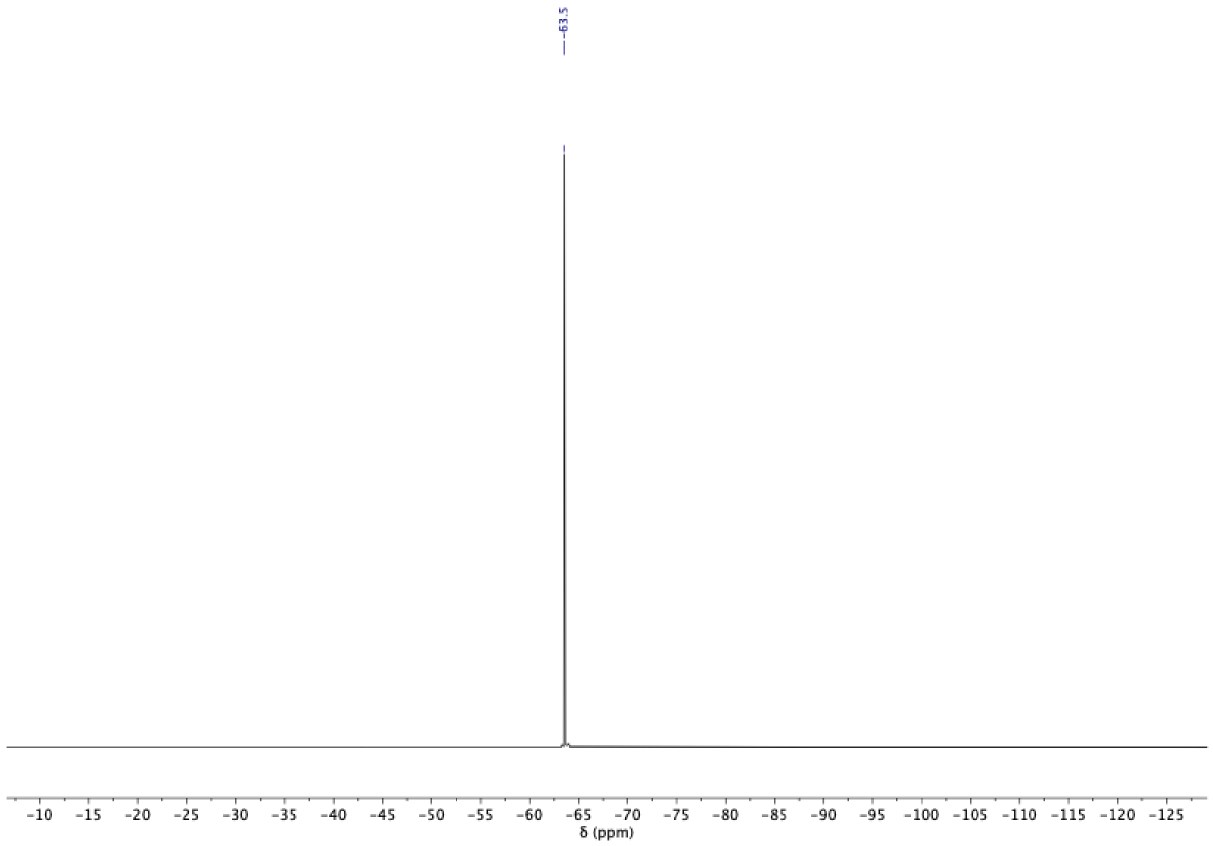
-63.5
-10 -15 -20 -25 -30 -35 -40 -45 -50 -55 -60 -65 -70 -75 -80 -85 -90 -95 -100 -105 -110 -115 -120 -125
δ (ppm)

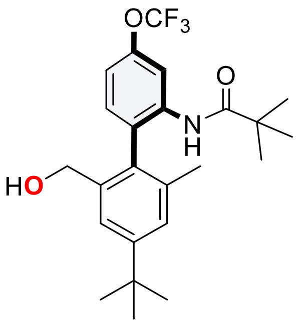
OCF3
O
N
H
HO

29a

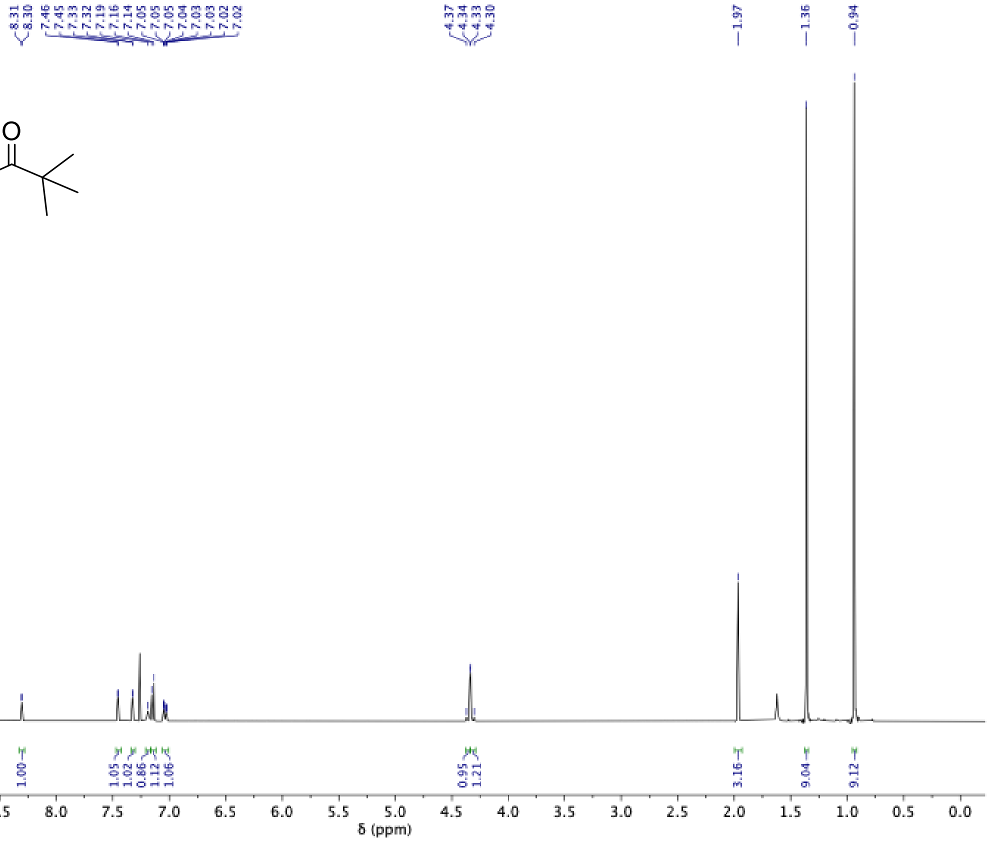
8.31
8.30
7.46
7.45
7.33
7.32
7.19
7.16
7.14
7.05
7.05
7.05
7.04
7.03
7.03
7.02
7.02
4.37
4.34
4.33
4.30
1.97
1.36
0.94
1.00
1.05
1.02
0.86
1.12
1.06
0.95
1.21
3.16
9.04
9.12
10.0 9.5 9.0 8.5 8.0 7.5 7.0 6.5 6.0 5.5 5.0 4.5 4.0 3.5 3.0 2.5 2.0 1.5 1.0 0.5 0.0
δ (ppm)

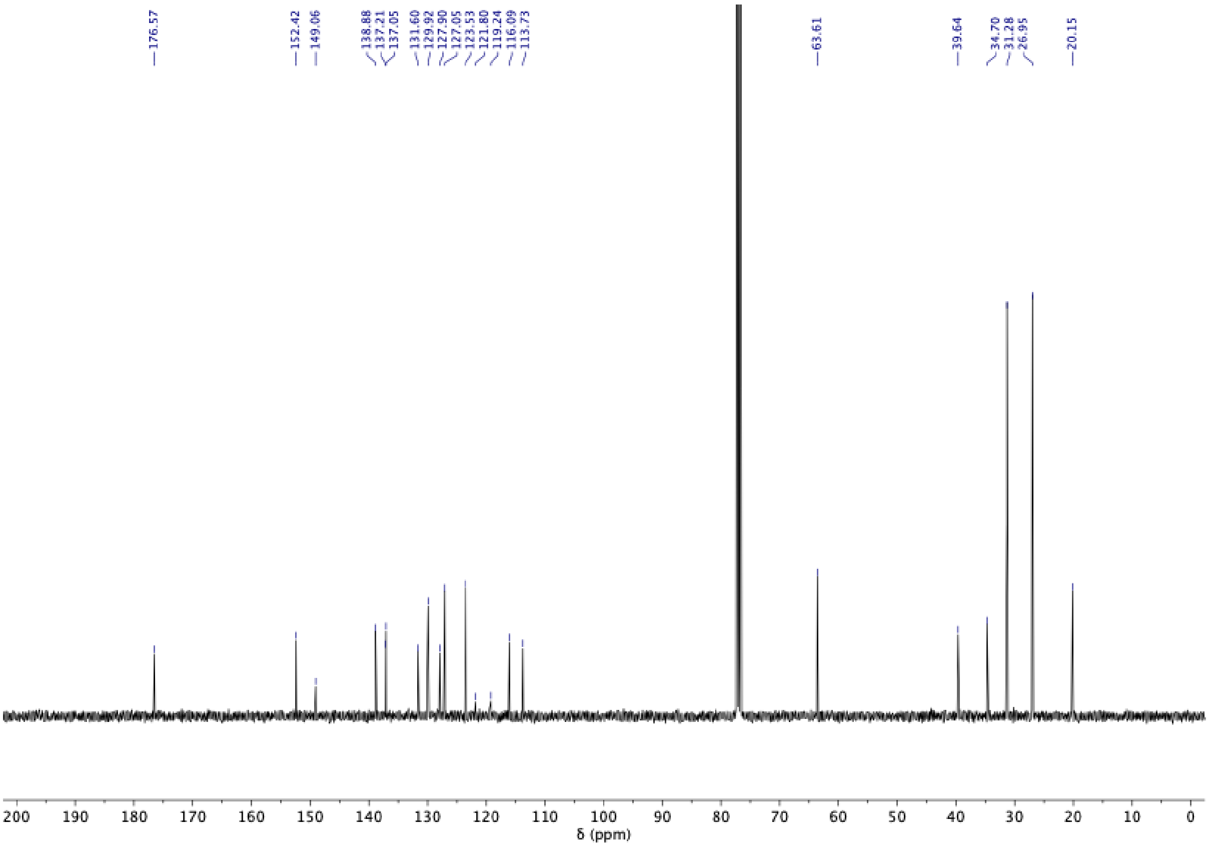
176.57
152.42
149.06
138.88
137.21
137.05
131.60
129.92
127.90
127.05
123.53
121.80
119.24
116.09
113.73
63.61
39.64
34.70
31.28
26.95
20.15
200 190 180 170 160 150 140 130 120 110 100 90 80 70 60 50 40 30 20 10 0
δ (ppm)

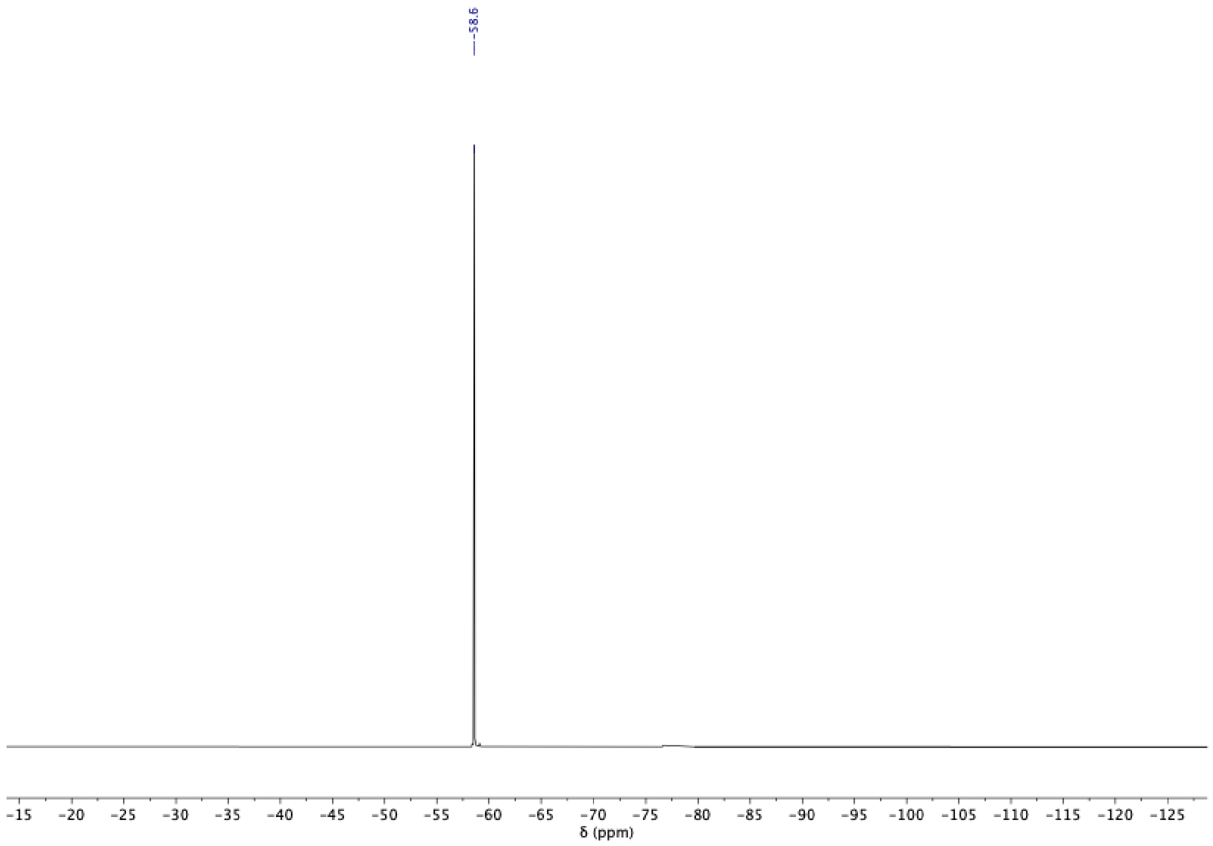
-58.6
-15 -20 -25 -30 -35 -40 -45 -50 -55 -60 -65 -70 -75 -80 -85 -90 -95 -100 -105 -110 -115 -120 -125
δ (ppm)

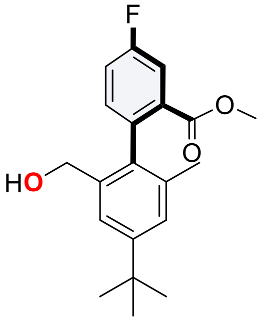
F
O
O
HO

30a

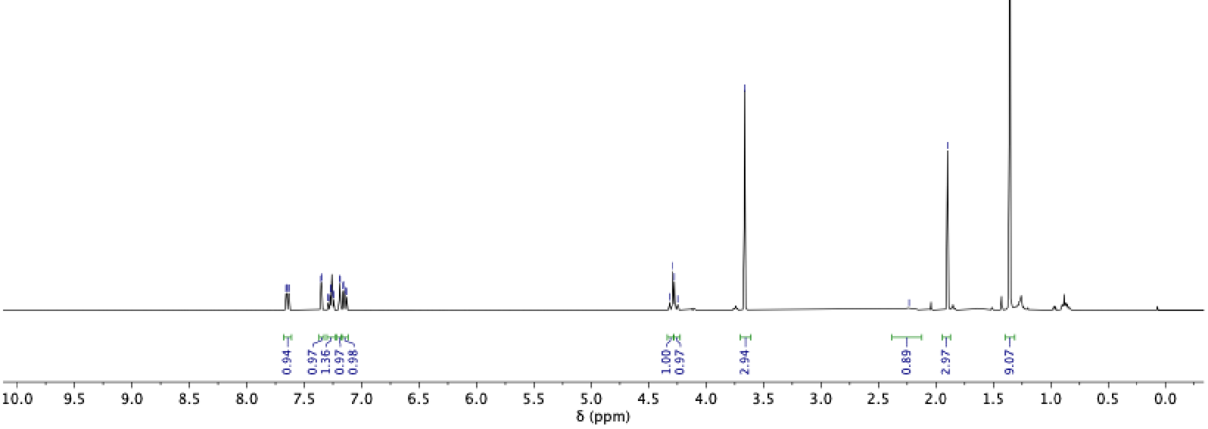
7.66
7.65
7.64
7.63
7.35
7.35
7.29
7.29
7.27
7.27
7.25
7.24
7.19
7.19
7.16
7.15
7.14
7.13
4.32
4.29
4.28
4.25
3.67
2.23
1.90
1.36
0.94
0.97
1.36
0.97
0.98
1.00
0.97
2.94
0.89
2.97
9.07
10.0 9.5 9.0 8.5 8.0 7.5 7.0 6.5 6.0 5.5 5.0 4.5 4.0 3.5 3.0 2.5 2.0 1.5 1.0 0.5 0.0
δ (ppm)

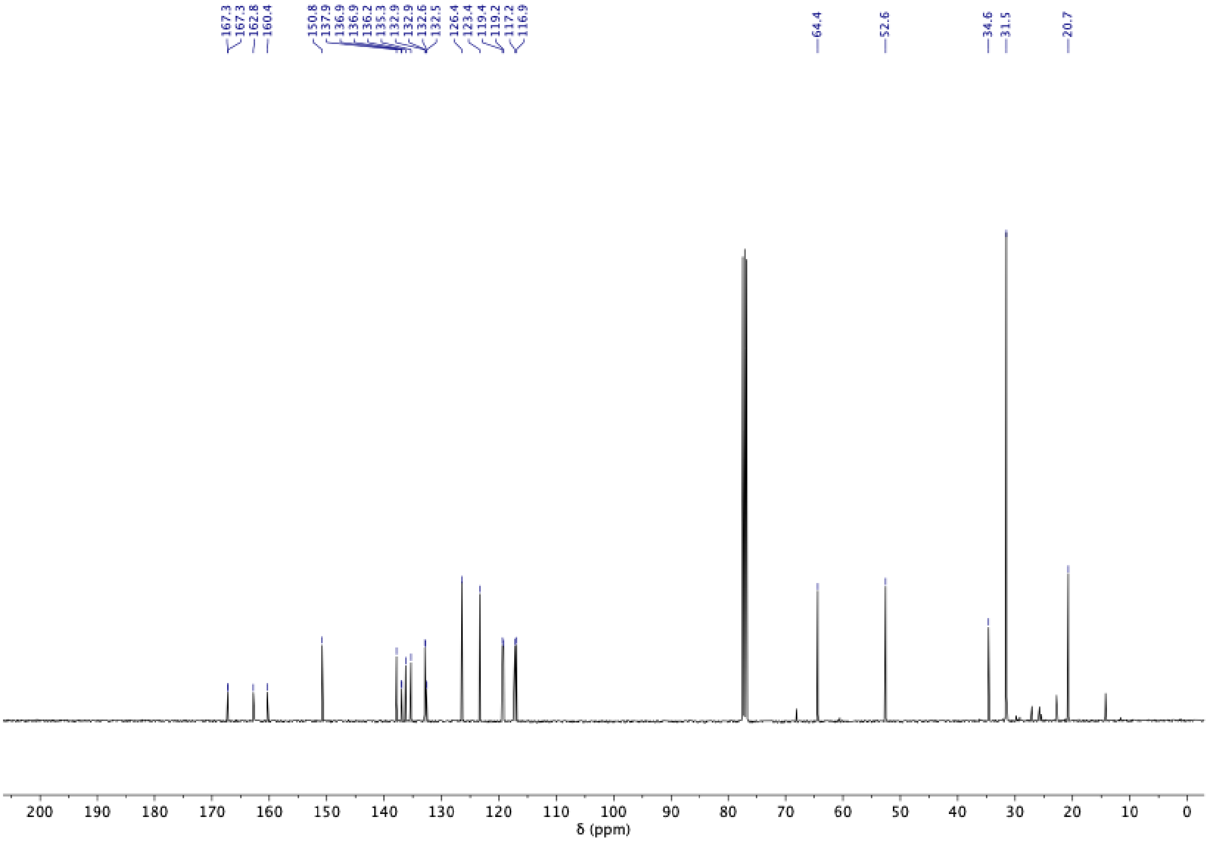
167.3
167.3
162.8
160.4
150.8
137.9
136.9
136.9
136.2
135.3
132.9
132.9
132.6
132.5
126.4
123.4
119.4
119.2
117.2
116.9
64.4
52.6
34.6
31.5
20.7
200 190 180 170 160 150 140 130 120 110 100 90 80 70 60 50 40 30 20 10 0
δ (ppm)

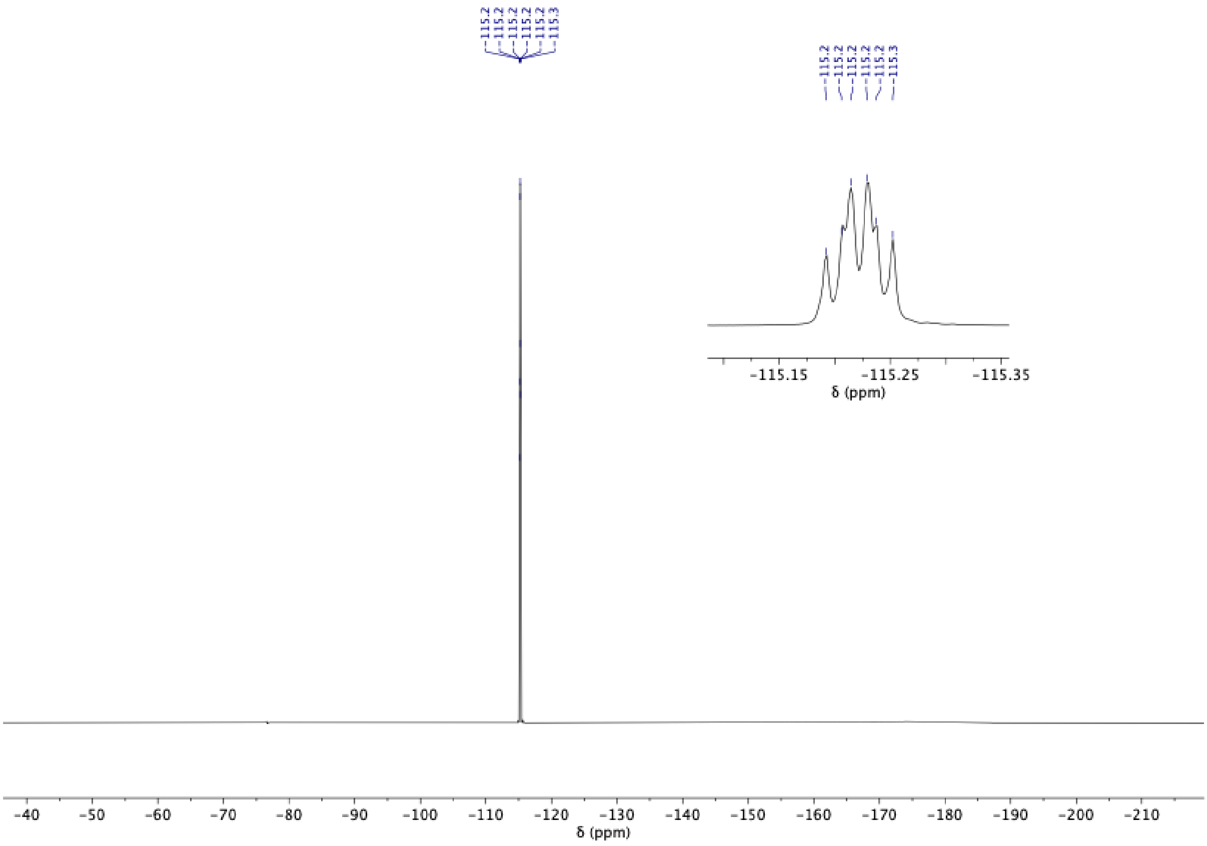
-115.2
-115.2
-115.2
-115.2
-115.2
-115.3
-115.15
-115.25
-115.35
δ (ppm)
-40
-50
-60
-70
-80
-90
-100
-110
-120
-130
-140
-150
-160
-170
-180
-190
-200
-210
δ (ppm)

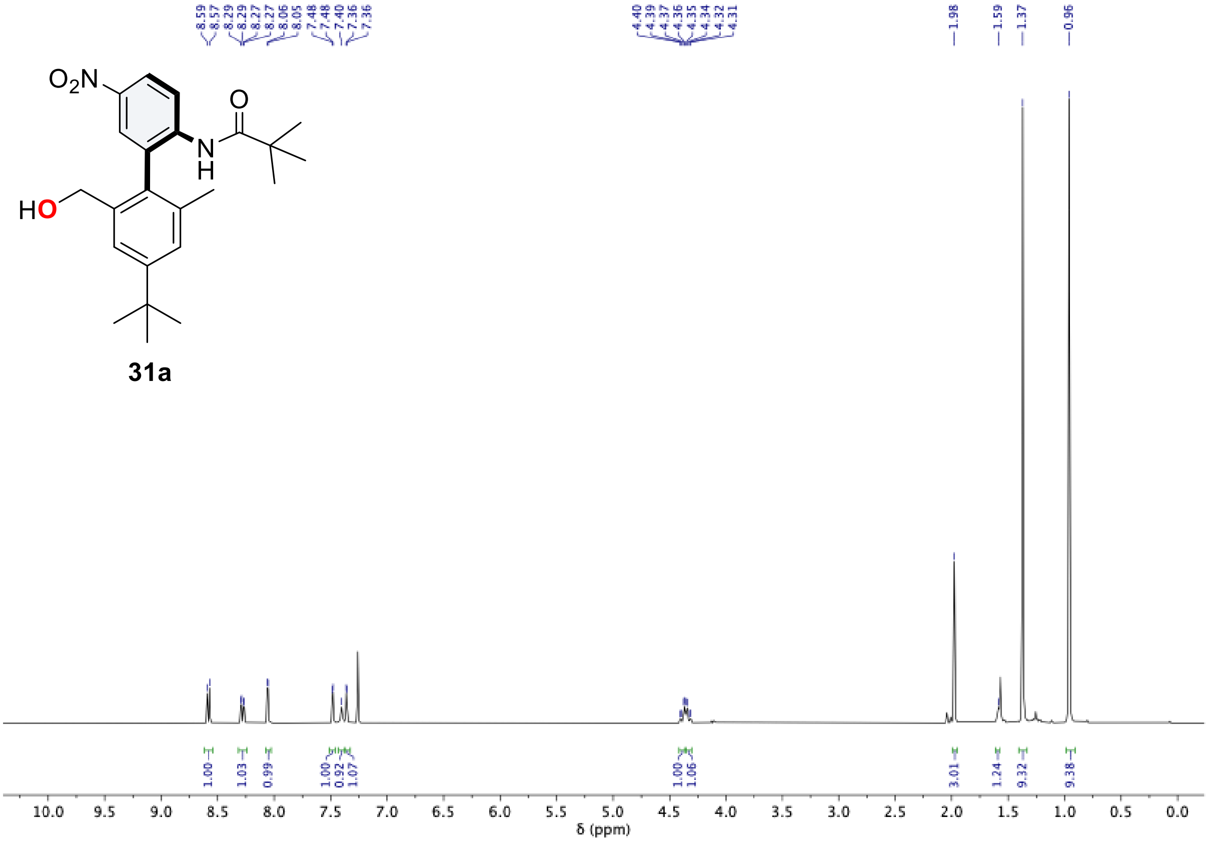

O2N
O
N
H
HO
31a
8.59 8.57 8.29 8.29 8.27 8.27 8.06 8.05 7.48 7.48 7.40 7.36 7.36
4.40 4.39 4.37 4.36 4.35 4.34 4.32 4.31
1.98
1.59
1.37
0.96
1.00 1.03 0.99 1.00 0.92 1.07
1.00 1.06
3.01 1.24 9.32 9.38
10.0 9.5 9.0 8.5 8.0 7.5 7.0 6.5 6.0 5.5 5.0 4.5 4.0 3.5 3.0 2.5 2.0 1.5 1.0 0.5 0.0
δ (ppm)

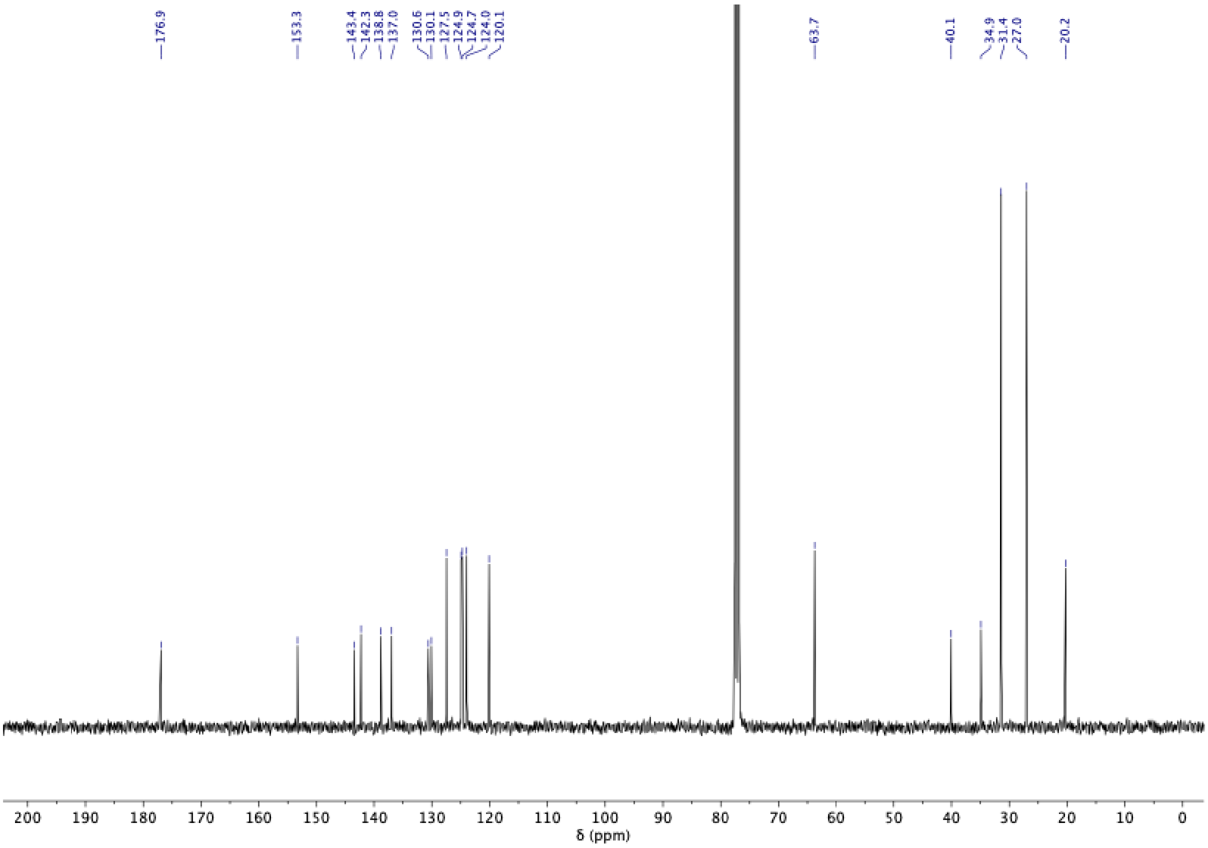

176.9
153.3
143.4 142.3 138.8 137.0 130.6 130.1 127.5 124.9 124.7 124.0 120.1
63.7
40.1
34.9 31.4 27.0
20.2
200 190 180 170 160 150 140 130 120 110 100 90 80 70 60 50 40 30 20 10 0
δ (ppm)

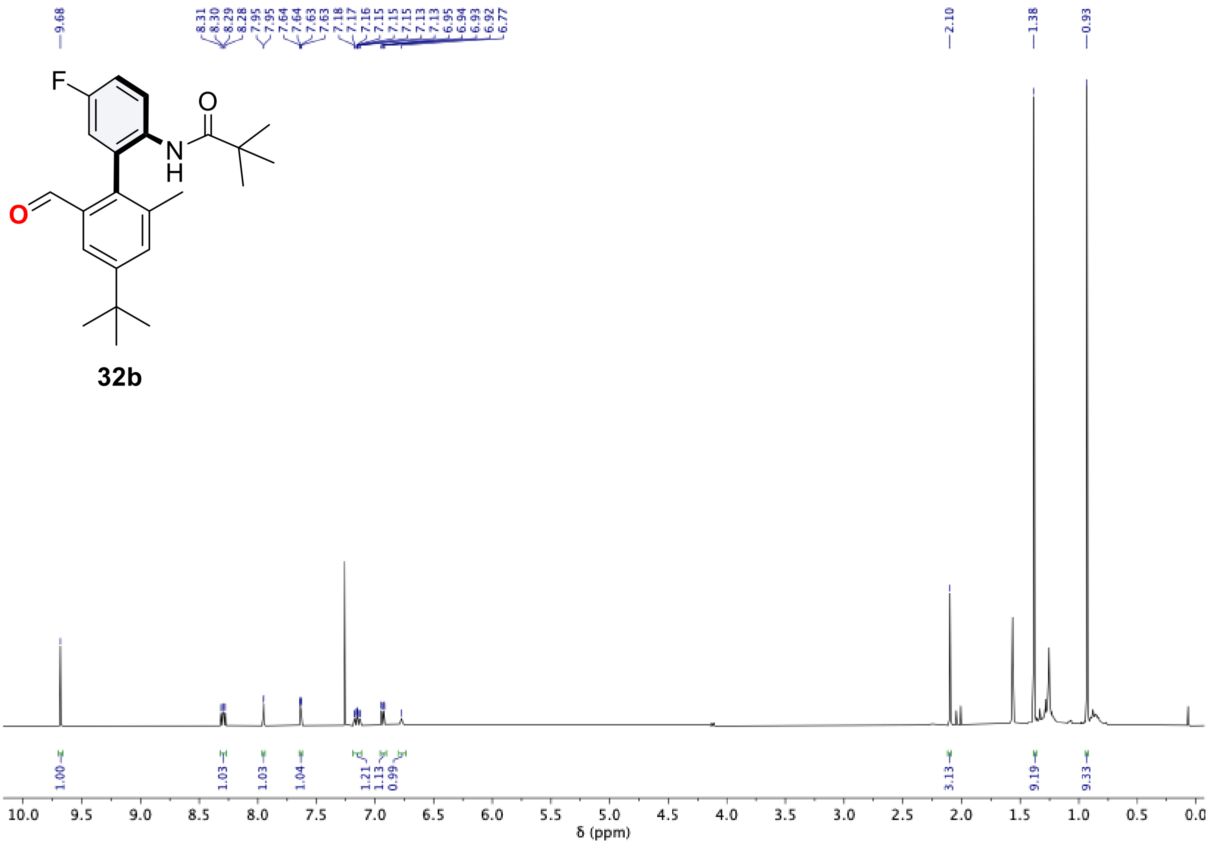

32b

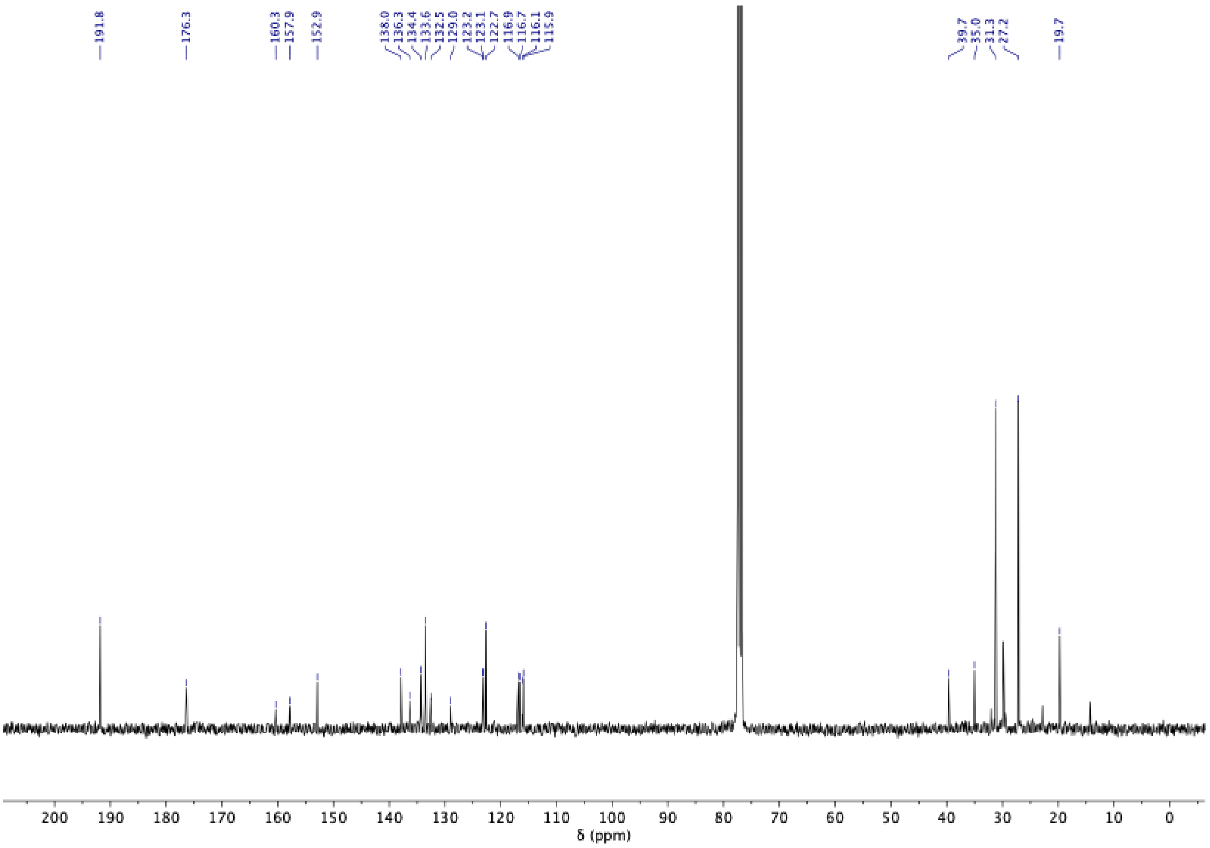

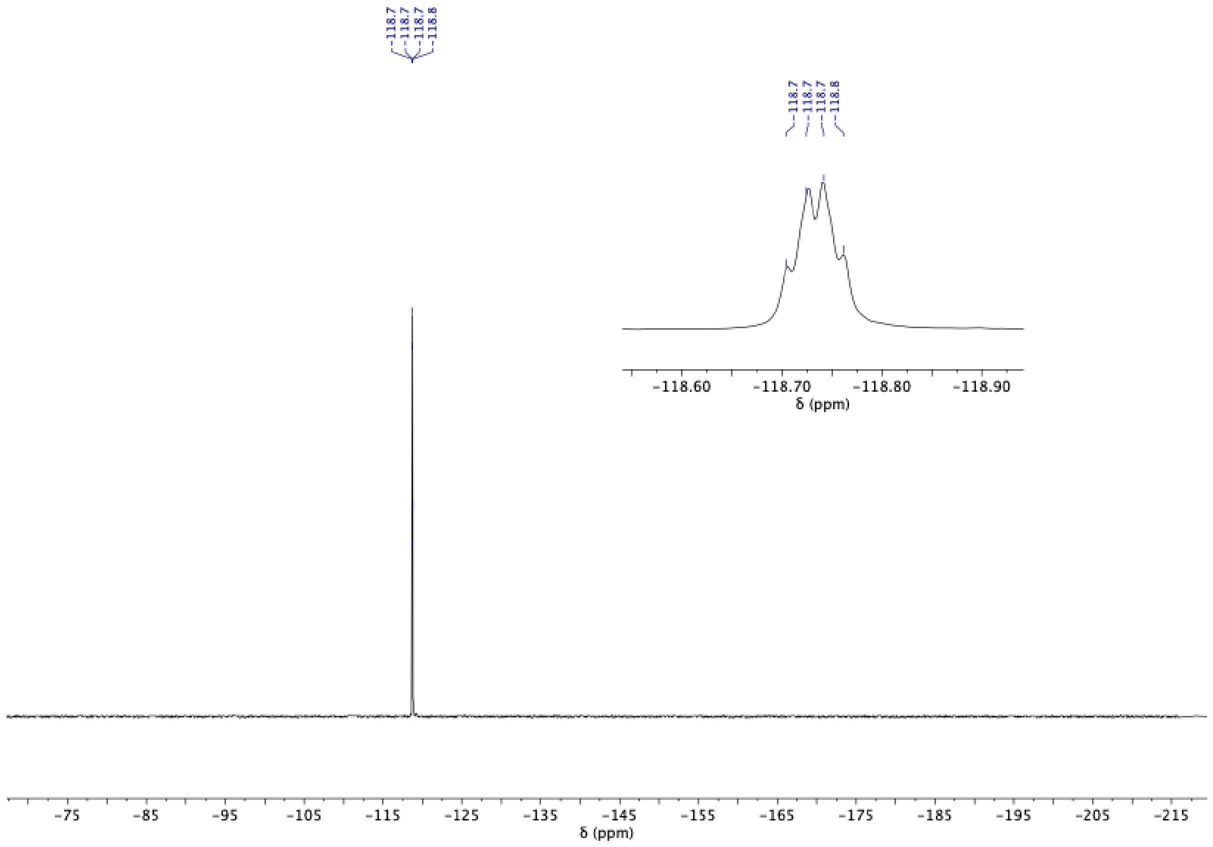
-118.7
-118.7
-118.7
-118.8
-75
-85
-95
-105
-115
-125
-135
-145
-155
-165
-175
-185
-195
-205
-215
δ (ppm)
-118.60
-118.70
-118.80
-118.90
δ (ppm)

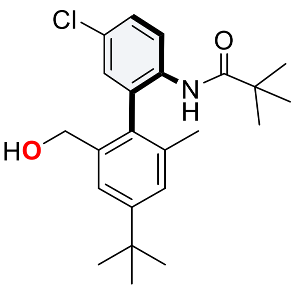

33a

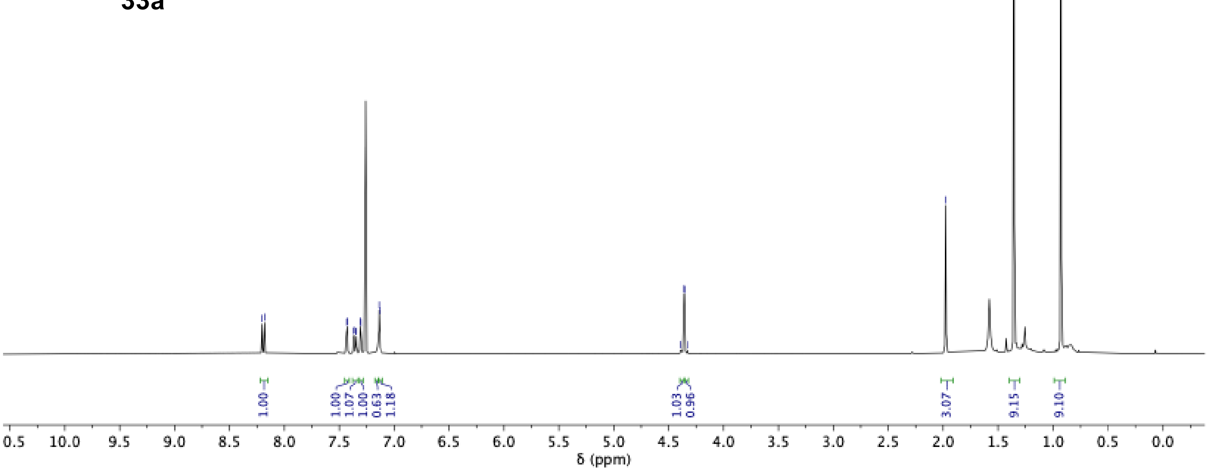
8.20
8.18
7.43
7.43
7.37
7.37
7.35
7.34
7.31
7.30
7.14
7.13
4.39
4.36
4.36
4.33
1.98
1.36
0.93
1.00
1.00
1.07
1.00
0.63
1.18
1.03
0.96
3.07
9.15
9.10
δ (ppm)

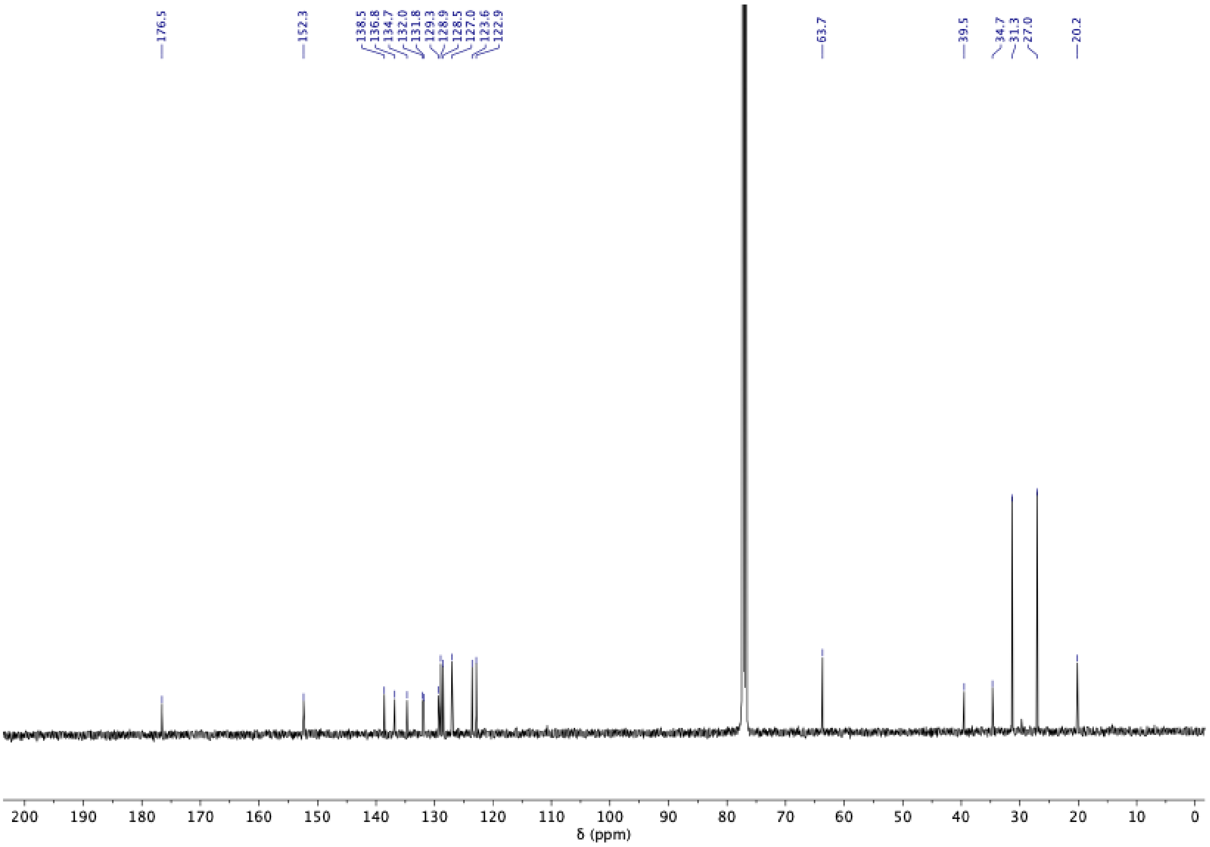
176.5
152.3
138.5
136.8
134.7
132.0
131.8
129.3
128.9
128.5
127.0
123.6
122.9
63.7
39.5
34.7
31.3
27.0
20.2
δ (ppm)

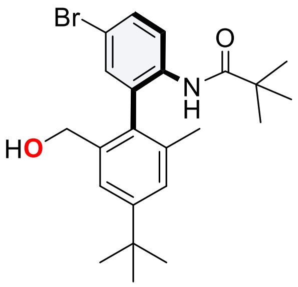
Br
O
N
H
HO

34a

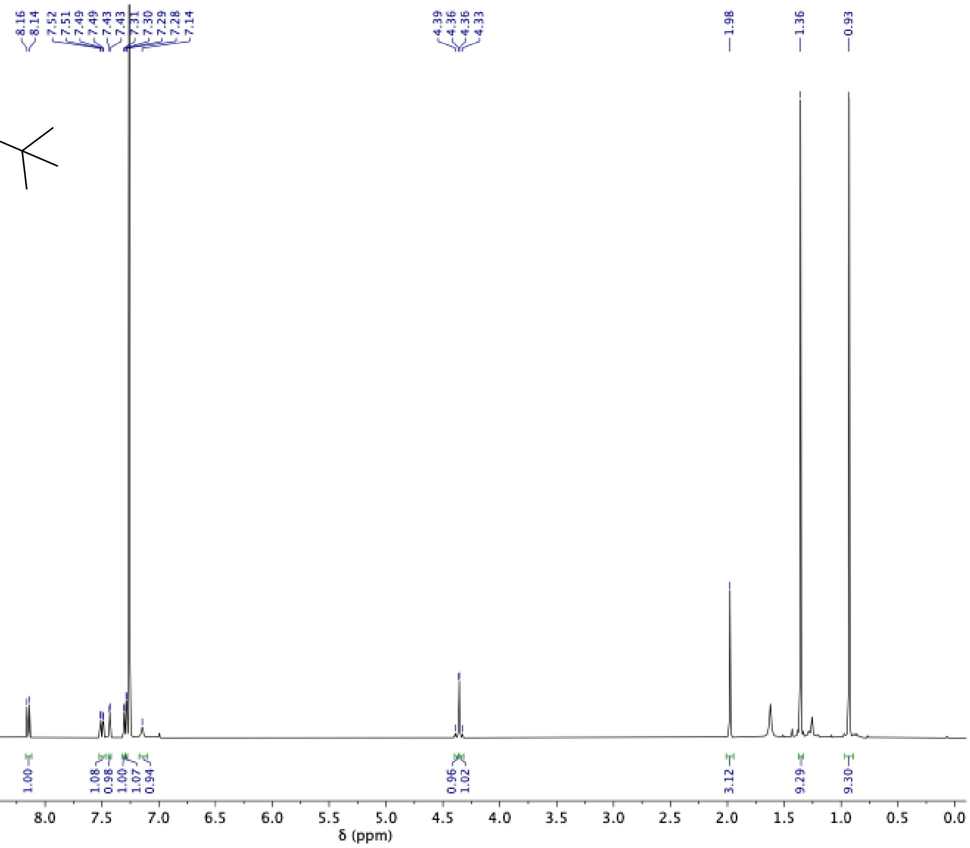
8.16
8.14
7.52
7.51
7.49
7.49
7.43
7.43
7.31
7.30
7.29
7.29
7.28
7.14
4.39
4.36
4.36
4.33
1.98
1.36
0.93
1.00
1.08
0.98
1.00
1.07
0.94
0.96
1.02
3.12
9.29
9.30
10.0 9.5 9.0 8.5 8.0 7.5 7.0 6.5 6.0 5.5 5.0 4.5 4.0 3.5 3.0 2.5 2.0 1.5 1.0 0.5 0.0
δ (ppm)

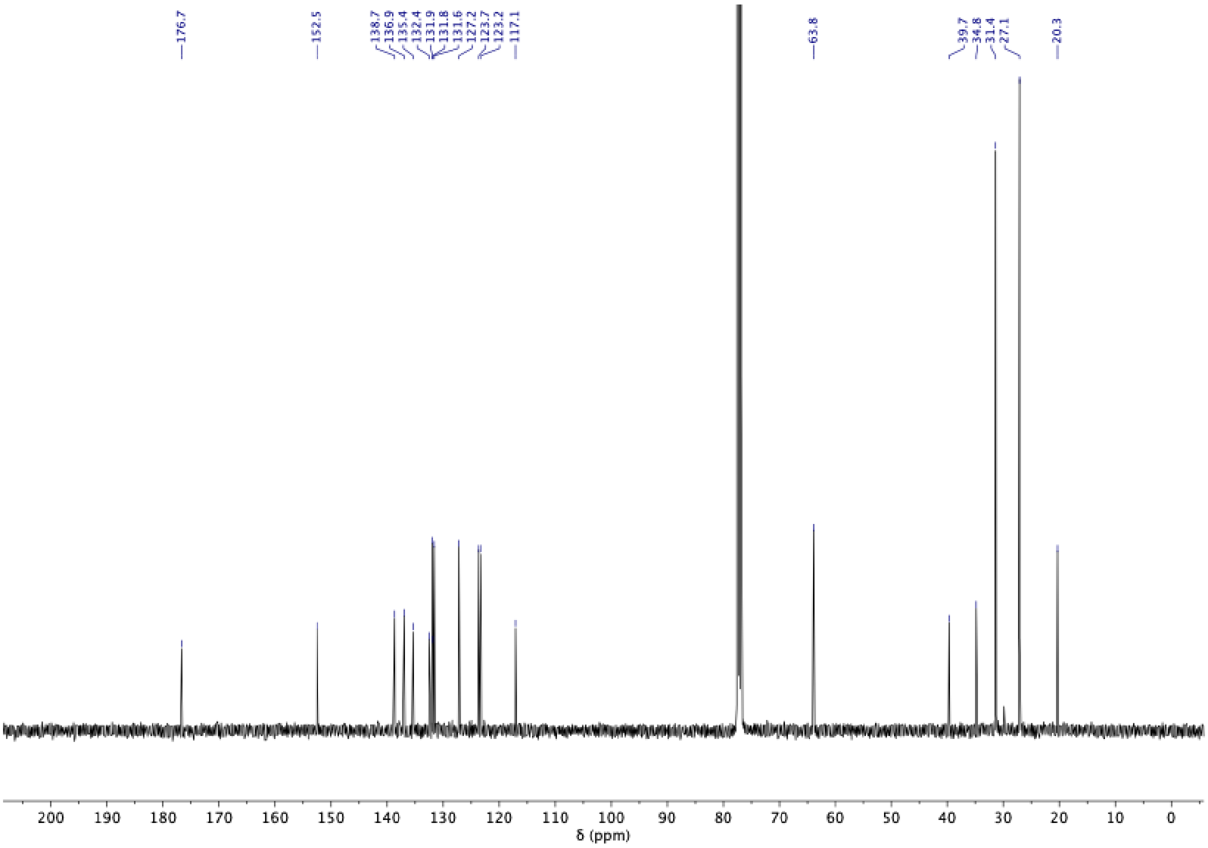
176.7
152.5
138.7
136.9
135.4
132.4
131.9
131.8
131.6
127.2
123.7
123.2
117.1
63.8
39.7
34.8
31.4
27.1
20.3
200 190 180 170 160 150 140 130 120 110 100 90 80 70 60 50 40 30 20 10 0
δ (ppm)

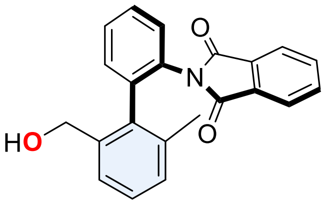

35a

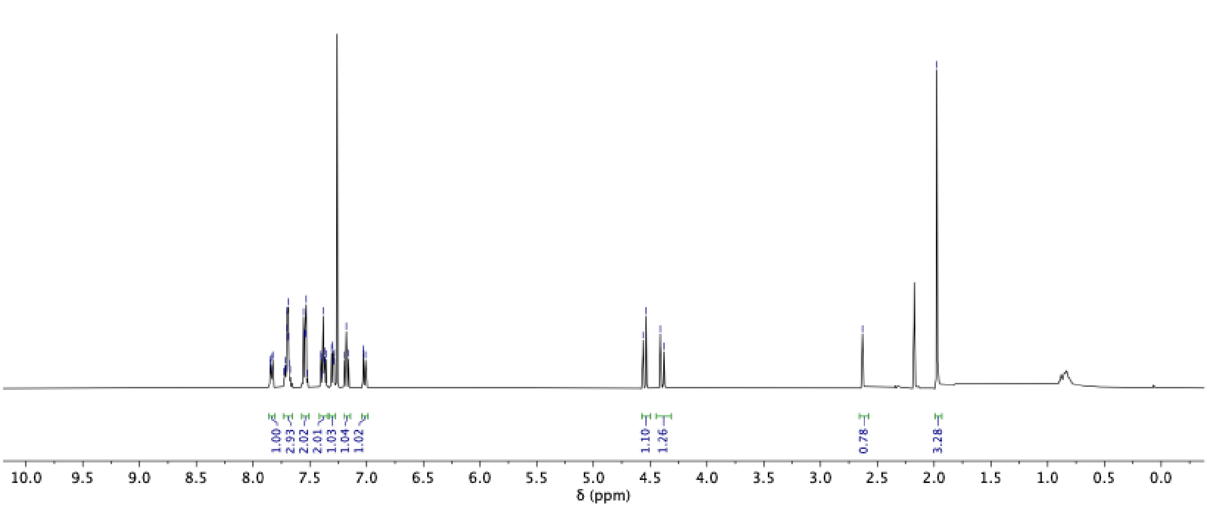
7.85
7.84
7.84
7.83
7.83
7.73
7.72
7.72
7.71
7.71
7.70
7.70
7.69
7.69
7.68
7.67
7.55
7.55
7.55
7.54
7.54
7.53
7.52
7.40
7.40
7.39
7.38
7.37
7.37
7.36
7.31
7.30
7.29
7.29
7.28
7.20
7.18
7.16
7.03
7.03
7.01
4.57
4.53
4.41
4.38
2.63
1.98
1.00
2.93
2.02
2.01
1.03
1.04
1.02
1.10
1.26
0.78
3.28
δ (ppm)

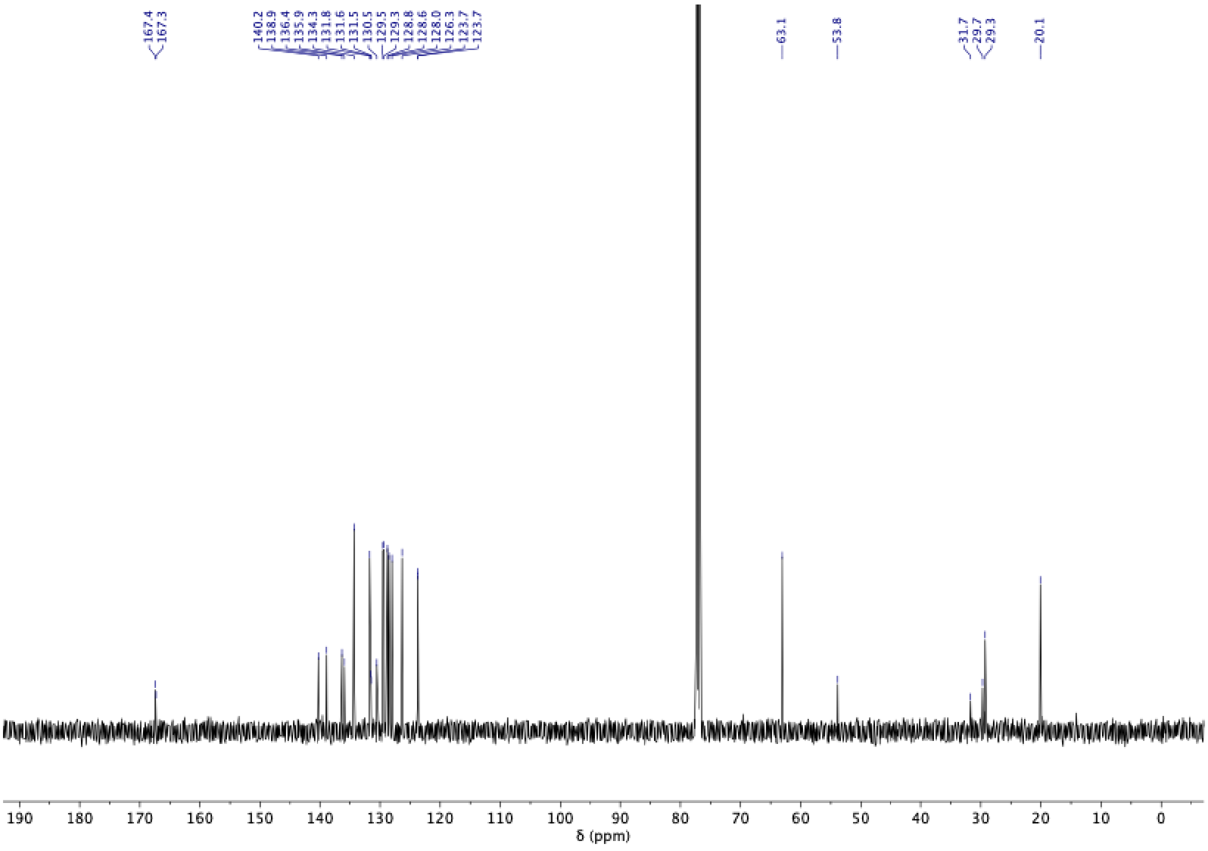
167.4
167.3
140.2
138.9
136.4
135.9
134.3
131.8
131.6
131.5
130.5
129.5
129.3
128.8
128.6
128.0
126.3
123.7
123.7
63.1
53.8
31.7
29.7
29.3
20.1
δ (ppm)

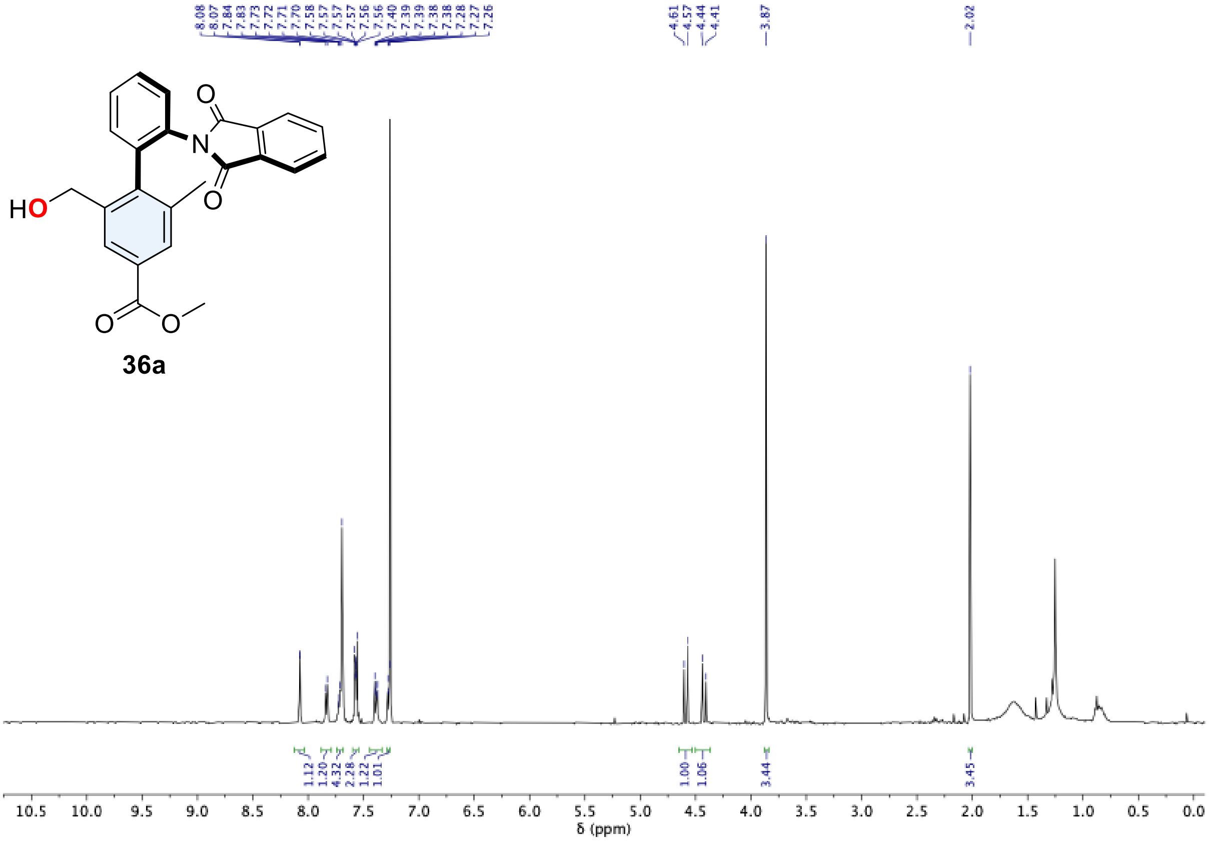

36a

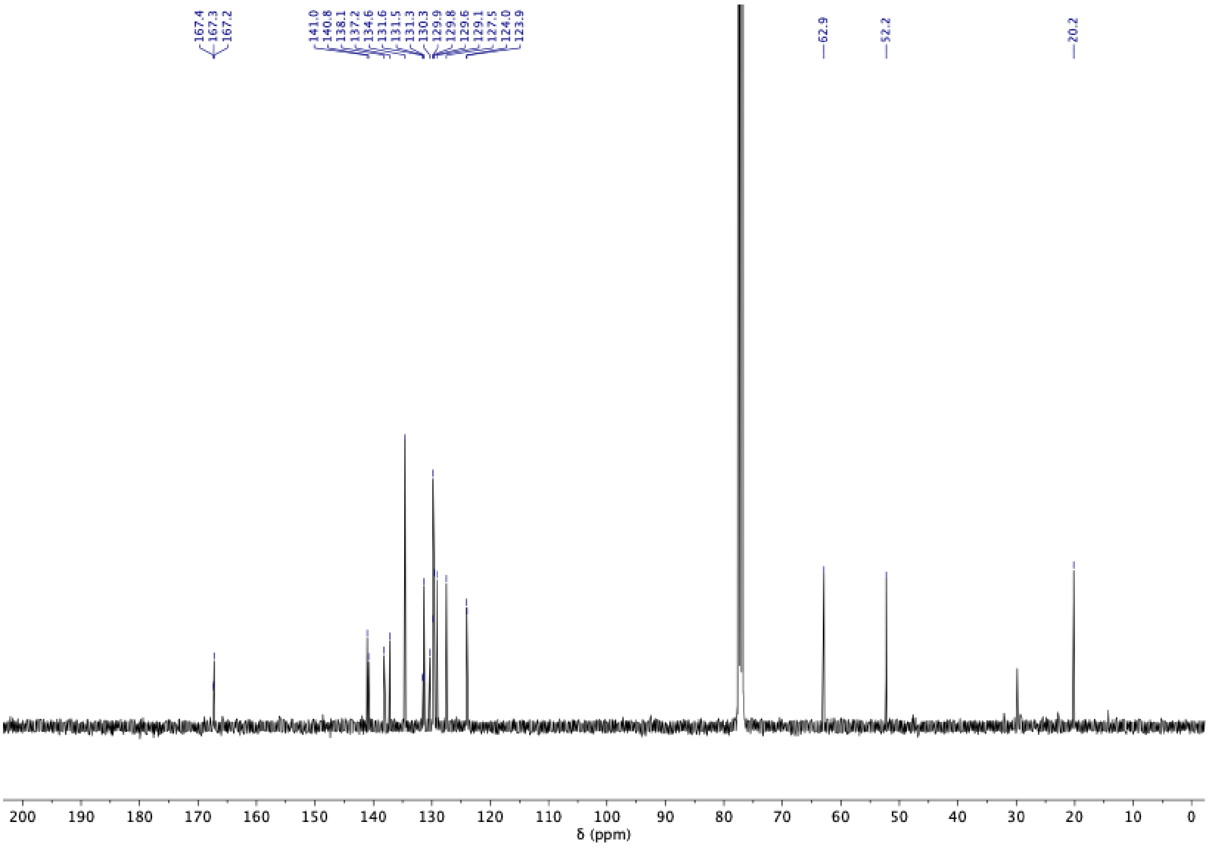

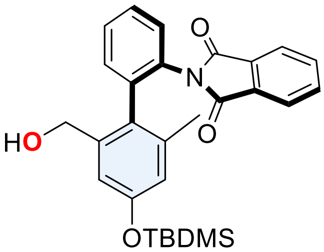
O
N
O
HO
OTBDMS

37a

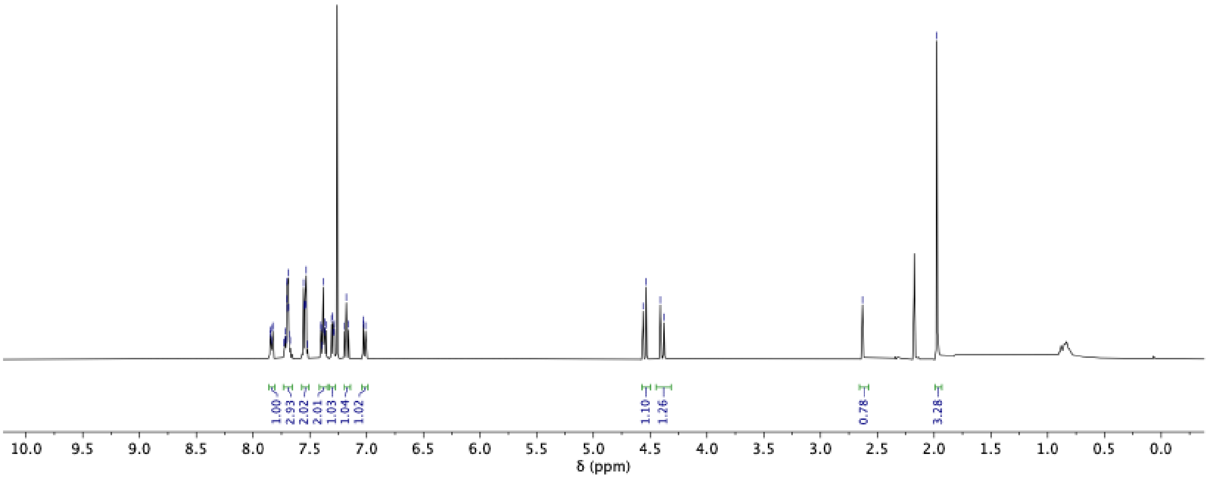
7.85 7.84 7.84 7.83 7.83 7.73 7.72 7.72 7.71 7.71 7.70 7.69 7.69 7.68 7.67 7.55 7.55 7.55 7.54 7.54 7.53 7.52 7.40 7.40 7.39 7.38 7.37 7.37 7.36 7.31 7.30 7.29 7.29 7.28 7.20 7.18 7.16 7.03 7.01
4.57 4.53 4.41 4.38
2.63
1.98
1.00 2.93 2.02 2.01 1.03 1.04 1.02
1.10 1.26
0.78
3.28
δ (ppm)

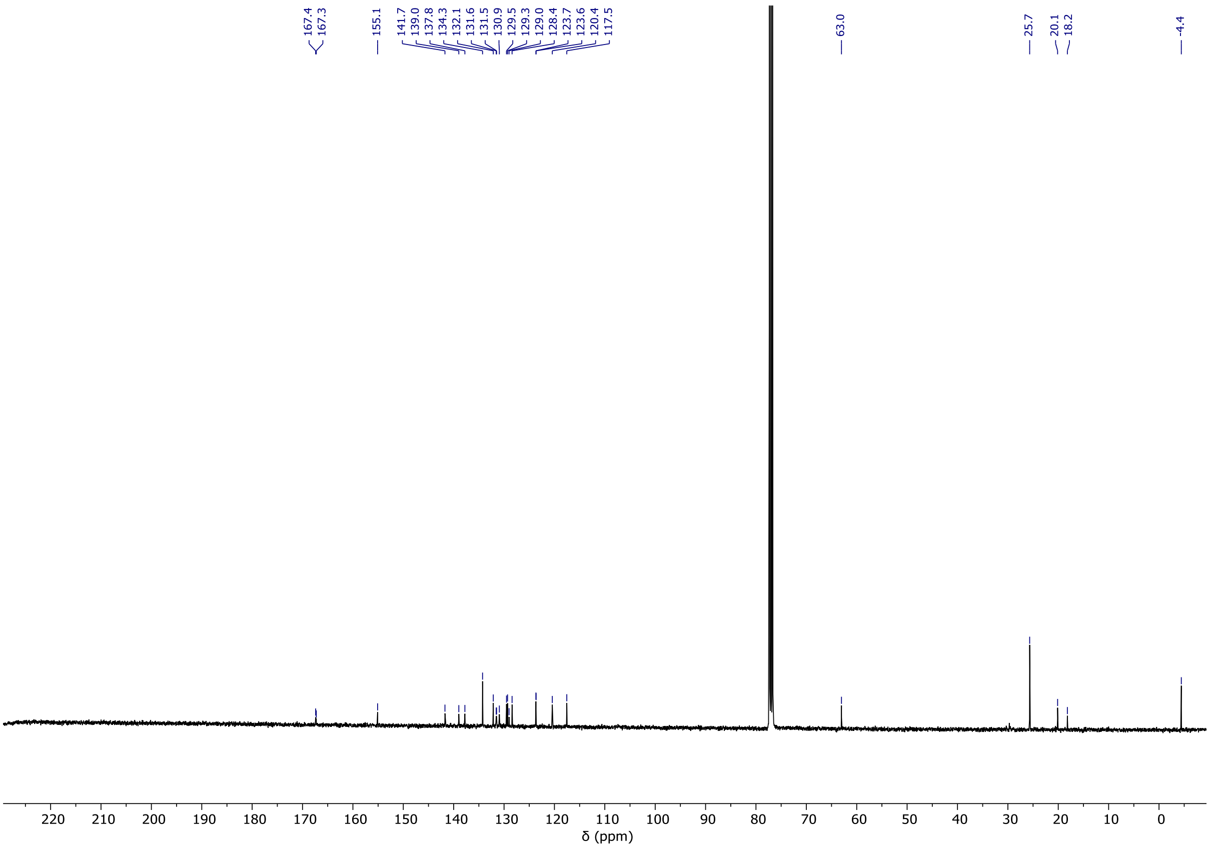
167.4 167.3
155.1
141.7 139.0 137.8 134.3 132.1 131.6 131.5 130.9 129.5 129.3 129.0 128.4 123.7 123.6 120.4 117.5
63.0
25.7 20.1 18.2
-4.4
δ (ppm)

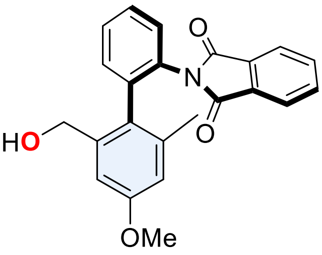
HO
N
O
O
OMe

38a

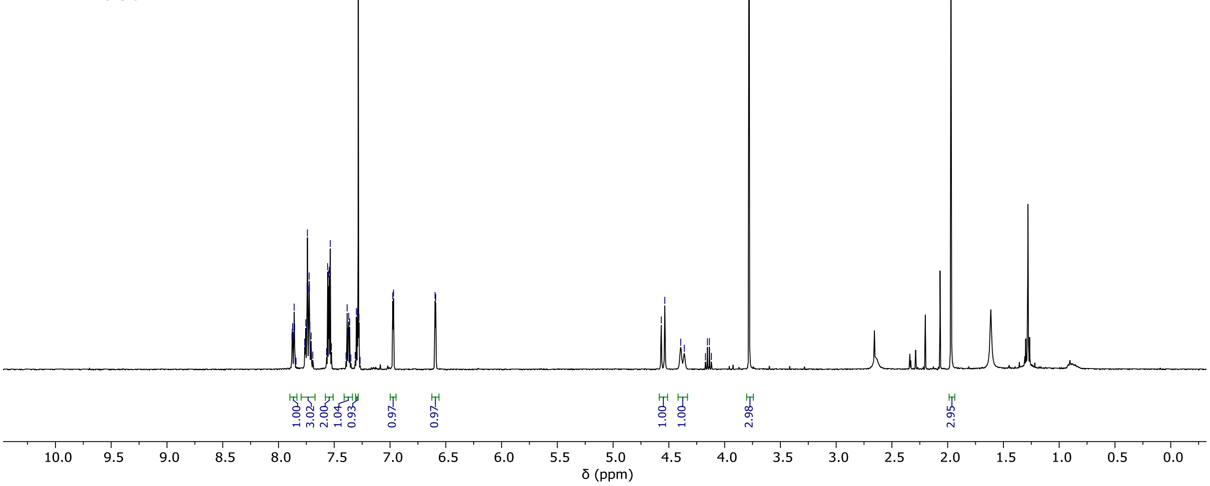
7.88 7.87 7.86 7.86 7.86 7.76 7.75 7.74 7.74 7.73 7.73 7.72 7.72 7.56 7.55 7.54 7.54 7.38 7.38 7.37 7.37 7.36 7.30 7.29 7.28 6.98 6.97 6.60 6.59
4.57 4.54 4.39 4.36 4.17 4.15 4.14 4.12 3.78
1.97
1.00 3.02 2.00 1.04 0.93 0.97 0.97 1.00 1.00 2.98 2.95
10.0 9.5 9.0 8.5 8.0 7.5 7.0 6.5 6.0 5.5 5.0 4.5 4.0 3.5 3.0 2.5 2.0 1.5 1.0 0.5 0.0
δ (ppm)

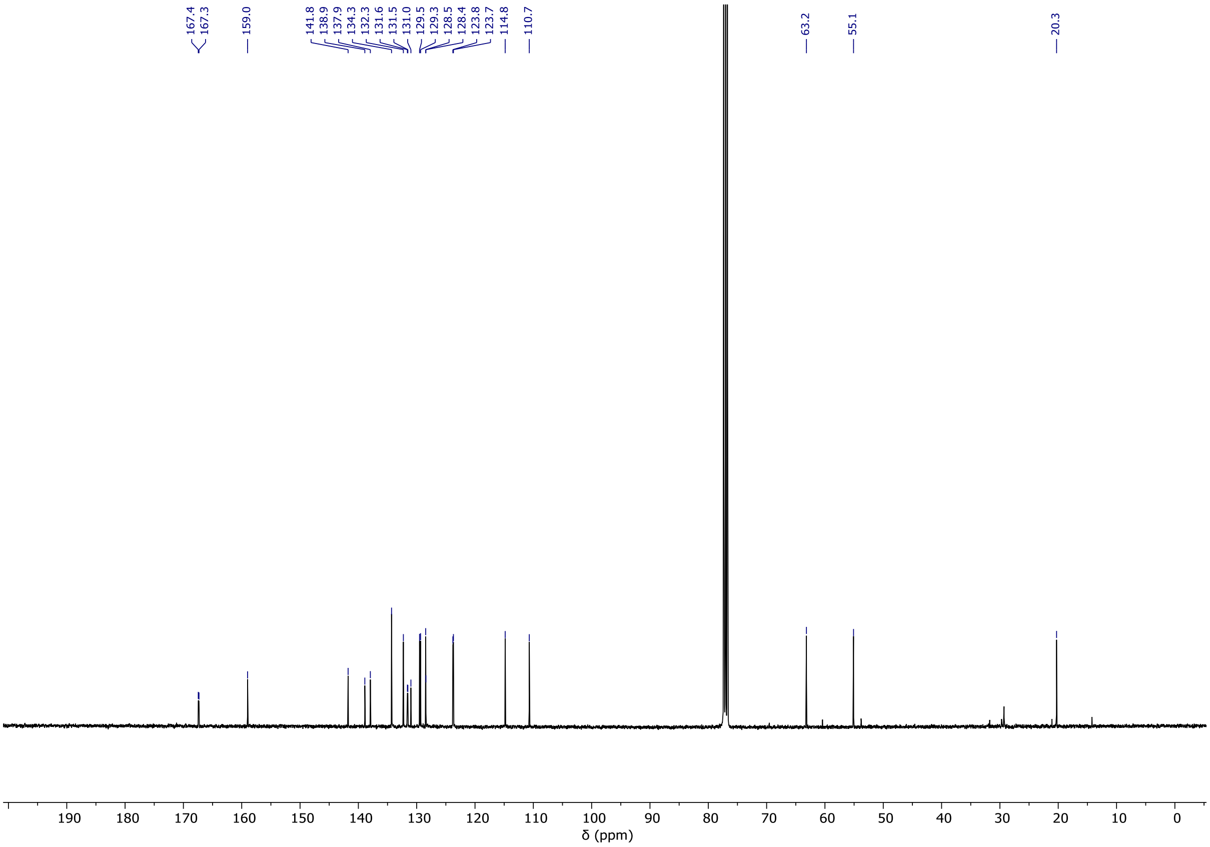
167.4 167.3 159.0
141.8 138.9 137.9 134.3 132.3 131.6 131.5 131.0 129.5 129.3 128.5 128.4 123.8 123.7 114.8 110.7
63.2 55.1 20.3
190 180 170 160 150 140 130 120 110 100 90 80 70 60 50 40 30 20 10 0
δ (ppm)

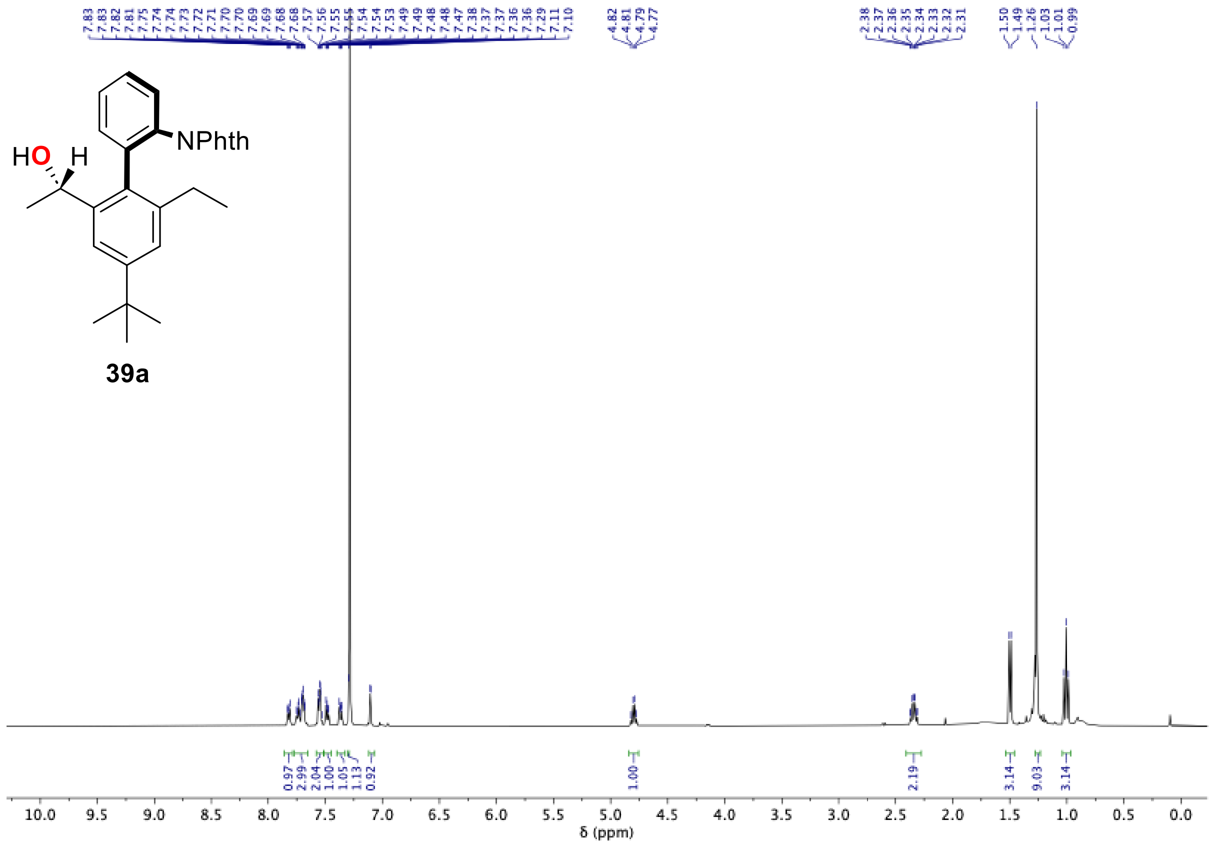

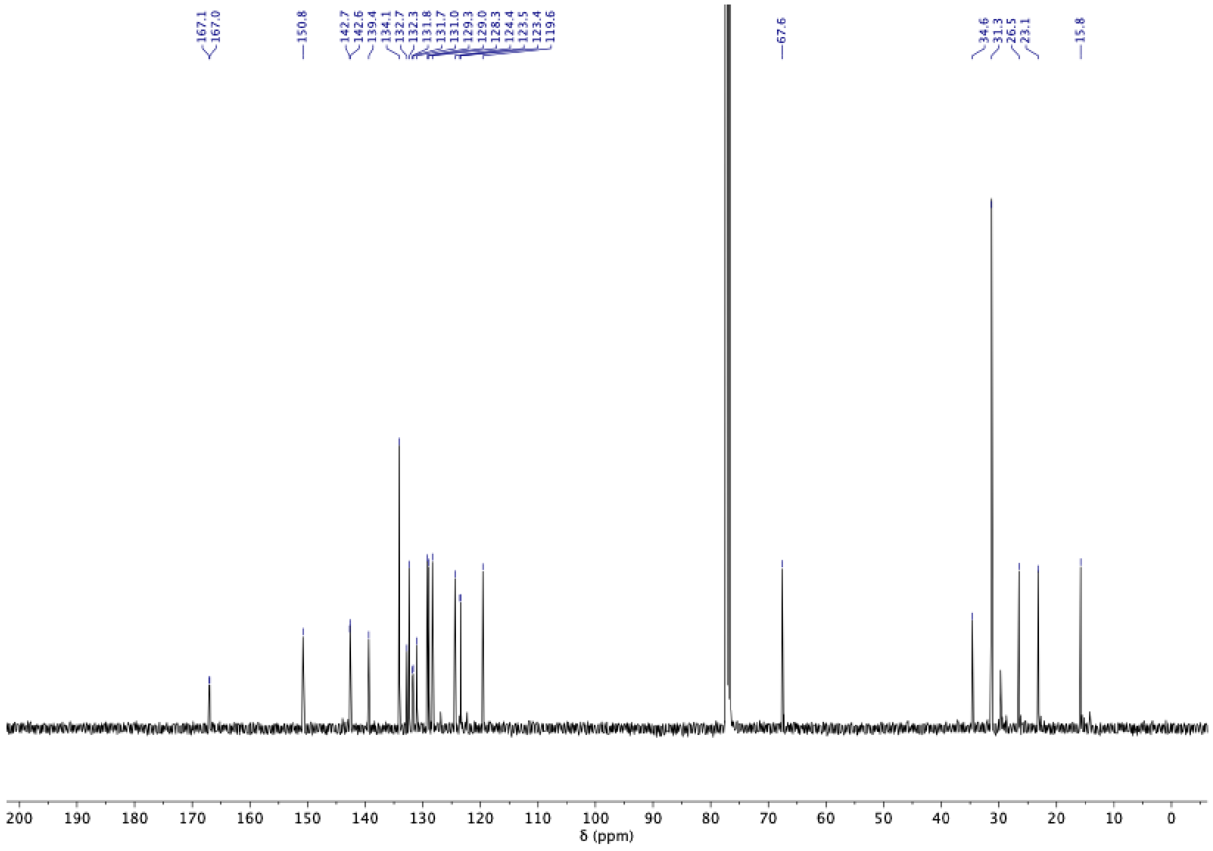

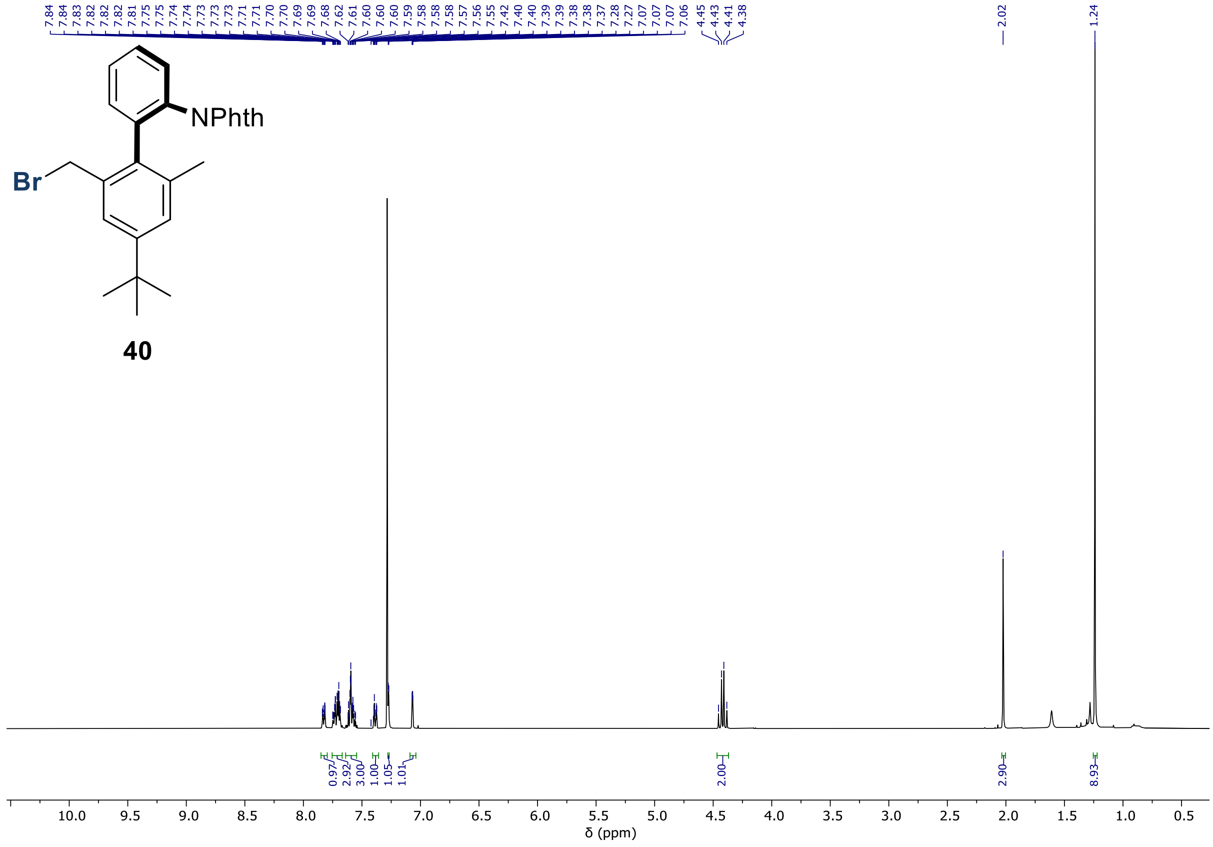
NPhth
Br
40
7.84 7.84 7.83 7.82 7.82 7.82 7.81 7.75 7.75 7.74 7.74 7.73 7.73 7.73 7.71 7.71 7.70 7.70 7.69 7.69 7.68 7.62 7.61 7.60 7.60 7.60 7.59 7.58 7.58 7.58 7.57 7.56 7.55 7.42 7.40 7.40 7.39 7.39 7.38 7.38 7.37 7.28 7.27 7.07 7.07 7.07 7.06
4.45 4.43 4.41 4.38
2.02
1.24
0.97 2.92 3.00 1.00 1.05 1.01
2.00
2.90
8.93
10.0 9.5 9.0 8.5 8.0 7.5 7.0 6.5 6.0 5.5 5.0 4.5 4.0 3.5 3.0 2.5 2.0 1.5 1.0 0.5
δ (ppm)

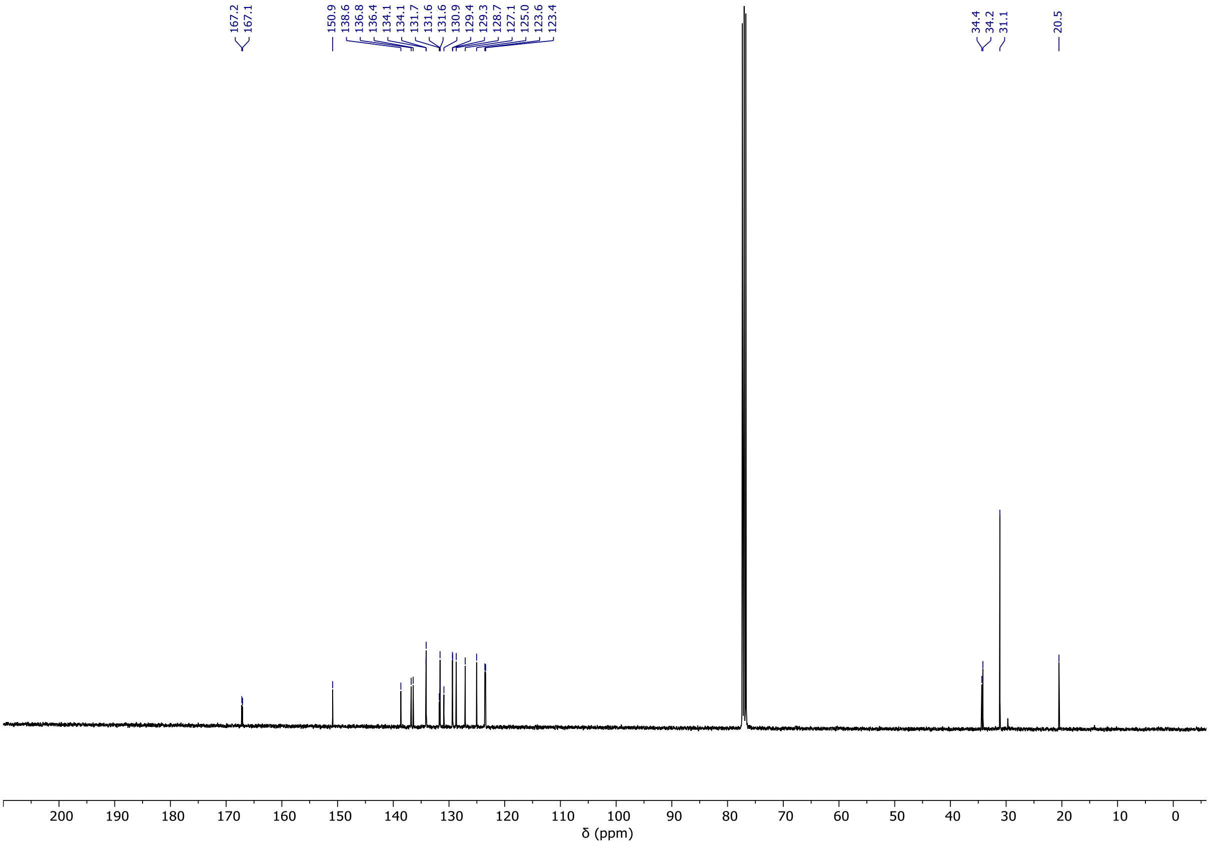
167.2 167.1
150.9 138.6 136.8 136.4 134.1 134.1 131.7 131.6 131.6 130.9 129.4 129.3 128.7 127.1 125.0 123.6 123.4
34.4 34.2 31.1
20.5
200 190 180 170 160 150 140 130 120 110 100 90 80 70 60 50 40 30 20 10 0
δ (ppm)

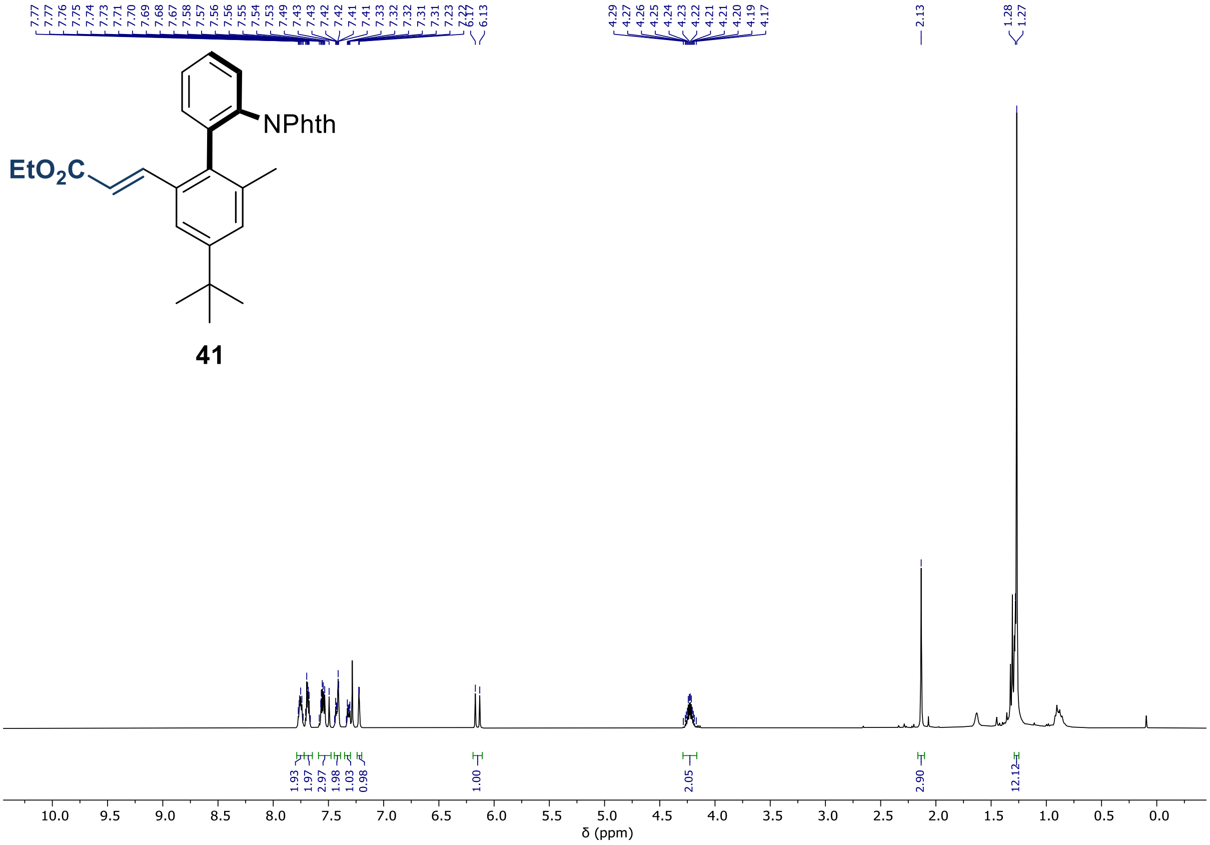
EtO2C
NPhth
7.77 7.77 7.76 7.75 7.74 7.73 7.71 7.70 7.69 7.68 7.67 7.58 7.57 7.56 7.56 7.55 7.54 7.53 7.49 7.43 7.43 7.42 7.42 7.41 7.41 7.33 7.32 7.32 7.31 7.31 7.23 7.22 6.17 6.13
4.29 4.27 4.26 4.25 4.24 4.23 4.22 4.21 4.21 4.20 4.19 4.17
2.13
1.28 1.27
1.93 1.97 2.97 1.98 1.03 0.98
1.00
2.05
2.90
12.12
10.0 9.5 9.0 8.5 8.0 7.5 7.0 6.5 6.0 5.5 5.0 4.5 4.0 3.5 3.0 2.5 2.0 1.5 1.0 0.5 0.0
δ (ppm)

41

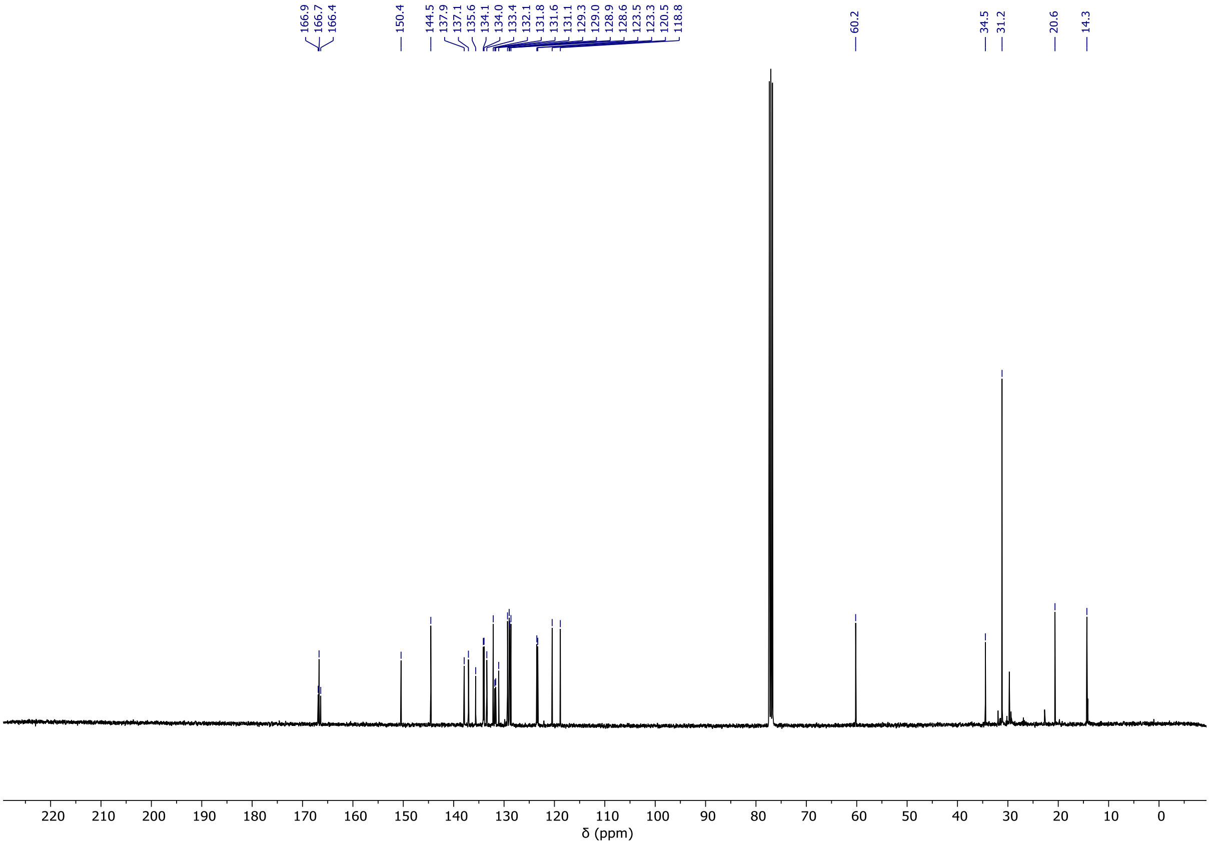
166.9 166.7 166.4
150.4
144.5 137.9 137.1 135.6 134.1 134.0 133.4 132.1 131.8 131.6 131.1 129.3 129.0 128.9 128.6 123.5 123.3 120.5 118.8
60.2
34.5 31.2
20.6
14.3
220 210 200 190 180 170 160 150 140 130 120 110 100 90 80 70 60 50 40 30 20 10 0
δ (ppm)

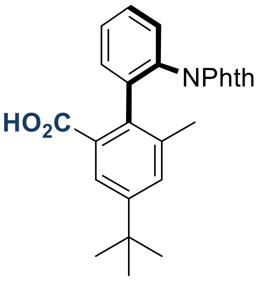
HO2C
NPhth

18c

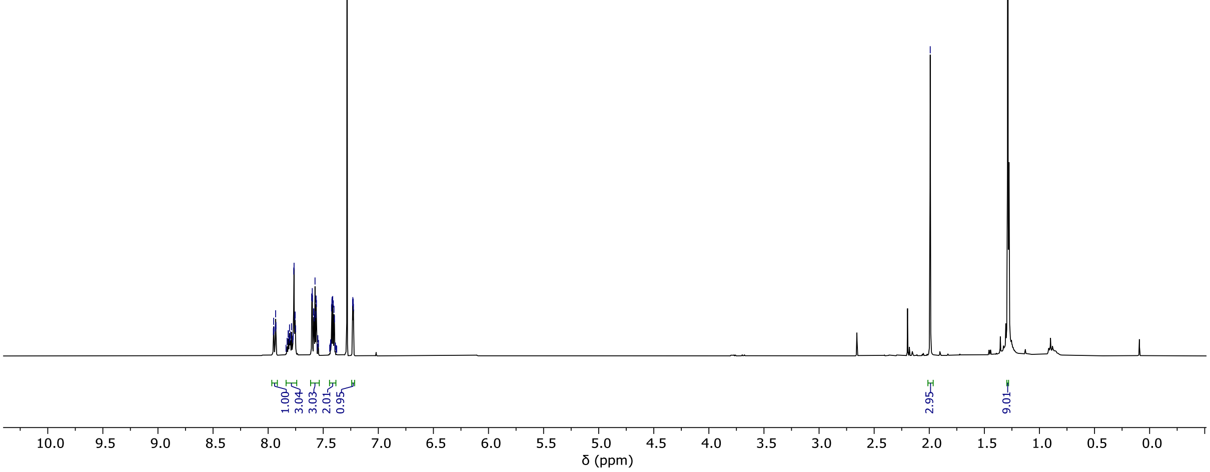
1.99
1.29
1.00
3.04
3.03
2.01
0.95
2.95
9.01
δ (ppm)

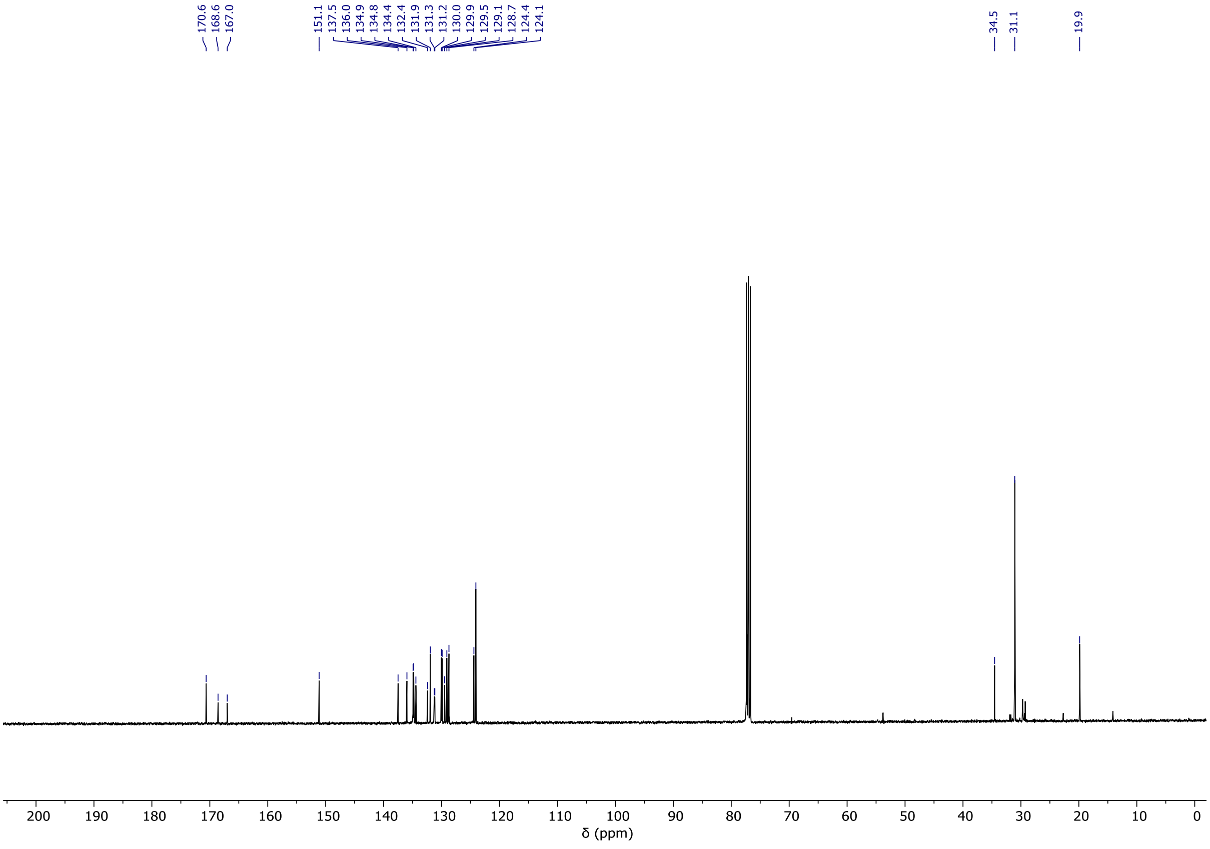
170.6
168.6
167.0
151.1
137.5
136.0
134.9
134.8
134.4
132.4
131.9
131.3
131.2
130.0
129.9
129.5
129.1
128.7
124.4
124.1
34.5
31.1
19.9
δ (ppm)

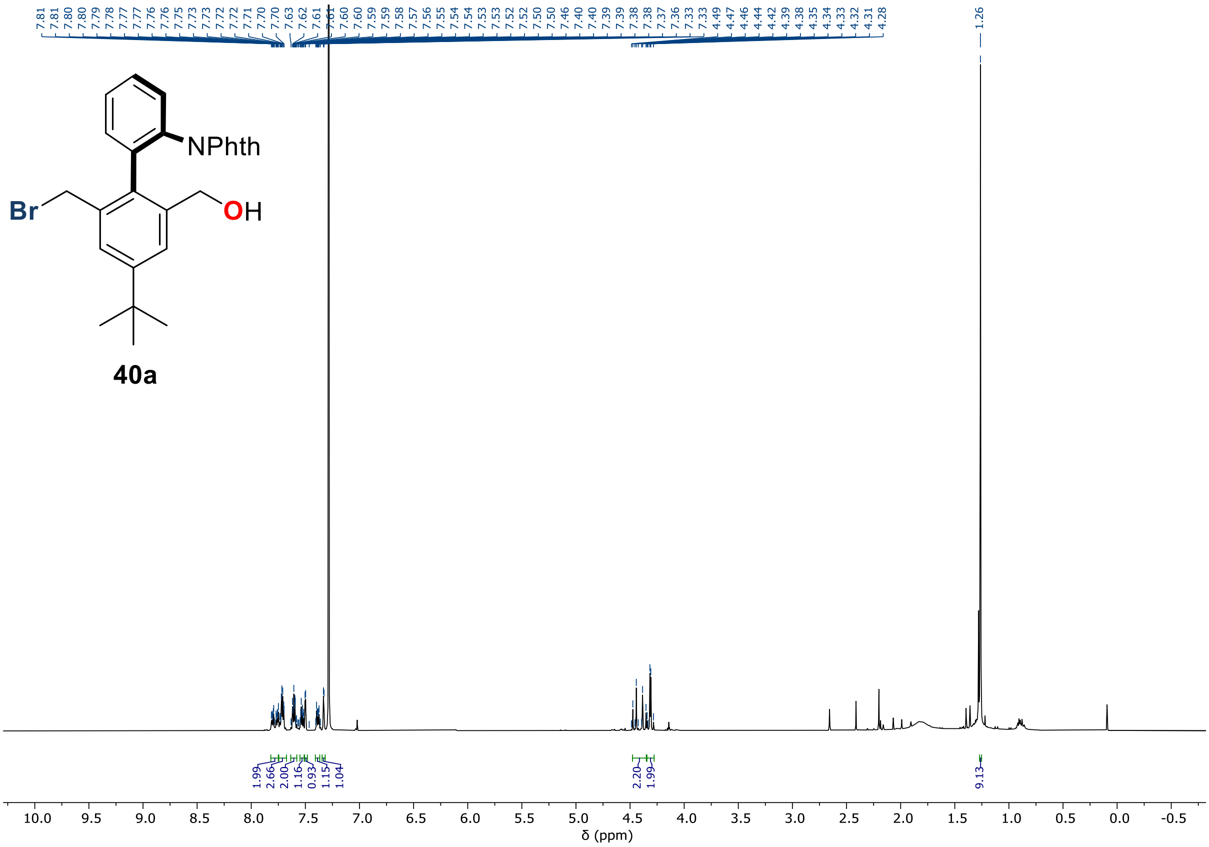
7.81 7.81 7.80 7.79 7.78 7.77 7.77 7.76 7.75 7.73 7.73 7.72 7.72 7.71 7.70 7.70 7.63 7.62 7.61 7.61 7.60 7.60 7.59 7.59 7.58 7.57 7.56 7.55 7.54 7.54 7.53 7.53 7.52 7.52 7.50 7.50 7.46 7.40 7.40 7.39 7.39 7.38 7.38 7.37 7.36 7.33 7.33 4.49 4.47 4.46 4.44 4.42 4.39 4.38 4.35 4.34 4.33 4.32 4.31 4.28
1.26
1.99 2.66 2.00 1.16 0.93 1.15 1.04
2.20 1.99
9.13
10.0 9.5 9.0 8.5 8.0 7.5 7.0 6.5 6.0 5.5 5.0 4.5 4.0 3.5 3.0 2.5 2.0 1.5 1.0 0.5 0.0 -0.5
δ (ppm)
NPhth
Br
OH

40a

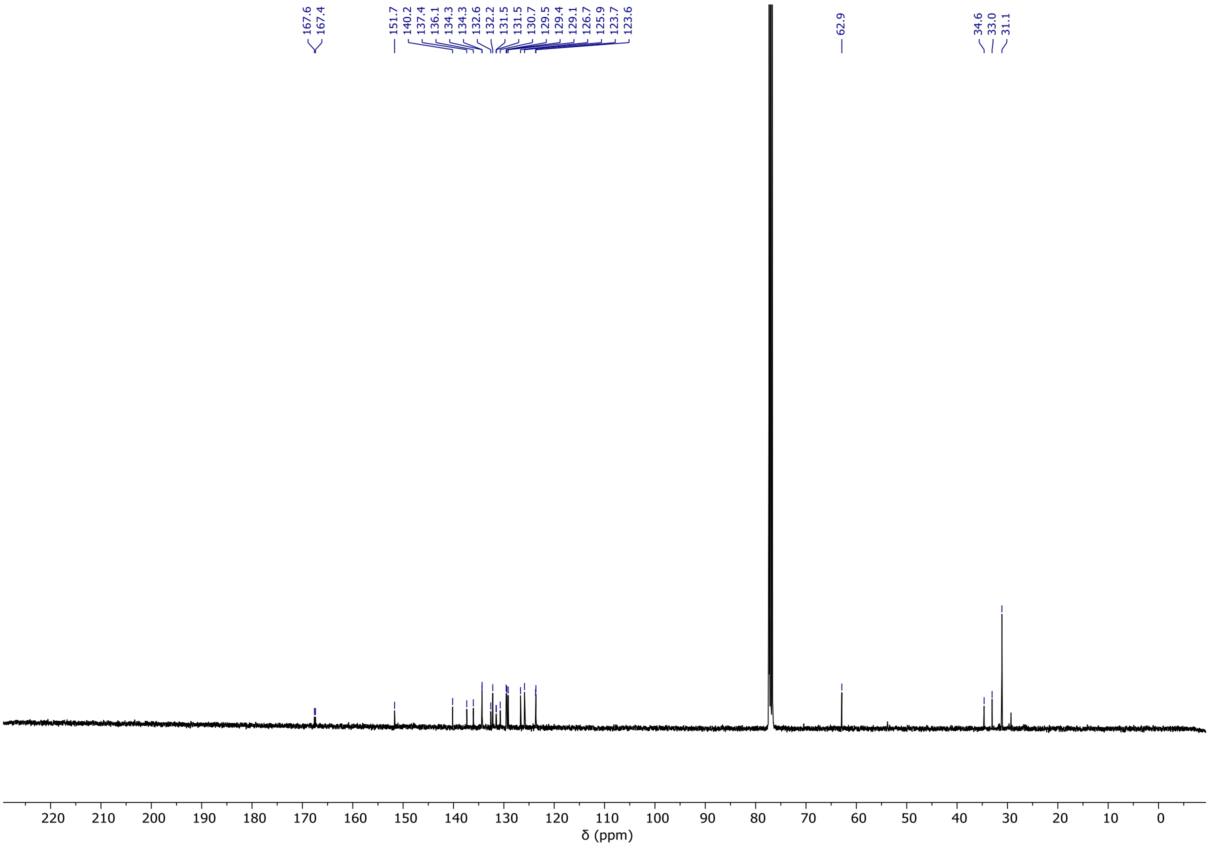
167.6 167.4
151.7 140.2 137.4 136.1 134.3 134.3 132.6 132.2 131.5 131.5 130.7 129.5 129.4 129.1 126.7 125.9 123.7 123.6
62.9
34.6 33.0 31.1
220 210 200 190 180 170 160 150 140 130 120 110 100 90 80 70 60 50 40 30 20 10 0
δ (ppm)

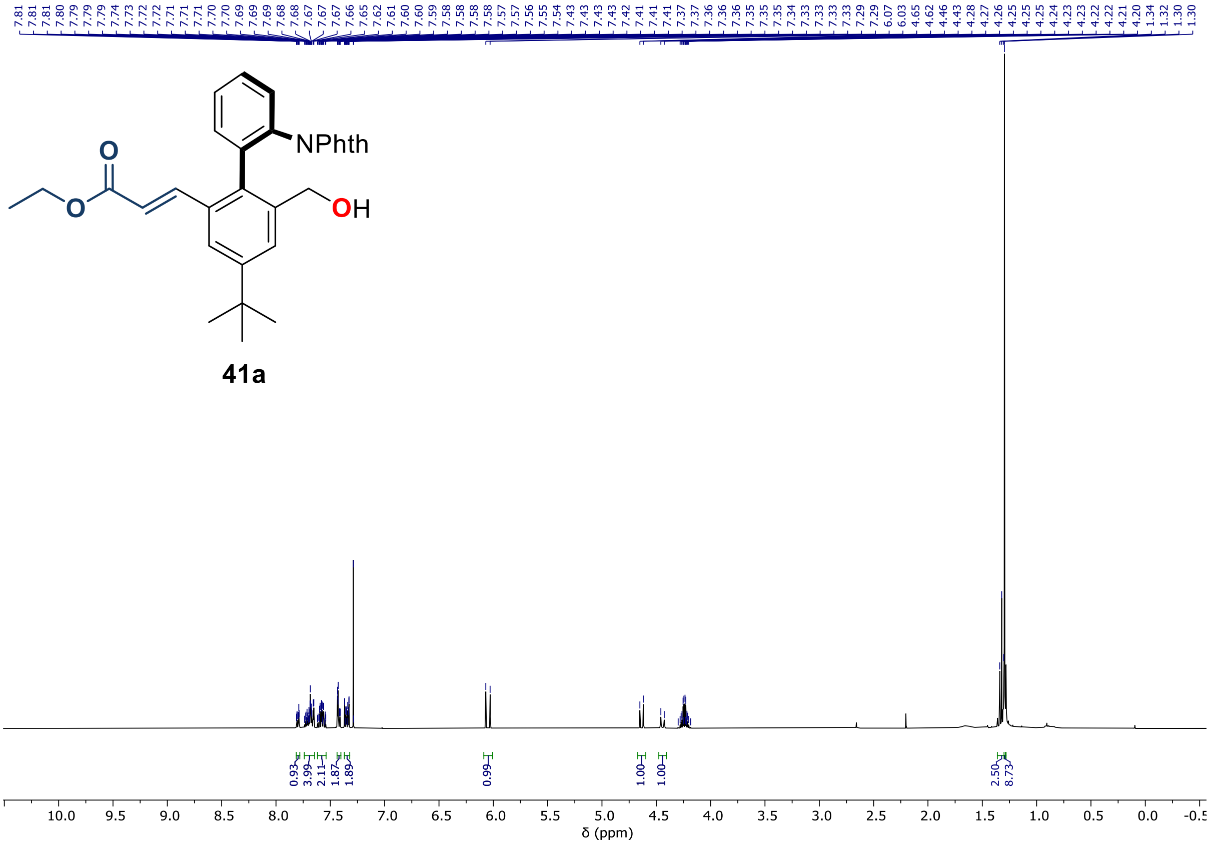

7.81 7.81 7.81 7.80 7.79 7.79 7.79 7.74 7.73 7.72 7.72 7.71 7.71 7.71 7.70 7.70 7.69 7.69 7.69 7.68 7.67 7.67 7.67 7.66 7.65 7.62 7.61 7.60 7.60 7.59 7.58 7.58 7.58 7.58 7.57 7.57 7.56 7.55 7.54 7.43 7.43 7.43 7.42 7.41 7.41 7.41 7.37 7.37 7.36 7.36 7.35 7.35 7.34 7.33 7.33 7.33 7.33 7.29 7.29 6.07 6.03 4.65 4.62 4.46 4.43 4.28 4.27 4.26 4.25 4.25 4.25 4.24 4.23 4.23 4.22 4.22 4.21 4.20 1.34 1.32 1.30 1.30
O
O
NPhth
OH
41a
0.93 3.99 2.11 1.87 1.89
0.99
1.00 1.00
2.50 8.73
10.0 9.5 9.0 8.5 8.0 7.5 7.0 6.5 6.0 5.5 5.0 4.5 4.0 3.5 3.0 2.5 2.0 1.5 1.0 0.5 0.0 -0.5
δ (ppm)

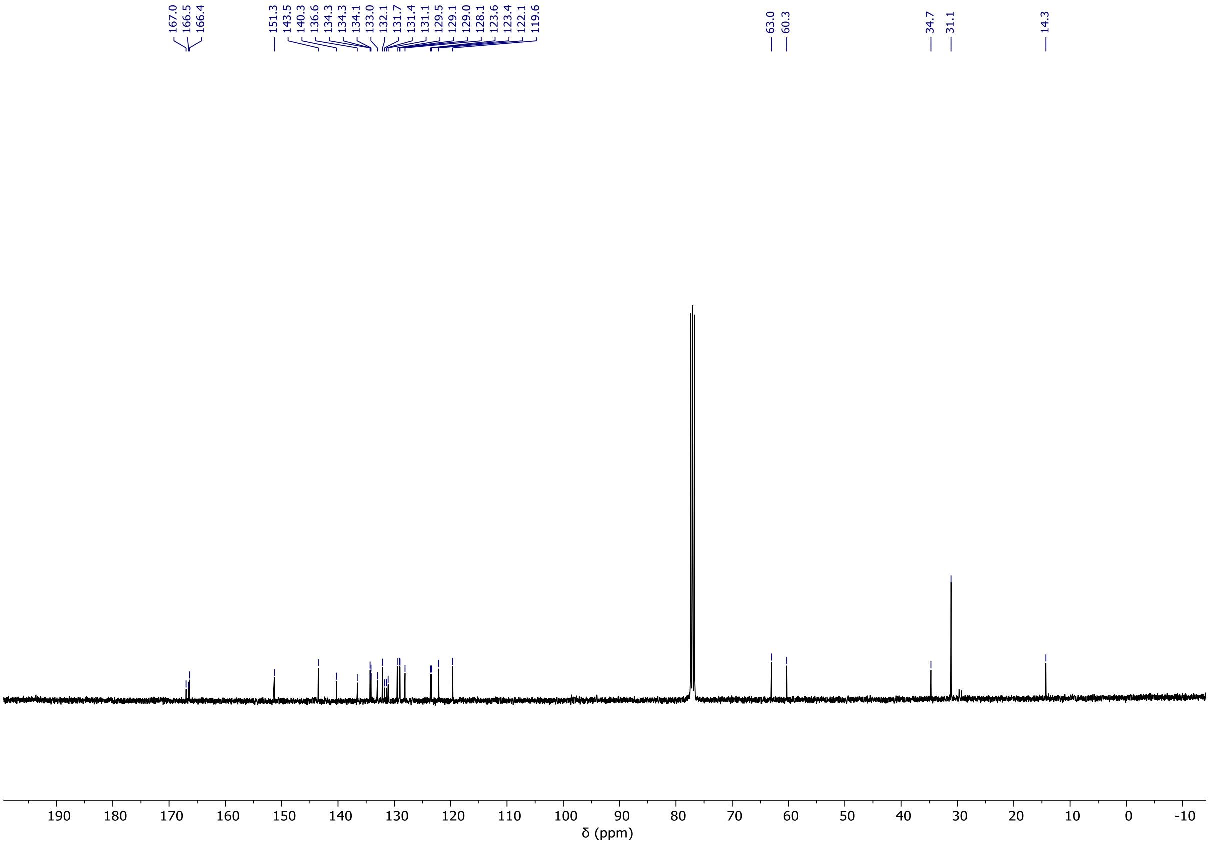

167.0 166.5 166.4
151.3 143.5 140.3 136.6 134.3 134.1 133.0 132.1 131.7 131.4 131.1 129.5 129.1 129.0 128.1 123.6 123.4 122.1 119.6
63.0 60.3
34.7 31.1
14.3
190 180 170 160 150 140 130 120 110 100 90 80 70 60 50 40 30 20 10 0 -10
δ (ppm)

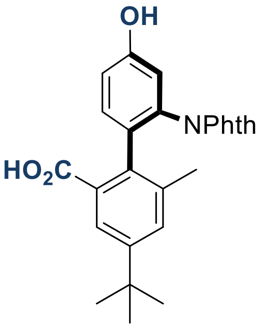
OH
NPhth
HO2C

42

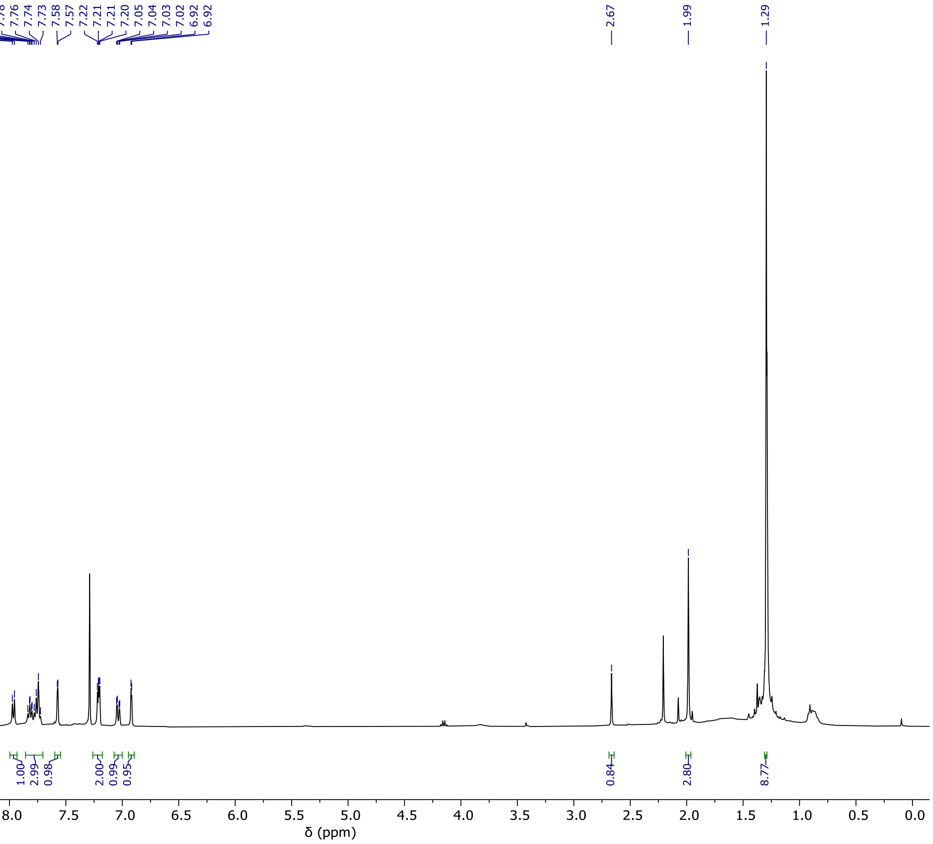
7.97
7.96
7.84
7.82
7.82
7.80
7.80
7.78
7.76
7.74
7.73
7.58
7.57
7.22
7.21
7.21
7.20
7.05
7.04
7.03
7.02
6.92
6.92
2.67
1.99
1.29
1.00
2.99
0.98
2.00
0.99
0.95
0.84
2.80
8.77
δ (ppm)

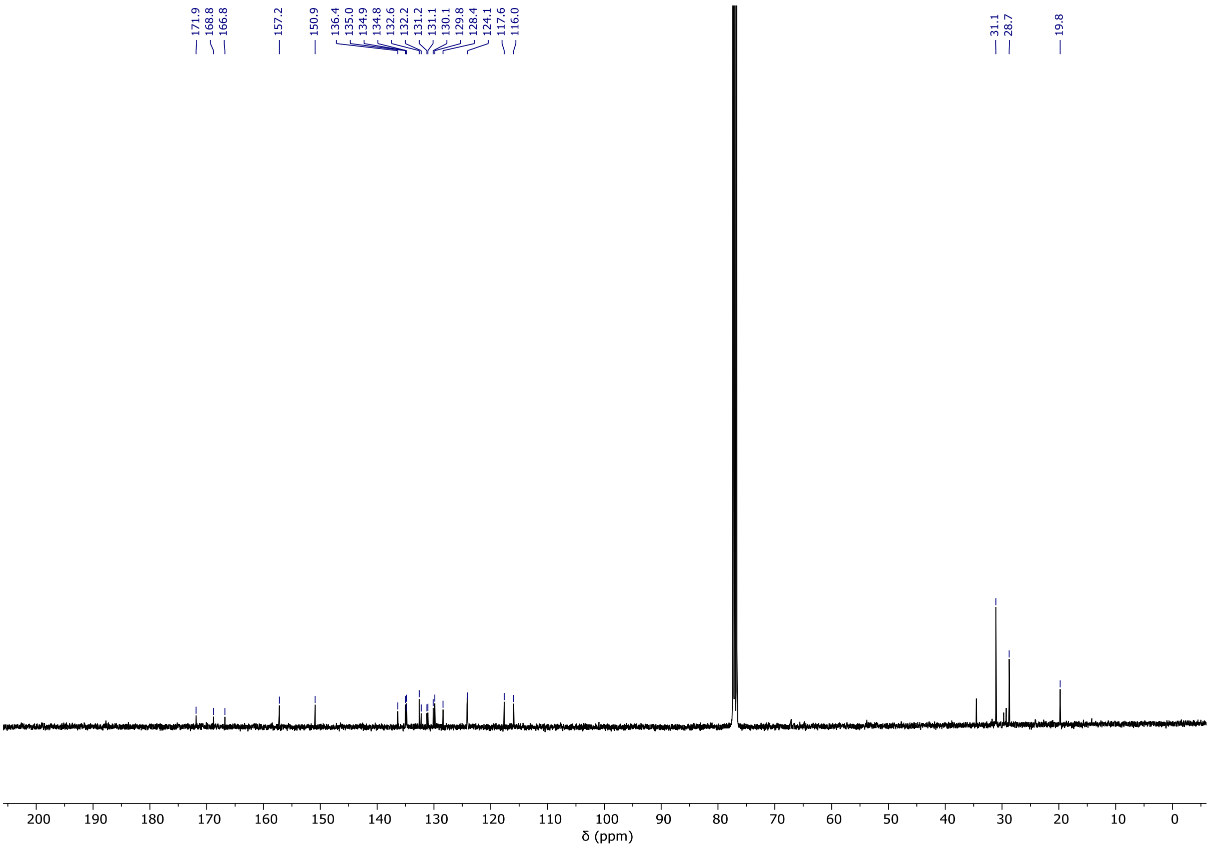
171.9
168.8
166.8
157.2
150.9
136.4
135.0
134.9
134.8
132.6
132.2
131.2
131.1
130.1
129.8
128.4
124.1
117.6
116.0
31.1
28.7
19.8
δ (ppm)

12. SFC traces of the isolated products

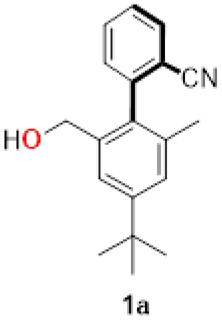

1a

SFC Analysis: Chiralpak IG, CO_2:2-propanol (10%), 35ºC, flow rate = 1.2 mL/min, λ = 210 nm. rt_1 = 1.5 min, rt_2 = 2.6 min.

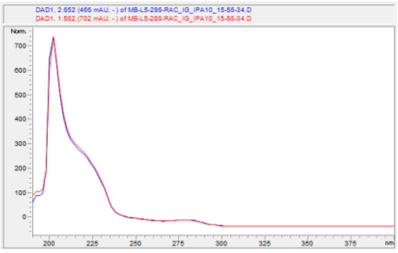

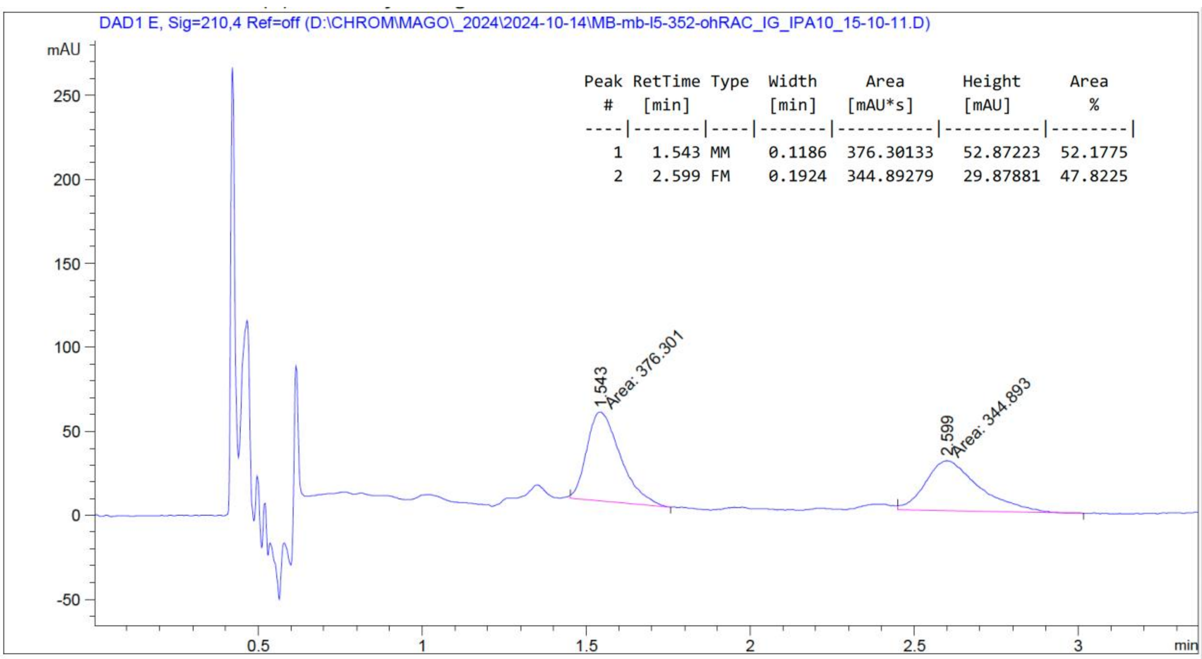

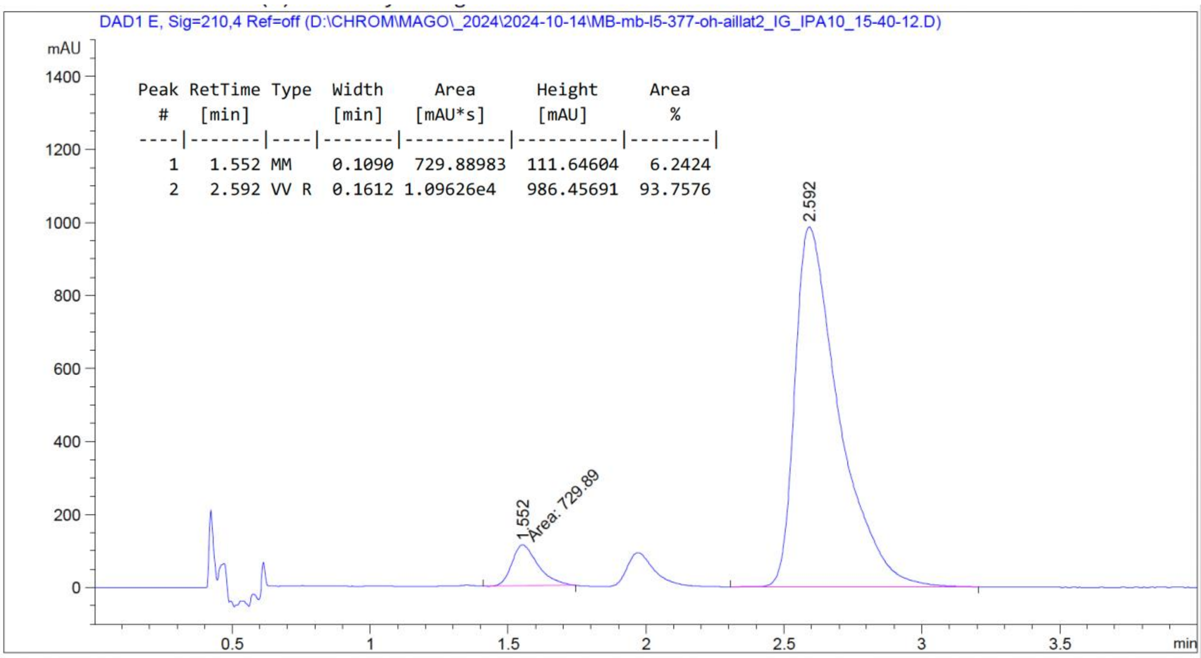

SFC Analysis: Chiralpak IA, CO_2:MeOH (5%), 35ºC, flow rate = 1.4mL/min, λ = 210 nm. rt_1 = 1.0 min, rt_2 = 1.4 min.

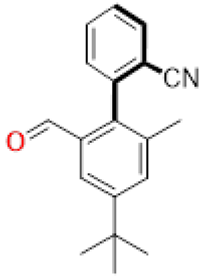

1b

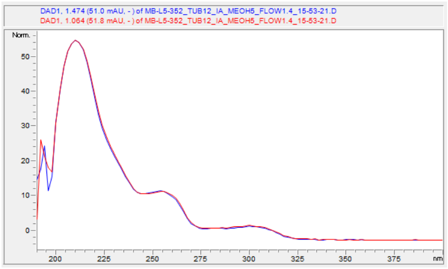

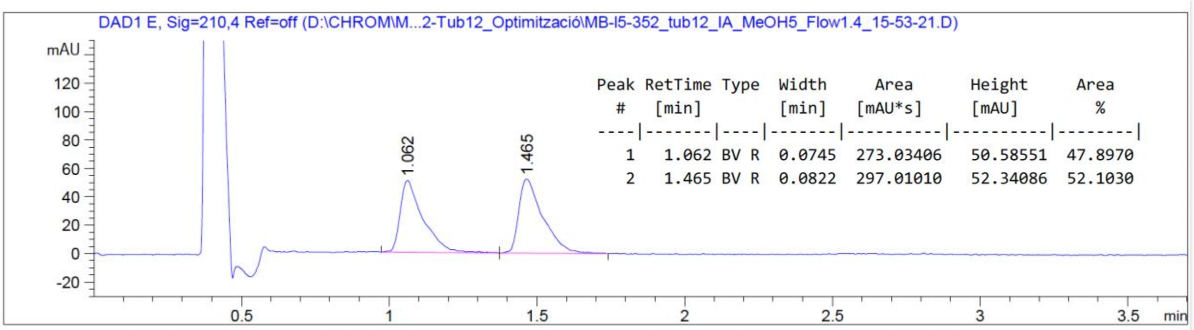

Peak #	RetTime [min]	Type	Width [min]	Area [mAU*s]	Height [mAU]	Area %
1	1.062	BV R	0.0745	273.03406	50.58551	47.8970
2	1.465	BV R	0.0822	297.01010	52.34086	52.1030

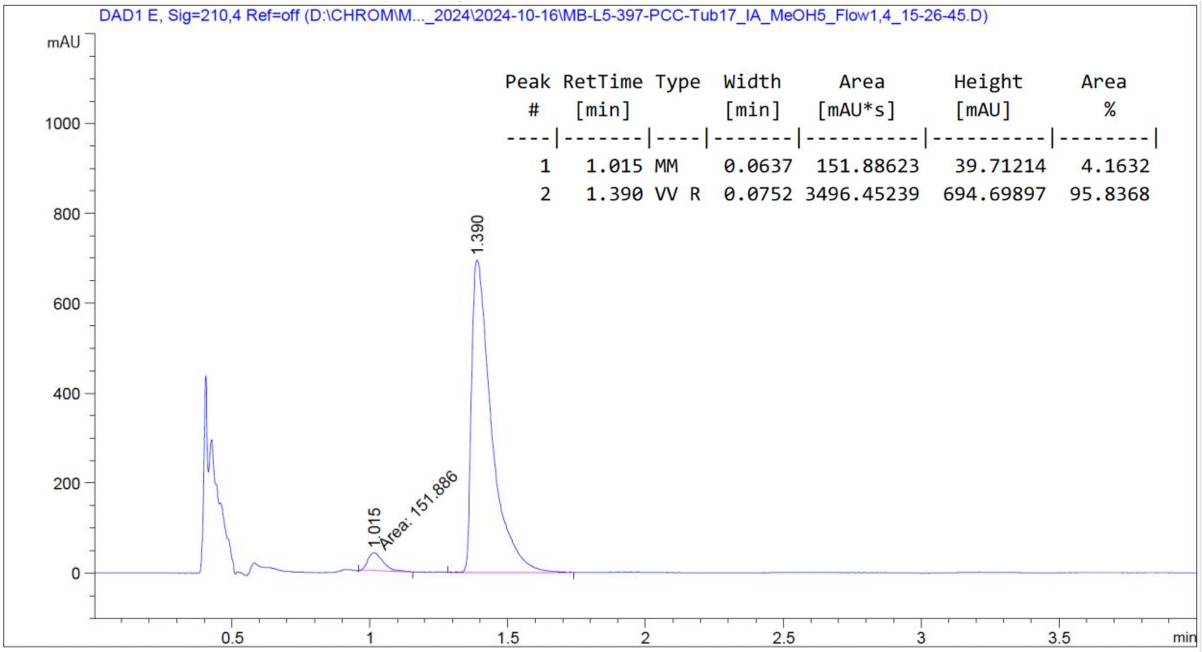

Peak #	RetTime [min]	Type	Width [min]	Area [mAU*s]	Height [mAU]	Area %
1	1.015	MM	0.0637	151.88623	39.71214	4.1632
2	1.390	VV R	0.0752	3496.45239	694.69897	95.8368

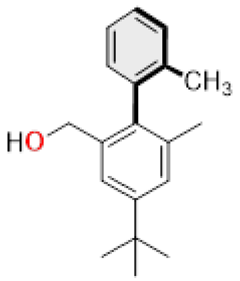

2a

SFC Analysis: Chiralpak IC, CO_2:2-propanol (3%), 35ºC, flow rate = 1.4 mL/min, λ = 210 nm. rt_1 = 2.7 min, rt_2 = 3.7 min.

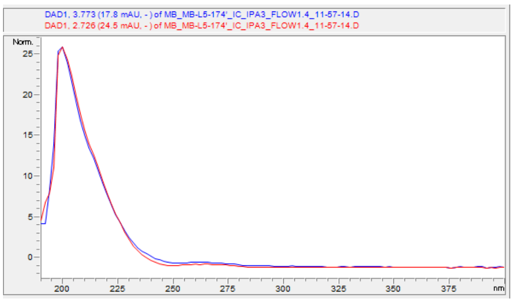

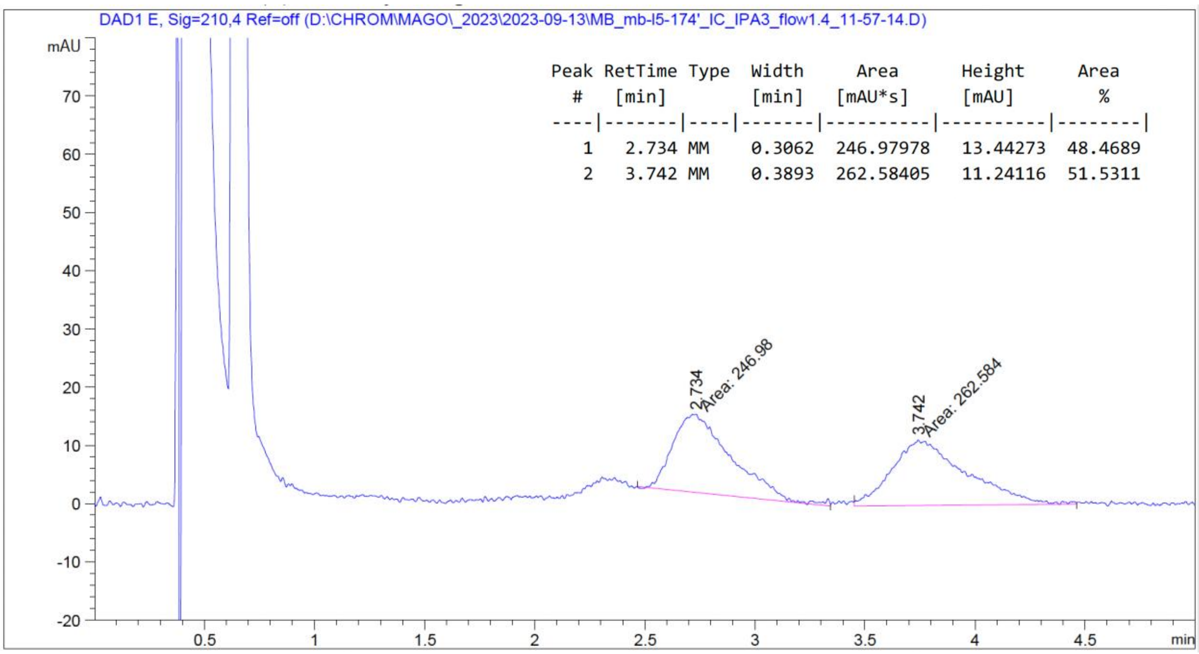

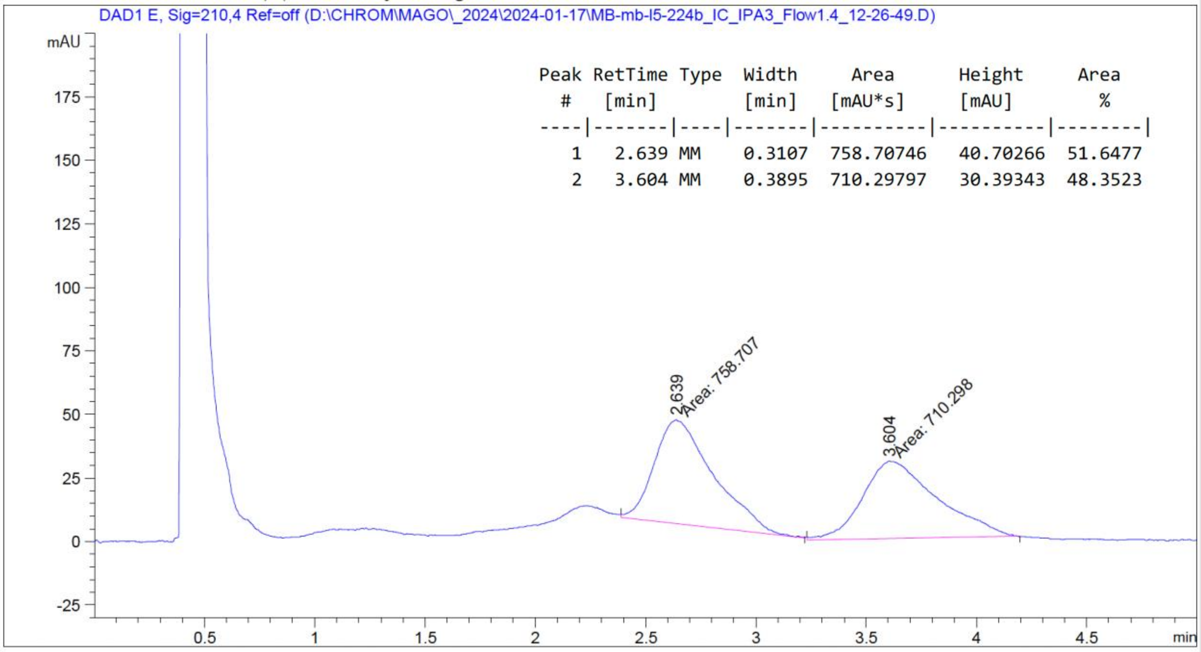

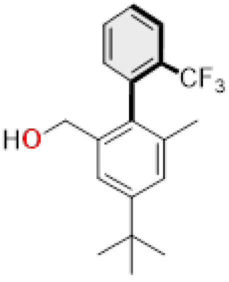

3a

SFC Analysis: Chiralpak IB, CO_2:EtOH (1.5%), 35ºC, flow rate = 1.9 mL/min, λ = 210 nm. rt_1 = 1.5 min, rt_2 = 1.9 min.

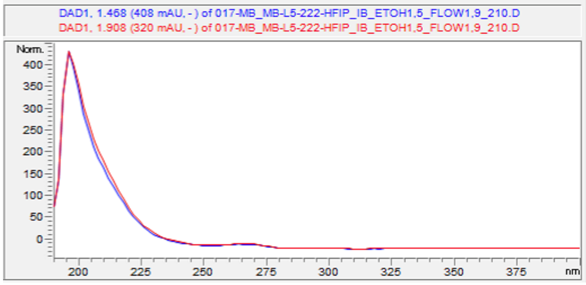

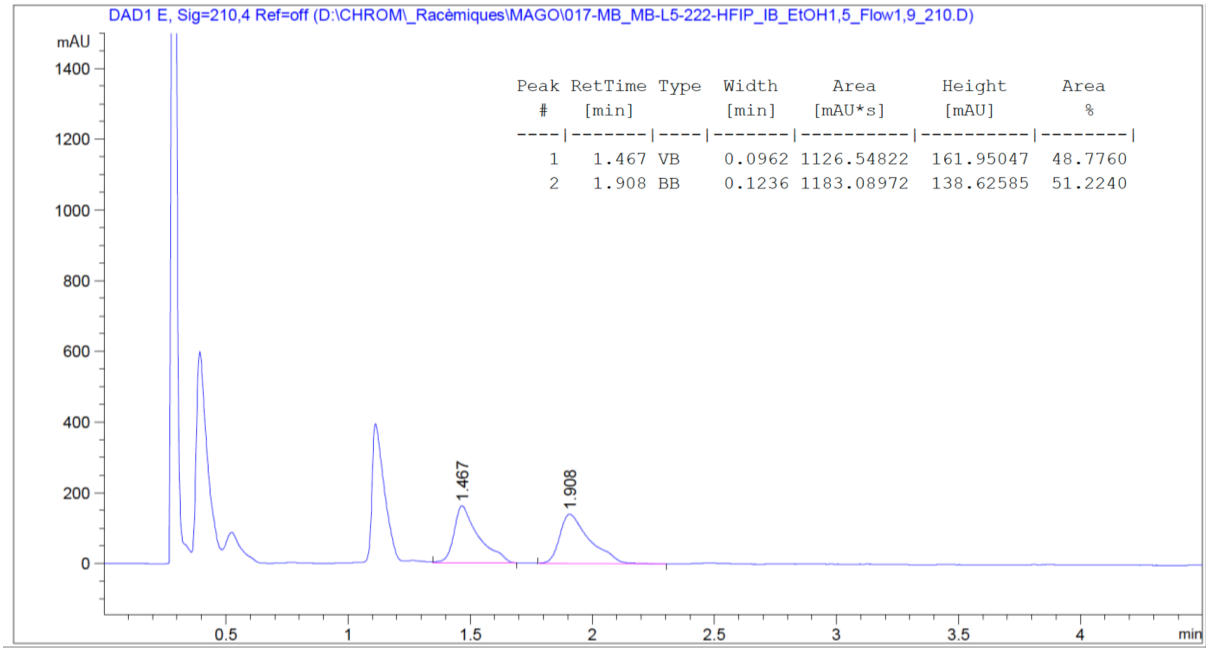

Peak #	RetTime [min]	Type	Width [min]	Area [mAU*s]	Height [mAU]	Area %
1	1.467	VB	0.0962	1126.54822	161.95047	48.7760
2	1.908	BB	0.1236	1183.08972	138.62585	51.2240

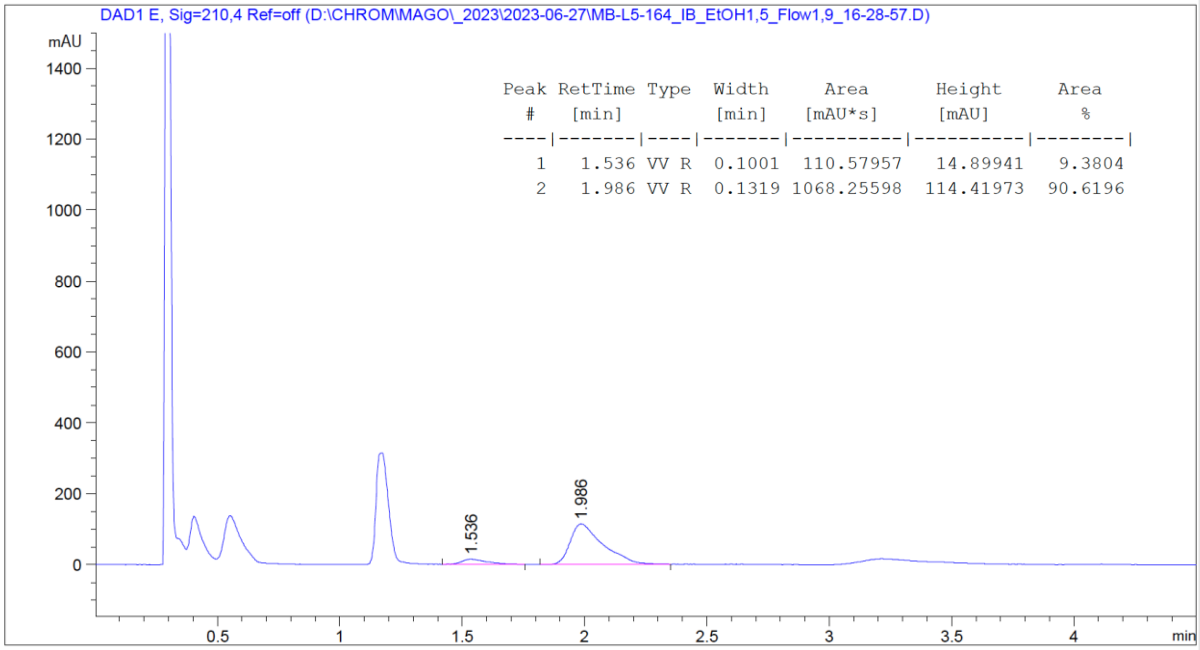

Peak #	RetTime [min]	Type	Width [min]	Area [mAU*s]	Height [mAU]	Area %
1	1.536	VV R	0.1001	110.57957	14.89941	9.3804
2	1.986	VV R	0.1319	1068.25598	114.41973	90.6196

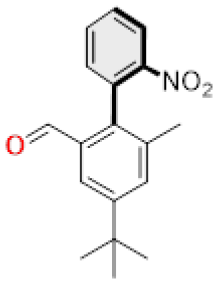

4b

SFC Analysis: Chiralpak IG, CO_2:MeOH (10%), 35ºC, flow rate = 1.2 mL/min, λ = 210 nm. rt_1 = 0.9 min, rt_2 = 1.2 min.

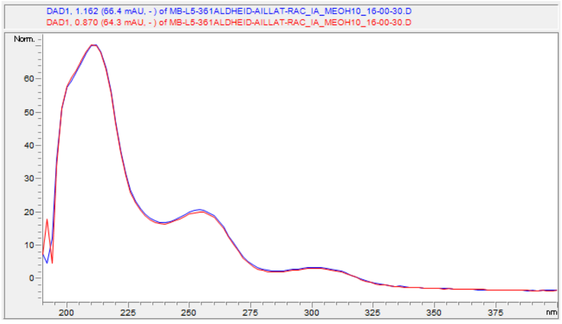

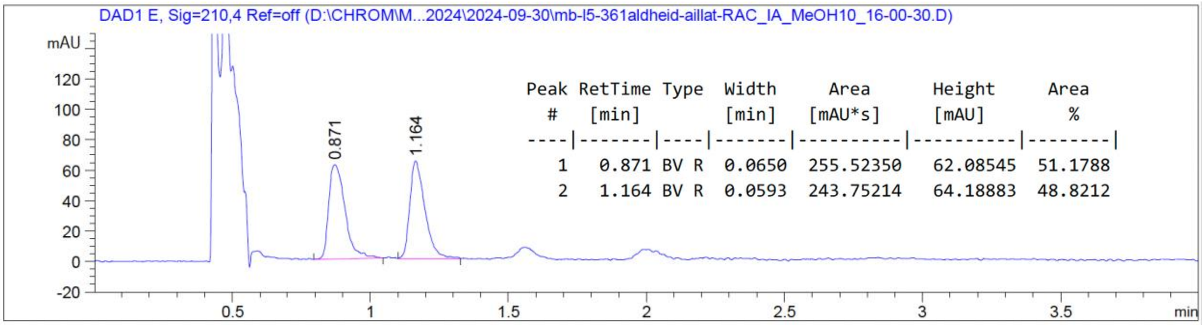

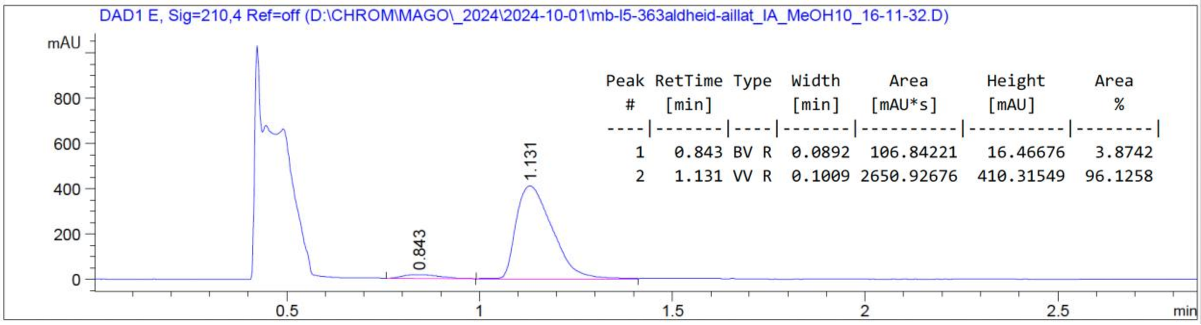

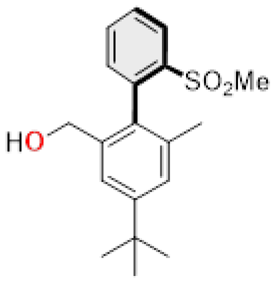

5a

SFC Analysis: Chiralpak IG, CO_2:MeOH (20%), 35ºC, flow rate = 1.2 mL/min, λ = 210 nm. rt_1 = 1.4 min, rt_2 = 1.8 min.

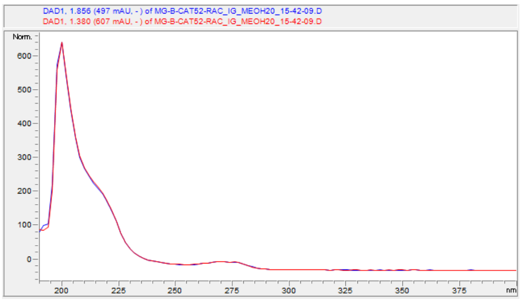

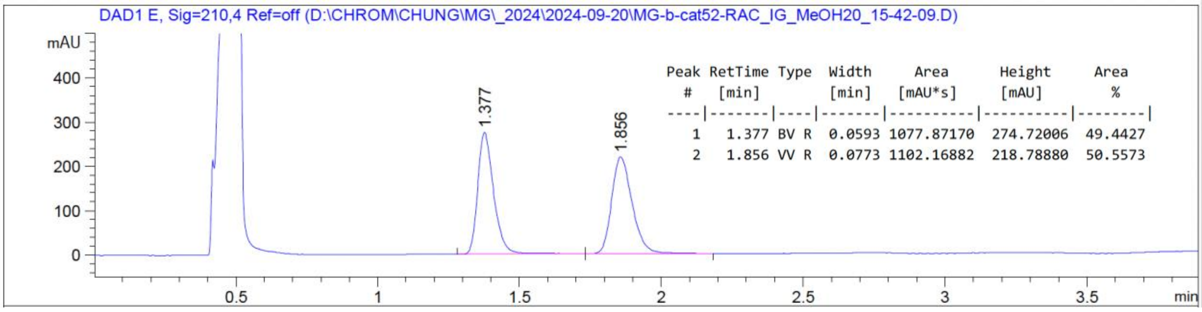

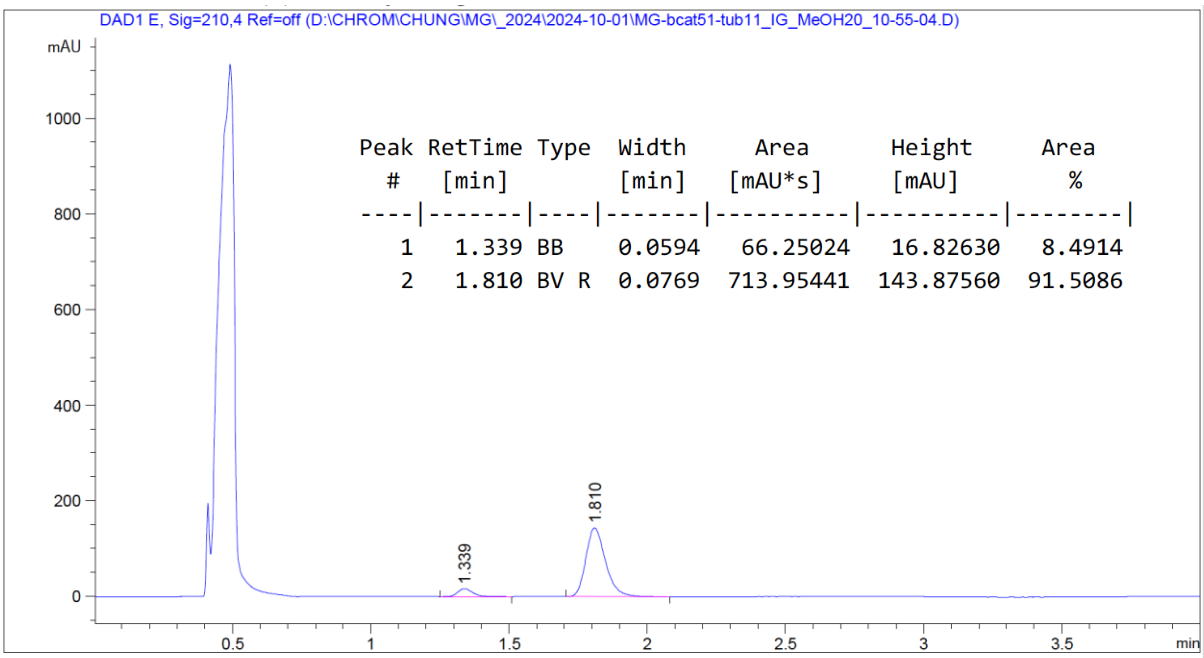

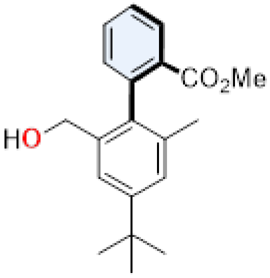

6a

SFC Analysis: Chiralpak IB, CO_2:2-propanol (10%), 35ºC, flow rate = 1.2 mL/min, λ = 210 nm. rt_1 = 1.2 min, rt_2 = 1.6 min.

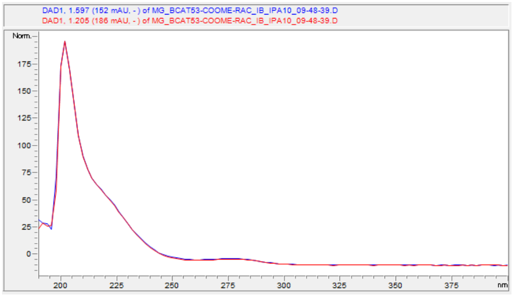

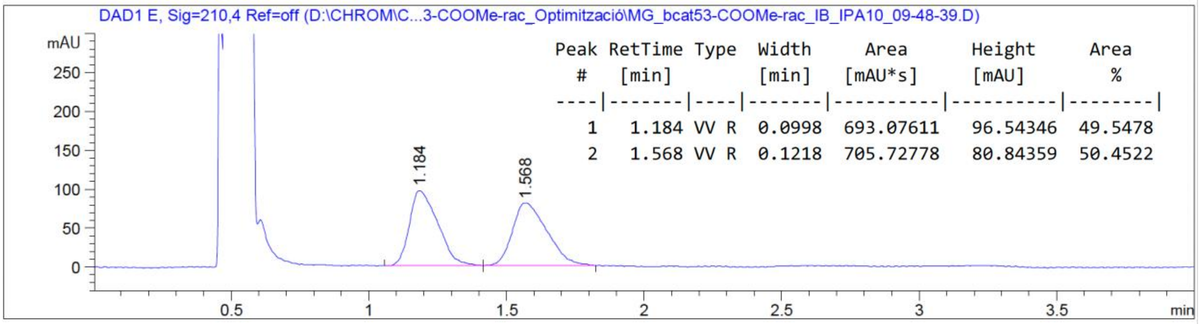

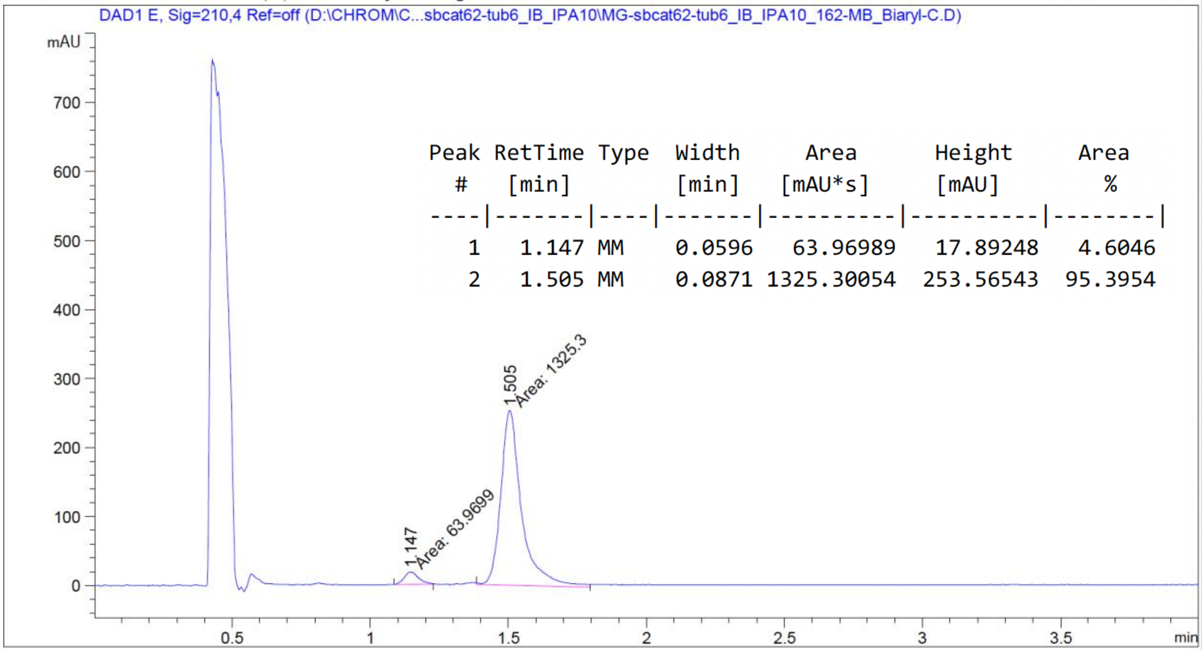

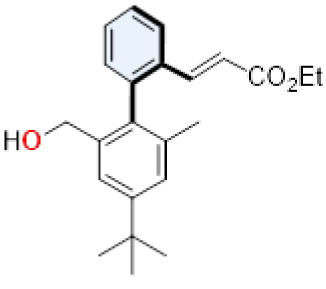

7a

SFC Analysis: Chiralpak IC, CO_2:IPA (4%), 35ºC, flow rate=1.5 mL/min, λ = 210 nm. rt_1 = 3.9 min, rt_2 = 4.5 min.

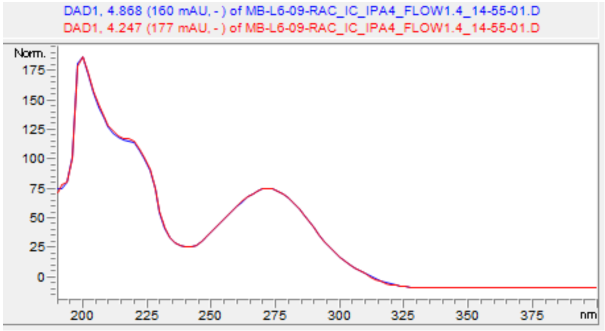

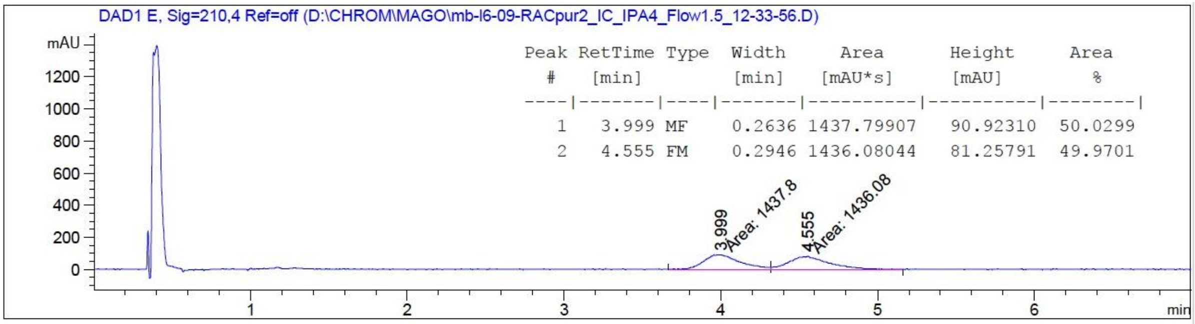

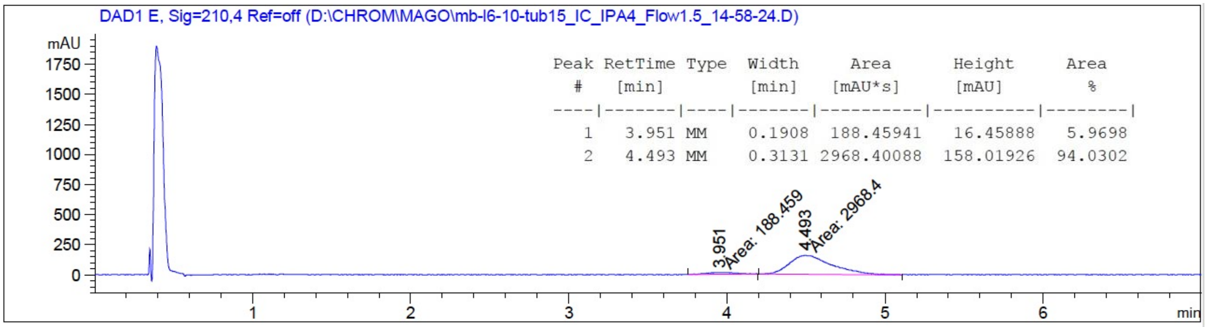

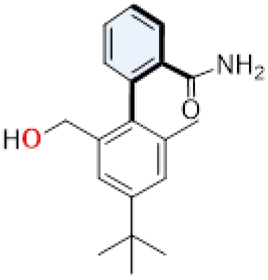

8a

SFC Analysis: Chiralpak IC, CO_2:EtOH (15%), 35ºC, flow rate = 1.2 mL/min, λ = 210 nm. rt_1 = 3.4 min, rt_2 = 4.0 min.

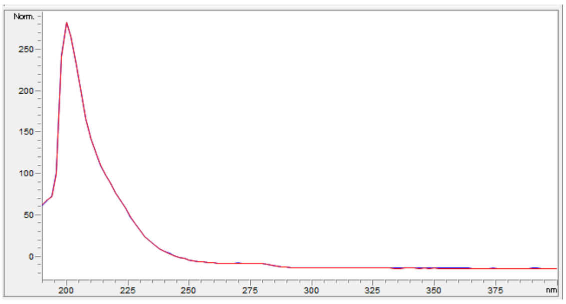

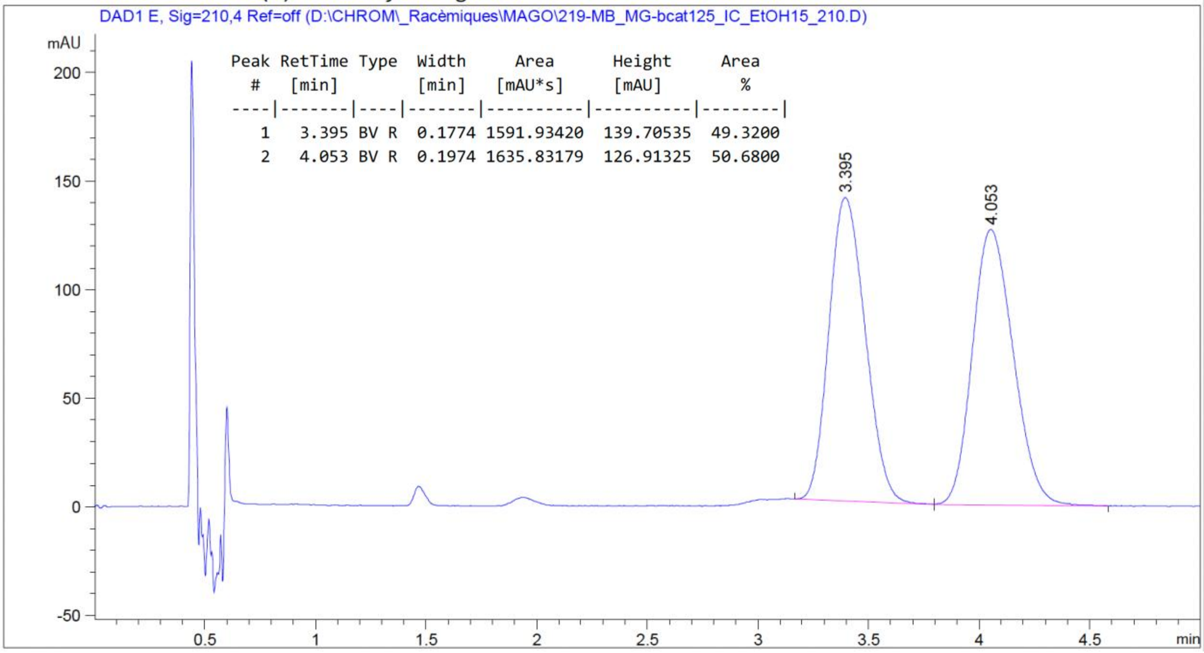

Peak #	RetTime [min]	Type	Width [min]	Area [mAU*s]	Height [mAU]	Area %
1	3.395	BV R	0.1774	1591.93420	139.70535	49.3200
2	4.053	BV R	0.1974	1635.83179	126.91325	50.6800

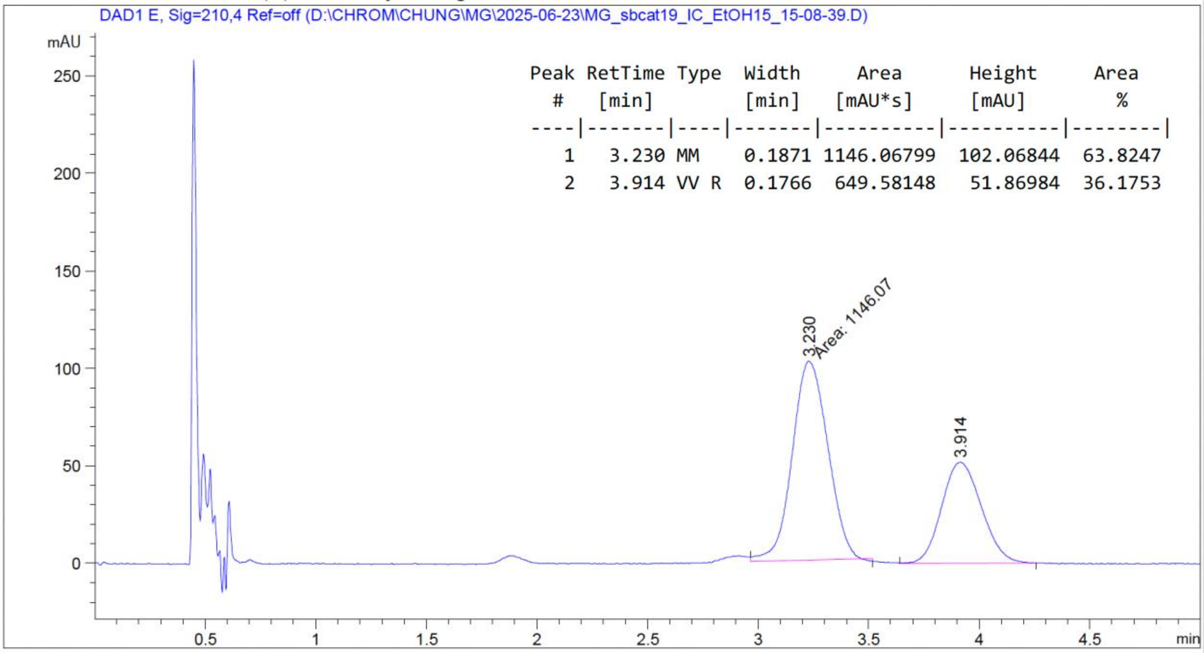

Peak #	RetTime [min]	Type	Width [min]	Area [mAU*s]	Height [mAU]	Area %
1	3.230	MM	0.1871	1146.06799	102.06844	63.8247
2	3.914	VV R	0.1766	649.58148	51.86984	36.1753

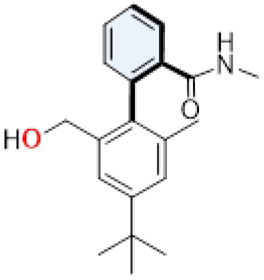

9a

SFC Analysis: Chiralpak IC, CO_2:2-propanol (30%), 35ºC, flow rate = 1.2 mL/min, λ = 210 nm. rt_1 = 1.3 min, rt_2 = 1.9 min.

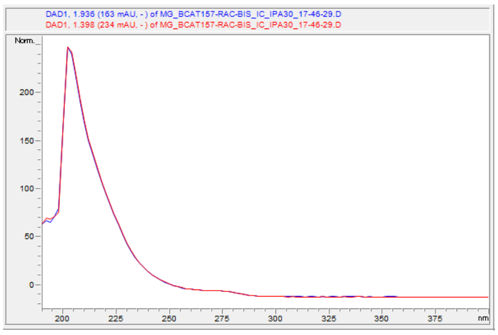

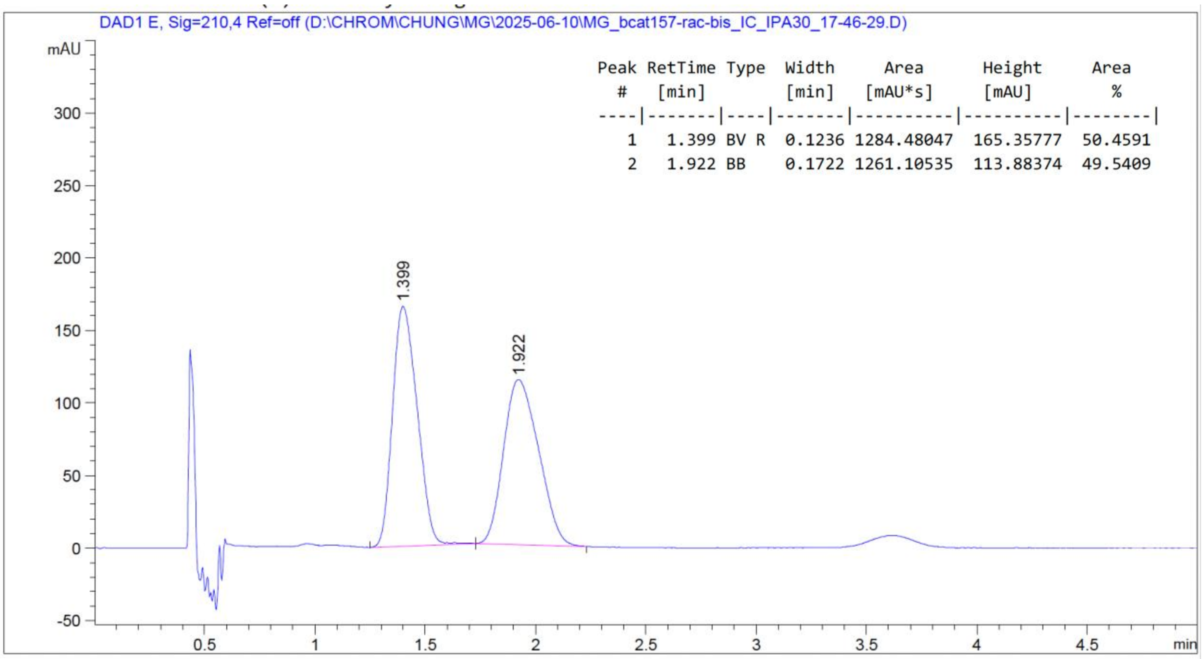

Peak #	RetTime [min]	Type	Width [min]	Area [mAU*s]	Height [mAU]	Area %
1	1.399	BV R	0.1236	1284.48047	165.35777	50.4591
2	1.922	BB	0.1722	1261.10535	113.88374	49.5409

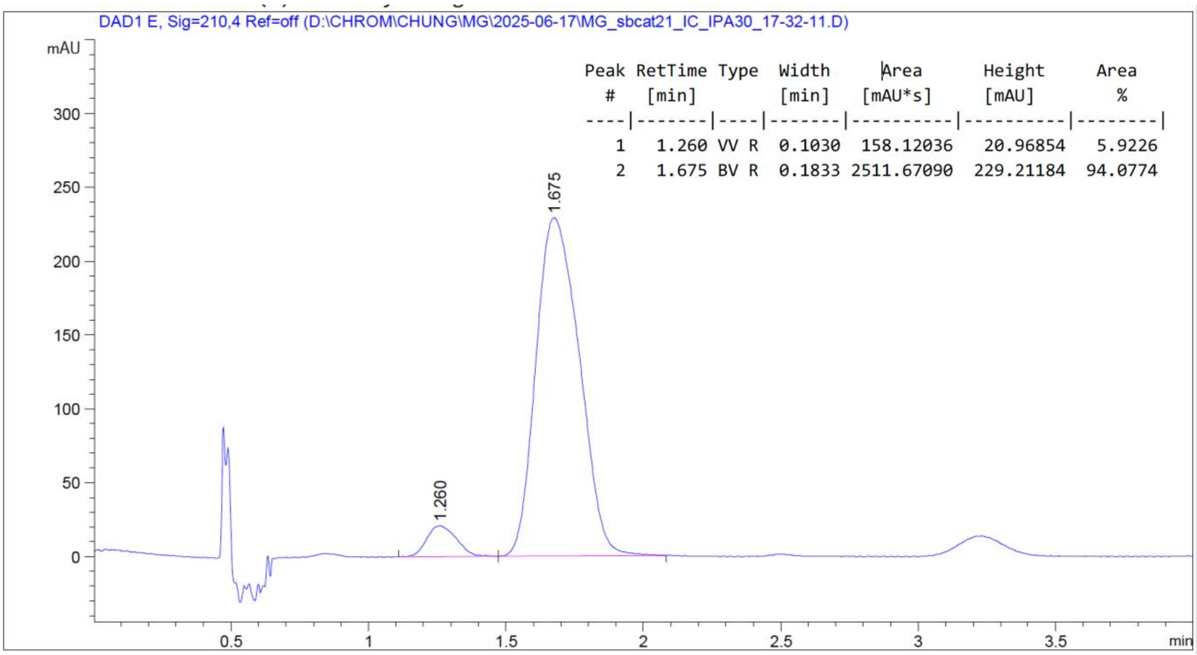

Peak #	RetTime [min]	Type	Width [min]	Area [mAU*s]	Height [mAU]	Area %
1	1.260	VV R	0.1030	158.12036	20.96854	5.9226
2	1.675	BV R	0.1833	2511.67090	229.21184	94.0774

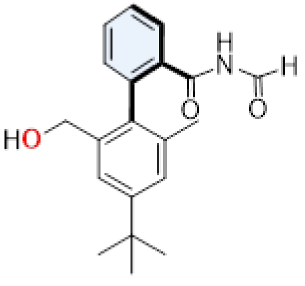

10a

SFC Analysis: Chiralpak IC, CO_2:2-propanol (15%), 35ºC, flow rate = 1.2 mL/min, λ = 210 nm. rt_1 = 1.5 min, rt_2 = 1.9 min.

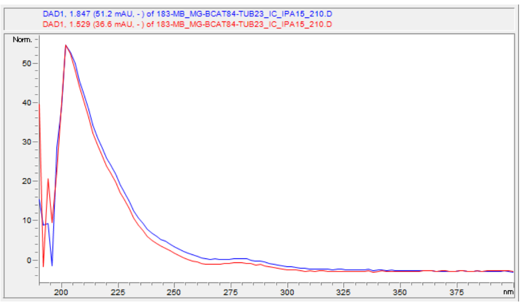

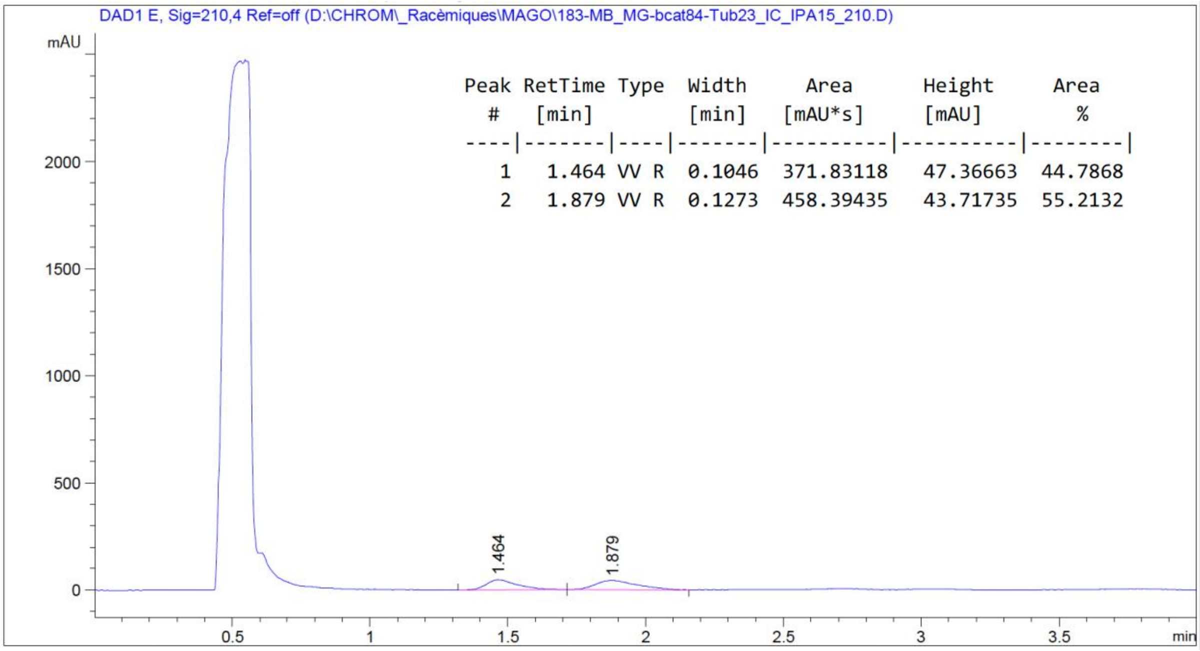

Peak #	RetTime [min]	Type	Width [min]	Area [mAU*s]	Height [mAU]	Area %
1	1.464	VV R	0.1046	371.83118	47.36663	44.7868
2	1.879	VV R	0.1273	458.39435	43.71735	55.2132

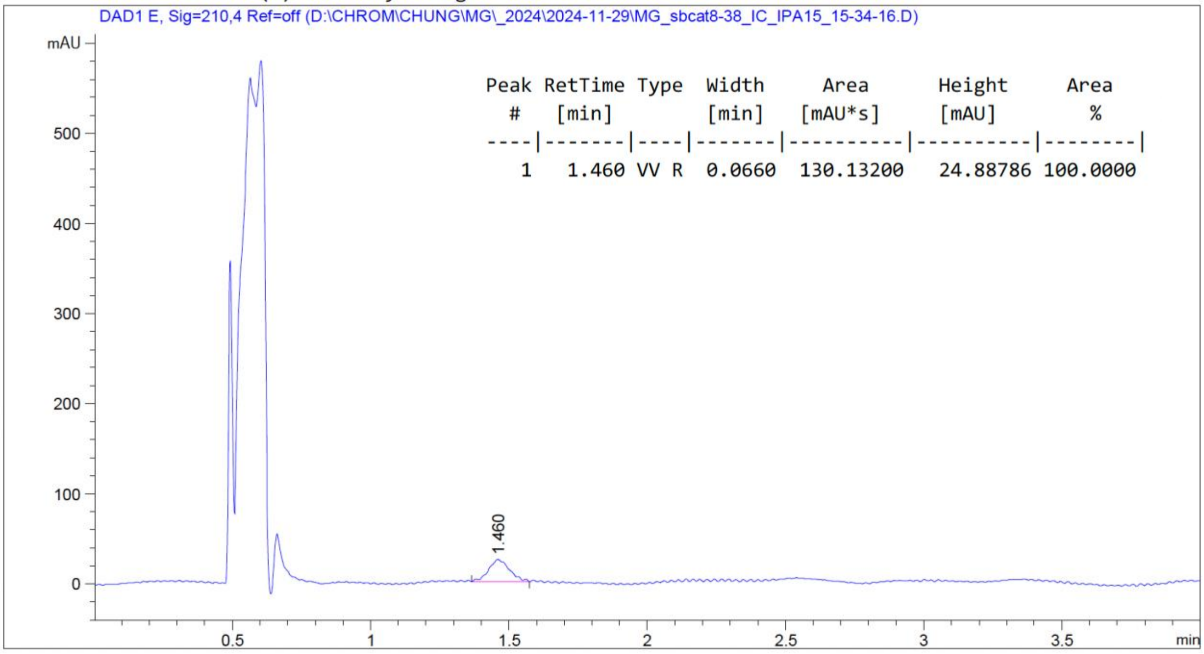

Peak #	RetTime [min]	Type	Width [min]	Area [mAU*s]	Height [mAU]	Area %
1	1.460	VV R	0.0660	130.13200	24.88786	100.0000

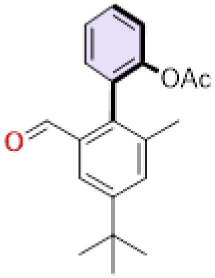

11b

SFC Analysis: Chiralpak IC, CO_2:2-propanol (10%), 35ºC, flow rate = 1.2 mL/min, λ = 210 nm. rt_1 = 1.3 min, rt_2 = 1.7 min.

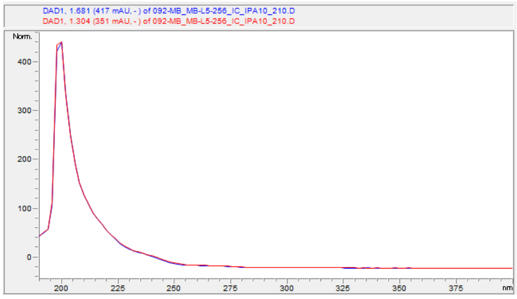

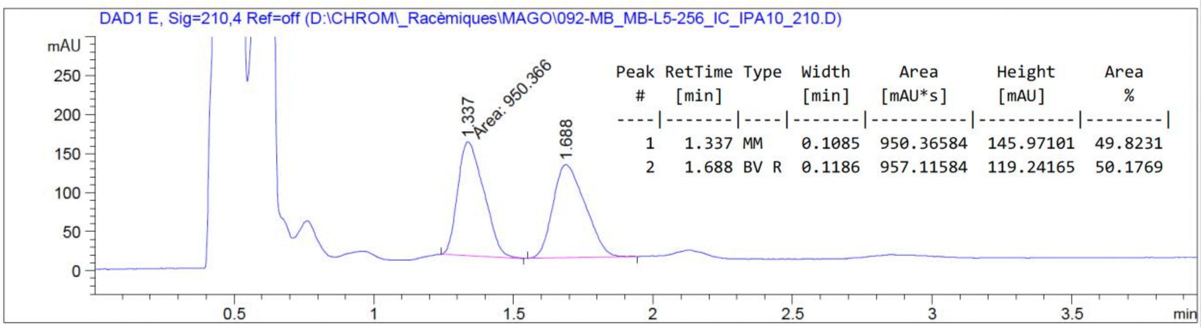

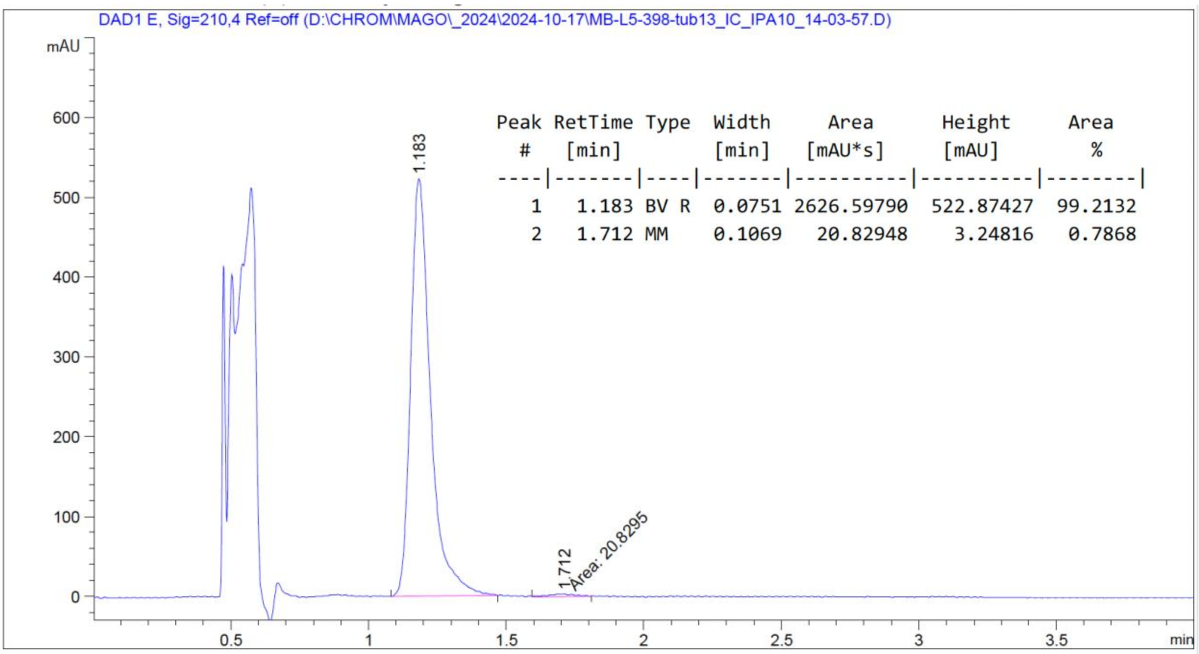

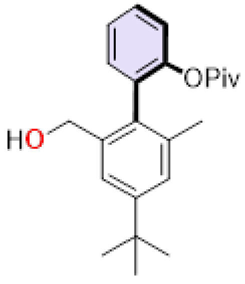

12a

SFC Analysis: Chiralpak IB, CO_2:MeOH (5%), 35ºC, flow rate = 0.8 mL/min, λ = 210 nm. rt_1 = 1.4 min, rt_2 = 1.7 min.

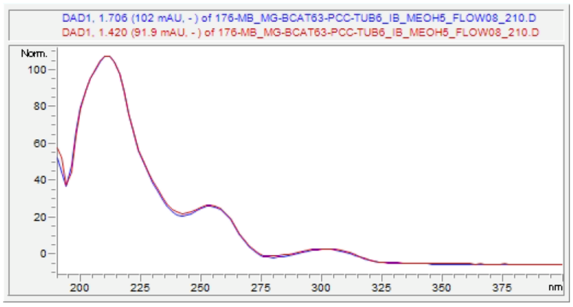

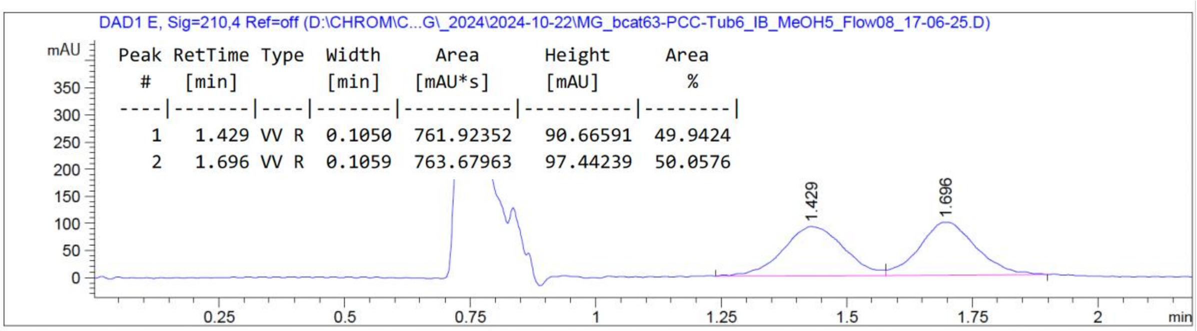

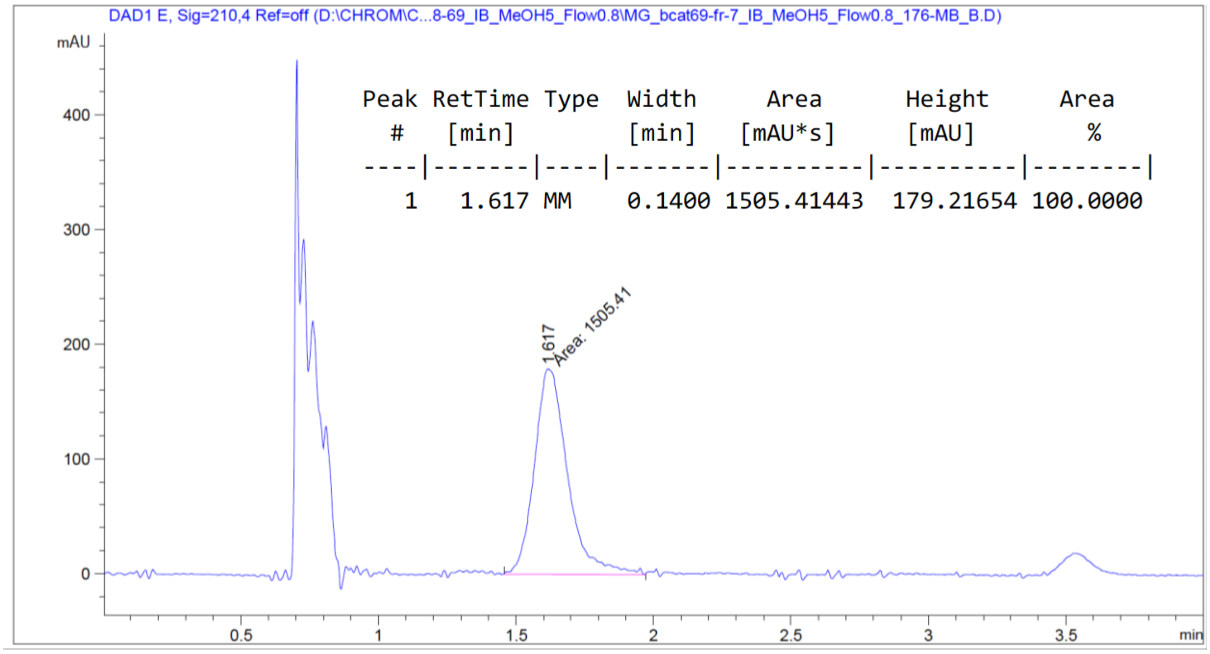

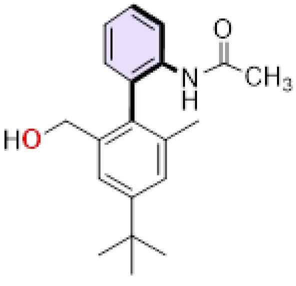

13a

SFC Analysis: Chiralpak IC, CO_2:2-propanol (20 %), 35ºC, flow rate = 2 mL/min, λ = 210 nm. rt_1 = 1.1 min, rt_2 = 1.7 min.

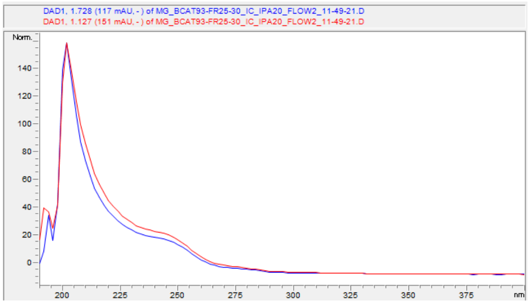

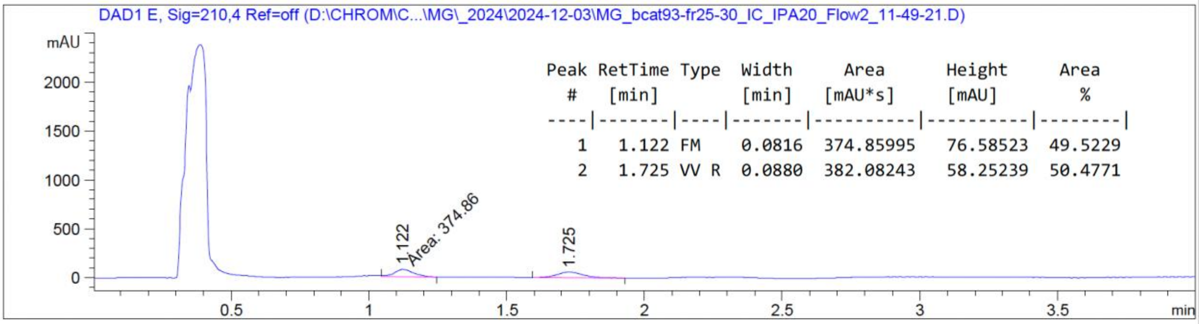

Peak #	RetTime [min]	Type	Width [min]	Area [mAU*s]	Height [mAU]	Area %
1	1.122	FM	0.0816	374.85995	76.58523	49.5229
2	1.725	VV R	0.0880	382.08243	58.25239	50.4771

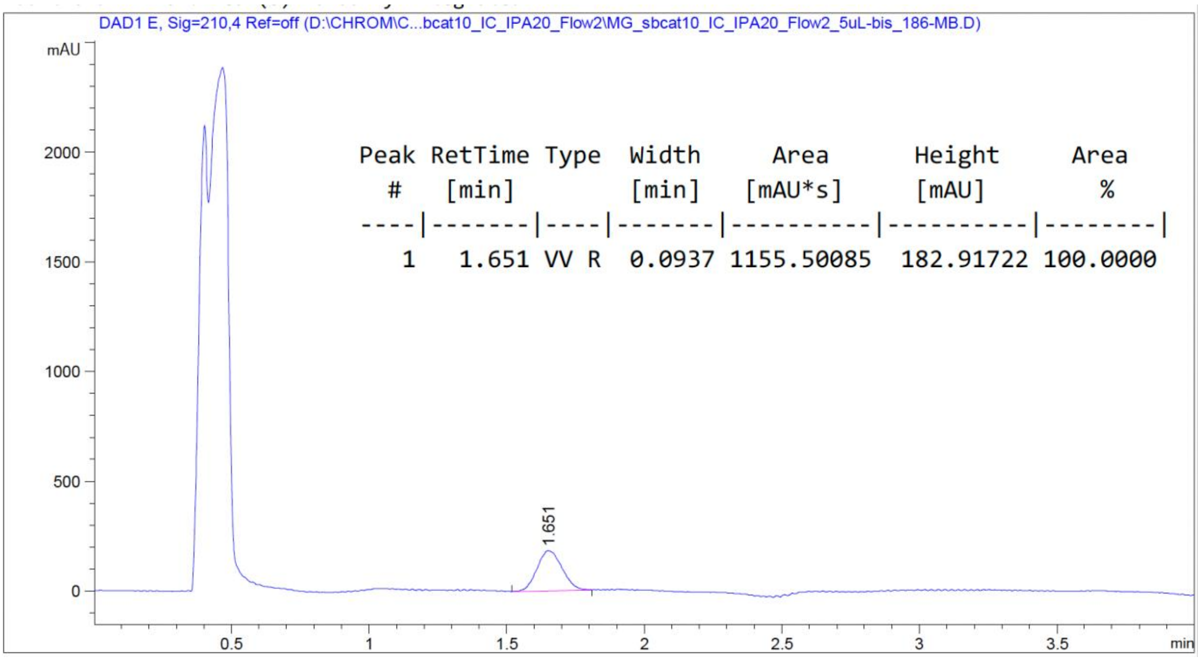

Peak #	RetTime [min]	Type	Width [min]	Area [mAU*s]	Height [mAU]	Area %
1	1.651	VV R	0.0937	1155.50085	182.91722	100.0000

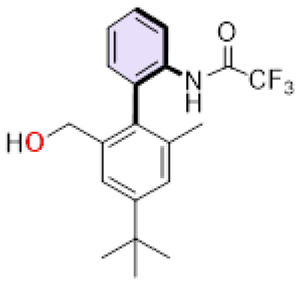

14a

SFC Analysis: Chiralpak IB, CO_2:MeOH (5%), 35ºC, flow rate = 0.8 mL/min, λ = 210 nm. rt_1 = 1.3 min, rt_2 = 1.6 min.

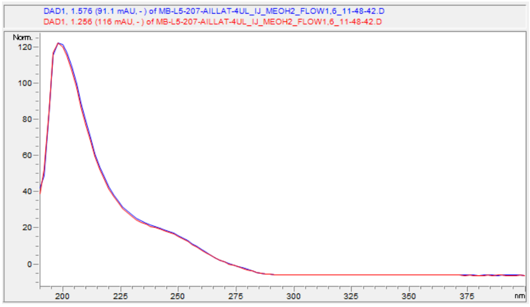

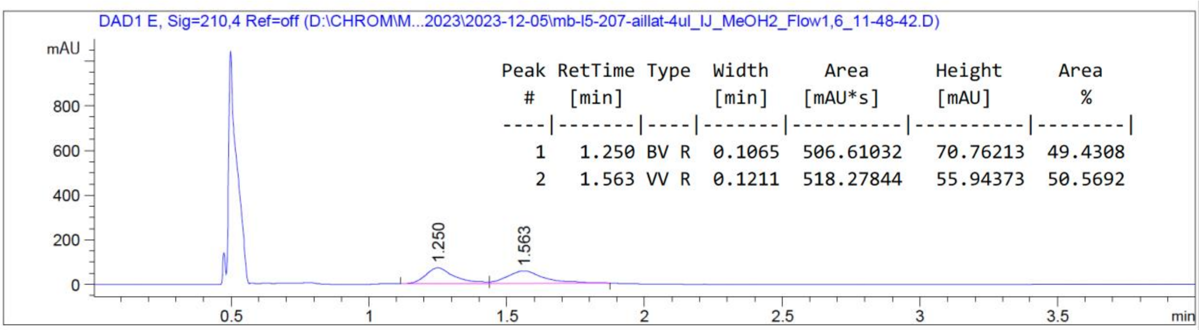

Peak #	RetTime [min]	Type	Width [min]	Area [mAU*s]	Height [mAU]	Area %
1	1.250	BV R	0.1065	506.61032	70.76213	49.4308
2	1.563	VV R	0.1211	518.27844	55.94373	50.5692

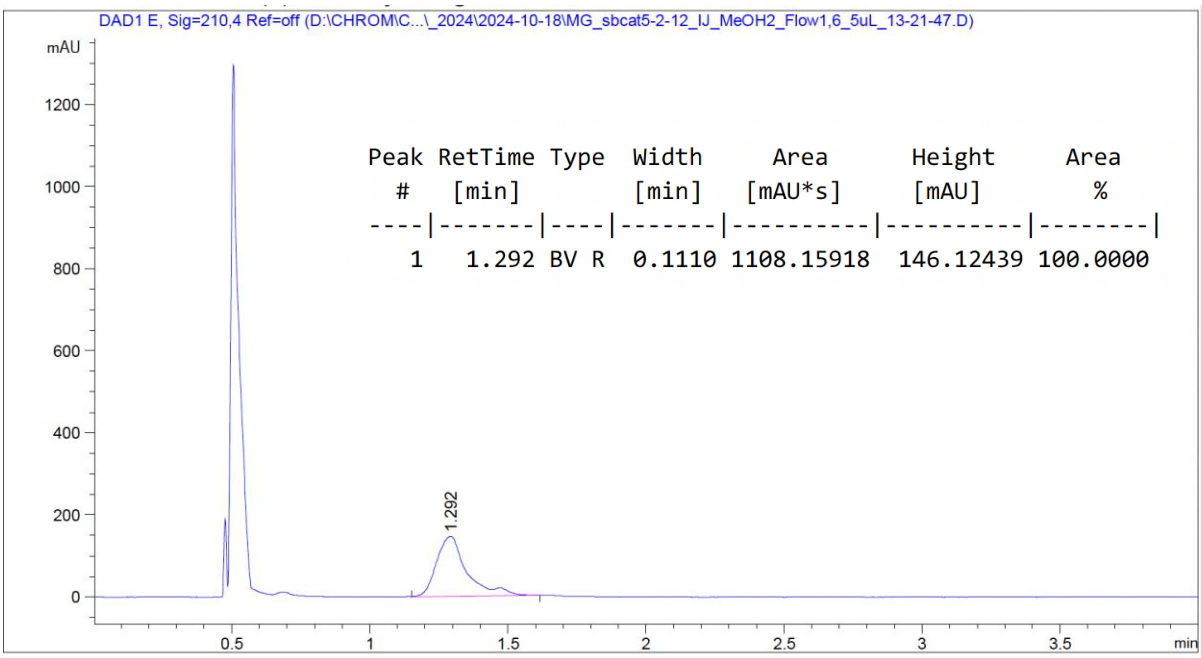

Peak #	RetTime [min]	Type	Width [min]	Area [mAU*s]	Height [mAU]	Area %
1	1.292	BV R	0.1110	1108.15918	146.12439	100.0000

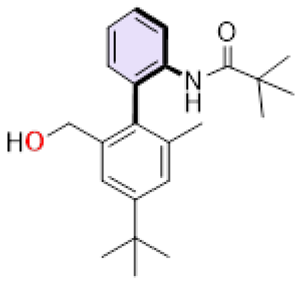

15a

SFC Analysis: Chiralpak IC, CO_2:MeOH (8%), 35ºC, flow rate = 1.5 mL/min, λ = 210 nm. rt_1 = 1.4 min, rt_2 = 1.9 min.

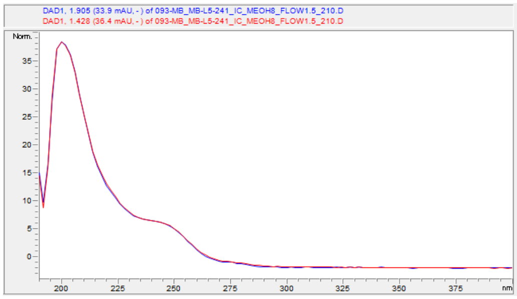

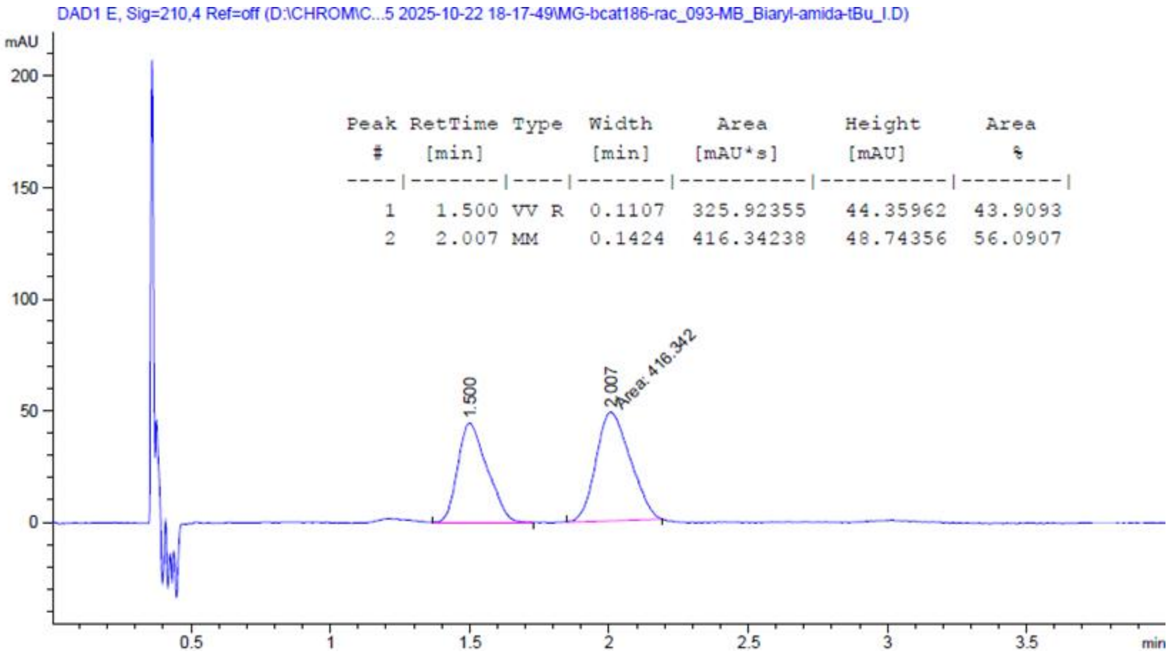

Peak #	RetTime [min]	Type	Width [min]	Area [mAU*s]	Height [mAU]	Area %
1	1.500	VV R	0.1107	325.92355	44.35962	43.9093
2	2.007	MM	0.1424	416.34238	48.74356	56.0907

Racemic **15a** was obtained by using a mixture of (*R*,*R*)- and (*S*,*S*)-**C6**.

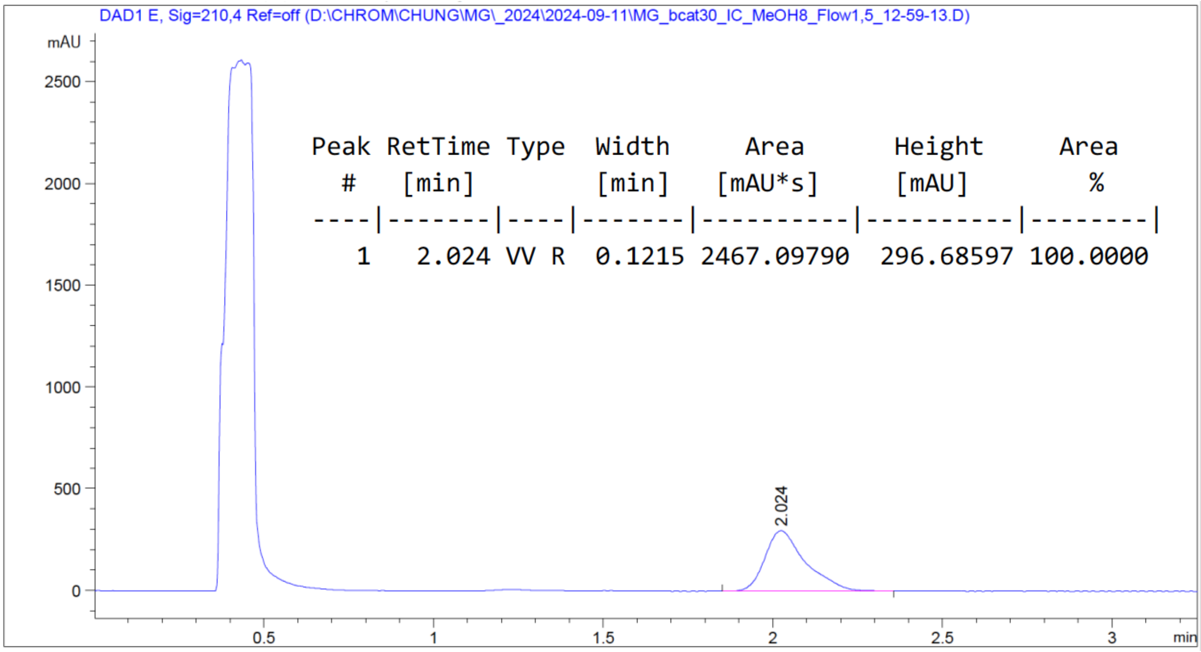

Peak #	RetTime [min]	Type	Width [min]	Area [mAU*s]	Height [mAU]	Area %
1	2.024	VV R	0.1215	2467.09790	296.68597	100.0000

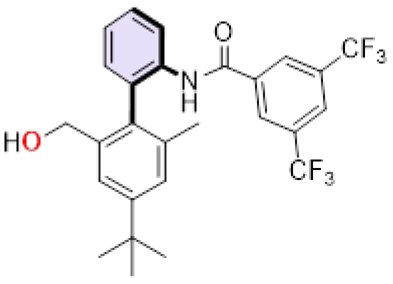

16a

SFC Analysis: Chiralpak IB, CO_2:MeOH (3%), 20ºC, flow rate = 1.5 mL/min, λ = 210 nm. rt_1 = 1.3 min, rt_2 = 2.5 min.

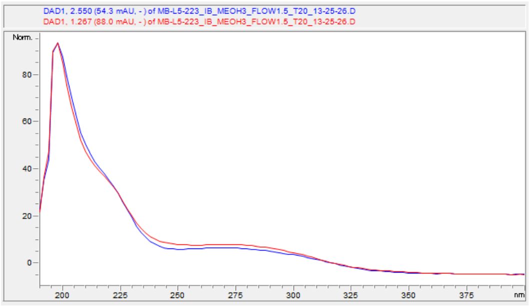

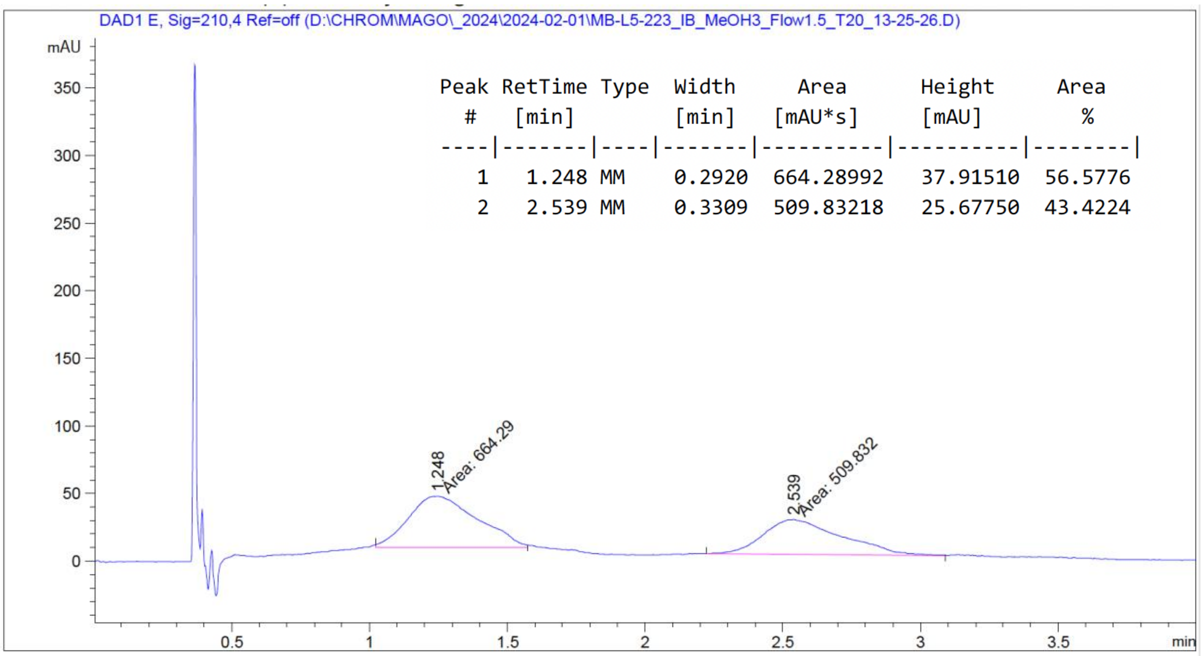

Peak #	RetTime [min]	Type	Width [min]	Area [mAU*s]	Height [mAU]	Area %
1	1.248	MM	0.2920	664.28992	37.91510	56.5776
2	2.539	MM	0.3309	509.83218	25.67750	43.4224

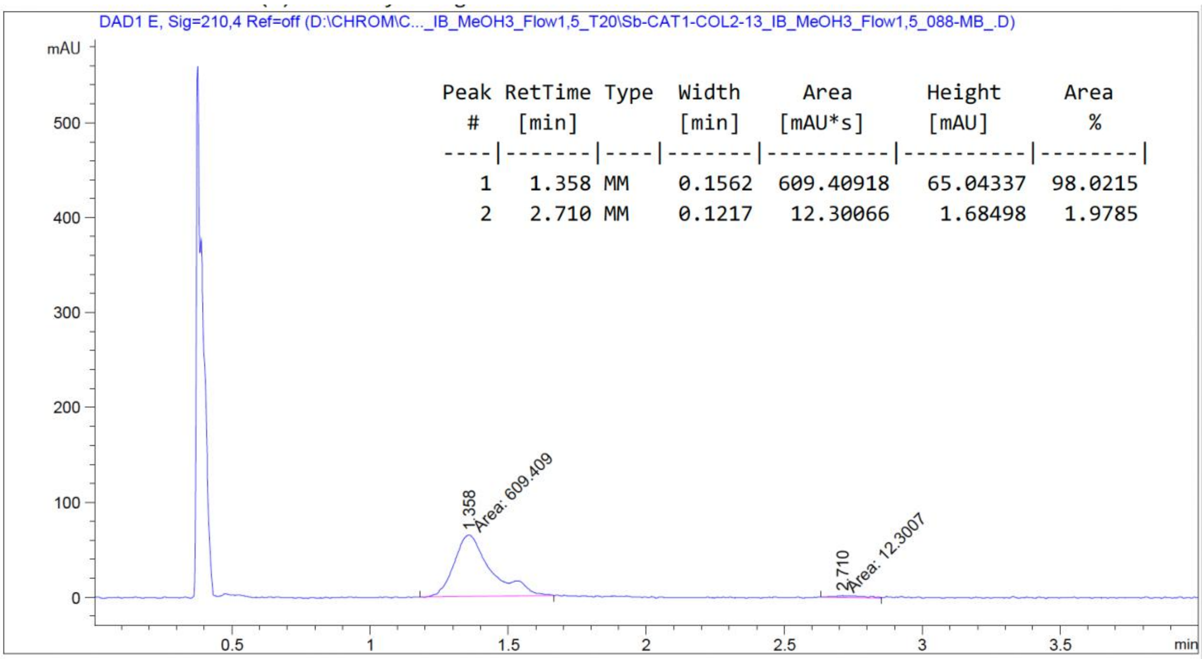

Peak #	RetTime [min]	Type	Width [min]	Area [mAU*s]	Height [mAU]	Area %
1	1.358	MM	0.1562	609.40918	65.04337	98.0215
2	2.710	MM	0.1217	12.30066	1.68498	1.9785

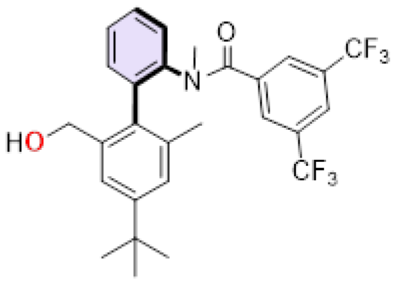

17a

SFC Analysis: Chiralpak IC, CO_2:2-propanol (5%), 35ºC, flow rate = 1.5 mL/min, λ = 210 nm. rt_1 = 1.7 min, rt_2 = 2.9 min.

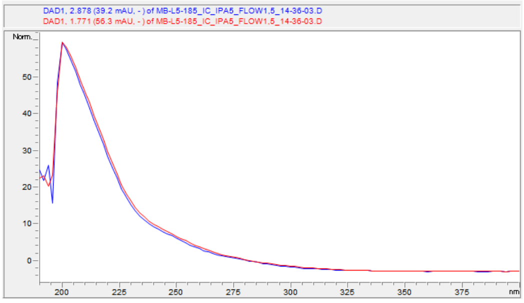

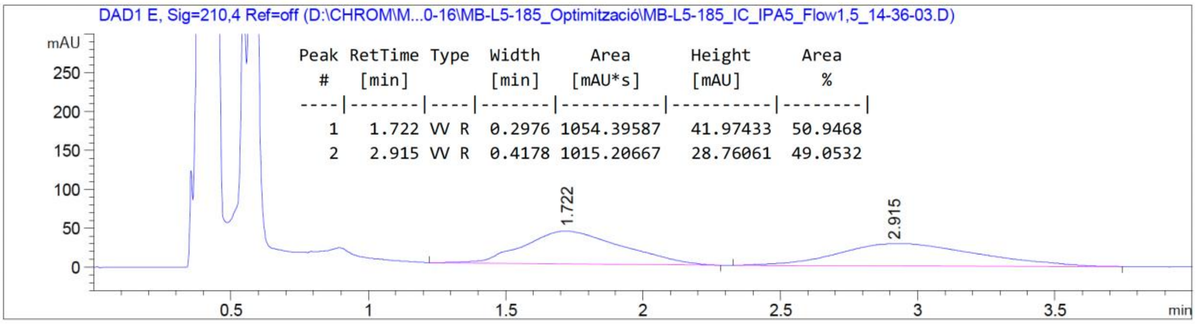

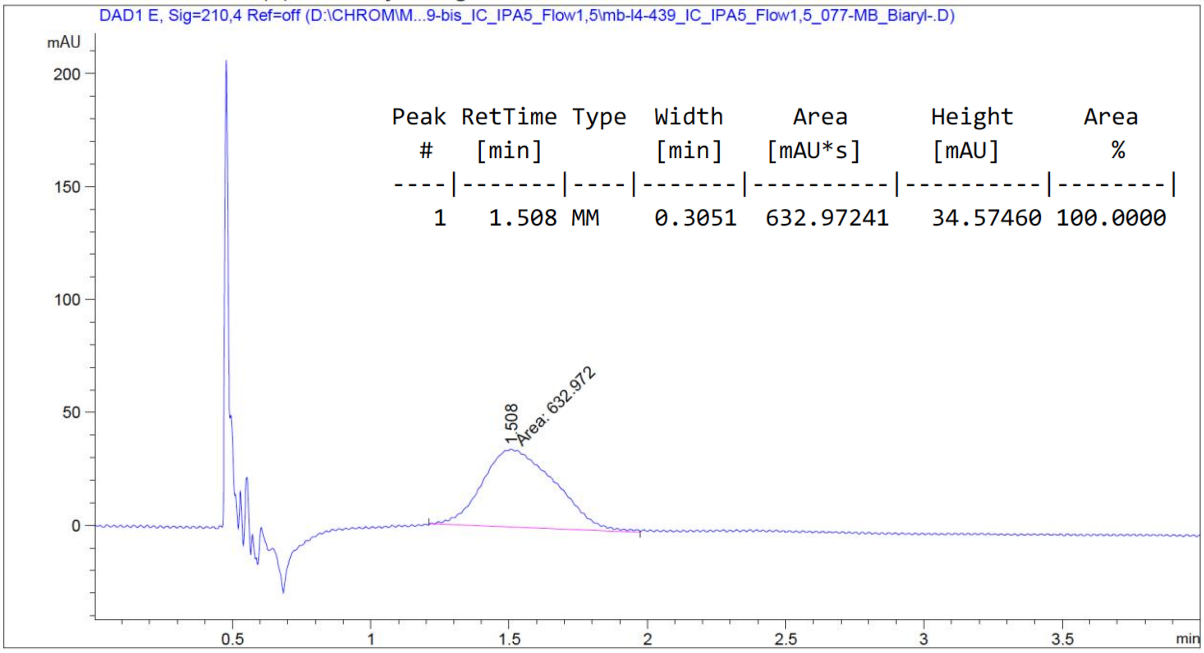

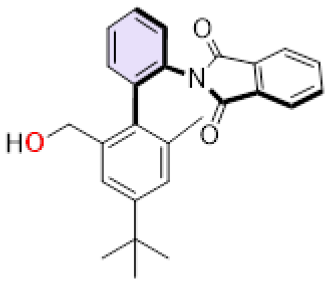

18a

SFC Analysis: Chiralpak IB, CO_2:EtOH (20%), 35ºC, flow rate = 1.2 mL/min, λ = 220 nm. rt_1 = 1.5 min, rt_2 = 1.9 min.

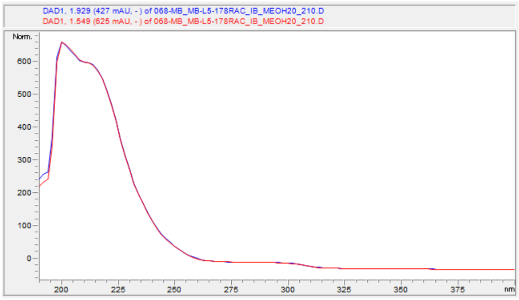

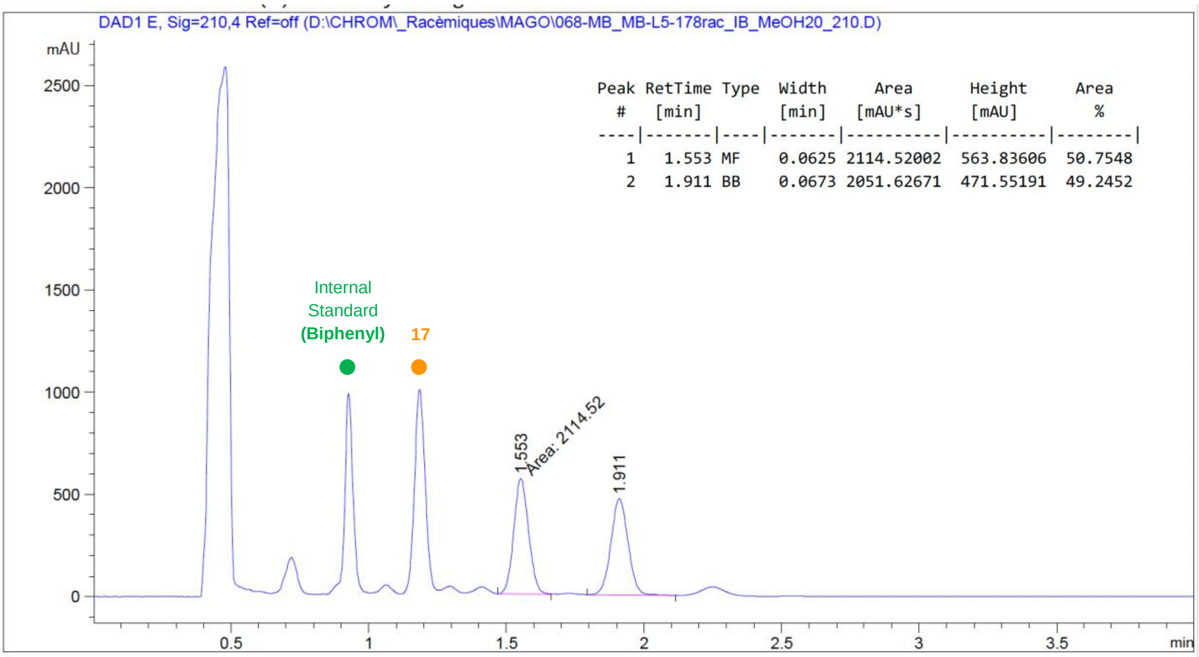

Peak #	RetTime [min]	Type	Width [min]	Area [mAU*s]	Height [mAU]	Area %
1	1.553	MF	0.0625	2114.52002	563.83606	50.7548
2	1.911	BB	0.0673	2051.62671	471.55191	49.2452

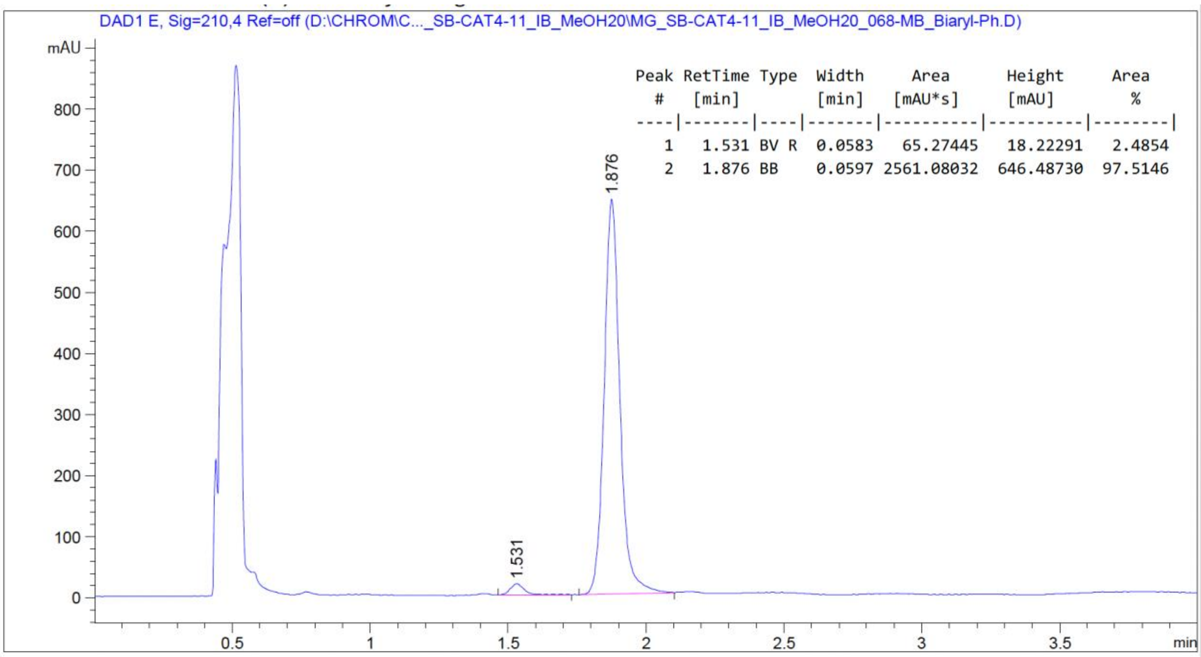

Peak #	RetTime [min]	Type	Width [min]	Area [mAU*s]	Height [mAU]	Area %
1	1.531	BV R	0.0583	65.27445	18.22291	2.4854
2	1.876	BB	0.0597	2561.08032	646.48730	97.5146

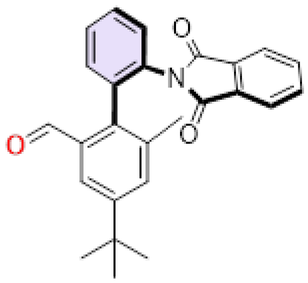

18b

SFC Analysis: Chiralpak IB, CO_2:MeOH (20%), 35ºC, flow rate = 1.2 mL/min, λ = 210 nm. rt_1 = 1.2 min, rt_2 = 1.4 min.

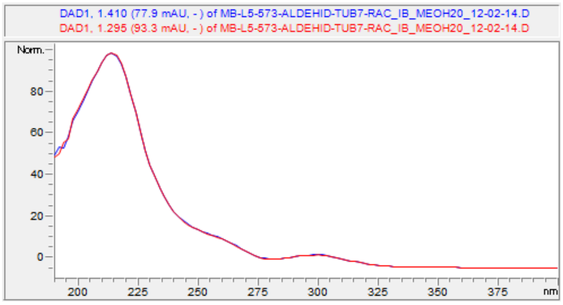

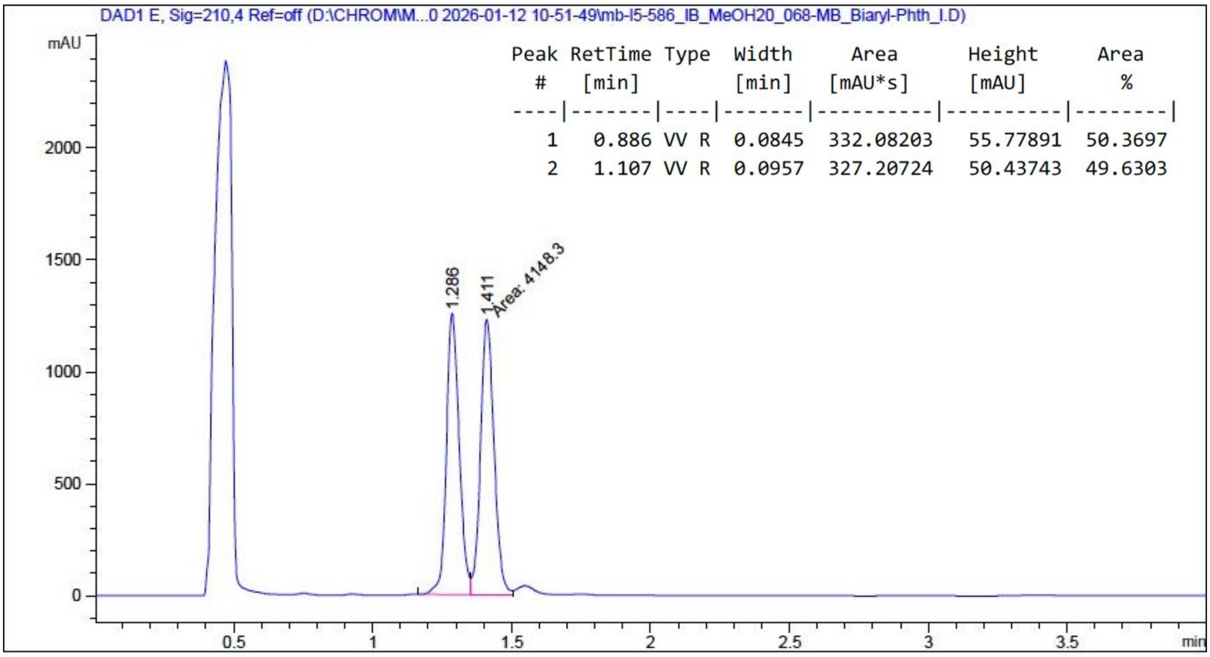

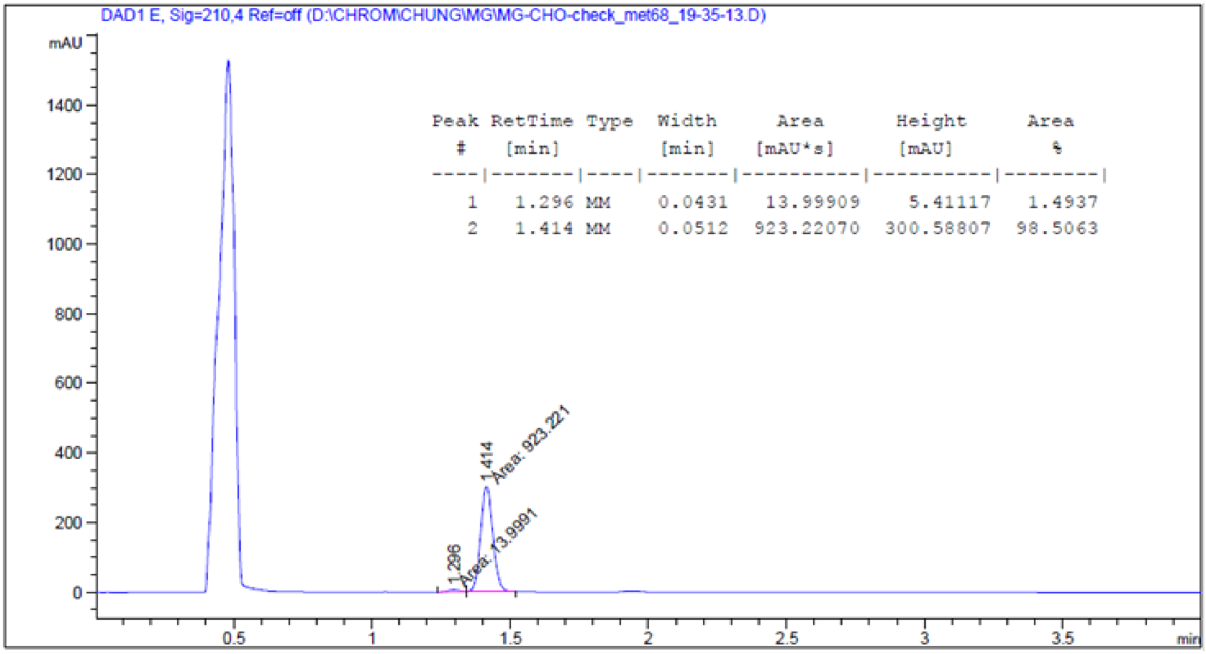

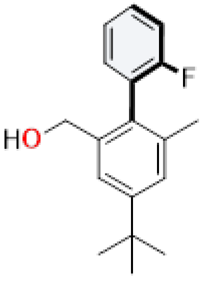

19a

SFC Analysis: Chiralpak IG, CO_2:EtOH (5%), 35ºC, flow rate = 1 mL/min, λ = 210 nm. rt_1 = 1.8 min, rt_2 = 2.1 min.

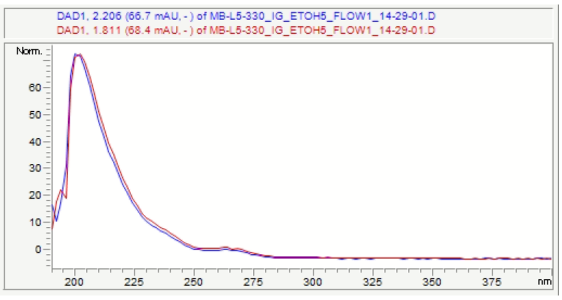

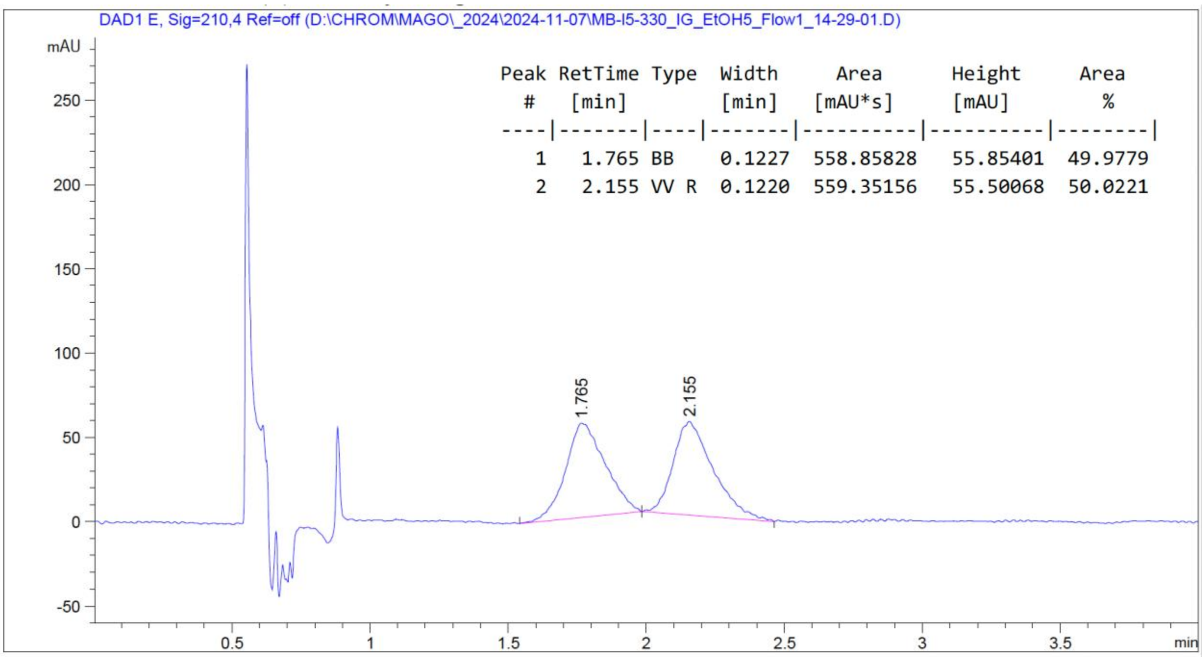

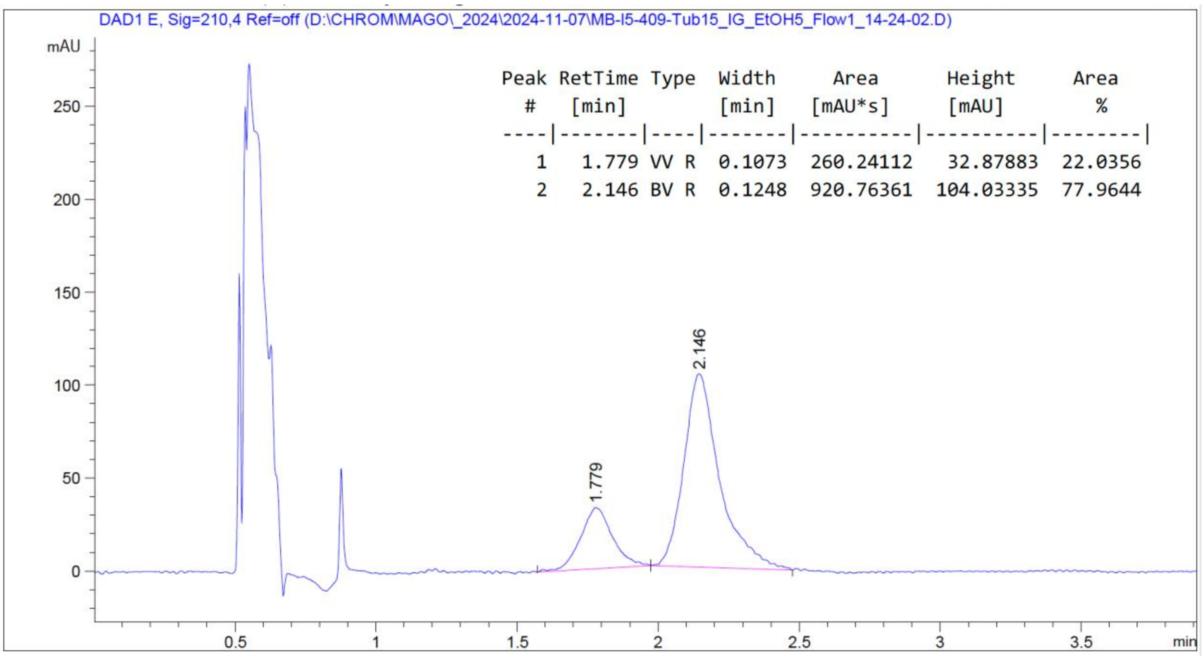

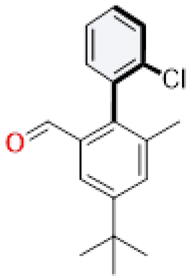

20b

SFC Analysis: Chiralpak IA, CO_2:2-propanol (5%), 35ºC, flow rate = 1.2 mL/min, λ = 210 nm. rt_1 = 1.1 min, rt_2 = 1.4 min.

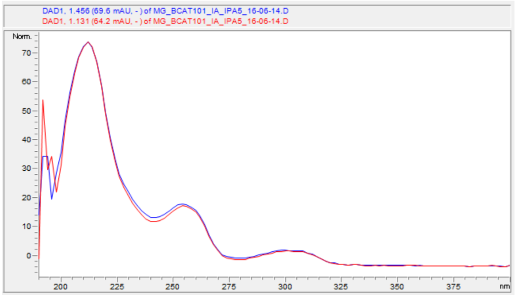

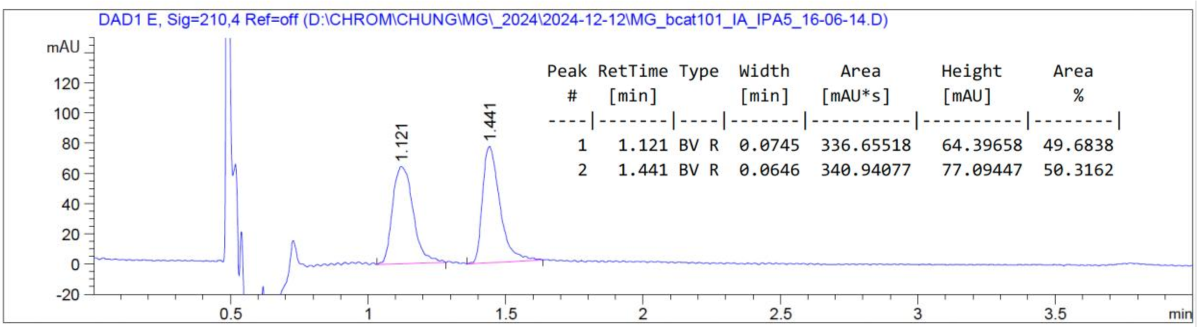

Peak #	RetTime [min]	Type	Width [min]	Area [mAU*s]	Height [mAU]	Area %
1	1.121	BV R	0.0745	336.65518	64.39658	49.6838
2	1.441	BV R	0.0646	340.94077	77.09447	50.3162

(*R*,*R*)-**C2** catalysts

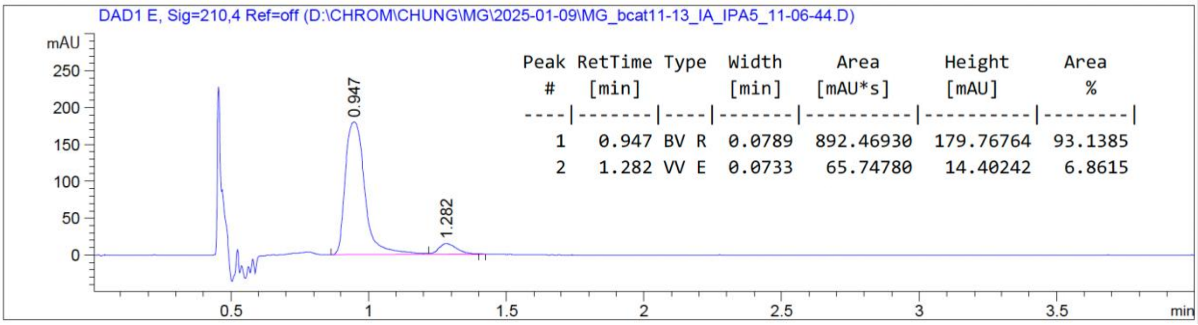

Peak #	RetTime [min]	Type	Width [min]	Area [mAU*s]	Height [mAU]	Area %
1	0.947	BV R	0.0789	892.46930	179.76764	93.1385
2	1.282	VV E	0.0733	65.74780	14.40242	6.8615

(*R*,*R*)-**C6** catalysts

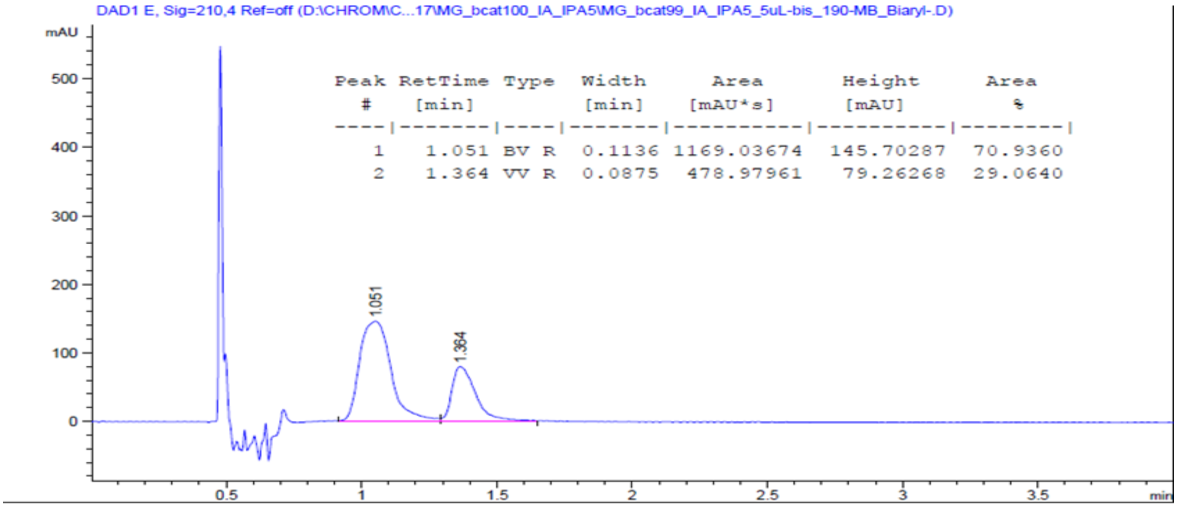

Peak #	RetTime [min]	Type	Width [min]	Area [mAU*s]	Height [mAU]	Area %
1	1.051	BV R	0.1136	1169.03674	145.70287	70.9360
2	1.364	VV R	0.0875	478.97961	79.26268	29.0640

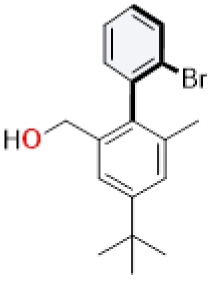

21a

SFC Analysis: Chiralpak IG, CO_2:2-propanol (5%), 35ºC, flow rate = 1.5 mL/min, λ = 210 nm. rt_1 = 2.1 min, rt_2 = 2.9 min.

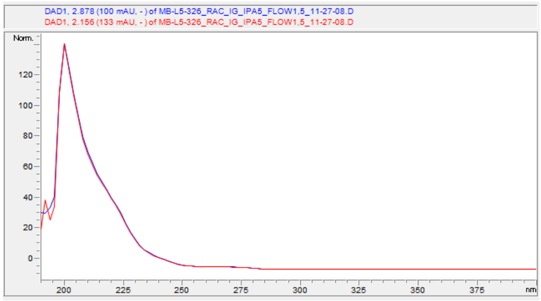

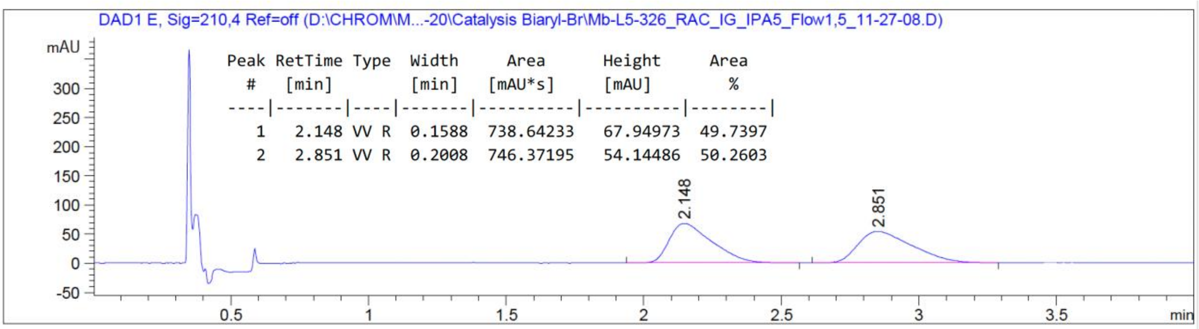

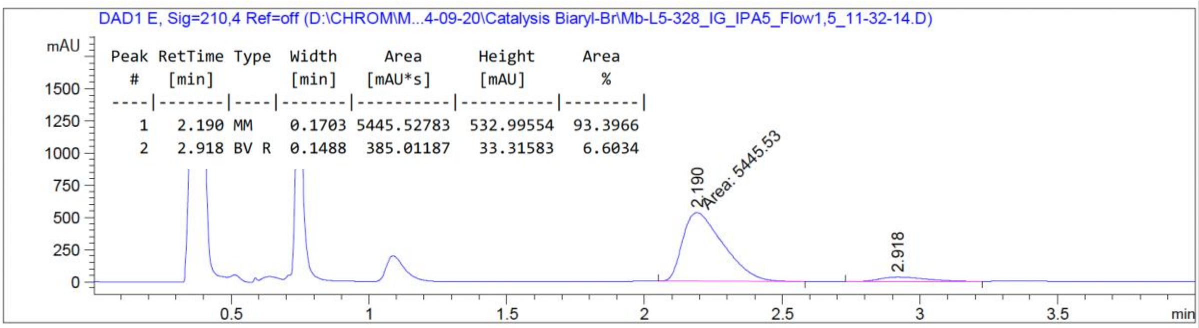

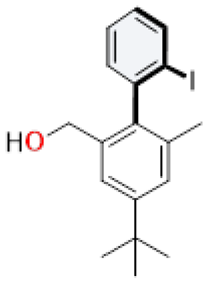

22a

SFC Analysis: Chiralpak IG, CO_2:2-propanol (15 %), 35ºC, flow rate = 1.2 mL/min, λ = 210 nm. rt_1 = 1.2 min, rt_2 = 1.4 min.

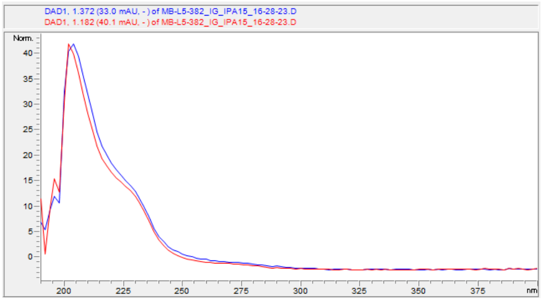

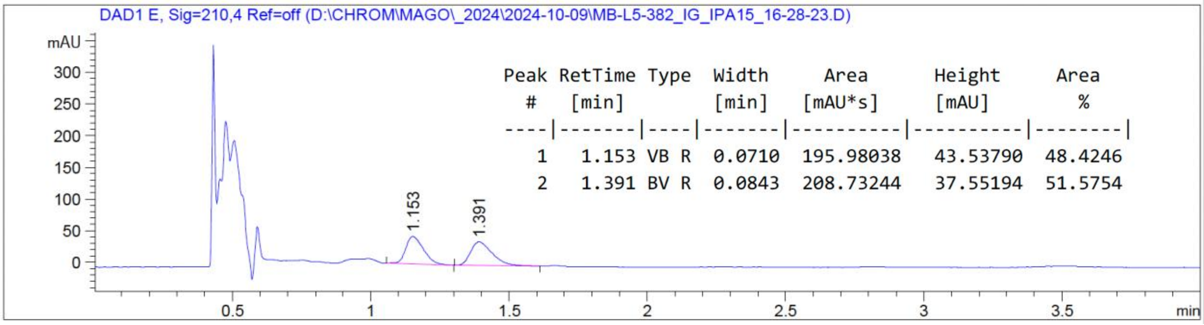

Peak #	RetTime [min]	Type	Width [min]	Area [mAU*s]	Height [mAU]	Area %
1	1.153	VB R	0.0710	195.98038	43.53790	48.4246
2	1.391	BV R	0.0843	208.73244	37.55194	51.5754

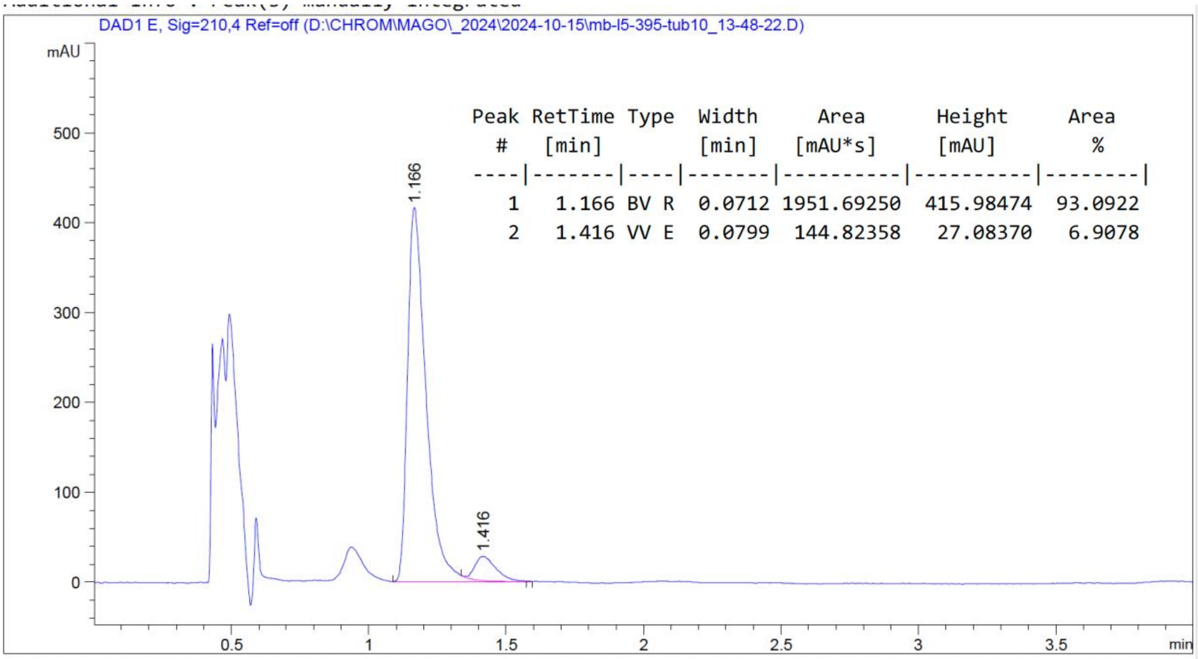

Peak #	RetTime [min]	Type	Width [min]	Area [mAU*s]	Height [mAU]	Area %
1	1.166	BV R	0.0712	1951.69250	415.98474	93.0922
2	1.416	VV E	0.0799	144.82358	27.08370	6.9078

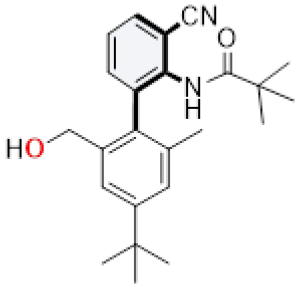

23a

SFC Analysis: Chiralpak X, CO_2:2-propanol (20%), 35ºC, flow rate = 2 mL/min, λ = 210 nm. rt_1 = 1.6 min, rt_2 = 2.2 min.

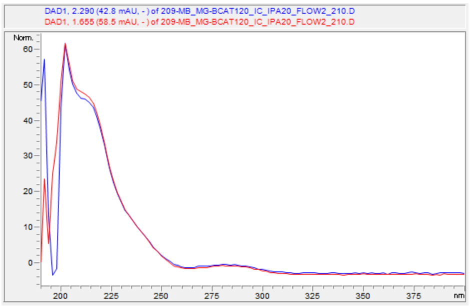

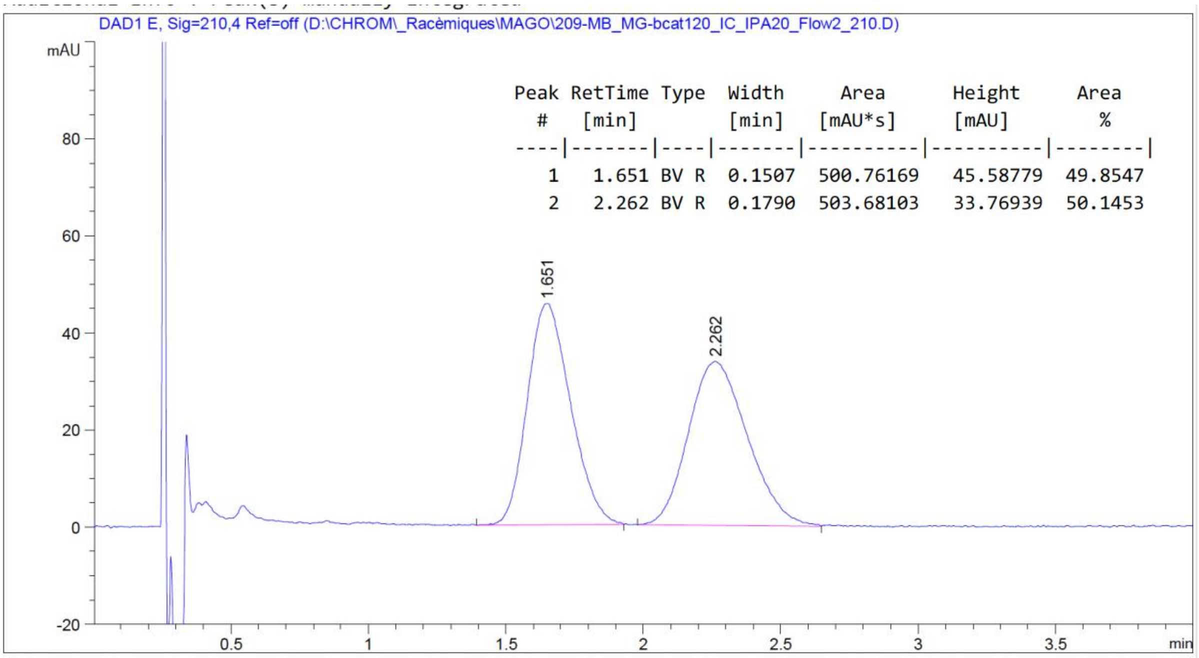

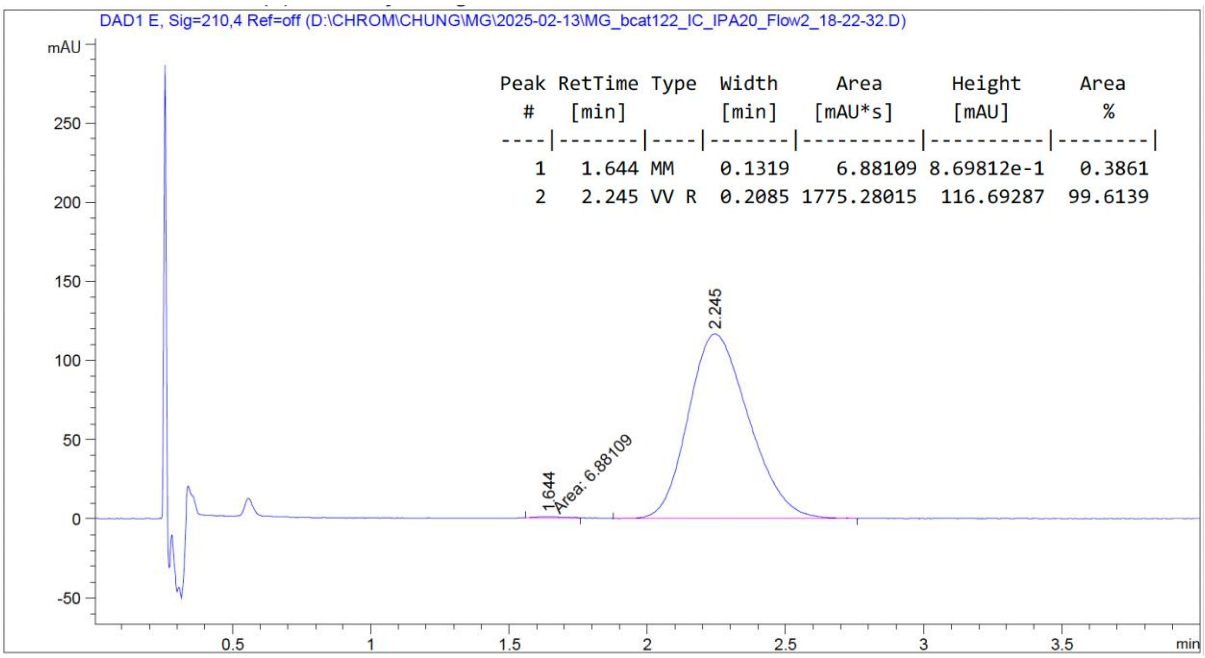

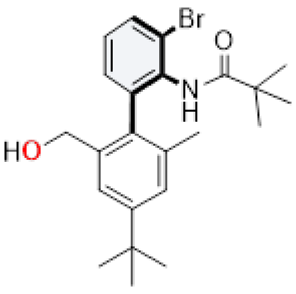

24a

SFC Analysis: Chiralpak IC, CO_2:2-propanol (15%), 35ºC, flow rate = 1.2 mL/min, λ = 210 nm. rt_1 = 3.2 min, rt_2 = 4.0 min.

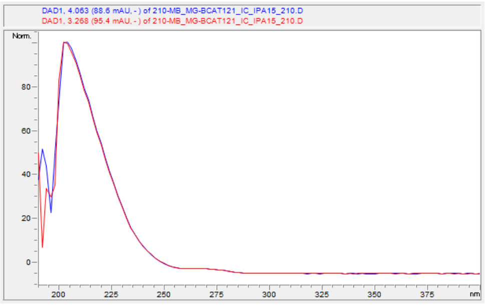

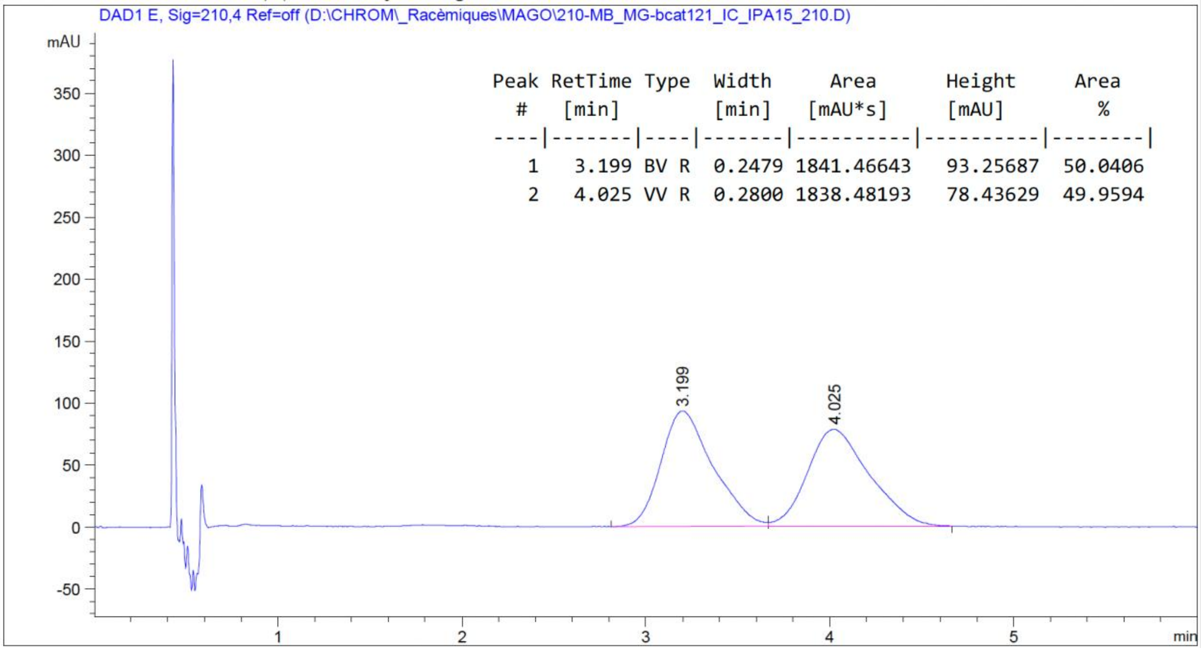

Peak #	RetTime [min]	Type	Width [min]	Area [mAU*s]	Height [mAU]	Area %
1	3.199	BV R	0.2479	1841.46643	93.25687	50.0406
2	4.025	VV R	0.2800	1838.48193	78.43629	49.9594

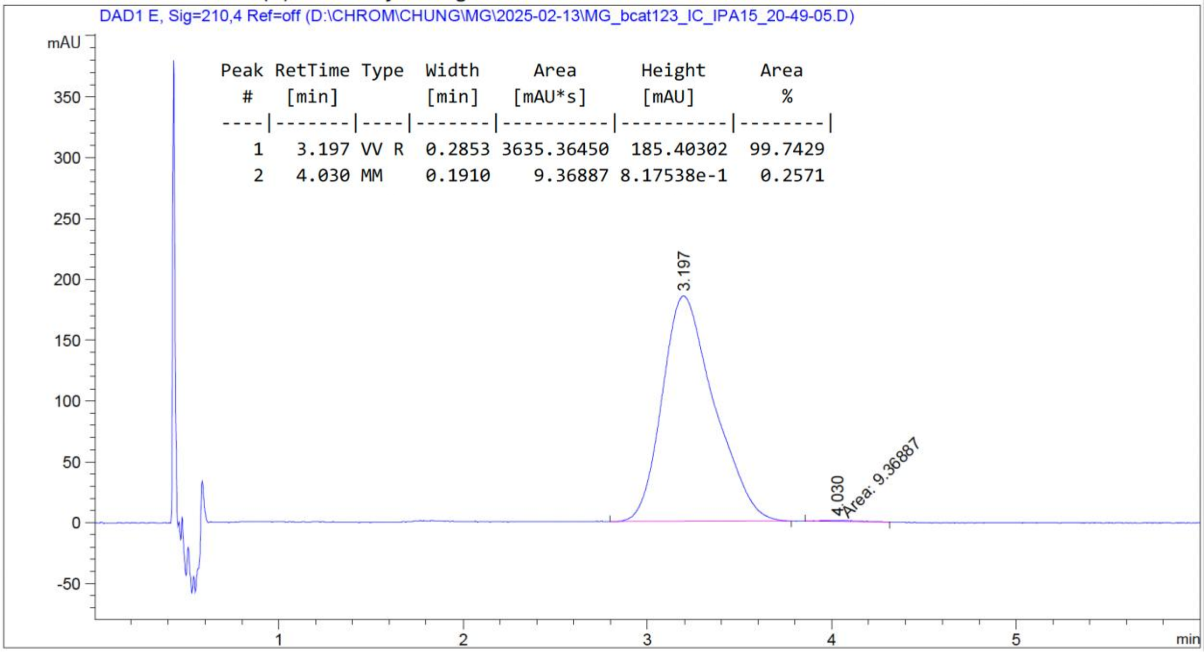

Peak #	RetTime [min]	Type	Width [min]	Area [mAU*s]	Height [mAU]	Area %
1	3.197	VV R	0.2853	3635.36450	185.40302	99.7429
2	4.030	MM	0.1910	9.36887	8.17538e-1	0.2571

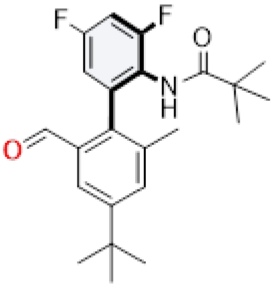

25b

SFC Analysis: Chiralpak IC, CO_2:10%, 35ºC, flow rate = 1.2 mL/min, λ = 210 nm. rt_1 = 0.8 min, rt_2 = 1.3 min.

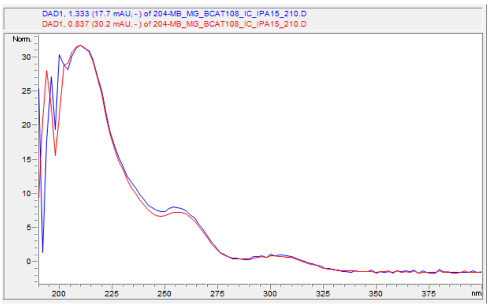

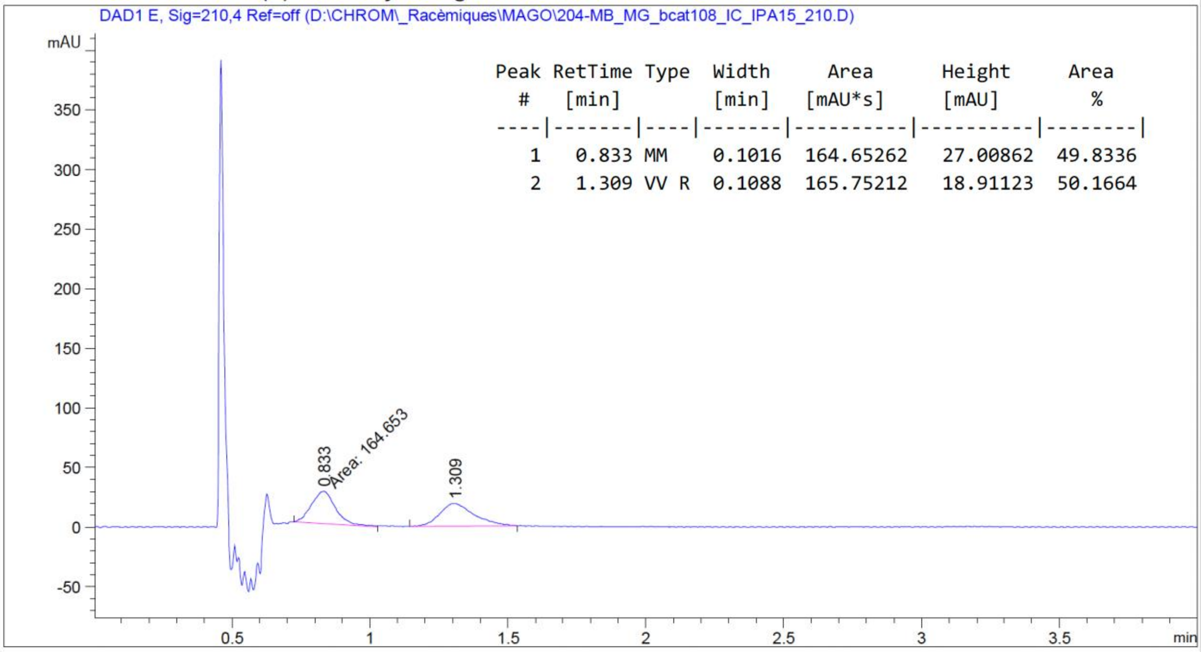

Peak #	RetTime [min]	Type	Width [min]	Area [mAU*s]	Height [mAU]	Area %
1	0.833	MM	0.1016	164.65262	27.00862	49.8336
2	1.309	VV R	0.1088	165.75212	18.91123	50.1664

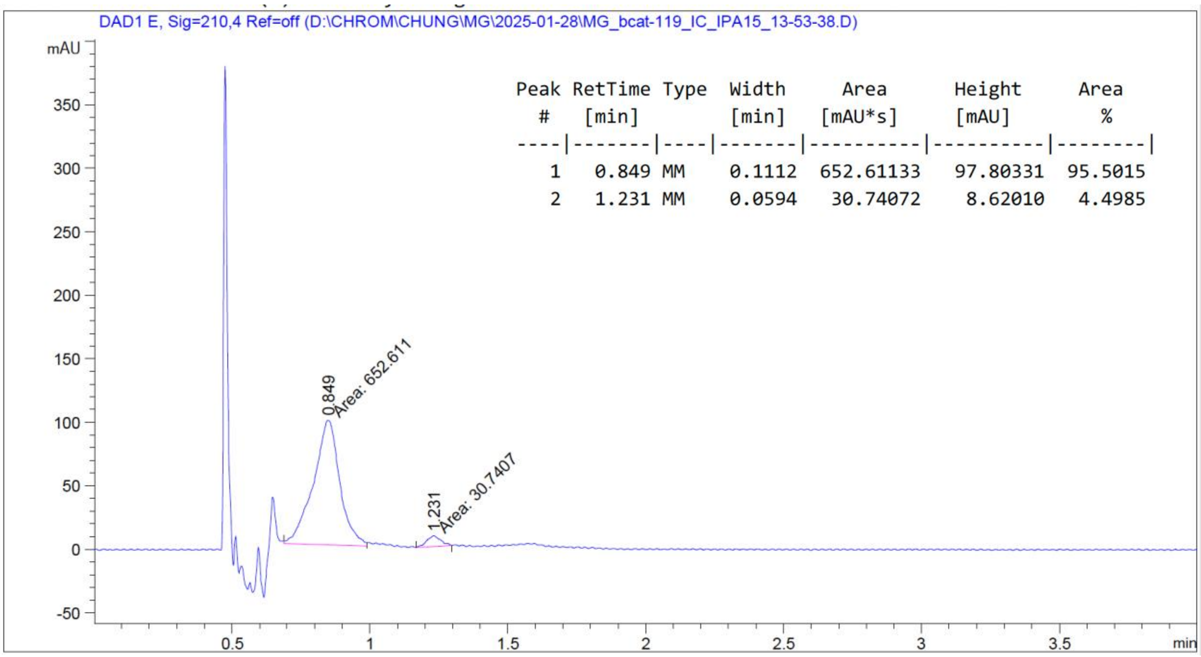

Peak #	RetTime [min]	Type	Width [min]	Area [mAU*s]	Height [mAU]	Area %
1	0.849	MM	0.1112	652.61133	97.80331	95.5015
2	1.231	MM	0.0594	30.74072	8.62010	4.4985

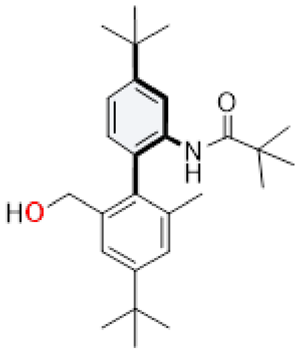

26a

SFC Analysis: Chiralpak IC, CO_2:2-propanol (15%), 35ºC, flow rate = 1.5 mL/min, λ = 210 nm. rt_1 = 0.9 min, rt_2 = 1.6 min.

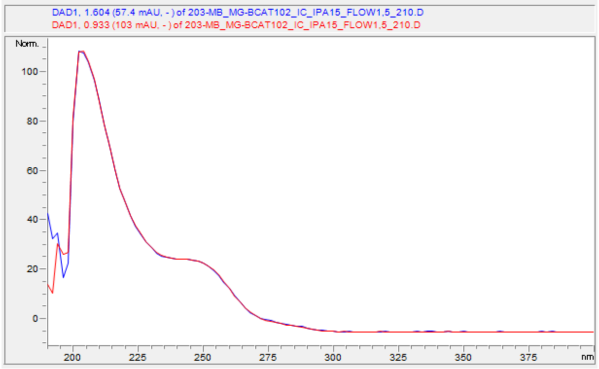

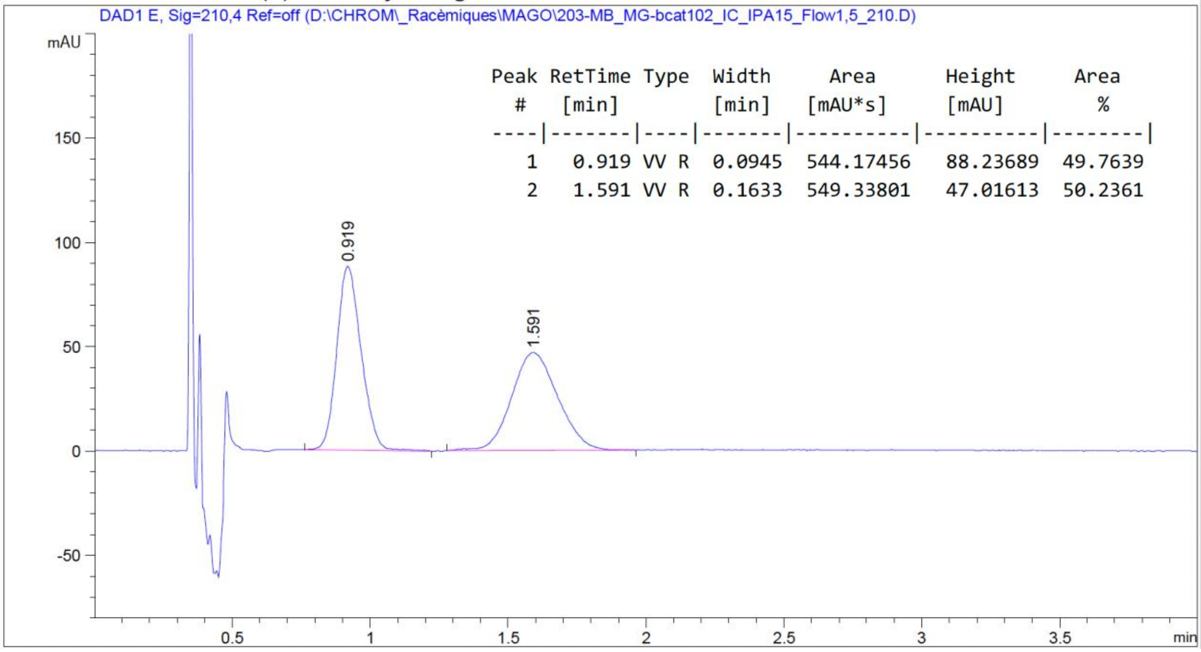

Peak #	RetTime [min]	Type	Width [min]	Area [mAU*s]	Height [mAU]	Area %
1	0.919	VV R	0.0945	544.17456	88.23689	49.7639
2	1.591	VV R	0.1633	549.33801	47.01613	50.2361

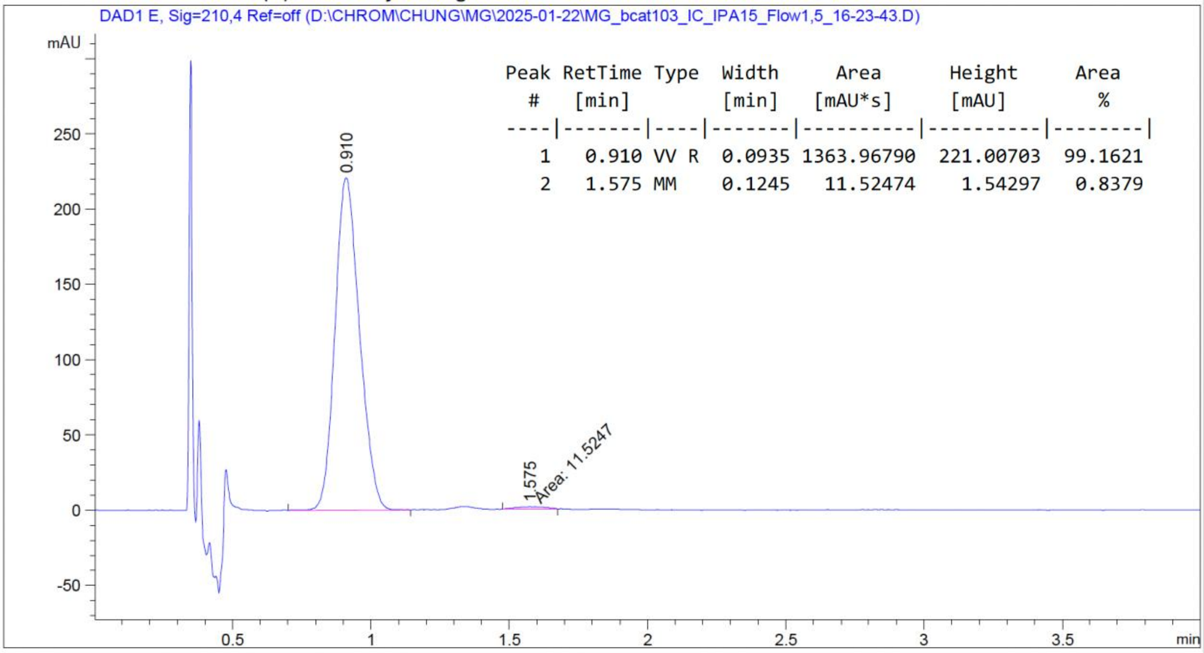

Peak #	RetTime [min]	Type	Width [min]	Area [mAU*s]	Height [mAU]	Area %
1	0.910	VV R	0.0935	1363.96790	221.00703	99.1621
2	1.575	MM	0.1245	11.52474	1.54297	0.8379

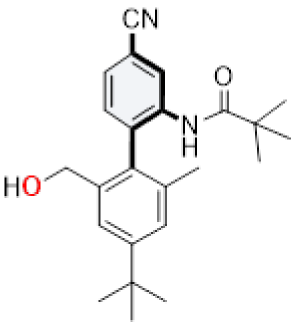

27a

SFC Analysis: Chiralpak IC, CO_2:2-propanol (25%), 35ºC, flow rate = 1.2 mL/min, λ = 220 nm. rt_1 = 0.9 min, rt_2 = 1.1 min.

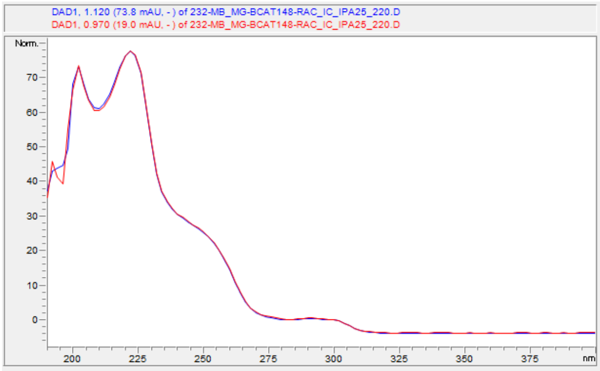

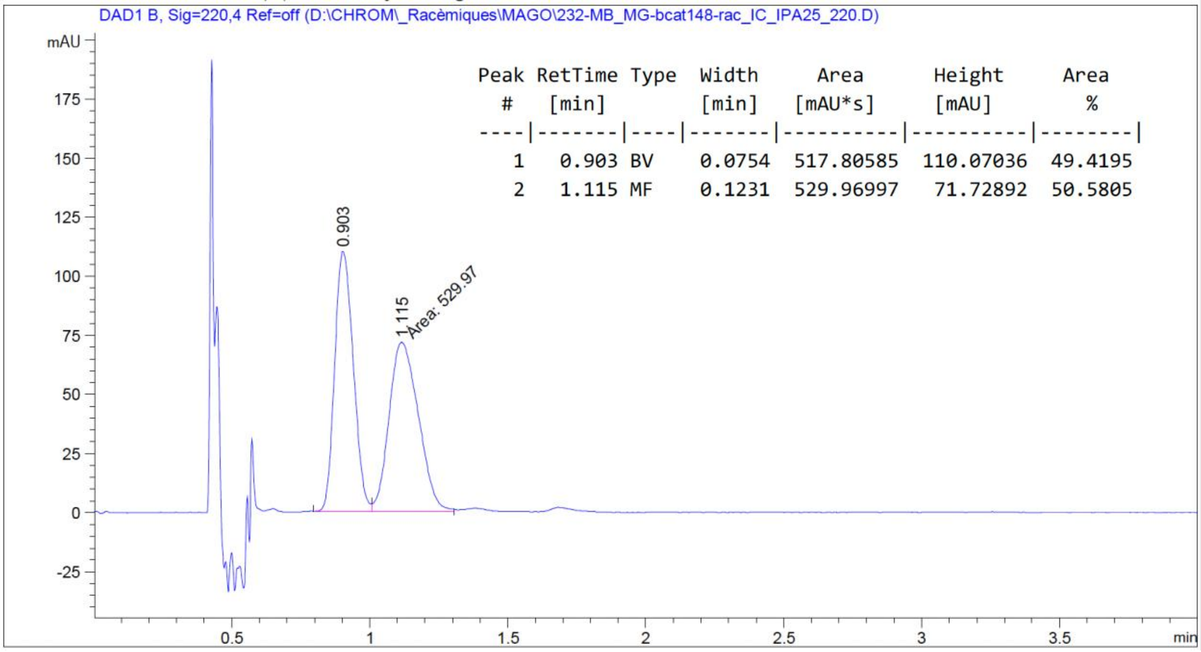

Peak #	RetTime [min]	Type	Width [min]	Area [mAU*s]	Height [mAU]	Area %
1	0.903	BV	0.0754	517.80585	110.07036	49.4195
2	1.115	MF	0.1231	529.96997	71.72892	50.5805

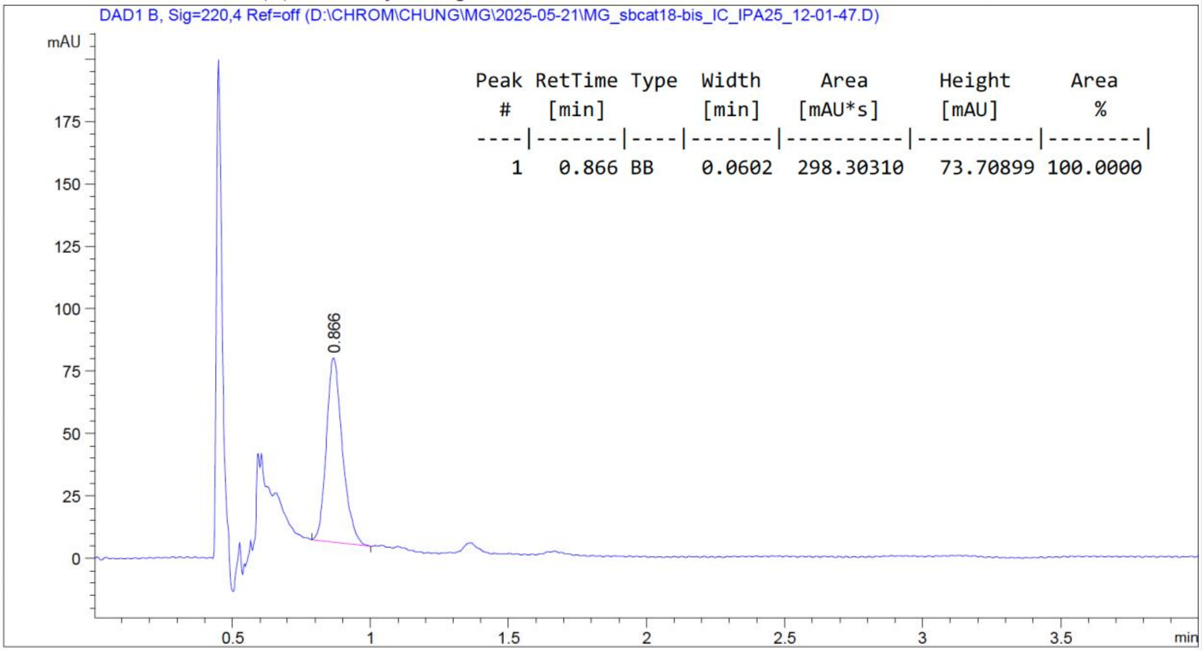

Peak #	RetTime [min]	Type	Width [min]	Area [mAU*s]	Height [mAU]	Area %
1	0.866	BB	0.0602	298.30310	73.70899	100.0000

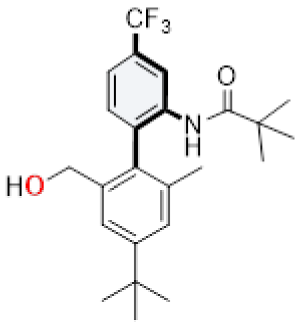

28a

SFC Analysis: Chiralpak IC, CO_2:MeOH (2%), 35ºC, flow rate = 1.5 mL/min, λ = 210 nm. rt_1 = 2.2 min, rt_2 = 3.3 min.

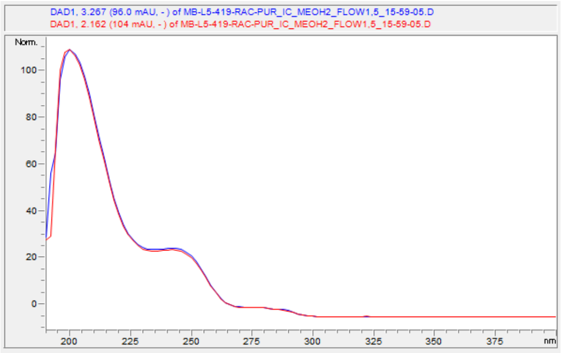

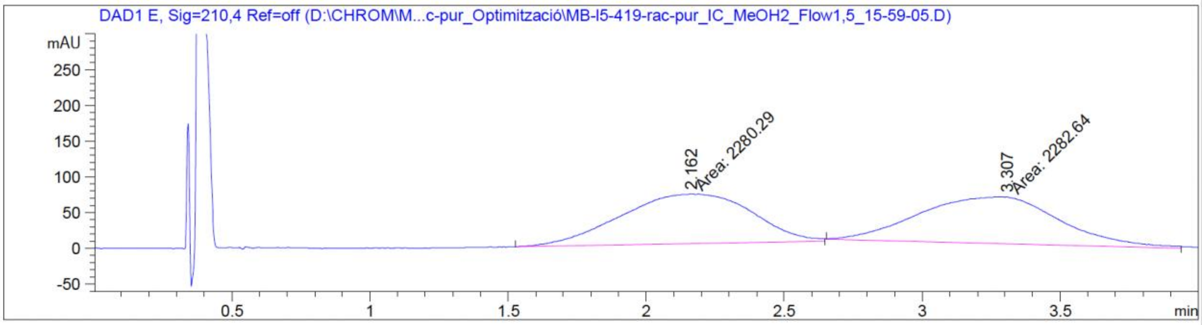

Peak #	RetTime [min]	Type	Width [min]	Area [mAU*s]	Height [mAU]	Area %
1	2.162	MM	0.5501	2280.29272	69.09252	49.9742
2	3.307	MM	0.5846	2282.64429	65.08212	50.0258

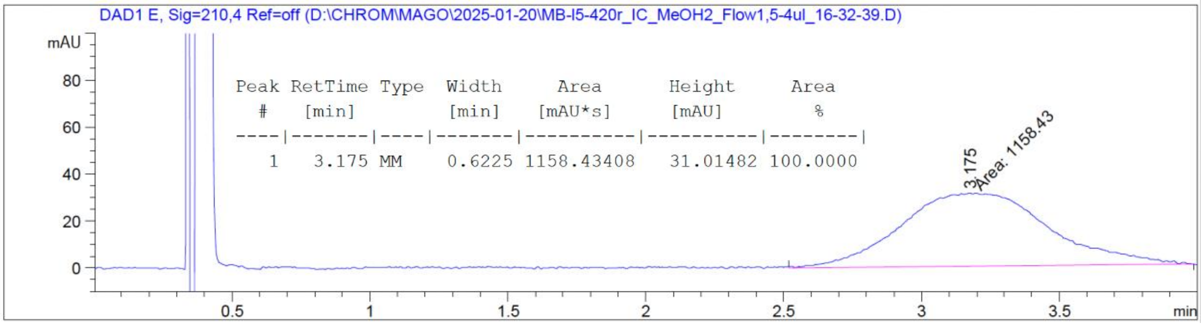

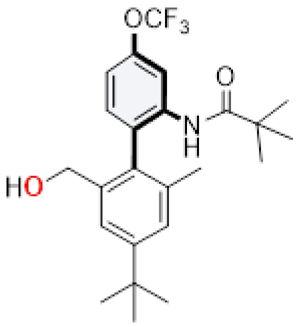

29a

SFC Analysis: Chiralpak IB, CO_2:2-isopropaonl (3%), 35ºC, flow rate = 1.5 mL/min, λ = 210 nm. rt_1 = 1.5 min, rt_2 = 2.4 min.

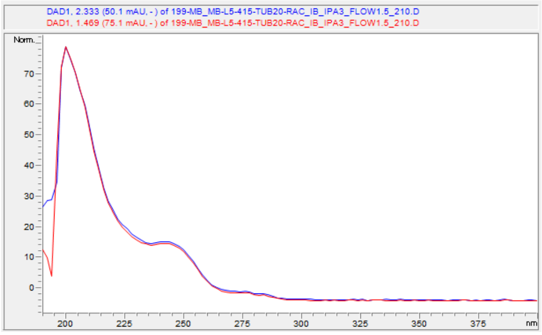

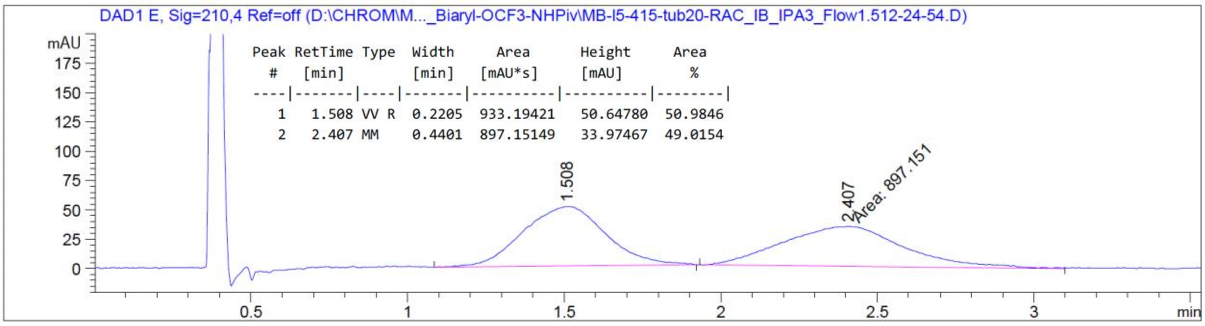

Peak #	RetTime [min]	Type	Width [min]	Area [mAU*s]	Height [mAU]	Area %
1	1.508	VV R	0.2205	933.19421	50.64780	50.9846
2	2.407	MM	0.4401	897.15149	33.97467	49.0154

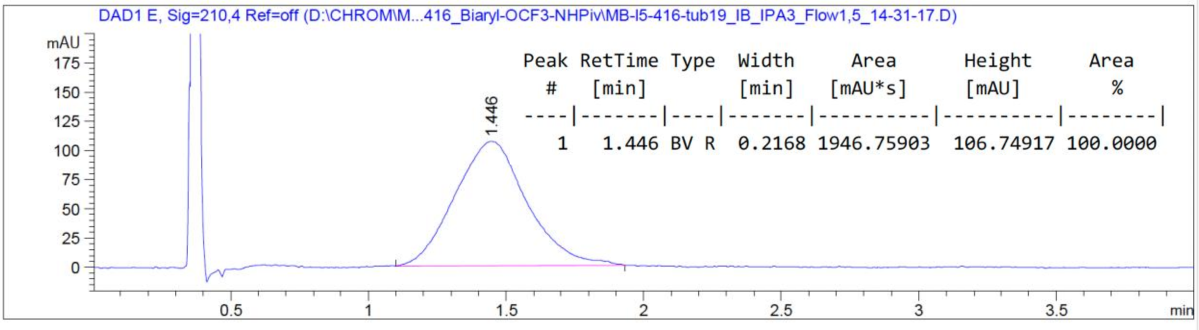

Peak #	RetTime [min]	Type	Width [min]	Area [mAU*s]	Height [mAU]	Area %
1	1.446	BV R	0.2168	1946.75903	106.74917	100.0000

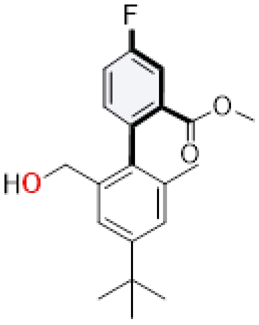

30a

SFC Analysis: Chiralpak iG, CO_2:IPA (15%), 35ºC, flow rate = 1.2 mL/min, λ = 210 nm. rt_1 = 1.6 min, rt_2 = 2.1 min.

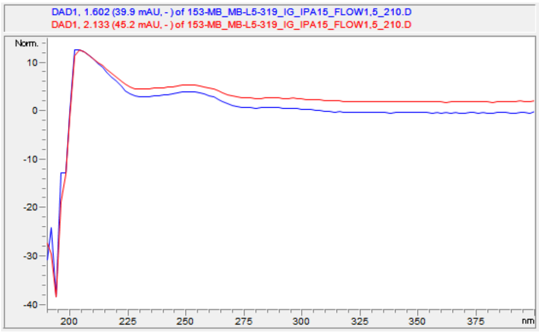

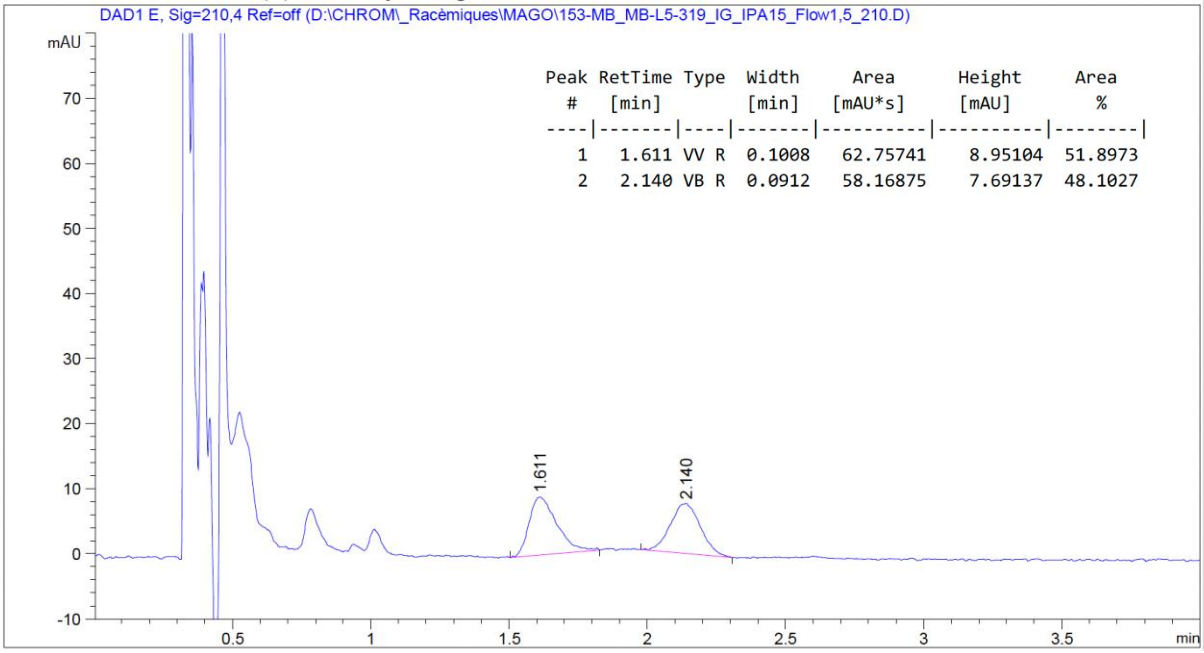

Peak #	RetTime [min]	Type	Width [min]	Area [mAU*s]	Height [mAU]	Area %
1	1.611	VV R	0.1008	62.75741	8.95104	51.8973
2	2.140	VB R	0.0912	58.16875	7.69137	48.1027

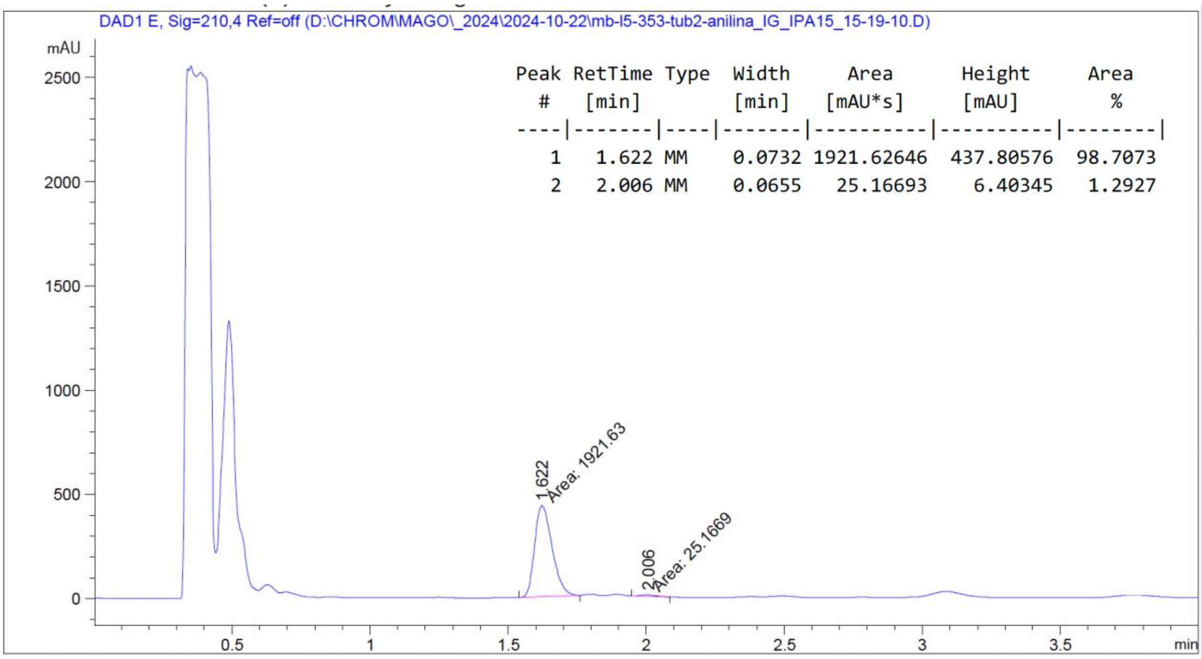

Peak #	RetTime [min]	Type	Width [min]	Area [mAU*s]	Height [mAU]	Area %
1	1.622	MM	0.0732	1921.62646	437.80576	98.7073
2	2.006	MM	0.0655	25.16693	6.40345	1.2927

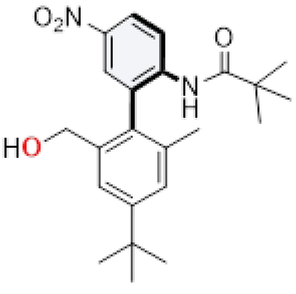

31a

SFC Analysis: Chiralpak IC, CO_2:2-propanol (20%), 35ºC, flow rate = 1.2 mL/min, λ = 210 nm. rt_1 = 1.1 min, rt_2 = 1.5 min.

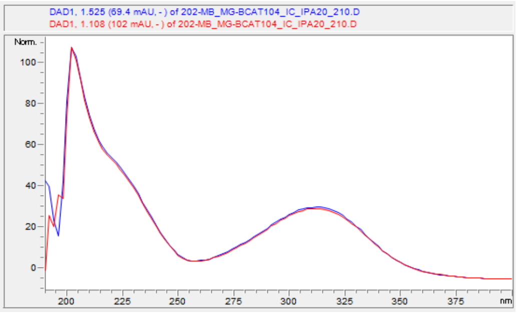

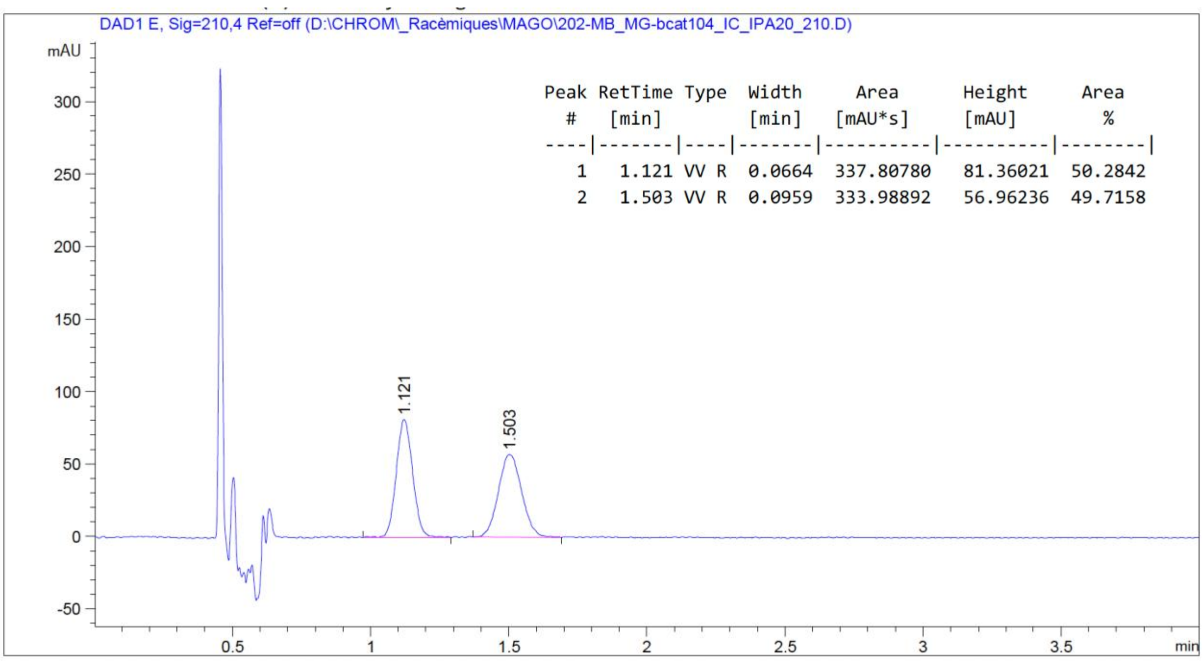

Peak #	RetTime [min]	Type	Width [min]	Area [mAU*s]	Height [mAU]	Area %
1	1.121	VV R	0.0664	337.80780	81.36021	50.2842
2	1.503	VV R	0.0959	333.98892	56.96236	49.7158

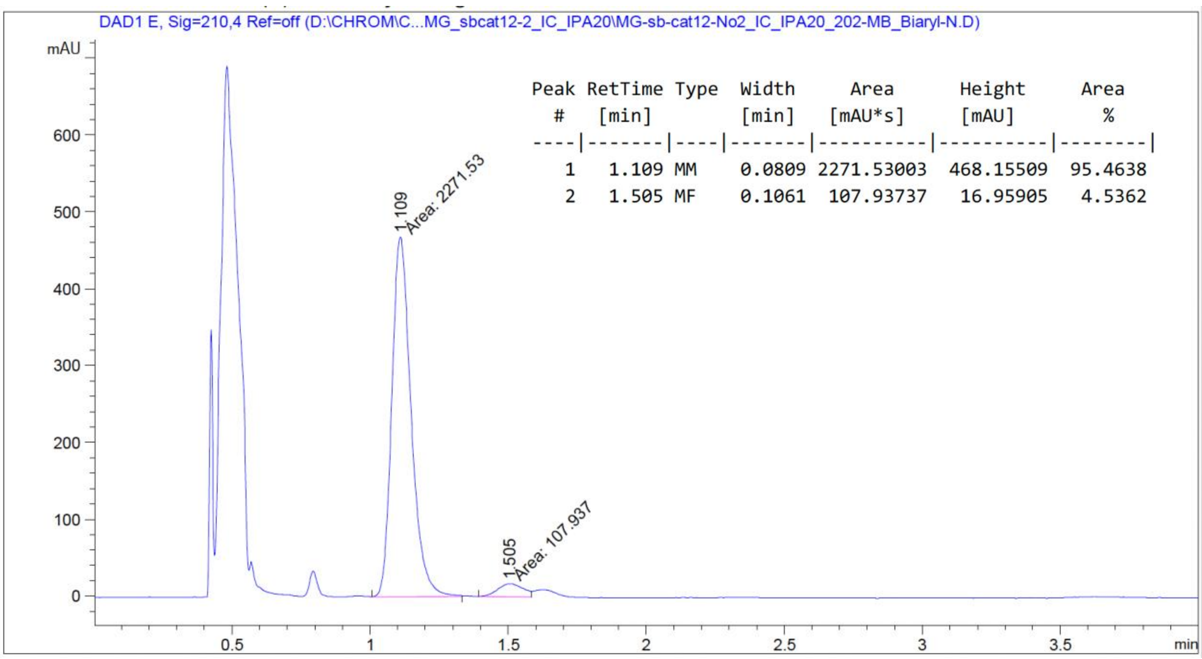

Peak #	RetTime [min]	Type	Width [min]	Area [mAU*s]	Height [mAU]	Area %
1	1.109	MM	0.0809	2271.53003	468.15509	95.4638
2	1.505	MF	0.1061	107.93737	16.95905	4.5362

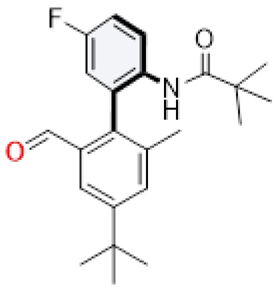

32b

SFC Analysis: Chiralpak IC, CO_2:2-propanol (15%), 35ºC, flow rate = 1.2 mL/min, λ = 210 nm. rt_1 = 1.0 min, rt_2 = 1.4 min.

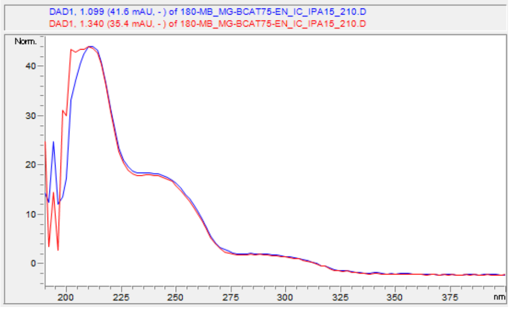

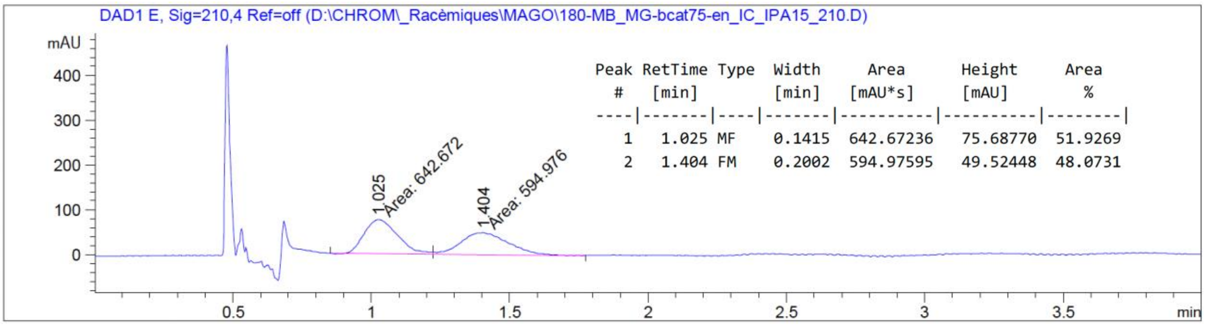

Peak #	RetTime [min]	Type	Width [min]	Area [mAU*s]	Height [mAU]	Area %
1	1.025	MF	0.1415	642.67236	75.68770	51.9269
2	1.404	FM	0.2002	594.97595	49.52448	48.0731

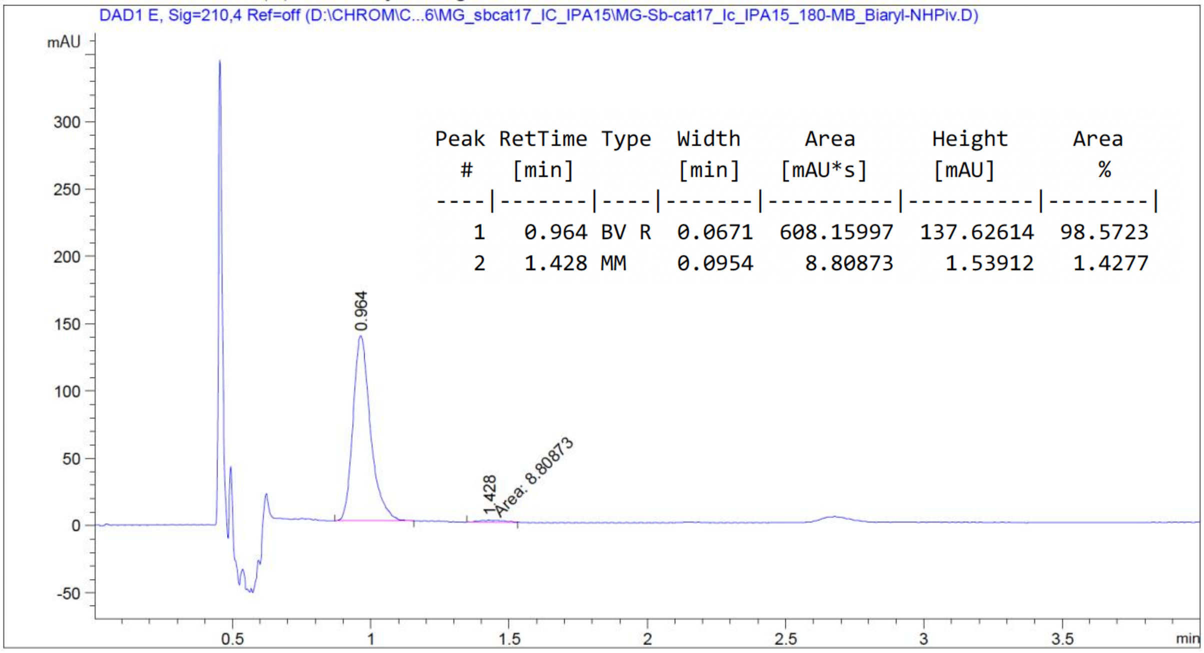

Peak #	RetTime [min]	Type	Width [min]	Area [mAU*s]	Height [mAU]	Area %
1	0.964	BV R	0.0671	608.15997	137.62614	98.5723
2	1.428	MM	0.0954	8.80873	1.53912	1.4277

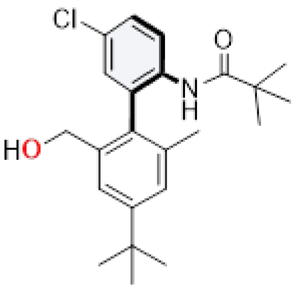

33a

SFC Analysis: Chiralpak IG, CO_2:EtOH (5%), 35ºC, flow rate = 1.2 mL/min, λ = 210 nm. rt_1 = 2.9 min, rt_2 = 3.6 min.

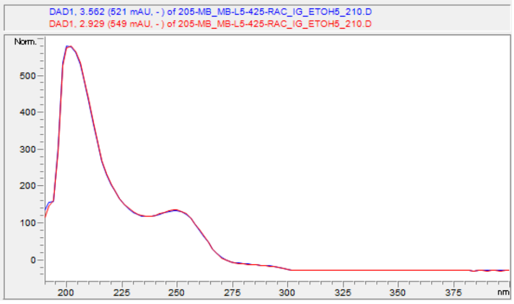

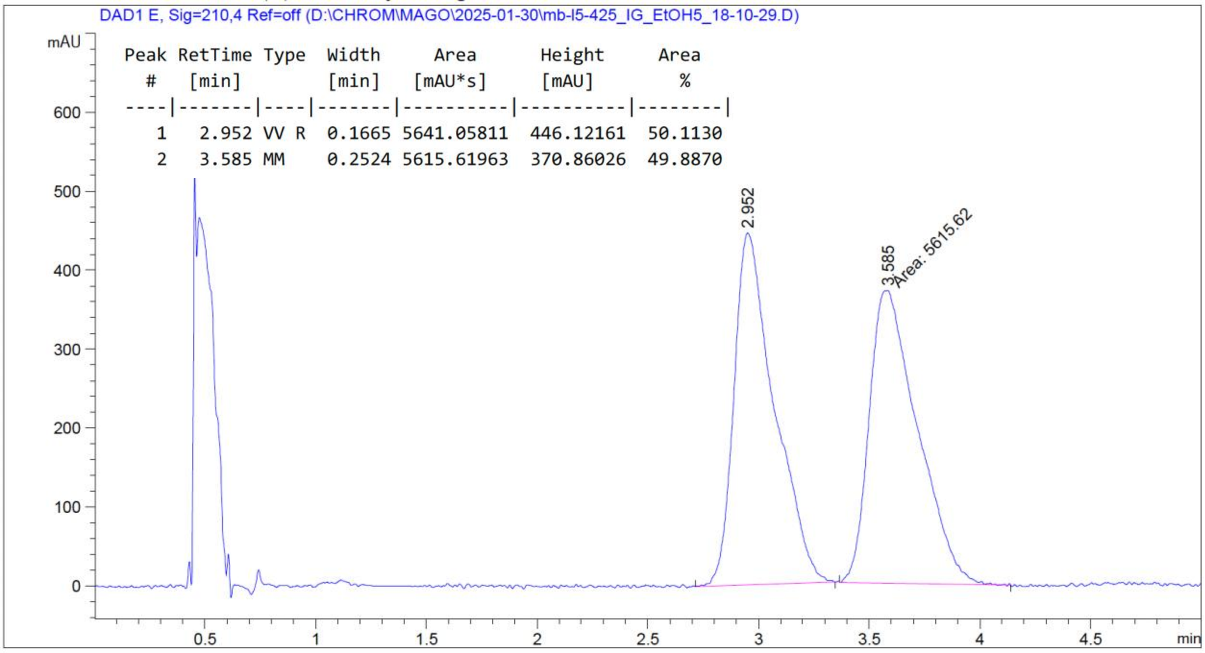

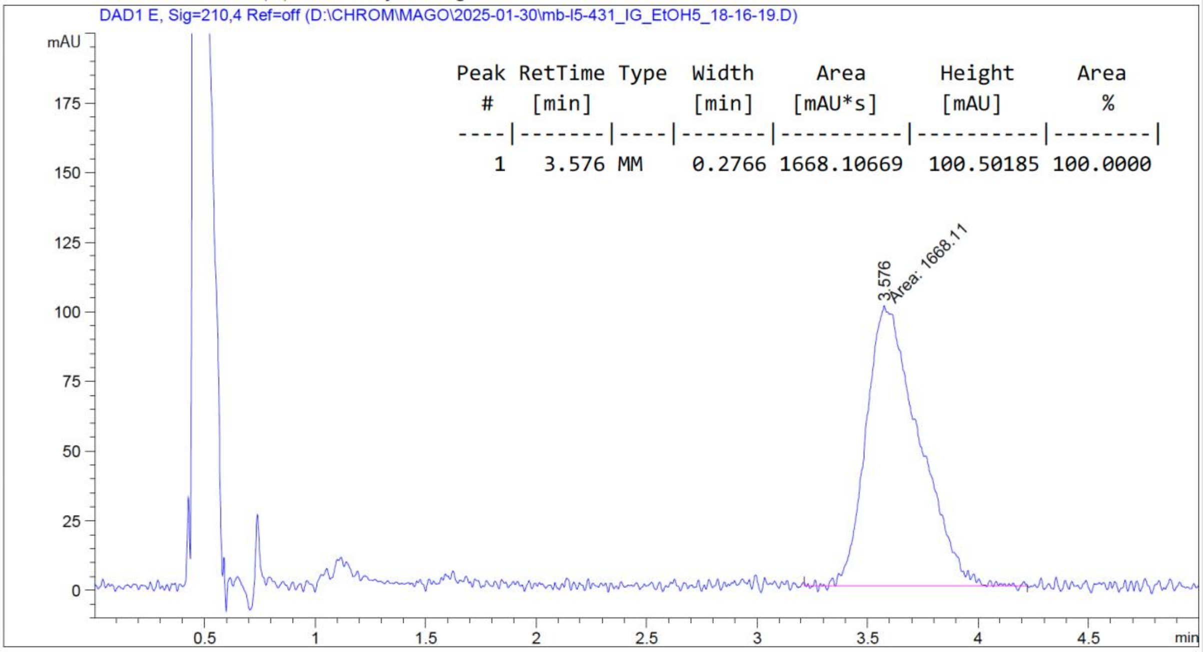

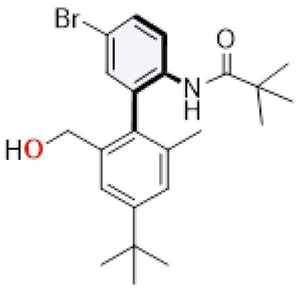

34a

SFC Analysis: Chiralpak IA, CO_2:2-propanol (15%), 35ºC, flow rate = 1.5 mL/min, λ = 210 nm. rt_1 = 1.1 min, rt_2 = 1.5 min.

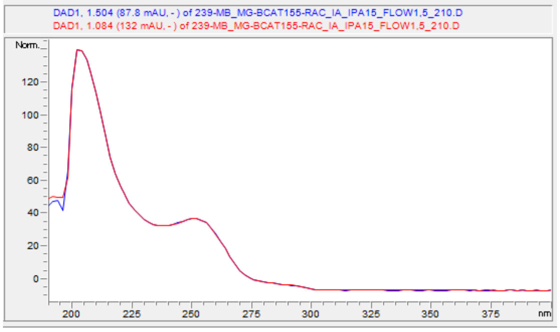

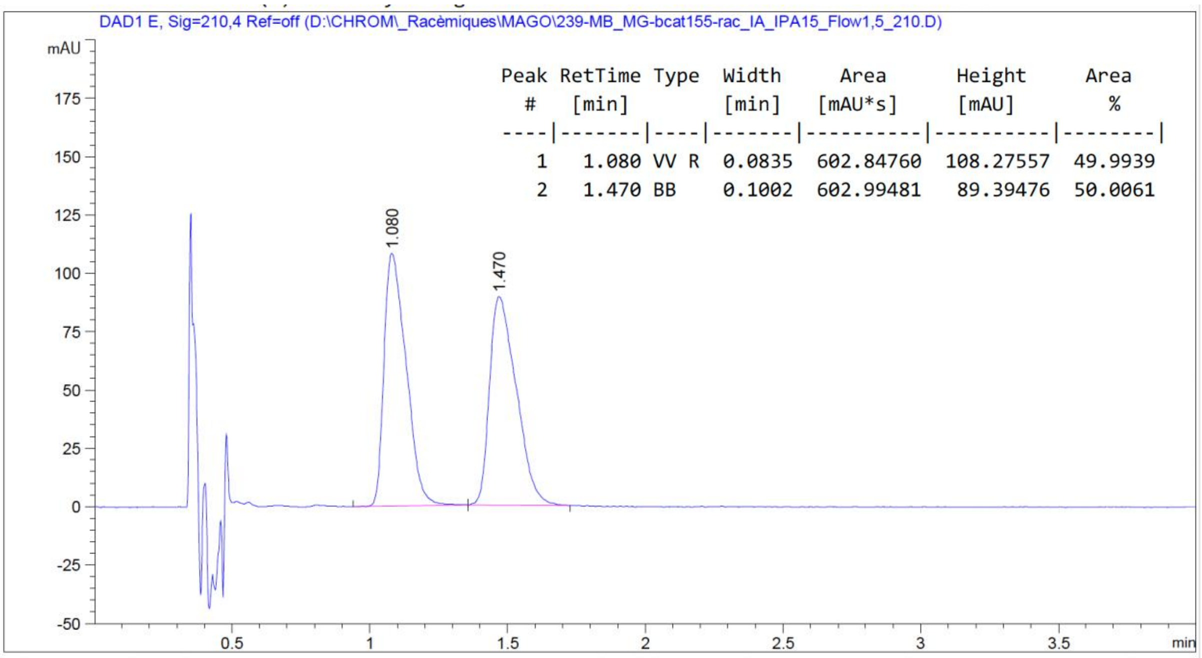

Peak #	RetTime [min]	Type	Width [min]	Area [mAU*s]	Height [mAU]	Area %
1	1.080	VV R	0.0835	602.84760	108.27557	49.9939
2	1.470	BB	0.1002	602.99481	89.39476	50.0061

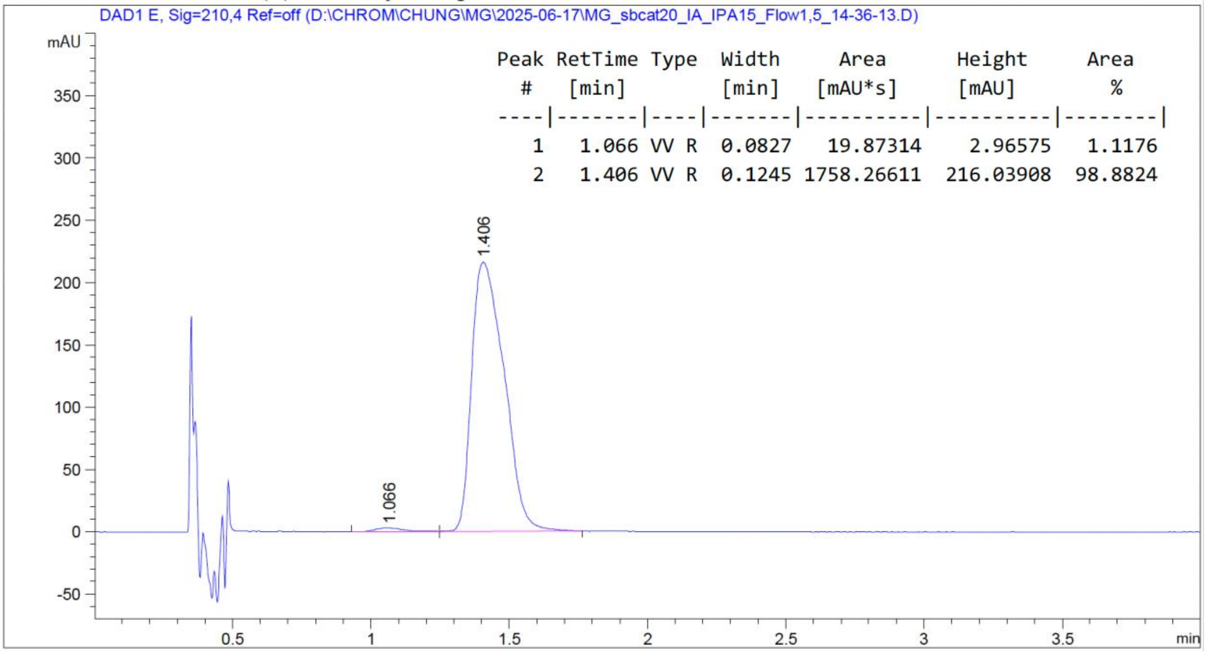

Peak #	RetTime [min]	Type	Width [min]	Area [mAU*s]	Height [mAU]	Area %
1	1.066	VV R	0.0827	19.87314	2.96575	1.1176
2	1.406	VV R	0.1245	1758.26611	216.03908	98.8824

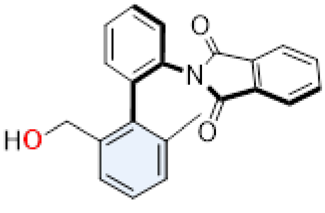

35a

SFC Analysis: Chiralpak IJ, CO_2:MeOH (10%), 35ºC, flow rate = 1.2 mL/min, λ = 220 nm. rt_1 = 2.5 min, rt_2 = 3.1 min.

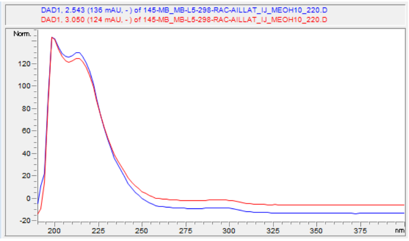

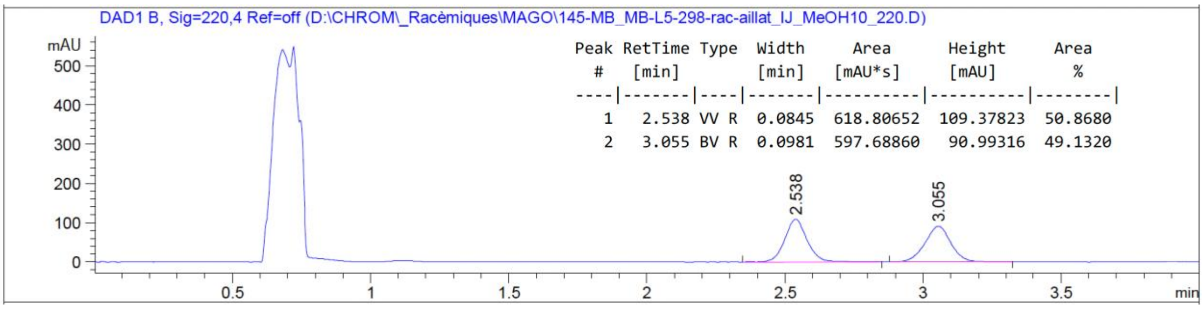

Peak #	RetTime [min]	Type	Width [min]	Area [mAU*s]	Height [mAU]	Area %
1	2.538	VV R	0.0845	618.80652	109.37823	50.8680
2	3.055	BV R	0.0981	597.68860	90.99316	49.1320

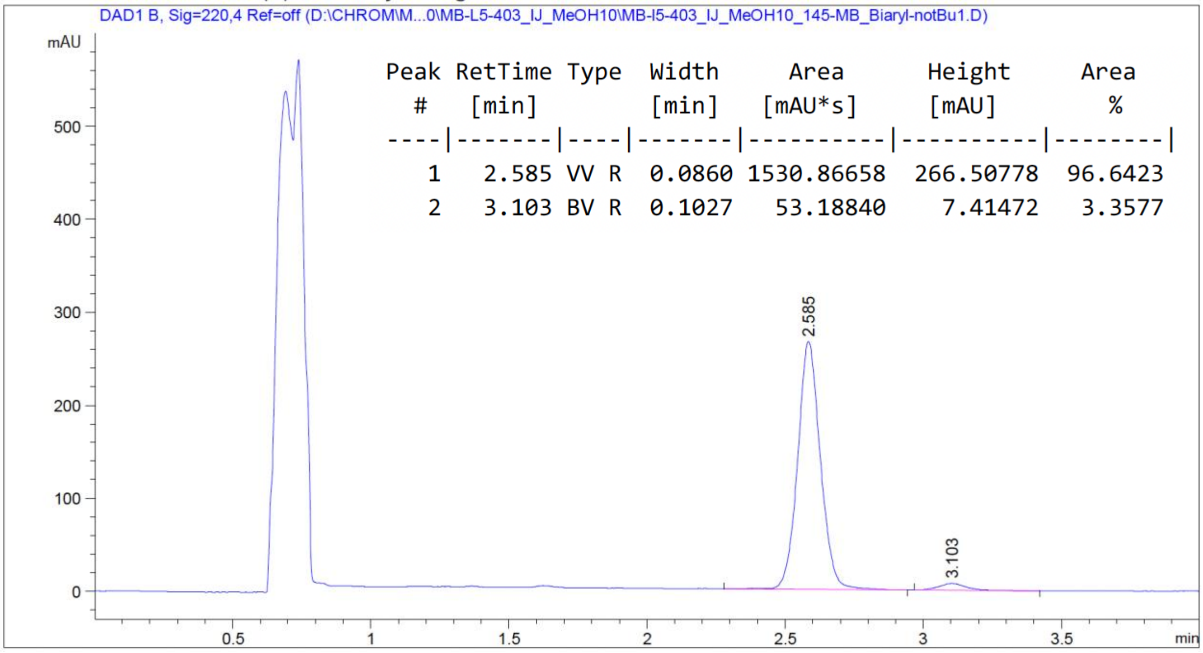

Peak #	RetTime [min]	Type	Width [min]	Area [mAU*s]	Height [mAU]	Area %
1	2.585	VV R	0.0860	1530.86658	266.50778	96.6423
2	3.103	BV R	0.1027	53.18840	7.41472	3.3577

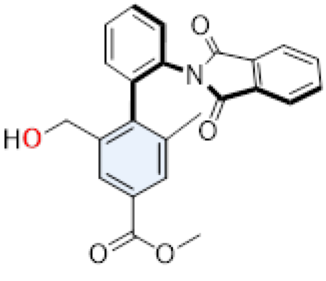

36a

SFC Analysis: Chiralpak IJ, CO_2:MeOH (15%), 35ºC, flow rate = 1.2 mL/min, λ = 220 nm. rt_1 = 1.4 min, rt_2 = 1.7 min.

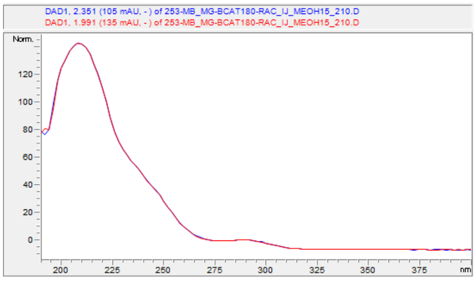

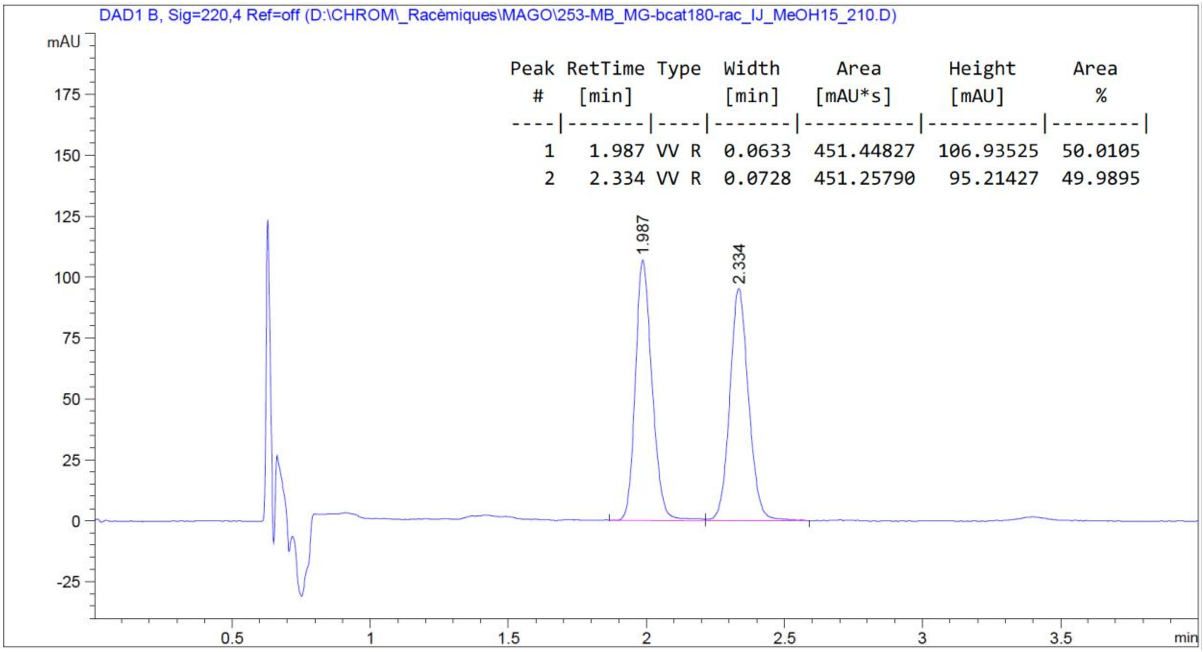

Peak #	RetTime [min]	Type	Width [min]	Area [mAU*s]	Height [mAU]	Area %
1	1.987	VV R	0.0633	451.44827	106.93525	50.0105
2	2.334	VV R	0.0728	451.25790	95.21427	49.9895

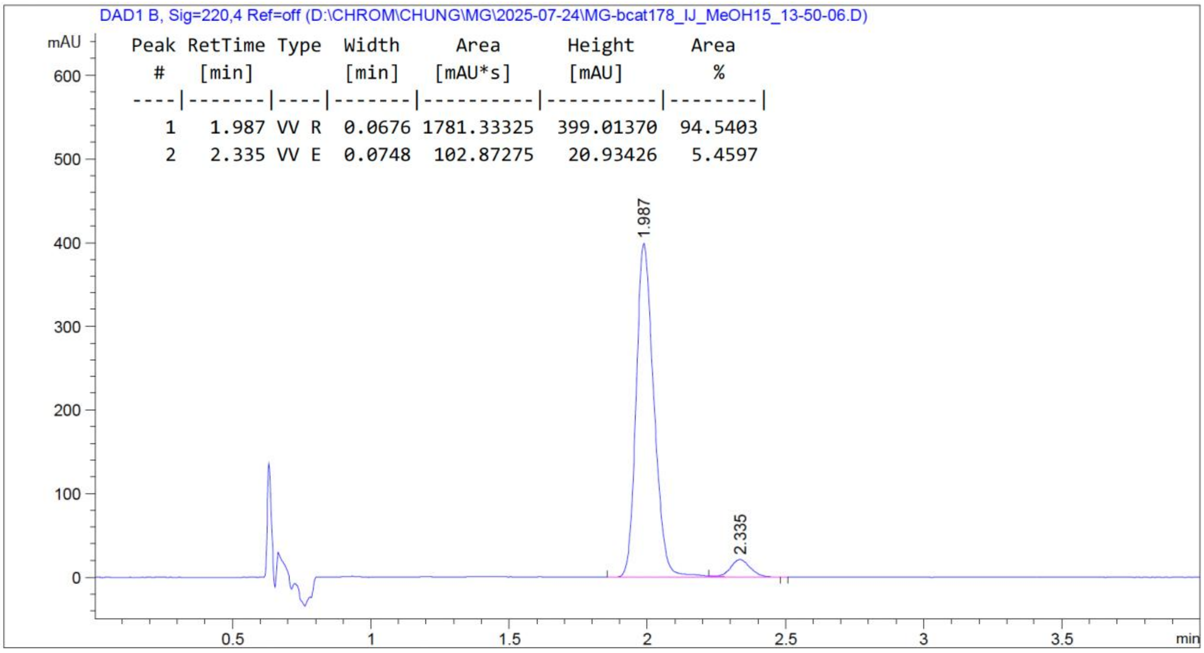

Peak #	RetTime [min]	Type	Width [min]	Area [mAU*s]	Height [mAU]	Area %
1	1.987	VV R	0.0676	1781.33325	399.01370	94.5403
2	2.335	VV E	0.0748	102.87275	20.93426	5.4597

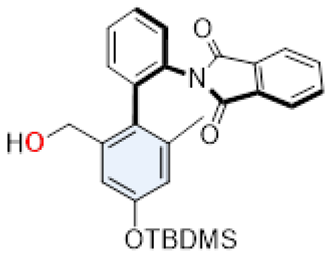

37a

SFC Analysis: Chiralpak IJ, CO_2:MeOH (10 %), 35ºC, flow rate = 1.2 mL/min, λ = 210 nm. rt_1 = 1.6 min, rt_2 = 1.9 min.

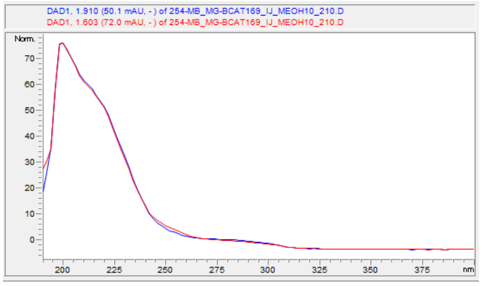

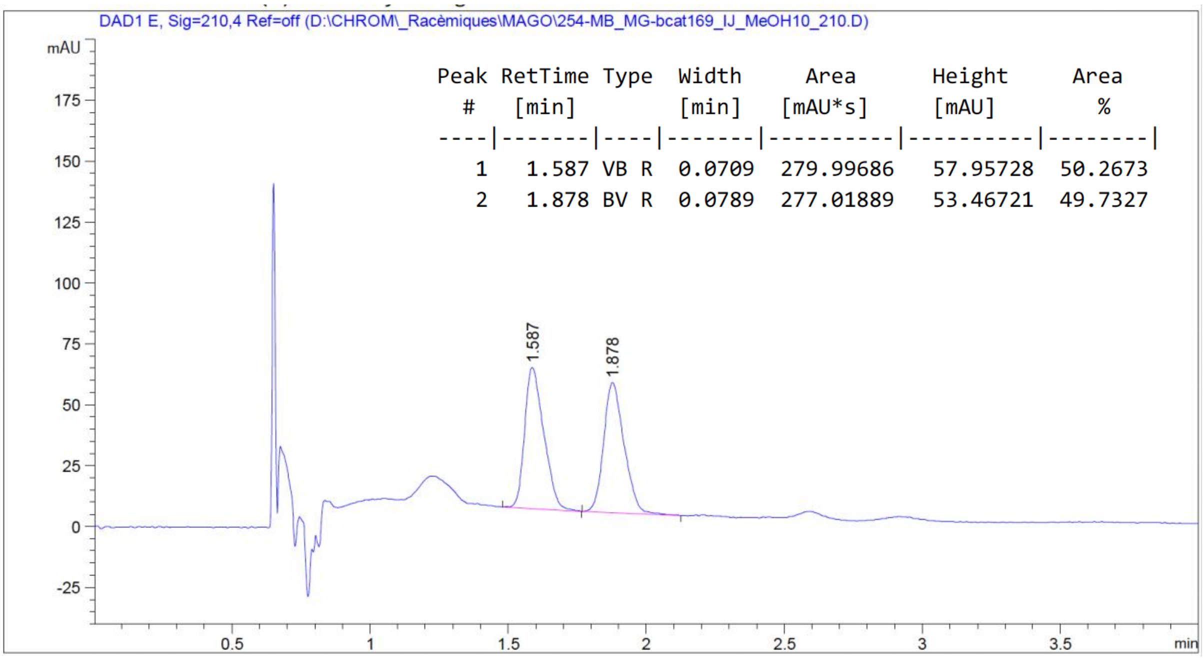

Peak #	RetTime [min]	Type	Width [min]	Area [mAU*s]	Height [mAU]	Area %
1	1.587	VB R	0.0709	279.99686	57.95728	50.2673
2	1.878	BV R	0.0789	277.01889	53.46721	49.7327

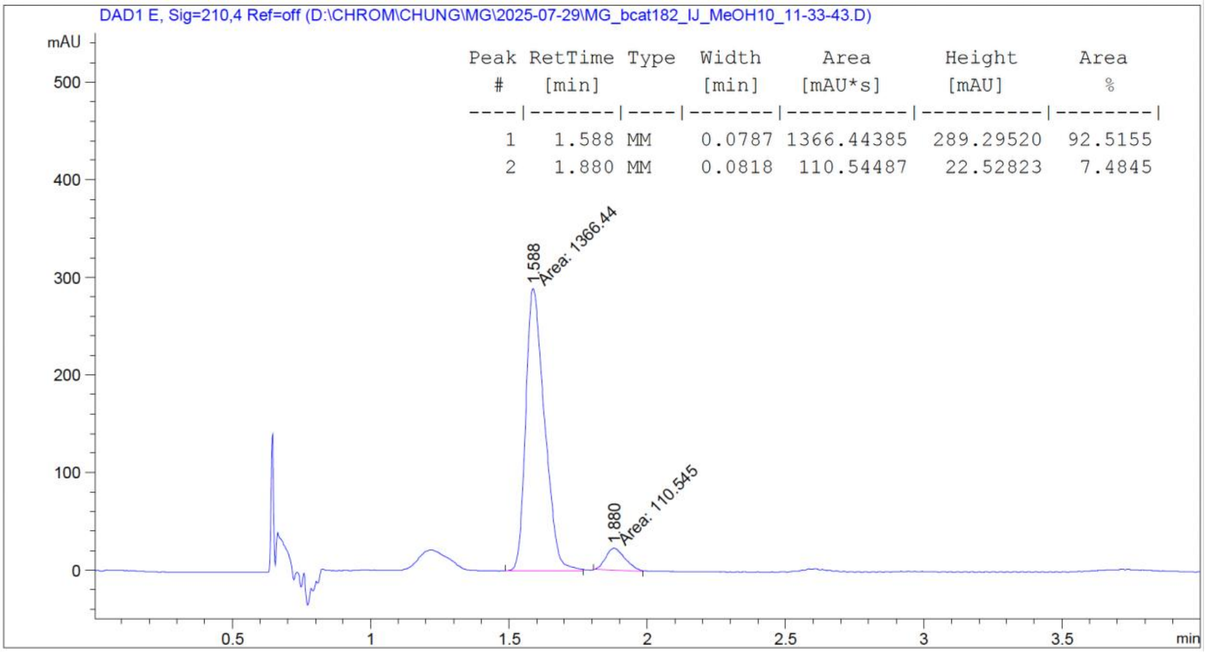

Peak #	RetTime [min]	Type	Width [min]	Area [mAU*s]	Height [mAU]	Area %
1	1.588	MM	0.0787	1366.44385	289.29520	92.5155
2	1.880	MM	0.0818	110.54487	22.52823	7.4845

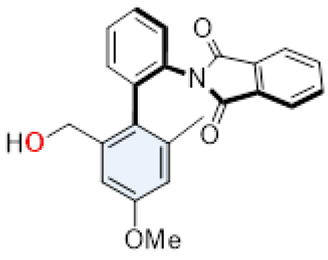

38a

SFC Analysis: Chiralpak IJ, CO_2:MeOH (10 %), 35ºC, flow rate = 1.2 mL/min, λ = 210 nm. rt_1 = 2.9 min, rt_2 = 3.4 min.

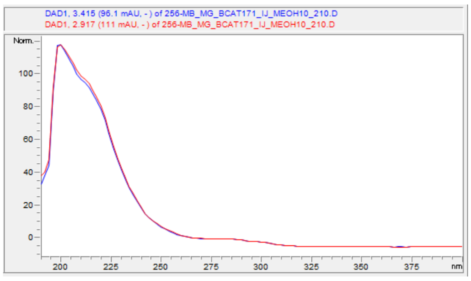

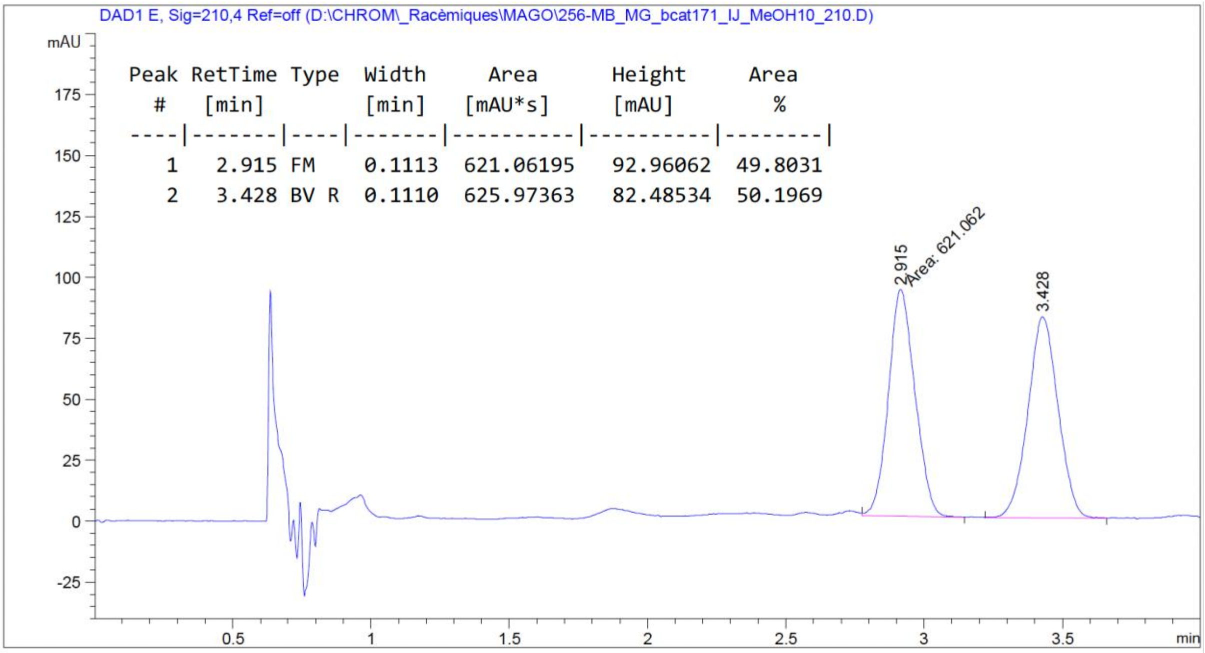

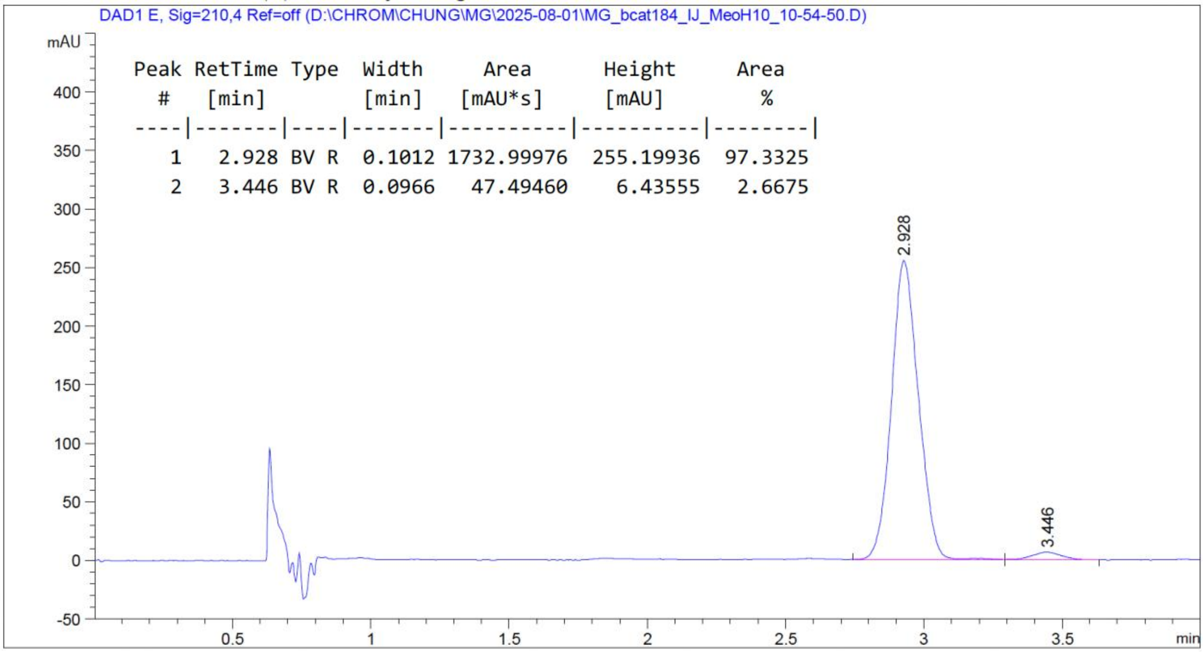

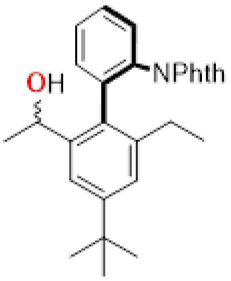

39a+39a'

SFC Analysis: Chiralpak IC, CO_2:EtOH (5%), 35ºC, flow rate = 1.5 mL/min, λ = 210 nm. Major diastereoisomer: rt_1 = 5.4 min, rt_2 = 6.8 min; minor diastereoisomer: rt_1 = 7.8 min, rt_2 = 9.2 min

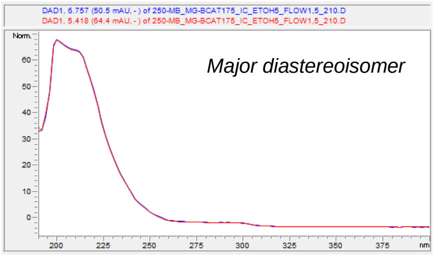

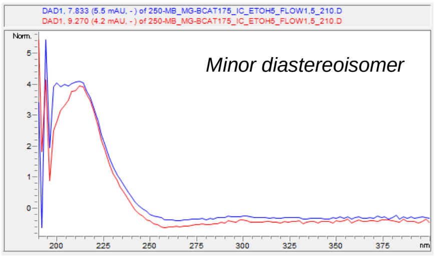

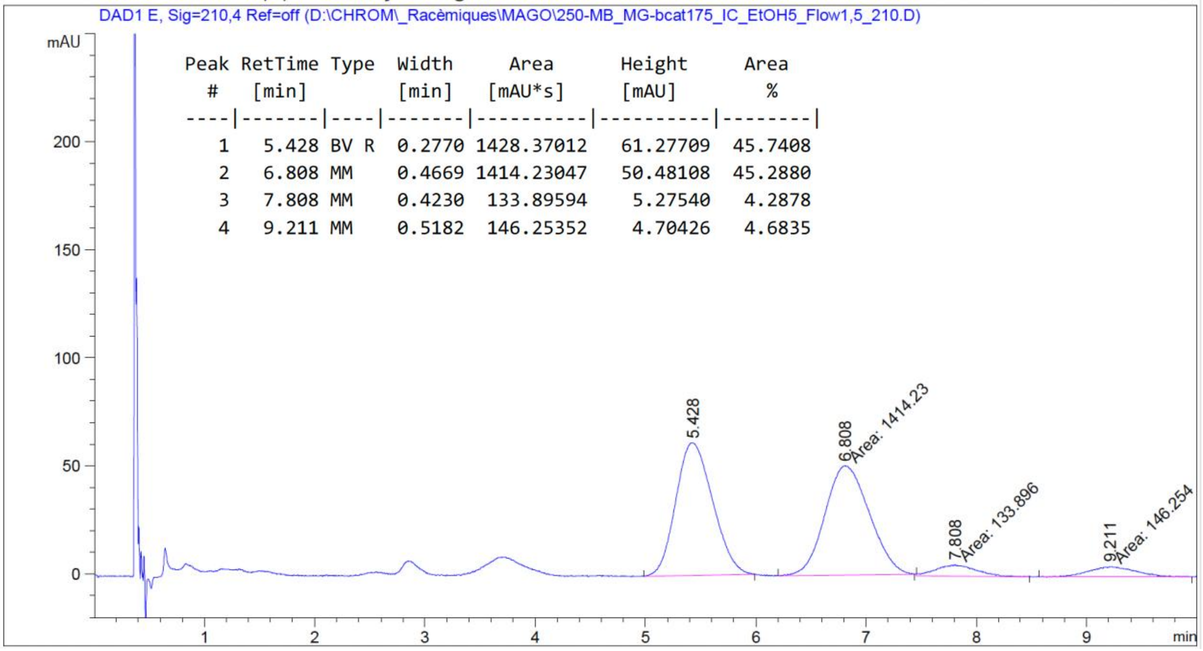

Peak #	RetTime [min]	Type	Width [min]	Area [mAU*s]	Height [mAU]	Area %
1	5.428	BV R	0.2770	1428.37012	61.27709	45.7408
2	6.808	MM	0.4669	1414.23047	50.48108	45.2880
3	7.808	MM	0.4230	133.89594	5.27540	4.2878
4	9.211	MM	0.5182	146.25352	4.70426	4.6835

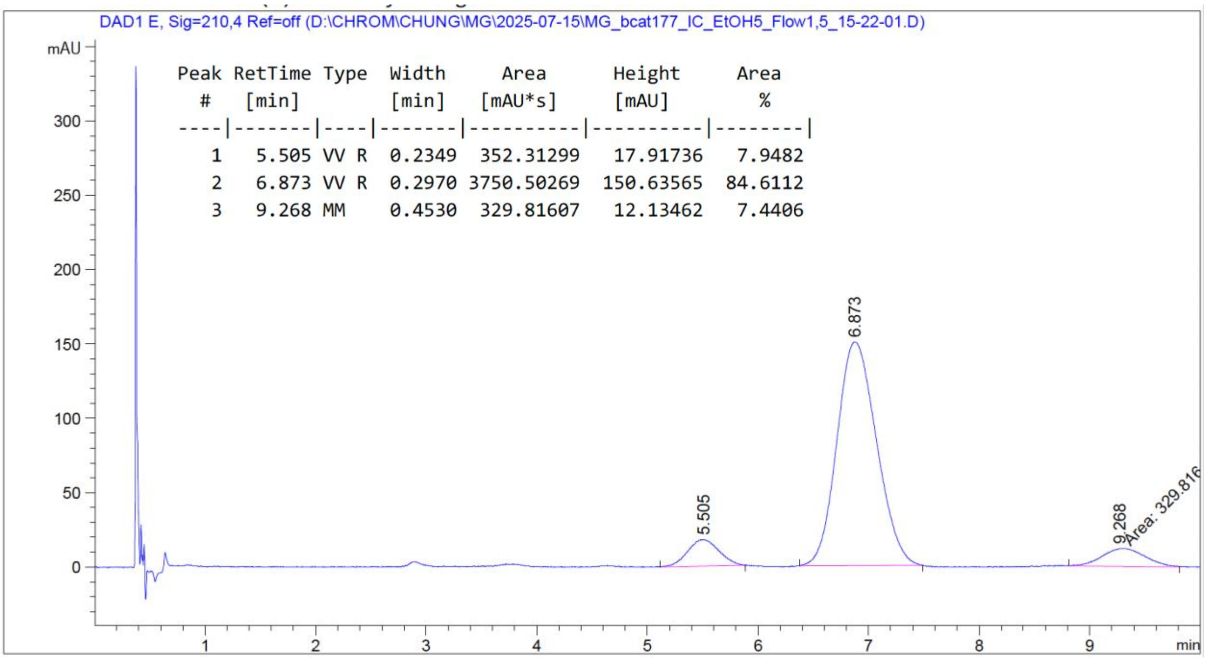

Peak #	RetTime [min]	Type	Width [min]	Area [mAU*s]	Height [mAU]	Area %
1	5.505	VV R	0.2349	352.31299	17.91736	7.9482
2	6.873	VV R	0.2970	3750.50269	150.63565	84.6112
3	9.268	MM	0.4530	329.81607	12.13462	7.4406

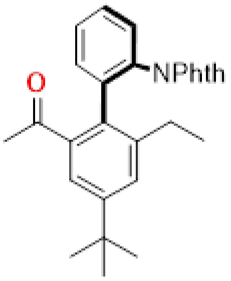

39b

SFC Analysis: Chiralpak IB, CO_2:EtOH (10%), 35ºC, flow rate = 1.2 mL/min, λ = 210 nm. rt_1 = 1.8 min, rt_2 = 2.1 min.

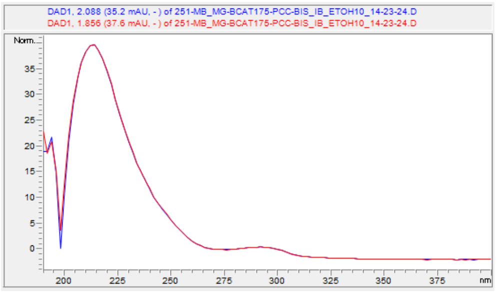

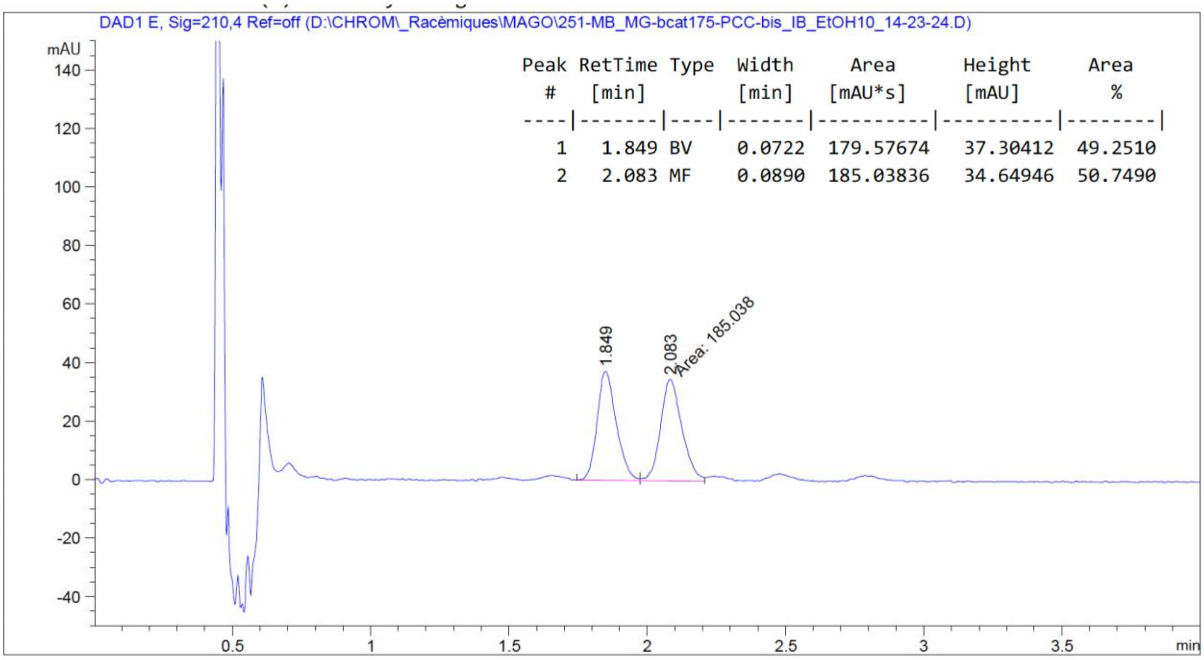

Peak #	RetTime [min]	Type	Width [min]	Area [mAU*s]	Height [mAU]	Area %
1	1.849	BV	0.0722	179.57674	37.30412	49.2510
2	2.083	MF	0.0890	185.03836	34.64946	50.7490

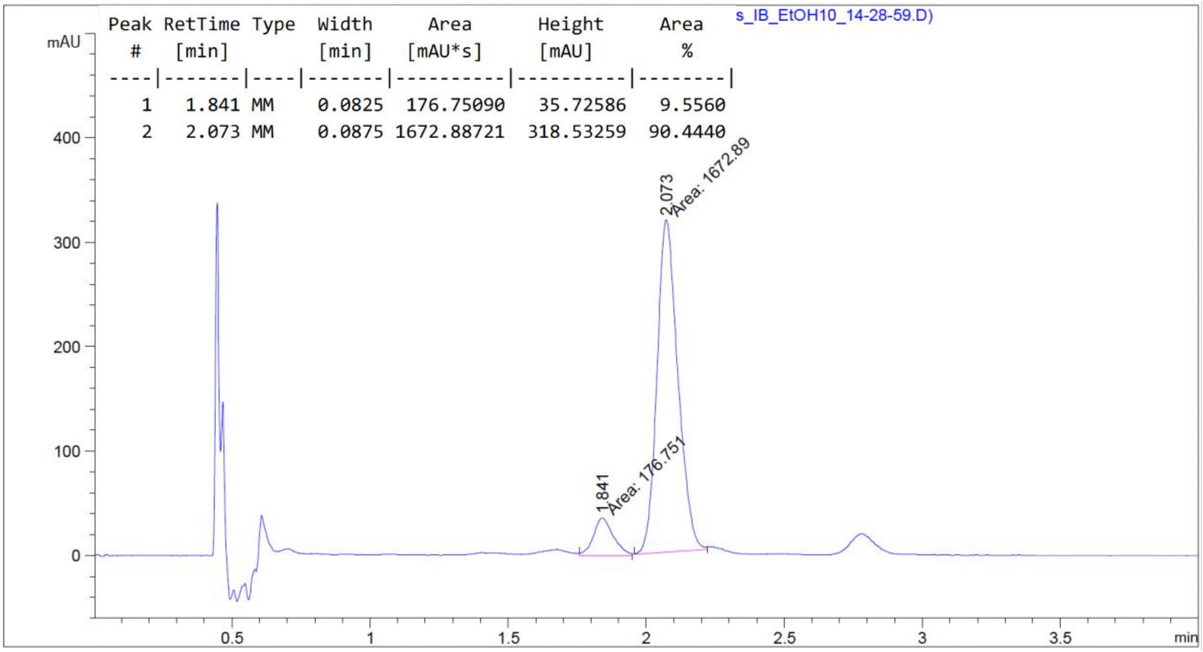

Peak #	RetTime [min]	Type	Width [min]	Area [mAU*s]	Height [mAU]	Area %
1	1.841	MM	0.0825	176.75090	35.72586	9.5560
2	2.073	MM	0.0875	1672.88721	318.53259	90.4440

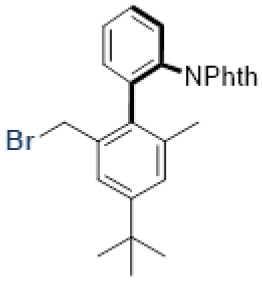

40

SFC Analysis: Chiralpak IJ, CO_2:MeOH (5%), 35ºC, flow rate = 1.2 mL/min, λ = 210 nm. rt_1 = 2.7 min, rt_2 = 3.2 min.

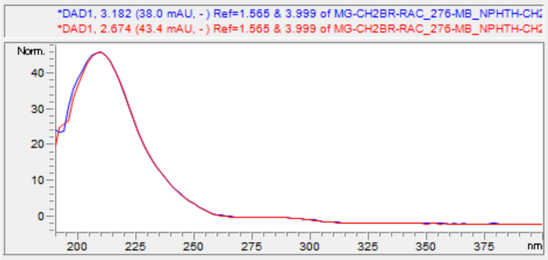

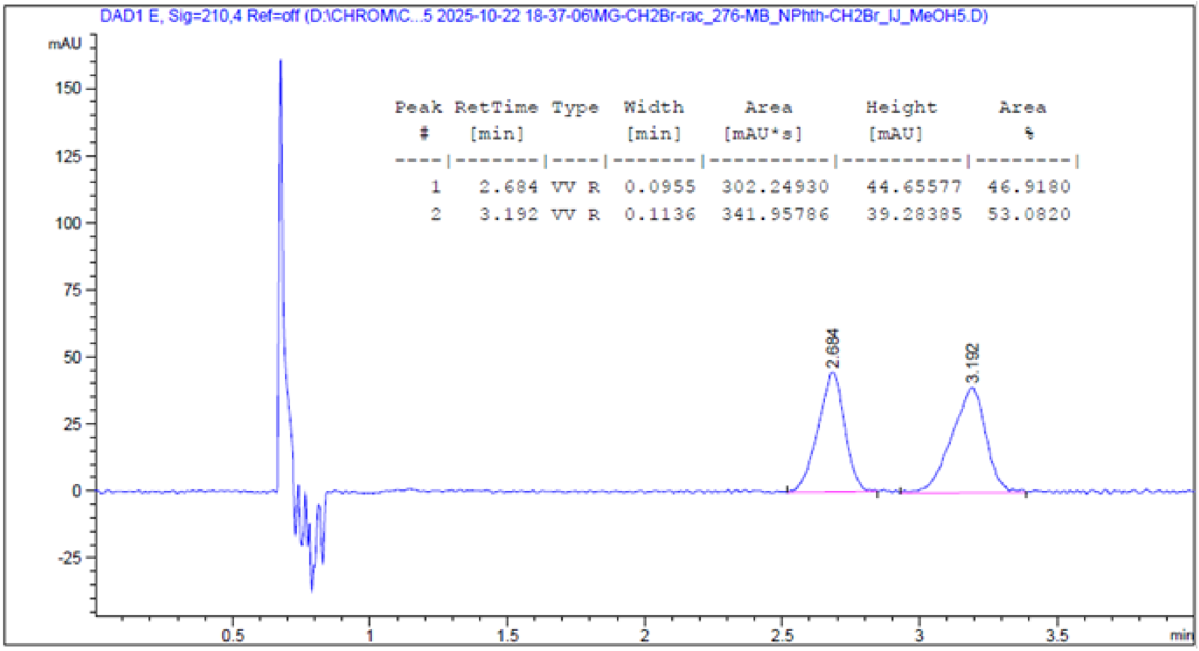

Racemic **40** was obtained by using a mixture of (*R*,*R*)- and (*S*,*S*)-**C6**.

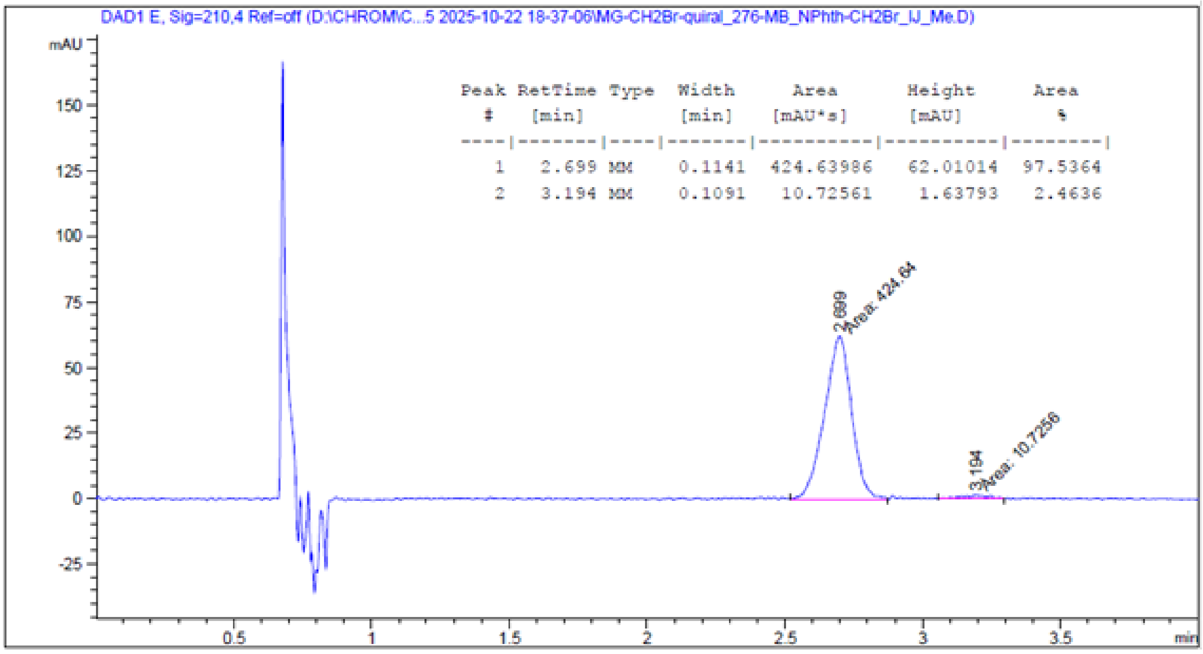

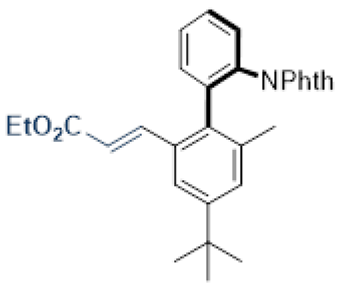

41

SFC Analysis: Chiralpak IC, CO_2:MeOH (5%), 35ºC, flow rate = 1.2 mL/min, λ = 210 nm. rt_1 = 5.7 min, rt_2 = 6.5 min.

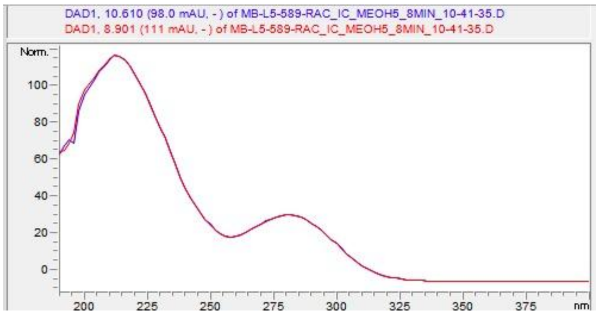

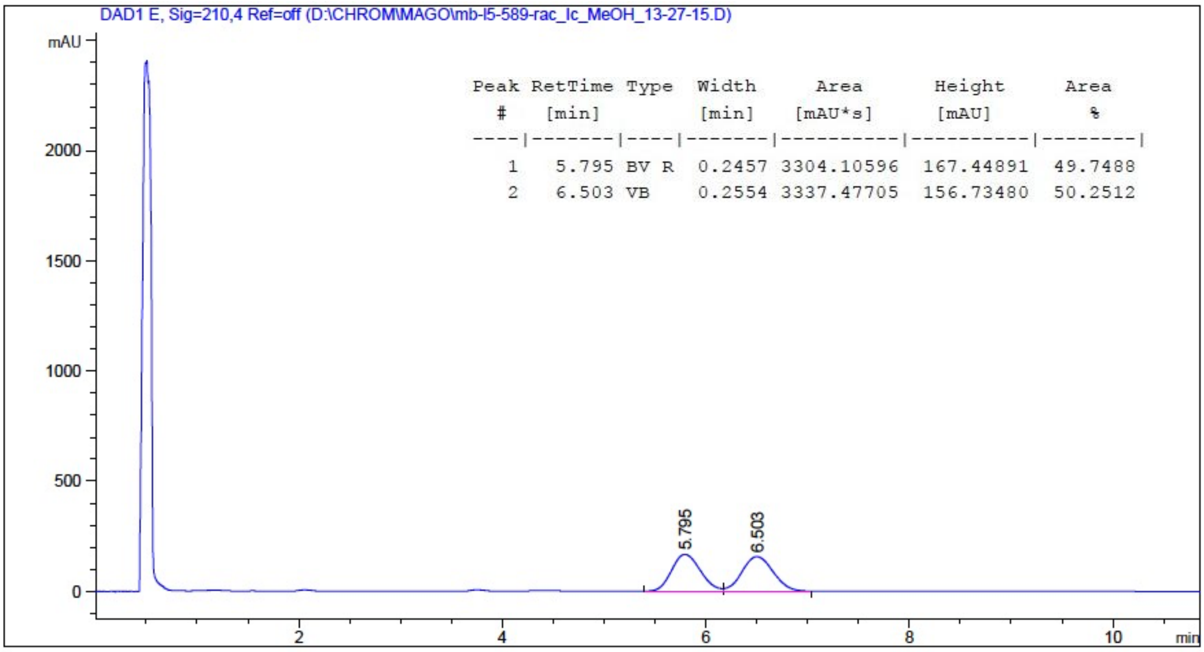

Peak #	RetTime [min]	Type	Width [min]	Area [mAU*s]	Height [mAU]	Area %
1	5.795	BV R	0.2457	3304.10596	167.44891	49.7488
2	6.503	VB	0.2554	3337.47705	156.73480	50.2512

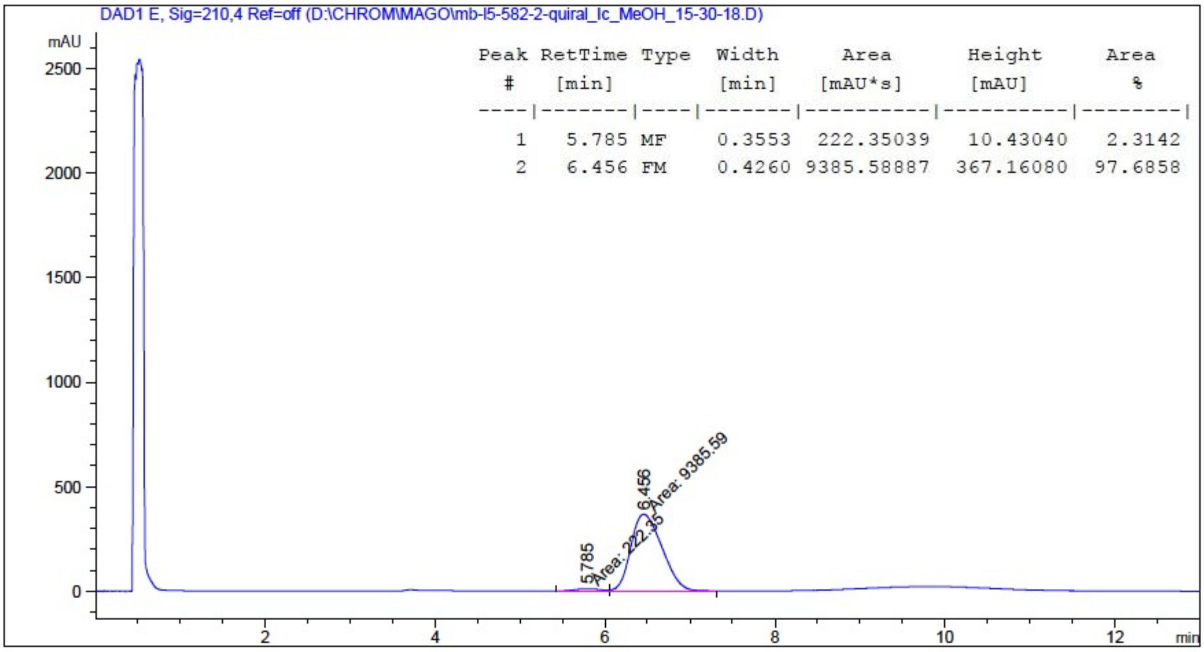

Peak #	RetTime [min]	Type	Width [min]	Area [mAU*s]	Height [mAU]	Area %
1	5.785	MF	0.3553	222.35039	10.43040	2.3142
2	6.456	FM	0.4260	9385.58887	367.16080	97.6858

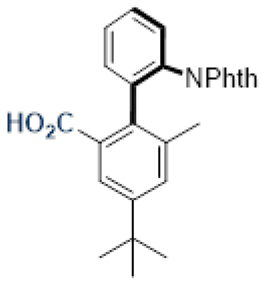

18c

SFC Analysis: Chiralpak IC, CO_2:MeOH (5%), 35ºC, flow rate = 1.2 mL/min, λ = 210 nm. rt_1 = 11.3 min, rt_2 = 13 min.

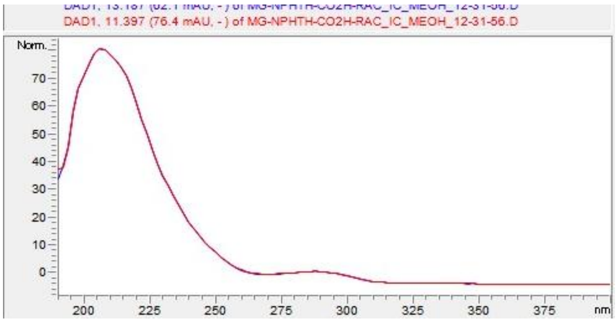

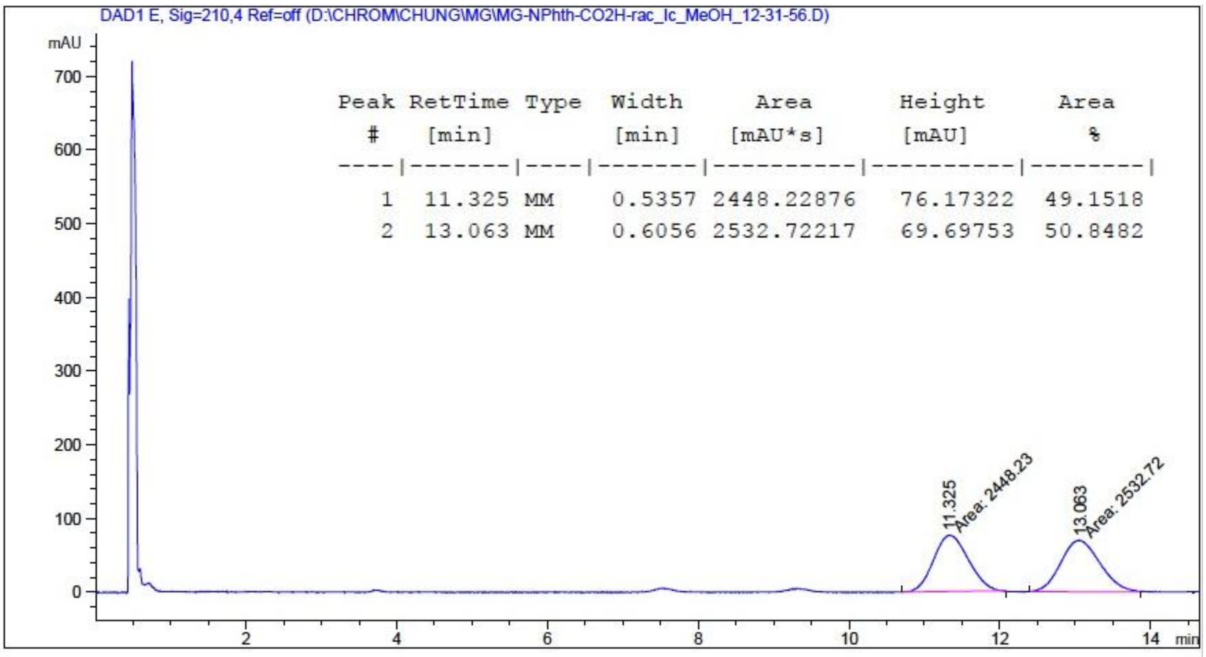

Peak #	RetTime [min]	Type	Width [min]	Area [mAU*s]	Height [mAU]	Area %
1	11.325	MM	0.5357	2448.22876	76.17322	49.1518
2	13.063	MM	0.6056	2532.72217	69.69753	50.8482

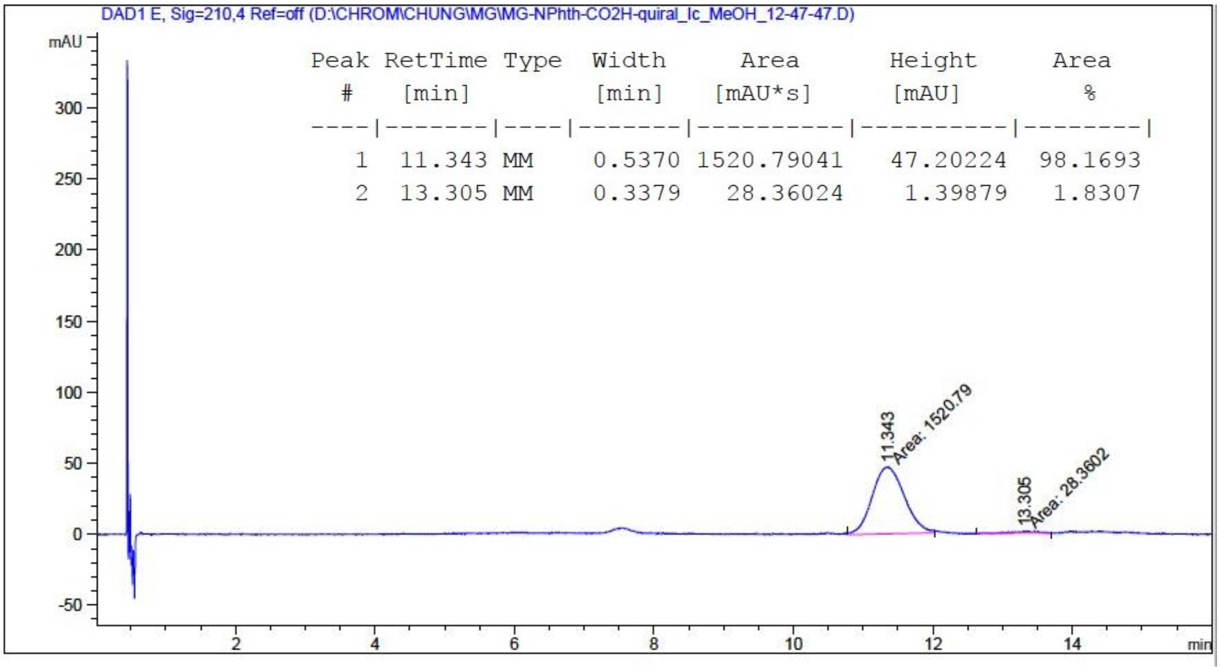

Peak #	RetTime [min]	Type	Width [min]	Area [mAU*s]	Height [mAU]	Area %
1	11.343	MM	0.5370	1520.79041	47.20224	98.1693
2	13.305	MM	0.3379	28.36024	1.39879	1.8307

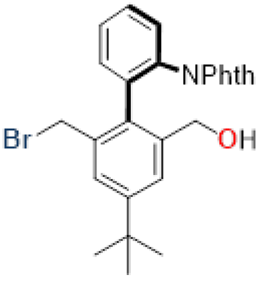

40a

SFC Analysis: Chiralpak IC, CO_2:MeOH (4%), 35ºC, flow rate =1.4 mL/min, λ = 210 nm. rt_1 = 15.1 min, rt_2 = 16.3 min

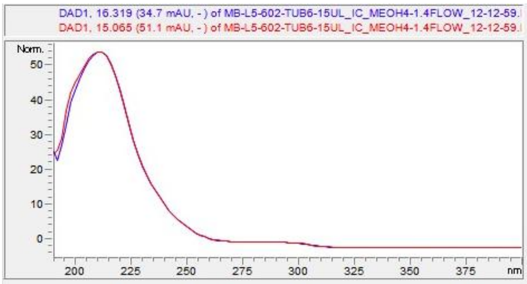

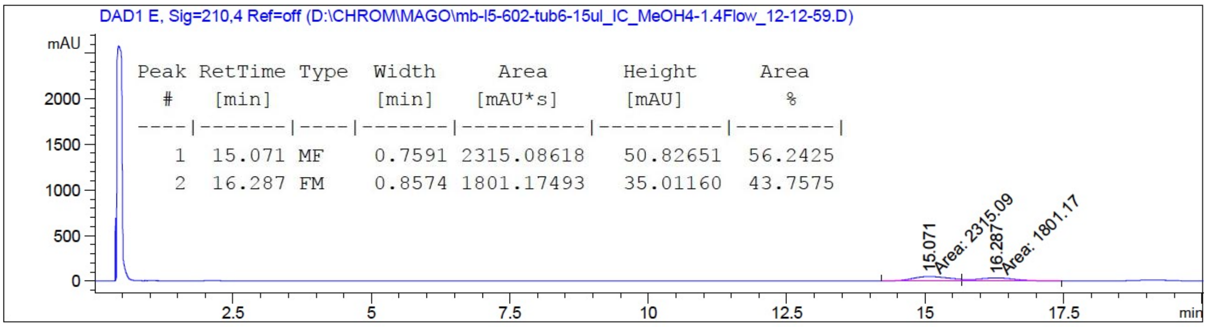

Racemic **40a** was obtained by using a mixture of (*R*,*R*)- and (*S*,*S*)-**C6**.

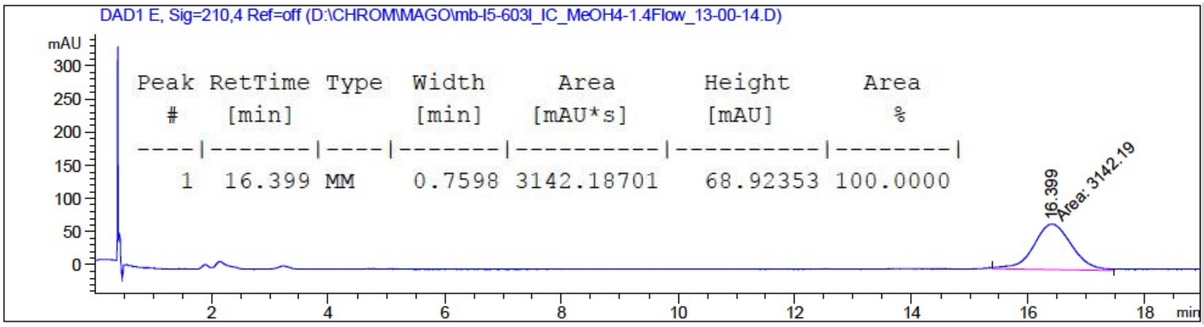

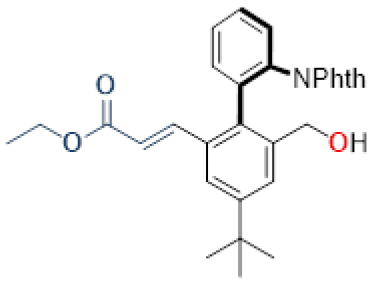

41a

SFC Analysis: Chiralpak IC, CO_2:MeOH (12%), 35ºC, flow rate =1.2 mL/min, λ = 275 nm. rt_1 = 5.8 min, rt_2 = 7.0 min.

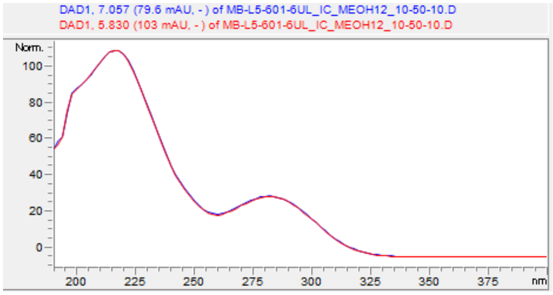

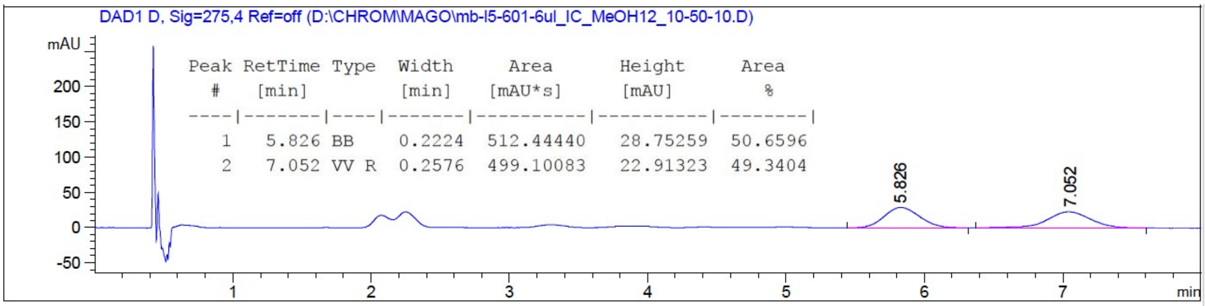

Peak #	RetTime [min]	Type	Width [min]	Area [mAU*s]	Height [mAU]	Area %
1	5.826	BB	0.2224	512.44440	28.75259	50.6596
2	7.052	VV R	0.2576	499.10083	22.91323	49.3404

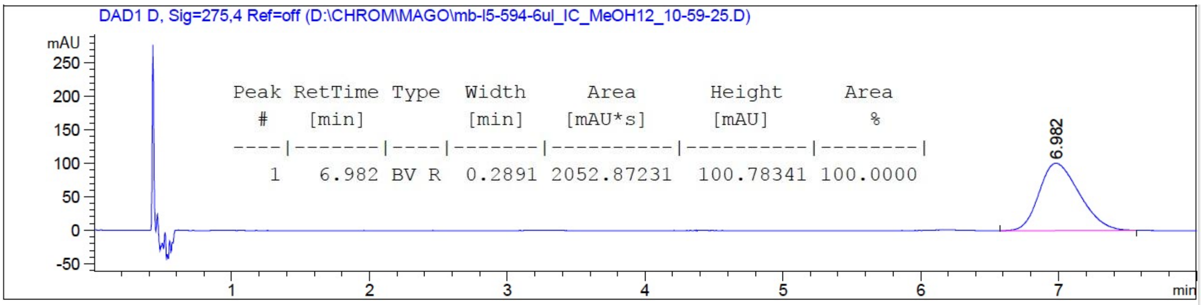

Peak #	RetTime [min]	Type	Width [min]	Area [mAU*s]	Height [mAU]	Area %
1	6.982	BV R	0.2891	2052.87231	100.78341	100.0000

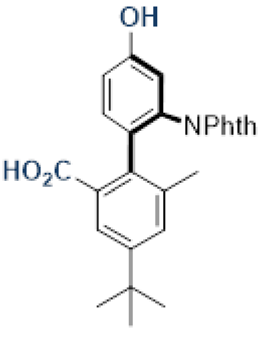

42

SFC Analysis: Chiralpak IC, CO_2:MeOH (10%), 35ºC, flow rate =1.2 mL/min, λ = 210 nm. rt_1 = 6.3 min, rt_2 = 7.5 min.

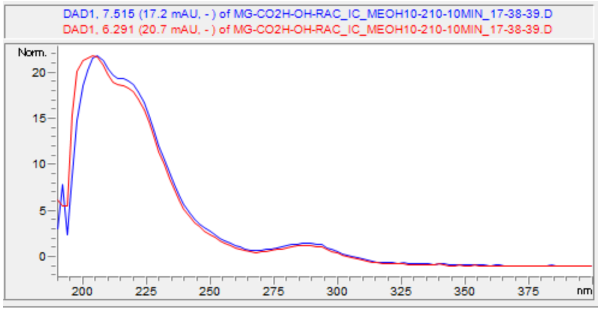

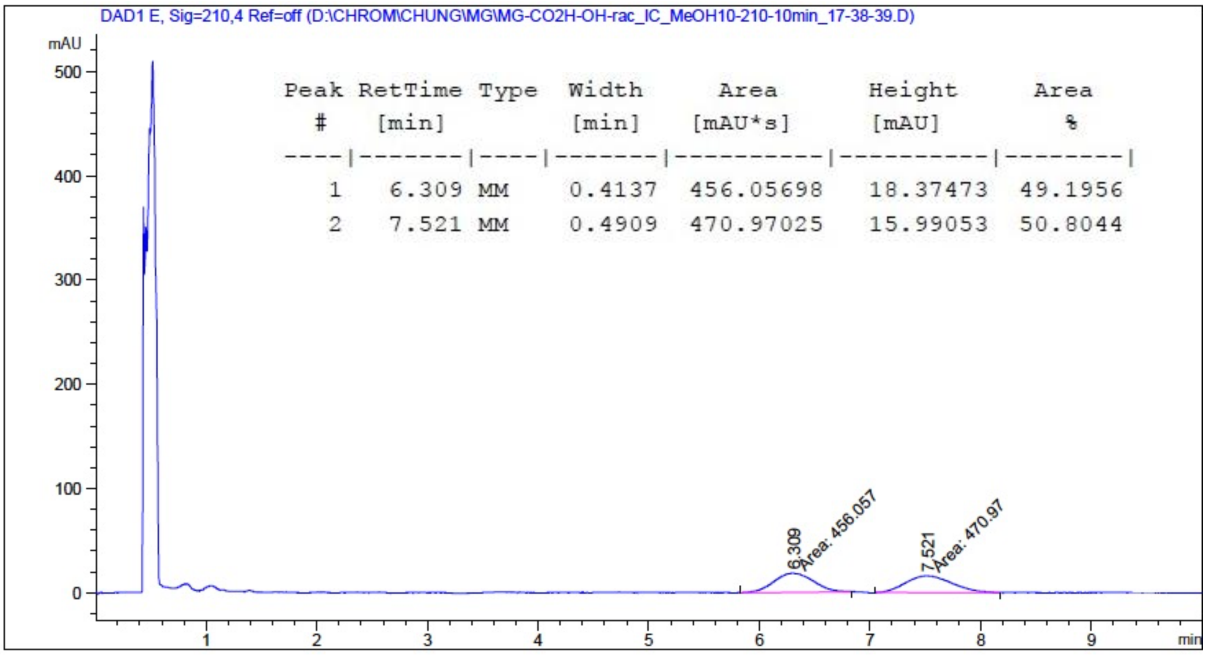

Peak #	RetTime [min]	Type	Width [min]	Area [mAU*s]	Height [mAU]	Area %
1	6.309	MM	0.4137	456.05698	18.37473	49.1956
2	7.521	MM	0.4909	470.97025	15.99053	50.8044

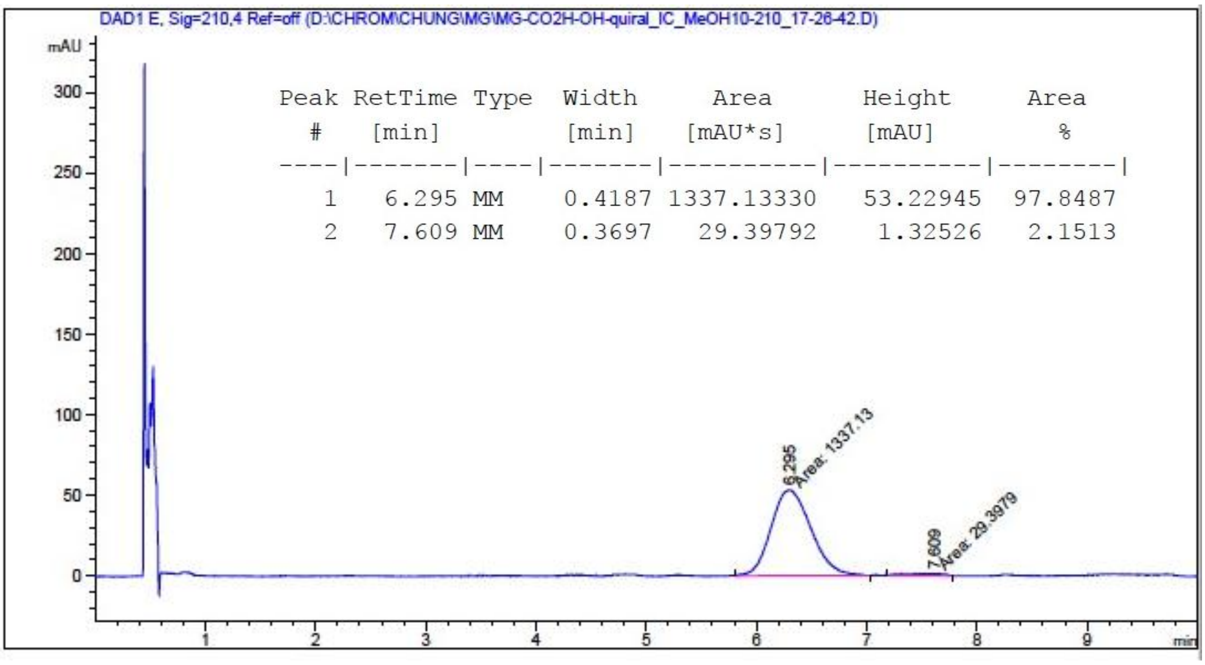

Peak #	RetTime [min]	Type	Width [min]	Area [mAU*s]	Height [mAU]	Area %
1	6.295	MM	0.4187	1337.13330	53.22945	97.8487
2	7.609	MM	0.3697	29.39792	1.32526	2.1513
